# TorsiFlex: an automatic generator of torsional conformers. Application to the twenty proteinogenic amino acids

**DOI:** 10.1186/s13321-021-00578-0

**Published:** 2021-12-24

**Authors:** David Ferro-Costas, Irea Mosquera-Lois, Antonio Fernández-Ramos

**Affiliations:** Centro Singular de Investigación en Química Biolóxica e Materiais Moleculares (CIQUS), Universidade de Santiago de Compostela, 15782 Santiago de Compostela, Spain

**Keywords:** Conformations, Flexible molecules, Torsions, Preconditioned search, Stochastic search, Validation tests, Amino acids

## Abstract

In this work, we introduce TorsiFlex, a user-friendly software written in Python 3 and designed to find *all* the torsional conformers of flexible acyclic molecules in an automatic fashion. For the mapping of the torsional potential energy surface, the algorithm implemented in TorsiFlex combines two searching strategies: preconditioned and stochastic. The former is a type of systematic search based on chemical knowledge and should be carried out before the stochastic (random) search. The algorithm applies several validation tests to accelerate the exploration of the torsional space. For instance, the optimized structures are stored and this information is used to prevent revisiting these points and their surroundings in future iterations. TorsiFlex operates with a dual-level strategy by which the initial search is carried out at an inexpensive electronic structure level of theory and the located conformers are reoptimized at a higher level. Additionally, the program takes advantage of conformational enantiomerism, when possible. As a case study, and in order to exemplify the effectiveness and capabilities of this program, we have employed TorsiFlex to locate the conformers of the twenty proteinogenic amino acids in their neutral canonical form. TorsiFlex has produced a number of conformers that roughly doubles the amount of the most complete work to date.

**Graphical Abstract:**

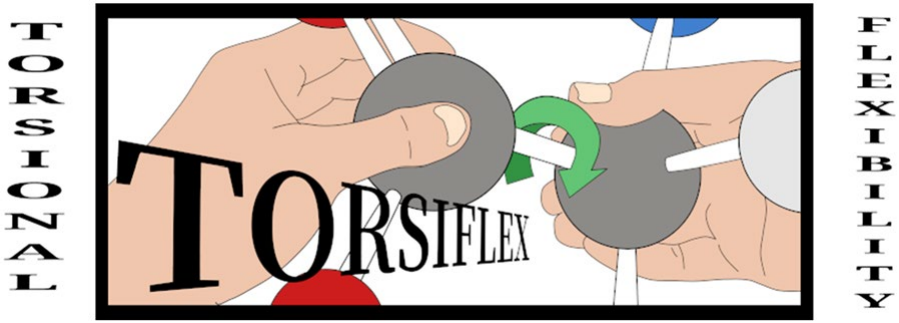

**Supplementary Information:**

The online version contains supplementary material available at 10.1186/s13321-021-00578-0.

## Introduction

Flexible molecules are prone to adopt different geometries due to the internal rotation about single bonds. Each of these equilibrium structures is represented by a unique spatial configuration, that is, a conformational isomer. Temperature and relative stability decide the conformations that possess a significant population; thus, in astrochemical studies (low temperature), it suffices to identify the most stable conformers, whereas in combustion studies (high temperature) most or all conformers need to be characterized. However, even if we just focus on the most stable equilibrium structures, it is very complex to know a priori their location, and the computational effort is, in many situations, similar to the exploration of the whole configurational space. Therefore, the search and location of equilibrium structures in flexible molecules with multiple internal rotations demands an efficient sampling of the torsional potential energy surface (PES).

Some of the first and still valid ideas are based on the Metropolis Monte Carlo sampling [1–3], the eigenvector-following algorithm [4–6], or the systematic search [7], although very recently machine learning [8–10], genetic [11], and meta-dynamics algorithms [12] are probing new routes. The application of any of these algorithms to large systems (cyclic or acyclic) usually requires improving their efficiency, and sometimes they are combined with knowledge-based methods that profit from databanks and/or diverse force field implementations but, at the same time, also rely heavily on them [13–19]. For systems of more modest size and for the location of the low-energy conformers, it is possible to adopt semiempirical tight-binding [20], or a combination of low-level quantum mechanics (QM) and molecular mechanics methods (MM) [21].

TorsiFlex is a *Python 3* code designed in the line of methods that handle flexible acyclic molecules of modest size (for instance, compounds of astronomical interest, amino acids and small peptides, organic molecules of a few tenths of atoms, etc.), with the objective of locating *all* the equilibrium structures of the torsional PES. The algorithm of TorsiFlex combines preconditioned and stochastic approaches for the sampling of configurational space [22, 23]. By preconditioned search, we mean a combination of systematic search and chemistry-based knowledge. It has been reported that accounting just for the anticipated dispositions may miss relevant parts of the conformational space (an example is the 3$^N$ estimator for $sp^3$ carbon atoms) [24]. For this reason, the preconditioned search followed by the stochastic exploration allows locating, not just the expected conformers, but also conformers that go beyond the chemically intuitive arrangements. As many other methods, our approach is complemented by dual-level (or two-level) calculations such that the conformational search can be carried out at an inexpensive level of calculation (low level, LL) whereas the final conformer refinement relies on more accurate calculations (high level, HL).

Most algorithms (ours included) entrust sampling strategies to LL (usually MM) with the idea of a fast exploration of the PES. This initial step is key in the location of the stationary points because these LL minima are adopted as the starting point for HL calculations. The main drawback of this procedure is that the LL and HL PESs should bear a certain degree of resemblance to grant that the most relevant LL regions are also of relevance at HL; otherwise there is a risk of overlooking some of the low-energy equilibrium conformations. For this reason, we prefer to employ LL ab initio methods instead of semiempirical or MM methods, although the latter are substantially faster.

Independently of the subsequent handling of the LL information, the generation of a suitable set of LL structures is crucial at any rate, although the demand for HL information can be adjusted. Thus, we may be concerned only about the identification of the most stable conformer(s) among the bevy of them, as for instance when dealing with astrochemical studies, [25] or when interpreting high-resolution microwave spectra at cryogenic temperatures. [26] In other situations, as for instance when studying the mechanism of bioalcohols or other combustion biofuels, most of the HL conformers, if not all, need to be identified. [27] The latter is required to calculate thermodynamic properties from the gathered structural information. Particularly, these macroscopic properties can be extracted from a single generating function, that is, the partition function. There exist different strategies to incorporate the effect of several conformers in the partition function, such as the multi-structural harmonic-oscillator approximation (MS-HO) [27–29], the extended two-dimensional torsional method (E2DT) [30–32], the coupled torsional anharmonic approximation (MS-T) [33, 34], or composite methods [35, 36] among others. However, all of them share a common starting point toward the evaluation of more accurate partition functions: the search and location of the torsional conformers.

In this work, we aim to find *all* the conformations of the twenty proteinogenic amino acids, pAAs, in their neutral canonical form. From a biological point of view, they constitute the building blocks of proteins and are associated with the machinery of life [37]. From a chemical point of view, pAAs display remarkable conformational flexibility, a situation that is hampered by the presence of hydrogen bond interactions. These properties are crucial when studying the dynamics of the protein backbones. The number of torsions in amino acids, and consequently the number of conformers, varies greatly from one system to another. For example, glycine (Gly), the simplest amino acid, contains a total of three torsions, whereas other amino acids, like aspartic acid (Asp), glutamine (Gln) and methionine (Met), comprise up to 6 torsions. The most complicated situation can be encountered in arginine (Arg), whose conformational flexibility is determined by a total of 9 torsions. These aspects, together with their moderate molecular size, render pAAs as attractive targets to test TorsiFlex.

From the experimental point of view, the studies by modern techniques as the laser ablation molecular-beam Fourier transform microwave (LA-MB-FTMW) carried out at cryogenic temperatures are contributing to reveal the intrinsic properties of pAAs in the absence of an interacting environment [38–47].

From a theoretical point of view, conformational studies on individual pAAs are extensive in literature, especially in the gas phase where their canonical (neutral uncharged) form prevails [48–72]. There are also studies that considered amino acids in their zwitterionic, cationic or anionic forms (the prevailing forms in aqueous solution). For example, Turan and Selçuki [69] studied glutamic acid (Glu) at the B3LYP/cc-pVTZ level and reported a total of 165 conformers for its canonical form, 63 for the zwitterionic forms, 199 for the anionic forms, 24 for the dianionic and 135 for the cationic form. However, the study of individual compounds prevents a fair comparison between amino acids due to the fact that each of them was analyzed at different electronic structure levels and by different searching algorithms.

Theoretical works that considered the twenty pAAs are scarce; Jamróz et al. [70] reported a total of 1 684 canonical conformers on their study about chirality measures of $\alpha$-amino acids. More recently, Ropo et al. [71, 72] studied all proteinogenic amino acids at the PBE generalized-gradient exchange-correlation functional [73] with *tier2* basis set and with corrections for van der Waals interactions. Notably, their search was not restricted to the arrangements arising from chemically intuitive dihedral angles, a common practice in conformational studies. Their dataset contains conformers for the twenty amino acids in their uncharged and zwitterionic forms, in addition to including dipeptides and the interaction with several divalent cations. Regarding the canonical forms, their database for the 20 pAAs contains a total of 3 315 geometries, which is twice the number of conformers reported by Jamróz et al. a clear indication of the conformational richness accessible to the pAAs in the gaseous phase. In order to exemplify the effectiveness and capabilities of TorsiFlex, we have searched for *all* conformers of the 20 pAAs in their canonical form and the results are compared with these previous works.

The manuscript is organized as follows:  "Computational details" Section briefly describes the technicalities associated with the electronic structure calculations, "Algorithm and implementation" Section introduces TorsiFlex and analyzes the algorithm implemented in it, "Results and discussion" Section presents the results for the pAAs establishing a comparison with previous studies, and "Conclusions" Section summarizes the main findings of this work.

## Computational details

TorsiFlex is a program written in Python 3 and includes a completely automated interface with *Gaussian* (versions *09* [74] and *16* [75]) and the two programs were employed for the location of the conformers of the twenty pAAs. The LL calculations involve geometry optimizations and Hessian calculations at the optimized geometries to ensure that the converged structure corresponds to an equilibrium geometry. TorsiFlex can function with any of the MM, semiempirical, Density Functional Theory (DFT), or *ab initio* methods implemented in *Gaussian*. Our recommendation is to employ the split-valence 3-21G basis set [76, 77] together with the Hartree-Fock (HF) method, since this basis set is the smallest of its kind but provides reasonable geometries and energies. It is well known that HF, due to the lack of electron correlation, increases the barrier heights between conformers (an effect that becomes more acute with large basis sets, as for intance 6-31G*) but HF/3-21G tends to correct that effect by error compensation. Another important aspect is that HF/3-21G is superior to the traditional semiempirical methods when accounting for hydrogen bond interactions [78]. This is an important aspect because in molecules presenting -OH and -$\hbox {NH}_2$ groups, some of the most stable conformers may contain strong hydrogen bonds. More recent semiempirical [79] and tight-binding methods [80] have improved this and other aspects by which they traditionally failed, so they are also viable options as LL methods. However, in a recent work, Mancini et al. [11] tested a few semiempirical methods (including PM7 [79]) against a reference set of data on threonine (Thr) [63] and none of the semiempirical methods was able to completely retrieve the conformers of the reference study. In this aspect, the HF/3-21G level produced 68 conformers for Thr versus the 56 HL conformers of the reference data.

It is noteworthy that HF methods tend to generate more minima than electron correlation methods but the spurious minima disappear at the HL optimization. However, adopting HF as the LL method forced us to find efficient sampling strategies that accomplish the challenge of exploring the PES within tractable limits. In TorsiFlex, this involved the design of a battery of tests that avoid ill-generated geometries and different types of redundancies, as detailed in the next Section.

The HL calculations, that also require geometry optimizations starting from the converged LL structures and Hessian calculation over the final geometries, were performed at the M08-HX functional [81] in combination with the MG3S [82] basis set (same as MG3 [83] but with improvements for molecules containing sulfur atoms). This global-hybrid-meta-GGA functional has been specially designed for thermochemistry, kinetics, and noncovalent interactions [84–86]. For the evaluation of the partition functions, all frequencies were scaled by the recommended scale factor of 0.973 [87].

Additionally, a comparison of the current M08-HX/MG3S calculations for the eleven most stable conformers of cysteine (Cys) against the benchmark CCSD(T) energies carried out by Wilke et al. [62] who also employed the HF/3-21G method as LL searching method, shows that the mean unsigned error (MUE) of M08-HX is only 0.25 kcal/mol, whereas B3LYP//aug-cc-pVTZ yields a MUE of 0.36 kcal/mol.

Our final dataset contains a total of 6 508 confirmed minima obtained at the M08-HX/MG3s level. This information is available in GitHub [88] and as Additional file 1 accompanying this work. Additionally, this file contains the zero-point energy, rotational constants, and dipole moments of each conformer.

## Algorithm and Implementation

**Fig. 1:**
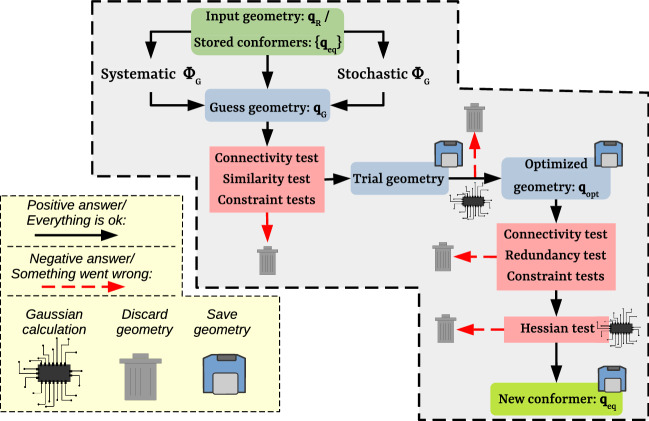
Flowchart for the LL conformational search algorithm as implemented in TorsiFlex

Figure 1 schematically illustrates the design of the search algorithm implemented in TorsiFlex. The algorithm requires an initial geometry to work with, termed the reference (or input) geometry ("Reference geometry and connectivity" Section). This geometry is the working framework to generate structures (Generation of geometries for sampling" Section) along the configurational space. However, they are validated through a series of tests ("Validation tests for the initial geometries" Section) before proceeding with the electronic structure calculations. The geometries that fail the tests are discarded, whereas those that pass them turn into trial structures that are optimized. In this manner, TorsiFlex avoids unnecessary calculations, accelerating the search process. If the optimization succeeds, the resulting geometry is treated as a potential conformer, condition that is verified by submitting the structure to a new set of tests ("Validation of the optimized geometry" Section). TorsiFlex also accounts for amine inversion ("Umbrella inversion of the NH_2_ group" Section), and conformational enantiomerism ("Conformational enantiomers" Section). Finally, the parallelization of the search algorithm ("Parallelization within the search algorithm" Section) and the dual-level method ("Dual-level approach" Section) are also discussed.

### Reference geometry and connectivity

TorsiFlex reads the reference geometry in internal coordinates, $\mathbf{q }_{\mathrm{R}}$, employing the Z-matrix format.^1^ These internal coordinates should explicitly include the *K* proper torsions of interest univocally defined, i.e. the torsion about a given single bond can only be defined once.^2^ The reference geometry, in addition to its role in the generation of the first candidates in the search process, defines the molecular connectivity of the system, represented by its adjacency (or connectivity) matrix, $\mathbf{A }_{\mathrm{R}}$. This square matrix is employed to build finite graphs and its elements reveal whether pairs of nodes (atoms) are adjacent (bonded) or nonadjacent (not bonded) in the graph (molecule). For a system with *N* atoms, $\mathbf{A }_{\mathrm{R}}$ is a ${N}\times {N}$ symmetric square matrix where each element is given by:

1 $$\begin{aligned} (\mathbf{A }_{\mathrm{R}})_{ij} =\left\{ \begin{matrix} 1 &{} \text{ if } \text{ bonded } \text{ and } i \ne j \\ 0 &{} \text{ otherwise } \end{matrix} \right. \end{aligned}$$

**Fig. 2:**
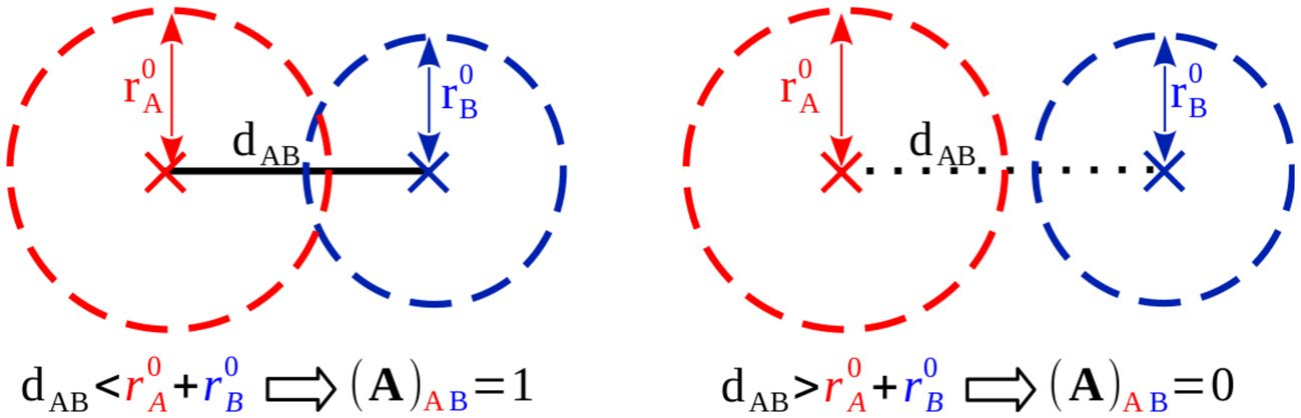
Bonding criterium for a pair of atoms, A and B

TorsiFlex considers a pair of atoms (A, B) to be bonded when the distance between them, $d_{AB}$, is smaller than the sum of their corresponding covalent radii, $r_{A}^{0}$ and $r_{B}^{0}$,

2 $$\begin{aligned} d_{AB} < (r_{A}^{0} + r_{B}^{0}) \end{aligned}$$

as illustrated in Fig. 2. In particular, TorsiFlex applies the criterium of Eq. 2, but considering a scale factor, $f_{\mathrm{c}}$, that facilitates the control of this connectivity by the user. Specifically, the program assumes that a pair of atoms (A,B) is bonded when:

3 $$\begin{aligned} d_{AB} < f_{\mathrm{c}} \cdot (r_{A}^{0} + r_{B}^{0}) \end{aligned}$$

In this inequality, a larger $f_{\mathrm{c}}$ value increases the probability for two atoms to be considered as bonded.

**Fig. 3:**
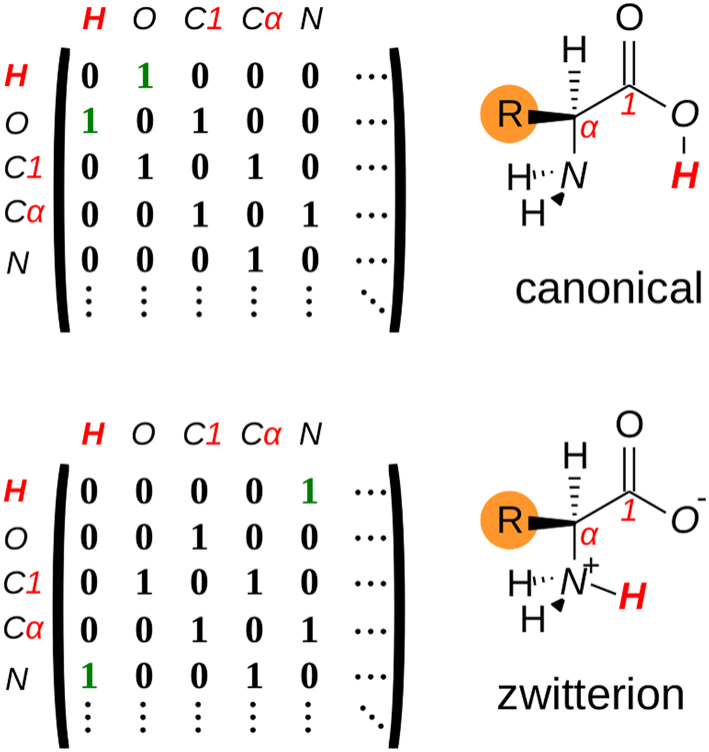
Adjacency matrices of the canonical and zwitterion forms for a general amino acid. The numbering of carbon atoms follows the IUPAC & IUB recommendation [90]

The adjacency matrix avoids the optimization of highly unstable geometries and discriminates between constitutional isomers (i.e. structures with different bonding patterns) [89]. An example of this type of isomerism can be found in the canonical and zwitterionic forms of general amino acids, both represented by different adjacency matrix (see Fig. 3).

A similar situation may appear if two atoms are close enough to be spuriously considered as bonded by Eq. 3, but such undesired arrangements are also discarded by applying this connectivity test.

### Generation of geometries for sampling

TorsiFlex builds a *K*-dimensional torsional vector, $\varvec{\Phi }^{\mathrm{R}}=(\phi _1,...,\phi _i,...,\phi _{\mathrm{K}})$ from the target torsions specified in the reference geometry. Therefore, this vector is contained in the internal coordinates definition of the Z-matrix:

4 $$\begin{aligned} \varvec{\Phi }^{\mathrm{R}} \subset \mathbf{q }_{\mathrm{R}} \end{aligned}$$

and its replacement by a vector with guessed dihedral angles, $\varvec{\Phi }^{\mathrm{G}}$, renders an initial geometry, $\mathbf{q }_{\mathrm{G}}$ (i.e. the dihedral angles in the reference Z-matrix are modified). In mathematical form:

5 $$\begin{aligned} \mathbf{q }_{\mathrm{G}} = (\mathbf{q }_{\mathrm{R}} ~\backslash ~ \varvec{\Phi }^{\mathrm{R}}) \cup \varvec{\Phi }^{\mathrm{G}} \end{aligned}$$

In practice, TorsiFlex operates with the structural parameters of the previously optimized geometries instead of resorting to the reference Z-matrix. Thus, the Z-matrix of the new structure is generated from that of the closest optimized conformer but substituting the dihedral angles of the target torsions by the new guessed ones. This strategy pursues two goals: (i) the improvement of the starting geometry by employing more suitable bond lengths, bond angles, and improper torsions than those of the reference geometry, and (ii) the speed-up of geometry optimizations. Tradicionally, many algorithms based on systematic searches employ exclusively the reference geometry in the generation of new structures, an approach sometimes referred as ‘rigid rotor’ approximation [18]. However, this update of the geometric parameters gains relevance as the size of the torsional vector increases. Otherwise, we run the risk of leaving important parts of the potential energy surface unexplored, since the reference values of the $3N-K-6$ degrees of freedom (*N* is the number of atoms) may be substantially far from the equilibrium configuration that matches a particular torsional vector.

**Fig. 4:**
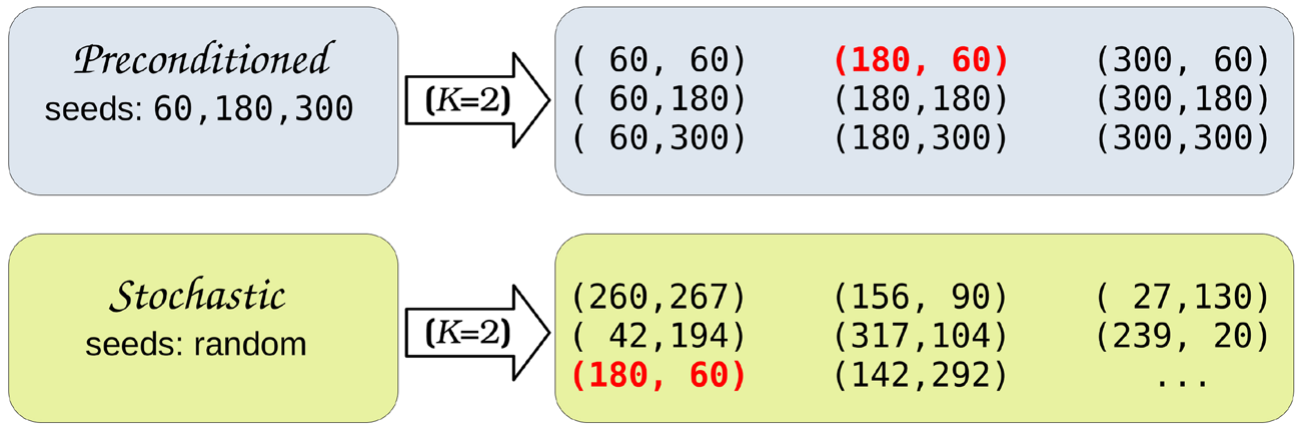
Preconditioned and stochastic generation of torsional vectors for a system with two torsions ($K=2$). Seeds in the preconditioned structures correspond to the two *gauche* and *anti* conformations. The bold-red vector that appears in the preconditioned and stochastic searches is skipped in the latter. Ellipsis in the stochastic algorithm indicates that we can generate as many guesses as desired

The vectors employed as initial guesses, $\varvec{\Phi }^{\mathrm{G}}$, can be generated in two different fashions: preconditionally or stochastically (see Fig. 4). The former is based on chemically intuitive dihedral angles as, for example, the expected *gauche* (60$^\circ$ and 300$^\circ$) and *anti* (180$^\circ$) configurations in *sp*$^3$ carbon atom chains or the *syn* (0$^\circ$) and *anti* (180$^\circ$) arrangements of carboxylic acids, respectively. The latter is based on the random generation of torsional angles and may lead to values already sampled, as indicated in Fig. 4. A similarity test needs to be performed before starting the optimization of the geometry to avoid this redundancy.

### Validation tests for the initial geometries

**Fig. 5:**
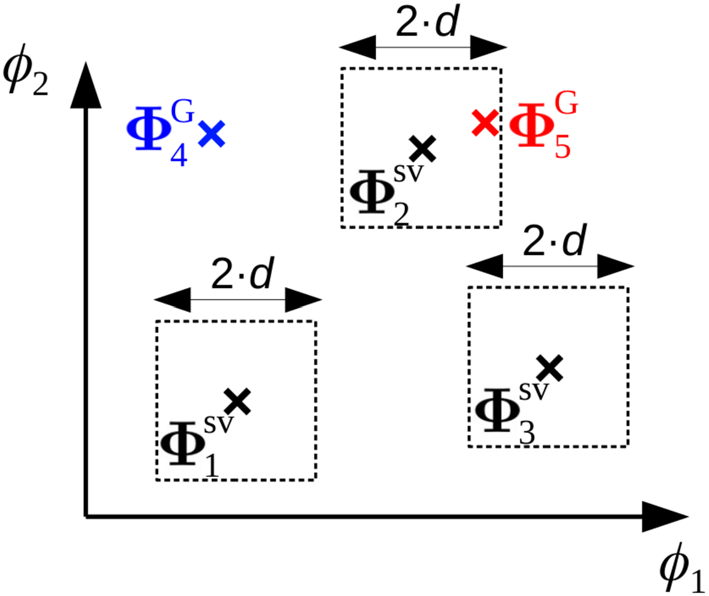
Scheme for the **similarity test** in a system with $K=2$. The test results positive for vector $\varvec{\Phi }^{\mathrm{G}}_{4}$. On the contrary, the test is negative for the fifth guess point, $\varvec{\Phi }^{\mathrm{G}}_{5}$, because it is inside the domain of the second saved point, $\varvec{\Phi }^{\mathrm{sv}}_{2}$

The initial geometry generated from a preconditioned or a stochastic seed, $\mathbf{q }_{\mathrm{G}}$, needs to be geometrically optimized at LL by an electronic structure program. If this optimization succeeds, the Hessian matrix of the optimized geometry, $\mathbf{q }_{\mathrm{opt}}$, must be calculated to assert that we are indeed dealing with an equilibrium structure. These two steps should be carried out only if necessary and, for this reason, the initial geometries must overcome the series of validation tests listed below:

- **Connectivity test**. The initial geometry should contain the same adjacency matrix as the reference geometry. Thus, if $\mathbf{A }_{\mathrm{G}}$ is the adjacency matrix associated with $\mathbf{q }_{\mathrm{G}}$, this test is positive if:
  6 $$\begin{aligned} \mathbf{A }_{\mathrm{R}} = \mathbf{A }_{\mathrm{G}} \end{aligned}$$
- **Similarity test**. The torsional vector of the generated structure, $\varvec{\Phi }^{\mathrm{G}}$, is compared with a pool of saved torsional vectors from previous iterations, $\{\varvec{\Phi }^{\mathrm{sv}}\}$; if $\varvec{\Phi }^{\mathrm{G}}$ falls outside of the domain *d* that surrounds each stored vector, then the test is positive (see Fig. 5). Mathematically, this condition can be written as:
  7 $$\begin{aligned} \forall ~p, ~~ \exists ~\tau : |(\varvec{\Phi }^{\mathrm{G}})_{\tau } - (\varvec{\Phi }^{\mathrm{sv}}_{p})_{\tau }| > d \end{aligned}$$
  where *p* and $\tau$ run over all saved points and over each target torsion, respectively. The domain associated with the stored values is provided by the user and by default is $d = 15^\circ$.
- **Constraints test**. For different reasons, the user may be interested in a set of conformers presenting certain characteristics. For example, the conformers with the hydrogen of the carboxylic group (dihedral O=C1-O-H) in *anti* position (dihedral angle in the 150$^\circ$-210$^\circ$ interval), and with the distance between the O-H and the N atom of the amino group smaller than 2.5 Å. For such situations, TorsiFlex accepts the definition of constraints based on the distance between a pair of atoms, the angle between a triad of atoms and the dihedral angle between four atoms. Specifically, TorsiFlex differenciates between two types of constraints:
  - *Hard* constraints: the test is positive if all of the defined constraints are fulfilled.
  - *Soft* constraints: the tests is positive if at least one of the defined constraints is fulfilled.

If any of the previous tests delivers a negative result, TorsiFlex discards $\mathbf{q }_{\mathrm{G}}$. Therefore, LL optimizations that could lead to constitutional isomers, previously located conformers, or undesired conformers, are skipped, speeding up the searching process. The initial geometry that passes all tests turns into a trial geometry, ready to be optimized.

### Validation of the optimized geometry

Optimum performance of the search algorithm is achieved when the preconditioned generation of structures precedes the stochastic one for two reasons. Firstly, structure optimizations from preconditioned seeds rarely fail and, secondly, initial geometries with different preconditioned seeds infrequently lead to the same conformer. Geometries built from random seeds have the disadvantage that are alien to any chemical characteristic of the system and should be our second choice. However, they are crucial to improve the sampling of the PES, mainly to access conformers that involve chemical interactions that went unnoticed in the first screening.

Beyond the optimum performance of the algorithm, the aforementioned order also enables estimating the importance of the chemically intuitive conformers. Particularly, TorsiFlex classifies the located conformers depending on whether they were obtained from preconditioned or stochastic seeds. Such classification can be used to estimate the importance of the chemically-intuitive conformers, associated with the preconditioned search.

Once a trial geometry is optimized, the resulting geometry, $\mathbf{q }_{\mathrm{opt}}$, is available. In order to assert if $\mathbf{q }_{\mathrm{opt}}$ corresponds to a new conformer, a second set of tests is sequentially executed:

- **Connectivity test**. It ensures that the optimized geometry still represents the original constitutional isomer by comparing its adjacency matrix $\mathbf{A }_{\mathrm{opt}}$ with the reference one:
  8 $$\begin{aligned} \mathbf{A }_{\mathrm{R}} = \mathbf{A }_{\mathrm{opt}} \end{aligned}$$
- **Redundancy test**. It compares the optimized torsion vector, $\varvec{\Phi }^{\mathrm{opt}}$, against the pool of saved equilibrium structures, $\{\varvec{\Phi }^{\mathrm{eq}}\}$. If $\varvec{\Phi }^{\mathrm{opt}}$ is missing from $\{\varvec{\Phi }^{\mathrm{eq}}\}$, i.e., if $\varvec{\Phi }^{\mathrm{opt}} \not \subset \{\varvec{\Phi }^{\mathrm{eq}}\}$, the test is positive and the optimized geometry is a new conformer incorporated into $\{\varvec{\Phi }^{\mathrm{eq}}\}$. However, to account for numerical errors, this test is considered positive when:
  9 $$\begin{aligned} \forall ~p, ~~ \exists ~\tau : |(\varvec{\Phi }^{\mathrm{opt}})_{\tau } - (\varvec{\Phi }^{\mathrm{eq}}_{p})_{\tau }| > \epsilon \end{aligned}$$
  with $\epsilon$ being not greater than 2 degrees. This test is essentially equivalent to the **similarity test**, but it is controlled by a different threshold ($\epsilon < d$) and the comparison is against the located conformers instead of all the previously saved vectors.
- **Constraints test**. TorsiFlex verifies the hard and soft constraints also on the optimized geometry.
- **Hessian test**. It inspects the vibrational frequencies resulting from the diagonalization of the Hessian matrix of the LL optimized geometry. If all vibrational frequencies are real, the test is positive.^3^

If any of the first three tests is negative, $\mathbf{q }_{\mathrm{opt}}$ is automatically discarded and no computer time is wasted carrying out the Hessian test. The validation of initial geometries is only carried out at the LL, whereas the validation of the optimized geometries is performed at both, LL and HL.

Finally, we highlight that along the search process (Fig. 1), all torsional vectors associated with the initial and optimized geometries are saved in $\{\varvec{\Phi }^{\mathrm{sv}}\}$. This storage, combined with the similarity test, prevents visiting the same region of the torsional space twice when performing the stochastic sampling.

### Umbrella inversion of the $\hbox {NH}_2$ group

**Fig. 6:**
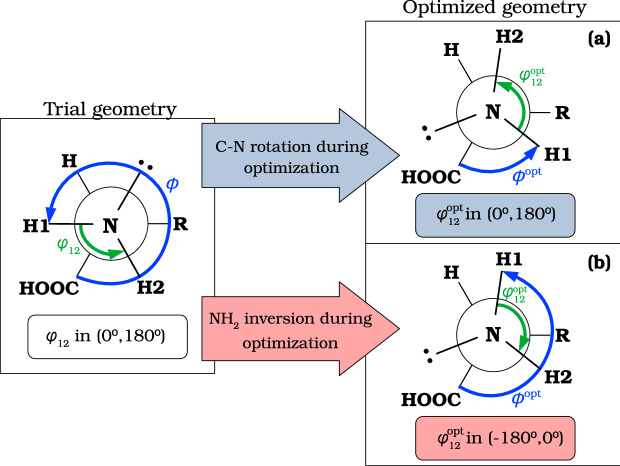
Umbrella inversion motion of $\hbox {NH}_2$ belonging to a generic amino acid backbone. The torsional angle $\phi$ = C1-$\hbox {C}_\alpha$-N-H1 differs in structures** a** and **b** but the structure is the same. The improper torsion $\varphi _{12}$ of structure **b** changed sign during the optimization in such a way that $\varphi _{12}^{\mathrm{opt,(a)}}=-\varphi _{12}^{\mathrm{opt,(b)}}$. Therefore structure **b** can be transformed into structure **a** by exchanging H1 and H2

The umbrella inversion, an internal motion commonly ascribed to the amino group, may interfere with the internal rotation, as both motions present low barriers. The effect of this inversion is illustrated in Fig. 6, showing that the optimization of the trial geometry of a general amino acid may follow two different paths. Structure (**a**) arises from the internal rotation about the $\hbox {C}_\alpha$-N bond, whereas structure **b** is obtained as a result of umbrella inversion motion. Both structures correspond to the same conformer but the dihedral angle that describes the proper torsion about the $\hbox {C}_\alpha$-N group, $\phi$= C1-$\hbox {C}_\alpha$-N-H1, differs from **a** to **b**. Therefore, the optimized value of $\phi$, without any additional information, provides an ambiguous description of the $\hbox {NH}_2$ internal motion.

For this reason, TorsiFlex automatically monitors the $\varphi _{12}$= H1-H2-$\hbox {C}_\alpha$-N improper torsion that changes sign upon umbrella inversion.^4^ Specifically, TorsiFlex compares the improper torsion at both, the trial $\varphi _{12}$ and the optimized $\varphi _{12}^{\mathrm{opt}}$ structures: if $\mathrm{sgn}(\varphi _{12})=\mathrm{sgn}(\varphi _{12}^{\mathrm{opt}})$ then no inversion occurred [structure **a** of Fig. 6], if $\mathrm{sgn}(\varphi _{12})\ne \mathrm{sgn}(\varphi _{12}^{\mathrm{opt}})$ then the inversion took place during one optimization step [structure **b** of Fig. 6], and TorsiFlex exchanges the H1 and H2 hydrogen atoms. In summary, $\phi$ can be used as the torsional target in the amino group but only after verifying that $\varphi _{12}$ preserves its sign in the optimized geometries.

### Conformational enantiomers

Some flexible molecules have a plane of symmetry for specific values of their dihedral angles; such molecules exhibit conformational enantiomerism. In the case of pAAs, only glycine (Gly) possesses this type of enantiomers, due to the absence of asymmetric carbon atoms. As a consequence of the plane of symmetry, only half of the torsional space needs to be explored.

For a given geometry defined by the torsional vector, $\varvec{\Phi }=(\phi _1,...,\phi _i,...,\phi _{\mathrm{K}})$, TorsiFlex retrieves the vector associated with the conformational enantiomer, $\varvec{\Phi }^{*}=(\phi _1^*,...,\phi _i^*,...,\phi _{\mathrm{K}}^*)$, and stores it together with $\varvec{\Phi }$. The similarity and redundancy tests take advantage of this double storage, reducing the computer time. The torsional angles of the enantiomer can be calculated considering that for the configuration of $C_s$ symmetry, there are target torsions: (i) with the 4 atoms lying in the plane of symmetry or (ii) with one or more atoms out of the plane. In case (i), for a given structure with torsion $\phi _i$, the torsional angle of the enantioner is simply

10 $$\begin{aligned} \phi _i^* = -\phi _i \end{aligned}$$

In case (ii), the out-of-plane atoms have replicas at the other side of the plane, and in addition to the dihedral angle of the four atoms, $\phi _{i}$, we need to know the dihedral angle ${\bar{\phi }}_{i}$ between the atoms that are identical under reflection. The relation is:

11 $$\begin{aligned} \phi _i^* = -{\bar{\phi }}_{i};~~~~~{\bar{\phi }}_{i}^* = -\phi _{i} \end{aligned}$$

**Fig. 7:**
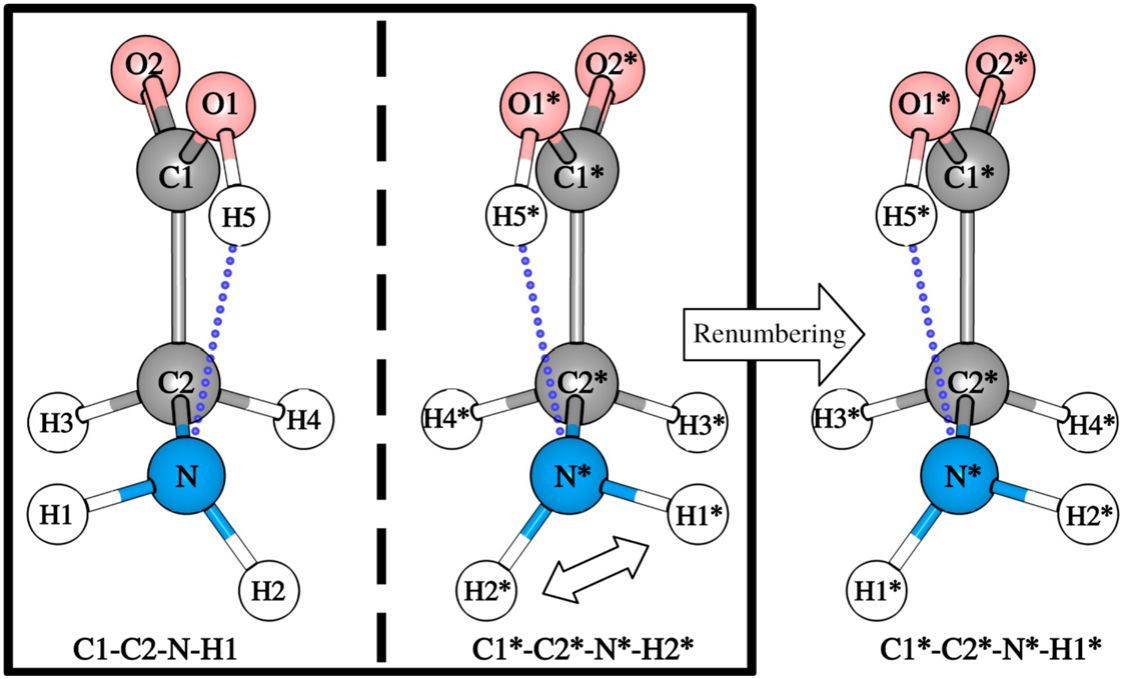
Enantiomer generation and renumbering in glycine. Notice that C2 is the $\hbox {C}_\alpha$ carbon.

For the case of Gly displayed in Figure 7, the target torsional angles are $\varvec{\Phi }= (\phi _1,\phi _2,\phi _3)$, where $\phi _1$=H5-O1-C1-C2, $\phi _2$=O1-C1-C2-N, $\phi _3$=C1-C2-N-H1, and ${\bar{\phi }}_3$=C1-C2-N-H2. With the rules of Eqs. 10 and 11, the torsional set of dihedrals of the enantiomer corresponds to $\varvec{\Phi }^{*}=(-\phi _1,-\phi _2,-{\bar{\phi }}_3)$.

In practice, for a given optimized structure TorsiFlex generates the enantiomer by projecting the geometry onto the *YZ* plane, i.e, by changing the sign of the *x* Cartesian coordinate of every atom. The reflection on the plane generates a non-superimposable structure (that cannot be obtained by internal rotations) with respect to the original structure, as shown in Fig. 7. However, conformational enantiomers can interconvert between them, and some atoms of the reflected structure need to be renumbered to meet this condition.

TorsiFlex renumbers the atoms in three steps:

1. Correlation of atoms of the same type. For example, H1$^*$ can only be correlated to H atoms in the original structure, i.e. H1 to H5. In this step, the N atom is automatically correlated.
2. Inspection of the connectivity. As H1$^*$ is bonded to the nitrogen atom, it can only correlate to H1 and H2. In this step, TorsiFlex also correlates (univocally) C1$^{*}$, C2$^{*}$, O1$^{*}$, O2$^{*}$ and H5$^{*}$.
3. Inspection of the spatial distribution through improper torsions. The dihedral angle associated with the improper torsion $\varphi _{12}^*$=H1$^{*}$-H2$^{*}$-N$^{*}$-C2$^{*}$ is compared against the $\varphi _{12}$=H1-H2-N-C2, and $\varphi _{21}$=H2-H1-N-C2 dihedral angles of the original structure. The improper torsional angles have the following property,
  12 $$\begin{aligned} \varphi _{12} = -\varphi _{21}, \end{aligned}$$
  but are invariant under reflection. Therefore, the correlation between improper torsions is $\varphi _{12} = \varphi _{21}^*$ and $\varphi _{21} = \varphi _{12}^*$. This relation allows an unambiguous assignment of H2$^*$ to H1 and H1$^*$ to H2. Finally, H2$^*$ and H1$^*$ are renumbered as H1$^*$ and H2$^*$, respectively, to preserve the same numbering as in the original structure. The same procedure can be applied to H3 and H4.

After the correct assignment of all atoms of the enantiomer, $\varvec{\Phi }^{*}$ is readily calculated. Notice that the above procedure reduces by half the computational effort for molecules presenting conformational enantiomerism.

### Parallelization within the search algorithm

The previous algorithm for systems with several torsions may be computationally expensive when resorting to LL ab initio methods (or even semi-empirical methods, depending on the number of torsions). TorsiFlex allows different batches to be executed at the same time, achieving in this manner an artificial 100% parallelization.

The standard execution for a preconditioned search is:

python3 torsiflex.py --prec

which sequentially generates the initial geometries. However, this procedure may be too slow in systems with $K>3$. In order to speed up the search, TorsiFlex can split the set of seeds into *M* groups $\{n_1,n_2,\ldots ,n_{\mathrm{m}},\ldots ,n_{\mathrm{M}}\}$ and deal with each of them separately. TorsiFlex can manage the *m*-th group ($m\le M$) by:

python3 torsiflex.py --prec M m

For example, to divide the seeds into M=10 groups and to perform calculations on the elements $n_2$ (m=2) the command line is:

python3 torsiflex.py –-prec 10 2

Regarding the stochastic search, each execution generates random seeds, and therefore, different batches can be carried out simultaneously. The number of seeds generated in each execution within the stochastic search flow can be controlled in the input file.

### Dual-level approach

The dual-level approach consists on performing more accurate HL electronic structure calculations employing as trial the set of structures of the already known LL equilibrium geometries. As for the LL case, each HL optimized geometry must overcome the validation tests ("Validation of the optimized geometry" Section) to be stored as a new conformer of the system. The dual-level method is a double-edged sword, a claim that can be extended to all methods that rely on PES that are sampled at LL. On the one hand, it plays a key role in accelerating the search process but, on the other hand, it may miss conformers existing in the HL PES. For this reason, it is of capital importance to select a LL torsional PES characterized by a similar topology than that of the HL torsional PES.

We have found that HF/3-21G calculations are a good compromise for this case. This method is fast enough and tends to produce more minima than electronic correlated methods. Unfortunately, the size of the molecular system or the availability of computational resources can render even HF unfeasible. In such cases, MM or semiempirical methods may be the only choice. However, even in the most favorable situation, it is almost impossible to assure that a given LL PES will completely map the HL PES without loss of conformers.

## Results and discussion

This section summarizes the results for the twenty proteinogenic amino acids. Firstly, we compare our results with previous works. Secondly, we briefly analyze the conformational hierarchy and the preferred conformations of the twenty pAAs. Finally, we discuss the importance of the stochastic search.

### Comparison with previous works

Table 1 contains the number of conformers located for each pAA using TorsiFlex, including both the number of conformers located in the LL search (7 665 in total) and the resulting conformers after the HL refinement (6 508). We notice that around 85% of the LL conformers led to a new conformer in the M08-HX/MG3S PES. These results do not correlate completely due to the HF tendency of producing more minima than electronic correlated methods.

**Table 1:** Number of canonical conformers obtained for the 20 pAAs employing TorsiFlex

Amino acid	HF	DFT	Number of conformers [Ref.]
Asp	169	127	139 [58], 37 [70], 76 [71]
Glu	513	415	143 [70], 165 [69], 197 [71]
Ala	11	11	10 [49], 13 [50], 9 [70], 15 [71]
Gly	12	9	8 [60, 71]
Ile	114	95	12 [65], 59 [70], 76 [71]
Leu	130	95	8 [65], 53 [70], 85 [71]
Pro	20	16	18 [52], 3 [70], 22 [71]
Val	35	30	6 [65], 19 [70], 22 [71]
Asn	91	64	62 [55], 23 [70], 49 [71]
Gln	271	181	72 [70], 143 [68], 134 [71]
Phe	41	35	37 [57], 20 [70], 25 [71]
Trp	95	76	45 [53], 37 [70], 57 [71]
Tyr	80	65	76 [54], 38 [70], 43 [71]
Arg	3 199	2 811	520 [70], 1 218 [71]
HisD	76	60	10 [64], 57 [71]
HisE	93	70	15 [64], 47 [71]
Lys	2 224	1 944	927 [61], 391 [70], 733 [71]
Ser	73	64	51 [49], 74 [66], 30 [70], 59 [71]
Thr	68	58	56 [63], 65 [66], 29 [70], 47 [71]
Cys	79	66	42 [49], 71 [62], 87 [66], 50 [70], 99 [71]
Met	271	216	27 [48], 113 [70], 246 [71]

Second and third columns list the number of conformers obtained at the LL and HL, respectively. The last row indicates the number of conformers obtained in previous works with the reference between square brackets. Two histidine tautomers, HisD, and HisE have been considered (also referred in literature as His[N$^\tau$H] and His[N$^\pi$H], respectively). HF and DFT refer to the HF/3-21G and M08-HX/MG3S levels, respectively

The most complete work on conformations of pAAs has been carried out by Ropo et al. [71], but their numbers do not correspond exclusively to conformers (minima), but rather to stationary points in the PES, as they pointed out in their work: *“The present data contains stationary-point geometries (mainly minima, but also saddle points since no routine normal-mode analysis was performed) on the potential energy surface of the 20 proteinogenic amino acids”* [71]. In this sense, their numbers are an upper limit to the total number of conformers. For example, they have found a total of 246 stationary points for methionine (Met), whereas we have located 216 conformers; this difference cannot be associated with a poor performance of TorsiFlex for this system, as the percentage of minima in their geometries is unknown. Nevertheless, we should remark that the total number of HL conformers found with TorsiFlex (6 508) surpasses their number of stationary points (3 315) by roughly a factor of two.

Regarding other works, the number of conformers located with TorsiFlex in the LL search is, in general, greater than numbers previously reported for the twenty amino acids. This result still holds when we consider the number of conformers after the HL refinement with M08-HX/MG3S, with the exception of seven amino acids: Asp (127 vs. 139 [58]), Ala (11 vs. 13 [50], Pro (16 vs. 18 [52]), Phe (35 vs. 37 [57]), Ser (73 vs 74 [66]), Cys (66 vs. 87 [66]), and Tyr (65 vs. 76 [54]), although the difference in five of them is very small and may be attributed to the difference in the electronic structure calculations. It is surprising the case of Cys with 87 conformers, since Wilke et al. [62] found 71 conformers, a number which is very close to the 66 conformers located in this study. For the remaining amino acids the variation in the number of reported conformers favors TorsiFlex, and in the cases of Glu, Gln, Arg, HisE, and Lys the difference is substantial.

### Classification of the conformers

**Fig. 8:**
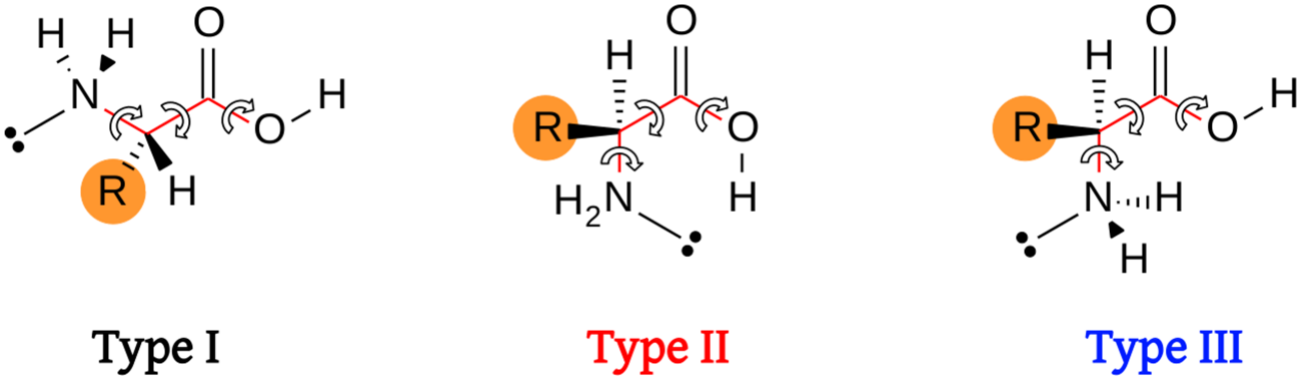
Classification of amino acids backbones. The torsional bonds that describe types I, II, and III are highlighted in red

We have selected the three torsions highlighted in Fig. 8 to classify the amino acids based on the backbone configurations, following the same nomenclature as in Ref. [39], that is, H-O-$\hbox {C}_1$-$\hbox {C}_\alpha$ ($\phi _1$), O-$\hbox {C}_1$-$\hbox {C}_\alpha$-N ($\phi _2$) and $\hbox {C}_1$-$\hbox {C}_\alpha$-N-*lp* ($\phi _3$), *lp* denoting the position of the N lone pair. According to these arrangements, we define the following types based on a subvector of $\varvec{\Phi }^{\mathrm{eq}}$ given by the dihedrals $(\phi _1,\phi _2,\phi _3)$:

- **Type I**. It is characterized by the reference $(\phi _1^{\mathrm{R}},\phi _2^{\mathrm{R}},\phi _3^{\mathrm{R}}) = (x,180,180)$, where *x* can take any value in the [0,360) interval; in this configuration, the hydrogen(s) of the amino group is(are) directed towards the carbonylic oxygen.
- **Type II**. It is specified by the reference $(\phi _1^{\mathrm{R}},\phi _2^{\mathrm{R}},\phi _3^{\mathrm{R}}) = (0,0,0)$; in this arrangement, the hydrogen of the carboxylic acid is directed towards the nitrogen lone pair.
- **Type III**. It is associated with the reference $(\phi _1^{\mathrm{R}},\phi _2^{\mathrm{R}},\phi _3^{\mathrm{R}}) = (180,0,180)$; the hydrogen(s) of the amino group is(are) directed towards the hydroxy group, and the hydrogen of the carboxylic acid is in *syn* configuration.
- **Type IV**. It corresponds to any configuration far from the three types.

The criterion to classify the conformers is based on the Euclidean distance calculated from the reference $(\phi _1^{\mathrm{R}},\phi _2^{\mathrm{R}},\phi _3^{\mathrm{R}})$ dihedral angles as:

13 $$\begin{aligned} d_{\mathrm{{J}}} = \sqrt{\sum _{i=1}^{3} \texttt {min} \left( \texttt {mod}(\phi _i-\phi _{i,\mathrm{J}}^{\mathrm{R}},360) , \texttt {mod}(\phi _{i,\mathrm{J}}^{\mathrm{R}}-\phi _i,360) \right) ^2} \end{aligned}$$

where $\phi _{i,\mathrm{J}}^{\mathrm{R}}$ denotes the reference dihedral angle for the *i*-th torsion in the J-th type.^5^ Notice that the mod operation and the min function are compulsory due to the fact that angles $\phi$ and $\phi \pm 2\pi n$, with $n \in {\mathbb {N}}$, are equivalent. Thus, the conformer is assigned to the type with the smallest distance. However, if the three $d_{\mathrm{{J}}}$ values are greater than a given threshold, the conformer is classified as type IV. In this work we have set this threshold to 75 degrees.

**Fig. 9:**
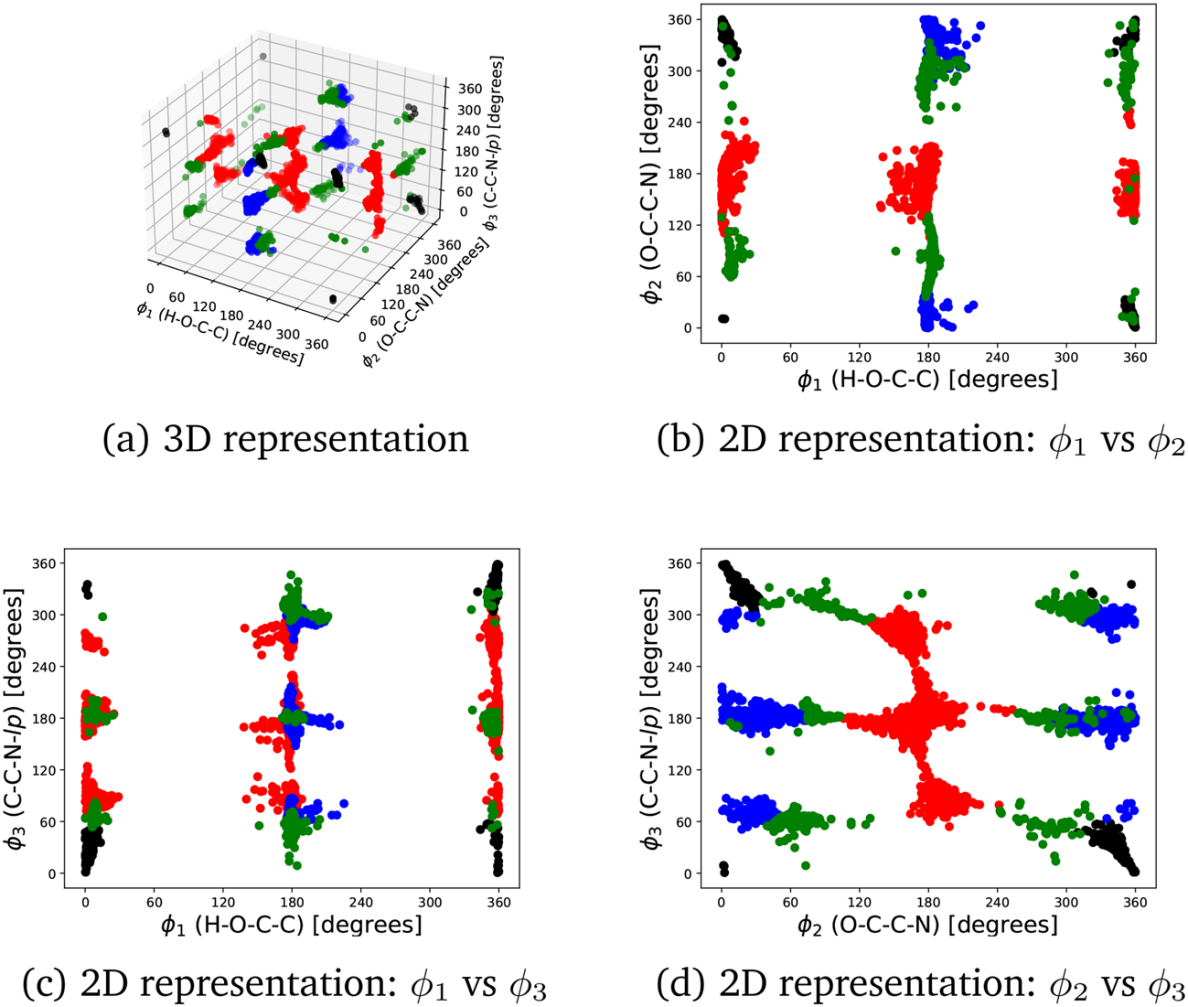
Distribution of conformers according to the backbone type of Fig. 8. Dihedral angles $\phi _1$, $\phi _2$ and $\phi _3$ correspond to the H-O-$\hbox {C}_1$-$\hbox {C}_\alpha$, O-$\hbox {C}_1$-$\hbox {C}_\alpha$-N and $\hbox {C}_1$-$\hbox {C}_\alpha$-N-*lp* torsions, respectively (*lp* denotes the position of the N lone pair). Black, red, blue and green colors identify the I, II, III and IV types, respectively

The distribution of the previous three angles for all the conformers is shown in Fig. 9, together with the corresponding type classification. As expected, conformers in amino acids are not uniformly distributed among the torsional space, although this behavior is less noticeable for the $\phi _2$ torsion. We have encountered a total of 2 794 conformers of type I, 977 of type II, 1 680 of type III, and 1 057 of type IV.

**Fig. 10:**
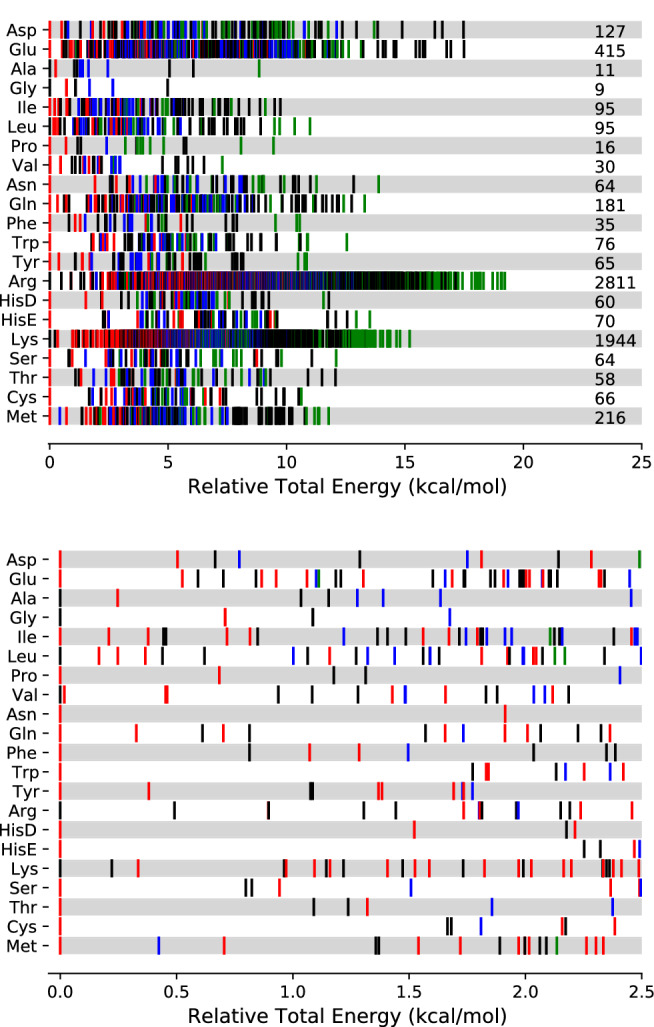
Conformational hierarchies for each pAA. Type I, II, III and IV backbone configurations are represented by black, red, blue and green colors, respectively. The top figure shows the relative energy of all conformers and their total number is summarized at the end of the row. The bottom figure zooms in the most stable types within a window of 2.5 kcal/mol

The energy distribution of these arrangements is represented in Fig. 10. Interestingly, type I and type IV configurations are, on average, the most unstable ones, with an average relative energy of 8.03 and 8.83 kcal/mol, respectively. On the contrary, type II and type III conformers are characterized by smaller relative energies (5.73 and 7.05 kcal/mol on average, respectively).

For the smallest amino acids, Gly and Ala (and also for Leu and Val), type I is the most stable arrangement, characterized by the -$\hbox {NH}_2\cdots$O=$\hbox {C}_1$ hydrogen bond and by the carboxylic acid group in *syn* disposition. The appearance of new interactions between the R fragment and the backbone for pAAs with long tails favors type II rearrangements, with a strong hydrogen bond between the nitrogen of the amino group and the hydrogen atom of the hydroxy group. The exceptions are Lys and Arg that include additional functional groups containing nitrogen at the end of the R fragments; these terminal nitrogen atoms form a strong hydrogen bond with the OH group (with the carboxylic acid group in *anti* disposition), leaving the amino group of the backbone in a type I arrangement. It may be argued that Asn and Gln also have terminal groups containing nitrogen in the side chain, but the interaction of the OH group with the acetamide seems weaker than with the amine or imine groups. For this reason, the most stable conformations of Asn and Gln are of type II.

Types I to III just account for hydrogen bonding within the common backbone. This interaction may be also of relevance between the backbone and the R fragment moiety, leading to geometrical distortions that are excluded from the three types. Therefore, the high number of type IV conformers is somehow expected.

In order to properly account for the significance of the conformers located during the HL search, we consider the multi-structural harmonic-oscillator (MS-HO) partition function. It takes into account all conformers and is given by [27–29]:

14 $$\begin{aligned} Q^{\mathrm{MS-HO}}_{\mathrm{rv}} = \sum _{j} Q^{\mathrm{RR-HO}}_{\mathrm{rv},j} e^{-U_j \beta } \end{aligned}$$

where $Q^{\mathrm{RR-HO}}_{\mathrm{rv},j}$ is the rigid-rotor harmonic-oscillator (RR-HO) rovibrational partition function of the *j*-th conformer, $U_j$ is its relative energy with regard to the most stable conformer and $\beta$ is $(k_B T)^{-1}$, with $k_B$ being the Boltzmann constant and T the temperature. Within this approximation, the contribution of a given conformer to the partition function can be obtained as:

15 $$\begin{aligned} \chi _i(T) = \frac{Q^{\mathrm{RR-HO}}_{\mathrm{rv},i} e^{-U_i \beta }}{Q^{\mathrm{MS-HO}}_{\mathrm{rv}}} = \frac{Q^{\mathrm{RR-HO}}_{\mathrm{rv},i} e^{-U_i \beta }}{\sum _{j} Q^{\mathrm{RR-HO}}_{\mathrm{rv},j} e^{-U_j \beta }} \end{aligned}$$

where we notice that this contribution is temperature-dependent and that:

16 $$\begin{aligned} \sum _i \chi _i(T) = 1 \end{aligned}$$

**Table 2:** Number of conformers required to achieve 90% of the MS-HO partition function, $N_{90}$, at 300 K. The table also collects the highest relative electronic, $E^{\mathrm{max}}$, and free, $G^{\mathrm{max}}$, energies within the considered conformers. Finally, it also includes the maximum contribution of a single conformer ($\chi _i^{\mathrm{max}}$) to the partition function

Amino acid	$N_{90}$	$E^{\mathrm{max}}$	$G^{\mathrm{max}}$	$\chi _i^{\mathrm{max}}$ (%)
Asp	16	2.782	3.220	17.4
Glu	66	3.312	4.601	16.9
Ala	6	1.388	1.361	57.8
Gly	5	1.086	1.138	57.3
Ile	23	2.106	3.131	33.1
Leu	19	1.931	3.298	52.4
Pro	4	1.313	0.676	45.1
Val	12	1.830	1.794	32.1
Asn	9	3.266	1.963	53.1
Gln	53	4.208	3.965	13.0
Phe	10	2.745	1.542	28.2
Trp	14	3.429	2.571	48.6
Tyr	16	2.980	1.809	18.5
Arg	80	4.333	5.653	47.3
HisD	4	2.213	1.875	78.9
HisE	5	2.491	2.261	70.6
Lys	667	5.490	5.232	1.7
Ser	12	2.627	2.118	29.6
Thr	11	3.036	2.303	38.7
Cys	16	3.597	2.708	31.1
Met	79	4.120	3.829	15.0

Table 2 lists the number of conformers, sorted by their electronic energy, needed to reach 0.9 in Eq. 16, i.e., 90% of the MS-HO partition function at 300 K. For the pAAs with few torsions the number of conformers is small (usually below 20 minima) and the largest contribution of a single conformer is well above 10%, often reaching 50%, and even 70% for HisD and HisE. This result justifies the common practice of considering that substantial contributions to the free energy are due to the most stable conformers in a window of 2.5 to 3.0 kcal/mol with respect to the absolute minimum, as the one showed in Fig. 10. However, in conformers with many torsional degrees of freedom, like Glu, Gln, Arg, Met, and Lys, the MS-HO partition function collects the contribution of many conformers, even if the most stable conformer contributes substantially to the partition function, as it is the case of Arg. The most notorious case is Lys for which the conformer with the largest contribution to MS-HO accounts for less than 2% of the total value. In fact, more than 600 conformations are needed to recover 90% of the partition function. This result shows that the theoretical evaluation of partition functions for flexible molecules involves a thorough exploration of the torsional PES, and its characterization by just a few stable conformers may handicap the accuracy of the results.

### The importance of the stochastic search

In this section we analyze and quantify the importance of the conformers located employing an stochastic algorithm, strategy avoided in many conformational studies because the search is completely random and detached from any chemical knowledge about the system. As a result, the stochastic exploration is less efficient than the preconditioned search. However, it is an ideal technique when employed after the preconditioned algorithm has already inspected the regions of the PES where conformers were expected. It allows reaching chemical structures that go beyond chemical intuition or that are stable due to unexpected intramolecular forces (like for instance hydrogen bonds between two distant fragments). When carried out in a second stage, the search is not completely random because exploits the information supplied by the similarity test, avoiding the search across previously inspected areas.

We notice that about 10% (680) of the HL conformers appeared after the stochastic search. Although the previous percentage seems significant, it does not really quantify the importance of these conformers. Usually, they are high energy conformers that barely contribute to the macroscopic properties of the system at room temperature (see Table 3).

**Table 3:** Contribution of the conformers encountered during the stochastic search $\chi _{\mathrm{st}}$ (in percentage) to the MS-HO partition function at *T* = 300 K

Amino acid	$\chi _{\mathrm{st}}$ (%)
Asp	0.3
Glu	1.3
Ala	0.0
Gly	0.0
Ile	3.0
Leu	0.6
Pro	0.2
Val	3.0
Asn	0.1
Gln	1.7
Phe	0.2
Trp	9.4
Tyr	0.2
Arg	3.5
HisD	0.0
HisE	0.4
Lys	1.7
Ser	0.2
Thr	0.0
Cys	0.5
Met	7.0

If the summation of Eq. 16 is restricted to the conformers associated with the stochastic search, $\chi _{\mathrm{st}}$, we find that at *T* = 300 K this contribution is generally small, with the exception of two amino acids, Trp, and Met, for which the contribution is larger than 5%, but none of the cases reached 10%. These results clearly indicate that the preconditioned search already retrieves the most stable conformers and that the stochastic algorithm, at least in the case of amino acids, has a minor contribution to the partition function.

Additionally for the case of Lys, we have performed a preconditioned search at two different LL methods. At the HF/3-21G level 2 224 minima were located; however, the same initial torsional angles only produced 1 412 minima when the search was carried out at the PM6 [91] semiempirical method. These numbers point toward the same direction as the study of Mancini et al. [11] when studying Thr, as the semiempirical methods employed produced fewer conformers than the benchmark study. These results indicate that a proper selection of the LL method is critical. To this conclusion we may add that the additional conformers obtained during the stochastic search have a modest influence on the room-temperature MS-HO partition function.

## Limitations and future work

In its current version, Torsiflex cannot automatically deal with different conformations arising from ring deformations, although it is possible to include them by finely tuning the torsions inside the ring (as was the case in proline). The input of the program also assumes some user skills in the construction of the Z-Matrix, which is a drawback.

Torsiflex is an open-source code written in Python 3 that can be easily modified to incorporate other electronic structure software besides *Gaussian*. It can be combined to structure generation programs, as for instance RDKit [92] or MAYGEN [93]. In particular, we are developing a structure-builder software that tries to avoid the definition of an initial Z-Matrix by the user. In addition to the generation of the chemical graph, this task involves, first, spotting the internal rotations, and second, modeling the rest of the structural parameters taking into account the targeted torsions. Another extension that is in our sights, is the automatic search and location of conformations as a result of ring puckering in 5, 6 and 7 member rings.

## Conclusions

We have presented a new program, TorsiFlex, intended for the search of conformers of acyclic molecules with several torsional degrees of freedom. The proposed algorithm is based on the synergy between preconditioned and stochastic methods at an inexpensive level that, combined with a dual-level approach, is able to find the conformers of the torsional PES at a high level method. TorsiFlex automatizes the whole process and almost no actions are required from the user. The combination of a battery of validation tests together with a suitable low-level electronic structure method accelerates the searching process, making it more efficient. Additionally, issues like umbrella inversion and conformational enantiomerism are automatically taken into account by the program.

In order to show the effectiveness and capabilities of this software, we have employed TorsiFlex to automatically locate the conformers of the twenty proteinogenic amino acids. Within the dual-level approximation we have employed the HF/3-21G as a low-level method and the M08HX/MG3S method as the high level. A total of 6 508 conformers were found, a number that improves substantially the total amount of previously reported conformers. For the most flexible amino acids a large number of conformers is required to recover 90% of the MS-HO partition function. The results also indicate that, at least in the case of the amino acids, a well designed preconditioned search suffices to obtain converged MS-HO partition functions and that search of new conformers through a stochastic search is less important than the choice of the low-level method adopted for the exploration of the PES.

### Availability and requirements

- **Project name:** Torsiflex (version 2021.3)
- **Project home page:** https://github.com/cathedralpkg/TorsiFlex
- **Operating systems:** Linux and MacOS
- **Programing language:** Python 3
- **Other requirements:** None
- **License:** MIT

## Supplementary Information

**Additional file 1.** It contains for each of the pAAs: the Z-matrix for the reference geometry indicating the target torsions, the M08-HX/MG3S absolute energy of the most stable conformer, the relative energy, moments of inertia, dipole moments, zero-point energy, normal mode vibrational frequencies and Cartesian coordinates of the M08-HX/MG3S optimized geometries of each conformer.

## Data Availability

The manual of Torsiflex and the source code, are available at: https://github.com/cathedralpkg/TorsiFlex The LL and HL Cartesian coordinates of the conformers, the reference Z-matrix and the Torsiflex input files for the 20 amino acids are available at: https://github.com/cathedralpkg/Aminoacids.

## Footnotes

1 All reference Z-matrix geometries for the 20 amino acids are listed in the Additional file 1.

2 As for example, in Gly, the target torsion about the C-C single bond can be defined as N-C-C-O. However, the two methylene $\hbox {H}_1$ and $\hbox {H}_2$ atoms need to be specified as improper torsions (e.g. $\hbox {H}_1$-C-N-C, and $\hbox {H}_2$-C-N-C).

3 When dealing with the conformers of a transition state, the test is positive when all frequencies but one (which is imaginary) are real.

4 Assuming that dihedral angles are defined between − 180$^\circ$ and +180$^\circ$.

5 When J = I, the summation excludes $\phi _1$, because this angle can take any value.
