# Correction to: TorsiFlex: an automatic generator of torsional conformers. Application to the twenty proteinogenic amino acids

**DOI:** 10.1186/s13321-022-00585-9

**Published:** 2022-03-14

**Authors:** David Ferro‑Costas, Irea Mosquera‑Lois, Antonio Fernandez‑Ramos

**Affiliations:** Centro Singular de Investigacion en Quimica Bioloxica E Materiais Moleculares (CIQUS), Universidade de Santiago de Compostela, 15782 Santiago de Compostela, Spain

## Correction to: Journal of Cheminformatics (2021) 13:100 https://doi.org/10.1186/s13321-021-00578-0

Following publication of the original article [1], the have been notified by the authors that some corrections were missed.

1. The capitation of Fig. 6 has been corrected.
2. A few other minor corrections in the body of the text.

The original article has been corrected.
