# Supplementary material for: TorsiFlex: an automatic generator of torsional conformers. Application to the twenty proteinogenic amino acids
Source: J Cheminform. 2021 Dec 24;13:100. doi: 10.1186/s13321-021-00578-0 (PMC8710030; doi:10.1186/s13321-021-00578-0)
Supplement: Supplementary file 1 — Additional file 1. It contains for each of the pAAs: the Z-matrix for the reference geometry indicating the target torsions, the M08-HX/MG3S absolute energy of the most stable conformer, the relative energy, moments of inertia, dipole moments, zero-point energy, normal mode vibrational frequencies and Cartesian coordinates of the M08-HX/MG3S optimized geometries of each conformer. [file 13321_2021_578_MOESM1_ESM.pdf]

Additional file 1 for
TorsiFlex: an automatic generator of torsional conformers. Application to the twenty proteinogenic amino acids

David Ferro-Costas*, Irea Mosquera-Lois, and Antonio Fernández-Ramos*

Centro Singular de Investigación en Química Biolóxica e Materiais Moleculares (CIQUS), Universidade de Santiago de Compostela, 15782 Santiago de Compostela, Spain

Contents

1 Asp **6**
- 1.1 Reference Z-matrix for Asp 6
- 1.2 Reference energy and target torsions for Asp 7
- 1.3 Properties of Asp conformers 8
- 1.4 Vibrational frequencies of Asp conformers 11
- 1.5 Cartesian coordinates of Asp conformers 43

2 Glu **101**
- 2.1 Reference Z-matrix for Glu 101
- 2.2 Reference energy and target torsions for Glu 103
- 2.3 Properties of Glu conformers 103
- 2.4 Vibrational frequencies of Glu conformers 114
- 2.5 Cartesian coordinates of Glu conformers 253

*david.ferro@usc.es, qf.ramos@usc.es

3 Ala **472**
3.1 Reference Z-matrix for Ala 472
3.2 Reference energy and target torsions for Ala 473
3.3 Properties of Ala conformers 473
3.4 Vibrational frequencies of Ala conformers 474
3.5 Cartesian coordinates of Ala conformers 476

4 Gly **481**
4.1 Reference Z-matrix for Gly 481
4.2 Reference energy and target torsions for Gly 482
4.3 Properties of Gly conformers 482
4.4 Vibrational frequencies of Gly conformers 482
4.5 Cartesian coordinates of Gly conformers 484

5 Ile **487**
5.1 Reference Z-matrix for Ile 487
5.2 Reference energy and target torsions for Ile 489
5.3 Properties of Ile conformers 489
5.4 Vibrational frequencies of Ile conformers 492
5.5 Cartesian coordinates of Ile conformers 524

6 Leu **582**
6.1 Reference Z-matrix for Leu 582
6.2 Reference energy and target torsions for Leu 584
6.3 Properties of Leu conformers 584
6.4 Vibrational frequencies of Leu conformers 587
6.5 Cartesian coordinates of Leu conformers 619

7 Pro **677**
7.1 Reference Z-matrix for Pro 677
7.2 Reference energy and target torsions for Pro 678
7.3 Properties of Pro conformers 679
7.4 Vibrational frequencies of Pro conformers 679
7.5 Cartesian coordinates of Pro conformers 684

8 Val **692**
8.1 Reference Z-matrix for Val 692
8.2 Reference energy and target torsions for Val 694

8.3 Properties of Val conformers 694
8.4 Vibrational frequencies of Val conformers 695
8.5 Cartesian coordinates of Val conformers 705

9 Asn **722**
9.1 Reference Z-matrix for Asn 722
9.2 Reference energy and target torsions for Asn 723
9.3 Properties of Asn conformers 724
9.4 Vibrational frequencies of Asn conformers 726
9.5 Cartesian coordinates of Asn conformers 742

10 Gln **773**
10.1 Reference Z-matrix for Gln 773
10.2 Reference energy and target torsions for Gln 775
10.3 Properties of Gln conformers 775
10.4 Vibrational frequencies of Gln conformers 780
10.5 Cartesian coordinates of Gln conformers 840

11 Phe **941**
11.1 Reference Z-matrix for Phe 941
11.2 Reference energy and target torsions for Phe 943
11.3 Properties of Phe conformers 943
11.4 Vibrational frequencies of Phe conformers 944
11.5 Cartesian coordinates of Phe conformers 956

12 Trp **979**
12.1 Reference Z-matrix for Trp 979
12.2 Reference energy and target torsions for Trp 981
12.3 Properties of Trp conformers 982
12.4 Vibrational frequencies of Trp conformers 984
12.5 Cartesian coordinates of Trp conformers 1022

13 Tyr **1078**
13.1 Reference Z-matrix for Tyr 1078
13.2 Reference energy and target torsions for Tyr 1080
13.3 Properties of Tyr conformers 1080
13.4 Vibrational frequencies of Tyr conformers 1082
13.5 Cartesian coordinates of Tyr conformers 1104

14 Arg **1148**
14.1 Reference Z-matrix for Arg 1148
14.2 Reference energy and target torsions for Arg 1150
14.3 Properties of Arg conformers 1151
14.4 Vibrational frequencies of Arg conformers 1227
14.5 Cartesian coordinates of Arg conformers 2632

15 HisD **4601**
15.1 Reference Z-matrix for HisD 4601
15.2 Reference energy and target torsions for HisD 4603
15.3 Properties of HisD conformers 4603
15.4 Vibrational frequencies of HisD conformers 4605
15.5 Cartesian coordinates of HisD conformers 4625

16 HisE **4659**
16.1 Reference Z-matrix for HisE 4659
16.2 Reference energy and target torsions for HisE 4661
16.3 Properties of HisE conformers 4661
16.4 Vibrational frequencies of HisE conformers 4663
16.5 Cartesian coordinates of HisE conformers 4686

17 Lys **4726**
17.1 Reference Z-matrix for Lys 4726
17.2 Reference energy and target torsions for Lys 4728
17.3 Properties of Lys conformers 4728
17.4 Vibrational frequencies of Lys conformers 4781
17.5 Cartesian coordinates of Lys conformers 5430

18 Ser **6694**
18.1 Reference Z-matrix for Ser 6694
18.2 Reference energy and target torsions for Ser 6695
18.3 Properties of Ser conformers 6695
18.4 Vibrational frequencies of Ser conformers 6697
18.5 Cartesian coordinates of Ser conformers 6710

19 Thr **6737**
19.1 Reference Z-matrix for Thr 6737
19.2 Reference energy and target torsions for Thr 6738

19.3 Properties of Thr conformers 6739
19.4 Vibrational frequencies of Thr conformers 6740
19.5 Cartesian coordinates of Thr conformers 6755

20 Cys **6784**
20.1 Reference Z-matrix for Cys 6784
20.2 Reference energy and target torsions for Cys 6785
20.3 Properties of Cys conformers 6785
20.4 Vibrational frequencies of Cys conformers 6787
20.5 Cartesian coordinates of Cys conformers 6801

21 Met **6828**
21.1 Reference Z-matrix for Met 6828
21.2 Reference energy and target torsions for Met 6830
21.3 Properties of Met conformers 6830
21.4 Vibrational frequencies of Met conformers 6836
21.5 Cartesian coordinates of Met conformers 6908

1 Asp

1.1 Reference Z-matrix for Asp

Bond distances are given in Å; bond and dihedral angles, in degrees.

h						
o	1	oh2				
c	2	co3	1	coh3		
c	3	cc4	2	cco4	1	dih4
n	4	nc5	3	ncc5	2	dih5
h	5	hn6	4	hnc6	3	dih6
o	3	oc7	2	oco7	4	dih7
c	4	cc8	3	ccc8	5	dih8
h	4	hc9	3	hcc9	5	dih9
h	5	hn10	4	hnc10	6	dih10
c	8	cc11	4	ccc11	3	dih11
h	8	hc12	4	hcc12	11	dih12
h	8	hc13	4	hcc13	11	dih13
o	11	oc14	8	occ14	4	dih14
h	14	ho15	11	hoc15	8	dih15
o	11	oc16	8	occ16	14	dih16

oh2	0.96837938
co3	1.35572702
coh3	112.1417013
cc4	1.50670316
cco4	110.85002087
dih4	180.85047015
nc5	1.46789701
ncc5	107.5118252
dih5	49.53628326
hn6	1.00395139
hnc6	111.11513163
dih6	183.32161963
oc7	1.20159336
oco7	122.40533925
dih7	181.4526388
cc8	1.52195432
ccc8	109.02085691
dih8	118.88218168

hc9	1.08884005
hcc9	107.28899741
dih9	237.6873375
hn10	1.00340287
hnc10	112.26313682
dih10	126.93707791
cc11	1.49697889
ccc11	111.36812983
dih11	174.64880857
hc12	1.08268627
hcc12	108.74318882
dih12	119.25040935
hc13	1.08072263
hcc13	111.6727679
dih13	237.87698666
oc14	1.35115421
occ14	111.32818094
dih14	186.05093943
ho15	0.96871306
hoc15	112.12762083
dih15	181.22501527
oc16	1.20680598
occ16	126.47970545
dih16	180.38555848

1.2 Reference energy and target torsions for Asp

- Reference M08-HX/MG3S energy: -512.3318989 hartree
- Target torsions:
  - torsion1: dih4
  - torsion2: dih5
  - torsion3: dih6
  - torsion4: dih11
  - torsion5: dih14
  - torsion6: dih15

1.3 Properties of Asp conformers

Table S1: Relative energy (kcal mol^{-1}), rotational constants (B1 to B3, GHz) and dipolar moment (Debye) for the conformers of Asp located at the M08-HX/MG3S level.

Conformer	Rel. Energy	B1	B2	B3	Dip. Mom.
1	0.000	1.086	1.224	2.635	5.318
2	0.504	0.770	0.914	3.468	4.429
3	0.666	1.093	1.241	2.591	2.542
4	0.770	1.090	1.221	2.649	0.661
5	1.288	0.783	0.918	3.443	2.241
6	1.750	0.765	0.909	3.570	0.785
7	1.811	0.800	0.916	3.292	4.250
8	2.142	0.784	0.956	3.219	5.232
9	2.283	1.115	1.257	2.585	5.322
10	2.490	0.973	1.155	2.642	1.128
11	2.507	0.901	1.129	2.709	4.057
12	2.640	1.131	1.340	2.367	5.690
13	2.733	0.808	0.903	3.448	4.631
14	2.739	1.090	1.381	2.413	4.812
15	2.762	1.112	1.254	2.535	1.577
16	2.782	0.821	0.891	3.287	1.578
17	2.801	0.807	0.910	3.249	3.068
18	3.060	0.955	1.136	2.792	5.078
19	3.167	0.836	0.906	3.331	2.691
20	3.185	1.112	1.243	2.646	2.619
21	3.266	1.107	1.229	2.601	2.248
22	3.307	1.100	1.218	2.628	3.308
23	3.483	0.935	1.126	2.726	2.070
24	3.601	0.952	1.125	2.777	1.961
25	3.601	0.989	1.192	2.586	2.777
26	3.627	1.163	1.408	2.275	2.807
27	3.753	0.992	1.206	2.551	1.815
28	3.816	0.819	0.890	3.288	3.190
29	3.838	0.852	1.127	2.880	6.099
30	3.864	0.794	0.877	3.459	2.047
31	3.960	1.106	1.235	2.556	3.736

32	3.976	0.993	1.180	2.641	3.391
33	4.000	0.965	1.153	2.781	5.790
34	4.142	0.763	0.929	3.423	6.879
35	4.159	0.819	0.887	3.501	3.103
36	4.193	1.030	1.080	2.863	4.657
37	4.228	0.781	0.910	3.558	3.742
38	4.234	0.806	0.926	3.230	6.486
39	4.330	0.902	1.128	2.733	5.581
40	4.485	0.985	1.192	2.535	2.318
41	4.595	0.839	0.899	3.282	1.862
42	4.657	1.111	1.221	2.701	2.932
43	4.712	1.097	1.274	2.522	7.331
44	4.820	1.087	1.232	2.605	6.485
45	4.827	0.973	1.161	2.742	4.140
46	4.993	0.798	0.961	3.207	1.281
47	4.998	0.769	0.919	3.436	2.850
48	5.000	1.020	1.236	2.496	2.011
49	5.010	1.168	1.396	2.276	1.304
50	5.223	1.113	1.198	2.649	1.452
51	5.312	0.990	1.206	2.550	2.314
52	5.353	1.161	1.370	2.302	2.513
53	5.408	1.098	1.226	2.631	3.079
54	5.423	1.095	1.243	2.596	4.579
55	5.512	0.863	0.877	3.322	1.024
56	5.514	1.027	1.306	2.567	4.947
57	5.566	0.997	1.206	2.931	5.846
58	5.647	1.161	1.384	2.308	1.648
59	5.738	0.877	1.149	2.767	5.555
60	5.955	0.966	1.197	2.529	1.287
61	5.973	0.787	0.918	3.415	3.842
62	6.083	0.767	0.912	3.538	2.548
63	6.148	0.972	1.130	3.147	4.857
64	6.155	0.919	1.171	2.603	6.083
65	6.285	1.165	1.376	2.303	3.136
66	6.451	0.799	0.922	3.262	2.264
67	6.468	0.987	1.205	2.528	4.249
68	6.537	1.049	1.244	2.410	2.498

69	6.628	1.038	1.292	2.357	3.238
70	6.882	0.965	1.164	2.543	3.682
71	7.004	0.899	1.127	2.709	4.400
72	7.283	1.101	1.399	2.304	4.833
73	7.289	1.122	1.226	2.547	4.531
74	7.317	1.118	1.400	2.375	6.068
75	7.330	0.787	0.962	3.185	2.950
76	7.345	0.983	1.160	2.604	3.747
77	7.461	0.797	0.907	3.393	4.064
78	7.570	0.935	1.186	2.531	4.593
79	7.711	0.810	0.911	3.223	4.486
80	7.839	0.845	1.116	2.898	5.626
81	7.848	0.831	0.893	3.224	2.543
82	8.061	1.112	1.236	2.586	3.973
83	8.127	0.821	0.899	3.217	4.290
84	8.206	1.086	1.219	2.543	4.704
85	8.409	0.948	1.142	2.722	2.587
86	8.550	0.992	1.194	2.578	4.873
87	8.631	0.786	0.909	3.348	4.103
88	8.634	0.841	0.908	3.274	3.698
89	8.709	0.935	1.121	2.727	3.870
90	8.740	0.844	1.123	2.831	3.546
91	8.789	1.032	1.081	2.847	4.943
92	8.904	1.034	1.256	2.636	5.395
93	8.951	0.846	1.111	2.804	4.790
94	9.034	0.793	0.880	3.410	4.423
95	9.055	1.110	1.245	2.542	5.606
96	9.114	1.152	1.364	2.341	2.398
97	9.308	0.912	1.132	2.700	6.009
98	9.317	0.812	0.968	3.169	4.403
99	9.419	0.802	0.969	3.169	4.776
100	9.421	0.980	1.059	3.113	2.840
101	9.499	1.181	1.440	2.205	3.665
102	9.540	0.995	1.192	2.518	4.340
103	9.614	1.142	1.259	2.411	6.770
104	9.718	0.808	0.964	3.176	3.126
105	9.743	0.999	1.225	2.388	5.515

106	9.862	0.856	1.116	2.788	3.318
107	9.900	1.022	1.243	2.434	3.656
108	10.070	0.852	0.890	3.253	4.201
109	10.112	0.967	1.076	3.192	4.320
110	10.281	0.994	1.090	3.105	2.521
111	10.749	0.822	0.898	3.364	4.820
112	10.753	0.820	0.884	3.466	5.169
113	10.936	0.763	0.946	3.293	6.878
114	11.220	1.026	1.236	2.465	3.306
115	11.370	1.169	1.393	2.232	4.505
116	11.461	1.030	1.256	2.334	5.260
117	11.465	1.098	1.185	2.523	5.872
118	11.758	0.998	1.208	2.514	6.243
119	12.113	0.995	1.154	2.567	5.874
120	12.357	0.808	0.904	3.354	3.066
121	12.951	0.832	0.888	3.207	3.249
122	13.698	1.038	1.204	2.768	9.371
123	13.850	1.071	1.201	2.621	6.745
124	14.882	1.155	1.411	2.265	5.542
125	16.264	1.030	1.231	2.475	5.432
126	16.581	1.087	1.272	2.512	6.153
127	17.472	1.094	1.177	2.543	7.812

1.4 Vibrational frequencies of Asp conformers

Harmonic vibrational zero point energy (ZPE) and frequencies for each conformer in cm^{-1}.

- Conformer (1); ZPE = 27608.535 cm^{-1}:

51.69	76.84	92.23	212.49	289.88	339.99
357.46	386.14	479.95	515.95	559.31	582.61
648.36	685.37	772.55	800.35	826.26	884.77
899.82	939.48	1065.22	1124.18	1178.56	1208.22
1246.16	1261.57	1310.34	1328.33	1402.43	1423.77
1436.70	1453.07	1643.00	1849.07	1888.18	3049.81
3067.55	3122.50	3554.22	3652.62	3709.51	3840.58

- Conformer (2); ZPE = 27530.777 cm^{-1}:

54.09	84.29	89.02	154.79	255.48	322.27
353.95	376.71	434.09	522.78	547.42	606.95
653.05	671.90	750.09	800.55	873.42	906.84
917.49	935.46	1079.25	1150.94	1174.60	1186.46
1229.91	1269.28	1282.09	1343.41	1397.70	1425.12
1443.64	1445.87	1661.23	1849.73	1890.73	3044.34
3059.47	3106.86	3538.01	3626.99	3702.59	3842.71

- Conformer (3); ZPE = 27431.202 cm^{-1}:

53.02	78.84	99.75	171.91	257.67	266.42
328.66	363.82	455.06	489.75	529.62	578.64
616.16	661.12	677.54	776.13	823.20	898.00
922.28	970.07	1050.29	1138.85	1171.41	1197.59
1206.58	1281.19	1307.57	1324.00	1388.64	1403.42
1433.14	1450.64	1670.65	1852.67	1877.14	3037.11
3077.43	3125.67	3540.44	3619.73	3844.46	3846.12

- Conformer (4); ZPE = 27465.832 cm^{-1}:

44.62	65.16	108.25	186.95	262.56	297.71
329.98	361.14	454.25	505.73	543.27	566.16
613.99	659.43	682.85	779.52	824.71	894.65
906.26	960.39	1050.06	1134.07	1187.73	1202.55
1210.49	1275.75	1316.51	1325.13	1398.09	1405.54
1424.77	1450.09	1662.54	1849.23	1857.45	3041.71
3080.83	3131.35	3551.85	3635.68	3845.95	3846.69

- Conformer (5); ZPE = 27412.119 cm^{-1}:

52.78	74.67	89.54	157.13	242.97	321.76
343.98	372.06	444.89	493.91	522.00	588.71
618.91	658.96	666.24	742.06	865.65	879.16
920.65	954.18	1067.37	1158.87	1184.85	1189.60
1225.34	1247.22	1284.68	1345.35	1368.34	1415.43
1443.44	1450.98	1631.38	1854.99	1871.52	2966.44
3068.58	3119.31	3564.95	3658.67	3847.06	3849.67

- Conformer (6); ZPE = 27420.999 cm^{-1}:

44.04	73.70	92.25	154.75	249.30	318.32
330.09	368.15	407.75	527.44	543.53	584.96
614.15	662.77	676.27	753.17	878.02	881.56
917.80	967.72	1062.00	1162.82	1176.30	1199.16
1208.40	1258.30	1289.27	1339.44	1370.31	1418.17
1442.44	1467.43	1650.09	1851.46	1870.57	2954.53
3069.34	3115.82	3555.73	3640.01	3844.86	3849.81

- Conformer (7); ZPE = 27550.388 cm^{-1}:

47.33	60.68	107.72	152.06	237.40	313.81
348.20	354.73	449.92	507.64	561.79	622.49
663.52	685.74	752.18	803.48	879.59	899.23
925.09	986.25	1033.71	1142.90	1185.11	1190.24
1243.94	1277.96	1291.05	1358.31	1378.38	1420.87
1437.77	1451.64	1674.80	1853.18	1897.91	3039.67
3052.75	3110.51	3544.39	3617.83	3694.90	3844.11

- Conformer (8); ZPE = 27568.923 cm^{-1}:

51.07	68.97	99.81	168.40	256.55	290.72
315.15	374.72	452.50	503.03	564.78	618.32
649.58	707.49	766.24	861.99	895.93	903.95
942.86	991.30	1022.88	1145.69	1161.71	1191.86
1259.45	1290.36	1316.74	1344.49	1376.84	1435.65
1457.48	1459.86	1663.92	1868.00	1886.31	3037.23
3064.03	3145.91	3526.69	3543.96	3614.49	3840.94

- Conformer (9); ZPE = 27580.998 cm^{-1}:

33.25	75.95	110.01	204.24	283.43	336.77
357.18	376.89	478.87	519.42	560.17	587.99
636.07	680.87	776.40	797.51	818.81	877.44
887.50	925.63	1064.17	1127.04	1176.04	1205.32
1246.12	1264.91	1311.48	1340.69	1392.37	1400.50
1429.38	1439.47	1652.32	1883.62	1892.07	3048.13
3072.18	3126.18	3560.33	3653.26	3710.07	3841.95

- Conformer (10); ZPE = 27417.039 cm^{-1}:

31.19	65.33	98.03	153.09	246.54	280.71
316.55	349.77	397.75	516.75	551.94	589.31
626.44	656.95	677.23	777.16	832.23	884.69
913.90	999.29	1055.24	1137.87	1164.55	1180.50
1211.25	1252.63	1303.17	1325.23	1396.79	1424.10
1441.60	1452.59	1660.06	1859.84	1864.73	3066.72
3085.69	3117.17	3542.46	3633.21	3846.42	3847.40

- Conformer (11); ZPE = 27458.812 cm^{-1}:

51.16	84.87	102.45	143.95	221.04	236.07
305.40	349.56	446.63	503.78	526.33	640.22
666.63	686.37	742.01	777.95	836.98	907.14
927.84	1003.74	1050.34	1162.85	1177.78	1212.11
1221.95	1255.91	1316.24	1346.30	1396.79	1425.56
1444.19	1458.77	1675.79	1825.93	1884.56	3047.18
3057.98	3103.42	3532.35	3612.33	3712.21	3837.00

- Conformer (12); ZPE = 27592.588 cm^{-1}:

51.30	70.30	74.91	241.98	284.42	311.12
357.74	405.87	478.12	510.11	563.69	599.44
619.80	707.99	785.09	825.43	872.14	893.65
949.35	968.52	1010.27	1122.95	1189.69	1210.59
1226.39	1287.55	1322.53	1348.42	1369.37	1435.78
1451.98	1463.92	1628.55	1868.96	1887.75	2989.64
3059.77	3148.31	3542.23	3562.31	3642.40	3844.86

- Conformer (13); ZPE = 27531.146 cm^{-1}:

33.56	78.69	79.47	148.08	244.95	321.53
368.78	380.87	442.44	516.19	559.54	612.87
640.48	691.25	750.45	795.47	859.72	901.13
907.02	928.88	1071.23	1131.03	1170.37	1211.54
1235.30	1264.94	1279.07	1356.81	1385.01	1402.13
1431.04	1452.60	1659.67	1877.55	1893.05	3058.01
3067.24	3119.34	3543.76	3636.77	3710.76	3843.70

- Conformer (14); ZPE = 27702.816 cm^{-1}:

57.85	75.61	140.52	189.11	260.24	308.23
362.75	438.06	467.86	534.38	561.30	600.00
678.12	744.23	803.98	820.35	836.83	899.52
925.85	945.55	1066.35	1107.13	1170.01	1226.17
1253.58	1287.64	1300.53	1337.30	1391.63	1409.48
1446.25	1471.62	1674.02	1854.80	1875.51	3048.38
3085.21	3147.43	3522.67	3632.72	3672.17	3774.70

- Conformer (15); ZPE = 27367.972 cm^{-1}:

12.19	74.08	104.55	173.75	221.57	257.25
327.55	360.89	463.39	493.10	524.95	582.50
623.57	648.14	673.36	773.34	814.23	884.66
907.88	955.81	1049.67	1140.23	1173.80	1197.27
1210.48	1282.23	1306.94	1339.54	1385.82	1393.62
1408.02	1439.30	1673.07	1873.96	1878.37	3031.87
3082.83	3130.48	3549.29	3625.53	3842.85	3844.01

- Conformer (16); ZPE = 27355.014 cm^{-1}:

39.50	62.42	71.72	148.09	168.41	224.96
312.52	369.66	430.85	505.81	534.74	590.00
623.68	660.71	692.49	755.11	853.25	905.71
925.29	953.86	1048.26	1153.38	1169.61	1184.86
1213.83	1255.67	1301.43	1326.35	1368.34	1427.17
1433.43	1457.38	1672.68	1864.37	1870.65	3062.42
3072.00	3119.87	3550.49	3641.62	3843.52	3843.94

- Conformer (17); ZPE = 27369.429 cm^{-1}:

47.21	63.77	97.51	149.25	228.58	275.91
338.74	355.41	440.69	501.56	546.17	601.15
624.05	673.49	677.07	763.75	809.62	878.44
905.14	942.88	1061.89	1149.70	1162.40	1178.63
1211.21	1267.17	1289.98	1335.43	1366.00	1417.45
1448.58	1454.36	1623.18	1856.80	1876.52	3047.00
3064.23	3097.46	3560.58	3662.61	3840.84	3846.43

- Conformer (18); ZPE = 27548.562 cm^{-1}:

53.86	79.30	96.28	163.02	281.04	296.38
356.88	370.73	415.66	519.53	562.39	592.51
643.98	702.79	747.83	764.50	835.74	863.22
920.65	973.26	1096.95	1139.62	1160.39	1206.10
1229.99	1243.80	1299.43	1341.60	1390.97	1432.26
1437.07	1458.77	1659.61	1861.52	1891.66	3053.55
3059.51	3120.81	3550.43	3643.51	3734.11	3845.90

- Conformer (19); ZPE = 27372.249 cm^{-1}:

44.71	64.42	75.14	147.73	205.99	221.79
298.68	370.14	428.83	505.47	542.40	596.56
609.19	649.12	711.30	755.65	852.93	905.91
927.32	965.49	1017.21	1126.74	1167.73	1200.68
1229.19	1265.79	1307.52	1340.90	1379.19	1397.27
1423.22	1460.11	1670.72	1869.70	1877.63	3061.54
3072.33	3128.40	3545.85	3628.68	3843.33	3852.00

- Conformer (20); ZPE = 27389.394 cm^{-1}:

58.58	93.56	105.68	158.27	250.69	252.95
334.92	391.50	477.11	498.92	520.13	582.14
607.24	623.32	698.69	781.87	814.03	850.12
930.88	951.33	1018.29	1125.01	1181.27	1188.24
1223.02	1242.97	1318.20	1339.08	1348.11	1409.87
1441.93	1466.11	1636.84	1870.73	1879.88	2963.48
3076.60	3136.78	3563.62	3660.33	3852.47	3854.01

- Conformer (21); ZPE = 27419.613 cm^{-1}:

47.66	71.26	105.77	181.45	251.58	285.03
345.80	351.23	478.94	514.77	541.36	566.31
622.34	667.47	687.63	782.58	808.80	845.57
895.18	941.50	1044.27	1136.38	1190.23	1194.42
1218.67	1266.19	1296.54	1328.80	1375.11	1421.94
1446.18	1458.00	1655.21	1850.95	1853.26	3035.61
3067.55	3114.67	3555.32	3648.14	3842.42	3847.14

- Conformer (22); ZPE = 27426.074 cm^{-1}:

20.03	57.32	110.95	187.08	258.93	275.87
329.44	361.40	467.90	502.14	543.56	579.25
613.35	668.72	672.70	776.15	814.40	879.08
889.24	946.72	1048.35	1133.25	1184.56	1207.22
1213.74	1276.95	1317.51	1338.68	1391.27	1396.22
1406.53	1432.22	1667.67	1860.32	1879.28	3036.91
3083.83	3136.29	3557.62	3639.43	3844.11	3845.95

- Conformer (23); ZPE = 27328.525 cm^{-1}:

28.92	41.96	93.24	147.43	206.36	257.03
308.67	348.41	420.71	499.19	527.86	589.75
623.41	650.90	688.80	750.69	827.46	907.46
919.15	1002.76	1044.63	1158.65	1170.34	1181.10
1226.90	1241.43	1304.30	1331.44	1382.31	1411.17
1436.75	1458.90	1672.37	1865.10	1868.23	3043.17
3061.26	3113.83	3539.11	3617.29	3843.65	3844.93

- Conformer (24); ZPE = 27384.378 cm^{-1}:

35.31	64.88	101.54	163.30	253.51	269.10
298.25	377.98	383.46	517.14	557.52	579.52
617.80	653.08	679.37	758.86	821.35	877.61
926.08	995.94	1061.30	1132.09	1173.03	1190.11
1218.96	1259.00	1288.98	1331.61	1367.93	1437.72
1447.79	1464.39	1654.03	1862.04	1877.92	2996.31
3060.77	3127.44	3552.73	3638.53	3846.78	3847.70

- Conformer (25); ZPE = 27412.490 cm^{-1}:

36.57	66.32	105.15	146.25	248.30	278.24
318.49	350.69	403.50	511.75	570.51	588.93
619.76	656.73	668.39	780.00	828.97	886.63
914.53	989.46	1040.71	1133.69	1160.41	1183.41
1219.38	1257.72	1306.79	1352.11	1380.95	1401.78
1442.81	1451.85	1658.80	1861.68	1876.82	3064.14
3090.54	3113.14	3538.35	3629.24	3844.94	3846.54

- Conformer (26); ZPE = 27418.891 cm^{-1}:

33.59	71.36	100.02	157.64	192.57	234.34
298.58	395.58	444.93	501.57	558.22	581.12
631.30	643.12	740.49	803.11	824.69	882.90
918.96	965.57	1020.02	1131.86	1168.94	1179.89
1229.92	1264.56	1317.10	1324.03	1371.16	1407.88
1416.15	1467.99	1677.77	1861.85	1871.76	3031.66
3095.68	3161.89	3543.20	3626.42	3840.10	3848.28

- Conformer (27); ZPE = 27355.514 cm^{-1}:

27.90	83.30	94.53	150.32	203.59	250.26
289.72	342.75	428.58	497.36	545.70	589.62
616.28	636.22	711.93	764.67	818.78	903.23
911.26	999.34	1042.78	1134.22	1166.13	1195.44
1229.68	1237.99	1299.92	1342.03	1377.40	1397.08
1426.65	1461.42	1673.24	1868.80	1874.30	3064.63
3071.25	3127.56	3541.21	3620.50	3842.31	3851.15

- Conformer (28); ZPE = 27351.210 cm^{-1}:

21.00	60.07	95.33	144.02	227.99	284.83
328.91	344.80	435.20	509.62	558.32	599.74
604.78	662.34	668.42	775.66	834.79	875.43
905.99	934.32	1063.50	1119.62	1171.23	1175.81
1223.29	1263.91	1291.34	1344.95	1370.22	1416.74
1437.65	1451.27	1642.98	1857.83	1875.63	3045.26
3086.02	3099.99	3555.15	3649.18	3842.78	3846.52

- Conformer (29); ZPE = 27721.007 cm^{-1}:

54.18	89.98	116.56	218.14	256.43	310.66
362.50	377.97	426.32	548.49	565.32	639.86
739.42	759.75	816.15	823.93	846.86	893.73
929.50	975.67	1072.50	1128.62	1187.84	1197.61
1262.28	1308.50	1316.12	1349.30	1401.03	1425.65
1456.28	1462.61	1661.90	1855.45	1884.49	3035.77
3056.50	3137.74	3549.11	3629.86	3641.76	3669.70

- Conformer (30); ZPE = 27358.588 cm^{-1}:

27.81	48.22	73.99	144.95	234.29	261.85
322.57	381.32	393.04	509.08	548.57	583.17
612.91	654.51	687.41	750.05	838.60	907.27
925.18	965.57	1033.16	1140.79	1175.41	1198.48
1204.75	1269.86	1293.37	1333.95	1389.37	1405.26
1439.10	1457.97	1665.35	1860.20	1868.01	3037.00
3069.22	3119.10	3551.77	3641.91	3846.00	3846.75

- Conformer (31); ZPE = 27288.494 cm^{-1}:

39.89	82.61	99.35	150.04	173.47	267.36
329.88	358.08	482.66	488.64	533.61	572.03
611.21	662.37	668.17	779.78	790.06	838.96
904.50	945.18	1037.13	1148.93	1164.88	1181.01
1211.70	1271.37	1297.15	1327.98	1349.11	1418.86
1445.77	1456.83	1645.46	1859.66	1892.33	3013.01
3062.99	3107.66	3557.17	3654.00	3842.87	3853.26

- Conformer (32); ZPE = 27385.475 cm^{-1}:

37.96	69.53	104.17	154.92	255.43	277.02
293.89	354.20	405.29	499.15	569.67	589.45
615.95	626.23	677.38	768.62	828.31	876.41
892.62	996.66	1055.54	1148.60	1155.09	1182.69
1225.84	1249.95	1308.68	1340.22	1389.61	1405.93
1423.11	1442.49	1660.45	1865.76	1881.56	3070.37
3074.96	3125.36	3542.80	3633.40	3845.67	3850.00

- Conformer (33); ZPE = 27530.820 cm^{-1}:

57.67	93.57	104.09	166.47	272.03	300.60
361.97	368.56	422.48	505.83	560.21	601.98
615.54	687.19	745.54	770.86	837.02	869.58
902.77	973.88	1096.74	1128.86	1164.60	1207.20
1232.66	1244.39	1304.32	1354.63	1389.52	1398.26
1431.80	1454.10	1659.72	1879.72	1895.66	3053.35
3058.46	3119.85	3548.50	3642.05	3728.54	3850.86

- Conformer (34); ZPE = 27527.723 cm^{-1}:

13.54	77.05	93.94	167.69	273.04	320.75
325.80	382.69	400.98	506.77	571.27	621.33
628.84	699.51	760.68	843.35	882.82	899.70
939.04	986.08	1013.24	1134.18	1161.54	1189.13
1271.20	1283.21	1307.14	1345.53	1399.46	1409.63
1449.11	1456.35	1655.16	1876.08	1886.77	3028.07
3048.75	3152.53	3535.46	3590.24	3624.90	3842.87

- Conformer (35); ZPE = 27353.254 cm^{-1}:

26.91	62.20	77.62	137.70	227.03	268.37
312.85	369.09	408.87	490.83	561.04	596.52
606.80	638.97	708.60	756.19	832.13	897.89
927.57	965.84	1016.32	1117.51	1171.77	1191.74
1226.69	1274.07	1290.20	1344.04	1390.56	1400.24
1410.84	1457.19	1660.77	1864.52	1876.65	3042.99
3078.99	3133.06	3547.16	3639.88	3846.28	3852.01

- Conformer (36); ZPE = 27572.692 cm^{-1}:

35.16	75.91	156.11	205.09	235.49	292.40
317.51	352.32	414.63	519.64	578.88	622.32
673.72	713.64	726.62	805.17	816.51	870.64
893.00	959.06	1035.23	1142.03	1187.76	1208.19
1241.84	1303.85	1325.95	1338.44	1404.50	1404.90
1431.07	1460.88	1657.55	1821.50	1879.64	3058.03
3092.51	3110.19	3562.28	3649.54	3729.00	3836.67

- Conformer (37); ZPE = 27365.822 cm^{-1}:

37.20	58.79	87.57	154.55	240.60	280.27
331.32	378.19	408.79	522.81	547.16	588.00
602.66	644.13	688.22	749.61	864.66	871.13
898.22	954.22	1057.83	1167.27	1180.67	1194.24
1221.68	1259.90	1290.89	1338.75	1374.19	1384.40
1430.73	1458.98	1656.84	1872.58	1877.37	2959.01
3074.88	3126.46	3562.21	3645.27	3840.88	3848.53

- Conformer (38); ZPE = 27573.195 cm^{-1}:

23.57	74.59	99.78	156.58	256.49	295.99
330.08	382.18	447.39	526.78	585.17	618.73
632.03	702.28	782.94	849.94	888.95	902.55
935.37	976.28	1005.76	1141.26	1151.46	1196.55
1273.72	1282.30	1314.00	1361.31	1383.58	1399.90
1455.83	1456.78	1656.03	1880.58	1888.09	3041.46
3087.16	3134.65	3538.30	3559.82	3626.26	3843.91

- Conformer (39); ZPE = 27482.391 cm^{-1}:

50.54	86.04	130.98	216.64	229.03	239.15
333.66	345.77	452.12	513.91	528.79	637.67
671.15	685.69	731.19	771.46	812.68	846.27
918.33	992.88	1069.73	1157.52	1196.48	1202.03
1222.93	1268.46	1301.63	1361.93	1380.74	1429.59
1451.75	1456.27	1633.79	1830.40	1889.14	3015.42
3050.71	3093.59	3551.94	3653.81	3718.41	3834.58

- Conformer (40); ZPE = 27374.894 cm^{-1}:

39.95	69.49	89.61	149.59	242.24	256.01
302.01	333.88	399.04	514.43	540.06	603.23
633.54	654.03	684.19	791.63	821.16	842.72
917.64	978.54	1110.40	1113.98	1164.56	1189.09
1201.52	1250.84	1299.19	1337.56	1375.71	1425.52
1446.25	1471.94	1649.50	1858.78	1861.35	3057.40
3093.33	3110.64	3542.78	3638.28	3843.23	3844.95

- Conformer (41); ZPE = 27332.179 cm^{-1}:

31.81	60.88	87.57	148.15	218.93	247.07
328.22	350.58	446.03	528.34	537.97	598.20
618.87	658.00	693.07	766.21	794.84	873.57
902.64	929.03	1055.07	1151.48	1163.07	1184.73
1218.56	1266.22	1287.40	1344.86	1369.67	1381.29
1441.99	1458.73	1627.99	1874.28	1877.28	3051.11
3067.62	3112.75	3560.80	3662.36	3842.88	3844.28

- Conformer (42); ZPE = 27369.440 cm^{-1}:

56.16	61.53	113.11	160.98	233.56	252.43
327.54	384.91	474.36	506.42	537.79	577.68
595.12	627.20	707.69	779.22	802.93	846.82
934.59	956.38	1007.07	1125.32	1180.56	1194.18
1210.72	1250.57	1326.57	1342.93	1353.23	1410.38
1443.27	1456.86	1649.43	1869.45	1875.20	2963.93
3076.72	3139.57	3566.16	3657.54	3848.13	3854.67

- Conformer (43); ZPE = 27554.522 cm^{-1}:

39.29	73.13	79.85	239.77	282.50	314.97
346.99	396.47	449.31	540.65	553.11	588.78
618.21	703.26	777.41	817.91	859.65	890.00
942.06	968.96	1004.00	1123.77	1177.94	1198.39
1235.93	1284.81	1335.01	1342.88	1368.26	1435.86
1446.96	1449.65	1643.28	1881.64	1895.38	2986.52
3058.80	3147.36	3551.57	3575.43	3640.47	3842.87

- Conformer (44); ZPE = 27562.796 cm^{-1}:

44.52	77.15	87.42	218.12	296.62	339.89
357.49	381.02	465.37	493.60	548.06	580.16
592.92	687.20	775.81	802.62	832.16	885.45
900.95	944.07	1065.26	1125.01	1170.45	1206.68
1249.70	1260.93	1312.34	1315.69	1401.88	1410.41
1433.73	1444.45	1639.77	1881.72	1886.27	3046.48
3054.30	3101.92	3548.84	3651.69	3708.79	3898.69

- Conformer (45); ZPE = 27388.399 cm^{-1}:

45.17	76.89	105.70	165.29	263.86	271.93
297.93	374.96	399.19	500.35	573.16	579.93
616.85	622.92	685.11	756.88	821.31	868.90
906.35	991.83	1060.41	1138.69	1175.95	1186.09
1232.98	1258.61	1294.40	1335.78	1378.61	1397.09
1447.34	1460.41	1654.61	1874.64	1881.96	2988.19
3056.74	3135.59	3555.15	3642.18	3847.55	3849.31

- Conformer (46); ZPE = 27405.256 cm^{-1}:

24.53	61.32	88.33	161.00	213.88	243.01
316.41	352.85	444.67	509.94	561.29	610.28
621.36	656.41	740.35	781.06	843.05	874.85
899.20	959.06	1034.33	1118.09	1175.28	1194.47
1245.40	1259.18	1291.00	1337.53	1356.98	1385.88
1457.98	1465.25	1649.03	1871.61	1874.81	3031.89
3067.45	3142.88	3554.65	3650.11	3837.46	3846.41

- Conformer (47); ZPE = 27498.385 cm^{-1}:

58.08	86.35	91.12	158.25	258.35	320.45
353.95	375.86	433.94	485.26	549.20	579.68
618.30	673.18	753.69	798.60	877.57	912.68
917.21	938.12	1079.32	1153.01	1172.14	1182.35
1228.72	1270.00	1286.59	1330.88	1395.68	1408.43
1435.58	1449.88	1662.25	1884.82	1888.77	3032.29
3049.96	3089.16	3537.52	3624.92	3701.95	3892.71

- Conformer (48); ZPE = 27377.436 cm^{-1}:

45.48	78.84	97.98	150.85	245.32	256.75
308.93	331.48	427.85	501.45	560.34	610.57
614.14	639.17	718.97	771.73	817.46	831.37
912.95	988.56	1042.20	1149.26	1156.90	1174.93
1233.54	1242.25	1292.00	1336.98	1360.98	1390.13
1456.66	1467.18	1638.77	1871.65	1876.14	3059.06
3062.72	3132.44	3554.40	3653.12	3842.64	3850.73

- Conformer (49); ZPE = 27460.319 cm^{-1}:

23.32	70.66	96.09	170.21	234.81	275.57
294.73	346.78	437.99	531.67	572.35	607.82
616.32	662.29	762.76	803.00	816.98	862.61
903.35	962.16	1012.39	1126.40	1159.80	1198.46
1226.08	1252.21	1320.94	1336.86	1376.20	1396.59
1414.72	1465.23	1663.34	1858.96	1867.89	3076.52
3085.62	3152.06	3549.83	3641.68	3840.84	3846.53

- Conformer (50); ZPE = 27381.125 cm^{-1}:

32.59	80.25	102.36	170.73	236.33	263.64
330.86	366.18	494.09	509.79	543.63	579.29
609.02	655.62	692.38	782.36	800.18	843.37
896.28	940.43	1035.67	1127.53	1179.32	1193.85
1234.23	1258.31	1284.97	1338.85	1370.89	1401.46
1443.06	1453.58	1651.23	1860.19	1875.69	3033.86
3063.90	3130.02	3555.19	3647.63	3846.26	3847.15

- Conformer (51); ZPE = 27351.177 cm^{-1}:

31.60	71.17	92.20	150.93	226.91	252.00
312.13	333.16	425.51	502.09	561.59	591.53
624.49	653.91	725.31	768.75	798.00	830.13
924.20	985.45	1048.84	1142.77	1157.54	1175.02
1223.01	1247.53	1292.32	1334.83	1352.00	1415.00
1456.85	1471.99	1636.67	1866.11	1874.53	3051.30
3064.01	3125.34	3559.07	3658.81	3841.39	3846.40

- Conformer (52); ZPE = 27447.460 cm^{-1}:

37.68	73.04	99.65	175.41	245.57	271.52
287.82	335.45	436.38	529.73	568.11	599.75
606.66	655.42	754.86	802.32	815.49	861.50
889.68	963.43	1008.88	1125.34	1150.52	1202.79
1233.27	1249.90	1327.06	1337.54	1383.96	1392.60
1407.08	1455.08	1662.12	1868.87	1874.86	3076.27
3087.85	3150.82	3557.43	3645.39	3842.15	3845.66

- Conformer (53); ZPE = 27451.020 cm^{-1}:

48.21	68.10	107.83	191.04	263.73	324.38
331.44	365.31	458.01	474.27	527.37	569.13
588.13	614.79	686.46	782.79	832.86	899.19
911.22	959.93	1050.50	1135.99	1183.90	1204.19
1210.18	1277.45	1311.36	1317.83	1398.16	1405.11
1412.19	1435.99	1665.00	1856.03	1885.19	3044.03
3058.95	3111.70	3552.04	3636.37	3846.82	3898.86

- Conformer (54); ZPE = 27399.214 cm^{-1}:

52.79	82.78	104.16	174.99	252.05	265.20
331.39	365.83	456.25	484.17	499.14	583.93
588.30	614.68	683.14	778.67	831.69	902.84
925.35	968.64	1051.33	1139.98	1172.14	1194.13
1205.89	1282.74	1309.49	1310.86	1388.74	1401.24
1416.02	1443.36	1669.67	1877.24	1891.15	3037.67
3055.33	3106.04	3540.45	3620.21	3844.05	3894.70

- Conformer (55); ZPE = 27370.308 cm^{-1}:

31.56	43.82	85.81	143.37	216.11	278.77
332.73	353.36	438.21	539.11	556.51	592.67
599.78	649.55	697.99	779.36	827.49	875.21
906.67	920.03	1053.02	1118.29	1159.14	1190.36
1232.53	1266.09	1286.87	1362.60	1372.79	1379.74
1434.02	1453.58	1644.30	1874.92	1878.88	3048.15
3099.28	3119.03	3555.05	3649.44	3843.97	3850.45

- Conformer (56); ZPE = 27533.799 cm^{-1}:

42.08	61.59	145.76	200.70	243.20	262.09
307.23	396.35	460.99	516.02	560.59	596.48
638.86	685.54	751.00	792.83	817.39	842.58
919.52	959.25	1026.16	1136.76	1164.77	1204.09
1243.72	1298.73	1332.15	1340.78	1380.24	1407.07
1424.75	1466.13	1661.35	1830.23	1880.84	3007.44
3085.93	3153.41	3551.43	3650.95	3781.75	3838.84

- Conformer (57); ZPE = 27532.127 cm^{-1}:

62.07	84.31	125.15	194.56	227.19	277.22
302.14	368.90	437.18	530.29	592.60	603.39
625.68	710.92	769.31	781.84	808.03	852.58
944.26	968.92	1009.59	1129.09	1171.87	1201.01
1223.65	1301.61	1333.88	1348.40	1356.79	1419.45
1462.12	1467.87	1648.77	1842.98	1884.85	2972.92
3074.80	3137.17	3556.64	3655.87	3758.44	3839.96

- Conformer (58); ZPE = 27429.555 cm^{-1}:

34.75	72.88	102.26	162.90	228.05	241.94
308.17	347.05	438.59	530.89	569.80	588.13
608.40	658.74	762.35	811.60	814.10	890.95
900.28	957.79	1022.61	1102.59	1136.45	1208.24
1222.13	1248.58	1285.73	1334.52	1376.54	1409.09
1438.97	1473.27	1662.77	1855.97	1879.55	3073.95
3094.14	3138.79	3544.40	3629.45	3842.01	3849.76

- Conformer (59); ZPE = 27576.712 cm^{-1}:

39.89	76.71	122.16	208.77	249.85	299.59
317.14	376.27	417.92	504.50	576.65	627.99
641.31	727.98	760.08	769.23	825.99	882.86
924.99	988.62	1053.95	1127.29	1180.56	1209.57
1238.58	1298.09	1311.89	1347.62	1376.24	1413.12
1458.74	1468.58	1657.61	1842.55	1887.39	2949.99
3069.34	3139.33	3557.76	3645.18	3742.15	3839.42

- Conformer (60); ZPE = 27372.119 cm^{-1}:

27.17	73.52	95.20	158.49	240.20	256.80
305.37	324.69	415.09	490.60	563.40	617.46
622.46	640.85	700.05	797.41	823.03	851.17
904.32	977.32	1097.52	1113.92	1168.53	1181.68
1218.20	1248.89	1307.16	1337.46	1375.73	1391.57
1439.79	1472.94	1644.75	1866.15	1873.40	3047.91
3085.47	3126.33	3538.65	3634.36	3842.61	3846.62

- Conformer (61); ZPE = 27380.151 cm^{-1}:

52.00	74.63	91.22	158.38	243.61	323.57
346.43	375.34	448.81	481.19	499.97	578.51
599.67	631.11	673.21	743.75	870.54	879.95
924.89	954.21	1066.48	1156.81	1177.76	1188.99
1228.28	1251.35	1280.13	1338.87	1360.80	1399.97
1434.28	1447.33	1631.53	1871.63	1892.24	2970.06
3045.64	3100.32	3564.44	3657.16	3849.03	3896.20

- Conformer (62); ZPE = 27369.724 cm^{-1}:

47.11	72.22	92.24	154.69	250.12	319.69
330.42	368.14	407.83	484.15	546.94	572.08
598.64	618.80	676.55	754.88	879.31	886.04
922.33	968.82	1061.05	1159.03	1176.23	1194.62
1209.33	1262.73	1282.83	1337.02	1362.71	1402.16
1436.20	1463.98	1650.41	1867.98	1886.96	2958.93
3046.53	3096.18	3553.78	3638.46	3848.59	3892.77

- Conformer (63); ZPE = 27565.745 cm^{-1}:

38.16	82.60	116.99	207.74	231.17	251.22
290.35	357.21	411.83	551.15	603.79	619.51
641.19	777.23	785.62	801.48	819.71	867.82
906.25	948.97	1058.38	1115.79	1186.38	1204.19
1221.59	1301.81	1306.78	1343.66	1381.61	1422.92
1462.44	1477.03	1659.76	1829.35	1886.43	3058.82
3075.14	3138.71	3535.70	3630.73	3686.11	3838.17

- Conformer (64); ZPE = 27528.267 cm^{-1}:

39.24	67.34	120.15	202.78	244.12	264.92
299.73	357.38	430.50	500.91	587.32	626.58
636.97	711.77	756.25	775.31	829.20	868.46
927.47	990.31	1032.25	1134.93	1163.43	1198.81
1224.15	1290.77	1314.85	1341.23	1396.17	1402.30
1431.17	1470.25	1656.62	1832.60	1889.77	3046.29
3059.95	3151.86	3542.56	3636.33	3764.66	3838.87

- Conformer (65); ZPE = 27431.694 cm^{-1}:

44.75	69.70	100.51	170.37	237.36	257.98
309.90	333.33	439.43	530.38	565.17	588.06
603.06	654.43	757.00	807.12	811.21	889.75
890.80	959.88	1014.16	1104.21	1128.22	1218.82
1222.44	1246.18	1295.18	1334.77	1374.69	1407.55
1435.56	1459.94	1660.69	1870.47	1884.04	3067.55
3095.29	3138.44	3555.95	3641.93	3838.96	3848.15

- Conformer (66); ZPE = 27518.556 cm^{-1}:

45.80	63.26	109.87	153.53	240.60	315.25
348.28	356.12	452.58	479.04	539.87	593.69
644.70	686.26	758.70	807.08	882.22	906.48
928.31	989.75	1033.39	1142.92	1179.38	1192.63
1248.22	1280.20	1288.69	1348.11	1375.10	1410.97
1434.64	1442.87	1675.21	1888.07	1895.72	3027.54
3044.14	3088.98	3542.80	3614.33	3688.41	3893.40

- Conformer (67); ZPE = 27307.277 cm^{-1}:

37.74	77.81	94.02	146.10	214.00	250.39
281.89	338.44	404.60	513.06	559.61	596.82
612.60	653.86	680.75	785.16	810.15	834.71
919.94	965.11	1082.92	1106.79	1149.12	1185.95
1206.17	1251.75	1300.10	1352.45	1367.16	1402.66
1435.00	1448.85	1650.23	1864.30	1901.66	3055.09
3095.04	3109.18	3544.20	3637.91	3842.74	3848.52

- Conformer (68); ZPE = 27350.966 cm^{-1}:

33.95	60.76	74.13	145.88	238.75	254.38
302.67	321.53	428.64	520.85	567.69	593.91
611.36	620.36	723.65	780.44	822.83	830.35
912.91	984.52	1045.03	1115.82	1140.80	1185.34
1235.56	1251.51	1295.19	1354.91	1374.24	1391.63
1439.75	1463.46	1648.87	1868.73	1880.86	3062.83
3092.71	3132.50	3553.37	3644.88	3846.38	3847.97

- Conformer (69); ZPE = 27394.417 cm^{-1}:

25.81	68.27	95.19	141.13	237.72	264.17
306.41	320.37	438.18	511.41	574.52	600.68
608.30	623.89	750.08	787.41	823.07	846.06
923.85	972.62	1052.70	1104.72	1131.08	1178.66
1235.87	1255.62	1296.74	1343.05	1372.05	1393.62
1431.86	1469.47	1650.06	1871.52	1888.07	3069.80
3094.61	3141.23	3549.67	3642.87	3845.02	3851.40

- Conformer (70); ZPE = 27450.459 cm^{-1}:

47.49	74.72	130.09	182.46	212.90	245.83
298.08	343.78	456.83	495.67	545.49	623.98
636.05	668.83	711.99	759.83	832.73	875.56
919.68	1001.20	1050.00	1149.69	1173.90	1204.93
1220.95	1239.70	1320.87	1344.33	1375.84	1385.59
1426.16	1451.06	1675.54	1887.08	1897.53	3056.44
3061.48	3114.65	3535.55	3614.82	3820.35	3831.30

- Conformer (71); ZPE = 27479.321 cm^{-1}:

51.79	85.85	134.15	177.65	223.79	238.43
310.23	352.15	447.84	500.97	515.37	578.64
657.65	697.79	750.58	786.06	841.20	915.53
935.63	1001.47	1053.29	1163.80	1175.53	1209.40
1221.63	1259.45	1307.77	1344.77	1389.61	1417.39
1433.88	1455.67	1676.07	1859.02	1884.86	3033.96
3057.70	3085.25	3533.69	3615.43	3688.79	3888.88

- Conformer (72); ZPE = 27483.382 cm^{-1}:

52.13	68.15	136.26	169.02	242.94	262.94
313.36	418.18	469.09	492.33	549.15	575.58
603.44	670.09	722.73	797.78	831.05	885.51
922.54	961.25	1020.38	1131.44	1159.23	1173.80
1248.76	1282.48	1315.77	1329.10	1369.50	1391.06
1400.20	1475.74	1673.13	1879.65	1893.17	2998.98
3084.15	3147.84	3539.48	3626.06	3826.85	3856.45

- Conformer (73); ZPE = 27321.818 cm^{-1}:

34.98	57.21	104.29	194.44	276.38	332.63
347.05	382.30	477.47	502.47	527.35	542.02
596.79	613.51	656.85	674.40	788.67	813.44
879.39	937.39	1039.27	1112.77	1182.79	1198.64
1211.84	1266.01	1298.08	1336.39	1375.98	1389.71
1393.71	1432.26	1634.68	1883.48	1888.82	3055.78
3084.75	3135.54	3590.76	3702.64	3840.66	3850.05

- Conformer (74); ZPE = 27577.509 cm^{-1}:

58.26	77.37	102.50	232.80	287.96	321.23
363.78	408.78	443.32	510.20	551.83	569.01
609.02	727.58	807.83	826.07	868.14	900.23
956.52	983.68	1017.97	1113.43	1194.33	1202.79
1233.74	1299.49	1314.87	1330.40	1373.11	1419.01
1458.99	1476.99	1636.90	1871.50	1883.16	2996.36
3064.15	3120.83	3528.27	3556.82	3623.70	3832.08

- Conformer (75); ZPE = 27537.061 cm^{-1}:

58.78	72.97	98.74	174.54	254.22	296.78
320.79	376.64	452.87	488.42	507.99	590.61
659.02	723.81	753.34	862.80	895.83	905.83
943.21	991.55	1024.63	1143.92	1163.80	1188.28
1260.45	1290.35	1316.73	1330.59	1367.05	1425.38
1458.55	1459.82	1666.68	1886.73	1897.70	3029.96
3049.32	3128.01	3521.61	3547.22	3609.96	3878.65

- Conformer (76); ZPE = 27371.379 cm^{-1}:

30.23	62.19	92.93	154.95	245.73	282.40
312.14	349.80	397.16	483.20	552.72	565.66
598.61	648.32	673.94	781.31	834.73	886.74
914.45	997.37	1057.08	1137.10	1161.09	1183.98
1209.25	1253.98	1297.38	1326.08	1382.10	1418.28
1439.97	1442.08	1659.36	1862.05	1893.64	3044.58
3091.35	3103.69	3542.00	3632.50	3843.57	3897.07

- Conformer (77); ZPE = 27316.414 cm^{-1}:

52.04	73.57	89.46	160.61	237.79	289.56
342.09	372.41	443.44	470.76	497.43	536.30
624.67	669.25	673.36	741.16	836.54	879.55
920.46	946.22	1066.09	1161.17	1174.17	1183.70
1226.16	1239.52	1287.96	1317.47	1362.03	1406.98
1437.76	1442.03	1620.68	1860.15	1902.11	2965.04
3043.18	3109.94	3566.38	3665.62	3844.86	3893.17

- Conformer (78); ZPE = 27516.856 cm^{-1}:

37.10	64.67	118.48	199.76	247.74	251.61
306.98	349.58	429.32	495.79	590.41	621.20
635.31	709.70	751.17	780.18	815.99	850.60
927.48	965.36	1090.83	1117.24	1161.39	1195.57
1229.15	1290.27	1307.20	1339.71	1380.92	1401.42
1459.77	1469.84	1660.64	1827.47	1885.76	3052.65
3065.11	3138.91	3552.85	3647.14	3769.17	3842.28

- Conformer (79); ZPE = 27318.673 cm^{-1}:

47.43	66.55	99.62	150.38	229.89	279.58
341.24	362.81	442.48	472.74	517.58	584.34
607.82	637.48	679.93	768.27	806.68	879.74
911.34	941.81	1057.71	1146.28	1160.82	1173.18
1209.97	1267.73	1287.11	1326.77	1364.68	1392.61
1442.69	1454.38	1622.99	1874.79	1894.29	3024.20
3064.44	3080.24	3561.51	3664.41	3845.49	3893.32

- Conformer (80); ZPE = 27521.313 cm^{-1}:

21.51	82.76	111.53	215.48	226.88	256.95
340.00	360.76	422.72	537.57	555.11	624.48
662.26	723.25	774.80	790.59	819.30	835.67
922.80	978.57	1054.34	1131.04	1179.42	1204.26
1239.55	1298.74	1306.13	1349.42	1382.77	1422.00
1459.76	1467.60	1660.38	1820.68	1883.07	3019.97
3055.43	3140.57	3555.15	3647.38	3671.83	3830.14

- Conformer (81); ZPE = 27327.912 cm^{-1}:

42.16	59.91	73.09	155.05	198.22	217.64
307.28	375.00	422.61	506.65	518.59	537.28
624.97	670.40	707.71	750.86	861.48	910.81
920.43	959.88	1038.99	1143.77	1164.57	1181.33
1215.61	1261.41	1301.37	1323.02	1347.32	1429.17
1431.45	1457.86	1669.66	1864.79	1900.54	3045.23
3051.19	3118.68	3538.21	3628.40	3840.80	3882.46

- Conformer (82); ZPE = 27359.712 cm^{-1}:

46.92	70.79	104.07	186.27	252.01	289.61
347.99	351.88	467.48	481.93	531.67	563.10
586.35	619.20	688.04	783.28	814.25	844.31
898.21	942.89	1044.27	1136.36	1176.95	1196.93
1218.99	1269.61	1294.56	1313.32	1376.75	1404.04
1435.60	1459.45	1654.52	1849.90	1886.72	3036.95
3046.92	3096.10	3556.29	3649.83	3846.05	3899.06

- Conformer (83); ZPE = 27312.421 cm^{-1}:

35.95	59.10	98.51	144.68	224.29	292.66
341.35	354.92	431.18	488.40	510.33	556.77
627.75	678.80	693.15	756.42	818.60	883.64
904.64	939.61	1060.03	1146.83	1161.91	1176.34
1211.78	1258.48	1292.59	1327.05	1352.70	1416.36
1444.31	1447.86	1619.95	1857.68	1903.46	3024.28
3042.35	3092.59	3554.97	3657.07	3839.47	3896.05

- Conformer (84); ZPE = 27336.248 cm^{-1}:

43.69	55.72	90.49	171.87	229.90	271.09
326.99	374.18	445.03	478.77	506.18	531.38
601.52	657.14	677.67	787.33	818.65	898.01
922.82	967.95	1045.02	1141.94	1164.35	1184.98
1205.80	1269.32	1292.11	1320.73	1362.65	1409.45
1437.36	1458.71	1671.78	1864.75	1904.84	3026.36
3053.22	3100.38	3537.75	3616.99	3840.72	3906.92

- Conformer (85); ZPE = 27339.106 cm^{-1}:

31.39	54.96	89.11	165.97	235.40	281.71
323.41	366.95	431.21	472.27	514.33	566.09
610.95	629.22	700.83	744.48	828.86	869.19
929.12	977.77	1072.67	1144.97	1165.56	1191.63
1219.29	1255.34	1287.65	1341.56	1353.60	1408.14
1443.14	1463.18	1645.37	1871.43	1893.44	2987.05
3053.77	3103.90	3554.39	3644.03	3846.88	3908.00

- Conformer (86); ZPE = 27363.480 cm^{-1}:

39.72	66.77	107.18	148.52	249.06	279.48
310.26	350.92	401.16	477.12	551.30	574.72
604.77	630.49	675.86	779.07	829.80	888.26
916.47	989.03	1041.33	1133.15	1158.46	1182.90
1214.72	1261.23	1297.74	1350.65	1376.59	1393.24
1422.43	1451.53	1658.98	1879.55	1896.41	3042.29
3087.61	3101.98	3537.72	3628.02	3844.99	3895.44

- Conformer (87); ZPE = 27329.394 cm^{-1}:

20.68	62.58	99.26	157.84	245.09	279.41
340.50	356.51	451.67	482.34	527.11	588.10
603.11	636.34	681.22	763.72	814.59	873.51
911.75	938.92	1064.27	1137.78	1171.07	1179.58
1224.80	1257.36	1287.86	1332.35	1372.75	1396.32
1438.64	1451.29	1644.28	1858.40	1890.60	3026.24
3057.88	3082.77	3558.75	3654.23	3842.00	3895.30

- Conformer (88); ZPE = 27311.627 cm^{-1}:

53.37	65.93	71.67	149.48	208.40	230.18
301.00	371.00	426.98	478.76	513.29	553.37
604.38	661.22	713.79	749.47	860.22	905.11
921.23	963.28	1016.20	1124.46	1161.13	1190.18
1229.29	1262.83	1301.30	1331.11	1358.88	1395.07
1419.53	1457.81	1672.19	1882.73	1901.68	3040.89
3056.94	3121.25	3539.83	3625.45	3849.62	3882.76

- Conformer (89); ZPE = 27313.485 cm^{-1}:

31.80	39.37	95.83	155.18	210.68	263.57
316.89	353.41	421.78	485.55	503.80	579.04
608.52	639.46	686.13	749.84	829.88	907.03
916.90	1000.45	1046.61	1152.34	1170.61	1181.28
1226.28	1243.64	1301.42	1329.94	1375.63	1404.64
1417.99	1454.73	1672.58	1866.73	1899.94	3036.94
3048.88	3094.57	3539.40	3618.83	3841.66	3907.25

- Conformer (90); ZPE = 27444.838 cm^{-1}:

39.74	66.73	109.56	202.95	245.36	253.40
329.93	386.42	423.02	511.83	539.18	614.93
632.81	651.97	725.10	764.50	819.00	864.52
917.24	979.35	1047.98	1123.50	1175.79	1198.32
1238.15	1286.65	1293.75	1330.77	1371.41	1393.49
1434.10	1471.15	1640.38	1884.56	1893.75	2935.68
3066.54	3148.20	3558.79	3652.19	3826.99	3840.03

- Conformer (91); ZPE = 27546.924 cm^{-1}:

35.92	74.88	159.68	206.35	239.76	294.17
317.26	352.20	416.36	497.64	570.38	594.87
633.62	720.77	731.66	806.19	824.66	868.16
899.95	961.25	1035.48	1143.80	1183.02	1207.73
1244.87	1309.86	1317.21	1338.59	1402.32	1409.49
1421.21	1443.02	1657.44	1855.43	1881.53	3039.04
3087.28	3090.60	3562.56	3650.14	3711.59	3895.92

- Conformer (92); ZPE = 27428.370 cm^{-1}:

47.70	78.77	107.90	170.47	243.13	260.79
328.27	426.22	449.15	506.40	552.44	597.88
621.15	664.53	726.38	780.60	796.45	846.36
939.59	949.48	1007.08	1117.34	1169.31	1200.26
1212.97	1280.12	1325.61	1335.92	1343.15	1400.91
1431.58	1476.33	1638.24	1885.11	1904.26	2926.74
3071.35	3129.30	3566.92	3662.83	3827.82	3849.94

- Conformer (93); ZPE = 27434.393 cm^{-1}:

44.13	69.32	104.59	198.85	230.13	236.97
296.36	370.00	421.82	504.86	554.10	608.60
635.31	656.54	717.71	769.80	832.59	895.98
917.21	994.25	1020.23	1149.90	1165.82	1199.11
1228.93	1283.31	1297.53	1340.73	1369.53	1389.34
1411.21	1462.39	1671.79	1885.20	1895.98	3026.27
3049.45	3142.66	3542.23	3622.03	3817.10	3838.94

- Conformer (94); ZPE = 27278.026 cm^{-1}:

28.06	47.65	71.58	145.74	202.54	240.45
323.77	375.71	391.92	483.63	535.23	589.90
602.43	629.38	679.63	754.09	824.40	905.14
922.85	956.79	1039.24	1140.28	1175.37	1188.23
1203.81	1268.40	1293.42	1322.59	1378.38	1408.12
1420.03	1450.67	1665.97	1858.83	1899.94	3038.75
3049.75	3098.15	3552.36	3644.06	3846.41	3902.42

- Conformer (95); ZPE = 27285.946 cm^{-1}:

48.52	84.43	101.66	168.41	222.46	279.56
337.71	359.21	471.72	483.55	497.35	578.87
585.56	610.12	671.96	780.08	803.57	844.87
905.16	945.04	1039.70	1144.14	1161.63	1173.09
1208.54	1278.19	1291.16	1314.84	1345.87	1405.97
1431.19	1455.92	1648.32	1886.74	1902.17	3016.74
3040.75	3088.69	3556.72	3653.59	3852.39	3895.74

- Conformer (96); ZPE = 27373.663 cm^{-1}:

29.97	67.01	85.80	172.70	255.24	272.59
340.47	352.49	444.65	497.67	513.37	543.69
603.23	635.09	713.07	791.98	818.95	898.92
922.07	968.45	1041.24	1148.81	1157.96	1175.34
1228.21	1267.01	1288.33	1335.76	1359.48	1400.13
1405.39	1462.58	1674.64	1873.76	1897.40	3027.22
3066.37	3116.37	3543.78	3619.85	3840.42	3889.86

- Conformer (97); ZPE = 27394.811 cm^{-1}:

48.13	82.77	130.77	216.17	227.12	237.83
331.27	342.92	452.13	484.06	521.37	569.54
649.62	695.51	731.17	771.28	783.60	846.67
921.54	991.86	1065.96	1152.12	1186.45	1204.43
1217.21	1268.33	1294.60	1354.48	1377.00	1409.99
1438.39	1458.31	1632.40	1863.93	1889.58	3023.60
3030.85	3073.70	3554.18	3656.37	3712.26	3890.14

- Conformer (98); ZPE = 27385.222 cm^{-1}:

35.74	67.47	81.62	156.53	204.87	276.15
320.09	365.70	448.02	503.60	518.39	584.05
624.76	669.06	744.80	774.85	857.34	881.97
910.74	955.89	1024.51	1127.83	1170.34	1210.03
1234.26	1253.56	1281.72	1325.29	1347.38	1394.96
1442.24	1469.47	1650.54	1860.27	1906.50	2982.22
3071.45	3138.34	3540.12	3628.37	3840.13	3889.28

- Conformer (99); ZPE = 27350.120 cm^{-1}:

17.94	65.22	76.53	161.47	212.28	276.79
310.29	359.23	460.51	483.17	516.14	578.28
623.10	644.67	737.75	772.10	854.54	876.04
906.58	952.93	1029.40	1120.59	1168.97	1193.48
1233.51	1258.10	1282.94	1338.30	1351.87	1364.44
1454.06	1477.64	1651.34	1875.58	1886.19	3019.64
3057.71	3123.31	3544.18	3633.11	3845.63	3904.72

- Conformer (100); ZPE = 27476.634 cm^{-1}:

29.28	62.48	136.34	200.28	221.38	271.32
310.16	345.79	401.37	513.05	588.11	610.79
631.87	658.83	741.89	798.28	807.89	854.50
882.70	951.79	1029.22	1137.12	1164.93	1194.53
1248.13	1280.13	1328.46	1339.59	1379.13	1394.48
1409.63	1449.76	1662.49	1883.18	1897.22	3052.17
3098.75	3139.40	3559.16	3645.35	3805.10	3837.22

- Conformer (101); ZPE = 27382.048 cm^{-1}:

42.98	55.26	86.54	165.47	228.07	239.84
302.40	387.04	443.15	474.21	503.99	576.39
620.39	649.56	736.46	814.22	825.43	902.62
914.74	968.22	1018.09	1130.81	1168.64	1177.02
1230.63	1257.04	1295.11	1324.30	1379.86	1393.53
1416.52	1467.04	1680.23	1864.81	1900.35	3002.49
3082.77	3147.24	3539.91	3622.42	3839.40	3888.91

- Conformer (102); ZPE = 27310.364 cm^{-1}:

39.50	66.23	87.05	151.72	240.21	255.95
300.07	336.16	397.67	475.97	540.67	561.70
604.48	656.46	672.45	789.72	813.94	840.58
917.65	979.52	1106.86	1114.26	1157.48	1189.57
1201.42	1249.63	1292.45	1337.83	1364.32	1405.74
1438.38	1472.50	1647.52	1859.24	1893.67	3037.49
3086.25	3104.52	3545.65	3641.78	3844.83	3901.59

- Conformer (103); ZPE = 27562.109 cm^{-1}:

46.41	81.46	135.43	200.91	254.08	280.66
323.74	382.55	441.70	541.90	575.25	588.13
633.97	728.07	806.50	819.59	876.63	898.22
953.64	982.35	996.28	1103.62	1157.63	1193.35
1264.83	1274.87	1314.86	1356.12	1380.45	1412.28
1440.40	1450.55	1651.09	1893.22	1906.03	3042.73
3061.19	3139.20	3534.60	3560.43	3616.25	3823.06

- Conformer (104); ZPE = 27369.531 cm^{-1}:

34.77	65.08	86.56	157.47	206.72	249.33
324.92	358.66	446.27	507.01	521.46	577.19
622.75	666.32	743.06	775.10	844.53	882.28
902.32	959.77	1029.71	1113.59	1175.06	1207.10
1244.55	1251.72	1294.84	1325.92	1341.58	1389.33
1442.93	1466.64	1648.07	1869.82	1904.48	2984.71
3070.62	3135.05	3548.22	3642.68	3837.28	3883.60

- Conformer (105); ZPE = 27462.611 cm^{-1}:

44.98	57.50	120.46	169.55	246.01	256.41
292.44	343.32	451.32	514.23	587.97	598.16
621.22	655.55	748.57	782.15	820.33	867.42
924.74	966.52	1023.87	1117.20	1141.08	1188.79
1232.23	1272.20	1317.75	1348.25	1366.09	1385.78
1397.86	1479.25	1654.15	1885.23	1901.26	3055.30
3090.26	3148.04	3541.50	3633.67	3828.12	3848.51

- Conformer (106); ZPE = 27394.165 cm^{-1}:

55.25	73.56	105.57	206.30	222.24	236.09
316.68	356.22	420.87	508.21	563.22	603.36
629.87	660.39	715.32	777.79	801.81	840.56
913.65	981.01	1024.03	1134.09	1150.74	1186.87
1236.45	1280.54	1297.69	1325.65	1359.45	1388.66
1444.86	1469.00	1640.69	1886.71	1898.68	3024.53
3045.89	3136.40	3558.79	3657.71	3810.14	3842.79

- Conformer (107); ZPE = 27319.191 cm^{-1}:

38.44	63.27	97.89	147.38	210.11	249.83
282.80	335.62	437.30	483.31	493.61	589.53
626.01	638.69	717.71	770.77	816.77	904.19
908.60	1001.03	1038.04	1126.42	1166.74	1182.36
1226.50	1238.05	1298.26	1331.44	1361.01	1383.37
1426.05	1471.67	1672.40	1874.81	1903.13	3039.99
3061.19	3124.73	3537.80	3618.97	3843.50	3899.10

- Conformer (108); ZPE = 27279.292 cm^{-1}:

30.16	57.13	87.22	146.27	215.65	268.53
328.52	352.12	438.79	500.21	536.54	544.84
604.73	670.85	699.22	756.65	805.42	878.09
901.54	926.17	1050.71	1151.91	1163.35	1179.62
1219.90	1255.83	1289.82	1331.78	1363.18	1377.00
1435.30	1452.35	1625.77	1878.45	1906.64	3030.09
3037.62	3113.11	3553.77	3655.92	3842.70	3895.10

- Conformer (109); ZPE = 27483.525 cm^{-1}:

20.26	80.08	125.63	199.44	217.06	261.63
337.07	351.45	401.52	565.30	585.16	606.84
611.23	675.52	771.81	787.80	791.04	839.79
890.77	956.90	1010.92	1140.27	1156.83	1190.22
1224.14	1286.71	1338.10	1347.36	1361.56	1387.44
1404.98	1460.08	1663.32	1883.83	1899.25	3056.35
3090.66	3143.46	3551.37	3647.34	3808.51	3838.09

- Conformer (110); ZPE = 27424.502 cm^{-1}:

44.92	70.93	114.57	195.92	209.74	244.05
284.18	360.98	408.93	558.68	577.22	593.14
615.74	649.77	768.60	797.24	801.34	865.98
890.05	955.63	1021.95	1097.52	1143.19	1187.40
1238.66	1286.82	1306.87	1343.20	1357.91	1388.82
1431.14	1469.47	1657.29	1889.36	1903.84	3045.95
3105.71	3138.11	3544.76	3634.42	3809.04	3839.94

- Conformer (111); ZPE = 27271.485 cm^{-1}:

13.49	47.30	64.52	144.87	216.53	228.71
298.91	385.87	436.19	465.50	523.30	574.34
613.06	643.25	707.97	756.62	811.97	893.40
926.18	959.76	999.04	1112.89	1183.83	1205.13
1226.04	1283.91	1297.62	1342.62	1363.74	1383.25
1398.11	1470.36	1666.00	1862.14	1902.37	3005.68
3066.11	3123.56	3554.77	3644.14	3838.82	3901.09

- Conformer (112); ZPE = 27260.208 cm^{-1}:

16.95	52.27	63.05	137.86	225.43	261.11
313.19	377.37	425.83	442.67	496.13	571.69
613.43	631.37	710.59	753.80	817.25	894.46
929.00	968.06	1005.05	1110.48	1176.61	1194.44
1222.85	1283.01	1293.30	1338.99	1359.71	1386.39
1412.17	1468.81	1660.87	1866.59	1904.78	2987.50
3072.03	3128.76	3552.44	3646.83	3845.52	3901.79

- Conformer (113); ZPE = 27477.905 cm^{-1}:

31.20	84.49	99.13	183.77	270.24	332.62
351.05	408.93	442.35	520.19	555.99	582.14
611.82	696.43	728.32	767.99	819.28	890.72
904.98	944.40	996.15	1129.56	1159.42	1192.91
1257.31	1272.95	1301.91	1343.93	1367.41	1389.46
1430.12	1454.98	1636.80	1891.75	1900.95	3034.29
3052.79	3153.24	3549.53	3657.44	3671.80	3885.08

- Conformer (114); ZPE = 27265.838 cm^{-1}:

44.11	63.93	99.28	153.49	208.10	252.46
305.99	330.09	436.95	442.27	501.29	582.70
625.00	635.05	711.65	768.68	815.43	832.31
911.83	991.57	1041.71	1149.65	1161.19	1176.39
1231.05	1237.79	1295.73	1328.67	1354.62	1369.70
1456.94	1469.21	1642.59	1878.83	1901.62	3012.39
3044.34	3126.06	3549.95	3648.56	3846.16	3896.36

- Conformer (115); ZPE = 27321.160 cm^{-1}:

10.85	47.17	75.60	162.35	215.86	250.15
325.14	349.44	445.40	478.24	513.05	570.50
613.78	668.34	737.41	808.27	821.24	884.74
903.31	974.59	1003.73	1118.51	1169.58	1208.99
1232.49	1252.19	1278.56	1322.39	1352.30	1410.34
1445.33	1466.83	1647.29	1871.67	1902.94	2998.66
3063.68	3125.56	3546.58	3636.17	3838.29	3894.79

- Conformer (116); ZPE = 27396.202 cm^{-1}:

53.63	73.21	113.63	152.22	218.06	250.72
268.01	329.27	453.03	509.65	572.58	597.96
628.36	655.09	739.59	793.14	802.48	843.33
922.87	959.29	1063.27	1094.31	1130.89	1170.72
1239.63	1269.26	1304.36	1343.20	1358.35	1374.55
1416.80	1474.98	1648.51	1895.90	1910.28	3052.56
3100.63	3134.13	3548.40	3643.21	3831.11	3851.26

- Conformer (117); ZPE = 27207.942 cm^{-1}:

27.85	47.15	115.86	137.25	169.22	260.53
331.38	357.85	448.98	488.38	508.03	539.21
615.15	646.24	666.53	790.96	796.66	839.44
903.77	951.82	1029.36	1160.15	1164.03	1176.03
1220.90	1257.00	1299.52	1309.01	1345.80	1424.68
1439.19	1459.16	1639.07	1874.65	1920.08	2979.65
3042.34	3085.75	3552.61	3650.60	3842.01	3902.04

- Conformer (118); ZPE = 27263.799 cm^{-1}:

43.48	74.58	93.56	146.95	224.51	258.45
290.19	340.39	402.39	473.98	549.81	563.77
602.85	632.43	673.17	782.82	805.48	832.48
919.34	967.85	1082.13	1107.03	1143.48	1186.75
1199.45	1252.04	1290.21	1349.94	1363.16	1392.29
1416.96	1448.45	1651.30	1895.69	1906.94	3035.30
3081.51	3108.11	3548.47	3643.41	3848.17	3898.31

- Conformer (119); ZPE = 27285.466 cm^{-1}:

27.52	51.55	81.38	158.89	251.12	271.46
296.76	351.83	406.82	452.00	518.83	590.19
605.86	622.87	671.08	766.30	828.43	876.40
895.18	998.79	1050.46	1140.36	1150.06	1177.68
1214.13	1258.86	1299.83	1335.77	1354.47	1410.25
1425.98	1446.15	1658.36	1875.19	1908.97	3011.23
3068.36	3128.34	3543.84	3635.01	3843.36	3910.99

- Conformer (120); ZPE = 27295.938 cm^{-1}:

54.21	77.85	87.13	159.88	234.84	280.73
338.92	375.36	453.66	479.61	501.75	512.45
591.05	639.54	677.44	748.14	830.54	880.23
926.48	943.16	1063.26	1159.70	1170.98	1179.77
1230.39	1244.63	1279.96	1318.16	1351.70	1396.14
1424.62	1440.12	1621.34	1891.59	1906.50	2974.43
3029.19	3089.76	3566.29	3664.76	3895.19	3900.43

- Conformer (121); ZPE = 27305.418 cm^{-1}:

21.39	61.94	98.74	144.45	225.36	295.15
342.35	376.47	425.26	486.62	516.74	540.16
596.65	639.88	702.43	769.05	835.16	886.43
911.53	937.51	1059.58	1144.21	1158.86	1170.32
1210.69	1261.56	1288.15	1328.49	1349.79	1392.84
1439.60	1450.12	1623.14	1888.42	1903.37	3020.77
3037.03	3074.89	3552.04	3653.40	3891.09	3899.21

- Conformer (122); ZPE = 27437.854 cm^{-1}:

59.85	79.48	99.70	220.74	250.19	279.45
302.26	387.14	392.97	486.24	541.00	591.46
626.33	727.99	779.47	802.59	828.13	868.05
932.13	954.91	1005.16	1112.21	1155.37	1200.51
1233.82	1280.50	1300.47	1332.80	1372.97	1430.52
1441.40	1448.20	1647.04	1893.40	1921.09	2967.76
3052.54	3136.03	3549.20	3636.63	3655.81	3892.18

- Conformer (123); ZPE = 27310.280 cm^{-1}:

51.13	54.44	95.23	170.19	266.25	287.21
330.29	382.18	415.74	463.98	482.31	507.64
583.20	607.83	673.80	786.46	827.78	904.30
931.49	970.48	1047.46	1139.40	1168.51	1187.55
1197.80	1268.79	1289.09	1303.43	1363.36	1406.03
1408.32	1460.59	1674.77	1894.32	1906.26	3025.48
3034.68	3085.00	3537.47	3616.01	3895.77	3918.55

- Conformer (124); ZPE = 27327.689 cm^{-1}:

54.74	88.11	105.07	155.78	201.17	248.65
302.61	403.88	454.45	490.72	514.64	554.08
593.50	636.75	717.04	804.92	830.64	886.07
919.85	960.62	1008.69	1120.69	1147.62	1173.03
1235.48	1262.71	1284.75	1315.96	1367.27	1379.84
1394.33	1471.37	1678.02	1888.77	1917.25	2985.74
3064.55	3139.78	3538.38	3622.23	3857.84	3877.80

- Conformer (125); ZPE = 27284.378 cm^{-1}:

57.31	86.63	112.42	161.04	191.33	258.89
327.90	342.27	434.82	496.68	522.60	569.77
626.89	645.17	710.89	753.14	812.65	843.18
892.91	990.88	1039.45	1138.17	1174.84	1190.88
1225.29	1240.49	1289.68	1313.74	1333.85	1374.80
1431.83	1466.28	1642.23	1897.87	1919.71	2970.00
3038.63	3129.99	3547.94	3648.69	3839.88	3877.11

- Conformer (126); ZPE = 27249.052 cm^{-1}:

58.55	77.62	100.32	147.27	223.31	263.66
332.88	410.21	466.80	483.16	502.23	568.57
582.86	621.13	695.14	766.53	804.15	841.76
930.68	942.50	1007.56	1111.88	1159.90	1185.69
1213.42	1250.65	1291.34	1307.30	1327.51	1392.48
1423.79	1473.86	1632.44	1902.41	1913.77	2927.90
3039.35	3123.69	3572.49	3672.21	3864.34	3884.79

- Conformer (127); ZPE = 27129.396 cm^{-1}:

26.48	48.61	116.70	142.65	172.68	258.32
332.78	355.56	435.49	458.70	486.64	520.57
582.06	617.89	656.65	793.56	799.77	842.84
904.69	949.39	1027.64	1158.85	1161.03	1162.82
1223.73	1259.12	1287.56	1304.66	1330.44	1410.05
1428.20	1458.07	1639.90	1903.08	1927.24	2978.91
3024.13	3065.95	3551.44	3650.04	3901.70	3902.21

1.5 Cartesian coordinates of Asp conformers

Each line starting by an asterisk contains the relative energy of the corresponding conformer and the dihedral angle for each of the target torsions (separated by underscores). Cartesian coordinates are given in Å.

```
16
 * E =  +0.000 kcal/mol ; (1) 353_022_082_297_172_180
 H    +0.00000    +0.00000    +0.00000
 O    +0.00000    +0.00000    +0.97110
 C    +1.26631    +0.00000    +1.36190
 C    +2.28454    -0.16107    +0.22089
 N    +1.68152    +0.23794    -1.04246
 H    +1.70874    +1.24620    -1.14789
 O    +1.60134    +0.06997    +2.50819
 C    +3.58558    +0.53557    +0.61180
 H    +2.47767    -1.23990    +0.16979
 H    +2.12590    -0.19740    -1.83818
 C    +3.37200    +2.01537    +0.79305
 H    +3.97111    +0.13059    +1.55123
 H    +4.35803    +0.38848    -0.15087
 O    +4.44264    +2.63003    +1.29904
 H    +4.23439    +3.56670    +1.39526
```

O +2.36127 +2.59853 +0.50415
16
∗ E = +0.504 kcal/mol ; (2) 355_022_083_169_176_179
H +0.00000 +0.00000 +0.00000
O +0.00000 +0.00000 +0.97160
C +1.26896 +0.00000 +1.36434
C +2.27820 −0.11555 +0.21552
N +1.66996 +0.32236 −1.03473
H +1.71169 +1.33159 −1.13028
O +1.59675 +0.04924 +2.51316
C +3.58500 +0.56848 +0.59097
H +2.47438 −1.19072 +0.11023
H +2.16418 −0.08054 −1.82139
C +4.66993 +0.23870 −0.39746
H +3.46707 +1.65790 +0.63656
H +3.90555 +0.26101 +1.59169
O +5.80945 +0.88720 −0.13547
H +6.46256 +0.62739 −0.79545
O +4.55174 −0.51732 −1.32379
16
∗ E = +0.666 kcal/mol ; (3) 181_199_301_301_183_180
H +0.00000 +0.00000 +0.00000
O +0.00000 +0.00000 +0.96398
C +1.27903 +0.00000 +1.37595
C +1.40401 −0.02876 +2.89904
N +2.70801 +0.39861 +3.35911
H +3.42360 −0.20474 +2.97149
O +2.20622 −0.02940 +0.62227
C +0.29704 +0.76975 +3.58062
H +1.26621 −1.08158 +3.18038
H +2.89484 +1.32743 +2.99307
C +0.30431 +2.20586 +3.13200
H −0.69319 +0.35722 +3.36684
H +0.43985 +0.74204 +4.66452
O −0.61390 +2.94518 +3.76261
H −0.55466 +3.84666 +3.42602
O +1.04132 +2.65632 +2.29741
16
∗ E = +0.770 kcal/mol ; (4) 179_023_295_297_182_181
H +0.00000 +0.00000 +0.00000

O	+0.00000	+0.00000	+0.96388
C	+1.27414	+0.00000	+1.37054
C	+1.41217	+0.02173	+2.89356
N	+0.27762	−0.47709	+3.64352
H	−0.53336	+0.11068	+3.50029
O	+2.20208	+0.02799	+0.61166
C	+2.68922	−0.71876	+3.27376
H	+1.55280	+1.07949	+3.15242
H	+0.03708	−1.40658	+3.31626
C	+2.61228	−2.16209	+2.85335
H	+3.56430	−0.27636	+2.78953
H	+2.84124	−0.67525	+4.35549
O	+3.70109	−2.85546	+3.19772
H	+3.58771	−3.76230	+2.89083
O	+1.68460	−2.65652	+2.27066

16

∗ E = +1.288 kcal/mol ; (5) 183_194_206_174_172_179

H	+0.00000	+0.00000	+0.00000
O	+0.00000	+0.00000	+0.96383
C	+1.27608	+0.00000	+1.37610
C	+1.39697	−0.06828	+2.88456
N	+2.75368	+0.25932	+3.26412
H	+2.98766	−0.17041	+4.14884
O	+2.20286	+0.01223	+0.61865
C	+0.40916	+0.87133	+3.57134
H	+1.10822	−1.10201	+3.14706
H	+3.40592	−0.03977	+2.54963
C	+0.45854	+0.67631	+5.06280
H	+0.65608	+1.91409	+3.34833
H	−0.61962	+0.69674	+3.24000
O	−0.26509	+1.59348	+5.71601
H	−0.19895	+1.40844	+6.65977
O	+1.05541	−0.19926	+5.62686

16

∗ E = +1.750 kcal/mol ; (6) 179_041_192_172_178_180

H	+0.00000	+0.00000	+0.00000
O	+0.00000	+0.00000	+0.96373
C	+1.27481	+0.00000	+1.37887
C	+1.37869	+0.02862	+2.89181
N	+0.38720	−0.85289	+3.48755

```
 H    +0.33736    -0.66926    +4.48280
 O    +2.20255    +0.01770    +0.62223
 C    +2.78070    -0.38526    +3.30804
 H    +1.23274    +1.08823    +3.17254
 H    -0.52812    -0.68793    +3.08829
 C    +2.99201    -0.18300    +4.78214
 H    +2.94967    -1.44102    +3.07201
 H    +3.53953    +0.18373    +2.76201
 O    +4.20302    -0.58965    +5.17979
 H    +4.27408    -0.43231    +6.12815
 O    +2.18949    +0.29174    +5.54031
16
 * E =  +1.811  kcal/mol  ;  (7)  002_343_164_173_188_181
 H    +0.00000    +0.00000    +0.00000
 O    +0.00000    +0.00000    +0.97188
 C    +1.26975    +0.00000    +1.36745
 C    +2.29052    +0.04194    +0.22333
 N    +1.65190    -0.33945    -1.03174
 H    +2.23455    -0.06281    -1.81388
 O    +1.59721    +0.01246    +2.51612
 C    +2.80658    +1.47720    +0.15417
 H    +3.12214    -0.61323    +0.51284
 H    +1.55810    -1.34749    -1.08927
 C    +3.94923    +1.63646    -0.81349
 H    +2.00526    +2.16002    -0.15665
 H    +3.13408    +1.80572    +1.14506
 O    +4.49472    +2.85396    -0.74465
 H    +5.20732    +2.90328    -1.39246
 O    +4.33653    +0.79199    -1.57468
16
 * E =  +2.142  kcal/mol  ;  (8)  178_169_287_171_039_358
 H    +0.00000    +0.00000    +0.00000
 O    +0.00000    +0.00000    +0.96437
 C    +1.26485    +0.00000    +1.39277
 C    +1.35542    +0.05060    +2.91104
 N    +2.70600    -0.21530    +3.38418
 H    +2.94014    -1.19585    +3.28382
 O    +2.21452    -0.01444    +0.66335
 C    +0.88511    +1.43415    +3.38935
 H    +0.67701    -0.71091    +3.31371
```

```
H    +3.37019    +0.29767    +2.81069
C    +0.72291    +1.55555    +4.90223
H    +1.62012    +2.18471    +3.06870
H    −0.07402    +1.69525    +2.94080
O    +1.64995    +0.94704    +5.63846
H    +2.28479    +0.48327    +5.05876
O    −0.17200    +2.17723    +5.39283
16
* E =  +2.283  kcal/mol ; (9) 354_021_084_302_349_177
H    +0.00000    +0.00000    +0.00000
O    +0.00000    +0.00000    +0.97103
C    +1.26762    +0.00000    +1.36185
C    +2.28579    −0.13847    +0.21900
N    +1.67793    +0.25947    −1.04447
H    +1.71829    +1.26398    −1.17041
O    +1.60252    +0.06536    +2.50781
C    +3.59272    +0.54987    +0.61160
H    +2.48546    −1.21569    +0.15576
H    +2.11415    −0.18952    −1.83739
C    +3.46745    +2.01835    +0.94754
H    +4.02003    +0.07316    +1.49692
H    +4.32965    +0.45830    −0.19314
O    +2.26180    +2.52724    +0.61717
H    +2.23553    +3.44730    +0.90473
O    +4.33210    +2.68284    +1.43259
16
* E =  +2.490  kcal/mol ; (10) 183_075_298_063_187_180
H    +0.00000    +0.00000    +0.00000
O    +0.00000    +0.00000    +0.96390
C    +1.27265    +0.00000    +1.38203
C    +1.36572    −0.06935    +2.90116
N    +1.06653    −1.41099    +3.38637
H    +0.11935    −1.67898    +3.14879
O    +2.20799    −0.01978    +0.63265
C    +2.75515    +0.34703    +3.34327
H    +0.62269    +0.62175    +3.31123
H    +1.69547    −2.09241    +2.97593
C    +3.02981    +1.77946    +2.97475
H    +2.85177    +0.23195    +4.42711
H    +3.52056    −0.28285    +2.87756
```

O +4.29749 +2.13405 +3.21667
H +4.40111 +3.05754 +2.96075
O +2.21454 +2.53814 +2.52938
16
* E = +2.507 kcal/mol ; (11) 003_167_289_078_173_180
H +0.00000 +0.00000 +0.00000
O +0.00000 +0.00000 +0.97005
C +1.24202 +0.00000 +1.44722
C +2.37374 +0.06228 +0.40460
N +3.65683 +0.36130 +1.00474
H +3.67650 +1.32475 +1.31699
O +1.46676 −0.05910 +2.62046
C +2.48090 −1.26600 −0.35858
H +2.12774 +0.84794 −0.32091
H +3.76447 −0.19733 +1.84668
C +1.44681 −1.44296 −1.43354
H +3.46764 −1.31063 −0.83040
H +2.42813 −2.12860 +0.31703
O +1.65623 −2.53246 −2.16636
H +0.96050 −2.59352 −2.83183
O +0.52550 −0.69264 −1.64683
16
* E = +2.640 kcal/mol ; (12) 180_198_202_306_325_357
H +0.00000 +0.00000 +0.00000
O +0.00000 +0.00000 +0.96436
C +1.26106 +0.00000 +1.39371
C +1.34747 +0.00175 +2.91268
N +2.69146 +0.43885 +3.27109
H +2.94707 +0.13863 +4.20240
O +2.21536 −0.02711 +0.67101
C +0.28383 +0.90105 +3.55409
H +1.13864 −1.03759 +3.21295
H +3.36741 +0.08093 +2.60441
C +0.26824 +2.33753 +3.03826
H −0.71614 +0.49584 +3.39771
H +0.46238 +0.93452 +4.63538
O +1.45628 +2.85149 +2.71790
H +2.16783 +2.21397 +2.91954
O −0.74247 +2.96562 +2.93546
16

∗ E = +2.733 kcal/mol ; (13) 355_021_087_176_325_183

H	+0.00000	+0.00000	+0.00000
O	+0.00000	+0.00000	+0.97099
C	+1.26809	+0.00000	+1.36638
C	+2.28555	−0.11636	+0.22295
N	+1.67717	+0.28630	−1.03992
H	+1.74735	+1.28743	−1.18710
O	+1.59479	+0.05082	+2.51480
C	+3.56581	+0.62545	+0.59739
H	+2.50843	−1.18762	+0.14724
H	+2.11747	−0.17583	−1.82468
C	+4.65491	+0.47684	−0.43208
H	+3.38043	+1.69453	+0.73406
H	+3.93814	+0.24443	+1.55463
O	+4.65312	−0.74209	−1.00867
H	+5.39927	−0.77929	−1.61815
O	+5.45204	+1.31923	−0.72353

16

∗ E = +2.739 kcal/mol ; (14) 352_025_091_293_088_352

H	+0.00000	+0.00000	+0.00000
O	+0.00000	+0.00000	+0.97348
C	+1.26008	+0.00000	+1.35896
C	+2.27445	−0.20048	+0.22221
N	+1.69042	+0.23664	−1.03717
H	+1.89051	+1.21815	−1.21266
O	+1.59515	+0.11774	+2.50809
C	+3.62123	+0.43855	+0.59738
H	+2.41640	−1.28744	+0.16929
H	+2.05315	−0.28930	−1.82048
C	+3.47028	+1.94737	+0.66589
H	+3.95932	+0.02933	+1.55256
H	+4.35548	+0.21326	−0.17746
O	+3.07837	+2.45035	+1.83614
H	+2.82809	+1.74503	+2.44925
O	+3.63910	+2.64917	−0.29116

16

∗ E = +2.762 kcal/mol ; (15) 182_196_300_301_003_179

H	+0.00000	+0.00000	+0.00000
O	+0.00000	+0.00000	+0.96403
C	+1.27695	+0.00000	+1.37993

C +1.39231 −0.04991 +2.90258
N +2.71098 +0.30537 +3.38001
H +3.39986 −0.33290 +3.00047
O +2.20739 −0.00184 +0.62901
C +0.30191 +0.76471 +3.59865
H +1.20890 −1.10095 +3.16561
H +2.95826 +1.22601 +3.03265
C +0.25790 +2.23421 +3.25365
H −0.69077 +0.36732 +3.37074
H +0.44103 +0.69311 +4.68042
O +1.16662 +2.58003 +2.32075
H +1.06569 +3.52124 +2.13734
O −0.49757 +3.02452 +3.73611
16
* E = +2.782 kcal/mol ; (16) 179_174_273_167_151_178
H +0.00000 +0.00000 +0.00000
O +0.00000 +0.00000 +0.96400
C +1.27368 +0.00000 +1.38355
C +1.37793 +0.02528 +2.90063
N +2.73639 −0.12384 +3.37414
H +2.98764 −1.09797 +3.47252
O +2.20709 −0.00132 +0.63308
C +0.78529 +1.33358 +3.43055
H +0.75920 −0.79018 +3.29224
H +3.38210 +0.28490 +2.70657
C +0.58555 +1.22950 +4.92236
H +1.44509 +2.17673 +3.20781
H −0.19171 +1.54058 +2.97920
O +0.66926 +2.42156 +5.52819
H +0.50539 +2.28675 +6.46860
O +0.34295 +0.21611 +5.51196
16
* E = +2.801 kcal/mol ; (17) 177_160_047_175_178_180
H +0.00000 +0.00000 +0.00000
O +0.00000 +0.00000 +0.96395
C +1.27505 +0.00000 +1.37987
C +1.37839 +0.07428 +2.88916
N +2.67986 −0.40006 +3.28614
H +3.41155 +0.01726 +2.72419
O +2.20772 −0.00139 +0.63050

C	+1.04418	+1.52355	+3.28729
H	+0.60824	−0.57824	+3.31682
H	+2.84339	−0.24145	+4.27087
C	+0.99969	+1.68425	+4.78348
H	+1.79362	+2.21723	+2.88755
H	+0.07373	+1.83761	+2.88506
O	+0.75417	+2.95044	+5.14389
H	+0.73456	+2.98853	+6.10712
O	+1.15875	+0.80250	+5.58102

16

* E = +3.060 kcal/mol ; (18) 353_028_083_069_215_180

H	+0.00000	+0.00000	+0.00000
O	+0.00000	+0.00000	+0.96986
C	+1.26856	+0.00000	+1.36648
C	+2.28882	−0.17094	+0.23482
N	+1.73486	+0.34882	−1.00761
H	+1.84314	+1.35414	−1.07783
O	+1.59554	+0.10465	+2.51155
C	+3.62789	+0.40952	+0.68363
H	+2.41497	−1.25575	+0.12251
H	+2.16639	−0.07075	−1.81966
C	+4.23015	−0.44905	+1.77139
H	+4.32953	+0.40573	−0.15808
H	+3.52138	+1.43737	+1.04025
O	+4.89635	+0.26502	+2.68110
H	+5.24396	−0.34564	+3.34119
O	+4.16164	−1.64563	+1.79296

16

* E = +3.167 kcal/mol ; (19) 180_175_296_179_309_179

H	+0.00000	+0.00000	+0.00000
O	+0.00000	+0.00000	+0.96405
C	+1.27404	+0.00000	+1.38459
C	+1.37254	+0.00688	+2.90472
N	+2.72358	−0.10770	+3.39875
H	+3.13111	−0.99789	+3.14148
O	+2.20924	−0.00097	+0.63772
C	+0.73641	+1.29157	+3.44850
H	+0.78404	−0.84249	+3.27176
H	+3.31009	+0.60957	+2.98502
C	+0.78380	+1.31701	+4.95595

H +1.27270 +2.17380 +3.08873
H −0.30673 +1.36241 +3.12491
O +0.29291 +0.18699 +5.48916
H +0.36651 +0.25686 +6.44725
O +1.17787 +2.23095 +5.61906
16
* E = +3.185 kcal/mol ; (20) 181_193_208_290_049_183
H +0.00000 +0.00000 +0.00000
O +0.00000 +0.00000 +0.96352
C +1.27249 +0.00000 +1.37628
C +1.38483 −0.03595 +2.89103
N +2.74995 +0.27261 +3.25815
H +3.01469 −0.14785 +4.13744
O +2.20612 −0.03786 +0.62853
C +0.41713 +0.92913 +3.58610
H +1.07197 −1.06097 +3.15708
H +3.39089 −0.02215 +2.53134
C +0.82279 +2.36631 +3.36904
H −0.60173 +0.78623 +3.21220
H +0.41851 +0.75182 +4.66289
O +1.06181 +2.61887 +2.07191
H +1.36281 +3.53058 +1.99227
O +0.90642 +3.20162 +4.22040
16
* E = +3.266 kcal/mol ; (21) 181_013_059_298_185_181
H +0.00000 +0.00000 +0.00000
O +0.00000 +0.00000 +0.96401
C +1.26928 +0.00000 +1.36104
C +1.45193 −0.01502 +2.87419
N +0.22354 −0.33632 +3.56567
H −0.12050 −1.24589 +3.28082
O +2.19798 +0.03420 +0.60052
C +2.65908 −0.90725 +3.18880
H +1.72924 +1.01601 +3.13222
H +0.35458 −0.33232 +4.56835
C +2.41096 −2.32195 +2.73800
H +3.55748 −0.54704 +2.67715
H +2.87410 −0.90954 +4.26208
O +3.40156 −3.15234 +3.08078
H +3.18603 −4.02964 +2.74392

O +1.43836 −2.68527 +2.13417
16
∗ E = +3.307 kcal/mol ; (22) 178_020_295_294_008_177
H +0.00000 +0.00000 +0.00000
O +0.00000 +0.00000 +0.96399
C +1.27591 +0.00000 +1.36926
C +1.41692 +0.04063 +2.89146
N +0.25766 −0.37172 +3.65575
H −0.51329 +0.26815 +3.51559
O +2.20123 +0.00707 +0.60765
C +2.66781 −0.73257 +3.30159
H +1.60929 +1.09704 +3.12272
H −0.05454 −1.28709 +3.35353
C +2.62516 −2.21595 +3.02281
H +3.54242 −0.34702 +2.76961
H +2.84499 −0.60933 +4.37204
O +1.55014 −2.58545 +2.29526
H +1.61871 −3.53231 +2.12699
O +3.44876 −3.00174 +3.38557
16
∗ E = +3.483 kcal/mol ; (23) 182_195_304_078_207_183
H +0.00000 +0.00000 +0.00000
O +0.00000 +0.00000 +0.96404
C +1.27190 +0.00000 +1.38777
C +1.37898 −0.04666 +2.90919
N +2.70289 +0.28765 +3.38766
H +3.38812 −0.32885 +2.96658
O +2.20768 +0.02824 +0.64121
C +0.32205 +0.84380 +3.56163
H +1.14693 −1.08135 +3.19667
H +2.95605 +1.22540 +3.09425
C −1.04118 +0.19775 +3.57658
H +0.61521 +0.99925 +4.60585
H +0.25534 +1.82808 +3.08927
O −2.02761 +1.10412 +3.58873
H −2.86433 +0.62790 +3.63799
O −1.24182 −0.98209 +3.61691
16
∗ E = +3.601 kcal/mol ; (24) 180_056_185_068_197_181
H +0.00000 +0.00000 +0.00000

O	+0.00000	+0.00000	+0.96378
C	+1.27415	+0.00000	+1.38243
C	+1.35855	−0.01002	+2.89906
N	+0.60598	−1.15916	+3.39095
H	+0.57456	−1.15427	+4.40297
O	+2.20820	+0.00670	+0.63580
C	+2.81491	−0.11068	+3.31563
H	+0.95871	+0.96196	+3.23051
H	−0.34830	−1.12831	+3.05281
C	+3.57474	+1.14075	+2.95979
H	+2.88710	−0.24468	+4.40155
H	+3.29384	−0.97523	+2.84998
O	+4.89884	+0.94572	+2.96822
H	+5.32152	+1.78191	+2.74203
O	+3.07539	+2.20556	+2.72688

16

* E = +3.601 kcal/mol ; (25) 177_266_302_058_181_180

H	+0.00000	+0.00000	+0.00000
O	+0.00000	+0.00000	+0.96390
C	+1.27935	+0.00000	+1.37759
C	+1.40786	+0.06359	+2.89542
N	+1.56496	+1.44334	+3.33951
H	+2.37826	+1.86609	+2.90817
O	+2.20615	+0.04475	+0.62268
C	+0.22887	−0.56579	+3.61769
H	+2.32438	−0.48316	+3.13577
H	+0.75493	+1.99902	+3.08673
C	+0.04456	−2.00370	+3.21992
H	+0.38489	−0.50885	+4.69926
H	−0.70124	−0.02715	+3.40453
O	−1.00339	−2.57200	+3.82988
H	−1.05917	−3.48750	+3.53323
O	+0.74915	−2.59384	+2.44965

16

* E = +3.627 kcal/mol ; (26) 179_173_322_299_087_185

H	+0.00000	+0.00000	+0.00000
O	+0.00000	+0.00000	+0.96369
C	+1.27137	+0.00000	+1.38564
C	+1.35733	+0.03297	+2.91002
N	+2.69688	−0.11721	+3.42035

```
 H    +3.21936    −0.78526    +2.86679
 O    +2.21180    −0.04146    +0.64682
 C    +0.72068    +1.33220    +3.44204
 H    +0.72942    −0.79554    +3.26456
 H    +3.20374    +0.76099    +3.38673
 C    +1.47426    +2.51994    +2.89943
 H    −0.32573    +1.38280    +3.13680
 H    +0.80650    +1.33503    +4.52938
 O    +1.00991    +2.90318    +1.70044
 H    +1.57979    +3.60761    +1.36994
 O    +2.41789    +3.03930    +3.42565
16
 * E =   +3.753  kcal/mol  ;  (27)  176_174_296_067_051_180
 H    +0.00000    +0.00000    +0.00000
 O    +0.00000    +0.00000    +0.96422
 C    +1.26971    +0.00000    +1.39282
 C    +1.35296    +0.08979    +2.91313
 N    +2.70135    −0.04617    +3.41374
 H    +3.06627    −0.96708    +3.20220
 O    +2.21218    −0.04378    +0.65645
 C    +0.76709    +1.43350    +3.37682
 H    +0.72784    −0.71137    +3.32431
 H    +3.31230    +0.61127    +2.93964
 C    −0.71449    +1.58615    +3.14178
 H    +0.94874    +1.50165    +4.45422
 H    +1.27367    +2.27160    +2.89142
 O    −1.40441    +0.54087    +3.62651
 H    −2.33832    +0.69346    +3.44522
 O    −1.24124    +2.52745    +2.62406
16
 * E =   +3.816  kcal/mol  ;  (28)  182_324_061_172_176_179
 H    +0.00000    +0.00000    +0.00000
 O    +0.00000    +0.00000    +0.96382
 C    +1.27529    +0.00000    +1.37967
 C    +1.38299    −0.04775    +2.89140
 N    +0.32740    +0.72446    +3.51142
 H    −0.58862    +0.36930    +3.26581
 O    +2.20542    −0.02916    +0.62722
 C    +1.43319    −1.53390    +3.28461
 H    +2.34451    +0.40662    +3.14684
```

H +0.43606 +0.71376 +4.51759
C +1.72457 −1.70125 +4.75224
H +0.48165 −2.03015 +3.05869
H +2.20978 −2.06813 +2.72384
O +1.68883 −2.98609 +5.12731
H +1.89290 −3.02869 +6.06873
O +1.96748 −0.80923 +5.51677
16
∗ E = +3.838 kcal/mol ; (29) 355_020_089_088_298_000
H +0.00000 +0.00000 +0.00000
O +0.00000 +0.00000 +0.97354
C +1.25756 +0.00000 +1.36138
C +2.28793 −0.11293 +0.22997
N +1.65648 +0.28695 −1.02293
H +1.74061 +1.28576 −1.17680
O +1.57538 +0.06575 +2.52081
C +3.58603 +0.64277 +0.58124
H +2.50555 −1.18725 +0.15420
H +2.07149 −0.18187 −1.81686
C +4.60016 −0.19130 +1.35872
H +4.08460 +0.93785 −0.34301
H +3.34564 +1.54619 +1.15442
O +4.21047 −0.62637 +2.55113
H +3.30127 −0.33993 +2.75110
O +5.67988 −0.44801 +0.91142
16
∗ E = +3.864 kcal/mol ; (30) 177_034_295_160_150_178
H +0.00000 +0.00000 +0.00000
O +0.00000 +0.00000 +0.96391
C +1.27729 +0.00000 +1.37441
C +1.38963 +0.06478 +2.89389
N +0.34669 −0.62762 +3.62678
H −0.55690 −0.19711 +3.48048
O +2.20021 −0.02054 +0.61024
C +2.75575 −0.42965 +3.34217
H +1.32324 +1.13099 +3.15265
H +0.27497 −1.59534 +3.33438
C +3.06103 +0.07450 +4.72949
H +2.80651 −1.52228 +3.32575
H +3.53554 −0.06073 +2.66760

O +3.84860 −0.76690 +5.41377
H +4.02908 −0.36788 +6.27258
O +2.68569 +1.11970 +5.17782
16
∗ E = +3.960 kcal/mol ; (31) 180_186_042_302_192_182
H +0.00000 +0.00000 +0.00000
O +0.00000 +0.00000 +0.96351
C +1.28256 +0.00000 +1.37032
C +1.37701 −0.00003 +2.88843
N +2.76392 +0.15436 +3.26117
H +3.21635 +0.84429 +2.67272
O +2.20647 −0.02740 +0.61699
C +0.40627 +1.01839 +3.50140
H +1.02775 −0.99598 +3.19903
H +2.86787 +0.41690 +4.23161
C +0.65911 +2.41114 +2.98944
H −0.63404 +0.76459 +3.26971
H +0.49398 +1.01862 +4.59251
O +0.00609 +3.34231 +3.69717
H +0.19203 +4.20167 +3.30151
O +1.35229 +2.67682 +2.04799
16
∗ E = +3.976 kcal/mol ; (32) 182_063_300_058_024_180
H +0.00000 +0.00000 +0.00000
O +0.00000 +0.00000 +0.96382
C +1.27427 +0.00000 +1.38337
C +1.36336 −0.04771 +2.90369
N +0.79015 −1.27002 +3.45063
H −0.18714 −1.35587 +3.19973
O +2.20829 −0.00155 +0.63326
C +2.81437 +0.07726 +3.33990
H +0.78317 +0.79900 +3.28673
H +1.27489 −2.08720 +3.09653
C +3.50752 +1.33361 +2.87599
H +2.85419 +0.05475 +4.43367
H +3.40685 −0.76547 +2.97291
O +2.64088 +2.32991 +2.61317
H +3.15206 +3.09357 +2.32274
O +4.69137 +1.46930 +2.78431
16

* E =	+4.000 kcal/mol ;	(33) 353_026_084_061_038_181	
H	+0.00000	+0.00000	+0.00000
O	+0.00000	+0.00000	+0.97017
C	+1.26869	+0.00000	+1.36638
C	+2.28913	−0.16146	+0.23232
N	+1.71326	+0.32070	−1.01742
H	+1.81290	+1.32534	−1.11081
O	+1.59566	+0.09049	+2.51218
C	+3.61598	+0.48229	+0.63351
H	+2.43637	−1.24416	+0.13473
H	+2.14305	−0.11072	−1.82423
C	+4.26219	−0.13047	+1.85432
H	+4.32710	+0.37523	−0.19413
H	+3.49190	+1.54958	+0.83514
O	+4.10887	−1.46561	+1.88321
H	+4.52281	−1.79194	+2.68987
O	+4.89669	+0.47138	+2.66759
16			
* E =	+4.142 kcal/mol ;	(34) 179_046_297_167_037_359	
H	+0.00000	+0.00000	+0.00000
O	+0.00000	+0.00000	+0.96410
C	+1.27455	+0.00000	+1.38607
C	+1.34860	+0.02966	+2.90927
N	+0.43617	−0.92084	+3.54622
H	−0.52996	−0.70284	+3.33374
O	+2.20948	−0.01637	+0.64203
C	+2.78089	−0.20154	+3.38023
H	+1.02437	+1.03641	+3.20838
H	+0.61580	−1.86488	+3.21977
C	+3.01802	+0.10660	+4.85491
H	+3.05362	−1.25052	+3.20297
H	+3.47813	+0.40269	+2.80022
O	+2.02282	−0.19958	+5.68897
H	+1.27230	−0.57829	+5.19578
O	+4.04289	+0.57986	+5.24617
16			
* E =	+4.159 kcal/mol ;	(35) 179_044_301_177_308_179	
H	+0.00000	+0.00000	+0.00000
O	+0.00000	+0.00000	+0.96383
C	+1.27616	+0.00000	+1.38026

C +1.37054 +0.03116 +2.90144
N +0.46236 −0.87206 +3.58499
H −0.50243 −0.67503 +3.35206
O +2.20495 −0.00874 +0.62424
C +2.80382 −0.25352 +3.33350
H +1.10637 +1.05381 +3.20216
H +0.65298 −1.83724 +3.34047
C +2.94503 −0.14843 +4.82990
H +3.10856 −1.25933 +3.03457
H +3.48583 +0.45523 +2.85467
O +2.46570 +1.01745 +5.29559
H +2.56855 +1.01616 +6.25352
O +3.44023 −0.97390 +5.54031
16
* E = +4.193 kcal/mol ; (36) 351_305_298_282_193_181
H +0.00000 +0.00000 +0.00000
O +0.00000 +0.00000 +0.96921
C +1.24400 +0.00000 +1.44930
C +2.38497 −0.22421 +0.43834
N +2.28092 −1.43078 −0.36527
H +2.30734 −2.26087 +0.21194
O +1.46237 +0.14168 +2.61698
C +2.62252 +0.98885 −0.46745
H +3.27115 −0.31884 +1.07119
H +1.43597 −1.45327 −0.92252
C +1.62477 +1.14040 −1.58299
H +2.62230 +1.92236 +0.10794
H +3.61068 +0.90443 −0.92925
O +2.01953 +2.01768 −2.49885
H +1.33552 +2.08524 −3.17598
O +0.57180 +0.55400 −1.67361
16
* E = +4.228 kcal/mol ; (37) 179_041_194_175_343_182
H +0.00000 +0.00000 +0.00000
O +0.00000 +0.00000 +0.96383
C +1.27519 +0.00000 +1.37950
C +1.37939 +0.02766 +2.89380
N +0.37917 −0.84859 +3.48461
H +0.29702 −0.65576 +4.47500
O +2.20311 +0.01987 +0.62424

C +2.78382 −0.39805 +3.29956
H +1.23650 +1.08867 +3.16794
H −0.52776 −0.70119 +3.06018
C +3.06892 −0.30048 +4.77583
H +2.96837 −1.43229 +2.99834
H +3.52805 +0.21729 +2.78321
O +2.17763 +0.46712 +5.43916
H +2.45498 +0.49990 +6.36206
O +3.99361 −0.81811 +5.32900
16
* E = +4.234 kcal/mol ; (38) 183_322_291_169_037_359
H +0.00000 +0.00000 +0.00000
O +0.00000 +0.00000 +0.96417
C +1.27545 +0.00000 +1.38722
C +1.36331 −0.07521 +2.90447
N +0.33938 +0.69006 +3.61014
H +0.47889 +1.68758 +3.50533
O +2.21263 +0.01561 +0.64655
C +1.27321 −1.55627 +3.30367
H +2.34877 +0.31508 +3.17667
H −0.58117 +0.46817 +3.24466
C +1.60964 −1.84636 +4.76457
H +0.25081 −1.91024 +3.11459
H +1.94798 −2.16181 +2.69602
O +1.20963 −0.93635 +5.65072
H +0.75880 −0.20021 +5.19563
O +2.18124 −2.84284 +5.09185
16
* E = +4.330 kcal/mol ; (39) 003_162_028_079_172_179
H +0.00000 +0.00000 +0.00000
O +0.00000 +0.00000 +0.96972
C +1.23913 +0.00000 +1.44800
C +2.36227 +0.07458 +0.40476
N +3.56937 +0.48088 +1.08383
H +3.54258 +0.18028 +2.05175
O +1.46376 −0.08123 +2.61958
C +2.49093 −1.27977 −0.32224
H +2.07468 +0.82867 −0.34217
H +4.40766 +0.13772 +0.63751
C +1.46232 −1.50142 −1.39796

H +3.47293 −1.34269 −0.80461
H +2.44810 −2.11970 +0.38170
O +1.70717 −2.59335 −2.11769
H +1.01299 −2.68630 −2.78132
O +0.51967 −0.78279 −1.62042
16
* E = +4.485 kcal/mol ; (40) 182_094_066_061_177_179
H +0.00000 +0.00000 +0.00000
O +0.00000 +0.00000 +0.96397
C +1.26316 +0.00000 +1.37806
C +1.39879 −0.05641 +2.89286
N +1.55947 −1.46462 +3.22861
H +2.43671 −1.81929 +2.86420
O +2.20818 −0.01147 +0.63798
C +2.56479 +0.83171 +3.32038
H +0.46383 +0.30523 +3.32991
H +1.54925 −1.60873 +4.22988
C +2.33012 +2.26664 +2.93457
H +2.72909 +0.77197 +4.40103
H +3.48768 +0.49661 +2.83238
O +3.30668 +3.08037 +3.35505
H +3.09825 +3.97363 +3.05822
O +1.37784 +2.65520 +2.31740
16
* E = +4.595 kcal/mol ; (41) 177_160_047_180_333_182
H +0.00000 +0.00000 +0.00000
O +0.00000 +0.00000 +0.96403
C +1.27402 +0.00000 +1.38106
C +1.37358 +0.07943 +2.89061
N +2.68047 −0.38566 +3.28610
H +3.40711 +0.03358 +2.71848
O +2.20896 −0.00528 +0.63482
C +1.03511 +1.53371 +3.27769
H +0.60715 −0.57805 +3.31525
H +2.85954 −0.22148 +4.26698
C +1.09386 +1.79803 +4.76155
H +1.72223 +2.23637 +2.79702
H +0.02010 +1.78713 +2.94686
O +0.85131 +0.69559 +5.49447
H +0.87064 +0.94991 +6.42435

O +1.31184 +2.86376 +5.25850
16
∗ E = +4.657 kcal/mol ; (42) 178_017_192_284_051_183
H +0.00000 +0.00000 +0.00000
O +0.00000 +0.00000 +0.96379
C +1.27394 +0.00000 +1.37157
C +1.41929 +0.05058 +2.88538
N +0.19183 −0.31122 +3.56789
H +0.24420 −0.08950 +4.55348
O +2.20266 +0.02991 +0.61748
C +2.54983 −0.88054 +3.32933
H +1.74941 +1.08628 +3.07857
H −0.61517 +0.15038 +3.17027
C +2.11578 −2.32522 +3.27007
H +3.41533 −0.73736 +2.67447
H +2.84532 −0.66921 +4.35830
O +1.57640 −2.63291 +2.08069
H +1.28267 −3.54979 +2.11407
O +2.24137 −3.12252 +4.15338
16
∗ E = +4.712 kcal/mol ; (43) 179_031_192_302_330_354
H +0.00000 +0.00000 +0.00000
O +0.00000 +0.00000 +0.96432
C +1.27386 +0.00000 +1.38340
C +1.38549 +0.02551 +2.90123
N +0.25433 −0.67285 +3.51279
H +0.21977 −0.49450 +4.50896
O +2.21080 +0.02334 +0.64423
C +2.71086 −0.60277 +3.33072
H +1.41012 +1.09593 +3.16259
H −0.62564 −0.38284 +3.10384
C +2.93240 −2.04155 +2.86496
H +3.54896 −0.02006 +2.94729
H +2.77516 −0.59197 +4.42524
O +1.83466 −2.78619 +2.70721
H +1.04616 −2.29767 +3.00805
O +4.02532 −2.47990 +2.67295
16
∗ E = +4.820 kcal/mol ; (44) 353_022_082_297_169_354
H +0.00000 +0.00000 +0.00000

```
 O   +0.00000   +0.00000   +0.97134
 C   +1.26431   +0.00000   +1.36261
 C   +2.28501   −0.16324   +0.22395
 N   +1.68792   +0.22257   −1.04390
 H   +1.71743   +1.22982   −1.15973
 O   +1.60180   +0.07130   +2.50867
 C   +3.57844   +0.54624   +0.62478
 H   +2.48676   −1.24099   +0.18303
 H   +2.12841   −0.22571   −1.83437
 C   +3.35343   +2.03629   +0.79188
 H   +3.94372   +0.12938   +1.56927
 H   +4.35372   +0.39566   −0.13588
 O   +4.35117   +2.71997   +1.36509
 H   +5.04228   +2.13232   +1.67834
 O   +2.36795   +2.59997   +0.41513
16
 * E =  +4.827  kcal/mol ; (45) 180_051_185_061_029_180
 H   +0.00000   +0.00000   +0.00000
 O   +0.00000   +0.00000   +0.96384
 C   +1.27399   +0.00000   +1.38268
 C   +1.36551   −0.00574   +2.89791
 N   +0.52488   −1.08007   +3.41280
 H   +0.50535   −1.06158   +4.42483
 O   +2.20802   +0.02116   +0.63594
 C   +2.81040   −0.23254   +3.32018
 H   +1.04372   +0.99851   +3.21979
 H   −0.42662   −0.98095   +3.08063
 C   +3.78367   +0.82202   +2.85419
 H   +2.86485   −0.25650   +4.41540
 H   +3.16636   −1.19774   +2.95504
 O   +3.20474   +2.02750   +2.70158
 H   +3.88466   +2.63994   +2.39923
 O   +4.95289   +0.64338   +2.68305
16
 * E =  +4.993  kcal/mol ; (46) 178_161_033_170_077_182
 H   +0.00000   +0.00000   +0.00000
 O   +0.00000   +0.00000   +0.96393
 C   +1.27041   +0.00000   +1.38310
 C   +1.36935   +0.05820   +2.89545
 N   +2.68650   −0.38059   +3.28518
```

H +3.36603 −0.11003 +2.58332
O +2.20875 −0.00417 +0.64002
C +0.98319 +1.50453 +3.31378
H +0.60160 −0.61709 +3.29627
H +2.96220 −0.00490 +4.18218
C +0.81244 +1.59408 +4.80650
H +1.77159 +2.18891 +2.98535
H +0.02977 +1.78169 +2.86013
O +1.99436 +1.63980 +5.45307
H +1.81176 +1.66603 +6.39985
O −0.23382 +1.59397 +5.38632
16
∗ E = +4.998 kcal/mol ; (47) 355_023_082_169_179_002
H +0.00000 +0.00000 +0.00000
O +0.00000 +0.00000 +0.97168
C +1.26746 +0.00000 +1.36563
C +2.27743 −0.11910 +0.21827
N +1.67955 +0.34135 −1.02662
H +1.71250 +1.35272 −1.10079
O +1.59478 +0.05519 +2.51489
C +3.59440 +0.53898 +0.60889
H +2.46136 −1.19511 +0.10022
H +2.18752 −0.04045 −1.81547
C +4.68553 +0.20506 −0.38541
H +3.47741 +1.62993 +0.66367
H +3.89004 +0.20887 +1.61161
O +5.88466 +0.75273 −0.13583
H +5.87247 +1.26533 +0.67586
O +4.52554 −0.49352 −1.34190
16
∗ E = +5.000 kcal/mol ; (48) 174_161_039_065_059_182
H +0.00000 +0.00000 +0.00000
O +0.00000 +0.00000 +0.96426
C +1.26901 +0.00000 +1.38829
C +1.35375 +0.15121 +2.89436
N +2.65512 −0.30245 +3.32030
H +3.36598 −0.01998 +2.65523
O +2.21249 −0.05411 +0.65445
C +1.05184 +1.63367 +3.23481
H +0.56506 −0.46616 +3.33999

H +2.89672 +0.03960 +4.24002
C −0.35430 +2.05515 +2.88905
H +1.18499 +1.75714 +4.31504
H +1.75008 +2.29418 +2.71589
O −1.26305 +1.30579 +3.53364
H −2.13932 +1.59448 +3.25526
O −0.65246 +2.94314 +2.14444
16
* E = +5.010 kcal/mol ; (49) 183_321_308_298_109_182
H +0.00000 +0.00000 +0.00000
O +0.00000 +0.00000 +0.96405
C +1.27642 +0.00000 +1.37897
C +1.38755 −0.07501 +2.90057
N +0.40982 +0.67981 +3.64853
H +0.36858 +1.64501 +3.34946
O +2.20794 +0.02623 +0.62756
C +1.33298 −1.56010 +3.31177
H +2.39241 +0.28990 +3.13453
H −0.51511 +0.27217 +3.56431
C +0.01461 −2.18458 +2.92159
H +2.15627 −2.10788 +2.85067
H +1.41633 −1.59993 +4.40046
O +0.13854 −3.05189 +1.90821
H −0.74119 −3.38471 +1.69493
O −1.04200 −1.93211 +3.43070
16
* E = +5.223 kcal/mol ; (50) 180_004_053_286_035_178
H +0.00000 +0.00000 +0.00000
O +0.00000 +0.00000 +0.96396
C +1.27142 +0.00000 +1.36399
C +1.44675 −0.00681 +2.87706
N +0.18203 −0.10557 +3.57309
H −0.35822 −0.89864 +3.24901
O +2.19927 +0.01885 +0.60476
C +2.50759 −1.05638 +3.25315
H +1.88570 +0.97472 +3.10066
H +0.31585 −0.18463 +4.57261
C +2.01461 −2.47654 +3.14278
H +3.37260 −0.95154 +2.58762
H +2.85095 −0.91029 +4.27918

O	+1.15934	−2.63569	+2.11477
H	+0.90965	−3.56582	+2.07290
O	+2.33329	−3.38049	+3.85811

16

∗ E = +5.312 kcal/mol ; (51) 176_166_038_074_234_180

H	+0.00000	+0.00000	+0.00000
O	+0.00000	+0.00000	+0.96402
C	+1.27099	+0.00000	+1.38479
C	+1.35977	+0.10251	+2.89447
N	+2.70264	−0.23985	+3.29048
H	+3.38010	+0.12539	+2.63180
O	+2.20980	−0.01776	+0.64290
C	+0.90141	+1.52808	+3.29233
H	+0.63695	−0.60118	+3.32586
H	+2.92187	+0.08069	+4.22301
C	−0.59486	+1.68424	+3.16399
H	+1.14467	+1.67170	+4.35001
H	+1.42842	+2.28827	+2.70996
O	−0.94736	+2.72627	+2.39935
H	−1.91076	+2.75545	+2.36865
O	−1.39160	+0.96471	+3.69443

16

∗ E = +5.353 kcal/mol ; (52) 185_316_302_304_293_178

H	+0.00000	+0.00000	+0.00000
O	+0.00000	+0.00000	+0.96407
C	+1.27603	+0.00000	+1.38376
C	+1.36993	−0.11271	+2.90298
N	+0.45156	+0.72291	+3.64683
H	+0.57891	+1.70142	+3.42338
O	+2.21013	+0.05722	+0.63857
C	+1.20808	−1.59517	+3.29507
H	+2.39450	+0.17549	+3.15478
H	−0.51139	+0.47163	+3.45798
C	−0.06675	−2.22296	+2.78412
H	+2.03596	−2.18031	+2.89266
H	+1.21556	−1.64215	+4.38803
O	−1.16436	−1.70044	+3.36055
H	−1.93336	−2.12915	+2.96745
O	−0.12577	−3.08911	+1.96060

16

∗ E = +5.408 kcal/mol ; (53) 180_021_295_297_182_359

H	+0.00000	+0.00000	+0.00000
O	+0.00000	+0.00000	+0.96398
C	+1.27159	+0.00000	+1.37231
C	+1.41001	+0.00775	+2.89562
N	+0.26615	−0.45742	+3.65014
H	−0.52326	+0.16279	+3.52501
O	+2.20419	+0.03971	+0.61932
C	+2.66152	−0.78435	+3.26113
H	+1.59144	+1.05755	+3.16113
H	−0.01224	−1.37308	+3.31365
C	+2.52207	−2.22851	+2.82402
H	+3.54252	−0.35949	+2.76813
H	+2.81096	−0.74684	+4.34518
O	+3.54494	−3.03615	+3.13826
H	+4.23841	−2.55539	+3.59547
O	+1.57248	−2.65173	+2.23369

16

∗ E = +5.423 kcal/mol ; (54) 181_199_300_301_184_001

H	+0.00000	+0.00000	+0.00000
O	+0.00000	+0.00000	+0.96403
C	+1.27819	+0.00000	+1.37781
C	+1.39822	−0.01494	+2.90166
N	+2.69829	+0.41582	+3.36683
H	+3.41562	−0.19886	+3.00107
O	+2.20807	−0.04370	+0.62924
C	+0.29145	+0.79778	+3.56794
H	+1.25579	−1.06417	+3.19397
H	+2.89301	+1.33676	+2.98488
C	+0.31327	+2.23725	+3.09766
H	−0.69607	+0.37915	+3.34538
H	+0.44004	+0.77062	+4.65296
O	−0.56398	+3.06014	+3.69191
H	−1.08149	+2.59654	+4.35396
O	+1.04902	+2.64231	+2.24800

16

∗ E = +5.512 kcal/mol ; (55) 182_320_063_180_325_182

H	+0.00000	+0.00000	+0.00000
O	+0.00000	+0.00000	+0.96379
C	+1.27416	+0.00000	+1.38271

C	+1.37065	−0.04892	+2.89546
N	+0.35020	+0.78524	+3.49744
H	−0.58149	+0.45102	+3.28217
O	+2.20792	−0.02630	+0.63545
C	+1.32076	−1.53770	+3.29356
H	+2.35254	+0.35448	+3.15412
H	+0.46565	+0.82730	+4.50179
C	+1.41462	−1.75388	+4.78262
H	+0.39099	−2.00293	+2.95399
H	+2.15522	−2.07825	+2.82916
O	+2.21383	−0.85100	+5.38116
H	+2.25716	−1.07107	+6.31886
O	+0.86494	−2.62588	+5.38897

16

∗ E = +5.514 kcal/mol ; (56) 179_042_316_290_086_352

H	+0.00000	+0.00000	+0.00000
O	+0.00000	+0.00000	+0.96453
C	+1.26118	+0.00000	+1.39207
C	+1.35516	+0.03531	+2.91603
N	+0.41781	−0.81737	+3.61319
H	−0.51213	−0.76624	+3.21991
O	+2.19873	+0.01448	+0.63489
C	+2.79022	−0.24345	+3.37522
H	+1.12432	+1.08043	+3.17346
H	+0.72409	−1.78363	+3.63456
C	+3.18765	−1.69239	+3.12834
H	+3.47702	+0.43758	+2.86604
H	+2.83852	−0.07582	+4.45150
O	+3.67133	−1.97517	+1.91571
H	+3.57225	−1.21659	+1.32486
O	+3.05624	−2.54578	+3.95681

16

∗ E = +5.566 kcal/mol ; (57) 183_063_162_280_076_355

H	+0.00000	+0.00000	+0.00000
O	+0.00000	+0.00000	+0.96458
C	+1.25847	+0.00000	+1.39579
C	+1.32729	−0.07996	+2.91176
N	+0.74077	−1.35586	+3.29271
H	+1.00685	−1.62025	+4.23363
O	+2.20224	+0.05382	+0.65019

C +2.76883 +0.01559 +3.41311
H +0.77311 +0.79492 +3.29108
H −0.26683 −1.35485 +3.21097
C +3.51307 −1.30980 +3.29497
H +3.29665 +0.80295 +2.86660
H +2.76059 +0.27007 +4.47418
O +3.92398 −1.64847 +2.07439
H +3.60296 −1.01541 +1.41673
O +3.72197 −2.00474 +4.24595
16
* E = +5.647 kcal/mol ; (58) 183_317_068_295_107_183
H +0.00000 +0.00000 +0.00000
O +0.00000 +0.00000 +0.96384
C +1.27329 +0.00000 +1.38344
C +1.36122 −0.06403 +2.89828
N +0.38184 +0.81652 +3.49535
H −0.55962 +0.48046 +3.32110
O +2.21025 −0.00596 +0.64031
C +1.27631 −1.55872 +3.32296
H +2.36431 +0.28778 +3.15205
H +0.50717 +0.85822 +4.49900
C −0.06776 −2.14231 +2.97347
H +2.07528 −2.13080 +2.84575
H +1.39088 −1.60591 +4.40885
O −0.00972 −2.98544 +1.93378
H −0.90727 −3.28239 +1.74349
O −1.09436 −1.87232 +3.53327
16
* E = +5.738 kcal/mol ; (59) 180_047_184_082_287_003
H +0.00000 +0.00000 +0.00000
O +0.00000 +0.00000 +0.96463
C +1.25923 +0.00000 +1.39340
C +1.35037 +0.01139 +2.90902
N +0.43221 −0.99392 +3.42543
H +0.42263 −0.97207 +4.43733
O +2.19722 +0.00888 +0.63887
C +2.77321 −0.30574 +3.38346
H +1.08820 +1.04179 +3.21346
H −0.51191 −0.83193 +3.09704
C +3.70636 +0.89719 +3.35990

H +2.72378 −0.63353 +4.42296
H +3.17999 −1.12515 +2.78177
O +4.10950 +1.30578 +2.15624
H +3.69003 +0.78175 +1.45788
O +4.05579 +1.45552 +4.35788
16
* E = +5.955 kcal/mol ; (60) 183_103_063_071_331_181
H +0.00000 +0.00000 +0.00000
O +0.00000 +0.00000 +0.96417
C +1.26533 +0.00000 +1.37698
C +1.40942 −0.08058 +2.89026
N +1.77723 −1.46430 +3.15975
H +2.67157 −1.67767 +2.73111
O +2.20767 +0.00561 +0.63518
C +2.44981 +0.93753 +3.37275
H +0.43344 +0.11622 +3.34391
H +1.85118 −1.63671 +4.15423
C +2.01444 +2.37788 +3.28886
H +2.73153 +0.73789 +4.40895
H +3.35095 +0.82964 +2.75506
O +1.13230 +2.57718 +2.29398
H +0.90370 +3.51342 +2.27483
O +2.39485 +3.26067 +4.00102
16
* E = +5.973 kcal/mol ; (61) 183_194_206_174_171_357
H +0.00000 +0.00000 +0.00000
O +0.00000 +0.00000 +0.96392
C +1.27678 +0.00000 +1.37642
C +1.39775 −0.07892 +2.88428
N +2.74779 +0.26462 +3.26954
H +2.98198 −0.16341 +4.15538
O +2.20335 +0.02098 +0.61956
C +0.40023 +0.84286 +3.58199
H +1.11856 −1.11613 +3.14215
H +3.40710 −0.02673 +2.55833
C +0.44908 +0.61952 +5.07983
H +0.65312 +1.88690 +3.36147
H −0.62392 +0.65809 +3.23808
O −0.23646 +1.50690 +5.81895
H −0.63062 +2.18633 +5.26760

O +1.02833 −0.28491 +5.60146
16
∗ E = +6.083 kcal/mol ; (62) 179_043_192_172_177_359
H +0.00000 +0.00000 +0.00000
O +0.00000 +0.00000 +0.96383
C +1.27253 +0.00000 +1.38160
C +1.37244 +0.02623 +2.89484
N +0.40275 −0.88202 +3.48430
H +0.35274 −0.70819 +4.48163
O +2.20382 +0.01626 +0.62838
C +2.78169 −0.36209 +3.31187
H +1.20653 +1.08000 +3.18468
H −0.51729 −0.73241 +3.08972
C +2.98810 −0.15818 +4.79607
H +2.95533 −1.41633 +3.06415
H +3.52172 +0.22821 +2.75959
O +4.18375 −0.54481 +5.26912
H +4.73035 −0.91157 +4.57043
O +2.17286 +0.31763 +5.52921
16
∗ E = +6.148 kcal/mol ; (63) 184_079_072_273_064_002
H +0.00000 +0.00000 +0.00000
O +0.00000 +0.00000 +0.96475
C +1.25287 +0.00000 +1.39170
C +1.36227 −0.08559 +2.90681
N +1.16349 −1.48271 +3.26252
H +1.96600 −2.04884 +3.00557
O +2.19628 +0.05210 +0.63971
C +2.66394 +0.56793 +3.42067
H +0.50998 +0.47551 +3.30328
H +1.02172 −1.58645 +4.25899
C +3.83651 −0.40146 +3.43790
H +2.91436 +1.44459 +2.81210
H +2.52120 +0.89364 +4.45181
O +4.22799 −0.86675 +2.24981
H +3.70175 −0.47865 +1.53156
O +4.35852 −0.76269 +4.45104
16
∗ E = +6.155 kcal/mol ; (64) 182_062_302_073_282_005
H +0.00000 +0.00000 +0.00000

O	+0.00000	+0.00000	+0.96456
C	+1.26066	+0.00000	+1.39493
C	+1.34220	−0.04639	+2.91654
N	+0.73668	−1.26803	+3.42946
H	−0.23315	−1.34527	+3.14852
O	+2.19997	−0.01181	+0.63984
C	+2.78886	+0.04693	+3.40274
H	+0.76374	+0.80126	+3.30453
H	+1.22994	−2.08822	+3.09468
C	+3.39428	+1.43699	+3.25868
H	+2.79346	−0.19637	+4.46513
H	+3.39905	−0.68826	+2.86410
O	+3.80203	+1.77936	+2.03324
H	+3.57208	+1.09343	+1.39129
O	+3.50072	+2.18892	+4.18092

16

∗ E = +6.285 kcal/mol ; (65) 184_314_066_303_291_178

H	+0.00000	+0.00000	+0.00000
O	+0.00000	+0.00000	+0.96397
C	+1.27315	+0.00000	+1.38693
C	+1.34816	−0.10783	+2.89982
N	+0.39793	+0.80034	+3.50722
H	−0.55372	+0.52545	+3.29420
O	+2.21267	+0.02511	+0.64899
C	+1.21761	−1.60870	+3.28431
H	+2.35942	+0.20534	+3.17124
H	+0.50156	+0.80564	+4.51399
C	−0.04596	−2.24961	+2.76831
H	+2.05879	−2.17199	+2.87539
H	+1.23297	−1.67769	+4.37656
O	−1.14615	−1.77942	+3.38552
H	−1.91081	−2.21003	+2.98561
O	−0.09884	−3.07640	+1.90491

16

∗ E = +6.451 kcal/mol ; (66) 001_345_163_173_188_003

H	+0.00000	+0.00000	+0.00000
O	+0.00000	+0.00000	+0.97226
C	+1.26876	+0.00000	+1.36752
C	+2.29176	+0.02386	+0.22426
N	+1.64665	−0.32165	−1.03664

H +2.23668 −0.04197 −1.81264
O +1.59777 +0.02796 +2.51595
C +2.85116 +1.44445 +0.17792
H +3.10556 −0.65645 +0.50640
H +1.53796 −1.32706 −1.11281
C +4.00743 +1.58666 −0.79069
H +2.06272 +2.14793 −0.12429
H +3.17583 +1.73000 +1.18515
O +4.64490 +2.76545 −0.75455
H +4.28582 +3.34003 −0.07439
O +4.34527 +0.73714 −1.55984
16
* E = +6.468 kcal/mol ; (67) 178_279_071_055_173_179
H +0.00000 +0.00000 +0.00000
O +0.00000 +0.00000 +0.96369
C +1.28373 +0.00000 +1.37616
C +1.36351 +0.06072 +2.89602
N +1.17604 +1.45931 +3.26175
H +0.21504 +1.74564 +3.11264
O +2.20998 +0.01782 +0.62752
C +0.36281 −0.88352 +3.56530
H +2.38707 −0.23077 +3.14582
H +1.40733 +1.62022 +4.23340
C +0.50373 −2.30501 +3.09411
H +0.47906 −0.85514 +4.65365
H −0.66326 −0.56181 +3.35060
O −0.27006 −3.15187 +3.78719
H −0.14279 −4.03577 +3.42375
O +1.21528 −2.65808 +2.19696
16
* E = +6.537 kcal/mol ; (68) 184_324_058_058_061_184
H +0.00000 +0.00000 +0.00000
O +0.00000 +0.00000 +0.96399
C +1.27279 +0.00000 +1.38157
C +1.38415 −0.10068 +2.89117
N +0.31389 +0.62940 +3.53536
H −0.59388 +0.28637 +3.24470
O +2.20907 +0.00066 +0.63676
C +1.43880 −1.60840 +3.24460
H +2.34530 +0.35026 +3.15371

H +0.37885 +0.56131 +4.54266
C +2.58512 −2.31406 +2.56342
H +1.56368 −1.69656 +4.32877
H +0.50861 −2.10090 +2.95123
O +3.76687 −1.79912 +2.93425
H +4.45740 −2.23553 +2.42276
O +2.47863 −3.21390 +1.78221
16
∗ E = +6.628 kcal/mol ; (69) 181_307_063_060_276_174
H +0.00000 +0.00000 +0.00000
O +0.00000 +0.00000 +0.96357
C +1.27426 +0.00000 +1.38517
C +1.34481 −0.03513 +2.90245
N +0.54049 +1.05109 +3.42865
H −0.43506 +0.93336 +3.18057
O +2.21233 +0.00126 +0.64591
C +0.91436 −1.43438 +3.40929
H +2.39278 +0.13536 +3.16379
H +0.61345 +1.10476 +4.43643
C +1.81577 −2.51366 +2.86849
H +0.98273 −1.44495 +4.49864
H −0.11862 −1.61861 +3.09873
O +1.43635 −2.90221 +1.64076
H +2.09317 −3.52266 +1.30422
O +2.77880 −2.95541 +3.42504
16
∗ E = +6.882 kcal/mol ; (70) 359_164_292_070_022_176
H +0.00000 +0.00000 +0.00000
O +0.00000 +0.00000 +0.96411
C +1.24978 +0.00000 +1.44252
C +2.37502 −0.01550 +0.39462
N +3.65418 +0.36502 +0.95356
H +3.64366 +1.34240 +1.21980
O +1.46624 −0.00979 +2.61615
C +2.51468 −1.42156 −0.20843
H +2.11810 +0.68633 −0.40821
H +3.80715 −0.15289 +1.81408
C +1.40994 −1.91337 −1.10663
H +3.42807 −1.42047 −0.81442
H +2.66046 −2.17195 +0.57419

O +0.67963 −0.90559 −1.65400
H +0.04131 −1.30538 −2.25754
O +1.19575 −3.05563 −1.37220
16
* E = +7.004 kcal/mol ; (71) 003_167_283_080_172_359
H +0.00000 +0.00000 +0.00000
O +0.00000 +0.00000 +0.97124
C +1.23898 +0.00000 +1.44877
C +2.37352 +0.06223 +0.40883
N +3.65589 +0.34943 +1.01728
H +3.72252 +1.33545 +1.23799
O +1.46650 −0.06442 +2.62178
C +2.46920 −1.26607 −0.36040
H +2.13337 +0.84754 −0.31817
H +3.70955 −0.13369 +1.90985
C +1.44277 −1.41742 −1.45926
H +3.47001 −1.31137 −0.80563
H +2.38736 −2.12430 +0.32007
O +1.59886 −2.47131 −2.26188
H +2.37045 −2.98884 −2.01892
O +0.53377 −0.65464 −1.64447
16
* E = +7.283 kcal/mol ; (72) 186_193_326_298_088_359
H +0.00000 +0.00000 +0.00000
O +0.00000 +0.00000 +0.96522
C +1.29018 +0.00000 +1.37746
C +1.42292 −0.13650 +2.89287
N +2.74798 +0.16238 +3.37464
H +3.44774 −0.12855 +2.70232
O +2.20446 +0.04694 +0.61266
C +0.36930 +0.68327 +3.65557
H +1.19684 −1.19706 +3.08532
H +2.86237 +1.15821 +3.53414
C +0.52418 +2.16357 +3.35668
H −0.63441 +0.33262 +3.40263
H +0.54469 +0.53861 +4.72253
O −0.14669 +2.61628 +2.28560
H −0.61835 +1.89951 +1.84999
O +1.22966 +2.88993 +3.99006
16

∗ E = +7.289 kcal/mol ; (73) 354_012_282_293_006_176

H	+0.00000	+0.00000	+0.00000
O	+0.00000	+0.00000	+0.96253
C	+1.26110	+0.00000	+1.39861
C	+2.35467	−0.15052	+0.32150
N	+1.94421	−0.01123	−1.05715
H	+1.47854	−0.80590	−1.46466
O	+1.52648	+0.07167	+2.56084
C	+3.51388	+0.78505	+0.66171
H	+2.72647	−1.17432	+0.44983
H	+1.50959	+0.86976	−1.28912
C	+3.18866	+2.25624	+0.56640
H	+3.84231	+0.60778	+1.68977
H	+4.36123	+0.59412	−0.00042
O	+1.87929	+2.48727	+0.32388
H	+1.73810	+3.44109	+0.33916
O	+3.97840	+3.14205	+0.69568

16

∗ E = +7.317 kcal/mol ; (74) 017_213_208_309_307_009

H	+0.00000	+0.00000	+0.00000
O	+0.00000	+0.00000	+0.96389
C	+1.21287	+0.00000	+1.51315
C	+2.40828	+0.39688	+0.63240
N	+3.55751	−0.38643	+1.08001
H	+4.42627	+0.10293	+0.90574
O	+1.37010	−0.24152	+2.67222
C	+2.18545	+0.19324	−0.86969
H	+2.53632	+1.47449	+0.81880
H	+3.47761	−0.55282	+2.07926
C	+1.68408	−1.20835	−1.18947
H	+1.46562	+0.90886	−1.27442
H	+3.13608	+0.36136	−1.38730
O	+2.41418	−2.20053	−0.69869
H	+3.08951	−1.84409	−0.08813
O	+0.67607	−1.39309	−1.81211

16

∗ E = +7.330 kcal/mol ; (75) 358_168_285_169_044_356

H	+0.00000	+0.00000	+0.00000
O	+0.00000	+0.00000	+0.96080
C	+1.24232	+0.00000	+1.46163

C	+2.37923	−0.05259	+0.43788
N	+3.66998	+0.23420	+1.04432
H	+3.76538	+1.22365	+1.24124
O	+1.43637	+0.01137	+2.63782
C	+2.40942	−1.44974	−0.20737
H	+2.18509	+0.69800	−0.34089
H	+3.72035	−0.23881	+1.94342
C	+3.32933	−1.54293	−1.42128
H	+2.75462	−2.16820	+0.54741
H	+1.41927	−1.77402	−0.53427
O	+4.51664	−0.95861	−1.28947
H	+4.57238	−0.50876	−0.42406
O	+3.00408	−2.11361	−2.41974

16

∗ E = +7.345 kcal/mol ; (76) 183_079_296_064_187_003

H	+0.00000	+0.00000	+0.00000
O	+0.00000	+0.00000	+0.96407
C	+1.26971	+0.00000	+1.38355
C	+1.36691	−0.06763	+2.90231
N	+1.15298	−1.42734	+3.38234
H	+0.21133	−1.73888	+3.17824
O	+2.20924	−0.02813	+0.63888
C	+2.73244	+0.43179	+3.33544
H	+0.58938	+0.57822	+3.32017
H	+1.79657	−2.07338	+2.93847
C	+2.91046	+1.88974	+2.96668
H	+2.83708	+0.31333	+4.41928
H	+3.51857	−0.16179	+2.85187
O	+4.13636	+2.39822	+3.17222
H	+4.74342	+1.72321	+3.48285
O	+2.03022	+2.57288	+2.53956

16

∗ E = +7.461 kcal/mol ; (77) 356_181_212_175_169_179

H	+0.00000	+0.00000	+0.00000
O	+0.00000	+0.00000	+0.95971
C	+1.25067	+0.00000	+1.45393
C	+2.36763	−0.08541	+0.41992
N	+3.64451	−0.10542	+1.09393
H	+4.36007	+0.34345	+0.54009
O	+1.44017	+0.08197	+2.62703

C +2.23101 −1.34637 −0.43959
H +2.24459 +0.79051 −0.24204
H +3.57473 +0.32218 +2.00892
C +3.27204 −1.33485 −1.53069
H +2.37966 −2.23653 +0.17898
H +1.25273 −1.45005 −0.92681
O +3.34682 −2.50890 −2.16598
H +4.01901 −2.43717 −2.85347
O +3.94950 −0.38955 −1.82327
16
* E = +7.570 kcal/mol ; (78) 181_066_078_072_280_005
H +0.00000 +0.00000 +0.00000
O +0.00000 +0.00000 +0.96447
C +1.25338 +0.00000 +1.38885
C +1.36429 −0.03068 +2.90935
N +0.83878 −1.31200 +3.35274
H +1.51197 −2.05488 +3.20496
O +2.19874 +0.01001 +0.63881
C +2.80688 +0.24767 +3.36008
H +0.69592 +0.75097 +3.28985
H +0.60736 −1.28940 +4.33674
C +3.24061 +1.69381 +3.16543
H +2.87220 +0.04408 +4.42998
H +3.48230 −0.43059 +2.82562
O +3.60665 +2.03791 +1.92794
H +3.46342 +1.30490 +1.31331
O +3.24588 +2.48728 +4.05948
16
* E = +7.711 kcal/mol ; (79) 177_160_047_175_176_000
H +0.00000 +0.00000 +0.00000
O +0.00000 +0.00000 +0.96404
C +1.27498 +0.00000 +1.38074
C +1.37740 +0.06616 +2.89056
N +2.67595 −0.40927 +3.28800
H +3.41233 +0.00775 +2.73231
O +2.20820 +0.00771 +0.63227
C +1.04475 +1.51584 +3.29561
H +0.60453 −0.58271 +3.31814
H +2.83484 −0.26541 +4.27579
C +1.00666 +1.66844 +4.80274

H	+1.80036	+2.19940	+2.88530
H	+0.06990	+1.81942	+2.89145
O	+0.79949	+2.91921	+5.24854
H	+0.70739	+3.53816	+4.52080
O	+1.14339	+0.76607	+5.57113

16

∗ E = +7.839 kcal/mol ; (80) 180_008_062_091_301_359

H	+0.00000	+0.00000	+0.00000
O	+0.00000	+0.00000	+0.96534
C	+1.25734	+0.00000	+1.37615
C	+1.44149	+0.00255	+2.88397
N	+0.17927	−0.17917	+3.56356
H	−0.25018	−1.06827	+3.33826
O	+2.18780	−0.01644	+0.60551
C	+2.56623	−1.00342	+3.26300
H	+1.79729	+1.01700	+3.11796
H	+0.30087	−0.12714	+4.56625
C	+3.96069	−0.39277	+3.32993
H	+2.35670	−1.39569	+4.25863
H	+2.57698	−1.84530	+2.56041
O	+4.40708	+0.17946	+2.21378
H	+3.75883	+0.10290	+1.49391
O	+4.61577	−0.41201	+4.33061

16

∗ E = +7.848 kcal/mol ; (81) 354_165_281_164_144_179

H	+0.00000	+0.00000	+0.00000
O	+0.00000	+0.00000	+0.96059
C	+1.25387	+0.00000	+1.44795
C	+2.36715	−0.13851	+0.40816
N	+3.67427	+0.20521	+0.91844
H	+3.80203	+1.20844	+0.94942
O	+1.45538	+0.06699	+2.62085
C	+2.39703	−1.58609	−0.09655
H	+2.14073	+0.50681	−0.45096
H	+3.76026	−0.13147	+1.87290
C	+3.20554	−1.65891	−1.37011
H	+2.82987	−2.24243	+0.66273
H	+1.39274	−1.96524	−0.32422
O	+3.90371	−2.79410	−1.46899
H	+4.37502	−2.78281	−2.31026

O +3.20075 −0.81602 −2.22140
16
∗ E = +8.061 kcal/mol ; (82) 181_014_060_298_182_357
H +0.00000 +0.00000 +0.00000
O +0.00000 +0.00000 +0.96414
C +1.26687 +0.00000 +1.36307
C +1.44816 −0.02210 +2.87688
N +0.22672 −0.35822 +3.56873
H −0.09948 −1.27757 +3.29464
O +2.19959 +0.04126 +0.60738
C +2.66537 −0.90986 +3.17529
H +1.71930 +1.00877 +3.14195
H +0.34873 −0.33327 +4.57199
C +2.42353 −2.32871 +2.70114
H +3.55375 −0.52070 +2.66433
H +2.87013 −0.91799 +4.25243
O +3.41109 −3.20400 +2.94442
H +4.16117 −2.77318 +3.36018
O +1.42453 −2.67877 +2.14700
16
∗ E = +8.127 kcal/mol ; (83) 351_150_045_176_176_180
H +0.00000 +0.00000 +0.00000
O +0.00000 +0.00000 +0.95976
C +1.25285 +0.00000 +1.44458
C +2.36512 −0.22555 +0.42668
N +3.54347 +0.46566 +0.88437
H +3.69783 +0.29221 +1.87073
O +1.45968 +0.09657 +2.61404
C +2.51274 −1.75133 +0.27921
H +2.07176 +0.18835 −0.54847
H +4.35672 +0.21287 +0.33951
C +3.51737 −2.10519 −0.78564
H +2.83631 −2.19028 +1.23016
H +1.56345 −2.23851 +0.02084
O +3.71369 −3.42570 −0.87139
H +4.36179 −3.58939 −1.56642
O +4.08348 −1.31535 −1.48904
16
∗ E = +8.206 kcal/mol ; (84) 002_192_301_303_179_180
H +0.00000 +0.00000 +0.00000

O	+0.00000	+0.00000	+0.95859
C	+1.25136	+0.00000	+1.46011
C	+2.40166	+0.04162	+0.43762
N	+3.68555	−0.23969	+1.04095
H	+3.87709	+0.44297	+1.76474
O	+1.43274	+0.02737	+2.63530
C	+2.18181	−0.87761	−0.76583
H	+2.41953	+1.07320	+0.05829
H	+3.62870	−1.13621	+1.51582
C	+1.97049	−2.31100	−0.34832
H	+1.34641	−0.57613	−1.40939
H	+3.07174	−0.83768	−1.40169
O	+1.74802	−3.11491	−1.39374
H	+1.62906	−4.01295	−1.06288
O	+1.99531	−2.69925	+0.78507

16

∗ E = +8.409 kcal/mol ; (85) 184_206_208_076_216_006

H	+0.00000	+0.00000	+0.00000
O	+0.00000	+0.00000	+0.96396
C	+1.26936	+0.00000	+1.39046
C	+1.37033	−0.10134	+2.90098
N	+2.62253	+0.50208	+3.31307
H	+2.96096	+0.09405	+4.17396
O	+2.20925	+0.05312	+0.65131
C	+0.17990	+0.56912	+3.59144
H	+1.29745	−1.18125	+3.11540
H	+3.32383	+0.36487	+2.59399
C	−1.07400	−0.28394	+3.50782
H	+0.42084	+0.68002	+4.65446
H	+0.02838	+1.57647	+3.18978
O	−2.23323	+0.37533	+3.35537
H	−2.08177	+1.31424	+3.23294
O	−1.05871	−1.47224	+3.61272

16

∗ E = +8.550 kcal/mol ; (86) 178_266_300_058_181_001

H	+0.00000	+0.00000	+0.00000
O	+0.00000	+0.00000	+0.96396
C	+1.27880	+0.00000	+1.37977
C	+1.40093	+0.04862	+2.89863
N	+1.54653	+1.42331	+3.36204

H	+2.37387	+1.84733	+2.95944
O	+2.20789	+0.05913	+0.62981
C	+0.22152	−0.60032	+3.60438
H	+2.31726	−0.49780	+3.13892
H	+0.74763	+1.98458	+3.08676
C	+0.05475	−2.04364	+3.18199
H	+0.38042	−0.54767	+4.68733
H	−0.70454	−0.05445	+3.38306
O	−0.98116	−2.69750	+3.73494
H	−1.47766	−2.12594	+4.32454
O	+0.78370	−2.59350	+2.41434

16

∗ E = +8.631 kcal/mol ; (87) 181_353_058_169_178_001

H	+0.00000	+0.00000	+0.00000
O	+0.00000	+0.00000	+0.96425
C	+1.27181	+0.00000	+1.36998
C	+1.43657	−0.02060	+2.88055
N	+0.18420	+0.13695	+3.57785
H	−0.48754	−0.57982	+3.33452
O	+2.19944	−0.02223	+0.60962
C	+2.24833	−1.28040	+3.22702
H	+2.06895	+0.84280	+3.12078
H	+0.34029	+0.13905	+4.57768
C	+2.69193	−1.26552	+4.67452
H	+1.64588	−2.18182	+3.05008
H	+3.13359	−1.34603	+2.58191
O	+3.37225	−2.35132	+5.07899
H	+3.48966	−2.97621	+4.35993
O	+2.47253	−0.37120	+5.43436

16

∗ E = +8.634 kcal/mol ; (88) 355_166_292_178_309_179

H	+0.00000	+0.00000	+0.00000
O	+0.00000	+0.00000	+0.96037
C	+1.25339	+0.00000	+1.45009
C	+2.37091	−0.11404	+0.40889
N	+3.67799	+0.21699	+0.92145
H	+3.74212	+1.20170	+1.14758
O	+1.45334	+0.05364	+2.62326
C	+2.39955	−1.55538	−0.12057
H	+2.14333	+0.55901	−0.42843

H	+3.84410	−0.28984	+1.78550
C	+3.44687	−1.71390	−1.19615
H	+2.64328	−2.25101	+0.68645
H	+1.42870	−1.84860	−0.53713
O	+3.32728	−0.77085	−2.14594
H	+4.02804	−0.91132	−2.79231
O	+4.26982	−2.57870	−1.23710

16

* E = +8.709 kcal/mol ; (89) 183_201_302_078_204_005

H	+0.00000	+0.00000	+0.00000
O	+0.00000	+0.00000	+0.96412
C	+1.27079	+0.00000	+1.39053
C	+1.38176	−0.08296	+2.91072
N	+2.65492	+0.39684	+3.40542
H	+3.40840	−0.13976	+2.99174
O	+2.20837	+0.05544	+0.64820
C	+0.21730	+0.62664	+3.59861
H	+1.28563	−1.14921	+3.15714
H	+2.80795	+1.35795	+3.11871
C	−1.05006	−0.21186	+3.58141
H	+0.49296	+0.77861	+4.64831
H	+0.03625	+1.61434	+3.15759
O	−2.20172	+0.47138	+3.69220
H	−2.04543	+1.41754	+3.69752
O	−1.05243	−1.40200	+3.52505

16

* E = +8.740 kcal/mol ; (90) 183_199_200_099_295_359

H	+0.00000	+0.00000	+0.00000
O	+0.00000	+0.00000	+0.96448
C	+1.28082	+0.00000	+1.39525
C	+1.38860	−0.07039	+2.90677
N	+2.71456	+0.38430	+3.26593
H	+2.97089	+0.07120	+4.19247
O	+2.20387	+0.01872	+0.64016
C	+0.31921	+0.77455	+3.64179
H	+1.20846	−1.13744	+3.14145
H	+3.40184	+0.05470	+2.59862
C	−0.87038	−0.03358	+4.13355
H	+0.78764	+1.21205	+4.52308
H	−0.02350	+1.59749	+3.00512

O −1.63887 −0.58971 +3.18738
H −1.31639 −0.37281 +2.30541
O −1.11386 −0.19080 +5.29182
16
∗ E = +8.789 kcal/mol ; (91) 349_306_298_283_193_002
H +0.00000 +0.00000 +0.00000
O +0.00000 +0.00000 +0.97002
C +1.24234 +0.00000 +1.44787
C +2.38383 −0.25240 +0.44244
N +2.26830 −1.45641 −0.36068
H +2.27432 −2.28691 +0.21637
O +1.46741 +0.16185 +2.61204
C +2.64760 +0.95326 −0.46782
H +3.26421 −0.35846 +1.08193
H +1.43107 −1.46401 −0.93002
C +1.64790 +1.11946 −1.59207
H +2.66348 +1.88442 +0.11296
H +3.64075 +0.82870 −0.91515
O +1.99295 +1.97288 −2.55560
H +2.86292 +2.34872 −2.40130
O +0.59228 +0.54805 −1.65686
16
∗ E = +8.904 kcal/mol ; (92) 185_211_190_288_071_005
H +0.00000 +0.00000 +0.00000
O +0.00000 +0.00000 +0.96533
C +1.28779 +0.00000 +1.37734
C +1.44262 −0.12743 +2.88358
N +2.64236 +0.58810 +3.25162
H +2.90552 +0.41883 +4.21279
O +2.20214 +0.04362 +0.61239
C +0.24951 +0.36578 +3.71191
H +1.50053 −1.22270 +3.03767
H +3.41292 +0.35977 +2.63656
C +0.12540 +1.88146 +3.72200
H −0.67600 −0.09209 +3.34850
H +0.39584 +0.05862 +4.74926
O −0.26569 +2.43561 +2.56204
H −0.47669 +1.76013 +1.90998
O +0.34536 +2.55142 +4.68203
16

∗ E = +8.951 kcal/mol ; (93) 180_179_304_102_306_356
H +0.00000 +0.00000 +0.00000
O +0.00000 +0.00000 +0.96444
C +1.27866 +0.00000 +1.40954
C +1.34680 +0.00389 +2.93236
N +2.70270 −0.01891 +3.42661
H +3.20489 −0.81793 +3.05766
O +2.21150 +0.00089 +0.66690
C +0.58770 +1.22682 +3.51020
H +0.84112 −0.91414 +3.26417
H +3.20518 +0.80456 +3.11239
C −0.79240 +0.92054 +4.07350
H +1.17719 +1.62635 +4.33553
H +0.48242 +2.01503 +2.75442
O −1.63924 +0.28013 +3.25528
H −1.26089 +0.14991 +2.37795
O −1.12129 +1.22796 +5.17792
16
∗ E = +9.034 kcal/mol ; (94) 178_039_289_158_155_356
H +0.00000 +0.00000 +0.00000
O +0.00000 +0.00000 +0.96392
C +1.27544 +0.00000 +1.37709
C +1.37968 +0.05739 +2.89687
N +0.38703 −0.72325 +3.61183
H −0.53275 −0.30915 +3.53959
O +2.20154 −0.02414 +0.61621
C +2.77760 −0.33487 +3.34787
H +1.23349 +1.11047 +3.17343
H +0.32626 −1.66692 +3.24761
C +3.06258 +0.22733 +4.72852
H +2.88006 −1.42696 +3.34133
H +3.52823 +0.06311 +2.65598
O +3.99133 −0.43175 +5.44392
H +4.29746 −1.21214 +4.97807
O +2.55047 +1.21221 +5.16147
16
∗ E = +9.055 kcal/mol ; (95) 180_189_047_303_187_000
H +0.00000 +0.00000 +0.00000
O +0.00000 +0.00000 +0.96359
C +1.28374 +0.00000 +1.37091

C	+1.37365	+0.00833	+2.89090
N	+2.74972	+0.21484	+3.26936
H	+3.15222	+0.98299	+2.74473
O	+2.20733	−0.03541	+0.61953
C	+0.35788	+0.99502	+3.48854
H	+1.06272	−0.99873	+3.20559
H	+2.84893	+0.39264	+4.25932
C	+0.55786	+2.39717	+2.95105
H	−0.66869	+0.68364	+3.26124
H	+0.46112	+1.00571	+4.58065
O	−0.19316	+3.35442	+3.52208
H	−0.76413	+2.98914	+4.20137
O	+1.31861	+2.66450	+2.07178

16

∗ E = +9.114 kcal/mol ; (96) 003_195_306_317_323_178

H	+0.00000	+0.00000	+0.00000
O	+0.00000	+0.00000	+0.96027
C	+1.24225	+0.00000	+1.47937
C	+2.41016	+0.06053	+0.47922
N	+3.67635	−0.29284	+1.08145
H	+3.83132	+0.28262	+1.90150
O	+1.40495	−0.01583	+2.65889
C	+2.14921	−0.77120	−0.78851
H	+2.47044	+1.11135	+0.16294
H	+3.63177	−1.25005	+1.41652
C	+1.52479	−2.12286	−0.53422
H	+1.51016	−0.25557	−1.51053
H	+3.11659	−0.92835	−1.27712
O	+2.00364	−2.69337	+0.58240
H	+1.54982	−3.53350	+0.71883
O	+0.69431	−2.63577	−1.22534

16

∗ E = +9.308 kcal/mol ; (97) 004_161_031_079_175_001

H	+0.00000	+0.00000	+0.00000
O	+0.00000	+0.00000	+0.97018
C	+1.23751	+0.00000	+1.44808
C	+2.36285	+0.08639	+0.40744
N	+3.55633	+0.52308	+1.08895
H	+3.55347	+0.19655	+2.04858
O	+1.46591	−0.09438	+2.61823

C +2.51208 −1.27785 −0.30041
H +2.06816 +0.82186 −0.35377
H +4.40742 +0.24269 +0.62362
C +1.46915 −1.53473 −1.36780
H +3.49515 −1.31157 −0.78801
H +2.49594 −2.09878 +0.42865
O +1.62475 −2.65322 −2.08007
H +2.39083 −3.15440 −1.79038
O +0.55327 −0.79732 −1.60548
16
* E = +9.317 kcal/mol ; (98) 353_158_031_174_265_176
H +0.00000 +0.00000 +0.00000
O +0.00000 +0.00000 +0.96037
C +1.25011 +0.00000 +1.44829
C +2.35947 −0.17031 +0.41526
N +3.58816 +0.34902 +0.95271
H +3.59441 +0.23526 +1.96069
O +1.45552 +0.07306 +2.61927
C +2.40142 −1.68118 +0.04587
H +2.07739 +0.38981 −0.49130
H +4.40015 −0.10906 +0.55641
C +3.38828 −1.86321 −1.07298
H +2.74245 −2.24291 +0.91852
H +1.41573 −2.03797 −0.26579
O +2.79836 −1.84372 −2.27759
H +3.48680 −1.90834 −2.95002
O +4.57597 −1.94999 −0.93258
16
* E = +9.419 kcal/mol ; (99) 178_162_035_174_259_356
H +0.00000 +0.00000 +0.00000
O +0.00000 +0.00000 +0.96405
C +1.27432 +0.00000 +1.38244
C +1.36904 +0.05211 +2.89412
N +2.68506 −0.36131 +3.30014
H +3.37417 −0.05324 +2.62364
O +2.20988 +0.00163 +0.63817
C +0.96759 +1.49341 +3.32361
H +0.61191 −0.64044 +3.28842
H +2.92420 +0.00179 +4.21524
C +0.91586 +1.55376 +4.83466

H +1.73737 +2.18915 +2.97930
H +0.00843 +1.76615 +2.87104
O −0.30446 +1.49778 +5.39500
H −0.99533 +1.48349 +4.72987
O +1.89424 +1.59124 +5.51911
16
* E = +9.421 kcal/mol ; (100) 355_294_292_266_041_178
H +0.00000 +0.00000 +0.00000
O +0.00000 +0.00000 +0.96486
C +1.24260 +0.00000 +1.46203
C +2.39883 −0.11529 +0.45598
N +2.45542 −1.39405 −0.23940
H +2.70451 −2.14125 +0.39551
O +1.43342 +0.05680 +2.64016
C +2.47157 +1.05374 −0.54776
H +3.29029 −0.04494 +1.08313
H +1.57398 −1.63382 −0.67647
C +1.78583 +0.84390 −1.87423
H +2.05565 +1.96866 −0.10675
H +3.51470 +1.25867 −0.79143
O +0.58444 +0.21348 −1.76118
H +0.19391 +0.14824 −2.64095
O +2.19466 +1.20613 −2.93224
16
* E = +9.499 kcal/mol ; (101) 000_168_320_303_094_186
H +0.00000 +0.00000 +0.00000
O +0.00000 +0.00000 +0.96001
C +1.24988 +0.00000 +1.45315
C +2.37857 −0.00059 +0.40885
N +3.67913 +0.26462 +0.96695
H +3.62157 +0.98832 +1.67341
O +1.44632 +0.00666 +2.62818
C +2.40127 −1.35106 −0.33991
H +2.13579 +0.77864 −0.32958
H +4.04765 −0.56363 +1.42412
C +2.55484 −2.47267 +0.65889
H +1.48743 −1.50139 −0.91982
H +3.26553 −1.35012 −1.00614
O +1.36938 −2.97080 +1.02875
H +1.51182 −3.61005 +1.73697

O +3.59778 −2.84246 +1.11859
16
∗ E = +9.540 kcal/mol ; (102) 182_093_066_062_182_003
H +0.00000 +0.00000 +0.00000
O +0.00000 +0.00000 +0.96405
C +1.26084 +0.00000 +1.38043
C +1.39317 −0.04764 +2.89610
N +1.51603 −1.45416 +3.24918
H +2.38415 −1.84094 +2.89670
O +2.20958 −0.01592 +0.64470
C +2.58200 +0.82031 +3.30621
H +0.47108 +0.34809 +3.32985
H +1.48504 −1.59189 +4.25065
C +2.36615 +2.26397 +2.90233
H +2.73719 +0.76389 +4.39020
H +3.48971 +0.44602 +2.81537
O +3.36966 +3.11130 +3.18608
H +4.11276 +2.64941 +3.57999
O +1.37152 +2.65258 +2.36982
16
∗ E = +9.614 kcal/mol ; (103) 354_316_195_299_319_358
H +0.00000 +0.00000 +0.00000
O +0.00000 +0.00000 +0.96448
C +1.23029 +0.00000 +1.48577
C +2.39985 −0.15038 +0.50657
N +2.11469 −1.19849 −0.48426
H +2.96688 −1.45963 −0.96872
O +1.40991 +0.11141 +2.65883
C +2.69408 +1.17747 −0.21161
H +3.25846 −0.38988 +1.14258
H +1.75292 −2.03773 −0.04489
C +1.55689 +1.74871 −1.05360
H +2.97579 +1.94421 +0.51143
H +3.55260 +1.02597 −0.87908
O +0.86008 +0.85237 −1.77765
H +1.23493 −0.03973 −1.65067
O +1.29741 +2.91003 −1.08702
16
∗ E = +9.718 kcal/mol ; (104) 353_159_029_168_085_183
H +0.00000 +0.00000 +0.00000

O +0.00000 +0.00000 +0.96072
C +1.24827 +0.00000 +1.45012
C +2.35940 −0.16159 +0.41674
N +3.58796 +0.34304 +0.97222
H +3.57759 +0.23119 +1.98037
O +1.45266 +0.06968 +2.62192
C +2.38531 −1.66209 +0.01234
H +2.08234 +0.41816 −0.47867
H +4.40231 −0.11834 +0.58929
C +3.25101 −1.83427 −1.20649
H +2.77126 −2.24503 +0.85282
H +1.38329 −2.01128 −0.25027
O +4.55260 −1.96070 −0.89432
H +5.05266 −2.02483 −1.71695
O +2.85546 −1.81062 −2.33597
16
* E = +9.743 kcal/mol ; (105) 173_274_300_063_278_000
H +0.00000 +0.00000 +0.00000
O +0.00000 +0.00000 +0.96515
C +1.29371 +0.00000 +1.37850
C +1.42501 +0.16051 +2.88821
N +1.42710 +1.57868 +3.22374
H +2.19957 +2.06111 +2.78065
O +2.20465 −0.02857 +0.60863
C +0.32225 −0.53139 +3.69343
H +2.39896 −0.25835 +3.15813
H +0.56318 +2.02440 +2.93512
C +0.32202 −2.04021 +3.51519
H +0.50446 −0.31954 +4.74722
H −0.65091 −0.10975 +3.41471
O −0.28645 −2.49531 +2.40673
H −0.63282 −1.76622 +1.88231
O +0.83872 −2.79131 +4.28317
16
* E = +9.862 kcal/mol ; (106) 177_167_043_104_306_358
H +0.00000 +0.00000 +0.00000
O +0.00000 +0.00000 +0.96444
C +1.27992 +0.00000 +1.40309
C +1.34422 +0.08096 +2.91312
N +2.69444 −0.22453 +3.31237

H +3.36903 +0.23509 +2.71254
O +2.21127 −0.00269 +0.65984
C +0.80208 +1.48045 +3.35700
H +0.66893 −0.69064 +3.30871
H +2.86896 +0.02107 +4.27684
C −0.60173 +1.45623 +3.94024
H +1.45493 +1.87734 +4.13496
H +0.82208 +2.19152 +2.52137
O −1.54970 +0.89371 +3.17953
H −1.20241 +0.59492 +2.33062
O −0.86182 +1.90241 +5.01637
16
* E = +9.900 kcal/mol ; (107) 174_163_296_060_065_001
H +0.00000 +0.00000 +0.00000
O +0.00000 +0.00000 +0.96440
C +1.26096 +0.00000 +1.40625
C +1.31463 +0.15490 +2.92357
N +2.58764 −0.21368 +3.49644
H +2.77412 −1.19880 +3.35144
O +2.22149 −0.08227 +0.69829
C +1.01863 +1.62542 +3.27710
H +0.53224 −0.49070 +3.34374
H +3.33102 +0.28811 +3.02051
C −0.33774 +2.12538 +2.81517
H +1.10010 +1.72155 +4.36523
H +1.77743 +2.26743 +2.82261
O −1.40009 +1.53887 +3.39946
H −1.12950 +0.88284 +4.04514
O −0.48927 +2.98427 +2.00406
16
* E = +10.070 kcal/mol ; (108) 351_153_044_181_330_182
H +0.00000 +0.00000 +0.00000
O +0.00000 +0.00000 +0.95984
C +1.25178 +0.00000 +1.44661
C +2.36328 −0.21073 +0.42415
N +3.55663 +0.42690 +0.92163
H +3.68506 +0.22207 +1.90611
O +1.45765 +0.08740 +2.61658
C +2.47247 −1.73539 +0.21178
H +2.07992 +0.25819 −0.52799

H	+4.37938	+0.16488	+0.39600
C	+3.54649	−2.13423	−0.77075
H	+2.68992	−2.23680	+1.15899
H	+1.52731	−2.15014	−0.16432
O	+3.75769	−1.19306	−1.71002
H	+4.42921	−1.52563	−2.31700
O	+4.14420	−3.16882	−0.75103

16

* E = +10.112 kcal/mol ; (109) 177_283_298_262_056_005

H	+0.00000	+0.00000	+0.00000
O	+0.00000	+0.00000	+0.96443
C	+1.28372	+0.00000	+1.40390
C	+1.38394	+0.07136	+2.92313
N	+1.12850	+1.40981	+3.42738
H	+1.80454	+2.08076	+3.08761
O	+2.20548	−0.01139	+0.64626
C	+0.50114	−0.96721	+3.64814
H	+2.42951	−0.16755	+3.13346
H	+0.19453	+1.73992	+3.21270
C	−0.84543	−0.44724	+4.13848
H	+0.32799	−1.83838	+3.00458
H	+1.02079	−1.31203	+4.54181
O	−1.64359	+0.11466	+3.21506
H	−1.27043	+0.03558	+2.32900
O	−1.19223	−0.53056	+5.27524

16

* E = +10.281 kcal/mol ; (110) 179_297_069_259_064_358

H	+0.00000	+0.00000	+0.00000
O	+0.00000	+0.00000	+0.96462
C	+1.28632	+0.00000	+1.39771
C	+1.36220	+0.03182	+2.91223
N	+0.76113	+1.27053	+3.37530
H	−0.24163	+1.28757	+3.22364
O	+2.20715	−0.04593	+0.64403
C	+0.79204	−1.28587	+3.52326
H	+2.43075	+0.07088	+3.13304
H	+0.92978	+1.41052	+4.36351
C	−0.62769	−1.13646	+4.04567
H	+0.83117	−2.10242	+2.79138
H	+1.39903	−1.58098	+4.37959

O	−1.57238	−0.85711	+3.13608
H	−1.19903	−0.75549	+2.25231
O	−0.90493	−1.23942	+5.20193

16

∗ E = +10.749 kcal/mol ; (111) 180_001_309_178_298_000

H	+0.00000	+0.00000	+0.00000
O	+0.00000	+0.00000	+0.96459
C	+1.27231	+0.00000	+1.37624
C	+1.41601	+0.01095	+2.89908
N	+0.20904	+0.00099	+3.68527
H	−0.44082	+0.72141	+3.39990
O	+2.20569	+0.00199	+0.62543
C	+2.29805	−1.18137	+3.29014
H	+1.95741	+0.94610	+3.10890
H	−0.26610	−0.89194	+3.62618
C	+2.54101	−1.23335	+4.78717
H	+1.80283	−2.11678	+3.01587
H	+3.24792	−1.12807	+2.74833
O	+3.19529	−0.17921	+5.30868
H	+3.42312	+0.46441	+4.63470
O	+2.20269	−2.14164	+5.47905

16

∗ E = +10.753 kcal/mol ; (112) 178_039_310_183_296_000

H	+0.00000	+0.00000	+0.00000
O	+0.00000	+0.00000	+0.96407
C	+1.27306	+0.00000	+1.38374
C	+1.36569	+0.04083	+2.90822
N	+0.37909	−0.74592	+3.61643
H	−0.55903	−0.56717	+3.28287
O	+2.20726	−0.00341	+0.63544
C	+2.77133	−0.37979	+3.33500
H	+1.19263	+1.09823	+3.16825
H	+0.56857	−1.73811	+3.53293
C	+2.90554	−0.42021	+4.84527
H	+2.98656	−1.38373	+2.96143
H	+3.51441	+0.29595	+2.90007
O	+2.75627	+0.75962	+5.47749
H	+2.58054	+1.47142	+4.85911
O	+3.14122	−1.41171	+5.46155

16

∗ E = +10.936 kcal/mol ; (113) 358_008_289_163_036_000

H	+0.00000	+0.00000	+0.00000
O	+0.00000	+0.00000	+0.96036
C	+1.25244	+0.00000	+1.43489
C	+2.38788	−0.03881	+0.39536
N	+2.00253	+0.10067	−1.00396
H	+1.51135	−0.68924	−1.39920
O	+1.47300	+0.00915	+2.60498
C	+3.43403	+1.01833	+0.76655
H	+2.84194	−1.03183	+0.50971
H	+1.53935	+0.97390	−1.22409
C	+4.79317	+0.84551	+0.09694
H	+3.05234	+2.01326	+0.49845
H	+3.59526	+1.01407	+1.84436
O	+4.77980	+0.39438	−1.16142
H	+3.86635	+0.24980	−1.45669
O	+5.81321	+1.11454	+0.65421

16

∗ E = +11.220 kcal/mol ; (114) 173_159_033_061_063_358

H	+0.00000	+0.00000	+0.00000
O	+0.00000	+0.00000	+0.96433
C	+1.26024	+0.00000	+1.40214
C	+1.31694	+0.16882	+2.91016
N	+2.59272	−0.31456	+3.37843
H	+3.30744	−0.14388	+2.67947
O	+2.22013	−0.07462	+0.69272
C	+1.05606	+1.66279	+3.22967
H	+0.50514	−0.44150	+3.33164
H	+2.86930	+0.11251	+4.25157
C	−0.29525	+2.18227	+2.77086
H	+1.13501	+1.79400	+4.31615
H	+1.82649	+2.27444	+2.75498
O	−1.36727	+1.60702	+3.34783
H	−1.11207	+0.91667	+3.96314
O	−0.43230	+3.04884	+1.96530

16

∗ E = +11.370 kcal/mol ; (115) 355_155_033_307_288_177

H	+0.00000	+0.00000	+0.00000
O	+0.00000	+0.00000	+0.95983
C	+1.24573	+0.00000	+1.44959

C +2.37330 −0.11361 +0.42324
N +3.55892 +0.48033 +0.98757
H +3.56772 +0.32683 +1.99063
O +1.45044 +0.03337 +2.62344
C +2.50134 −1.60766 +0.01457
H +2.08043 +0.43637 −0.48368
H +4.40079 +0.07590 +0.59925
C +2.64826 −2.51856 +1.20922
H +1.61588 −1.93791 −0.53447
H +3.37615 −1.70709 −0.63556
O +3.85118 −2.40244 +1.79259
H +3.85632 −2.96820 +2.57394
O +1.79586 −3.25465 +1.61178
16
* E = +11.461 kcal/mol ; (116) 174_284_074_058_279_359
H +0.00000 +0.00000 +0.00000
O +0.00000 +0.00000 +0.96500
C +1.29447 +0.00000 +1.37908
C +1.38361 +0.15653 +2.88966
N +1.12386 +1.56229 +3.16052
H +0.13974 +1.77885 +3.05184
O +2.20836 −0.08070 +0.62213
C +0.43702 −0.79939 +3.64332
H +2.42169 −0.06375 +3.15156
H +1.40590 +1.81386 +4.09880
C +0.69854 −2.26108 +3.32837
H +0.59047 −0.65979 +4.71487
H −0.59848 −0.53408 +3.39910
O +0.15934 −2.70572 +2.17925
H −0.33870 −2.00949 +1.73922
O +1.35479 −2.97898 +4.01724
16
* E = +11.465 kcal/mol ; (117) 356_175_033_301_188_181
H +0.00000 +0.00000 +0.00000
O +0.00000 +0.00000 +0.95926
C +1.25327 +0.00000 +1.45722
C +2.36534 −0.09900 +0.40900
N +3.63545 +0.02088 +1.08138
H +3.57728 −0.39415 +2.00493
O +1.44543 +0.09294 +2.62505

C +2.21842 −1.35341 −0.46886
H +2.22225 +0.76767 −0.25783
H +4.38859 −0.40788 +0.56174
C +2.24897 −2.63622 +0.32550
H +1.29469 −1.35539 −1.06234
H +3.02915 −1.38222 −1.20510
O +2.30048 −3.71129 −0.47471
H +2.29697 −4.49840 +0.08239
O +2.22167 −2.70775 +1.51852
16
* E = +11.758 kcal/mol ; (118) 178_277_072_055_179_001
H +0.00000 +0.00000 +0.00000
O +0.00000 +0.00000 +0.96384
C +1.28383 +0.00000 +1.37726
C +1.36425 +0.04495 +2.89837
N +1.24139 +1.44480 +3.28106
H +0.29557 +1.78139 +3.14264
O +2.21104 +0.02905 +0.63127
C +0.32103 −0.86648 +3.55076
H +2.37136 −0.29949 +3.14641
H +1.49525 +1.59061 +4.24913
C +0.41341 −2.29303 +3.05209
H +0.45089 −0.85293 +4.64001
H −0.68652 −0.48649 +3.33814
O −0.45291 −3.16259 +3.60257
H −1.02396 −2.72831 +4.23969
O +1.19258 −2.65040 +2.22360
16
* E = +12.113 kcal/mol ; (119) 182_066_303_056_036_003
H +0.00000 +0.00000 +0.00000
O +0.00000 +0.00000 +0.96424
C +1.27348 +0.00000 +1.38811
C +1.35096 −0.06048 +2.91086
N +0.83774 −1.31880 +3.43312
H −0.11601 −1.47809 +3.13155
O +2.21399 +0.00683 +0.65020
C +2.78945 +0.12891 +3.37467
H +0.69321 +0.72875 +3.30470
H +1.40331 −2.09655 +3.11145
C +3.48711 +1.39591 +2.91139

```
H    +2.80396    +0.10861    +4.46975
H    +3.40551    −0.70155    +3.02075
O    +2.72385    +2.50749    +2.83044
H    +1.82568    +2.34945    +3.12590
O    +4.64941    +1.44827    +2.66623
16
* E = +12.357 kcal/mol ; (120) 354_179_212_175_165_355
H    +0.00000    +0.00000    +0.00000
O    +0.00000    +0.00000    +0.95995
C    +1.25146    +0.00000    +1.45378
C    +2.36207    −0.14646    +0.42112
N    +3.64595    −0.10573    +1.07848
H    +4.35030    +0.31241    +0.48708
O    +1.44438    +0.11358    +2.62343
C    +2.23190    −1.47528    −0.33288
H    +2.22690    +0.66730    −0.31257
H    +3.58438    +0.37839    +1.96532
C    +3.26777    −1.52798    −1.44178
H    +2.39469    −2.29852    +0.37205
H    +1.24540    −1.61775    −0.79496
O    +3.49021    −2.73865    −1.97389
H    +2.99855    −3.42068    −1.51116
O    +3.84024    −0.56551    −1.85398
16
* E = +12.951 kcal/mol ; (121) 350_142_048_177_173_359
H    +0.00000    +0.00000    +0.00000
O    +0.00000    +0.00000    +0.95969
C    +1.25108    +0.00000    +1.44383
C    +2.37324    −0.23683    +0.43860
N    +3.47968    +0.61567    +0.79473
H    +3.68457    +0.53991    +1.78440
O    +1.46021    +0.10675    +2.61283
C    +2.67461    −1.74691    +0.47960
H    +2.04686    +0.02009    −0.57786
H    +4.29913    +0.40508    +0.24028
C    +3.72480    −2.12365    −0.54584
H    +3.02288    −2.01305    +1.48614
H    +1.77294    −2.34386    +0.28203
O    +4.16278    −3.39108    −0.48656
H    +3.74669    −3.87268    +0.23208
```

O +4.14839 −1.37167 −1.37031
16
∗ E = +13.698 kcal/mol ; (122) 011_066_195_301_326_002
H +0.00000 +0.00000 +0.00000
O +0.00000 +0.00000 +0.96004
C +1.25251 +0.00000 +1.46212
C +2.35690 +0.25979 +0.43580
N +2.21013 +1.63486 −0.06438
H +2.75154 +1.77952 −0.90792
O +1.45081 −0.17521 +2.61912
C +3.73183 +0.05220 +1.06238
H +2.23082 −0.48746 −0.36606
H +1.24926 +1.89082 −0.25616
C +4.08461 +0.91194 +2.27784
H +3.84515 −0.98571 +1.37956
H +4.49870 +0.24411 +0.30140
O +3.59440 +2.15173 +2.29676
H +3.04671 +2.31071 +1.51075
O +4.81704 +0.50842 +3.12844
16
∗ E = +13.850 kcal/mol ; (123) 006_194_299_301_189_003
H +0.00000 +0.00000 +0.00000
O +0.00000 +0.00000 +0.95791
C +1.24738 +0.00000 +1.46420
C +2.41009 +0.14214 +0.46344
N +3.68668 −0.19353 +1.05532
H +3.87591 +0.43150 +1.82996
O +1.42438 −0.02548 +2.64017
C +2.23181 −0.65439 −0.83086
H +2.42775 +1.20654 +0.19026
H +3.61906 −1.12209 +1.46304
C +2.06862 −2.13740 −0.55165
H +1.38054 −0.31416 −1.43682
H +3.12586 −0.49879 −1.44469
O +2.08921 −2.94211 −1.62467
H +2.25793 −2.45054 −2.43148
O +1.91649 −2.58044 +0.54505
16
∗ E = +14.882 kcal/mol ; (124) 346_179_325_300_083_358
H +0.00000 +0.00000 +0.00000

O +0.00000 +0.00000 +0.96111
C +1.26844 +0.00000 +1.45109
C +2.35414 −0.34243 +0.42406
N +3.68579 −0.31814 +0.96733
H +3.77392 +0.40785 +1.66849
O +1.47159 +0.21895 +2.59976
C +2.06916 −1.72068 −0.20901
H +2.27129 +0.40866 −0.37856
H +3.91332 −1.19966 +1.41626
C +2.04045 −2.80513 +0.85658
H +1.13084 −1.71536 −0.77389
H +2.88527 −1.95141 −0.89526
O +0.88641 −2.95547 +1.52331
H +0.21518 −2.32015 +1.25583
O +2.99334 −3.47118 +1.12562
16
* E = +16.264 kcal/mol ; (125) 358_161_025_061_052_015
H +0.00000 +0.00000 +0.00000
O +0.00000 +0.00000 +0.96314
C +1.22812 +0.00000 +1.48845
C +2.39137 −0.03790 +0.49081
N +3.58599 +0.40563 +1.16225
H +3.49863 +0.24648 +2.16029
O +1.39824 +0.00335 +2.66835
C +2.51587 −1.46544 −0.09876
H +2.14332 +0.66725 −0.32060
H +4.41991 −0.04887 +0.81803
C +1.28740 −1.92912 −0.85229
H +3.37304 −1.47616 −0.78451
H +2.71081 −2.18268 +0.70102
O +0.84233 −1.06194 −1.80654
H +1.50546 −0.39871 −2.01523
O +0.68926 −2.93050 −0.63540
16
* E = +16.581 kcal/mol ; (126) 349_194_202_293_065_004
H +0.00000 +0.00000 +0.00000
O +0.00000 +0.00000 +0.96066
C +1.25969 +0.00000 +1.45930
C +2.37125 −0.25372 +0.44460
N +3.57775 −0.54412 +1.17356

```
 H    +4.41441    −0.38173    +0.63256
 O    +1.44625    +0.18082    +2.61844
 C    +2.06582    −1.41100    −0.52623
 H    +2.41480    +0.67331    −0.15983
 H    +3.61463    −0.03861    +2.04928
 C    +2.01740    −2.77024    +0.15943
 H    +1.13573    −1.25000    −1.08785
 H    +2.86997    −1.46715    −1.26229
 O    +1.03659    −2.94911    +1.06091
 H    +0.43333    −2.20315    +1.12336
 O    +2.78255    −3.64666    −0.09023
16
 * E = +17.472 kcal/mol ; (127) 355_175_034_300_191_001
 H    +0.00000    +0.00000    +0.00000
 O    +0.00000    +0.00000    +0.95901
 C    +1.25240    +0.00000    +1.45980
 C    +2.36740    −0.11850    +0.41513
 N    +3.63463    +0.00287    +1.08969
 H    +3.57683    −0.41700    +2.01127
 O    +1.44425    +0.11206    +2.62513
 C    +2.21888    −1.38501    −0.44889
 H    +2.23076    +0.74030    −0.26327
 H    +4.39330    −0.41675    +0.57060
 C    +2.26395    −2.66390    +0.36928
 H    +1.28476    −1.38871    −1.02819
 H    +3.02861    −1.39742    −1.18968
 O    +2.37263    −3.80013    −0.34387
 H    +2.42463    −3.62360    −1.28548
 O    +2.19673    −2.69126    +1.55653
```

2 Glu

2.1 Reference Z-matrix for Glu

Bond distances are given in Å; bond and dihedral angles, in degrees.

h						
o	1	oh2				
c	2	co3	1	coh3		
c	3	cc4	2	cco4	1	dih4
n	4	nc5	3	ncc5	2	dih5
h	5	hn6	4	hnc6	3	dih6
o	3	oc7	2	oco7	4	dih7
c	4	cc8	3	ccc8	5	dih8
h	4	hc9	3	hcc9	5	dih9
h	5	hn10	4	hnc10	6	dih10
c	8	cc11	4	ccc11	3	dih11
h	8	hc12	4	hcc12	11	dih12
h	8	hc13	4	hcc13	11	dih13
c	11	cc14	8	ccc14	4	dih14
h	11	hc15	8	hcc15	14	dih15
h	11	hc16	8	hcc16	14	dih16
o	14	oc17	11	occ17	8	dih17
h	17	ho18	14	hoc18	11	dih18
o	14	oc19	11	occ19	17	dih19

oh2	0.96842597
co3	1.35957659
coh3	111.95160616
cc4	1.51091486
cco4	110.92207252
dih4	180.46474053
nc5	1.46817695
ncc5	107.17124694
dih5	46.53785957
hn6	1.00247685
hnc6	113.72355713
dih6	186.87185845
oc7	1.20007521
oco7	122.12378381
dih7	181.84336758

cc8	1.52797156
ccc8	109.18241073
dih8	120.31298926
hc9	1.09201238
hcc9	106.26025788
dih9	238.59535257
hn10	1.00279656
hnc10	112.62572657
dih10	128.80194382
cc11	1.52976577
ccc11	112.15226542
dih11	176.69661271
hc12	1.08063799
hcc12	108.04559714
dih12	121.06298157
hc13	1.08041506
hcc13	109.49618626
dih13	237.92021315
cc14	1.50107061
ccc14	111.36551379
dih14	185.48591876
hc15	1.08598289
hcc15	111.67322794
dih15	119.98800304
hc16	1.08300193
hcc16	111.66978095
dih16	240.12209656
oc17	1.35836528
occ17	110.69135737
dih17	182.89016708
ho18	0.96857741
hoc18	111.81190577
dih18	180.2134146
oc19	1.20151688
occ19	127.01194971
dih19	180.32931333

2.2 Reference energy and target torsions for Glu

- Reference M08-HX/MG3S energy: -551.6503579 hartree
- Target torsions:
  - torsion1: dih4
  - torsion2: dih5
  - torsion3: dih6
  - torsion4: dih11
  - torsion5: dih14
  - torsion6: dih17
  - torsion7: dih18

2.3 Properties of Glu conformers

Table S2: Relative energy (kcal mol^{-1}), rotational constants (B1 to B3, GHz) and dipolar moment (Debye) for the conformers of Glu located at the M08-HX/MG3S level.

Conformer	Rel. Energy	B1	B2	B3	Dip. Mom.
1	0.000	0.767	0.796	2.345	4.119
2	0.525	1.041	1.212	1.810	4.671
3	0.592	0.627	0.678	3.051	2.308
4	0.701	0.665	0.811	2.440	5.286
5	0.842	0.612	0.683	2.789	0.898
6	0.866	0.580	0.646	3.202	6.012
7	0.928	0.657	0.756	2.367	3.962
8	1.061	0.757	0.901	2.281	5.924
9	1.100	1.011	1.199	1.828	0.455
10	1.112	0.733	0.770	2.496	1.446
11	1.185	0.760	0.782	2.445	1.628
12	1.206	0.943	1.124	1.814	0.549
13	1.303	0.655	0.728	2.571	4.783
14	1.602	0.749	0.871	2.294	4.623
15	1.655	0.621	0.669	3.082	1.795

16	1.685	0.611	0.704	2.723	4.737
17	1.735	0.655	0.725	2.674	4.916
18	1.740	0.522	0.568	2.903	1.944
19	1.850	1.093	1.252	1.656	0.857
20	1.869	0.631	0.672	2.370	1.183
21	1.906	0.640	0.708	2.828	4.321
22	1.925	0.854	1.007	2.161	0.323
23	1.975	0.786	0.931	2.146	2.026
24	1.984	0.730	0.753	2.507	2.725
25	1.993	0.936	1.112	1.855	2.221
26	2.003	0.992	1.151	1.858	5.969
27	2.017	0.869	1.019	2.147	5.003
28	2.072	0.630	0.644	2.755	1.817
29	2.075	0.898	0.957	2.231	5.770
30	2.101	0.639	0.690	2.971	2.658
31	2.108	0.697	0.784	2.449	6.831
32	2.137	0.817	0.976	2.175	2.622
33	2.316	0.574	0.668	2.446	3.994
34	2.325	0.616	0.654	2.879	6.305
35	2.340	0.578	0.652	3.059	6.739
36	2.448	0.667	0.754	2.273	1.264
37	2.555	0.657	0.686	2.275	4.764
38	2.642	0.609	0.652	2.504	4.680
39	2.654	0.660	0.736	2.544	0.698
40	2.661	0.645	0.755	2.410	2.889
41	2.661	0.660	0.734	2.508	5.589
42	2.748	1.062	1.204	1.704	3.048
43	2.758	0.634	0.724	2.571	2.550
44	2.765	0.538	0.620	2.804	4.189
45	2.768	0.717	0.783	2.098	1.318
46	2.811	0.646	0.714	2.650	1.014
47	2.827	0.611	0.668	2.780	2.873
48	2.834	0.988	1.165	1.807	0.881
49	2.843	1.022	1.179	1.823	5.766
50	2.861	0.795	0.817	2.345	3.739
51	2.864	0.599	0.645	2.630	2.776
52	2.872	0.542	0.571	2.775	1.828

53	2.921	0.752	0.798	2.522	3.114
54	3.030	0.727	0.846	2.221	2.422
55	3.058	0.615	0.701	2.383	3.178
56	3.105	0.775	0.804	2.383	3.440
57	3.111	0.772	0.911	2.154	2.653
58	3.117	0.963	1.130	1.865	3.125
59	3.124	0.854	1.003	2.183	2.229
60	3.132	0.679	0.747	2.540	4.116
61	3.146	0.749	0.871	2.311	6.105
62	3.162	0.649	0.680	2.260	1.359
63	3.205	0.813	0.968	2.206	3.303
64	3.216	0.532	0.562	2.883	2.015
65	3.309	0.852	1.016	2.122	0.570
66	3.312	0.521	0.590	2.801	2.845
67	3.338	0.579	0.680	2.316	2.847
68	3.344	0.506	0.558	3.102	6.199
69	3.352	0.630	0.682	2.987	2.292
70	3.368	0.614	0.695	2.710	2.065
71	3.390	0.647	0.719	2.681	6.190
72	3.398	0.753	0.800	2.445	1.642
73	3.414	0.801	0.954	2.113	1.209
74	3.445	0.559	0.632	2.637	2.622
75	3.468	0.683	0.793	2.306	2.741
76	3.546	0.635	0.717	2.243	3.223
77	3.556	0.963	1.033	1.896	0.774
78	3.582	0.935	1.100	1.929	1.040
79	3.583	0.635	0.678	2.378	3.648
80	3.600	0.664	0.808	2.464	6.596
81	3.648	0.862	1.019	2.163	6.496
82	3.677	0.599	0.639	2.644	2.939
83	3.706	0.897	1.078	1.953	3.426
84	3.725	0.591	0.671	2.321	1.357
85	3.738	0.879	1.095	1.963	0.802
86	3.744	0.667	0.773	2.293	5.944
87	3.757	0.678	0.825	2.422	7.690
88	3.785	0.792	0.894	2.109	0.435
89	3.802	0.606	0.670	2.525	4.366

90	3.815	0.898	1.074	1.877	2.732
91	3.816	0.785	0.816	2.437	3.499
92	3.849	1.015	1.151	1.819	7.036
93	3.862	0.647	0.711	2.293	4.322
94	3.866	0.751	0.889	2.255	6.808
95	3.892	0.657	0.726	2.545	2.982
96	3.896	1.068	1.226	1.706	4.749
97	3.905	0.724	0.793	2.056	1.264
98	3.936	0.742	0.763	2.474	2.833
99	3.941	0.571	0.672	2.371	2.383
100	3.950	0.681	0.754	2.490	3.379
101	3.952	0.945	1.064	1.866	2.065
102	3.964	0.544	0.624	2.773	3.137
103	3.985	0.753	0.817	2.738	5.595
104	3.988	1.019	1.210	1.815	1.861
105	3.994	0.605	0.654	2.442	5.353
106	4.006	0.630	0.697	2.843	0.844
107	4.014	0.834	0.954	1.976	2.567
108	4.042	0.526	0.563	2.957	5.038
109	4.066	0.651	0.676	2.560	2.321
110	4.073	0.651	0.669	2.245	3.191
111	4.083	0.958	1.021	2.008	3.544
112	4.098	0.635	0.709	2.313	6.453
113	4.125	0.512	0.586	2.895	3.921
114	4.145	0.627	0.762	2.644	7.869
115	4.191	0.630	0.713	2.288	1.915
116	4.234	0.521	0.574	2.842	2.229
117	4.235	0.723	0.765	2.555	2.729
118	4.275	0.680	0.773	2.526	7.131
119	4.282	0.594	0.673	2.525	6.267
120	4.312	0.574	0.648	3.079	6.279
121	4.325	0.577	0.674	2.418	6.417
122	4.340	0.786	0.817	2.276	1.525
123	4.348	0.558	0.611	2.810	5.141
124	4.351	0.677	0.746	2.485	1.340
125	4.352	0.718	0.807	2.097	2.806
126	4.355	0.641	0.711	2.553	2.779

127	4.356	0.669	0.732	2.537	5.683
128	4.376	0.607	0.667	2.316	1.398
129	4.377	0.544	0.565	2.805	3.642
130	4.377	0.522	0.555	3.111	3.323
131	4.386	0.569	0.634	3.050	4.911
132	4.389	0.587	0.668	2.631	4.285
133	4.414	0.849	1.008	2.148	1.949
134	4.417	0.635	0.718	2.268	2.601
135	4.431	0.541	0.624	2.739	2.987
136	4.457	0.589	0.681	2.305	2.212
137	4.490	0.582	0.668	2.353	1.001
138	4.495	0.983	1.188	1.830	2.350
139	4.503	0.812	0.994	2.101	5.192
140	4.504	0.647	0.684	2.912	3.288
141	4.518	0.603	0.652	2.646	3.672
142	4.538	0.759	0.894	2.257	5.894
143	4.548	0.959	1.141	1.776	3.254
144	4.569	0.684	0.803	2.229	1.159
145	4.573	0.683	0.733	2.537	3.045
146	4.573	0.775	0.931	2.104	2.308
147	4.578	0.824	0.900	1.958	3.130
148	4.600	0.579	0.622	2.647	2.907
149	4.607	1.043	1.196	1.717	3.542
150	4.655	0.847	1.006	2.134	3.245
151	4.705	0.776	0.915	2.168	3.320
152	4.727	0.511	0.573	3.035	2.945
153	4.738	0.842	1.048	2.033	1.672
154	4.752	0.901	0.961	2.269	5.201
155	4.758	0.533	0.586	2.738	2.203
156	4.763	0.946	1.125	1.813	0.594
157	4.774	0.796	0.940	2.139	5.393
158	4.789	0.635	0.671	3.060	2.729
159	4.803	0.493	0.565	3.245	3.726
160	4.809	0.898	1.068	1.895	3.897
161	4.810	0.930	1.091	1.947	2.825
162	4.856	0.664	0.814	2.485	7.701
163	4.892	0.624	0.662	2.308	3.451

164	4.904	0.664	0.722	2.559	1.537
165	4.905	1.092	1.160	1.781	3.937
166	4.924	0.645	0.677	2.274	2.721
167	4.925	0.840	1.049	2.005	2.878
168	4.944	0.604	0.687	2.275	2.035
169	4.946	0.863	0.884	2.274	4.914
170	4.953	0.595	0.656	2.640	2.883
171	4.959	0.667	0.773	2.312	3.240
172	4.968	0.755	0.879	2.077	2.982
173	5.014	0.807	0.847	2.348	2.018
174	5.024	0.940	1.055	1.892	2.455
175	5.028	0.598	0.660	2.586	2.954
176	5.054	0.647	0.763	2.542	7.052
177	5.065	0.875	0.914	2.263	6.198
178	5.083	0.743	0.866	2.248	2.432
179	5.111	0.637	0.693	2.257	2.344
180	5.125	0.669	0.693	2.853	2.924
181	5.134	0.632	0.696	2.813	3.506
182	5.137	0.662	0.803	2.416	2.179
183	5.152	0.612	0.666	2.835	4.051
184	5.161	0.668	0.813	2.437	6.502
185	5.171	0.582	0.674	2.348	2.024
186	5.174	0.659	0.810	2.479	6.940
187	5.250	0.566	0.614	2.790	2.935
188	5.260	0.677	0.718	2.547	3.342
189	5.261	0.637	0.700	2.268	1.941
190	5.268	0.548	0.577	2.676	1.126
191	5.279	0.607	0.657	2.354	0.830
192	5.281	0.616	0.689	2.768	2.902
193	5.295	0.528	0.599	2.672	2.519
194	5.298	0.738	0.771	2.505	3.190
195	5.334	0.640	0.688	2.977	4.974
196	5.343	0.786	0.967	2.162	6.161
197	5.361	0.528	0.565	2.856	2.799
198	5.370	0.636	0.760	2.613	7.411
199	5.405	0.760	0.801	2.485	4.149
200	5.413	0.975	1.129	1.929	2.711

201	5.478	0.544	0.639	2.741	3.893
202	5.536	0.872	1.054	1.950	2.736
203	5.539	0.518	0.564	3.096	2.645
204	5.587	0.538	0.646	2.682	2.100
205	5.596	0.775	0.942	2.091	1.049
206	5.633	0.839	0.965	1.930	0.465
207	5.636	0.507	0.577	3.066	3.152
208	5.706	0.862	1.071	1.990	0.892
209	5.708	0.698	0.815	2.240	3.766
210	5.711	0.577	0.646	3.197	5.673
211	5.736	0.942	1.116	1.845	2.130
212	5.798	0.844	1.000	2.155	3.747
213	5.798	0.620	0.657	2.339	3.141
214	5.816	0.592	0.677	2.320	2.835
215	5.835	0.810	1.010	2.082	4.056
216	5.865	0.951	1.124	1.825	3.070
217	6.006	0.627	0.700	2.301	2.659
218	6.041	0.752	0.886	2.222	3.228
219	6.047	0.788	0.963	2.027	7.520
220	6.052	0.743	0.882	2.200	4.538
221	6.087	0.681	0.762	2.451	4.578
222	6.144	0.640	0.699	2.284	0.384
223	6.158	0.956	1.137	1.932	4.272
224	6.160	0.630	0.680	3.031	4.564
225	6.188	1.028	1.205	1.797	2.724
226	6.319	0.630	0.678	3.016	3.821
227	6.321	0.884	1.031	2.126	2.621
228	6.332	0.555	0.567	2.710	3.070
229	6.365	0.837	1.040	2.052	1.963
230	6.391	0.537	0.589	2.661	0.885
231	6.391	0.637	0.670	2.346	3.532
232	6.417	0.685	0.829	2.316	7.343
233	6.422	0.750	0.875	2.287	5.038
234	6.448	0.984	1.167	1.807	4.057
235	6.468	0.520	0.572	2.886	4.268
236	6.524	0.571	0.673	2.442	3.788
237	6.533	0.740	0.765	2.499	4.695

238	6.548	0.837	0.998	2.154	4.653
239	6.581	0.634	0.650	2.730	4.120
240	6.590	0.937	1.111	1.955	3.357
241	6.644	0.748	0.772	2.516	3.139
242	6.662	0.642	0.674	2.327	2.671
243	6.697	0.726	0.751	2.520	3.603
244	6.732	0.646	0.717	2.787	3.262
245	6.738	0.672	0.765	2.246	2.180
246	6.738	0.654	0.767	2.494	5.986
247	6.764	0.667	0.733	2.613	2.261
248	6.817	0.748	0.886	2.207	3.570
249	6.887	0.655	0.807	2.486	5.934
250	6.890	0.653	0.680	2.219	2.001
251	6.972	0.518	0.571	2.897	4.022
252	7.058	0.533	0.628	2.767	3.875
253	7.060	0.939	1.110	1.960	2.969
254	7.117	0.771	0.915	2.152	3.637
255	7.209	0.679	0.828	2.313	5.396
256	7.234	0.597	0.645	2.646	5.338
257	7.300	0.654	0.698	2.261	4.686
258	7.301	0.723	0.797	2.075	3.682
259	7.386	0.940	1.105	1.845	3.959
260	7.460	0.601	0.662	2.499	4.346
261	7.467	0.615	0.673	2.766	5.659
262	7.473	0.669	0.742	2.523	2.029
263	7.536	0.617	0.648	2.975	7.130
264	7.537	0.641	0.691	2.954	2.551
265	7.603	0.885	1.026	2.147	3.734
266	7.607	0.684	0.833	2.261	4.277
267	7.642	0.990	1.092	1.858	2.587
268	7.656	0.836	0.992	2.180	5.117
269	7.672	1.085	1.237	1.649	3.346
270	7.673	0.693	0.774	2.470	6.591
271	7.757	0.656	0.679	2.252	2.668
272	7.790	0.672	0.817	2.344	6.923
273	7.823	0.677	0.819	2.361	3.816
274	7.830	0.657	0.790	2.397	4.439

275	7.924	0.685	0.808	2.286	4.803
276	7.935	0.651	0.680	2.250	2.600
277	7.971	0.671	0.809	2.399	3.837
278	7.974	0.532	0.565	2.870	4.400
279	8.029	1.073	1.216	1.648	5.103
280	8.029	0.929	1.037	1.851	3.410
281	8.036	0.578	0.651	3.050	5.793
282	8.052	0.685	0.759	2.513	3.910
283	8.087	0.696	0.821	2.228	4.330
284	8.089	0.698	0.752	2.627	3.331
285	8.114	0.761	0.824	2.727	6.940
286	8.116	0.544	0.572	2.742	1.427
287	8.194	0.902	1.076	1.963	3.216
288	8.232	0.745	0.791	2.562	5.005
289	8.243	0.750	0.871	2.312	6.951
290	8.250	0.898	0.958	2.243	6.102
291	8.254	0.886	0.950	2.241	2.797
292	8.324	0.554	0.636	2.639	5.486
293	8.350	0.617	0.704	2.363	5.132
294	8.351	0.921	1.095	1.859	4.813
295	8.399	0.861	1.021	2.120	3.475
296	8.407	0.575	0.676	2.325	5.180
297	8.445	0.629	0.705	2.349	4.664
298	8.488	0.585	0.683	2.293	3.669
299	8.498	0.639	0.712	2.240	4.123
300	8.498	0.522	0.597	2.726	3.250
301	8.506	0.722	0.796	2.050	2.830
302	8.529	0.957	0.978	2.042	2.657
303	8.539	0.928	1.111	1.914	5.518
304	8.583	0.789	0.816	2.362	5.812
305	8.602	0.538	0.629	2.764	5.354
306	8.631	0.730	0.791	2.435	4.986
307	8.691	0.594	0.635	2.684	5.788
308	8.694	0.789	0.829	2.320	3.612
309	8.730	0.788	0.829	2.350	5.717
310	8.744	0.615	0.746	2.606	4.478
311	8.767	0.740	0.765	2.472	3.932

312	8.801	0.855	0.889	2.223	2.933
313	8.828	0.617	0.754	2.542	4.103
314	8.852	0.529	0.599	2.650	5.263
315	8.894	0.944	1.054	1.839	2.981
316	8.910	0.511	0.556	3.077	7.236
317	8.931	0.568	0.670	2.378	3.656
318	8.940	0.580	0.677	2.331	4.043
319	8.977	0.778	0.810	2.467	5.653
320	8.989	0.630	0.694	2.860	3.027
321	8.997	0.673	0.812	2.336	2.854
322	8.997	0.579	0.670	2.359	2.180
323	9.036	0.904	0.966	2.271	5.420
324	9.102	0.682	0.759	2.480	5.180
325	9.134	0.769	0.937	2.057	2.918
326	9.146	0.961	1.145	1.788	5.375
327	9.221	0.506	0.585	2.921	3.887
328	9.255	0.643	0.711	2.264	4.362
329	9.273	0.631	0.714	2.295	4.480
330	9.283	0.623	0.724	2.616	5.605
331	9.290	0.651	0.664	2.241	5.717
332	9.373	0.538	0.628	2.716	4.881
333	9.388	0.612	0.669	2.303	1.909
334	9.395	1.094	1.238	1.664	3.675
335	9.530	0.522	0.574	2.838	3.155
336	9.549	0.924	1.091	1.874	5.950
337	9.647	0.640	0.681	2.939	4.408
338	9.658	0.860	1.072	1.955	4.544
339	9.719	0.635	0.729	2.250	3.796
340	9.733	0.709	0.752	2.285	2.753
341	9.735	0.865	0.890	2.276	5.220
342	9.735	0.681	0.748	2.523	5.101
343	9.750	0.528	0.556	3.041	6.327
344	9.756	0.666	0.721	2.570	3.858
345	9.816	0.656	0.687	2.820	5.194
346	9.817	0.604	0.652	3.104	5.693
347	9.827	0.784	0.826	2.441	5.916
348	9.838	0.590	0.683	2.290	4.237

349	9.854	0.565	0.648	2.468	3.811
350	9.858	0.790	0.814	2.339	5.086
351	9.921	0.647	0.748	2.504	2.858
352	9.922	0.621	0.661	3.122	3.546
353	9.928	0.592	0.676	2.293	4.935
354	9.938	0.512	0.587	2.857	5.502
355	10.032	0.934	1.046	1.884	3.340
356	10.052	0.642	0.679	2.269	4.038
357	10.109	0.613	0.663	2.328	2.774
358	10.112	0.654	0.684	2.947	4.431
359	10.192	0.622	0.699	2.302	5.587
360	10.239	0.581	0.674	2.585	6.322
361	10.256	0.653	0.718	2.659	6.918
362	10.297	0.506	0.567	3.151	5.593
363	10.319	0.609	0.685	2.256	4.258
364	10.350	0.586	0.678	2.322	4.807
365	10.393	0.672	0.690	2.835	4.231
366	10.408	0.528	0.599	2.668	3.931
367	10.420	0.549	0.581	2.640	2.783
368	10.468	0.611	0.662	2.871	6.383
369	10.477	0.762	0.810	2.515	3.482
370	10.524	0.558	0.648	2.566	6.012
371	10.537	0.831	0.873	2.224	3.426
372	10.568	0.899	1.116	1.942	4.301
373	10.716	0.529	0.565	2.838	3.813
374	10.744	0.928	1.077	1.844	8.696
375	10.747	0.706	0.788	2.272	3.859
376	10.826	0.530	0.600	2.629	3.840
377	10.838	0.587	0.682	2.513	7.904
378	10.868	0.553	0.632	2.660	4.944
379	10.878	0.549	0.580	2.625	2.517
380	10.918	0.743	0.796	2.755	3.901
381	10.922	0.902	1.062	1.958	5.117
382	11.004	0.649	0.678	2.294	2.432
383	11.005	0.768	0.933	2.076	4.027
384	11.041	0.594	0.667	2.334	3.724
385	11.084	0.559	0.642	3.114	5.350

386	11.184	0.643	0.690	2.953	6.628
387	11.538	0.658	0.679	2.209	2.950
388	11.626	0.639	0.733	2.717	7.056
389	11.647	0.544	0.639	2.724	6.819
390	11.752	0.537	0.592	2.617	2.423
391	11.798	0.851	1.064	1.997	6.326
392	11.807	0.515	0.577	2.884	5.010
393	11.932	0.715	0.760	2.602	2.111
394	12.014	0.657	0.806	2.531	9.097
395	12.085	0.627	0.709	2.284	4.553
396	12.117	0.952	1.109	1.935	4.548
397	12.146	0.555	0.566	2.674	4.589
398	12.319	0.657	0.686	2.238	1.043
399	12.378	0.695	0.830	2.270	3.695
400	12.625	0.888	1.064	1.953	6.097
401	13.149	0.846	1.045	1.992	6.285
402	13.237	0.541	0.592	2.668	3.866
403	13.854	0.590	0.697	2.243	4.883
404	14.072	0.728	0.887	2.127	4.556
405	14.181	0.584	0.688	2.285	3.775
406	14.527	0.641	0.683	2.221	4.210
407	14.597	0.620	0.722	2.634	6.201
408	14.629	0.754	0.800	2.499	7.041
409	15.459	0.775	0.819	2.494	7.443
410	15.617	1.065	1.194	1.670	7.130
411	15.771	0.587	0.684	2.262	5.903
412	15.848	0.861	1.044	1.903	6.819
413	16.758	0.596	0.673	2.298	5.769
414	16.939	0.533	0.597	2.608	4.652
415	17.494	0.929	1.099	1.790	7.696

2.4 Vibrational frequencies of Glu conformers

Harmonic vibrational zero point energy (ZPE) and frequencies for each conformer in cm^{-1}.

- Conformer (1); ZPE = 33915.153 cm^{-1}:

39.17	44.93	86.64	151.92	185.59	246.35
313.32	360.18	378.46	418.87	495.85	525.41
555.34	626.24	648.27	662.39	766.73	818.60
832.30	853.83	907.95	927.18	961.69	1031.46
1061.63	1153.67	1172.68	1191.32	1223.19	1246.38
1289.54	1318.33	1360.81	1370.51	1385.50	1430.22
1446.58	1451.52	1466.82	1658.73	1847.11	1889.15
3038.61	3048.95	3062.68	3088.78	3119.60	3545.32
3633.95	3645.99	3844.04			

- Conformer (2); ZPE = 33959.954 cm^{-1}:

77.64	88.73	111.46	116.97	174.40	231.00
318.08	358.75	377.84	439.92	504.12	550.57
562.48	579.78	610.67	741.96	764.23	769.46
799.16	853.27	869.48	935.92	980.78	996.73
1098.81	1127.80	1159.80	1209.90	1236.02	1247.19
1257.90	1324.47	1343.80	1366.22	1398.39	1435.01
1436.52	1469.18	1489.37	1640.22	1835.81	1881.01
3029.78	3060.52	3074.75	3112.90	3137.94	3531.68
3644.08	3717.90	3839.53			

- Conformer (3); ZPE = 33706.106 cm^{-1}:

40.98	47.32	55.41	136.10	177.75	247.42
283.32	306.35	317.70	384.71	474.46	520.09
539.03	579.10	623.09	648.90	678.25	746.02
823.48	854.04	893.26	952.99	990.89	1015.03
1070.87	1141.53	1162.72	1197.26	1214.74	1236.50
1289.62	1311.60	1335.73	1358.86	1393.22	1411.41
1436.05	1450.32	1474.70	1665.23	1853.47	1868.47
3013.97	3052.21	3054.07	3108.80	3122.09	3535.56
3621.58	3846.32	3849.62			

- Conformer (4); ZPE = 33904.681 cm^{-1}:

64.19	86.83	110.14	137.67	200.05	231.25
252.47	298.69	320.50	404.78	503.19	524.52
536.08	634.24	652.37	688.26	737.25	766.30
825.42	865.72	899.53	948.54	1007.35	1016.33
1096.20	1167.53	1175.82	1213.53	1219.44	1236.13
1310.58	1319.15	1329.83	1390.09	1402.45	1428.36
1438.58	1457.68	1476.49	1672.55	1822.54	1889.19
3033.15	3049.82	3059.23	3097.81	3111.11	3534.40
3612.47	3715.41	3838.15			

- Conformer (5); ZPE = 33645.610 cm^{-1}:

40.32	48.83	50.71	107.48	187.15	203.91
241.00	285.24	309.02	386.57	483.22	508.04
536.59	594.17	634.83	648.89	674.57	748.51
805.74	858.88	878.25	958.04	994.04	1000.21
1080.16	1145.81	1164.12	1192.56	1200.91	1244.21
1276.08	1312.11	1334.81	1362.67	1382.95	1424.60
1435.54	1452.56	1468.25	1670.81	1856.60	1860.52
3040.27	3061.45	3073.12	3101.63	3111.86	3538.46
3623.79	3843.66	3847.53			

- Conformer (6); ZPE = 33842.622 cm^{-1}:

32.39	42.63	70.66	139.01	180.06	251.16
276.68	300.31	365.98	408.98	501.34	519.91
550.38	614.79	627.90	674.73	753.36	818.22
840.55	854.18	882.11	948.04	974.72	1031.23
1074.98	1146.91	1165.51	1203.44	1233.77	1253.62
1286.09	1306.68	1332.32	1382.22	1395.41	1434.70
1441.45	1447.13	1473.89	1670.40	1849.13	1885.76
3031.02	3047.23	3053.34	3089.43	3121.25	3556.50
3645.06	3654.46	3844.24			

- Conformer (7); ZPE = 33832.809 cm^{-1}:

45.91	52.35	66.13	108.34	203.83	278.09
293.63	312.55	360.81	375.56	503.57	521.92
567.85	568.71	634.61	666.07	743.26	793.80
807.64	851.89	874.39	943.25	978.61	1066.80
1095.03	1148.19	1175.11	1193.40	1217.88	1239.51
1264.61	1314.17	1343.63	1374.59	1412.17	1427.28
1440.72	1456.44	1465.26	1657.87	1845.49	1882.42
3044.94	3054.31	3065.25	3099.13	3110.90	3540.45
3632.23	3699.32	3845.77			

- Conformer (8); ZPE = 34079.571 cm^{-1}:

56.83	80.91	115.05	173.26	200.40	242.92
256.98	324.87	354.07	412.23	474.64	557.06
606.80	649.66	736.86	773.86	796.75	816.42
839.21	861.64	876.81	935.07	988.56	1045.21
1064.49	1136.26	1172.77	1179.50	1268.19	1279.16
1302.94	1341.81	1354.05	1372.48	1389.81	1424.21
1447.47	1483.34	1487.49	1659.79	1850.36	1882.89
3028.18	3040.78	3076.00	3087.51	3139.68	3560.69
3643.89	3645.08	3664.25			

- Conformer (9); ZPE = 33887.131 cm^{-1}:

65.52	71.52	105.91	127.24	171.87	232.64
288.68	332.82	362.61	433.84	458.33	537.28
557.09	576.48	607.83	632.03	750.38	770.17
795.79	863.82	897.43	943.77	981.32	1012.38
1081.19	1140.61	1166.30	1197.80	1227.29	1246.64
1264.35	1320.67	1340.69	1369.91	1391.10	1407.27
1430.52	1467.18	1488.27	1674.92	1834.92	1849.55
3028.83	3071.86	3078.71	3123.91	3140.68	3531.30
3625.94	3844.24	3852.88			

- Conformer (10); ZPE = 33780.754 cm^{-1}:

36.22	60.79	82.04	125.16	187.65	261.11
296.44	352.83	385.79	408.82	475.78	524.57
558.22	574.98	621.19	647.22	680.14	753.49
812.84	836.20	904.66	927.44	963.06	1017.61
1068.18	1149.65	1176.21	1191.89	1195.93	1230.68
1269.11	1316.13	1338.28	1362.26	1390.95	1433.22
1450.60	1454.67	1464.15	1645.86	1848.49	1868.53
2948.19	3058.23	3069.62	3114.07	3126.17	3552.33
3647.49	3847.82	3848.58			

- Conformer (11); ZPE = 33736.313 cm^{-1}:

43.49	45.76	79.62	115.01	178.51	264.30
280.13	337.53	394.85	431.41	499.19	518.59
540.77	576.38	621.99	646.43	683.42	745.16
800.38	836.40	879.03	928.47	970.43	1012.81
1069.01	1148.10	1174.92	1193.44	1205.01	1230.31
1256.37	1316.18	1343.39	1355.04	1387.90	1432.72
1447.20	1450.38	1479.02	1626.32	1851.97	1866.93
2943.60	3051.96	3068.51	3101.34	3128.38	3560.67
3658.78	3845.14	3849.96			

- Conformer (12); ZPE = 33695.361 cm^{-1}:

41.28	52.67	110.35	129.11	164.76	185.79
262.34	284.00	341.91	402.24	479.12	515.90
525.37	581.61	626.92	662.40	672.58	774.33
790.68	856.63	892.31	931.01	974.94	1006.25
1090.47	1150.30	1159.46	1187.86	1194.02	1236.49
1262.43	1306.28	1322.37	1361.47	1385.13	1433.61
1447.12	1457.02	1463.80	1671.53	1850.89	1855.42
3056.49	3066.20	3082.88	3104.58	3124.14	3538.33
3625.39	3843.67	3848.88			

- Conformer (13); ZPE = 33924.965 cm^{-1}:

28.10	57.48	85.63	123.43	176.56	205.72
327.81	357.03	390.52	415.35	444.50	530.42
582.81	605.52	644.46	741.74	766.41	799.26
806.38	860.79	884.74	958.61	978.93	1013.15
1090.32	1130.36	1162.63	1206.98	1232.08	1250.37
1263.51	1313.95	1342.97	1365.75	1397.10	1421.73
1434.28	1473.90	1485.19	1657.48	1844.51	1889.10
3037.15	3053.09	3077.00	3111.81	3137.24	3533.06
3619.75	3695.14	3838.14			

- Conformer (14); ZPE = 33949.162 cm^{-1}:

42.94	83.35	131.15	162.90	179.79	211.26
241.71	299.90	320.56	379.36	458.35	531.14
592.51	648.73	665.59	743.85	778.09	802.83
813.47	862.02	885.79	941.91	989.45	1063.79
1068.27	1155.53	1178.02	1187.01	1218.29	1250.43
1294.16	1334.49	1344.88	1358.29	1403.43	1429.28
1441.22	1476.47	1491.22	1669.61	1817.87	1878.61
3048.20	3061.60	3068.36	3105.32	3137.38	3530.00
3614.43	3674.49	3831.02			

- Conformer (15); ZPE = 33737.489 cm^{-1}:

20.58	44.20	63.18	142.13	184.03	253.54
295.61	330.09	353.76	394.27	488.04	497.99
534.87	582.37	622.52	643.26	673.89	747.70
815.31	850.37	886.40	949.97	982.71	1018.76
1070.12	1141.31	1179.10	1189.81	1206.67	1243.52
1299.32	1312.32	1328.52	1365.56	1394.70	1411.73
1437.84	1450.79	1475.16	1659.38	1852.83	1862.01
2996.23	3051.26	3063.65	3103.62	3126.72	3542.02
3639.89	3847.48	3847.85			

- Conformer (16); ZPE = 33915.449 cm^{-1}:

30.14	58.58	84.19	109.63	183.26	274.93
302.81	344.27	387.59	414.11	513.18	525.73
544.76	584.01	635.29	679.66	745.15	802.41
815.48	857.90	908.97	964.51	973.28	1060.45
1103.68	1150.12	1170.41	1194.40	1234.45	1249.90
1264.89	1316.68	1337.03	1389.11	1400.61	1430.63
1439.62	1454.43	1464.99	1666.11	1843.50	1886.73
3024.68	3048.25	3057.62	3096.03	3117.61	3535.33
3619.27	3690.75	3843.78			

- Conformer (17); ZPE = 33940.744 cm^{-1}:

44.53	59.66	102.37	158.15	180.05	209.65
284.85	316.48	351.09	409.38	462.84	525.49
578.81	606.67	649.17	742.83	756.59	795.94
839.57	871.87	930.05	968.17	1005.63	1026.58
1049.60	1138.65	1171.55	1190.26	1223.88	1284.44
1298.69	1317.71	1358.97	1370.33	1393.16	1424.97
1439.71	1475.32	1483.61	1662.63	1866.33	1885.93
3026.78	3040.42	3065.78	3109.28	3139.01	3528.30
3601.64	3612.39	3845.71			

- Conformer (18); ZPE = 33580.997 cm^{-1}:

30.53	46.04	68.48	101.71	122.09	209.48
229.58	261.55	299.75	393.43	469.33	511.38
523.64	588.38	633.50	652.09	666.78	765.57
790.60	854.93	903.67	962.48	976.62	1025.71
1098.32	1146.38	1158.14	1177.86	1193.36	1258.09
1272.15	1300.51	1333.37	1356.89	1380.64	1422.14
1434.73	1459.72	1482.71	1669.56	1861.07	1866.58
3037.87	3049.75	3057.97	3092.61	3113.55	3538.42
3620.55	3843.57	3848.17			

- Conformer (19); ZPE = 33801.322 cm^{-1}:

36.37	83.22	96.09	117.10	170.74	230.99
272.83	281.86	356.24	419.75	457.43	521.99
559.44	583.51	625.68	641.24	729.60	760.41
788.23	858.44	899.48	940.90	981.46	1014.58
1079.72	1142.44	1166.43	1181.79	1221.37	1237.17
1271.05	1308.02	1329.70	1366.86	1383.10	1413.93
1433.87	1467.76	1483.07	1680.58	1855.66	1858.18
3060.36	3071.03	3076.56	3121.99	3137.24	3530.84
3613.73	3836.48	3846.14			

- Conformer (20); ZPE = 33640.200 cm^{-1}:

34.33	56.18	61.93	113.94	162.02	206.74
251.66	292.35	354.41	446.22	490.84	505.26
525.24	586.97	640.60	654.21	663.60	761.52
785.36	833.86	868.34	938.14	967.70	1029.25
1097.52	1143.83	1171.63	1181.34	1213.30	1224.87
1279.36	1305.30	1332.54	1345.54	1376.54	1431.29
1446.18	1459.38	1480.31	1636.27	1861.06	1865.80
2966.82	3045.89	3068.65	3086.47	3117.70	3561.58
3656.74	3845.80	3847.99			

- Conformer (21); ZPE = 33876.799 cm^{-1}:

35.87	55.89	88.06	108.12	182.32	219.08
326.18	355.17	384.76	414.00	458.37	535.42
572.96	598.16	614.57	712.15	756.37	820.87
824.56	854.23	877.02	946.40	970.03	1044.56
1055.81	1117.42	1161.29	1203.34	1233.46	1244.69
1282.24	1297.50	1338.85	1375.50	1395.27	1403.25
1432.28	1465.63	1480.22	1665.64	1860.81	1889.58
3031.06	3049.97	3063.11	3118.63	3127.21	3546.88
3639.06	3676.33	3843.48			

- Conformer (22); ZPE = 33696.902 cm^{-1}:

52.33	61.68	92.80	129.45	174.68	248.74
268.68	284.73	317.08	343.07	496.87	510.30
565.50	574.47	604.14	639.40	658.34	765.12
789.70	846.10	882.95	918.92	1002.87	1032.42
1079.57	1142.64	1176.03	1195.23	1207.75	1250.94
1275.26	1317.68	1339.46	1365.06	1391.64	1408.46
1439.85	1447.47	1472.44	1664.73	1847.07	1854.58
3047.26	3056.46	3062.61	3102.32	3114.17	3545.78
3629.64	3847.66	3851.70			

- Conformer (23); ZPE = 33719.540 cm^{-1}:

45.25	57.74	65.92	130.88	205.15	233.47
252.66	284.24	325.12	347.09	472.56	519.99
553.84	605.14	634.36	647.70	664.31	775.99
787.38	844.13	872.15	930.09	976.83	1070.37
1080.40	1145.62	1162.93	1182.00	1216.42	1218.28
1290.29	1306.71	1327.74	1364.18	1368.99	1437.70
1442.76	1460.93	1471.27	1669.73	1862.07	1862.57
3049.81	3057.26	3090.02	3099.41	3119.16	3540.47
3626.92	3835.87	3847.22			

- Conformer (24); ZPE = 33709.690 cm^{-1}:

40.76	48.11	73.45	123.95	175.69	246.53
281.41	294.47	349.27	402.51	486.41	500.50
547.05	598.76	624.41	652.45	677.11	763.44
817.60	827.25	876.55	928.34	963.20	1017.79
1079.53	1158.55	1176.23	1188.43	1213.52	1243.10
1268.97	1314.75	1315.94	1343.97	1387.05	1434.11
1448.17	1459.74	1480.98	1649.28	1852.90	1860.90
3035.36	3045.62	3058.05	3088.22	3107.06	3551.66
3645.18	3847.27	3847.83			

- Conformer (25); ZPE = 33688.143 cm^{-1}:

44.55	69.05	112.61	127.44	167.13	229.21
283.93	302.38	338.23	397.85	484.73	501.38
528.19	572.11	623.76	651.13	678.21	771.70
783.72	836.36	872.21	925.95	963.10	998.47
1092.59	1138.88	1176.03	1192.10	1210.58	1233.30
1256.14	1318.76	1320.59	1348.82	1386.56	1435.50
1446.50	1458.14	1483.24	1638.59	1853.39	1858.77
3049.20	3057.13	3061.78	3097.15	3103.92	3549.86
3646.63	3848.45	3850.28			

- Conformer (26); ZPE = 33939.486 cm^{-1}:

65.20	92.26	118.73	133.32	186.62	233.08
317.28	344.21	378.45	444.24	504.09	549.56
562.66	572.32	599.38	745.31	757.08	769.37
791.71	853.23	864.16	923.04	970.80	993.55
1095.88	1128.72	1150.75	1204.77	1232.00	1241.80
1256.17	1323.18	1339.41	1372.89	1394.27	1403.64
1436.36	1468.78	1479.08	1646.15	1882.14	1898.03
3029.94	3055.33	3058.09	3111.17	3139.64	3554.51
3650.89	3716.89	3838.84			

- Conformer (27); ZPE = 33865.671 cm^{-1}:

59.72	68.11	89.11	119.33	198.35	265.41
283.31	334.99	341.11	357.70	505.47	513.96
568.08	618.60	652.76	666.92	743.67	781.40
798.65	853.98	882.49	916.46	1016.71	1025.53
1082.56	1130.04	1160.27	1202.72	1235.78	1261.03
1287.15	1322.50	1339.25	1361.10	1388.67	1432.39
1443.45	1448.38	1476.47	1656.74	1857.37	1877.93
3028.44	3042.05	3060.23	3093.02	3114.25	3560.05
3646.24	3711.37	3850.07			

- Conformer (28); ZPE = 33657.964 cm^{-1}:

31.92	47.65	51.40	98.15	188.71	240.87
278.09	293.34	312.73	369.03	484.68	515.64
526.22	586.30	623.89	641.72	671.19	767.65
802.06	848.61	871.91	959.35	992.02	1003.56
1077.53	1141.29	1156.15	1179.21	1202.55	1244.19
1291.10	1310.88	1329.82	1360.58	1407.44	1422.86
1428.29	1453.47	1466.87	1657.50	1856.53	1864.37
3049.42	3061.53	3066.49	3101.34	3114.72	3538.14
3631.76	3847.47	3847.74			

- Conformer (29); ZPE = 33883.288 cm^{-1}:

56.28	70.52	103.45	157.17	168.93	253.19
288.01	326.86	351.91	430.65	473.81	548.81
563.11	611.02	662.82	705.57	736.86	780.48
816.94	834.06	899.50	913.44	965.79	1017.38
1050.09	1132.95	1158.81	1178.43	1237.32	1240.60
1282.56	1337.91	1351.00	1368.87	1375.77	1402.68
1417.24	1468.99	1475.08	1654.88	1869.15	1889.25
3037.89	3053.40	3061.93	3112.55	3122.12	3549.06
3634.49	3724.58	3842.41			

- Conformer (30); ZPE = 33694.230 cm^{-1}:

42.93	49.29	81.28	129.32	173.36	237.62
272.69	289.58	322.05	390.07	465.62	510.21
555.27	584.15	592.10	654.61	676.41	751.50
825.19	846.98	887.90	939.18	995.23	1008.22
1064.67	1124.44	1163.28	1200.55	1219.80	1237.94
1287.55	1317.91	1343.71	1364.19	1390.34	1400.07
1421.21	1451.44	1481.47	1666.36	1867.61	1872.12
3034.99	3050.91	3052.78	3107.94	3118.34	3543.28
3627.92	3845.48	3851.37			

- Conformer (31); ZPE = 33892.051 cm^{-1}:

42.56	59.67	79.22	122.20	209.49	244.14
285.67	305.17	380.31	427.52	483.25	511.31
585.58	602.81	644.18	711.46	754.93	832.58
856.70	905.68	928.98	947.30	989.38	1021.77
1068.10	1141.64	1187.72	1198.24	1223.19	1249.16
1306.72	1329.59	1336.17	1363.39	1380.49	1451.04
1461.26	1470.70	1485.38	1634.79	1863.68	1881.41
3012.52	3023.03	3058.87	3115.28	3141.42	3456.87
3536.89	3630.41	3844.24			

- Conformer (32); ZPE = 33676.625 cm^{-1}:

51.58	63.33	75.94	135.08	187.21	209.74
262.28	287.92	319.34	338.95	472.74	514.53
558.61	586.98	622.37	652.84	660.80	756.89
778.99	855.23	897.17	929.72	1010.59	1036.88
1091.25	1143.70	1160.51	1181.80	1203.47	1248.55
1276.21	1310.56	1317.61	1370.66	1380.36	1425.79
1437.01	1448.56	1475.50	1671.24	1859.80	1878.11
3038.66	3055.35	3055.96	3099.38	3115.66	3537.70
3617.31	3838.09	3848.73			

- Conformer (33); ZPE = 33785.477 cm^{-1}:

32.31	53.21	85.40	112.58	133.00	198.89
286.33	328.51	340.24	359.07	481.63	524.85
568.17	637.31	654.12	663.16	748.15	802.36
809.06	848.87	901.56	921.47	1005.24	1051.21
1087.05	1135.62	1157.29	1181.77	1234.37	1256.88
1291.92	1310.54	1337.09	1364.69	1384.30	1424.95
1446.20	1453.48	1486.46	1658.27	1858.98	1887.44
3032.10	3040.17	3059.55	3082.33	3107.23	3558.89
3644.17	3692.89	3849.58			

- Conformer (34); ZPE = 33780.701 cm^{-1}:

36.64	41.00	67.13	111.43	200.89	234.45
290.58	315.25	375.06	405.91	493.71	522.48
543.33	593.14	637.99	673.42	742.44	792.35
799.21	843.05	855.47	947.14	973.47	1011.28
1095.02	1140.84	1159.87	1194.28	1233.20	1237.97
1263.62	1308.20	1328.27	1386.54	1398.21	1428.27
1431.94	1454.32	1468.25	1653.81	1861.05	1891.29
3042.67	3050.19	3067.43	3091.33	3119.56	3544.97
3643.76	3714.60	3845.12			

- Conformer (35); ZPE = 33890.359 cm^{-1}:

51.29	56.01	76.72	141.98	171.47	248.32
276.97	294.69	325.23	409.13	449.22	510.45
593.08	607.44	657.60	713.97	753.05	832.39
872.14	911.24	936.70	955.18	1009.64	1044.99
1065.21	1159.58	1167.49	1197.88	1201.67	1268.10
1314.66	1324.01	1360.33	1364.90	1378.41	1438.40
1464.18	1467.30	1482.32	1666.84	1866.05	1880.60
3033.61	3041.42	3061.24	3109.94	3138.41	3456.32
3520.11	3612.32	3840.52			

- Conformer (36); ZPE = 33680.909 cm^{-1}:

44.41	54.34	61.89	100.36	189.50	272.25
286.85	295.46	327.23	372.14	486.75	523.51
538.71	571.64	606.04	637.80	664.17	753.17
790.55	840.04	895.74	916.53	1000.32	1047.11
1093.13	1147.37	1173.60	1183.09	1218.66	1225.66
1284.95	1314.41	1332.11	1343.04	1405.01	1426.98
1454.77	1455.61	1462.50	1651.05	1851.64	1867.24
2962.37	3061.70	3063.36	3103.43	3118.23	3552.54
3638.48	3847.01	3847.34			

- Conformer (37); ZPE = 33773.957 cm^{-1}:

35.83	57.41	79.24	99.04	152.94	194.85
271.56	301.02	357.78	445.33	464.61	525.52
551.81	644.26	651.66	658.22	763.07	801.04
807.34	840.61	898.45	928.72	973.21	1036.63
1064.44	1138.04	1159.77	1189.65	1206.12	1267.99
1285.94	1307.56	1350.57	1363.03	1374.31	1421.99
1440.13	1457.90	1478.08	1656.63	1864.88	1894.67
3043.01	3050.51	3061.24	3083.57	3117.94	3552.69
3634.95	3694.83	3847.33			

- Conformer (38); ZPE = 33759.618 cm^{-1}:

33.77	53.87	62.20	118.27	149.89	210.72
270.49	296.48	374.47	440.62	494.00	522.10
550.94	574.66	651.11	656.94	758.87	787.16
798.84	842.07	874.02	924.01	970.81	1039.54
1103.91	1141.99	1172.05	1186.19	1229.76	1239.79
1279.94	1306.92	1333.04	1372.82	1397.19	1424.67
1441.39	1452.70	1470.92	1657.46	1865.38	1887.30
3034.65	3045.99	3067.09	3085.46	3120.54	3551.55
3642.14	3705.56	3847.00			

- Conformer (39); ZPE = 33726.704 cm^{-1}:

25.45	53.45	80.17	115.80	187.07	203.37
258.69	312.65	360.52	421.86	445.12	512.42
567.27	583.48	624.86	640.69	735.94	764.75
801.21	850.84	875.45	930.46	987.63	1014.77
1088.53	1146.72	1169.21	1191.99	1218.14	1240.68
1260.32	1318.10	1322.12	1357.12	1375.94	1398.51
1439.36	1469.61	1485.18	1637.86	1865.25	1871.36
2956.79	3055.55	3080.71	3113.91	3143.05	3558.12
3649.31	3837.52	3848.55			

- Conformer (40); ZPE = 33687.553 cm^{-1}:

43.76	50.70	64.17	98.79	197.53	256.55
290.10	297.15	337.65	377.74	490.43	514.89
553.64	574.94	632.01	635.79	671.17	741.00
788.18	838.89	884.02	913.03	992.42	1053.85
1093.30	1156.03	1175.84	1189.40	1217.58	1233.62
1266.53	1314.70	1333.49	1344.54	1397.31	1429.58
1441.53	1457.89	1474.41	1633.29	1853.42	1868.79
2952.84	3062.94	3065.59	3104.87	3116.03	3555.69
3648.29	3843.58	3845.60			

- Conformer (41); ZPE = 33884.559 cm^{-1}:

32.74	57.27	84.82	122.99	178.46	209.78
319.57	351.66	382.51	414.83	444.05	527.96
578.36	600.17	640.13	740.47	761.49	793.31
800.92	853.86	880.03	949.73	983.60	1003.90
1084.13	1133.12	1157.09	1187.69	1233.03	1255.48
1260.57	1316.80	1323.75	1372.79	1394.88	1398.10
1429.83	1473.77	1481.75	1658.24	1879.40	1892.81
3032.70	3050.85	3073.01	3116.11	3137.70	3539.52
3631.82	3706.82	3834.75			

- Conformer (42); ZPE = 33751.494 cm^{-1}:

64.29	77.39	82.07	112.71	170.79	238.07
270.32	278.59	345.50	411.62	456.90	527.10
557.17	577.90	615.44	649.29	708.31	752.23
784.82	853.61	894.70	930.67	980.54	1006.04
1076.27	1145.14	1164.23	1167.33	1228.59	1240.71
1272.57	1306.97	1342.50	1363.11	1377.74	1402.46
1427.01	1465.89	1478.61	1682.83	1857.70	1871.98
3055.59	3066.78	3073.31	3120.27	3141.45	3524.57
3613.92	3839.78	3849.61			

- Conformer (43); ZPE = 33660.753 cm^{-1}:

29.76	40.28	50.82	99.45	183.62	200.35
247.14	288.64	329.89	388.42	486.02	523.74
551.99	600.08	603.16	656.02	688.13	752.46
804.56	852.81	876.68	950.43	988.52	999.15
1075.91	1138.55	1166.08	1183.27	1223.71	1240.14
1280.76	1316.63	1344.01	1366.75	1385.99	1395.22
1430.78	1447.16	1476.57	1670.35	1862.48	1869.23
3038.60	3058.00	3060.66	3110.93	3133.30	3540.71
3626.41	3841.56	3845.60			

- Conformer (44); ZPE = 33745.106 cm^{-1}:

36.57	47.70	65.80	127.79	136.01	220.06
286.80	308.25	352.87	377.60	503.72	514.59
560.45	578.29	644.79	652.70	736.39	789.52
802.97	856.06	894.76	915.27	1023.39	1077.81
1093.14	1139.25	1163.02	1191.25	1207.05	1243.65
1283.46	1299.98	1337.56	1367.65	1398.55	1430.09
1440.01	1458.82	1476.06	1658.75	1859.53	1886.44
3025.95	3042.19	3051.99	3087.36	3115.49	3541.70
3635.16	3696.43	3849.54			

- Conformer (45); ZPE = 33661.569 cm^{-1}:

38.94	46.50	57.60	114.44	187.25	251.59
265.06	280.18	305.97	363.13	482.29	526.05
543.13	589.57	617.51	647.54	669.57	772.88
798.63	839.56	859.89	927.42	978.18	1061.04
1081.93	1141.15	1155.44	1174.79	1209.51	1217.58
1291.08	1310.34	1326.30	1368.19	1416.06	1417.22
1434.41	1460.06	1464.14	1654.10	1851.77	1861.06
3050.67	3061.09	3074.58	3097.99	3115.61	3538.69
3632.56	3845.33	3847.52			

- Conformer (46); ZPE = 33830.008 cm^{-1}:

28.30	44.53	83.68	122.45	181.69	201.39
308.07	346.89	367.03	414.99	452.96	495.02
578.65	583.85	624.42	642.98	745.29	764.30
802.98	862.19	927.24	931.06	988.35	1020.68
1084.25	1147.92	1181.81	1189.70	1215.63	1250.42
1265.76	1320.11	1330.90	1353.09	1375.87	1417.29
1449.32	1471.24	1487.61	1667.21	1844.50	1868.77
2946.20	3063.53	3081.64	3115.17	3142.87	3547.52
3632.46	3840.78	3849.48			

- Conformer (47); ZPE = 33653.863 cm^{-1}:

23.59	47.78	50.11	99.78	177.78	236.87
276.52	285.78	305.78	369.08	496.41	514.91
542.37	591.02	627.15	638.70	667.69	786.43
806.03	845.34	865.66	937.16	983.91	998.34
1072.75	1143.82	1150.12	1183.87	1208.97	1240.13
1297.78	1311.80	1332.04	1374.21	1388.61	1400.82
1427.31	1451.97	1466.06	1658.02	1859.21	1875.38
3046.32	3062.37	3093.60	3104.53	3108.21	3544.34
3634.19	3847.48	3849.67			

- Conformer (48); ZPE = 33820.238 cm^{-1}:

55.74	73.73	99.83	121.89	174.97	221.17
264.72	288.88	366.82	436.60	483.73	495.84
570.31	575.84	601.81	660.92	740.85	761.78
785.49	863.47	900.75	934.89	973.87	1016.57
1080.26	1125.68	1175.18	1180.94	1216.67	1253.60
1265.38	1317.90	1335.80	1377.14	1380.29	1389.42
1416.55	1473.22	1483.16	1677.60	1862.72	1878.36
3022.72	3069.61	3071.89	3122.32	3141.44	3542.76
3620.27	3842.67	3846.46			

- Conformer (49); ZPE = 33989.714 cm^{-1}:

71.77	88.51	121.53	134.98	204.14	234.10
302.93	325.77	409.43	447.76	488.83	519.39
578.63	599.35	637.72	748.26	754.83	787.57
856.81	870.03	905.31	937.77	988.17	1007.16
1065.42	1130.61	1169.49	1206.12	1232.89	1240.10
1291.34	1333.18	1363.00	1371.75	1380.19	1419.46
1446.52	1475.32	1484.34	1627.44	1865.42	1888.46
2969.31	3053.33	3060.45	3114.41	3136.03	3541.15
3619.00	3633.65	3840.31			

- Conformer (50); ZPE = 33697.043 cm^{-1}:

45.88	53.75	96.47	114.61	170.96	248.25
263.48	319.69	385.35	430.60	498.70	507.04
552.77	575.02	594.34	646.51	680.31	746.14
805.46	826.78	872.95	920.61	966.95	1005.71
1066.87	1136.64	1173.12	1191.66	1212.87	1242.28
1250.63	1330.54	1349.47	1352.32	1386.09	1401.28
1444.76	1454.02	1485.52	1630.09	1865.70	1874.58
2945.12	3052.60	3068.76	3108.45	3121.64	3563.48
3660.97	3845.31	3850.95			

- Conformer (51); ZPE = 33618.476 cm^{-1}:

28.79	47.83	61.86	119.90	160.99	208.84
270.04	285.74	337.13	416.82	473.08	523.97
549.34	558.00	620.30	656.70	666.25	754.69
784.31	831.42	888.15	942.03	970.07	1024.25
1091.19	1143.61	1172.42	1179.25	1208.00	1227.59
1285.17	1306.82	1339.48	1348.92	1378.45	1434.20
1448.22	1461.14	1475.32	1650.50	1857.83	1869.30
2944.53	3054.74	3064.01	3097.78	3116.32	3558.82
3646.93	3846.53	3849.35			

- Conformer (52); ZPE = 33633.959 cm^{-1}:

26.01	46.98	64.89	99.38	120.55	205.84
239.83	258.96	305.38	393.50	444.02	511.79
571.78	598.50	618.47	662.63	703.10	773.81
785.34	855.19	900.73	964.77	971.85	1026.13
1084.56	1123.49	1163.97	1192.52	1225.03	1250.55
1272.26	1309.63	1337.24	1365.59	1384.66	1396.98
1425.27	1464.32	1484.77	1669.13	1865.14	1867.35
3040.82	3051.40	3061.59	3099.96	3126.27	3540.62
3623.11	3843.21	3849.09			

- Conformer (53); ZPE = 33710.487 cm^{-1}:

33.36	53.81	64.64	121.58	159.13	232.05
260.21	292.78	353.27	418.96	451.08	518.86
567.76	591.99	595.76	655.28	710.02	760.15
819.40	841.70	885.70	924.30	968.71	1034.71
1051.51	1145.94	1166.02	1174.90	1209.57	1242.95
1284.33	1292.38	1343.80	1360.96	1382.55	1405.49
1430.55	1462.99	1477.15	1679.44	1860.99	1867.42
3053.64	3063.25	3072.54	3107.08	3123.87	3528.31
3620.35	3842.99	3854.82			

- Conformer (54); ZPE = 33661.267 cm^{-1}:

19.20	32.86	56.21	127.84	194.21	230.75
269.54	281.22	317.12	345.73	493.50	511.67
564.13	605.97	612.80	645.98	655.09	778.37
785.20	839.17	856.08	914.44	975.45	1049.95
1079.54	1140.77	1173.63	1185.92	1213.04	1227.27
1292.02	1317.34	1338.04	1359.11	1388.65	1409.80
1439.55	1455.03	1470.10	1664.00	1854.45	1862.41
3040.28	3059.07	3094.88	3100.47	3113.68	3549.38
3635.55	3847.00	3849.09			

- Conformer (55); ZPE = 33665.776 cm^{-1}:

29.52	53.63	55.94	111.86	171.81	202.11
216.82	294.17	351.92	438.84	479.97	504.18
575.34	586.28	627.54	650.75	709.69	760.21
791.12	825.05	863.62	928.82	970.49	1025.08
1085.01	1131.76	1177.32	1198.62	1222.17	1239.57
1277.50	1306.99	1334.74	1350.70	1386.68	1398.37
1445.26	1463.57	1484.34	1636.36	1865.10	1867.80
2970.80	3051.32	3065.11	3106.54	3124.37	3562.28
3658.51	3847.24	3848.78			

- Conformer (56); ZPE = 33733.475 cm^{-1}:

44.49	59.40	98.36	126.38	181.44	257.09
293.79	327.01	365.09	418.09	466.33	540.58
555.81	567.65	599.93	642.95	682.46	749.46
808.94	824.89	893.41	917.13	957.24	1007.25
1065.01	1139.89	1173.78	1194.01	1201.90	1239.91
1265.10	1330.39	1345.50	1362.89	1386.84	1400.85
1449.50	1455.65	1470.35	1643.47	1868.48	1873.86
2942.00	3053.73	3068.84	3117.89	3127.09	3558.60
3650.30	3847.02	3848.89			

- Conformer (57); ZPE = 33693.297 cm^{-1}:

24.48	53.06	57.25	131.52	195.78	246.23
257.74	302.61	316.38	339.97	477.24	526.79
547.57	615.92	633.45	652.66	671.83	780.52
787.83	834.09	865.96	901.80	999.44	1046.44
1099.68	1147.76	1164.38	1170.19	1213.82	1227.33
1267.72	1313.86	1336.01	1347.85	1368.78	1435.26
1456.70	1460.63	1480.55	1638.19	1861.16	1869.88
3042.64	3057.18	3085.87	3098.61	3108.57	3542.82
3638.76	3840.05	3845.76			

- Conformer (58); ZPE = 33839.927 cm^{-1}:

55.08	73.43	112.22	131.45	182.74	228.70
282.27	293.96	358.07	428.98	467.21	536.65
561.11	567.50	600.33	646.83	743.72	759.56
787.02	859.15	892.56	919.22	967.63	1013.45
1085.14	1127.20	1171.07	1193.12	1218.33	1254.10
1263.73	1321.48	1338.20	1380.05	1386.55	1393.78
1414.34	1467.50	1480.31	1673.59	1857.38	1885.03
3023.70	3066.29	3070.70	3121.79	3142.16	3552.12
3633.60	3840.53	3849.23			

- Conformer (59); ZPE = 33670.907 cm^{-1}:

52.69	68.96	96.39	130.57	171.64	245.46
261.85	301.75	315.15	339.19	502.11	510.04
553.92	565.45	599.24	637.87	661.36	765.93
794.08	845.44	858.12	913.19	992.52	1069.60
1079.61	1120.21	1171.11	1184.53	1215.55	1245.32
1256.98	1319.49	1337.29	1366.88	1378.79	1438.27
1441.41	1449.09	1472.00	1652.12	1853.63	1859.55
3044.75	3054.86	3060.96	3097.90	3102.50	3547.45
3636.60	3850.59	3851.84			

- Conformer (60); ZPE = 33905.072 cm^{-1}:

36.09	60.08	67.20	109.14	176.71	192.52
288.47	329.34	357.26	410.15	444.54	555.25
581.37	635.14	660.17	740.80	755.75	781.89
810.27	867.94	907.40	951.58	987.97	1020.71
1061.73	1116.63	1176.08	1192.67	1242.21	1252.33
1289.63	1315.11	1333.56	1372.32	1386.72	1418.59
1423.50	1469.58	1489.53	1676.90	1854.60	1897.07
3015.65	3053.46	3081.30	3112.22	3137.86	3544.48
3614.84	3713.20	3840.61			

- Conformer (61); ZPE = 33939.472 cm^{-1}:

42.22	85.05	132.03	163.05	178.58	241.15
264.01	316.53	322.67	378.23	459.01	531.39
592.13	648.32	662.09	741.25	778.41	795.77
808.56	841.67	880.84	904.60	1010.13	1037.46
1090.63	1149.72	1173.90	1199.73	1223.54	1254.66
1275.69	1331.90	1342.32	1365.73	1391.23	1431.07
1456.03	1475.17	1491.34	1636.04	1821.80	1883.61
3036.47	3041.64	3070.76	3093.86	3136.39	3541.06
3636.78	3681.99	3830.73			

- Conformer (62); ZPE = 33592.391 cm^{-1}:

24.23	47.89	70.70	92.58	156.03	194.00
232.28	288.31	335.44	436.33	457.41	519.62
530.32	597.59	643.56	654.71	665.55	768.94
786.51	806.37	860.88	934.49	955.51	1034.32
1095.42	1150.46	1172.10	1175.06	1208.67	1249.82
1266.17	1310.66	1318.44	1341.81	1378.45	1433.48
1449.10	1460.60	1485.13	1638.86	1863.29	1865.54
3038.74	3042.21	3060.08	3079.55	3108.20	3553.97
3650.69	3846.96	3847.77			

- Conformer (63); ZPE = 33657.938 cm^{-1}:

48.26	67.18	79.59	135.15	181.74	217.09
265.93	308.46	319.56	336.85	481.05	514.99
555.04	584.62	619.94	656.88	662.13	758.87
780.53	843.34	865.19	912.09	998.58	1074.44
1078.80	1162.55	1164.72	1175.37	1217.92	1244.26
1248.76	1318.41	1333.56	1341.45	1371.42	1435.70
1448.65	1456.43	1474.22	1642.44	1860.35	1880.67
3019.00	3045.80	3054.16	3095.18	3100.25	3546.23
3641.44	3842.56	3848.07			

- Conformer (64); ZPE = 33599.213 cm^{-1}:

21.43	34.77	66.91	93.33	125.11	213.88
257.05	275.73	290.44	386.35	466.00	526.69
547.29	603.84	632.58	652.51	658.91	787.28
790.21	834.85	896.02	952.71	961.34	1020.68
1093.43	1144.88	1159.32	1178.95	1201.46	1261.54
1271.66	1311.32	1325.95	1370.37	1385.08	1394.07
1434.94	1459.36	1482.58	1662.77	1861.92	1868.73
3042.39	3054.88	3068.13	3088.40	3106.60	3546.71
3633.30	3845.99	3847.79			

- Conformer (65); ZPE = 33693.741 cm^{-1}:

61.00	71.85	81.46	129.26	182.65	199.85
259.99	286.69	329.51	349.25	480.98	522.35
578.63	588.00	600.92	646.71	697.88	756.71
777.41	847.24	887.12	920.48	997.95	1031.42
1089.96	1137.66	1161.14	1186.82	1213.50	1247.94
1278.82	1307.16	1336.20	1372.39	1383.07	1392.60
1428.32	1446.36	1482.94	1669.88	1866.29	1872.94
3044.29	3056.84	3061.12	3107.24	3113.89	3538.17
3618.96	3839.68	3847.98			

- Conformer (66); ZPE = 33625.371 cm^{-1}:

3.00	44.88	70.79	105.05	111.62	223.06
239.90	259.04	304.16	392.36	458.46	511.09
558.42	601.84	622.46	664.21	693.25	762.72
795.33	855.70	893.71	954.10	976.46	1027.29
1091.31	1142.95	1163.40	1185.86	1219.01	1254.74
1276.99	1304.89	1336.56	1365.77	1384.49	1399.52
1423.94	1461.75	1483.52	1671.36	1865.52	1869.41
3029.67	3042.86	3050.59	3110.82	3135.01	3541.47
3622.99	3842.94	3844.48			

- Conformer (67); ZPE = 33588.543 cm^{-1}:

28.64	38.86	80.64	98.42	131.15	192.72
233.17	281.12	330.90	334.60	448.50	516.83
550.74	615.37	640.26	648.96	653.52	760.33
802.27	841.43	906.46	912.19	1018.21	1055.05
1091.56	1145.11	1165.42	1173.14	1188.49	1241.36
1289.53	1295.27	1321.02	1360.33	1378.28	1425.84
1437.69	1451.69	1481.72	1668.45	1864.65	1868.64
3043.63	3052.69	3062.15	3088.22	3116.90	3539.56
3623.71	3834.86	3846.81			

- Conformer (68); ZPE = 33701.773 cm^{-1}:

24.80	48.87	69.35	100.87	117.02	246.73
249.21	284.94	363.71	423.57	472.67	523.64
548.55	595.13	652.05	663.38	749.41	783.97
810.18	845.18	879.19	934.22	977.86	1052.84
1097.59	1138.42	1157.20	1179.42	1234.91	1251.99
1260.29	1304.00	1318.46	1370.69	1390.47	1427.49
1435.65	1456.06	1485.00	1654.42	1868.83	1892.91
3029.72	3035.99	3049.72	3082.62	3117.13	3551.34
3650.20	3698.19	3847.48			

- Conformer (69); ZPE = 33700.810 cm^{-1}:

14.28	47.32	83.34	132.01	179.63	239.11
290.99	324.54	342.66	391.62	472.10	496.04
557.04	581.82	600.62	637.42	685.35	749.45
817.18	838.36	878.22	938.10	987.54	1011.22
1060.91	1121.82	1176.87	1188.53	1220.00	1246.04
1295.78	1312.50	1340.91	1371.72	1392.63	1399.01
1415.00	1451.54	1480.44	1658.79	1862.26	1873.44
3008.23	3052.46	3062.06	3112.25	3119.93	3546.33
3640.69	3846.17	3849.36			

- Conformer (70); ZPE = 33707.325 cm^{-1}:

30.82	47.47	67.18	104.63	183.07	272.16
287.18	339.79	347.06	417.76	484.75	519.15
526.61	562.18	615.81	631.46	662.45	744.16
804.36	850.48	916.03	931.43	980.95	1063.60
1070.34	1150.04	1182.97	1189.62	1210.34	1249.19
1275.33	1319.85	1326.83	1338.32	1393.84	1430.44
1452.49	1456.91	1475.32	1648.71	1847.92	1865.48
2916.01	3051.82	3071.95	3094.32	3126.84	3548.62
3634.89	3845.03	3850.65			

- Conformer (71); ZPE = 33949.494 cm^{-1}:

29.56	60.42	112.27	157.59	180.17	218.44
303.64	345.03	372.50	401.37	472.76	501.65
586.20	606.14	634.32	745.27	758.37	795.47
831.37	866.65	921.91	964.04	1002.78	1014.38
1052.21	1133.94	1173.95	1183.67	1210.96	1284.12
1296.69	1320.72	1354.78	1367.15	1404.64	1414.70
1433.04	1475.81	1483.75	1655.10	1874.24	1887.46
3018.93	3052.89	3059.05	3111.97	3137.16	3532.82
3618.19	3634.42	3844.33			

- Conformer (72); ZPE = 33778.601 cm^{-1}:

33.13	51.62	59.29	134.81	170.48	237.97
277.85	346.71	358.18	420.74	443.47	545.33
553.21	575.48	589.30	649.95	723.86	752.51
820.47	845.90	885.45	921.69	966.58	1035.62
1049.61	1145.28	1170.07	1188.33	1204.81	1244.05
1283.22	1315.08	1343.70	1375.56	1391.59	1408.20
1412.32	1466.60	1474.33	1680.32	1851.61	1857.79
3030.27	3067.24	3073.68	3123.44	3132.07	3541.18
3629.72	3845.68	3851.83			

- Conformer (73); ZPE = 33710.846 cm^{-1}:

40.55	51.99	76.48	128.11	193.76	223.02
250.64	284.36	322.67	352.02	479.91	523.37
573.88	592.95	616.77	647.19	685.59	778.69
788.14	837.20	869.38	928.31	963.82	1067.91
1081.08	1151.01	1165.95	1177.35	1217.36	1231.24
1291.07	1306.07	1347.62	1364.53	1372.89	1402.12
1441.51	1462.07	1473.32	1668.47	1865.34	1868.69
3047.77	3056.93	3087.06	3102.62	3119.37	3538.51
3624.34	3837.09	3843.57			

- Conformer (74); ZPE = 33610.253 cm^{-1}:

36.55	46.09	60.41	122.62	136.74	209.03
276.41	278.81	301.15	357.45	477.01	517.23
547.99	588.30	618.77	646.87	649.55	769.25
786.67	836.21	899.51	910.10	1021.69	1056.97
1087.14	1144.60	1154.06	1171.26	1195.76	1239.62
1289.16	1304.21	1320.60	1361.01	1400.19	1420.02
1439.15	1455.69	1476.99	1656.58	1861.60	1866.87
3037.21	3045.27	3061.72	3091.29	3116.33	3542.64
3634.60	3845.92	3849.63			

- Conformer (75); ZPE = 33637.335 cm^{-1}:

33.92	49.26	58.10	114.99	182.71	235.48
280.00	299.68	308.58	339.39	491.91	518.15
559.60	600.65	607.70	637.99	662.39	779.72
789.86	824.33	855.45	907.20	986.96	1057.92
1084.32	1122.09	1162.48	1180.61	1213.64	1234.43
1266.26	1316.89	1341.26	1366.46	1375.05	1432.72
1445.98	1453.61	1468.61	1646.60	1855.24	1866.49
3040.07	3057.12	3084.96	3096.08	3101.85	3545.64
3636.69	3847.85	3849.71			

- Conformer (76); ZPE = 33677.635 cm^{-1}:

22.52	43.24	54.40	93.73	154.40	202.44
219.36	263.51	333.77	425.10	437.38	519.12
577.79	597.69	630.39	659.68	712.54	772.92
790.26	845.37	904.10	923.60	953.48	1042.03
1075.83	1159.29	1160.09	1176.98	1236.49	1239.56
1283.21	1304.18	1324.39	1365.07	1388.03	1396.71
1435.55	1464.60	1480.44	1674.81	1860.57	1870.52
3034.10	3063.24	3073.05	3115.89	3136.49	3544.15
3624.24	3840.83	3848.13			

- Conformer (77); ZPE = 33823.836 cm^{-1}:

35.64	68.54	82.57	127.20	200.46	247.48
288.72	306.95	355.01	428.16	483.00	511.48
550.58	556.81	628.97	643.98	694.13	747.49
784.58	864.96	886.64	945.56	962.32	1061.53
1074.49	1169.59	1177.34	1199.81	1211.49	1249.44
1301.00	1314.76	1341.08	1366.92	1394.70	1408.77
1436.37	1458.05	1482.05	1663.26	1836.31	1850.70
3018.81	3064.98	3068.01	3105.59	3117.06	3548.56
3631.09	3844.80	3849.88			

- Conformer (78); ZPE = 33753.988 cm^{-1}:

62.62	70.55	84.78	130.20	165.14	244.12
261.77	298.68	318.00	381.66	446.85	507.16
576.77	593.35	597.06	655.81	722.75	763.39
804.37	851.36	886.87	935.42	994.66	1039.88
1072.59	1120.77	1165.19	1186.62	1224.12	1237.52
1281.24	1311.54	1328.42	1371.68	1380.57	1397.00
1443.27	1461.99	1474.42	1665.48	1855.80	1859.41
3056.93	3064.20	3074.69	3118.93	3126.57	3536.21
3624.27	3825.20	3850.13			

- Conformer (79); ZPE = 33622.848 cm^{-1}:

15.24	52.55	60.13	113.95	166.60	204.28
224.79	292.95	351.93	450.85	477.52	505.12
550.53	581.54	622.72	645.63	684.44	762.27
782.06	831.56	860.12	918.72	966.75	1033.09
1093.69	1142.31	1177.63	1202.24	1213.91	1234.43
1281.67	1307.81	1336.37	1346.41	1385.70	1402.19
1444.99	1459.94	1480.64	1638.70	1864.31	1868.62
2967.37	3037.13	3057.99	3109.56	3125.31	3561.82
3656.38	3845.96	3847.29			

- Conformer (80); ZPE = 33794.686 cm^{-1}:

59.00	79.65	108.95	134.26	171.86	199.31
266.30	304.28	338.21	401.44	502.95	530.23
535.97	631.45	650.89	685.72	736.63	759.78
815.63	823.53	882.06	919.71	978.30	1032.07
1085.04	1157.20	1203.05	1210.26	1224.32	1256.13
1277.14	1323.20	1336.62	1382.14	1393.49	1439.53
1441.14	1455.86	1480.38	1636.07	1827.95	1890.97
2993.25	3045.52	3052.43	3093.92	3105.22	3540.23
3641.93	3710.83	3837.37			

- Conformer (81); ZPE = 33860.469 cm^{-1}:

56.48	75.88	94.40	135.08	182.89	259.32
280.62	336.08	346.17	357.02	509.92	518.21
581.88	587.72	651.65	670.79	746.20	787.63
797.10	848.19	878.60	909.69	1002.52	1023.87
1083.91	1128.12	1152.84	1214.50	1241.68	1254.58
1289.31	1332.63	1351.25	1364.01	1391.93	1399.48
1430.69	1444.94	1485.83	1657.32	1875.72	1887.49
3028.37	3040.22	3063.61	3091.62	3115.30	3558.10
3643.38	3707.55	3848.64			

- Conformer (82); ZPE = 33671.559 cm^{-1}:

32.29	41.63	60.79	120.96	143.93	215.18
267.08	292.91	337.52	392.69	468.41	530.27
558.29	564.84	627.25	659.91	671.25	760.40
778.68	837.54	904.76	921.29	959.76	1056.47
1079.70	1158.33	1168.62	1185.17	1195.29	1238.47
1291.93	1312.84	1326.86	1374.16	1392.92	1404.96
1435.19	1455.22	1470.23	1667.89	1858.61	1866.89
3034.78	3044.37	3078.62	3087.91	3128.87	3550.86
3634.57	3847.49	3848.26			

- Conformer (83); ZPE = 33692.246 cm^{-1}:

42.12	56.17	79.87	125.98	182.44	211.14
236.88	303.61	371.97	429.97	480.64	505.17
566.80	576.60	594.64	630.05	729.42	757.32
801.09	844.25	876.76	930.49	955.33	1008.82
1086.30	1124.43	1164.53	1190.03	1214.65	1240.82
1248.57	1328.00	1335.13	1345.69	1379.43	1399.19
1445.57	1473.93	1483.38	1638.96	1860.50	1874.78
2967.69	3061.55	3069.49	3112.09	3130.66	3561.06
3656.29	3841.49	3852.74			

- Conformer (84); ZPE = 33569.003 cm^{-1}:

25.93	36.23	64.44	110.49	122.71	197.89
233.42	278.74	326.24	337.25	466.32	521.88
562.52	599.62	617.59	644.66	653.83	761.72
801.78	832.49	890.87	909.59	1009.53	1036.95
1088.76	1144.06	1161.87	1182.31	1205.40	1240.51
1290.28	1308.45	1336.63	1354.94	1387.06	1395.43
1439.90	1454.56	1485.78	1661.68	1857.09	1864.87
3044.29	3054.14	3067.92	3087.13	3111.48	3545.85
3631.43	3846.23	3847.23			

- Conformer (85); ZPE = 33752.285 cm^{-1}:

44.59	67.32	83.09	129.83	161.24	196.92
255.06	289.39	311.87	420.50	454.35	501.29
573.02	584.64	605.73	632.16	763.31	778.51
824.90	846.46	886.38	922.00	1018.23	1027.32
1065.53	1121.48	1146.24	1184.04	1230.02	1237.02
1267.63	1309.84	1336.85	1360.86	1397.55	1426.77
1434.97	1470.46	1482.90	1654.22	1847.92	1855.68
3052.44	3065.40	3073.98	3113.97	3136.48	3533.85
3624.15	3847.38	3848.80			

- Conformer (86); ZPE = 33814.471 cm^{-1}:

20.45	49.13	63.19	103.58	197.46	279.83
306.14	309.90	369.95	378.41	509.08	539.59
569.21	575.28	602.43	690.73	742.92	784.90
801.48	841.59	871.00	937.40	961.17	1064.70
1094.41	1151.36	1177.04	1182.04	1228.49	1238.33
1265.05	1323.68	1354.44	1372.09	1390.43	1406.61
1434.43	1453.07	1472.94	1657.24	1871.51	1887.02
3036.99	3051.08	3065.15	3099.00	3123.10	3539.39
3632.95	3711.28	3840.31			

- Conformer (87); ZPE = 34024.747 cm^{-1}:

67.43	96.22	114.69	127.78	205.09	228.67
303.33	328.22	377.44	432.86	481.41	563.03
568.25	623.50	706.68	760.88	776.52	786.33
819.29	866.01	878.52	938.22	964.91	1009.57
1103.47	1126.56	1177.24	1205.80	1255.19	1276.69
1298.18	1308.28	1330.57	1386.25	1396.65	1402.84
1443.89	1475.04	1485.06	1661.48	1858.63	1888.95
3020.97	3038.86	3066.08	3108.06	3135.97	3541.72
3637.91	3690.77	3703.53			

- Conformer (88); ZPE = 33800.934 cm^{-1}:

19.67	44.53	68.34	146.59	188.32	235.65
263.70	271.44	322.61	384.11	482.34	499.38
570.98	596.54	622.44	654.61	759.36	775.40
795.23	845.74	869.35	929.06	992.69	1061.95
1078.09	1146.18	1162.78	1175.59	1197.16	1247.89
1289.41	1315.17	1337.96	1353.45	1406.02	1426.27
1439.04	1470.54	1481.65	1655.46	1844.33	1856.32
3048.48	3073.90	3093.21	3107.20	3138.67	3538.69
3630.81	3838.74	3848.82			

- Conformer (89); ZPE = 33821.706 cm^{-1}:

23.74	49.06	59.22	117.63	163.92	203.53
286.52	297.41	375.77	431.84	479.94	548.23
566.84	583.28	636.07	708.61	762.45	786.18
799.59	846.00	878.03	919.52	970.55	1032.55
1095.37	1137.40	1164.81	1215.63	1236.17	1244.72
1281.45	1305.37	1335.96	1390.92	1394.55	1403.31
1433.04	1458.88	1472.83	1660.37	1865.97	1887.11
3042.73	3049.16	3057.04	3106.76	3125.07	3553.34
3644.23	3704.16	3850.59			

- Conformer (90); ZPE = 33699.505 cm^{-1}:

41.77	51.51	78.30	135.18	175.63	213.33
259.62	281.66	329.46	391.82	485.38	508.15
555.45	580.71	623.43	643.70	681.83	775.86
785.77	842.15	883.82	923.73	973.20	996.38
1078.92	1144.42	1159.69	1189.66	1197.12	1253.08
1258.42	1318.25	1337.78	1375.97	1393.00	1400.98
1436.52	1450.96	1461.05	1669.04	1861.11	1881.13
3057.51	3063.73	3090.92	3102.62	3119.40	3552.65
3632.39	3846.01	3848.87			

- Conformer (91); ZPE = 33696.995 cm^{-1}:

37.00	56.05	69.82	121.32	165.66	258.28
289.51	312.17	351.42	413.41	466.40	528.09
560.65	583.74	608.17	664.61	689.03	760.84
801.38	821.39	860.80	915.00	959.31	1009.87
1068.53	1141.18	1168.19	1172.86	1227.42	1234.73
1266.48	1313.58	1329.09	1352.00	1385.59	1395.66
1451.85	1458.91	1484.83	1637.77	1866.69	1873.19
3034.69	3053.44	3060.55	3104.41	3113.64	3550.27
3652.11	3845.52	3846.89			

- Conformer (92); ZPE = 33961.215 cm^{-1}:

64.15	86.70	125.11	140.11	216.28	239.00
300.45	323.54	396.99	446.83	482.82	535.77
563.60	596.36	619.85	742.34	749.27	783.82
857.32	875.15	900.15	934.70	986.22	1009.79
1065.29	1131.94	1166.84	1184.29	1229.71	1243.68
1293.11	1335.95	1348.84	1370.71	1383.98	1426.19
1441.79	1472.36	1478.19	1640.01	1877.98	1895.39
2976.77	3051.80	3056.57	3112.93	3135.20	3546.29
3601.74	3632.74	3845.82			

- Conformer (93); ZPE = 33833.581 cm^{-1}:

20.52	55.96	73.46	95.79	164.27	193.22
283.04	301.88	359.37	436.12	453.14	549.52
575.91	626.61	651.09	714.57	769.33	797.50
807.02	845.96	894.73	923.80	969.12	1044.08
1050.43	1124.81	1161.31	1197.05	1236.18	1267.15
1296.87	1303.05	1353.88	1365.15	1386.93	1402.53
1428.12	1466.05	1482.10	1658.13	1868.76	1894.36
3034.00	3047.47	3053.47	3113.68	3131.21	3556.67
3640.89	3689.28	3851.65			

- Conformer (94); ZPE = 33918.225 cm^{-1}:

49.90	74.41	113.81	164.08	192.99	229.41
258.49	285.37	313.06	406.70	471.56	549.64
603.66	613.11	647.07	740.24	774.30	777.23
804.02	841.49	866.95	908.88	981.89	1047.94
1061.25	1151.76	1167.23	1190.73	1220.76	1273.89
1298.53	1334.12	1348.09	1360.71	1402.93	1413.58
1415.23	1480.64	1485.96	1666.34	1820.66	1886.00
3033.74	3058.11	3069.94	3105.82	3135.05	3553.07
3640.20	3712.87	3833.06			

- Conformer (95); ZPE = 33737.232 cm^{-1}:

31.12	46.68	80.47	116.97	178.65	203.76
277.25	321.70	369.22	411.95	450.35	490.97
576.38	579.55	617.81	634.98	746.56	758.01
800.38	852.42	899.61	927.48	983.38	1018.71
1083.85	1138.10	1175.58	1183.32	1207.70	1245.96
1267.22	1314.39	1323.14	1351.89	1378.80	1397.12
1446.25	1470.24	1487.45	1664.59	1871.71	1875.63
2933.18	3063.74	3080.19	3117.18	3142.32	3555.15
3635.36	3840.13	3849.90			

- Conformer (96); ZPE = 33867.547 cm^{-1}:

48.64	91.36	99.17	131.22	174.56	257.08
269.37	315.43	349.34	415.16	476.05	551.65
565.03	591.81	648.75	700.17	747.37	791.34
802.99	846.74	896.07	905.41	984.11	999.94
1062.45	1126.20	1160.48	1190.60	1233.14	1243.55
1279.25	1345.97	1352.14	1357.99	1388.75	1412.12
1417.93	1463.65	1477.25	1659.69	1864.22	1894.49
3034.95	3053.39	3066.61	3107.44	3130.00	3557.93
3641.83	3709.63	3844.66			

- Conformer (97); ZPE = 33631.738 cm^{-1}:

22.94	49.30	51.19	110.16	170.15	233.86
262.84	268.22	315.77	360.38	474.46	525.44
542.56	601.90	622.97	650.27	668.52	784.33
798.09	824.71	848.72	924.76	979.80	1060.92
1112.81	1129.19	1162.18	1176.83	1208.62	1219.12
1273.95	1311.79	1331.04	1358.79	1396.69	1430.90
1452.78	1459.52	1464.56	1650.46	1854.30	1860.14
3052.57	3053.35	3084.26	3091.25	3109.53	3541.66
3637.60	3841.99	3845.37			

- Conformer (98); ZPE = 33691.230 cm^{-1}:

36.59	38.20	60.95	121.74	177.05	248.63
278.95	299.93	345.99	400.33	494.20	509.07
558.22	576.54	623.24	650.99	672.29	761.31
816.20	828.66	870.18	931.66	962.50	1017.82
1077.80	1130.08	1165.26	1196.10	1209.41	1245.85
1266.92	1314.35	1317.45	1361.51	1387.17	1430.42
1442.70	1451.48	1472.52	1659.53	1856.02	1868.22
3050.33	3053.30	3062.52	3090.70	3111.12	3552.54
3639.12	3843.53	3845.26			

- Conformer (99); ZPE = 33581.834 cm^{-1}:

28.96	38.53	79.88	97.92	141.66	193.51
245.40	300.83	322.14	331.49	455.81	513.48
550.51	599.00	639.25	644.66	652.07	761.93
801.45	845.26	874.73	908.51	1025.73	1082.28
1086.04	1148.41	1170.35	1178.15	1181.06	1238.06
1278.46	1302.75	1335.71	1342.38	1368.70	1435.51
1448.05	1464.41	1483.28	1641.79	1864.31	1869.99
3038.58	3045.38	3052.36	3086.36	3104.52	3542.65
3634.52	3839.40	3847.45			

- Conformer (100); ZPE = 33665.585 cm^{-1}:

33.00	50.29	63.00	100.48	177.10	193.67
243.88	338.28	341.83	400.24	441.01	520.74
576.01	593.32	630.60	654.12	740.03	758.31
781.08	803.03	870.40	926.04	980.83	1010.15
1077.83	1130.15	1150.97	1172.82	1214.91	1242.93
1267.56	1308.78	1341.77	1354.84	1366.03	1409.60
1451.38	1471.78	1481.90	1628.15	1859.13	1874.95
3036.45	3046.68	3081.55	3099.90	3141.55	3552.95
3653.28	3841.38	3844.52			

- Conformer (101); ZPE = 33767.332 cm^{-1}:

50.35	61.05	74.90	161.29	185.97	209.83
273.83	308.04	350.39	396.30	495.94	517.70
541.80	604.87	625.58	651.63	678.34	769.08
796.50	838.30	881.45	914.07	948.28	1036.05
1066.31	1148.03	1175.40	1200.38	1215.61	1223.68
1295.36	1316.57	1346.45	1361.87	1390.07	1396.45
1441.56	1460.96	1466.14	1659.06	1856.59	1859.34
3050.01	3064.80	3072.34	3091.13	3122.86	3552.94
3643.00	3841.26	3844.96			

- Conformer (102); ZPE = 33589.980 cm^{-1}:

34.21	51.75	60.71	127.30	138.79	215.25
282.46	287.34	306.69	359.54	492.22	518.88
540.24	577.38	612.39	646.99	652.56	759.39
776.21	823.12	893.87	914.68	1019.73	1071.82
1083.93	1142.52	1169.42	1175.06	1200.83	1251.41
1276.69	1305.27	1324.52	1340.93	1386.67	1438.50
1454.32	1458.55	1474.05	1650.66	1863.27	1873.57
2933.63	3043.14	3057.00	3099.11	3115.03	3556.99
3646.62	3846.28	3848.45			

- Conformer (103); ZPE = 34007.897 cm^{-1}:

53.41	90.72	116.45	159.12	212.99	273.72
294.03	309.91	348.03	364.05	503.62	510.00
612.77	617.28	665.10	684.17	758.38	789.53
811.90	853.29	880.20	949.71	968.98	1005.76
1076.95	1161.02	1178.73	1212.31	1222.89	1272.41
1284.79	1320.64	1331.36	1388.00	1394.99	1417.26
1431.59	1461.19	1494.05	1663.49	1816.52	1884.61
3056.25	3062.45	3098.16	3112.37	3116.74	3552.76
3637.72	3691.04	3842.37			

- Conformer (104); ZPE = 33809.959 cm^{-1}:

72.41	75.70	99.83	125.71	171.80	231.35
282.75	341.57	354.31	437.62	470.39	531.23
558.08	575.90	609.29	645.01	752.02	766.10
787.11	827.75	862.92	928.69	976.70	995.43
1089.72	1131.50	1166.14	1208.10	1214.52	1245.75
1256.03	1317.26	1341.40	1356.44	1376.62	1427.01
1455.24	1466.95	1483.10	1654.95	1836.46	1844.90
3007.70	3057.29	3075.17	3111.20	3136.74	3543.56
3642.06	3843.56	3850.87			

- Conformer (105); ZPE = 33798.808 cm^{-1}:

21.82	49.62	63.53	116.68	134.58	198.02
294.17	335.40	341.15	364.99	452.63	533.55
590.76	617.91	653.41	701.38	754.90	791.86
799.11	852.15	893.74	925.51	1005.46	1048.13
1072.85	1132.62	1142.26	1222.46	1233.51	1254.77
1296.36	1308.12	1349.90	1367.42	1385.02	1402.45
1429.71	1461.17	1488.35	1659.78	1870.62	1887.02
3026.64	3035.62	3066.72	3085.04	3130.66	3558.78
3643.64	3697.63	3848.05			

- Conformer (106); ZPE = 33732.226 cm^{-1}:

36.98	42.78	74.11	113.68	171.29	219.60
296.10	361.40	369.71	408.02	450.46	488.78
569.10	584.46	591.31	634.28	727.02	751.56
818.04	847.86	893.44	925.28	969.70	1042.59
1066.02	1147.47	1170.89	1183.10	1229.09	1247.97
1255.44	1316.55	1344.17	1348.45	1385.17	1408.05
1445.66	1466.83	1471.71	1670.42	1852.10	1871.59
2929.04	3062.06	3067.04	3118.11	3124.60	3556.12
3640.71	3847.59	3850.94			

- Conformer (107); ZPE = 33740.033 cm^{-1}:

36.74	55.71	66.31	132.36	169.42	234.51
255.83	271.03	298.86	419.18	461.99	494.13
579.20	590.28	616.99	640.89	757.52	789.35
800.55	840.12	864.78	926.57	986.43	1046.41
1071.18	1137.52	1159.58	1171.57	1187.26	1246.11
1285.50	1310.77	1325.35	1353.51	1396.88	1414.04
1429.12	1468.73	1485.46	1653.78	1860.17	1870.52
3051.22	3067.14	3086.15	3109.93	3148.60	3538.86
3630.27	3840.98	3844.70			

- Conformer (108); ZPE = 33759.740 cm^{-1}:

26.48	46.36	71.68	99.50	114.51	238.49
275.81	299.90	366.74	413.10	451.75	533.38
580.83	591.03	632.56	714.44	757.90	779.41
798.46	847.32	879.69	933.72	979.09	1045.94
1084.06	1121.28	1161.75	1216.86	1238.92	1252.33
1258.33	1317.66	1318.10	1381.76	1392.79	1398.66
1428.03	1463.35	1486.96	1657.05	1872.34	1891.64
3034.25	3039.60	3052.09	3107.15	3123.37	3549.47
3647.06	3699.38	3847.20			

- Conformer (109); ZPE = 33654.601 cm^{-1}:

27.50	29.76	47.13	93.20	184.42	242.80
280.83	290.76	328.78	375.87	487.67	510.95
553.97	585.04	600.86	645.81	688.77	767.38
799.57	841.86	867.46	950.67	986.73	1001.31
1072.67	1130.45	1162.38	1181.30	1215.85	1243.79
1289.23	1312.32	1340.86	1368.63	1393.29	1401.67
1422.79	1446.57	1472.92	1658.18	1866.55	1868.78
3041.42	3056.51	3062.95	3114.09	3128.90	3541.01
3634.84	3844.54	3847.60			

- Conformer (110); ZPE = 33633.501 cm^{-1}:

21.91	35.96	62.60	89.09	139.73	197.30
234.16	274.85	332.50	435.48	456.47	528.12
546.00	591.08	636.25	659.49	674.75	777.14
780.54	829.88	889.83	921.78	949.80	1055.77
1071.59	1156.42	1169.67	1182.76	1213.54	1238.16
1287.00	1307.23	1329.75	1370.44	1379.79	1401.91
1433.52	1451.23	1475.16	1672.47	1863.17	1868.49
3038.09	3070.18	3077.48	3080.91	3127.35	3557.01
3635.79	3840.47	3846.97			

- Conformer (111); ZPE = 33733.985 cm^{-1}:

63.31	66.40	106.41	125.20	190.13	230.04
284.86	297.49	356.53	427.23	495.83	517.60
548.79	582.64	606.28	644.63	669.70	742.65
786.47	834.19	868.48	933.40	957.13	1003.10
1082.60	1140.11	1181.87	1196.86	1230.57	1237.00
1254.18	1325.38	1344.15	1350.17	1388.42	1401.01
1447.12	1447.96	1488.44	1633.87	1859.62	1875.60
2955.96	3057.61	3068.53	3116.19	3133.79	3558.02
3653.36	3846.64	3854.41			

- Conformer (112); ZPE = 33794.834 cm^{-1}:

20.32	53.21	74.27	93.86	170.56	189.91
250.81	302.03	357.69	433.20	454.30	553.39
570.86	620.84	652.46	709.52	758.96	802.07
805.79	838.51	898.05	920.58	977.66	1026.99
1049.15	1130.25	1167.42	1185.85	1234.77	1271.03
1282.02	1316.76	1350.58	1369.58	1384.24	1396.13
1425.62	1463.98	1483.67	1654.90	1875.27	1896.06
3043.76	3048.85	3058.70	3106.04	3125.94	3550.77
3633.83	3700.31	3848.35			

- Conformer (113); ZPE = 33781.980 cm^{-1}:

18.06	43.94	72.81	101.46	112.83	228.19
276.99	303.50	373.91	416.10	439.07	537.29
589.95	592.07	634.39	733.87	748.33	793.49
808.93	857.40	884.07	934.12	974.90	1046.48
1079.84	1122.24	1157.70	1218.68	1232.72	1250.28
1268.77	1300.69	1327.47	1383.33	1391.18	1402.11
1430.54	1466.92	1487.53	1658.59	1868.66	1891.59
3023.58	3040.12	3052.59	3113.14	3130.51	3548.68
3647.13	3699.87	3847.37			

- Conformer (114); ZPE = 34026.577 cm^{-1}:

60.64	93.50	103.19	136.02	195.54	256.53
295.74	312.51	375.72	387.86	457.17	556.68
562.15	626.60	719.41	753.26	779.80	791.05
812.38	866.67	889.07	950.90	995.98	1041.71
1101.16	1141.62	1181.15	1187.56	1245.34	1266.50
1304.19	1315.12	1338.39	1381.35	1398.36	1411.60
1444.13	1476.11	1486.71	1659.95	1858.88	1888.94
3023.50	3042.14	3059.62	3098.58	3140.50	3544.49
3638.20	3691.81	3707.14			

- Conformer (115); ZPE = 33608.264 cm^{-1}:

27.05	45.67	59.76	86.45	169.18	186.40
218.81	287.65	336.10	429.03	440.66	526.22
571.52	592.96	619.90	660.65	715.71	763.32
787.32	801.38	862.39	928.62	961.32	1022.69
1077.37	1133.30	1171.69	1191.38	1234.67	1247.52
1259.99	1308.68	1324.76	1347.86	1385.23	1394.79
1448.36	1463.54	1486.36	1638.01	1867.37	1871.23
3034.60	3047.23	3056.58	3099.71	3124.29	3554.16
3650.85	3846.83	3849.43			

- Conformer (116); ZPE = 33566.923 cm^{-1}:

32.03	52.37	78.08	101.25	109.43	203.98
227.27	256.16	319.81	397.01	469.11	518.98
531.83	596.76	635.69	662.29	665.08	769.84
784.26	820.45	870.81	926.62	955.92	1081.06
1095.25	1156.36	1163.21	1173.88	1193.99	1251.56
1273.04	1311.68	1319.76	1359.98	1367.57	1433.58
1450.89	1455.13	1478.93	1636.36	1867.61	1871.79
3026.75	3041.95	3052.46	3081.33	3105.87	3552.60
3653.40	3845.93	3846.93			

- Conformer (117); ZPE = 33772.230 cm^{-1}:

31.73	58.44	70.10	110.32	172.92	250.62
274.41	319.86	350.13	404.37	447.31	552.85
564.14	573.61	626.47	653.25	729.01	756.14
823.29	840.74	884.38	928.43	964.88	1045.45
1050.34	1136.24	1163.54	1187.09	1192.59	1234.13
1278.05	1309.35	1325.14	1380.26	1395.03	1397.49
1423.14	1467.98	1477.12	1673.50	1858.69	1871.98
3040.80	3067.57	3075.12	3119.34	3130.92	3539.21
3628.86	3841.02	3847.10			

- Conformer (118); ZPE = 33913.899 cm^{-1}:

40.66	63.57	70.91	131.76	214.40	241.19
292.70	311.70	371.01	416.06	473.87	538.20
551.92	604.36	644.85	721.41	747.85	826.88
853.75	888.00	928.07	938.69	992.45	1016.30
1069.09	1143.05	1178.98	1187.70	1226.69	1251.13
1311.77	1340.43	1347.36	1361.51	1382.37	1445.00
1453.12	1464.77	1472.86	1650.25	1876.90	1882.88
2978.70	3044.49	3056.33	3117.86	3144.84	3535.51
3552.75	3628.92	3841.96			

- Conformer (119); ZPE = 33773.003 cm^{-1}:

7.24	54.89	58.82	105.58	162.13	207.35
280.39	296.59	370.70	436.89	478.96	547.61
567.90	579.06	634.66	699.29	752.68	786.06
793.02	840.35	867.39	913.42	971.20	1031.24
1095.40	1133.78	1176.77	1202.94	1236.00	1248.77
1270.98	1316.23	1330.32	1384.70	1396.56	1400.17
1429.34	1453.42	1470.99	1658.95	1873.79	1889.88
3029.51	3045.23	3066.59	3110.53	3127.14	3553.75
3643.41	3711.19	3846.25			

- Conformer (120); ZPE = 33874.468 cm^{-1}:

19.37	52.86	72.31	139.43	171.18	245.21
283.88	297.73	339.92	407.34	449.40	533.14
595.28	601.03	643.16	718.41	752.83	831.53
858.03	892.33	926.89	946.22	1006.41	1027.16
1063.29	1155.71	1166.71	1201.26	1209.59	1278.22
1312.56	1323.38	1359.30	1374.76	1383.22	1406.19
1456.82	1464.36	1480.75	1662.87	1872.43	1881.61
3031.62	3042.94	3064.52	3111.44	3139.49	3499.21
3535.36	3618.97	3841.29			

- Conformer (121); ZPE = 33778.338 cm^{-1}:

14.28	50.23	71.38	105.29	145.85	190.05
297.07	333.47	341.26	366.55	444.48	533.72
590.41	616.86	650.27	709.29	742.97	787.50
810.01	843.10	901.67	913.26	1001.98	1043.79
1072.72	1127.83	1146.66	1206.18	1246.62	1252.86
1289.06	1314.08	1343.53	1365.90	1386.12	1401.32
1425.09	1462.27	1488.99	1658.45	1874.59	1889.95
3027.46	3037.52	3054.52	3085.73	3148.46	3556.14
3641.01	3700.88	3847.99			

- Conformer (122); ZPE = 33883.783 cm^{-1}:

37.64	43.62	85.28	156.29	186.47	248.11
318.28	367.99	379.76	417.60	493.77	516.81
543.88	584.74	640.71	646.95	769.80	824.41
836.19	857.95	906.07	928.39	960.31	1030.18
1060.54	1152.22	1169.77	1186.07	1223.36	1246.63
1289.02	1306.58	1353.31	1371.76	1383.14	1415.05
1435.84	1455.61	1462.65	1659.09	1881.36	1889.21
3026.66	3044.14	3063.73	3074.67	3124.02	3546.15
3634.18	3637.30	3894.29			

- Conformer (123); ZPE = 33781.966 cm^{-1}:

27.95	48.10	56.66	131.07	137.39	217.12
283.55	308.02	371.29	376.83	468.76	548.80
566.94	592.54	633.07	701.98	742.19	783.84
793.56	855.02	890.11	909.19	1024.22	1069.09
1092.63	1128.18	1169.70	1201.31	1227.16	1243.09
1279.15	1307.34	1341.68	1377.63	1393.24	1404.33
1433.44	1464.31	1479.05	1658.41	1866.81	1885.95
3028.07	3035.32	3068.60	3086.51	3121.83	3543.67
3636.68	3706.50	3846.05			

- Conformer (124); ZPE = 33706.010 cm^{-1}:

35.27	54.03	67.70	106.34	174.71	194.91
253.97	338.82	344.31	400.82	441.39	520.74
575.10	597.12	632.05	655.70	747.77	763.27
798.57	801.38	868.60	923.68	982.32	1012.40
1081.40	1133.97	1155.30	1175.96	1204.39	1243.47
1265.85	1312.37	1323.22	1362.01	1368.36	1402.61
1451.74	1473.24	1485.42	1627.65	1872.10	1873.80
3035.72	3046.08	3081.59	3099.91	3140.02	3558.88
3661.57	3838.53	3845.87			

- Conformer (125); ZPE = 33679.567 cm^{-1}:

30.52	49.74	63.66	94.31	183.39	264.28
287.37	288.90	328.51	393.51	482.16	516.34
565.27	577.05	611.31	625.36	689.17	757.40
789.37	829.75	884.21	914.05	991.81	1039.77
1088.99	1138.78	1161.07	1196.41	1213.73	1238.81
1278.48	1324.87	1326.24	1352.08	1386.55	1407.71
1456.46	1461.85	1468.97	1651.77	1868.47	1874.63
2939.12	3058.31	3065.68	3116.07	3140.20	3553.37
3640.82	3845.29	3847.17			

- Conformer (126); ZPE = 33643.948 cm^{-1}:

22.59	24.67	44.78	92.05	172.01	234.54
276.81	286.64	331.07	375.66	483.85	542.44
549.47	589.66	602.58	643.58	679.05	783.10
804.81	838.87	863.49	934.29	977.34	997.60
1066.14	1132.95	1154.39	1191.46	1216.52	1240.68
1295.77	1314.21	1345.25	1375.23	1390.43	1394.78
1397.47	1447.46	1476.65	1659.65	1868.17	1872.15
3044.01	3060.67	3079.00	3100.97	3133.28	3547.33
3636.09	3846.25	3849.96			

- Conformer (127); ZPE = 33876.024 cm^{-1}:

37.85	60.34	65.64	113.57	176.70	199.11
268.90	327.47	358.45	406.67	446.50	554.21
574.18	633.61	660.30	738.38	761.89	787.62
804.25	861.41	897.32	943.27	981.17	1020.22
1058.95	1114.97	1169.21	1186.21	1237.40	1257.97
1288.23	1315.33	1325.18	1369.83	1390.56	1407.63
1422.48	1470.14	1488.92	1668.65	1878.91	1899.41
3021.36	3050.78	3075.26	3111.48	3136.46	3552.66
3631.06	3707.01	3836.96			

- Conformer (128); ZPE = 33659.686 cm^{-1}:

25.17	39.05	65.27	101.65	128.68	189.90
257.08	283.80	326.05	352.90	437.43	506.21
588.48	614.17	634.67	651.69	709.27	760.03
802.59	843.67	901.49	918.69	1014.33	1060.17
1077.52	1129.38	1166.59	1180.64	1227.57	1238.88
1294.78	1299.60	1321.99	1374.85	1379.44	1400.22
1433.32	1457.78	1484.68	1668.25	1865.05	1869.84
3045.93	3053.34	3066.98	3099.68	3124.52	3539.00
3623.63	3832.77	3850.73			

- Conformer (129); ZPE = 33643.912 cm^{-1}:

9.90	30.45	60.91	97.34	124.37	236.86
249.49	263.78	299.24	396.59	443.98	540.52
572.10	599.38	627.38	649.78	705.74	778.07
786.96	834.65	891.46	949.87	966.55	1017.66
1081.89	1122.21	1172.68	1200.62	1227.27	1255.03
1271.94	1319.74	1332.18	1379.58	1386.73	1394.73
1399.89	1463.80	1485.33	1665.22	1864.11	1869.77
3041.38	3057.81	3062.26	3098.79	3127.21	3549.68
3634.38	3841.24	3849.31			

- Conformer (130); ZPE = 33610.419 cm^{-1}:

18.46	31.04	55.06	88.59	125.86	236.82
255.63	294.14	321.77	379.10	429.86	521.30
571.23	585.87	612.79	641.88	714.44	760.98
782.28	847.16	897.44	969.00	971.82	1019.97
1078.15	1116.94	1165.55	1186.11	1222.81	1257.79
1268.72	1316.88	1324.00	1368.75	1391.21	1400.62
1416.92	1464.29	1483.70	1659.25	1865.78	1868.11
3020.32	3051.74	3060.44	3103.12	3125.65	3541.02
3633.33	3846.00	3851.16			

- Conformer (131); ZPE = 33829.892 cm^{-1}:

29.72	47.40	66.70	101.94	172.25	264.13
265.05	296.76	356.49	405.16	436.37	542.69
580.03	604.06	641.53	699.45	757.68	809.00
831.01	840.25	880.01	963.09	972.15	1010.77
1061.49	1145.80	1162.18	1196.58	1250.77	1252.81
1287.61	1312.98	1340.77	1371.45	1378.17	1394.36
1434.14	1459.28	1484.36	1664.16	1880.01	1888.59
3031.32	3039.36	3053.33	3122.50	3141.99	3568.24
3658.52	3661.31	3843.99			

- Conformer (132); ZPE = 33655.806 cm^{-1}:

21.31	46.01	55.24	117.06	171.02	206.45
269.01	285.35	339.91	435.98	443.63	542.93
559.93	568.64	613.81	653.20	699.44	752.52
786.37	829.46	884.08	930.56	972.01	1020.33
1080.36	1132.10	1179.50	1202.45	1205.40	1244.70
1285.10	1311.45	1342.07	1353.53	1383.80	1402.93
1447.40	1464.43	1478.08	1649.38	1867.10	1870.72
2944.29	3051.38	3062.72	3107.66	3134.75	3560.97
3649.80	3846.54	3848.74			

- Conformer (133); ZPE = 33697.982 cm^{-1}:

65.53	71.15	85.89	130.06	180.27	229.95
262.88	306.33	332.83	346.90	488.60	519.61
574.98	586.23	600.64	650.34	694.36	759.14
779.66	845.11	857.23	904.46	984.65	1070.56
1074.98	1162.27	1167.28	1176.47	1222.01	1243.52
1253.52	1328.93	1336.83	1344.15	1376.30	1393.80
1445.83	1460.99	1481.73	1640.61	1867.88	1876.32
3023.06	3045.95	3056.97	3098.55	3110.27	3547.19
3641.17	3844.85	3847.16			

- Conformer (134); ZPE = 33634.361 cm^{-1}:

19.13	45.65	49.75	93.27	159.77	189.30
230.79	283.24	332.22	429.46	434.81	525.76
578.53	594.87	622.46	664.95	727.90	773.46
788.85	817.30	867.00	930.35	947.98	1033.62
1075.02	1128.25	1174.03	1193.94	1234.40	1254.20
1258.49	1304.39	1326.54	1343.64	1386.55	1398.80
1452.83	1470.10	1486.04	1639.19	1866.40	1869.69
3034.61	3042.71	3055.41	3100.67	3132.74	3554.90
3650.13	3846.73	3847.87			

- Conformer (135); ZPE = 33591.908 cm^{-1}:

27.45	44.28	60.45	130.07	137.62	218.33
256.33	303.52	335.30	359.98	487.27	520.77
538.12	580.90	627.90	651.03	653.38	745.35
774.86	822.16	875.34	914.01	1023.66	1073.82
1085.73	1149.72	1171.83	1185.58	1200.35	1245.88
1272.35	1301.86	1326.83	1348.09	1383.16	1437.67
1446.12	1457.95	1479.23	1638.32	1864.21	1868.54
2920.58	3046.10	3061.41	3100.25	3117.59	3563.24
3658.73	3842.51	3848.09			

- Conformer (136); ZPE = 33647.341 cm^{-1}:

20.62	41.97	62.93	98.29	144.06	184.49
238.38	283.96	325.45	352.39	434.85	502.79
588.03	608.45	625.50	650.57	715.70	762.87
809.40	838.37	906.71	912.11	1020.68	1047.97
1070.23	1134.64	1169.48	1178.73	1215.08	1249.88
1282.62	1298.97	1334.30	1373.38	1380.37	1399.59
1431.23	1457.95	1485.59	1668.95	1864.17	1870.82
3036.32	3053.32	3066.99	3113.86	3137.73	3538.10
3622.63	3832.88	3850.33			

- Conformer (137); ZPE = 33577.636 cm^{-1}:

28.58	40.43	71.35	101.63	133.24	197.94
242.60	298.37	318.74	334.79	470.62	520.80
561.75	592.90	611.00	643.48	654.75	764.21
803.66	846.92	861.44	907.93	1021.45	1074.29
1086.66	1115.54	1163.41	1170.87	1194.21	1245.94
1280.93	1306.19	1338.63	1364.37	1368.65	1430.31
1444.11	1450.60	1480.37	1651.41	1861.88	1869.94
3041.69	3053.22	3067.03	3084.23	3102.08	3546.74
3635.23	3847.69	3850.46			

- Conformer (138); ZPE = 33682.747 cm^{-1}:

27.01	72.47	84.91	137.00	163.21	187.39
234.78	307.30	361.03	432.56	476.60	516.60
554.77	571.93	590.21	622.57	744.67	757.21
790.43	833.75	870.98	919.43	975.72	1000.31
1077.37	1123.00	1172.37	1190.15	1221.52	1243.16
1255.13	1333.29	1340.90	1356.03	1377.35	1412.00
1445.37	1465.65	1472.74	1651.20	1855.40	1859.13
2951.31	3056.99	3082.08	3110.82	3150.06	3570.52
3659.16	3849.02	3850.93			

- Conformer (139); ZPE = 33886.053 cm^{-1}:

55.67	70.21	106.99	114.64	152.29	239.09
304.35	328.80	382.45	410.67	444.76	546.49
563.20	577.29	591.65	732.72	767.66	773.00
829.31	854.02	859.37	931.15	1005.64	1035.45
1090.19	1120.22	1170.11	1193.37	1234.82	1243.09
1264.10	1316.49	1343.83	1373.86	1395.19	1428.98
1433.86	1467.78	1482.35	1657.50	1855.25	1888.02
3030.36	3052.30	3069.34	3104.05	3131.91	3543.34
3637.51	3720.56	3846.88			

- Conformer (140); ZPE = 33640.330 cm^{-1}:

33.70	48.41	56.74	98.50	173.36	231.91
257.69	307.96	340.09	391.33	469.69	522.77
549.51	590.40	631.68	658.43	700.28	760.55
802.87	822.43	852.51	932.07	963.14	1053.06
1068.63	1139.07	1158.06	1160.40	1216.89	1222.86
1277.56	1308.03	1320.80	1361.13	1372.43	1424.83
1450.86	1459.87	1477.58	1638.33	1865.18	1881.45
3039.32	3049.66	3053.39	3101.34	3105.63	3547.64
3645.15	3839.27	3846.20			

- Conformer (141); ZPE = 33637.456 cm^{-1}:

21.88	49.01	56.64	124.52	163.16	205.22
254.50	282.41	337.54	419.65	458.70	548.36
554.72	566.02	616.79	658.54	692.47	757.54
777.60	829.96	877.92	928.58	966.04	1028.88
1084.07	1135.90	1179.25	1192.55	1221.68	1235.97
1291.82	1308.03	1340.49	1350.28	1387.57	1405.81
1449.70	1463.77	1475.22	1650.30	1864.35	1869.34
2942.75	3042.73	3058.15	3110.78	3134.29	3559.07
3647.62	3847.66	3849.11			

- Conformer (142); ZPE = 33885.179 cm^{-1}:

54.46	77.11	116.29	163.15	190.24	226.18
248.96	297.59	313.41	399.79	471.25	547.79
599.28	606.57	630.14	739.66	773.43	782.80
807.03	831.36	871.21	900.15	1005.57	1034.26
1079.70	1132.33	1158.84	1185.59	1216.51	1263.12
1291.07	1334.95	1347.37	1361.68	1396.56	1412.25
1450.74	1480.26	1484.88	1651.71	1827.11	1883.70
3032.12	3060.77	3069.91	3091.61	3134.46	3549.44
3640.40	3708.63	3836.97			

- Conformer (143); ZPE = 33697.236 cm^{-1}:

26.59	68.87	100.51	127.41	168.04	240.73
282.58	300.32	347.92	393.95	480.35	532.55
544.55	573.89	603.76	664.76	690.70	766.05
776.49	832.24	863.83	928.21	957.83	983.16
1095.89	1132.31	1174.36	1190.79	1223.76	1238.26
1256.28	1320.21	1339.35	1345.72	1394.85	1398.26
1448.89	1464.11	1483.17	1639.00	1861.92	1877.30
3044.41	3052.75	3057.82	3099.40	3113.81	3546.80
3643.53	3844.35	3851.89			

- Conformer (144); ZPE = 33681.218 cm^{-1}:

29.02	42.84	56.73	91.25	198.16	265.23
296.06	304.44	347.04	386.61	484.05	521.35
568.03	574.55	611.66	635.91	685.35	742.04
786.75	832.29	874.04	911.56	982.76	1044.98
1093.22	1149.22	1171.67	1195.93	1224.92	1236.43
1263.47	1322.57	1330.95	1359.41	1384.53	1401.68
1443.88	1460.22	1478.25	1632.59	1866.06	1869.40
2925.76	3056.66	3068.48	3113.72	3144.37	3557.80
3651.89	3842.12	3844.50			

- Conformer (145); ZPE = 33723.643 cm^{-1}:

31.85	41.65	66.03	105.15	175.80	191.64
268.78	330.43	359.52	402.89	438.59	539.26
579.70	589.73	618.27	641.05	745.06	779.04
805.08	820.25	867.07	925.54	977.96	1011.39
1075.68	1123.05	1127.38	1205.07	1207.86	1246.19
1268.79	1315.50	1347.26	1363.67	1371.37	1412.54
1428.23	1471.17	1483.14	1644.24	1856.50	1875.52
3040.05	3071.82	3082.26	3098.07	3142.14	3545.13
3640.13	3842.74	3850.06			

- Conformer (146); ZPE = 33678.678 cm^{-1}:

27.60	50.69	63.02	125.15	184.22	237.34
269.96	298.82	312.83	354.24	479.31	511.40
570.90	598.57	620.30	641.54	679.93	782.11
787.00	831.55	862.79	901.51	990.65	1043.94
1103.27	1146.70	1162.66	1171.76	1225.52	1233.81
1263.39	1321.50	1340.05	1360.05	1370.70	1396.67
1460.71	1463.90	1481.01	1638.47	1869.57	1873.55
3041.71	3056.86	3073.01	3096.08	3119.31	3541.11
3635.44	3839.96	3845.22			

- Conformer (147); ZPE = 33895.347 cm^{-1}:

31.58	41.91	70.73	192.29	203.51	241.60
304.35	333.57	380.77	431.35	492.06	522.84
542.44	568.99	600.47	641.12	771.39	800.43
855.46	866.56	898.16	941.54	956.83	1049.16
1079.54	1153.10	1179.66	1210.94	1220.58	1254.18
1296.42	1304.26	1333.26	1384.93	1405.34	1417.16
1444.40	1457.43	1460.65	1672.97	1869.79	1881.29
3034.08	3048.05	3061.74	3077.07	3116.22	3541.49
3608.25	3645.06	3893.73			

- Conformer (148); ZPE = 33658.619 cm^{-1}:

24.28	43.93	59.63	125.49	133.52	205.28
273.94	277.39	301.12	379.15	446.96	525.77
586.01	596.18	623.50	628.53	705.95	766.44
789.16	835.34	895.28	907.34	1019.47	1058.84
1079.09	1137.01	1153.04	1178.22	1227.14	1240.99
1296.47	1302.92	1320.38	1378.77	1392.61	1413.09
1422.28	1460.93	1478.33	1655.91	1861.62	1866.02
3044.91	3050.43	3061.21	3100.88	3120.09	3540.90
3633.14	3844.57	3847.78			

- Conformer (149); ZPE = 33690.394 cm^{-1}:

31.27	78.07	86.93	133.10	171.77	247.48
257.35	297.49	334.00	405.47	462.91	537.49
560.04	575.05	613.10	660.86	694.74	754.53
776.70	824.76	861.31	927.06	974.64	984.98
1084.46	1128.61	1171.72	1184.68	1222.59	1243.52
1262.09	1312.21	1336.18	1346.74	1385.67	1403.98
1457.73	1459.13	1480.30	1638.95	1864.77	1878.14
3037.59	3054.97	3063.97	3103.52	3131.64	3539.76
3642.60	3838.66	3855.47			

- Conformer (150); ZPE = 33683.659 cm^{-1}:

45.77	66.01	84.24	128.54	167.28	236.46
264.73	286.28	321.83	352.06	506.89	508.72
572.47	584.37	594.69	620.95	675.79	766.21
781.16	838.78	876.24	910.73	993.65	1025.82
1080.15	1136.06	1177.66	1192.87	1219.02	1252.73
1278.13	1334.03	1341.38	1374.57	1387.86	1395.62
1404.52	1443.99	1483.18	1663.61	1855.17	1876.56
3050.47	3059.51	3062.53	3106.50	3111.91	3546.35
3629.91	3845.06	3848.30			

- Conformer (151); ZPE = 33735.020 cm^{-1}:

39.81	68.65	83.54	102.40	206.89	226.75
282.03	304.30	340.49	409.64	453.74	531.35
550.07	575.87	624.36	649.93	699.31	758.28
803.26	856.34	908.59	932.03	961.87	1019.39
1086.06	1132.94	1171.98	1178.51	1212.79	1236.16
1252.38	1314.31	1333.59	1356.99	1379.44	1432.41
1454.52	1471.00	1481.85	1650.63	1863.77	1873.32
2956.86	3056.21	3062.76	3109.11	3117.45	3556.08
3644.04	3847.34	3848.63			

- Conformer (152); ZPE = 33601.515 cm^{-1}:

20.04	25.62	56.32	101.50	105.84	225.61
255.05	268.16	319.80	363.95	438.19	513.47
573.04	587.36	613.41	641.79	731.12	756.90
798.65	840.60	900.48	952.35	978.32	1023.63
1074.08	1116.54	1165.05	1183.81	1223.37	1259.35
1272.20	1307.52	1328.19	1366.20	1390.72	1403.78
1420.22	1468.02	1485.43	1657.27	1867.27	1869.31
3023.26	3041.14	3054.71	3111.96	3150.99	3540.21
3633.32	3846.67	3851.20			

- Conformer (153); ZPE = 33738.370 cm^{-1}:

47.56	59.73	93.12	126.75	157.73	215.76
281.73	293.00	328.37	417.01	454.80	498.31
569.27	582.09	593.03	619.72	753.61	762.24
812.08	840.73	901.77	916.58	993.08	1054.17
1063.35	1126.69	1162.93	1186.74	1229.94	1244.44
1262.73	1321.21	1331.74	1343.55	1384.51	1428.21
1458.82	1466.93	1481.56	1654.74	1851.38	1871.97
2967.87	3059.97	3070.88	3111.94	3134.19	3551.72
3638.65	3848.22	3849.60			

- Conformer (154); ZPE = 33864.943 cm^{-1}:

54.63	81.56	121.11	154.23	177.59	210.76
252.43	294.99	338.37	426.32	463.50	536.44
581.45	631.36	642.10	689.97	726.96	779.49
828.73	840.32	878.99	921.74	951.73	1031.81
1060.05	1143.05	1162.25	1185.08	1235.03	1257.87
1268.67	1319.54	1338.92	1353.27	1380.48	1403.03
1455.51	1484.27	1488.78	1653.88	1831.03	1881.96
3043.10	3053.17	3061.56	3096.92	3118.40	3563.55
3648.02	3790.86	3835.06			

- Conformer (155); ZPE = 33598.469 cm^{-1}:

12.87	17.59	64.69	97.01	109.65	231.17
247.28	264.85	290.17	380.60	444.51	544.36
560.64	600.43	617.69	646.35	696.18	784.69
799.21	832.43	889.53	943.50	958.00	1022.55
1079.08	1129.59	1160.24	1195.40	1218.65	1259.78
1272.87	1318.02	1330.34	1377.71	1387.66	1391.04
1400.95	1464.51	1483.94	1660.59	1867.99	1870.33
3029.39	3046.63	3072.45	3102.14	3144.52	3547.19
3634.46	3846.75	3848.79			

- Conformer (156); ZPE = 33753.994 cm^{-1}:

40.83	74.42	77.14	123.74	174.68	239.12
282.99	289.51	346.45	398.85	484.05	541.50
554.58	580.57	602.87	650.89	700.08	772.38
785.46	843.63	885.55	921.43	968.41	984.62
1077.63	1137.86	1155.78	1189.62	1208.59	1250.41
1257.91	1330.56	1340.59	1377.56	1384.94	1396.66
1406.74	1457.35	1470.36	1671.16	1866.54	1875.01
3052.41	3066.29	3089.64	3112.36	3122.65	3553.99
3634.48	3846.87	3850.23			

- Conformer (157); ZPE = 33850.904 cm^{-1}:

41.51	78.66	82.40	98.02	200.60	221.59
299.21	321.54	377.10	432.73	488.03	533.88
558.26	573.12	614.68	706.56	763.10	773.00
816.84	862.42	873.18	942.31	957.44	1017.63
1106.95	1132.45	1166.52	1189.19	1220.50	1241.01
1262.93	1316.73	1324.85	1374.27	1393.60	1429.48
1436.21	1464.61	1477.54	1660.02	1870.45	1888.98
3040.12	3050.39	3062.89	3097.16	3112.65	3542.93
3636.83	3720.82	3847.92			

- Conformer (158); ZPE = 33642.336 cm^{-1}:

17.06	35.39	53.04	114.47	171.19	243.10
264.48	290.75	333.05	373.80	493.99	523.72
540.45	590.60	620.92	649.04	674.92	769.97
828.42	839.04	848.31	932.97	960.57	1050.47
1070.88	1120.06	1159.33	1185.91	1212.70	1238.66
1283.09	1313.26	1324.62	1375.91	1378.83	1428.52
1432.98	1453.77	1473.58	1651.43	1860.91	1871.54
3046.78	3047.35	3069.96	3090.54	3104.52	3544.04
3635.44	3841.02	3849.29			

- Conformer (159); ZPE = 33583.142 cm^{-1}:

31.57	45.10	80.32	95.65	116.61	234.87
249.65	274.15	334.15	410.58	457.97	528.91
534.44	577.13	623.13	650.90	662.24	745.98
785.58	848.17	892.60	924.39	965.44	1082.82
1092.98	1151.43	1172.05	1182.95	1189.49	1246.11
1273.26	1309.30	1324.18	1348.19	1374.89	1431.54
1452.76	1457.34	1473.45	1657.95	1869.22	1879.10
2923.26	3031.62	3071.11	3076.78	3123.43	3562.81
3645.53	3846.07	3847.17			

- Conformer (160); ZPE = 33692.787 cm^{-1}:

43.88	65.39	83.20	128.68	178.30	235.54
277.33	312.08	326.39	387.19	486.67	514.10
557.33	574.48	623.63	648.20	688.23	768.01
779.86	837.16	858.87	931.93	963.39	989.73
1103.77	1105.40	1151.20	1187.12	1212.44	1245.73
1250.46	1318.72	1330.30	1360.33	1390.05	1428.09
1437.59	1455.13	1467.33	1647.77	1863.61	1891.72
3047.63	3055.95	3083.31	3093.64	3101.22	3555.04
3648.19	3846.01	3848.25			

- Conformer (161); ZPE = 33706.243 cm^{-1}:

59.09	73.35	78.52	129.54	165.00	243.83
252.96	289.38	314.57	379.07	445.60	505.89
575.35	594.25	600.18	651.07	725.88	765.14
804.73	855.85	865.81	913.40	1001.33	1052.41
1057.71	1139.24	1168.58	1184.37	1218.72	1240.66
1259.11	1323.01	1332.61	1356.98	1368.12	1397.84
1461.42	1467.34	1483.05	1640.91	1857.98	1863.05
3050.01	3062.12	3064.60	3104.35	3124.74	3536.46
3629.27	3828.36	3849.68			

- Conformer (162); ZPE = 33873.860 cm^{-1}:

56.47	96.91	114.02	124.17	201.39	226.26
271.77	284.14	345.47	407.80	452.59	553.06
576.92	609.62	667.44	704.39	739.93	765.36
796.04	852.70	907.10	941.85	955.21	1016.97
1067.13	1130.17	1184.27	1205.62	1218.91	1266.77
1294.28	1315.63	1345.25	1359.14	1394.36	1399.69
1460.25	1477.04	1488.45	1651.19	1842.49	1887.87
2949.93	3055.00	3062.33	3098.71	3146.69	3555.80
3644.44	3740.90	3837.82			

- Conformer (163); ZPE = 33652.029 cm^{-1}:

21.87	28.26	52.25	100.90	128.36	199.74
269.75	278.58	323.14	362.39	435.85	529.42
591.73	607.13	619.89	625.39	717.44	762.86
805.82	833.11	893.02	910.10	1010.82	1037.86
1069.15	1124.94	1166.60	1193.10	1228.18	1243.35
1297.88	1305.01	1341.27	1370.64	1386.67	1394.02
1407.17	1462.31	1489.19	1663.78	1862.43	1871.55
3043.91	3052.31	3074.18	3099.42	3130.25	3549.38
3635.61	3845.92	3850.17			

- Conformer (164); ZPE = 33695.150 cm^{-1}:

26.83	56.76	73.42	80.57	173.70	191.38
240.53	302.17	356.21	426.31	441.70	520.00
570.24	585.45	609.29	662.64	702.26	758.65
819.88	821.23	882.97	925.45	976.88	1010.22
1064.35	1153.91	1177.29	1209.87	1229.17	1253.70
1266.12	1310.22	1330.36	1345.18	1383.05	1387.37
1455.05	1458.21	1486.76	1659.54	1860.07	1873.84
3018.42	3031.21	3065.10	3111.78	3141.63	3560.19
3649.49	3846.11	3847.57			

- Conformer (165); ZPE = 33985.335 cm^{-1}:

22.53	80.92	111.24	151.94	171.22	232.16
299.36	313.43	382.24	460.07	477.83	554.24
568.90	595.27	646.39	737.58	772.09	802.06
825.66	863.85	882.62	943.37	973.30	1007.47
1098.70	1133.42	1179.81	1187.33	1243.11	1260.24
1289.15	1303.36	1340.66	1365.50	1392.84	1401.19
1443.03	1464.72	1491.80	1675.75	1854.54	1871.63
3038.83	3062.28	3086.62	3112.01	3144.09	3534.51
3637.02	3649.03	3833.79			

- Conformer (166); ZPE = 33593.163 cm^{-1}:

31.41	42.59	74.89	87.07	148.42	190.57
254.77	295.14	323.27	427.90	452.69	526.02
552.73	582.82	631.99	655.27	667.89	761.13
794.67	817.88	851.73	931.72	952.13	1034.01
1098.95	1121.29	1163.82	1174.09	1221.76	1243.09
1263.27	1311.49	1320.19	1362.70	1376.22	1430.55
1440.21	1448.95	1478.69	1649.96	1866.28	1871.70
3040.45	3054.64	3070.20	3080.59	3107.34	3558.59
3650.75	3844.63	3847.21			

- Conformer (167); ZPE = 33738.851 cm^{-1}:

58.20	59.33	68.49	132.15	171.79	199.39
260.04	294.50	315.81	404.05	467.97	498.01
576.07	587.74	606.91	634.42	759.27	777.99
817.20	844.96	885.73	907.33	1003.74	1037.82
1070.80	1127.11	1139.33	1177.47	1214.74	1243.13
1275.30	1309.05	1334.36	1362.88	1396.75	1409.47
1429.77	1471.14	1478.48	1654.79	1860.45	1883.46
3051.28	3060.31	3063.10	3112.29	3135.36	3534.64
3625.32	3843.05	3844.98			

- Conformer (168); ZPE = 33635.279 cm^{-1}:

25.39	28.41	53.32	85.19	139.19	189.86
264.64	289.04	320.50	365.01	434.93	527.58
594.01	599.28	613.88	622.63	723.81	766.68
817.17	826.85	887.66	911.55	1017.36	1027.26
1064.98	1122.74	1161.69	1188.80	1220.10	1256.26
1279.28	1318.04	1344.55	1366.12	1386.55	1395.75
1405.36	1460.03	1484.96	1659.81	1867.78	1871.29
3038.81	3055.32	3076.41	3109.23	3128.65	3546.21
3634.77	3847.75	3848.06			

- Conformer (169); ZPE = 33933.428 cm^{-1}:

29.37	72.31	133.92	165.18	177.94	214.81
286.51	328.33	335.53	401.99	458.90	548.27
581.11	633.87	667.74	682.78	761.11	770.66
811.50	847.06	852.47	913.59	958.29	1042.66
1050.33	1149.78	1183.52	1189.28	1241.99	1266.97
1300.03	1313.64	1356.53	1361.14	1391.57	1408.05
1435.77	1466.09	1488.08	1662.00	1815.06	1875.80
3055.75	3062.39	3082.18	3111.37	3132.57	3572.05
3659.58	3727.10	3832.36			

- Conformer (170); ZPE = 33664.195 cm^{-1}:

22.15	32.57	57.02	113.91	150.35	210.12
253.53	269.30	340.96	402.85	453.92	550.58
558.38	565.56	620.27	656.95	694.42	757.74
778.25	834.21	897.53	910.88	959.23	1050.31
1075.96	1160.95	1163.68	1187.20	1225.57	1248.58
1298.24	1311.51	1329.50	1388.31	1391.53	1399.20
1411.85	1456.86	1469.69	1669.58	1858.91	1864.80
3029.95	3043.10	3072.22	3111.60	3133.19	3553.74
3635.96	3847.62	3848.11			

- Conformer (171); ZPE = 33853.077 cm^{-1}:

43.79	54.56	67.57	113.01	215.98	286.46
296.49	326.57	362.25	380.46	504.76	520.82
554.43	572.31	599.34	649.35	745.55	798.26
811.65	860.25	881.15	946.19	980.33	1068.39
1095.98	1146.49	1175.52	1193.58	1220.97	1243.55
1268.51	1308.72	1341.46	1376.50	1404.49	1413.26
1438.80	1458.28	1462.36	1661.43	1872.99	1886.10
3042.40	3045.86	3054.77	3091.57	3104.58	3543.35
3635.80	3687.80	3891.11			

- Conformer (172); ZPE = 33674.343 cm^{-1}:

15.74	27.94	38.98	124.66	164.49	258.41
266.08	305.34	307.79	360.82	506.60	520.56
577.20	591.16	608.46	623.29	689.60	782.05
797.12	833.61	856.37	915.07	963.79	1044.61
1078.55	1138.35	1162.36	1186.28	1216.93	1235.70
1295.78	1326.56	1356.16	1360.65	1384.56	1399.55
1408.82	1457.55	1474.96	1661.50	1863.95	1874.66
3044.17	3058.20	3094.30	3100.28	3118.91	3546.37
3634.25	3843.63	3845.95			

- Conformer (173); ZPE = 33694.078 cm^{-1}:

33.27	41.53	63.47	120.14	163.76	250.76
298.21	308.85	351.62	418.96	473.20	545.30
561.10	577.55	609.93	659.23	694.25	761.80
805.37	821.46	851.31	914.90	960.05	1012.13
1064.81	1128.01	1153.80	1185.02	1228.53	1240.79
1261.40	1312.80	1337.58	1365.04	1382.90	1397.31
1439.94	1457.73	1476.74	1655.03	1863.27	1865.28
3046.18	3056.31	3058.86	3105.09	3115.57	3554.93
3646.82	3844.13	3846.11			

- Conformer (174); ZPE = 33705.200 cm^{-1}:

54.32	60.64	72.08	157.67	187.09	204.27
215.92	299.14	333.81	390.36	486.47	520.33
544.97	583.07	621.85	648.92	678.36	764.12
800.55	850.74	866.23	937.11	954.24	1037.00
1070.79	1132.81	1152.94	1202.78	1211.99	1231.74
1292.64	1303.72	1336.72	1362.53	1382.29	1434.97
1441.96	1460.20	1467.55	1666.63	1855.34	1867.89
3048.87	3053.89	3085.87	3091.82	3108.01	3551.90
3633.10	3844.65	3847.54			

- Conformer (175); ZPE = 33669.044 cm^{-1}:

21.30	29.21	56.22	112.14	152.00	212.20
267.73	274.45	343.28	415.38	451.99	546.35
559.26	561.27	615.04	652.58	691.47	760.00
780.60	837.22	899.49	909.47	961.84	1051.30
1076.54	1159.14	1181.20	1186.04	1210.16	1253.74
1289.54	1316.26	1330.73	1383.28	1388.83	1400.86
1410.87	1455.72	1471.43	1669.90	1859.81	1867.52
3031.84	3039.65	3076.04	3105.17	3131.29	3552.31
3634.42	3845.83	3848.17			

- Conformer (176); ZPE = 33879.300 cm^{-1}:

52.49	94.27	101.26	127.67	192.95	252.44
271.31	285.90	317.46	391.15	458.77	514.87
581.52	626.14	627.74	722.21	757.38	767.97
803.32	832.61	895.25	942.59	1002.21	1040.42
1066.88	1142.58	1170.90	1190.26	1214.84	1273.77
1304.59	1312.54	1323.24	1377.61	1400.74	1409.01
1426.46	1476.83	1492.62	1656.92	1829.40	1890.71
3033.80	3042.91	3049.95	3118.99	3140.34	3545.01
3638.22	3731.95	3837.64			

- Conformer (177); ZPE = 33885.960 cm^{-1}:

41.92	73.84	143.50	163.40	178.55	225.71
259.01	273.61	340.02	397.26	469.12	523.67
593.89	627.88	671.89	727.50	754.70	776.42
820.99	844.43	860.32	919.53	941.15	1042.76
1056.12	1139.49	1180.73	1186.18	1203.61	1264.81
1301.38	1304.58	1355.74	1362.53	1386.17	1405.42
1440.40	1468.71	1481.14	1678.43	1825.95	1889.98
3052.70	3054.33	3076.20	3109.24	3124.86	3561.60
3645.48	3715.14	3829.96			

- Conformer (178); ZPE = 33837.508 cm^{-1}:

34.09	76.58	118.45	155.84	176.57	229.48
248.80	295.65	315.78	380.18	454.87	520.05
577.38	598.40	658.89	690.01	755.42	768.34
802.29	847.94	883.72	939.69	973.84	1057.30
1067.03	1145.55	1155.71	1186.43	1200.55	1243.82
1297.96	1320.97	1331.23	1358.64	1380.37	1391.82
1428.97	1476.34	1484.37	1669.01	1882.04	1892.18
3045.41	3057.83	3069.48	3107.39	3139.01	3532.37
3615.57	3798.63	3836.79			

- Conformer (179); ZPE = 33657.807 cm^{-1}:

22.59	32.30	55.78	87.45	151.04	197.59
235.58	274.50	331.64	434.78	443.93	543.31
567.78	589.08	618.31	666.43	701.50	776.30
782.84	828.63	886.96	915.00	954.21	1045.51
1066.73	1151.85	1171.91	1202.38	1213.67	1250.07
1280.17	1316.20	1324.96	1380.19	1387.38	1398.21
1401.39	1458.48	1475.59	1671.97	1862.83	1869.40
3041.09	3065.80	3073.48	3101.37	3129.95	3554.50
3633.97	3840.39	3848.65			

- Conformer (180); ZPE = 33670.335 cm^{-1}:

34.89	41.62	63.06	108.36	165.15	232.50
265.96	308.87	353.97	397.41	459.15	536.16
563.58	587.52	608.14	643.62	704.49	767.26
813.52	829.56	845.68	921.80	963.23	1036.61
1060.71	1112.14	1153.96	1175.62	1230.43	1237.27
1277.71	1314.82	1339.36	1372.59	1379.64	1399.41
1429.49	1458.82	1479.00	1651.11	1859.39	1873.49
3041.00	3057.92	3074.39	3096.06	3119.76	3552.94
3648.34	3844.38	3848.81			

- Conformer (181); ZPE = 33700.667 cm^{-1}:

42.09	44.57	86.59	101.89	175.13	234.79
284.19	330.06	356.21	402.13	455.66	489.37
560.57	574.24	615.20	634.47	727.03	748.34
812.64	844.08	882.66	930.82	968.18	1053.63
1069.34	1138.18	1172.24	1182.13	1210.81	1246.86
1256.40	1320.66	1321.06	1352.86	1380.13	1415.61
1443.96	1472.05	1475.52	1665.58	1873.02	1876.29
2929.36	3057.28	3066.45	3110.57	3122.45	3556.60
3640.65	3841.59	3849.18			

- Conformer (182); ZPE = 33779.688 cm^{-1}:

33.82	69.87	91.25	125.29	196.61	212.46
253.36	294.04	327.10	411.72	519.80	521.40
540.76	590.80	626.42	667.60	689.07	750.45
807.63	851.36	888.97	932.86	999.90	1010.05
1095.14	1151.66	1179.60	1197.38	1215.04	1236.45
1310.58	1317.40	1334.33	1370.92	1382.83	1397.60
1428.49	1448.54	1485.28	1671.57	1882.65	1898.63
3024.00	3059.25	3062.17	3102.18	3109.52	3536.45
3617.29	3801.04	3830.81			

- Conformer (183); ZPE = 33599.097 cm^{-1}:

31.70	42.34	45.96	104.33	178.32	232.09
245.72	304.08	325.04	370.26	502.42	514.37
536.30	582.71	625.58	637.10	665.85	776.33
788.49	808.70	857.18	918.25	974.17	1012.32
1088.28	1118.41	1150.14	1179.08	1221.21	1229.38
1270.38	1312.07	1334.28	1368.99	1380.55	1421.30
1435.45	1458.48	1469.08	1644.94	1865.56	1882.61
3039.84	3056.21	3087.84	3096.11	3106.73	3552.65
3652.33	3845.12	3851.57			

- Conformer (184); ZPE = 33884.811 cm^{-1}:

65.33	86.93	110.12	139.38	202.35	240.52
254.78	299.93	323.61	408.16	498.86	515.02
525.86	582.81	645.06	664.57	739.93	769.00
835.13	870.86	905.34	952.06	1009.17	1017.41
1096.30	1164.07	1176.96	1208.43	1222.86	1235.83
1303.78	1321.59	1323.89	1388.80	1402.02	1410.20
1431.96	1452.25	1476.20	1672.01	1859.65	1890.28
3029.52	3036.86	3061.20	3079.60	3113.04	3533.52
3612.52	3711.47	3892.63			

- Conformer (185); ZPE = 33648.279 cm^{-1}:

27.49	42.66	62.24	99.39	149.45	188.25
263.92	303.20	323.40	348.42	435.83	505.85
586.96	603.29	622.33	649.10	713.13	763.65
806.71	839.56	870.65	909.75	1017.81	1061.81
1082.54	1146.59	1157.20	1173.77	1227.68	1242.99
1274.96	1302.03	1341.47	1352.82	1376.47	1395.66
1458.04	1463.63	1486.70	1642.63	1868.15	1873.84
3034.55	3046.52	3051.75	3094.64	3138.09	3544.21
3637.84	3837.94	3848.99			

- Conformer (186); ZPE = 33895.028 cm^{-1}:

48.56	95.68	119.41	124.87	200.65	232.32
265.52	272.06	353.88	394.82	454.31	555.76
574.25	612.76	664.77	708.07	745.80	764.16
815.69	832.43	900.23	944.52	948.52	1020.44
1069.01	1149.33	1174.19	1197.41	1220.52	1279.74
1306.61	1311.68	1327.79	1387.35	1395.64	1407.07
1419.31	1478.38	1485.14	1661.10	1827.40	1890.64
3030.79	3033.24	3068.48	3120.26	3140.02	3550.79
3639.13	3731.74	3837.84			

- Conformer (187); ZPE = 33655.092 cm^{-1}:

25.34	50.34	56.06	133.42	135.21	214.70
285.00	289.05	310.39	381.31	458.38	525.28
569.57	590.95	617.12	627.21	703.28	760.89
770.27	833.82	890.74	916.14	1027.09	1064.09
1074.83	1140.43	1165.94	1189.50	1231.62	1250.70
1276.41	1305.75	1332.50	1351.46	1389.31	1410.59
1456.85	1461.89	1476.31	1652.10	1864.00	1874.05
2940.54	3041.70	3061.52	3105.97	3123.19	3556.63
3645.28	3846.84	3848.59			

- Conformer (188); ZPE = 33726.335 cm^{-1}:

30.81	43.32	71.00	109.16	174.22	192.21
260.34	328.69	341.57	395.65	443.19	538.17
580.28	598.35	626.79	650.54	751.05	783.00
799.24	819.84	864.01	922.20	980.46	1009.34
1078.34	1119.66	1132.15	1192.13	1207.94	1251.64
1267.65	1318.65	1330.66	1369.23	1374.58	1401.22
1425.76	1473.45	1483.71	1640.61	1873.16	1880.98
3040.29	3074.27	3079.27	3098.78	3138.44	3551.65
3648.32	3836.29	3850.36			

- Conformer (189); ZPE = 33667.943 cm^{-1}:

18.44	26.35	58.97	81.35	150.97	200.14
240.63	272.29	332.94	437.53	439.65	542.01
565.47	591.93	619.99	668.29	701.16	771.35
784.07	831.46	891.25	916.98	947.56	1046.02
1067.56	1152.78	1160.14	1203.73	1230.22	1242.00
1290.90	1306.39	1332.27	1380.67	1388.61	1395.05
1403.64	1456.27	1475.73	1672.81	1863.26	1868.36
3030.85	3065.84	3074.19	3116.09	3132.97	3559.34
3638.41	3839.55	3851.47			

- Conformer (190); ZPE = 33570.772 cm^{-1}:

21.26	48.14	66.92	102.72	110.80	201.50
244.04	249.85	322.18	390.29	435.51	522.30
573.88	599.04	615.70	657.59	713.30	771.61
782.54	811.41	866.65	923.25	963.30	1062.59
1076.71	1148.20	1155.69	1174.93	1226.23	1251.29
1265.98	1315.81	1321.42	1361.04	1375.82	1397.78
1452.49	1460.48	1481.26	1633.02	1869.79	1873.06
3031.42	3040.29	3051.10	3093.28	3124.90	3554.06
3654.42	3845.81	3848.90			

- Conformer (191); ZPE = 33606.109 cm^{-1}:

16.78	39.44	58.43	102.38	131.49	192.10
232.59	300.70	321.64	345.32	435.02	506.79
590.43	610.46	626.31	648.25	716.10	759.40
798.20	846.24	863.25	906.48	1032.26	1063.31
1066.52	1148.73	1158.05	1172.49	1229.06	1234.73
1279.99	1300.12	1337.35	1353.69	1373.64	1396.89
1456.38	1464.45	1482.73	1641.81	1869.53	1871.89
3034.49	3048.18	3059.91	3091.15	3127.03	3544.68
3638.03	3835.73	3851.57			

- Conformer (192); ZPE = 33600.822 cm^{-1}:

38.09	47.45	51.00	106.69	186.13	196.69
245.43	292.64	312.02	388.75	477.56	486.69
533.49	566.56	594.00	645.44	663.28	750.05
809.30	864.12	883.87	960.39	994.38	1001.55
1080.44	1141.66	1164.92	1190.61	1198.23	1245.36
1276.92	1300.98	1327.84	1363.26	1383.81	1407.09
1436.10	1446.10	1464.72	1670.11	1858.97	1892.40
3038.21	3044.21	3073.85	3085.79	3114.83	3537.08
3623.20	3844.83	3894.57			

- Conformer (193); ZPE = 33593.551 cm^{-1}:

21.08	47.95	67.11	100.53	116.98	214.05
244.73	247.72	323.95	392.55	434.73	519.20
571.70	597.53	617.94	659.51	711.72	765.18
793.61	824.89	872.40	925.04	951.96	1069.66
1078.85	1138.77	1161.95	1179.19	1227.90	1246.75
1276.63	1304.55	1326.29	1364.09	1379.80	1396.29
1453.17	1461.09	1482.03	1635.95	1868.87	1872.90
3023.60	3037.52	3050.01	3098.29	3128.46	3554.51
3653.58	3846.17	3848.19			

- Conformer (194); ZPE = 33769.425 cm^{-1}:

37.79	62.48	77.37	123.78	190.89	268.30
295.74	354.67	386.55	410.95	474.52	505.23
553.36	572.13	592.31	654.81	655.00	757.83
817.81	846.23	907.69	928.75	964.42	1016.40
1067.60	1146.83	1170.96	1190.39	1195.13	1235.50
1267.67	1311.99	1335.69	1361.69	1386.03	1411.66
1450.93	1455.37	1465.18	1646.18	1866.87	1882.12
2956.24	3044.29	3070.73	3091.24	3126.64	3549.16
3644.07	3847.61	3906.05			

- Conformer (195); ZPE = 33702.623 cm^{-1}:

42.69	48.65	56.72	129.96	179.59	255.32
297.00	318.22	321.77	388.83	471.48	492.07
540.10	569.66	586.47	648.63	663.49	745.96
829.58	861.94	895.92	956.26	992.48	1011.82
1069.87	1138.31	1163.40	1198.88	1207.13	1242.74
1290.88	1311.94	1328.77	1359.93	1392.02	1412.51
1413.64	1446.87	1476.22	1667.19	1869.95	1886.86
3012.37	3037.40	3056.37	3087.38	3123.00	3533.97
3620.82	3845.37	3906.84			

- Conformer (196); ZPE = 33858.655 cm^{-1}:

59.95	71.05	86.46	140.36	161.39	231.26
302.82	322.71	383.59	398.59	450.28	546.05
563.44	572.87	592.34	732.45	768.78	775.00
817.96	853.00	861.42	917.77	1007.50	1032.11
1093.78	1123.64	1161.24	1189.98	1227.16	1238.96
1271.92	1317.05	1338.76	1374.93	1397.69	1409.54
1433.13	1468.60	1477.07	1657.92	1881.37	1893.21
3031.98	3047.94	3056.27	3100.25	3132.77	3541.11
3636.22	3722.33	3843.34			

- Conformer (197); ZPE = 33537.834 cm^{-1}:

30.96	41.23	74.17	94.30	111.69	213.41
240.14	264.62	314.09	385.34	461.75	522.23
545.81	587.25	627.94	647.38	651.27	774.49
791.66	826.60	859.29	924.75	954.72	1075.48
1091.49	1110.20	1158.87	1167.94	1206.07	1253.13
1266.32	1312.13	1326.83	1368.86	1369.48	1425.68
1435.26	1455.94	1480.38	1646.64	1867.53	1878.17
3039.16	3047.80	3069.89	3079.67	3101.07	3552.13
3647.92	3847.89	3848.66			

- Conformer (198); ZPE = 33884.375 cm^{-1}:

57.53	95.65	104.64	126.46	194.48	257.96
278.49	291.39	321.95	395.97	464.73	512.02
576.76	624.69	627.65	720.30	750.05	768.10
789.97	850.52	896.24	936.14	1016.09	1051.54
1062.29	1138.89	1173.75	1211.09	1222.29	1274.40
1296.86	1308.36	1339.62	1351.12	1396.21	1407.60
1461.14	1477.96	1488.93	1653.67	1842.54	1889.43
2941.94	3045.47	3058.58	3102.21	3135.12	3559.54
3648.13	3734.08	3838.23			

- Conformer (199); ZPE = 33697.837 cm^{-1}:

35.84	45.67	72.96	110.51	175.66	270.58
291.49	342.84	392.50	436.92	470.43	497.02
539.05	570.96	589.81	642.31	676.60	746.52
804.10	849.05	877.21	925.29	969.96	1009.73
1063.87	1143.04	1169.67	1183.41	1207.75	1237.60
1251.01	1314.95	1339.29	1346.50	1381.74	1407.60
1444.34	1450.86	1482.36	1628.45	1865.35	1880.29
2961.29	3030.64	3070.70	3087.01	3128.86	3559.94
3657.47	3848.53	3910.13			

- Conformer (200); ZPE = 33695.211 cm^{-1}:

54.86	77.09	95.79	120.04	176.43	208.49
256.21	309.91	360.45	422.85	447.91	534.66
561.63	563.86	589.89	638.79	713.46	775.79
785.97	833.13	861.18	916.80	951.46	1004.08
1088.97	1114.72	1153.05	1191.51	1227.55	1240.85
1257.36	1311.57	1338.26	1368.63	1380.06	1402.33
1444.24	1461.42	1475.66	1648.27	1856.06	1860.47
3052.12	3062.02	3066.37	3106.60	3120.12	3560.20
3653.28	3835.77	3852.25			

- Conformer (201); ZPE = 33632.589 cm^{-1}:

16.17	51.62	56.71	131.64	137.88	215.42
284.06	290.09	307.47	381.77	466.54	518.29
569.67	585.86	615.98	623.94	690.03	759.51
779.47	828.55	892.72	907.51	1019.51	1070.86
1078.93	1137.43	1165.22	1198.21	1212.42	1258.65
1275.63	1307.20	1328.48	1351.67	1385.90	1411.33
1456.64	1457.72	1475.87	1651.37	1869.45	1875.51
2937.98	3033.28	3058.36	3105.55	3134.41	3556.70
3645.71	3846.69	3847.60			

- Conformer (202); ZPE = 33685.797 cm^{-1}:

26.07	57.56	72.70	113.70	153.25	228.53
253.70	298.80	305.10	400.81	447.68	522.11
570.19	582.71	604.33	611.78	736.81	768.88
807.35	839.62	874.81	926.96	993.21	1032.08
1058.18	1119.56	1151.54	1186.36	1222.22	1246.78
1277.02	1323.30	1344.29	1370.40	1387.09	1392.55
1408.63	1467.11	1481.68	1656.28	1863.95	1879.17
3057.13	3063.32	3078.78	3116.24	3121.19	3542.10
3632.25	3840.63	3855.07			

- Conformer (203); ZPE = 33601.808 cm^{-1}:

25.29	39.36	75.72	96.19	100.23	232.08
234.93	272.15	337.14	411.89	433.74	515.32
574.21	589.21	619.92	639.69	715.80	753.77
777.28	848.00	885.12	923.94	970.34	1068.09
1080.06	1161.47	1162.99	1181.88	1229.52	1247.94
1269.93	1304.06	1331.60	1357.44	1383.47	1402.63
1446.19	1462.46	1476.98	1657.40	1866.99	1875.54
2930.36	3042.02	3067.00	3101.76	3123.02	3561.93
3644.13	3847.13	3848.31			

- Conformer (204); ZPE = 33617.335 cm^{-1}:

20.64	43.36	47.47	118.97	145.18	209.81
252.65	296.79	336.08	385.93	449.22	514.19
572.41	594.78	621.12	640.85	712.11	739.20
779.47	824.88	875.33	908.47	1016.71	1065.70
1078.94	1132.04	1172.57	1195.91	1223.57	1249.75
1269.59	1296.60	1333.27	1356.62	1385.13	1398.95
1446.10	1467.87	1485.53	1638.09	1865.06	1869.80
2926.36	3039.46	3056.00	3103.87	3159.57	3563.38
3658.69	3842.16	3848.48			

- Conformer (205); ZPE = 33715.727 cm^{-1}:

39.61	59.29	88.65	95.78	150.42	186.67
234.88	295.18	330.67	395.70	440.71	506.80
572.10	593.50	602.64	650.81	739.45	766.20
823.33	859.00	892.54	937.84	997.70	1027.82
1073.94	1142.83	1166.80	1192.92	1207.01	1242.88
1285.18	1302.59	1329.04	1365.41	1387.38	1409.71
1426.88	1472.33	1479.77	1670.72	1866.91	1871.50
3041.99	3052.33	3071.01	3109.77	3135.87	3534.47
3614.24	3844.22	3846.47			

- Conformer (206); ZPE = 33719.403 cm^{-1}:

32.00	43.30	65.92	115.79	159.46	229.53
249.70	265.43	296.41	431.92	446.06	485.80
580.60	602.86	623.18	644.79	756.28	795.51
799.67	833.97	856.08	920.93	1006.21	1028.75
1112.91	1121.70	1155.75	1177.45	1193.71	1245.04
1267.90	1313.52	1332.98	1352.00	1395.58	1403.48
1454.29	1470.87	1487.79	1651.18	1859.67	1866.64
3045.20	3065.14	3085.94	3101.70	3147.09	3541.49
3635.19	3839.49	3844.92			

- Conformer (207); ZPE = 33626.306 cm^{-1}:

25.52	42.35	72.85	90.31	113.83	231.29
253.20	272.81	337.94	401.76	446.89	507.15
573.35	584.90	614.74	638.19	718.47	745.34
792.32	843.82	893.12	923.71	964.98	1068.27
1079.37	1148.19	1173.89	1182.28	1224.77	1258.40
1269.49	1307.44	1330.17	1361.53	1381.70	1402.46
1448.54	1463.31	1480.35	1657.56	1870.14	1876.61
2928.06	3032.93	3064.30	3114.94	3132.82	3563.60
3646.75	3846.03	3849.88			

- Conformer (208); ZPE = 33750.501 cm^{-1}:

46.13	72.00	76.19	128.40	169.81	197.58
265.54	302.18	318.39	406.62	467.97	521.54
570.85	581.06	602.07	659.92	759.59	778.29
817.40	842.84	886.73	909.94	1001.02	1026.90
1062.98	1124.51	1137.72	1186.40	1211.22	1246.36
1269.60	1325.53	1339.07	1364.99	1384.91	1400.66
1411.59	1472.09	1486.37	1653.57	1862.67	1877.48
3054.99	3064.07	3066.22	3109.71	3138.81	3531.66
3621.36	3841.41	3846.06			

- Conformer (209); ZPE = 33641.616 cm^{-1}:

29.54	33.21	50.02	116.06	172.99	246.52
283.41	302.87	312.82	346.77	506.49	518.74
574.79	590.26	604.03	616.30	683.63	780.17
790.11	825.60	849.86	902.39	975.64	1053.09
1089.94	1119.81	1158.75	1179.48	1225.65	1238.02
1268.81	1326.95	1361.23	1368.61	1371.90	1393.38
1439.53	1450.63	1476.45	1648.56	1867.94	1875.18
3039.78	3060.33	3081.02	3095.12	3109.26	3544.62
3634.79	3842.20	3850.00			

- Conformer (210); ZPE = 33798.374 cm^{-1}:

28.58	46.60	69.88	143.43	181.71	253.41
275.93	297.84	366.22	409.64	486.21	508.97
548.94	567.63	630.67	641.36	753.39	827.88
843.60	857.35	884.44	951.26	976.68	1023.52
1078.01	1147.76	1162.95	1196.70	1242.08	1255.59
1294.67	1298.93	1329.41	1379.54	1394.36	1413.17
1443.67	1450.73	1470.92	1665.74	1882.07	1885.82
3023.20	3026.88	3052.06	3069.87	3119.92	3555.40
3633.84	3653.74	3894.58			

- Conformer (211); ZPE = 33665.949 cm^{-1}:

50.91	70.27	86.25	118.85	177.90	219.96
271.38	299.93	341.52	388.63	486.95	535.08
554.23	580.49	595.01	650.79	701.64	765.58
778.59	830.25	855.92	929.35	951.06	978.53
1102.52	1103.64	1142.33	1195.90	1216.33	1242.83
1247.04	1329.19	1340.42	1360.84	1390.26	1396.43
1435.19	1448.09	1474.76	1646.57	1866.54	1886.38
3046.28	3058.34	3080.46	3099.60	3111.08	3552.36
3644.36	3846.07	3849.02			

- Conformer (212); ZPE = 33660.510 cm^{-1}:

49.16	68.37	89.00	128.54	162.07	247.61
257.00	304.91	318.53	347.34	507.90	514.21
558.64	579.79	589.11	618.62	682.93	766.54
787.10	842.61	847.15	908.08	976.20	1070.62
1075.96	1117.50	1164.18	1180.87	1223.03	1250.47
1258.14	1332.79	1344.27	1371.31	1374.97	1394.68
1441.49	1444.53	1479.35	1651.34	1864.11	1877.96
3045.69	3056.83	3059.99	3097.33	3108.35	3549.30
3638.62	3843.78	3852.15			

- Conformer (213); ZPE = 33626.564 cm^{-1}:

23.42	41.69	49.80	106.55	130.85	202.52
258.70	299.13	319.69	353.61	433.17	529.28
590.31	593.85	611.54	621.69	727.37	764.01
800.36	849.07	855.50	908.45	1025.05	1053.83
1067.51	1111.12	1149.81	1177.62	1230.11	1244.53
1281.59	1303.75	1347.40	1369.11	1372.17	1397.67
1435.95	1463.67	1485.37	1651.21	1869.86	1871.81
3034.56	3060.55	3070.38	3090.43	3130.90	3548.72
3638.81	3847.64	3851.44			

- Conformer (214); ZPE = 33628.642 cm^{-1}:

27.66	38.95	55.96	94.38	145.48	190.20
250.80	295.74	321.85	353.08	433.76	528.17
591.60	595.03	612.08	621.90	725.58	768.41
809.06	840.19	860.31	911.47	1009.61	1055.57
1083.66	1110.04	1143.13	1178.34	1229.17	1251.37
1274.08	1306.36	1353.53	1368.61	1370.76	1396.61
1434.86	1462.54	1485.49	1652.53	1872.16	1875.39
3033.64	3056.30	3071.20	3092.12	3134.78	3549.24
3638.81	3847.67	3848.03			

- Conformer (215); ZPE = 33740.768 cm^{-1}:

47.92	69.69	77.18	140.50	169.13	214.81
280.85	294.65	330.74	405.36	462.85	496.49
575.57	582.48	600.01	628.13	752.15	763.60
804.74	839.01	888.88	915.58	984.90	1050.72
1071.73	1133.52	1153.64	1183.35	1220.31	1249.35
1270.83	1326.65	1330.08	1338.52	1390.61	1408.70
1456.22	1468.54	1476.29	1654.62	1872.94	1883.82
2964.02	3057.56	3058.17	3112.22	3136.30	3553.68
3641.24	3842.80	3849.89			

- Conformer (216); ZPE = 33659.950 cm^{-1}:

39.23	64.69	106.22	124.74	164.32	173.16
256.50	293.32	346.68	407.86	476.32	492.23
525.01	558.31	598.96	643.50	670.91	778.75
802.18	863.50	890.41	932.88	973.66	1006.55
1092.72	1145.84	1160.45	1186.09	1195.11	1233.25
1264.21	1300.92	1315.67	1359.79	1382.01	1408.16
1450.35	1456.90	1466.86	1665.01	1847.33	1888.28
3038.26	3066.80	3080.79	3085.38	3126.46	3537.53
3632.67	3839.57	3903.59			

- Conformer (217); ZPE = 33602.566 cm^{-1}:

23.99	41.67	66.32	83.77	160.60	186.88
228.91	290.46	324.24	419.86	439.61	553.60
565.22	584.72	610.72	660.48	710.04	757.47
797.56	807.08	850.36	926.42	954.26	1026.52
1083.53	1115.91	1150.96	1203.76	1234.85	1247.24
1254.74	1314.75	1327.61	1364.44	1388.46	1395.46
1436.97	1457.08	1480.29	1648.26	1868.99	1874.83
3041.22	3050.87	3070.00	3097.62	3125.75	3557.43
3650.45	3843.24	3849.65			

- Conformer (218); ZPE = 33819.686 cm^{-1}:

36.54	79.34	114.29	150.73	174.22	250.34
259.16	308.16	321.29	388.82	450.88	524.25
580.97	618.39	656.99	683.22	753.01	771.21
803.89	833.87	869.43	901.60	1000.20	1033.07
1091.17	1130.96	1168.18	1190.28	1203.87	1249.76
1268.66	1315.99	1335.63	1358.14	1369.27	1390.79
1446.13	1475.51	1487.34	1638.66	1883.34	1892.98
3015.50	3040.26	3075.48	3096.34	3138.51	3542.76
3637.57	3801.24	3831.17			

- Conformer (219); ZPE = 33943.194 cm^{-1}:

22.58	50.21	81.68	172.22	199.74	245.91
257.30	329.04	346.00	402.39	446.71	542.37
602.48	634.91	663.84	741.34	766.94	797.45
823.29	857.89	897.51	928.38	1011.44	1030.58
1067.75	1133.56	1153.30	1199.94	1253.51	1274.52
1298.27	1326.64	1344.27	1356.44	1380.40	1407.76
1442.43	1475.96	1497.62	1656.85	1851.61	1881.99
3033.58	3041.22	3075.38	3090.61	3132.01	3562.32
3647.44	3662.64	3786.17			

- Conformer (220); ZPE = 33780.511 cm^{-1}:

42.44	67.29	107.74	147.95	182.04	190.98
247.34	285.30	304.46	394.18	462.21	520.33
600.97	602.75	644.28	673.98	744.18	764.24
802.59	855.42	865.94	918.83	980.33	1051.95
1063.86	1147.65	1154.96	1164.64	1191.47	1260.60
1294.89	1304.35	1337.26	1355.86	1370.23	1395.74
1428.00	1477.31	1486.37	1666.68	1880.06	1894.08
3041.12	3059.13	3068.01	3109.32	3133.78	3540.94
3625.82	3821.87	3829.32			

- Conformer (221); ZPE = 33848.945 cm^{-1}:

33.70	60.50	84.85	118.24	181.37	210.57
332.35	364.62	390.39	415.63	451.69	493.98
530.86	596.11	619.08	738.02	748.99	803.71
812.07	864.77	882.82	956.79	975.43	1009.91
1083.90	1121.06	1160.00	1202.88	1234.60	1246.64
1263.59	1308.71	1336.66	1357.66	1391.49	1398.86
1435.14	1476.20	1487.07	1660.79	1878.53	1889.01
3022.70	3047.38	3061.65	3095.36	3128.48	3531.91
3616.93	3691.97	3892.29			

- Conformer (222); ZPE = 33609.004 cm^{-1}:

17.20	41.17	59.61	83.65	154.92	186.71
230.82	287.71	324.60	425.62	431.65	550.35
570.71	589.05	616.73	662.35	714.36	765.66
789.52	822.94	852.23	926.62	945.88	1034.20
1083.69	1116.31	1150.52	1208.27	1236.77	1240.82
1262.33	1308.09	1327.79	1363.97	1387.55	1398.93
1438.19	1457.26	1477.51	1649.54	1868.58	1871.03
3036.39	3048.82	3068.72	3102.55	3126.72	3557.57
3649.25	3844.84	3851.71			

- Conformer (223); ZPE = 33724.269 cm^{-1}:

61.88	78.11	104.36	131.64	181.85	206.76
224.31	305.71	373.96	438.11	465.37	517.18
551.85	595.20	605.50	637.27	732.85	771.61
799.96	844.40	871.72	922.70	974.02	996.35
1079.95	1127.93	1148.47	1183.91	1216.87	1238.45
1255.14	1315.38	1324.72	1341.88	1367.65	1383.69
1452.91	1477.01	1486.07	1635.97	1845.30	1890.24
2998.64	3053.68	3063.28	3116.50	3118.23	3567.64
3663.71	3833.98	3868.63			

- Conformer (224); ZPE = 33688.235 cm^{-1}:

46.79	52.75	58.70	138.56	180.19	249.88
291.35	316.93	321.74	385.65	469.90	477.87
524.34	540.58	623.12	649.38	681.92	747.16
821.37	858.10	904.71	949.62	993.79	1016.70
1073.05	1142.87	1158.76	1197.27	1215.46	1238.56
1283.11	1312.81	1334.76	1347.14	1388.43	1411.84
1438.14	1452.05	1471.19	1668.61	1857.23	1902.05
2991.09	3044.31	3052.00	3093.43	3109.18	3534.59
3619.37	3847.68	3890.33			

- Conformer (225); ZPE = 33833.391 cm^{-1}:

58.28	77.45	104.52	115.13	178.19	229.98
288.22	331.30	365.72	442.19	465.47	481.75
538.72	565.84	598.97	625.56	747.06	752.81
798.63	863.46	894.23	943.82	980.60	1011.08
1079.40	1136.65	1164.44	1199.52	1225.45	1249.33
1268.34	1320.44	1329.12	1366.42	1390.99	1400.76
1415.61	1467.74	1489.86	1674.64	1847.96	1866.47
3030.83	3058.14	3069.67	3113.21	3125.31	3530.17
3626.67	3850.71	3909.97			

- Conformer (226); ZPE = 33707.574 cm^{-1}:

21.48	38.02	61.12	137.16	185.66	261.85
304.42	330.37	362.44	398.86	471.74	493.00
513.23	575.44	588.16	641.43	659.10	747.09
820.49	858.48	887.74	951.75	982.63	1014.59
1071.09	1136.18	1181.58	1191.47	1197.50	1249.22
1294.36	1314.24	1320.57	1364.80	1396.21	1404.00
1417.10	1447.76	1475.30	1661.34	1863.05	1885.74
2993.78	3033.61	3066.19	3083.26	3129.30	3541.19
3638.66	3846.56	3904.84			

- Conformer (227); ZPE = 33654.368 cm^{-1}:

47.86	63.86	90.55	118.97	187.66	253.02
268.64	286.17	325.21	348.44	445.36	500.41
558.77	572.99	585.50	609.62	655.19	768.77
790.22	849.55	882.01	918.91	1002.33	1033.46
1080.03	1137.21	1173.51	1198.99	1210.04	1247.94
1281.76	1308.17	1337.91	1362.06	1392.54	1410.31
1415.06	1446.90	1472.10	1666.11	1841.89	1886.38
3038.71	3050.42	3061.31	3086.58	3117.93	3546.73
3630.87	3844.16	3899.61			

- Conformer (228); ZPE = 33590.197 cm^{-1}:

23.96	33.73	69.46	94.22	110.12	219.06
241.60	251.49	311.56	385.79	437.41	548.44
575.65	597.07	620.99	642.19	716.95	769.57
796.67	816.98	859.10	921.61	962.83	1062.53
1078.77	1108.45	1149.09	1191.02	1233.54	1254.41
1260.36	1320.22	1332.00	1368.83	1379.43	1397.63
1429.25	1464.26	1484.16	1644.22	1870.63	1877.75
3035.46	3048.32	3071.67	3092.10	3122.16	3553.75
3649.80	3846.24	3847.89			

- Conformer (229); ZPE = 33757.037 cm^{-1}:

31.57	74.68	82.29	144.40	171.02	213.33
279.59	302.66	338.38	404.10	468.87	515.77
556.42	579.35	599.73	651.75	754.11	770.93
803.93	835.96	888.44	916.38	981.15	1046.33
1074.54	1130.17	1151.39	1196.28	1228.52	1245.17
1265.86	1325.66	1334.23	1355.57	1394.95	1409.47
1437.20	1472.24	1484.84	1645.83	1866.68	1877.04
2973.74	3056.45	3058.59	3116.44	3135.86	3544.39
3632.68	3843.24	3845.91			

- Conformer (230); ZPE = 33598.617 cm^{-1}:

23.49	38.09	64.61	97.06	109.84	231.03
240.24	257.18	315.02	381.12	433.91	541.04
575.72	592.47	617.46	641.98	719.12	782.30
793.27	834.76	861.10	927.32	951.00	1063.56
1077.13	1109.76	1134.15	1198.70	1226.51	1249.13
1272.38	1305.05	1339.71	1368.79	1379.14	1396.83
1429.29	1463.64	1483.40	1647.80	1870.22	1879.55
3030.83	3049.76	3071.34	3093.85	3130.61	3552.20
3646.54	3848.37	3849.85			

- Conformer (231); ZPE = 33603.457 cm^{-1}:

41.46	53.14	62.16	115.59	164.10	209.19
249.41	292.49	355.39	448.78	486.94	490.38
509.25	578.64	587.58	642.51	675.31	762.60
789.78	837.17	872.92	938.48	965.77	1029.54
1099.15	1142.58	1162.04	1180.83	1214.08	1223.81
1280.12	1309.43	1324.34	1343.25	1363.45	1414.96
1445.71	1459.83	1478.36	1635.96	1864.45	1897.32
2968.72	3021.40	3067.69	3071.36	3121.94	3560.90
3655.49	3845.18	3895.98			

- Conformer (232); ZPE = 33923.257 cm^{-1}:

51.78	67.67	95.35	133.76	197.61	266.98
293.35	333.78	361.78	419.32	539.13	544.05
557.29	602.28	615.69	754.81	757.49	826.01
830.95	855.77	861.40	921.04	993.34	1024.89
1108.85	1145.22	1166.14	1220.71	1248.55	1262.52
1292.20	1297.21	1338.31	1377.47	1391.56	1404.97
1447.34	1455.76	1490.24	1659.31	1863.12	1871.97
3023.27	3052.35	3059.12	3093.72	3106.62	3559.94
3651.63	3665.29	3687.60			

- Conformer (233); ZPE = 33903.689 cm^{-1}:

42.39	83.47	132.95	166.70	180.50	207.89
242.45	301.34	320.26	384.60	471.94	518.86
545.65	609.23	662.19	737.98	766.85	805.21
824.67	867.44	882.23	937.65	987.09	1061.76
1067.18	1153.58	1167.10	1185.72	1220.10	1248.33
1295.32	1327.95	1344.31	1353.93	1400.77	1408.61
1439.19	1476.64	1494.84	1671.34	1850.26	1878.50
3046.46	3056.35	3065.49	3098.07	3110.77	3531.19
3616.90	3653.82	3903.39			

- Conformer (234); ZPE = 33703.154 cm^{-1}:

41.09	54.60	80.75	120.20	165.80	228.93
248.22	313.02	359.47	433.65	490.12	495.88
562.52	575.35	607.15	649.40	738.43	765.01
776.10	814.09	864.12	926.01	977.42	996.43
1088.76	1130.98	1161.06	1177.32	1218.43	1247.66
1263.21	1318.01	1337.68	1340.89	1378.50	1420.90
1436.27	1474.89	1485.64	1648.36	1852.31	1889.75
2989.20	3052.69	3075.39	3107.98	3137.34	3547.43
3648.43	3844.02	3849.45			

- Conformer (235); ZPE = 33548.857 cm^{-1}:

37.51	45.57	67.97	102.27	120.46	211.47
230.54	262.59	300.49	394.05	473.38	483.92
514.92	576.83	590.09	642.05	674.98	764.11
796.59	859.31	908.36	960.64	979.63	1024.70
1101.07	1145.90	1153.87	1171.20	1195.09	1258.26
1278.63	1296.77	1334.69	1344.75	1376.57	1412.46
1423.08	1460.88	1480.98	1669.27	1867.34	1897.86
3027.27	3038.05	3055.03	3076.66	3116.62	3536.91
3618.35	3843.52	3894.19			

- Conformer (236); ZPE = 33717.234 cm^{-1}:

30.96	51.02	82.71	110.13	136.07	199.06
265.65	326.38	341.64	357.64	476.70	485.43
565.59	578.87	643.35	666.64	746.53	807.67
811.88	847.53	901.17	921.70	1005.68	1048.61
1090.55	1136.56	1155.83	1174.26	1237.89	1255.65
1289.88	1318.57	1331.02	1361.23	1373.48	1414.72
1435.19	1458.67	1487.24	1657.62	1882.78	1894.92
3031.22	3036.10	3046.80	3078.23	3097.73	3559.10
3643.86	3685.54	3891.21			

- Conformer (237); ZPE = 33676.576 cm^{-1}:

36.08	50.90	73.98	127.04	176.88	254.28
277.77	295.42	348.34	399.33	473.07	489.12
536.70	569.78	603.90	642.87	677.99	766.76
812.87	837.13	875.11	926.54	964.44	1017.40
1078.76	1155.50	1178.22	1182.40	1214.31	1241.53
1268.27	1306.47	1318.57	1343.39	1383.87	1413.16
1451.16	1459.65	1480.37	1648.15	1856.69	1888.96
3025.64	3044.03	3058.96	3075.65	3109.74	3553.32
3645.61	3844.43	3892.62			

- Conformer (238); ZPE = 33654.420 cm^{-1}:

57.25	66.00	76.00	136.57	196.10	222.36
265.52	288.40	323.68	343.14	472.16	493.68
559.51	566.64	593.74	636.08	655.70	756.60
781.02	861.25	898.12	932.54	1010.05	1036.26
1090.40	1139.04	1158.35	1179.17	1202.61	1246.18
1281.70	1305.40	1313.78	1367.52	1377.72	1412.25
1423.82	1449.58	1473.17	1671.19	1877.25	1897.52
3034.08	3036.00	3057.08	3080.98	3116.85	3538.44
3617.44	3833.31	3899.64			

- Conformer (239); ZPE = 33627.241 cm^{-1}:

33.34	46.98	51.91	97.22	189.69	244.65
281.58	298.60	315.37	370.91	479.22	498.50
519.37	569.50	580.41	629.33	659.75	767.83
806.78	857.10	879.51	959.66	994.25	1004.50
1078.13	1137.62	1156.98	1180.02	1197.74	1246.12
1289.95	1303.26	1324.70	1360.85	1404.21	1408.17
1423.17	1446.95	1463.99	1658.01	1866.04	1892.95
3037.13	3054.13	3067.99	3082.62	3120.07	3538.12
3631.65	3849.53	3898.44			

- Conformer (240); ZPE = 33728.042 cm^{-1}:

57.75	75.61	97.00	124.39	154.98	183.61
210.24	286.72	375.32	428.25	442.77	510.51
555.96	608.55	610.83	658.43	728.46	784.43
818.41	854.49	889.34	921.34	965.84	1015.05
1066.22	1133.36	1152.80	1189.88	1215.78	1238.71
1280.00	1300.15	1321.39	1359.01	1377.42	1390.83
1440.67	1470.67	1479.73	1672.80	1830.40	1887.32
3051.91	3068.63	3083.89	3118.49	3127.70	3545.08
3622.07	3824.57	3848.33			

- Conformer (241); ZPE = 33650.916 cm^{-1}:

43.06	50.45	72.67	110.28	179.65	264.52
279.84	330.51	385.91	429.47	490.58	509.88
526.15	558.10	608.27	620.49	677.65	743.88
787.40	833.55	871.96	925.64	970.89	1006.67
1073.40	1148.97	1178.34	1188.14	1205.22	1228.13
1256.09	1310.46	1321.49	1343.41	1384.55	1427.00
1447.11	1450.58	1480.70	1625.26	1850.96	1899.12
2934.59	3048.30	3053.32	3093.56	3104.03	3564.37
3665.63	3851.11	3890.50			

- Conformer (242); ZPE = 33550.292 cm^{-1}:

28.98	51.95	63.34	114.95	159.02	206.73
213.81	313.57	363.06	447.97	477.69	506.86
524.10	529.57	609.87	652.19	657.80	765.30
791.99	832.35	863.30	936.25	960.83	1031.52
1097.53	1143.22	1173.42	1182.62	1200.33	1221.66
1275.38	1303.79	1316.77	1331.37	1376.86	1427.20
1442.56	1457.10	1487.40	1633.14	1858.03	1894.82
2959.24	3034.81	3043.19	3086.63	3099.51	3558.52
3657.23	3848.56	3886.73			

- Conformer (243); ZPE = 33670.577 cm^{-1}:

41.93	51.83	75.94	127.00	177.71	247.72
299.34	308.96	355.37	402.61	486.58	493.68
502.82	555.11	625.03	652.16	673.84	762.87
820.45	825.27	880.57	925.93	963.15	1013.85
1081.48	1164.69	1173.70	1193.35	1213.17	1244.31
1267.52	1312.90	1316.34	1327.12	1387.08	1432.06
1447.90	1451.53	1474.18	1650.92	1850.45	1891.25
2978.52	3045.19	3048.78	3092.07	3095.60	3551.95
3645.69	3847.67	3886.04			

- Conformer (244); ZPE = 33821.342 cm^{-1}:

29.36	61.87	87.31	117.57	182.99	216.96
321.81	349.18	388.42	418.78	454.73	515.55
537.74	595.79	598.50	706.95	756.82	821.25
834.04	853.87	883.00	943.17	967.32	1042.35
1053.79	1112.34	1162.79	1191.80	1234.23	1246.58
1279.83	1291.02	1321.79	1366.54	1386.54	1394.89
1434.21	1472.57	1478.16	1663.64	1887.50	1890.74
3013.27	3037.82	3052.09	3113.80	3126.50	3546.03
3635.24	3674.80	3888.83			

- Conformer (245); ZPE = 33659.685 cm^{-1}:

44.39	57.02	59.65	104.08	197.96	276.90
288.20	293.29	328.32	371.65	486.32	509.88
534.09	573.33	585.53	608.29	649.82	752.11
794.99	845.58	897.51	917.22	1000.60	1046.48
1095.72	1145.80	1169.81	1183.46	1222.37	1224.15
1285.50	1306.03	1333.56	1343.69	1396.71	1408.75
1452.73	1459.33	1462.57	1653.30	1860.33	1889.51
2962.00	3041.54	3061.78	3086.22	3117.80	3552.98
3638.57	3848.42	3893.53			

- Conformer (246); ZPE = 33822.944 cm^{-1}:

49.22	91.39	99.02	128.20	192.15	210.27
253.96	281.91	326.24	385.15	457.43	504.89
587.40	619.77	626.82	719.37	752.79	774.05
796.65	811.46	876.80	938.85	997.97	1045.01
1111.18	1127.56	1169.67	1186.56	1223.89	1266.73
1299.75	1306.46	1328.71	1370.83	1392.55	1402.43
1459.27	1474.93	1487.06	1660.35	1825.32	1889.65
3038.89	3042.00	3053.51	3102.20	3139.59	3550.68
3646.26	3723.74	3839.37			

- Conformer (247); ZPE = 33924.013 cm^{-1}:

54.99	56.91	101.74	160.87	178.29	207.94
282.48	319.77	350.07	410.74	464.97	512.86
531.06	599.56	664.23	736.30	756.89	801.35
828.06	872.25	931.13	969.14	1005.89	1029.86
1051.60	1142.04	1170.56	1189.49	1218.06	1283.85
1296.40	1320.45	1348.08	1369.58	1386.19	1419.10
1437.67	1475.46	1484.38	1665.71	1885.10	1896.99
3012.75	3036.26	3067.75	3089.52	3137.31	3527.36
3599.56	3624.52	3884.91			

- Conformer (248); ZPE = 33793.432 cm^{-1}:

46.71	69.47	108.83	144.54	179.27	238.12
253.80	297.22	307.90	392.56	463.58	517.73
599.74	604.91	641.10	668.92	744.51	768.70
802.53	834.74	872.36	895.67	1005.02	1032.31
1086.89	1134.75	1156.07	1170.45	1200.03	1247.61
1280.05	1320.13	1337.79	1342.20	1368.28	1394.02
1454.39	1476.44	1487.89	1640.38	1884.44	1893.58
3036.29	3047.10	3068.37	3094.22	3132.49	3546.26
3641.19	3823.73	3831.58			

- Conformer (249); ZPE = 33861.769 cm^{-1}:

41.99	94.93	121.62	126.97	193.39	211.33
233.11	286.73	357.68	397.10	456.85	552.72
578.59	611.13	670.44	712.13	754.96	769.47
794.65	830.97	869.70	943.80	964.79	1007.70
1104.84	1128.29	1181.67	1202.54	1225.71	1273.27
1294.51	1308.52	1326.01	1376.47	1389.85	1406.69
1458.62	1479.01	1487.76	1665.24	1820.24	1889.50
3035.22	3036.33	3057.16	3102.02	3137.09	3549.57
3641.95	3721.41	3841.31			

- Conformer (250); ZPE = 33570.769 cm^{-1}:

32.30	47.21	61.12	102.87	141.49	184.83
210.41	279.05	339.79	429.44	455.38	501.46
526.58	531.18	644.90	655.33	655.52	773.37
788.38	846.85	913.08	925.29	953.17	1056.51
1084.54	1160.61	1167.00	1177.62	1198.30	1243.79
1273.09	1297.34	1313.93	1348.46	1377.27	1425.04
1440.73	1455.64	1473.31	1675.60	1862.11	1890.81
3036.37	3039.69	3052.78	3089.58	3110.32	3537.39
3619.25	3848.71	3886.75			

- Conformer (251); ZPE = 33537.616 cm^{-1}:

29.77	51.92	71.51	109.60	117.02	196.62
228.67	262.17	309.05	397.37	463.60	497.18
515.53	520.84	629.02	650.28	680.19	749.79
790.49	863.88	908.65	959.95	978.41	1027.52
1098.13	1146.99	1160.13	1176.45	1194.91	1260.86
1270.83	1297.55	1332.31	1342.38	1379.84	1414.90
1437.54	1457.28	1478.57	1672.49	1862.41	1898.23
3008.32	3043.53	3053.94	3085.24	3107.44	3535.38
3616.10	3847.36	3887.10			

- Conformer (252); ZPE = 33710.912 cm^{-1}:

36.84	37.40	68.46	121.10	141.59	218.69
271.76	304.81	354.55	372.66	490.40	507.27
561.17	581.25	584.73	668.02	735.34	796.42
819.43	856.09	891.22	919.30	1019.96	1081.76
1088.99	1134.92	1161.99	1181.74	1212.03	1247.55
1283.50	1303.96	1332.59	1356.38	1393.31	1415.84
1436.49	1461.56	1477.57	1658.82	1880.26	1894.64
3025.47	3029.19	3046.50	3088.15	3107.07	3543.39
3636.97	3688.18	3894.56			

- Conformer (253); ZPE = 33747.402 cm^{-1}:

55.57	84.06	100.09	152.46	182.47	194.69
229.97	307.33	369.78	436.73	445.18	522.20
563.34	604.05	615.32	662.23	730.42	773.77
783.26	840.70	874.34	912.08	964.32	1000.69
1080.26	1130.77	1163.74	1177.36	1218.65	1251.45
1263.88	1311.13	1322.32	1349.05	1370.03	1390.25
1460.27	1474.77	1494.40	1637.83	1833.68	1887.00
3047.91	3050.24	3056.94	3105.52	3117.67	3560.57
3658.76	3830.30	3845.02			

- Conformer (254); ZPE = 33678.443 cm^{-1}:

36.39	44.31	53.96	134.38	209.90	243.90
262.13	289.39	327.01	345.08	467.15	483.41
561.07	578.90	610.32	636.76	663.26	770.40
790.66	852.36	876.83	932.25	974.76	1068.45
1081.21	1142.73	1162.25	1180.48	1211.77	1220.46
1288.38	1304.35	1319.94	1365.59	1371.11	1410.33
1442.74	1457.51	1468.69	1670.76	1863.03	1894.76
3038.53	3050.37	3078.69	3092.22	3118.36	3541.07
3627.67	3834.89	3906.00			

- Conformer (255); ZPE = 33786.000 cm^{-1}:

53.62	82.65	93.15	118.35	187.49	222.97
265.24	301.74	367.54	431.62	481.34	507.82
575.77	589.10	634.75	676.15	711.90	749.14
799.98	850.56	886.89	944.77	951.95	1009.51
1074.10	1126.28	1170.09	1202.21	1216.63	1253.92
1282.16	1307.20	1336.00	1346.91	1380.57	1394.80
1436.81	1474.77	1497.41	1640.38	1881.27	1899.07
2944.84	3030.26	3061.70	3102.44	3154.10	3560.37
3652.65	3818.97	3832.09			

- Conformer (256); ZPE = 33588.860 cm^{-1}:

36.47	48.44	60.82	121.63	165.88	209.16
261.06	281.89	332.48	413.60	472.79	486.57
556.46	563.43	579.75	634.33	684.29	756.55
788.97	830.12	891.93	942.03	972.22	1023.28
1092.80	1141.99	1164.41	1179.95	1208.22	1229.76
1284.42	1311.89	1338.18	1343.53	1364.18	1418.92
1450.09	1463.48	1473.89	1649.88	1868.52	1895.04
2950.28	3034.15	3064.10	3079.97	3116.13	3555.97
3644.11	3845.75	3893.96			

- Conformer (257); ZPE = 33744.184 cm^{-1}:

32.75	56.43	80.26	100.28	157.74	193.11
278.86	301.78	358.52	447.24	464.04	485.69
549.86	586.27	649.26	670.90	763.36	806.88
810.04	844.58	907.26	928.44	976.39	1030.36
1065.01	1136.55	1158.60	1182.35	1208.55	1268.22
1281.00	1314.38	1345.83	1358.44	1370.18	1414.92
1427.14	1458.74	1478.42	1656.61	1895.01	1897.01
3022.08	3052.42	3062.30	3066.25	3120.14	3551.83
3633.35	3689.26	3893.51			

- Conformer (258); ZPE = 33609.461 cm^{-1}:

38.17	45.94	54.08	115.97	197.45	252.37
265.70	276.72	303.77	362.43	469.21	494.43
541.16	574.13	592.17	622.10	660.52	770.68
799.92	840.81	863.47	929.82	977.11	1061.14
1081.96	1140.80	1153.28	1178.17	1204.29	1224.33
1292.76	1304.42	1323.65	1368.80	1401.57	1416.77
1423.88	1459.11	1462.39	1654.60	1849.12	1894.23
3039.61	3051.03	3073.01	3081.90	3117.28	3539.44
3633.63	3844.37	3895.24			

- Conformer (259); ZPE = 33603.649 cm^{-1}:

45.49	62.18	74.23	112.41	176.24	214.86
269.57	307.33	350.71	407.71	445.71	487.69
509.22	547.89	623.82	657.99	689.26	756.61
788.65	832.64	878.28	924.25	962.33	1003.52
1092.08	1142.75	1178.95	1196.28	1211.42	1232.93
1252.87	1313.28	1319.57	1331.28	1385.10	1438.77
1440.98	1453.98	1473.89	1638.25	1858.16	1882.17
3000.97	3046.93	3057.78	3093.82	3102.31	3546.88
3643.33	3846.58	3897.41			

- Conformer (260); ZPE = 33734.345 cm^{-1}:

25.65	55.35	64.18	120.38	159.97	209.55
285.20	294.37	374.93	443.54	470.84	490.08
551.61	575.17	586.21	676.42	758.85	790.17
792.48	842.97	879.21	929.04	974.36	1037.79
1101.70	1140.12	1172.08	1178.90	1235.36	1239.85
1272.35	1305.50	1338.08	1356.93	1394.57	1413.77
1435.27	1457.00	1471.25	1660.19	1885.67	1897.89
3014.95	3045.09	3069.75	3078.87	3119.82	3550.77
3640.94	3709.55	3894.14			

- Conformer (261); ZPE = 33628.813 cm^{-1}:

24.47	48.78	52.11	100.06	179.15	241.06
278.61	286.94	309.93	370.04	482.47	507.82
536.98	573.24	590.20	634.65	654.93	786.41
810.56	849.66	870.83	939.14	985.48	998.76
1073.78	1141.54	1150.08	1182.59	1203.71	1247.01
1297.28	1303.72	1328.02	1372.56	1387.13	1401.39
1410.88	1448.96	1464.62	1658.37	1877.80	1895.04
3038.38	3048.39	3083.58	3097.83	3109.25	3543.37
3632.69	3849.16	3898.20			

- Conformer (262); ZPE = 33732.381 cm^{-1}:

31.92	48.05	81.09	116.06	185.12	203.87
301.87	332.87	376.46	415.87	460.16	493.80
504.46	581.74	604.52	633.48	738.20	746.67
807.28	864.52	918.40	927.57	989.56	1014.27
1076.25	1138.48	1182.49	1185.44	1213.53	1247.43
1261.82	1310.68	1327.37	1353.30	1362.03	1390.80
1450.91	1467.82	1490.08	1666.79	1868.39	1882.36
2932.08	3056.27	3063.50	3108.40	3133.86	3541.49
3628.48	3849.43	3897.47			

- Conformer (263); ZPE = 33727.459 cm^{-1}:

7.40	42.49	71.49	116.56	202.46	228.07
292.11	326.80	376.60	401.43	466.93	504.49
542.56	562.07	598.37	668.94	743.29	777.40
800.79	835.41	860.22	949.17	977.70	1008.84
1095.99	1143.57	1163.43	1187.12	1232.18	1248.49
1261.97	1299.86	1324.71	1387.70	1401.61	1412.99
1429.09	1454.00	1467.63	1652.66	1890.35	1894.93
3023.85	3046.26	3068.66	3080.16	3116.08	3547.14
3644.06	3723.60	3895.25			

- Conformer (264); ZPE = 33639.615 cm^{-1}:

40.88	57.96	83.04	129.72	172.51	237.28
261.62	292.27	322.72	390.21	464.60	486.57
514.10	559.25	591.61	660.91	681.43	749.11
822.24	850.45	894.74	935.60	995.02	1008.59
1063.92	1124.89	1158.25	1195.11	1221.88	1241.16
1279.76	1317.20	1335.62	1354.11	1385.10	1403.80
1417.57	1451.16	1475.45	1666.57	1874.51	1900.49
3004.20	3044.82	3052.90	3090.72	3115.98	3536.72
3623.78	3850.30	3890.80			

- Conformer (265); ZPE = 33625.140 cm^{-1}:

48.29	71.15	93.34	117.37	185.06	242.52
267.77	300.91	322.41	346.10	447.54	504.94
551.08	571.57	580.13	604.08	658.22	770.91
794.59	845.58	865.58	911.69	994.67	1067.95
1073.68	1123.74	1164.92	1188.23	1216.57	1244.41
1259.54	1309.90	1339.39	1361.43	1382.23	1410.94
1443.01	1448.71	1471.31	1651.79	1849.87	1886.21
3037.52	3046.96	3056.71	3085.17	3103.27	3546.30
3635.09	3846.40	3903.52			

- Conformer (266); ZPE = 33790.058 cm^{-1}:

52.14	83.39	94.43	116.34	185.61	217.71
235.06	280.95	373.97	434.69	475.60	489.33
580.39	593.27	631.32	682.76	711.51	747.35
813.22	854.46	911.33	939.36	944.28	1026.17
1062.14	1148.95	1159.96	1177.08	1227.81	1269.18
1300.03	1307.81	1319.31	1371.73	1384.02	1387.63
1402.85	1480.03	1493.92	1672.14	1878.51	1901.05
3006.97	3024.08	3075.50	3120.58	3137.62	3540.02
3620.18	3810.37	3825.98			

- Conformer (267); ZPE = 33776.399 cm^{-1}:

40.15	71.10	75.42	120.72	204.96	256.52
299.88	307.68	352.91	427.44	481.23	495.50
532.28	557.50	585.35	632.90	669.79	748.00
794.52	867.74	889.89	945.78	969.04	1057.13
1074.54	1168.28	1170.18	1205.23	1211.73	1250.97
1296.87	1316.02	1325.68	1368.20	1393.13	1407.09
1422.83	1449.41	1483.15	1660.88	1835.42	1879.65
3022.61	3042.02	3068.72	3094.59	3117.89	3537.35
3624.78	3849.29	3892.88			

- Conformer (268); ZPE = 33642.095 cm^{-1}:

54.90	69.34	81.22	135.93	191.18	223.78
269.64	309.35	326.13	342.49	476.56	492.61
555.66	567.71	600.93	636.04	660.10	759.90
782.91	844.25	871.24	910.95	999.52	1070.31
1077.47	1159.36	1163.97	1174.09	1218.01	1243.86
1249.33	1309.36	1334.55	1342.14	1368.99	1410.30
1448.87	1454.93	1473.06	1643.57	1879.40	1898.58
3013.83	3032.70	3048.21	3079.36	3102.60	3546.40
3641.81	3833.91	3902.88			

- Conformer (269); ZPE = 33751.012 cm^{-1}:

32.97	71.85	92.19	106.58	176.77	233.90
277.66	287.11	356.57	423.30	454.83	478.54
536.82	563.00	627.02	633.66	726.94	753.68
792.61	863.23	904.43	948.02	980.24	1014.83
1081.35	1145.86	1168.62	1180.94	1223.17	1240.23
1266.90	1294.55	1329.85	1358.25	1380.18	1413.05
1428.00	1463.08	1486.31	1680.89	1861.77	1886.79
3031.61	3048.29	3078.67	3109.73	3138.86	3527.28
3609.79	3835.21	3896.06			

- Conformer (270); ZPE = 33877.327 cm^{-1}:

37.43	66.18	83.23	127.67	208.92	246.78
303.11	321.96	386.34	434.26	474.32	502.60
526.29	604.94	625.00	710.46	763.75	832.76
858.06	901.16	928.51	953.69	993.48	1018.70
1064.51	1143.44	1186.03	1202.02	1222.89	1238.37
1305.24	1321.89	1327.12	1353.62	1382.63	1437.08
1467.99	1472.04	1492.12	1634.48	1884.33	1893.92
3000.98	3030.13	3044.14	3083.89	3143.56	3462.57
3528.95	3630.00	3891.14			

- Conformer (271); ZPE = 33577.875 cm^{-1}:

31.97	51.81	78.28	94.38	157.72	195.99
258.67	298.34	344.35	436.62	458.20	512.44
525.14	537.55	646.65	655.14	661.26	767.62
795.87	807.35	865.98	934.60	951.42	1034.09
1095.36	1152.25	1172.30	1178.08	1213.20	1243.56
1266.57	1311.47	1312.81	1327.98	1379.64	1431.78
1445.53	1452.82	1479.91	1639.32	1861.92	1896.61
2987.23	3042.03	3050.17	3079.04	3096.18	3552.01
3649.78	3847.55	3889.18			

- Conformer (272); ZPE = 33776.710 cm^{-1}:

41.76	63.17	93.43	121.25	195.60	256.55
266.96	314.64	330.86	417.09	525.45	535.04
550.66	578.57	611.57	669.47	711.21	745.58
826.50	842.90	871.92	919.59	986.79	1011.99
1099.70	1164.70	1177.32	1217.60	1228.96	1234.12
1303.34	1304.61	1329.90	1372.35	1388.75	1401.66
1420.18	1452.23	1489.60	1667.21	1834.16	1874.26
3003.97	3055.98	3063.22	3100.46	3110.01	3550.38
3637.22	3748.85	3834.13			

- Conformer (273); ZPE = 33750.187 cm^{-1}:

41.61	77.60	89.24	115.35	187.20	237.93
317.94	323.49	378.23	423.19	450.84	532.56
560.13	581.69	597.72	618.88	695.75	771.80
806.94	856.28	902.43	944.40	951.43	1009.18
1076.62	1122.82	1178.92	1206.73	1219.42	1243.97
1256.72	1308.84	1321.54	1346.47	1378.91	1391.81
1429.91	1462.97	1505.30	1641.60	1876.81	1889.61
2950.96	3037.89	3056.35	3094.54	3156.72	3553.18
3643.34	3826.19	3850.43			

- Conformer (274); ZPE = 33667.915 cm^{-1}:

41.21	50.65	75.72	118.00	198.02	220.22
247.44	292.55	330.05	411.53	507.93	517.80
540.09	566.80	599.26	627.62	654.08	739.62
824.03	849.36	869.88	932.49	994.63	1006.34
1094.21	1142.80	1166.59	1183.63	1209.57	1237.09
1288.56	1313.81	1323.63	1356.23	1372.39	1392.37
1431.10	1451.56	1499.06	1671.92	1882.82	1889.36
3024.28	3044.21	3066.26	3091.59	3112.27	3544.38
3629.00	3843.29	3858.52			

- Conformer (275); ZPE = 33601.149 cm^{-1}:

34.93	46.10	53.83	112.52	193.88	236.50
288.58	303.92	313.77	342.56	460.36	497.16
561.10	570.69	605.00	611.48	647.41	779.72
790.60	825.79	861.80	909.02	987.43	1057.14
1081.79	1125.45	1159.12	1183.14	1213.91	1235.42
1264.91	1308.66	1339.93	1366.26	1377.18	1412.35
1442.69	1455.32	1467.69	1648.17	1856.52	1891.97
3038.84	3042.90	3079.34	3085.06	3102.58	3547.34
3638.64	3848.29	3897.57			

- Conformer (276); ZPE = 33543.254 cm^{-1}:

28.23	46.59	68.76	88.26	157.45	191.96
249.41	292.35	332.36	429.54	460.53	481.39
529.99	572.79	597.53	653.30	679.61	768.00
789.86	807.81	861.90	934.94	953.90	1032.32
1097.27	1144.98	1163.78	1174.11	1206.16	1250.22
1261.13	1305.03	1318.81	1338.50	1368.45	1412.79
1450.20	1458.42	1483.86	1637.64	1862.35	1898.00
3021.41	3045.64	3060.66	3062.21	3110.13	3555.09
3651.73	3846.22	3892.97			

- Conformer (277); ZPE = 33702.489 cm^{-1}:

38.91	65.52	94.66	124.34	192.16	199.21
262.80	306.33	335.29	409.26	515.12	528.92
536.17	591.34	621.11	665.31	684.91	748.81
805.35	811.66	862.81	917.43	964.96	1021.41
1092.61	1146.61	1188.96	1200.65	1225.32	1243.23
1276.64	1325.50	1339.67	1370.72	1382.76	1387.53
1442.09	1448.85	1486.14	1633.41	1883.82	1902.06
2989.57	3056.28	3057.31	3096.61	3106.77	3542.51
3640.99	3803.97	3830.59			

- Conformer (278); ZPE = 33551.581 cm^{-1}:

24.37	35.70	65.89	92.31	127.43	213.79
258.38	287.00	290.62	384.43	467.54	473.23
548.17	577.47	600.35	633.92	667.55	788.98
794.05	839.78	900.85	954.55	959.91	1020.85
1095.08	1143.23	1151.47	1172.76	1201.03	1260.69
1275.94	1303.27	1328.01	1356.82	1385.53	1393.87
1413.58	1461.45	1480.93	1662.45	1869.10	1897.45
3027.94	3048.55	3068.62	3073.16	3106.79	3544.80
3631.16	3846.25	3896.12			

- Conformer (279); ZPE = 33705.462 cm^{-1}:

47.53	64.49	73.17	118.60	178.35	245.24
273.54	291.65	366.06	425.81	467.85	483.61
519.88	584.61	590.62	633.48	698.93	742.58
790.12	861.21	902.67	936.82	981.00	1004.11
1073.09	1141.40	1161.42	1167.04	1230.16	1235.47
1276.01	1310.66	1327.43	1367.14	1376.64	1384.20
1413.36	1472.57	1484.21	1682.78	1873.76	1896.42
3036.19	3054.51	3059.75	3107.29	3135.98	3525.00
3613.12	3838.23	3885.20			

- Conformer (280); ZPE = 33694.395 cm^{-1}:

32.66	50.60	71.72	173.01	185.47	212.10
247.20	281.45	345.34	403.34	497.99	507.45
518.13	533.90	628.04	663.91	668.20	769.79
789.83	859.19	896.35	926.15	950.60	1050.00
1070.83	1150.99	1167.59	1182.75	1217.48	1233.17
1296.14	1303.19	1330.78	1361.29	1384.06	1412.53
1448.81	1465.96	1473.80	1669.03	1856.41	1897.46
2995.40	3049.95	3055.60	3090.04	3107.94	3546.52
3628.76	3847.52	3882.36			

- Conformer (281); ZPE = 33832.199 cm^{-1}:

51.02	59.10	81.86	141.48	173.11	249.87
278.51	293.99	332.94	412.59	448.78	493.14
511.78	605.99	667.38	703.57	750.61	827.18
874.89	911.44	936.41	958.25	1012.53	1044.84
1064.93	1160.93	1165.59	1193.02	1204.32	1260.36
1316.09	1324.06	1340.43	1359.55	1374.44	1428.20
1463.75	1467.29	1479.32	1667.03	1882.21	1895.94
3026.54	3037.00	3043.94	3087.71	3137.78	3454.57
3510.92	3610.55	3886.71			

- Conformer (282); ZPE = 33839.596 cm^{-1}:

40.34	62.35	70.43	108.35	177.24	198.83
273.62	331.75	358.92	407.76	453.83	496.80
556.79	601.29	657.15	731.24	749.38	787.57
808.63	869.53	907.42	949.49	988.73	1017.92
1059.83	1112.28	1176.63	1195.52	1243.21	1251.28
1291.20	1309.21	1328.72	1370.64	1377.10	1403.00
1424.22	1473.88	1492.39	1677.91	1886.34	1895.78
3013.15	3047.83	3063.82	3096.03	3127.51	3544.58
3613.38	3707.53	3890.83			

- Conformer (283); ZPE = 33619.211 cm^{-1}:

23.22	45.85	55.19	118.91	195.37	248.57
286.59	305.36	316.56	334.74	469.44	472.50
557.27	570.37	623.18	638.71	657.08	777.99
788.14	833.85	867.64	907.80	993.48	1051.90
1095.82	1145.64	1161.64	1166.61	1212.68	1225.78
1263.32	1303.76	1331.70	1352.36	1376.53	1407.54
1454.99	1459.40	1475.04	1635.19	1870.06	1895.15
3038.92	3045.69	3075.64	3083.69	3103.71	3544.91
3642.02	3833.52	3897.41			

- Conformer (284); ZPE = 33633.030 cm^{-1}:

37.61	39.98	80.94	125.84	196.65	231.34
274.95	310.22	344.68	381.89	480.91	482.22
512.24	562.38	619.57	647.27	674.84	759.68
799.42	829.44	897.88	928.64	959.69	1016.73
1068.87	1146.91	1170.23	1184.87	1205.88	1222.59
1276.23	1298.90	1321.09	1362.72	1394.57	1431.64
1447.34	1449.76	1464.60	1655.17	1843.44	1901.31
2933.85	3060.89	3065.73	3115.43	3121.38	3545.22
3638.41	3847.07	3896.96			

- Conformer (285); ZPE = 33993.786 cm^{-1}:

50.69	90.28	116.78	160.04	213.48	274.93
299.06	315.92	358.51	366.37	504.34	513.02
564.88	623.33	637.67	670.60	760.77	793.66
816.72	860.94	885.89	952.92	972.15	1006.36
1076.00	1160.25	1175.95	1210.09	1222.25	1271.72
1286.22	1317.62	1324.62	1385.71	1397.14	1415.38
1426.01	1437.58	1492.76	1664.63	1852.01	1884.56
3043.14	3061.87	3094.84	3099.24	3116.21	3550.18
3635.78	3683.47	3893.03			

- Conformer (286); ZPE = 33572.294 cm^{-1}:

27.84	49.89	67.86	104.53	117.78	174.54
240.45	260.41	308.74	393.67	442.42	502.37
516.48	577.88	614.90	672.49	706.55	765.84
783.62	860.55	902.13	965.09	968.30	1026.10
1081.56	1123.43	1161.34	1187.97	1225.88	1250.08
1269.42	1306.28	1332.59	1348.88	1383.52	1398.43
1419.16	1465.09	1484.20	1669.45	1868.12	1899.18
3011.53	3037.92	3064.89	3086.79	3126.43	3534.18
3618.47	3848.71	3890.66			

- Conformer (287); ZPE = 33749.437 cm^{-1}:

49.73	65.01	85.48	166.15	175.22	244.33
250.05	285.88	311.87	403.55	449.52	532.57
565.51	592.66	603.49	622.86	742.28	780.94
803.56	850.21	868.68	919.49	998.07	1050.21
1066.11	1114.44	1154.79	1191.63	1202.18	1258.12
1275.05	1320.54	1338.10	1350.34	1378.71	1396.86
1442.64	1472.16	1484.19	1653.17	1840.61	1885.71
3045.21	3057.56	3076.33	3103.89	3117.79	3544.84
3634.40	3835.90	3840.28			

- Conformer (288); ZPE = 33713.544 cm^{-1}:

48.25	64.52	69.52	121.57	163.50	245.78
282.45	301.12	348.23	415.47	451.14	499.82
529.13	570.91	594.54	656.96	706.55	760.08
822.95	842.95	894.56	930.18	968.81	1031.97
1053.17	1147.87	1162.54	1177.58	1211.07	1244.73
1276.25	1288.43	1342.84	1345.46	1385.87	1405.72
1424.99	1463.10	1474.15	1682.26	1866.33	1897.35
3033.40	3051.24	3058.49	3106.91	3116.88	3527.10
3617.74	3853.36	3891.30			

- Conformer (289); ZPE = 33866.237 cm^{-1}:

41.11	84.37	133.14	165.69	179.96	242.51
244.93	315.53	321.00	379.64	474.16	519.07
542.70	608.03	659.59	734.44	767.74	794.94
812.14	829.78	879.13	901.66	1004.56	1037.91
1087.53	1140.78	1171.37	1198.15	1215.50	1255.99
1278.07	1324.91	1342.35	1362.42	1394.20	1402.94
1457.54	1474.74	1494.26	1632.71	1853.78	1884.65
3032.56	3050.02	3055.08	3089.72	3108.28	3540.79
3638.60	3667.17	3904.66			

- Conformer (290); ZPE = 33790.001 cm^{-1}:

70.58	75.35	96.84	152.49	173.02	255.88
276.46	312.62	348.65	442.49	459.96	501.44
552.10	585.79	662.37	697.48	733.58	781.28
814.83	824.04	894.24	903.43	954.50	1010.71
1047.98	1131.49	1151.59	1176.02	1226.84	1242.52
1282.84	1317.04	1342.68	1366.72	1374.50	1385.89
1416.42	1470.70	1478.88	1663.54	1886.19	1900.81
3022.10	3036.45	3048.71	3105.54	3120.44	3555.11
3641.22	3719.19	3888.44			

- Conformer (291); ZPE = 33723.393 cm^{-1}:

50.92	61.46	104.14	142.72	171.12	196.14
257.92	290.09	328.06	425.50	459.95	532.97
555.04	584.72	607.44	668.23	719.99	758.16
819.88	828.99	855.43	921.87	949.81	1026.24
1060.87	1114.57	1134.22	1180.62	1235.81	1252.14
1266.37	1319.12	1332.07	1353.03	1383.16	1391.38
1428.68	1467.32	1483.66	1657.35	1883.04	1891.48
3046.69	3057.46	3068.71	3101.25	3115.06	3563.02
3649.78	3830.66	3862.46			

- Conformer (292); ZPE = 33552.894 cm^{-1}:

35.41	41.75	62.27	123.14	141.66	209.73
275.47	279.63	298.71	355.43	469.48	481.98
549.67	572.56	586.30	615.59	663.84	769.29
787.33	834.73	898.05	911.84	1022.55	1055.11
1089.06	1144.70	1153.33	1166.31	1192.32	1240.96
1284.14	1308.12	1322.44	1346.42	1393.01	1415.87
1425.82	1456.49	1475.92	1657.40	1858.38	1900.80
3022.38	3031.23	3061.21	3076.09	3118.60	3544.03
3636.24	3845.72	3897.29			

- Conformer (293); ZPE = 33609.488 cm^{-1}:

26.65	47.56	58.52	107.16	173.77	203.55
246.84	313.37	363.14	437.61	459.22	495.29
515.14	578.28	610.02	634.53	713.88	763.01
794.86	832.24	866.00	928.36	966.60	1025.52
1081.35	1131.46	1179.87	1188.93	1219.51	1235.55
1271.29	1302.85	1315.73	1341.38	1387.29	1397.83
1430.49	1465.36	1493.41	1634.51	1870.00	1896.57
2962.58	3021.71	3054.37	3094.85	3120.43	3560.90
3659.89	3847.86	3891.87			

- Conformer (294); ZPE = 33686.863 cm^{-1}:

46.35	60.59	79.82	138.66	181.76	224.11
263.96	288.56	336.88	395.19	478.21	491.89
548.94	560.33	591.40	644.19	668.51	778.03
792.61	849.27	887.16	926.35	973.96	995.90
1079.43	1142.38	1153.79	1181.22	1198.37	1251.63
1259.07	1306.27	1335.39	1369.75	1390.86	1399.54
1414.42	1455.89	1459.97	1669.45	1881.06	1898.00
3037.15	3063.27	3079.04	3094.17	3121.12	3555.86
3635.47	3840.32	3898.20			

- Conformer (295); ZPE = 33625.570 cm^{-1}:

52.51	74.32	95.35	138.31	164.13	214.46
245.71	295.98	324.62	356.68	478.07	490.80
548.57	589.07	609.20	638.11	660.92	777.88
794.21	849.17	880.74	916.49	993.37	1030.02
1085.46	1130.31	1167.65	1184.44	1201.24	1236.49
1278.34	1301.55	1324.33	1364.16	1373.43	1378.43
1441.24	1448.12	1495.14	1667.66	1855.50	1889.90
3006.76	3055.44	3075.02	3099.50	3112.31	3534.80
3620.06	3824.06	3881.11			

- Conformer (296); ZPE = 33518.929 cm^{-1}:

24.54	34.41	79.78	101.31	132.85	193.73
234.90	280.71	330.93	332.57	450.52	462.52
553.91	570.71	598.69	645.22	662.85	759.50
796.50	846.13	908.31	913.52	1016.81	1054.51
1093.62	1142.70	1159.66	1167.65	1188.24	1238.15
1288.73	1296.02	1318.64	1348.46	1377.19	1411.33
1425.07	1456.11	1481.38	1669.48	1867.39	1902.20
3028.77	3046.76	3059.71	3072.08	3118.08	3540.93
3624.27	3834.03	3895.75			

- Conformer (297); ZPE = 33578.056 cm^{-1}:

23.01	47.59	56.74	115.59	165.03	198.01
212.77	312.10	360.68	434.34	469.90	503.12
511.83	576.34	607.92	631.77	711.43	769.97
786.51	835.61	864.31	924.11	957.78	1029.94
1081.55	1131.87	1178.27	1188.89	1215.04	1235.71
1274.21	1302.37	1313.80	1335.89	1383.32	1398.83
1431.46	1468.22	1494.83	1634.82	1876.01	1895.20
2959.79	3032.18	3038.31	3081.26	3145.65	3559.14
3657.60	3849.28	3886.21			

- Conformer (298); ZPE = 33543.921 cm^{-1}:

35.63	43.24	88.70	104.38	129.29	195.47
217.20	287.39	325.35	336.65	448.46	507.34
529.27	555.21	639.74	657.08	665.23	753.14
803.74	848.13	908.40	915.52	1018.43	1049.89
1090.47	1142.92	1163.24	1170.30	1191.58	1241.24
1288.44	1295.17	1320.59	1359.86	1363.92	1413.85
1437.15	1459.08	1481.19	1667.31	1871.24	1895.63
3005.21	3029.97	3055.84	3075.61	3117.05	3536.22
3618.48	3845.23	3888.16			

- Conformer (299); ZPE = 33619.304 cm^{-1}:

24.77	46.55	58.10	90.86	152.93	190.59
209.41	276.20	340.84	433.32	440.70	510.59
523.51	568.80	626.13	651.29	700.83	775.26
784.89	848.92	908.44	921.52	952.32	1047.44
1075.89	1159.68	1164.27	1175.82	1236.74	1239.70
1276.47	1307.19	1313.42	1354.65	1386.12	1402.30
1425.99	1460.49	1476.57	1677.95	1873.96	1892.55
3031.35	3037.14	3053.37	3100.39	3130.72	3542.00
3621.78	3850.79	3887.10			

- Conformer (300); ZPE = 33549.170 cm^{-1}:

16.30	49.02	70.92	100.05	116.35	182.64
241.96	253.15	311.70	391.95	437.48	501.85
514.70	571.07	613.16	671.61	703.03	747.33
798.22	862.86	901.40	949.91	975.29	1026.20
1081.49	1124.55	1162.73	1186.03	1222.36	1253.64
1276.03	1293.81	1338.00	1344.01	1383.54	1396.96
1416.55	1464.73	1483.93	1672.21	1873.26	1898.21
3007.73	3032.66	3045.49	3089.98	3151.89	3536.91
3618.33	3848.40	3886.75			

- Conformer (301); ZPE = 33583.272 cm^{-1}:

35.73	49.30	56.13	110.91	184.46	212.29
259.18	276.82	318.02	360.08	476.41	497.35
542.80	579.66	594.04	611.23	657.89	779.39
795.34	815.20	856.17	928.38	977.42	1062.10
1112.36	1131.14	1157.85	1182.01	1203.19	1223.13
1274.57	1302.47	1327.84	1363.02	1397.04	1403.57
1453.02	1461.41	1462.10	1655.23	1849.64	1892.13
3035.54	3050.51	3075.22	3079.20	3107.96	3546.00
3641.55	3848.28	3894.25			

- Conformer (302); ZPE = 33782.594 cm^{-1}:

12.71	65.83	85.44	153.11	178.72	225.78
269.29	289.18	351.53	436.60	471.94	513.45
539.70	552.77	594.76	633.02	746.84	751.35
802.97	850.60	873.51	931.63	958.68	1044.48
1064.24	1146.61	1179.57	1191.45	1209.22	1253.81
1281.80	1304.91	1332.98	1357.12	1391.93	1401.50
1417.80	1466.19	1487.10	1673.99	1848.03	1872.19
3029.21	3058.41	3065.57	3111.81	3125.43	3559.80
3639.16	3848.60	3912.86			

- Conformer (303); ZPE = 33652.246 cm^{-1}:

45.22	74.22	84.19	129.80	188.28	214.04
232.34	306.75	370.08	442.27	479.69	500.97
505.18	578.71	590.57	610.76	716.86	757.01
800.89	846.08	872.19	928.56	955.64	1005.97
1087.59	1124.23	1161.56	1185.24	1212.68	1234.77
1251.04	1312.83	1333.12	1342.63	1373.05	1385.18
1443.16	1475.01	1478.99	1643.66	1872.63	1896.81
2976.35	3045.95	3051.10	3097.51	3127.54	3558.21
3656.52	3842.34	3898.53			

- Conformer (304); ZPE = 33627.457 cm^{-1}:

38.08	53.76	92.23	110.69	171.81	246.30
271.25	331.97	389.74	433.75	483.55	500.89
518.95	565.00	587.85	598.10	679.75	751.90
804.99	824.11	868.90	919.59	968.90	999.86
1067.60	1134.07	1175.22	1183.31	1214.52	1239.07
1245.21	1309.97	1337.35	1344.84	1387.06	1399.71
1433.29	1456.56	1492.75	1626.98	1875.71	1899.64
2945.26	3037.82	3054.16	3091.26	3108.33	3564.31
3664.65	3852.69	3901.64			

- Conformer (305); ZPE = 33531.782 cm^{-1}:

25.02	48.28	61.43	123.38	145.67	213.33
281.02	287.43	308.53	360.20	467.66	495.32
538.09	569.73	585.58	612.61	663.40	756.75
783.96	821.81	894.96	916.44	1015.94	1073.00
1086.19	1140.90	1165.77	1172.16	1198.64	1253.46
1271.92	1312.73	1316.23	1337.93	1371.29	1422.34
1453.65	1461.08	1475.12	1651.20	1868.11	1897.33
2926.75	3022.69	3060.14	3087.21	3113.41	3558.53
3648.22	3845.53	3895.52			

- Conformer (306); ZPE = 33987.687 cm^{-1}:

38.64	65.44	80.79	151.30	198.95	228.34
294.02	335.40	340.41	427.47	468.77	543.96
594.70	637.42	702.29	729.17	762.59	792.89
825.86	858.07	912.59	936.40	1006.83	1021.02
1058.27	1128.83	1153.51	1196.92	1227.69	1264.70
1305.79	1336.57	1353.44	1366.59	1377.85	1403.46
1414.40	1471.20	1476.07	1657.42	1889.99	1901.91
3036.85	3041.78	3050.27	3112.89	3139.69	3518.54
3593.28	3740.11	3804.02			

- Conformer (307); ZPE = 33616.050 cm^{-1}:

30.88	39.58	60.69	133.56	150.63	215.10
262.20	276.66	325.79	383.08	467.33	486.44
560.90	576.62	582.55	631.61	689.85	764.13
785.19	834.80	897.69	922.63	961.28	1056.92
1079.63	1156.13	1162.91	1180.35	1192.28	1239.00
1284.58	1307.07	1331.79	1353.34	1392.02	1405.17
1422.77	1457.00	1468.51	1666.86	1857.88	1899.68
3025.09	3042.93	3073.55	3077.81	3127.44	3550.48
3635.28	3848.11	3898.33			

- Conformer (308); ZPE = 33708.917 cm^{-1}:

26.72	49.23	52.91	137.61	172.05	239.15
274.18	344.56	359.10	432.15	447.19	476.82
544.37	552.42	591.04	633.07	709.38	749.26
822.29	846.13	881.44	920.94	966.14	1035.19
1051.32	1147.60	1173.36	1186.57	1206.23	1239.61
1285.05	1312.29	1328.41	1370.22	1387.90	1397.45
1411.78	1464.70	1482.12	1681.09	1854.70	1883.66
3024.83	3049.26	3052.34	3113.95	3127.43	3547.44
3635.04	3845.60	3894.55			

- Conformer (309); ZPE = 33606.690 cm^{-1}:

19.87	35.88	51.56	116.57	158.78	226.91
261.15	291.15	366.25	432.35	437.20	470.22
517.33	584.34	588.52	643.58	688.86	752.10
825.11	842.20	884.39	922.82	967.21	1033.67
1054.46	1147.40	1166.53	1176.88	1210.94	1231.62
1288.65	1292.10	1328.77	1361.33	1379.29	1387.57
1426.95	1468.56	1476.18	1682.54	1871.91	1891.01
3042.04	3045.59	3054.69	3102.60	3108.33	3533.46
3624.32	3841.93	3899.71			

- Conformer (310); ZPE = 33786.164 cm^{-1}:

43.51	74.74	99.54	109.67	198.05	252.12
278.26	299.27	332.85	399.46	458.36	514.64
559.79	621.22	634.00	641.47	717.80	751.87
781.92	841.93	880.07	929.72	1010.26	1051.60
1070.33	1142.87	1166.49	1203.49	1223.95	1259.51
1281.54	1305.78	1339.52	1343.00	1383.03	1399.03
1435.90	1479.42	1496.46	1641.79	1881.92	1901.10
2922.36	3051.32	3058.34	3104.18	3139.07	3558.69
3651.39	3819.91	3829.82			

- Conformer (311); ZPE = 33670.832 cm^{-1}:

37.90	43.56	61.15	124.29	179.48	249.67
284.77	299.83	346.84	399.38	468.31	496.10
544.28	575.53	597.40	640.95	673.05	763.22
819.75	838.30	873.42	932.65	963.84	1016.64
1079.35	1126.17	1162.38	1192.75	1211.56	1247.52
1267.56	1308.05	1317.70	1358.95	1383.74	1411.15
1438.11	1455.54	1473.34	1657.45	1870.51	1891.54
3025.58	3055.48	3063.10	3073.39	3111.45	3553.98
3639.35	3841.40	3894.26			

- Conformer (312); ZPE = 33709.994 cm^{-1}:

32.29	43.42	80.18	151.97	166.53	226.28
269.52	325.38	341.75	411.28	444.28	515.14
545.59	568.78	627.95	645.68	738.52	761.31
765.09	810.36	848.28	908.52	933.06	1037.45
1042.21	1125.67	1171.61	1179.21	1212.85	1258.58
1300.01	1309.17	1338.07	1355.15	1380.50	1393.87
1403.26	1465.16	1480.26	1660.43	1880.94	1887.61
3058.75	3065.40	3074.33	3118.01	3132.03	3581.70
3677.12	3827.61	3841.86			

- Conformer (313); ZPE = 33779.779 cm^{-1}:

34.92	73.41	101.18	105.46	191.82	222.15
258.26	286.24	321.01	385.05	447.99	506.74
567.89	621.00	640.78	653.62	715.50	750.32
789.41	862.93	911.78	941.24	1009.75	1031.98
1062.90	1153.05	1163.11	1197.06	1219.95	1259.70
1299.41	1315.27	1321.71	1376.06	1382.45	1392.40
1402.72	1480.90	1498.74	1669.10	1877.38	1900.80
3002.77	3038.62	3059.19	3110.52	3143.57	3539.10
3620.78	3816.25	3825.62			

- Conformer (314); ZPE = 33584.887 cm^{-1}:

32.39	40.57	62.56	100.92	115.55	204.73
239.37	250.54	310.16	390.05	442.92	494.87
511.43	589.57	617.98	659.50	720.69	765.70
800.28	855.99	902.97	952.58	977.05	1019.60
1068.84	1109.33	1162.85	1190.22	1222.76	1250.89
1274.62	1295.50	1334.90	1349.35	1373.06	1388.99
1420.59	1474.78	1491.68	1670.82	1870.03	1902.57
3027.18	3037.83	3044.00	3089.07	3152.91	3541.31
3623.85	3842.04	3901.84			

- Conformer (315); ZPE = 33707.058 cm^{-1}:

31.48	44.19	68.99	160.70	187.77	209.14
272.20	307.08	345.22	401.09	495.25	505.75
531.07	582.50	607.02	641.23	667.30	766.78
803.21	844.91	883.46	916.35	948.27	1038.80
1069.58	1150.24	1169.90	1204.76	1210.43	1229.42
1294.58	1311.45	1335.37	1362.93	1393.36	1396.24
1417.34	1464.07	1467.62	1659.36	1851.17	1894.77
3025.54	3059.51	3063.75	3074.61	3123.35	3549.87
3639.77	3840.21	3895.12			

- Conformer (316); ZPE = 33653.354 cm^{-1}:

26.68	47.42	66.18	97.64	127.72	246.96
252.53	279.16	363.59	424.09	464.62	480.48
545.75	581.88	602.15	677.13	750.52	785.74
806.74	838.64	880.28	936.69	977.49	1049.69
1097.27	1132.67	1155.07	1175.45	1237.78	1252.37
1258.11	1299.08	1322.87	1354.04	1389.35	1413.94
1426.90	1457.86	1484.68	1653.52	1891.36	1903.69
3015.56	3030.13	3053.16	3061.69	3120.12	3553.40
3653.74	3699.11	3904.01			

- Conformer (317); ZPE = 33542.996 cm^{-1}:

30.74	38.58	81.25	99.88	145.95	194.28
241.77	302.40	321.08	334.05	458.57	473.56
552.26	573.97	602.59	643.98	662.45	761.76
799.89	845.71	875.02	910.69	1024.59	1080.35
1086.42	1149.28	1167.92	1173.00	1177.67	1236.27
1277.30	1309.52	1322.47	1341.10	1363.13	1410.61
1452.44	1462.41	1482.59	1641.80	1872.61	1900.21
3026.63	3034.26	3052.01	3071.11	3102.79	3545.00
3637.06	3837.12	3897.90			

- Conformer (318); ZPE = 33534.324 cm^{-1}:

32.74	36.86	84.02	101.11	137.16	197.98
251.18	306.18	322.06	336.78	454.81	493.09
521.79	555.22	640.04	650.27	661.61	754.01
805.35	849.29	877.96	908.71	1022.32	1080.88
1087.09	1153.49	1165.53	1182.41	1186.09	1236.94
1275.09	1300.31	1327.02	1335.68	1361.95	1432.50
1447.27	1457.99	1478.49	1643.11	1868.81	1898.87
2982.64	3028.55	3050.66	3077.28	3103.11	3538.48
3630.09	3844.98	3892.79			

- Conformer (319); ZPE = 33680.384 cm^{-1}:

43.30	54.65	74.10	124.42	169.70	263.68
298.89	333.53	355.08	410.96	468.22	496.81
535.31	562.74	606.23	668.41	687.90	760.50
803.50	822.06	865.79	912.93	958.38	1006.06
1071.88	1146.53	1163.62	1178.51	1232.03	1237.90
1260.56	1309.70	1320.63	1345.68	1387.72	1395.69
1442.50	1455.15	1479.57	1637.41	1874.41	1899.79
2987.96	3043.16	3056.67	3096.24	3116.87	3548.46
3652.19	3842.91	3893.88			

- Conformer (320); ZPE = 33654.240 cm^{-1}:

33.58	46.07	63.79	108.28	173.68	219.12
283.45	358.64	371.94	408.64	452.99	481.80
488.82	575.54	595.42	633.15	706.33	748.77
825.26	845.70	891.36	924.10	967.78	1040.34
1068.07	1146.67	1169.20	1186.10	1227.32	1246.73
1251.01	1312.01	1336.13	1346.57	1381.89	1388.24
1448.27	1468.56	1473.43	1673.72	1870.63	1881.81
2935.14	3039.22	3047.58	3108.76	3116.19	3558.84
3641.78	3847.88	3892.17			

- Conformer (321); ZPE = 33703.007 cm^{-1}:

39.30	68.68	81.01	109.47	182.87	204.87
242.01	299.48	380.60	426.96	444.64	514.90
558.20	575.07	596.99	617.68	699.74	772.19
807.80	861.19	913.97	942.25	949.03	1029.09
1062.51	1145.38	1170.65	1196.60	1213.30	1250.98
1293.16	1302.53	1308.70	1366.96	1378.02	1389.15
1406.28	1470.79	1502.00	1673.58	1876.57	1889.09
3016.98	3023.51	3067.66	3113.34	3140.58	3530.20
3607.88	3843.03	3848.61			

- Conformer (322); ZPE = 33542.619 cm^{-1}:

34.96	42.02	72.31	105.11	135.86	196.77
235.84	299.88	321.43	332.66	474.94	487.76
563.54	575.62	591.90	611.05	661.14	764.05
803.84	846.39	859.39	911.92	1020.70	1073.62
1086.19	1119.31	1161.77	1169.06	1190.06	1246.60
1278.48	1314.64	1325.93	1356.82	1370.78	1410.63
1440.93	1456.18	1479.38	1652.66	1863.83	1897.66
3029.24	3046.40	3062.95	3071.94	3100.55	3547.74
3636.23	3848.94	3897.66			

- Conformer (323); ZPE = 33828.954 cm^{-1}:

52.38	89.75	123.59	155.22	182.02	232.45
256.85	292.72	344.59	429.89	463.85	537.50
544.03	584.36	649.34	692.01	725.10	773.55
822.33	839.27	886.23	917.43	952.56	1031.85
1058.39	1145.81	1165.59	1184.95	1238.14	1259.73
1265.85	1317.92	1332.57	1348.88	1377.02	1406.10
1442.78	1477.90	1485.10	1655.13	1860.75	1882.48
2980.83	3039.26	3063.05	3089.09	3119.24	3562.20
3645.82	3787.14	3887.37			

- Conformer (324); ZPE = 33620.675 cm^{-1}:

41.90	54.77	65.18	104.53	179.25	198.48
244.10	342.09	346.23	406.42	453.50	516.47
525.66	589.83	604.63	652.89	725.94	753.58
769.25	802.77	871.30	924.14	976.66	1007.37
1073.83	1122.59	1151.99	1172.39	1209.19	1239.27
1268.01	1303.80	1332.16	1351.03	1365.10	1385.33
1453.04	1476.95	1485.41	1628.53	1876.90	1890.66
3030.49	3039.62	3061.38	3083.23	3133.89	3555.14
3656.77	3844.21	3893.49			

- Conformer (325); ZPE = 33656.878 cm^{-1}:

33.71	46.36	66.08	114.82	181.91	237.15
265.77	313.11	317.24	366.44	471.24	500.70
551.49	584.91	606.93	645.28	666.75	781.88
789.53	845.48	876.52	931.73	969.69	1059.26
1074.91	1141.63	1172.34	1178.13	1198.12	1224.61
1284.63	1304.22	1325.68	1356.20	1375.94	1389.47
1441.79	1466.43	1494.98	1668.13	1864.63	1899.74
3031.03	3055.85	3058.48	3078.97	3124.24	3534.38
3621.52	3838.22	3885.51			

- Conformer (326); ZPE = 33627.840 cm^{-1}:

29.66	70.23	94.32	126.89	174.21	235.88
295.01	304.88	354.30	396.59	461.81	485.23
530.70	553.90	592.19	665.19	685.16	755.79
787.42	835.45	869.08	926.10	954.04	983.96
1093.39	1136.96	1173.61	1198.45	1221.11	1236.52
1253.16	1316.14	1326.33	1344.15	1393.17	1395.48
1443.92	1448.39	1475.83	1639.36	1874.97	1894.56
2991.85	3043.39	3059.17	3088.71	3112.74	3545.15
3641.72	3844.89	3894.56			

- Conformer (327); ZPE = 33711.763 cm^{-1}:

16.18	48.60	74.03	102.72	112.22	237.76
275.12	301.24	368.81	418.19	445.95	488.57
543.54	597.30	623.27	705.96	745.28	782.79
806.11	852.73	881.39	928.93	981.09	1051.25
1083.11	1128.99	1156.72	1214.21	1233.20	1253.63
1266.40	1308.69	1329.83	1355.08	1393.65	1394.27
1430.86	1465.51	1481.80	1660.39	1888.87	1896.86
3022.89	3035.29	3040.95	3084.68	3119.85	3551.61
3649.76	3697.34	3890.02			

- Conformer (328); ZPE = 33557.298 cm^{-1}:

20.59	48.53	54.20	91.28	165.41	187.78
209.67	290.75	343.55	431.09	437.32	501.06
532.15	574.23	625.92	657.14	712.54	769.37
791.42	805.33	868.71	925.72	946.72	1034.93
1079.19	1133.20	1172.02	1204.30	1226.99	1249.42
1263.88	1306.58	1315.79	1336.74	1389.83	1399.02
1436.05	1461.23	1484.77	1639.83	1872.62	1896.70
2988.05	3035.17	3049.38	3082.48	3132.16	3551.12
3647.70	3849.37	3885.61			

- Conformer (329); ZPE = 33601.124 cm^{-1}:

29.15	45.59	68.65	87.85	176.92	184.24
251.60	295.93	346.45	428.88	440.48	512.50
535.73	572.21	619.17	658.58	718.63	761.36
802.85	803.71	869.36	928.60	961.07	1019.76
1074.69	1134.67	1169.28	1197.89	1231.93	1244.62
1259.27	1309.56	1314.66	1339.34	1385.00	1395.67
1433.01	1461.29	1485.77	1640.22	1874.76	1897.89
2985.35	3034.48	3055.17	3086.85	3127.54	3553.26
3650.38	3850.86	3889.56			

- Conformer (330); ZPE = 33620.132 cm^{-1}:

36.76	59.22	68.33	102.40	179.10	278.74
300.36	314.62	343.70	420.80	463.37	497.25
518.75	538.74	606.35	632.96	668.75	732.05
802.44	841.43	890.57	919.68	982.70	1056.18
1067.49	1155.78	1177.93	1187.65	1222.80	1238.36
1264.36	1301.59	1325.24	1327.83	1393.12	1421.15
1436.95	1449.78	1487.63	1624.00	1856.45	1901.23
2909.90	3032.33	3055.65	3088.54	3117.90	3556.01
3651.32	3842.19	3891.83			

- Conformer (331); ZPE = 33594.077 cm^{-1}:

24.62	38.65	64.87	98.19	140.05	198.46
255.31	277.62	326.09	423.18	457.92	483.63
550.87	578.54	593.37	638.13	678.68	780.10
784.29	834.31	888.28	921.93	948.37	1052.73
1072.62	1152.59	1161.62	1174.04	1214.01	1234.67
1283.20	1302.30	1331.37	1351.58	1377.02	1402.27
1412.83	1455.79	1474.44	1673.56	1868.79	1901.95
3019.15	3062.93	3073.08	3086.64	3127.53	3557.22
3636.83	3840.29	3901.63			

- Conformer (332); ZPE = 33541.432 cm^{-1}:

28.53	44.09	63.66	125.86	143.21	216.16
254.37	303.93	334.49	359.97	476.12	488.40
541.05	575.79	585.13	628.68	666.67	744.34
779.47	817.06	875.50	914.44	1020.10	1074.89
1087.23	1148.90	1163.44	1183.62	1197.58	1245.94
1270.36	1308.41	1316.39	1344.53	1372.47	1418.02
1445.58	1460.98	1479.13	1638.51	1869.77	1900.06
2916.76	3023.89	3064.27	3087.20	3112.22	3565.21
3661.02	3840.35	3899.14			

- Conformer (333); ZPE = 33598.651 cm^{-1}:

27.89	41.93	75.01	104.92	127.32	191.36
238.88	290.74	328.47	350.12	441.00	508.19
512.23	590.37	620.06	661.15	709.01	757.24
799.65	849.45	905.25	922.22	1015.36	1054.17
1077.89	1130.72	1163.28	1176.37	1230.06	1239.45
1291.51	1298.67	1320.42	1360.64	1380.00	1401.54
1421.52	1464.43	1482.85	1668.50	1870.36	1895.14
3015.71	3035.94	3053.81	3090.55	3110.95	3535.51
3618.71	3850.33	3890.42			

- Conformer (334); ZPE = 33701.480 cm^{-1}:

57.10	64.67	87.60	129.62	177.78	249.67
268.39	315.02	352.12	420.15	496.63	520.73
542.35	564.23	591.69	649.92	692.64	761.69
784.26	817.63	861.56	926.96	975.11	983.02
1087.47	1124.10	1165.72	1212.34	1218.64	1236.55
1254.12	1307.74	1328.66	1361.91	1383.86	1385.11
1451.31	1466.58	1490.71	1658.20	1841.92	1884.91
3039.25	3047.08	3058.98	3091.06	3130.09	3542.91
3638.40	3842.75	3862.03			

- Conformer (335); ZPE = 33496.791 cm^{-1}:

35.66	45.72	70.73	104.48	114.12	170.26
224.58	259.64	321.96	400.38	472.50	477.94
521.49	576.85	593.12	643.61	671.59	767.76
787.87	823.84	875.41	926.76	958.76	1078.10
1095.44	1156.84	1164.83	1172.18	1196.70	1252.17
1270.66	1309.27	1321.07	1350.50	1363.44	1411.34
1449.64	1456.20	1477.06	1639.94	1870.14	1902.32
3016.90	3020.00	3055.23	3061.99	3109.32	3552.50
3652.00	3846.93	3895.85			

- Conformer (336); ZPE = 33642.153 cm^{-1}:

50.28	69.31	84.22	129.53	184.66	236.67
278.08	310.62	333.30	386.76	479.20	495.70
544.03	562.22	587.18	642.33	671.39	769.04
781.30	831.36	861.45	928.81	962.04	988.78
1101.10	1103.51	1146.38	1178.64	1214.95	1245.23
1247.54	1304.88	1334.68	1357.33	1385.89	1408.04
1431.12	1454.45	1464.97	1646.26	1888.81	1904.17
3033.08	3049.24	3076.04	3085.38	3103.69	3556.90
3651.47	3838.93	3903.36			

- Conformer (337); ZPE = 33580.811 cm^{-1}:

25.67	42.45	89.92	118.84	177.90	243.22
278.37	293.05	332.44	376.23	456.55	492.96
513.70	574.21	586.27	638.72	665.02	766.20
801.04	831.06	864.25	936.95	985.04	1005.45
1057.71	1112.14	1167.63	1175.50	1208.60	1245.58
1287.76	1300.37	1322.44	1365.29	1375.51	1403.23
1422.49	1459.39	1483.14	1660.84	1862.29	1902.56
3019.98	3038.55	3045.00	3097.78	3114.59	3547.23
3644.95	3847.12	3898.46			

- Conformer (338); ZPE = 33668.899 cm^{-1}:

52.84	66.71	74.26	132.96	178.99	206.26
249.52	276.49	313.62	398.90	463.27	474.60
502.32	581.80	597.78	640.82	763.22	786.04
817.11	851.37	888.09	906.63	1000.33	1034.90
1070.39	1123.53	1134.38	1165.10	1215.60	1239.29
1276.12	1306.78	1331.75	1361.57	1385.34	1402.51
1413.65	1471.36	1477.65	1660.80	1880.56	1896.10
3017.44	3054.07	3061.35	3111.89	3139.09	3531.15
3618.09	3835.00	3898.45			

- Conformer (339); ZPE = 33572.879 cm^{-1}:

31.41	40.21	57.46	97.31	158.02	187.05
209.48	285.13	337.81	441.49	445.67	491.99
524.44	599.87	620.85	667.33	724.42	768.45
786.16	803.15	863.28	927.68	951.44	1028.13
1071.74	1123.21	1169.85	1189.69	1230.38	1255.56
1257.85	1300.46	1321.86	1336.99	1365.66	1388.67
1446.99	1475.52	1487.25	1637.47	1871.13	1902.53
3026.09	3029.92	3042.48	3086.59	3123.52	3559.72
3656.15	3844.26	3895.97			

- Conformer (340); ZPE = 33655.486 cm^{-1}:

22.74	39.47	66.55	102.02	163.89	250.93
266.91	286.33	319.61	436.59	458.88	500.11
503.68	577.21	598.13	628.06	741.52	758.60
805.99	843.31	902.26	908.46	993.41	1040.77
1076.18	1121.68	1156.58	1190.11	1209.25	1238.91
1273.56	1306.55	1325.16	1343.12	1366.29	1403.86
1462.09	1471.79	1491.09	1654.37	1869.23	1891.98
2970.60	3036.65	3057.36	3104.75	3138.74	3551.06
3636.19	3846.20	3902.19			

- Conformer (341); ZPE = 33890.229 cm^{-1}:

30.13	72.10	136.62	165.40	177.12	220.37
286.38	324.39	330.08	405.14	474.89	529.89
550.77	603.21	664.38	685.51	743.41	769.70
818.22	841.72	853.54	910.77	960.39	1040.57
1048.71	1144.22	1178.41	1188.95	1237.22	1271.04
1300.05	1315.21	1344.14	1362.45	1394.06	1406.55
1409.44	1466.37	1492.81	1667.15	1847.93	1875.12
3050.90	3050.93	3081.17	3100.29	3108.98	3571.60
3658.35	3709.13	3904.56			

- Conformer (342); ZPE = 33652.165 cm^{-1}:

25.68	48.22	68.54	105.29	175.55	198.61
266.98	335.48	354.88	406.02	451.14	521.15
540.85	586.95	603.97	636.95	725.69	768.50
801.80	804.84	869.30	921.56	974.61	1008.22
1070.99	1119.45	1130.68	1204.29	1209.39	1242.86
1266.42	1309.05	1339.95	1354.52	1374.29	1387.09
1434.76	1474.72	1483.48	1645.41	1868.82	1888.29
3031.05	3058.27	3062.29	3083.81	3133.62	3547.67
3643.98	3845.95	3892.42			

- Conformer (343); ZPE = 33608.307 cm^{-1}:

20.75	33.59	46.18	87.41	134.57	230.05
250.78	294.44	314.45	372.55	441.98	501.17
532.76	586.91	610.57	651.22	739.31	768.48
785.94	848.69	900.76	967.17	975.86	1024.56
1065.55	1108.31	1158.01	1182.34	1219.28	1256.64
1266.55	1311.40	1321.48	1345.99	1374.28	1404.92
1421.02	1472.85	1493.18	1657.94	1868.09	1900.54
3030.18	3046.96	3051.25	3103.26	3127.29	3539.06
3630.90	3846.22	3892.99			

- Conformer (344); ZPE = 33672.427 cm^{-1}:

33.13	64.96	77.07	88.50	174.89	214.09
242.46	308.38	358.65	426.07	440.44	504.48
527.91	573.24	608.14	664.19	698.77	763.35
815.86	825.04	884.15	923.69	976.47	1006.25
1065.86	1155.29	1177.64	1212.19	1226.63	1254.09
1265.22	1315.18	1320.13	1335.52	1383.30	1389.47
1440.79	1458.38	1482.46	1661.07	1876.30	1891.82
2969.59	3036.35	3052.19	3096.60	3144.11	3556.94
3647.36	3845.46	3884.73			

- Conformer (345); ZPE = 33573.297 cm^{-1}:

34.60	39.72	61.82	108.71	165.07	220.84
247.08	321.88	342.45	406.71	447.72	492.97
521.12	587.06	603.44	654.21	695.41	758.09
772.02	831.88	853.76	916.44	963.93	1034.81
1062.32	1136.73	1157.38	1162.22	1213.31	1234.04
1279.50	1298.51	1324.25	1359.82	1375.07	1379.58
1452.67	1469.49	1479.37	1636.50	1875.21	1887.48
3009.85	3045.25	3053.44	3081.48	3120.39	3567.04
3668.12	3843.01	3892.84			

- Conformer (346); ZPE = 33533.875 cm^{-1}:

28.10	47.84	53.47	106.75	170.64	183.16
259.62	280.12	332.80	381.28	473.65	493.98
524.42	566.98	599.10	639.38	659.63	757.87
826.63	852.90	864.97	928.25	963.99	1059.78
1080.05	1144.79	1155.98	1163.69	1206.26	1230.95
1282.55	1302.79	1315.76	1362.09	1371.69	1404.25
1448.14	1453.10	1469.97	1640.13	1880.01	1900.47
3018.07	3032.94	3049.96	3068.98	3105.23	3546.50
3637.66	3844.74	3895.67			

- Conformer (347); ZPE = 33633.708 cm^{-1}:

34.89	61.45	65.77	125.99	166.11	257.72
293.43	327.74	346.42	422.70	449.42	494.09
535.91	582.93	598.63	665.03	674.04	758.78
807.95	826.81	854.74	912.41	958.05	1003.38
1063.72	1136.88	1159.58	1172.08	1222.94	1229.62
1266.74	1311.38	1318.69	1340.71	1370.95	1386.23
1456.49	1464.44	1481.83	1641.72	1874.69	1900.15
3023.08	3038.95	3046.75	3087.95	3111.70	3549.82
3645.57	3845.10	3895.25			

- Conformer (348); ZPE = 33608.196 cm^{-1}:

20.43	46.21	77.82	101.20	141.36	187.20
226.89	292.21	328.56	349.31	439.69	505.60
519.97	590.66	618.24	661.43	719.79	751.16
810.75	845.08	909.73	915.52	1022.39	1044.31
1069.99	1134.52	1165.65	1177.73	1213.07	1251.39
1282.15	1293.58	1333.85	1362.70	1377.60	1398.14
1419.08	1466.53	1485.26	1670.22	1874.34	1896.36
3012.73	3023.43	3047.37	3110.80	3128.07	3534.82
3617.93	3848.07	3895.49			

- Conformer (349); ZPE = 33544.172 cm^{-1}:

27.45	36.98	65.54	110.01	142.82	199.67
234.29	283.82	290.56	349.44	470.01	480.84
543.11	579.80	614.67	626.97	662.27	782.02
801.16	844.90	854.03	912.77	1019.37	1081.50
1110.95	1127.08	1164.68	1169.15	1185.72	1239.22
1278.40	1309.73	1323.43	1345.50	1381.60	1417.00
1456.55	1457.20	1471.70	1653.49	1859.94	1900.09
3018.44	3052.73	3060.65	3065.55	3109.61	3540.69
3633.95	3842.09	3899.18			

- Conformer (350); ZPE = 33602.346 cm^{-1}:

42.21	60.87	98.00	120.18	183.57	261.29
271.92	308.35	366.58	421.78	436.70	483.47
553.43	563.11	576.49	641.71	655.87	747.28
790.40	825.76	878.74	915.08	950.57	1000.34
1063.08	1134.48	1170.70	1187.25	1201.92	1229.16
1259.85	1316.13	1338.96	1361.16	1371.58	1391.10
1448.84	1461.12	1479.47	1654.93	1868.64	1905.40
2938.01	3023.80	3046.69	3104.65	3128.86	3564.14
3655.76	3845.78	3899.50			

- Conformer (351); ZPE = 33743.321 cm^{-1}:

30.13	59.43	94.88	107.76	180.04	259.42
285.52	295.31	330.63	388.09	453.30	526.39
557.02	586.06	609.97	635.89	717.34	778.99
794.10	856.55	902.49	939.24	1000.43	1039.40
1058.44	1130.40	1165.03	1198.52	1206.34	1246.91
1282.26	1300.94	1339.36	1351.56	1370.70	1388.35
1415.36	1472.19	1507.01	1659.78	1873.49	1892.69
3030.33	3033.61	3051.80	3113.15	3145.50	3532.32
3619.41	3823.58	3849.24			

- Conformer (352); ZPE = 33580.999 cm^{-1}:

32.71	41.87	49.97	119.03	170.37	234.04
259.42	280.87	325.48	372.25	479.29	507.93
531.58	567.38	587.22	636.76	651.97	769.51
832.13	850.39	855.28	931.99	962.77	1050.78
1075.38	1121.35	1156.24	1188.92	1203.41	1242.54
1284.75	1305.59	1321.74	1375.30	1379.47	1406.81
1431.67	1453.07	1469.31	1652.96	1869.82	1896.13
3017.51	3047.25	3065.38	3069.12	3103.98	3545.28
3633.83	3849.92	3894.25			

- Conformer (353); ZPE = 33592.026 cm^{-1}:

28.59	40.77	66.08	98.46	149.23	181.93
240.87	281.77	321.77	357.64	440.70	468.23
513.34	609.27	628.48	651.76	717.12	756.57
808.02	839.54	910.53	913.31	1017.70	1046.09
1066.33	1127.94	1168.04	1178.24	1215.66	1241.12
1282.32	1298.13	1331.55	1348.20	1377.01	1386.17
1432.83	1466.56	1485.50	1670.40	1868.66	1902.73
3031.97	3042.08	3061.21	3088.75	3134.71	3539.22
3622.66	3833.74	3894.55			

- Conformer (354); ZPE = 33572.163 cm^{-1}:

19.53	26.85	58.80	105.48	112.93	231.43
242.21	262.33	323.60	400.90	443.95	485.21
525.40	580.71	616.65	657.03	716.22	752.96
792.34	838.26	896.23	953.19	966.38	1018.33
1073.16	1113.81	1177.08	1206.79	1219.66	1257.42
1270.10	1309.14	1334.70	1351.47	1381.92	1396.92
1398.28	1472.39	1488.20	1665.26	1859.31	1899.69
3016.06	3029.12	3039.62	3090.50	3148.04	3548.98
3636.31	3838.41	3895.05			

- Conformer (355); ZPE = 33666.844 cm^{-1}:

47.71	50.00	64.43	160.34	189.77	214.32
220.78	303.39	335.34	393.87	479.72	497.81
535.68	582.70	595.77	642.31	664.53	763.37
807.08	852.89	867.73	936.34	955.18	1038.17
1072.77	1132.82	1151.80	1191.40	1214.15	1232.02
1288.20	1310.58	1320.80	1362.76	1381.96	1414.53
1437.87	1462.55	1468.20	1667.87	1863.96	1889.16
3024.76	3053.22	3067.79	3084.78	3109.36	3552.79
3633.56	3843.88	3900.93			

- Conformer (356); ZPE = 33523.494 cm^{-1}:

26.63	40.39	75.09	90.04	154.89	190.48
250.10	295.21	322.14	424.37	454.23	463.81
552.47	578.32	583.74	639.25	679.87	760.99
796.13	816.24	852.13	931.52	951.70	1031.68
1099.81	1117.73	1158.08	1167.46	1221.94	1240.82
1260.30	1305.30	1325.27	1353.64	1368.64	1411.69
1434.12	1450.60	1475.96	1646.67	1874.09	1899.61
3019.99	3058.99	3061.73	3073.81	3110.97	3559.25
3651.81	3842.43	3894.87			

- Conformer (357); ZPE = 33588.056 cm^{-1}:

19.66	41.64	70.98	103.38	131.56	196.51
253.05	306.40	327.38	344.78	439.98	508.61
514.32	591.33	620.00	660.27	714.64	758.94
799.89	850.09	870.82	907.91	1029.40	1062.45
1072.04	1152.14	1167.14	1170.10	1230.39	1237.69
1276.40	1303.73	1325.89	1342.80	1371.44	1400.64
1450.29	1464.09	1482.62	1643.08	1869.68	1900.08
2992.19	3033.68	3045.36	3090.49	3109.28	3540.38
3633.82	3850.62	3896.06			

- Conformer (358); ZPE = 33548.881 cm^{-1}:

21.40	36.85	85.82	120.17	166.43	229.90
243.86	293.76	308.19	386.77	431.43	487.89
541.15	573.88	600.58	638.43	665.60	770.33
810.64	834.45	853.43	927.32	967.26	1001.65
1057.34	1114.38	1171.28	1183.39	1208.36	1239.55
1291.57	1302.94	1323.02	1373.71	1378.20	1383.47
1398.03	1456.31	1489.42	1664.71	1870.40	1902.62
3008.09	3045.62	3075.74	3090.61	3116.63	3556.62
3648.04	3848.96	3901.55			

- Conformer (359); ZPE = 33635.034 cm^{-1}:

22.37	34.26	55.43	84.55	167.87	179.28
225.64	278.38	330.11	424.17	446.01	499.17
550.15	594.99	616.87	666.51	720.28	767.18
793.90	831.95	884.48	920.82	957.49	1037.38
1054.28	1149.24	1160.30	1202.58	1218.43	1244.97
1275.58	1310.83	1325.39	1355.76	1378.93	1388.73
1405.47	1465.28	1481.16	1670.81	1868.66	1902.51
3036.87	3050.36	3085.11	3102.65	3125.21	3559.34
3637.62	3839.12	3885.63			

- Conformer (360); ZPE = 33680.174 cm^{-1}:

25.53	30.03	60.43	108.65	168.48	194.23
252.66	276.06	347.68	434.25	445.48	520.98
539.48	565.55	619.76	655.41	718.60	760.61
779.94	842.33	902.10	919.18	965.32	1039.46
1062.34	1158.68	1166.84	1188.24	1218.74	1252.24
1280.67	1310.95	1329.96	1351.87	1383.15	1392.47
1410.56	1464.39	1482.07	1672.75	1864.41	1904.22
3034.23	3037.80	3056.03	3110.67	3130.89	3555.34
3634.29	3843.24	3891.09			

- Conformer (361); ZPE = 33844.343 cm^{-1}:

39.89	62.73	107.33	155.88	184.49	208.17
316.56	333.81	388.04	416.72	466.96	534.37
585.90	591.66	617.21	725.70	748.54	767.65
784.67	799.17	866.51	938.37	969.57	996.40
1042.94	1129.02	1167.76	1188.45	1209.67	1270.53
1291.62	1318.84	1348.49	1361.91	1375.83	1389.97
1418.87	1474.92	1482.03	1636.29	1892.18	1900.65
3031.86	3054.55	3058.66	3115.94	3136.92	3544.69
3647.79	3711.17	3880.85			

- Conformer (362); ZPE = 33563.975 cm^{-1}:

17.42	48.24	87.01	96.22	109.45	243.69
255.59	278.14	331.41	408.78	451.72	470.58
541.34	578.37	603.96	638.96	668.51	750.74
779.50	851.91	890.44	915.93	970.58	1078.54
1088.48	1159.46	1175.53	1186.73	1221.26	1253.89
1271.66	1311.08	1330.71	1347.59	1366.12	1397.37
1449.93	1461.21	1478.04	1657.90	1875.71	1894.94
2938.52	3023.71	3043.66	3087.14	3104.49	3560.07
3642.33	3844.24	3889.17			

- Conformer (363); ZPE = 33585.958 cm^{-1}:

27.19	30.11	55.85	89.04	140.82	188.82
264.82	291.17	319.42	369.06	441.00	483.27
537.41	602.96	610.61	632.32	727.74	761.22
816.93	827.81	889.27	911.96	1012.92	1026.92
1059.32	1115.03	1160.70	1188.86	1218.42	1249.50
1279.11	1316.21	1344.67	1347.21	1373.87	1389.41
1402.61	1469.15	1485.04	1662.08	1869.18	1901.49
3033.17	3043.17	3070.26	3090.38	3121.40	3546.55
3634.97	3846.32	3895.19			

- Conformer (364); ZPE = 33611.150 cm^{-1}:

26.05	42.94	76.62	98.39	146.55	191.63
277.67	308.09	329.72	347.49	441.66	504.52
511.69	589.33	617.45	660.81	717.49	753.47
808.81	844.69	874.54	909.49	1016.30	1060.42
1085.66	1152.49	1158.58	1173.38	1228.87	1243.19
1272.54	1296.13	1332.01	1344.99	1373.16	1395.08
1447.79	1466.30	1483.81	1643.83	1874.87	1900.17
2985.65	3024.72	3045.75	3096.77	3123.42	3541.62
3634.60	3847.40	3893.73			

- Conformer (365); ZPE = 33621.073 cm^{-1}:

36.25	41.15	68.63	114.13	164.86	229.95
261.73	310.50	343.54	399.91	456.13	521.61
539.48	585.73	593.50	636.04	698.86	767.30
803.37	837.12	847.39	918.40	961.77	1032.29
1058.52	1107.40	1152.71	1170.63	1222.71	1241.20
1277.60	1307.46	1326.92	1370.86	1375.63	1380.23
1426.32	1467.77	1479.80	1649.64	1878.67	1889.94
3011.03	3046.95	3072.68	3083.95	3120.14	3558.00
3652.42	3851.14	3892.18			

- Conformer (366); ZPE = 33536.683 cm^{-1}:

24.91	45.64	63.35	101.00	124.90	185.04
245.75	250.77	334.11	398.74	434.67	488.31
526.16	578.00	616.57	672.41	709.40	753.44
795.25	822.32	880.11	924.71	951.58	1068.21
1075.72	1140.60	1169.04	1195.09	1229.64	1240.32
1279.96	1300.44	1321.77	1357.63	1376.35	1397.11
1432.72	1460.54	1481.61	1639.33	1874.19	1901.12
2967.29	3035.07	3048.96	3087.25	3129.67	3550.24
3651.32	3846.65	3888.38			

- Conformer (367); ZPE = 33536.142 cm^{-1}:

22.50	46.64	62.35	104.82	111.91	207.13
244.08	252.75	329.47	391.28	434.33	484.23
529.71	583.48	617.54	672.79	716.82	772.94
777.40	819.01	874.45	925.00	965.72	1059.32
1071.51	1149.80	1162.53	1184.95	1230.60	1245.91
1267.92	1313.27	1318.28	1349.64	1375.11	1400.65
1432.50	1462.01	1480.45	1633.55	1871.90	1901.49
2977.78	3028.30	3056.65	3087.57	3126.81	3549.84
3651.15	3845.94	3890.49			

- Conformer (368); ZPE = 33529.410 cm^{-1}:

28.14	42.94	49.23	107.54	179.16	233.94
243.66	303.53	326.00	369.61	473.03	507.43
530.65	564.76	584.54	632.25	650.87	767.44
778.17	807.74	861.74	917.30	975.11	1008.81
1085.66	1119.59	1147.69	1170.29	1217.06	1236.08
1267.01	1299.32	1330.02	1368.43	1381.76	1402.70
1428.07	1458.15	1467.92	1640.81	1883.00	1899.33
3030.42	3039.44	3071.56	3094.29	3113.64	3555.01
3656.68	3852.65	3898.66			

- Conformer (369); ZPE = 33627.114 cm^{-1}:

42.03	53.25	72.64	114.42	175.99	262.16
293.80	333.23	379.94	432.68	451.06	489.14
509.30	555.30	581.85	630.42	678.24	750.49
792.39	846.07	873.96	919.33	967.94	1006.56
1065.92	1145.31	1174.74	1177.46	1209.12	1234.76
1252.43	1309.22	1322.08	1340.28	1378.87	1409.86
1437.66	1452.11	1478.33	1629.61	1880.30	1896.95
2940.95	3041.48	3053.88	3094.34	3102.39	3564.56
3664.61	3884.73	3900.07			

- Conformer (370); ZPE = 33581.899 cm^{-1}:

12.67	39.84	61.32	120.71	140.68	197.04
273.23	274.33	295.60	384.64	446.29	474.71
526.96	596.47	621.03	630.60	700.18	775.74
790.84	838.07	900.26	905.89	1020.06	1045.73
1066.38	1135.69	1153.69	1180.87	1207.98	1245.94
1293.15	1305.17	1330.13	1346.61	1380.64	1411.80
1424.49	1463.19	1476.17	1655.41	1866.78	1902.30
3027.17	3038.77	3048.01	3088.33	3134.52	3539.40
3630.15	3840.64	3897.52			

- Conformer (371); ZPE = 33625.242 cm^{-1}:

24.06	35.51	69.87	132.74	168.85	237.32
278.93	307.31	362.36	441.39	483.49	507.05
524.29	554.69	588.65	659.77	691.94	752.76
799.68	823.19	850.90	913.80	959.55	1012.32
1064.80	1128.26	1160.59	1190.69	1221.81	1233.69
1263.81	1315.08	1319.84	1360.00	1369.32	1386.57
1448.59	1469.18	1475.56	1659.16	1852.64	1892.84
3007.71	3029.97	3048.81	3105.73	3120.61	3561.25
3651.43	3841.32	3890.78			

- Conformer (372); ZPE = 33641.319 cm^{-1}:

59.70	65.20	81.75	118.90	169.73	214.97
226.71	271.55	324.96	413.18	458.26	471.85
502.21	581.68	604.81	632.55	759.41	771.39
804.84	836.91	887.52	901.35	993.25	1048.86
1067.67	1120.97	1153.82	1189.33	1230.83	1238.87
1258.26	1307.70	1322.08	1339.55	1383.33	1430.90
1449.62	1466.04	1482.14	1654.78	1839.68	1901.12
2941.41	3059.10	3072.32	3114.86	3138.75	3548.25
3637.70	3841.99	3890.03			

- Conformer (373); ZPE = 33472.397 cm^{-1}:

31.67	39.85	75.63	93.97	113.99	213.19
233.54	260.53	312.13	384.75	462.42	472.80
546.30	578.55	586.67	629.99	665.08	773.21
792.47	820.04	861.80	924.09	954.85	1072.86
1092.82	1109.48	1155.40	1160.52	1206.87	1254.41
1263.60	1305.78	1327.50	1356.43	1369.11	1408.16
1428.96	1455.85	1478.50	1645.79	1878.17	1901.66
3018.11	3048.27	3059.49	3069.87	3101.89	3552.56
3649.23	3848.53	3897.40			

- Conformer (374); ZPE = 33761.223 cm^{-1}:

45.49	66.15	90.96	107.97	180.51	244.45
302.08	323.66	346.29	414.42	452.09	498.55
542.56	579.22	642.52	677.05	744.60	778.23
804.61	856.35	907.19	924.08	964.03	1008.87
1069.72	1118.58	1157.56	1173.69	1218.33	1245.58
1275.42	1303.15	1353.76	1366.52	1378.54	1408.62
1415.16	1460.75	1462.32	1654.75	1900.10	1919.37
3036.37	3045.62	3054.63	3079.01	3111.39	3544.80
3627.82	3738.48	3900.51			

- Conformer (375); ZPE = 33744.873 cm^{-1}:

51.87	81.05	90.78	104.74	168.78	210.01
243.92	294.98	315.31	376.28	453.03	526.97
583.49	599.75	625.69	682.40	731.29	772.72
798.86	824.06	864.29	923.58	998.91	1043.20
1068.94	1105.39	1160.10	1176.09	1226.80	1264.85
1294.49	1300.12	1336.19	1359.93	1372.58	1391.01
1417.23	1479.73	1494.60	1651.48	1895.89	1903.24
3021.03	3045.20	3086.26	3103.74	3140.92	3548.55
3645.45	3805.73	3828.25			

- Conformer (376); ZPE = 33530.964 cm^{-1}:

25.71	46.08	68.28	98.33	120.52	201.33
240.59	252.20	324.52	394.02	441.59	479.47
519.95	593.46	616.93	658.42	712.39	764.07
790.68	816.88	872.69	924.70	953.91	1061.50
1070.71	1134.87	1161.96	1177.54	1225.38	1247.45
1274.62	1303.41	1323.01	1351.11	1366.42	1384.16
1450.98	1466.57	1483.33	1636.73	1874.78	1900.86
3020.01	3030.42	3038.16	3083.20	3128.45	3555.83
3655.82	3844.32	3893.59			

- Conformer (377); ZPE = 33733.309 cm^{-1}:

21.04	57.67	61.02	114.26	169.41	199.80
280.44	298.53	372.51	439.33	476.45	480.10
548.75	575.33	626.27	714.79	746.88	782.35
790.20	841.61	852.51	917.48	967.25	1025.49
1086.43	1130.67	1170.41	1199.56	1237.09	1240.97
1273.13	1313.53	1324.71	1354.52	1393.74	1397.27
1431.60	1460.46	1474.86	1654.37	1894.05	1909.41
3020.38	3032.44	3049.63	3101.19	3128.31	3554.09
3647.07	3724.49	3902.79			

- Conformer (378); ZPE = 33490.902 cm^{-1}:

37.63	61.43	64.54	125.31	138.30	184.44
213.25	286.39	291.93	361.78	482.88	502.69
523.83	547.26	610.34	647.65	660.64	751.97
785.14	803.53	884.22	912.66	1018.66	1069.23
1084.30	1133.33	1159.42	1174.07	1203.22	1244.56
1274.01	1298.25	1310.00	1332.82	1385.96	1440.02
1445.41	1456.73	1472.95	1655.35	1864.91	1908.15
2910.43	3040.90	3057.23	3099.13	3117.96	3556.23
3646.42	3849.35	3895.02			

- Conformer (379); ZPE = 33524.663 cm^{-1}:

24.64	47.53	68.11	98.73	113.56	228.16
242.44	256.40	322.62	388.12	440.36	483.92
523.18	598.54	618.35	657.51	710.92	760.51
784.44	792.85	866.04	921.24	962.08	1055.95
1069.87	1144.84	1149.90	1174.79	1225.51	1248.93
1264.11	1316.14	1318.05	1344.49	1368.62	1383.83
1453.33	1466.63	1482.57	1630.80	1871.81	1901.81
3017.39	3036.81	3045.24	3083.70	3123.47	3557.44
3662.44	3844.31	3896.31			

- Conformer (380); ZPE = 33781.397 cm^{-1}:

48.42	52.10	84.74	150.54	213.92	266.01
293.55	315.11	345.57	370.54	511.44	524.26
578.75	587.64	595.56	629.76	656.08	782.24
807.41	834.91	850.16	930.88	942.69	992.63
1073.24	1152.42	1165.34	1176.13	1205.06	1239.83
1280.63	1308.65	1323.96	1359.24	1372.28	1388.66
1412.03	1442.54	1516.54	1669.16	1883.27	1892.49
3039.00	3062.94	3090.99	3107.55	3130.71	3554.63
3646.76	3847.82	3856.04			

- Conformer (381); ZPE = 33619.447 cm^{-1}:

31.13	66.92	79.29	131.51	163.29	244.50
266.86	291.28	315.49	383.42	439.23	471.25
514.87	582.88	595.07	650.10	712.73	764.63
803.79	848.46	865.67	909.57	1001.52	1050.30
1056.16	1138.99	1173.35	1184.52	1211.09	1238.11
1254.27	1313.24	1331.92	1355.54	1368.13	1372.26
1465.59	1469.22	1481.97	1641.02	1865.52	1888.87
3024.70	3049.87	3063.84	3090.09	3120.12	3540.11
3634.95	3826.36	3895.31			

- Conformer (382); ZPE = 33528.395 cm^{-1}:

36.24	55.11	63.58	114.17	162.11	211.24
226.82	311.94	362.76	452.42	474.12	480.42
503.82	525.60	580.32	615.70	669.77	769.70
796.25	836.21	868.94	938.05	959.57	1030.41
1098.74	1141.62	1168.94	1180.89	1201.84	1221.51
1278.84	1308.76	1318.58	1323.72	1364.45	1419.59
1431.60	1458.18	1484.98	1631.81	1894.22	1894.99
2961.21	3020.18	3043.44	3072.08	3099.38	3559.02
3657.33	3883.85	3891.75			

- Conformer (383); ZPE = 33616.908 cm^{-1}:

32.73	43.47	70.05	113.16	177.47	257.30
283.91	304.54	317.96	365.49	467.00	485.34
537.54	583.72	610.27	642.69	669.20	783.92
789.12	829.84	858.77	901.60	988.43	1040.77
1096.77	1135.12	1157.10	1175.81	1211.57	1227.82
1260.50	1312.15	1332.44	1348.46	1370.36	1379.56
1464.61	1472.93	1491.05	1642.16	1868.15	1903.22
3009.09	3046.50	3053.68	3076.62	3124.22	3543.75
3639.23	3841.20	3895.47			

- Conformer (384); ZPE = 33566.878 cm^{-1}:

31.44	42.48	60.63	101.90	150.67	184.01
241.11	301.40	318.44	358.00	442.83	460.36
512.14	604.63	627.90	650.15	729.62	759.43
806.41	838.35	862.80	913.72	1013.95	1051.97
1074.49	1139.04	1151.87	1172.47	1224.80	1238.55
1273.58	1297.85	1334.69	1343.05	1360.11	1377.17
1465.16	1468.60	1487.87	1642.80	1873.54	1902.92
3025.60	3037.38	3040.24	3083.71	3135.17	3545.60
3638.71	3837.54	3896.88			

- Conformer (385); ZPE = 33810.619 cm^{-1}:

31.47	48.23	90.72	135.08	180.54	241.53
296.81	340.99	369.36	413.89	448.91	514.37
575.20	595.23	617.87	703.18	741.08	792.03
826.58	853.03	872.33	936.24	977.24	1013.37
1063.48	1145.75	1157.67	1194.93	1200.65	1264.92
1307.40	1316.44	1346.89	1359.57	1379.34	1391.33
1431.72	1464.68	1481.52	1638.71	1888.22	1895.97
3027.75	3037.80	3048.24	3122.68	3138.05	3534.98
3635.79	3644.22	3887.24			

- Conformer (386); ZPE = 33638.807 cm^{-1}:

46.51	47.85	57.96	127.17	182.99	256.41
305.51	319.06	323.85	388.61	463.97	466.69
487.17	538.98	579.38	644.48	667.63	746.51
825.65	863.66	906.32	951.58	994.44	1011.28
1071.81	1137.21	1159.08	1196.93	1207.70	1243.63
1279.33	1311.38	1327.60	1343.81	1385.89	1409.44
1413.06	1447.26	1470.12	1668.08	1887.67	1903.65
2990.26	3039.16	3046.57	3088.91	3096.82	3530.57
3616.20	3892.06	3909.75			

- Conformer (387); ZPE = 33524.912 cm^{-1}:

31.96	47.52	57.65	102.81	138.24	167.56
206.76	284.00	341.36	431.43	458.60	477.03
501.36	533.77	580.73	658.80	665.03	777.19
793.49	848.89	907.29	927.49	952.67	1054.43
1088.79	1158.44	1167.45	1173.82	1197.76	1243.98
1268.09	1292.98	1322.15	1341.67	1361.81	1419.96
1433.22	1459.22	1471.90	1674.28	1887.55	1896.79
3020.24	3039.43	3054.78	3070.83	3113.38	3537.62
3625.35	3888.20	3894.10			

- Conformer (388); ZPE = 33779.905 cm^{-1}:

42.59	68.56	89.73	117.64	206.65	231.84
293.83	313.55	373.04	400.35	458.46	518.02
561.74	580.58	616.37	689.64	753.95	773.36
831.21	840.46	861.52	928.30	967.13	1025.84
1056.53	1139.74	1172.65	1176.99	1202.40	1247.28
1289.84	1305.36	1337.55	1354.06	1385.81	1405.13
1428.21	1462.68	1476.20	1649.03	1885.38	1900.97
3033.13	3037.98	3064.18	3125.92	3144.70	3558.60
3630.24	3659.80	3885.09			

- Conformer (389); ZPE = 33556.024 cm^{-1}:

15.97	47.20	59.16	126.88	148.35	202.04
273.37	281.88	307.20	403.37	446.02	481.66
521.69	572.98	614.75	628.00	713.42	755.62
776.49	825.84	897.35	907.62	1010.67	1058.92
1072.39	1121.72	1156.50	1191.31	1220.11	1251.92
1271.94	1302.41	1324.07	1342.55	1356.38	1402.64
1456.74	1465.21	1481.72	1652.09	1878.94	1904.02
2935.49	3025.59	3027.93	3097.75	3150.45	3557.39
3645.25	3843.51	3899.58			

- Conformer (390); ZPE = 33527.700 cm^{-1}:

26.09	26.32	63.77	96.45	111.78	231.17
240.50	255.26	318.18	387.49	442.64	475.49
542.39	588.78	606.29	646.02	715.75	774.13
789.99	826.14	861.79	926.09	952.64	1058.75
1072.14	1114.37	1131.31	1199.57	1223.94	1248.37
1268.61	1302.72	1336.76	1354.69	1370.11	1383.34
1434.69	1469.24	1485.67	1649.72	1872.86	1900.98
3023.43	3034.50	3061.18	3080.05	3127.50	3557.05
3650.78	3845.90	3892.04			

- Conformer (391); ZPE = 33610.308 cm^{-1}:

60.53	67.64	77.54	136.92	179.69	215.84
220.51	273.55	324.81	402.79	470.69	479.77
497.49	577.68	593.92	629.18	754.70	765.94
796.41	830.03	888.14	891.14	981.55	1050.67
1073.60	1126.45	1143.68	1178.15	1219.62	1237.66
1267.21	1301.92	1326.27	1337.21	1386.38	1405.01
1448.61	1467.21	1475.03	1655.49	1883.22	1910.94
2930.62	3055.70	3056.51	3113.26	3136.08	3550.13
3640.38	3837.27	3889.87			

- Conformer (392); ZPE = 33512.068 cm^{-1}:

30.52	51.68	71.87	107.77	112.67	206.01
230.18	263.35	311.05	398.11	466.54	481.08
503.06	519.46	576.71	641.56	685.98	746.38
797.74	869.02	915.10	956.79	984.55	1026.56
1101.07	1144.13	1157.96	1171.56	1197.78	1258.37
1279.42	1293.69	1328.02	1339.53	1370.65	1412.67
1417.76	1458.34	1476.28	1673.27	1895.71	1897.89
3008.35	3027.97	3048.63	3079.39	3098.93	3534.41
3613.82	3889.66	3895.14			

- Conformer (393); ZPE = 33648.548 cm^{-1}:

42.09	62.96	78.91	125.42	198.30	251.35
282.30	328.69	340.28	386.44	476.09	490.48
503.90	550.27	581.69	647.87	664.67	763.51
803.60	842.05	898.28	928.84	960.79	1013.86
1067.78	1142.76	1166.34	1183.75	1204.61	1229.35
1269.85	1297.93	1317.74	1359.37	1389.20	1412.20
1445.55	1451.26	1463.09	1651.90	1879.47	1900.54
2935.00	3046.68	3067.55	3098.52	3122.77	3544.11
3638.14	3892.54	3896.47			

- Conformer (394); ZPE = 33747.272 cm^{-1}:

55.75	95.46	119.96	126.82	137.75	209.37
233.66	268.19	333.50	395.49	453.12	482.99
555.51	601.22	664.34	716.27	754.15	758.38
791.42	841.28	899.47	944.34	954.53	1016.91
1063.42	1124.18	1175.47	1194.56	1214.83	1267.22
1296.18	1305.60	1318.83	1351.83	1395.81	1403.90
1446.80	1478.70	1486.53	1658.85	1874.51	1891.68
2935.65	3055.24	3071.92	3097.46	3147.23	3556.74
3644.65	3737.13	3889.78			

- Conformer (395); ZPE = 33531.537 cm^{-1}:

19.51	42.80	61.64	84.42	166.14	182.21
246.88	291.37	324.22	416.86	444.86	464.51
553.47	584.79	612.09	665.41	717.40	747.67
789.80	799.95	844.96	923.17	953.24	1019.49
1074.20	1111.02	1145.10	1201.10	1235.28	1236.99
1254.72	1309.01	1318.07	1354.87	1366.71	1385.56
1438.91	1462.55	1479.37	1644.73	1875.03	1904.43
3028.50	3034.79	3073.84	3090.43	3125.89	3560.93
3657.68	3840.73	3895.74			

- Conformer (396); ZPE = 33675.070 cm^{-1}:

49.33	74.88	99.94	131.48	178.02	198.46
239.49	317.95	379.83	437.34	453.06	496.11
529.43	538.18	599.36	671.74	720.56	770.35
780.54	842.46	884.12	911.85	963.80	1005.07
1081.85	1134.96	1173.41	1181.33	1221.94	1248.84
1260.19	1312.73	1313.07	1333.47	1373.28	1396.36
1441.80	1472.05	1491.56	1640.16	1860.32	1883.86
3003.60	3042.34	3055.99	3092.21	3113.68	3559.21
3656.04	3853.70	3878.87			

- Conformer (397); ZPE = 33519.443 cm^{-1}:

21.93	39.61	70.06	93.87	113.62	230.38
244.62	260.79	315.14	380.70	438.81	479.66
548.69	585.22	606.68	644.50	710.02	762.78
792.85	808.86	858.99	919.91	959.14	1056.41
1071.16	1104.95	1143.26	1191.04	1225.67	1252.76
1259.81	1317.53	1327.91	1353.24	1369.31	1384.91
1427.08	1468.56	1483.46	1643.86	1883.78	1901.83
3015.77	3037.32	3072.58	3084.43	3120.74	3555.42
3653.23	3848.66	3897.38			

- Conformer (398); ZPE = 33518.034 cm^{-1}:

39.46	49.33	77.83	92.10	161.17	193.50
240.46	296.45	341.86	433.50	461.80	494.88
517.51	536.74	579.07	659.74	670.45	766.03
791.90	805.73	867.21	935.49	951.49	1030.30
1095.60	1148.95	1166.41	1173.62	1212.17	1243.67
1262.97	1303.39	1320.49	1325.24	1367.13	1414.81
1438.56	1454.53	1479.48	1634.27	1893.26	1896.82
2991.92	3025.41	3048.32	3065.26	3097.61	3554.02
3651.87	3884.02	3892.26			

- Conformer (399); ZPE = 33727.469 cm^{-1}:

20.62	75.24	105.50	112.81	192.60	215.34
226.95	258.20	280.57	367.97	444.42	552.72
601.29	626.06	636.21	676.72	732.88	767.55
794.40	825.12	858.84	944.66	959.01	994.70
1077.30	1102.78	1145.87	1190.18	1231.85	1259.20
1283.11	1313.69	1335.43	1354.91	1385.42	1387.32
1425.40	1479.14	1484.83	1649.53	1897.53	1904.99
3037.62	3063.77	3089.49	3096.94	3145.13	3554.80
3647.31	3814.39	3826.64			

- Conformer (400); ZPE = 33566.443 cm^{-1}:

37.35	73.01	83.57	105.47	155.49	229.89
245.24	272.07	306.97	401.16	437.28	479.03
523.91	587.35	591.55	608.80	718.96	769.02
805.63	828.15	859.41	911.96	992.91	1049.06
1057.34	1098.79	1158.63	1186.45	1204.88	1238.87
1259.18	1308.27	1340.11	1362.86	1368.08	1376.49
1427.86	1470.43	1474.68	1649.36	1889.27	1904.14
3029.42	3050.16	3077.49	3094.00	3114.55	3543.83
3636.61	3836.72	3901.16			

- Conformer (401); ZPE = 33640.930 cm^{-1}:

37.02	77.16	84.71	120.94	170.00	210.46
265.74	277.97	323.31	421.86	473.27	489.87
499.58	591.25	600.02	631.54	740.13	770.78
802.87	840.90	885.87	906.92	980.64	1045.09
1068.70	1133.58	1140.54	1177.12	1216.79	1243.91
1261.42	1310.50	1329.49	1334.86	1372.00	1399.97
1455.38	1467.71	1482.62	1653.79	1888.96	1911.29
2923.01	3029.72	3054.83	3113.11	3129.97	3551.26
3640.03	3842.33	3901.07			

- Conformer (402); ZPE = 33606.956 cm^{-1}:

30.16	43.17	63.30	99.91	132.03	206.16
248.28	266.77	318.52	396.65	449.50	499.11
519.45	540.73	619.77	672.14	719.60	780.42
781.32	867.76	906.87	958.51	973.97	1035.23
1065.43	1118.78	1165.67	1179.85	1226.44	1248.47
1265.12	1304.02	1328.27	1339.69	1358.35	1385.57
1423.89	1475.97	1495.09	1672.73	1896.33	1900.03
3018.69	3032.71	3052.19	3081.38	3124.46	3530.76
3619.56	3884.52	3890.59			

- Conformer (403); ZPE = 33462.419 cm^{-1}:

34.23	45.90	85.01	101.39	130.64	194.23
205.10	285.65	321.80	339.62	450.77	465.54
510.39	556.10	580.68	654.98	663.34	753.58
810.53	842.49	900.46	911.41	1017.62	1046.30
1090.23	1138.62	1160.85	1163.97	1183.87	1242.62
1281.57	1300.19	1319.76	1351.18	1357.90	1408.59
1420.77	1455.67	1477.19	1666.04	1897.03	1903.57
3004.58	3009.08	3054.45	3059.88	3123.58	3536.72
3621.46	3892.06	3895.65			

- Conformer (404); ZPE = 33648.994 cm^{-1}:

52.41	63.20	82.12	116.03	175.50	179.84
216.29	281.01	359.47	430.62	453.64	477.07
500.83	606.29	623.08	653.78	729.53	776.57
809.85	859.72	909.81	930.48	952.31	1017.62
1054.60	1141.11	1147.89	1175.24	1216.13	1253.38
1275.90	1289.80	1303.54	1356.08	1358.56	1387.44
1409.85	1481.66	1491.69	1673.62	1898.22	1903.99
3023.71	3025.75	3048.89	3119.63	3143.90	3539.45
3616.93	3830.96	3873.00			

- Conformer (405); ZPE = 33485.946 cm^{-1}:

31.30	38.39	80.47	96.91	139.78	196.36
263.80	298.74	322.72	338.82	454.43	460.42
497.48	550.77	584.50	654.23	664.01	756.74
811.58	850.48	878.55	910.96	1018.37	1081.33
1087.44	1148.94	1160.61	1176.07	1184.34	1239.06
1268.85	1306.63	1319.81	1333.39	1354.45	1410.48
1446.76	1455.74	1476.43	1644.57	1899.46	1901.67
2987.93	3009.47	3053.79	3058.67	3107.20	3537.87
3628.73	3896.03	3896.34			

- Conformer (406); ZPE = 33580.361 cm^{-1}:

21.06	43.06	66.13	103.83	122.70	202.97
256.34	294.31	321.49	359.58	443.07	496.62
507.44	527.10	632.30	659.65	733.97	761.03
807.25	846.89	900.52	919.12	1010.70	1052.16
1062.53	1117.00	1166.93	1167.93	1221.81	1244.73
1288.88	1298.03	1316.98	1357.73	1362.46	1384.77
1418.02	1468.96	1485.41	1665.66	1889.23	1902.19
3019.99	3029.39	3038.23	3102.04	3117.64	3537.39
3624.62	3889.40	3893.51			

- Conformer (407); ZPE = 33574.492 cm^{-1}:

40.54	59.80	71.30	104.47	181.39	281.32
300.55	319.86	344.25	422.90	455.46	473.16
498.86	540.47	565.83	614.21	650.95	733.41
808.93	847.16	893.89	921.92	980.18	1055.50
1066.20	1153.59	1174.39	1181.85	1222.78	1238.59
1266.64	1294.86	1313.71	1330.02	1392.78	1405.82
1422.97	1450.17	1486.43	1624.55	1889.98	1903.90
2908.60	3009.07	3054.03	3070.02	3119.46	3557.73
3652.88	3895.75	3895.92			

- Conformer (408); ZPE = 33598.570 cm^{-1}:

36.76	50.54	64.95	123.78	163.75	241.21
280.69	308.57	344.60	419.48	436.20	464.00
504.90	533.17	585.20	653.47	686.84	759.17
824.03	840.77	886.66	922.42	966.53	1027.24
1054.12	1148.04	1161.82	1177.15	1211.27	1229.17
1276.96	1292.27	1327.89	1342.32	1381.14	1386.96
1425.57	1467.75	1471.12	1682.54	1893.08	1902.97
3026.26	3039.63	3047.24	3082.38	3107.82	3524.08
3620.95	3888.71	3903.03			

- Conformer (409); ZPE = 33584.479 cm^{-1}:

39.49	60.87	68.17	127.92	168.05	263.44
295.94	332.80	351.46	415.29	446.03	491.65
494.03	543.85	584.26	667.03	678.96	757.31
816.56	827.94	860.29	911.86	958.10	998.09
1065.29	1139.66	1154.89	1170.77	1221.55	1233.72
1257.93	1307.19	1311.19	1331.37	1369.45	1388.41
1445.18	1462.23	1478.35	1638.76	1899.55	1906.04
2990.73	3024.24	3047.43	3067.20	3119.61	3544.90
3640.75	3896.24	3896.95			

- Conformer (410); ZPE = 33623.525 cm^{-1}:

54.23	71.38	86.53	97.77	170.91	246.26
279.75	295.35	348.14	420.60	432.32	460.18
501.07	548.23	584.27	623.81	700.83	748.20
788.24	859.46	899.83	939.81	978.30	1003.42
1076.35	1143.35	1153.60	1165.25	1226.06	1231.95
1263.55	1294.19	1325.41	1351.73	1369.86	1388.75
1417.67	1469.17	1478.13	1686.95	1892.94	1914.12
3018.22	3027.47	3057.63	3096.66	3139.27	3519.89
3613.50	3886.38	3900.10			

- Conformer (411); ZPE = 33558.412 cm^{-1}:

16.67	44.01	83.15	99.27	142.88	190.07
238.97	293.66	326.79	351.28	443.85	480.49
517.26	523.47	627.11	660.89	699.41	747.60
805.90	848.40	907.85	920.38	1020.65	1043.29
1070.56	1136.02	1167.70	1176.39	1210.06	1242.33
1289.64	1293.91	1332.79	1350.28	1367.42	1386.09
1421.56	1469.15	1482.98	1670.85	1895.15	1904.37
3014.03	3014.76	3037.44	3084.50	3120.00	3533.52
3617.56	3893.77	3900.71			

- Conformer (412); ZPE = 33684.544 cm^{-1}:

33.53	75.12	91.24	144.48	186.17	240.65
250.80	291.04	314.76	387.09	442.48	503.39
526.51	571.19	609.98	662.81	742.28	765.86
789.99	859.15	892.66	920.27	1003.34	1031.39
1065.36	1115.63	1137.43	1162.43	1205.14	1262.62
1283.29	1291.35	1318.53	1346.24	1364.46	1391.27
1417.63	1477.19	1482.55	1668.03	1886.27	1914.16
3005.34	3048.07	3055.65	3112.73	3117.56	3539.76
3625.01	3856.06	3883.14			

- Conformer (413); ZPE = 33511.574 cm^{-1}:

31.12	38.77	72.92	98.81	148.12	188.74
244.63	307.07	325.30	354.96	446.40	468.27
499.12	518.05	626.76	660.85	728.18	750.53
806.51	842.93	867.03	911.31	1012.59	1051.82
1078.50	1140.20	1154.75	1171.96	1223.29	1238.06
1270.94	1292.11	1326.35	1337.91	1351.40	1375.77
1448.45	1470.87	1487.82	1642.99	1899.87	1909.29
2983.98	3014.02	3026.28	3084.90	3122.38	3541.78
3634.76	3896.04	3897.68			

- Conformer (414); ZPE = 33477.912 cm^{-1}:

22.96	47.66	61.87	102.85	118.43	210.24
246.34	248.73	328.87	390.73	440.32	482.78
491.39	526.81	617.06	673.99	709.39	760.53
787.59	823.19	878.14	923.64	950.79	1061.77
1067.49	1134.37	1158.45	1186.78	1224.07	1241.17
1275.26	1299.36	1317.33	1345.36	1359.59	1382.79
1435.19	1463.82	1481.16	1638.69	1902.56	1906.92
2974.35	3015.69	3033.49	3078.87	3128.03	3549.29
3650.09	3894.51	3905.09			

- Conformer (415); ZPE = 33503.098 cm^{-1}:

10.54	55.53	105.60	124.43	174.02	239.21
278.38	304.02	358.80	399.50	420.07	490.64
519.80	548.04	577.09	614.53	666.97	760.89
779.47	822.93	867.31	921.45	949.52	977.97
1096.71	1140.11	1152.42	1196.88	1216.29	1226.32
1250.93	1299.14	1318.34	1338.24	1373.63	1389.48
1439.87	1447.84	1491.12	1640.04	1891.40	1913.00
2983.20	3012.59	3057.81	3070.22	3110.71	3549.70
3648.43	3881.97	3903.08			

2.5 Cartesian coordinates of Glu conformers

Each line starting by an asterisk contains the relative energy of the corresponding conformer and the dihedral angle for each of the target torsions (separated by underscores). Cartesian coordinates are given in Å.

```
19
 * E =  +0.000 kcal/mol ; (1) 002_348_146_297_275_177_179
 H   +0.00000   +0.00000   +0.00000
 O   +0.00000   +0.00000   +0.97384
 C   +1.27562   +0.00000   +1.35045
 C   +2.28421   +0.04950   +0.19162
 N   +1.60618   -0.18948   -1.07855
 H   +2.02767   +0.33721   -1.83534
 O   +1.61842   +0.00695   +2.49618
 C   +2.96274   +1.42435   +0.23151
 H   +3.04639   -0.70528   +0.42097
 H   +1.60566   -1.17009   -1.32673
 C   +2.01005   +2.59622   +0.00171
 H   +3.42129   +1.52278   +1.21834
 H   +3.76185   +1.45156   -0.51663
 C   +1.77449   +2.91280   -1.45225
 H   +1.03437   +2.44352   +0.48013
 H   +2.40826   +3.51272   +0.45127
 O   +0.89093   +3.90332   -1.60964
 H   +0.79408   +4.07289   -2.55369
 O   +2.31543   +2.38068   -2.38530
19
 * E =  +0.525 kcal/mol ; (2) 351_027_074_281_051_096_189
 H   +0.00000   +0.00000   +0.00000
```

O +0.00000 +0.00000 +0.97063
C +1.26536 +0.00000 +1.36524
C +2.28250 −0.21072 +0.23015
N +1.70553 +0.26983 −1.01780
H +1.68963 +1.28468 −1.04443
O +1.58828 +0.10208 +2.51421
C +3.66766 +0.31720 +0.62582
H +2.36011 −1.30390 +0.14895
H +2.18633 −0.08720 −1.83142
C +3.82596 +1.84238 +0.50404
H +3.85942 +0.00895 +1.65761
H +4.42559 −0.15262 −0.00877
C +2.69786 +2.54795 +1.20691
H +4.78532 +2.14754 +0.92556
H +3.79274 +2.14348 −0.54689
O +2.99251 +2.91771 +2.44900
H +2.18455 +3.23285 +2.87185
O +1.61495 +2.72226 +0.70731
19
∗ E = +0.592 kcal/mol ; (3) 180_178_308_183_081_202_182
H +0.00000 +0.00000 +0.00000
O +0.00000 +0.00000 +0.96389
C +1.27731 +0.00000 +1.37968
C +1.38291 −0.01089 +2.89873
N +2.74366 −0.06022 +3.37917
H +3.24914 −0.82563 +2.95001
O +2.20577 −0.00262 +0.62402
C +0.64506 +1.19918 +3.48702
H +0.86003 −0.91884 +3.23443
H +3.23291 +0.79548 +3.13516
C +0.79573 +1.24807 +5.00183
H +1.05584 +2.12195 +3.05924
H −0.40868 +1.14521 +3.20534
C +2.11651 +1.84020 +5.42538
H +0.01954 +1.88162 +5.44750
H +0.69700 +0.25660 +5.45376
O +2.46564 +1.46288 +6.66136
H +3.29862 +1.89696 +6.87699
O +2.77294 +2.60705 +4.77538
19

```
 * E =  +0.701  kcal/mol  ;  (4)  000_171_294_140_289_182_180
 H    +0.00000    +0.00000    +0.00000
 O    +0.00000    +0.00000    +0.97059
 C    +1.24745    +0.00000    +1.44813
 C    +2.33698    −0.00973    +0.37291
 N    +3.67505    +0.20518    +0.87758
 H    +3.76391    +1.14124    +1.25521
 O    +1.47899    −0.00243    +2.62085
 C    +2.27862    −1.33596    −0.41653
 H    +2.09617    +0.81561    −0.31106
 H    +3.83929    −0.42269    +1.65895
 C    +2.50358    −1.10935    −1.90201
 H    +3.05305    −1.99722    −0.01855
 H    +1.31666    −1.83888    −0.27216
 C    +1.32164    −0.40946    −2.51765
 H    +3.39557    −0.49457    −2.07596
 H    +2.66171    −2.04151    −2.45429
 O    +1.45136    −0.22135    −3.82883
 H    +0.66318    +0.22997    −4.15374
 O    +0.34140    −0.04518    −1.91380
19
 * E =  +0.842  kcal/mol  ;  (5)  178_170_286_178_289_179_180
 H    +0.00000    +0.00000    +0.00000
 O    +0.00000    +0.00000    +0.96391
 C    +1.27292    +0.00000    +1.38087
 C    +1.38630    +0.03685    +2.89741
 N    +2.74078    −0.18821    +3.35793
 H    +2.99622    −1.16274    +3.26116
 O    +2.20478    −0.00312    +0.62598
 C    +0.86398    +1.39643    +3.39396
 H    +0.72582    −0.74058    +3.29803
 H    +3.38857    +0.33393    +2.77594
 C    +0.89216    +1.49546    +4.91088
 H    +1.49529    +2.18765    +2.97141
 H    −0.15375    +1.54546    +3.02157
 C    −0.14261    +0.62103    +5.56599
 H    +1.87394    +1.20191    +5.29778
 H    +0.71581    +2.52142    +5.25071
 O    −0.09965    +0.70945    +6.90449
 H    −0.78231    +0.12937    +7.25983
```

O −0.93068 −0.08120 +4.99497
19
∗ E = +0.866 kcal/mol ; (6) 358_006_110_169_084_182_182
H +0.00000 +0.00000 +0.00000
O +0.00000 +0.00000 +0.97432
C +1.27205 +0.00000 +1.34609
C +2.27886 −0.04634 +0.18079
N +1.58547 +0.05832 −1.10128
H +1.77171 +0.94589 −1.55464
O +1.62307 +0.01699 +2.49038
C +3.35311 +1.01749 +0.41526
H +2.74923 −1.03586 +0.25884
H +1.83068 −0.68232 −1.74176
C +4.55331 +0.89364 −0.51552
H +2.90362 +2.01184 +0.30229
H +3.67739 +0.92919 +1.45462
C +4.36815 +1.55464 −1.85729
H +5.44255 +1.35652 −0.07259
H +4.82972 −0.15332 −0.69150
O +5.42909 +1.36940 −2.65187
H +5.26806 +1.83330 −3.48153
O +3.41242 +2.19538 −2.20422
19
∗ E = +0.928 kcal/mol ; (7) 354_023_086_076_069_180_180
H +0.00000 +0.00000 +0.00000
O +0.00000 +0.00000 +0.97165
C +1.27124 +0.00000 +1.35873
C +2.28062 −0.14169 +0.21230
N +1.65851 +0.29929 −1.03446
H +1.75905 +1.30122 −1.15731
O +1.59451 +0.07573 +2.50947
C +3.61005 +0.54272 +0.55299
H +2.45756 −1.22024 +0.11098
H +2.09175 −0.14972 −1.83086
C +4.47159 −0.25059 +1.52694
H +4.16289 +0.67734 −0.38275
H +3.40850 +1.53415 +0.97304
C +5.00968 −1.50759 +0.89837
H +5.33047 +0.33574 +1.87056
H +3.90832 −0.51976 +2.42630

O +5.77095 −2.21294 +1.74460
H +6.07915 −3.00066 +1.28249
O +4.80234 −1.86527 −0.23055
19
* E = +1.061 kcal/mol ; (8) 004_344_145_058_059_269_004
H +0.00000 +0.00000 +0.00000
O +0.00000 +0.00000 +0.97474
C +1.26161 +0.00000 +1.35361
C +2.28397 +0.10074 +0.21725
N +1.63309 −0.21029 −1.05183
H +2.00729 +0.34652 −1.80928
O +1.58656 −0.03949 +2.51391
C +2.84760 +1.53076 +0.16731
H +3.09726 −0.59447 +0.46350
H +1.73146 −1.18530 −1.30411
C +3.51304 +2.01507 +1.46057
H +3.58944 +1.55939 −0.63964
H +2.03649 +2.21613 −0.10770
C +4.66771 +1.09913 +1.84098
H +3.93389 +3.00689 +1.28769
H +2.78368 +2.08222 +2.27224
O +4.36400 +0.07600 +2.64075
H +3.40821 +0.03841 +2.81873
O +5.77645 +1.25598 +1.41820
19
* E = +1.100 kcal/mol ; (9) 180_034_291_279_056_096_187
H +0.00000 +0.00000 +0.00000
O +0.00000 +0.00000 +0.96357
C +1.27295 +0.00000 +1.37370
C +1.39001 +0.00169 +2.89945
N +0.34109 −0.72628 +3.59142
H −0.54472 −0.24603 +3.49893
O +2.20031 +0.03805 +0.61202
C +2.78342 −0.46722 +3.32935
H +1.30159 +1.06137 +3.17689
H +0.22460 −1.64062 +3.16385
C +2.94444 −1.99678 +3.32016
H +3.53155 −0.00836 +2.67726
H +2.95889 −0.12204 +4.35122
C +2.60533 −2.56103 +1.96866

H +3.96861 −2.26205 +3.58817
H +2.25592 −2.43610 +4.04652
O +3.67131 −2.76036 +1.19409
H +3.36186 −3.01970 +0.31843
O +1.48034 −2.77758 +1.59357
19
* E = +1.112 kcal/mol ; (10) 180_047_179_288_279_162_178
H +0.00000 +0.00000 +0.00000
O +0.00000 +0.00000 +0.96368
C +1.27597 +0.00000 +1.38019
C +1.36900 +0.01229 +2.89800
N +0.45857 −0.99387 +3.42551
H +0.50646 −1.02377 +4.43713
O +2.20212 +0.02104 +0.62132
C +2.80250 −0.25699 +3.34716
H +1.10009 +1.04364 +3.19122
H −0.49542 −0.81827 +3.13844
C +3.21480 −1.70838 +3.13289
H +3.46864 +0.40574 +2.79041
H +2.89334 −0.01370 +4.41062
C +2.71328 −2.61558 +4.22590
H +2.87447 −2.09447 +2.16789
H +4.30721 −1.80224 +3.13354
O +2.72774 −3.90362 +3.85997
H +2.42409 −4.42559 +4.61097
O +2.37856 −2.26642 +5.32635
19
* E = +1.185 kcal/mol ; (11) 183_197_192_290_280_155_178
H +0.00000 +0.00000 +0.00000
O +0.00000 +0.00000 +0.96386
C +1.27776 +0.00000 +1.37208
C +1.41177 −0.08171 +2.88297
N +2.76012 +0.31801 +3.22182
H +2.98276 +0.11101 +4.18648
O +2.19962 +0.01745 +0.60771
C +0.39185 +0.80223 +3.60895
H +1.17721 −1.13614 +3.12022
H +3.43966 −0.10139 +2.60056
C +0.71723 +2.27991 +3.42857
H −0.61089 +0.57623 +3.24117

```
 H    +0.41762    +0.56494    +4.67742
 C    +1.81325    +2.72817    +4.36308
 H    +1.01427    +2.51546    +2.40116
 H    −0.15762    +2.89952    +3.65762
 O    +2.47826    +3.79034    +3.89295
 H    +3.13954    +4.03640    +4.54921
 O    +2.04554    +2.24632    +5.43849
19
 * E =  +1.206  kcal/mol ; (12) 169_158_275_296_081_195_181
 H    +0.00000    +0.00000    +0.00000
 O    +0.00000    +0.00000    +0.96428
 C    +1.26385    +0.00000    +1.38545
 C    +1.38480    +0.26726    +2.87683
 N    +2.63729    −0.22599    +3.42182
 H    +2.56117    −1.20636    +3.66041
 O    +2.20923    −0.14530    +0.65851
 C    +1.25093    +1.78578    +3.10927
 H    +0.54464    −0.22114    +3.37976
 H    +3.36034    −0.16156    +2.71140
 C    +2.38335    +2.58810    +2.47789
 H    +0.29049    +2.13571    +2.71724
 H    +1.26258    +1.93747    +4.19118
 C    +2.18461    +2.83768    +1.00345
 H    +2.46042    +3.57744    +2.94413
 H    +3.35774    +2.11408    +2.63229
 O    +3.30836    +3.25502    +0.40839
 H    +3.11245    +3.40323    −0.52353
 O    +1.14579    +2.71927    +0.41350
19
 * E =  +1.303  kcal/mol ; (13) 355_021_086_182_307_266_177
 H    +0.00000    +0.00000    +0.00000
 O    +0.00000    +0.00000    +0.97187
 C    +1.27175    +0.00000    +1.35877
 C    +2.27832    −0.10998    +0.20700
 N    +1.65662    +0.29685    −1.04793
 H    +1.71992    +1.29951    −1.18744
 O    +1.60338    +0.05009    +2.50672
 C    +3.57564    +0.61195    +0.57905
 H    +2.48191    −1.18560    +0.11572
 H    +2.10877    −0.16182    −1.83075
```

C +4.61447 +0.59107 −0.55328
H +3.35123 +1.65566 +0.82847
H +3.98165 +0.15894 +1.48744
C +4.85680 −0.81746 −1.02473
H +4.24763 +1.15808 −1.41311
H +5.54992 +1.03646 −0.20998
O +5.89502 −1.40178 −0.41867
H +5.95893 −2.30991 −0.73752
O +4.17127 −1.39243 −1.82918
19
∗ E = +1.602 kcal/mol ; (14) 001_154_289_056_059_106_182
H +0.00000 +0.00000 +0.00000
O +0.00000 +0.00000 +0.97219
C +1.23964 +0.00000 +1.46152
C +2.38264 +0.03207 +0.43494
N +3.59412 +0.61661 +0.97420
H +3.47813 +1.61417 +1.10601
O +1.44981 −0.05255 +2.63897
C +2.70928 −1.40328 −0.01088
H +2.06430 +0.62052 −0.43372
H +3.75827 +0.22736 +1.89841
C +1.53877 −2.21775 −0.58629
H +3.52402 −1.33988 −0.73835
H +3.10148 −1.94105 +0.85979
C +0.95241 −1.54030 −1.79349
H +1.88245 −3.21584 −0.86157
H +0.74106 −2.31786 +0.15708
O +1.26413 −2.12860 −2.94437
H +0.87234 −1.61929 −3.66457
O +0.28156 −0.53480 −1.75392
19
∗ E = +1.655 kcal/mol ; (15) 178_034_308_181_081_200_182
H +0.00000 +0.00000 +0.00000
O +0.00000 +0.00000 +0.96379
C +1.27917 +0.00000 +1.37468
C +1.39766 +0.05259 +2.89487
N +0.34833 −0.63346 +3.62441
H −0.56915 −0.33841 +3.31669
O +2.20044 −0.00661 +0.60902
C +2.77657 −0.44732 +3.32042

H	+1.32418	+1.12293	+3.14597
H	+0.42123	−1.63839	+3.50450
C	+2.93163	−0.42526	+4.83457
H	+2.92126	−1.47085	+2.95598
H	+3.53887	+0.16953	+2.84060
C	+2.29858	−1.62313	+5.49625
H	+3.99085	−0.44578	+5.11756
H	+2.50421	+0.47932	+5.27849
O	+2.05541	−1.41037	+6.79598
H	+1.68274	−2.22044	+7.16149
O	+2.07404	−2.67638	+4.96468

19

∗ E = +1.685 kcal/mol ; (16) 355_022_083_116_062_177_179

H	+0.00000	+0.00000	+0.00000
O	+0.00000	+0.00000	+0.97221
C	+1.27178	+0.00000	+1.35862
C	+2.27645	−0.12874	+0.20554
N	+1.65088	+0.30581	−1.03968
H	+1.69152	+1.31630	−1.12358
O	+1.60164	+0.04596	+2.50777
C	+3.63075	+0.51663	+0.56105
H	+2.41653	−1.21495	+0.10844
H	+2.16862	−0.07751	−1.82240
C	+4.77268	−0.48804	+0.62152
H	+3.86005	+1.27577	−0.19418
H	+3.55178	+1.01303	+1.53143
C	+5.00778	−1.16232	−0.70392
H	+5.71374	−0.02166	+0.93123
H	+4.57130	−1.27033	+1.36385
O	+6.04844	−2.00171	−0.67291
H	+6.13703	−2.40363	−1.54474
O	+4.34970	−0.99076	−1.69631

19

∗ E = +1.735 kcal/mol ; (17) 180_178_311_189_305_079_355

H	+0.00000	+0.00000	+0.00000
O	+0.00000	+0.00000	+0.96421
C	+1.26829	+0.00000	+1.38839
C	+1.36050	−0.01209	+2.90550
N	+2.73627	−0.06326	+3.37508
H	+3.24368	−0.79498	+2.88843

O +2.21160 +0.00665 +0.65019
C +0.62574 +1.20621 +3.48559
H +0.84667 −0.92208 +3.24640
H +3.21340 +0.80306 +3.14368
C +0.85592 +1.40060 +4.99082
H +0.96282 +2.11058 +2.96302
H −0.44193 +1.09909 +3.27752
C +0.51296 +0.16089 +5.80297
H +1.90031 +1.67180 +5.17767
H +0.22157 +2.21152 +5.35098
O +1.44087 −0.80242 +5.78504
H +2.16105 −0.56545 +5.17255
O −0.50876 +0.03460 +6.41078
19
* E = +1.740 kcal/mol ; (18) 178_169_293_173_174_175_179
H +0.00000 +0.00000 +0.00000
O +0.00000 +0.00000 +0.96398
C +1.27386 +0.00000 +1.38325
C +1.37776 +0.04480 +2.90175
N +2.71959 −0.20638 +3.38188
H +3.00163 −1.15901 +3.18628
O +2.20751 −0.01787 +0.63314
C +0.88302 +1.41015 +3.39615
H +0.70615 −0.73080 +3.29331
H +3.37281 +0.39005 +2.88335
C +0.79783 +1.44404 +4.91247
H +1.57484 +2.18830 +3.05099
H −0.09069 +1.63948 +2.95384
C +0.45009 +2.81261 +5.42714
H +0.04990 +0.73745 +5.29119
H +1.75143 +1.13990 +5.35826
O +0.27960 +2.82138 +6.75966
H +0.07086 +3.72460 +7.02292
O +0.33556 +3.80089 +4.75812
19
* E = +1.850 kcal/mol ; (19) 174_171_290_283_061_237_173
H +0.00000 +0.00000 +0.00000
O +0.00000 +0.00000 +0.96393
C +1.26775 +0.00000 +1.38522
C +1.36940 +0.14999 +2.90242

N +2.71413 −0.06197 +3.39574
H +2.96987 −1.03858 +3.31818
O +2.20808 −0.10092 +0.64720
C +0.83203 +1.52549 +3.34402
H +0.70497 −0.61020 +3.32943
H +3.37541 +0.44426 +2.81242
C +1.81863 +2.66651 +3.07625
H −0.11363 +1.72780 +2.83260
H +0.64057 +1.48038 +4.41892
C +2.11021 +2.77451 +1.59960
H +1.37190 +3.61320 +3.39377
H +2.74548 +2.52073 +3.63445
O +3.41492 +2.66806 +1.31841
H +3.51108 +2.63903 +0.35878
O +1.26959 +2.89593 +0.75192
19
* E = +1.869 kcal/mol ; (20) 182_192_207_302_190_186_181
H +0.00000 +0.00000 +0.00000
O +0.00000 +0.00000 +0.96390
C +1.27538 +0.00000 +1.37654
C +1.38974 −0.06049 +2.88777
N +2.76134 +0.23427 +3.25323
H +3.01653 −0.20238 +4.12761
O +2.20280 +0.01821 +0.61819
C +0.41811 +0.91046 +3.56911
H +1.07762 −1.08725 +3.14673
H +3.40081 −0.06661 +2.52763
C +0.65685 +2.34365 +3.12343
H −0.61460 +0.61289 +3.36744
H +0.56196 +0.83943 +4.65190
C −0.12262 +3.32986 +3.94647
H +1.72121 +2.59395 +3.20910
H +0.38907 +2.49533 +2.07076
O −0.01613 +4.58101 +3.46857
H −0.52487 +5.15993 +4.04705
O −0.76845 +3.06899 +4.92256
19
* E = +1.906 kcal/mol ; (21) 356_016_093_166_064_060_177
H +0.00000 +0.00000 +0.00000
O +0.00000 +0.00000 +0.97224

C	+1.27066	+0.00000	+1.35686
C	+2.28360	−0.08833	+0.20185
N	+1.62815	+0.22686	−1.06628
H	+1.71835	+1.21116	−1.29333
O	+1.60525	+0.03337	+2.50448
C	+3.51954	+0.74935	+0.52689
H	+2.57491	−1.14746	+0.16764
H	+2.00187	−0.30863	−1.83855
C	+4.69671	+0.45030	−0.40491
H	+3.25916	+1.81393	+0.47097
H	+3.80930	+0.54737	+1.56077
C	+4.42542	+0.81420	−1.84102
H	+5.56996	+1.02643	−0.08165
H	+4.95667	−0.61069	−0.38082
O	+4.15418	+2.12502	−1.98567
H	+4.03002	+2.29988	−2.92569
O	+4.43401	+0.05915	−2.77275

19

∗ E = +1.925 kcal/mol ; (22) 188_331_300_053_277_171_180

H	+0.00000	+0.00000	+0.00000
O	+0.00000	+0.00000	+0.96395
C	+1.27014	+0.00000	+1.37673
C	+1.40886	−0.20662	+2.87872
N	+0.29929	+0.25866	+3.68561
H	+0.14761	+1.25271	+3.56799
O	+2.20462	+0.11534	+0.63041
C	+1.63527	−1.70119	+3.15707
H	+2.31619	+0.33773	+3.16547
H	−0.55591	−0.20842	+3.40494
C	+2.78730	−2.31609	+2.37203
H	+1.81275	−1.79136	+4.23154
H	+0.71051	−2.24454	+2.92794
C	+2.38890	−2.72944	+0.97704
H	+3.64080	−1.63487	+2.28895
H	+3.15894	−3.21921	+2.86911
O	+3.44704	−3.07637	+0.23648
H	+3.12892	−3.32469	−0.63852
O	+1.26267	−2.77997	+0.56381

19

∗ E = +1.975 kcal/mol ; (23) 177_153_289_048_058_195_181

```
 H    +0.00000    +0.00000    +0.00000
 O    +0.00000    +0.00000    +0.96458
 C    +1.27617    +0.00000    +1.38621
 C    +1.38385    +0.06482    +2.90265
 N    +2.60496    -0.53145    +3.40223
 H    +2.58310    -1.53917    +3.30907
 O    +2.20697    -0.01788    +0.63129
 C    +1.33429    +1.53672    +3.35163
 H    +0.51773    -0.45325    +3.32513
 H    +3.39357    -0.20135    +2.85487
 C    +0.21359    +2.37039    +2.74330
 H    +1.25147    +1.52679    +4.44142
 H    +2.29669    +1.99784    +3.10150
 C    -1.15360    +1.80309    +3.02590
 H    +0.22927    +3.38412    +3.16035
 H    +0.31810    +2.49176    +1.66071
 O    -2.09599    +2.34271    +2.23901
 H    -2.94219    +1.95238    +2.48507
 O    -1.40487    +0.98467    +3.86538
19
 * E =  +1.984  kcal/mol ; (24) 177_160_030_293_272_175_179
 H    +0.00000    +0.00000    +0.00000
 O    +0.00000    +0.00000    +0.96386
 C    +1.27365    +0.00000    +1.37887
 C    +1.38421    +0.06760    +2.88676
 N    +2.69437    -0.40271    +3.27610
 H    +3.37613    -0.20292    +2.55340
 O    +2.20384    +0.00020    +0.62146
 C    +1.04560    +1.51225    +3.32247
 H    +0.60440    -0.58893    +3.29347
 H    +2.99468    +0.01252    +4.14828
 C    +2.07455    +2.53528    +2.85062
 H    +0.05260    +1.76983    +2.94167
 H    +0.99354    +1.53312    +4.41576
 C    +3.22604    +2.70793    +3.80832
 H    +2.49443    +2.28187    +1.86865
 H    +1.62437    +3.52712    +2.73277
 O    +4.15644    +3.53789    +3.31820
 H    +4.85586    +3.62314    +3.97595
 O    +3.32505    +2.20200    +4.89271
```

```
19
 * E =  +1.993  kcal/mol  ;  (25)  169_151_035_300_084_191_180
 H    +0.00000    +0.00000    +0.00000
 O    +0.00000    +0.00000    +0.96416
 C    +1.26241    +0.00000    +1.38307
 C    +1.38223    +0.27208    +2.86688
 N    +2.57574    -0.38946    +3.35153
 H    +3.30271    -0.37513    +2.64541
 O    +2.20876    -0.13755    +0.65557
 C    +1.35810    +1.80685    +3.06896
 H    +0.49493    -0.14311    +3.35768
 H    +2.92393    +0.03004    +4.20312
 C    +2.48991    +2.53181    +2.34934
 H    +0.39433    +2.20287    +2.73162
 H    +1.43154    +1.98880    +4.14627
 C    +2.18136    +2.81708    +0.89996
 H    +2.69896    +3.50076    +2.81662
 H    +3.42873    +1.96860    +2.39150
 O    +3.26516    +3.22935    +0.23240
 H    +3.00689    +3.39376    -0.68141
 O    +1.10022    +2.72439    +0.38755
19
 * E =  +2.003  kcal/mol  ;  (26)  354_024_078_281_060_285_173
 H    +0.00000    +0.00000    +0.00000
 O    +0.00000    +0.00000    +0.97068
 C    +1.27062    +0.00000    +1.36283
 C    +2.28495    -0.15691    +0.21941
 N    +1.68264    +0.30209    -1.02616
 H    +1.67276    +1.31436    -1.08113
 O    +1.59329    +0.07121    +2.51211
 C    +3.65236    +0.42365    +0.60222
 H    +2.40947    -1.24491    +0.12911
 H    +2.15018    -0.07043    -1.84085
 C    +3.78222    +1.95056    +0.49740
 H    +3.87421    +0.10772    +1.62575
 H    +4.41306    -0.01680    -0.05047
 C    +2.80400    +2.69404    +1.37017
 H    +4.78564    +2.25070    +0.80149
 H    +3.63070    +2.27230    -0.53947
 O    +1.55888    +2.65513    +0.84083
```

H	+0.94671	+3.04881	+1.47437
O	+3.05135	+3.25822	+2.39141

19

∗ E = +2.017 kcal/mol ; (27) 001_343_149_049_278_166_182

H	+0.00000	+0.00000	+0.00000
O	+0.00000	+0.00000	+0.97114
C	+1.26418	+0.00000	+1.36225
C	+2.29950	+0.02288	+0.22964
N	+1.67437	−0.37139	−1.02868
H	+2.10984	+0.08067	−1.82209
O	+1.59444	−0.01298	+2.51491
C	+2.90616	+1.42490	+0.07363
H	+3.09443	−0.66240	+0.55325
H	+1.70556	−1.37144	−1.17858
C	+3.37733	+2.05064	+1.37995
H	+3.74701	+1.33977	−0.62313
H	+2.15601	+2.08070	−0.38433
C	+2.23401	+2.69014	+2.13077
H	+3.85891	+1.32430	+2.04078
H	+4.10934	+2.84238	+1.18508
O	+2.55833	+2.98072	+3.39311
H	+1.78135	+3.36028	+3.81867
O	+1.16418	+2.94729	+1.65268

19

∗ E = +2.072 kcal/mol ; (28) 181_060_298_175_289_180_180

H	+0.00000	+0.00000	+0.00000
O	+0.00000	+0.00000	+0.96361
C	+1.27905	+0.00000	+1.37940
C	+1.36850	−0.03127	+2.89845
N	+0.72792	−1.21824	+3.45773
H	−0.26199	−1.23100	+3.24597
O	+2.20346	−0.00459	+0.61746
C	+2.82444	+0.05225	+3.34013
H	+0.82600	+0.84731	+3.26782
H	+1.13647	−2.06208	+3.07040
C	+2.95879	+0.14872	+4.85185
H	+3.35436	−0.83525	+2.97549
H	+3.29268	+0.91570	+2.85955
C	+2.50517	+1.48096	+5.38619
H	+2.36774	−0.62824	+5.34923

H	+3.99504	+0.00490	+5.17501
O	+2.62751	+1.55541	+6.72068
H	+2.32327	+2.42742	+6.99601
O	+2.08291	+2.39295	+4.73005

19

∗ E = +2.075 kcal/mol ; (29) 000_338_161_304_297_305_182

H	+0.00000	+0.00000	+0.00000
O	+0.00000	+0.00000	+0.97070
C	+1.25646	+0.00000	+1.40682
C	+2.32947	+0.00954	+0.31265
N	+1.77634	−0.48660	−0.94092
H	+2.34670	−0.21050	−1.73093
O	+1.53716	+0.04633	+2.56807
C	+2.80822	+1.45762	+0.14210
H	+3.16516	−0.58720	+0.69912
H	+1.69547	−1.49582	−0.94539
C	+1.67761	+2.44890	−0.15989
H	+3.29912	+1.76121	+1.07030
H	+3.55858	+1.49078	−0.65614
C	+0.95222	+2.18369	−1.45442
H	+0.93009	+2.45846	+0.63743
H	+2.10152	+3.45637	−0.23178
O	+1.79616	+2.13376	−2.50555
H	+1.26349	+1.99582	−3.29717
O	−0.23153	+2.05611	−1.57996

19

∗ E = +2.101 kcal/mol ; (30) 179_172_297_179_072_039_177

H	+0.00000	+0.00000	+0.00000
O	+0.00000	+0.00000	+0.96397
C	+1.27559	+0.00000	+1.38212
C	+1.37847	+0.02276	+2.90115
N	+2.72528	−0.15936	+3.38709
H	+3.08918	−1.06839	+3.13037
O	+2.20723	−0.00586	+0.62994
C	+0.79892	+1.34142	+3.43025
H	+0.75205	−0.79731	+3.27763
H	+3.33929	+0.53414	+2.97219
C	+0.84634	+1.39913	+4.95424
H	+1.36481	+2.18100	+3.00890
H	−0.23411	+1.44027	+3.08650

C	+2.21689	+1.59064	+5.55530
H	+0.24398	+2.24548	+5.30608
H	+0.42718	+0.49481	+5.40290
O	+2.97389	+2.45440	+4.84843
H	+3.80663	+2.55542	+5.32246
O	+2.58790	+1.11512	+6.58849

19

* E = +2.108 kcal/mol ; (31) 181_188_221_301_259_055_001

H	+0.00000	+0.00000	+0.00000
O	+0.00000	+0.00000	+0.96429
C	+1.26672	+0.00000	+1.38551
C	+1.36709	−0.01254	+2.90110
N	+2.76792	+0.17202	+3.26835
H	+3.03102	−0.43117	+4.03676
O	+2.20750	+0.00484	+0.64305
C	+0.47399	+1.07846	+3.50869
H	+0.98187	−0.99402	+3.21508
H	+3.36569	−0.03839	+2.47467
C	+0.83160	+2.49709	+3.01929
H	−0.56524	+0.85352	+3.25852
H	+0.55830	+1.01793	+4.59930
C	+1.65313	+3.30841	+4.01200
H	+1.40480	+2.44423	+2.08212
H	−0.06786	+3.07742	+2.81530
O	+2.78342	+2.73642	+4.42276
H	+2.91163	+1.86776	+3.98824
O	+1.31331	+4.38703	+4.40411

19

* E = +2.137 kcal/mol ; (32) 177_161_300_059_275_172_178

H	+0.00000	+0.00000	+0.00000
O	+0.00000	+0.00000	+0.96445
C	+1.28261	+0.00000	+1.37370
C	+1.40003	+0.08212	+2.89142
N	+2.69588	−0.35039	+3.36985
H	+2.87428	−1.31067	+3.10059
O	+2.20501	−0.08164	+0.61761
C	+1.13785	+1.50705	+3.40274
H	+0.62541	−0.57702	+3.30534
H	+3.41912	+0.20432	+2.92258
C	−0.23112	+2.08239	+3.06286

H +1.26684 +1.46281 +4.48689
H +1.91690 +2.16981 +3.00763
C −0.28095 +2.70978 +1.69406
H −1.02557 +1.33061 +3.12070
H −0.50548 +2.87030 +3.77357
O −1.52936 +3.05403 +1.34516
H −1.48768 +3.47846 +0.48096
O +0.66585 +2.91379 +0.98704
19
* E = +2.316 kcal/mol ; (33) 005_339_151_061_175_179_180
H +0.00000 +0.00000 +0.00000
O +0.00000 +0.00000 +0.97200
C +1.27121 +0.00000 +1.36020
C +2.28573 +0.12698 +0.21795
N +1.66380 −0.27435 −1.04022
H +2.07790 +0.20363 −1.83007
O +1.60141 −0.04930 +2.50936
C +2.73438 +1.58820 +0.11152
H +3.14780 −0.49171 +0.50033
H +1.73553 −1.27068 −1.20264
C +3.41628 +2.11340 +1.36491
H +3.42633 +1.67462 −0.73465
H +1.86987 +2.21583 −0.13675
C +3.94664 +3.50446 +1.14822
H +2.74330 +2.12790 +2.22803
H +4.25400 +1.47361 +1.66674
O +4.54118 +3.99233 +2.24641
H +4.85518 +4.88067 +2.04391
O +3.86491 +4.12746 +0.12645
19
* E = +2.325 kcal/mol ; (34) 355_021_089_179_295_180_179
H +0.00000 +0.00000 +0.00000
O +0.00000 +0.00000 +0.97061
C +1.27276 +0.00000 +1.36034
C +2.28598 −0.12561 +0.21404
N +1.66313 +0.28424 −1.04329
H +1.76240 +1.27989 −1.20736
O +1.59746 +0.06551 +2.50874
C +3.57570 +0.60770 +0.58858
H +2.50469 −1.19787 +0.14007

H	+2.04266	−0.20757	−1.84018
C	+4.65632	+0.52333	−0.47871
H	+3.34059	+1.66218	+0.77899
H	+3.93799	+0.19395	+1.53313
C	+5.17200	−0.87591	−0.69589
H	+4.31062	+0.90187	−1.44884
H	+5.51758	+1.14708	−0.21656
O	+6.11806	−0.91627	−1.64678
H	+6.41560	−1.82927	−1.73186
O	+4.80524	−1.85592	−0.11050

19

∗ E = +2.340 kcal/mol ; (35) 178_169_276_172_099_305_000

H	+0.00000	+0.00000	+0.00000
O	+0.00000	+0.00000	+0.96440
C	+1.26728	+0.00000	+1.38934
C	+1.36406	+0.04899	+2.90661
N	+2.72690	−0.20207	+3.35658
H	+2.91325	−1.19508	+3.42463
O	+2.21136	−0.01187	+0.65205
C	+0.85655	+1.41011	+3.40854
H	+0.70016	−0.72667	+3.30657
H	+3.37258	+0.15930	+2.65725
C	+0.74006	+1.48010	+4.94318
H	+1.52867	+2.19457	+3.03717
H	−0.12291	+1.59618	+2.96063
C	+1.90090	+2.17393	+5.64424
H	−0.15782	+2.02426	+5.23644
H	+0.65673	+0.46558	+5.35523
O	+3.11108	+1.68545	+5.38289
H	+3.05799	+0.93034	+4.76127
O	+1.74162	+3.09761	+6.38885

19

∗ E = +2.448 kcal/mol ; (36) 180_049_186_074_071_183_181

H	+0.00000	+0.00000	+0.00000
O	+0.00000	+0.00000	+0.96376
C	+1.27612	+0.00000	+1.37989
C	+1.37101	+0.00398	+2.89460
N	+0.49547	−1.03764	+3.41853
H	+0.47374	−0.98708	+4.42969
O	+2.20057	+0.02256	+0.61787

C +2.80695 −0.24086 +3.34824
H +1.06462 +1.01796 +3.20975
H −0.45167 −0.91822 +3.08085
C +3.71566 +0.96300 +3.14696
H +2.78181 −0.49660 +4.41263
H +3.20341 −1.10455 +2.80786
C +3.39463 +2.08369 +4.09982
H +4.76682 +0.69848 +3.30533
H +3.65979 +1.34653 +2.12269
O +4.15868 +3.16510 +3.88498
H +3.90979 +3.83601 +4.53059
O +2.56493 +2.04736 +4.96716
19
∗ E = +2.555 kcal/mol ; (37) 003_345_151_302_186_185_180
H +0.00000 +0.00000 +0.00000
O +0.00000 +0.00000 +0.97163
C +1.27276 +0.00000 +1.36217
C +2.29470 +0.06910 +0.21970
N +1.64835 −0.23679 −1.05617
H +2.10922 +0.23355 −1.82532
O +1.60108 +0.00203 +2.51107
C +2.88084 +1.48393 +0.20578
H +3.09577 −0.63347 +0.47687
H +1.65888 −1.22932 −1.25496
C +1.81535 +2.55877 +0.02745
H +3.41289 +1.64834 +1.14649
H +3.62516 +1.56044 −0.59473
C +2.42874 +3.92354 −0.13837
H +1.18497 +2.37211 −0.85118
H +1.12956 +2.59493 +0.88262
O +1.49906 +4.88928 −0.17785
H +1.95160 +5.73319 −0.28760
O +3.60171 +4.14868 −0.23599
19
∗ E = +2.642 kcal/mol ; (38) 355_021_087_300_184_182_180
H +0.00000 +0.00000 +0.00000
O +0.00000 +0.00000 +0.97103
C +1.27042 +0.00000 +1.36260
C +2.28993 −0.11233 +0.21806
N +1.66454 +0.29808 −1.03847

H +1.72835 +1.29958 −1.18022
O +1.59526 +0.05375 +2.51232
C +3.59788 +0.58710 +0.58709
H +2.48934 −1.18855 +0.13588
H +2.09587 −0.15165 −1.83446
C +3.41999 +2.07191 +0.86916
H +4.01582 +0.10885 +1.47683
H +4.32301 +0.44485 −0.22074
C +4.73942 +2.74904 +1.13099
H +2.94946 +2.61113 +0.03643
H +2.77776 +2.23316 +1.74256
O +4.58351 +4.04811 +1.42974
H +5.45712 +4.42191 +1.59096
O +5.81343 +2.22097 +1.08027
19
* E = +2.654 kcal/mol ; (39) 182_191_215_185_299_092_182
H +0.00000 +0.00000 +0.00000
O +0.00000 +0.00000 +0.96383
C +1.27409 +0.00000 +1.37653
C +1.38980 −0.04033 +2.88688
N +2.76557 +0.21235 +3.26250
H +3.02245 −0.31881 +4.08358
O +2.20234 +0.00760 +0.61894
C +0.43657 +0.97559 +3.53462
H +1.04971 −1.05441 +3.16532
H +3.38813 −0.02384 +2.49899
C +0.62255 +1.04275 +5.05598
H +0.64757 +1.96707 +3.12044
H −0.59916 +0.71973 +3.29393
C +0.33287 −0.29276 +5.68733
H +1.64757 +1.34527 +5.28416
H −0.07421 +1.76728 +5.48065
O +1.44289 −1.05523 +5.79872
H +1.18042 −1.90038 +6.18211
O −0.74352 −0.68928 +6.02925
19
* E = +2.661 kcal/mol ; (40) 182_197_202_087_068_182_181
H +0.00000 +0.00000 +0.00000
O +0.00000 +0.00000 +0.96395
C +1.28047 +0.00000 +1.37288

C	+1.41039	−0.05565	+2.88094
N	+2.75835	+0.34081	+3.23118
H	+3.00294	−0.01231	+4.14695
O	+2.19879	+0.00794	+0.60465
C	+0.38487	+0.83638	+3.60255
H	+1.18782	−1.10752	+3.13968
H	+3.42300	−0.00572	+2.54986
C	−0.95107	+0.15600	+3.86172
H	+0.83292	+1.11668	+4.56010
H	+0.23736	+1.76051	+3.03610
C	−0.82283	−0.94601	+4.88078
H	−1.69306	+0.86502	+4.24491
H	−1.38470	−0.26830	+2.95035
O	−1.98536	−1.58167	+5.08914
H	−1.83504	−2.26358	+5.75353
O	+0.18505	−1.24384	+5.46134

19

* E = +2.661 kcal/mol ; (41) 355_021_086_180_302_091_182

H	+0.00000	+0.00000	+0.00000
O	+0.00000	+0.00000	+0.97122
C	+1.27137	+0.00000	+1.36189
C	+2.28292	−0.11475	+0.21299
N	+1.66243	+0.29613	−1.04259
H	+1.72516	+1.30002	−1.17454
O	+1.59907	+0.05370	+2.50987
C	+3.57907	+0.60670	+0.58490
H	+2.48441	−1.19177	+0.12761
H	+2.10977	−0.15241	−1.83228
C	+4.64418	+0.53338	−0.51813
H	+3.35708	+1.66038	+0.79208
H	+3.96368	+0.18042	+1.51500
C	+4.97572	−0.89762	−0.85260
H	+4.29627	+1.04013	−1.42315
H	+5.56170	+1.01461	−0.17580
O	+4.19999	−1.37780	−1.85174
H	+4.44573	−2.29924	−1.99754
O	+5.79193	−1.57533	−0.30225

19

* E = +2.748 kcal/mol ; (42) 179_170_293_276_054_054_184

H	+0.00000	+0.00000	+0.00000

O	+0.00000	+0.00000	+0.96356
C	+1.27393	+0.00000	+1.38255
C	+1.36897	+0.02619	+2.90657
N	+2.70855	−0.20491	+3.39982
H	+3.00366	−1.15188	+3.19729
O	+2.20736	−0.02569	+0.63202
C	+0.79290	+1.34577	+3.46040
H	+0.71640	−0.78332	+3.25482
H	+3.37066	+0.40341	+2.92464
C	+1.80274	+2.49797	+3.44356
H	−0.10464	+1.61444	+2.89603
H	+0.49756	+1.17580	+4.49888
C	+2.41146	+2.73433	+2.08522
H	+1.30150	+3.42074	+3.75365
H	+2.62363	+2.30489	+4.13588
O	+1.47708	+2.92009	+1.13582
H	+1.93524	+2.99606	+0.29033
O	+3.58464	+2.74190	+1.84379

19

∗ E = +2.758 kcal/mol ; (43) 178_168_286_175_294_334_182

H	+0.00000	+0.00000	+0.00000
O	+0.00000	+0.00000	+0.96404
C	+1.27361	+0.00000	+1.38147
C	+1.38511	+0.04881	+2.89868
N	+2.72421	−0.22904	+3.37080
H	+2.94400	−1.21320	+3.28514
O	+2.20600	−0.00960	+0.62858
C	+0.92447	+1.43561	+3.37785
H	+0.69130	−0.69966	+3.30170
H	+3.39670	+0.26660	+2.79389
C	+0.90106	+1.52830	+4.89881
H	+1.62480	+2.18419	+2.98741
H	−0.06211	+1.65616	+2.96126
C	−0.12127	+0.64781	+5.57512
H	+1.87373	+1.26864	+5.32161
H	+0.67307	+2.55406	+5.21154
O	−1.17452	+0.36237	+4.78502
H	−1.78917	−0.17014	+5.30245
O	−0.04793	+0.25089	+6.70229

19

```
 * E =  +2.765  kcal/mol  ;  (44)  354_022_087_071_173_180_180
 H    +0.00000    +0.00000    +0.00000
 O    +0.00000    +0.00000    +0.97147
 C    +1.26995    +0.00000    +1.36236
 C    +2.28432    -0.13875    +0.21572
 N    +1.65055    +0.28683    -1.03256
 H    +1.74332    +1.28935    -1.15862
 O    +1.58914    +0.07356    +2.51325
 C    +3.60389    +0.56845    +0.52965
 H    +2.46708    -1.21983    +0.13209
 H    +2.07613    -0.15826    -1.83461
 C    +4.43515    -0.11518    +1.60584
 H    +4.19143    +0.61983    -0.39380
 H    +3.40365    +1.60532    +0.82522
 C    +5.78178    +0.53796    +1.75240
 H    +3.92918    -0.09707    +2.57510
 H    +4.60284    -1.17409    +1.37135
 O    +6.52957    -0.05635    +2.69475
 H    +7.37099    +0.41104    +2.74234
 O    +6.17205    +1.47796    +1.11772
19
 * E =  +2.768  kcal/mol  ;  (45)  184_071_299_062_068_182_181
 H    +0.00000    +0.00000    +0.00000
 O    +0.00000    +0.00000    +0.96389
 C    +1.27650    +0.00000    +1.38183
 C    +1.36827    -0.09592    +2.89893
 N    +0.97997    -1.43282    +3.33935
 H    +0.02555    -1.64307    +3.07461
 O    +2.20265    +0.01536    +0.61850
 C    +2.77711    +0.21451    +3.38517
 H    +0.65916    +0.61767    +3.33228
 H    +1.58323    -2.13617    +2.92712
 C    +3.24961    +1.62599    +3.06528
 H    +2.78735    +0.04955    +4.46610
 H    +3.46974    -0.50037    +2.92685
 C    +2.49277    +2.68518    +3.82196
 H    +4.30828    +1.75025    +3.31806
 H    +3.17869    +1.84654    +1.99462
 O    +2.90027    +3.92188    +3.49430
 H    +2.38675    +4.54887    +4.01584
```

```
 O    +1.62950    +2.48825    +4.63145
19
 * E =  +2.811  kcal/mol ; (46) 179_040_202_186_305_266_177
 H    +0.00000    +0.00000    +0.00000
 O    +0.00000    +0.00000    +0.96370
 C    +1.27807    +0.00000    +1.37586
 C    +1.39707    +0.03286    +2.88710
 N    +0.38713    −0.81075    +3.50980
 H    +0.24721    −0.50684    +4.46766
 O    +2.20013    +0.01311    +0.61227
 C    +2.80808    −0.40154    +3.28607
 H    +1.26417    +1.09925    +3.15088
 H    −0.49889    −0.71951    +3.02880
 C    +2.97117    −0.54962    +4.80576
 H    +3.02063    −1.36924    +2.82206
 H    +3.53312    +0.31393    +2.88736
 C    +2.58524    +0.72139    +5.51028
 H    +2.31029    −1.34241    +5.16465
 H    +4.00428    −0.80708    +5.04567
 O    +3.62759    +1.52566    +5.75524
 H    +3.29284    +2.32998    +6.16870
 O    +1.45758    +1.02872    +5.79573
19
 * E =  +2.827  kcal/mol ; (47) 180_308_292_175_288_178_180
 H    +0.00000    +0.00000    +0.00000
 O    +0.00000    +0.00000    +0.96350
 C    +1.28321    +0.00000    +1.37933
 C    +1.37433    −0.01120    +2.89742
 N    +0.58911    +1.04005    +3.52879
 H    +0.97217    +1.95656    +3.33580
 O    +2.20447    +0.00298    +0.61619
 C    +0.91303    −1.38206    +3.41038
 H    +2.43037    +0.12808    +3.14406
 H    −0.36552    +1.02428    +3.18674
 C    +1.08337    −1.51257    +4.91607
 H    −0.14336    −1.51185    +3.14811
 H    +1.48168    −2.16611    +2.89947
 C    +2.52741    −1.63888    +5.32226
 H    +0.66154    −0.64292    +5.43100
 H    +0.55767    −2.39072    +5.30568
```

O +2.66900 −1.71026 +6.65525
H +3.60976 −1.79405 +6.84715
O +3.46355 −1.68086 +4.57372
19
∗ E = +2.834 kcal/mol ; (48) 183_203_296_282_063_281_177
H +0.00000 +0.00000 +0.00000
O +0.00000 +0.00000 +0.96412
C +1.27539 +0.00000 +1.38264
C +1.40421 −0.08065 +2.90405
N +2.67317 +0.43431 +3.37815
H +3.43137 −0.13653 +3.02458
O +2.20412 +0.01558 +0.62694
C +0.22325 +0.52351 +3.67619
H +1.38255 −1.16071 +3.10940
H +2.82379 +1.36254 +2.99488
C +0.21280 +2.05674 +3.77160
H −0.71764 +0.17228 +3.24475
H +0.28344 +0.14038 +4.69843
C +0.08686 +2.72425 +2.42901
H −0.63567 +2.37611 +4.37808
H +1.13709 +2.39513 +4.25070
O +1.27286 +2.73670 +1.78258
H +1.14501 +3.12851 +0.91092
O −0.90809 +3.18144 +1.94877
19
∗ E = +2.843 kcal/mol ; (49) 179_211_192_282_056_284_001
H +0.00000 +0.00000 +0.00000
O +0.00000 +0.00000 +0.96461
C +1.26125 +0.00000 +1.38953
C +1.38673 +0.03543 +2.90965
N +2.62820 +0.74808 +3.20203
H +2.89209 +0.64403 +4.17386
O +2.21261 −0.06887 +0.66476
C +0.19295 +0.64803 +3.65114
H +1.43556 −1.03014 +3.19254
H +3.37579 +0.38264 +2.62071
C +0.13138 +2.18295 +3.65011
H −0.72807 +0.23762 +3.22938
H +0.23664 +0.31637 +4.69446
C +0.13706 +2.78308 +2.25570

H −0.78782 +2.50562 +4.14043
H +0.98132 +2.58772 +4.21034
O +1.33183 +2.77730 +1.64953
H +2.00431 +2.36184 +2.21895
O −0.83338 +3.22189 +1.71395
19
* E = +2.861 kcal/mol ; (50) 183_198_200_295_287_320_183
H +0.00000 +0.00000 +0.00000
O +0.00000 +0.00000 +0.96396
C +1.27670 +0.00000 +1.37322
C +1.40694 −0.07175 +2.88484
N +2.74752 +0.34856 +3.23117
H +3.01653 +0.03006 +4.15154
O +2.20093 +0.01329 +0.61215
C +0.36890 +0.79732 +3.59999
H +1.19168 −1.12940 +3.12504
H +3.42094 +0.03629 +2.54267
C +0.58065 +2.28299 +3.31861
H −0.63317 +0.49748 +3.28377
H +0.44079 +0.61353 +4.67656
C +1.77493 +2.90420 +3.99961
H +0.68382 +2.48425 +2.24808
H −0.29191 +2.84837 +3.66702
O +1.94036 +2.45647 +5.26195
H +2.68718 +2.93762 +5.63444
O +2.46157 +3.76764 +3.53662
19
* E = +2.864 kcal/mol ; (51) 178_038_189_297_191_186_181
H +0.00000 +0.00000 +0.00000
O +0.00000 +0.00000 +0.96385
C +1.27658 +0.00000 +1.37847
C +1.38866 +0.04749 +2.89229
N +0.34920 −0.77298 +3.49920
H +0.32344 −0.62210 +4.49976
O +2.20148 +0.01281 +0.61814
C +2.77271 −0.40356 +3.34932
H +1.28347 +1.12140 +3.13366
H −0.56383 −0.55306 +3.12215
C +3.06875 −1.84948 +2.97995
H +3.53006 +0.25571 +2.91642

H	+2.83871	−0.28490	+4.43630
C	+4.31351	−2.35427	+3.65250
H	+2.23893	−2.50314	+3.27358
H	+3.18996	−1.97083	+1.89851
O	+4.65336	−3.58268	+3.22721
H	+5.44878	−3.84706	+3.70248
O	+4.95040	−1.76507	+4.48097

19

∗ E = +2.872 kcal/mol ; (52) 178_169_292_173_176_314_181

H	+0.00000	+0.00000	+0.00000
O	+0.00000	+0.00000	+0.96402
C	+1.27376	+0.00000	+1.38367
C	+1.37749	+0.03967	+2.90249
N	+2.71742	−0.21723	+3.38193
H	+2.98641	−1.17794	+3.20975
O	+2.20781	−0.01132	+0.63393
C	+0.88878	+1.40904	+3.39614
H	+0.70092	−0.73080	+3.29459
H	+3.37663	+0.35958	+2.86854
C	+0.81325	+1.43833	+4.92039
H	+1.58518	+2.17956	+3.04465
H	−0.08973	+1.62816	+2.95712
C	+0.43262	+2.78233	+5.47758
H	+0.08863	+0.71308	+5.29792
H	+1.79579	+1.17022	+5.32601
O	+1.12731	+3.78538	+4.90871
H	+0.84929	+4.60707	+5.32846
O	−0.37659	+2.97689	+6.33945

19

∗ E = +2.921 kcal/mol ; (53) 178_169_284_290_285_304_178

H	+0.00000	+0.00000	+0.00000
O	+0.00000	+0.00000	+0.96407
C	+1.27622	+0.00000	+1.38120
C	+1.38374	+0.03885	+2.90107
N	+2.72962	−0.20407	+3.37325
H	+2.96118	−1.18754	+3.31871
O	+2.20590	−0.00079	+0.62625
C	+0.87020	+1.39892	+3.40361
H	+0.71399	−0.73644	+3.28973
H	+3.40287	+0.28114	+2.78569

C +1.81718 +2.54623 +3.03491
H −0.12042 +1.58857 +2.98092
H +0.77039 +1.34605 +4.49091
C +3.06581 +2.59728 +3.88282
H +2.13563 +2.49443 +1.98894
H +1.30052 +3.50086 +3.18136
O +2.78007 +2.66157 +5.19178
H +3.61400 +2.66678 +5.67414
O +4.18910 +2.61926 +3.46476
19
∗ E = +3.030 kcal/mol ; (54) 183_329_295_054_064_187_180
H +0.00000 +0.00000 +0.00000
O +0.00000 +0.00000 +0.96391
C +1.27940 +0.00000 +1.37525
C +1.41620 −0.06949 +2.88805
N +0.35203 +0.56235 +3.64158
H +0.33301 +1.56268 +3.49068
O +2.20077 +0.00210 +0.60671
C +1.50820 −1.54822 +3.30146
H +2.36777 +0.41223 +3.13053
H −0.55137 +0.19505 +3.36537
C +2.59614 −2.34613 +2.59590
H +1.67727 −1.55341 +4.38182
H +0.53510 −2.02099 +3.11950
C +3.98172 −1.84998 +2.92053
H +2.55626 −3.39862 +2.89869
H +2.48815 −2.34164 +1.50644
O +4.91770 −2.46818 +2.18696
H +5.77593 −2.11149 +2.44178
O +4.25038 −1.02193 +3.74537
19
∗ E = +3.058 kcal/mol ; (55) 182_191_209_301_186_048_179
H +0.00000 +0.00000 +0.00000
O +0.00000 +0.00000 +0.96393
C +1.27313 +0.00000 +1.38005
C +1.38008 −0.04040 +2.89309
N +2.75415 +0.23000 +3.26315
H +3.00667 −0.22184 +4.13025
O +2.20482 +0.00590 +0.62708
C +0.42309 +0.96405 +3.54987

```
H    +1.04305    −1.05466    +3.16855
H    +3.38961    −0.06391    +2.53128
C    +0.71084    +2.38853    +3.07918
H    −0.61157    +0.68556    +3.32776
H    +0.55767    +0.90122    +4.63399
C    −0.09498    +3.43156    +3.80154
H    +1.77330    +2.60098    +3.25031
H    +0.50765    +2.50893    +2.01181
O    −0.05640    +3.27027    +5.13816
H    −0.57520    +3.98105    +5.53079
O    −0.70254    +4.32718    +3.28725
19
* E =  +3.105  kcal/mol ; (56) 179_041_185_291_288_324_184
H    +0.00000    +0.00000    +0.00000
O    +0.00000    +0.00000    +0.96381
C    +1.27508    +0.00000    +1.38023
C    +1.37703    +0.02413    +2.89707
N    +0.38054    −0.86847    +3.46610
H    +0.37673    −0.80092    +4.47607
O    +2.20247    +0.02533    +0.62342
C    +2.77910    −0.38002    +3.34332
H    +1.21944    +1.08553    +3.16547
H    −0.54753    −0.66960    +3.11618
C    +3.08271    −1.85207    +3.07061
H    +3.50303    +0.24145    +2.81037
H    +2.88597    −0.16993    +4.41226
C    +2.37729    −2.84265    +3.96093
H    +2.85698    −2.12520    +2.03730
H    +4.15462    −2.03168    +3.21783
O    +2.24677    −2.40285    +5.23315
H    +1.84851    −3.12118    +5.73700
O    +2.03263    −3.94144    +3.63666
19
* E =  +3.111  kcal/mol ; (57) 177_147_048_053_059_196_181
H    +0.00000    +0.00000    +0.00000
O    +0.00000    +0.00000    +0.96450
C    +1.27510    +0.00000    +1.38295
C    +1.38331    +0.07661    +2.88959
N    +2.55932    −0.67094    +3.28890
H    +3.35364    −0.39778    +2.72011
```

O	+2.20758	−0.00184	+0.63086
C	+1.42097	+1.56682	+3.29943
H	+0.48802	−0.38250	+3.31912
H	+2.78268	−0.50272	+4.26138
C	+0.28643	+2.42219	+2.74706
H	+1.40353	+1.59992	+4.39399
H	+2.37899	+1.98305	+2.96785
C	−1.07198	+1.92718	+3.17301
H	+0.37498	+3.45343	+3.10894
H	+0.30465	+2.48598	+1.65491
O	−2.05682	+2.45786	+2.43499
H	−2.89357	+2.11711	+2.77087
O	−1.27917	+1.17088	+4.08031

19

∗ E = +3.117 kcal/mol ; (58) 176_032_292_278_064_284_177

H	+0.00000	+0.00000	+0.00000
O	+0.00000	+0.00000	+0.96372
C	+1.27920	+0.00000	+1.36943
C	+1.40552	+0.09094	+2.88944
N	+0.33052	−0.53604	+3.63722
H	−0.53516	−0.02727	+3.51313
O	+2.19849	−0.02005	+0.60030
C	+2.78315	−0.39304	+3.35199
H	+1.36109	+1.17046	+3.09237
H	+0.16913	−1.47651	+3.29283
C	+2.92032	−1.91678	+3.48634
H	+3.54411	−0.00873	+2.66723
H	+2.96867	+0.03488	+4.34060
C	+2.76431	−2.65297	+2.18309
H	+3.90989	−2.15931	+3.87554
H	+2.16657	−2.28476	+4.19047
O	+1.46468	−2.72590	+1.80601
H	+1.43458	−3.16291	+0.94694
O	+3.63984	−3.13656	+1.53035

19

∗ E = +3.124 kcal/mol ; (59) 189_325_056_056_276_169_180

H	+0.00000	+0.00000	+0.00000
O	+0.00000	+0.00000	+0.96378
C	+1.26715	+0.00000	+1.38053
C	+1.39247	−0.21199	+2.87492

N +0.28770 +0.41421 +3.57636
H −0.59901 +0.06758 +3.22881
O +2.20658 +0.08718 +0.63822
C +1.54305 −1.72624 +3.13977
H +2.32649 +0.28399 +3.16309
H +0.33639 +0.21792 +4.56829
C +2.70491 −2.37188 +2.39215
H +1.68217 −1.84926 +4.21880
H +0.60680 −2.22671 +2.86621
C +2.34104 −2.75933 +0.97972
H +3.57951 −1.71395 +2.34556
H +3.03387 −3.29059 +2.89093
O +3.41655 −3.09892 +0.26159
H +3.12081 −3.32926 −0.62613
O +1.22566 −2.79764 +0.53766
19
∗ E = +3.132 kcal/mol ; (60) 003_338_169_184_312_282_177
H +0.00000 +0.00000 +0.00000
O +0.00000 +0.00000 +0.97094
C +1.27102 +0.00000 +1.37153
C +2.28969 +0.08169 +0.23243
N +1.68226 −0.37549 −1.01252
H +2.27512 −0.15476 −1.80479
O +1.58989 −0.00776 +2.52241
C +2.71036 +1.55485 +0.13588
H +3.15792 −0.51723 +0.54344
H +1.59216 −1.38589 −1.00970
C +3.66893 +1.84253 −1.03020
H +1.81890 +2.18065 +0.01606
H +3.18068 +1.82575 +1.08539
C +4.78116 +0.82895 −1.04955
H +3.15167 +1.78614 −1.99063
H +4.08387 +2.84727 −0.92090
O +5.71696 +1.08185 −0.12732
H +6.36949 +0.37228 −0.15297
O +4.81329 −0.13660 −1.76297
19
∗ E = +3.146 kcal/mol ; (61) 000_151_037_058_057_103_182
H +0.00000 +0.00000 +0.00000
O +0.00000 +0.00000 +0.97190

C	+1.23498	+0.00000	+1.46671
C	+2.37391	−0.00690	+0.44552
N	+3.51300	+0.65406	+1.04731
H	+3.55166	+0.42494	+2.03512
O	+1.44126	−0.04372	+2.64474
C	+2.67073	−1.46874	+0.04317
H	+2.05305	+0.55585	−0.44039
H	+4.38218	+0.37948	+0.60872
C	+1.49556	−2.25831	−0.56102
H	+3.50588	−1.45760	−0.66738
H	+3.01578	−1.99730	+0.93846
C	+0.94893	−1.57085	−1.78177
H	+1.82237	−3.26475	−0.82722
H	+0.68157	−2.33637	+0.16651
O	+1.32328	−2.13632	−2.92649
H	+0.95430	−1.62320	−3.65599
O	+0.25896	−0.57904	−1.75700

19

∗ E = +3.162 kcal/mol ; (62) 177_165_030_303_189_186_181

H	+0.00000	+0.00000	+0.00000
O	+0.00000	+0.00000	+0.96392
C	+1.27257	+0.00000	+1.38069
C	+1.37390	+0.06235	+2.88964
N	+2.71988	−0.30513	+3.27325
H	+3.38565	−0.04461	+2.55545
O	+2.20575	+0.00856	+0.62854
C	+0.92277	+1.46516	+3.34482
H	+0.65563	−0.66374	+3.29107
H	+2.98870	+0.10721	+4.15611
C	+1.73027	+2.57666	+2.69229
H	−0.14198	+1.59865	+3.12904
H	+1.02837	+1.52488	+4.43333
C	+1.42662	+3.92199	+3.29421
H	+2.80866	+2.40274	+2.79923
H	+1.55119	+2.63962	+1.61217
O	+2.04329	+4.91650	+2.63641
H	+1.81816	+5.74487	+3.07463
O	+0.72901	+4.11538	+4.24925

19

∗ E = +3.205 kcal/mol ; (63) 175_156_035_064_274_172_178

H	+0.00000	+0.00000	+0.00000
O	+0.00000	+0.00000	+0.96443
C	+1.28033	+0.00000	+1.37033
C	+1.39885	+0.12354	+2.87664
N	+2.67282	−0.44192	+3.26733
H	+3.36709	−0.25348	+2.55245
O	+2.20452	−0.08071	+0.61633
C	+1.21311	+1.59323	+3.31090
H	+0.57632	−0.46330	+3.30979
H	+2.99512	−0.05936	+4.14612
C	−0.16158	+2.18151	+3.01351
H	+1.38844	+1.61983	+4.39162
H	+1.98926	+2.20322	+2.83532
C	−0.26524	+2.75349	+1.62315
H	−0.96137	+1.44249	+3.13566
H	−0.39686	+3.00039	+3.70279
O	−1.52361	+3.10398	+1.31976
H	−1.51798	+3.49072	+0.43706
O	+0.64984	+2.91218	+0.86503

19

∗ E = +3.216 kcal/mol ; (64) 183_325_295_170_174_174_179

H	+0.00000	+0.00000	+0.00000
O	+0.00000	+0.00000	+0.96386
C	+1.27828	+0.00000	+1.37807
C	+1.39452	−0.08407	+2.89372
N	+0.35422	+0.59994	+3.63889
H	+0.38630	+1.60086	+3.49296
O	+2.20504	+0.02143	+0.62018
C	+1.41466	−1.56989	+3.27370
H	+2.36411	+0.36106	+3.14351
H	−0.56043	+0.27856	+3.34137
C	+1.76237	−1.75731	+4.74067
H	+0.42932	−2.00613	+3.06846
H	+2.13070	−2.10469	+2.64158
C	+1.63926	−3.19252	+5.17001
H	+2.78210	−1.42065	+4.96018
H	+1.10307	−1.15228	+5.37325
O	+2.06193	−3.37848	+6.43137
H	+1.94870	−4.31142	+6.64468
O	+1.21344	−4.08787	+4.49602

19
∗ E = +3.309 kcal/mol ; (65) 178_161_299_058_280_342_182

H	+0.00000	+0.00000	+0.00000
O	+0.00000	+0.00000	+0.96435
C	+1.27276	+0.00000	+1.39522
C	+1.34984	+0.05130	+2.91617
N	+2.63403	−0.38114	+3.42397
H	+2.81666	−1.34380	+3.16689
O	+2.21283	−0.04415	+0.65634
C	+1.06123	+1.46489	+3.44723
H	+0.56778	−0.62002	+3.29400
H	+3.36916	+0.17050	+2.99293
C	−0.28899	+2.06372	+3.05923
H	+1.12370	+1.38757	+4.53588
H	+1.87217	+2.13013	+3.13074
C	−0.39593	+2.64641	+1.67004
H	−1.10342	+1.33985	+3.15668
H	−0.52931	+2.89000	+3.73842
O	+0.80403	+2.90210	+1.11794
H	+0.65356	+3.30060	+0.25334
O	−1.42396	+2.89774	+1.11049

19
∗ E = +3.312 kcal/mol ; (66) 178_168_293_172_175_029_178

H	+0.00000	+0.00000	+0.00000
O	+0.00000	+0.00000	+0.96402
C	+1.27395	+0.00000	+1.38325
C	+1.37892	+0.04356	+2.90200
N	+2.71511	−0.23151	+3.38263
H	+2.97644	−1.19211	+3.19788
O	+2.20754	−0.01340	+0.63296
C	+0.90575	+1.41960	+3.39097
H	+0.69152	−0.71647	+3.29635
H	+3.38117	+0.34583	+2.87886
C	+0.81377	+1.45369	+4.91202
H	+1.61922	+2.17754	+3.04152
H	−0.06119	+1.66143	+2.94210
C	+0.46305	+2.80105	+5.48752
H	+0.03610	+0.75742	+5.25356
H	+1.74950	+1.12843	+5.37029
O	−0.27932	+3.54746	+4.64875

H	−0.47934	+4.37655	+5.09783
O	+0.77481	+3.18797	+6.57712

19

∗ E = +3.338 kcal/mol ; (67) 178_149_297_058_173_180_180

H	+0.00000	+0.00000	+0.00000
O	+0.00000	+0.00000	+0.96464
C	+1.27625	+0.00000	+1.38259
C	+1.39760	+0.04732	+2.90109
N	+2.57764	−0.63724	+3.38603
H	+2.54252	−1.62553	+3.16773
O	+2.20605	−0.01252	+0.62715
C	+1.45014	+1.51092	+3.35548
H	+0.50344	−0.42588	+3.32398
H	+3.39986	−0.26421	+2.92222
C	+0.23920	+2.33254	+2.94161
H	+1.55076	+1.51224	+4.44402
H	+2.36497	+1.96994	+2.96123
C	+0.25991	+3.70370	+3.56328
H	+0.17313	+2.46602	+1.85599
H	−0.69964	+1.84425	+3.23035
O	−0.80932	+4.43195	+3.20101
H	−0.73644	+5.29378	+3.62615
O	+1.11057	+4.12607	+4.29294

19

∗ E = +3.344 kcal/mol ; (68) 357_014_096_168_173_171_179

H	+0.00000	+0.00000	+0.00000
O	+0.00000	+0.00000	+0.97149
C	+1.27098	+0.00000	+1.35872
C	+2.29002	−0.07384	+0.20798
N	+1.63146	+0.19552	−1.07011
H	+1.72815	+1.16822	−1.33874
O	+1.60252	+0.02047	+2.50668
C	+3.48868	+0.82209	+0.50995
H	+2.61911	−1.12214	+0.19933
H	+1.98931	−0.37556	−1.82229
C	+4.64436	+0.55921	−0.44109
H	+3.18380	+1.87403	+0.44145
H	+3.79910	+0.66405	+1.54596
C	+5.77047	+1.54287	−0.25810
H	+5.04848	−0.45221	−0.31798

H +4.32940 +0.63084 −1.49086
O +6.85833 +1.19256 −0.96340
H +7.53499 +1.86327 −0.81697
O +5.72549 +2.53185 +0.41488
19
∗ E = +3.352 kcal/mol ; (69) 178_038_303_175_072_038_176
H +0.00000 +0.00000 +0.00000
O +0.00000 +0.00000 +0.96382
C +1.27863 +0.00000 +1.37660
C +1.38776 +0.04700 +2.89796
N +0.38613 −0.72715 +3.60641
H −0.55208 −0.44359 +3.35551
O +2.20153 −0.01216 +0.61308
C +2.78979 −0.36247 +3.33509
H +1.23272 +1.10366 +3.16575
H +0.48509 −1.71575 +3.40383
C +2.97684 −0.21417 +4.84191
H +2.97832 −1.39823 +3.03013
H +3.51891 +0.25356 +2.80377
C +2.25845 −1.22975 +5.69497
H +4.04029 −0.31709 +5.08955
H +2.65934 +0.77154 +5.19247
O +2.27369 −2.46667 +5.15398
H +1.84845 −3.05562 +5.78706
O +1.79141 −1.01610 +6.77555
19
∗ E = +3.368 kcal/mol ; (70) 178_041_187_116_063_181_180
H +0.00000 +0.00000 +0.00000
O +0.00000 +0.00000 +0.96356
C +1.27686 +0.00000 +1.37660
C +1.38049 +0.04202 +2.89186
N +0.37536 −0.82543 +3.48557
H +0.38765 −0.68482 +4.48973
O +2.19881 +0.02199 +0.61185
C +2.79072 −0.34381 +3.36867
H +1.20719 +1.11047 +3.13216
H −0.54972 −0.60029 +3.14128
C +3.51608 +0.79244 +4.07348
H +2.68690 −1.19344 +4.04945
H +3.38756 −0.67193 +2.51463

```
 C    +2.80527    +1.21703    +5.33087
 H    +4.53800    +0.51180    +4.35077
 H    +3.61572    +1.67438    +3.42943
 O    +3.42307    +2.22991    +5.95242
 H    +2.91840    +2.44584    +6.74486
 O    +1.79579    +0.72277    +5.75693
19
 * E =   +3.390  kcal/mol  ;  (71)  178_043_310_187_306_079_355
 H    +0.00000    +0.00000    +0.00000
 O    +0.00000    +0.00000    +0.96398
 C    +1.27587    +0.00000    +1.38503
 C    +1.36247    +0.04272    +2.90521
 N    +0.41242    -0.84983    +3.57131
 H    -0.52289    -0.71547    +3.20348
 O    +2.20786    -0.01602    +0.63613
 C    +2.79430    -0.23586    +3.35698
 H    +1.08532    +1.06915    +3.18858
 H    +0.66260    -1.82074    +3.41325
 C    +2.94781    -0.37490    +4.87792
 H    +3.14638    -1.15600    +2.87607
 H    +3.43712    +0.56512    +2.98274
 C    +2.38888    +0.81711    +5.63872
 H    +2.44470    -1.28437    +5.22393
 H    +4.00626    -0.45439    +5.12850
 O    +1.05633    +0.82879    +5.77253
 H    +0.66283    +0.08548    +5.28216
 O    +3.06722    +1.69516    +6.08303
19
 * E =   +3.398  kcal/mol  ;  (72)  178_039_293_291_287_297_178
 H    +0.00000    +0.00000    +0.00000
 O    +0.00000    +0.00000    +0.96385
 C    +1.27702    +0.00000    +1.37605
 C    +1.38798    +0.03874    +2.90006
 N    +0.39319    -0.76111    +3.59556
 H    -0.53376    -0.37740    +3.46416
 O    +2.20040    -0.00597    +0.61126
 C    +2.80847    -0.31448    +3.33623
 H    +1.20704    +1.08922    +3.16738
 H    +0.37125    -1.71580    +3.25192
 C    +3.17385    -1.78415    +3.08993
```

```
 H    +3.51023    +0.31464    +2.78354
 H    +2.90718    −0.08702    +4.40132
 C    +2.50298    −2.73240    +4.05053
 H    +2.91943    −2.09111    +2.07277
 H    +4.25400    −1.90628    +3.21918
 O    +2.89050    −2.52263    +5.31596
 H    +2.40013    −3.13015    +5.88077
 O    +1.71763    −3.59061    +3.75143
19
 * E =  +3.414  kcal/mol ; (73) 176_152_290_044_057_022_178
 H    +0.00000    +0.00000    +0.00000
 O    +0.00000    +0.00000    +0.96462
 C    +1.26885    +0.00000    +1.40021
 C    +1.34542    +0.11082    +2.91566
 N    +2.52678    −0.51477    +3.47149
 H    +2.47725    −1.52291    +3.39285
 O    +2.21452    −0.04297    +0.66556
 C    +1.34539    +1.60002    +3.30651
 H    +0.45014    −0.36207    +3.33036
 H    +3.34510    −0.21811    +2.94892
 C    +0.34056    +2.48785    +2.57598
 H    +1.18971    +1.64603    +4.38732
 H    +2.35121    +1.98980    +3.11064
 C    −1.11200    +2.08961    +2.68177
 H    +0.40412    +3.50722    +2.97422
 H    +0.56104    +2.57083    +1.50769
 O    −1.36544    +1.31067    +3.74828
 H    −2.31149    +1.12532    +3.75362
 O    −1.97237    +2.44633    +1.93004
19
 * E =  +3.445  kcal/mol ; (74) 184_068_305_065_177_181_180
 H    +0.00000    +0.00000    +0.00000
 O    +0.00000    +0.00000    +0.96393
 C    +1.27684    +0.00000    +1.38325
 C    +1.36175    −0.08731    +2.90303
 N    +0.90662    −1.39390    +3.36632
 H    −0.02640    −1.59932    +3.03017
 O    +2.20431    +0.01603    +0.62385
 C    +2.77745    +0.17382    +3.38930
 H    +0.67598    +0.66651    +3.31211
```

H	+1.52823	−2.12440	+3.03728
C	+3.24988	+1.58938	+3.09875
H	+2.80873	−0.02145	+4.46469
H	+3.46135	−0.54006	+2.91614
C	+4.62399	+1.84435	+3.65568
H	+3.28698	+1.78729	+2.02169
H	+2.57389	+2.33938	+3.52806
O	+5.03167	+3.10402	+3.42629
H	+5.91495	+3.19927	+3.79961
O	+5.30060	+1.04449	+4.23682

19

∗ E = +3.468 kcal/mol ; (75) 182_322_062_065_071_184_180

H	+0.00000	+0.00000	+0.00000
O	+0.00000	+0.00000	+0.96368
C	+1.27611	+0.00000	+1.37861
C	+1.39138	−0.05577	+2.88635
N	+0.34962	+0.74680	+3.50007
H	−0.57009	+0.39037	+3.26744
O	+2.20212	−0.03187	+0.61744
C	+1.38557	−1.54286	+3.30536
H	+2.36459	+0.37460	+3.13996
H	+0.44940	+0.73909	+4.50727
C	+2.59849	−2.32383	+2.81735
H	+1.35600	−1.56823	+4.39978
H	+0.46305	−2.00870	+2.93884
C	+3.85555	−1.91682	+3.54268
H	+2.47005	−3.40033	+2.97499
H	+2.77415	−2.19219	+1.74367
O	+4.93851	−2.54000	+3.05953
H	+5.70389	−2.23801	+3.56145
O	+3.91048	−1.14112	+4.45645

19

∗ E = +3.546 kcal/mol ; (76) 177_167_284_294_177_306_181

H	+0.00000	+0.00000	+0.00000
O	+0.00000	+0.00000	+0.96417
C	+1.27221	+0.00000	+1.38477
C	+1.37757	+0.06376	+2.90346
N	+2.70936	−0.23606	+3.38458
H	+2.89264	−1.23031	+3.33557
O	+2.20714	−0.00853	+0.63452

C	+0.92466	+1.45910	+3.36319
H	+0.67146	−0.66727	+3.31184
H	+3.39914	+0.20662	+2.78562
C	+1.88203	+2.55244	+2.88278
H	−0.08566	+1.65706	+2.99409
H	+0.89456	+1.45240	+4.45680
C	+1.41868	+3.93315	+3.26548
H	+2.87927	+2.41780	+3.30560
H	+1.96684	+2.52784	+1.78764
O	+0.15886	+4.17287	+2.85648
H	−0.06999	+5.06970	+3.12477
O	+2.06543	+4.75051	+3.85463

19

* E = +3.556 kcal/mol ; (77) 178_020_302_336_310_190_182

H	+0.00000	+0.00000	+0.00000
O	+0.00000	+0.00000	+0.96371
C	+1.27568	+0.00000	+1.36746
C	+1.41791	+0.05131	+2.88459
N	+0.24512	−0.35930	+3.63398
H	−0.55240	+0.21325	+3.38680
O	+2.19302	+0.02243	+0.59061
C	+2.68627	−0.68513	+3.38774
H	+1.56749	+1.12113	+3.08832
H	+0.01633	−1.31614	+3.38556
C	+3.29496	−1.78955	+2.52506
H	+3.47874	+0.04584	+3.56755
H	+2.41133	−1.09216	+4.36508
C	+2.31696	−2.82038	+2.04499
H	+3.77273	−1.38065	+1.62935
H	+4.08072	−2.30952	+3.08245
O	+2.92118	−3.86828	+1.47309
H	+2.23723	−4.47233	+1.16257
O	+1.11842	−2.74418	+2.12772

19

* E = +3.582 kcal/mol ; (78) 166_141_292_047_280_304_177

H	+0.00000	+0.00000	+0.00000
O	+0.00000	+0.00000	+0.96574
C	+1.26370	+0.00000	+1.39552
C	+1.37387	+0.33034	+2.87721
N	+2.38191	−0.44785	+3.56764

H +2.11719 −1.42348 +3.62594
O +2.21193 −0.16661 +0.67887
C +1.73774 +1.81988 +2.99181
H +0.39641 +0.15785 +3.34004
H +3.25326 −0.40398 +3.04817
C +0.88798 +2.75293 +2.11651
H +1.64280 +2.09772 +4.04405
H +2.79399 +1.93280 +2.72162
C +1.33012 +2.79323 +0.67059
H −0.16843 +2.47411 +2.13000
H +0.97780 +3.77625 +2.49575
O +2.62132 +3.13209 +0.55518
H +2.85589 +3.09759 −0.37895
O +0.63550 +2.56958 −0.28205
19
∗ E = +3.583 kcal/mol ; (79) 182_192_209_302_187_335_181
H +0.00000 +0.00000 +0.00000
O +0.00000 +0.00000 +0.96397
C +1.27511 +0.00000 +1.37723
C +1.38759 −0.05782 +2.88907
N +2.76025 +0.22576 +3.25777
H +3.02036 −0.23575 +4.11763
O +2.20330 +0.01631 +0.62026
C +0.41679 +0.92100 +3.56304
H +1.06704 −1.08165 +3.14925
H +3.39637 −0.05454 +2.52101
C +0.66657 +2.35390 +3.10597
H −0.61495 +0.62510 +3.35743
H +0.56334 +0.84453 +4.64607
C −0.12503 +3.39897 +3.84675
H +1.72428 +2.61273 +3.19461
H +0.39942 +2.46481 +2.04583
O −1.26723 +2.91455 +4.36971
H −1.71942 +3.64712 +4.80314
O +0.18705 +4.54982 +3.95941
19
∗ E = +3.600 kcal/mol ; (80) 000_167_025_138_291_180_180
H +0.00000 +0.00000 +0.00000
O +0.00000 +0.00000 +0.97068
C +1.24179 +0.00000 +1.45252

```
C    +2.33726   −0.00612   +0.38918
N    +3.60401   +0.30944   +1.01101
H    +3.57060   +0.05632   +1.99329
O    +1.46730   −0.02193   +2.62643
C    +2.28766   −1.35652   −0.37801
H    +2.07375   +0.79413   −0.32018
H    +4.38012   −0.16308   +0.56874
C    +2.46497   −1.17468   −1.87874
H    +3.07276   −2.00564   +0.01963
H    +1.33620   −1.87086   −0.20245
C    +1.26684   −0.49668   −2.48915
H    +3.34766   −0.56971   −2.12093
H    +2.60096   −2.12824   −2.40032
O    +1.38220   −0.32914   −3.80420
H    +0.58660   +0.10858   −4.12990
O    +0.28926   −0.13613   −1.88086
19
 * E =  +3.648  kcal/mol ; (81) 003_341_151_053_279_346_177
H    +0.00000   +0.00000   +0.00000
O    +0.00000   +0.00000   +0.97127
C    +1.26864   +0.00000   +1.36096
C    +2.29499   +0.07387   +0.22542
N    +1.67550   −0.32971   −1.03310
H    +2.11100   +0.12253   −1.82665
O    +1.60042   −0.03372   +2.51074
C    +2.83380   +1.50422   +0.08292
H    +3.12323   −0.57748   +0.53512
H    +1.71965   −1.33015   −1.17915
C    +3.38835   +2.11098   +1.36908
H    +3.63458   +1.46756   −0.66492
H    +2.04069   +2.14104   −0.32455
C    +2.37264   +2.67540   +2.33781
H    +3.98218   +1.38791   +1.93560
H    +4.05840   +2.94287   +1.12621
O    +1.15523   +2.83031   +1.78827
H    +0.55908   +3.14388   +2.47778
O    +2.62268   +2.99978   +3.46033
19
 * E =  +3.677  kcal/mol ; (82) 178_044_298_290_178_180_180
H    +0.00000   +0.00000   +0.00000
```

O	+0.00000	+0.00000	+0.96379
C	+1.27859	+0.00000	+1.37792
C	+1.38059	+0.03904	+2.90156
N	+0.46020	−0.86884	+3.57233
H	−0.50029	−0.62100	+3.37016
O	+2.20070	−0.01507	+0.61246
C	+2.81991	−0.18180	+3.35239
H	+1.08694	+1.05960	+3.18190
H	+0.59112	−1.82227	+3.25492
C	+3.30386	−1.60367	+3.10933
H	+3.47785	+0.51485	+2.82630
H	+2.88615	+0.04807	+4.41902
C	+4.74079	−1.77900	+3.52101
H	+2.71800	−2.33763	+3.67564
H	+3.22719	−1.88044	+2.05101
O	+5.17215	−3.03415	+3.30932
H	+6.09337	−3.07881	+3.58852
O	+5.44273	−0.92700	+3.98590

19

∗ E = +3.706 kcal/mol ; (83) 177_192_211_303_087_064_181

H	+0.00000	+0.00000	+0.00000
O	+0.00000	+0.00000	+0.96452
C	+1.27628	+0.00000	+1.37259
C	+1.38955	+0.08126	+2.88438
N	+2.76997	+0.36164	+3.22397
H	+3.03261	−0.05156	+4.10718
O	+2.20468	−0.08361	+0.62248
C	+0.45362	+1.14615	+3.47700
H	+1.04339	−0.90366	+3.24238
H	+3.39201	+0.02633	+2.49799
C	+0.70212	+2.54117	+2.88358
H	−0.59174	+0.85360	+3.34140
H	+0.64768	+1.17573	+4.55323
C	−0.08445	+2.78747	+1.62188
H	+0.39645	+3.30603	+3.60393
H	+1.76457	+2.67264	+2.66581
O	−1.40965	+2.77526	+1.85358
H	−1.85584	+2.92894	+1.01388
O	+0.37048	+2.98032	+0.52955

19

∗ E = +3.725 kcal/mol ; (84) 183_324_302_062_181_184_181
H +0.00000 +0.00000 +0.00000
O +0.00000 +0.00000 +0.96389
C +1.27735 +0.00000 +1.37845
C +1.39617 −0.07586 +2.89497
N +0.37360 +0.63870 +3.63230
H +0.35817 +1.62199 +3.39213
O +2.20371 +0.01229 +0.61687
C +1.37228 −1.55394 +3.30295
H +2.37751 +0.34709 +3.13794
H −0.54356 +0.26102 +3.42262
C +2.53414 −2.35534 +2.73719
H +1.38388 −1.59123 +4.39603
H +0.42134 −2.00208 +2.98920
C +2.45696 −3.80287 +3.14028
H +2.57422 −2.31574 +1.64207
H +3.49892 −1.95883 +3.07401
O +3.52799 −4.49729 +2.72346
H +3.41321 −5.41230 +3.00327
O +1.55947 −4.30828 +3.75310
19
∗ E = +3.738 kcal/mol ; (85) 182_077_301_078_303_268_172
H +0.00000 +0.00000 +0.00000
O +0.00000 +0.00000 +0.96376
C +1.27034 +0.00000 +1.38243
C +1.36019 −0.05222 +2.90329
N +1.09471 −1.41560 +3.36111
H +0.17926 −1.72695 +3.05818
O +2.20628 −0.04145 +0.63137
C +2.73245 +0.39755 +3.39058
H +0.57807 +0.60157 +3.30680
H +1.78159 −2.05641 +2.97769
C +2.91868 +1.92260 +3.38492
H +2.85330 +0.03360 +4.41422
H +3.50307 −0.07351 +2.76951
C +2.68382 +2.48583 +2.00901
H +2.18953 +2.38556 +4.05509
H +3.92698 +2.17402 +3.72008
O +3.80301 +2.57018 +1.28547
H +3.55947 +2.82053 +0.38690

O +1.60271 +2.78821 +1.57905
19
∗ E = +3.744 kcal/mol ; (86) 354_025_084_076_067_013_178
H +0.00000 +0.00000 +0.00000
O +0.00000 +0.00000 +0.97094
C +1.27038 +0.00000 +1.36330
C +2.28366 −0.15458 +0.22040
N +1.67634 +0.31737 −1.02254
H +1.77176 +1.32338 −1.11340
O +1.59023 +0.08680 +2.51293
C +3.62739 +0.49131 +0.57825
H +2.42617 −1.23806 +0.10773
H +2.12321 −0.10316 −1.82676
C +4.46915 −0.33328 +1.54719
H +4.18487 +0.64701 −0.35085
H +3.44119 +1.47517 +1.02314
C +4.99281 −1.63907 +1.00023
H +5.35333 +0.23284 +1.86114
H +3.91205 −0.55993 +2.45960
O +4.86157 −1.75240 −0.34003
H +5.24811 −2.59896 −0.59241
O +5.49878 −2.50143 +1.65690
19
∗ E = +3.757 kcal/mol ; (87) 353_028_083_299_134_301_358
H +0.00000 +0.00000 +0.00000
O +0.00000 +0.00000 +0.97217
C +1.26015 +0.00000 +1.36382
C +2.28390 −0.17881 +0.23508
N +1.71175 +0.35011 −0.99943
H +1.83380 +1.35585 −1.06064
O +1.57425 +0.11328 +2.51956
C +3.63423 +0.38576 +0.66580
H +2.37424 −1.26614 +0.10799
H +2.14114 −0.06182 −1.81704
C +3.57638 +1.89414 +0.98724
H +3.96860 −0.17519 +1.54413
H +4.36775 +0.19592 −0.12261
C +4.29823 +2.23470 +2.28293
H +4.02374 +2.50038 +0.19915
H +2.53357 +2.22139 +1.10849

O +3.82816 +1.61857 +3.36937
H +3.06025 +1.05899 +3.16786
O +5.22171 +2.99111 +2.33996
19
* E = +3.785 kcal/mol ; (88) 183_076_298_051_047_123_179
H +0.00000 +0.00000 +0.00000
O +0.00000 +0.00000 +0.96367
C +1.27227 +0.00000 +1.38030
C +1.36554 −0.06175 +2.89692
N +1.09256 −1.41544 +3.37240
H +0.15414 −1.70548 +3.12567
O +2.20383 −0.03149 +0.62249
C +2.74424 +0.36211 +3.39288
H +0.58944 +0.59599 +3.29714
H +1.74337 −2.07761 +2.96420
C +3.27058 +1.70424 +2.86450
H +2.70622 +0.36472 +4.48633
H +3.46507 −0.41055 +3.10129
C +2.25353 +2.81018 +2.94157
H +4.17219 +1.99539 +3.40536
H +3.52157 +1.59456 +1.80485
O +2.66687 +3.86704 +3.65505
H +1.96553 +4.52869 +3.63321
O +1.17425 +2.77905 +2.41304
19
* E = +3.802 kcal/mol ; (89) 356_020_088_299_186_313_181
H +0.00000 +0.00000 +0.00000
O +0.00000 +0.00000 +0.97124
C +1.26979 +0.00000 +1.36226
C +2.28971 −0.10869 +0.21698
N +1.66097 +0.28001 −1.04365
H +1.72800 +1.27806 −1.20676
O +1.59697 +0.05091 +2.51142
C +3.58576 +0.61699 +0.58255
H +2.50720 −1.18226 +0.14753
H +2.08293 −0.18845 −1.83366
C +3.36905 +2.10915 +0.84271
H +4.00594 +0.15885 +1.48113
H +4.30636 +0.47272 −0.23077
C +4.65913 +2.85805 +1.04985

H	+2.85235	+2.61361	+0.02181
H	+2.76556	+2.22829	+1.75101
O	+5.47470	+2.22901	+1.91108
H	+6.26784	+2.76688	+2.01376
O	+4.95088	+3.89609	+0.52757

19

* E = +3.815 kcal/mol ; (90) 185_319_289_293_084_191_183

H	+0.00000	+0.00000	+0.00000
O	+0.00000	+0.00000	+0.96407
C	+1.28660	+0.00000	+1.37117
C	+1.40041	−0.12182	+2.88498
N	+0.41885	+0.66020	+3.62266
H	+0.62209	+1.64951	+3.55564
O	+2.20471	+0.08623	+0.61147
C	+1.34386	−1.59727	+3.31333
H	+2.39823	+0.25153	+3.13085
H	−0.51038	+0.52614	+3.24134
C	−0.00040	−2.27685	+3.07536
H	+2.12745	−2.15614	+2.79187
H	+1.56501	−1.61047	+4.38363
C	−0.16520	−2.79132	+1.66863
H	−0.10872	−3.14864	+3.73133
H	−0.85054	−1.62979	+3.31421
O	−1.42719	−3.17312	+1.41959
H	−1.45579	−3.52921	+0.52454
O	+0.70995	−2.88993	+0.85527

19

* E = +3.816 kcal/mol ; (91) 177_162_036_295_287_317_184

H	+0.00000	+0.00000	+0.00000
O	+0.00000	+0.00000	+0.96390
C	+1.27526	+0.00000	+1.37818
C	+1.38032	+0.06360	+2.88738
N	+2.70997	−0.35620	+3.26830
H	+3.40090	−0.04714	+2.59344
O	+2.20549	+0.00251	+0.62406
C	+0.98101	+1.48605	+3.33853
H	+0.63422	−0.63401	+3.28893
H	+2.95892	−0.03214	+4.19266
C	+1.95705	+2.55831	+2.85110
H	−0.02445	+1.70692	+2.96655

H	+0.93190	+1.50159	+4.43181
C	+3.28800	+2.57735	+3.56072
H	+2.17006	+2.46828	+1.78179
H	+1.51652	+3.54895	+3.01404
O	+3.16357	+2.41965	+4.89582
H	+4.04372	+2.50765	+5.27911
O	+4.35122	+2.75733	+3.04159

19

* E = +3.849 kcal/mol ; (92) 181_030_183_278_056_286_001

H	+0.00000	+0.00000	+0.00000
O	+0.00000	+0.00000	+0.96420
C	+1.27333	+0.00000	+1.38128
C	+1.37814	−0.02341	+2.90283
N	+0.22850	−0.71826	+3.48321
H	+0.27034	−0.68952	+4.49512
O	+2.20973	+0.07734	+0.64426
C	+2.69920	−0.65808	+3.34753
H	+1.40741	+1.04304	+3.18227
H	−0.64189	−0.28938	+3.19117
C	+2.70923	−2.19464	+3.37127
H	+3.48455	−0.29025	+2.68164
H	+2.93619	−0.30264	+4.35595
C	+2.34621	−2.84239	+2.04394
H	+3.71052	−2.53977	+3.63163
H	+2.01434	−2.56121	+4.13563
O	+1.04467	−2.78168	+1.72457
H	+0.54486	−2.30962	+2.41554
O	+3.13826	−3.37656	+1.32816

19

* E = +3.862 kcal/mol ; (93) 003_346_147_300_185_308_181

H	+0.00000	+0.00000	+0.00000
O	+0.00000	+0.00000	+0.97205
C	+1.27202	+0.00000	+1.36093
C	+2.29315	+0.06879	+0.21636
N	+1.64407	−0.21911	−1.06144
H	+2.06983	+0.29497	−1.82263
O	+1.60453	+0.00017	+2.50871
C	+2.89682	+1.47663	+0.22265
H	+3.08617	−0.64557	+0.46625
H	+1.68350	−1.20325	−1.29324

C +1.83703 +2.56441 +0.01645
H +3.40104 +1.63291 +1.17938
H +3.65900 +1.53463 −0.56453
C +2.44869 +3.93527 −0.10487
H +1.24022 +2.39539 −0.88249
H +1.15534 +2.57764 +0.87694
O +3.28076 +4.20413 +0.91442
H +3.63005 +5.09324 +0.78642
O +2.24163 +4.71393 −0.99104
19
* E = +3.866 kcal/mol ; (94) 182_337_299_056_060_272_002
H +0.00000 +0.00000 +0.00000
O +0.00000 +0.00000 +0.96497
C +1.26309 +0.00000 +1.38476
C +1.41033 −0.05891 +2.89911
N +0.27049 +0.39850 +3.65993
H +0.05480 +1.36926 +3.47325
O +2.19255 +0.00799 +0.61338
C +1.73659 −1.50811 +3.30654
H +2.27402 +0.57360 +3.13903
H −0.55534 −0.14736 +3.44395
C +2.97861 −2.12213 +2.64870
H +1.86116 −1.49907 +4.39319
H +0.86317 −2.13529 +3.08763
C +4.23008 −1.32643 +2.98867
H +3.12462 −3.12803 +3.04565
H +2.85471 −2.19618 +1.56364
O +4.52038 −0.31101 +2.16773
H +3.85250 −0.21439 +1.47085
O +4.90491 −1.56079 +3.94729
19
* E = +3.892 kcal/mol ; (95) 179_042_199_183_301_093_182
H +0.00000 +0.00000 +0.00000
O +0.00000 +0.00000 +0.96373
C +1.27848 +0.00000 +1.37706
C +1.38833 +0.02347 +2.89050
N +0.41335 −0.88321 +3.48104
H +0.27319 −0.64982 +4.45695
O +2.20168 +0.01588 +0.61659
C +2.81241 −0.34744 +3.29735

H	+1.20123	+1.08006	+3.16535
H	−0.48179	−0.79767	+3.01594
C	+2.99537	−0.42063	+4.81914
H	+3.05366	−1.32414	+2.86769
H	+3.50901	+0.37995	+2.87121
C	+2.65768	+0.89900	+5.46027
H	+2.36200	−1.20768	+5.23680
H	+4.03811	−0.64392	+5.05075
O	+1.37085	+0.94961	+5.87025
H	+1.21337	+1.82699	+6.23839
O	+3.39636	+1.83317	+5.57584

19

* E = +3.896 kcal/mol ; (96) 000_343_155_280_057_051_186

H	+0.00000	+0.00000	+0.00000
O	+0.00000	+0.00000	+0.97157
C	+1.26400	+0.00000	+1.38934
C	+2.32455	−0.00480	+0.27670
N	+1.72470	−0.41138	−0.98871
H	+2.23963	−0.05996	−1.78553
O	+1.56332	+0.01643	+2.54567
C	+2.97395	+1.38496	+0.16530
H	+3.09822	−0.69541	+0.63549
H	+1.67265	−1.41861	−1.07480
C	+2.15324	+2.42570	−0.60819
H	+3.17375	+1.74142	+1.17995
H	+3.94077	+1.27351	−0.33720
C	+0.77410	+2.65426	−0.04000
H	+2.68785	+3.38125	−0.57578
H	+2.02139	+2.15761	−1.65814
O	+0.81983	+2.90058	+1.27465
H	−0.08253	+2.94276	+1.61273
O	−0.24658	+2.60956	−0.66604

19

* E = +3.905 kcal/mol ; (97) 185_087_074_064_069_185_181

H	+0.00000	+0.00000	+0.00000
O	+0.00000	+0.00000	+0.96411
C	+1.26903	+0.00000	+1.37838
C	+1.39633	−0.11071	+2.89144
N	+1.38123	−1.53958	+3.18506
H	+2.26463	−1.96381	+2.92470

O +2.20195 +0.05368 +0.62442
C +2.65029 +0.62127 +3.36940
H +0.50229 +0.33256 +3.34120
H +1.23559 −1.69916 +4.17339
C +2.61027 +2.12039 +3.10685
H +2.75995 +0.43847 +4.44326
H +3.51625 +0.18862 +2.85715
C +1.59502 +2.82716 +3.96692
H +3.58046 +2.58731 +3.30988
H +2.39562 +2.34449 +2.05528
O +1.48634 +4.12677 +3.65394
H +0.83453 +4.52050 +4.24481
O +0.95223 +2.32090 +4.84452
19
* E = +3.936 kcal/mol ; (98) 181_336_045_288_275_170_178
H +0.00000 +0.00000 +0.00000
O +0.00000 +0.00000 +0.96413
C +1.27907 +0.00000 +1.37447
C +1.42965 −0.02866 +2.88393
N +0.25532 +0.50625 +3.54593
H −0.59946 +0.14673 +3.13899
O +2.19919 −0.03528 +0.60869
C +1.83425 −1.46416 +3.28894
H +2.28739 +0.62101 +3.09141
H +0.26284 +0.26757 +4.53011
C +0.69637 −2.46948 +3.14932
H +2.68667 −1.76740 +2.67368
H +2.16661 −1.44636 +4.33183
C −0.20975 −2.50949 +4.35446
H +0.07540 −2.28289 +2.26459
H +1.08317 −3.48790 +3.02904
O −1.31444 −3.23261 +4.12083
H −1.83527 −3.24965 +4.93179
O +0.00692 −1.99535 +5.41693
19
* E = +3.941 kcal/mol ; (99) 178_148_042_062_169_175_180
H +0.00000 +0.00000 +0.00000
O +0.00000 +0.00000 +0.96455
C +1.27464 +0.00000 +1.37906
C +1.39786 +0.05731 +2.88829

N	+2.59106	−0.67847	+3.25728
H	+3.34196	−0.45704	+2.61204
O	+2.20505	−0.00057	+0.62465
C	+1.40993	+1.53203	+3.33248
H	+0.51163	−0.43154	+3.31288
H	+2.89077	−0.43761	+4.19330
C	+0.13886	+2.29456	+2.98877
H	+1.55722	+1.55445	+4.41773
H	+2.28312	+2.02713	+2.89186
C	+0.08524	+3.63219	+3.67848
H	+0.03116	+2.46678	+1.91278
H	−0.75686	+1.73223	+3.28233
O	−0.98623	+4.34701	+3.29970
H	−0.96793	+5.18328	+3.77851
O	+0.88216	+4.03767	+4.47608

19

∗ E = +3.950 kcal/mol ; (100) 176_156_055_187_309_287_177

H	+0.00000	+0.00000	+0.00000
O	+0.00000	+0.00000	+0.96402
C	+1.27605	+0.00000	+1.37755
C	+1.38685	+0.09254	+2.88434
N	+2.65478	−0.46238	+3.28870
H	+3.42850	−0.02014	+2.80832
O	+2.20691	−0.00365	+0.62546
C	+1.13176	+1.57527	+3.25148
H	+0.58279	−0.51537	+3.31958
H	+2.78255	−0.42721	+4.29166
C	+1.37709	+1.85151	+4.74027
H	+1.80108	+2.21297	+2.66226
H	+0.10145	+1.83596	+2.98673
C	+0.62945	+0.85339	+5.58197
H	+2.43698	+1.75732	+4.98338
H	+1.04558	+2.86282	+4.98933
O	−0.69224	+1.08119	+5.58528
H	−1.11528	+0.38459	+6.10051
O	+1.12567	−0.08082	+6.14855

19

∗ E = +3.952 kcal/mol ; (101) 183_328_309_328_294_165_178

H	+0.00000	+0.00000	+0.00000
O	+0.00000	+0.00000	+0.96436

C	+1.28065	+0.00000	+1.37402
C	+1.41521	−0.07047	+2.89009
N	+0.36635	+0.56647	+3.65627
H	+0.19596	+1.51420	+3.34642
O	+2.20081	−0.00260	+0.60552
C	+1.56299	−1.56137	+3.28739
H	+2.36348	+0.43048	+3.11052
H	−0.49954	+0.04295	+3.59999
C	+0.83569	−2.56761	+2.39740
H	+2.62374	−1.82283	+3.27518
H	+1.20947	−1.64755	+4.31924
C	−0.66615	−2.46238	+2.43556
H	+1.16179	−2.50629	+1.35303
H	+1.07847	−3.58818	+2.71761
O	−1.24019	−3.12003	+1.41949
H	−2.19570	−3.04186	+1.52034
O	−1.30874	−1.89768	+3.27778

19

* E = +3.964 kcal/mol ; (102) 181_054_180_069_173_177_180

H	+0.00000	+0.00000	+0.00000
O	+0.00000	+0.00000	+0.96381
C	+1.27608	+0.00000	+1.38166
C	+1.35763	−0.02625	+2.89892
N	+0.56702	−1.16045	+3.36133
H	+0.58704	−1.21665	+4.37160
O	+2.20285	+0.03005	+0.62473
C	+2.79695	−0.16038	+3.37453
H	+0.95464	+0.94843	+3.23543
H	−0.39763	−1.08136	+3.06470
C	+3.63070	+1.08395	+3.11099
H	+2.79039	−0.36750	+4.45058
H	+3.25400	−1.03063	+2.89421
C	+4.99114	+0.98810	+3.74547
H	+3.76445	+1.25817	+2.03892
H	+3.14880	+1.98572	+3.51086
O	+5.75419	+2.05634	+3.45960
H	+6.60516	+1.93375	+3.89479
O	+5.37925	+0.08660	+4.43330

19

* E = +3.985 kcal/mol ; (103) 355_295_282_226_070_182_180

H	+0.00000	+0.00000	+0.00000
O	+0.00000	+0.00000	+0.97141
C	+1.24112	+0.00000	+1.47213
C	+2.36007	−0.11093	+0.43386
N	+2.33153	−1.40298	−0.25610
H	+2.72525	−2.12051	+0.33862
O	+1.43903	+0.05338	+2.65013
C	+2.32474	+1.10436	−0.53923
H	+3.29112	−0.07647	+1.00261
H	+1.38637	−1.68708	−0.48714
C	+2.45217	+0.73478	−2.00808
H	+1.39639	+1.67039	−0.40357
H	+3.13506	+1.78665	−0.27263
C	+1.22005	+0.04145	−2.51180
H	+2.59743	+1.62761	−2.62658
H	+3.30192	+0.07320	−2.19838
O	+1.31108	−0.32807	−3.78519
H	+0.48595	−0.75747	−4.04007
O	+0.22049	−0.16046	−1.86086

19

∗ E = +3.988 kcal/mol ; (104) 180_027_055_281_055_096_187

H	+0.00000	+0.00000	+0.00000
O	+0.00000	+0.00000	+0.96384
C	+1.26863	+0.00000	+1.36225
C	+1.43336	−0.00228	+2.87839
N	+0.29262	−0.62136	+3.52183
H	+0.13872	−1.55727	+3.16192
O	+2.19456	+0.03889	+0.59589
C	+2.82139	−0.55100	+3.25557
H	+1.42892	+1.06641	+3.14244
H	+0.41548	−0.66543	+4.52478
C	+2.92177	−2.08435	+3.20260
H	+3.56969	−0.10457	+2.59434
H	+3.05209	−0.23584	+4.27840
C	+2.51538	−2.60050	+1.84948
H	+3.94299	−2.39595	+3.42956
H	+2.24274	−2.52790	+3.93575
O	+3.54740	−2.80361	+1.03123
H	+3.20085	−3.02512	+0.15907
O	+1.37210	−2.77907	+1.51500

19
∗ E = +3.994 kcal/mol ; (105) 005_339_152_063_177_045_178

H	+0.00000	+0.00000	+0.00000
O	+0.00000	+0.00000	+0.97171
C	+1.27065	+0.00000	+1.36239
C	+2.28702	+0.12846	+0.22183
N	+1.66872	−0.28098	−1.03550
H	+2.09282	+0.18141	−1.82925
O	+1.59850	−0.04923	+2.51190
C	+2.72161	+1.59552	+0.11469
H	+3.15420	−0.48134	+0.50697
H	+1.73270	−1.28013	−1.18442
C	+3.44844	+2.10069	+1.35962
H	+3.38511	+1.68350	−0.75559
H	+1.84363	+2.21820	−0.09153
C	+3.93414	+3.51817	+1.21367
H	+2.78072	+2.06511	+2.22791
H	+4.31466	+1.48024	+1.59881
O	+2.99916	+4.32264	+0.67479
H	+3.36515	+5.21350	+0.63834
O	+5.01297	+3.92174	+1.54024

19
∗ E = +4.006 kcal/mol ; (106) 179_038_198_175_073_065_183

H	+0.00000	+0.00000	+0.00000
O	+0.00000	+0.00000	+0.96373
C	+1.27777	+0.00000	+1.37621
C	+1.39577	+0.03285	+2.89023
N	+0.36127	−0.79179	+3.49725
H	+0.23035	−0.55962	+4.47473
O	+2.19979	+0.01521	+0.61338
C	+2.79332	−0.42791	+3.29535
H	+1.29431	+1.10503	+3.14993
H	−0.52549	−0.66622	+3.02634
C	+3.04114	−0.30846	+4.80273
H	+2.92930	−1.46660	+2.97647
H	+3.52864	+0.17664	+2.75869
C	+2.30171	−1.34406	+5.61162
H	+4.10890	−0.44542	+5.00103
H	+2.74577	+0.67579	+5.17653
O	+2.73612	−2.58353	+5.35456

H +2.19955 −3.19958 +5.86572
O +1.42906 −1.11599 +6.40509
19
∗ E = +4.014 kcal/mol ; (107) 184_079_299_055_051_273_177
H +0.00000 +0.00000 +0.00000
O +0.00000 +0.00000 +0.96398
C +1.27464 +0.00000 +1.37998
C +1.37346 −0.09296 +2.89653
N +1.17848 −1.47177 +3.33180
H +0.26323 −1.81558 +3.06780
O +2.20513 −0.00879 +0.62278
C +2.72376 +0.40611 +3.39594
H +0.56010 +0.50564 +3.32182
H +1.87597 −2.07970 +2.91661
C +3.08577 +1.83152 +2.94899
H +2.71249 +0.34047 +4.48747
H +3.50284 −0.27234 +3.03063
C +1.96883 +2.79200 +3.25680
H +3.97432 +2.16883 +3.48429
H +3.28098 +1.84150 +1.87411
O +1.06091 +2.83044 +2.25927
H +0.35023 +3.42478 +2.52638
O +1.83917 +3.42965 +4.26185
19
∗ E = +4.042 kcal/mol ; (108) 357_015_094_170_178_306_180
H +0.00000 +0.00000 +0.00000
O +0.00000 +0.00000 +0.97136
C +1.27015 +0.00000 +1.36075
C +2.29032 −0.08204 +0.21178
N +1.64136 +0.22430 −1.06150
H +1.73273 +1.20616 −1.29779
O +1.59962 +0.03119 +2.50915
C +3.50981 +0.77769 +0.53697
H +2.59510 −1.13722 +0.18405
H +2.01330 −0.31858 −1.82785
C +4.64656 +0.54081 −0.45600
H +3.21691 +1.83488 +0.52480
H +3.83184 +0.55155 +1.55734
C +5.84339 +1.41677 −0.19215
H +4.98912 −0.49652 −0.42824

H	+4.30512	+0.75903	−1.47627
O	+5.50690	+2.71785	−0.13881
H	+6.30893	+3.22418	+0.03277
O	+6.96946	+1.03665	−0.05209

19

∗ E = +4.066 kcal/mol ; (109) 182_060_298_174_294_337_182

H	+0.00000	+0.00000	+0.00000
O	+0.00000	+0.00000	+0.96369
C	+1.27812	+0.00000	+1.38132
C	+1.36418	−0.03996	+2.90141
N	+0.73481	−1.23423	+3.45342
H	−0.25259	−1.26518	+3.23258
O	+2.20457	+0.00189	+0.62233
C	+2.81619	+0.05753	+3.35115
H	+0.80412	+0.82927	+3.27097
H	+1.16406	−2.07272	+3.07764
C	+2.94134	+0.16329	+4.86631
H	+3.35051	−0.83384	+3.00190
H	+3.28930	+0.91172	+2.86038
C	+2.38789	+1.43070	+5.47130
H	+2.43853	−0.66761	+5.36647
H	+3.99599	+0.12491	+5.16265
O	+2.32865	+2.45378	+4.59591
H	+2.00126	+3.22374	+5.07463
O	+2.05366	+1.55592	+6.61414

19

∗ E = +4.073 kcal/mol ; (110) 182_345_290_292_178_181_180

H	+0.00000	+0.00000	+0.00000
O	+0.00000	+0.00000	+0.96434
C	+1.28129	+0.00000	+1.37112
C	+1.43396	−0.04350	+2.89030
N	+0.23996	+0.24958	+3.65874
H	−0.01353	+1.22617	+3.58152
O	+2.19970	−0.00329	+0.60242
C	+2.00916	−1.41601	+3.26515
H	+2.19556	+0.70689	+3.12624
H	−0.55179	−0.28234	+3.31714
C	+1.01990	−2.54272	+3.00775
H	+2.92649	−1.59112	+2.69590
H	+2.27620	−1.39823	+4.32481

C +1.60706 −3.88922 +3.33771
H +0.11210 −2.42891 +3.61235
H +0.68864 −2.57247 +1.96129
O +0.74354 −4.88700 +3.08451
H +1.17556 −5.71635 +3.31767
O +2.70439 −4.08129 +3.77666
19
* E = +4.083 kcal/mol ; (111) 183_191_208_261_052_024_183
H +0.00000 +0.00000 +0.00000
O +0.00000 +0.00000 +0.96331
C +1.27351 +0.00000 +1.37207
C +1.40016 −0.07402 +2.88425
N +2.78376 +0.18676 +3.22559
H +3.06030 −0.26756 +4.08454
O +2.19979 −0.02654 +0.61091
C +0.42872 +0.86912 +3.63735
H +1.08568 −1.10940 +3.10502
H +3.39553 −0.11190 +2.47520
C +1.07442 +2.17287 +4.10279
H −0.43492 +1.07564 +3.00053
H +0.04626 +0.35108 +4.52193
C +1.81090 +2.94296 +3.03814
H +0.30070 +2.83989 +4.50101
H +1.79494 +2.00966 +4.90600
O +1.36634 +2.65238 +1.79926
H +1.90430 +3.14674 +1.17035
O +2.66432 +3.75691 +3.23655
19
* E = +4.098 kcal/mol ; (112) 003_345_151_301_187_052_179
H +0.00000 +0.00000 +0.00000
O +0.00000 +0.00000 +0.97141
C +1.27161 +0.00000 +1.36375
C +2.29596 +0.06515 +0.22305
N +1.65607 −0.24749 −1.05334
H +2.11917 +0.21819 −1.82422
O +1.60052 +0.00069 +2.51226
C +2.88363 +1.47993 +0.20226
H +3.09725 −0.63453 +0.48832
H +1.66446 −1.24127 −1.24541
C +1.81175 +2.55339 −0.01581

H +3.38959 +1.65070 +1.15698
H +3.63915 +1.53741 −0.58885
C +2.39752 +3.92782 −0.20678
H +1.22627 +2.32383 −0.91527
H +1.12075 +2.60479 +0.82924
O +3.33838 +3.94135 −1.17151
H +3.66193 +4.84568 −1.25184
O +2.08242 +4.91349 +0.39275
19
* E = +4.125 kcal/mol ; (113) 356_016_093_171_177_065_181
H +0.00000 +0.00000 +0.00000
O +0.00000 +0.00000 +0.97148
C +1.27011 +0.00000 +1.36093
C +2.28907 −0.08944 +0.21131
N +1.64078 +0.22706 −1.05978
H +1.73046 +1.21148 −1.28652
O +1.59977 +0.03497 +2.50912
C +3.52083 +0.75073 +0.53991
H +2.58146 −1.14820 +0.17952
H +2.01961 −0.30456 −1.83077
C +4.64920 +0.51729 −0.46631
H +3.23394 +1.81125 +0.54290
H +3.85349 +0.51087 +1.55280
C +5.83852 +1.39377 −0.17265
H +4.97003 −0.53016 −0.41780
H +4.32994 +0.73946 −1.48715
O +6.40188 +1.08729 +1.00653
H +7.13900 +1.69098 +1.15228
O +6.24525 +2.27413 −0.87560
19
* E = +4.145 kcal/mol ; (114) 352_029_083_073_226_066_003
H +0.00000 +0.00000 +0.00000
O +0.00000 +0.00000 +0.97202
C +1.26018 +0.00000 +1.36455
C +2.28101 −0.19800 +0.23594
N +1.70951 +0.34013 −0.99445
H +1.84427 +1.34394 −1.05314
O +1.56760 +0.12735 +2.52055
C +3.65165 +0.34273 +0.64604
H +2.35262 −1.28764 +0.10478

H +2.13227 −0.07431 −1.81423
C +4.37049 −0.55220 +1.68093
H +4.26940 +0.43287 −0.25171
H +3.52139 +1.35808 +1.04144
C +4.96366 +0.25990 +2.81933
H +3.66168 −1.26893 +2.11123
H +5.18237 −1.11390 +1.21848
O +4.06468 +0.86097 +3.60237
H +3.16068 +0.63065 +3.33372
O +6.13876 +0.38012 +3.00393
19
∗ E = +4.191 kcal/mol ; (115) 177_166_029_303_188_058_180
H +0.00000 +0.00000 +0.00000
O +0.00000 +0.00000 +0.96399
C +1.27140 +0.00000 +1.38295
C +1.36718 +0.07052 +2.89249
N +2.71679 −0.26792 +3.28431
H +3.37917 −0.01635 +2.56023
O +2.20760 +0.00072 +0.63527
C +0.88751 +1.46716 +3.34075
H +0.65921 −0.66575 +3.29478
H +2.98131 +0.16682 +4.15769
C +1.68266 +2.59135 +2.67304
H −0.17867 +1.57352 +3.11438
H +0.99962 +1.52864 +4.42845
C +1.34847 +3.94292 +3.24334
H +2.75547 +2.41818 +2.82385
H +1.49544 +2.63376 +1.59737
O +1.57616 +3.99606 +4.56946
H +1.34701 +4.88200 +4.87163
O +0.93172 +4.88121 +2.62758
19
∗ E = +4.234 kcal/mol ; (116) 176_159_039_174_182_180_180
H +0.00000 +0.00000 +0.00000
O +0.00000 +0.00000 +0.96389
C +1.27302 +0.00000 +1.37964
C +1.37887 +0.08744 +2.88770
N +2.68600 −0.40191 +3.27037
H +3.38542 −0.08794 +2.60689
O +2.20679 −0.01752 +0.63011

C	+1.05634	+1.54071	+3.28695
H	+0.60100	−0.56508	+3.30798
H	+2.95088	−0.10963	+4.20029
C	+1.00021	+1.71475	+4.79562
H	+1.81437	+2.20908	+2.86204
H	+0.09408	+1.83639	+2.85585
C	+0.64139	+3.12453	+5.18757
H	+0.25995	+1.04400	+5.24924
H	+1.95521	+1.47920	+5.28023
O	+0.60181	+3.27651	+6.52117
H	+0.36590	+4.19209	+6.70822
O	+0.41307	+4.02062	+4.42688

19

* E = +4.235 kcal/mol ; (117) 179_048_294_286_277_126_180

H	+0.00000	+0.00000	+0.00000
O	+0.00000	+0.00000	+0.96375
C	+1.27793	+0.00000	+1.37830
C	+1.37661	+0.01807	+2.90294
N	+0.51242	−0.96408	+3.54314
H	−0.46300	−0.74002	+3.39310
O	+2.20110	−0.01068	+0.61386
C	+2.82755	−0.14437	+3.34476
H	+1.02512	+1.01221	+3.20880
H	+0.66574	−1.89401	+3.16654
C	+3.33176	−1.58176	+3.17467
H	+3.45295	+0.53205	+2.75811
H	+2.91073	+0.13345	+4.39936
C	+2.92828	−2.45246	+4.34090
H	+2.99571	−2.01992	+2.23049
H	+4.42586	−1.58383	+3.16278
O	+2.26894	−3.56464	+3.96116
H	+2.05767	−4.06226	+4.75961
O	+3.17563	−2.21504	+5.48710

19

* E = +4.275 kcal/mol ; (118) 178_031_204_299_261_061_359

H	+0.00000	+0.00000	+0.00000
O	+0.00000	+0.00000	+0.96427
C	+1.27467	+0.00000	+1.38378
C	+1.39248	+0.05087	+2.89961
N	+0.25540	−0.61355	+3.53833

H +0.12176 −0.24922 +4.47497
O +2.20556 +0.00443 +0.63410
C +2.72267 −0.56288 +3.33613
H +1.41462 +1.12940 +3.12995
H −0.60204 −0.45523 +3.02251
C +2.88994 −2.04712 +2.94935
H +3.52199 +0.01837 +2.87104
H +2.82533 −0.43519 +4.41998
C +2.59720 −3.02632 +4.07553
H +2.23657 −2.29315 +2.10220
H +3.91289 −2.24205 +2.62885
O +1.35843 −2.98183 +4.57096
H +0.83290 −2.29327 +4.12052
O +3.41213 −3.79252 +4.50059
19
* E = +4.282 kcal/mol ; (119) 356_021_086_297_182_043_179
H +0.00000 +0.00000 +0.00000
O +0.00000 +0.00000 +0.97075
C +1.26979 +0.00000 +1.36459
C +2.29167 −0.10678 +0.22108
N +1.66961 +0.30150 −1.03619
H +1.71389 +1.30496 −1.17089
O +1.59379 +0.04484 +2.51433
C +3.59786 +0.59524 +0.59645
H +2.49543 −1.18233 +0.13930
H +2.11185 −0.13569 −1.83313
C +3.41676 +2.09636 +0.82093
H +3.97829 +0.14003 +1.51572
H +4.33615 +0.41992 −0.19178
C +4.70229 +2.81296 +1.14898
H +3.02775 +2.58278 −0.08446
H +2.71805 +2.29514 +1.63691
O +5.72647 +2.39929 +0.37897
H +6.50427 +2.90992 +0.63070
O +4.82780 +3.67152 +1.97222
19
* E = +4.312 kcal/mol ; (120) 182_348_286_166_098_302_001
H +0.00000 +0.00000 +0.00000
O +0.00000 +0.00000 +0.96448
C +1.27573 +0.00000 +1.37930

C	+1.41479	−0.04835	+2.89698
N	+0.18558	+0.18818	+3.64316
H	−0.08009	+1.16516	+3.62252
O	+2.20644	+0.01191	+0.62809
C	+2.04434	−1.39437	+3.28484
H	+2.13348	+0.73933	+3.15148
H	−0.58094	−0.33001	+3.22363
C	+2.54569	−1.43659	+4.74101
H	+1.31504	−2.19531	+3.10450
H	+2.88246	−1.57292	+2.60667
C	+1.59806	−2.09816	+5.73223
H	+3.48194	−1.99132	+4.80444
H	+2.74501	−0.41565	+5.09272
O	+0.37875	−1.56641	+5.81429
H	+0.28046	−0.81930	+5.19152
O	+1.91993	−3.03416	+6.40486

19

∗ E = +4.325 kcal/mol ; (121) 006_337_154_060_173_311_181

H	+0.00000	+0.00000	+0.00000
O	+0.00000	+0.00000	+0.97137
C	+1.27090	+0.00000	+1.36384
C	+2.28744	+0.13949	+0.22546
N	+1.68185	−0.29282	−1.03019
H	+2.11721	+0.15361	−1.82719
O	+1.59844	−0.05870	+2.51235
C	+2.70211	+1.60993	+0.09755
H	+3.16506	−0.45026	+0.52146
H	+1.74628	−1.29478	−1.15950
C	+3.33673	+2.17695	+1.36720
H	+3.41779	+1.68578	−0.72886
H	+1.82165	+2.20191	−0.18417
C	+3.89011	+3.56361	+1.16319
H	+2.63184	+2.21677	+2.19786
H	+4.16901	+1.53382	+1.67978
O	+4.66907	+3.64374	+0.06585
H	+5.00175	+4.54655	+0.01050
O	+3.69727	+4.50464	+1.87630

19

∗ E = +4.340 kcal/mol ; (122) 003_349_144_299_277_173_357

H	+0.00000	+0.00000	+0.00000

O	+0.00000	+0.00000	+0.97492
C	+1.27667	+0.00000	+1.34771
C	+2.27928	+0.06983	+0.18413
N	+1.59206	−0.14008	−1.08589
H	+1.98052	+0.43173	−1.82761
O	+1.62334	+0.00296	+2.49250
C	+2.96100	+1.44211	+0.25703
H	+3.04135	−0.69138	+0.39020
H	+1.61817	−1.10894	−1.37456
C	+1.99931	+2.62002	+0.10723
H	+3.45182	+1.50249	+1.23116
H	+3.73090	+1.50536	−0.51853
C	+1.68070	+2.96861	−1.33399
H	+1.06110	+2.44485	+0.65226
H	+2.44004	+3.52132	+0.55150
O	+0.73107	+3.90006	−1.50195
H	+0.36211	+4.17825	−0.66036
O	+2.23419	+2.49704	−2.28384

19

∗ E = +4.348 kcal/mol ; (123) 354_023_086_072_173_038_178

H	+0.00000	+0.00000	+0.00000
O	+0.00000	+0.00000	+0.97115
C	+1.26947	+0.00000	+1.36425
C	+2.28482	−0.14699	+0.21941
N	+1.66137	+0.29901	−1.02623
H	+1.75561	+1.30293	−1.13825
O	+1.58678	+0.07900	+2.51503
C	+3.61551	+0.53481	+0.54904
H	+2.45084	−1.22977	+0.12588
H	+2.08703	−0.13813	−1.83256
C	+4.43580	−0.20955	+1.60243
H	+4.19267	+0.60191	−0.38129
H	+3.42780	+1.56132	+0.88327
C	+5.80771	+0.37188	+1.81256
H	+3.91068	−0.18041	+2.56290
H	+4.57238	−1.26117	+1.33591
O	+5.80786	+1.71664	+1.76064
H	+6.70773	+2.01465	+1.93485
O	+6.80492	−0.25855	+2.02033

19

∗ E = +4.351 kcal/mol ; (124) 176_156_051_184_304_100_183
H +0.00000 +0.00000 +0.00000
O +0.00000 +0.00000 +0.96401
C +1.27371 +0.00000 +1.37945
C +1.38290 +0.09209 +2.88678
N +2.65604 −0.46078 +3.28277
H +3.41580 −0.05271 +2.75163
O +2.20762 −0.00690 +0.63039
C +1.12785 +1.57326 +3.25386
H +0.57748 −0.51631 +3.31888
H +2.82151 −0.36496 +4.27516
C +1.30757 +1.85208 +4.75122
H +1.82493 +2.20611 +2.69213
H +0.10980 +1.84363 +2.95331
C +0.41850 +0.93775 +5.55360
H +2.34900 +1.70881 +5.04942
H +1.01831 +2.88195 +4.96969
O +1.09468 −0.11811 +6.04577
H +0.46757 −0.68856 +6.50585
O −0.75967 +1.07676 +5.71070
19
∗ E = +4.352 kcal/mol ; (125) 180_054_183_073_062_038_178
H +0.00000 +0.00000 +0.00000
O +0.00000 +0.00000 +0.96385
C +1.27562 +0.00000 +1.38217
C +1.35706 −0.01016 +2.89964
N +0.57043 −1.13851 +3.38231
H +0.55457 −1.14449 +4.39450
O +2.20422 +0.01639 +0.62760
C +2.80270 −0.13570 +3.36552
H +0.95156 +0.96656 +3.22721
H −0.38530 −1.08412 +3.05210
C +3.60960 +1.14353 +3.15498
H +2.79721 −0.39529 +4.42887
H +3.27408 −0.96155 +2.82588
C +3.08171 +2.32977 +3.92192
H +4.64089 +0.98834 +3.49296
H +3.65331 +1.42484 +2.10142
O +2.62046 +1.99155 +5.14379
H +2.33398 +2.80284 +5.57826

O +3.07043 +3.46255 +3.53382
19
* E = +4.355 kcal/mol ; (126) 181_313_292_173_295_330_182
H +0.00000 +0.00000 +0.00000
O +0.00000 +0.00000 +0.96363
C +1.28040 +0.00000 +1.38069
C +1.37460 −0.03510 +2.89862
N +0.50468 +0.91253 +3.57395
H +0.79717 +1.86838 +3.41758
O +2.20572 +0.00359 +0.62165
C +1.05432 −1.45995 +3.37095
H +2.41686 +0.19890 +3.13990
H −0.44858 +0.81987 +3.24136
C +1.30450 −1.62777 +4.86586
H −0.00059 −1.66600 +3.15382
H +1.65364 −2.17647 +2.80083
C +2.75059 −1.49297 +5.27700
H +0.73874 −0.89528 +5.44452
H +0.98351 −2.62428 +5.19113
O +3.60338 −1.89264 +4.31513
H +4.49859 −1.80196 +4.66064
O +3.13030 −1.10265 +6.34328
19
* E = +4.356 kcal/mol ; (127) 003_339_164_181_306_094_182
H +0.00000 +0.00000 +0.00000
O +0.00000 +0.00000 +0.97088
C +1.27143 +0.00000 +1.36935
C +2.29139 +0.08053 +0.22874
N +1.67431 −0.35474 −1.01956
H +2.22528 −0.06978 −1.81965
O +1.59316 −0.01207 +2.51904
C +2.73234 +1.54731 +0.14680
H +3.14941 −0.53477 +0.53407
H +1.61766 −1.36614 −1.05896
C +3.75765 +1.83763 −0.95924
H +1.85154 +2.17803 −0.01973
H +3.15568 +1.81844 +1.11825
C +4.95521 +0.93226 −0.82775
H +3.31637 +1.72009 −1.95310
H +4.10556 +2.86764 −0.86211

O +4.83713 −0.17199 −1.59341
H +5.61523 −0.72175 −1.44213
O +5.89346 +1.11908 −0.11106
19
∗ E = +4.376 kcal/mol ; (128) 178_148_294_058_172_047_179
H +0.00000 +0.00000 +0.00000
O +0.00000 +0.00000 +0.96472
C +1.27463 +0.00000 +1.38402
C +1.39461 +0.04595 +2.90268
N +2.56729 −0.65086 +3.38883
H +2.50397 −1.64474 +3.20505
O +2.20725 −0.01193 +0.63193
C +1.46759 +1.51083 +3.35199
H +0.49481 −0.41390 +3.32707
H +3.38833 −0.31343 +2.89618
C +0.26386 +2.34781 +2.91858
H +1.55702 +1.50235 +4.44268
H +2.39100 +1.95230 +2.95991
C +0.26659 +3.72384 +3.53065
H +0.26073 +2.48137 +1.82990
H −0.68070 +1.86990 +3.18856
O +1.46819 +4.31857 +3.42748
H +1.39873 +5.19355 +3.82553
O −0.67259 +4.26002 +4.04493
19
∗ E = +4.377 kcal/mol ; (129) 183_342_295_170_176_314_181
H +0.00000 +0.00000 +0.00000
O +0.00000 +0.00000 +0.96415
C +1.27878 +0.00000 +1.37233
C +1.42413 −0.06167 +2.88910
N +0.25174 +0.29294 +3.66101
H −0.00455 +1.26259 +3.52737
O +2.20207 +0.01307 +0.60955
C +1.88521 −1.48070 +3.25001
H +2.23442 +0.63562 +3.13422
H −0.54334 −0.26923 +3.37871
C +2.29077 −1.56353 +4.71921
H +1.06644 −2.18271 +3.05042
H +2.72089 −1.75917 +2.60015
C +2.65404 −2.95319 +5.16515

H	+3.14643	−0.91880	+4.93268
H	+1.45312	−1.22024	+5.33764
O	+1.75869	−3.87115	+4.75399
H	+2.04379	−4.72721	+5.09242
O	+3.60883	−3.24710	+5.82607

19

∗ E = +4.377 kcal/mol ; (130) 180_050_301_171_176_309_181

H	+0.00000	+0.00000	+0.00000
O	+0.00000	+0.00000	+0.96371
C	+1.27853	+0.00000	+1.38017
C	+1.36778	+0.00550	+2.90282
N	+0.54908	−1.02399	+3.52774
H	−0.42590	−0.92084	+3.27558
O	+2.20371	−0.00536	+0.62047
C	+2.81878	−0.12096	+3.34705
H	+0.97034	+0.97879	+3.22587
H	+0.84860	−1.94621	+3.23007
C	+2.94959	+0.09479	+4.85372
H	+3.19113	−1.11529	+3.07425
H	+3.43013	+0.59880	+2.79498
C	+4.34865	−0.12061	+5.36111
H	+2.65258	+1.10677	+5.13879
H	+2.28828	−0.60801	+5.37362
O	+4.85653	−1.30098	+4.95984
H	+5.74387	−1.37707	+5.32780
O	+4.96755	+0.63660	+6.05340

19

∗ E = +4.386 kcal/mol ; (131) 000_355_127_170_113_312_180

H	+0.00000	+0.00000	+0.00000
O	+0.00000	+0.00000	+0.97351
C	+1.27240	+0.00000	+1.34780
C	+2.28384	+0.01184	+0.18846
N	+1.60399	−0.08858	−1.09947
H	+1.87626	+0.64549	−1.74134
O	+1.62274	+0.00194	+2.49177
C	+3.12843	+1.28240	+0.30530
H	+2.93887	−0.85196	+0.36305
H	+1.73634	−0.98315	−1.54824
C	+4.33840	+1.29389	−0.65015
H	+2.48186	+2.14819	+0.11701

H	+3.46600	+1.36065	+1.34046
C	+4.26428	+2.33208	−1.73899
H	+5.27452	+1.46547	−0.11874
H	+4.42599	+0.32205	−1.15451
O	+3.06396	+2.32895	−2.36313
H	+3.08191	+3.00770	−3.04751
O	+5.14001	+3.07896	−2.05949

19

∗ E = +4.389 kcal/mol ; (132) 178_036_190_296_188_044_178

H	+0.00000	+0.00000	+0.00000
O	+0.00000	+0.00000	+0.96393
C	+1.27635	+0.00000	+1.37812
C	+1.39292	+0.04792	+2.89206
N	+0.32180	−0.71817	+3.51102
H	+0.29876	−0.55069	+4.50889
O	+2.20203	+0.01379	+0.61942
C	+2.75926	−0.46384	+3.34411
H	+1.34219	+1.12732	+3.12705
H	−0.58121	−0.47301	+3.12604
C	+2.96783	−1.93703	+2.99460
H	+3.53775	+0.14854	+2.87955
H	+2.83608	−0.32587	+4.42755
C	+4.21544	−2.52880	+3.58959
H	+2.11758	−2.51692	+3.37420
H	+3.02097	−2.08591	+1.91443
O	+4.39444	−2.16669	+4.87616
H	+5.19462	−2.60324	+5.18863
O	+4.97736	−3.26313	+3.02831

19

∗ E = +4.414 kcal/mol ; (133) 177_156_036_062_279_341_182

H	+0.00000	+0.00000	+0.00000
O	+0.00000	+0.00000	+0.96423
C	+1.27120	+0.00000	+1.39138
C	+1.35039	+0.07789	+2.90252
N	+2.62100	−0.48281	+3.31068
H	+3.33630	−0.24923	+2.63079
O	+2.21223	−0.03521	+0.65340
C	+1.13175	+1.53066	+3.37811
H	+0.52480	−0.53270	+3.29387
H	+2.90531	−0.13691	+4.21745

C	−0.22827	+2.13793	+3.03562
H	+1.23792	+1.51173	+4.46851
H	+1.94175	+2.15575	+2.98829
C	−0.39052	+2.67519	+1.63313
H	−1.04419	+1.42600	+3.19299
H	−0.43078	+2.98909	+3.69618
O	+0.78595	+2.91455	+1.02696
H	+0.60127	+3.28794	+0.15778
O	−1.44065	+2.90825	+1.10813

19

* E = +4.417 kcal/mol ; (134) 177_164_032_301_185_294_179

H	+0.00000	+0.00000	+0.00000
O	+0.00000	+0.00000	+0.96397
C	+1.27172	+0.00000	+1.38187
C	+1.37065	+0.07132	+2.89104
N	+2.70762	−0.31629	+3.28167
H	+3.38205	−0.04851	+2.57446
O	+2.20682	+0.00213	+0.63261
C	+0.93661	+1.48588	+3.33055
H	+0.63800	−0.63819	+3.29649
H	+2.97368	+0.08170	+4.17215
C	+1.80623	+2.58252	+2.70770
H	−0.11336	+1.64599	+3.06616
H	+1.00850	+1.53133	+4.42391
C	+1.42792	+3.93780	+3.24039
H	+2.86304	+2.42117	+2.92888
H	+1.67766	+2.59004	+1.61874
O	+0.18418	+4.28962	+2.86748
H	−0.00560	+5.15342	+3.25049
O	+2.11324	+4.63705	+3.93006

19

* E = +4.431 kcal/mol ; (135) 182_198_201_080_173_179_180

H	+0.00000	+0.00000	+0.00000
O	+0.00000	+0.00000	+0.96422
C	+1.27788	+0.00000	+1.37258
C	+1.41925	−0.04938	+2.88321
N	+2.76266	+0.38797	+3.20384
H	+3.04823	+0.06953	+4.11924
O	+2.19704	+0.00300	+0.60516
C	+0.39005	+0.81209	+3.62673

H +1.22819 −1.11076 +3.13893
H +3.42454 +0.06093 +2.51064
C −0.97992 +0.16749 +3.77448
H +0.79496 +1.01312 +4.62394
H +0.30589 +1.78999 +3.14211
C −1.88615 +0.98355 +4.65589
H −1.48104 +0.02753 +2.81260
H −0.90421 −0.83274 +4.22039
O −3.10733 +0.43254 +4.76469
H −3.63469 +0.99917 +5.33872
O −1.58962 +2.00354 +5.21027
19
∗ E = +4.457 kcal/mol ; (136) 178_149_294_057_172_304_180
H +0.00000 +0.00000 +0.00000
O +0.00000 +0.00000 +0.96477
C +1.27441 +0.00000 +1.38408
C +1.39447 +0.06079 +2.90200
N +2.56658 −0.63321 +3.39413
H +2.49731 −1.62963 +3.22674
O +2.20758 −0.02154 +0.63299
C +1.46739 +1.52917 +3.34067
H +0.49402 −0.39298 +3.33177
H +3.38599 −0.30863 +2.89014
C +0.27444 +2.37034 +2.88225
H +1.53920 +1.53477 +4.43110
H +2.39952 +1.95781 +2.95071
C +0.28504 +3.75033 +3.48656
H +0.25009 +2.49552 +1.79848
H −0.65921 +1.87908 +3.18368
O +0.32498 +3.71203 +4.83028
H +0.32593 +4.62091 +5.15061
O +0.25493 +4.77970 +2.87528
19
∗ E = +4.490 kcal/mol ; (137) 182_319_060_061_175_179_180
H +0.00000 +0.00000 +0.00000
O +0.00000 +0.00000 +0.96374
C +1.27506 +0.00000 +1.38151
C +1.37897 −0.04910 +2.89219
N +0.37700 +0.81961 +3.48572
H −0.55447 +0.52985 +3.21021

O	+2.20503	−0.03051	+0.62698
C	+1.29437	−1.52078	+3.33579
H	+2.37429	+0.34067	+3.13257
H	+0.42954	+0.78024	+4.49607
C	+2.41379	−2.38578	+2.77586
H	+1.32559	−1.54818	+4.43049
H	+0.31856	−1.93000	+3.04757
C	+2.35946	−3.78708	+3.32162
H	+2.38757	−2.44548	+1.68188
H	+3.40008	−1.96935	+3.01504
O	+3.33205	−4.56140	+2.81581
H	+3.24450	−5.43843	+3.20559
O	+1.55812	−4.19370	+4.11504

19

∗ E = +4.495 kcal/mol ; (138) 182_021_204_289_068_079_187

H	+0.00000	+0.00000	+0.00000
O	+0.00000	+0.00000	+0.96383
C	+1.26969	+0.00000	+1.37647
C	+1.41548	−0.05290	+2.88999
N	+0.22795	−0.57928	+3.53974
H	+0.16447	−0.25891	+4.49647
O	+2.20150	+0.09055	+0.62711
C	+2.66151	−0.86341	+3.27279
H	+1.61949	+0.99951	+3.15756
H	−0.61769	−0.31466	+3.05315
C	+2.51453	−2.36876	+3.00567
H	+3.52626	−0.46397	+2.73427
H	+2.84171	−0.71941	+4.34311
C	+2.46382	−2.65402	+1.52844
H	+3.36977	−2.89564	+3.43641
H	+1.58774	−2.73377	+3.45079
O	+3.68021	−2.62736	+0.96708
H	+3.56738	−2.69874	+0.01270
O	+1.46225	−2.84813	+0.89513

19

∗ E = +4.503 kcal/mol ; (139) 353_027_083_095_306_269_170

H	+0.00000	+0.00000	+0.00000
O	+0.00000	+0.00000	+0.97026
C	+1.26917	+0.00000	+1.36450
C	+2.28575	−0.16883	+0.22597

N +1.70038 +0.35083 −1.00835
H +1.80892 +1.35696 −1.07383
O +1.59027 +0.10306 +2.51204
C +3.65523 +0.40629 +0.63013
H +2.37743 −1.25830 +0.10675
H +2.12984 −0.05962 −1.82646
C +4.61613 −0.65005 +1.19959
H +4.12345 +0.84565 −0.25634
H +3.51440 +1.20377 +1.36685
C +3.98099 −1.39710 +2.34495
H +4.85291 −1.38709 +0.42721
H +5.53933 −0.17174 +1.53395
O +4.23313 −0.83482 +3.52822
H +3.67536 −1.26454 +4.18696
O +3.30354 −2.37893 +2.20872
19
∗ E = +4.504 kcal/mol ; (140) 176_159_059_185_081_216_183
H +0.00000 +0.00000 +0.00000
O +0.00000 +0.00000 +0.96389
C +1.27826 +0.00000 +1.37658
C +1.38098 +0.09025 +2.88494
N +2.67900 −0.40074 +3.28736
H +3.42496 +0.13694 +2.86077
O +2.20717 −0.00705 +0.62403
C +1.04794 +1.54888 +3.27416
H +0.61097 −0.56373 +3.31338
H +2.79361 −0.40318 +4.29226
C +1.24262 +1.78534 +4.76796
H +1.70236 +2.23383 +2.72136
H +0.01505 +1.76651 +2.98664
C +2.69314 +2.01759 +5.11724
H +0.71014 +2.69017 +5.08262
H +0.84964 +0.95971 +5.37006
O +3.02672 +1.47986 +6.30297
H +3.94744 +1.70525 +6.48059
O +3.47535 +2.62690 +4.44535
19
∗ E = +4.518 kcal/mol ; (141) 178_038_188_296_187_327_182
H +0.00000 +0.00000 +0.00000
O +0.00000 +0.00000 +0.96385

C	+1.27562	+0.00000	+1.37942
C	+1.38503	+0.04444	+2.89384
N	+0.34509	−0.77705	+3.49614
H	+0.32550	−0.64321	+4.49919
O	+2.20210	+0.01608	+0.62107
C	+2.77204	−0.40300	+3.34873
H	+1.28034	+1.11842	+3.13655
H	−0.56888	−0.54770	+3.12736
C	+3.06268	−1.85942	+2.99188
H	+3.52486	+0.25011	+2.90070
H	+2.83455	−0.26390	+4.43488
C	+4.34761	−2.38349	+3.57303
H	+2.25741	−2.52030	+3.31721
H	+3.14815	−1.95763	+1.90238
O	+5.30112	−1.43683	+3.65957
H	+6.09397	−1.85629	+4.01191
O	+4.54430	−3.51500	+3.91515

19

∗ E = +4.538 kcal/mol ; (142) 183_330_063_058_059_270_003

H	+0.00000	+0.00000	+0.00000
O	+0.00000	+0.00000	+0.96474
C	+1.25921	+0.00000	+1.38827
C	+1.39470	−0.06566	+2.89369
N	+0.25825	+0.56030	+3.53897
H	−0.60353	+0.07446	+3.31945
O	+2.19495	−0.02250	+0.62532
C	+1.62857	−1.54247	+3.29068
H	+2.29373	+0.51212	+3.13861
H	+0.37655	+0.55781	+4.54409
C	+2.85700	−2.20738	+2.65413
H	+1.73667	−1.56608	+4.38069
H	+0.72782	−2.11692	+3.04216
C	+4.12848	−1.45951	+3.02618
H	+2.95695	−3.22105	+3.04584
H	+2.74910	−2.26764	+1.56664
O	+4.49282	−0.47130	+2.20318
H	+3.84483	−0.33940	+1.49303
O	+4.75424	−1.70571	+4.01535

19

∗ E = +4.548 kcal/mol ; (143) 175_153_037_292_075_027_179

H	+0.00000	+0.00000	+0.00000
O	+0.00000	+0.00000	+0.96370
C	+1.26925	+0.00000	+1.37898
C	+1.38114	+0.12019	+2.88323
N	+2.61810	−0.50982	+3.29251
H	+3.35004	−0.33182	+2.61370
O	+2.20823	−0.04308	+0.63420
C	+1.23493	+1.61025	+3.27505
H	+0.52793	−0.41553	+3.31541
H	+2.92094	−0.19296	+4.20397
C	+2.39340	+2.49775	+2.82574
H	+0.28431	+1.99591	+2.89223
H	+1.17739	+1.64625	+4.36860
C	+2.45679	+2.85412	+1.35902
H	+2.33924	+3.45818	+3.35234
H	+3.36272	+2.06353	+3.08478
O	+1.24724	+2.85405	+0.76686
H	+1.38118	+3.11263	−0.15219
O	+3.45440	+3.16504	+0.77798

19

* E = +4.569 kcal/mol ; (144) 183_199_201_084_060_032_178

H	+0.00000	+0.00000	+0.00000
O	+0.00000	+0.00000	+0.96409
C	+1.27593	+0.00000	+1.37610
C	+1.40750	−0.06528	+2.88607
N	+2.73945	+0.38625	+3.23089
H	+3.01022	+0.05244	+4.14589
O	+2.19989	+0.01612	+0.61460
C	+0.35267	+0.77274	+3.62652
H	+1.23304	−1.13256	+3.12747
H	+3.41494	+0.07266	+2.54433
C	−0.98784	+0.06947	+3.82076
H	+0.77582	+1.02108	+4.60373
H	+0.20409	+1.71993	+3.09921
C	−0.90222	−1.20906	+4.61745
H	−1.67123	+0.72750	+4.37089
H	−1.47273	−0.17504	+2.87474
O	+0.07742	−1.18203	+5.54386
H	+0.04067	−2.01565	+6.02657
O	−1.62187	−2.15684	+4.48546

19
∗ E = +4.573 kcal/mol ; (145) 182_317_068_184_308_283_176

H	+0.00000	+0.00000	+0.00000
O	+0.00000	+0.00000	+0.96360
C	+1.27682	+0.00000	+1.37920
C	+1.37378	−0.03899	+2.89092
N	+0.40159	+0.85631	+3.48538
H	−0.54741	+0.53507	+3.33721
O	+2.20564	−0.03181	+0.62505
C	+1.27332	−1.52216	+3.31240
H	+2.37385	+0.33459	+3.13627
H	+0.57511	+0.96871	+4.47710
C	+1.26257	−1.68759	+4.83820
H	+0.34959	−1.95104	+2.90648
H	+2.11507	−2.07470	+2.88083
C	+2.42477	−0.94761	+5.44290
H	+0.34906	−1.26861	+5.26443
H	+1.32400	−2.74718	+5.09777
O	+3.58233	−1.60428	+5.28810
H	+4.29020	−1.05535	+5.64516
O	+2.35878	+0.13495	+5.95808

19
∗ E = +4.573 kcal/mol ; (146) 176_146_046_054_057_030_178

H	+0.00000	+0.00000	+0.00000
O	+0.00000	+0.00000	+0.96447
C	+1.26689	+0.00000	+1.39502
C	+1.35410	+0.10254	+2.90287
N	+2.50315	−0.66749	+3.33581
H	+3.30214	−0.45148	+2.74901
O	+2.21376	−0.02017	+0.66109
C	+1.42402	+1.59758	+3.28322
H	+0.43981	−0.32862	+3.32446
H	+2.74099	−0.45790	+4.29685
C	+0.30598	+2.47202	+2.71672
H	+1.42313	+1.65610	+4.37679
H	+2.38754	+1.98579	+2.93335
C	−1.09751	+2.04351	+3.07084
H	+0.41724	+3.49459	+3.09684
H	+0.34658	+2.54218	+1.62698
O	−1.16360	+1.42932	+4.26750

H −2.08895 +1.21698 +4.43490
O −2.06798 +2.24889 +2.40237
19
* E = +4.578 kcal/mol ; (147) 357_010_104_314_287_178_357
H +0.00000 +0.00000 +0.00000
O +0.00000 +0.00000 +0.97638
C +1.27131 +0.00000 +1.34250
C +2.26997 −0.07122 +0.17527
N +1.57641 +0.12426 −1.09526
H +1.74109 +1.05513 −1.46537
O +1.62687 +0.04174 +2.48744
C +3.48154 +0.83692 +0.45863
H +2.63485 −1.10537 +0.20257
H +1.87251 −0.54667 −1.78934
C +3.16995 +2.24189 +0.97099
H +4.08895 +0.34573 +1.22301
H +4.08335 +0.90087 −0.45371
C +2.60091 +3.18904 −0.06095
H +2.49048 +2.19402 +1.83154
H +4.09232 +2.70518 +1.34479
O +2.28369 +4.40831 +0.39653
H +2.43265 +4.47432 +1.34280
O +2.44453 +2.92784 −1.21942
19
* E = +4.600 kcal/mol ; (148) 184_070_303_064_174_038_178
H +0.00000 +0.00000 +0.00000
O +0.00000 +0.00000 +0.96400
C +1.27585 +0.00000 +1.38403
C +1.36057 −0.09246 +2.90330
N +0.95856 −1.42074 +3.35429
H +0.02164 −1.64717 +3.04332
O +2.20515 +0.01306 +0.62681
C +2.76485 +0.22066 +3.39412
H +0.64567 +0.62946 +3.31837
H +1.58999 −2.12646 +2.99147
C +3.17955 +1.65932 +3.09399
H +2.78678 +0.02978 +4.47153
H +3.47346 −0.47032 +2.92472
C +4.50607 +2.04602 +3.69214
H +3.26744 +1.79956 +2.00961

```
H    +2.44438    +2.38008    +3.46227
O    +5.39929    +1.04238    +3.65149
H    +6.22435    +1.37092    +4.02584
O    +4.77415    +3.12282    +4.14406
19
 * E =  +4.607  kcal/mol ;  (149)  177_163_042_279_057_050_184
H    +0.00000    +0.00000    +0.00000
O    +0.00000    +0.00000    +0.96338
C    +1.27298    +0.00000    +1.37927
C    +1.36683    +0.06913    +2.89150
N    +2.69614    −0.34551    +3.27978
H    +3.39488    +0.04558    +2.65588
O    +2.20712    −0.02597    +0.63038
C    +0.92036    +1.47168    +3.37995
H    +0.62986    −0.65067    +3.26997
H    +2.91243    −0.08365    +4.23197
C    +2.00205    +2.54730    +3.25854
H    +0.01818    +1.77185    +2.83732
H    +0.64496    +1.38868    +4.43647
C    +2.53557    +2.73619    +1.86059
H    +1.58655    +3.50856    +3.58106
H    +2.86153    +2.32924    +3.89506
O    +1.54658    +2.87832    +0.95943
H    +1.95646    +2.93617    +0.08832
O    +3.69321    +2.76969    +1.56056
19
 * E =  +4.655  kcal/mol ;  (150)  183_334_301_056_278_345_181
H    +0.00000    +0.00000    +0.00000
O    +0.00000    +0.00000    +0.96385
C    +1.27560    +0.00000    +1.37206
C    +1.42215    −0.08145    +2.88511
N    +0.30457    +0.42224    +3.65662
H    +0.12178    +1.39672    +3.45199
O    +2.20298    +0.03548    +0.61146
C    +1.69684    −1.53584    +3.29625
H    +2.31499    +0.51074    +3.11843
H    −0.53933    −0.09244    +3.43106
C    +2.90750    −2.17624    +2.62214
H    +1.84766    −1.51545    +4.37899
H    +0.79770    −2.13469    +3.11107
```

C	+2.70926	−2.72774	+1.22939
H	+3.75113	−1.48220	+2.55937
H	+3.25464	−3.02726	+3.21897
O	+1.41191	−2.86483	+0.88933
H	+1.39091	−3.24021	+0.00178
O	+3.59670	−3.06658	+0.50347

19

∗ E = +4.705 kcal/mol ; (151) 180_050_186_304_106_212_181

H	+0.00000	+0.00000	+0.00000
O	+0.00000	+0.00000	+0.96388
C	+1.27675	+0.00000	+1.38010
C	+1.37136	−0.00125	+2.89520
N	+0.51371	−1.06372	+3.40988
H	+0.47800	−1.02781	+4.42088
O	+2.20314	+0.02223	+0.62286
C	+2.81239	−0.20229	+3.35471
H	+1.04074	+1.00611	+3.20575
H	−0.43135	−0.97531	+3.05758
C	+3.44438	−1.48222	+2.79741
H	+3.42136	+0.65546	+3.05320
H	+2.80577	−0.22083	+4.44928
C	+4.44992	−1.19227	+1.71018
H	+3.99689	−2.01643	+3.57840
H	+2.68545	−2.17633	+2.42725
O	+4.49731	−2.16703	+0.79011
H	+5.16939	−1.92189	+0.14457
O	+5.17623	−0.23962	+1.67743

19

∗ E = +4.727 kcal/mol ; (152) 181_056_302_174_180_059_180

H	+0.00000	+0.00000	+0.00000
O	+0.00000	+0.00000	+0.96373
C	+1.27805	+0.00000	+1.38160
C	+1.36294	−0.02017	+2.90383
N	+0.63830	−1.14060	+3.48917
H	−0.33670	−1.12901	+3.21668
O	+2.20478	+0.00093	+0.62371
C	+2.81817	−0.02351	+3.34871
H	+0.87653	+0.90154	+3.25393
H	+1.03561	−2.02201	+3.18238
C	+2.93499	+0.10539	+4.86813

H	+3.29041	−0.95414	+3.00822
H	+3.35373	+0.79017	+2.85266
C	+4.37002	+0.10184	+5.32096
H	+2.48411	+1.05298	+5.18816
H	+2.41444	−0.70780	+5.37383
O	+5.07263	+1.10774	+4.76728
H	+5.97856	+1.04576	+5.08965
O	+4.86718	−0.68323	+6.07684

19

∗ E = +4.738 kcal/mol ; (153) 181_060_183_085_304_267_172

H	+0.00000	+0.00000	+0.00000
O	+0.00000	+0.00000	+0.96368
C	+1.27352	+0.00000	+1.38153
C	+1.34898	−0.01376	+2.90120
N	+0.68104	−1.23650	+3.34309
H	+0.65228	−1.27488	+4.35427
O	+2.20509	−0.00858	+0.63010
C	+2.80013	−0.00296	+3.37509
H	+0.84346	+0.90879	+3.23708
H	−0.27316	−1.26556	+3.00404
C	+3.40069	+1.40782	+3.47166
H	+2.84078	−0.45767	+4.37016
H	+3.39872	−0.62860	+2.70663
C	+3.29352	+2.13955	+2.15908
H	+2.84776	+1.99387	+4.21112
H	+4.44811	+1.34549	+3.77390
O	+4.38396	+2.00273	+1.40045
H	+4.19721	+2.39125	+0.53823
O	+2.32548	+2.75953	+1.80938

19

∗ E = +4.752 kcal/mol ; (154) 176_147_033_307_298_108_347

H	+0.00000	+0.00000	+0.00000
O	+0.00000	+0.00000	+0.96486
C	+1.26046	+0.00000	+1.38847
C	+1.37939	+0.09778	+2.89202
N	+2.54990	−0.63916	+3.32264
H	+3.29322	−0.60637	+2.63634
O	+2.19886	−0.00004	+0.63055
C	+1.37564	+1.60292	+3.25538
H	+0.47683	−0.34898	+3.32472

H +2.91353 −0.28514 +4.19847
C +2.41167 +2.44453 +2.50128
H +0.37702 +2.01496 +3.07557
H +1.57129 +1.67897 +4.33101
C +3.83363 +1.99056 +2.79593
H +2.21174 +2.44423 +1.42472
H +2.34365 +3.47771 +2.85051
O +4.47823 +1.34693 +1.81297
H +3.86651 +1.08470 +1.11244
O +4.36202 +2.19386 +3.84920
19
* E = +4.758 kcal/mol ; (155) 183_322_295_171_176_041_178
H +0.00000 +0.00000 +0.00000
O +0.00000 +0.00000 +0.96382
C +1.27844 +0.00000 +1.37936
C +1.38745 −0.07018 +2.89631
N +0.39264 +0.69400 +3.62464
H +0.49247 +1.68806 +3.46395
O +2.20553 +0.01333 +0.62195
C +1.31242 −1.54896 +3.29768
H +2.38366 +0.31327 +3.14290
H −0.54163 +0.42887 +3.33305
C +1.62897 −1.73293 +4.77934
H +0.30168 −1.91835 +3.07802
H +2.00688 −2.13197 +2.68545
C +1.48953 −3.15565 +5.25269
H +2.66761 −1.43276 +4.97090
H +0.98868 −1.10614 +5.40101
O +1.96699 −4.04095 +4.35820
H +1.86702 −4.92156 +4.73687
O +1.02948 −3.49757 +6.30413
19
* E = +4.763 kcal/mol ; (156) 183_322_290_292_077_026_177
H +0.00000 +0.00000 +0.00000
O +0.00000 +0.00000 +0.96386
C +1.27646 +0.00000 +1.39108
C +1.35829 −0.07008 +2.91085
N +0.34172 +0.70058 +3.60770
H +0.50193 +1.69414 +3.50024
O +2.21100 +0.03218 +0.64523

C	+1.32932	−1.53578	+3.37566
H	+2.34155	+0.33383	+3.16774
H	−0.57946	+0.50861	+3.23126
C	−0.00061	−2.25728	+3.15402
H	+2.13984	−2.08779	+2.89103
H	+1.53176	−1.51379	+4.45004
C	−0.29744	−2.71123	+1.74486
H	−0.01266	−3.17319	+3.75749
H	−0.85617	−1.66823	+3.49433
O	+0.81969	−2.92764	+1.02798
H	+0.55749	−3.25896	+0.16184
O	−1.39185	−2.91015	+1.30217

19

* E = +4.774 kcal/mol ; (157) 353_026_083_307_103_218_179

H	+0.00000	+0.00000	+0.00000
O	+0.00000	+0.00000	+0.97055
C	+1.27011	+0.00000	+1.36396
C	+2.28775	−0.16030	+0.22615
N	+1.69393	+0.33414	−1.01602
H	+1.78843	+1.34141	−1.09362
O	+1.59441	+0.09319	+2.51117
C	+3.63617	+0.44827	+0.61542
H	+2.41046	−1.24501	+0.10828
H	+2.13620	−0.07638	−1.82748
C	+3.51504	+1.90192	+1.09242
H	+4.08953	−0.13854	+1.41987
H	+4.29852	+0.37735	−0.25242
C	+3.61888	+2.00922	+2.59839
H	+4.33180	+2.51213	+0.69244
H	+2.58165	+2.37624	+0.76866
O	+2.78628	+2.92972	+3.09935
H	+2.89731	+2.93661	+4.05678
O	+4.39269	+1.39049	+3.26942

19

* E = +4.789 kcal/mol ; (158) 182_319_073_181_083_201_183

H	+0.00000	+0.00000	+0.00000
O	+0.00000	+0.00000	+0.96359
C	+1.27303	+0.00000	+1.38057
C	+1.37422	−0.05364	+2.89253
N	+0.37295	+0.80586	+3.49779

H	−0.55297	+0.39980	+3.41980
O	+2.20644	−0.02258	+0.63012
C	+1.31680	−1.54097	+3.29810
H	+2.36483	+0.34233	+3.14015
H	+0.56940	+0.96576	+4.47755
C	+1.39776	−1.71966	+4.80826
H	+0.37857	−1.97801	+2.93569
H	+2.14227	−2.07007	+2.81173
C	+0.06596	−1.53483	+5.49351
H	+1.72516	−2.73450	+5.06367
H	+2.12792	−1.04291	+5.26625
O	+0.20940	−1.24816	+6.79689
H	−0.67076	−1.19111	+7.18635
O	−1.00919	−1.65264	+4.97641

19

∗ E = +4.803 kcal/mol ; (159) 179_040_194_178_186_181_180

H	+0.00000	+0.00000	+0.00000
O	+0.00000	+0.00000	+0.96371
C	+1.27936	+0.00000	+1.37632
C	+1.38998	+0.02864	+2.89037
N	+0.37392	−0.83526	+3.47813
H	+0.27797	−0.65542	+4.46910
O	+2.20160	+0.01985	+0.61586
C	+2.79109	−0.39322	+3.31381
H	+1.24829	+1.09462	+3.15727
H	−0.52921	−0.67686	+3.04911
C	+2.95468	−0.33797	+4.82367
H	+2.98260	−1.40897	+2.95412
H	+3.52908	+0.25296	+2.83130
C	+4.37045	−0.61732	+5.25434
H	+2.67745	+0.64560	+5.22649
H	+2.31290	−1.06824	+5.33048
O	+4.49729	−0.59406	+6.59273
H	+5.41944	−0.77808	+6.80367
O	+5.29227	−0.83616	+4.52301

19

∗ E = +4.809 kcal/mol ; (160) 183_315_054_295_085_189_182

H	+0.00000	+0.00000	+0.00000
O	+0.00000	+0.00000	+0.96406
C	+1.28542	+0.00000	+1.37223

C	+1.39077	−0.07981	+2.88341
N	+0.43508	+0.84077	+3.47845
H	−0.50474	+0.67850	+3.13927
O	+2.20620	+0.03710	+0.61423
C	+1.28127	−1.54630	+3.35750
H	+2.40120	+0.26762	+3.11739
H	+0.43529	+0.76706	+4.48778
C	−0.06480	−2.20675	+3.08343
H	+2.07730	−2.13662	+2.89118
H	+1.46656	−1.54032	+4.43701
C	−0.16338	−2.78808	+1.69628
H	−0.24355	−3.03524	+3.77829
H	−0.90460	−1.51739	+3.22436
O	−1.41215	−3.18693	+1.41033
H	−1.40231	−3.57817	+0.52964
O	+0.74718	−2.91635	+0.92795

19

∗ E = +4.810 kcal/mol ; (161) 167_137_048_050_278_304_177

H	+0.00000	+0.00000	+0.00000
O	+0.00000	+0.00000	+0.96565
C	+1.26121	+0.00000	+1.39339
C	+1.37517	+0.32601	+2.86845
N	+2.34775	−0.57523	+3.45532
H	+3.18285	−0.59101	+2.87855
O	+2.21220	−0.14809	+0.67654
C	+1.75418	+1.81950	+2.97658
H	+0.39454	+0.16529	+3.32977
H	+2.60951	−0.27377	+4.38560
C	+0.87177	+2.75999	+2.14002
H	+1.69429	+2.10304	+4.03214
H	+2.79943	+1.92806	+2.66633
C	+1.28317	+2.82338	+0.68569
H	−0.17991	+2.46460	+2.17301
H	+0.95517	+3.77942	+2.53099
O	+2.56546	+3.18834	+0.54892
H	+2.78217	+3.16869	−0.38997
O	+0.57449	+2.59569	−0.25505

19

∗ E = +4.856 kcal/mol ; (162) 180_049_182_295_136_299_358

H	+0.00000	+0.00000	+0.00000

O	+0.00000	+0.00000	+0.96468
C	+1.26077	+0.00000	+1.39402
C	+1.35124	+0.00144	+2.90976
N	+0.48130	−1.04494	+3.42667
H	+0.48916	−1.04409	+4.43896
O	+2.19707	+0.02508	+0.63783
C	+2.79371	−0.21679	+3.34873
H	+1.04037	+1.01854	+3.21171
H	−0.47453	−0.92134	+3.11693
C	+3.34107	−1.60726	+2.96508
H	+3.40643	+0.57679	+2.90831
H	+2.85144	−0.07974	+4.43290
C	+4.74684	−1.53203	+2.39875
H	+3.36312	−2.28136	+3.82046
H	+2.69208	−2.06868	+2.21122
O	+4.86317	−0.80885	+1.27717
H	+4.00250	−0.47184	+0.98672
O	+5.69629	−2.06156	+2.89569

19

∗ E = +4.892 kcal/mol ; (163) 183_319_299_060_179_053_179

H	+0.00000	+0.00000	+0.00000
O	+0.00000	+0.00000	+0.96382
C	+1.27705	+0.00000	+1.38146
C	+1.38280	−0.06760	+2.89912
N	+0.42666	+0.75363	+3.61597
H	+0.53942	+1.73537	+3.39660
O	+2.20564	+0.00855	+0.62395
C	+1.21344	−1.52919	+3.32955
H	+2.39755	+0.26412	+3.14378
H	−0.52121	+0.49222	+3.36879
C	+2.27218	−2.45389	+2.72726
H	+1.26462	−1.54766	+4.42277
H	+0.21093	−1.87102	+3.04591
C	+2.10992	−3.88036	+3.18013
H	+2.20981	−2.44658	+1.63199
H	+3.28053	−2.13474	+2.99889
O	+0.86037	−4.33516	+2.97131
H	+0.82745	−5.24988	+3.27281
O	+2.96600	−4.56292	+3.66457

19

∗ E = +4.904 kcal/mol ; (164) 178_166_015_298_244_046_179
H +0.00000 +0.00000 +0.00000
O +0.00000 +0.00000 +0.96386
C +1.27216 +0.00000 +1.38139
C +1.37472 +0.04464 +2.89236
N +2.71933 −0.29704 +3.29772
H +3.35466 −0.25330 +2.51003
O +2.20560 −0.00006 +0.62827
C +0.91681 +1.43714 +3.37094
H +0.64988 −0.68772 +3.27431
H +3.05894 +0.31097 +4.03101
C +1.80957 +2.57469 +2.83639
H −0.12076 +1.59557 +3.06539
H +0.92838 +1.42265 +4.46513
C +2.55521 +3.33039 +3.90361
H +2.57543 +2.16112 +2.16424
H +1.24305 +3.31072 +2.26591
O +3.14198 +2.50426 +4.79612
H +3.61622 +3.04893 +5.43444
O +2.65820 +4.51989 +3.97884
19
∗ E = +4.905 kcal/mol ; (165) 352_023_089_318_317_106_358
H +0.00000 +0.00000 +0.00000
O +0.00000 +0.00000 +0.97433
C +1.26371 +0.00000 +1.35623
C +2.27281 −0.19454 +0.21598
N +1.65083 +0.21444 −1.04244
H +1.79646 +1.20134 −1.23532
O +1.59443 +0.13038 +2.50525
C +3.64844 +0.41964 +0.54121
H +2.40145 −1.28365 +0.16254
H +2.02150 −0.30777 −1.82433
C +3.63119 +1.80015 +1.22496
H +4.20320 −0.26427 +1.18876
H +4.19628 +0.49051 −0.40363
C +2.59541 +2.70060 +0.58456
H +3.44291 +1.68243 +2.29353
H +4.60443 +2.27775 +1.09186
O +1.47742 +2.92125 +1.28633
H +1.48153 +2.43218 +2.11724

O +2.71443 +3.14917 −0.52121
19
* E = +4.924 kcal/mol ; (166) 182_334_051_300_189_186_181

H	+0.00000	+0.00000	+0.00000
O	+0.00000	+0.00000	+0.96410
C	+1.27769	+0.00000	+1.37622
C	+1.41473	−0.05473	+2.88533
N	+0.24380	+0.50062	+3.53851
H	−0.61529	+0.09954	+3.18473
O	+2.20204	−0.02837	+0.61584
C	+1.77377	−1.50471	+3.27070
H	+2.27953	+0.57378	+3.12175
H	+0.28463	+0.35518	+4.53906
C	+0.71945	−2.51103	+2.83596
H	+2.74084	−1.76608	+2.82906
H	+1.89997	−1.55282	+4.35750
C	+0.96975	−3.87812	+3.41489
H	−0.28605	−2.20480	+3.15276
H	+0.66923	−2.61065	+1.74532
O	+0.12001	−4.79308	+2.92087
H	+0.32432	−5.63975	+3.33360
O	+1.80998	−4.14841	+4.22479

19
* E = +4.925 kcal/mol ; (167) 185_074_303_081_298_078_183

H	+0.00000	+0.00000	+0.00000
O	+0.00000	+0.00000	+0.96380
C	+1.27627	+0.00000	+1.37986
C	+1.36885	−0.12199	+2.89507
N	+1.04930	−1.49182	+3.29589
H	+0.13071	−1.76195	+2.96447
O	+2.20442	+0.01509	+0.62112
C	+2.75962	+0.24848	+3.39641
H	+0.61329	+0.54307	+3.33049
H	+1.72339	−2.14118	+2.90429
C	+3.01716	+1.75595	+3.52628
H	+2.87480	−0.20210	+4.38599
H	+3.50879	−0.19943	+2.73378
C	+2.91782	+2.50153	+2.22060
H	+2.29043	+2.18904	+4.22226
H	+4.02151	+1.92508	+3.91700

O	+1.63108	+2.70209	+1.86110
H	+1.63490	+3.13876	+1.00141
O	+3.83432	+2.89257	+1.56112

19

∗ E = +4.944 kcal/mol ; (168) 181_307_298_053_176_300_180

H	+0.00000	+0.00000	+0.00000
O	+0.00000	+0.00000	+0.96381
C	+1.27674	+0.00000	+1.38587
C	+1.36308	−0.03003	+2.90599
N	+0.59721	+1.01564	+3.56054
H	+0.93506	+1.93735	+3.31348
O	+2.20826	−0.00053	+0.63316
C	+0.88777	−1.39457	+3.40863
H	+2.42337	+0.09828	+3.14750
H	−0.37941	+0.96058	+3.29378
C	+1.62745	−2.55418	+2.73719
H	+1.03397	−1.42132	+4.49149
H	−0.18973	−1.48062	+3.21897
C	+1.21653	−3.88585	+3.30708
H	+1.43882	−2.58880	+1.66155
H	+2.70741	−2.43684	+2.88779
O	+1.46343	−3.96195	+4.62708
H	+1.17770	−4.83066	+4.93101
O	+0.72446	−4.78865	+2.69337

19

∗ E = +4.946 kcal/mol ; (169) 352_320_297_311_296_254_179

H	+0.00000	+0.00000	+0.00000
O	+0.00000	+0.00000	+0.96953
C	+1.23598	+0.00000	+1.47560
C	+2.39898	−0.17904	+0.48607
N	+2.21143	−1.13663	−0.58924
H	+2.09578	−2.07568	−0.23264
O	+1.42535	+0.16050	+2.64638
C	+2.84336	+1.20203	−0.03279
H	+3.21999	−0.54952	+1.10666
H	+1.43523	−0.92549	−1.20105
C	+1.74316	+2.14826	−0.55048
H	+3.33484	+1.72442	+0.79277
H	+3.59328	+1.03292	−0.81199
C	+1.03343	+1.62987	−1.77002

H	+0.98519	+2.30093	+0.22610
H	+2.18673	+3.11636	−0.78913
O	+1.37034	+2.25839	−2.89058
H	+0.88641	+1.85904	−3.62407
O	+0.23133	+0.72340	−1.76762

19

∗ E = +4.953 kcal/mol ; (170) 178_039_298_292_181_324_182

H	+0.00000	+0.00000	+0.00000
O	+0.00000	+0.00000	+0.96391
C	+1.27802	+0.00000	+1.37705
C	+1.38697	+0.05170	+2.90056
N	+0.39369	−0.74751	+3.60027
H	−0.54165	−0.41923	+3.39561
O	+2.20033	−0.01651	+0.61187
C	+2.80588	−0.28856	+3.34791
H	+1.19515	+1.10216	+3.15968
H	+0.43350	−1.71825	+3.31305
C	+3.16934	−1.74884	+3.08498
H	+3.51340	+0.35908	+2.82500
H	+2.87797	−0.07463	+4.41845
C	+4.55375	−2.12689	+3.54482
H	+2.48612	−2.44208	+3.58165
H	+3.13312	−1.94898	+2.00603
O	+5.44384	−1.13476	+3.37639
H	+6.30288	−1.46184	+3.66609
O	+4.86270	−3.19368	+3.99453

19

∗ E = +4.959 kcal/mol ; (171) 355_022_088_074_068_186_003

H	+0.00000	+0.00000	+0.00000
O	+0.00000	+0.00000	+0.97212
C	+1.26948	+0.00000	+1.35888
C	+2.28154	−0.12623	+0.21265
N	+1.65096	+0.29139	−1.03736
H	+1.74452	+1.29159	−1.17807
O	+1.59180	+0.06565	+2.51163
C	+3.59385	+0.59498	+0.54326
H	+2.48704	−1.20055	+0.12018
H	+2.07971	−0.16918	−1.82950
C	+4.45579	−0.14501	+1.55826
H	+4.15942	+0.69607	−0.38863

H	+3.37097	+1.60027	+0.91770
C	+4.99570	−1.44169	+0.99100
H	+5.31732	+0.46797	+1.85097
H	+3.88382	−0.35037	+2.47010
O	+5.66049	−2.21956	+1.86163
H	+5.65165	−1.83576	+2.74154
O	+4.86978	−1.78210	−0.14815

19

* E = +4.968 kcal/mol ; (172) 181_310_296_051_064_019_178

H	+0.00000	+0.00000	+0.00000
O	+0.00000	+0.00000	+0.96381
C	+1.27954	+0.00000	+1.38188
C	+1.37938	−0.03234	+2.89960
N	+0.55746	+0.95640	+3.57397
H	+0.84870	+1.89958	+3.35047
O	+2.20628	−0.01051	+0.62340
C	+0.98507	−1.42953	+3.39167
H	+2.43075	+0.15303	+3.13752
H	−0.41381	+0.85856	+3.30018
C	+1.72129	−2.57101	+2.69782
H	+1.15767	−1.45321	+4.47067
H	−0.09222	−1.55539	+3.22916
C	+3.21758	−2.62109	+2.90619
H	+1.33862	−3.53208	+3.05978
H	+1.56094	−2.57389	+1.61477
O	+3.62437	−1.92132	+3.98088
H	+4.57960	−2.03002	+4.05268
O	+3.97648	−3.24496	+2.22358

19

* E = +5.014 kcal/mol ; (173) 182_343_046_293_289_312_183

H	+0.00000	+0.00000	+0.00000
O	+0.00000	+0.00000	+0.96410
C	+1.27237	+0.00000	+1.37852
C	+1.42082	−0.04727	+2.88913
N	+0.18405	+0.30245	+3.55989
H	−0.61821	−0.17052	+3.16157
O	+2.20215	−0.01891	+0.62251
C	+2.01528	−1.42323	+3.25368
H	+2.18004	+0.70894	+3.12225
H	+0.23431	+0.09373	+4.54860

C	+1.05390	−2.57916	+2.96939
H	+2.93586	−1.56469	+2.67944
H	+2.28373	−1.41948	+4.31501
C	−0.12898	−2.64990	+3.90164
H	+0.65889	−2.54204	+1.94991
H	+1.58904	−3.52928	+3.07899
O	+0.23451	−2.54949	+5.19559
H	−0.56078	−2.65650	+5.72969
O	−1.26980	−2.81204	+3.57324

19

∗ E = +5.024 kcal/mol ; (174) 183_322_072_326_295_163_178

H	+0.00000	+0.00000	+0.00000
O	+0.00000	+0.00000	+0.96408
C	+1.27692	+0.00000	+1.37912
C	+1.38726	−0.06651	+2.88921
N	+0.34346	+0.72795	+3.51122
H	−0.55871	+0.27554	+3.41554
O	+2.20309	−0.03244	+0.61982
C	+1.47146	−1.56727	+3.29788
H	+2.34882	+0.39539	+3.12949
H	+0.52732	+0.81630	+4.50266
C	+0.66812	−2.54292	+2.44002
H	+2.51892	−1.88011	+3.25414
H	+1.14676	−1.64076	+4.34095
C	−0.82340	−2.34630	+2.51270
H	+0.97274	−2.51021	+1.38828
H	+0.85799	−3.57242	+2.76775
O	−1.45887	−2.94587	+1.49786
H	−2.40524	−2.80917	+1.62041
O	−1.41003	−1.76081	+3.38149

19

∗ E = +5.028 kcal/mol ; (175) 178_037_297_289_176_031_179

H	+0.00000	+0.00000	+0.00000
O	+0.00000	+0.00000	+0.96395
C	+1.27881	+0.00000	+1.37539
C	+1.39294	+0.05559	+2.89887
N	+0.37155	−0.69998	+3.60682
H	−0.54849	−0.32039	+3.42240
O	+2.20033	−0.01719	+0.60984
C	+2.79727	−0.34660	+3.34087

H +1.24753 +1.11452 +3.15304
H +0.35387 −1.66559 +3.29956
C +3.05611 −1.83741 +3.13699
H +3.52889 +0.23527 +2.77332
H +2.91096 −0.09333 +4.39734
C +4.46107 −2.27389 +3.46404
H +2.39898 −2.42615 +3.79054
H +2.86475 −2.14709 +2.10550
O +5.02095 −1.53130 +4.43501
H +5.90183 −1.88586 +4.60028
O +5.03318 −3.19278 +2.95165
19
∗ E = +5.054 kcal/mol ; (176) 182_062_303_065_224_062_002
H +0.00000 +0.00000 +0.00000
O +0.00000 +0.00000 +0.96469
C +1.26155 +0.00000 +1.39485
C +1.35053 −0.06004 +2.91661
N +0.76315 −1.28698 +3.43250
H −0.20161 −1.39181 +3.14403
O +2.19646 +0.00650 +0.63337
C +2.80050 +0.06617 +3.35867
H +0.76355 +0.78288 +3.30539
H +1.28052 −2.09981 +3.11721
C +3.42081 +1.43725 +3.02238
H +2.83898 −0.11646 +4.43440
H +3.37301 −0.74020 +2.88264
C +4.82243 +1.31748 +2.44891
H +2.80762 +1.95983 +2.27684
H +3.47900 +2.07426 +3.90495
O +4.89988 +0.64209 +1.29615
H +4.02158 +0.36589 +0.99296
O +5.79529 +1.77629 +2.96900
19
∗ E = +5.065 kcal/mol ; (177) 177_144_295_311_295_091_356
H +0.00000 +0.00000 +0.00000
O +0.00000 +0.00000 +0.96529
C +1.26064 +0.00000 +1.39267
C +1.37763 +0.08001 +2.90867
N +2.47303 −0.68088 +3.46473
H +2.34918 −1.67470 +3.32424

O +2.19672 −0.01299 +0.63077
C +1.45828 +1.57118 +3.30024
H +0.44484 −0.31310 +3.32522
H +3.37516 −0.42123 +3.08483
C +2.46781 +2.43344 +2.52430
H +0.46775 +2.01719 +3.16621
H +1.69698 +1.60012 +4.36769
C +3.91709 +2.04002 +2.76305
H +2.24394 +2.42303 +1.45152
H +2.37288 +3.46304 +2.87467
O +4.43524 +1.13685 +1.91517
H +3.77239 +0.81101 +1.28631
O +4.57121 +2.48810 +3.65580
19
∗ E = +5.083 kcal/mol ; (178) 001_153_291_059_066_279_179
H +0.00000 +0.00000 +0.00000
O +0.00000 +0.00000 +0.96562
C +1.24547 +0.00000 +1.45500
C +2.38912 +0.01537 +0.42973
N +3.59235 +0.62553 +0.95724
H +3.45688 +1.61946 +1.09816
O +1.44795 −0.04020 +2.63222
C +2.72686 −1.43027 +0.02833
H +2.07149 +0.57926 −0.45609
H +3.78224 +0.23294 +1.87487
C +1.58733 −2.25728 −0.58813
H +3.57400 −1.38621 −0.66228
H +3.07525 −1.94794 +0.92963
C +1.16242 −1.71459 −1.92603
H +1.93438 −3.27738 −0.75761
H +0.72182 −2.29225 +0.08192
O +0.31965 −0.65226 −1.80603
H +0.09851 −0.34246 −2.69249
O +1.51024 −2.11070 −2.99603
19
∗ E = +5.111 kcal/mol ; (179) 182_347_290_290_175_042_179
H +0.00000 +0.00000 +0.00000
O +0.00000 +0.00000 +0.96443
C +1.27941 +0.00000 +1.37259
C +1.43191 −0.04660 +2.89236

```
N    +0.23020    +0.19994    +3.66352
H    −0.05913    +1.16672    +3.58924
O    +2.20112    +0.00354    +0.60781
C    +2.05394    −1.40088    +3.26102
H    +2.16759    +0.72849    +3.13186
H    −0.54201    −0.35963    +3.32080
C    +1.07270    −2.55111    +3.03495
H    +2.95603    −1.54766    +2.65849
H    +2.34806    −1.36949    +4.31258
C    +1.67643    −3.91034    +3.27217
H    +0.22917    −2.45560    +3.73071
H    +0.66727    −2.55684    +2.01845
O    +2.46293    −3.93300    +4.36196
H    +2.79980    −4.83051    +4.46154
O    +1.48525    −4.88192    +2.59864
19
 * E =   +5.125  kcal/mol  ;  (180)  182_319_069_177_072_053_178
H    +0.00000    +0.00000    +0.00000
O    +0.00000    +0.00000    +0.96366
C    +1.27522    +0.00000    +1.37995
C    +1.37506    −0.04953    +2.89229
N    +0.37461    +0.81302    +3.49143
H    −0.56163    +0.44927    +3.35967
O    +2.20611    −0.02730    +0.62764
C    +1.31312    −1.53573    +3.29859
H    +2.36582    +0.34421    +3.14189
H    +0.54130    +0.96179    +4.47794
C    +1.47690    −1.73024    +4.80658
H    +0.35573    −1.95938    +2.97204
H    +2.10800    −2.07871    +2.77640
C    +0.28514    −1.28444    +5.61554
H    +1.61810    −2.79553    +5.02090
H    +2.34955    −1.19334    +5.18753
O    −0.85886    −1.83909    +5.17860
H    −1.57049    −1.54040    +5.75613
O    +0.31728    −0.54349    +6.55801
19
 * E =   +5.134  kcal/mol  ;  (181)  179_036_198_183_082_235_180
H    +0.00000    +0.00000    +0.00000
O    +0.00000    +0.00000    +0.96378
```

```
C    +1.27806    +0.00000    +1.37645
C    +1.39920    +0.03064    +2.88853
N    +0.34409    −0.75761    +3.50874
H    +0.22859    −0.50862    +4.48380
O    +2.20122    +0.01972    +0.61569
C    +2.78037    −0.47222    +3.30063
H    +1.33052    +1.10571    +3.14626
H    −0.54303    −0.61171    +3.04435
C    +2.93038    −0.51956    +4.82308
H    +2.93823    −1.47667    +2.89398
H    +3.53885    +0.18122    +2.86363
C    +2.32922    −1.77780    +5.40578
H    +3.99218    −0.54744    +5.08620
H    +2.49296    +0.36287    +5.30129
O    +1.41427    −1.52594    +6.36292
H    +1.09003    −2.37578    +6.68314
O    +2.63643    −2.88878    +5.08855
19
* E =  +5.137  kcal/mol  ;  (182)  000_171_293_147_288_355_181
H    +0.00000    +0.00000    +0.00000
O    +0.00000    +0.00000    +0.96559
C    +1.25844    +0.00000    +1.43303
C    +2.33151    +0.00626    +0.34356
N    +3.67588    +0.21412    +0.82975
H    +3.77862    +1.14729    +1.21018
O    +1.49340    −0.01164    +2.60265
C    +2.26616    −1.30727    −0.46263
H    +2.08051    +0.84043    −0.32724
H    +3.85595    −0.42291    +1.59974
C    +2.65721    −1.08468    −1.91526
H    +2.96196    −2.01387    −0.00011
H    +1.27218    −1.76237    −0.40847
C    +1.63773    −0.32846    −2.73354
H    +3.59730    −0.52491    −1.97676
H    +2.82836    −2.02825    −2.44341
O    +0.48828    −0.05337    −2.05808
H    −0.09687    +0.40614    −2.67260
O    +1.77873    +0.00678    −3.87020
19
* E =  +5.152  kcal/mol  ;  (183)  181_313_067_180_292_172_179
```

H	+0.00000	+0.00000	+0.00000
O	+0.00000	+0.00000	+0.96347
C	+1.28043	+0.00000	+1.37837
C	+1.36947	−0.02488	+2.89155
N	+0.44966	+0.96031	+3.43596
H	−0.51579	+0.70272	+3.26843
O	+2.20487	−0.03044	+0.62097
C	+1.13331	−1.48262	+3.34115
H	+2.39247	+0.26566	+3.14459
H	+0.58844	+1.09932	+4.42729
C	+1.20270	−1.65741	+4.85000
H	+0.14507	−1.79949	+2.98718
H	+1.88217	−2.12437	+2.86409
C	+2.59059	−1.44478	+5.39879
H	+0.51635	−0.98389	+5.37530
H	+0.89694	−2.66813	+5.14314
O	+2.60236	−1.44267	+6.74142
H	+3.51462	−1.32360	+7.02937
O	+3.58386	−1.30313	+4.74426

19

∗ E = +5.161 kcal/mol ; (184) 359_170_293_140_290_180_000

H	+0.00000	+0.00000	+0.00000
O	+0.00000	+0.00000	+0.97092
C	+1.24523	+0.00000	+1.45112
C	+2.33751	−0.01610	+0.37879
N	+3.67535	+0.20145	+0.88356
H	+3.76830	+1.14531	+1.24019
O	+1.47629	−0.00032	+2.62403
C	+2.28025	−1.34851	−0.39888
H	+2.09756	+0.80362	−0.31175
H	+3.83186	−0.40903	+1.68032
C	+2.52826	−1.12837	−1.88158
H	+3.04273	−2.01381	+0.01523
H	+1.31109	−1.84092	−0.27076
C	+1.35531	−0.40766	−2.51444
H	+3.43622	−0.52763	−2.03052
H	+2.67823	−2.07313	−2.41826
O	+1.45184	−0.15380	−3.82209
H	+2.28367	−0.47449	−4.17895
O	+0.37459	−0.06601	−1.91178

19
∗ E = +5.171 kcal/mol ; (185) 177_147_043_062_171_305_180

H	+0.00000	+0.00000	+0.00000
O	+0.00000	+0.00000	+0.96467
C	+1.27284	+0.00000	+1.38057
C	+1.39509	+0.06829	+2.88988
N	+2.57258	−0.68785	+3.26520
H	+3.33131	−0.48277	+2.62381
O	+2.20650	−0.00915	+0.63040
C	+1.43852	+1.54807	+3.31903
H	+0.49883	−0.39561	+3.32077
H	+2.87081	−0.45931	+4.20460
C	+0.17188	+2.33003	+2.96282
H	+1.58431	+1.57034	+4.40422
H	+2.31892	+2.01606	+2.86158
C	+0.15395	+3.70611	+3.57500
H	+0.05594	+2.45445	+1.88531
H	−0.70772	+1.78767	+3.33243
O	+0.32250	+3.66712	+4.90980
H	+0.29308	+4.57355	+5.23579
O	+0.00341	+4.73360	+2.97918

19
∗ E = +5.174 kcal/mol ; (186) 180_050_304_293_137_298_358

H	+0.00000	+0.00000	+0.00000
O	+0.00000	+0.00000	+0.96465
C	+1.26172	+0.00000	+1.39354
C	+1.35748	+0.01015	+2.91785
N	+0.52899	−0.99639	+3.55927
H	−0.44134	−0.91192	+3.28387
O	+2.19540	−0.00242	+0.63048
C	+2.81349	−0.11678	+3.34404
H	+0.97930	+0.99417	+3.22717
H	+0.83830	−1.93321	+3.32764
C	+3.43102	−1.48640	+2.98520
H	+3.38351	+0.69041	+2.87325
H	+2.86583	+0.03971	+4.42342
C	+4.83500	−1.35021	+2.42129
H	+3.48265	−2.14505	+3.85217
H	+2.82941	−1.98984	+2.21586
O	+4.91161	−0.65089	+1.28262

H +4.03344 −0.36846 +0.98462
O +5.80807 −1.81563 +2.93452
19
∗ E = +5.250 kcal/mol ; (187) 181_054_182_071_174_038_178
H +0.00000 +0.00000 +0.00000
O +0.00000 +0.00000 +0.96390
C +1.27575 +0.00000 +1.38221
C +1.35654 −0.01982 +2.89972
N +0.56147 −1.14825 +3.36937
H +0.55579 −1.17969 +4.38099
O +2.20331 +0.02663 +0.62646
C +2.79771 −0.15839 +3.37249
H +0.95866 +0.95797 +3.23190
H −0.39659 −1.07858 +3.04939
C +3.62242 +1.10279 +3.12328
H +2.78021 −0.38134 +4.44642
H +3.25559 −1.01812 +2.87612
C +5.00595 +1.04830 +3.71377
H +3.73878 +1.25626 +2.04417
H +3.13887 +1.99371 +3.53307
O +5.56003 −0.17217 +3.61287
H +6.44718 −0.12141 +3.98598
O +5.58363 +1.97316 +4.21117
19
∗ E = +5.260 kcal/mol ; (188) 181_315_067_182_302_095_183
H +0.00000 +0.00000 +0.00000
O +0.00000 +0.00000 +0.96362
C +1.27764 +0.00000 +1.37965
C +1.37049 −0.03484 +2.89225
N +0.42312 +0.90143 +3.46746
H −0.53389 +0.61818 +3.29382
O +2.20608 −0.02868 +0.62608
C +1.21090 −1.50811 +3.32510
H +2.38217 +0.30545 +3.13640
H +0.57208 +0.99986 +4.46347
C +1.23432 −1.68691 +4.84874
H +0.25709 −1.89006 +2.94235
H +2.01302 −2.10331 +2.87457
C +2.51709 −1.14184 +5.42073
H +0.38333 −1.17580 +5.30637

H	+1.18299	−2.74910	+5.09454
O	+2.37748	+0.12833	+5.85271
H	+3.23758	+0.43376	+6.16479
O	+3.56281	−1.72094	+5.46245

19

∗ E = +5.261 kcal/mol ; (189) 182_347_292_293_180_314_181

H	+0.00000	+0.00000	+0.00000
O	+0.00000	+0.00000	+0.96440
C	+1.27980	+0.00000	+1.37232
C	+1.43041	−0.03919	+2.89225
N	+0.23365	+0.23122	+3.66187
H	−0.05955	+1.19388	+3.55617
O	+2.20086	−0.00361	+0.60660
C	+2.03395	−1.40062	+3.26480
H	+2.17797	+0.72554	+3.12856
H	−0.53982	−0.34341	+3.34926
C	+1.07533	−2.55360	+2.96492
H	+2.96564	−1.54030	+2.71048
H	+2.26796	−1.37957	+4.33331
C	+1.64422	−3.90604	+3.31024
H	+0.13783	−2.45985	+3.51716
H	+0.83582	−2.57441	+1.89235
O	+2.90826	−4.05081	+2.87850
H	+3.20069	−4.93753	+3.11729
O	+1.05932	−4.77720	+3.88758

19

∗ E = +5.268 kcal/mol ; (190) 177_159_042_175_182_305_180

H	+0.00000	+0.00000	+0.00000
O	+0.00000	+0.00000	+0.96396
C	+1.27318	+0.00000	+1.38012
C	+1.37844	+0.08135	+2.88878
N	+2.68046	−0.41718	+3.27012
H	+3.39106	−0.07293	+2.63434
O	+2.20731	−0.00899	+0.63129
C	+1.06748	+1.53949	+3.28723
H	+0.59614	−0.56391	+3.31043
H	+2.92735	−0.17152	+4.21827
C	+1.02627	+1.71126	+4.80564
H	+1.83032	+2.19861	+2.85752
H	+0.09935	+1.82724	+2.86085

C +0.68710 +3.12129 +5.21319
H +0.28414 +1.05120 +5.26063
H +2.00659 +1.47919 +5.23975
O +1.52216 +4.01648 +4.65704
H +1.25739 +4.89353 +4.95640
O −0.20630 +3.44164 +5.94282
19
∗ E = +5.279 kcal/mol ; (191) 178_146_044_062_172_052_179
H +0.00000 +0.00000 +0.00000
O +0.00000 +0.00000 +0.96465
C +1.27344 +0.00000 +1.37977
C +1.39755 +0.05603 +2.88952
N +2.57065 −0.71167 +3.25678
H +3.33654 −0.48875 +2.63001
O +2.20596 +0.00119 +0.62815
C +1.45410 +1.53361 +3.32453
H +0.49999 −0.40635 +3.31826
H +2.85874 −0.50871 +4.20523
C +0.18966 +2.32439 +2.98008
H +1.60965 +1.54520 +4.41028
H +2.33318 +2.00304 +2.86944
C +0.19235 +3.69890 +3.59434
H +0.10504 +2.44964 +1.89445
H −0.71102 +1.81008 +3.32306
O +1.32426 +4.36956 +3.31450
H +1.26142 +5.23885 +3.72589
O −0.68735 +4.17256 +4.25437
19
∗ E = +5.281 kcal/mol ; (192) 178_171_283_179_291_173_358
H +0.00000 +0.00000 +0.00000
O +0.00000 +0.00000 +0.96396
C +1.27112 +0.00000 +1.38289
C +1.38111 +0.03761 +2.89947
N +2.73706 −0.18196 +3.36104
H +2.98179 −1.16222 +3.30212
O +2.20597 −0.00131 +0.63162
C +0.85131 +1.39515 +3.39388
H +0.72253 −0.74126 +3.30041
H +3.38532 +0.30910 +2.75289
C +0.90616 +1.49545 +4.90997

H +1.46343 +2.19346 +2.95688
H −0.17658 +1.52710 +3.04476
C −0.09202 +0.57660 +5.58461
H +1.91372 +1.24224 +5.26173
H +0.68951 +2.51848 +5.24111
O +0.00247 +0.51691 +6.92653
H +0.73293 +1.05354 +7.24184
O −0.92493 −0.05708 +5.01090
19
* E = +5.295 kcal/mol ; (193) 177_159_039_174_180_056_180
H +0.00000 +0.00000 +0.00000
O +0.00000 +0.00000 +0.96394
C +1.27308 +0.00000 +1.37977
C +1.37933 +0.07930 +2.88850
N +2.68089 −0.42169 +3.26974
H +3.38454 −0.10849 +2.61051
O +2.20693 −0.01094 +0.63034
C +1.06775 +1.53506 +3.29270
H +0.59400 −0.56471 +3.30767
H +2.94630 −0.13995 +4.20281
C +1.00146 +1.69451 +4.81183
H +1.84673 +2.18768 +2.87858
H +0.11060 +1.83867 +2.85640
C +0.70197 +3.11184 +5.22723
H +0.20554 +1.05300 +5.21013
H +1.93998 +1.41821 +5.29682
O −0.42969 +3.57004 +4.66478
H −0.56659 +4.47383 +4.97032
O +1.36707 +3.77678 +5.96813
19
* E = +5.298 kcal/mol ; (194) 180_051_178_287_280_158_353
H +0.00000 +0.00000 +0.00000
O +0.00000 +0.00000 +0.96375
C +1.27440 +0.00000 +1.38324
C +1.35902 −0.00033 +2.90159
N +0.52128 −1.08500 +3.39591
H +0.56592 −1.14165 +4.40696
O +2.20327 +0.01739 +0.62693
C +2.80468 −0.17559 +3.35787
H +1.01141 +1.00057 +3.21422

H −0.44225 −0.96810 +3.11022
C +3.30757 −1.59894 +3.14600
H +3.43144 +0.53001 +2.80806
H +2.87497 +0.06349 +4.42349
C +2.83612 −2.53834 +4.24001
H +3.00500 −1.97597 +2.16225
H +4.40394 −1.62045 +3.17022
O +2.83166 −3.84349 +3.92477
H +3.04101 −3.97029 +2.99723
O +2.53461 −2.18497 +5.34205
19
∗ E = +5.334 kcal/mol ; (195) 180_179_307_185_080_207_009
H +0.00000 +0.00000 +0.00000
O +0.00000 +0.00000 +0.96396
C +1.27680 +0.00000 +1.38058
C +1.37956 −0.01073 +2.89966
N +2.73961 −0.03562 +3.38520
H +3.25743 −0.79954 +2.96828
O +2.20700 −0.00438 +0.62776
C +0.61744 +1.18293 +3.49156
H +0.87481 −0.93058 +3.23138
H +3.22076 +0.82234 +3.13162
C +0.82004 +1.25638 +4.99997
H +0.98654 +2.11517 +3.04758
H −0.44185 +1.09278 +3.24224
C +2.15325 +1.89247 +5.35592
H +0.04677 +1.88324 +5.46002
H +0.74228 +0.25844 +5.44844
O +2.69045 +1.51962 +6.52770
H +2.20152 +0.78955 +6.91165
O +2.68419 +2.72674 +4.68540
19
∗ E = +5.343 kcal/mol ; (196) 352_029_082_093_297_082_188
H +0.00000 +0.00000 +0.00000
O +0.00000 +0.00000 +0.97020
C +1.27049 +0.00000 +1.36529
C +2.28424 −0.18874 +0.22818
N +1.70449 +0.34340 −1.00448
H +1.81647 +1.35011 −1.05779
O +1.59075 +0.11382 +2.51126

C	+3.66443	+0.36347	+0.62104
H	+2.35566	−1.27857	+0.10288
H	+2.14049	−0.05751	−1.82404
C	+4.61450	−0.67383	+1.23744
H	+4.14363	+0.75122	−0.28385
H	+3.54093	+1.20006	+1.31688
C	+4.10373	−1.24732	+2.53648
H	+4.76521	−1.49632	+0.52948
H	+5.57911	−0.20837	+1.44639
O	+3.22242	−2.24345	+2.32426
H	+2.84242	−2.48315	+3.17748
O	+4.43457	−0.89203	+3.62785

19

∗ E = +5.361 kcal/mol ; (197) 182_317_064_174_180_178_180

H	+0.00000	+0.00000	+0.00000
O	+0.00000	+0.00000	+0.96368
C	+1.27594	+0.00000	+1.38101
C	+1.37058	−0.05521	+2.89294
N	+0.39525	+0.85044	+3.47655
H	−0.55060	+0.55606	+3.26254
O	+2.20691	−0.01737	+0.62983
C	+1.24547	−1.53282	+3.30710
H	+2.37418	+0.30541	+3.14267
H	+0.49787	+0.91229	+4.48045
C	+1.48004	−1.72521	+4.79539
H	+0.24833	−1.90015	+3.03635
H	+1.96547	−2.13636	+2.74331
C	+1.35708	−3.16896	+5.20653
H	+2.47427	−1.37296	+5.09657
H	+0.76228	−1.15697	+5.39945
O	+1.61595	−3.33913	+6.51333
H	+1.51781	−4.27652	+6.71503
O	+1.06170	−4.07538	+4.48202

19

∗ E = +5.370 kcal/mol ; (198) 181_055_179_070_223_063_002

H	+0.00000	+0.00000	+0.00000
O	+0.00000	+0.00000	+0.96469
C	+1.26048	+0.00000	+1.39449
C	+1.34409	−0.03291	+2.91104
N	+0.57502	−1.18336	+3.36333

H	+0.60745	−1.25689	+4.37218
O	+2.19678	+0.02884	+0.63857
C	+2.79750	−0.14793	+3.34959
H	+0.93489	+0.93486	+3.25767
H	−0.39483	−1.11540	+3.08069
C	+3.60987	+1.13387	+3.07516
H	+2.82059	−0.37700	+4.41903
H	+3.23563	−1.01299	+2.84006
C	+4.98311	+0.83444	+2.49974
H	+3.08210	+1.76701	+2.35079
H	+3.75303	+1.71961	+3.98334
O	+4.96854	+0.22409	+1.30971
H	+4.06061	+0.10074	+0.99354
O	+6.00857	+1.10748	+3.04927

19

∗ E = +5.405 kcal/mol ; (199) 183_189_192_288_281_145_347

H	+0.00000	+0.00000	+0.00000
O	+0.00000	+0.00000	+0.96379
C	+1.27616	+0.00000	+1.37593
C	+1.39295	−0.06699	+2.88791
N	+2.78150	+0.14231	+3.23303
H	+2.96919	−0.07590	+4.20297
O	+2.20162	+0.02197	+0.61546
C	+0.51677	+0.99829	+3.56284
H	+0.99647	−1.06139	+3.15922
H	+3.40488	−0.37403	+2.62636
C	+1.11447	+2.38732	+3.36077
H	−0.49930	+0.94180	+3.16720
H	+0.47209	+0.79751	+4.63782
C	+2.26989	+2.62398	+4.31974
H	+1.43694	+2.52640	+2.32063
H	+0.36758	+3.15943	+3.57671
O	+3.29403	+3.35552	+3.85691
H	+3.23136	+3.46389	+2.90604
O	+2.25812	+2.24687	+5.45434

19

∗ E = +5.413 kcal/mol ; (200) 194_341_049_306_082_059_184

H	+0.00000	+0.00000	+0.00000
O	+0.00000	+0.00000	+0.96510
C	+1.26693	+0.00000	+1.38245

C +1.40890 −0.32879 +2.85666
N +0.16129 −0.13199 +3.56645
H −0.61884 −0.57011 +3.09361
O +2.20500 +0.13245 +0.64654
C +2.00707 −1.75388 +2.93107
H +2.15647 +0.36542 +3.25543
H +0.21677 −0.48717 +4.51183
C +1.20644 −2.79648 +2.13551
H +3.03880 −1.73329 +2.56663
H +2.03483 −2.04267 +3.98664
C +1.52830 −2.79132 +0.65780
H +1.44427 −3.79868 +2.50645
H +0.12852 −2.64430 +2.23925
O +2.82645 −3.03041 +0.43304
H +2.98545 −2.95235 −0.51425
O +0.73986 −2.61081 −0.22851
19
∗ E = +5.478 kcal/mol ; (201) 181_055_182_071_178_328_181
H +0.00000 +0.00000 +0.00000
O +0.00000 +0.00000 +0.96389
C +1.27633 +0.00000 +1.38197
C +1.35629 −0.02674 +2.89962
N +0.58649 −1.17716 +3.35775
H +0.58323 −1.22072 +4.36887
O +2.20417 +0.02713 +0.62736
C +2.79913 −0.13496 +3.37363
H +0.93594 +0.93919 +3.23945
H −0.37308 −1.12571 +3.03884
C +3.58749 +1.15170 +3.14615
H +2.79256 −0.37709 +4.44146
H +3.27723 −0.97377 +2.85785
C +5.00622 +1.10771 +3.65287
H +3.63310 +1.41452 +2.08779
H +3.10141 +1.98695 +3.66908
O +5.14443 +0.33538 +4.74678
H +6.06889 +0.37832 +5.01588
O +5.92788 +1.70443 +3.17531
19
∗ E = +5.536 kcal/mol ; (202) 182_291_299_051_274_298_179
H +0.00000 +0.00000 +0.00000

O	+0.00000	+0.00000	+0.96446
C	+1.28251	+0.00000	+1.37611
C	+1.38001	−0.05701	+2.89497
N	+0.90986	+1.17142	+3.51830
H	+1.46362	+1.96965	+3.23317
O	+2.20771	+0.07010	+0.62178
C	+0.59882	−1.23829	+3.46884
H	+2.44546	−0.17578	+3.11730
H	−0.05537	+1.35119	+3.26450
C	+0.93124	−2.57652	+2.79041
H	+0.82974	−1.28706	+4.53577
H	−0.47543	−1.03827	+3.38251
C	+0.11061	−2.83071	+1.54962
H	+1.98587	−2.63107	+2.50753
H	+0.71895	−3.39672	+3.48309
O	−1.20143	−2.89836	+1.83211
H	−1.67377	−3.05205	+1.00676
O	+0.53687	−2.96997	+0.43863

19

∗ E = +5.539 kcal/mol ; (203) 179_040_194_179_186_311_180

H	+0.00000	+0.00000	+0.00000
O	+0.00000	+0.00000	+0.96375
C	+1.27777	+0.00000	+1.37790
C	+1.38772	+0.02805	+2.89200
N	+0.37357	−0.83573	+3.48124
H	+0.27515	−0.65133	+4.47124
O	+2.20213	+0.01837	+0.61927
C	+2.78949	−0.39838	+3.31135
H	+1.24949	+1.09351	+3.16105
H	−0.52907	−0.68090	+3.04990
C	+2.94908	−0.35062	+4.83040
H	+2.97770	−1.41257	+2.94741
H	+3.51828	+0.25933	+2.82794
C	+4.36082	−0.61939	+5.28021
H	+2.66231	+0.62276	+5.23866
H	+2.31506	−1.11786	+5.29235
O	+4.87477	−1.71421	+4.69585
H	+5.77125	−1.83323	+5.02889
O	+4.97748	+0.03795	+6.06940

19

∗ E = +5.587 kcal/mol ; (204) 182_198_203_081_175_307_180

H	+0.00000	+0.00000	+0.00000
O	+0.00000	+0.00000	+0.96421
C	+1.27439	+0.00000	+1.37719
C	+1.40715	−0.04378	+2.88933
N	+2.75213	+0.38197	+3.21758
H	+3.04028	+0.04030	+4.12366
O	+2.19944	+0.00213	+0.61654
C	+0.38080	+0.83347	+3.62173
H	+1.20143	−1.10006	+3.15267
H	+3.41066	+0.07069	+2.51383
C	−0.99679	+0.18675	+3.77935
H	+0.79170	+1.04225	+4.61408
H	+0.29997	+1.79926	+3.11067
C	−1.93330	+1.02952	+4.60465
H	−1.47930	+0.00503	+2.81995
H	−0.88675	−0.77636	+4.29499
O	−1.39523	+1.37197	+5.79035
H	−2.04534	+1.90205	+6.26469
O	−3.03698	+1.36665	+4.28429

19

∗ E = +5.596 kcal/mol ; (205) 176_174_300_112_293_094_185

H	+0.00000	+0.00000	+0.00000
O	+0.00000	+0.00000	+0.96392
C	+1.27152	+0.00000	+1.39279
C	+1.34986	+0.08972	+2.91031
N	+2.70034	−0.03826	+3.41502
H	+3.09457	−0.93296	+3.14813
O	+2.21127	−0.04424	+0.65199
C	+0.71225	+1.42141	+3.38115
H	+0.74925	−0.74277	+3.30008
H	+3.29325	+0.66494	+2.98539
C	−0.59509	+1.24203	+4.16366
H	+1.44045	+1.91036	+4.03342
H	+0.52552	+2.09001	+2.53355
C	−1.68819	+0.71239	+3.27027
H	−0.43583	+0.55705	+5.00117
H	−0.92830	+2.20867	+4.54681
O	−1.78289	−0.62745	+3.32379
H	−2.43792	−0.90087	+2.67142

O −2.38541 +1.38193 +2.56389
19
∗ E = +5.633 kcal/mol ; (206) 183_086_072_057_053_263_176
H +0.00000 +0.00000 +0.00000
O +0.00000 +0.00000 +0.96396
C +1.26693 +0.00000 +1.37763
C +1.39419 −0.08424 +2.89232
N +1.36350 −1.50538 +3.21953
H +2.22941 −1.95112 +2.93704
O +2.20501 +0.02134 +0.62882
C +2.65318 +0.64268 +3.36923
H +0.49571 +0.37193 +3.32396
H +1.25132 −1.64386 +4.21555
C +2.72034 +2.11988 +2.95086
H +2.69350 +0.56766 +4.46130
H +3.52796 +0.12513 +2.96132
C +1.45366 +2.82108 +3.36320
H +3.55738 +2.60896 +3.45228
H +2.85060 +2.19594 +1.86908
O +0.57438 +2.90264 +2.34816
H −0.23485 +3.30609 +2.68342
O +1.20081 +3.21485 +4.46639
19
∗ E = +5.636 kcal/mol ; (207) 179_042_191_177_185_055_180
H +0.00000 +0.00000 +0.00000
O +0.00000 +0.00000 +0.96374
C +1.27751 +0.00000 +1.37851
C +1.38161 +0.02184 +2.89355
N +0.39512 −0.88534 +3.46309
H +0.31912 −0.75671 +4.46362
O +2.20252 +0.02065 +0.62090
C +2.79526 −0.35545 +3.31677
H +1.20475 +1.07953 +3.17234
H −0.51997 −0.72650 +3.06057
C +2.96225 −0.27958 +4.83471
H +3.00496 −1.37149 +2.96551
H +3.50600 +0.31429 +2.82621
C +4.38216 −0.52757 +5.27403
H +2.68044 +0.72215 +5.18662
H +2.34146 −1.01645 +5.34841

O +5.24699 +0.30753 +4.67287
H +6.13096 +0.09798 +4.99430
O +4.73295 −1.35368 +6.06679
19
* E = +5.706 kcal/mol ; (208) 176_260_307_077_300_078_182
H +0.00000 +0.00000 +0.00000
O +0.00000 +0.00000 +0.96414
C +1.27592 +0.00000 +1.38427
C +1.41041 +0.10885 +2.89976
N +1.72999 +1.49341 +3.24616
H +2.53685 +1.81294 +2.72215
O +2.20490 +0.00413 +0.62755
C +0.17732 −0.32827 +3.68750
H +2.27182 −0.51376 +3.16800
H +0.95268 +2.10498 +3.01947
C −0.02988 −1.84549 +3.80091
H +0.29834 +0.06984 +4.69911
H −0.71670 +0.13792 +3.25983
C −0.19525 −2.53267 +2.47047
H +0.83305 −2.29059 +4.30747
H −0.92675 −2.05015 +4.38720
O +0.99510 −2.69912 +1.85982
H +0.84209 −3.11105 +1.00169
O −1.22553 −2.89428 +1.98238
19
* E = +5.708 kcal/mol ; (209) 182_319_060_063_068_014_179
H +0.00000 +0.00000 +0.00000
O +0.00000 +0.00000 +0.96372
C +1.27655 +0.00000 +1.38029
C +1.38324 −0.05053 +2.88966
N +0.37435 +0.80528 +3.48854
H −0.55718 +0.50357 +3.22676
O +2.20469 −0.03150 +0.62399
C +1.30508 −1.52969 +3.32382
H +2.37360 +0.34253 +3.13867
H +0.44387 +0.77867 +4.49808
C +2.45419 −2.39539 +2.81603
H +1.28002 −1.54839 +4.41818
H +0.35188 −1.94271 +2.97182
C +3.81348 −2.08930 +3.40113

H	+2.26376	−3.45011	+3.04424
H	+2.56796	−2.33559	+1.72913
O	+3.75899	−1.30843	+4.49770
H	+4.66319	−1.18640	+4.80919
O	+4.84706	−2.51126	+2.97179

19

∗ E = +5.711 kcal/mol ; (210) 359_002_117_166_084_182_000

H	+0.00000	+0.00000	+0.00000
O	+0.00000	+0.00000	+0.97500
C	+1.27125	+0.00000	+1.34497
C	+2.27790	−0.03425	+0.17770
N	+1.58067	−0.00366	−1.10486
H	+1.80052	+0.83125	−1.63611
O	+1.62666	+0.01393	+2.48824
C	+3.28488	+1.10117	+0.37618
H	+2.80726	−0.98955	+0.29617
H	+1.77257	−0.81061	−1.67984
C	+4.52661	+0.98929	−0.50177
H	+2.78968	+2.06004	+0.18018
H	+3.57592	+1.09508	+1.42921
C	+4.35269	+1.54171	−1.90363
H	+5.36022	+1.54262	−0.05056
H	+4.86102	−0.05479	−0.57781
O	+5.41967	+1.40572	−2.70673
H	+6.14363	+0.96603	−2.25503
O	+3.36641	+2.08393	−2.30667

19

∗ E = +5.736 kcal/mol ; (211) 181_316_055_296_079_022_178

H	+0.00000	+0.00000	+0.00000
O	+0.00000	+0.00000	+0.96387
C	+1.27528	+0.00000	+1.39342
C	+1.34385	−0.03583	+2.90825
N	+0.37122	+0.89724	+3.45308
H	−0.56133	+0.71141	+3.10539
O	+2.21231	−0.00954	+0.65260
C	+1.22150	−1.48981	+3.41736
H	+2.34767	+0.31864	+3.16004
H	+0.35555	+0.85899	+4.46429
C	−0.11297	−2.17326	+3.12086
H	+2.04692	−2.08612	+3.01635

H	+1.35627	−1.44764	+4.50408
C	−0.30038	−2.70502	+1.71910
H	−0.23442	−3.04196	+3.77865
H	−0.96645	−1.52041	+3.32620
O	+0.86545	−2.92796	+1.08946
H	+0.66994	−3.29338	+0.21931
O	−1.35947	−2.94728	+1.21622

19
∗ E = +5.798 kcal/mol ; (212) 184_327_057_060_278_343_181

H	+0.00000	+0.00000	+0.00000
O	+0.00000	+0.00000	+0.96357
C	+1.27236	+0.00000	+1.37654
C	+1.40149	−0.08725	+2.88242
N	+0.30068	+0.60122	+3.52993
H	−0.58973	+0.22760	+3.22278
O	+2.20516	+0.00520	+0.62337
C	+1.55538	−1.56920	+3.28498
H	+2.33690	+0.43145	+3.12262
H	+0.35681	+0.49896	+4.53544
C	+2.77057	−2.26852	+2.67791
H	+1.64717	−1.58504	+4.37659
H	+0.63430	−2.10670	+3.03368
C	+2.63054	−2.78446	+1.26416
H	+3.65231	−1.62009	+2.68150
H	+3.03415	−3.14750	+3.27725
O	+1.34963	−2.88279	+0.85800
H	+1.36378	−3.23624	−0.03870
O	+3.54616	−3.13052	+0.57796

19
∗ E = +5.798 kcal/mol ; (213) 182_318_060_062_179_058_180

H	+0.00000	+0.00000	+0.00000
O	+0.00000	+0.00000	+0.96377
C	+1.27482	+0.00000	+1.38238
C	+1.37326	−0.05122	+2.89372
N	+0.38627	+0.83731	+3.48137
H	−0.55099	+0.56250	+3.21055
O	+2.20607	−0.02732	+0.63012
C	+1.25224	−1.52312	+3.33341
H	+2.37514	+0.31588	+3.14053
H	+0.44245	+0.81448	+4.49181

C +2.38440 −2.40297 +2.79607
H +1.25852 −1.54028 +4.42986
H +0.28064 −1.91472 +3.00998
C +2.25856 −3.82636 +3.26769
H +2.37327 −2.40577 +1.69992
H +3.35943 −2.03151 +3.11818
O +1.08547 −4.37345 +2.89794
H +1.06735 −5.27932 +3.22619
O +3.07567 −4.43189 +3.89987
19
∗ E = +5.816 kcal/mol ; (214) 182_315_060_059_175_299_180
H +0.00000 +0.00000 +0.00000
O +0.00000 +0.00000 +0.96384
C +1.27491 +0.00000 +1.38410
C +1.36653 −0.05031 +2.89602
N +0.42158 +0.88921 +3.47410
H −0.52595 +0.66110 +3.19565
O +2.20762 −0.02505 +0.63458
C +1.17566 −1.50914 +3.34962
H +2.38457 +0.26962 +3.14274
H +0.46752 +0.86479 +4.48508
C +2.21228 −2.46262 +2.74735
H +1.24587 −1.53274 +4.44238
H +0.16155 −1.83075 +3.08029
C +2.07533 −3.85477 +3.30300
H +2.12167 −2.52838 +1.66160
H +3.22016 −2.09810 +2.97952
O +2.25532 −3.88034 +4.63724
H +2.15266 −4.79267 +4.93016
O +1.83002 −4.84206 +2.67184
19
∗ E = +5.835 kcal/mol ; (215) 182_058_183_086_297_080_184
H +0.00000 +0.00000 +0.00000
O +0.00000 +0.00000 +0.96370
C +1.27670 +0.00000 +1.38055
C +1.35659 −0.05497 +2.89767
N +0.65780 −1.26916 +3.31321
H +0.63165 −1.33247 +4.32309
O +2.20445 +0.02910 +0.62647
C +2.80831 −0.09830 +3.36855

H +0.87634 +0.87116 +3.26100
H −0.29694 −1.26888 +2.97509
C +3.47069 +1.27703 +3.52716
H +2.82911 −0.58983 +4.34685
H +3.38841 −0.72243 +2.68265
C +3.57535 +2.05130 +2.23764
H +2.89699 +1.87840 +4.24109
H +4.48542 +1.15266 +3.90823
O +2.40947 +2.65341 +1.92136
H +2.52575 +3.07012 +1.05974
O +4.55553 +2.15597 +1.56210
19
∗ E = +5.865 kcal/mol ; (216) 167_158_266_298_083_194_006
H +0.00000 +0.00000 +0.00000
O +0.00000 +0.00000 +0.96478
C +1.25996 +0.00000 +1.38884
C +1.38616 +0.30674 +2.87094
N +2.63742 −0.19487 +3.41729
H +2.51297 −1.11400 +3.82007
O +2.20998 −0.16236 +0.67027
C +1.29331 +1.83718 +3.04361
H +0.53639 −0.14243 +3.39306
H +3.31303 −0.29533 +2.66566
C +2.41865 +2.57402 +2.32478
H +0.32970 +2.19792 +2.67019
H +1.35311 +2.03569 +4.11618
C +2.13046 +2.80188 +0.85054
H +2.56751 +3.56761 +2.76506
H +3.36928 +2.03886 +2.44570
O +3.18590 +3.16464 +0.10461
H +3.99694 +3.12960 +0.61590
O +1.04379 +2.72014 +0.36117
19
∗ E = +6.006 kcal/mol ; (217) 182_334_050_298_187_053_179
H +0.00000 +0.00000 +0.00000
O +0.00000 +0.00000 +0.96420
C +1.27642 +0.00000 +1.37754
C +1.41295 −0.05604 +2.88714
N +0.23978 +0.48759 +3.54275
H −0.61750 +0.09027 +3.18065

O	+2.20345	−0.02266	+0.62063
C	+1.78663	−1.50388	+3.27134
H	+2.27443	+0.57758	+3.12314
H	+0.27606	+0.33436	+4.54234
C	+0.70861	−2.51353	+2.87470
H	+2.73718	−1.76102	+2.79115
H	+1.94331	−1.53725	+4.35452
C	+0.99251	−3.90053	+3.38698
H	−0.25780	−2.20401	+3.29310
H	+0.59923	−2.58554	+1.79013
O	+1.20964	−3.91129	+4.71553
H	+1.37516	−4.82370	+4.97802
O	+1.01857	−4.89833	+2.72621

19

* E = +6.041 kcal/mol ; (218) 358_151_037_064_062_268_178

H	+0.00000	+0.00000	+0.00000
O	+0.00000	+0.00000	+0.96545
C	+1.23958	+0.00000	+1.46247
C	+2.38176	−0.04297	+0.44689
N	+3.51536	+0.64003	+1.03200
H	+3.54958	+0.44627	+2.02737
O	+1.43571	−0.01755	+2.64087
C	+2.67406	−1.51719	+0.09374
H	+2.06774	+0.49129	−0.46150
H	+4.38674	+0.35039	+0.60776
C	+1.53129	−2.27503	−0.60293
H	+3.55604	−1.53648	−0.55685
H	+2.93761	−2.04059	+1.01922
C	+1.20459	−1.63847	−1.92773
H	+1.84960	−3.29862	−0.80777
H	+0.63802	−2.30469	+0.02773
O	+0.22220	−0.70531	−1.82318
H	+0.08053	−0.31681	−2.69502
O	+1.74429	−1.86296	−2.96826

19

* E = +6.047 kcal/mol ; (219) 003_343_145_053_258_109_346

H	+0.00000	+0.00000	+0.00000
O	+0.00000	+0.00000	+0.97395
C	+1.26016	+0.00000	+1.35845
C	+2.28928	+0.07829	+0.22716

N	+1.65077	−0.25482	−1.04145
H	+2.03232	+0.28372	−1.80823
O	+1.58733	−0.03745	+2.51694
C	+2.88953	+1.49083	+0.14284
H	+3.08994	−0.61900	+0.50569
H	+1.73303	−1.23627	−1.27235
C	+3.46419	+2.02706	+1.46638
H	+3.68156	+1.44711	−0.61188
H	+2.13146	+2.18615	−0.23933
C	+2.52049	+3.02518	+2.12971
H	+3.72395	+1.20449	+2.13799
H	+4.37237	+2.59535	+1.25540
O	+1.88633	+2.63597	+3.23499
H	+1.94811	+1.67917	+3.35647
O	+2.36343	+4.12163	+1.67647

19

∗ E = +6.052 kcal/mol ; (220) 179_155_294_062_064_270_001

H	+0.00000	+0.00000	+0.00000
O	+0.00000	+0.00000	+0.96517
C	+1.28654	+0.00000	+1.39257
C	+1.40733	+0.02687	+2.91177
N	+2.65583	−0.53905	+3.37163
H	+2.70739	−1.52990	+3.16990
O	+2.20316	−0.00081	+0.62745
C	+1.31424	+1.48106	+3.40770
H	+0.57468	−0.55224	+3.33035
H	+3.43282	−0.09886	+2.88963
C	+0.00032	+2.21515	+3.11076
H	+1.47694	+1.44949	+4.48879
H	+2.14889	+2.04265	+2.97103
C	−1.16561	+1.55157	+3.82514
H	+0.07084	+3.23192	+3.50070
H	−0.18677	+2.27792	+2.03370
O	−1.80060	+0.58263	+3.14537
H	−1.39984	+0.44138	+2.28012
O	−1.49293	+1.83300	+4.93787

19

∗ E = +6.087 kcal/mol ; (221) 356_021_086_181_311_283_357

H	+0.00000	+0.00000	+0.00000
O	+0.00000	+0.00000	+0.97188

C	+1.26944	+0.00000	+1.36145
C	+2.28026	−0.10451	+0.21288
N	+1.66677	+0.30719	−1.04337
H	+1.72232	+1.31118	−1.17587
O	+1.60107	+0.04932	+2.50967
C	+3.57612	+0.61655	+0.59353
H	+2.48840	−1.18076	+0.12087
H	+2.11963	−0.14232	−1.83156
C	+4.61588	+0.55480	−0.53602
H	+3.35953	+1.66518	+0.82759
H	+3.95810	+0.17215	+1.51829
C	+4.76174	−0.87043	−1.02399
H	+4.29245	+1.15165	−1.39131
H	+5.57692	+0.95106	−0.19181
O	+5.47658	−1.69256	−0.23893
H	+5.84117	−1.22639	+0.51715
O	+4.22413	−1.29115	−2.00628

19

∗ E = +6.144 kcal/mol ; (222) 182_335_052_299_187_305_180

H	+0.00000	+0.00000	+0.00000
O	+0.00000	+0.00000	+0.96416
C	+1.27636	+0.00000	+1.37734
C	+1.41241	−0.05173	+2.88725
N	+0.23715	+0.49143	+3.53994
H	−0.61690	+0.07108	+3.19625
O	+2.20291	−0.02907	+0.61976
C	+1.79157	−1.49860	+3.26910
H	+2.27138	+0.58530	+3.12315
H	+0.28407	+0.35994	+4.54213
C	+0.72731	−2.51607	+2.85186
H	+2.74983	−1.75357	+2.80641
H	+1.93294	−1.53029	+4.35617
C	+1.02497	−3.89516	+3.37767
H	−0.26391	−2.23800	+3.21666
H	+0.68323	−2.57993	+1.75727
O	+2.25001	−4.31399	+3.01689
H	+2.37930	−5.19762	+3.37930
O	+0.28621	−4.56461	+4.04151

19

∗ E = +6.158 kcal/mol ; (223) 172_180_206_307_090_247_005

H	+0.00000	+0.00000	+0.00000
O	+0.00000	+0.00000	+0.96521
C	+1.25779	+0.00000	+1.40021
C	+1.33485	+0.20614	+2.89733
N	+2.72826	+0.18659	+3.29460
H	+2.84034	−0.09629	+4.25762
O	+2.21178	−0.08838	+0.67381
C	+0.70583	+1.57325	+3.23660
H	+0.72221	−0.58357	+3.35859
H	+3.27718	−0.41892	+2.69752
C	+1.32176	+2.70497	+2.39929
H	−0.37765	+1.54815	+3.08618
H	+0.89323	+1.75834	+4.29799
C	+0.56201	+2.91325	+1.09699
H	+1.24180	+3.65064	+2.94052
H	+2.38637	+2.49748	+2.23936
O	+1.20903	+2.61806	−0.04640
H	+2.06933	+2.22392	+0.12583
O	−0.56161	+3.31456	+1.06444

19

∗ E = +6.160 kcal/mol ; (224) 359_172_306_183_081_203_182

H	+0.00000	+0.00000	+0.00000
O	+0.00000	+0.00000	+0.95993
C	+1.25645	+0.00000	+1.44716
C	+2.37153	−0.03537	+0.39728
N	+3.68866	+0.15452	+0.95468
H	+3.72453	+1.00489	+1.50382
O	+1.45335	+0.02433	+2.62168
C	+2.31931	−1.36024	−0.38031
H	+2.17815	+0.78656	−0.31117
H	+3.91045	−0.61165	+1.58392
C	+3.46681	−1.45615	−1.37836
H	+2.38862	−2.19676	+0.32551
H	+1.36211	−1.45800	−0.90277
C	+4.75227	−1.89177	−0.71886
H	+3.24889	−2.20503	−2.14897
H	+3.64112	−0.50633	−1.89242
O	+5.82917	−1.50986	−1.41443
H	+6.60938	−1.84333	−0.95723
O	+4.82581	−2.55255	+0.28005

19
∗ E = +6.188 kcal/mol ; (225) 181_032_289_280_053_097_356

H	+0.00000	+0.00000	+0.00000
O	+0.00000	+0.00000	+0.96388
C	+1.27052	+0.00000	+1.37433
C	+1.39561	−0.03348	+2.89922
N	+0.34004	−0.74865	+3.59215
H	−0.53035	−0.23548	+3.54088
O	+2.20193	+0.06004	+0.61901
C	+2.78232	−0.55116	+3.29247
H	+1.34356	+1.02146	+3.20205
H	+0.18164	−1.64398	+3.13828
C	+2.90113	−2.08215	+3.21201
H	+3.53079	−0.07219	+2.65497
H	+2.97697	−0.25813	+4.32722
C	+2.47269	−2.62403	+1.86330
H	+3.92455	−2.38732	+3.45258
H	+2.23353	−2.52753	+3.95440
O	+3.43197	−2.87372	+0.96587
H	+4.29248	−2.60466	+1.29281
O	+1.32189	−2.81581	+1.58418

19
∗ E = +6.319 kcal/mol ; (226) 178_031_308_183_081_203_007

H	+0.00000	+0.00000	+0.00000
O	+0.00000	+0.00000	+0.96390
C	+1.27789	+0.00000	+1.37548
C	+1.39822	+0.05170	+2.89552
N	+0.32416	−0.58307	+3.63437
H	−0.58107	−0.24317	+3.33675
O	+2.20150	−0.00356	+0.61279
C	+2.75660	−0.50517	+3.32034
H	+1.37269	+1.12585	+3.14070
H	+0.34336	−1.59052	+3.51283
C	+2.88881	−0.52850	+4.83709
H	+2.86989	−1.52652	+2.94012
H	+3.54599	+0.09478	+2.86371
C	+2.19079	−1.73008	+5.45060
H	+3.94323	−0.60148	+5.13023
H	+2.49284	+0.39587	+5.27626
O	+1.83580	−1.60385	+6.73950

H +1.98766 −0.70944 +7.05050
O +2.00655 −2.75979 +4.87323
19
∗ E = +6.321 kcal/mol ; (227) 189_334_300_049_278_164_351
H +0.00000 +0.00000 +0.00000
O +0.00000 +0.00000 +0.96447
C +1.26596 +0.00000 +1.38049
C +1.40960 −0.21703 +2.88104
N +0.28453 +0.18967 +3.69568
H +0.09087 +1.17805 +3.59381
O +2.20488 +0.11869 +0.63816
C +1.70203 −1.70425 +3.14204
H +2.29338 +0.36284 +3.17274
H −0.55117 −0.30973 +3.41210
C +2.82144 −2.27848 +2.28159
H +1.94298 −1.79539 +4.20382
H +0.78764 −2.28020 +2.95776
C +2.33699 −2.66566 +0.89350
H +3.65509 −1.57117 +2.19319
H +3.21943 −3.19293 +2.73769
O +3.29302 −2.85657 −0.02621
H +4.14868 −2.57411 +0.30319
O +1.18714 −2.84272 +0.62000
19
∗ E = +6.332 kcal/mol ; (228) 182_318_064_175_181_308_181
H +0.00000 +0.00000 +0.00000
O +0.00000 +0.00000 +0.96370
C +1.27586 +0.00000 +1.38064
C +1.37354 −0.05043 +2.89274
N +0.37562 +0.82366 +3.48139
H −0.56338 +0.50403 +3.27411
O +2.20686 −0.02437 +0.62959
C +1.29260 −1.53395 +3.30317
H +2.36753 +0.33527 +3.14257
H +0.48453 +0.89334 +4.48403
C +1.50509 −1.71164 +4.80520
H +0.31284 −1.93251 +3.01510
H +2.05285 −2.09865 +2.75036
C +1.46274 −3.15487 +5.23399
H +2.46813 −1.30533 +5.12385

H +0.71326 −1.18963 +5.35677
O +0.35560 −3.77716 +4.79128
H +0.38461 −4.68829 +5.10427
O +2.29092 −3.71051 +5.89607
19
∗ E = +6.365 kcal/mol ; (229) 178_243_190_084_301_078_181
H +0.00000 +0.00000 +0.00000
O +0.00000 +0.00000 +0.96418
C +1.26915 +0.00000 +1.38408
C +1.42790 +0.04647 +2.89901
N +2.10850 +1.31169 +3.16450
H +2.40312 +1.35966 +4.13182
O +2.20351 +0.00252 +0.63378
C +0.11634 −0.01003 +3.68157
H +2.04054 −0.83692 +3.14800
H +2.93420 +1.38868 +2.58106
C −0.43501 −1.42467 +3.91495
H +0.30244 +0.43157 +4.66655
H −0.63132 +0.62031 +3.19337
C −0.71896 −2.19231 +2.64917
H +0.29039 −2.00221 +4.49872
H −1.36969 −1.36803 +4.47461
O +0.41552 −2.67466 +2.10053
H +0.17900 −3.13499 +1.28724
O −1.79574 −2.38232 +2.16504
19
∗ E = +6.391 kcal/mol ; (230) 182_316_064_174_180_059_180
H +0.00000 +0.00000 +0.00000
O +0.00000 +0.00000 +0.96370
C +1.27590 +0.00000 +1.38182
C +1.36834 −0.04324 +2.89446
N +0.39933 +0.87230 +3.46965
H −0.54841 +0.58689 +3.25192
O +2.20728 −0.02491 +0.63165
C +1.23658 −1.51856 +3.31951
H +2.37484 +0.30926 +3.14395
H +0.49634 +0.93387 +4.47421
C +1.45568 −1.69273 +4.82257
H +0.23644 −1.87572 +3.04159
H +1.96756 −2.12255 +2.77103

C	+1.33599	−3.13305	+5.24768
H	+2.46131	−1.34334	+5.08738
H	+0.72484	−1.12777	+5.40437
O	+2.22934	−3.91509	+4.61790
H	+2.10210	−4.81916	+4.92670
O	+0.55100	−3.56334	+6.04300

19

∗ E = +6.391 kcal/mol ; (231) 183_192_207_301_189_180_359

H	+0.00000	+0.00000	+0.00000
O	+0.00000	+0.00000	+0.96396
C	+1.27437	+0.00000	+1.37885
C	+1.38480	−0.07056	+2.88990
N	+2.75787	+0.21293	+3.26001
H	+3.00335	−0.22046	+4.13896
O	+2.20363	+0.02941	+0.62289
C	+0.41976	+0.90682	+3.57133
H	+1.06315	−1.09530	+3.14422
H	+3.39784	−0.10134	+2.54055
C	+0.68193	+2.34054	+3.14041
H	−0.61581	+0.62916	+3.35904
H	+0.55175	+0.82815	+4.65498
C	−0.11633	+3.33430	+3.95423
H	+1.75115	+2.56754	+3.24318
H	+0.42632	+2.48476	+2.08096
O	+0.06571	+4.62705	+3.61670
H	+0.70674	+4.70804	+2.90716
O	−0.86195	+3.04105	+4.83740

19

∗ E = +6.417 kcal/mol ; (232) 358_012_098_125_298_345_005

H	+0.00000	+0.00000	+0.00000
O	+0.00000	+0.00000	+0.97373
C	+1.26001	+0.00000	+1.35483
C	+2.29512	−0.05607	+0.22335
N	+1.64858	+0.18415	−1.06228
H	+1.74447	+1.14806	−1.35776
O	+1.59904	+0.00728	+2.50919
C	+3.50220	+0.84484	+0.56635
H	+2.62270	−1.10597	+0.23290
H	+2.01497	−0.40573	−1.79599
C	+4.83713	+0.11424	+0.51490

H +3.52537 +1.68175 −0.13953
H +3.35927 +1.28730 +1.55775
C +5.02210 −1.04836 +1.48771
H +5.03047 −0.29590 −0.48145
H +5.65938 +0.80853 +0.71793
O +4.16953 −1.17600 +2.50427
H +3.42017 −0.55824 +2.52487
O +5.92681 −1.81834 +1.34863
19
* E = +6.422 kcal/mol ; (233) 001_154_286_057_059_104_002
H +0.00000 +0.00000 +0.00000
O +0.00000 +0.00000 +0.97329
C +1.23727 +0.00000 +1.46419
C +2.38430 +0.03037 +0.44127
N +3.59620 +0.60665 +0.98854
H +3.50341 +1.61130 +1.07590
O +1.44748 −0.05514 +2.64189
C +2.70665 −1.40721 −0.00206
H +2.07328 +0.61902 −0.42941
H +3.72682 +0.25067 +1.93143
C +1.53345 −2.20684 −0.59369
H +3.53219 −1.34439 −0.71807
H +3.08491 −1.94875 +0.87244
C +0.96629 −1.51132 −1.81127
H +1.86829 −3.21942 −0.83913
H +0.72520 −2.29782 +0.13897
O +1.27814 −2.00911 −3.00874
H +1.81548 −2.80015 −2.93115
O +0.28784 −0.52026 −1.74046
19
* E = +6.448 kcal/mol ; (234) 183_196_046_284_060_097_182
H +0.00000 +0.00000 +0.00000
O +0.00000 +0.00000 +0.96373
C +1.28965 +0.00000 +1.35899
C +1.41873 −0.06730 +2.87473
N +2.76551 +0.32239 +3.23038
H +3.04366 +1.15897 +2.72909
O +2.19797 +0.00379 +0.58665
C +0.28225 +0.64670 +3.63472
H +1.29647 −1.14085 +3.09251

H	+2.86119	+0.47590	+4.22520
C	+0.37812	+2.17869	+3.65295
H	−0.68425	+0.33275	+3.23011
H	+0.31783	+0.30416	+4.67477
C	+0.34308	+2.74559	+2.26118
H	−0.44143	+2.59246	+4.24351
H	+1.32827	+2.48482	+4.09934
O	−0.87404	+3.16376	+1.89206
H	−0.81673	+3.47343	+0.98074
O	+1.29493	+2.79733	+1.53031

19

∗ E = +6.468 kcal/mol ; (235) 178_169_294_174_175_181_001

H	+0.00000	+0.00000	+0.00000
O	+0.00000	+0.00000	+0.96404
C	+1.27250	+0.00000	+1.38519
C	+1.37261	+0.04718	+2.90408
N	+2.71531	−0.19600	+3.38763
H	+3.00584	−1.14442	+3.18338
O	+2.20898	−0.02008	+0.63895
C	+0.86844	+1.40963	+3.39568
H	+0.70503	−0.73262	+3.29448
H	+3.36458	+0.40893	+2.89383
C	+0.80637	+1.45712	+4.91286
H	+1.54392	+2.19565	+3.03647
H	−0.11505	+1.62596	+2.97000
C	+0.41682	+2.82820	+5.42005
H	+0.07756	+0.72881	+5.29453
H	+1.77802	+1.17582	+5.33715
O	+0.36272	+2.94808	+6.76195
H	+0.60698	+2.12471	+7.18995
O	+0.16609	+3.76046	+4.72038

19

∗ E = +6.524 kcal/mol ; (236) 005_340_149_058_171_175_358

H	+0.00000	+0.00000	+0.00000
O	+0.00000	+0.00000	+0.97246
C	+1.26973	+0.00000	+1.35961
C	+2.28696	+0.11840	+0.21853
N	+1.65993	−0.25480	−1.04525
H	+2.06411	+0.24651	−1.82577
O	+1.60121	−0.04299	+2.50970

C	+2.77473	+1.56835	+0.13068
H	+3.13348	−0.52421	+0.49469
H	+1.73620	−1.24588	−1.23463
C	+3.41636	+2.07988	+1.41082
H	+3.50530	+1.64055	−0.68285
H	+1.93667	+2.21723	−0.15139
C	+4.06159	+3.43291	+1.19698
H	+2.68682	+2.14992	+2.22535
H	+4.18767	+1.38321	+1.76538
O	+4.55883	+4.00942	+2.30635
H	+4.39295	+3.46775	+3.08142
O	+4.15156	+3.97669	+0.13889

19

∗ E = +6.533 kcal/mol ; (237) 177_157_032_293_275_169_357

H	+0.00000	+0.00000	+0.00000
O	+0.00000	+0.00000	+0.96401
C	+1.27171	+0.00000	+1.38101
C	+1.38319	+0.07370	+2.88820
N	+2.66489	−0.46444	+3.28269
H	+3.36750	−0.28291	+2.57546
O	+2.20420	+0.00504	+0.62503
C	+1.12489	+1.54239	+3.30261
H	+0.56915	−0.53319	+3.30317
H	+2.97310	−0.08743	+4.16948
C	+2.22317	+2.49023	+2.83030
H	+0.15316	+1.85605	+2.90875
H	+1.06942	+1.58548	+4.39479
C	+3.40053	+2.55580	+3.78671
H	+2.59028	+2.20991	+1.83303
H	+1.83870	+3.51407	+2.74486
O	+4.48924	+3.18162	+3.30823
H	+4.36177	+3.44941	+2.39535
O	+3.39549	+2.12642	+4.90106

19

∗ E = +6.548 kcal/mol ; (238) 175_162_300_058_277_165_356

H	+0.00000	+0.00000	+0.00000
O	+0.00000	+0.00000	+0.96500
C	+1.28217	+0.00000	+1.37961
C	+1.39175	+0.13225	+2.89484
N	+2.68714	−0.27361	+3.39546

H +2.87491 −1.24067 +3.15872
O +2.20732 −0.11175 +0.63230
C +1.11585 +1.57201 +3.35619
H +0.61975 −0.51930 +3.32619
H +3.40915 +0.27213 +2.93529
C −0.25043 +2.12434 +2.96897
H +1.22787 +1.56779 +4.44310
H +1.89178 +2.22910 +2.94667
C −0.28045 +2.66049 +1.54961
H −1.03291 +1.36408 +3.08696
H −0.51790 +2.96131 +3.62576
O −1.50565 +2.86356 +1.03261
H −2.18783 +2.57124 +1.64062
O +0.69441 +2.92235 +0.91376
19
* E = +6.581 kcal/mol ; (239) 181_060_296_175_290_175_358
H +0.00000 +0.00000 +0.00000
O +0.00000 +0.00000 +0.96365
C +1.27780 +0.00000 +1.38086
C +1.36495 −0.02475 +2.90005
N +0.71778 −1.20531 +3.46622
H −0.27612 −1.20176 +3.27356
O +2.20457 −0.00938 +0.62206
C +2.82052 +0.05486 +3.34330
H +0.82750 +0.85890 +3.26473
H +1.10471 −2.05337 +3.06602
C +2.94193 +0.15022 +4.85633
H +3.35227 −0.83166 +2.97929
H +3.29051 +0.92208 +2.87218
C +2.46462 +1.48679 +5.38853
H +2.35526 −0.64489 +5.33357
H +3.98371 +0.02135 +5.17416
O +2.45631 +1.59789 +6.73068
H +2.73526 +0.77750 +7.14302
O +2.12102 +2.40442 +4.70704
19
* E = +6.590 kcal/mol ; (240) 170_167_288_312_092_246_009
H +0.00000 +0.00000 +0.00000
O +0.00000 +0.00000 +0.96569
C +1.25471 +0.00000 +1.40593

C	+1.34818	+0.24219	+2.90287
N	+2.65959	−0.05275	+3.43993
H	+2.82481	−1.05177	+3.45561
O	+2.21197	−0.06984	+0.67705
C	+0.98288	+1.72294	+3.14074
H	+0.60171	−0.37912	+3.40727
H	+3.37796	+0.33924	+2.83988
C	+1.71760	+2.68940	+2.19252
H	−0.09439	+1.87401	+3.02392
H	+1.25014	+1.94033	+4.17687
C	+0.90066	+2.96039	+0.93351
H	+1.82650	+3.66061	+2.68079
H	+2.72486	+2.32751	+1.95484
O	+1.39661	+2.52498	−0.23710
H	+2.16535	+1.95961	−0.10819
O	−0.14317	+3.53817	+0.97049

19

∗ E = +6.644 kcal/mol ; (241) 000_183_195_289_278_156_178

H	+0.00000	+0.00000	+0.00000
O	+0.00000	+0.00000	+0.95985
C	+1.25306	+0.00000	+1.44795
C	+2.36664	−0.00704	+0.40208
N	+3.62824	−0.07072	+1.09847
H	+4.41082	+0.16560	+0.50395
O	+1.44615	+0.03395	+2.62355
C	+2.24420	−1.20820	−0.55322
H	+2.21937	+0.91875	−0.18606
H	+3.62291	+0.49123	+1.93932
C	+2.60396	−2.50820	+0.15635
H	+1.23757	−1.27088	−0.98099
H	+2.93276	−1.06229	−1.39180
C	+4.09875	−2.70791	+0.20582
H	+2.21373	−2.54189	+1.17882
H	+2.19074	−3.37181	−0.37721
O	+4.46672	−3.51331	+1.20727
H	+5.42369	−3.61860	+1.16566
O	+4.88281	−2.25602	−0.58397

19

∗ E = +6.662 kcal/mol ; (242) 001_188_213_303_185_180_180

H	+0.00000	+0.00000	+0.00000

O	+0.00000	+0.00000	+0.96035
C	+1.25103	+0.00000	+1.44772
C	+2.36604	+0.01787	+0.40390
N	+3.63039	−0.18404	+1.07954
H	+4.38484	+0.31717	+0.63339
O	+1.44579	−0.00100	+2.62439
C	+2.15329	−1.06610	−0.66496
H	+2.29026	+1.00472	−0.08578
H	+3.56231	+0.09693	+2.05068
C	+2.04606	−2.44942	−0.04426
H	+1.27603	−0.85961	−1.29253
H	+3.00465	−1.03944	−1.35221
C	+1.97003	−3.52544	−1.09064
H	+2.91118	−2.64094	+0.60013
H	+1.16241	−2.54478	+0.60052
O	+1.88265	−4.74985	−0.55143
H	+1.83595	−5.38850	−1.27170
O	+1.98099	−3.34034	−2.27586

19

∗ E = +6.697 kcal/mol ; (243) 353_158_026_294_271_178_179

H	+0.00000	+0.00000	+0.00000
O	+0.00000	+0.00000	+0.96026
C	+1.25400	+0.00000	+1.44008
C	+2.35706	−0.16300	+0.40188
N	+3.58768	+0.35612	+0.94461
H	+3.57845	+0.30169	+1.95670
O	+1.45743	+0.06077	+2.61504
C	+2.40425	−1.65352	−0.01127
H	+2.06682	+0.41732	−0.48908
H	+4.39655	−0.13070	+0.58140
C	+2.83841	−2.57534	+1.12513
H	+1.41916	−1.96003	−0.38159
H	+3.10660	−1.75281	−0.84532
C	+4.33227	−2.76339	+1.19651
H	+2.50121	−2.21152	+2.10416
H	+2.40543	−3.57529	+1.01590
O	+4.68335	−3.54331	+2.22555
H	+5.64281	−3.63585	+2.21780
O	+5.13808	−2.30279	+0.43408

19

∗ E = +6.732 kcal/mol ; (244) 356_018_092_165_061_066_005
H +0.00000 +0.00000 +0.00000
O +0.00000 +0.00000 +0.97281
C +1.26923 +0.00000 +1.35705
C +2.27987 −0.09954 +0.19960
N +1.62899 +0.24285 −1.06142
H +1.71491 +1.23005 −1.27755
O +1.60593 +0.04266 +2.50420
C +3.53886 +0.69668 +0.54667
H +2.54574 −1.16491 +0.15145
H +2.00605 −0.27546 −1.84401
C +4.72917 +0.35824 −0.35901
H +3.30022 +1.76956 +0.49942
H +3.80009 +0.48960 +1.58735
C +4.45417 +0.67318 −1.81379
H +5.61389 +0.91857 −0.03420
H +4.96471 −0.70645 −0.29601
O +4.30784 +1.98368 −2.10134
H +4.48688 +2.53037 −1.33241
O +4.32384 −0.14509 −2.67321
19
∗ E = +6.738 kcal/mol ; (245) 179_047_187_074_069_186_001
H +0.00000 +0.00000 +0.00000
O +0.00000 +0.00000 +0.96381
C +1.27389 +0.00000 +1.38145
C +1.37170 +0.01604 +2.89548
N +0.45252 −0.97521 +3.44006
H +0.42974 −0.89906 +4.44967
O +2.20007 +0.01963 +0.61962
C +2.79565 −0.28956 +3.35192
H +1.11562 +1.04811 +3.19691
H −0.48786 −0.82520 +3.09587
C +3.76356 +0.86210 +3.12135
H +2.76141 −0.51186 +4.42317
H +3.14699 −1.18658 +2.83490
C +3.47894 +2.03867 +4.03303
H +4.79461 +0.54140 +3.31565
H +3.73405 +1.19296 +2.07619
O +4.21720 +3.13948 +3.79775
H +4.80680 +3.00331 +3.05273

O +2.67470 +2.03106 +4.91587
19
* E = +6.738 kcal/mol ; (246) 182_067_079_063_224_061_002
H +0.00000 +0.00000 +0.00000
O +0.00000 +0.00000 +0.96460
C +1.25446 +0.00000 +1.38912
C +1.37180 −0.05161 +2.90933
N +0.88287 −1.35317 +3.33800
H +1.58756 −2.06832 +3.19951
O +2.19445 +0.02999 +0.63213
C +2.80378 +0.27491 +3.32691
H +0.67882 +0.70698 +3.29547
H +0.63485 −1.34594 +4.31798
C +3.24801 +1.70092 +2.94206
H +2.88665 +0.13575 +4.40825
H +3.46421 −0.47017 +2.86569
C +4.64691 +1.73286 +2.35015
H +2.56598 +2.12062 +2.19106
H +3.23956 +2.36961 +3.80283
O +4.79661 +1.02447 +1.22562
H +3.95888 +0.62221 +0.94834
O +5.55980 +2.33069 +2.83722
19
* E = +6.764 kcal/mol ; (247) 357_170_309_189_306_082_354
H +0.00000 +0.00000 +0.00000
O +0.00000 +0.00000 +0.96071
C +1.24587 +0.00000 +1.45805
C +2.37206 −0.07437 +0.42691
N +3.67929 +0.16229 +1.01664
H +3.66304 +1.01930 +1.56019
O +1.44075 +0.03687 +2.63374
C +2.34022 −1.44779 −0.26625
H +2.19585 +0.70834 −0.32672
H +3.88842 −0.57574 +1.68324
C +3.55614 −1.70104 −1.16829
H +2.29743 −2.23160 +0.49998
H +1.42505 −1.53919 −0.86135
C +3.74795 −0.58412 −2.18235
H +4.45837 −1.79965 −0.55697
H +3.41013 −2.62911 −1.72242

O +4.37800 +0.50019 −1.71853
H +4.51932 +0.42003 −0.75803
O +3.34859 −0.64435 −3.30779
19
* E = +6.817 kcal/mol ; (248) 178_150_046_064_062_267_003
H +0.00000 +0.00000 +0.00000
O +0.00000 +0.00000 +0.96511
C +1.28593 +0.00000 +1.38719
C +1.40813 +0.04327 +2.89586
N +2.63520 −0.64003 +3.24847
H +3.39444 −0.32934 +2.65234
O +2.20297 +0.01183 +0.62336
C +1.35597 +1.51786 +3.35641
H +0.55482 −0.49986 +3.32219
H +2.88451 −0.46675 +4.21354
C +0.03951 +2.25655 +3.07787
H +1.53564 +1.52471 +4.43740
H +2.18768 +2.05295 +2.88390
C −1.11039 +1.60073 +3.82450
H +0.11862 +3.27823 +3.45353
H −0.16583 +2.30491 +2.00362
O −1.79628 +0.66290 +3.15299
H −1.41772 +0.50895 +2.27993
O −1.38066 +1.86393 +4.95737
19
* E = +6.887 kcal/mol ; (249) 179_051_076_292_137_298_358
H +0.00000 +0.00000 +0.00000
O +0.00000 +0.00000 +0.96464
C +1.25628 +0.00000 +1.38274
C +1.38961 +0.03282 +2.90241
N +0.57186 −1.01528 +3.48802
H +1.00210 −1.92676 +3.38265
O +2.19004 +0.00588 +0.61662
C +2.86913 +0.03315 +3.28993
H +0.94079 +0.99122 +3.19706
H +0.42927 −0.85236 +4.47583
C +3.57775 −1.29878 +2.96282
H +3.36638 +0.86471 +2.77857
H +2.94571 +0.23700 +4.36165
C +4.95242 −1.07906 +2.35433

H +3.70591 −1.91988 +3.84943
H +2.99162 −1.87295 +2.23228
O +4.94588 −0.42971 +1.18549
H +4.03974 −0.22981 +0.90288
O +5.97063 −1.44246 +2.86332
19
∗ E = +6.890 kcal/mol ; (250) 358_169_281_294_176_176_179
H +0.00000 +0.00000 +0.00000
O +0.00000 +0.00000 +0.96039
C +1.25272 +0.00000 +1.44703
C +2.37592 −0.04266 +0.40479
N +3.67592 +0.22339 +0.98461
H +3.80292 +1.21672 +1.13279
O +1.44845 −0.01013 +2.62432
C +2.36153 −1.41700 −0.28451
H +2.16598 +0.72180 −0.35473
H +3.72186 −0.20093 +1.90659
C +2.70918 −2.53881 +0.68247
H +1.38588 −1.62276 −0.74037
H +3.08984 −1.39677 −1.09954
C +2.59703 −3.88859 +0.02669
H +3.73040 −2.43058 +1.06383
H +2.05398 −2.54203 +1.56409
O +2.99174 −4.87727 +0.84206
H +2.88644 −5.71129 +0.37064
O +2.20006 −4.08704 −1.08694
19
∗ E = +6.972 kcal/mol ; (251) 000_170_294_174_178_180_180
H +0.00000 +0.00000 +0.00000
O +0.00000 +0.00000 +0.96013
C +1.25337 +0.00000 +1.44838
C +2.37625 −0.00904 +0.40519
N +3.67893 +0.23241 +0.98500
H +3.73390 +1.17216 +1.35909
O +1.44981 −0.00496 +2.62418
C +2.37624 −1.35127 −0.33916
H +2.16547 +0.79395 −0.31799
H +3.80612 −0.38848 +1.77901
C +3.38126 −1.35101 −1.47872
H +2.62299 −2.15040 +0.37030

H	+1.37895	−1.59503	−0.72323
C	+3.42565	−2.67746	−2.18526
H	+3.14748	−0.58002	−2.22337
H	+4.38398	−1.11242	−1.10987
O	+4.30767	−2.67997	−3.19672
H	+4.28849	−3.55344	−3.60374
O	+2.76282	−3.63585	−1.90355

19

∗ E = +7.058 kcal/mol ; (252) 355_019_089_067_170_172_357

H	+0.00000	+0.00000	+0.00000
O	+0.00000	+0.00000	+0.97216
C	+1.26838	+0.00000	+1.36145
C	+2.28564	−0.11113	+0.21385
N	+1.63470	+0.26184	−1.04209
H	+1.71923	+1.25911	−1.20848
O	+1.58838	+0.05045	+2.51456
C	+3.57136	+0.66587	+0.50267
H	+2.52137	−1.18327	+0.15327
H	+2.05176	−0.21133	−1.83233
C	+4.41201	+0.10356	+1.64035
H	+4.17958	+0.67196	−0.40809
H	+3.32567	+1.71381	+0.71454
C	+5.77089	+0.76955	+1.69950
H	+3.89840	+0.21688	+2.59992
H	+4.57698	−0.97459	+1.50307
O	+6.51839	+0.44717	+2.77162
H	+6.03576	−0.13265	+3.36507
O	+6.19163	+1.51966	+0.87257

19

∗ E = +7.060 kcal/mol ; (253) 170_164_032_312_093_246_009

H	+0.00000	+0.00000	+0.00000
O	+0.00000	+0.00000	+0.96546
C	+1.25421	+0.00000	+1.40201
C	+1.34538	+0.23934	+2.89044
N	+2.66163	−0.15797	+3.33633
H	+3.35593	−0.00621	+2.61487
O	+2.21049	−0.06338	+0.67119
C	+0.99569	+1.73149	+3.12306
H	+0.57691	−0.36908	+3.38179
H	+2.93795	+0.31889	+4.18311

C	+1.75388	+2.68409	+2.18284
H	−0.08052	+1.89223	+3.00220
H	+1.24232	+1.95761	+4.16499
C	+0.94271	+2.97821	+0.92511
H	+1.89106	+3.65201	+2.67059
H	+2.74851	+2.28589	+1.94894
O	+1.42532	+2.53025	−0.24544
H	+2.17958	+1.94525	−0.11721
O	−0.08471	+3.58484	+0.96437

19

∗ E = +7.117 kcal/mol ; (254) 180_155_288_050_059_193_003

H	+0.00000	+0.00000	+0.00000
O	+0.00000	+0.00000	+0.96463
C	+1.27793	+0.00000	+1.38387
C	+1.39055	+0.00314	+2.90162
N	+2.64164	−0.55723	+3.36671
H	+2.66163	−1.56066	+3.23520
O	+2.20514	+0.02094	+0.62476
C	+1.27416	+1.44518	+3.42745
H	+0.55268	−0.57517	+3.30235
H	+3.41021	−0.17383	+2.82564
C	+0.08390	+2.24196	+2.90792
H	+1.22632	+1.37277	+4.51681
H	+2.19978	+1.97527	+3.17464
C	−1.24477	+1.58649	+3.23584
H	+0.07516	+3.23615	+3.37206
H	+0.14885	+2.40754	+1.82575
O	−2.31064	+2.08154	+2.58038
H	−2.04579	+2.75840	+1.95448
O	−1.38007	+0.71422	+4.03796

19

∗ E = +7.209 kcal/mol ; (255) 185_205_200_300_141_301_000

H	+0.00000	+0.00000	+0.00000
O	+0.00000	+0.00000	+0.96534
C	+1.28947	+0.00000	+1.37315
C	+1.44033	−0.11030	+2.87989
N	+2.71742	+0.47463	+3.23294
H	+3.02894	+0.16189	+4.14233
O	+2.19749	+0.04947	+0.59924
C	+0.29748	+0.57847	+3.62433

H +1.37762 −1.19669 +3.07869
H +3.42390 +0.24649 +2.54374
C +0.21137 +2.08251 +3.30034
H −0.64029 +0.06592 +3.38634
H +0.45331 +0.44025 +4.69825
C −1.21976 +2.56581 +3.17570
H +0.71084 +2.68745 +4.05522
H +0.71932 +2.29029 +2.34797
O −1.93806 +1.94735 +2.21822
H −1.40312 +1.28698 +1.76301
O −1.70779 +3.42241 +3.84700
19
* E = +7.234 kcal/mol ; (256) 178_042_188_297_193_186_001
H +0.00000 +0.00000 +0.00000
O +0.00000 +0.00000 +0.96388
C +1.27535 +0.00000 +1.38123
C +1.37767 +0.04541 +2.89576
N +0.38735 −0.84911 +3.48225
H +0.35711 −0.72680 +4.48682
O +2.20250 +0.00796 +0.62319
C +2.78258 −0.32112 +3.36214
H +1.19980 +1.10674 +3.14819
H −0.53787 −0.66742 +3.11423
C +3.17736 −1.74232 +2.98790
H +3.50266 +0.38700 +2.94340
H +2.83302 −0.21112 +4.45059
C +4.42940 −2.18711 +3.71010
H +2.36775 −2.43836 +3.24400
H +3.34108 −1.82445 +1.90645
O +4.91167 −3.38714 +3.32858
H +4.37519 −3.76159 +2.62659
O +4.97611 −1.56497 +4.56873
19
* E = +7.300 kcal/mol ; (257) 004_345_151_304_191_192_003
H +0.00000 +0.00000 +0.00000
O +0.00000 +0.00000 +0.97191
C +1.27431 +0.00000 +1.36080
C +2.29211 +0.08675 +0.21629
N +1.64150 −0.20514 −1.06074
H +2.10391 +0.26987 −1.82626

O	+1.60330	−0.00191	+2.50931
C	+2.86405	+1.50802	+0.21830
H	+3.10014	−0.61235	+0.46013
H	+1.64867	−1.19596	−1.26872
C	+1.78020	+2.57595	+0.11957
H	+3.43494	+1.65286	+1.13911
H	+3.56754	+1.62227	−0.61329
C	+2.36877	+3.94919	−0.13541
H	+1.08466	+2.35976	−0.70346
H	+1.17654	+2.60117	+1.03666
O	+1.51435	+4.97789	+0.01206
H	+0.65455	+4.67580	+0.31300
O	+3.49702	+4.14136	−0.46946

19

∗ E = +7.301 kcal/mol ; (258) 183_068_299_061_066_185_002

H	+0.00000	+0.00000	+0.00000
O	+0.00000	+0.00000	+0.96387
C	+1.27418	+0.00000	+1.38414
C	+1.36177	−0.08353	+2.90245
N	+0.89199	−1.38424	+3.36836
H	−0.07743	−1.53504	+3.11835
O	+2.20218	+0.01292	+0.62159
C	+2.78585	+0.14774	+3.39013
H	+0.69927	+0.68088	+3.32344
H	+1.44198	−2.13381	+2.96338
C	+3.35411	+1.51623	+3.03811
H	+2.77678	+0.01725	+4.47553
H	+3.43433	−0.62448	+2.96083
C	+2.63375	+2.65252	+3.73547
H	+4.40887	+1.57264	+3.33509
H	+3.33162	+1.68243	+1.95418
O	+3.02930	+3.88781	+3.37055
H	+3.70672	+3.84434	+2.69212
O	+1.78237	+2.51257	+4.55965

19

∗ E = +7.386 kcal/mol ; (259) 004_149_031_305_085_193_179

H	+0.00000	+0.00000	+0.00000
O	+0.00000	+0.00000	+0.95984
C	+1.24155	+0.00000	+1.44489
C	+2.38005	+0.08724	+0.42909

N	+3.47353	+0.79467	+1.06095
H	+3.47180	+0.62202	+2.06003
O	+1.44580	−0.08791	+2.62063
C	+2.74754	−1.33160	−0.06182
H	+2.02715	+0.66392	−0.43869
H	+4.37034	+0.53278	+0.67421
C	+3.08468	−2.28680	+1.07707
H	+1.92836	−1.75459	−0.65427
H	+3.60839	−1.22371	−0.73019
C	+1.84642	−2.92203	+1.66214
H	+3.72650	−3.10563	+0.73388
H	+3.62848	−1.78508	+1.88462
O	+2.09424	−3.53614	+2.82263
H	+1.26538	−3.90680	+3.14643
O	+0.76358	−2.91239	+1.14588

19

∗ E = +7.460 kcal/mol ; (260) 355_021_087_303_191_194_005

H	+0.00000	+0.00000	+0.00000
O	+0.00000	+0.00000	+0.97122
C	+1.26984	+0.00000	+1.36255
C	+2.28948	−0.11278	+0.21887
N	+1.66732	+0.31054	−1.03477
H	+1.73996	+1.31242	−1.17154
O	+1.59451	+0.05906	+2.51267
C	+3.60291	+0.57619	+0.58985
H	+2.48211	−1.18956	+0.12860
H	+2.09855	−0.13605	−1.83279
C	+3.42844	+2.04514	+0.95285
H	+4.04850	+0.05685	+1.44266
H	+4.30661	+0.48732	−0.24325
C	+4.76328	+2.75693	+1.06429
H	+2.85085	+2.58971	+0.19118
H	+2.87658	+2.13895	+1.89575
O	+4.72391	+3.96112	+1.66409
H	+3.84482	+4.15388	+1.99715
O	+5.79015	+2.32534	+0.63990

19

∗ E = +7.467 kcal/mol ; (261) 181_309_291_176_289_173_358

H	+0.00000	+0.00000	+0.00000
O	+0.00000	+0.00000	+0.96354

C	+1.28343	+0.00000	+1.37948
C	+1.37323	−0.01437	+2.89772
N	+0.56741	+1.01958	+3.53325
H	+0.94742	+1.94180	+3.36245
O	+2.20505	+0.00291	+0.61769
C	+0.93857	−1.39650	+3.40387
H	+2.42616	+0.14248	+3.14731
H	−0.37994	+1.00320	+3.17135
C	+1.08871	−1.51252	+4.91299
H	−0.10872	−1.55836	+3.12361
H	+1.54223	−2.16574	+2.91183
C	+2.54104	−1.57030	+5.34320
H	+0.60674	−0.65736	+5.40231
H	+0.59892	−2.42084	+5.28464
O	+2.74482	−1.52289	+6.67377
H	+1.91429	−1.42106	+7.14351
O	+3.46455	−1.66512	+4.59434

19

∗ E = +7.473 kcal/mol ; (262) 179_039_201_185_308_281_357

H	+0.00000	+0.00000	+0.00000
O	+0.00000	+0.00000	+0.96380
C	+1.27522	+0.00000	+1.37880
C	+1.39101	+0.03467	+2.89032
N	+0.37494	−0.79818	+3.51523
H	+0.24397	−0.50673	+4.47865
O	+2.20212	+0.01071	+0.62064
C	+2.79597	−0.41391	+3.29451
H	+1.27353	+1.10466	+3.15235
H	−0.51356	−0.69190	+3.04194
C	+2.94139	−0.50724	+4.82009
H	+2.99387	−1.39768	+2.85903
H	+3.52717	+0.27943	+2.86519
C	+2.50075	+0.78751	+5.46627
H	+2.29983	−1.30220	+5.20467
H	+3.97662	−0.74285	+5.08843
O	+3.38832	+1.79791	+5.43052
H	+4.21241	+1.51944	+5.02487
O	+1.41232	+0.96036	+5.93092

19

∗ E = +7.536 kcal/mol ; (263) 354_023_087_185_298_182_002

H	+0.00000	+0.00000	+0.00000
O	+0.00000	+0.00000	+0.97030
C	+1.27193	+0.00000	+1.36253
C	+2.28686	−0.13393	+0.22073
N	+1.68213	+0.30411	−1.03403
H	+1.77608	+1.30276	−1.17996
O	+1.59475	+0.07151	+2.51130
C	+3.59355	+0.55816	+0.62269
H	+2.47950	−1.20858	+0.12727
H	+2.06702	−0.17840	−1.83458
C	+4.64171	+0.59441	−0.48095
H	+3.37136	+1.58587	+0.93465
H	+3.98908	+0.04818	+1.50471
C	+5.09647	−0.77695	−0.93977
H	+4.26954	+1.13266	−1.36412
H	+5.52527	+1.14857	−0.14099
O	+6.05605	−0.77430	−1.88425
H	+6.32805	+0.11993	−2.10178
O	+4.65755	−1.80899	−0.53259

19

∗ E = +7.537 kcal/mol ; (264) 357_167_296_178_072_040_177

H	+0.00000	+0.00000	+0.00000
O	+0.00000	+0.00000	+0.96008
C	+1.25542	+0.00000	+1.44722
C	+2.37253	−0.07382	+0.40175
N	+3.67815	+0.22074	+0.93976
H	+3.73004	+1.17668	+1.26932
O	+1.45387	+0.03029	+2.62180
C	+2.38013	−1.47452	−0.22910
H	+2.15166	+0.66125	−0.38761
H	+3.85447	−0.37599	+1.74238
C	+3.45067	−1.59934	−1.30970
H	+2.55425	−2.21770	+0.55785
H	+1.39906	−1.69896	−0.66226
C	+4.87285	−1.65289	−0.80628
H	+3.29555	−2.52806	−1.87191
H	+3.39885	−0.77391	−2.02451
O	+4.99502	−2.40461	+0.30495
H	+5.93007	−2.42323	+0.53692
O	+5.81521	−1.16848	−1.36085

19
∗ E = +7.603 kcal/mol ; (265) 189_327_056_052_277_162_349

H	+0.00000	+0.00000	+0.00000
O	+0.00000	+0.00000	+0.96431
C	+1.26297	+0.00000	+1.38436
C	+1.39376	−0.22868	+2.87581
N	+0.26864	+0.34180	+3.59022
H	−0.60505	−0.02979	+3.23526
O	+2.20682	+0.09238	+0.64582
C	+1.60442	−1.74144	+3.11231
H	+2.30898	+0.29789	+3.17084
H	+0.32463	+0.12626	+4.57776
C	+2.73530	−2.34077	+2.28164
H	+1.80797	−1.87741	+4.17930
H	+0.67309	−2.26937	+2.87834
C	+2.28060	−2.69411	+0.87365
H	+3.59087	−1.65586	+2.23132
H	+3.09328	−3.27351	+2.73367
O	+3.25458	−2.85889	−0.03167
H	+4.09969	−2.56421	+0.31377
O	+1.13709	−2.87129	+0.57553

19
∗ E = +7.607 kcal/mol ; (266) 184_198_311_302_141_301_000

H	+0.00000	+0.00000	+0.00000
O	+0.00000	+0.00000	+0.96558
C	+1.29221	+0.00000	+1.37703
C	+1.43930	−0.10696	+2.89493
N	+2.74904	+0.30008	+3.35122
H	+3.47333	−0.15116	+2.80435
O	+2.19885	+0.06217	+0.60280
C	+0.33807	+0.67293	+3.61010
H	+1.30898	−1.17469	+3.12299
H	+2.87476	+1.29902	+3.22539
C	+0.33136	+2.16787	+3.22758
H	−0.62729	+0.21259	+3.38098
H	+0.49228	+0.56416	+4.68580
C	−1.07206	+2.72606	+3.07688
H	+0.85367	+2.78011	+3.96323
H	+0.83592	+2.31761	+2.26041
O	−1.82508	+2.10015	+2.15434

H −1.33468 +1.38288 +1.73531
O −1.50281 +3.64610 +3.70060
19
∗ E = +7.642 kcal/mol ; (267) 179_017_304_343_309_178_357
H +0.00000 +0.00000 +0.00000
O +0.00000 +0.00000 +0.96390
C +1.27184 +0.00000 +1.37111
C +1.41181 +0.02970 +2.89048
N +0.22001 −0.33503 +3.63109
H −0.56117 +0.25203 +3.36642
O +2.19326 +0.03454 +0.59769
C +2.62992 −0.78850 +3.39906
H +1.61600 +1.08871 +3.10213
H −0.02728 −1.29182 +3.39745
C +3.26567 −1.80497 +2.45406
H +3.41776 −0.10629 +3.72774
H +2.27334 −1.30691 +4.29336
C +2.26219 −2.75556 +1.84500
H +3.80306 −1.31184 +1.63781
H +3.99758 −2.40485 +3.00994
O +2.75527 −3.64086 +0.96601
H +3.69211 −3.49215 +0.81913
O +1.09325 −2.75659 +2.10556
19
∗ E = +7.656 kcal/mol ; (268) 173_157_033_062_276_163_356
H +0.00000 +0.00000 +0.00000
O +0.00000 +0.00000 +0.96502
C +1.27953 +0.00000 +1.37751
C +1.38805 +0.16805 +2.88087
N +2.66596 −0.36671 +3.29801
H +3.35841 −0.21050 +2.57362
O +2.20707 −0.10827 +0.63262
C +1.17777 +1.64718 +3.27078
H +0.56977 −0.41776 +3.32473
H +2.98698 +0.06039 +4.15642
C −0.19800 +2.20499 +2.92175
H +1.33023 +1.70965 +4.35342
H +1.94971 +2.25714 +2.78908
C −0.27970 +2.68301 +1.48319
H −0.98096 +1.45715 +3.10261

H −0.43240 +3.07091 +3.55301
O −1.52262 +2.85879 +1.00044
H −2.18280 +2.58260 +1.63944
O +0.67160 +2.92465 +0.80540
19
∗ E = +7.672 kcal/mol ; (269) 006_170_290_284_062_237_173
H +0.00000 +0.00000 +0.00000
O +0.00000 +0.00000 +0.95988
C +1.24642 +0.00000 +1.45100
C +2.38220 +0.14058 +0.42108
N +3.66684 +0.36547 +1.04895
H +3.69427 +1.28789 +1.46634
O +1.44115 −0.07643 +2.62680
C +2.45808 −1.07260 −0.52770
H +2.13531 +1.02028 −0.18904
H +3.77757 −0.28457 +1.82362
C +3.08845 −2.30218 +0.13135
H +1.46165 −1.34337 −0.89707
H +3.06723 −0.78514 −1.38823
C +2.24288 −2.74886 +1.29934
H +3.11994 −3.12488 −0.58866
H +4.10942 −2.08986 +0.45347
O +2.92455 −2.79717 +2.44897
H +2.29822 −2.97657 +3.16109
O +1.07121 −2.99070 +1.21931
19
∗ E = +7.673 kcal/mol ; (270) 000_184_225_301_257_055_002
H +0.00000 +0.00000 +0.00000
O +0.00000 +0.00000 +0.96011
C +1.24176 +0.00000 +1.45653
C +2.37441 +0.00743 +0.42834
N +3.64185 −0.08708 +1.14295
H +4.32183 +0.57221 +0.78789
O +1.43180 −0.00445 +2.63462
C +2.22346 −1.15735 −0.56541
H +2.28568 +0.95734 −0.12115
H +3.48946 +0.10316 +2.12972
C +2.22105 −2.53925 +0.12118
H +1.30891 −1.03667 −1.15791
H +3.04521 −1.08869 −1.28665

C	+3.54059	−3.28794	−0.01672
H	+2.00830	−2.42989	+1.19415
H	+1.45147	−3.18671	−0.29819
O	+4.61628	−2.62857	+0.40829
H	+4.36567	−1.75533	+0.77475
O	+3.61405	−4.38834	−0.48012

19

∗ E = +7.757 kcal/mol ; (271) 354_164_026_304_187_181_180

H	+0.00000	+0.00000	+0.00000
O	+0.00000	+0.00000	+0.96049
C	+1.25192	+0.00000	+1.44436
C	+2.35537	−0.14041	+0.40290
N	+3.60666	+0.26065	+1.00134
H	+3.57858	+0.13164	+2.00629
O	+1.45377	+0.04083	+2.61951
C	+2.33134	−1.58740	−0.13189
H	+2.11441	+0.53206	−0.43557
H	+4.39661	−0.23420	+0.61079
C	+2.46296	−2.62137	+0.97596
H	+1.41387	−1.77509	−0.70290
H	+3.15258	−1.70461	−0.84744
C	+2.60439	−4.01332	+0.42226
H	+3.33252	−2.41815	+1.61251
H	+1.59972	−2.61197	+1.65279
O	+2.69623	−4.93322	+1.39299
H	+2.78243	−5.79807	+0.97634
O	+2.63481	−4.29514	−0.74273

19

∗ E = +7.790 kcal/mol ; (272) 177_009_301_130_294_350_003

H	+0.00000	+0.00000	+0.00000
O	+0.00000	+0.00000	+0.96503
C	+1.26251	+0.00000	+1.38389
C	+1.43056	+0.06357	+2.89758
N	+0.24199	−0.08946	+3.70402
H	−0.45041	+0.61735	+3.49105
O	+2.20224	−0.01501	+0.62984
C	+2.52647	−0.93458	+3.33158
H	+1.81110	+1.08414	+3.06220
H	−0.18877	−0.99405	+3.55269
C	+3.57052	−0.29539	+4.23424

H +2.03783 −1.75285 +3.86923
H +3.00752 −1.38120 +2.45482
C +4.44862 +0.78126 +3.60333
H +3.09494 +0.16733 +5.10488
H +4.25848 −1.04991 +4.62901
O +4.39724 +0.95626 +2.27741
H +3.75964 +0.40405 +1.80277
O +5.17946 +1.45004 +4.27194
19
* E = +7.823 kcal/mol ; (273) 014_211_204_300_136_312_183
H +0.00000 +0.00000 +0.00000
O +0.00000 +0.00000 +0.96407
C +1.21837 +0.00000 +1.50875
C +2.41654 +0.33135 +0.60864
N +3.55448 −0.40724 +1.12203
H +4.42849 +0.02705 +0.85724
O +1.36303 −0.19846 +2.67870
C +2.20655 +0.01359 −0.87025
H +2.52604 +1.42676 +0.70869
H +3.50481 −0.45377 +2.13366
C +1.92463 −1.48068 −1.12162
H +1.40645 +0.64248 −1.27704
H +3.11010 +0.31106 −1.41075
C +0.80279 −1.71856 −2.09531
H +2.80289 −2.01325 −1.48256
H +1.63410 −1.96753 −0.18006
O −0.28444 −0.96663 −1.78522
H −0.99198 −1.17989 −2.40378
O +0.80317 −2.47163 −3.02109
19
* E = +7.830 kcal/mol ; (274) 180_173_293_148_291_350_002
H +0.00000 +0.00000 +0.00000
O +0.00000 +0.00000 +0.96408
C +1.27523 +0.00000 +1.40731
C +1.30767 −0.00398 +2.92743
N +2.62140 −0.17249 +3.49746
H +2.98910 −1.09676 +3.30955
O +2.22083 +0.00796 +0.67853
C +0.65960 +1.29045 +3.46509
H +0.68601 −0.85829 +3.23266

H	+3.26956	+0.49031	+3.08525
C	−0.02703	+1.05698	+4.80187
H	+1.45496	+2.03312	+3.58518
H	−0.04151	+1.71662	+2.73726
C	−1.27638	+0.18411	+4.77413
H	+0.66718	+0.58378	+5.50342
H	−0.33069	+2.00324	+5.26084
O	−1.80201	−0.13495	+3.57683
H	−1.29941	+0.20371	+2.82969
O	−1.79632	−0.20287	+5.77555

19

∗ E = +7.924 kcal/mol ; (275) 182_327_061_067_069_192_005

H	+0.00000	+0.00000	+0.00000
O	+0.00000	+0.00000	+0.96379
C	+1.27384	+0.00000	+1.37915
C	+1.39749	−0.06001	+2.88605
N	+0.30154	+0.64693	+3.51890
H	−0.59093	+0.22934	+3.28273
O	+2.20122	−0.03395	+0.61732
C	+1.52102	−1.54904	+3.28398
H	+2.33504	+0.44376	+3.14172
H	+0.40781	+0.62906	+4.52531
C	+2.81374	−2.19636	+2.80496
H	+1.48652	−1.59414	+4.37703
H	+0.65254	−2.09664	+2.89904
C	+4.01615	−1.64818	+3.55223
H	+2.79290	−3.27917	+2.97926
H	+2.95933	−2.04830	+1.72802
O	+5.21397	−1.96721	+3.03396
H	+5.11593	−2.43942	+2.20427
O	+3.94215	−0.99757	+4.55042

19

∗ E = +7.935 kcal/mol ; (276) 177_161_032_303_190_185_001

H	+0.00000	+0.00000	+0.00000
O	+0.00000	+0.00000	+0.96406
C	+1.27014	+0.00000	+1.38381
C	+1.37080	+0.06892	+2.89217
N	+2.69198	−0.37389	+3.28143
H	+3.37942	−0.13816	+2.57587
O	+2.20670	+0.01252	+0.63462

C +1.00545 +1.50425 +3.32592
H +0.61075 −0.60696 +3.30242
H +2.97228 +0.00371 +4.17622
C +1.88018 +2.55580 +2.65994
H −0.04861 +1.70252 +3.10794
H +1.11640 +1.57705 +4.41278
C +1.68484 +3.92795 +3.27088
H +2.94269 +2.29074 +2.75260
H +1.67787 +2.61907 +1.58209
O +2.35022 +4.92667 +2.65851
H +2.84660 +4.60025 +1.90478
O +1.00885 +4.14910 +4.22734
19
∗ E = +7.971 kcal/mol ; (277) 000_166_031_147_293_346_183
H +0.00000 +0.00000 +0.00000
O +0.00000 +0.00000 +0.96533
C +1.25493 +0.00000 +1.43515
C +2.32694 −0.00207 +0.35369
N +3.60021 +0.33561 +0.94530
H +3.63099 +0.00738 +1.90473
O +1.48768 −0.03112 +2.60463
C +2.27350 −1.36190 −0.39112
H +2.04444 +0.78872 −0.35997
H +4.37956 −0.05061 +0.42975
C +2.63580 −1.21906 −1.86320
H +2.97118 −2.04466 +0.10299
H +1.28108 −1.81820 −0.31142
C +1.64008 −0.42995 −2.68189
H +3.60635 −0.73127 −2.00440
H +2.71653 −2.19769 −2.34974
O +0.44994 −0.23988 −2.05382
H −0.12366 +0.23216 −2.67006
O +1.83259 −0.01750 −3.78488
19
∗ E = +7.974 kcal/mol ; (278) 183_323_295_171_174_179_001
H +0.00000 +0.00000 +0.00000
O +0.00000 +0.00000 +0.96389
C +1.27722 +0.00000 +1.38049
C +1.38604 −0.08349 +2.89662
N +0.36987 +0.64612 +3.63381

```
 H    +0.44088    +1.64382    +3.47895
 O    +2.20678    +0.02292    +0.62652
 C    +1.34256    -1.56631    +3.28430
 H    +2.37122    +0.32518    +3.14757
 H    -0.55511    +0.35781    +3.33358
 C    +1.65985    -1.76087    +4.75734
 H    +0.34412    -1.96611    +3.06953
 H    +2.04504    -2.13628    +2.66854
 C    +1.47365    -3.19839    +5.19105
 H    +2.69464    -1.46310    +4.97432
 H    +1.01529    -1.11625    +5.36786
 O    +1.75768    -3.44704    +6.48534
 H    +2.02875    -2.64532    +6.93765
 O    +1.10313    -4.07668    +4.47502
19
 * E =   +8.029  kcal/mol  ;  (279)  181_185_296_279_053_058_356
 H    +0.00000    +0.00000    +0.00000
 O    +0.00000    +0.00000    +0.96449
 C    +1.28505    +0.00000    +1.37048
 C    +1.41659    -0.02820    +2.89509
 N    +2.78705    +0.08497    +3.34106
 H    +3.32533    -0.71290    +3.02626
 O    +2.20444    -0.01869    +0.60869
 C    +0.52757    +1.01935    +3.58613
 H    +1.03302    -1.01561    +3.18549
 H    +3.22740    +0.89815    +2.91724
 C    +1.13929    +2.42547    +3.57683
 H    -0.46927    +1.00633    +3.13116
 H    +0.40150    +0.71881    +4.62940
 C    +1.54071    +2.90618    +2.19574
 H    +0.42510    +3.14151    +3.99930
 H    +2.03862    +2.44825    +4.19413
 O    +0.55275    +2.94331    +1.27917
 H    -0.26703    +2.59158    +1.63593
 O    +2.65063    +3.22711    +1.89881
19
 * E =   +8.029  kcal/mol  ;  (280)  354_162_314_330_294_169_180
 H    +0.00000    +0.00000    +0.00000
 O    +0.00000    +0.00000    +0.96045
 C    +1.26148    +0.00000    +1.44010
```

C	+2.35742	−0.15506	+0.38450
N	+3.66538	+0.25702	+0.82961
H	+3.62010	+1.14875	+1.30729
O	+1.46443	+0.05302	+2.61354
C	+2.36405	−1.63517	−0.08324
H	+2.07688	+0.47083	−0.47662
H	+4.04391	−0.42222	+1.48091
C	+1.91519	−2.66428	+0.95431
H	+1.71395	−1.74481	−0.95706
H	+3.38384	−1.85376	−0.41433
C	+2.83737	−2.80183	+2.13830
H	+0.90655	−2.46289	+1.33270
H	+1.86082	−3.65708	+0.49178
O	+2.28316	−3.52549	+3.11466
H	+2.91414	−3.58100	+3.84134
O	+3.95347	−2.36488	+2.21056

19

∗ E = +8.036 kcal/mol ; (281) 358_168_275_172_100_305_000

H	+0.00000	+0.00000	+0.00000
O	+0.00000	+0.00000	+0.96024
C	+1.24549	+0.00000	+1.45592
C	+2.37824	−0.05748	+0.42780
N	+3.66392	+0.22649	+1.04964
H	+3.83610	+1.22358	+1.09400
O	+1.43979	+0.01488	+2.63234
C	+2.39865	−1.44088	−0.24413
H	+2.18442	+0.69848	−0.34597
H	+3.62462	−0.09453	+2.01598
C	+3.38841	−1.52407	−1.42213
H	+2.64006	−2.19097	+0.51958
H	+1.39361	−1.68853	−0.60197
C	+4.71920	−2.19118	−1.09398
H	+2.96171	−2.09472	−2.24717
H	+3.60378	−0.51500	−1.79812
O	+5.38814	−1.67415	−0.06733
H	+4.90374	−0.91780	+0.32384
O	+5.14147	−3.11767	−1.72265

19

∗ E = +8.052 kcal/mol ; (282) 002_341_166_182_316_288_358

H	+0.00000	+0.00000	+0.00000

O	+0.00000	+0.00000	+0.97131
C	+1.26938	+0.00000	+1.37090
C	+2.29265	+0.05627	+0.23335
N	+1.67263	−0.34662	−1.02343
H	+2.25965	−0.10264	−1.81351
O	+1.59180	+0.01249	+2.52122
C	+2.78228	+1.50973	+0.17265
H	+3.13280	−0.58724	+0.53385
H	+1.57480	−1.35570	−1.05852
C	+3.78480	+1.76498	−0.96403
H	+1.92575	+2.18067	+0.04309
H	+3.22341	+1.74092	+1.14829
C	+4.81141	+0.65362	−1.02824
H	+3.28212	+1.79498	−1.93293
H	+4.27330	+2.73459	−0.81980
O	+5.72216	+0.63476	−0.04100
H	+5.60709	+1.37426	+0.56045
O	+4.79930	−0.20554	−1.85838

19

∗ E = +8.087 kcal/mol ; (283) 179_149_048_068_068_189_003

H	+0.00000	+0.00000	+0.00000
O	+0.00000	+0.00000	+0.96469
C	+1.27938	+0.00000	+1.37455
C	+1.41359	+0.02566	+2.88267
N	+2.62371	−0.69557	+3.21935
H	+3.39344	−0.38086	+2.63909
O	+2.20157	+0.02772	+0.61095
C	+1.41086	+1.49808	+3.35166
H	+0.54814	−0.48352	+3.31977
H	+2.86646	−0.56315	+4.19241
C	+0.08320	+2.22126	+3.16316
H	+1.65485	+1.49291	+4.41894
H	+2.21206	+2.03196	+2.82801
C	−0.99545	+1.66800	+4.07698
H	+0.19225	+3.28642	+3.40291
H	−0.26496	+2.16838	+2.12480
O	−2.24101	+2.11507	+3.83565
H	−2.25734	+2.69693	+3.07289
O	−0.78854	+0.90917	+4.97378

19

∗ E = +8.089 kcal/mol ; (284) 006_063_174_287_276_171_179
H +0.00000 +0.00000 +0.00000
O +0.00000 +0.00000 +0.95938
C +1.25395 +0.00000 +1.45470
C +2.36144 +0.14422 +0.40970
N +2.20445 +1.43977 −0.24590
H +2.97722 +1.61463 −0.87862
O +1.43581 −0.11098 +2.62695
C +3.73496 +0.04629 +1.06937
H +2.25244 −0.71290 −0.28135
H +1.33776 +1.53724 −0.75788
C +4.10026 +1.28432 +1.88089
H +3.73797 −0.83269 +1.71784
H +4.48970 −0.10635 +0.29092
C +4.68681 +2.38402 +1.03662
H +3.24167 +1.68354 +2.42781
H +4.85376 +1.04112 +2.63875
O +4.82360 +3.52424 +1.72374
H +5.22225 +4.17703 +1.13757
O +5.03798 +2.28275 −0.10958
19
∗ E = +8.114 kcal/mol ; (285) 355_294_282_226_069_186_003
H +0.00000 +0.00000 +0.00000
O +0.00000 +0.00000 +0.97191
C +1.23778 +0.00000 +1.47651
C +2.36311 −0.11044 +0.44427
N +2.34565 −1.40431 −0.24344
H +2.74086 −2.11774 +0.35531
O +1.43377 +0.05273 +2.65507
C +2.32848 +1.10063 −0.53352
H +3.29069 −0.06960 +1.01835
H +1.40329 −1.69645 −0.47774
C +2.47011 +0.71458 −1.99672
H +1.39282 +1.65770 −0.41622
H +3.13011 +1.79220 −0.26442
C +1.24799 −0.02389 −2.48994
H +2.58633 +1.61159 −2.61815
H +3.34664 +0.07872 −2.16091
O +1.31348 −0.52602 −3.72307
H +2.17906 −0.37886 −4.11181

O +0.23855 −0.15604 −1.84861
19
∗ E = +8.116 kcal/mol ; (286) 357_167_290_173_177_311_181
H +0.00000 +0.00000 +0.00000
O +0.00000 +0.00000 +0.96020
C +1.25350 +0.00000 +1.44875
C +2.37531 −0.06100 +0.40693
N +3.67348 +0.24790 +0.96180
H +3.73454 +1.22713 +1.21240
O +1.45073 +0.02068 +2.62411
C +2.39633 −1.46496 −0.21788
H +2.15015 +0.67210 −0.38234
H +3.79501 −0.27187 +1.82618
C +3.38490 −1.53048 −1.37954
H +2.68287 −2.18537 +0.55692
H +1.39279 −1.75125 −0.55613
C +3.50455 −2.90485 −1.97945
H +3.10304 −0.84739 −2.18443
H +4.37216 −1.22791 −1.01256
O +3.69087 −3.85136 −1.04221
H +3.77698 −4.69903 −1.49266
O +3.45531 −3.16174 −3.14845
19
∗ E = +8.194 kcal/mol ; (287) 187_313_063_045_269_116_349
H +0.00000 +0.00000 +0.00000
O +0.00000 +0.00000 +0.96507
C +1.25694 +0.00000 +1.40323
C +1.33832 −0.17328 +2.90295
N +0.40044 +0.71600 +3.56216
H −0.55213 +0.48417 +3.30301
O +2.21069 +0.02251 +0.66855
C +1.13853 −1.67394 +3.21514
H +2.35804 +0.11684 +3.17565
H +0.47876 +0.63666 +4.56831
C +1.94444 −2.61227 +2.30080
H +1.41991 −1.83121 +4.26069
H +0.07381 −1.91982 +3.12427
C +1.12869 −3.01764 +1.07674
H +2.90270 −2.15887 +2.02407
H +2.15475 −3.54601 +2.82796

O	+1.57528	−2.62748	−0.12589
H	+2.28831	−1.98464	−0.04424
O	+0.12920	−3.66433	+1.17454

19

∗ E = +8.232 kcal/mol ; (288) 356_166_283_289_283_307_179

H	+0.00000	+0.00000	+0.00000
O	+0.00000	+0.00000	+0.96008
C	+1.25576	+0.00000	+1.44645
C	+2.37406	−0.08592	+0.40174
N	+3.67377	+0.23823	+0.94594
H	+3.78054	+1.23851	+1.05677
O	+1.45280	+0.02368	+2.62192
C	+2.38983	−1.50719	−0.19324
H	+2.14105	+0.62176	−0.40531
H	+3.76672	−0.16178	+1.87692
C	+2.90878	−2.54063	+0.81193
H	+1.38245	−1.79385	−0.51603
H	+3.03237	−1.50443	−1.07768
C	+4.40961	−2.51630	+0.98442
H	+2.46167	−2.40781	+1.80185
H	+2.65266	−3.54612	+0.46118
O	+5.05092	−2.62474	−0.18934
H	+5.99695	−2.58719	−0.01093
O	+4.98547	−2.45510	+2.03300

19

∗ E = +8.243 kcal/mol ; (289) 000_151_038_058_058_104_003

H	+0.00000	+0.00000	+0.00000
O	+0.00000	+0.00000	+0.97276
C	+1.23459	+0.00000	+1.46556
C	+2.37281	+0.00109	+0.44303
N	+3.50659	+0.66940	+1.04481
H	+3.56364	+0.42237	+2.02725
O	+1.44516	−0.05364	+2.64265
C	+2.67727	−1.46217	+0.04828
H	+2.05071	+0.55806	−0.44520
H	+4.37570	+0.42818	+0.58726
C	+1.50093	−2.25923	−0.54465
H	+3.51198	−1.44826	−0.66340
H	+3.02824	−1.98201	+0.94653
C	+0.94776	−1.58475	−1.78135

H +1.82413 −3.28142 −0.76556
H +0.68676 −2.32348 +0.18416
O +1.26460 −2.11352 −2.96534
H +1.78589 −2.91261 −2.86535
O +0.27569 −0.58891 −1.73819
19
∗ E = +8.250 kcal/mol ; (290) 000_338_160_304_298_299_353
H +0.00000 +0.00000 +0.00000
O +0.00000 +0.00000 +0.97168
C +1.25136 +0.00000 +1.41499
C +2.33371 −0.00523 +0.32740
N +1.79236 −0.52374 −0.91826
H +2.33802 −0.25221 −1.72661
O +1.52862 +0.05878 +2.57694
C +2.80503 +1.44496 +0.14940
H +3.16983 −0.58854 +0.73369
H +1.71442 −1.53264 −0.91248
C +1.67447 +2.43210 −0.18198
H +3.27921 +1.76105 +1.08236
H +3.58584 +1.46290 −0.62370
C +0.96145 +2.13343 −1.48665
H +0.91945 +2.43750 +0.60806
H +2.08912 +3.44569 −0.24281
O +1.74735 +2.16907 −2.58802
H +2.62661 +2.48956 −2.37411
O −0.19771 +1.87724 −1.58491
19
∗ E = +8.254 kcal/mol ; (291) 179_324_049_302_296_111_352
H +0.00000 +0.00000 +0.00000
O +0.00000 +0.00000 +0.96499
C +1.29494 +0.00000 +1.36833
C +1.44707 +0.03636 +2.87518
N +0.42144 +0.86440 +3.48213
H −0.51007 +0.65161 +3.15039
O +2.19474 −0.08592 +0.58831
C +1.54684 −1.41828 +3.38917
H +2.42233 +0.50404 +3.04638
H +0.42412 +0.76530 +4.48975
C +0.35109 −2.30971 +3.03748
H +2.46044 −1.87000 +2.98942

H +1.64587 −1.37957 +4.47986
C −0.92192 −1.81874 +3.70615
H +0.23056 −2.39593 +1.95203
H +0.53374 −3.31418 +3.42772
O −1.86702 −1.30235 +2.89917
H −1.53077 −1.20568 +2.00285
O −1.09109 −1.87030 +4.88665
19
∗ E = +8.324 kcal/mol ; (292) 183_065_305_064_175_176_358
H +0.00000 +0.00000 +0.00000
O +0.00000 +0.00000 +0.96394
C +1.27564 +0.00000 +1.38488
C +1.35764 −0.08086 +2.90559
N +0.83050 −1.35195 +3.38776
H −0.10990 −1.51419 +3.04892
O +2.20403 +0.01904 +0.62598
C +2.78512 +0.10645 +3.39281
H +0.71564 +0.71609 +3.30540
H +1.41459 −2.12119 +3.07921
C +3.35099 +1.48299 +3.08062
H +2.80357 −0.06615 +4.47196
H +3.42809 −0.65606 +2.93837
C +4.72630 +1.67214 +3.68720
H +3.42140 +1.63848 +1.99711
H +2.69918 +2.27411 +3.47774
O +5.32984 +2.83842 +3.38093
H +4.78743 +3.35984 +2.78550
O +5.26748 +0.87971 +4.39358
19
∗ E = +8.350 kcal/mol ; (293) 002_188_214_304_187_051_179
H +0.00000 +0.00000 +0.00000
O +0.00000 +0.00000 +0.96006
C +1.24854 +0.00000 +1.45135
C +2.37065 +0.03745 +0.41422
N +3.63020 −0.16754 +1.09452
H +4.38517 +0.34804 +0.66640
O +1.43995 −0.01054 +2.62835
C +2.17507 −1.03182 −0.67512
H +2.29276 +1.03035 −0.06276
H +3.55178 +0.08883 +2.07163

C +2.05443 −2.42744 −0.06493
H +1.30403 −0.80695 −1.30667
H +3.04413 −0.99422 −1.33866
C +2.03358 −3.52257 −1.09537
H +2.92218 −2.58391 +0.58743
H +1.14954 −2.53145 +0.54008
O +3.05003 −3.41235 −1.97155
H +2.98685 −4.15188 −2.58627
O +1.23015 −4.40824 −1.15852
19
∗ E = +8.351 kcal/mol ; (294) 187_318_284_294_082_196_003
H +0.00000 +0.00000 +0.00000
O +0.00000 +0.00000 +0.96457
C +1.28524 +0.00000 +1.37955
C +1.38352 −0.17219 +2.88934
N +0.39679 +0.59676 +3.63784
H +0.65451 +1.57484 +3.67230
O +2.20829 +0.11589 +0.63151
C +1.30417 −1.66134 +3.26548
H +2.38156 +0.18116 +3.16084
H −0.51077 +0.55055 +3.18959
C −0.04620 −2.30807 +2.97479
H +2.08442 −2.21571 +2.73471
H +1.50562 −1.71698 +4.33825
C −0.19294 −2.73917 +1.52710
H −0.16578 −3.21480 +3.58088
H −0.87371 −1.64441 +3.25557
O −1.45099 −2.99941 +1.12561
H −2.08014 −2.81659 +1.82638
O +0.72141 −2.88004 +0.77422
19
∗ E = +8.399 kcal/mol ; (295) 171_149_290_064_287_335_005
H +0.00000 +0.00000 +0.00000
O +0.00000 +0.00000 +0.96595
C +1.25927 +0.00000 +1.40441
C +1.35522 +0.21988 +2.90729
N +2.49029 −0.45695 +3.50038
H +2.35534 −1.46060 +3.50120
O +2.21712 −0.08750 +0.68617
C +1.48989 +1.73063 +3.16374

H +0.42887 −0.13349 +3.37302
H +3.32057 −0.27427 +2.94478
C +0.29861 +2.58889 +2.74042
H +1.64053 +1.84111 +4.24107
H +2.43063 +2.07646 +2.70754
C +0.04292 +2.83840 +1.26111
H −0.63426 +2.17729 +3.13567
H +0.40282 +3.58631 +3.18366
O +1.09268 +2.76540 +0.42205
H +1.89983 +2.47779 +0.85653
O −1.03354 +3.13497 +0.84259
19
∗ E = +8.407 kcal/mol ; (296) 179_152_298_060_172_181_000
H +0.00000 +0.00000 +0.00000
O +0.00000 +0.00000 +0.96473
C +1.27997 +0.00000 +1.37884
C +1.40611 +0.02155 +2.89784
N +2.62928 −0.59583 +3.36258
H +2.66799 −1.57335 +3.10067
O +2.20414 +0.00134 +0.61772
C +1.36206 +1.47152 +3.39651
H +0.54689 −0.52169 +3.31015
H +3.42634 −0.14525 +2.92462
C +0.08062 +2.21511 +3.05254
H +1.49461 +1.44768 +4.48123
H +2.22524 +2.01512 +2.99421
C +0.02505 +3.57014 +3.72931
H −0.02264 +2.37271 +1.97171
H −0.80143 +1.63544 +3.35749
O −1.09334 +4.28305 +3.48656
H −1.69265 +3.80092 +2.91289
O +0.88330 +4.01134 +4.42771
19
∗ E = +8.445 kcal/mol ; (297) 002_189_215_303_182_309_181
H +0.00000 +0.00000 +0.00000
O +0.00000 +0.00000 +0.96030
C +1.24876 +0.00000 +1.45182
C +2.37176 +0.04890 +0.41577
N +3.63054 −0.18045 +1.09102
H +4.38601 +0.34995 +0.68216

O	+1.43901	−0.01977	+2.62895
C	+2.15874	−0.99042	−0.69749
H	+2.30178	+1.05349	−0.03774
H	+3.55021	+0.04755	+2.07507
C	+2.03495	−2.40450	−0.12980
H	+1.28477	−0.74928	−1.31612
H	+3.02203	−0.93486	−1.36975
C	+1.87218	−3.44630	−1.20354
H	+2.91405	−2.65275	+0.46579
H	+1.15239	−2.47052	+0.52158
O	+0.88404	−3.12625	−2.06414
H	+0.81988	−3.83571	−2.71330
O	+2.51157	−4.45125	−1.31352

19

∗ E = +8.488 kcal/mol ; (298) 356_148_298_059_176_182_181

H	+0.00000	+0.00000	+0.00000
O	+0.00000	+0.00000	+0.96002
C	+1.25759	+0.00000	+1.44302
C	+2.38795	−0.08841	+0.41023
N	+3.57734	+0.62339	+0.82446
H	+3.38940	+1.61246	+0.93692
O	+1.45712	+0.03094	+2.61822
C	+2.74334	−1.56432	+0.19663
H	+2.04118	+0.33828	−0.54338
H	+3.86825	+0.27955	+1.73458
C	+1.57963	−2.41681	−0.28517
H	+3.56762	−1.60975	−0.52001
H	+3.12875	−1.96771	+1.14052
C	+2.01017	−3.83066	−0.58154
H	+0.76763	−2.47103	+0.45041
H	+1.13131	−2.01359	−1.20351
O	+0.99166	−4.57348	−1.04232
H	+1.32268	−5.46417	−1.20407
O	+3.11555	−4.26447	−0.43238

19

∗ E = +8.498 kcal/mol ; (299) 358_170_283_297_178_314_181

H	+0.00000	+0.00000	+0.00000
O	+0.00000	+0.00000	+0.96024
C	+1.25263	+0.00000	+1.44818
C	+2.37697	−0.05030	+0.40672

N	+3.67973	+0.19547	+0.98575
H	+3.80821	+1.18345	+1.16535
O	+1.44749	−0.00451	+2.62508
C	+2.34400	−1.41914	−0.29491
H	+2.17746	+0.72204	−0.34796
H	+3.73596	−0.25879	+1.89240
C	+2.62107	−2.56144	+0.68253
H	+1.37707	−1.58432	−0.78333
H	+3.10809	−1.40568	−1.07784
C	+2.56008	−3.92074	+0.03580
H	+3.60968	−2.46439	+1.13520
H	+1.87808	−2.55671	+1.49232
O	+1.47492	−4.05986	−0.74910
H	+1.48760	−4.95359	−1.10969
O	+3.35451	−4.80202	+0.19011

19

∗ E = +8.498 kcal/mol ; (300) 358_168_293_174_180_048_179

H	+0.00000	+0.00000	+0.00000
O	+0.00000	+0.00000	+0.96022
C	+1.25362	+0.00000	+1.44862
C	+2.37623	−0.03628	+0.40534
N	+3.67638	+0.24544	+0.96920
H	+3.72632	+1.20361	+1.29359
O	+1.45059	+0.00711	+2.62420
C	+2.38261	−1.41277	−0.27563
H	+2.15523	+0.72720	−0.35635
H	+3.81377	−0.33466	+1.79172
C	+3.38625	−1.45759	−1.42604
H	+2.64181	−2.16773	+0.47808
H	+1.38356	−1.67136	−0.64294
C	+3.42512	−2.79451	−2.11852
H	+3.11100	−0.70900	−2.18063
H	+4.39120	−1.22202	−1.07621
O	+2.19062	−3.23911	−2.42536
H	+2.28705	−4.08624	−2.87485
O	+4.41237	−3.41195	−2.39377

19

∗ E = +8.506 kcal/mol ; (301) 184_075_078_064_068_186_001

H	+0.00000	+0.00000	+0.00000
O	+0.00000	+0.00000	+0.96382

C	+1.26687	+0.00000	+1.38012
C	+1.38253	−0.09058	+2.89660
N	+1.08953	−1.47237	+3.25708
H	+1.88871	−2.06870	+3.07513
O	+2.20133	+0.05099	+0.62592
C	+2.75313	+0.40382	+3.35818
H	+0.59315	+0.53689	+3.32438
H	+0.86195	−1.54492	+4.23982
C	+3.00298	+1.87629	+3.05981
H	+2.82336	+0.23761	+4.43767
H	+3.52377	−0.19891	+2.86579
C	+2.12142	+2.78939	+3.88928
H	+4.04437	+2.14272	+3.27958
H	+2.85275	+2.08931	+1.99349
O	+2.19888	+4.09779	+3.58291
H	+2.80277	+4.24472	+2.85161
O	+1.40609	+2.41815	+4.76978

19

∗ E = +8.529 kcal/mol ; (302) 180_025_283_311_314_253_001

H	+0.00000	+0.00000	+0.00000
O	+0.00000	+0.00000	+0.96374
C	+1.27155	+0.00000	+1.37131
C	+1.40793	−0.01180	+2.89064
N	+0.28816	−0.58078	+3.62113
H	−0.47885	+0.07662	+3.66483
O	+2.20070	+0.05818	+0.61262
C	+2.75819	−0.62064	+3.30687
H	+1.46219	+1.05066	+3.16555
H	−0.06033	−1.41198	+3.15807
C	+3.12758	−1.96775	+2.66024
H	+3.55840	+0.08045	+3.05431
H	+2.72563	−0.71483	+4.39598
C	+1.98656	−2.95930	+2.66269
H	+3.37837	−1.79519	+1.61047
H	+4.01339	−2.37617	+3.15747
O	+2.06470	−3.99395	+3.51277
H	+2.86952	−3.95297	+4.03234
O	+1.03290	−2.84360	+1.94617

19

∗ E = +8.539 kcal/mol ; (303) 174_193_218_301_081_068_359

H	+0.00000	+0.00000	+0.00000
O	+0.00000	+0.00000	+0.96476
C	+1.26938	+0.00000	+1.38788
C	+1.35659	+0.13961	+2.89968
N	+2.72665	+0.43781	+3.26210
H	+3.00390	−0.02399	+4.11591
O	+2.21166	−0.12120	+0.66200
C	+0.39735	+1.21726	+3.43489
H	+1.00993	−0.83220	+3.28920
H	+3.35587	+0.16397	+2.51607
C	+0.67419	+2.60471	+2.83619
H	−0.63833	+0.90277	+3.26064
H	+0.54289	+1.26495	+4.51823
C	+0.06435	+2.79697	+1.46121
H	+0.27273	+3.38193	+3.49692
H	+1.75328	+2.75368	+2.75547
O	−1.28445	+2.83934	+1.42909
H	−1.65433	+2.72777	+2.30726
O	+0.68822	+2.90415	+0.45000

19

∗ E = +8.583 kcal/mol ; (304) 001_189_207_296_286_320_183

H	+0.00000	+0.00000	+0.00000
O	+0.00000	+0.00000	+0.95953
C	+1.25037	+0.00000	+1.44945
C	+2.37432	+0.01988	+0.41234
N	+3.62137	−0.20627	+1.10521
H	+4.41098	+0.19830	+0.62309
O	+1.44162	+0.01503	+2.62578
C	+2.18699	−1.05367	−0.67091
H	+2.30459	+1.01456	−0.06584
H	+3.57545	+0.13144	+2.05880
C	+2.26270	−2.46282	−0.08613
H	+1.23774	−0.92105	−1.20519
H	+2.97068	−0.92492	−1.42383
C	+3.65384	−2.92099	+0.28058
H	+1.64560	−2.56652	+0.81180
H	+1.89019	−3.18693	−0.82014
O	+4.57463	−2.54615	−0.63104
H	+5.41836	−2.91486	−0.34784
O	+3.92485	−3.61894	+1.21248

```
19
 * E =   +8.602  kcal/mol  ;  (305)  181_051_181_068_168_169_357
 H    +0.00000    +0.00000    +0.00000
 O    +0.00000    +0.00000    +0.96388
 C    +1.27417    +0.00000    +1.38326
 C    +1.35718    -0.01908    +2.90041
 N    +0.52229    -1.11233    +3.37933
 H    +0.54115    -1.15581    +4.39022
 O    +2.20225    +0.03296    +0.62658
 C    +2.78937    -0.20900    +3.37992
 H    +0.99812    +0.97671    +3.22654
 H    -0.43850    -1.00199    +3.08067
 C    +3.69499    +0.97540    +3.07704
 H    +2.77707    -0.37252    +4.46294
 H    +3.19865    -1.11976    +2.93242
 C    +5.01791    +0.87116    +3.80834
 H    +3.87643    +1.05948    +2.00003
 H    +3.22506    +1.91766    +3.39426
 O    +5.95493    +1.75956    +3.42180
 H    +5.63888    +2.29308    +2.68953
 O    +5.24859    +0.09323    +4.68179
19
 * E =   +8.631  kcal/mol  ;  (306)  359_345_173_302_263_068_358
 H    +0.00000    +0.00000    +0.00000
 O    +0.00000    +0.00000    +0.96544
 C    +1.26170    +0.00000    +1.40832
 C    +2.35127    -0.02053    +0.33252
 N    +1.78806    -0.37914    -0.97138
 H    +2.49664    -0.28482    -1.69322
 O    +1.52768    +0.04612    +2.56935
 C    +3.00945    +1.36500    +0.29298
 H    +3.10044    -0.74142    +0.68332
 H    +1.49145    -1.34990    -0.98819
 C    +2.05773    +2.54738    +0.00611
 H    +3.46425    +1.51408    +1.27529
 H    +3.83043    +1.34981    -0.43441
 C    +2.02412    +2.98363    -1.44969
 H    +1.03669    +2.31304    +0.33596
 H    +2.38215    +3.42166    +0.57018
 O    +1.47118    +2.11774    -2.31372
```

H +1.19760 +1.30543 −1.86535
O +2.46522 +4.02602 −1.82893
19
∗ E = +8.691 kcal/mol ; (307) 180_052_296_290_180_185_002
H +0.00000 +0.00000 +0.00000
O +0.00000 +0.00000 +0.96382
C +1.27678 +0.00000 +1.38136
C +1.36830 +0.01081 +2.90495
N +0.56592 −1.03621 +3.52690
H −0.41931 −0.88899 +3.34624
O +2.20202 −0.01241 +0.61888
C +2.81892 −0.03844 +3.36916
H +0.93943 +0.97105 +3.21902
H +0.80121 −1.94905 +3.15508
C +3.49278 −1.37763 +3.10627
H +3.39018 +0.74887 +2.86971
H +2.84675 +0.17083 +4.44139
C +4.93255 −1.37323 +3.57897
H +2.97918 −2.18895 +3.64012
H +3.47678 −1.61936 +2.03569
O +5.63051 −2.49205 +3.29631
H +5.09565 −3.11317 +2.79775
O +5.44642 −0.47452 +4.16875
19
∗ E = +8.694 kcal/mol ; (308) 178_034_291_294_292_293_004
H +0.00000 +0.00000 +0.00000
O +0.00000 +0.00000 +0.96406
C +1.27380 +0.00000 +1.37741
C +1.38808 +0.03800 +2.90150
N +0.34296 −0.66701 +3.61937
H −0.54425 −0.18994 +3.52938
O +2.20199 +0.00202 +0.61784
C +2.78385 −0.41622 +3.33248
H +1.29757 +1.10320 +3.15704
H +0.21109 −1.61051 +3.27057
C +3.07791 −1.89026 +3.02328
H +3.52862 +0.19452 +2.81602
H +2.87447 −0.21451 +4.40613
C +2.25376 −2.87375 +3.83169
H +2.89982 −2.09892 +1.96634

H	+4.13666	−2.09474	+3.22291
O	+2.49917	−2.89573	+5.15234
H	+3.13820	−2.22329	+5.39797
O	+1.43795	−3.60914	+3.35916

19

∗ E = +8.730 kcal/mol ; (309) 180_179_283_295_290_301_007

H	+0.00000	+0.00000	+0.00000
O	+0.00000	+0.00000	+0.96417
C	+1.27771	+0.00000	+1.38049
C	+1.38446	−0.00435	+2.90328
N	+2.75112	−0.02217	+3.37215
H	+3.15312	−0.94640	+3.28600
O	+2.20634	+0.01182	+0.62728
C	+0.62254	+1.19739	+3.48254
H	+0.87425	−0.91327	+3.24516
H	+3.33230	+0.59456	+2.81077
C	+1.24842	+2.53838	+3.08422
H	−0.41456	+1.17152	+3.13770
H	+0.60486	+1.07535	+4.57191
C	+2.59159	+2.84626	+3.72709
H	+1.39832	+2.59191	+2.00143
H	+0.56741	+3.35341	+3.35747
O	+2.60345	+2.88389	+5.07017
H	+1.76486	+2.59461	+5.43526
O	+3.58100	+3.09313	+3.10680

19

∗ E = +8.744 kcal/mol ; (310) 183_211_198_080_218_063_002

H	+0.00000	+0.00000	+0.00000
O	+0.00000	+0.00000	+0.96544
C	+1.29049	+0.00000	+1.37098
C	+1.46465	−0.06840	+2.87928
N	+2.68010	+0.65602	+3.19193
H	+3.01913	+0.42201	+4.11520
O	+2.19270	+0.02659	+0.58959
C	+0.27227	+0.50205	+3.65300
H	+1.52942	−1.15283	+3.09739
H	+3.40446	+0.45301	+2.51333
C	−0.91445	−0.47670	+3.76121
H	+0.62118	+0.76264	+4.65571
H	−0.02674	+1.44883	+3.18932

C	−2.25271	+0.22468	+3.64013
H	−0.86207	−1.22357	+2.95970
H	−0.90266	−1.01011	+4.71209
O	−2.46004	+0.81589	+2.44961
H	−1.71016	+0.65881	+1.86424
O	−3.07429	+0.27326	+4.50330

19

∗ E = +8.767 kcal/mol ; (311) 180_333_046_288_275_170_358

H	+0.00000	+0.00000	+0.00000
O	+0.00000	+0.00000	+0.96419
C	+1.28151	+0.00000	+1.37323
C	+1.43110	−0.00910	+2.88275
N	+0.28532	+0.59671	+3.53191
H	−0.58788	+0.26648	+3.13994
O	+2.19838	−0.05194	+0.60529
C	+1.76763	−1.45484	+3.31547
H	+2.32036	+0.60019	+3.07848
H	+0.28457	+0.38643	+4.52272
C	+0.58255	−2.40839	+3.20223
H	+2.60431	−1.81165	+2.70718
H	+2.09824	−1.43301	+4.35858
C	−0.32420	−2.37470	+4.42003
H	−0.01948	−2.19813	+2.30701
H	+0.93022	−3.44421	+3.10339
O	−1.48548	−3.03973	+4.28328
H	−1.56664	−3.41800	+3.40497
O	−0.05904	−1.83631	+5.45167

19

∗ E = +8.801 kcal/mol ; (312) 357_331_301_308_286_090_178

H	+0.00000	+0.00000	+0.00000
O	+0.00000	+0.00000	+0.96345
C	+1.25066	+0.00000	+1.44222
C	+2.38782	−0.07913	+0.41209
N	+2.10913	−0.75917	−0.83574
H	+1.82275	−1.71988	−0.71047
O	+1.46418	+0.10127	+2.61331
C	+2.91771	+1.34672	+0.18620
H	+3.18136	−0.63394	+0.92244
H	+1.46109	−0.28384	−1.44618
C	+1.86684	+2.41873	−0.16270

H	+3.39737	+1.66576	+1.11520
H	+3.68570	+1.30650	−0.59247
C	+1.32771	+2.31897	−1.56507
H	+1.03560	+2.38942	+0.54999
H	+2.34150	+3.39939	−0.09509
O	+0.23116	+1.51743	−1.65334
H	−0.06943	+1.53828	−2.57033
O	+1.77800	+2.86021	−2.52893

19

∗ E = +8.828 kcal/mol ; (313) 183_200_315_082_219_065_003

H	+0.00000	+0.00000	+0.00000
O	+0.00000	+0.00000	+0.96570
C	+1.29362	+0.00000	+1.37348
C	+1.46479	−0.07999	+2.89030
N	+2.77056	+0.38453	+3.30672
H	+3.49019	+0.00277	+2.70301
O	+2.19315	+0.04111	+0.58979
C	+0.35314	+0.66097	+3.64192
H	+1.39036	−1.15151	+3.13279
H	+2.82746	+1.39400	+3.22366
C	−0.94746	−0.15602	+3.79376
H	+0.74475	+0.90761	+4.63138
H	+0.15411	+1.61646	+3.13977
C	−2.18552	+0.70032	+3.61631
H	−0.98446	−0.95079	+3.03952
H	−1.00131	−0.62553	+4.77608
O	−2.32440	+1.21674	+2.38107
H	−1.61401	+0.90477	+1.80847
O	−2.98376	+0.92821	+4.47227

19

∗ E = +8.852 kcal/mol ; (314) 179_171_295_174_178_073_003

H	+0.00000	+0.00000	+0.00000
O	+0.00000	+0.00000	+0.96416
C	+1.27461	+0.00000	+1.38497
C	+1.37367	+0.02499	+2.90596
N	+2.71817	−0.18012	+3.39136
H	+3.05242	−1.10564	+3.15290
O	+2.20923	−0.00779	+0.63772
C	+0.81502	+1.35810	+3.42241
H	+0.73106	−0.78341	+3.28041

H	+3.34934	+0.47520	+2.94101
C	+0.75189	+1.38014	+4.95233
H	+1.46406	+2.17225	+3.07434
H	−0.17359	+1.52803	+2.98167
C	+0.23509	+2.71289	+5.45093
H	+0.09929	+0.57163	+5.30797
H	+1.74639	+1.21880	+5.36785
O	−1.08523	+2.92826	+5.25725
H	−1.50626	+2.16181	+4.86268
O	+0.91150	+3.55524	+5.95303

19

∗ E = +8.894 kcal/mol ; (315) 181_334_309_329_294_170_358

H	+0.00000	+0.00000	+0.00000
O	+0.00000	+0.00000	+0.96436
C	+1.27548	+0.00000	+1.37882
C	+1.41018	−0.03246	+2.89702
N	+0.30896	+0.51343	+3.65677
H	+0.06020	+1.44217	+3.34245
O	+2.20129	−0.03303	+0.61561
C	+1.70635	−1.49549	+3.31875
H	+2.30770	+0.55922	+3.10738
H	−0.50857	−0.08280	+3.59307
C	+1.10425	−2.58489	+2.43335
H	+2.78846	−1.64544	+3.33019
H	+1.34112	−1.60631	+4.34386
C	−0.40951	−2.65053	+2.45808
H	+1.44532	−2.47818	+1.39420
H	+1.46580	−3.56653	+2.76612
O	−0.96075	−3.45458	+1.53194
H	−0.28865	−3.84767	+0.97102
O	−1.10216	−2.07691	+3.24315

19

∗ E = +8.910 kcal/mol ; (316) 357_012_098_166_169_168_357

H	+0.00000	+0.00000	+0.00000
O	+0.00000	+0.00000	+0.97141
C	+1.26979	+0.00000	+1.36004
C	+2.29256	−0.06759	+0.21125
N	+1.63488	+0.17400	−1.07237
H	+1.73636	+1.13781	−1.36917
O	+1.60149	+0.01680	+2.50795

C	+3.46889	+0.86083	+0.50491
H	+2.64318	−1.10888	+0.21952
H	+1.97571	−0.42401	−1.81094
C	+4.65372	+0.58931	−0.40867
H	+3.15075	+1.90382	+0.38560
H	+3.75998	+0.74852	+1.55234
C	+5.71539	+1.66844	−0.30646
H	+5.10195	−0.38750	−0.18360
H	+4.33796	+0.55218	−1.46153
O	+6.89530	+1.37283	−0.88570
H	+6.89410	+0.48335	−1.24491
O	+5.54618	+2.72470	+0.21686

19
* E = +8.931 kcal/mol ; (317) 179_150_041_063_168_177_359

H	+0.00000	+0.00000	+0.00000
O	+0.00000	+0.00000	+0.96462
C	+1.27821	+0.00000	+1.37533
C	+1.40567	+0.03508	+2.88479
N	+2.63391	−0.64803	+3.23582
H	+3.36747	−0.39584	+2.58227
O	+2.20304	+0.01406	+0.61540
C	+1.34408	+1.49778	+3.36466
H	+0.54562	−0.50623	+3.30084
H	+2.93450	−0.40140	+4.16987
C	+0.02483	+2.19771	+3.07168
H	+1.51000	+1.50383	+4.44717
H	+2.17715	+2.05608	+2.92269
C	−0.09270	+3.51054	+3.82072
H	−0.09892	+2.39661	+2.00019
H	−0.82424	+1.56173	+3.35889
O	−1.20576	+4.22184	+3.55474
H	−1.75620	+3.77376	+2.90896
O	+0.71239	+3.91702	+4.59961

19
* E = +8.940 kcal/mol ; (318) 353_144_039_062_172_177_180

H	+0.00000	+0.00000	+0.00000
O	+0.00000	+0.00000	+0.95986
C	+1.25435	+0.00000	+1.44002
C	+2.37441	−0.17466	+0.41817
N	+3.48959	+0.64223	+0.84804

H	+3.56817	+0.59413	+1.85888
O	+1.46050	+0.07299	+2.61228
C	+2.70140	−1.67497	+0.31213
H	+2.02295	+0.18011	−0.56452
H	+4.35843	+0.31754	+0.44313
C	+1.53591	−2.52531	−0.17067
H	+3.54630	−1.79598	−0.37455
H	+3.04233	−2.02475	+1.29302
C	+1.95229	−3.95017	−0.43288
H	+0.70939	−2.54630	+0.54942
H	+1.11191	−2.13583	−1.10686
O	+0.91272	−4.71187	−0.80488
H	+1.23780	−5.60654	−0.95682
O	+3.06724	−4.37519	−0.33447

19

* E = +8.977 kcal/mol ; (319) 353_159_033_294_286_320_184

H	+0.00000	+0.00000	+0.00000
O	+0.00000	+0.00000	+0.96004
C	+1.25374	+0.00000	+1.44398
C	+2.35821	−0.16796	+0.40740
N	+3.58688	+0.33883	+0.96750
H	+3.62060	+0.17789	+1.96856
O	+1.45621	+0.06237	+2.61724
C	+2.39511	−1.65494	−0.01689
H	+2.08458	+0.42739	−0.47799
H	+4.40364	−0.05403	+0.52056
C	+2.81765	−2.58426	+1.12200
H	+1.40630	−1.95834	−0.38145
H	+3.08757	−1.76018	−0.85794
C	+4.28704	−2.54947	+1.46213
H	+2.27153	−2.37783	+2.04680
H	+2.60221	−3.62188	+0.84115
O	+5.07183	−2.46869	+0.36521
H	+5.98742	−2.52182	+0.66247
O	+4.74643	−2.63206	+2.56322

19

* E = +8.989 kcal/mol ; (320) 179_041_199_173_068_065_354

H	+0.00000	+0.00000	+0.00000
O	+0.00000	+0.00000	+0.96383
C	+1.27521	+0.00000	+1.37957

C	+1.38465	+0.02433	+2.89519
N	+0.39390	−0.86376	+3.48296
H	+0.22768	−0.64503	+4.45819
O	+2.20174	+0.01570	+0.62189
C	+2.80241	−0.37141	+3.29961
H	+1.22922	+1.08565	+3.17053
H	−0.49025	−0.79575	+2.99576
C	+3.05847	−0.20718	+4.80067
H	+2.96945	−1.40675	+2.97524
H	+3.51294	+0.24680	+2.74494
C	+2.28858	−1.17415	+5.68091
H	+4.12649	−0.34883	+5.00514
H	+2.79118	+0.80147	+5.12610
O	+2.60274	−2.47107	+5.53957
H	+3.22719	−2.60268	+4.82258
O	+1.45913	−0.83636	+6.47332

19

∗ E = +8.997 kcal/mol ; (321) 012_204_313_302_139_310_182

H	+0.00000	+0.00000	+0.00000
O	+0.00000	+0.00000	+0.96297
C	+1.22747	+0.00000	+1.50009
C	+2.42888	+0.29138	+0.57728
N	+3.65897	−0.27346	+1.09702
H	+3.74007	−0.04857	+2.08300
O	+1.37458	−0.17919	+2.67214
C	+2.21210	−0.14128	−0.86969
H	+2.53258	+1.38580	+0.59011
H	+3.62530	−1.28714	+1.04837
C	+1.89843	−1.64597	−1.00719
H	+1.41356	+0.45678	−1.32072
H	+3.12043	+0.09981	−1.42680
C	+0.81338	−1.93401	−2.01168
H	+2.77483	−2.23029	−1.28797
H	+1.52976	−2.04598	−0.05055
O	−0.27219	−1.15463	−1.78932
H	−0.95714	−1.39377	−2.42386
O	+0.84710	−2.74323	−2.88781

19

∗ E = +8.997 kcal/mol ; (322) 182_323_058_061_174_177_359

H	+0.00000	+0.00000	+0.00000

O	+0.00000	+0.00000	+0.96388
C	+1.27229	+0.00000	+1.38305
C	+1.38367	−0.05780	+2.89276
N	+0.32564	+0.72300	+3.50720
H	−0.58509	+0.38279	+3.22075
O	+2.20463	−0.02929	+0.62935
C	+1.41742	−1.53917	+3.31455
H	+2.34928	+0.40098	+3.13438
H	+0.37960	+0.66022	+4.51624
C	+2.60193	−2.30535	+2.74440
H	+1.45459	−1.58256	+4.40818
H	+0.47870	−2.02440	+3.02167
C	+2.67313	−3.71561	+3.29173
H	+2.56156	−2.35298	+1.64906
H	+3.54474	−1.79483	+2.98439
O	+3.66410	−4.47560	+2.78656
H	+4.17054	−3.99102	+2.13090
O	+1.92995	−4.15801	+4.11239

19

∗ E = +9.036 kcal/mol ; (323) 352_148_026_307_298_109_346

H	+0.00000	+0.00000	+0.00000
O	+0.00000	+0.00000	+0.96056
C	+1.24319	+0.00000	+1.44197
C	+2.36352	−0.19609	+0.43107
N	+3.53686	+0.52252	+0.87229
H	+3.55033	+0.63626	+1.87825
O	+1.43417	+0.06608	+2.62514
C	+2.56354	−1.72166	+0.25715
H	+2.03169	+0.20632	−0.53924
H	+4.39435	+0.06426	+0.59075
C	+2.76740	−2.48412	+1.57078
H	+1.70666	−2.14928	−0.27637
H	+3.44362	−1.86621	−0.37999
C	+4.02122	−2.01979	+2.29828
H	+1.88306	−2.40918	+2.21124
H	+2.92341	−3.54081	+1.34017
O	+3.84362	−1.31218	+3.41916
H	+2.92754	−1.01367	+3.49747
O	+5.11822	−2.27321	+1.89319

19

∗ E = +9.102 kcal/mol ; (324) 177_158_055_186_310_289_357

H	+0.00000	+0.00000	+0.00000
O	+0.00000	+0.00000	+0.96405
C	+1.27669	+0.00000	+1.37857
C	+1.38122	+0.07928	+2.88708
N	+2.66702	−0.42546	+3.29268
H	+3.42904	+0.05527	+2.83183
O	+2.20786	+0.00777	+0.62796
C	+1.06550	+1.54721	+3.27115
H	+0.60088	−0.56451	+3.31447
H	+2.78178	−0.42053	+4.29781
C	+1.28808	+1.80564	+4.76711
H	+1.71337	+2.22057	+2.69778
H	+0.03122	+1.77242	+2.98156
C	+0.59155	+0.74782	+5.59649
H	+2.35092	+1.75560	+5.00940
H	+0.92686	+2.80367	+5.03847
O	−0.75277	+0.81494	+5.62032
H	−1.07237	+1.57535	+5.12934
O	+1.16139	−0.13897	+6.15725

19

∗ E = +9.134 kcal/mol ; (325) 176_148_291_057_055_038_359

H	+0.00000	+0.00000	+0.00000
O	+0.00000	+0.00000	+0.96468
C	+1.26062	+0.00000	+1.40543
C	+1.33682	+0.08913	+2.92532
N	+2.48141	−0.60551	+3.47634
H	+2.38308	−1.60807	+3.37316
O	+2.21872	−0.02965	+0.68578
C	+1.42717	+1.57324	+3.31525
H	+0.41540	−0.34196	+3.33493
H	+3.31646	−0.33836	+2.96376
C	+0.28515	+2.44979	+2.80046
H	+1.51823	+1.61236	+4.40842
H	+2.37626	+1.96584	+2.93122
C	−1.11458	+1.99189	+3.17689
H	+0.40352	+3.46719	+3.19193
H	+0.29718	+2.53143	+1.71148
O	−1.25478	+1.47435	+4.41537
H	−0.41817	+1.46144	+4.88615

O −2.05893 +2.09825 +2.45941
19
∗ E = +9.146 kcal/mol ; (326) 358_151_033_296_076_023_183
H +0.00000 +0.00000 +0.00000
O +0.00000 +0.00000 +0.95981
C +1.24820 +0.00000 +1.44282
C +2.37136 −0.05788 +0.41257
N +3.52061 +0.61686 +0.97242
H +3.54528 +0.48868 +1.97828
O +1.45363 +0.00432 +2.61927
C +2.62228 −1.52848 +0.00331
H +2.03787 +0.48079 −0.48772
H +4.38863 +0.29027 +0.56904
C +3.11152 −2.42100 +1.14180
H +1.71894 −1.95489 −0.44747
H +3.38676 −1.50950 −0.78180
C +2.07190 −2.86312 +2.14715
H +3.53439 −3.34418 +0.72921
H +3.91510 −1.94589 +1.71145
O +0.82487 −2.83396 +1.64605
H +0.21634 −3.08513 +2.35047
O +2.31821 −3.24467 +3.25244
19
∗ E = +9.221 kcal/mol ; (327) 357_016_093_168_173_053_359
H +0.00000 +0.00000 +0.00000
O +0.00000 +0.00000 +0.97163
C +1.26708 +0.00000 +1.36280
C +2.29099 −0.08518 +0.21672
N +1.65101 +0.22098 −1.05864
H +1.74119 +1.20220 −1.29762
O +1.59783 +0.03102 +2.51159
C +3.51380 +0.76854 +0.55157
H +2.59338 −1.14133 +0.19272
H +2.02480 −0.32246 −1.82367
C +4.67885 +0.48498 −0.39601
H +3.23024 +1.82836 +0.49803
H +3.77377 +0.56989 +1.59723
C +5.85191 +1.43000 −0.21778
H +5.03787 −0.54193 −0.24387
H +4.36976 +0.57489 −1.43998

O +6.32992 +1.53838 +1.03570
H +5.83061 +0.99970 +1.65378
O +6.34662 +2.04868 −1.10845
19
∗ E = +9.255 kcal/mol ; (328) 354_164_026_304_186_306_181
H +0.00000 +0.00000 +0.00000
O +0.00000 +0.00000 +0.96041
C +1.25097 +0.00000 +1.44619
C +2.35731 −0.13767 +0.40648
N +3.60793 +0.25579 +1.00927
H +3.57611 +0.12032 +2.01338
O +1.45141 +0.03863 +2.62133
C +2.32599 −1.58237 −0.13773
H +2.11942 +0.53934 −0.42952
H +4.39714 −0.24235 +0.62106
C +2.45940 −2.62448 +0.97472
H +1.40317 −1.76282 −0.70131
H +3.15096 −1.68588 −0.85312
C +2.57731 −4.02190 +0.42779
H +3.33704 −2.43444 +1.59548
H +1.57600 −2.59182 +1.62507
O +1.56051 −4.31937 −0.40261
H +1.68614 −5.22405 −0.71046
O +3.45220 −4.80023 +0.67493
19
∗ E = +9.273 kcal/mol ; (329) 354_164_024_306_191_060_180
H +0.00000 +0.00000 +0.00000
O +0.00000 +0.00000 +0.96038
C +1.25046 +0.00000 +1.44653
C +2.35749 −0.14176 +0.40811
N +3.60785 +0.25288 +1.00930
H +3.56729 +0.15039 +2.01686
O +1.45173 +0.04544 +2.62127
C +2.33670 −1.59035 −0.12706
H +2.11970 +0.52951 −0.43257
H +4.39462 −0.26335 +0.64044
C +2.42375 −2.62274 +1.00006
H +1.43082 −1.76507 −0.72131
H +3.18675 −1.70649 −0.80814
C +2.67963 −4.01004 +0.47591

H +3.24886 −2.35485 +1.67136
H +1.50436 −2.65228 +1.58917
O +3.83665 −4.07674 −0.21084
H +3.94615 −4.98433 −0.51598
O +1.96832 −4.96041 +0.62627
19
* E = +9.283 kcal/mol ; (330) 004_191_201_111_063_182_180
H +0.00000 +0.00000 +0.00000
O +0.00000 +0.00000 +0.95984
C +1.25561 +0.00000 +1.45038
C +2.36624 +0.08510 +0.41008
N +3.62865 −0.18084 +1.05518
H +4.38841 +0.19931 +0.50502
O +1.44424 −0.01291 +2.62638
C +2.15252 −0.87195 −0.78987
H +2.28970 +1.12374 +0.02878
H +3.64308 +0.20354 +1.99190
C +1.83451 −0.14726 −2.09140
H +3.08337 −1.43102 −0.90999
H +1.37511 −1.61306 −0.57285
C +2.98667 +0.73068 −2.51635
H +1.63104 −0.84680 −2.90926
H +0.94463 +0.49403 −2.02517
O +2.71962 +1.40815 −3.63892
H +3.49142 +1.94202 −3.86037
O +4.03081 +0.82411 −1.93265
19
* E = +9.290 kcal/mol ; (331) 182_338_287_290_179_181_000
H +0.00000 +0.00000 +0.00000
O +0.00000 +0.00000 +0.96438
C +1.28361 +0.00000 +1.37157
C +1.43090 −0.03879 +2.89010
N +0.28311 +0.42184 +3.64870
H +0.18696 +1.42749 +3.59244
O +2.20014 −0.01210 +0.60211
C +1.82132 −1.46786 +3.29412
H +2.28422 +0.60844 +3.11387
H −0.57599 +0.02619 +3.28579
C +0.68519 −2.46138 +3.09999
H +2.69352 −1.78709 +2.71635

H +2.11245 −1.45812 +4.34747
C +1.10167 −3.86766 +3.48266
H −0.17976 −2.18661 +3.71862
H +0.33711 −2.47794 +2.05732
O +0.14901 −4.80918 +3.32630
H −0.66258 −4.42623 +2.98699
O +2.17917 −4.16117 +3.89655
19
∗ E = +9.373 kcal/mol ; (332) 182_197_201_079_170_175_359
H +0.00000 +0.00000 +0.00000
O +0.00000 +0.00000 +0.96435
C +1.28032 +0.00000 +1.37026
C +1.42517 −0.04419 +2.88062
N +2.77614 +0.36974 +3.19591
H +3.05720 +0.05733 +4.11468
O +2.19596 −0.00139 +0.59966
C +0.41766 +0.84194 +3.62462
H +1.21344 −1.10039 +3.14282
H +3.43253 +0.02764 +2.50498
C −0.97770 +0.24949 +3.75528
H +0.81487 +1.01524 +4.62978
H +0.37257 +1.82871 +3.15315
C −1.84363 +1.06285 +4.69582
H −1.47978 +0.17891 +2.78454
H −0.92711 −0.77421 +4.15342
O −3.12660 +0.65603 +4.78408
H −3.29093 −0.08988 +4.20337
O −1.46099 +1.99277 +5.33514
19
∗ E = +9.388 kcal/mol ; (333) 357_148_295_058_173_047_179
H +0.00000 +0.00000 +0.00000
O +0.00000 +0.00000 +0.96009
C +1.25548 +0.00000 +1.44531
C +2.38995 −0.08179 +0.41633
N +3.57907 +0.62369 +0.84290
H +3.40718 +1.61976 +0.90953
O +1.45394 +0.02506 +2.62097
C +2.74354 −1.55786 +0.19523
H +2.04983 +0.35049 −0.53639
H +3.83039 +0.31069 +1.77588

C	+1.56492	−2.40440	−0.28553
H	+3.56206	−1.58854	−0.53037
H	+3.13160	−1.96382	+1.13617
C	+1.96525	−3.81218	−0.64624
H	+0.79945	−2.47843	+0.49771
H	+1.09357	−1.97656	−1.17603
O	+2.74195	−4.36966	+0.29600
H	+2.95117	−5.26786	+0.01565
O	+1.63687	−4.39222	−1.64072

19

* E = +9.395 kcal/mol ; (334) 185_356_055_278_051_055_351

H	+0.00000	+0.00000	+0.00000
O	+0.00000	+0.00000	+0.96468
C	+1.25998	+0.00000	+1.38168
C	+1.41603	−0.12436	+2.89241
N	+0.13744	−0.12334	+3.56816
H	−0.48832	−0.83633	+3.20850
O	+2.20413	+0.05212	+0.63855
C	+2.34593	−1.32605	+3.18747
H	+1.96318	+0.78267	+3.17974
H	+0.24570	−0.25451	+4.56524
C	+1.62372	−2.67804	+3.16309
H	+3.18473	−1.29508	+2.48156
H	+2.78077	−1.19678	+4.18365
C	+0.78639	−2.93371	+1.92272
H	+2.35809	−3.48723	+3.24912
H	+0.94021	−2.76618	+4.00946
O	+1.41680	−2.82222	+0.74031
H	+2.30370	−2.46037	+0.83099
O	−0.37083	−3.22688	+1.96023

19

* E = +9.530 kcal/mol ; (335) 177_161_034_173_181_179_000

H	+0.00000	+0.00000	+0.00000
O	+0.00000	+0.00000	+0.96391
C	+1.27168	+0.00000	+1.38141
C	+1.37255	+0.07978	+2.89095
N	+2.69458	−0.36741	+3.27603
H	+3.37038	−0.10383	+2.56734
O	+2.20758	−0.01966	+0.63465
C	+1.00110	+1.51503	+3.30847

H +0.61222 −0.60225 +3.29836
H +2.98675 +0.00728 +4.16733
C +0.90276 +1.66067 +4.81884
H +1.74865 +2.21445 +2.91755
H +0.04041 +1.79416 +2.86401
C +0.50781 +3.06794 +5.22467
H +0.16150 +0.96030 +5.22775
H +1.85587 +1.42829 +5.31313
O +0.37549 +3.26661 +6.55092
H +0.55055 +2.46153 +7.04271
O +0.32104 +3.95930 +4.45721
19
* E = +9.549 kcal/mol ; (336) 185_314_053_297_083_195_003
H +0.00000 +0.00000 +0.00000
O +0.00000 +0.00000 +0.96461
C +1.28522 +0.00000 +1.37817
C +1.37967 −0.12182 +2.88792
N +0.43659 +0.80149 +3.49904
H −0.50048 +0.68150 +3.13651
O +2.20913 +0.06399 +0.62748
C +1.23580 −1.59814 +3.32107
H +2.39454 +0.19883 +3.13955
H +0.41760 +0.69832 +4.50546
C −0.11262 −2.22614 +2.98590
H +2.02937 −2.19266 +2.85656
H +1.39145 −1.62623 +4.40489
C −0.17913 −2.73139 +1.55561
H −0.30417 −3.09041 +3.63370
H −0.93374 −1.51893 +3.16165
O −1.41493 −2.99795 +1.09415
H −2.08235 −2.77069 +1.74464
O +0.77617 −2.91900 +0.86744
19
* E = +9.647 kcal/mol ; (337) 182_061_296_179_070_045_000
H +0.00000 +0.00000 +0.00000
O +0.00000 +0.00000 +0.96392
C +1.27338 +0.00000 +1.38626
C +1.35530 −0.05021 +2.90710
N +0.74735 −1.24749 +3.46140
H −0.24605 −1.29056 +3.27595

```
 O     +2.20776     +0.00845     +0.63594
 C     +2.80381     +0.07411     +3.36169
 H     +0.78431     +0.80716     +3.28827
 H     +1.17900     −2.09367     +3.10690
 C     +2.93311     +0.04334     +4.88452
 H     +3.39623     −0.71344     +2.87380
 H     +3.22023     +1.00850     +2.97463
 C     +2.64528     −1.28558     +5.56217
 H     +3.95517     +0.32226     +5.17016
 H     +2.26247     +0.77342     +5.34425
 O     +3.19927     −2.37343     +4.97843
 H     +3.70917     −2.12610     +4.20456
 O     +2.04144     −1.39509     +6.58200
19
 * E =   +9.658  kcal/mol ;  (338)  014_088_303_079_298_076_186
 H     +0.00000     +0.00000     +0.00000
 O     +0.00000     +0.00000     +0.95980
 C     +1.25958     +0.00000     +1.43464
 C     +2.35676     +0.33913     +0.42016
 N     +2.54763     +1.78915     +0.38547
 H     +1.68665     +2.27641     +0.16487
 O     +1.47018     −0.15380     +2.59960
 C     +3.67753     −0.32571     +0.79280
 H     +2.04835     +0.01008     −0.58300
 H     +2.84983     +2.11337     +1.29888
 C     +3.78189     −1.81224     +0.42575
 H     +4.46957     +0.21502     +0.26744
 H     +3.83738     −0.19493     +1.86922
 C     +2.76306     −2.66904     +1.13125
 H     +3.64495     −1.93353     −0.65455
 H     +4.76779     −2.19041     +0.69875
 O     +1.53729     −2.53204     +0.57652
 H     +0.91007     −3.02693     +1.11770
 O     +2.96743     −3.38541     +2.06423
19
 * E =   +9.719  kcal/mol ;  (339)  179_169_030_302_184_286_357
 H     +0.00000     +0.00000     +0.00000
 O     +0.00000     +0.00000     +0.96410
 C     +1.27568     +0.00000     +1.38006
 C     +1.37446     +0.01580     +2.89101
```

N	+2.74251	−0.25410	+3.26606
H	+3.38939	+0.08535	+2.56446
O	+2.20559	+0.04089	+0.62748
C	+0.80317	+1.35750	+3.39617
H	+0.71622	−0.78114	+3.26226
H	+2.97758	+0.13664	+4.16807
C	+1.53133	+2.56545	+2.79617
H	−0.26867	+1.39527	+3.16940
H	+0.89963	+1.37769	+4.48771
C	+1.00485	+3.85357	+3.39239
H	+2.60031	+2.51366	+3.01198
H	+1.40897	+2.58298	+1.70443
O	−0.21574	+4.23127	+2.95772
H	−0.54768	+3.62848	+2.28879
O	+1.57697	+4.49654	+4.21625

19

∗ E = +9.733 kcal/mol ; (340) 180_053_187_074_068_082_004

H	+0.00000	+0.00000	+0.00000
O	+0.00000	+0.00000	+0.96385
C	+1.27353	+0.00000	+1.38326
C	+1.35586	+0.00118	+2.90020
N	+0.54750	−1.10819	+3.39856
H	+0.49135	−1.07131	+4.40892
O	+2.20426	+0.00888	+0.62997
C	+2.80421	−0.16118	+3.35114
H	+0.97999	+0.99070	+3.21564
H	−0.39699	−1.05206	+3.03674
C	+3.64168	+1.11141	+3.14668
H	+2.80212	−0.44503	+4.41140
H	+3.25311	−0.99001	+2.79717
C	+3.16609	+2.20412	+4.07932
H	+4.69990	+0.89197	+3.32599
H	+3.53928	+1.46468	+2.11943
O	+3.65107	+2.14727	+5.33837
H	+4.29320	+1.44015	+5.42749
O	+2.37191	+3.04449	+3.78291

19

∗ E = +9.735 kcal/mol ; (341) 352_320_296_310_296_258_359

H	+0.00000	+0.00000	+0.00000
O	+0.00000	+0.00000	+0.97048

C +1.23289 +0.00000 +1.47952
C +2.40170 −0.19308 +0.49782
N +2.22300 −1.15361 −0.57464
H +2.10927 −2.09229 −0.21685
O +1.42184 +0.17181 +2.64916
C +2.85712 +1.18374 −0.02334
H +3.21595 −0.56203 +1.12821
H +1.45270 −0.94874 −1.19631
C +1.75922 +2.11626 −0.57063
H +3.33183 +1.71413 +0.80713
H +3.62260 +1.00452 −0.78574
C +1.06843 +1.56839 −1.79956
H +0.98721 +2.27080 +0.19093
H +2.20034 +3.09480 −0.78648
O +1.42159 +2.07407 −2.98042
H +2.08633 +2.75946 −2.88552
O +0.23841 +0.69721 −1.75482
19
∗ E = +9.735 kcal/mol ; (342) 183_328_066_183_310_288_358
H +0.00000 +0.00000 +0.00000
O +0.00000 +0.00000 +0.96384
C +1.27441 +0.00000 +1.37743
C +1.39308 −0.06114 +2.88730
N +0.29558 +0.61853 +3.53710
H −0.59184 +0.15830 +3.37739
O +2.20495 −0.03019 +0.62380
C +1.57463 −1.55552 +3.24590
H +2.31903 +0.47052 +3.13687
H +0.46299 +0.71670 +4.53126
C +1.64129 −1.77117 +4.76349
H +0.73390 −2.13356 +2.84388
H +2.48208 −1.92398 +2.75109
C +2.63875 −0.81926 +5.38960
H +0.67236 −1.56521 +5.22148
H +1.90889 −2.80910 +4.98942
O +3.93433 −1.08124 +5.14135
H +4.03612 −1.87831 +4.61610
O +2.32857 +0.14647 +6.02046
19
∗ E = +9.750 kcal/mol ; (343) 182_064_295_170_172_289_357

H	+0.00000	+0.00000	+0.00000
O	+0.00000	+0.00000	+0.96395
C	+1.27547	+0.00000	+1.38518
C	+1.35565	−0.05360	+2.90510
N	+0.79343	−1.29494	+3.42885
H	−0.20110	−1.35260	+3.24755
O	+2.20831	+0.00327	+0.63500
C	+2.79506	+0.11345	+3.37223
H	+0.74314	+0.77155	+3.29153
H	+1.23143	−2.09739	+2.98903
C	+2.85434	+0.31873	+4.88890
H	+3.36368	−0.77572	+3.06708
H	+3.25961	+0.95703	+2.85385
C	+4.27628	+0.28300	+5.40355
H	+2.43335	+1.28994	+5.15724
H	+2.25999	−0.46130	+5.38027
O	+4.85823	−0.93599	+5.38820
H	+4.23957	−1.60725	+5.09206
O	+4.88509	+1.23438	+5.78384

19

∗ E = +9.756 kcal/mol ; (344) 354_162_014_298_244_046_178

H	+0.00000	+0.00000	+0.00000
O	+0.00000	+0.00000	+0.96049
C	+1.25231	+0.00000	+1.44237
C	+2.35707	−0.13677	+0.39991
N	+3.61289	+0.28833	+0.96610
H	+3.54021	+0.36073	+1.97426
O	+1.45589	+0.05701	+2.61731
C	+2.37363	−1.59550	−0.10511
H	+2.08352	+0.50646	−0.45341
H	+4.36993	−0.33962	+0.73075
C	+2.68750	−2.60944	+1.01236
H	+1.41201	−1.84040	−0.56912
H	+3.12366	−1.65399	−0.90006
C	+3.97595	−3.36310	+0.81664
H	+2.77939	−2.08353	+1.97336
H	+1.89898	−3.35276	+1.12854
O	+4.99181	−2.54833	+0.45789
H	+5.78683	−3.08688	+0.37365
O	+4.13027	−4.53900	+0.96948

19
∗ E = +9.816 kcal/mol ; (345) 176_154_054_177_069_060_000

H	+0.00000	+0.00000	+0.00000
O	+0.00000	+0.00000	+0.96418
C	+1.27459	+0.00000	+1.38030
C	+1.38442	+0.08673	+2.88977
N	+2.63320	−0.51306	+3.28562
H	+3.42201	−0.10859	+2.79699
O	+2.20830	+0.00230	+0.63213
C	+1.17930	+1.57684	+3.25118
H	+0.55617	−0.48543	+3.32570
H	+2.78880	−0.50763	+4.28371
C	+1.19342	+1.83019	+4.76144
H	+1.95816	+2.16298	+2.74186
H	+0.21877	+1.91289	+2.84548
C	+2.55014	+1.64108	+5.41264
H	+0.87046	+2.85891	+4.96352
H	+0.49627	+1.16027	+5.27034
O	+3.53423	+2.43286	+4.94724
H	+3.21856	+3.01186	+4.24975
O	+2.77220	+0.85936	+6.28746

19
∗ E = +9.817 kcal/mol ; (346) 177_158_056_180_087_189_001

H	+0.00000	+0.00000	+0.00000
O	+0.00000	+0.00000	+0.96394
C	+1.27862	+0.00000	+1.37610
C	+1.38450	+0.08356	+2.88536
N	+2.67613	−0.43113	+3.27810
H	+3.41591	+0.07709	+2.80499
O	+2.20671	−0.00601	+0.62276
C	+1.06956	+1.54557	+3.27603
H	+0.60075	−0.55939	+3.30783
H	+2.82861	−0.34347	+4.27417
C	+1.12981	+1.77215	+4.78201
H	+1.79269	+2.21067	+2.78990
H	+0.07228	+1.79907	+2.90326
C	+2.52402	+2.10268	+5.28789
H	+0.48944	+2.61457	+5.07220
H	+0.74819	+0.89721	+5.32782
O	+2.65195	+2.11923	+6.62902

H	+1.82350	+1.89904	+7.06042
O	+3.45668	+2.35645	+4.59112

19

∗ E = +9.827 kcal/mol ; (347) 178_162_038_293_290_307_357

H	+0.00000	+0.00000	+0.00000
O	+0.00000	+0.00000	+0.96399
C	+1.27566	+0.00000	+1.37914
C	+1.37531	+0.05865	+2.89060
N	+2.70175	−0.35906	+3.27472
H	+3.40149	−0.01450	+2.62653
O	+2.20730	+0.00617	+0.62893
C	+0.96272	+1.48114	+3.33625
H	+0.62759	−0.64092	+3.28577
H	+2.94525	−0.08200	+4.21632
C	+1.95286	+2.55931	+2.88102
H	−0.03303	+1.70593	+2.94054
H	+0.85915	+1.47127	+4.42941
C	+3.29293	+2.53105	+3.59355
H	+2.16557	+2.47356	+1.81174
H	+1.51727	+3.55183	+3.04990
O	+3.23274	+2.55090	+4.94503
H	+2.32700	+2.62924	+5.25207
O	+4.35015	+2.51344	+3.04535

19

∗ E = +9.838 kcal/mol ; (348) 357_148_295_059_175_305_180

H	+0.00000	+0.00000	+0.00000
O	+0.00000	+0.00000	+0.95995
C	+1.25526	+0.00000	+1.44496
C	+2.39050	−0.07611	+0.41629
N	+3.57669	+0.63162	+0.84853
H	+3.39865	+1.62609	+0.92379
O	+1.45446	+0.02453	+2.62044
C	+2.75112	−1.54879	+0.18441
H	+2.05100	+0.35912	−0.53555
H	+3.82782	+0.31343	+1.77992
C	+1.58153	−2.39857	−0.31622
H	+3.57408	−1.57462	−0.53396
H	+3.12861	−1.95673	+1.13062
C	+2.00104	−3.80655	−0.65853
H	+0.77835	−2.47392	+0.42028

H +1.17001 −1.95498 −1.23404
O +3.01685 −3.82570 −1.53879
H +3.23586 −4.74712 −1.71770
O +1.50536 −4.80498 −0.22423
19
∗ E = +9.854 kcal/mol ; (349) 185_086_072_060_172_173_358
H +0.00000 +0.00000 +0.00000
O +0.00000 +0.00000 +0.96420
C +1.26828 +0.00000 +1.38057
C +1.39241 −0.11517 +2.89375
N +1.36255 −1.54494 +3.18323
H +2.23321 −1.97778 +2.89450
O +2.20405 +0.05921 +0.63143
C +2.64230 +0.60745 +3.38707
H +0.49137 +0.33134 +3.33122
H +1.25236 −1.70743 +4.17597
C +2.64529 +2.09147 +3.05153
H +2.72530 +0.47833 +4.47082
H +3.52499 +0.13526 +2.94165
C +3.79567 +2.81970 +3.71767
H +2.71153 +2.24107 +1.96610
H +1.71349 +2.57191 +3.38317
O +3.95035 +4.10631 +3.34793
H +3.31177 +4.35079 +2.67482
O +4.52308 +2.33200 +4.52583
19
∗ E = +9.858 kcal/mol ; (350) 179_040_189_291_292_315_359
H +0.00000 +0.00000 +0.00000
O +0.00000 +0.00000 +0.96394
C +1.27195 +0.00000 +1.38315
C +1.37232 +0.02129 +2.90124
N +0.35871 −0.84361 +3.47401
H +0.30337 −0.74758 +4.47973
O +2.20438 +0.02914 +0.63289
C +2.76860 −0.42283 +3.33392
H +1.24893 +1.08826 +3.16662
H −0.55621 −0.67866 +3.07633
C +3.03387 −1.90591 +3.06183
H +3.50649 +0.17482 +2.79227
H +2.89867 −0.17383 +4.39639

C	+2.24551	−2.90003	+3.89524
H	+2.83771	−2.14254	+2.01405
H	+4.09509	−2.12011	+3.24108
O	+2.18387	−2.63250	+5.22295
H	+2.68161	−1.84084	+5.43491
O	+1.76382	−3.89994	+3.46614

19

∗ E = +9.921 kcal/mol ; (351) 354_247_307_064_221_054_178

H	+0.00000	+0.00000	+0.00000
O	+0.00000	+0.00000	+0.96404
C	+1.21871	+0.00000	+1.51889
C	+2.46217	−0.13282	+0.61695
N	+3.12725	−1.40519	+0.86268
H	+3.30908	−1.51061	+1.85471
O	+1.34209	+0.04682	+2.70714
C	+2.20637	+0.03547	−0.87417
H	+3.14065	+0.66011	+0.95579
H	+2.53469	−2.17960	+0.58197
C	+1.72051	+1.45149	−1.24468
H	+3.13831	−0.19023	−1.39714
H	+1.48652	−0.72401	−1.20790
C	+0.62622	+1.44941	−2.27797
H	+1.30214	+1.95202	−0.36006
H	+2.53308	+2.07550	−1.61598
O	−0.39672	+0.64965	−1.88629
H	−1.09637	+0.68961	−2.54791
O	+0.60122	+2.05796	−3.30326

19

∗ E = +9.922 kcal/mol ; (352) 182_319_074_179_085_190_001

H	+0.00000	+0.00000	+0.00000
O	+0.00000	+0.00000	+0.96364
C	+1.27164	+0.00000	+1.38183
C	+1.37125	−0.05097	+2.89472
N	+0.37288	+0.81277	+3.49654
H	−0.55182	+0.40167	+3.42725
O	+2.20719	−0.02423	+0.63382
C	+1.31283	−1.53923	+3.29766
H	+2.36407	+0.34240	+3.13933
H	+0.56643	+0.97171	+4.47690
C	+1.43097	−1.73514	+4.80358

H +0.36151 −1.96600 +2.95890
H +2.12126 −2.07181 +2.78674
C +0.10404 −1.60460 +5.53166
H +1.81295 −2.73734 +5.03499
H +2.15040 −1.02561 +5.23700
O +0.19481 −1.55246 +6.87412
H +1.10908 −1.59503 +7.16269
O −0.96273 −1.57378 +5.00003
19
* E = +9.928 kcal/mol ; (353) 178_150_294_059_173_294_359
H +0.00000 +0.00000 +0.00000
O +0.00000 +0.00000 +0.96492
C +1.27300 +0.00000 +1.38667
C +1.38710 +0.05659 +2.90594
N +2.56871 −0.61704 +3.40179
H +2.51757 −1.61433 +3.23299
O +2.20991 −0.02124 +0.64146
C +1.43264 +1.52467 +3.35106
H +0.49296 −0.41588 +3.32932
H +3.38491 −0.27848 +2.90156
C +0.21094 +2.34457 +2.92512
H +1.54789 +1.51054 +4.44063
H +2.34422 +1.98284 +2.94758
C +0.23033 +3.73545 +3.52735
H +0.17268 +2.46810 +1.84200
H −0.71046 +1.83098 +3.23140
O +0.10556 +3.78606 +4.87016
H +0.00375 +2.90845 +5.24463
O +0.35485 +4.73884 +2.89731
19
* E = +9.938 kcal/mol ; (354) 179_009_301_167_176_063_001
H +0.00000 +0.00000 +0.00000
O +0.00000 +0.00000 +0.96441
C +1.27560 +0.00000 +1.37338
C +1.42181 +0.03392 +2.89541
N +0.21131 −0.15151 +3.66473
H −0.48292 +0.54970 +3.44084
O +2.20102 −0.00454 +0.61189
C +2.48508 −0.98615 +3.31063
H +1.81350 +1.03854 +3.11365

H −0.20421 −1.05567 +3.47030
C +2.90429 −0.79309 +4.76936
H +2.07762 −1.99695 +3.17486
H +3.33329 −0.89448 +2.62402
C +3.88169 −1.85606 +5.22561
H +3.36979 +0.19438 +4.89097
H +2.03177 −0.83549 +5.42156
O +5.06692 −1.86756 +4.57843
H +5.10989 −1.17263 +3.91802
O +3.65996 −2.65486 +6.08171
19
* E = +10.032 kcal/mol ; (355) 180_324_072_326_294_166_357
H +0.00000 +0.00000 +0.00000
O +0.00000 +0.00000 +0.96401
C +1.27400 +0.00000 +1.38374
C +1.38024 −0.01099 +2.89575
N +0.32210 +0.78485 +3.48732
H −0.57440 +0.32066 +3.39273
O +2.20319 −0.07559 +0.63002
C +1.49672 −1.49551 +3.35603
H +2.33350 +0.47565 +3.12077
H +0.49366 +0.90033 +4.47815
C +0.72156 −2.51772 +2.52734
H +2.55164 −1.78448 +3.33165
H +1.16093 −1.54383 +4.39671
C −0.78476 −2.36868 +2.61123
H +1.03627 −2.48559 +1.47537
H +0.95497 −3.53097 +2.87934
O −1.48153 −3.04086 +1.67925
H −0.89562 −3.48518 +1.06296
O −1.35473 −1.74414 +3.45449
19
* E = +10.052 kcal/mol ; (356) 181_332_051_301_192_188_002
H +0.00000 +0.00000 +0.00000
O +0.00000 +0.00000 +0.96416
C +1.28014 +0.00000 +1.37471
C +1.41739 −0.02958 +2.88418
N +0.26561 +0.57697 +3.52499
H −0.60808 +0.20073 +3.18050
O +2.20105 −0.04717 +0.61195

C +1.73103 −1.48139 +3.30121
H +2.30202 +0.57539 +3.10746
H +0.30243 +0.45640 +4.52891
C +0.65475 −2.47030 +2.87861
H +2.69441 −1.78099 +2.87649
H +1.84218 −1.51456 +4.38983
C +0.83797 −3.82382 +3.53653
H −0.34470 −2.10304 +3.15189
H +0.64593 −2.60517 +1.78864
O +0.01744 −4.79425 +3.08854
H −0.55221 −4.46994 +2.38787
O +1.62680 −4.05077 +4.40018
19
∗ E = +10.109 kcal/mol ; (357) 353_143_040_061_173_050_179
H +0.00000 +0.00000 +0.00000
O +0.00000 +0.00000 +0.95992
C +1.25248 +0.00000 +1.44232
C +2.37708 −0.16563 +0.42341
N +3.47783 +0.67104 +0.85114
H +3.57361 +0.60870 +1.85963
O +1.45794 +0.06573 +2.61513
C +2.72252 −1.66393 +0.33438
H +2.02608 +0.17489 −0.56359
H +4.34871 +0.38194 +0.42445
C +1.55012 −2.52776 −0.13497
H +3.56441 −1.77107 −0.36029
H +3.06742 −1.99796 +1.31862
C +1.94713 −3.95919 −0.38803
H +0.75957 −2.53722 +0.62613
H +1.11791 −2.15027 −1.06680
O +2.63127 −4.47745 +0.64462
H +2.84394 −5.39304 +0.43108
O +1.69496 −4.58580 −1.37653
19
∗ E = +10.112 kcal/mol ; (358) 182_322_288_176_069_047_358
H +0.00000 +0.00000 +0.00000
O +0.00000 +0.00000 +0.96393
C +1.27756 +0.00000 +1.38247
C +1.37828 −0.05518 +2.90097
N +0.39284 +0.71418 +3.62686

H +0.54873 +1.71041 +3.55551
O +2.20765 −0.00320 +0.62927
C +1.29668 −1.52861 +3.32398
H +2.37597 +0.31910 +3.15294
H −0.54909 +0.51488 +3.31056
C +1.49306 −1.69755 +4.83061
H +0.33185 −1.93738 +2.98684
H +2.06426 −2.09775 +2.78996
C +0.38450 −1.17088 +5.72695
H +1.60309 −2.76372 +5.06603
H +2.41197 −1.20157 +5.15205
O −0.87372 −1.51189 +5.36952
H −0.87706 −2.02071 +4.55683
O +0.57966 −0.57063 +6.73625
19
* E = +10.192 kcal/mol ; (359) 183_341_282_293_187_068_002
H +0.00000 +0.00000 +0.00000
O +0.00000 +0.00000 +0.96450
C +1.27796 +0.00000 +1.37775
C +1.41537 −0.06927 +2.89587
N +0.24161 +0.30635 +3.66173
H +0.13389 +1.31166 +3.69501
O +2.20482 +0.00691 +0.62097
C +1.86906 −1.49155 +3.26073
H +2.23508 +0.61084 +3.14659
H −0.60208 −0.06251 +3.23914
C +0.78667 −2.53519 +2.96361
H +2.78155 −1.73490 +2.70755
H +2.11468 −1.48264 +4.32809
C +1.18011 −3.90618 +3.47308
H −0.15463 −2.24203 +3.44645
H +0.60972 −2.62361 +1.88913
O +1.23510 −4.01901 +4.81677
H +0.97984 −3.19806 +5.24447
O +1.44311 −4.83703 +2.77778
19
* E = +10.239 kcal/mol ; (360) 178_030_290_291_187_066_002
H +0.00000 +0.00000 +0.00000
O +0.00000 +0.00000 +0.96426
C +1.27785 +0.00000 +1.37469

C +1.40029 +0.05747 +2.89700
N +0.30672 −0.56190 +3.62778
H −0.54067 −0.01606 +3.53784
O +2.20213 −0.01872 +0.61387
C +2.75268 −0.50206 +3.33688
H +1.38882 +1.12910 +3.13786
H +0.09300 −1.48125 +3.25914
C +2.86095 −2.01255 +3.09977
H +3.55237 +0.00664 +2.79166
H +2.86687 −0.26714 +4.40042
C +4.13358 −2.58360 +3.69059
H +2.01145 −2.52803 +3.56716
H +2.86511 −2.23314 +2.03024
O +4.20303 −2.54030 +5.03892
H +3.40101 −2.17016 +5.41508
O +5.03362 −3.03739 +3.05624
19
∗ E = +10.256 kcal/mol ; (361) 001_352_303_183_306_079_356
H +0.00000 +0.00000 +0.00000
O +0.00000 +0.00000 +0.96050
C +1.25590 +0.00000 +1.43076
C +2.38908 +0.02406 +0.39611
N +2.01817 −0.13471 −1.00419
H +1.50998 −0.98400 −1.21371
O +1.48062 +0.00058 +2.60066
C +3.17122 +1.33026 +0.59884
H +3.04077 −0.81723 +0.66334
H +1.55904 +0.67273 −1.40794
C +4.31106 +1.54651 −0.40571
H +2.47776 +2.17824 +0.52639
H +3.55574 +1.32653 +1.62168
C +5.28208 +0.37798 −0.45868
H +3.90483 +1.71665 −1.40908
H +4.88050 +2.43074 −0.11658
O +4.85950 −0.67529 −1.17557
H +3.95540 −0.52200 −1.49093
O +6.34067 +0.36916 +0.09215
19
∗ E = +10.297 kcal/mol ; (362) 179_045_193_180_189_331_003
H +0.00000 +0.00000 +0.00000

O	+0.00000	+0.00000	+0.96398
C	+1.27373	+0.00000	+1.38359
C	+1.36968	+0.01508	+2.89933
N	+0.42811	−0.94558	+3.45673
H	+0.31799	−0.80657	+4.45315
O	+2.20585	+0.01742	+0.63435
C	+2.79795	−0.30727	+3.32305
H	+1.14535	+1.05834	+3.19308
H	−0.48486	−0.84499	+3.03066
C	+2.94383	−0.29866	+4.84201
H	+3.04062	−1.29523	+2.90818
H	+3.47340	+0.42033	+2.86070
C	+4.36595	−0.38662	+5.36780
H	+2.51557	+0.60979	+5.27710
H	+2.40226	−1.14782	+5.27767
O	+5.25317	−1.03535	+4.59285
H	+4.85786	−1.32161	+3.76617
O	+4.69880	+0.06353	+6.42044

19

∗ E = +10.319 kcal/mol ; (363) 182_308_297_054_178_291_358

H	+0.00000	+0.00000	+0.00000
O	+0.00000	+0.00000	+0.96395
C	+1.27378	+0.00000	+1.38969
C	+1.35448	−0.04166	+2.91038
N	+0.55663	+0.96944	+3.57889
H	+0.86888	+1.90461	+3.34944
O	+2.21091	+0.00401	+0.64438
C	+0.91642	−1.42702	+3.39207
H	+2.41015	+0.11438	+3.15658
H	−0.41700	+0.89193	+3.30650
C	+1.70644	−2.56090	+2.73032
H	+1.02746	−1.43809	+4.48204
H	−0.15258	−1.55247	+3.17991
C	+1.27951	−3.91282	+3.26391
H	+1.54322	−2.57547	+1.65031
H	+2.78190	−2.41888	+2.90019
O	+1.62441	−4.16192	+4.54478
H	+2.12799	−3.43505	+4.91735
O	+0.66178	−4.71798	+2.63996

19

∗ E = +10.350 kcal/mol ; (364) 353_144_039_062_173_306_180

H	+0.00000	+0.00000	+0.00000
O	+0.00000	+0.00000	+0.95984
C	+1.25215	+0.00000	+1.44235
C	+2.37712	−0.15631	+0.42210
N	+3.48408	+0.66139	+0.86793
H	+3.56179	+0.60046	+1.87796
O	+1.45778	+0.06430	+2.61516
C	+2.71471	−1.65381	+0.29645
H	+2.02910	+0.20600	−0.55885
H	+4.35714	+0.35917	+0.45533
C	+1.54962	−2.49959	−0.22245
H	+3.56655	−1.75071	−0.38484
H	+3.03668	−2.01281	+1.28129
C	+1.94529	−3.92930	−0.49301
H	+0.71480	−2.52407	+0.48151
H	+1.18772	−2.08132	−1.17253
O	+3.01066	−4.00766	−1.31093
H	+3.20811	−4.94093	−1.44902
O	+1.39590	−4.89840	−0.05755

19

∗ E = +10.393 kcal/mol ; (365) 181_314_069_175_068_058_001

H	+0.00000	+0.00000	+0.00000
O	+0.00000	+0.00000	+0.96376
C	+1.27559	+0.00000	+1.38308
C	+1.36126	−0.01992	+2.89783
N	+0.43226	+0.94058	+3.45657
H	−0.53351	+0.66677	+3.32282
O	+2.20788	−0.04288	+0.63473
C	+1.17622	−1.48584	+3.34303
H	+2.37952	+0.29535	+3.14730
H	+0.59897	+1.11516	+4.43851
C	+1.35217	−1.66868	+4.85319
H	+0.18345	−1.82467	+3.01236
H	+1.90734	−2.11334	+2.82140
C	+0.25999	−1.02936	+5.68936
H	+1.36987	−2.73856	+5.09476
H	+2.30169	−1.24155	+5.18453
O	−0.99204	−1.45787	+5.43467
H	−0.99866	−2.13117	+4.75055

O +0.45155 −0.20595 +6.53189
19
* E = +10.408 kcal/mol ; (366) 354_159_030_174_181_056_180
H +0.00000 +0.00000 +0.00000
O +0.00000 +0.00000 +0.96016
C +1.25123 +0.00000 +1.44605
C +2.36150 −0.13252 +0.40833
N +3.58491 +0.36298 +0.99183
H +3.57005 +0.21410 +1.99516
O +1.45324 +0.04993 +2.61976
C +2.39393 −1.60307 −0.05363
H +2.08930 +0.49030 −0.46139
H +4.40968 −0.06885 +0.60011
C +3.37564 −1.81313 −1.20761
H +2.67372 −2.22886 +0.80261
H +1.39541 −1.92202 −0.37539
C +3.39314 −3.24152 −1.68960
H +3.08664 −1.17747 −2.05395
H +4.40084 −1.56258 −0.92796
O +2.16700 −3.66011 −2.04986
H +2.24065 −4.57417 −2.34698
O +4.36007 −3.94272 −1.75816
19
* E = +10.420 kcal/mol ; (367) 354_158_034_174_181_298_179
H +0.00000 +0.00000 +0.00000
O +0.00000 +0.00000 +0.96012
C +1.25094 +0.00000 +1.44701
C +2.36258 −0.15119 +0.41371
N +3.57615 +0.38094 +0.98455
H +3.59308 +0.20130 +1.98273
O +1.45280 +0.05666 +2.62030
C +2.41295 −1.63940 +0.00988
H +2.08896 +0.43821 −0.47745
H +4.40990 +0.00231 +0.55815
C +3.39723 −1.88480 −1.13565
H +2.69869 −2.23233 +0.88581
H +1.41374 −1.97511 −0.29782
C +3.41555 −3.33423 −1.54692
H +3.13377 −1.29497 −2.01659
H +4.41396 −1.61570 −0.82671

O	+3.81333	−4.12948	−0.53993
H	+3.79494	−5.04084	−0.85335
O	+3.10848	−3.75740	−2.62368

19

∗ E = +10.468 kcal/mol ; (368) 181_314_069_182_292_175_000

H	+0.00000	+0.00000	+0.00000
O	+0.00000	+0.00000	+0.96350
C	+1.28075	+0.00000	+1.37837
C	+1.36909	−0.02183	+2.89223
N	+0.44778	+0.96208	+3.43423
H	−0.51938	+0.69819	+3.28986
O	+2.20524	−0.03308	+0.62160
C	+1.13403	−1.48135	+3.33906
H	+2.39040	+0.26937	+3.14858
H	+0.60932	+1.13659	+4.41611
C	+1.15316	−1.65538	+4.84995
H	+0.16200	−1.81159	+2.95334
H	+1.90677	−2.11517	+2.89099
C	+2.52440	−1.41741	+5.45382
H	+0.43143	−0.98209	+5.33179
H	+0.84539	−2.67289	+5.12131
O	+2.57349	−1.46576	+6.79958
H	+1.70834	−1.64921	+7.17191
O	+3.51204	−1.20814	+4.82070

19

∗ E = +10.477 kcal/mol ; (369) 358_177_192_289_281_143_346

H	+0.00000	+0.00000	+0.00000
O	+0.00000	+0.00000	+0.96035
C	+1.25242	+0.00000	+1.44861
C	+2.35946	−0.05673	+0.39963
N	+3.62823	+0.03105	+1.07964
H	+4.39868	+0.19543	+0.44531
O	+1.44708	+0.04022	+2.62430
C	+2.28838	−1.38706	−0.37537
H	+2.16045	+0.77225	−0.30485
H	+3.62001	+0.71595	+1.82342
C	+2.72420	−2.54543	+0.51746
H	+1.27928	−1.55699	−0.76574
H	+2.96405	−1.33981	−1.23543
C	+4.24051	−2.60048	+0.61320

H +2.26975 −2.46265 +1.51236
H +2.40396 −3.50020 +0.08640
O +4.75501 −2.95097 +1.79881
H +4.08507 −2.91384 +2.48478
O +4.95278 −2.41018 −0.32860
19
∗ E = +10.524 kcal/mol ; (370) 184_074_301_066_178_305_001
H +0.00000 +0.00000 +0.00000
O +0.00000 +0.00000 +0.96424
C +1.27439 +0.00000 +1.38558
C +1.35777 −0.10193 +2.90429
N +1.03577 −1.45699 +3.33945
H +0.10348 −1.72438 +3.04718
O +2.20846 +0.01298 +0.63602
C +2.74268 +0.28759 +3.39896
H +0.60302 +0.57229 +3.32814
H +1.69042 −2.12358 +2.94447
C +3.06032 +1.76272 +3.14377
H +2.77206 +0.03906 +4.46635
H +3.48980 −0.33975 +2.89835
C +4.41629 +2.19030 +3.67146
H +3.05621 +1.97737 +2.07380
H +2.29934 +2.39514 +3.62207
O +4.63288 +1.95050 +4.98234
H +3.87477 +1.52613 +5.38922
O +5.25712 +2.71746 +3.01295
19
∗ E = +10.537 kcal/mol ; (371) 180_011_046_296_295_300_357
H +0.00000 +0.00000 +0.00000
O +0.00000 +0.00000 +0.96436
C +1.26881 +0.00000 +1.37167
C +1.43254 −0.00624 +2.88534
N +0.17858 −0.27079 +3.55835
H −0.32972 −1.04747 +3.15431
O +2.19909 +0.00227 +0.61320
C +2.61268 −0.92936 +3.23916
H +1.74516 +1.02026 +3.12904
H +0.30902 −0.44353 +4.54631
C +2.36887 −2.39637 +2.86617
H +3.50300 −0.57645 +2.71157

H	+2.81520	−0.82183	+4.31324
C	+1.24797	−3.05096	+3.64834
H	+2.12694	−2.48666	+1.80433
H	+3.28371	−2.97574	+3.04061
O	+1.42080	−3.08097	+4.98769
H	+2.27078	−2.70965	+5.23467
O	+0.26490	−3.52140	+3.16298

19

∗ E = +10.568 kcal/mol ; (372) 018_081_185_080_309_263_172

H	+0.00000	+0.00000	+0.00000
O	+0.00000	+0.00000	+0.96016
C	+1.25657	+0.00000	+1.43532
C	+2.32883	+0.42604	+0.42392
N	+2.30356	+1.88659	+0.39661
H	+2.92819	+2.24508	−0.31462
O	+1.48043	−0.24914	+2.57899
C	+3.70497	−0.05380	+0.87166
H	+2.08069	−0.04033	−0.54788
H	+1.37659	+2.25036	+0.21155
C	+3.96771	−1.53208	+0.54181
H	+4.46829	+0.55260	+0.37330
H	+3.79128	+0.12539	+1.94717
C	+2.83866	−2.39554	+1.04033
H	+4.01570	−1.66631	−0.54264
H	+4.91152	−1.85421	+0.98499
O	+3.08656	−2.95894	+2.22125
H	+2.27912	−3.39076	+2.52465
O	+1.79555	−2.53182	+0.45489

19

∗ E = +10.716 kcal/mol ; (373) 182_317_065_173_179_176_359

H	+0.00000	+0.00000	+0.00000
O	+0.00000	+0.00000	+0.96374
C	+1.27474	+0.00000	+1.38275
C	+1.36580	−0.05561	+2.89535
N	+0.39176	+0.85349	+3.47557
H	−0.55483	+0.56193	+3.26113
O	+2.20821	−0.01538	+0.63501
C	+1.23356	−1.53287	+3.30810
H	+2.37004	+0.30278	+3.14619
H	+0.49330	+0.92362	+4.47882

C +1.48097 −1.73622 +4.79331
H +0.23113 −1.89351 +3.04980
H +1.94251 −2.14310 +2.73787
C +1.31464 −3.18746 +5.20133
H +2.49247 −1.40731 +5.06896
H +0.78144 −1.14171 +5.39677
O +1.59673 −3.45694 +6.49142
H +1.88121 −2.66804 +6.95753
O +0.95517 −4.05339 +4.46697
19
* E = +10.744 kcal/mol ; (374) 010_331_164_297_086_194_004
H +0.00000 +0.00000 +0.00000
O +0.00000 +0.00000 +0.96914
C +1.27981 +0.00000 +1.36715
C +2.28134 +0.24806 +0.23281
N +1.72258 −0.24489 −1.02687
H +2.22248 +0.13572 −1.82150
O +1.60736 −0.15068 +2.50124
C +2.57488 +1.75046 +0.11625
H +3.20708 −0.25714 +0.53172
H +1.77891 −1.25471 −1.08805
C +1.35692 +2.59866 −0.24866
H +2.97786 +2.10291 +1.07027
H +3.35470 +1.87769 −0.64312
C +0.54454 +3.00675 +0.97205
H +1.67665 +3.53218 −0.72756
H +0.71449 +2.08334 −0.97404
O −0.68308 +3.49062 +0.70895
H −0.88339 +3.43997 −0.22758
O +0.95696 +2.96119 +2.08731
19
* E = +10.747 kcal/mol ; (375) 174_282_077_052_222_059_000
H +0.00000 +0.00000 +0.00000
O +0.00000 +0.00000 +0.96535
C +1.29619 +0.00000 +1.37832
C +1.39490 +0.14310 +2.88973
N +1.16774 +1.54881 +3.19130
H +0.18150 +1.77643 +3.13524
O +2.20292 −0.09524 +0.61230
C +0.43057 −0.82213 +3.58717

H	+2.43352	−0.09910	+3.13445
H	+1.49488	+1.78344	+4.11898
C	+0.57367	−2.28354	+3.11599
H	+0.58883	−0.75537	+4.66720
H	−0.58858	−0.45822	+3.40677
C	−0.76738	−2.96402	+2.91333
H	+1.10407	−2.32529	+2.15329
H	+1.14789	−2.88425	+3.82100
O	−1.57344	−2.36022	+2.02097
H	−1.15546	−1.57453	+1.64817
O	−1.11174	−3.95621	+3.47779

19

* E = +10.826 kcal/mol ; (376) 177_161_038_173_179_068_002

H	+0.00000	+0.00000	+0.00000
O	+0.00000	+0.00000	+0.96408
C	+1.27396	+0.00000	+1.38118
C	+1.37362	+0.06774	+2.89168
N	+2.69272	−0.37688	+3.27404
H	+3.38419	−0.04866	+2.60945
O	+2.20831	−0.00475	+0.63408
C	+0.99419	+1.50199	+3.31782
H	+0.61533	−0.61669	+3.29818
H	+2.95117	−0.08126	+4.20459
C	+0.90133	+1.63296	+4.84048
H	+1.74679	+2.19910	+2.92973
H	+0.03534	+1.76476	+2.85331
C	+0.54545	+3.04552	+5.26073
H	+0.14639	+0.93571	+5.22772
H	+1.85508	+1.39856	+5.31727
O	−0.69930	+3.44848	+4.93254
H	−1.18987	+2.74900	+4.49552
O	+1.28976	+3.78544	+5.82406

19

* E = +10.838 kcal/mol ; (377) 356_021_084_296_182_063_001

H	+0.00000	+0.00000	+0.00000
O	+0.00000	+0.00000	+0.97019
C	+1.26769	+0.00000	+1.36962
C	+2.29560	−0.10263	+0.23003
N	+1.68491	+0.31505	−1.02761
H	+1.71151	+1.32004	−1.15310

O	+1.58862	+0.04155	+2.51946
C	+3.60123	+0.59263	+0.62577
H	+2.49770	−1.17802	+0.14304
H	+2.12497	−0.11566	−1.82895
C	+3.43062	+2.09949	+0.84728
H	+3.96460	+0.13958	+1.55280
H	+4.34673	+0.38571	−0.15276
C	+4.73951	+2.78160	+1.19747
H	+3.03318	+2.58833	−0.05360
H	+2.73922	+2.28210	+1.67247
O	+5.69223	+2.72672	+0.23961
H	+5.37719	+2.26545	−0.53999
O	+4.95704	+3.33578	+2.22734

19

∗ E = +10.868 kcal/mol ; (378) 007_070_180_066_172_176_180

H	+0.00000	+0.00000	+0.00000
O	+0.00000	+0.00000	+0.95979
C	+1.25698	+0.00000	+1.44993
C	+2.35248	+0.18077	+0.39800
N	+2.27082	+1.55974	−0.07498
H	+2.97908	+1.74332	−0.77431
O	+1.44977	−0.11478	+2.61867
C	+3.72794	−0.07095	+0.99351
H	+2.16648	−0.56714	−0.39921
H	+1.36927	+1.79917	−0.46734
C	+3.92720	−1.51563	+1.42307
H	+4.48926	+0.19741	+0.25235
H	+3.86587	+0.59985	+1.84626
C	+5.35182	−1.78634	+1.82514
H	+3.27818	−1.77563	+2.26544
H	+3.68073	−2.21790	+0.61566
O	+5.51120	−3.03468	+2.29305
H	+6.43887	−3.14422	+2.52994
O	+6.25941	−1.00832	+1.74175

19

∗ E = +10.878 kcal/mol ; (379) 176_157_043_176_183_296_359

H	+0.00000	+0.00000	+0.00000
O	+0.00000	+0.00000	+0.96418
C	+1.26999	+0.00000	+1.38414
C	+1.37161	+0.08658	+2.89348

N	+2.65570	−0.44718	+3.28082
H	+3.38578	−0.12289	+2.65742
O	+2.20986	−0.00688	+0.64239
C	+1.10137	+1.55835	+3.27603
H	+0.56916	−0.52849	+3.32092
H	+2.89776	−0.24280	+4.23945
C	+1.08749	+1.75755	+4.79329
H	+1.87349	+2.18026	+2.80191
H	+0.13499	+1.86676	+2.86092
C	+0.76151	+3.18734	+5.17691
H	+0.33112	+1.11931	+5.25544
H	+2.06312	+1.49767	+5.22465
O	+1.66836	+4.10907	+4.79185
H	+2.40839	+3.70355	+4.33493
O	−0.22008	+3.51741	+5.76514

19

∗ E = +10.918 kcal/mol ; (380) 177_296_285_218_069_016_357

H	+0.00000	+0.00000	+0.00000
O	+0.00000	+0.00000	+0.96375
C	+1.28200	+0.00000	+1.40216
C	+1.32163	+0.06303	+2.92038
N	+0.75482	+1.30941	+3.41759
H	+1.39064	+2.08270	+3.27667
O	+2.21504	−0.02327	+0.65756
C	+0.62670	−1.19619	+3.51305
H	+2.37733	+0.05211	+3.19959
H	−0.13152	+1.54069	+2.98327
C	−0.20476	−0.92312	+4.75976
H	+0.00762	−1.68776	+2.75148
H	+1.39940	−1.93011	+3.75660
C	−1.47111	−0.10153	+4.57585
H	−0.52443	−1.87504	+5.19847
H	+0.37769	−0.40263	+5.52263
O	−1.94946	+0.04185	+3.32134
H	−1.38961	−0.36316	+2.65188
O	−2.06125	+0.37737	+5.49410

19

∗ E = +10.922 kcal/mol ; (381) 166_137_046_053_276_300_003

H	+0.00000	+0.00000	+0.00000
O	+0.00000	+0.00000	+0.96597

C	+1.25822	+0.00000	+1.39741
C	+1.36863	+0.34149	+2.87048
N	+2.33413	−0.55612	+3.46950
H	+3.16148	−0.60815	+2.88420
O	+2.21572	−0.16332	+0.69421
C	+1.75465	+1.83460	+2.95796
H	+0.38596	+0.19849	+3.33351
H	+2.60768	−0.24651	+4.39330
C	+0.83219	+2.76957	+2.15623
H	+1.74265	+2.12209	+4.01418
H	+2.79601	+1.91652	+2.61717
C	+1.18367	+2.88706	+0.67961
H	−0.20595	+2.43291	+2.21361
H	+0.87689	+3.78231	+2.57407
O	+2.41318	+3.36329	+0.41532
H	+2.91012	+3.52223	+1.22057
O	+0.44058	+2.61687	−0.21449

19

∗ E = +11.004 kcal/mol ; (382) 000_187_212_304_185_178_359

H	+0.00000	+0.00000	+0.00000
O	+0.00000	+0.00000	+0.96054
C	+1.24993	+0.00000	+1.45020
C	+2.36590	−0.00817	+0.40821
N	+3.63095	−0.17374	+1.09277
H	+4.38522	+0.30039	+0.61736
O	+1.44246	+0.00798	+2.62745
C	+2.16081	−1.14159	−0.60991
H	+2.28262	+0.95576	−0.12367
H	+3.56586	+0.16012	+2.04713
C	+2.06497	−2.49401	+0.07760
H	+1.27976	−0.97654	−1.24311
H	+3.01076	−1.14494	−1.29906
C	+1.99193	−3.62729	−0.92125
H	+2.93540	−2.63192	+0.73128
H	+1.17595	−2.54973	+0.72272
O	+1.95311	−4.86228	−0.38847
H	+1.99695	−4.82663	+0.56976
O	+1.96164	−3.47621	−2.10440

19

∗ E = +11.005 kcal/mol ; (383) 176_144_045_058_055_041_000

H	+0.00000	+0.00000	+0.00000
O	+0.00000	+0.00000	+0.96462
C	+1.25940	+0.00000	+1.40106
C	+1.34239	+0.09374	+2.91326
N	+2.46240	−0.71770	+3.34217
H	+3.26493	−0.53949	+2.74763
O	+2.21763	−0.01587	+0.68149
C	+1.46809	+1.58707	+3.28609
H	+0.40941	−0.30957	+3.32669
H	+2.71730	−0.52685	+4.30240
C	+0.32948	+2.47790	+2.78238
H	+1.57062	+1.65057	+4.37942
H	+2.41644	+1.95665	+2.87951
C	−1.07140	+2.04195	+3.17910
H	+0.46685	+3.49761	+3.16199
H	+0.33125	+2.54600	+1.69245
O	−1.21240	+1.58842	+4.44330
H	−0.37696	+1.60445	+4.91494
O	−2.01545	+2.11233	+2.45768

19

∗ E = +11.041 kcal/mol ; (384) 177_148_042_061_171_286_356

H	+0.00000	+0.00000	+0.00000
O	+0.00000	+0.00000	+0.96481
C	+1.27209	+0.00000	+1.38253
C	+1.38869	+0.06481	+2.89338
N	+2.57256	−0.67757	+3.27101
H	+3.33017	−0.46879	+2.62951
O	+2.20842	−0.00772	+0.63696
C	+1.41082	+1.54475	+3.32634
H	+0.49558	−0.41218	+3.31721
H	+2.86943	−0.45119	+4.21121
C	+0.14934	+2.32754	+2.93970
H	+1.54649	+1.55861	+4.41579
H	+2.29524	+2.02543	+2.89214
C	+0.15224	+3.69896	+3.58252
H	+0.09651	+2.47434	+1.86014
H	−0.74671	+1.76993	+3.24363
O	−0.11499	+3.71602	+4.90659
H	−0.33088	+2.83846	+5.22862
O	+0.40133	+4.71134	+3.00664

19
∗ E = +11.084 kcal/mol ; (385) 358_010_286_162_099_301_001

H	+0.00000	+0.00000	+0.00000
O	+0.00000	+0.00000	+0.96011
C	+1.25216	+0.00000	+1.43706
C	+2.39359	−0.03758	+0.40377
N	+2.01194	+0.16058	−0.99014
H	+1.54031	−0.62392	−1.41971
O	+1.46458	+0.00068	+2.60961
C	+3.48952	+0.94918	+0.81765
H	+2.80742	−1.05146	+0.48362
H	+1.49957	+1.02097	−1.15165
C	+4.84882	+0.69978	+0.13509
H	+3.14113	+1.97256	+0.62322
H	+3.60414	+0.86402	+1.89967
C	+5.14211	+1.58660	−1.06555
H	+5.66254	+0.87102	+0.83991
H	+4.91467	−0.34664	−0.19187
O	+4.26320	+1.51162	−2.07122
H	+3.54736	+0.89023	−1.85619
O	+6.09369	+2.30682	−1.12648

19
∗ E = +11.184 kcal/mol ; (386) 359_174_305_186_081_208_009

H	+0.00000	+0.00000	+0.00000
O	+0.00000	+0.00000	+0.95980
C	+1.25507	+0.00000	+1.44961
C	+2.37378	−0.01578	+0.40295
N	+3.69228	+0.12689	+0.97281
H	+3.74529	+0.96143	+1.54451
O	+1.45024	+0.01510	+2.62422
C	+2.30509	−1.30342	−0.43472
H	+2.19853	+0.84068	−0.26838
H	+3.89132	−0.65857	+1.58642
C	+3.49987	−1.39863	−1.37704
H	+2.31632	−2.17560	+0.22957
H	+1.37230	−1.34191	−1.00674
C	+4.73458	−1.91005	−0.65201
H	+3.29701	−2.11417	−2.18262
H	+3.70277	−0.42665	−1.84242
O	+5.91638	−1.50483	−1.13854

H +5.80073 −0.82125 −1.80094
O +4.68459 −2.68189 +0.25795
19
∗ E = +11.538 kcal/mol ; (387) 357_168_273_295_178_178_000
H +0.00000 +0.00000 +0.00000
O +0.00000 +0.00000 +0.96049
C +1.25010 +0.00000 +1.44892
C +2.37715 −0.06005 +0.41330
N +3.67218 +0.21380 +1.00451
H +3.86113 +1.20769 +1.01366
O +1.44365 −0.00829 +2.62740
C +2.36318 −1.45167 −0.24311
H +2.17215 +0.68607 −0.36504
H +3.65958 −0.07926 +1.97732
C +2.68840 −2.54983 +0.75773
H +1.39495 −1.66539 −0.71075
H +3.10661 −1.45992 −1.04412
C +2.63316 −3.91989 +0.11510
H +3.69287 −2.39587 +1.17264
H +1.99081 −2.53922 +1.60759
O +2.94020 −4.94827 +0.92740
H +3.15103 −4.64039 +1.81166
O +2.34059 −4.11409 −1.02444
19
∗ E = +11.626 kcal/mol ; (388) 000_042_258_289_257_047_003
H +0.00000 +0.00000 +0.00000
O +0.00000 +0.00000 +0.96052
C +1.25479 +0.00000 +1.44259
C +2.36954 −0.00666 +0.38607
N +2.09097 +0.89742 −0.73774
H +1.76312 +0.43798 −1.57511
O +1.45990 −0.00685 +2.61525
C +3.71458 +0.30125 +1.04081
H +2.39961 −1.03175 −0.00667
H +1.47159 +1.66439 −0.50621
C +3.85603 +1.76806 +1.49054
H +3.82751 −0.35622 +1.90492
H +4.50753 +0.04558 +0.33001
C +4.71739 +2.62548 +0.57413
H +2.86886 +2.24487 +1.56940

H	+4.30038	+1.83358	+2.48315
O	+4.46092	+2.52145	−0.73557
H	+3.70619	+1.93013	−0.89868
O	+5.57371	+3.35356	+0.98033

19

∗ E = +11.647 kcal/mol ; (389) 181_054_184_071_176_295_358

H	+0.00000	+0.00000	+0.00000
O	+0.00000	+0.00000	+0.96403
C	+1.27520	+0.00000	+1.38361
C	+1.35063	−0.02528	+2.90213
N	+0.57066	−1.16640	+3.36317
H	+0.53164	−1.18969	+4.37415
O	+2.20604	+0.02604	+0.63406
C	+2.79439	−0.14714	+3.37586
H	+0.94130	+0.94644	+3.23889
H	−0.37769	−1.12734	+3.01065
C	+3.60591	+1.13266	+3.14888
H	+2.76908	−0.40598	+4.44306
H	+3.26671	−0.98860	+2.85947
C	+5.00632	+1.03015	+3.71889
H	+3.69996	+1.33739	+2.08224
H	+3.09920	+1.98571	+3.62173
O	+5.07318	+0.90293	+5.06410
H	+4.19876	+0.92671	+5.45750
O	+6.00523	+1.05018	+3.07153

19

∗ E = +11.752 kcal/mol ; (390) 183_324_061_173_179_068_002

H	+0.00000	+0.00000	+0.00000
O	+0.00000	+0.00000	+0.96396
C	+1.27203	+0.00000	+1.38315
C	+1.37917	−0.06501	+2.89468
N	+0.30348	+0.68038	+3.51498
H	−0.60249	+0.29825	+3.27073
O	+2.20792	−0.02163	+0.63709
C	+1.47067	−1.55965	+3.26850
H	+2.33116	+0.41595	+3.14646
H	+0.38985	+0.69738	+4.52208
C	+1.76383	−1.75473	+4.75754
H	+0.52350	−2.04980	+3.00971
H	+2.25329	−2.02388	+2.65454

C	+1.82207	−3.22282	+5.13071
H	+2.71735	−1.27431	+5.01528
H	+0.97921	−1.30731	+5.37061
O	+2.86228	−3.90907	+4.61592
H	+3.43527	−3.34085	+4.09642
O	+1.01393	−3.77348	+5.81123

19
∗ E = +11.798 kcal/mol ; (391) 011_079_186_081_299_076_185

H	+0.00000	+0.00000	+0.00000
O	+0.00000	+0.00000	+0.95997
C	+1.25980	+0.00000	+1.43956
C	+2.34178	+0.26988	+0.38994
N	+2.35451	+1.71496	+0.17553
H	+2.98167	+1.96483	−0.57845
O	+1.46963	−0.15241	+2.60070
C	+3.70891	−0.18178	+0.89081
H	+2.08023	−0.30883	−0.51688
H	+1.43762	+2.08661	−0.04033
C	+3.97432	−1.68570	+0.73756
H	+4.47476	+0.35521	+0.32097
H	+3.80695	+0.12346	+1.93668
C	+3.00787	−2.54599	+1.51175
H	+3.90552	−1.96524	−0.32001
H	+4.97616	−1.92283	+1.09762
O	+1.80446	−2.61359	+0.89836
H	+1.20768	−3.10195	+1.47824
O	+3.23827	−3.11700	+2.53455

19
∗ E = +11.807 kcal/mol ; (392) 000_171_295_176_181_186_002

H	+0.00000	+0.00000	+0.00000
O	+0.00000	+0.00000	+0.96014
C	+1.25096	+0.00000	+1.45167
C	+2.37891	+0.00727	+0.41325
N	+3.68099	+0.21963	+1.00758
H	+3.73490	+1.14303	+1.42105
O	+1.44527	−0.01288	+2.62779
C	+2.36952	−1.31057	−0.37212
H	+2.17989	+0.83374	−0.28644
H	+3.79992	−0.43133	+1.77872
C	+3.41682	−1.30751	−1.47354

H	+2.56594	−2.14066	+0.31735
H	+1.38512	−1.51479	−0.80737
C	+3.38986	−2.59113	−2.27531
H	+3.24843	−0.47177	−2.16718
H	+4.41355	−1.15248	−1.04528
O	+4.37632	−2.72313	−3.18235
H	+4.97841	−1.97662	−3.14869
O	+2.56361	−3.44195	−2.15089

19
∗ E = +11.932 kcal/mol ; (393) 007_066_177_286_279_164_355

H	+0.00000	+0.00000	+0.00000
O	+0.00000	+0.00000	+0.95977
C	+1.25403	+0.00000	+1.45300
C	+2.35588	+0.17695	+0.40765
N	+2.21868	+1.51792	−0.15414
H	+2.95994	+1.70979	−0.81884
O	+1.43960	−0.12134	+2.62408
C	+3.73408	+0.00543	+1.04286
H	+2.21982	−0.62888	−0.33787
H	+1.32676	+1.68341	−0.60135
C	+4.14019	+1.19666	+1.90324
H	+3.72309	−0.90445	+1.64738
H	+4.47790	−0.12175	+0.24997
C	+4.69323	+2.34172	+1.07823
H	+3.29717	+1.54258	+2.51081
H	+4.93432	+0.90295	+2.60079
O	+4.75088	+3.52732	+1.70438
H	+4.34494	+3.47475	+2.57243
O	+5.10544	+2.23230	−0.03987

19
∗ E = +12.014 kcal/mol ; (394) 007_062_182_292_137_296_357

H	+0.00000	+0.00000	+0.00000
O	+0.00000	+0.00000	+0.96005
C	+1.24127	+0.00000	+1.45936
C	+2.36704	+0.15579	+0.43857
N	+2.21962	+1.44733	−0.22575
H	+2.93429	+1.57049	−0.93230
O	+1.40610	−0.12902	+2.63807
C	+3.72306	+0.07007	+1.12933
H	+2.27294	−0.70515	−0.25013

H	+1.32009	+1.58780	−0.66688
C	+4.01352	+1.27001	+2.05546
H	+3.75690	−0.87038	+1.68945
H	+4.50024	−0.00316	+0.36200
C	+4.62799	+0.82680	+3.37012
H	+4.69828	+1.97785	+1.58962
H	+3.08410	+1.80862	+2.27002
O	+3.83376	+0.06116	+4.12774
H	+2.96262	−0.04757	+3.71708
O	+5.73466	+1.11342	+3.71985

19

∗ E = +12.085 kcal/mol ; (395) 182_333_052_300_187_069_003

H	+0.00000	+0.00000	+0.00000
O	+0.00000	+0.00000	+0.96434
C	+1.27541	+0.00000	+1.37988
C	+1.40315	−0.05872	+2.89099
N	+0.23930	+0.51306	+3.53599
H	−0.62776	+0.12133	+3.19251
O	+2.20597	−0.02302	+0.62871
C	+1.74205	−1.51673	+3.27322
H	+2.27662	+0.55532	+3.13334
H	+0.28118	+0.41538	+4.54163
C	+0.66375	−2.51318	+2.83376
H	+2.70069	−1.79137	+2.82051
H	+1.88051	−1.54638	+4.36188
C	+0.93704	−3.90385	+3.36824
H	−0.32332	−2.18136	+3.18355
H	+0.62699	−2.58759	+1.74478
O	+0.79554	−4.04386	+4.70455
H	+0.49505	−3.22754	+5.10925
O	+1.27300	−4.82999	+2.69950

19

∗ E = +12.117 kcal/mol ; (396) 358_162_026_314_092_237_017

H	+0.00000	+0.00000	+0.00000
O	+0.00000	+0.00000	+0.96134
C	+1.23505	+0.00000	+1.45850
C	+2.37287	−0.05854	+0.44947
N	+3.58281	+0.37873	+1.10476
H	+3.53261	+0.21497	+2.10319
O	+1.41644	−0.05385	+2.64326

C +2.43012 −1.50702 −0.09694
H +2.13558 +0.61795 −0.38514
H +4.40839 −0.06220 +0.72444
C +2.34703 −2.55780 +1.01742
H +1.62200 −1.69557 −0.81410
H +3.36884 −1.60370 −0.65129
C +0.89813 −2.97104 +1.26806
H +2.85703 −3.47357 +0.70673
H +2.83896 −2.19696 +1.92773
O +0.41584 −2.82740 +2.50482
H +0.96365 −2.23771 +3.03325
O +0.22552 −3.43250 +0.39354
19
∗ E = +12.146 kcal/mol ; (397) 181_315_064_176_183_299_000
H +0.00000 +0.00000 +0.00000
O +0.00000 +0.00000 +0.96387
C +1.27628 +0.00000 +1.38409
C +1.36026 −0.03442 +2.89808
N +0.41527 +0.91249 +3.45805
H −0.54149 +0.66432 +3.23657
O +2.20932 −0.03050 +0.63799
C +1.18241 −1.50260 +3.33614
H +2.37445 +0.28939 +3.15292
H +0.51535 +1.00260 +4.45974
C +1.35506 −1.66268 +4.84745
H +0.18605 −1.83443 +3.01083
H +1.91615 −2.12647 +2.81231
C +1.26944 −3.10696 +5.29925
H +2.33111 −1.28713 +5.16350
H +0.58291 −1.09075 +5.37858
O +0.10090 −3.72768 +5.03503
H −0.51559 −3.14037 +4.59281
O +2.14930 −3.69055 +5.85030
19
∗ E = +12.319 kcal/mol ; (398) 353_162_026_306_190_184_001
H +0.00000 +0.00000 +0.00000
O +0.00000 +0.00000 +0.96061
C +1.25058 +0.00000 +1.44519
C +2.35608 −0.16124 +0.40958
N +3.60207 +0.27091 +0.99635

H	+3.57771	+0.17160	+2.00446
O	+1.45140	+0.04491	+2.62113
C	+2.34368	−1.62910	−0.06817
H	+2.11246	+0.47752	−0.45360
H	+4.39970	−0.21977	+0.61644
C	+2.44673	−2.61632	+1.08499
H	+1.44020	−1.84121	−0.65220
H	+3.18350	−1.77961	−0.75457
C	+2.67834	−4.03068	+0.59566
H	+3.27229	−2.34105	+1.75556
H	+1.53891	−2.60128	+1.70311
O	+2.69834	−4.97330	+1.55549
H	+2.54452	−4.59037	+2.42220
O	+2.84177	−4.32592	−0.54814

19

∗ E = +12.378 kcal/mol ; (399) 174_291_071_280_130_290_355

H	+0.00000	+0.00000	+0.00000
O	+0.00000	+0.00000	+0.96574
C	+1.29936	+0.00000	+1.37439
C	+1.42102	+0.14215	+2.88083
N	+0.99909	+1.49045	+3.22500
H	+0.00071	+1.61711	+3.11379
O	+2.19809	−0.10337	+0.60043
C	+0.71575	−1.01310	+3.62414
H	+2.49484	+0.06965	+3.07247
H	+1.24704	+1.72130	+4.17829
C	−0.82115	−0.88370	+3.75129
H	+0.98215	−1.95804	+3.13319
H	+1.14761	−1.06914	+4.62709
C	−1.52096	−2.15380	+3.31089
H	−1.11688	−0.68525	+4.78097
H	−1.19936	−0.06243	+3.13194
O	−1.45265	−2.39291	+1.98863
H	−1.02077	−1.65786	+1.53820
O	−2.07302	−2.91283	+4.04647

19

∗ E = +12.625 kcal/mol ; (400) 186_295_064_048_274_298_000

H	+0.00000	+0.00000	+0.00000
O	+0.00000	+0.00000	+0.96500
C	+1.27567	+0.00000	+1.39055

C	+1.33007	−0.15182	+2.90116
N	+0.72173	+1.02698	+3.49630
H	−0.26210	+1.08081	+3.25726
O	+2.21747	+0.07527	+0.66294
C	+0.68054	−1.48300	+3.32156
H	+2.39394	−0.16655	+3.15722
H	+0.80482	+1.01361	+4.50491
C	+1.16260	−2.68870	+2.49382
H	+0.90280	−1.64450	+4.38123
H	−0.40810	−1.36393	+3.23938
C	+0.37986	−2.88918	+1.20628
H	+2.21460	−2.57564	+2.21807
H	+1.07013	−3.60958	+3.08126
O	−0.93373	−3.15015	+1.36808
H	−1.17650	−3.16225	+2.29622
O	+0.84969	−2.83795	+0.11241

19

∗ E = +13.149 kcal/mol ; (401) 182_072_180_083_298_084_007

H	+0.00000	+0.00000	+0.00000
O	+0.00000	+0.00000	+0.96436
C	+1.27697	+0.00000	+1.38116
C	+1.34820	−0.04736	+2.90359
N	+0.93941	−1.39576	+3.28032
H	+0.95699	−1.50827	+4.28611
O	+2.21169	+0.00051	+0.64054
C	+2.76535	+0.23526	+3.38847
H	+0.65579	+0.72639	+3.28926
H	+0.00053	−1.59530	+2.95643
C	+3.11478	+1.72783	+3.47145
H	+2.87860	−0.19468	+4.38940
H	+3.46915	−0.27994	+2.72843
C	+3.05255	+2.43796	+2.13058
H	+2.44270	+2.22518	+4.18389
H	+4.13553	+1.84196	+3.83946
O	+1.82063	+2.86532	+1.75758
H	+1.18300	+2.74280	+2.46368
O	+3.99046	+2.61934	+1.42318

19

∗ E = +13.237 kcal/mol ; (402) 357_167_279_170_168_285_355

H	+0.00000	+0.00000	+0.00000

O	+0.00000	+0.00000	+0.96029
C	+1.24871	+0.00000	+1.45391
C	+2.37904	−0.06307	+0.42230
N	+3.67088	+0.23899	+1.00240
H	+3.80574	+1.23835	+1.09020
O	+1.44257	+0.01232	+2.63035
C	+2.40956	−1.47047	−0.19777
H	+2.16560	+0.66541	−0.37335
H	+3.69509	−0.13284	+1.94823
C	+3.36167	−1.51585	−1.39612
H	+2.73667	−2.16809	+0.58473
H	+1.40868	−1.79560	−0.50416
C	+3.62639	−2.94279	−1.82342
H	+2.92964	−0.98708	−2.24846
H	+4.29888	−1.02162	−1.11445
O	+4.46376	−3.63048	−1.02014
H	+4.81971	−3.06679	−0.32898
O	+3.12554	−3.46903	−2.76847

19

* E = +13.854 kcal/mol ; (403) 354_143_297_054_175_174_357

H	+0.00000	+0.00000	+0.00000
O	+0.00000	+0.00000	+0.95978
C	+1.26023	+0.00000	+1.43936
C	+2.38555	−0.14501	+0.40817
N	+3.55225	+0.64487	+0.73505
H	+3.33819	+1.63479	+0.74087
O	+1.46400	+0.06135	+2.61237
C	+2.78935	−1.62237	+0.34799
H	+2.01901	+0.16870	−0.58117
H	+3.86526	+0.40555	+1.67081
C	+1.62963	−2.56855	+0.07501
H	+3.55967	−1.73967	−0.41788
H	+3.25461	−1.88854	+1.30440
C	+2.10670	−3.99860	−0.10104
H	+0.89046	−2.54420	+0.88726
H	+1.09433	−2.28569	−0.84380
O	+1.12977	−4.91743	−0.22315
H	+0.26142	−4.51790	−0.13901
O	+3.25203	−4.32116	−0.14447

19

∗ E = +14.072 kcal/mol ; (404) 348_181_302_305_135_291_354

H	+0.00000	+0.00000	+0.00000
O	+0.00000	+0.00000	+0.96161
C	+1.26352	+0.00000	+1.45584
C	+2.37044	−0.27757	+0.43237
N	+3.68836	−0.28662	+1.01831
H	+3.88332	+0.59699	+1.47455
O	+1.44632	+0.12523	+2.62488
C	+2.07400	−1.62910	−0.23708
H	+2.33171	+0.50952	−0.33405
H	+3.73938	−0.99113	+1.74765
C	+1.91370	−2.77803	+0.78797
H	+1.17064	−1.55343	−0.85422
H	+2.89555	−1.84773	−0.92188
C	+0.71309	−3.64193	+0.45378
H	+2.79261	−3.42212	+0.80370
H	+1.77795	−2.37776	+1.80338
O	−0.48384	−3.04816	+0.63152
H	−0.40692	−2.17969	+1.04164
O	+0.78198	−4.75181	+0.02555

19

∗ E = +14.181 kcal/mol ; (405) 350_139_042_058_171_170_357

H	+0.00000	+0.00000	+0.00000
O	+0.00000	+0.00000	+0.95966
C	+1.25667	+0.00000	+1.43693
C	+2.37305	−0.22601	+0.42172
N	+3.45778	+0.67254	+0.75803
H	+3.58358	+0.68038	+1.76533
O	+1.46727	+0.10357	+2.60588
C	+2.75274	−1.71634	+0.46399
H	+2.00401	+0.01778	−0.58742
H	+4.32534	+0.37251	+0.33118
C	+1.58891	−2.65317	+0.17303
H	+3.55250	−1.90004	−0.26072
H	+3.16523	−1.93543	+1.45522
C	+2.05084	−4.08988	+0.00971
H	+0.83259	−2.60708	+0.96785
H	+1.07881	−2.36928	−0.75988
O	+1.06780	−5.00746	−0.04357
H	+0.20597	−4.60464	+0.08221

O +3.19210 −4.41703 −0.08806
19
∗ E = +14.527 kcal/mol ; (406) 357_135_293_056_181_070_003
H +0.00000 +0.00000 +0.00000
O +0.00000 +0.00000 +0.95994
C +1.25173 +0.00000 +1.44566
C +2.39999 −0.08233 +0.43232
N +3.46886 +0.84997 +0.72440
H +3.17089 +1.80944 +0.59716
O +1.45093 +0.01554 +2.62294
C +2.95352 −1.50931 +0.49238
H +2.02747 +0.11862 −0.58229
H +3.74426 +0.74827 +1.69657
C +1.88629 −2.57876 +0.23775
H +3.76348 −1.59241 −0.23760
H +3.39933 −1.63905 +1.48775
C +2.47593 −3.97199 +0.32901
H +1.06898 −2.49022 +0.96675
H +1.46253 −2.47281 −0.76432
O +2.82919 −4.36297 +1.56897
H +2.60346 −3.69968 +2.22575
O +2.65223 −4.68829 −0.60645
19
∗ E = +14.597 kcal/mol ; (407) 004_191_201_113_063_181_359
H +0.00000 +0.00000 +0.00000
O +0.00000 +0.00000 +0.95951
C +1.25702 +0.00000 +1.44932
C +2.36722 +0.08743 +0.40811
N +3.62953 −0.17647 +1.05263
H +4.38874 +0.20166 +0.50017
O +1.44545 −0.01495 +2.62482
C +2.15583 −0.87249 −0.79058
H +2.28861 +1.12550 +0.02516
H +3.64479 +0.20966 +1.98852
C +1.87378 −0.14896 −2.10213
H +3.07920 −1.44673 −0.89464
H +1.36178 −1.59922 −0.58480
C +3.05704 +0.71116 −2.51342
H +1.66919 −0.86129 −2.91078
H +0.99027 +0.50428 −2.03776

O +2.90357 +1.40908 −3.65018
H +2.04536 +1.24813 −4.04853
O +4.07175 +0.78454 −1.88931
19
∗ E = +14.629 kcal/mol ; (408) 355_166_283_291_286_302_005
H +0.00000 +0.00000 +0.00000
O +0.00000 +0.00000 +0.95995
C +1.25505 +0.00000 +1.44981
C +2.37573 −0.11382 +0.40835
N +3.67867 +0.21018 +0.93705
H +3.79676 +1.21066 +1.03138
O +1.45100 +0.04395 +2.62374
C +2.38177 −1.54735 −0.15673
H +2.14564 +0.57651 −0.41471
H +3.78794 −0.18261 +1.86927
C +2.83866 −2.57940 +0.88172
H +1.38132 −1.82151 −0.51078
H +3.03813 −1.54922 −1.03480
C +4.33005 −2.58131 +1.18425
H +2.32317 −2.42826 +1.83483
H +2.58465 −3.58643 +0.52970
O +5.14162 −2.78913 +0.13203
H +4.64833 −2.83355 −0.68916
O +4.78187 −2.46520 +2.28155
19
∗ E = +15.459 kcal/mol ; (409) 352_157_037_292_289_307_356
H +0.00000 +0.00000 +0.00000
O +0.00000 +0.00000 +0.95984
C +1.25295 +0.00000 +1.44724
C +2.36024 −0.20186 +0.41764
N +3.57873 +0.35251 +0.94874
H +3.64642 +0.18737 +1.94776
O +1.45464 +0.08131 +2.61839
C +2.40866 −1.71205 +0.07506
H +2.07809 +0.33986 −0.49864
H +4.40865 +0.00230 +0.48846
C +2.88818 −2.57354 +1.24970
H +1.41501 −2.05341 −0.23969
H +3.05829 −1.83540 −0.80151
C +4.36530 −2.43242 +1.57090

H +2.34099 −2.32991 +2.16410
H +2.70361 −3.63138 +1.02589
O +5.20778 −2.62616 +0.52897
H +4.73244 −2.89666 −0.25934
O +4.80244 −2.18505 +2.65011
19
* E = +15.617 kcal/mol ; (410) 353_174_293_278_053_060_000
H +0.00000 +0.00000 +0.00000
O +0.00000 +0.00000 +0.95989
C +1.26107 +0.00000 +1.44787
C +2.37223 −0.15996 +0.39602
N +3.69892 −0.02570 +0.94998
H +3.85293 +0.92422 +1.26505
O +1.46453 +0.12165 +2.61221
C +2.24357 −1.49606 −0.36646
H +2.21729 +0.64926 −0.33238
H +3.78730 −0.60932 +1.77953
C +2.84313 −2.68379 +0.39543
H +1.19350 −1.69004 −0.62578
H +2.78201 −1.40190 −1.31325
C +2.31476 −2.84181 +1.80988
H +2.64803 −3.61015 −0.15699
H +3.92441 −2.56285 +0.47350
O +0.98730 −3.03535 +1.92397
H +0.55358 −3.04560 +1.06798
O +3.00540 −2.79265 +2.78034
19
* E = +15.771 kcal/mol ; (411) 357_147_294_061_178_306_001
H +0.00000 +0.00000 +0.00000
O +0.00000 +0.00000 +0.95994
C +1.25179 +0.00000 +1.44981
C +2.39565 −0.07097 +0.42882
N +3.57029 +0.65412 +0.86391
H +3.38697 +1.64944 +0.90888
O +1.44927 +0.02348 +2.62515
C +2.77689 −1.54258 +0.21783
H +2.05973 +0.35027 −0.53041
H +3.80545 +0.36217 +1.80825
C +1.63864 −2.40053 −0.33727
H +3.64853 −1.53389 −0.44684

H	+3.11669	−1.94805	+1.17897
C	+2.03142	−3.84524	−0.59465
H	+0.79041	−2.43368	+0.35031
H	+1.28373	−1.97976	−1.29006
O	+3.11425	−4.02795	−1.37565
H	+3.50232	−3.19258	−1.64390
O	+1.43440	−4.78082	−0.16383

19

∗ E = +15.848 kcal/mol ; (412) 347_147_297_047_266_112_347

H	+0.00000	+0.00000	+0.00000
O	+0.00000	+0.00000	+0.96049
C	+1.27612	+0.00000	+1.44016
C	+2.36913	−0.31227	+0.41703
N	+3.60703	+0.38561	+0.67453
H	+3.49166	+1.38919	+0.60359
O	+1.48001	+0.16797	+2.59828
C	+2.64416	−1.82606	+0.43308
H	+2.01123	−0.03082	−0.58498
H	+3.91490	+0.18698	+1.62135
C	+1.39671	−2.72471	+0.39599
H	+3.29741	−2.03640	−0.41654
H	+3.21609	−2.06152	+1.33864
C	+0.94240	−3.12607	+1.79818
H	+0.58469	−2.27496	−0.18715
H	+1.65474	−3.66674	−0.09304
O	−0.21622	−2.63298	+2.24994
H	−0.54423	−1.91426	+1.70086
O	+1.59186	−3.87484	+2.46331

19

∗ E = +16.758 kcal/mol ; (413) 353_143_039_062_174_290_357

H	+0.00000	+0.00000	+0.00000
O	+0.00000	+0.00000	+0.95969
C	+1.25049	+0.00000	+1.44471
C	+2.37990	−0.17063	+0.42967
N	+3.47654	+0.66839	+0.85834
H	+3.55474	+0.63088	+1.86957
O	+1.45635	+0.07416	+2.61627
C	+2.72901	−1.66914	+0.34695
H	+2.03116	+0.16347	−0.56109
H	+4.35513	+0.37380	+0.45224

C +1.56760 −2.54471 −0.13811
H +3.59276 −1.76870 −0.32352
H +3.05541 −2.00019 +1.33958
C +2.00148 −3.98510 −0.33215
H +0.75177 −2.54951 +0.58805
H +1.17619 −2.15758 −1.09064
O +2.83929 −4.19988 −1.36934
H +3.00091 −3.39355 −1.86325
O +1.66964 −4.88612 +0.37061
19
* E = +16.939 kcal/mol ; (414) 351_155_035_173_175_067_002
H +0.00000 +0.00000 +0.00000
O +0.00000 +0.00000 +0.95972
C +1.25192 +0.00000 +1.44614
C +2.36223 −0.21357 +0.42153
N +3.56753 +0.38099 +0.94341
H +3.59374 +0.27373 +1.95191
O +1.45655 +0.09631 +2.61557
C +2.43737 −1.73118 +0.14339
H +2.07876 +0.29527 −0.51542
H +4.40781 −0.00984 +0.54114
C +3.40635 −2.05364 −0.99765
H +2.75819 −2.23252 +1.06422
H +1.43277 −2.11114 −0.09147
C +3.58106 −3.54827 −1.18932
H +3.04715 −1.60449 −1.93322
H +4.40138 −1.65317 −0.79416
O +2.48828 −4.20932 −1.62287
H +1.75285 −3.61275 −1.77575
O +4.59197 −4.13602 −0.96568
19
* E = +17.494 kcal/mol ; (415) 344_160_032_294_077_022_349
H +0.00000 +0.00000 +0.00000
O +0.00000 +0.00000 +0.96075
C +1.26224 +0.00000 +1.44333
C +2.33281 −0.38827 +0.43550
N +3.60444 +0.09033 +0.91275
H +3.63096 +0.08465 +1.92642
O +1.47956 +0.19275 +2.59642
C +2.24944 −1.91985 +0.20584

H	+2.09278	+0.09835	−0.52332
H	+4.38363	−0.43829	+0.54496
C	+2.60412	−2.77090	+1.42359
H	+1.26450	−2.18745	−0.20532
H	+2.96030	−2.15764	−0.59352
C	+1.60510	−2.94662	+2.56338
H	+2.82308	−3.79272	+1.09340
H	+3.52394	−2.40710	+1.88960
O	+0.30261	−2.74501	+2.30809
H	+0.14243	−2.34262	+1.45225
O	+1.95578	−3.33335	+3.63448

3 Ala

3.1 Reference Z-matrix for Ala

Bond distances are given in Å; bond and dihedral angles, in degrees.

h						
o	1	oh2				
c	2	co3	1	coh3		
c	3	cc4	2	cco4	1	dih4
n	4	nc5	3	ncc5	2	dih5
h	5	hn6	4	hnc6	3	dih6
o	3	oc7	2	oco7	4	dih7
c	4	cc8	3	ccc8	5	dih8
h	4	hc9	3	hcc9	5	dih9
h	5	hn10	4	hnc10	6	dih10
h	8	hc11	4	hcc11	3	dih11
h	8	hc12	4	hcc12	11	dih12
h	8	hc13	4	hcc13	11	dih13

oh2	0.96831095
co3	1.36030912
coh3	111.91107106
cc4	1.50817026
cco4	110.44746386
dih4	181.66847674
nc5	1.46955971
ncc5	106.21958447
dih5	52.24738606
hn6	1.00334632
hnc6	113.29994021
dih6	174.22216263
oc7	1.2005964
oco7	122.1338143
dih7	181.20436871
cc8	1.52544932
ccc8	109.98628363
dih8	118.94210791
hc9	1.08977021
hcc9	106.86760327
dih9	238.57650318

```
hn10     1.00286975
hnc10    113.00633475
dih10    129.62721763
hc11     1.08390454
hcc11    110.22227297
dih11    177.78923293
hc12      1.0808178
hcc12    108.98672573
dih12    119.56307255
hc13     1.08132627
hcc13    110.8248062
dih13    239.46413686
```

3.2 Reference energy and target torsions for Ala

- Reference M08-HX/MG3S energy: -323.7586543 hartree
- Target torsions:
  - torsion1: dih4
  - torsion2: dih5
  - torsion3: dih6

3.3 Properties of Ala conformers

Table S3: Relative energy (kcal mol^{-1}), rotational constants (B1 to B3, GHz) and dipolar moment (Debye) for the conformers of Ala located at the M08-HX/MG3S level.

Conformer	Rel. Energy	B1	B2	B3	Dip. Mom.
1	0.000	2.251	3.188	5.101	1.413
2	0.247	2.172	3.469	4.911	5.319
3	1.035	2.175	3.400	5.061	2.111
4	1.154	2.322	3.172	5.099	2.219
5	1.277	2.213	3.257	5.094	1.363
6	1.388	2.480	2.917	5.112	1.733
7	1.635	2.174	3.382	5.108	2.399

8	2.454	2.499	2.958	5.079	2.622
9	5.054	2.269	3.172	5.045	3.304
10	6.060	2.322	3.185	5.044	4.292
11	8.841	2.245	3.232	5.093	5.102

3.4 Vibrational frequencies of Ala conformers

Harmonic vibrational zero point energy (ZPE) and frequencies for each conformer in cm^{-1}.

- Conformer (1); ZPE = 23839.248 cm^{-1}:

47.54	195.61	213.09	249.97	306.03	380.60
498.35	595.05	640.04	761.54	808.11	908.73
943.20	1019.77	1090.87	1167.56	1194.90	1283.80
1301.09	1376.94	1387.97	1419.30	1485.35	1490.18
1669.70	1864.66	3041.96	3050.56	3127.53	3155.00
3540.34	3619.24	3843.92			

- Conformer (2); ZPE = 23975.584 cm^{-1}:

58.07	217.73	255.81	284.70	366.01	404.47
538.68	567.72	746.70	805.34	822.95	873.94
941.32	1016.51	1101.91	1168.87	1229.26	1250.65
1314.01	1388.21	1400.49	1427.35	1479.36	1490.31
1657.83	1891.13	3041.44	3048.94	3121.71	3152.30
3548.16	3640.97	3698.34			

- Conformer (3); ZPE = 23830.070 cm^{-1}:

63.44	203.71	227.41	277.16	332.07	415.28
500.16	570.67	625.04	745.71	811.69	837.23
954.02	1030.20	1109.81	1169.60	1200.93	1239.35
1337.81	1351.83	1400.97	1430.36	1485.88	1494.70
1634.39	1865.01	2944.74	3053.21	3132.61	3150.33
3561.04	3656.26	3847.52			

- Conformer (4); ZPE = 23835.748 cm^{-1}:

62.85	208.44	231.92	253.07	317.86	377.11
507.88	589.85	640.30	767.37	808.64	850.45
932.87	1041.48	1098.44	1173.17	1189.34	1251.26
1328.01	1348.39	1389.81	1443.09	1485.44	1498.17
1641.30	1868.19	3033.84	3039.61	3121.99	3130.52
3548.53	3644.46	3847.85			

- Conformer (5); ZPE = 23841.257 cm^{-1}:

35.27	222.11	247.19	277.50	309.79	377.05
510.37	570.34	611.24	761.89	814.75	884.26
944.97	1023.15	1082.43	1162.57	1191.11	1257.74
1311.10	1384.14	1402.01	1417.95	1479.71	1482.57
1656.86	1865.91	3037.24	3054.36	3131.93	3154.88
3539.36	3630.92	3849.86			

- Conformer (6); ZPE = 23858.045 cm^{-1}:

29.38	228.56	230.51	277.59	292.35	374.43
535.46	592.21	615.86	784.99	800.34	885.40
935.41	1016.97	1056.35	1167.95	1206.53	1271.52
1324.33	1372.52	1389.78	1399.56	1481.38	1489.40
1664.29	1865.59	3044.15	3081.26	3126.28	3148.68
3544.31	3630.22	3852.53			

- Conformer (7); ZPE = 23823.590 cm^{-1}:

54.93	213.08	253.03	271.48	319.53	402.60
501.57	559.23	602.64	751.19	810.33	860.00
955.19	1029.25	1095.35	1170.53	1191.20	1250.47
1328.35	1355.52	1405.65	1439.73	1479.56	1489.37
1649.33	1875.34	2942.29	3053.08	3132.64	3150.59
3557.57	3646.17	3850.40			

- Conformer (8); ZPE = 23831.098 cm^{-1}:

47.51	231.81	235.59	255.04	305.58	360.53
534.10	584.73	612.10	785.42	807.51	860.12
926.16	1027.58	1097.00	1114.53	1206.52	1254.92
1332.84	1367.09	1390.94	1421.49	1482.04	1492.64
1649.32	1877.65	3036.35	3083.54	3123.02	3126.37
3544.57	3633.32	3854.26			

- Conformer (9); ZPE = 23787.559 cm^{-1}:

60.03	189.22	209.41	250.00	305.95	384.81
488.77	502.14	644.41	749.38	816.54	914.03
940.80	1021.15	1086.68	1165.77	1192.16	1279.85
1293.72	1358.59	1397.45	1415.77	1476.38	1493.24
1672.69	1898.83	3015.38	3044.99	3119.69	3145.78
3537.85	3615.66	3888.01			

- Conformer (10); ZPE = 23779.949 cm^{-1}:

64.02	193.06	235.56	252.97	329.50	385.13
492.34	512.90	649.37	750.86	814.05	852.40
934.38	1040.62	1094.09	1173.34	1211.63	1241.05
1314.73	1344.56	1397.69	1422.10	1485.32	1491.97
1644.36	1900.67	2976.17	3034.73	3115.22	3125.74
3546.29	3643.57	3889.51			

- Conformer (11); ZPE = 23661.530 cm^{-1}:

66.24	105.48	219.17	248.78	300.11	386.51
476.17	505.66	611.84	748.73	797.25	842.95
951.03	1025.92	1089.56	1157.47	1178.43	1240.45
1295.02	1353.81	1402.91	1433.72	1476.79	1483.98
1655.60	1911.75	2927.87	3054.87	3136.20	3153.38
3552.69	3640.97	3891.75			

3.5 Cartesian coordinates of Ala conformers

Each line starting by an asterisk contains the relative energy of the corresponding conformer and the dihedral angle for each of the target torsions (separated by underscores). Cartesian coordinates are given in Å.

```
13
 * E =   +0.000  kcal/mol ;  (1)  179_172_305
 H    +0.00000    +0.00000    +0.00000
 O    +0.00000    +0.00000    +0.96381
 C    +1.27671    +0.00000    +1.37970
 C    +1.38420    +0.01525    +2.89925
 N    +2.73588    -0.17971    +3.37308
 H    +3.12725    -1.03630    +2.99930
 O    +2.20484    -0.00519    +0.62210
 C    +0.82142    +1.33224    +3.43365
```

H +0.75587 −0.80677 +3.26523
H +3.32835 +0.57098 +3.03394
H +0.86032 +1.32387 +4.52417
H +1.42649 +2.17243 +3.07604
H −0.20886 +1.48465 +3.10712
13
∗ E = +0.247 kcal/mol ; (2) 356_017_092
H +0.00000 +0.00000 +0.00000
O +0.00000 +0.00000 +0.97139
C +1.27226 +0.00000 +1.35893
C +2.28873 −0.09601 +0.20929
N +1.63882 +0.24041 −1.05804
H +1.73643 +1.22824 −1.26435
O +1.60085 +0.03829 +2.50763
C +3.53304 +0.72029 +0.53265
H +2.55461 −1.16011 +0.16820
H +2.03523 −0.27275 −1.83316
H +4.30800 +0.55246 −0.21932
H +3.29030 +1.78829 +0.54987
H +3.91980 +0.45125 +1.51685
13
∗ E = +1.035 kcal/mol ; (3) 182_194_203
H +0.00000 +0.00000 +0.00000
O +0.00000 +0.00000 +0.96369
C +1.27654 +0.00000 +1.37228
C +1.40199 −0.06051 +2.88139
N +2.76901 +0.26736 +3.23351
H +3.01926 −0.10461 +4.13884
O +2.19996 +0.00624 +0.60860
C +0.41954 +0.87824 +3.57743
H +1.12159 −1.10089 +3.13108
H +3.41461 −0.08363 +2.53644
H +0.55623 +0.80973 +4.65971
H +0.61668 +1.91053 +3.27747
H −0.61437 +0.62317 +3.33782
13
∗ E = +1.154 kcal/mol ; (4) 177_162_033
H +0.00000 +0.00000 +0.00000
O +0.00000 +0.00000 +0.96375
C +1.27467 +0.00000 +1.37736

C	+1.38176	+0.06872	+2.88522
N	+2.70857	−0.36659	+3.26999
H	+3.38196	−0.09829	+2.56088
O	+2.20459	−0.00714	+0.62181
C	+1.02075	+1.49721	+3.32096
H	+0.62884	−0.61893	+3.29146
H	+2.98665	+0.03570	+4.15478
H	+1.08213	+1.57429	+4.40996
H	+1.72385	+2.21361	+2.88477
H	+0.00562	+1.76240	+3.01387

13

∗ E = +1.277 kcal/mol ; (5) 181_057_306

H	+0.00000	+0.00000	+0.00000
O	+0.00000	+0.00000	+0.96351
C	+1.28015	+0.00000	+1.38019
C	+1.36872	−0.03046	+2.90166
N	+0.68081	−1.18457	+3.46884
H	−0.27776	−1.23454	+3.14579
O	+2.20358	+0.00600	+0.61741
C	+2.81795	−0.00389	+3.34815
H	+0.84842	+0.86779	+3.25932
H	+1.14659	−2.04303	+3.19626
H	+2.86429	+0.01358	+4.43821
H	+3.34191	−0.89389	+2.98589
H	+3.33789	+0.86695	+2.94495

13

∗ E = +1.388 kcal/mol ; (6) 183_321_301

H	+0.00000	+0.00000	+0.00000
O	+0.00000	+0.00000	+0.96363
C	+1.28077	+0.00000	+1.37538
C	+1.39718	−0.07040	+2.89213
N	+0.41365	+0.70581	+3.62312
H	+0.46460	+1.68955	+3.38929
O	+2.20338	+0.01615	+0.61141
C	+1.29457	−1.54015	+3.29876
H	+2.39742	+0.30380	+3.13026
H	−0.52296	+0.38849	+3.39973
H	+1.44076	−1.62680	+4.37696
H	+0.30086	−1.92850	+3.05135
H	+2.04224	−2.14615	+2.78190

```
13
 * E =  +1.635 kcal/mol ; (7) 179_046_182
 H   +0.00000   +0.00000   +0.00000
 O   +0.00000   +0.00000   +0.96354
 C   +1.27873   +0.00000   +1.37771
 C   +1.37687   +0.01503   +2.89238
 N   +0.44197   −0.96168   +3.43711
 H   +0.46878   −0.94773   +4.44865
 O   +2.20247   +0.02486   +0.61781
 C   +2.79474   −0.29752   +3.33470
 H   +1.12918   +1.05413   +3.17977
 H   −0.50691   −0.76836   +3.14202
 H   +2.86981   −0.23709   +4.42401
 H   +3.06701   −1.30794   +3.02085
 H   +3.50296   +0.40574   +2.89356
13
 * E =  +2.454 kcal/mol ; (8) 182_317_060
 H   +0.00000   +0.00000   +0.00000
 O   +0.00000   +0.00000   +0.96353
 C   +1.27810   +0.00000   +1.37886
 C   +1.37902   −0.04657   +2.88988
 N   +0.40121   +0.85720   +3.47610
 H   −0.53845   +0.58607   +3.20918
 O   +2.20451   −0.02395   +0.62186
 C   +1.24717   −1.51294   +3.32544
 H   +2.38194   +0.31702   +3.13108
 H   +0.45802   +0.83097   +4.48655
 H   +1.35866   −1.58699   +4.41062
 H   +0.25947   −1.89843   +3.05284
 H   +2.01088   −2.13932   +2.85548
13
 * E =  +5.054 kcal/mol ; (9) 358_169_304
 H   +0.00000   +0.00000   +0.00000
 O   +0.00000   +0.00000   +0.96010
 C   +1.25746   +0.00000   +1.44335
 C   +2.36955   −0.03877   +0.38961
 N   +3.67985   +0.21931   +0.93930
 H   +3.69425   +1.11126   +1.42018
 O   +1.45747   +0.01459   +2.61844
 C   +2.36247   −1.40197   −0.30612
```

H	+2.14637	+0.74155	−0.35294
H	+3.89128	−0.47932	+1.64473
H	+3.12847	−1.41620	−1.08308
H	+2.59111	−2.18973	+0.41888
H	+1.39536	−1.63697	−0.75971

13

∗ E = +6.060 kcal/mol ; (10) 355_162_024

H	+0.00000	+0.00000	+0.00000
O	+0.00000	+0.00000	+0.96018
C	+1.25409	+0.00000	+1.44123
C	+2.35468	−0.12140	+0.39378
N	+3.60322	+0.31320	+0.97750
H	+3.56351	+0.21064	+1.98599
O	+1.45883	+0.04689	+2.61506
C	+2.37282	−1.56895	−0.12021
H	+2.08682	+0.54350	−0.44382
H	+4.38813	−0.21529	+0.62254
H	+3.15471	−1.68165	−0.87623
H	+2.58457	−2.25499	+0.70508
H	+1.42087	−1.85932	−0.57670

13

∗ E = +8.841 kcal/mol ; (11) 008_067_180

H	+0.00000	+0.00000	+0.00000
O	+0.00000	+0.00000	+0.95956
C	+1.25886	+0.00000	+1.45078
C	+2.35340	+0.18233	+0.40056
N	+2.23243	+1.53697	−0.13763
H	+2.94291	+1.70756	−0.83808
O	+1.45105	−0.12826	+2.61732
C	+3.72164	+0.00089	+1.02754
H	+2.19626	−0.60170	−0.36483
H	+1.33056	+1.72655	−0.55616
H	+4.50331	+0.13291	+0.27388
H	+3.86062	+0.74006	+1.81884
H	+3.82011	−0.99475	+1.46424

4 Gly

4.1 Reference Z-matrix for Gly

Bond distances are given in Å; bond and dihedral angles, in degrees.

h						
o	1	oh2				
c	2	co3	1	coh3		
c	3	cc4	2	cco4	1	dih4
n	4	nc5	3	ncc5	2	dih5
h	5	hn6	4	hnc6	3	dih6
o	3	oc7	2	oco7	4	dih7
h	4	hc8	3	hcc8	5	dih8
h	4	hc9	3	hcc9	5	dih9
h	5	hn10	4	hnc10	6	dih10

oh2	0.96836476
co3	1.36001934
coh3	112.01654315
cc4	1.50425064
cco4	110.29411407
dih4	181.47905327
nc5	1.46331264
ncc5	108.54489116
dih5	47.54914819
hn6	1.00165654
hnc6	114.17559438
dih6	172.28949467
oc7	1.20034302
oco7	122.29217275
dih7	182.02216791
hc8	1.07801507
hcc8	107.39726344
dih8	118.94185156
hc9	1.09015057
hcc9	107.46569489
dih9	235.57927647
hn10	1.00177138
hnc10	112.96035617
dih10	131.26204088

4.2 Reference energy and target torsions for Gly

- Reference M08-HX/MG3S energy: -284.4378457 hartree
- Target torsions:
  - torsion1: dih4
  - torsion2: dih5
  - torsion3: dih6

4.3 Properties of Gly conformers

Table S4: Relative energy (kcal mol^{-1}), rotational constants (B1 to B3, GHz) and dipolar moment (Debye) for the conformers of Gly located at the M08-HX/MG3S level.

Conformer	Rel. Energy	B1	B2	B3	Dip. Mom.
1	0.000	2.945	3.913	10.495	1.282
2	0.709	3.035	4.094	10.191	5.561
3	0.709	3.035	4.094	10.191	5.561
4	1.086	2.986	4.010	10.582	2.063
5	1.086	2.986	4.010	10.582	2.063
6	1.675	2.979	4.032	10.099	1.958
7	2.665	3.055	4.108	9.957	2.477
8	2.665	3.055	4.108	9.957	2.477
9	4.978	2.943	3.938	10.283	3.140

4.4 Vibrational frequencies of Gly conformers

Harmonic vibrational zero point energy (ZPE) and frequencies for each conformer in cm^{-1}.

- Conformer (1); ZPE = 17708.256 cm^{-1}:

88.41	238.30	259.55	471.53	516.95	647.90
661.21	844.61	918.97	938.62	1162.34	1187.62
1189.47	1318.13	1381.04	1422.45	1450.36	1677.49
1869.54	3050.60	3091.38	3554.28	3630.10	3845.65

- Conformer (2); ZPE = 17824.782 cm^{-1}:

79.83	243.18	333.43	515.95	559.42	654.87
801.51	836.47	893.04	948.65	1107.60	1170.42
1250.99	1326.09	1369.76	1426.92	1463.50	1662.38
1900.09	3060.60	3109.89	3577.15	3662.99	3694.82

- Conformer (3); ZPE = 17824.789 cm^{-1}:

79.79	243.18	333.43	515.95	559.41	654.87
801.51	836.47	893.04	948.65	1107.60	1170.42
1250.99	1326.08	1369.76	1426.91	1463.50	1662.38
1900.09	3060.66	3109.93	3577.15	3662.99	3694.82

- Conformer (4); ZPE = 17695.514 cm^{-1}:

103.74	212.68	289.35	477.46	526.57	636.90
671.68	834.41	864.11	1029.91	1133.94	1175.39
1238.17	1260.24	1344.65	1465.31	1489.49	1643.97
1870.91	2985.66	3064.74	3561.53	3661.09	3849.11

- Conformer (5); ZPE = 17695.531 cm^{-1}:

103.73	212.67	289.34	477.46	526.57	636.91
671.69	834.41	864.12	1029.91	1133.94	1175.41
1238.18	1260.24	1344.66	1465.32	1489.49	1643.97
1870.91	2985.66	3064.74	3561.53	3661.09	3849.11

- Conformer (6); ZPE = 17697.183 cm^{-1}:

42.17	261.94	269.77	509.40	520.83	604.60
683.26	819.55	906.39	911.36	1163.35	1192.91
1197.42	1356.94	1380.40	1382.75	1450.51	1673.12
1866.19	3057.48	3098.67	3561.46	3641.97	3841.94

- Conformer (7); ZPE = 17689.499 cm^{-1}:

71.44	228.78	291.67	462.38	549.90	600.27
701.45	826.17	857.06	1026.29	1099.03	1161.51
1240.13	1291.94	1368.12	1437.16	1485.49	1652.49
1876.19	2968.44	3114.39	3565.69	3654.02	3848.98

- Conformer (8); ZPE = 17689.425 cm^{-1}:

71.46	228.72	291.66	462.37	549.90	600.26
701.45	826.15	857.05	1026.28	1099.02	1161.50
1240.13	1291.94	1368.12	1437.15	1485.49	1652.48
1876.19	2968.44	3114.39	3565.69	3654.02	3848.98

- Conformer (9); ZPE = 17643.717 cm^{-1}:

95.20	227.45	263.59	454.67	471.96	567.79
661.69	858.07	923.17	942.48	1164.09	1185.38
1185.81	1301.44	1389.44	1394.80	1457.00	1679.29
1904.72	3024.31	3066.11	3553.31	3627.73	3887.93

4.5 Cartesian coordinates of Gly conformers

Each line starting by an asterisk contains the relative energy of the corresponding conformer and the dihedral angle for each of the target torsions (separated by underscores). Cartesian coordinates are given in Å.

```
10
 * E =  +0.000 kcal/mol ; (1) 180_180_302
 H    +0.00000    +0.00000    +0.00000
 O    +0.00000    +0.00000    +0.96373
 C    +1.27493    +0.00000    +1.38121
 C    +1.37348    +0.00012    +2.89444
 N    +2.71739    +0.00031    +3.41472
 H    +3.22491    −0.80919    +3.07852
 O    +2.20805    +0.00010    +0.63058
 H    +0.82112    +0.87223    +3.26195
 H    +0.82135    −0.87209    +3.26205
 H    +3.22476    +0.80982    +3.07831
10
 * E =  +0.709 kcal/mol ; (2) 002_348_141
 H    +0.00000    +0.00000    +0.00000
 O    +0.00000    +0.00000    +0.97168
 C    +1.27146    +0.00000    +1.36101
 C    +2.28250    +0.04740    +0.21400
 N    +1.65133    −0.20073    −1.07663
 H    +2.02631    +0.38367    −1.80940
 O    +1.60566    −0.01409    +2.50746
 H    +2.71219    +1.05335    +0.23121
 H    +3.09225    −0.64444    +0.46537
 H    +1.74327    −1.16463    −1.36749
10
 * E =  +0.709 kcal/mol ; (3) 358_012_097
 H    +0.00000    +0.00000    +0.00000
 O    +0.00000    +0.00000    +0.97168
```

C +1.27146 +0.00000 +1.36101
C +2.28251 -0.04740 +0.21400
N +1.65133 +0.20073 -1.07662
H +1.74328 +1.16463 -1.36748
O +1.60566 +0.01409 +2.50746
H +3.09225 +0.64442 +0.46536
H +2.71218 -1.05337 +0.23121
H +2.02631 -0.38367 -1.80940
10
* E = +1.086 kcal/mol ; (4) 178_166_030
H +0.00000 +0.00000 +0.00000
O +0.00000 +0.00000 +0.96366
C +1.27388 +0.00000 +1.37727
C +1.38072 +0.05382 +2.87963
N +2.72653 -0.27611 +3.29077
H +3.38623 +0.00369 +2.57412
O +2.20572 -0.01232 +0.62476
H +1.04153 +1.06330 +3.16441
H +0.64825 -0.64671 +3.29636
H +2.97686 +0.16780 +4.16194
10
* E = +1.086 kcal/mol ; (5) 182_194_209
H +0.00000 +0.00000 +0.00000
O +0.00000 +0.00000 +0.96366
C +1.27387 +0.00000 +1.37726
C +1.38071 -0.05382 +2.87963
N +2.72652 +0.27611 +3.29077
H +2.97685 -0.16779 +4.16194
O +2.20571 +0.01232 +0.62475
H +0.64824 +0.64671 +3.29636
H +1.04152 -1.06330 +3.16440
H +3.38622 -0.00371 +2.57412
10
* E = +1.675 kcal/mol ; (6) 180_000_300
H +0.00000 +0.00000 +0.00000
O +0.00000 +0.00000 +0.96408
C +1.28035 +0.00000 +1.36837
C +1.43145 +0.00006 +2.88131
N +0.23451 +0.00010 +3.68763
H -0.33607 +0.81309 +3.49468

```
 O    +2.20045    -0.00004    +0.60179
 H    +2.04905    -0.87063    +3.12522
 H    +2.04904    +0.87082    +3.12510
 H    -0.33593    -0.81306    +3.49501
10
 * E =  +2.665  kcal/mol ; (7) 179_034_183
 H    +0.00000    +0.00000    +0.00000
 O    +0.00000    +0.00000    +0.96364
 C    +1.27755    +0.00000    +1.37616
 C    +1.39109    +0.03587    +2.88155
 N    +0.31218    -0.68987    +3.52563
 H    +0.37572    -0.61334    +4.53154
 O    +2.20292    +0.02184    +0.61777
 H    +2.35394    -0.41248    +3.13487
 H    +1.45349    +1.10572    +3.14646
 H    -0.58954    -0.33231    +3.23676
10
 * E =  +2.665  kcal/mol ; (8) 181_326_057
 H    +0.00000    +0.00000    +0.00000
 O    +0.00000    +0.00000    +0.96364
 C    +1.27755    +0.00000    +1.37615
 C    +1.39110    -0.03588    +2.88155
 N    +0.31218    +0.68983    +3.52565
 H    -0.58954    +0.33220    +3.23685
 O    +2.20292    -0.02184    +0.61776
 H    +1.45355    -1.10572    +3.14645
 H    +2.35395    +0.41251    +3.13487
 H    +0.37578    +0.61338    +4.53156
10
 * E =  +4.978  kcal/mol ; (9) 000_180_302
 H    +0.00000    +0.00000    +0.00000
 O    +0.00000    +0.00000    +0.96022
 C    +1.25534    +0.00000    +1.44723
 C    +2.36035    -0.00027    +0.39517
 N    +3.69999    -0.00058    +0.92185
 H    +3.83978    +0.80794    +1.51630
 O    +1.45682    -0.00023    +2.62129
 H    +2.21436    -0.87707    -0.24939
 H    +2.21476    +0.87659    -0.24938
 H    +3.83953    -0.80935    +1.51603
```

5 Ile

5.1 Reference Z-matrix for Ile

Bond distances are given in Å; bond and dihedral angles, in degrees.

h						
o	1	oh2				
c	2	co3	1	coh3		
c	3	cc4	2	cco4	1	dih4
n	4	nc5	3	ncc5	2	dih5
h	5	hn6	4	hnc6	3	dih6
o	3	oc7	2	oco7	4	dih7
c	4	cc8	3	ccc8	5	dih8
h	4	hc9	3	hcc9	5	dih9
h	5	hn10	4	hnc10	6	dih10
c	8	cc11	4	ccc11	3	dih11
h	8	hc12	4	hcc12	11	dih12
c	8	cc13	4	ccc13	11	dih13
c	11	cc14	8	ccc14	4	dih14
h	11	hc15	8	hcc15	14	dih15
h	11	hc16	8	hcc16	14	dih16
h	14	hc17	11	hcc17	8	dih17
h	14	hc18	11	hcc18	17	dih18
h	14	hc19	11	hcc19	17	dih19
h	13	hc20	8	hcc20	4	dih20
h	13	hc21	8	hcc21	20	dih21
h	13	hc22	8	hcc22	20	dih22

oh2	0.96856641
co3	1.36227708
coh3	111.80082897
cc4	1.51403154
cco4	110.22239137
dih4	181.79617429
nc5	1.47539334
ncc5	104.93831114
dih5	54.58670633
hn6	1.00246262
hnc6	113.13570805
dih6	188.47073406

oc7	1.20111077
oco7	121.52652784
dih7	179.99526403
cc8	1.54035777
ccc8	110.40890837
dih8	121.26096712
hc9	1.09027436
hcc9	106.88858613
dih9	240.08411389
hn10	1.00367509
hnc10	111.78812276
dih10	126.69104962
cc11	1.54271908
ccc11	111.04911447
dih11	195.2294551
hc12	1.08283188
hcc12	107.27462396
dih12	118.46869285
cc13	1.54162751
ccc13	109.26549828
dih13	236.89515521
cc14	1.54212837
ccc14	113.28516631
dih14	177.92324395
hc15	1.08787717
hcc15	109.47660736
dih15	122.36490789
hc16	1.08232476
hcc16	108.48989637
dih16	240.09929684
hc17	1.08456106
hcc17	110.19323336
dih17	187.38484664
hc18	1.08494233
hcc18	110.41945065
dih18	119.29695006
hc19	1.08230133
hcc19	111.75680134
dih19	240.09836964
hc20	1.08193435

```
hcc20    111.04770927
dih20    185.70352059
hc21     1.08050749
hcc21    110.44955758
dih21    119.30312872
hc22     1.08646521
hcc22    110.25924982
dih22    240.02463486
```

5.2 Reference energy and target torsions for Ile

- Reference M08-HX/MG3S energy: -441.7144529 hartree
- Target torsions:
  - torsion1: dih4
  - torsion2: dih5
  - torsion3: dih6
  - torsion4: dih11
  - torsion5: dih14

5.3 Properties of Ile conformers

Table S5: Relative energy (kcal mol^{-1}), rotational constants (B1 to B3, GHz) and dipolar moment (Debye) for the conformers of Ile located at the M08-HX/MG3S level.

Conformer	Rel. Energy	B1	B2	B3	Dip. Mom.
1	0.000	1.134	1.270	2.047	5.066
2	0.209	1.105	1.333	2.007	4.990
3	0.378	0.927	1.120	2.251	5.103
4	0.444	0.988	1.120	2.110	1.309
5	0.455	1.142	1.268	2.010	1.379
6	0.717	0.988	1.132	2.160	5.301
7	0.816	0.974	1.127	2.370	5.028
8	0.849	1.097	1.394	1.876	1.550

9	1.219	1.130	1.263	2.054	1.568
10	1.364	0.848	1.000	2.694	1.401
11	1.407	0.948	1.107	2.487	1.236
12	1.486	0.967	1.178	2.168	1.559
13	1.560	1.169	1.242	2.100	5.140
14	1.671	0.912	1.055	2.594	5.025
15	1.716	0.984	1.131	2.149	2.416
16	1.744	1.101	1.361	1.926	1.499
17	1.793	0.927	1.124	2.526	5.153
18	1.807	1.192	1.245	2.061	1.383
19	1.816	1.101	1.361	1.924	2.236
20	1.834	0.974	1.120	2.141	1.677
21	1.912	0.862	0.988	2.723	1.622
22	1.941	0.962	1.087	2.513	1.778
23	2.106	0.919	1.077	2.568	1.228
24	2.124	0.966	1.130	2.296	2.211
25	2.146	0.929	1.083	2.510	2.050
26	2.157	1.094	1.272	2.039	2.065
27	2.158	1.130	1.264	2.084	1.955
28	2.380	1.042	1.108	2.305	1.394
29	2.456	0.860	1.110	2.613	5.082
30	2.470	0.973	1.116	2.325	1.534
31	2.481	0.911	1.101	2.353	2.028
32	2.625	0.791	0.985	2.866	5.025
33	2.641	0.942	1.103	2.505	2.228
34	2.741	1.107	1.337	1.957	2.329
35	2.804	0.847	0.997	2.700	2.167
36	2.824	1.131	1.542	1.845	1.493
37	2.884	0.917	1.133	2.466	5.239
38	3.007	1.188	1.252	2.065	2.375
39	3.022	0.978	1.133	2.156	2.329
40	3.080	0.881	1.093	2.633	2.261
41	3.081	0.976	1.114	2.332	2.355
42	3.086	1.116	1.455	1.970	4.925
43	3.101	0.882	1.068	2.565	1.406
44	3.166	1.136	1.272	2.037	1.877
45	3.179	0.944	1.093	2.528	2.856

46	3.210	0.907	1.046	2.624	2.136
47	3.256	0.854	0.989	2.715	2.910
48	3.312	1.095	1.279	2.052	1.566
49	3.336	0.800	0.969	2.963	2.264
50	3.367	0.930	1.060	2.420	1.275
51	3.496	1.166	1.245	2.076	1.655
52	3.518	0.902	1.060	2.561	1.623
53	3.672	1.133	1.525	1.869	2.127
54	3.731	0.880	1.042	2.574	1.374
55	3.855	0.887	1.117	2.438	5.097
56	3.918	1.042	1.103	2.308	1.624
57	3.931	1.139	1.520	1.854	1.496
58	3.957	0.913	1.127	2.467	2.326
59	4.264	1.040	1.081	2.352	2.088
60	4.310	0.857	1.112	2.392	4.960
61	4.333	1.079	1.288	2.099	1.435
62	4.483	1.166	1.258	2.066	2.444
63	4.531	1.040	1.263	2.182	5.009
64	4.539	0.888	1.069	2.530	2.154
65	4.761	0.871	1.082	2.485	2.240
66	4.901	1.138	1.503	1.877	2.598
67	4.976	0.899	1.079	2.497	2.901
68	5.147	0.991	1.116	2.103	3.265
69	5.255	1.057	1.121	2.263	2.323
70	5.335	1.143	1.265	1.998	3.305
71	5.447	1.045	1.284	2.160	2.085
72	5.513	1.088	1.334	1.941	3.663
73	5.632	0.960	1.150	2.245	3.484
74	5.699	1.056	1.294	2.121	2.203
75	5.712	0.937	1.118	2.481	3.298
76	5.870	0.843	1.005	2.677	3.371
77	6.030	0.966	1.137	2.291	4.445
78	6.344	1.094	1.311	1.978	4.655
79	6.420	0.988	1.128	2.149	4.477
80	6.460	1.196	1.247	2.055	3.286
81	6.893	0.938	1.114	2.495	4.595
82	6.949	0.933	1.116	2.507	4.187

83	7.130	0.847	1.001	2.682	4.509
84	7.138	1.040	1.111	2.300	3.316
85	7.185	1.097	1.401	1.871	4.339
86	7.405	1.130	1.532	1.853	3.255
87	7.501	0.876	1.083	2.530	3.319
88	7.535	1.187	1.250	2.067	4.214
89	7.667	0.856	0.971	2.754	3.819
90	7.982	1.134	1.513	1.876	4.244
91	8.418	1.074	1.271	2.119	3.833
92	8.878	0.889	1.078	2.503	4.550
93	9.105	0.900	1.045	2.563	3.802
94	9.495	1.039	1.262	2.171	5.014
95	9.735	1.086	1.286	2.112	4.897

5.4 Vibrational frequencies of Ile conformers

Harmonic vibrational zero point energy (ZPE) and frequencies for each conformer in cm^{-1}.

- Conformer (1); ZPE = 42739.366 cm^{-1}:

80.10	97.95	141.71	185.19	231.29	249.61
271.43	291.44	303.65	347.42	376.09	464.25
530.43	537.54	606.81	737.93	797.90	801.47
821.32	858.61	886.89	969.47	1002.52	1018.35
1049.88	1074.51	1094.05	1156.40	1203.62	1215.88
1242.19	1271.63	1290.01	1321.07	1360.44	1384.35
1398.93	1407.00	1414.40	1427.64	1484.43	1486.42
1493.49	1496.23	1504.24	1656.78	1885.34	2999.92
3021.93	3032.39	3034.55	3054.96	3065.72	3097.28
3105.97	3117.11	3129.18	3555.81	3646.91	3688.71

- Conformer (2); ZPE = 42722.137 cm^{-1}:

64.10	92.41	141.80	190.00	218.46	240.66
256.81	275.31	295.69	347.51	406.08	447.94
472.68	551.33	664.30	739.83	793.59	803.71
820.71	858.74	915.10	932.54	978.26	1025.18
1048.32	1097.42	1115.36	1137.45	1201.01	1206.38
1259.10	1281.64	1310.04	1335.93	1358.27	1377.15
1389.04	1405.39	1410.95	1429.30	1478.03	1485.92
1487.99	1494.36	1507.85	1653.75	1884.95	2983.77
3027.65	3031.73	3033.83	3057.92	3078.43	3107.09
3108.28	3120.87	3126.04	3552.85	3637.95	3689.53

- Conformer (3); ZPE = 42670.238 cm^{-1}:

51.63	57.97	91.12	177.98	209.24	220.98
267.75	291.15	312.71	331.69	375.40	428.33
528.76	550.88	634.62	757.53	775.08	808.83
822.95	862.81	892.12	966.31	985.54	1026.95
1034.35	1075.87	1104.85	1168.85	1206.21	1209.77
1245.46	1280.96	1308.54	1327.29	1365.13	1386.95
1398.53	1403.75	1410.07	1431.17	1469.70	1485.37
1490.84	1494.11	1503.19	1657.26	1886.69	3000.72
3024.55	3033.46	3041.61	3048.87	3063.34	3095.06
3115.43	3123.40	3129.89	3553.69	3645.78	3691.41

- Conformer (4); ZPE = 42496.812 cm^{-1}:

40.01	58.57	84.07	166.68	200.53	207.86
223.52	256.79	291.65	319.73	346.99	420.43
453.82	562.42	626.36	656.59	747.49	780.40
826.89	865.56	913.66	971.78	976.85	1006.70
1051.35	1084.21	1104.65	1165.81	1172.95	1209.46
1240.75	1271.15	1302.73	1332.10	1356.20	1373.97
1390.95	1400.75	1404.65	1425.00	1480.13	1482.42
1489.88	1498.66	1501.54	1671.98	1858.56	3012.42
3030.85	3037.50	3041.65	3049.38	3058.45	3111.04
3115.97	3122.44	3131.72	3542.02	3621.43	3843.53

- Conformer (5); ZPE = 42596.368 cm^{-1}:

67.32	97.01	131.69	185.00	213.65	225.84
246.48	258.06	291.19	324.62	338.90	451.81
477.67	534.66	603.65	650.18	719.44	806.12
827.95	865.65	917.00	970.92	987.35	1021.16
1050.45	1082.20	1107.57	1155.05	1173.89	1214.17
1247.69	1281.15	1299.00	1323.52	1363.92	1378.47
1394.74	1397.38	1410.51	1414.25	1482.22	1488.82
1496.13	1497.79	1504.75	1671.21	1855.91	3017.26
3026.58	3033.44	3034.93	3046.41	3068.02	3105.57
3112.07	3118.93	3121.14	3542.72	3619.19	3842.34

- Conformer (6); ZPE = 42631.734 cm^{-1}:

42.79	67.83	88.92	162.49	187.20	206.99
231.47	275.17	316.19	326.43	371.16	429.28
497.99	602.02	645.39	750.78	779.92	811.85
826.34	847.13	921.16	958.40	972.96	1012.16
1039.90	1072.12	1109.38	1168.68	1184.34	1215.86
1260.16	1289.09	1300.47	1338.26	1367.78	1375.49
1394.07	1405.18	1409.78	1432.21	1469.31	1484.34
1489.32	1495.08	1508.46	1655.37	1888.12	3012.63
3025.70	3039.00	3040.05	3045.44	3061.29	3099.14
3113.18	3124.54	3129.02	3568.14	3649.84	3670.69

- Conformer (7); ZPE = 42672.500 cm^{-1}:

61.71	73.72	108.30	169.28	211.36	218.29
266.08	272.27	321.97	348.26	364.55	443.58
483.50	552.24	667.56	761.57	788.10	797.67
826.42	860.32	918.28	934.62	973.84	1010.43
1052.82	1102.73	1115.89	1125.76	1197.86	1209.01
1259.62	1283.49	1299.88	1339.08	1358.86	1372.60
1391.04	1405.69	1408.13	1428.63	1476.82	1487.89
1490.99	1496.89	1508.15	1652.62	1885.31	2980.10
3031.18	3032.51	3035.05	3039.53	3084.89	3106.85
3110.79	3124.12	3131.66	3551.66	3635.91	3697.05

- Conformer (8); ZPE = 42603.082 cm^{-1}:

40.29	101.71	150.36	183.31	201.04	227.38
243.58	261.02	293.90	313.20	384.97	432.58
460.30	533.05	614.92	676.37	733.05	805.51
822.32	853.85	911.04	953.35	976.31	1026.14
1045.27	1091.20	1145.79	1153.75	1173.66	1198.22
1243.33	1268.49	1291.25	1324.38	1344.19	1369.52
1387.12	1404.98	1409.36	1439.19	1478.35	1486.92
1489.81	1499.25	1507.51	1669.80	1855.80	3020.90
3028.36	3037.02	3037.80	3062.51	3076.86	3110.37
3111.15	3123.77	3125.12	3540.87	3620.24	3834.49

- Conformer (9); ZPE = 42655.079 cm^{-1}:

70.20	103.96	143.63	188.06	223.43	248.35
265.54	282.26	297.16	335.06	337.57	465.45
499.13	517.10	607.22	662.91	725.76	806.61
823.17	846.84	906.33	967.12	975.48	1021.36
1049.09	1078.08	1102.55	1158.34	1196.32	1214.59
1233.61	1284.07	1299.22	1323.26	1367.16	1380.08
1387.08	1398.34	1404.53	1413.15	1482.68	1488.88
1495.80	1498.05	1507.20	1667.68	1856.48	3018.16
3023.77	3031.02	3033.89	3048.38	3067.30	3101.78
3112.76	3117.82	3120.93	3551.58	3632.79	3844.03

- Conformer (10); ZPE = 42519.703 cm^{-1}:

54.15	64.64	75.06	156.14	202.62	242.27
262.91	264.95	286.80	325.42	365.70	405.82
431.38	543.19	614.11	670.01	763.56	784.01
854.03	861.16	901.59	966.34	987.43	1004.13
1063.06	1073.02	1141.10	1165.87	1173.16	1189.23
1229.07	1275.91	1298.93	1322.01	1360.20	1368.30
1379.40	1403.93	1410.50	1428.60	1476.36	1486.07
1489.10	1498.01	1505.16	1662.50	1857.26	2997.67
3015.45	3034.41	3038.53	3057.49	3088.54	3107.68
3112.46	3120.69	3130.87	3534.67	3621.43	3835.37

- Conformer (11); ZPE = 42557.602 cm^{-1}:

55.81	63.27	86.83	168.74	215.66	231.92
250.96	255.50	277.42	323.31	376.20	385.21
521.67	534.45	609.06	672.67	756.70	769.01
847.62	854.39	902.71	975.41	996.97	1001.32
1033.16	1068.63	1142.35	1153.64	1171.90	1188.86
1237.62	1286.67	1295.77	1337.13	1362.46	1369.54
1382.36	1404.46	1410.67	1429.39	1473.00	1485.30
1490.84	1495.26	1503.83	1661.33	1858.75	3006.82
3035.83	3038.62	3041.31	3065.29	3093.62	3112.06
3117.75	3119.74	3121.42	3532.69	3617.81	3836.45

- Conformer (12); ZPE = 42552.029 cm^{-1}:

40.73	77.51	108.27	162.35	201.04	220.83
240.44	258.94	293.54	331.48	358.04	411.86
457.31	535.21	627.41	677.15	769.96	786.36
823.37	857.38	916.75	949.00	981.92	1010.27
1052.88	1097.53	1131.16	1156.75	1176.72	1199.53
1243.68	1275.10	1295.96	1307.12	1356.18	1377.23
1384.30	1406.59	1411.60	1436.71	1472.32	1485.01
1489.38	1497.70	1509.12	1666.27	1854.78	3016.72
3027.28	3038.31	3040.64	3061.67	3068.69	3112.08
3116.50	3122.01	3129.75	3537.31	3617.61	3834.77

- Conformer (13); ZPE = 42815.193 cm^{-1}:

52.28	89.92	151.63	198.87	229.46	260.18
278.31	291.53	340.00	353.87	390.87	416.14
539.82	567.31	646.53	747.95	767.53	803.59
824.18	860.83	884.78	970.04	981.81	1016.70
1033.07	1063.50	1104.56	1174.79	1190.96	1218.30
1246.47	1272.69	1299.26	1355.16	1360.70	1381.33
1396.99	1406.85	1410.03	1429.89	1480.00	1489.30
1492.68	1503.45	1508.02	1660.71	1884.68	3020.33
3030.22	3038.61	3039.06	3056.24	3081.77	3099.29
3103.63	3116.33	3118.19	3575.58	3657.97	3665.61

- Conformer (14); ZPE = 42658.239 cm^{-1}:

42.44	80.02	104.44	180.70	198.75	232.13
263.39	272.94	304.59	352.45	367.34	447.08
496.94	565.62	604.48	759.52	786.38	809.66
833.52	860.85	903.80	955.73	975.22	1021.21
1057.30	1087.73	1131.09	1143.95	1197.22	1210.09
1242.44	1284.06	1290.27	1321.24	1369.15	1377.32
1399.15	1404.11	1407.45	1433.22	1476.73	1486.01
1494.38	1496.08	1506.45	1654.57	1882.75	2988.87
3027.15	3031.52	3033.89	3040.38	3092.90	3101.60
3113.16	3122.29	3132.62	3544.26	3639.10	3676.78

- Conformer (15); ZPE = 42476.390 cm^{-1}:

52.53	68.78	89.29	160.89	192.23	199.73
226.85	273.39	304.40	323.06	353.59	425.52
463.53	575.68	617.60	660.17	741.06	780.10
804.95	830.68	894.11	954.87	966.35	1010.70
1045.08	1075.96	1145.78	1167.93	1174.15	1189.36
1254.59	1273.61	1310.36	1312.51	1353.69	1368.58
1393.75	1404.48	1404.75	1448.46	1471.94	1486.46
1490.33	1496.41	1509.65	1633.80	1865.58	3015.75
3019.11	3023.98	3030.16	3037.37	3060.79	3098.91
3110.25	3122.08	3127.40	3557.74	3654.52	3847.46

- Conformer (16); ZPE = 42597.072 cm^{-1}:

38.72	89.91	142.51	184.14	210.29	225.53
255.39	269.69	290.05	311.25	389.61	445.11
455.81	548.89	596.18	653.42	734.64	802.53
820.80	840.62	903.29	937.94	976.20	1025.45
1045.65	1090.51	1129.85	1153.98	1195.75	1204.09
1237.94	1273.83	1304.18	1330.24	1348.71	1382.80
1387.82	1402.33	1405.81	1410.04	1477.07	1485.17
1488.49	1497.97	1509.56	1666.76	1855.04	3022.94
3027.99	3034.79	3035.88	3065.20	3079.40	3108.62
3110.41	3119.62	3126.23	3553.69	3633.24	3844.58

- Conformer (17); ZPE = 42721.983 cm^{-1}:

57.42	76.95	104.24	180.01	205.31	245.48
255.47	289.66	314.47	362.84	393.03	418.37
517.97	565.15	681.47	729.76	771.77	778.67
842.17	855.22	913.57	934.26	992.69	995.31
1026.58	1093.88	1114.11	1141.47	1175.87	1216.72
1267.69	1283.91	1302.69	1331.61	1355.02	1365.39
1388.19	1410.03	1415.17	1419.68	1483.13	1487.77
1492.49	1498.87	1508.01	1656.62	1889.83	3008.77
3027.26	3032.43	3039.62	3045.99	3065.31	3110.27
3116.42	3125.89	3144.75	3559.13	3645.71	3716.41

- Conformer (18); ZPE = 42591.021 cm^{-1}:

65.58	93.53	135.74	154.44	176.99	217.23
244.79	279.99	295.30	309.45	372.63	400.01
481.30	572.91	642.77	659.78	728.93	778.10
812.68	853.07	899.67	964.40	980.80	1010.03
1031.80	1076.83	1118.45	1165.88	1177.32	1202.45
1239.65	1267.65	1298.28	1347.50	1361.81	1374.20
1378.07	1405.16	1409.38	1431.01	1473.81	1486.40
1491.66	1492.72	1515.63	1670.82	1858.16	3021.16
3036.21	3040.38	3043.29	3049.62	3061.16	3114.04
3116.02	3120.78	3136.16	3559.52	3635.88	3843.06

- Conformer (19); ZPE = 42548.133 cm^{-1}:

38.25	97.19	145.52	185.27	200.83	228.59
237.93	264.41	301.17	304.96	387.68	429.28
460.48	529.72	602.45	675.88	732.58	803.94
807.30	869.49	882.21	933.23	970.41	1024.29
1060.03	1108.78	1121.13	1168.99	1179.67	1195.99
1243.59	1270.05	1290.33	1331.10	1343.53	1350.21
1387.13	1406.71	1410.21	1465.56	1478.94	1487.28
1489.50	1490.12	1507.24	1640.19	1862.39	2987.55
3026.29	3034.75	3037.38	3058.81	3064.83	3109.39
3110.91	3121.33	3124.65	3543.80	3635.57	3839.24

- Conformer (20); ZPE = 42506.757 cm^{-1}:

37.52	48.96	78.44	166.38	204.93	213.31
238.56	268.17	294.89	321.27	342.14	419.69
486.51	553.30	627.01	663.10	747.77	776.68
824.22	846.04	910.70	960.21	975.24	1005.16
1050.17	1079.78	1101.87	1173.86	1201.40	1211.89
1228.59	1282.76	1304.54	1329.96	1367.97	1379.73
1385.99	1399.30	1404.35	1407.37	1475.85	1483.55
1490.40	1495.65	1500.22	1665.09	1857.01	3003.74
3035.19	3038.56	3042.01	3044.23	3052.33	3112.96
3116.31	3120.26	3130.08	3554.89	3633.84	3841.63

- Conformer (21); ZPE = 42453.098 cm^{-1}:

50.68	52.06	72.61	146.50	201.79	212.17
255.36	265.58	279.58	315.46	365.56	400.50
445.35	562.70	594.34	636.45	770.85	788.64
828.61	858.27	893.58	946.80	978.81	1002.47
1057.79	1072.28	1132.45	1155.23	1181.67	1202.25
1227.67	1278.61	1305.39	1332.61	1357.05	1372.31
1380.59	1395.36	1403.84	1411.00	1474.87	1485.95
1490.22	1498.79	1505.17	1655.32	1867.13	2996.55
3017.60	3034.56	3037.58	3066.30	3087.39	3106.86
3111.46	3119.35	3132.36	3544.62	3633.55	3849.74

- Conformer (22); ZPE = 42529.396 cm^{-1}:

42.32	65.56	90.93	163.43	202.37	237.91
248.98	259.74	272.83	319.20	367.28	407.76
516.19	544.70	599.90	645.38	766.73	771.29
831.36	844.90	899.50	950.18	997.20	1001.55
1029.19	1068.07	1135.37	1146.13	1178.60	1199.11
1237.42	1294.27	1303.65	1348.77	1364.06	1370.68
1385.71	1399.10	1407.25	1414.19	1470.50	1485.81
1491.10	1493.89	1504.35	1655.50	1864.58	3013.02
3034.10	3038.22	3040.45	3071.58	3088.91	3108.35
3114.40	3117.79	3121.64	3538.94	3627.40	3849.50

- Conformer (23); ZPE = 42557.969 cm^{-1}:

39.07	82.32	97.20	179.86	198.22	237.74
252.46	264.84	283.85	310.02	353.39	429.17
506.42	524.62	622.66	655.73	765.50	796.37
826.85	842.62	905.24	972.99	994.71	1002.75
1030.44	1071.58	1130.49	1139.21	1179.21	1186.17
1239.66	1286.89	1297.21	1337.42	1360.20	1367.34
1399.96	1403.97	1406.71	1423.38	1473.02	1487.65
1490.29	1499.11	1506.87	1650.65	1862.17	3021.05
3035.17	3038.64	3041.33	3050.28	3085.63	3108.64
3117.00	3119.63	3124.28	3534.14	3626.23	3839.72

- Conformer (24); ZPE = 42480.943 cm^{-1}:

27.49	60.87	110.60	162.89	200.14	218.80
244.88	251.28	307.42	321.64	357.49	427.79
459.04	533.35	610.10	675.68	762.85	785.61
806.12	871.55	880.67	934.01	969.98	1018.92
1053.21	1101.69	1134.69	1171.25	1180.18	1191.96
1239.19	1282.63	1289.25	1311.87	1338.95	1370.71
1386.07	1404.95	1410.08	1462.68	1471.81	1483.36
1487.49	1494.94	1503.76	1641.32	1860.52	2988.35
3027.15	3033.09	3034.53	3037.60	3072.04	3107.73
3111.91	3121.13	3128.20	3548.01	3640.16	3840.27

- Conformer (25); ZPE = 42467.589 cm^{-1}:

53.92	71.92	103.93	173.02	190.00	214.15
230.51	269.30	299.60	334.67	356.12	434.43
483.80	534.85	600.60	639.62	748.50	782.50
799.75	860.58	881.24	942.88	968.29	1023.11
1054.39	1096.70	1145.04	1156.71	1185.62	1195.94
1226.54	1281.68	1303.26	1314.16	1338.23	1360.60
1384.56	1400.46	1404.94	1455.57	1478.15	1489.35
1490.52	1497.48	1506.82	1633.54	1860.62	2958.24
3004.01	3025.74	3028.56	3036.38	3085.04	3105.63
3112.31	3120.05	3128.23	3566.05	3663.18	3843.61

- Conformer (26); ZPE = 42507.823 cm^{-1}:

40.43	76.19	131.98	142.33	197.53	223.63
247.36	265.34	288.65	324.90	414.62	426.57
478.69	539.93	594.00	649.38	729.63	792.10
813.61	857.50	877.08	940.02	971.24	1022.36
1060.52	1106.32	1133.69	1158.55	1182.74	1201.58
1228.96	1280.52	1290.27	1333.21	1338.43	1354.12
1385.43	1398.12	1403.72	1457.25	1481.49	1489.41
1491.36	1493.41	1505.69	1635.77	1860.77	3004.05
3010.75	3024.86	3029.19	3035.44	3079.69	3098.79
3104.97	3117.89	3128.05	3562.98	3660.35	3842.26

- Conformer (27); ZPE = 42578.037 cm^{-1}:

68.20	95.22	137.43	187.57	224.21	234.73
263.54	288.93	298.26	340.29	345.62	465.35
504.08	516.73	599.26	640.93	721.85	791.07
817.25	844.66	912.97	946.47	975.72	1020.10
1052.43	1075.15	1123.85	1166.20	1181.76	1205.54
1230.82	1290.16	1313.63	1330.43	1338.93	1366.18
1396.44	1401.43	1406.01	1436.93	1472.80	1485.19
1488.20	1494.82	1511.94	1648.65	1861.48	2925.60
3024.50	3028.60	3035.30	3047.09	3071.09	3097.76
3111.19	3116.00	3118.58	3563.41	3649.90	3847.60

- Conformer (28); ZPE = 42636.136 cm^{-1}:

44.79	70.60	144.00	177.64	212.88	243.60
254.10	283.83	289.13	309.04	354.04	408.04
482.88	576.00	635.87	658.41	717.29	793.36
813.09	867.69	913.15	972.95	984.87	1017.76
1031.20	1094.48	1116.74	1164.02	1171.89	1194.19
1244.08	1273.69	1310.92	1349.87	1371.29	1376.07
1380.09	1403.17	1409.93	1429.49	1478.46	1484.26
1493.18	1496.51	1512.75	1670.29	1857.56	3017.98
3036.39	3042.11	3046.56	3048.24	3062.65	3110.86
3117.82	3125.71	3134.13	3533.10	3613.76	3843.81

- Conformer (29); ZPE = 42719.886 cm^{-1}:

56.51	69.01	123.49	176.82	218.42	243.88
284.93	292.76	338.79	360.12	400.95	449.02
506.74	544.92	620.14	743.54	778.00	789.06
835.07	870.14	889.10	968.39	991.86	1002.79
1034.55	1081.06	1139.54	1145.62	1188.63	1208.88
1224.88	1271.72	1293.52	1328.33	1365.86	1380.83
1389.00	1401.30	1411.16	1425.08	1480.66	1484.22
1490.27	1499.55	1510.95	1659.31	1888.38	3007.42
3022.05	3030.95	3035.05	3038.23	3070.58	3100.11
3105.14	3127.64	3160.84	3539.13	3631.42	3713.48

- Conformer (30); ZPE = 42534.418 cm^{-1}:

40.66	72.46	101.54	166.62	209.41	216.62
235.15	262.90	297.78	324.90	360.30	437.60
454.41	548.75	609.65	652.34	762.85	788.99
826.30	840.96	907.97	936.75	973.12	1009.15
1053.56	1093.03	1120.96	1153.09	1195.01	1201.38
1234.96	1278.30	1298.84	1319.68	1363.93	1381.40
1386.81	1398.36	1404.73	1412.19	1471.70	1485.75
1488.46	1496.11	1508.69	1668.83	1856.34	3024.29
3029.49	3033.74	3035.81	3066.80	3076.06	3102.91
3110.43	3121.52	3128.71	3552.93	3631.74	3845.11

- Conformer (31); ZPE = 42513.539 cm^{-1}:

41.32	63.53	98.67	177.71	198.06	215.06
249.00	292.71	310.35	327.74	357.28	420.39
491.30	540.87	618.19	644.07	740.79	779.10
811.54	847.77	911.89	954.56	967.06	1009.81
1041.60	1075.82	1137.87	1168.56	1185.10	1198.28
1234.09	1289.83	1310.94	1330.58	1347.42	1367.62
1392.69	1403.15	1403.31	1439.14	1476.14	1488.85
1493.33	1496.54	1508.88	1650.08	1865.55	2924.85
3023.21	3032.12	3037.28	3043.90	3081.92	3093.89
3108.13	3118.31	3125.77	3565.13	3650.23	3848.18

- Conformer (32); ZPE = 42658.962 cm^{-1}:

64.49	72.17	115.02	171.98	201.19	242.21
275.45	317.08	341.32	358.15	378.81	413.43
479.61	553.11	609.53	745.58	775.66	783.98
843.86	864.04	891.32	960.89	991.37	1003.50
1066.77	1093.29	1134.46	1172.93	1185.63	1202.16
1225.04	1263.07	1298.92	1334.15	1354.68	1378.72
1390.83	1400.13	1403.64	1423.80	1475.34	1480.35
1488.15	1498.17	1514.14	1657.24	1888.32	2992.28
3003.68	3016.87	3027.24	3040.38	3061.69	3111.53
3115.39	3128.87	3151.06	3537.43	3632.71	3715.09

- Conformer (33); ZPE = 42535.536 cm^{-1}:

51.86	64.06	82.22	170.24	209.17	231.59
251.50	260.80	285.23	330.76	378.02	387.54
520.17	527.74	612.57	670.24	761.90	771.27
827.06	866.73	879.60	942.73	989.23	998.43
1029.33	1112.02	1135.75	1162.88	1174.54	1182.21
1235.61	1267.64	1293.00	1341.62	1348.47	1365.87
1376.91	1409.73	1411.08	1458.13	1481.67	1486.08
1494.38	1497.55	1502.56	1636.51	1865.43	3014.81
3034.51	3037.11	3038.47	3055.97	3069.09	3107.71
3111.74	3119.75	3124.11	3541.45	3637.06	3839.62

- Conformer (34); ZPE = 42536.031 cm^{-1}:

43.83	84.98	138.43	182.83	206.85	230.22
234.53	268.76	298.19	311.17	386.91	439.84
454.93	545.53	592.32	653.38	734.82	798.59
803.36	860.46	876.09	934.20	971.15	1024.06
1061.14	1101.68	1114.81	1141.86	1187.56	1201.22
1246.54	1270.97	1291.19	1331.98	1352.52	1366.99
1386.23	1406.14	1408.95	1443.37	1476.87	1484.72
1487.58	1491.89	1508.85	1649.25	1861.73	2990.33
3025.60	3032.71	3035.16	3065.95	3075.44	3108.34
3108.99	3117.83	3125.40	3550.48	3640.18	3846.19

- Conformer (35); ZPE = 42473.172 cm^{-1}:

44.81	56.72	74.81	151.54	203.28	222.15
237.70	269.74	298.02	329.07	366.36	407.07
424.38	539.72	616.56	666.82	763.25	786.11
827.48	866.34	880.17	939.35	978.01	1000.49
1063.38	1120.93	1138.67	1152.92	1172.61	1187.40
1227.62	1264.32	1301.73	1327.77	1353.80	1365.73
1378.83	1404.25	1408.12	1456.58	1469.84	1482.11
1488.57	1497.89	1509.36	1637.10	1864.74	3003.62
3017.98	3032.88	3041.00	3047.58	3066.06	3108.72
3114.77	3123.54	3133.42	3546.30	3648.44	3837.80

- Conformer (36); ZPE = 42727.189 cm^{-1}:

77.56	94.56	156.20	182.94	212.41	258.25
264.94	298.28	307.91	333.93	352.85	383.84
512.67	552.04	625.99	680.79	734.49	799.40
818.91	857.30	909.71	951.49	997.98	1012.44
1030.59	1106.49	1130.44	1148.06	1168.66	1192.56
1246.65	1280.92	1306.47	1329.98	1356.44	1367.06
1379.61	1409.38	1416.22	1439.58	1478.35	1484.53
1489.37	1505.50	1509.16	1666.01	1853.96	3029.42
3038.55	3042.76	3049.49	3067.36	3071.49	3111.79
3121.53	3125.62	3128.26	3538.42	3620.54	3836.25

- Conformer (37); ZPE = 42748.357 cm^{-1}:

64.01	78.35	127.92	199.08	226.48	271.73
287.11	301.80	323.89	365.67	383.45	410.34
474.27	552.35	682.65	735.45	776.31	787.44
845.90	861.10	907.45	948.37	980.72	1028.82
1054.20	1090.87	1108.86	1142.25	1186.44	1211.04
1263.04	1293.54	1318.32	1325.61	1341.12	1376.04
1382.06	1405.29	1413.28	1422.81	1474.77	1487.19
1495.38	1499.06	1503.82	1655.51	1892.98	2994.17
3018.37	3020.13	3026.20	3040.62	3066.09	3097.37
3116.77	3121.28	3123.36	3556.43	3646.74	3705.06

- Conformer (38); ZPE = 42579.036 cm^{-1}:

74.03	77.33	129.29	153.91	171.69	240.17
249.56	294.02	300.32	318.77	365.27	403.36
487.26	584.09	629.77	673.04	721.94	776.50
803.17	827.79	897.63	943.69	981.00	1013.50
1025.01	1073.59	1142.44	1166.53	1171.65	1188.19
1255.74	1284.80	1302.98	1330.20	1361.78	1372.81
1383.13	1403.66	1412.30	1447.58	1477.53	1487.43
1492.39	1503.01	1507.79	1636.01	1862.62	3011.37
3020.49	3033.05	3036.69	3044.17	3059.47	3104.84
3113.81	3115.89	3130.76	3569.13	3663.31	3848.81

- Conformer (39); ZPE = 42456.303 cm^{-1}:

41.76	57.92	89.04	158.45	195.23	227.02
238.35	277.56	310.76	318.65	344.04	421.39
476.78	565.17	609.31	651.29	741.35	778.57
804.68	836.82	873.35	959.02	963.62	1010.37
1039.56	1076.65	1122.88	1145.44	1189.98	1207.46
1238.68	1275.69	1309.96	1316.87	1364.94	1366.97
1391.35	1400.68	1403.95	1436.78	1470.42	1486.57
1490.59	1494.36	1507.40	1648.36	1861.14	3011.87
3023.14	3029.70	3038.44	3046.20	3059.22	3096.81
3112.05	3121.01	3126.44	3556.75	3648.04	3845.75

- Conformer (40); ZPE = 42587.470 cm^{-1}:

58.93	81.33	117.03	188.73	200.91	231.95
274.77	281.30	288.37	325.12	372.25	455.42
491.79	524.42	597.89	641.23	761.35	783.54
825.48	853.78	916.55	935.06	989.15	997.05
1026.70	1100.89	1137.06	1148.55	1178.65	1189.00
1244.23	1284.44	1305.96	1321.78	1338.55	1365.58
1387.26	1401.95	1409.67	1446.14	1485.25	1488.04
1491.43	1504.68	1508.00	1652.64	1876.29	2938.15
3031.26	3036.22	3038.93	3053.24	3073.96	3103.53
3106.89	3123.14	3139.74	3555.29	3638.72	3849.74

- Conformer (41); ZPE = 42538.194 cm^{-1}:

51.43	79.40	106.06	169.33	214.80	221.26
261.58	275.74	311.36	323.76	359.03	439.04
452.91	551.40	598.72	654.13	764.87	790.06
801.18	862.53	879.29	935.63	970.03	1019.66
1054.47	1095.30	1114.93	1153.91	1180.59	1198.50
1243.38	1276.59	1291.53	1311.97	1360.17	1374.58
1382.57	1404.78	1410.34	1438.82	1472.94	1485.88
1488.88	1495.73	1507.63	1650.80	1864.66	2992.48
3028.64	3029.80	3034.32	3058.24	3074.84	3103.52
3109.11	3121.26	3127.65	3552.76	3643.59	3848.05

- Conformer (42); ZPE = 42815.435 cm^{-1}:

38.36	103.47	155.43	172.36	228.43	278.23
292.64	312.91	322.88	347.62	352.75	421.31
526.50	562.47	676.64	733.94	769.94	798.12
808.49	862.21	914.57	931.54	992.04	1022.56
1032.63	1109.26	1117.38	1133.40	1170.90	1210.30
1270.12	1290.28	1325.96	1347.75	1358.95	1375.75
1382.47	1412.39	1415.92	1425.47	1477.10	1485.06
1490.24	1501.63	1508.28	1654.47	1887.87	2996.70
3033.70	3037.88	3040.31	3049.12	3076.38	3115.24
3118.17	3122.17	3138.70	3547.73	3633.01	3712.77

- Conformer (43); ZPE = 42515.211 cm^{-1}:

54.94	56.25	79.62	160.26	213.14	228.60
249.52	260.80	281.24	326.68	376.09	384.27
464.63	532.06	620.87	671.02	748.62	782.23
852.53	856.45	909.07	980.95	994.77	1038.76
1049.15	1076.12	1138.95	1163.67	1176.19	1185.65
1232.72	1282.40	1302.18	1318.90	1356.59	1378.90
1381.86	1398.09	1409.82	1427.69	1475.51	1484.70
1487.38	1498.09	1504.00	1659.63	1858.99	2999.22
3023.62	3031.21	3036.00	3056.91	3065.13	3106.62
3108.01	3120.18	3136.02	3528.26	3615.14	3833.58

- Conformer (44); ZPE = 42557.386 cm^{-1}:

75.32	90.72	139.45	189.91	203.10	228.71
253.82	278.48	300.10	330.44	342.34	465.43
495.44	531.52	592.85	660.26	721.18	797.23
802.54	846.20	868.59	963.41	976.26	1020.67
1051.03	1076.85	1115.47	1148.84	1193.54	1208.70
1239.92	1269.58	1304.30	1322.42	1343.09	1371.31
1397.29	1401.98	1411.22	1440.26	1479.52	1485.81
1494.63	1496.69	1509.01	1652.47	1853.95	3012.17
3017.04	3023.84	3036.31	3041.59	3062.77	3096.98
3108.88	3117.20	3120.96	3550.16	3640.47	3844.54

- Conformer (45); ZPE = 42516.816 cm^{-1}:

46.55	71.38	88.25	167.25	200.26	231.38
254.25	261.81	277.58	323.14	372.44	403.10
512.59	541.37	592.28	646.15	764.16	771.73
823.07	866.45	869.81	942.33	990.34	998.25
1030.51	1096.45	1118.96	1143.49	1174.83	1198.79
1242.81	1272.91	1296.84	1343.20	1366.59	1369.08
1380.11	1410.78	1412.74	1421.64	1480.60	1487.01
1492.43	1498.48	1501.32	1647.14	1875.44	3010.61
3033.21	3036.63	3038.80	3067.78	3076.72	3106.57
3112.96	3119.67	3123.10	3544.04	3635.58	3849.92

- Conformer (46); ZPE = 42500.413 cm^{-1}:

35.15	82.79	108.21	178.82	198.20	235.95
260.23	264.60	301.78	329.02	351.91	429.44
474.34	555.81	579.72	644.44	744.75	783.84
802.56	861.58	903.71	939.71	970.15	1023.28
1055.00	1097.80	1137.87	1149.43	1179.30	1201.75
1234.46	1283.83	1306.38	1333.88	1340.67	1363.47
1386.64	1396.98	1404.25	1447.37	1480.76	1486.21
1493.84	1499.55	1505.43	1648.46	1866.91	2927.68
2982.70	3024.00	3033.68	3043.02	3086.64	3110.54
3120.31	3124.18	3127.34	3560.93	3650.43	3849.16

- Conformer (47); ZPE = 42453.051 cm^{-1}:

43.17	64.86	73.45	145.85	188.80	215.11
237.55	263.14	301.50	330.91	371.52	409.49
428.87	559.77	593.30	635.61	766.00	790.78
814.00	861.33	874.13	942.56	978.78	997.55
1065.64	1092.72	1128.92	1143.88	1178.97	1199.39
1235.68	1266.45	1302.28	1340.41	1360.15	1366.16
1380.96	1404.55	1410.53	1417.08	1472.19	1484.92
1487.00	1494.92	1510.12	1646.55	1879.47	3000.11
3014.81	3034.23	3039.88	3065.39	3068.11	3108.87
3113.75	3123.43	3131.73	3550.64	3647.59	3850.59

- Conformer (48); ZPE = 42517.845 cm^{-1}:

43.80	65.11	131.61	186.51	203.12	215.97
233.72	271.63	292.12	314.50	406.98	441.47
469.01	550.44	592.36	649.35	735.06	794.59
806.88	855.31	885.38	929.25	973.06	1021.39
1061.15	1106.80	1118.09	1163.25	1175.36	1201.97
1238.96	1279.93	1298.58	1343.22	1348.35	1366.02
1386.39	1400.00	1405.09	1444.30	1476.32	1486.48
1488.85	1493.00	1508.04	1648.72	1854.02	2991.56
3007.93	3026.87	3028.96	3032.88	3074.82	3101.19
3105.65	3120.54	3135.17	3558.14	3648.44	3842.02

- Conformer (49); ZPE = 42596.403 cm^{-1}:

56.73	81.79	122.54	167.08	211.64	243.42
273.27	297.62	333.91	342.26	353.22	402.69
471.32	539.13	592.98	633.14	759.89	790.54
839.83	855.23	923.87	943.77	971.06	1005.25
1064.85	1112.18	1140.91	1156.28	1180.59	1197.46
1237.99	1282.76	1303.97	1319.59	1341.77	1367.56
1381.69	1404.72	1406.34	1445.86	1480.58	1485.18
1490.61	1499.47	1513.81	1656.79	1876.13	2929.20
2992.47	3025.16	3038.11	3041.16	3072.37	3110.75
3113.55	3124.94	3139.56	3558.38	3641.05	3846.84

- Conformer (50); ZPE = 42522.379 cm^{-1}:

31.21	41.72	106.58	168.92	198.99	214.28
245.66	270.26	294.95	331.93	346.58	395.88
445.26	558.35	605.75	687.61	758.61	778.96
817.46	845.20	911.79	949.88	977.79	1011.15
1051.87	1100.81	1121.97	1150.22	1170.98	1207.29
1228.12	1290.89	1306.29	1312.06	1370.50	1378.21
1395.86	1402.80	1405.26	1420.92	1482.28	1487.42
1495.38	1499.37	1506.53	1658.91	1861.70	3012.83
3017.13	3035.91	3041.69	3047.69	3077.42	3110.59
3118.27	3122.18	3132.98	3547.97	3634.04	3845.67

- Conformer (51); ZPE = 42608.196 cm^{-1}:

48.90	92.87	130.87	166.76	205.66	220.20
243.96	283.35	292.69	307.88	369.16	395.89
502.96	582.59	632.08	671.84	724.47	778.11
808.81	834.83	896.23	946.49	978.18	1007.62
1028.80	1071.35	1114.65	1174.03	1194.06	1205.71
1228.81	1281.51	1298.04	1355.51	1364.54	1376.82
1380.64	1401.06	1406.16	1410.14	1473.73	1485.30
1491.42	1493.08	1516.74	1666.41	1858.19	3023.97
3040.10	3041.11	3043.76	3057.39	3063.18	3114.03
3115.71	3118.59	3135.25	3568.26	3652.72	3843.24

- Conformer (52); ZPE = 42474.784 cm^{-1}:

45.96	59.19	75.23	156.86	202.74	219.84
229.33	259.17	290.86	324.39	367.16	381.11
480.11	540.54	605.21	639.41	756.23	788.38
834.66	851.30	900.70	955.94	990.82	1038.76
1047.96	1076.46	1130.91	1158.05	1178.49	1203.62
1232.28	1288.26	1312.30	1324.39	1361.24	1378.61
1383.78	1396.97	1399.09	1410.57	1475.04	1485.13
1487.39	1494.31	1503.12	1654.02	1866.09	3006.60
3024.05	3031.32	3036.39	3063.56	3066.30	3104.48
3108.40	3120.16	3136.10	3536.55	3625.00	3848.66

- Conformer (53); ZPE = 42688.864 cm^{-1}:

70.14	97.91	157.34	181.28	215.11	258.37
273.33	295.35	306.28	314.87	349.46	388.87
517.62	544.63	624.49	684.38	736.59	799.33
807.41	869.60	884.01	934.92	992.14	1022.66
1029.41	1105.60	1138.06	1154.94	1173.39	1189.01
1246.03	1280.26	1315.09	1323.56	1344.04	1364.07
1377.19	1411.31	1416.58	1472.56	1478.25	1482.56
1489.44	1501.04	1506.19	1641.80	1859.46	3006.12
3032.38	3041.20	3050.13	3052.04	3070.16	3115.58
3121.65	3124.92	3127.39	3539.66	3631.17	3839.43

- Conformer (54); ZPE = 42497.459 cm^{-1}:

31.19	68.30	79.74	172.09	213.10	219.11
236.07	267.37	301.87	328.79	364.42	388.46
491.41	507.39	607.79	651.74	754.34	797.99
840.68	852.39	909.54	978.46	989.33	1038.43
1048.87	1073.34	1126.14	1148.88	1179.52	1188.97
1233.27	1278.00	1302.93	1315.98	1356.17	1380.25
1397.34	1400.92	1405.54	1419.80	1474.64	1486.27
1488.08	1498.58	1503.68	1650.93	1861.48	3013.45
3023.50	3030.29	3037.25	3047.68	3063.74	3106.86
3108.92	3125.75	3131.81	3531.74	3623.03	3841.28

- Conformer (55); ZPE = 42703.176 cm^{-1}:

34.78	78.95	121.80	190.75	200.12	238.79
257.24	308.73	337.88	366.40	381.39	387.00
469.88	559.33	675.29	737.00	764.83	782.33
851.99	859.34	923.55	936.79	989.62	1037.50
1047.69	1091.34	1115.96	1154.24	1177.30	1215.09
1261.57	1284.26	1310.37	1319.72	1341.76	1378.36
1386.02	1411.83	1413.56	1418.60	1486.80	1488.04
1489.52	1495.81	1507.45	1658.76	1890.40	2997.14
3014.47	3019.45	3037.14	3038.12	3068.43	3110.16
3111.09	3120.33	3144.59	3551.42	3637.84	3720.47

- Conformer (56); ZPE = 42652.563 cm^{-1}:

38.57	64.83	149.55	173.41	209.21	253.41
276.00	284.36	294.07	324.18	350.15	413.67
510.65	570.02	634.29	655.25	719.52	793.24
808.55	851.78	907.18	956.96	985.99	1017.48
1027.46	1088.09	1112.60	1163.90	1192.96	1199.84
1232.74	1283.01	1305.37	1358.68	1369.50	1373.48
1381.41	1397.31	1406.90	1414.66	1478.27	1483.55
1492.45	1496.00	1512.67	1668.85	1858.69	3010.64
3036.09	3046.36	3050.40	3051.66	3065.61	3110.66
3120.01	3124.19	3133.26	3547.05	3625.63	3842.84

- Conformer (57); ZPE = 42684.215 cm^{-1}:

70.60	78.96	149.42	160.04	205.93	260.48
264.70	295.61	306.41	327.82	359.29	380.25
527.08	553.47	609.44	663.06	736.21	791.68
818.00	840.66	899.15	936.07	995.51	1012.57
1028.61	1102.74	1121.30	1142.77	1175.53	1209.97
1240.86	1291.61	1325.66	1335.37	1360.48	1371.27
1391.06	1399.18	1408.75	1416.23	1475.33	1484.10
1489.48	1503.34	1506.64	1661.98	1857.81	3027.68
3039.93	3041.69	3051.22	3071.01	3076.59	3113.05
3121.37	3126.20	3131.21	3549.04	3632.71	3844.27

- Conformer (58); ZPE = 42647.197 cm^{-1}:

62.31	80.93	128.82	189.76	221.52	252.19
286.10	297.02	324.68	343.05	367.52	410.61
458.84	527.55	611.76	674.25	745.15	782.57
839.59	870.26	903.95	929.74	983.11	1031.38
1052.67	1105.51	1143.98	1173.20	1177.42	1187.19
1236.33	1270.31	1317.54	1321.89	1340.06	1361.01
1376.52	1405.11	1407.81	1455.48	1474.15	1490.11
1498.00	1500.39	1509.49	1650.60	1862.55	3007.62
3017.24	3025.85	3030.76	3033.90	3061.16	3099.68
3112.34	3115.23	3116.41	3545.34	3649.49	3839.41

- Conformer (59); ZPE = 42575.529 cm^{-1}:

40.30	61.45	113.57	184.10	225.26	251.81
266.52	292.63	314.54	333.52	358.81	423.76
487.79	549.96	626.83	639.96	722.36	778.74
794.39	849.75	922.89	948.05	980.44	1014.24
1021.26	1086.69	1132.57	1158.50	1185.10	1205.98
1225.54	1293.29	1319.36	1332.27	1368.67	1374.37
1380.59	1402.71	1405.65	1442.46	1475.80	1487.21
1488.33	1503.82	1510.53	1652.36	1866.29	2908.41
3027.66	3030.12	3042.28	3055.37	3070.99	3102.86
3110.32	3111.11	3135.09	3561.65	3646.50	3847.68

- Conformer (60); ZPE = 42673.971 cm^{-1}:

67.68	71.59	105.38	191.69	217.46	234.50
248.72	325.87	349.27	370.84	388.34	407.86
481.12	542.24	603.77	745.42	760.97	773.28
841.43	866.57	886.40	966.82	998.46	1034.56
1055.11	1097.12	1123.68	1164.72	1190.93	1202.00
1221.46	1262.36	1301.85	1314.96	1374.00	1378.11
1390.95	1399.26	1406.30	1424.04	1479.09	1487.85
1491.11	1498.38	1504.25	1660.95	1889.66	2997.25
3003.08	3020.88	3029.70	3041.30	3058.88	3103.38
3113.12	3122.62	3153.82	3544.11	3635.33	3726.09

- Conformer (61); ZPE = 42606.417 cm^{-1}:

54.84	64.46	109.54	191.53	207.68	240.85
247.85	270.88	281.18	331.05	360.01	404.63
485.82	563.41	623.92	672.61	739.53	786.69
833.34	863.27	910.56	961.52	987.66	1011.60
1032.40	1106.49	1134.33	1165.44	1169.98	1195.29
1228.91	1282.14	1305.59	1343.18	1350.48	1371.88
1380.62	1408.40	1416.81	1428.38	1475.37	1487.39
1493.95	1495.37	1505.56	1669.89	1857.37	3020.46
3036.31	3042.63	3045.70	3046.48	3061.87	3112.03
3116.06	3120.71	3125.33	3529.56	3607.91	3838.16

- Conformer (62); ZPE = 42603.362 cm^{-1}:

60.55	97.16	127.91	170.34	207.21	233.92
255.39	287.51	290.42	325.26	364.82	395.59
502.89	577.28	622.75	672.01	720.50	777.80
799.45	847.50	870.59	952.63	975.33	1012.31
1020.99	1071.03	1117.81	1153.34	1174.00	1216.78
1239.35	1277.99	1299.33	1350.92	1364.13	1372.52
1382.24	1400.19	1408.25	1438.83	1477.92	1490.64
1491.59	1497.56	1513.87	1652.22	1862.88	3023.19
3032.62	3035.79	3042.74	3053.45	3062.28	3104.85
3113.35	3117.47	3121.49	3570.93	3661.15	3845.92

- Conformer (63); ZPE = 42735.781 cm^{-1}:

53.88	74.07	122.59	202.72	221.89	262.15
270.36	285.57	300.03	339.31	390.71	459.97
504.80	566.50	623.56	751.73	778.29	799.45
828.86	861.14	900.44	960.76	995.25	1023.07
1036.26	1093.31	1127.76	1151.09	1185.04	1206.27
1236.34	1291.19	1313.44	1339.01	1370.75	1377.03
1403.08	1408.73	1419.35	1433.29	1471.74	1480.78
1490.09	1491.64	1500.97	1656.30	1886.74	3000.12
3015.65	3026.83	3030.98	3050.08	3053.43	3101.97
3123.69	3133.26	3145.63	3532.95	3630.84	3678.82

- Conformer (64); ZPE = 42492.248 cm^{-1}:

48.83	52.07	104.11	158.57	195.14	230.42
237.36	265.54	319.29	324.08	373.45	379.59
461.33	523.44	617.88	667.17	755.63	785.90
828.36	864.69	876.21	950.42	993.03	1030.88
1050.42	1119.87	1135.89	1151.03	1170.97	1187.41
1229.66	1265.44	1303.27	1320.24	1350.20	1371.01
1383.26	1403.83	1407.59	1458.86	1479.83	1487.09
1490.04	1492.82	1501.32	1639.31	1864.46	3006.05
3014.24	3030.68	3040.10	3051.16	3060.69	3106.06
3113.45	3120.26	3122.38	3539.27	3637.43	3835.52

- Conformer (65); ZPE = 42573.239 cm^{-1}:

59.82	78.18	144.20	174.57	214.07	229.45
251.55	309.20	318.57	332.51	379.04	389.15
486.84	505.59	593.24	635.11	755.46	782.63
838.04	854.51	926.25	943.90	983.51	1039.37
1049.25	1113.43	1130.10	1152.38	1183.93	1191.22
1239.82	1277.73	1306.40	1316.11	1329.53	1375.14
1386.40	1400.44	1407.53	1446.25	1484.29	1486.59
1492.75	1498.86	1509.00	1654.33	1876.92	2928.14
2999.92	3027.22	3040.94	3042.66	3056.21	3105.40
3111.63	3131.64	3136.23	3550.46	3633.57	3849.27

- Conformer (66); ZPE = 42644.546 cm^{-1}:

60.27	86.42	157.26	160.32	205.50	261.63
271.52	290.29	307.48	321.59	356.38	383.29
532.07	543.70	604.02	664.55	735.35	793.35
802.43	861.28	876.70	937.37	991.74	1020.26
1033.90	1100.24	1110.94	1141.30	1178.01	1194.96
1247.71	1286.06	1322.05	1322.54	1361.40	1367.16
1378.75	1410.58	1415.34	1437.31	1476.42	1483.16
1489.12	1499.78	1504.13	1651.46	1868.99	3004.39
3031.68	3041.23	3050.73	3066.90	3070.48	3113.34
3121.77	3125.74	3130.44	3545.26	3635.43	3845.61

- Conformer (67); ZPE = 42509.002 cm^{-1}:

41.68	67.10	106.11	160.54	196.95	225.89
236.80	275.75	323.85	342.34	370.16	378.98
470.08	538.16	597.94	638.83	758.68	787.90
829.51	864.20	870.97	948.22	993.20	1033.39
1053.02	1097.88	1127.08	1140.15	1176.48	1198.77
1237.58	1265.08	1308.23	1318.80	1366.22	1369.19
1380.33	1405.20	1410.64	1420.61	1482.64	1489.14
1490.37	1492.20	1500.70	1650.20	1876.65	3003.60
3014.02	3031.04	3039.39	3060.98	3062.01	3106.27
3112.69	3119.97	3122.14	3544.60	3637.95	3848.95

- Conformer (68); ZPE = 42453.425 cm^{-1}:

45.10	61.35	88.46	168.83	191.58	207.91
217.53	256.70	294.05	322.51	348.21	422.09
453.93	510.60	593.16	663.46	750.36	779.31
823.04	872.68	914.76	968.71	976.86	1007.90
1050.91	1084.48	1106.07	1165.19	1171.51	1210.99
1240.25	1265.35	1300.40	1329.93	1340.33	1365.25
1387.16	1401.36	1405.67	1421.35	1480.36	1482.45
1490.49	1499.28	1501.03	1673.75	1891.57	2986.91
3016.24	3020.08	3038.09	3041.99	3063.94	3111.43
3116.19	3125.07	3135.05	3539.13	3618.39	3890.13

- Conformer (69); ZPE = 42612.329 cm^{-1}:

44.31	59.96	129.33	175.93	219.17	248.22
268.78	306.60	309.51	326.88	347.40	410.02
511.11	574.82	624.21	659.26	715.18	784.72
801.48	840.37	870.21	954.35	984.77	1015.82
1020.86	1082.47	1125.24	1154.32	1161.92	1212.91
1242.57	1284.47	1306.23	1347.63	1369.16	1371.90
1379.38	1403.30	1410.09	1437.77	1476.95	1485.69
1488.48	1495.95	1507.86	1651.94	1863.64	3025.32
3030.06	3036.14	3041.07	3048.99	3066.16	3101.76
3113.37	3119.66	3128.30	3556.33	3648.85	3845.50

- Conformer (70); ZPE = 42537.238 cm^{-1}:

62.18	100.05	127.70	189.22	218.27	222.78
247.85	255.50	292.91	319.83	341.09	450.74
458.68	496.50	562.18	628.18	727.18	806.67
826.38	874.23	921.54	966.83	989.58	1022.46
1053.60	1084.95	1109.49	1156.01	1170.41	1217.19
1245.27	1275.91	1299.62	1305.66	1340.15	1383.40
1386.39	1397.30	1414.66	1418.50	1482.84	1488.34
1495.14	1497.80	1505.22	1671.48	1889.50	2986.46
3000.28	3030.50	3036.50	3036.93	3069.66	3109.51
3113.75	3123.84	3124.33	3541.17	3616.34	3887.85

- Conformer (71); ZPE = 42583.705 cm^{-1}:

51.96	74.33	123.14	194.28	222.20	247.64
264.85	280.75	281.36	344.30	368.19	447.12
486.34	550.89	615.20	648.16	732.70	780.63
802.86	846.79	894.12	930.29	987.60	1021.16
1032.58	1100.09	1146.44	1161.87	1185.90	1193.24
1229.21	1298.14	1304.43	1334.02	1352.97	1363.70
1377.26	1403.20	1416.77	1454.13	1472.62	1489.29
1494.44	1495.54	1503.25	1633.70	1859.09	2939.67
3013.55	3032.63	3036.49	3045.75	3055.11	3112.44
3117.47	3122.58	3127.62	3562.21	3659.47	3843.69

- Conformer (72); ZPE = 42529.002 cm^{-1}:

44.67	93.20	151.63	177.19	202.05	221.68
224.85	254.82	300.18	310.01	393.12	430.82
466.70	513.60	536.19	671.12	735.78	801.18
819.81	861.42	912.99	954.09	970.62	1022.47
1043.18	1088.00	1141.70	1158.72	1171.51	1195.97
1243.25	1269.38	1288.29	1318.85	1333.27	1364.16
1388.65	1411.21	1413.84	1430.35	1479.30	1486.90
1495.11	1497.83	1503.89	1674.01	1887.85	3021.17
3024.33	3031.67	3037.98	3041.34	3064.12	3091.22
3111.15	3124.65	3126.28	3538.45	3615.39	3874.86

- Conformer (73); ZPE = 42550.108 cm^{-1}:

39.63	79.62	112.84	165.88	207.60	226.45
237.31	262.71	306.53	329.38	357.83	419.76
466.63	525.78	538.67	676.84	768.63	799.79
824.62	869.48	925.12	956.90	978.76	1010.62
1053.52	1096.16	1135.51	1160.40	1174.29	1204.85
1244.37	1276.44	1292.87	1307.80	1341.98	1375.31
1378.92	1406.53	1413.28	1427.12	1478.42	1486.72
1490.75	1501.28	1506.03	1674.80	1888.06	2983.49
3020.35	3028.10	3038.29	3042.02	3050.03	3115.02
3117.83	3124.97	3133.04	3540.55	3617.33	3886.46

- Conformer (74); ZPE = 42586.676 cm^{-1}:

32.82	83.75	135.69	198.37	212.99	254.79
270.91	290.45	313.38	326.04	344.94	417.69
497.68	553.71	603.14	656.57	741.82	774.88
798.11	852.14	918.53	933.96	988.59	1020.64
1034.74	1093.85	1139.13	1144.96	1178.79	1189.62
1234.78	1294.44	1308.85	1328.99	1358.44	1368.01
1387.77	1403.23	1416.37	1450.76	1473.64	1486.74
1489.80	1492.41	1503.79	1651.76	1876.06	2920.49
2995.41	3018.69	3043.63	3045.61	3058.19	3114.61
3115.78	3131.69	3143.83	3558.54	3648.04	3850.35

- Conformer (75); ZPE = 42539.898 cm^{-1}:

56.89	72.64	93.85	184.28	216.80	236.44
255.21	267.26	286.87	329.87	379.62	388.79
504.50	519.21	545.56	693.46	727.49	768.06
844.68	867.30	907.62	976.71	996.21	1000.30
1031.57	1068.01	1141.90	1157.24	1165.90	1191.23
1235.24	1288.18	1295.38	1330.16	1358.89	1368.22
1374.37	1403.14	1406.75	1417.93	1473.82	1488.27
1494.50	1499.48	1506.93	1666.10	1890.73	3000.83
3015.54	3024.87	3037.72	3040.41	3095.10	3099.86
3115.73	3121.71	3122.45	3531.03	3611.86	3889.16

- Conformer (76); ZPE = 42470.507 cm^{-1}:

55.66	62.47	69.19	165.78	205.79	236.16
251.27	268.49	292.34	333.78	365.67	414.53
427.78	518.86	550.08	690.15	742.64	780.16
857.92	864.29	903.80	963.81	984.60	1003.71
1061.68	1070.88	1141.46	1163.58	1172.97	1187.05
1227.24	1276.85	1297.40	1322.86	1349.30	1361.48
1376.81	1404.86	1409.60	1414.05	1478.39	1487.81
1492.06	1499.77	1509.29	1664.72	1889.14	2994.81
3004.38	3011.38	3021.06	3039.43	3093.29	3101.06
3114.42	3121.91	3131.37	3530.91	3615.25	3893.56

- Conformer (77); ZPE = 42500.227 cm^{-1}:

47.82	76.04	121.03	165.06	199.55	224.87
240.96	259.19	308.14	330.57	361.07	428.35
470.80	538.60	540.91	681.25	769.12	795.12
804.32	868.01	884.63	934.23	972.61	1017.82
1054.28	1104.07	1133.88	1176.12	1189.90	1197.74
1240.73	1286.69	1292.11	1310.01	1328.39	1368.16
1381.96	1406.10	1411.44	1447.62	1477.81	1488.22
1489.60	1500.57	1502.42	1643.20	1892.98	2973.57
2991.03	2995.99	3037.13	3040.72	3050.98	3111.82
3115.61	3124.11	3130.93	3544.90	3637.19	3882.41

- Conformer (78); ZPE = 42520.163 cm^{-1}:

50.57	93.62	141.17	176.44	211.07	233.06
237.17	259.97	298.56	323.22	394.89	430.55
468.58	520.69	534.77	678.01	736.40	795.87
809.34	866.12	886.51	929.05	966.02	1022.60
1054.10	1109.42	1120.69	1173.73	1186.20	1196.59
1240.40	1273.24	1290.48	1320.83	1335.76	1351.97
1388.94	1410.42	1414.49	1450.56	1481.28	1486.69
1489.21	1495.45	1501.19	1643.79	1892.75	2990.18
3006.36	3026.12	3030.90	3036.05	3063.28	3093.33
3109.79	3123.87	3124.78	3544.83	3637.41	3880.99

- Conformer (79); ZPE = 42426.249 cm^{-1}:

50.83	68.97	94.59	161.42	196.91	212.63
230.60	275.26	309.15	325.69	356.97	428.21
460.39	492.79	605.81	652.85	744.42	779.51
796.18	826.27	900.47	952.42	964.39	1011.54
1046.43	1075.95	1143.90	1167.21	1182.96	1194.04
1247.10	1269.60	1309.03	1321.08	1339.28	1368.03
1389.14	1403.80	1405.70	1433.08	1471.83	1486.90
1490.36	1496.01	1509.88	1634.07	1895.38	2965.93
2985.86	3025.25	3029.38	3036.88	3067.55	3101.19
3109.75	3124.19	3129.04	3553.95	3654.80	3889.66

- Conformer (80); ZPE = 42569.448 cm^{-1}:

68.99	97.98	135.31	161.96	186.63	227.88
243.91	286.68	294.01	311.29	374.81	400.79
477.99	494.22	629.50	655.29	734.58	776.91
811.93	867.61	902.50	965.40	980.39	1012.28
1033.40	1077.79	1120.48	1164.00	1179.61	1202.66
1239.93	1262.21	1304.23	1331.25	1354.41	1369.96
1379.42	1402.84	1411.49	1426.38	1473.73	1485.85
1491.12	1491.67	1516.63	1675.39	1890.61	2984.22
3019.53	3027.43	3041.78	3044.45	3065.10	3113.67
3120.07	3122.86	3142.10	3556.65	3631.70	3885.42

- Conformer (81); ZPE = 42534.451 cm^{-1}:

51.16	70.48	90.99	182.09	230.14	246.33
254.96	263.75	296.73	335.58	380.98	393.12
518.35	518.84	544.38	690.82	736.85	772.64
826.19	864.36	886.46	936.51	988.61	997.88
1027.81	1115.69	1138.18	1166.55	1177.14	1182.99
1236.38	1264.06	1292.16	1332.64	1342.80	1362.77
1373.31	1407.12	1410.59	1437.13	1485.54	1491.00
1496.45	1499.15	1506.38	1636.86	1893.81	2985.54
3019.40	3027.98	3035.96	3039.88	3070.15	3098.29
3108.26	3120.72	3123.50	3544.91	3641.01	3898.58

- Conformer (82); ZPE = 42474.654 cm^{-1}:

52.84	67.15	95.64	177.40	212.36	252.38
255.49	282.00	325.30	356.54	387.38	401.08
498.39	532.70	563.33	608.92	701.57	730.49
768.69	844.16	887.17	927.41	994.23	1002.66
1025.67	1065.59	1132.16	1152.54	1178.88	1202.48
1239.99	1282.74	1297.08	1339.93	1348.42	1364.80
1371.56	1383.45	1406.13	1416.43	1476.33	1485.93
1491.61	1494.30	1506.12	1640.22	1890.78	2976.81
3036.65	3042.33	3045.54	3053.12	3077.98	3115.39
3121.11	3123.61	3135.76	3569.45	3676.33	3856.79

- Conformer (83); ZPE = 42453.617 cm^{-1}:

50.82	57.13	70.89	156.19	209.21	220.94
261.05	274.22	301.92	336.45	369.99	414.99
424.23	513.31	550.61	687.14	750.88	778.03
826.66	869.04	883.79	935.67	976.01	999.87
1062.70	1124.39	1141.21	1155.77	1173.55	1187.40
1228.50	1261.58	1299.80	1327.11	1343.04	1361.39
1378.03	1404.46	1407.56	1436.69	1470.34	1486.01
1488.44	1497.45	1512.71	1638.34	1894.26	2978.42
3006.62	3014.90	3023.94	3042.65	3067.59	3103.00
3116.88	3125.49	3133.53	3546.53	3647.79	3900.11

- Conformer (84); ZPE = 42601.572 cm^{-1}:

49.36	75.08	145.87	181.83	215.97	243.49
257.93	284.99	290.83	310.72	354.61	408.63
483.64	489.31	628.85	653.22	721.20	793.77
808.98	875.88	917.22	975.20	981.39	1019.82
1031.64	1097.19	1119.88	1163.71	1171.51	1195.33
1244.37	1271.10	1307.97	1336.41	1356.82	1375.41
1384.34	1403.32	1407.61	1428.21	1478.45	1485.33
1493.87	1496.38	1513.01	1671.68	1888.61	3000.36
3012.92	3026.43	3037.13	3049.67	3065.89	3110.74
3119.52	3128.05	3136.66	3529.34	3610.67	3885.84

- Conformer (85); ZPE = 42488.079 cm^{-1}:

64.78	86.12	119.92	162.95	207.58	220.84
239.01	288.47	300.62	313.40	353.41	436.31
464.73	503.32	605.11	654.80	724.03	790.62
793.92	829.64	905.42	949.95	973.42	1032.85
1049.80	1072.47	1130.79	1167.74	1185.12	1191.76
1248.01	1266.59	1306.50	1312.93	1333.34	1360.61
1392.28	1403.17	1413.80	1432.27	1478.73	1483.86
1493.88	1500.51	1500.77	1636.81	1892.86	2965.66
2989.82	3027.52	3030.38	3041.35	3066.76	3102.76
3113.95	3118.06	3136.07	3559.68	3661.58	3886.76

- Conformer (86); ZPE = 42648.145 cm^{-1}:

67.61	99.52	162.35	180.01	207.76	235.13
265.45	298.45	310.41	323.74	355.90	389.90
501.85	521.73	563.06	687.73	736.27	803.55
812.27	861.79	911.42	950.56	996.99	1008.77
1029.71	1101.78	1128.98	1151.56	1163.45	1190.32
1245.38	1279.54	1301.98	1321.11	1346.78	1362.68
1376.33	1410.18	1419.71	1428.66	1480.31	1485.68
1493.99	1505.54	1509.06	1668.28	1884.21	3000.79
3014.54	3039.37	3041.86	3052.62	3061.33	3115.05
3122.47	3130.35	3135.01	3534.93	3615.67	3894.87

- Conformer (87); ZPE = 42473.846 cm^{-1}:

54.57	61.81	80.44	174.56	212.86	228.34
255.26	266.14	283.68	330.13	375.01	392.06
462.06	504.59	542.47	690.98	731.02	772.49
851.37	868.41	912.36	980.07	994.03	1038.33
1047.91	1074.13	1140.25	1161.97	1174.67	1187.03
1229.17	1283.72	1301.47	1318.73	1350.75	1363.64
1381.93	1398.61	1405.32	1414.72	1476.27	1487.41
1490.57	1501.67	1504.87	1664.43	1889.54	2993.15
3007.48	3016.71	3024.84	3037.38	3064.69	3097.41
3109.60	3123.64	3137.94	3526.32	3609.04	3887.68

- Conformer (88); ZPE = 42531.255 cm^{-1}:

53.14	80.78	127.31	141.16	173.64	229.83
255.61	288.13	301.22	327.27	370.58	409.43
490.91	504.42	641.49	646.86	730.34	776.85
808.04	828.92	910.52	941.14	979.52	1014.86
1026.74	1073.50	1141.78	1173.17	1180.92	1200.48
1250.11	1286.91	1311.02	1324.43	1361.91	1373.27
1376.58	1404.10	1411.36	1425.86	1476.65	1486.89
1493.77	1501.27	1505.67	1648.68	1891.71	2950.11
2992.78	3025.04	3037.84	3045.73	3062.75	3110.54
3116.89	3119.28	3134.83	3560.30	3660.64	3887.06

- Conformer (89); ZPE = 42375.895 cm^{-1}:

43.67	53.82	77.39	156.48	196.90	207.64
227.95	254.99	284.19	312.55	360.09	398.27
452.41	499.46	565.18	652.83	770.24	783.35
822.33	854.83	889.36	946.39	980.91	1002.41
1055.98	1073.43	1128.38	1148.76	1179.25	1214.95
1229.56	1275.05	1302.77	1333.15	1338.32	1354.40
1378.61	1398.34	1405.23	1414.64	1477.80	1486.61
1490.62	1498.44	1505.97	1653.92	1895.35	2935.25
3023.14	3036.27	3039.32	3066.47	3092.01	3107.87
3113.14	3124.29	3135.22	3540.41	3631.53	3873.68

- Conformer (90); ZPE = 42618.467 cm^{-1}:

60.37	100.73	163.45	178.41	211.65	266.01
277.05	301.71	308.29	317.23	353.09	393.91
485.37	523.07	556.83	694.37	737.66	798.20
803.83	869.29	886.55	931.32	990.97	1021.07
1026.19	1107.07	1135.37	1160.75	1170.89	1193.03
1244.43	1277.81	1304.64	1318.90	1328.31	1361.94
1371.54	1411.98	1419.40	1458.53	1479.92	1482.90
1493.42	1498.99	1506.97	1643.93	1889.12	2986.46
3000.29	3009.85	3042.47	3052.62	3060.78	3117.93
3123.05	3127.28	3133.92	3537.26	3628.98	3899.60

- Conformer (91); ZPE = 42628.966 cm^{-1}:

64.12	75.11	138.02	200.63	209.19	244.75
258.86	279.84	303.23	341.61	368.09	413.52
489.23	532.53	570.79	673.91	738.70	780.04
831.15	870.36	913.53	967.78	987.12	1008.50
1031.32	1099.18	1138.18	1168.10	1177.97	1197.47
1227.23	1289.68	1303.16	1333.73	1347.27	1373.19
1377.83	1403.05	1413.24	1419.85	1475.58	1490.29
1497.25	1505.07	1520.93	1672.32	1888.46	3006.95
3011.18	3030.28	3038.61	3051.44	3067.42	3088.88
3113.33	3118.10	3120.79	3528.57	3604.87	3866.55

- Conformer (92); ZPE = 42459.699 cm^{-1}:

47.13	55.59	107.08	166.06	200.19	239.92
244.23	284.26	314.70	328.18	377.58	380.49
461.83	502.43	536.83	687.63	745.10	777.39
827.41	864.18	882.36	944.16	993.04	1030.07
1049.51	1124.01	1137.69	1153.13	1171.65	1186.40
1229.41	1261.07	1301.01	1320.00	1337.87	1360.78
1382.38	1404.42	1406.15	1439.80	1484.05	1487.25
1491.26	1496.90	1503.03	1637.98	1894.84	2981.96
3008.40	3014.89	3024.07	3040.62	3060.50	3097.15
3113.96	3120.32	3123.28	3540.10	3638.73	3897.03

- Conformer (93); ZPE = 42439.073 cm^{-1}:

36.88	64.24	87.33	168.76	218.34	222.18
242.17	253.52	289.75	326.14	364.27	385.22
480.95	496.97	537.09	655.91	759.31	782.47
833.62	849.11	897.73	957.88	992.69	1039.71
1048.10	1079.67	1128.13	1152.45	1180.29	1214.11
1233.92	1284.07	1305.36	1318.74	1340.12	1366.80
1384.50	1398.81	1404.21	1414.40	1476.72	1488.10
1489.17	1496.70	1504.11	1654.74	1891.70	2940.35
3028.43	3031.82	3037.84	3062.81	3071.15	3106.34
3111.31	3120.11	3138.02	3533.12	3623.31	3876.37

- Conformer (94); ZPE = 42623.247 cm^{-1}:

63.41	78.62	139.49	199.74	217.56	254.31
260.03	289.55	311.91	368.78	383.69	456.52
489.44	550.84	572.72	643.51	741.63	776.81
807.36	849.20	895.50	929.17	985.24	1016.50
1030.51	1097.76	1145.60	1161.13	1188.74	1198.10
1234.13	1298.51	1307.48	1317.11	1345.17	1363.96
1375.26	1401.35	1409.77	1437.36	1475.27	1493.04
1495.69	1513.10	1527.42	1628.86	1886.70	2921.89
3008.10	3025.82	3037.42	3046.22	3063.01	3086.12
3113.93	3115.75	3118.09	3560.56	3659.18	3876.83

- Conformer (95); ZPE = 42610.933 cm^{-1}:

62.08	83.28	138.77	193.35	213.22	233.08
275.15	295.79	311.93	345.63	377.66	417.27
502.66	521.56	565.29	682.74	745.68	781.50
807.39	853.33	892.83	922.45	983.96	1013.09
1029.97	1095.51	1148.45	1167.25	1182.03	1187.99
1240.24	1296.06	1299.66	1325.32	1334.23	1367.36
1372.53	1401.77	1412.08	1443.00	1475.43	1490.45
1496.62	1501.19	1524.11	1639.28	1890.69	2977.30
3011.93	3022.94	3034.45	3037.88	3067.08	3094.26
3113.08	3120.71	3121.43	3548.66	3648.46	3882.78

5.5 Cartesian coordinates of Ile conformers

Each line starting by an asterisk contains the relative energy of the corresponding conformer and the dihedral angle for each of the target torsions (separated by underscores). Cartesian coordinates are given in Å.

```
22
 * E =  +0.000 kcal/mol ; (1) 357_014_094_281_052
 H   +0.00000   +0.00000   +0.00000
 O   +0.00000   +0.00000   +0.97204
 C   +1.27382   +0.00000   +1.35499
 C   +2.28662   -0.07155   +0.19945
 N   +1.62279   +0.21831   -1.07108
 H   +1.69700   +1.19997   -1.31270
 O   +1.60368   +0.00789   +2.50472
 C   +3.55375   +0.74918   +0.49980
 H   +2.57706   -1.13103   +0.17863
 H   +2.01197   -0.31756   -1.83411
 C   +3.35641   +2.25369   +0.27988
 H   +3.77489   +0.58063   +1.56064
 C   +4.71685   +0.24767   -0.35162
 C   +2.15228   +2.85645   +0.99819
 H   +4.27161   +2.75741   +0.61177
 H   +3.29659   +2.45866   -0.79996
 H   +2.12804   +3.94236   +0.87770
 H   +1.20141   +2.46983   +0.61125
 H   +2.17841   +2.62806   +2.06744
 H   +5.62168   +0.83260   -0.16280
 H   +4.94406   -0.80299   -0.14651
```

H +4.48428 +0.34327 −1.41984
22
∗ E = +0.209 kcal/mol ; (2) 004_341_149_064_053

H	+0.00000	+0.00000	+0.00000
O	+0.00000	+0.00000	+0.97209
C	+1.27439	+0.00000	+1.35541
C	+2.28680	+0.10250	+0.21022
N	+1.64609	−0.27177	−1.05151
H	+2.05508	+0.22481	−1.83339
O	+1.60485	−0.03982	+2.50532
C	+2.81731	+1.55027	+0.12499
H	+3.12055	−0.55855	+0.47257
H	+1.72899	−1.26324	−1.23648
C	+3.59772	+1.98111	+1.37088
H	+3.51896	+1.54957	−0.72490
C	+1.69012	+2.54346	−0.15977
C	+4.72323	+1.03335	+1.77136
H	+4.00408	+2.97878	+1.16422
H	+2.90020	+2.09595	+2.20850
H	+5.36981	+1.49162	+2.52376
H	+4.32386	+0.11115	+2.20233
H	+5.34990	+0.77230	+0.91011
H	+2.09782	+3.54894	−0.29608
H	+1.11774	+2.28599	−1.05591
H	+0.99234	+2.58114	+0.68498

22
∗ E = +0.378 kcal/mol ; (3) 357_016_091_296_171

H	+0.00000	+0.00000	+0.00000
O	+0.00000	+0.00000	+0.97188
C	+1.27298	+0.00000	+1.35537
C	+2.28759	−0.07008	+0.20252
N	+1.62580	+0.24708	−1.06312
H	+1.67983	+1.23796	−1.26987
O	+1.60327	+0.02324	+2.50485
C	+3.53784	+0.75826	+0.53523
H	+2.58106	−1.12786	+0.15766
H	+2.03818	−0.25184	−1.83912
C	+3.20208	+2.24795	+0.66820
H	+3.88026	+0.40630	+1.51634
C	+4.62710	+0.50722	−0.50402

C +4.36288 +3.07315 +1.21616
H +2.91005 +2.65303 −0.31220
H +2.33731 +2.36166 +1.33343
H +4.06809 +4.11364 +1.37321
H +4.69790 +2.67127 +2.17762
H +5.21829 +3.07330 +0.53472
H +5.56753 +0.98739 −0.22420
H +4.82547 −0.56354 −0.62077
H +4.33239 +0.90529 −1.48296
22
∗ E = +0.444 kcal/mol ; (4) 179_171_288_294_169
H +0.00000 +0.00000 +0.00000
O +0.00000 +0.00000 +0.96393
C +1.27696 +0.00000 +1.37802
C +1.39307 +0.03120 +2.89606
N +2.75197 −0.17963 +3.35025
H +3.02972 −1.14286 +3.20994
O +2.20234 −0.00539 +0.61565
C +0.82486 +1.36673 +3.42780
H +0.75730 −0.77558 +3.28239
H +3.38768 +0.37925 +2.79011
C +1.69740 +2.54258 +2.97413
H −0.17478 +1.48851 +2.98906
C +0.70098 +1.30201 +4.94704
C +1.05811 +3.90139 +3.24334
H +2.66515 +2.48095 +3.48914
H +1.90960 +2.45156 +1.89962
H +1.67610 +4.71387 +2.85254
H +0.07456 +3.96987 +2.76652
H +0.92247 +4.07891 +4.31374
H +0.26866 +2.22088 +5.35102
H +0.05872 +0.47025 +5.25359
H +1.68722 +1.15085 +5.39584
22
∗ E = +0.455 kcal/mol ; (5) 183_183_300_283_058
H +0.00000 +0.00000 +0.00000
O +0.00000 +0.00000 +0.96393
C +1.27877 +0.00000 +1.37314
C +1.41057 −0.07057 +2.89171
N +2.78713 −0.00516 +3.33440

H	+3.31852	−0.77457	+2.94521
O	+2.19891	+0.00633	+0.60432
C	+0.52965	+0.95615	+3.63719
H	+1.01594	−1.06421	+3.15143
H	+3.22514	+0.83898	+2.98030
C	+1.10841	+2.37376	+3.59169
H	−0.44876	+0.96619	+3.14068
C	+0.35520	+0.50892	+5.08632
C	+1.31979	+2.92577	+2.18420
H	+0.42193	+3.02792	+4.14199
H	+2.05059	+2.39013	+4.15553
H	+1.58377	+3.98604	+2.21117
H	+2.12407	+2.40733	+1.65132
H	+0.40828	+2.82329	+1.58446
H	−0.23517	+1.23756	+5.64974
H	−0.15233	−0.45882	+5.14793
H	+1.33399	+0.41153	+5.56616

22

* E = +0.717 kcal/mol ; (6) 001_351_137_298_176

H	+0.00000	+0.00000	+0.00000
O	+0.00000	+0.00000	+0.97300
C	+1.27454	+0.00000	+1.34906
C	+2.28810	+0.03138	+0.19592
N	+1.60902	−0.16625	−1.08558
H	+1.95691	+0.46012	−1.79955
O	+1.61745	+0.00640	+2.49532
C	+3.04298	+1.37184	+0.26386
H	+3.00856	−0.76956	+0.40466
H	+1.71067	−1.11160	−1.42877
C	+2.08996	+2.55885	+0.07857
H	+3.45016	+1.42198	+1.28188
C	+4.20144	+1.39726	−0.73149
C	+2.78368	+3.90779	+0.24126
H	+1.61909	+2.50694	−0.91337
H	+1.27571	+2.48014	+0.80996
H	+2.06107	+4.72718	+0.22268
H	+3.31824	+3.95460	+1.19597
H	+3.50813	+4.09017	−0.55763
H	+4.86184	+2.24685	−0.54261
H	+4.80306	+0.48567	−0.66191

H +3.84819 +1.49132 −1.76617
22
∗ E = +0.816 kcal/mol ; (7) 004_340_153_072_175
H +0.00000 +0.00000 +0.00000
O +0.00000 +0.00000 +0.97169
C +1.27391 +0.00000 +1.35869
C +2.28820 +0.09578 +0.21181
N +1.64448 −0.29793 −1.04160
H +2.08254 +0.15097 −1.83625
O +1.59836 −0.03336 +2.50975
C +2.80598 +1.54688 +0.10927
H +3.13111 −0.55064 +0.48813
H +1.68472 −1.29860 −1.18876
C +3.73038 +1.89301 +1.27926
H +3.40927 +1.58135 −0.81286
C +1.65199 +2.54180 −0.02459
C +4.36217 +3.27511 +1.14472
H +3.16606 +1.82227 +2.21581
H +4.51822 +1.13212 +1.34119
H +5.10967 +3.44042 +1.92443
H +4.85985 +3.39064 +0.17495
H +3.61721 +4.07060 +1.23393
H +2.01956 +3.53610 −0.28768
H +0.94026 +2.23504 −0.79735
H +1.10699 +2.62398 +0.92372
22
∗ E = +0.849 kcal/mol ; (8) 178_151_288_061_056
H +0.00000 +0.00000 +0.00000
O +0.00000 +0.00000 +0.96458
C +1.27754 +0.00000 +1.37774
C +1.41498 +0.04492 +2.89368
N +2.62892 −0.61024 +3.34599
H +2.55238 −1.61450 +3.24114
O +2.20047 −0.02109 +0.61188
C +1.39269 +1.51187 +3.38723
H +0.55203 −0.47389 +3.32402
H +3.40736 −0.31984 +2.76313
C +0.09085 +2.24887 +3.06011
H +1.47517 +1.42585 +4.47930
C +2.59793 +2.29394 +2.87018

C	−1.16583	+1.56572	+3.59112
H	+0.17111	+3.25440	+3.49057
H	+0.00909	+2.39341	+1.97466
H	−2.03984	+2.21401	+3.48779
H	−1.38113	+0.64024	+3.05016
H	−1.05637	+1.32192	+4.65394
H	+2.57738	+3.31540	+3.26115
H	+3.54183	+1.84034	+3.18387
H	+2.59373	+2.35397	+1.77602

22

∗ E = +1.219 kcal/mol ; (9) 177_012_299_280_057

H	+0.00000	+0.00000	+0.00000
O	+0.00000	+0.00000	+0.96400
C	+1.28343	+0.00000	+1.36494
C	+1.44014	+0.06129	+2.88437
N	+0.23498	−0.15989	+3.65878
H	−0.46808	+0.53572	+3.44505
O	+2.19476	−0.00286	+0.58617
C	+2.58903	−0.85374	+3.35361
H	+1.76602	+1.09582	+3.06753
H	−0.17260	−1.06258	+3.44365
C	+2.16593	−2.32425	+3.43100
H	+3.38776	−0.75905	+2.60752
C	+3.09556	−0.38242	+4.71339
C	+1.61509	−2.89661	+2.12717
H	+3.04195	−2.90536	+3.74179
H	+1.43145	−2.43937	+4.23972
H	+1.43970	−3.97217	+2.21187
H	+0.65811	−2.43954	+1.84676
H	+2.31124	−2.72817	+1.29924
H	+3.88006	−1.04647	+5.08820
H	+3.50716	+0.63014	+4.65782
H	+2.27381	−0.37744	+5.43671

22

∗ E = +1.364 kcal/mol ; (10) 178_141_290_170_165

H	+0.00000	+0.00000	+0.00000
O	+0.00000	+0.00000	+0.96458
C	+1.27505	+0.00000	+1.37922
C	+1.41392	+0.05443	+2.89281
N	+2.49511	−0.79289	+3.36374

H +2.26814 −1.77343 +3.25375
O +2.20377 −0.02472 +0.62003
C +1.67093 +1.51455 +3.32332
H +0.46825 −0.27837 +3.33833
H +3.32344 −0.61429 +2.80413
C +1.57268 +1.61775 +4.84783
H +2.70394 +1.74665 +3.01984
C +0.72186 +2.48932 +2.62893
C +2.15243 +2.91354 +5.40529
H +0.51449 +1.52717 +5.13227
H +2.09503 +0.76126 +5.28516
H +2.09947 +2.93084 +6.49704
H +3.20503 +3.01927 +5.12222
H +1.61951 +3.79594 +5.03907
H +0.86673 +3.50564 +3.00258
H +0.87782 +2.51598 +1.54602
H −0.32148 +2.20587 +2.81045
22
* E = +1.407 kcal/mol ; (11) 178_140_296_182_305
H +0.00000 +0.00000 +0.00000
O +0.00000 +0.00000 +0.96457
C +1.27587 +0.00000 +1.37895
C +1.41473 +0.04628 +2.89373
N +2.47490 −0.82192 +3.37134
H +2.27255 −1.79306 +3.16727
O +2.20363 −0.02264 +0.61884
C +1.69759 +1.50298 +3.31139
H +0.46054 −0.26997 +3.33126
H +3.33798 −0.59323 +2.88743
C +1.90799 +1.60851 +4.82867
H +2.63927 +1.78886 +2.81906
C +0.59520 +2.44199 +2.82022
C +0.75878 +1.04196 +5.65735
H +2.83054 +1.08713 +5.09888
H +2.05543 +2.66833 +5.07004
H +0.89893 +1.26298 +6.71850
H −0.20790 +1.46147 +5.35958
H +0.70590 −0.04638 +5.55590
H +0.75592 +3.45205 +3.20771
H +0.56858 +2.50555 +1.72846

H −0.39296 +2.10413 +3.15058
22
∗ E = +1.486 kcal/mol ; (12) 178_148_288_059_163
H +0.00000 +0.00000 +0.00000
O +0.00000 +0.00000 +0.96451
C +1.27649 +0.00000 +1.37747
C +1.41336 +0.04892 +2.89380
N +2.59575 −0.65629 +3.35343
H +2.47597 −1.65691 +3.25402
O +2.20130 −0.02594 +0.61334
C +1.44175 +1.51798 +3.37641
H +0.52303 −0.42467 +3.32209
H +3.38751 −0.40223 +2.77121
C +0.17253 +2.27631 +2.97387
H +1.47172 +1.44481 +4.47214
C +2.70528 +2.23623 +2.90626
C −0.01698 +3.57206 +3.75644
H +0.20434 +2.49590 +1.89817
H −0.70002 +1.62766 +3.12255
H −0.95165 +4.06734 +3.48090
H −0.04777 +3.37461 +4.83307
H +0.79532 +4.28061 +3.57201
H +2.72739 +3.26348 +3.27836
H +3.60700 +1.74023 +3.27500
H +2.75465 +2.27156 +1.81198
22
∗ E = +1.560 kcal/mol ; (13) 359_009_100_311_309
H +0.00000 +0.00000 +0.00000
O +0.00000 +0.00000 +0.97286
C +1.27462 +0.00000 +1.34999
C +2.28704 −0.02067 +0.19494
N +1.60420 +0.18018 −1.08599
H +1.68218 +1.13439 −1.41221
O +1.61336 −0.00690 +2.49781
C +3.47746 +0.91400 +0.50156
H +2.67537 −1.04759 +0.19296
H +1.97632 −0.42217 −1.80648
C +3.06664 +2.31876 +0.97262
H +4.00428 +0.44836 +1.34223
C +4.42652 +0.96453 −0.69359

C	+2.05751	+3.04812	+0.08603
H	+2.65662	+2.24113	+1.98424
H	+3.97986	+2.92068	+1.05293
H	+1.89277	+4.06573	+0.44856
H	+2.39976	+3.13185	−0.95275
H	+1.08001	+2.55093	+0.09400
H	+5.34898	+1.48790	−0.42773
H	+4.69551	−0.04131	−1.03473
H	+3.98421	+1.49378	−1.54498

22

∗ E = +1.671 kcal/mol ; (14) 358_014_093_075_173

H	+0.00000	+0.00000	+0.00000
O	+0.00000	+0.00000	+0.97230
C	+1.27234	+0.00000	+1.35691
C	+2.28679	−0.05660	+0.19982
N	+1.59886	+0.23707	−1.06067
H	+1.65867	+1.22522	−1.28214
O	+1.59379	+0.01433	+2.50963
C	+3.52623	+0.82202	+0.46023
H	+2.60764	−1.10641	+0.16281
H	+2.00940	−0.27042	−1.83251
C	+4.48266	+0.19560	+1.48014
H	+4.05610	+0.86238	−0.50475
C	+3.12350	+2.24553	+0.85032
C	+5.78810	+0.97333	+1.61588
H	+3.97580	+0.12654	+2.44753
H	+4.70169	−0.83366	+1.16640
H	+6.49732	+0.44273	+2.25606
H	+6.26453	+1.12114	+0.63975
H	+5.62397	+1.95951	+2.05941
H	+3.97560	+2.92690	+0.79558
H	+2.34727	+2.65153	+0.19048
H	+2.73480	+2.26120	+1.87366

22

∗ E = +1.716 kcal/mol ; (15) 178_165_036_302_179

H	+0.00000	+0.00000	+0.00000
O	+0.00000	+0.00000	+0.96372
C	+1.27681	+0.00000	+1.37472
C	+1.38668	+0.05666	+2.88236
N	+2.74298	−0.29129	+3.24720

H	+3.39914	+0.06350	+2.56158
O	+2.20212	+0.00120	+0.61318
C	+0.87886	+1.44301	+3.36311
H	+0.69249	−0.69635	+3.28191
H	+2.99016	+0.05469	+4.16386
C	+1.67690	+2.58487	+2.72904
H	−0.16614	+1.52349	+3.03270
C	+0.90790	+1.50470	+4.88942
C	+1.19691	+3.96703	+3.16162
H	+2.73767	+2.46337	+2.98718
H	+1.61895	+2.50672	+1.63581
H	+1.70888	+4.75508	+2.60371
H	+0.12095	+4.07894	+2.98793
H	+1.38217	+4.14463	+4.22481
H	+0.35820	+2.37298	+5.26065
H	+0.45720	+0.61054	+5.33184
H	+1.93532	+1.59121	+5.26421

22

∗ E = +1.744 kcal/mol ; (16) 182_331_294_059_054

H	+0.00000	+0.00000	+0.00000
O	+0.00000	+0.00000	+0.96408
C	+1.28176	+0.00000	+1.37104
C	+1.42746	−0.04304	+2.88679
N	+0.33261	+0.55484	+3.62917
H	+0.28612	+1.55301	+3.46789
O	+2.19703	+0.00964	+0.59551
C	+1.65294	−1.50197	+3.34841
H	+2.34008	+0.51982	+3.10718
H	−0.55563	+0.16709	+3.33467
C	+2.89159	−2.14850	+2.72097
H	+1.81548	−1.41613	+4.43140
C	+0.41992	−2.37079	+3.10896
C	+4.17986	−1.35525	+2.91706
H	+2.99736	−3.14489	+3.16718
H	+2.71908	−2.31051	+1.64914
H	+5.05252	−1.94766	+2.63053
H	+4.18403	−0.45029	+2.30334
H	+4.30544	−1.06284	+3.96604
H	+0.59301	−3.38077	+3.49180
H	−0.46787	−1.97794	+3.61257

H +0.19822 −2.45689 +2.03900
22
* E = +1.793 kcal/mol ; (17) 007_331_162_185_308
H +0.00000 +0.00000 +0.00000
O +0.00000 +0.00000 +0.97074
C +1.27466 +0.00000 +1.36431
C +2.28033 +0.17336 +0.22297
N +1.69775 −0.37577 −0.99864
H +2.17309 −0.04099 −1.82579
O +1.59279 −0.08885 +2.51315
C +2.57813 +1.67838 +0.04625
H +3.20264 −0.33101 +0.54379
H +1.73774 −1.38781 −1.00885
C +3.48638 +1.93650 −1.16956
H +1.61734 +2.17785 −0.14548
C +3.19218 +2.26835 +1.31604
C +4.76917 +1.10780 −1.18315
H +2.93218 +1.77819 −2.10341
H +3.73968 +3.00285 −1.16782
H +5.41475 +1.40208 −2.01421
H +5.34059 +1.23748 −0.25947
H +4.56067 +0.03805 −1.29436
H +3.42368 +3.32619 +1.16193
H +2.52219 +2.18269 +2.17321
H +4.12056 +1.75222 +1.58160
22
* E = +1.807 kcal/mol ; (18) 178_169_285_312_304
H +0.00000 +0.00000 +0.00000
O +0.00000 +0.00000 +0.96397
C +1.27675 +0.00000 +1.37938
C +1.38493 +0.04963 +2.89544
N +2.72836 −0.20709 +3.37397
H +2.94421 −1.19329 +3.30486
O +2.20382 −0.00659 +0.61908
C +0.79771 +1.39814 +3.39070
H +0.72835 −0.73609 +3.28980
H +3.40946 +0.27594 +2.80042
C +1.30401 +2.62387 +2.61327
H −0.28151 +1.33634 +3.20127
C +1.01404 +1.54003 +4.89363

C	+2.82182	+2.80618	+2.56709
H	+0.91818	+2.58488	+1.58585
H	+0.84379	+3.50820	+3.07060
H	+3.07811	+3.80914	+2.21599
H	+3.27869	+2.67143	+3.55217
H	+3.28410	+2.09888	+1.87118
H	+0.50045	+2.43078	+5.26790
H	+0.62516	+0.66824	+5.42941
H	+2.07611	+1.61637	+5.13656

22

∗ E = +1.816 kcal/mol ; (19) 178_151_039_066_054

H	+0.00000	+0.00000	+0.00000
O	+0.00000	+0.00000	+0.96438
C	+1.27651	+0.00000	+1.37512
C	+1.40616	+0.05605	+2.88210
N	+2.64129	−0.61835	+3.23942
H	+3.36392	−0.39890	+2.56281
O	+2.20107	−0.00833	+0.61187
C	+1.31844	+1.53156	+3.36299
H	+0.55788	−0.49258	+3.30654
H	+2.95907	−0.33158	+4.15665
C	−0.04911	+2.18088	+3.12576
H	+1.46935	+1.47132	+4.45262
C	+2.42879	+2.38968	+2.76311
C	−1.22751	+1.40409	+3.70579
H	−0.01468	+3.18316	+3.57022
H	−0.19762	+2.33417	+2.04926
H	−2.14202	+2.00212	+3.67988
H	−1.42112	+0.48677	+3.14326
H	−1.04015	+1.12940	+4.75020
H	+2.41653	+3.39006	+3.20521
H	+3.41891	+1.95789	+2.93321
H	+2.29708	+2.50038	+1.68102

22

∗ E = +1.834 kcal/mol ; (20) 180_356_295_291_168

H	+0.00000	+0.00000	+0.00000
O	+0.00000	+0.00000	+0.96419
C	+1.28239	+0.00000	+1.36548
C	+1.44918	−0.01118	+2.88368
N	+0.22514	+0.06385	+3.65616

H	−0.23928	+0.95221	+3.51899
O	+2.19544	−0.00099	+0.58855
C	+2.27961	−1.25379	+3.26565
H	+2.05730	+0.87343	+3.11260
H	−0.42736	−0.65358	+3.36245
C	+1.47343	−2.53488	+3.02074
H	+3.14904	−1.26202	+2.59525
C	+2.75171	−1.14269	+4.71153
C	+2.32330	−3.79958	+3.09462
H	+0.66282	−2.59336	+3.75968
H	+0.99585	−2.48361	+2.03136
H	+1.73225	−4.68952	+2.86337
H	+3.15107	−3.75096	+2.37980
H	+2.75022	−3.93915	+4.09158
H	+3.37139	−1.99724	+4.99460
H	+3.34796	−0.23689	+4.86007
H	+1.89157	−1.09442	+5.38596

22

* E = +1.912 kcal/mol ; (21) 180_304_296_169_167

H	+0.00000	+0.00000	+0.00000
O	+0.00000	+0.00000	+0.96358
C	+1.28164	+0.00000	+1.37969
C	+1.37359	+0.00609	+2.89688
N	+0.66665	+1.12772	+3.49726
H	+1.08132	+2.01359	+3.23734
O	+2.20419	−0.00637	+0.61584
C	+0.84172	−1.32331	+3.46223
H	+2.44072	+0.08963	+3.13415
H	−0.30093	+1.13554	+3.19406
C	+1.18050	−1.41914	+4.95233
H	−0.25391	−1.30299	+3.34985
C	+1.39868	−2.50941	+2.67699
C	+0.42854	−2.53351	+5.67276
H	+2.26438	−1.57415	+5.05040
H	+0.95495	−0.45655	+5.42176
H	+0.66636	−2.54340	+6.73969
H	−0.65363	−2.39628	+5.57498
H	+0.67536	−3.52192	+5.27428
H	+1.07059	−3.45736	+3.10941
H	+1.07661	−2.49939	+1.63088

H +2.49490 −2.49265 +2.68818
22
∗ E = +1.941 kcal/mol ; (22) 180_303_301_180_306

H	+0.00000	+0.00000	+0.00000
O	+0.00000	+0.00000	+0.96356
C	+1.28147	+0.00000	+1.37860
C	+1.38147	+0.00811	+2.89627
N	+0.68620	+1.12893	+3.50899
H	+1.05101	+2.01670	+3.18694
O	+2.20301	−0.00625	+0.61290
C	+0.84721	−1.32150	+3.45360
H	+2.45139	+0.08811	+3.11882
H	−0.30148	+1.09866	+3.28026
C	+0.93466	−1.35517	+4.98626
H	−0.21538	−1.38045	+3.17541
C	+1.57636	−2.50027	+2.80923
C	+2.32409	−1.05213	+5.54008
H	+0.22422	−0.63429	+5.40163
H	+0.60945	−2.34953	+5.31601
H	+2.35718	−1.22177	+6.61928
H	+3.09465	−1.68315	+5.08521
H	+2.59182	−0.00556	+5.36584
H	+1.22077	−3.44628	+3.22692
H	+1.41984	−2.53396	+1.72618
H	+2.65664	−2.43908	+2.97828

22
∗ E = +2.106 kcal/mol ; (23) 185_079_303_188_306

H	+0.00000	+0.00000	+0.00000
O	+0.00000	+0.00000	+0.96406
C	+1.27968	+0.00000	+1.37911
C	+1.37465	−0.12057	+2.89485
N	+1.18238	−1.51783	+3.27787
H	+0.29779	−1.87365	+2.93566
O	+2.20173	+0.01302	+0.61315
C	+2.71069	+0.41828	+3.40533
H	+0.55081	+0.46081	+3.32841
H	+1.92257	−2.09437	+2.89152
C	+2.90557	+0.11388	+4.89766
H	+3.50182	−0.09519	+2.84088
C	+2.81851	+1.91484	+3.11089

C +1.75938 +0.59072 +5.78594
H +3.03080 −0.96467 +5.03437
H +3.84441 +0.58408 +5.21480
H +1.99702 +0.44015 +6.84220
H +1.54880 +1.65610 +5.64737
H +0.84530 +0.02804 +5.57256
H +3.76489 +2.31318 +3.48772
H +2.77746 +2.11359 +2.03654
H +2.00697 +2.47225 +3.59168
22
∗ E = +2.124 kcal/mol ; (24) 178_153_035_073_169
H +0.00000 +0.00000 +0.00000
O +0.00000 +0.00000 +0.96433
C +1.27571 +0.00000 +1.37346
C +1.41056 +0.05089 +2.88138
N +2.67319 −0.57407 +3.22396
H +3.36916 −0.37078 +2.51556
O +2.19988 −0.00492 +0.60931
C +1.26324 +1.51674 +3.37603
H +0.57942 −0.52773 +3.30549
H +3.01256 −0.25098 +4.12050
C −0.18050 +2.01832 +3.29419
H +1.53750 +1.47411 +4.44217
C +2.23691 +2.45069 +2.66093
C −0.38743 +3.34285 +4.02300
H −0.46989 +2.12576 +2.24206
H −0.84754 +1.25614 +3.71674
H −1.44078 +3.63454 +4.02234
H −0.06127 +3.27100 +5.06641
H +0.17724 +4.15311 +3.55304
H +2.30914 +3.41463 +3.17015
H +3.24182 +2.02056 +2.61377
H +1.90880 +2.63768 +1.63134
22
∗ E = +2.146 kcal/mol ; (25) 181_183_208_081_170
H +0.00000 +0.00000 +0.00000
O +0.00000 +0.00000 +0.96413
C +1.27857 +0.00000 +1.36892
C +1.41772 −0.02106 +2.87950
N +2.83148 +0.06523 +3.19192

H	+3.04721	−0.38206	+4.07126
O	+2.19441	−0.00894	+0.59567
C	+0.65838	+1.13983	+3.56711
H	+0.95049	−0.96991	+3.19893
H	+3.39646	−0.33985	+2.45607
C	−0.83899	+0.87634	+3.73943
H	+1.10177	+1.18853	+4.57298
C	+0.95368	+2.45900	+2.85694
C	−1.51415	+1.91962	+4.62515
H	−1.32586	+0.85099	+2.75906
H	−0.97560	−0.12059	+4.17929
H	−2.56020	+1.66384	+4.81283
H	−1.00937	+1.99829	+5.59446
H	−1.49689	+2.90995	+4.16107
H	+0.66798	+3.31887	+3.46741
H	+2.02303	+2.53746	+2.63909
H	+0.39626	+2.51997	+1.91378

22

∗ E = +2.157 kcal/mol ; (26) 181_180_218_074_048

H	+0.00000	+0.00000	+0.00000
O	+0.00000	+0.00000	+0.96416
C	+1.27810	+0.00000	+1.37240
C	+1.40011	−0.01411	+2.88164
N	+2.81137	−0.01459	+3.23023
H	+2.99855	−0.60245	+4.02939
O	+2.19745	−0.00595	+0.60314
C	+0.69866	+1.20503	+3.53351
H	+0.88272	−0.92930	+3.20789
H	+3.37559	−0.32386	+2.44813
C	−0.83370	+1.16484	+3.54543
H	+1.03863	+1.16788	+4.57883
C	+1.20534	+2.49952	+2.90653
C	−1.43171	−0.15311	+4.02751
H	−1.16707	+1.98035	+4.19917
H	−1.21681	+1.39999	+2.54685
H	−2.50388	−0.05409	+4.21578
H	−1.30491	−0.94496	+3.28261
H	−0.96094	−0.48603	+4.96007
H	+0.84221	+3.36980	+3.46074
H	+2.29785	+2.52243	+2.89258

H +0.84176 +2.59259 +1.87493
22
* E = +2.158 kcal/mol ; (27) 177_019_190_281_054
H +0.00000 +0.00000 +0.00000
O +0.00000 +0.00000 +0.96376
C +1.28028 +0.00000 +1.36825
C +1.43087 +0.07990 +2.87796
N +0.20745 −0.30714 +3.56001
H +0.27134 −0.09661 +4.54767
O +2.19589 +0.01048 +0.59547
C +2.63402 −0.75118 +3.36183
H +1.69664 +1.14305 +3.03709
H −0.59756 +0.17789 +3.18557
C +2.33045 −2.25164 +3.42416
H +3.43503 −0.57948 +2.63184
C +3.09489 −0.25401 +4.73086
C +1.78779 −2.84180 +2.12776
H +3.25686 −2.76444 +3.71089
H +1.61096 −2.43199 +4.23205
H +1.69268 −3.92826 +2.20305
H +0.79005 −2.44421 +1.91093
H +2.44266 −2.61505 +1.28038
H +3.97136 −0.81354 +5.07018
H +3.35815 +0.80818 +4.70949
H +2.31308 −0.39733 +5.48707
22
* E = +2.380 kcal/mol ; (28) 178_168_288_293_271
H +0.00000 +0.00000 +0.00000
O +0.00000 +0.00000 +0.96395
C +1.27628 +0.00000 +1.37866
C +1.39488 +0.04089 +2.89622
N +2.74363 −0.23964 +3.34587
H +2.96329 −1.21924 +3.21267
O +2.20246 −0.00993 +0.61680
C +0.88023 +1.40714 +3.40708
H +0.71946 −0.72842 +3.29188
H +3.40158 +0.27226 +2.76536
C +1.78301 +2.57769 +2.96070
H −0.10114 +1.54932 +2.93842
C +0.68922 +1.35553 +4.91944

C +2.94173 +2.91519 +3.90205
H +2.17412 +2.37207 +1.95429
H +1.15564 +3.47045 +2.85581
H +3.57344 +3.69267 +3.46403
H +2.57231 +3.29423 +4.85937
H +3.56617 +2.04553 +4.11888
H +0.35962 +2.32487 +5.30656
H −0.06507 +0.60992 +5.18941
H +1.62596 +1.08570 +5.41512
22
∗ E = +2.456 kcal/mol ; (29) 352_029_081_195_297
H +0.00000 +0.00000 +0.00000
O +0.00000 +0.00000 +0.97057
C +1.27367 +0.00000 +1.36288
C +2.27574 −0.19263 +0.21013
N +1.66231 +0.34570 −1.00549
H +1.76428 +1.35461 −1.04506
O +1.58366 +0.11869 +2.51110
C +3.68004 +0.33418 +0.56652
H +2.34028 −1.28278 +0.07149
H +2.09526 −0.03302 −1.83773
C +4.56164 +0.47086 −0.68382
H +3.55372 +1.33462 +1.00465
C +4.35519 −0.56429 +1.60641
C +4.83446 −0.84873 −1.40424
H +4.11375 +1.18662 −1.38340
H +5.51527 +0.91440 −0.37464
H +5.42650 −0.68715 −2.30861
H +5.38878 −1.54395 −0.76816
H +3.90956 −1.35490 −1.70631
H +5.39395 −0.25381 +1.75946
H +3.83254 −0.52564 +2.56157
H +4.36733 −1.60736 +1.26965
22
∗ E = +2.470 kcal/mol ; (30) 182_334_299_071_175
H +0.00000 +0.00000 +0.00000
O +0.00000 +0.00000 +0.96402
C +1.28239 +0.00000 +1.36930
C +1.42526 −0.05687 +2.88572
N +0.29763 +0.47109 +3.63161

H	+0.15044	+1.45140	+3.42551
O	+2.19687	+0.01397	+0.59350
C	+1.71727	−1.51377	+3.31338
H	+2.31251	+0.54166	+3.12101
H	−0.55671	−0.01014	+3.37631
C	+3.12784	−1.94820	+2.90690
H	+1.65961	−1.49098	+4.41000
C	+0.65501	−2.48016	+2.78745
C	+3.49226	−3.33822	+3.41990
H	+3.22020	−1.91466	+1.81450
H	+3.84494	−1.21436	+3.29522
H	+4.54383	−3.56488	+3.22682
H	+3.32608	−3.41443	+4.50035
H	+2.89654	−4.11718	+2.93563
H	+0.74360	−3.45751	+3.26739
H	−0.36152	−2.11897	+2.97137
H	+0.76742	−2.62847	+1.70589

22

* E = +2.481 kcal/mol ; (31) 178_027_194_297_178

H	+0.00000	+0.00000	+0.00000
O	+0.00000	+0.00000	+0.96378
C	+1.27947	+0.00000	+1.37170
C	+1.42001	+0.06053	+2.88193
N	+0.25698	−0.51009	+3.54376
H	+0.24841	−0.25717	+4.52317
O	+2.19631	+0.01166	+0.60095
C	+2.71783	−0.62854	+3.33092
H	+1.53776	+1.14326	+3.08524
H	−0.60424	−0.18254	+3.12629
C	+2.71497	−2.11880	+2.97701
H	+3.53389	−0.14361	+2.77954
C	+2.92460	−0.40133	+4.82821
C	+4.01000	−2.82387	+3.36895
H	+1.85778	−2.59409	+3.46785
H	+2.56111	−2.22457	+1.89686
H	+4.02654	−3.85184	+2.99777
H	+4.88048	−2.30516	+2.95194
H	+4.13583	−2.86710	+4.45489
H	+3.92911	−0.69603	+5.14033
H	+2.79294	+0.65375	+5.09384

H +2.21328 −0.99648 +5.41382
22
∗ E = +2.625 kcal/mol ; (32) 352_030_080_197_183
H +0.00000 +0.00000 +0.00000
O +0.00000 +0.00000 +0.97041
C +1.27353 +0.00000 +1.36342
C +2.27658 −0.19583 +0.21168
N +1.67268 +0.36226 −0.99941
H +1.76741 +1.37237 −1.02053
O +1.58415 +0.12430 +2.51085
C +3.68823 +0.29178 +0.59085
H +2.32862 −1.28510 +0.05654
H +2.11520 +0.00005 −1.83347
C +4.55335 +0.48452 −0.65976
H +3.58691 +1.26003 +1.10355
C +4.33969 −0.70427 +1.55271
C +5.98612 +0.90255 −0.34301
H +4.56219 −0.45570 −1.23303
H +4.10099 +1.24027 −1.31220
H +6.53082 +1.16438 −1.25357
H +5.99758 +1.77592 +0.31828
H +6.53977 +0.10261 +0.15500
H +5.26271 −0.30111 +1.97612
H +3.67149 −0.94834 +2.37921
H +4.59323 −1.62824 +1.01621
22
∗ E = +2.641 kcal/mol ; (33) 178_135_052_185_305
H +0.00000 +0.00000 +0.00000
O +0.00000 +0.00000 +0.96454
C +1.27424 +0.00000 +1.37501
C +1.42217 +0.04339 +2.88238
N +2.44075 −0.93232 +3.22936
H +3.27743 −0.76448 +2.68024
O +2.20247 −0.00168 +0.61598
C +1.72508 +1.50482 +3.29384
H +0.46522 −0.25915 +3.32432
H +2.68259 −0.87564 +4.21005
C +2.02322 +1.60881 +4.79631
H +2.63284 +1.80136 +2.74888
C +0.58103 +2.43303 +2.88266

C	+0.92889	+1.03815	+5.69637
H	+2.97635	+1.11406	+5.01760
H	+2.17879	+2.66776	+5.03425
H	+1.15277	+1.22538	+6.74951
H	−0.04498	+1.48855	+5.48136
H	+0.82470	−0.04542	+5.57347
H	+0.74523	+3.44348	+3.26850
H	+0.48719	+2.50272	+1.79523
H	−0.37978	+2.07416	+3.26772

22

* E = +2.741 kcal/mol ; (34) 181_328_055_062_053

H	+0.00000	+0.00000	+0.00000
O	+0.00000	+0.00000	+0.96391
C	+1.27817	+0.00000	+1.37410
C	+1.41709	−0.03611	+2.88143
N	+0.30707	+0.66048	+3.51052
H	−0.58279	+0.28568	+3.20567
O	+2.19911	−0.02388	+0.60646
C	+1.62584	−1.50471	+3.34315
H	+2.33537	+0.51752	+3.10096
H	+0.35983	+0.57228	+4.51786
C	+2.90029	−2.14039	+2.77728
H	+1.74160	−1.43530	+4.43629
C	+0.41481	−2.38109	+3.03513
C	+4.17157	−1.33372	+3.02453
H	+2.99718	−3.13577	+3.22823
H	+2.77427	−2.30352	+1.69943
H	+5.06091	−1.92321	+2.78797
H	+4.19858	−0.43685	+2.39965
H	+4.24681	−1.02585	+4.07410
H	+0.54160	−3.37358	+3.47699
H	−0.51468	−1.95798	+3.42717
H	+0.29444	−2.51390	+1.95399

22

* E = +2.804 kcal/mol ; (35) 178_134_052_174_171

H	+0.00000	+0.00000	+0.00000
O	+0.00000	+0.00000	+0.96459
C	+1.27343	+0.00000	+1.37543
C	+1.42152	+0.04439	+2.88308
N	+2.41603	−0.95919	+3.22116

H	+3.25112	−0.81130	+2.66431
O	+2.20292	−0.00168	+0.61767
C	+1.76012	+1.49750	+3.29974
H	+0.45852	−0.23157	+3.33170
H	+2.66750	−0.92264	+4.19928
C	+1.77925	+1.61154	+4.82653
H	+2.76993	+1.70584	+2.91384
C	+0.77583	+2.49019	+2.68169
C	+2.31309	+2.94748	+5.33565
H	+0.75930	+1.44705	+5.20195
H	+2.39669	+0.80927	+5.24774
H	+2.39771	+2.94787	+6.42540
H	+3.30697	+3.15030	+4.92293
H	+1.66132	+3.78024	+5.05920
H	+0.97431	+3.50969	+3.01978
H	+0.83549	+2.49445	+1.58933
H	−0.25358	+2.23328	+2.95930

22

∗ E = +2.824 kcal/mol ; (36) 178_143_290_055_270

H	+0.00000	+0.00000	+0.00000
O	+0.00000	+0.00000	+0.96452
C	+1.27656	+0.00000	+1.37998
C	+1.40484	+0.04342	+2.89523
N	+2.51582	−0.76545	+3.36554
H	+2.32109	−1.75075	+3.23493
O	+2.20313	−0.02821	+0.61757
C	+1.55675	+1.49951	+3.39829
H	+0.47298	−0.35301	+3.31298
H	+3.34194	−0.55734	+2.81336
C	+0.40926	+2.41373	+2.92674
H	+1.48811	+1.40273	+4.48852
C	+2.93252	+2.07574	+3.06769
C	+0.63727	+3.11150	+1.58416
H	−0.51996	+1.83167	+2.88780
H	+0.25425	+3.18544	+3.68943
H	−0.26653	+3.63965	+1.26922
H	+1.44241	+3.84829	+1.65452
H	+0.90879	+2.41715	+0.78397
H	+2.99861	+3.11440	+3.40673
H	+3.72277	+1.51400	+3.57170

H +3.12988 +2.06029 +1.99033
22
∗ E = +2.884 kcal/mol ; (37) 009_331_156_148_055
H +0.00000 +0.00000 +0.00000
O +0.00000 +0.00000 +0.97128
C +1.27589 +0.00000 +1.35734
C +2.27487 +0.20861 +0.21630
N +1.69115 −0.31733 −1.01851
H +2.09771 +0.11610 −1.83637
O +1.60586 −0.11146 +2.50077
C +2.59598 +1.71688 +0.03852
H +3.19403 −0.30964 +0.52567
H +1.81545 −1.31917 −1.10198
C +4.03973 +1.91658 −0.44155
H +1.90858 +2.08623 −0.73866
C +2.36987 +2.53797 +1.30722
C +4.42582 +1.16740 −1.71268
H +4.19879 +2.99094 −0.59157
H +4.71021 +1.62035 +0.37645
H +5.45008 +1.40623 −2.00972
H +4.37343 +0.08180 −1.57803
H +3.77335 +1.44057 −2.55105
H +2.65259 +3.57925 +1.12868
H +1.32492 +2.51994 +1.62938
H +2.97421 +2.15183 +2.13376
22
∗ E = +3.007 kcal/mol ; (38) 177_164_031_314_302
H +0.00000 +0.00000 +0.00000
O +0.00000 +0.00000 +0.96375
C +1.27566 +0.00000 +1.37624
C +1.38284 +0.06456 +2.88252
N +2.72370 −0.32534 +3.26552
H +3.39185 −0.06347 +2.55169
O +2.20272 −0.00106 +0.61580
C +0.87690 +1.46302 +3.34199
H +0.66590 −0.66673 +3.28308
H +3.00139 +0.08093 +4.14737
C +1.43048 +2.64559 +2.53210
H −0.20705 +1.44485 +3.16880
C +1.10235 +1.64082 +4.84171

C +2.95245 +2.78632 +2.49292
H +1.05375 +2.58189 +1.50273
H +0.99187 +3.55926 +2.95276
H +3.23327 +3.75143 +2.06250
H +3.39611 +2.73282 +3.49217
H +3.40813 +2.00921 +1.87443
H +0.58710 +2.53679 +5.19960
H +0.72273 +0.78288 +5.40638
H +2.16229 +1.76254 +5.08860
22
* E = +3.022 kcal/mol ; (39) 182_341_056_299_179
H +0.00000 +0.00000 +0.00000
O +0.00000 +0.00000 +0.96395
C +1.27776 +0.00000 +1.36933
C +1.43865 −0.05331 +2.87563
N +0.22197 +0.35469 +3.55124
H −0.57389 −0.19861 +3.25925
O +2.19790 −0.01628 +0.60088
C +2.00236 −1.45339 +3.23762
H +2.22192 +0.67845 +3.10931
H +0.32136 +0.27636 +4.55482
C +1.05300 −2.57308 +2.80343
H +2.93944 −1.55759 +2.67318
C +2.31369 −1.51708 +4.73083
C +1.58411 −3.96812 +3.12025
H +0.08031 −2.43100 +3.29507
H +0.87015 −2.49497 +1.72403
H +0.94022 −4.74246 +2.69571
H +2.58909 −4.10412 +2.70635
H +1.64084 −4.14256 +4.19841
H +2.91054 −2.39892 +4.97537
H +2.87586 −0.63553 +5.05688
H +1.39198 −1.57381 +5.32289
22
* E = +3.080 kcal/mol ; (40) 182_059_186_193_301
H +0.00000 +0.00000 +0.00000
O +0.00000 +0.00000 +0.96366
C +1.28116 +0.00000 +1.37773
C +1.35822 −0.05377 +2.89619
N +0.66273 −1.27454 +3.29858

H	+0.58991	−1.32729	+4.30673
O	+2.19941	+0.02892	+0.61237
C	+2.81296	−0.03377	+3.37635
H	+0.85408	+0.86331	+3.25693
H	−0.27709	−1.28684	+2.92118
C	+2.92148	−0.41541	+4.85964
H	+3.35312	−0.78885	+2.79215
C	+3.44138	+1.33509	+3.11139
C	+2.13062	+0.49070	+5.80327
H	+2.60935	−1.45782	+4.99047
H	+3.98169	−0.38610	+5.13691
H	+2.23888	+0.16512	+6.84107
H	+2.47424	+1.52759	+5.74768
H	+1.05875	+0.49038	+5.57161
H	+4.46436	+1.36758	+3.49867
H	+3.47686	+1.55455	+2.04253
H	+2.87415	+2.13288	+3.60422

22

∗ E = +3.081 kcal/mol ; (41) 182_329_053_071_174

H	+0.00000	+0.00000	+0.00000
O	+0.00000	+0.00000	+0.96379
C	+1.27912	+0.00000	+1.37289
C	+1.41478	−0.04516	+2.88189
N	+0.28951	+0.62746	+3.50772
H	−0.59332	+0.26129	+3.17362
O	+2.19807	−0.01958	+0.60372
C	+1.64404	−1.51513	+3.32835
H	+2.32934	+0.51505	+3.10916
H	+0.32229	+0.51315	+4.51296
C	+3.03688	−2.01075	+2.93196
H	+1.59555	−1.48560	+4.42827
C	+0.54162	−2.44215	+2.81986
C	+3.35104	−3.40138	+3.47535
H	+3.12469	−2.00545	+1.83870
H	+3.78275	−1.29437	+3.29872
H	+4.38958	−3.67716	+3.27580
H	+3.19599	−3.44459	+4.55942
H	+2.71615	−4.16566	+3.01807
H	+0.56944	−3.40790	+3.32993
H	−0.45291	−2.01294	+2.97668

H +0.66075 −2.62873 +1.74523
22
∗ E = +3.086 kcal/mol ; (42) 007_332_158_061_266
H +0.00000 +0.00000 +0.00000
O +0.00000 +0.00000 +0.97105
C +1.27484 +0.00000 +1.36161
C +2.28563 +0.16207 +0.22423
N +1.70464 −0.36486 −1.00933
H +2.15913 +0.02806 −1.82477
O +1.59691 −0.09275 +2.50971
C +2.67268 +1.64583 +0.02578
H +3.18741 −0.37373 +0.54764
H +1.78834 −1.37257 −1.06274
C +3.33222 +2.25636 +1.28145
H +3.44033 +1.61554 −0.76190
C +1.50708 +2.49076 −0.49014
C +2.38989 +2.98361 +2.24114
H +3.85956 +1.46343 +1.82451
H +4.09830 +2.96561 +0.94726
H +2.94191 +3.31367 +3.12491
H +1.95394 +3.87274 +1.77628
H +1.57562 +2.34365 +2.58823
H +1.83392 +3.52159 −0.65652
H +1.11094 +2.10804 −1.43458
H +0.68434 +2.51853 +0.23201
22
∗ E = +3.101 kcal/mol ; (43) 178_137_294_179_096
H +0.00000 +0.00000 +0.00000
O +0.00000 +0.00000 +0.96464
C +1.27544 +0.00000 +1.37940
C +1.41653 +0.04254 +2.89466
N +2.44122 −0.87196 +3.36569
H +2.17699 −1.83514 +3.19759
O +2.20380 −0.02058 +0.61989
C +1.74871 +1.48488 +3.32335
H +0.45339 −0.23643 +3.33912
H +3.29958 −0.70509 +2.84872
C +1.87256 +1.57902 +4.85665
H +2.72389 +1.72810 +2.87318
C +0.70797 +2.46605 +2.78485

C	+3.30718	+1.48334	+5.36728
H	+1.43684	+2.53068	+5.18403
H	+1.26700	+0.78706	+5.31517
H	+3.34855	+1.58587	+6.45544
H	+3.74768	+0.52071	+5.09858
H	+3.92524	+2.27757	+4.93474
H	+0.93863	+3.48204	+3.11596
H	+0.66679	+2.47416	+1.69149
H	−0.29178	+2.20993	+3.15478

22

∗ E = +3.166 kcal/mol ; (44) 179_002_056_281_058

H	+0.00000	+0.00000	+0.00000
O	+0.00000	+0.00000	+0.96404
C	+1.27884	+0.00000	+1.36097
C	+1.46476	+0.01710	+2.86981
N	+0.19461	−0.02474	+3.56905
H	−0.35838	−0.82740	+3.29292
O	+2.19281	−0.00142	+0.58374
C	+2.52303	−1.03405	+3.29252
H	+1.91518	+1.00292	+3.05926
H	+0.33692	−0.06695	+4.57013
C	+1.96590	−2.45906	+3.32767
H	+3.32337	−0.98555	+2.54282
C	+3.09732	−0.66508	+4.65782
C	+1.37622	−2.94297	+2.00614
H	+2.77951	−3.12764	+3.63311
H	+1.21015	−2.52770	+4.12283
H	+1.12880	−4.00663	+2.05128
H	+0.45318	−2.41080	+1.75075
H	+2.08212	−2.79164	+1.18291
H	+3.85793	−1.38717	+4.96853
H	+3.55599	+0.32832	+4.64746
H	+2.31675	−0.67001	+5.42909

22

∗ E = +3.179 kcal/mol ; (45) 180_309_067_183_304

H	+0.00000	+0.00000	+0.00000
O	+0.00000	+0.00000	+0.96352
C	+1.27943	+0.00000	+1.37912
C	+1.36810	−0.00144	+2.89168
N	+0.53538	+1.07702	+3.40406

H	−0.44371	+0.89113	+3.21643
O	+2.20385	−0.03676	+0.61993
C	+1.02748	−1.41762	+3.41210
H	+2.41486	+0.22304	+3.12349
H	+0.65078	+1.17365	+4.40498
C	+1.02531	−1.45761	+4.94660
H	+0.00642	−1.64509	+3.07232
C	+1.98241	−2.45420	+2.81928
C	+2.33113	−0.99793	+5.59210
H	+0.19528	−0.85016	+5.32652
H	+0.80739	−2.48676	+5.25567
H	+2.29506	−1.12502	+6.67693
H	+3.18772	−1.56919	+5.22213
H	+2.53395	+0.06071	+5.39626
H	+1.78143	−3.44686	+3.23294
H	+1.88933	−2.51688	+1.73128
H	+3.02558	−2.20046	+3.03599

22

∗ E = +3.210 kcal/mol ; (46) 178_035_189_073_173

H	+0.00000	+0.00000	+0.00000
O	+0.00000	+0.00000	+0.96375
C	+1.28124	+0.00000	+1.37178
C	+1.39395	+0.05438	+2.88695
N	+0.30651	−0.70753	+3.48634
H	+0.28548	−0.55763	+4.48680
O	+2.19271	+0.01402	+0.59497
C	+2.74565	−0.46194	+3.40701
H	+1.33723	+1.13694	+3.11508
H	−0.59167	−0.44112	+3.10443
C	+3.88595	+0.52409	+3.13803
H	+2.61803	−0.52076	+4.50028
C	+3.04970	−1.86939	+2.89319
C	+5.19215	+0.10333	+3.80448
H	+4.02972	+0.61843	+2.05660
H	+3.59152	+1.51619	+3.50586
H	+5.96090	+0.87185	+3.68979
H	+5.04908	−0.07002	+4.87730
H	+5.58327	−0.82013	+3.36828
H	+3.83448	−2.34307	+3.48826
H	+2.15621	−2.49729	+2.95224

H +3.38649 −1.83224 +1.85247
22
∗ E = +3.256 kcal/mol ; (47) 179_303_070_176_177
H +0.00000 +0.00000 +0.00000
O +0.00000 +0.00000 +0.96358
C +1.27978 +0.00000 +1.37993
C +1.35914 +0.02221 +2.89377
N +0.63033 +1.19639 +3.35330
H −0.36561 +1.08023 +3.20341
O +2.20481 −0.04288 +0.62257
C +0.88158 −1.33493 +3.45878
H +2.42032 +0.15333 +3.13559
H +0.78882 +1.36501 +4.33726
C +1.05009 −1.35554 +4.98008
H −0.19096 −1.42202 +3.22410
C +1.62978 −2.48908 +2.79367
C +0.53683 −2.63431 +5.63576
H +2.11487 −1.21641 +5.21714
H +0.51874 −0.50006 +5.41309
H +0.55618 −2.55374 +6.72558
H −0.49632 −2.83807 +5.33378
H +1.14111 −3.50272 +5.36084
H +1.26666 −3.45614 +3.14887
H +1.51649 −2.47666 +1.70548
H +2.70236 −2.42691 +3.01373
22
∗ E = +3.312 kcal/mol ; (48) 180_351_193_068_051
H +0.00000 +0.00000 +0.00000
O +0.00000 +0.00000 +0.96426
C +1.27796 +0.00000 +1.36527
C +1.45587 −0.00565 +2.86878
N +0.19607 +0.20834 +3.56468
H +0.36639 +0.41615 +4.54017
O +2.19560 −0.00088 +0.59118
C +2.05522 −1.36416 +3.30704
H +2.20254 +0.77863 +3.06292
H −0.33576 +0.97038 +3.16471
C +3.49608 −1.59145 +2.84010
H +2.05871 −1.32191 +4.40752
C +1.15742 −2.51531 +2.86116

C +4.46077 −0.45710 +3.17081
H +3.84154 −2.52186 +3.30755
H +3.49902 −1.76909 +1.75860
H +5.49773 −0.76620 +3.01636
H +4.28210 +0.41172 +2.53043
H +4.36143 −0.13988 +4.21579
H +1.49278 −3.45951 +3.29962
H +0.11816 −2.33916 +3.14771
H +1.20101 −2.62820 +1.77031
22
* E = +3.336 kcal/mol ; (49) 182_057_189_194_184
H +0.00000 +0.00000 +0.00000
O +0.00000 +0.00000 +0.96368
C +1.28127 +0.00000 +1.37739
C +1.36228 −0.04249 +2.89625
N +0.64151 −1.24217 +3.31727
H +0.53508 −1.25669 +4.32334
O +2.19924 +0.02490 +0.61150
C +2.81890 −0.03297 +3.37043
H +0.87971 +0.88885 +3.25261
H −0.28669 −1.25404 +2.91207
C +2.90700 −0.42021 +4.85065
H +3.36334 −0.78221 +2.78009
C +3.43259 +1.34508 +3.12100
C +4.32286 −0.33663 +5.41305
H +2.24081 +0.23599 +5.43276
H +2.53808 −1.44408 +4.97424
H +4.37096 −0.75196 +6.42289
H +5.02159 −0.90041 +4.78523
H +4.67984 +0.69548 +5.46313
H +4.51196 +1.33522 +3.28914
H +3.26451 +1.67626 +2.09389
H +2.99432 +2.08503 +3.80317
22
* E = +3.367 kcal/mol ; (50) 183_067_300_058_163
H +0.00000 +0.00000 +0.00000
O +0.00000 +0.00000 +0.96376
C +1.28247 +0.00000 +1.37746
C +1.35375 −0.07753 +2.90005
N +0.86540 −1.37222 +3.36777

H	−0.09536	−1.51807	+3.08260
O	+2.19699	+0.01382	+0.60257
C	+2.74149	+0.23318	+3.47001
H	+0.65525	+0.68226	+3.27405
H	+1.41863	−2.12821	+2.98140
C	+3.20984	+1.63084	+3.04638
H	+2.59569	+0.23966	+4.55983
C	+3.77685	−0.84397	+3.14263
C	+4.36409	+2.15454	+3.89550
H	+3.50026	+1.60829	+1.99006
H	+2.36355	+2.32758	+3.12358
H	+4.63588	+3.17361	+3.60826
H	+4.09570	+2.16587	+4.95732
H	+5.25896	+1.53558	+3.78524
H	+4.73380	−0.60919	+3.61571
H	+3.47278	−1.82312	+3.52482
H	+3.93870	−0.91643	+2.06367

22

* E = +3.496 kcal/mol ; (51) 182_341_292_308_301

H	+0.00000	+0.00000	+0.00000
O	+0.00000	+0.00000	+0.96417
C	+1.28202	+0.00000	+1.36829
C	+1.44134	−0.05819	+2.88210
N	+0.28113	+0.31432	+3.66798
H	+0.07565	+1.29947	+3.56473
O	+2.19777	+0.00492	+0.59454
C	+1.99550	−1.46186	+3.23556
H	+2.24174	+0.65428	+3.11440
H	−0.54453	−0.19552	+3.38366
C	+1.19459	−2.63053	+2.63788
H	+2.98639	−1.50066	+2.76556
C	+2.16988	−1.59145	+4.74486
C	−0.27921	−2.71983	+3.03780
H	+1.26982	−2.59581	+1.54294
H	+1.70544	−3.55281	+2.94006
H	−0.68747	−3.70133	+2.78265
H	−0.42084	−2.57282	+4.11294
H	−0.88403	−1.97948	+2.50490
H	+2.66211	−2.53739	+4.99024
H	+2.78161	−0.77358	+5.13837

H	+1.20962	−1.54968	+5.26382

22

∗ E = +3.518 kcal/mol ; (52) 179_300_299_177_094

H	+0.00000	+0.00000	+0.00000
O	+0.00000	+0.00000	+0.96360
C	+1.28130	+0.00000	+1.37934
C	+1.37729	+0.02207	+2.89756
N	+0.75608	+1.20531	+3.47436
H	+1.20338	+2.05398	+3.15026
O	+2.20361	−0.00656	+0.61470
C	+0.77108	−1.26085	+3.48779
H	+2.44872	+0.04129	+3.12919
H	−0.22297	+1.25106	+3.21232
C	+0.93629	−1.29401	+5.01861
H	−0.30315	−1.25463	+3.24745
C	+1.41013	−2.48526	+2.83311
C	−0.26754	−0.75789	+5.78798
H	+1.12352	−2.33049	+5.32438
H	+1.83336	−0.72452	+5.29383
H	−0.11652	−0.84280	+6.86792
H	−0.44246	+0.29388	+5.55193
H	−1.17101	−1.32296	+5.53403
H	+0.96965	−3.40459	+3.22780
H	+1.28172	−2.49279	+1.74567
H	+2.48614	−2.51207	+3.04063

22

∗ E = +3.672 kcal/mol ; (53) 178_140_045_058_270

H	+0.00000	+0.00000	+0.00000
O	+0.00000	+0.00000	+0.96443
C	+1.27471	+0.00000	+1.37676
C	+1.40541	+0.04709	+2.88427
N	+2.48899	−0.85032	+3.24300
H	+3.27976	−0.69722	+2.62625
O	+2.20274	−0.01348	+0.61627
C	+1.57583	+1.51107	+3.37997
H	+0.46522	−0.33163	+3.30248
H	+2.79110	−0.68832	+4.19587
C	+0.39662	+2.41225	+2.96403
H	+1.54870	+1.42144	+4.47546
C	+2.92870	+2.10215	+2.99057

C	+0.56519	+3.12146	+1.61898
H	−0.52212	+1.81226	+2.95274
H	+0.25325	+3.17545	+3.73747
H	−0.35996	+3.63048	+1.33665
H	+1.35598	+3.87544	+1.66636
H	+0.82425	+2.43527	+0.80779
H	+3.01046	+3.13302	+3.34926
H	+3.75363	+1.53346	+3.42745
H	+3.06700	+2.10893	+1.90463

22

∗ E = +3.731 kcal/mol ; (54) 185_083_300_185_093

H	+0.00000	+0.00000	+0.00000
O	+0.00000	+0.00000	+0.96417
C	+1.27917	+0.00000	+1.37886
C	+1.38129	−0.12724	+2.89344
N	+1.27235	−1.53847	+3.25667
H	+0.38447	−1.92385	+2.95735
O	+2.20231	+0.01434	+0.61398
C	+2.67311	+0.50190	+3.41539
H	+0.52461	+0.39529	+3.33857
H	+2.01074	−2.07386	+2.81139
C	+2.84137	+0.26327	+4.92682
H	+3.50991	+0.03125	+2.87838
C	+2.67285	+1.99729	+3.09642
C	+3.65733	−0.97653	+5.28157
H	+3.32838	+1.14438	+5.36180
H	+1.84906	+0.19777	+5.39218
H	+3.79399	−1.06318	+6.36334
H	+3.16243	−1.88536	+4.93290
H	+4.65144	−0.92822	+4.82421
H	+3.61239	+2.45748	+3.41376
H	+2.55222	+2.18945	+2.02657
H	+1.85973	+2.50002	+3.63417

22

∗ E = +3.855 kcal/mol ; (55) 007_330_166_188_100

H	+0.00000	+0.00000	+0.00000
O	+0.00000	+0.00000	+0.97047
C	+1.27418	+0.00000	+1.36733
C	+2.28054	+0.17412	+0.22692
N	+1.70448	−0.40323	−0.98607

H	+2.21634	−0.11966	−1.81112
O	+1.58772	−0.08682	+2.51750
C	+2.56338	+1.68130	+0.03813
H	+3.21352	−0.31144	+0.54848
H	+1.72443	−1.41597	−0.95294
C	+3.42237	+1.94001	−1.21887
H	+1.59186	+2.17907	−0.10422
C	+3.24127	+2.25741	+1.28057
C	+2.61657	+2.39878	−2.43426
H	+4.16281	+2.71145	−0.98123
H	+4.00935	+1.04146	−1.45812
H	+3.25415	+2.53344	−3.31210
H	+1.81936	+1.69632	−2.69725
H	+2.13202	+3.35746	−2.22404
H	+3.38377	+3.33513	+1.16175
H	+2.66629	+2.07975	+2.19127
H	+4.22901	+1.80206	+1.41619

22

* E = +3.918 kcal/mol ; (56) 181_352_294_290_270

H	+0.00000	+0.00000	+0.00000
O	+0.00000	+0.00000	+0.96420
C	+1.28259	+0.00000	+1.36650
C	+1.44712	−0.02338	+2.88514
N	+0.22676	+0.14264	+3.65057
H	−0.16098	+1.06774	+3.51408
O	+2.19580	+0.00285	+0.59017
C	+2.20348	−1.31581	+3.26083
H	+2.11478	+0.81750	+3.11268
H	−0.47908	−0.51327	+3.33499
C	+1.34257	−2.57870	+3.03913
H	+3.04790	−1.36495	+2.56352
C	+2.74749	−1.21530	+4.68165
C	+0.49059	−3.01831	+4.23251
H	+0.69915	−2.42979	+2.15951
H	+2.01058	−3.40569	+2.77308
H	−0.14929	−3.86054	+3.95561
H	+1.12191	−3.34622	+5.06332
H	−0.14490	−2.21503	+4.61336
H	+3.26174	−2.13747	+4.97022
H	+3.46272	−0.39116	+4.76358

H	+1.93854	−1.03336	+5.39484

22

∗ E = +3.931 kcal/mol ; (57) 180_316_296_051_271

H	+0.00000	+0.00000	+0.00000
O	+0.00000	+0.00000	+0.96400
C	+1.28213	+0.00000	+1.37545
C	+1.41451	+0.00000	+2.89138
N	+0.51447	+0.92364	+3.56206
H	+0.72388	+1.88237	+3.31305
O	+2.19996	−0.00304	+0.60391
C	+1.26869	−1.42080	+3.48441
H	+2.44485	+0.32136	+3.07621
H	−0.44605	+0.74549	+3.29209
C	+2.19656	−2.43817	+2.79269
H	+1.61182	−1.29998	+4.51927
C	−0.18269	−1.89588	+3.53717
C	+1.60725	−3.14177	+1.56926
H	+3.12932	−1.93442	+2.50945
H	+2.47022	−3.20312	+3.52846
H	+2.35650	−3.78968	+1.10713
H	+0.75311	−3.76753	+1.84367
H	+1.27227	−2.44072	+0.79998
H	−0.23045	−2.90829	+3.95094
H	−0.78444	−1.25073	+4.18262
H	−0.64247	−1.91946	+2.54410

22

∗ E = +3.957 kcal/mol ; (58) 177_144_038_154_054

H	+0.00000	+0.00000	+0.00000
O	+0.00000	+0.00000	+0.96434
C	+1.27381	+0.00000	+1.37503
C	+1.40865	+0.06350	+2.88291
N	+2.55613	−0.76132	+3.23016
H	+3.27685	−0.63380	+2.52700
O	+2.20062	−0.01916	+0.61389
C	+1.50079	+1.54914	+3.34387
H	+0.49326	−0.36991	+3.30934
H	+2.94185	−0.49957	+4.12633
C	+0.99793	+1.70201	+4.78468
H	+2.56840	+1.81013	+3.30225
C	+0.72713	+2.51693	+2.44748

C +1.64957 +0.79190 +5.82096
H +1.13526 +2.74879 +5.08173
H −0.08732 +1.52795 +4.78093
H +1.27477 +1.01533 +6.82306
H +1.43778 −0.26486 +5.62603
H +2.73744 +0.92596 +5.84205
H +0.80054 +3.53415 +2.84301
H +1.11390 +2.52901 +1.42373
H −0.33360 +2.24735 +2.40625
22
* E = +4.264 kcal/mol ; (59) 177_031_190_296_276
H +0.00000 +0.00000 +0.00000
O +0.00000 +0.00000 +0.96369
C +1.27977 +0.00000 +1.37230
C +1.40856 +0.06220 +2.88472
N +0.28255 −0.60650 +3.51947
H +0.27912 −0.42204 +4.51445
O +2.19645 +0.01051 +0.60172
C +2.76374 −0.50458 +3.33097
H +1.43040 +1.15085 +3.09552
H −0.59757 −0.28292 +3.13874
C +2.94794 −2.00035 +3.00973
H +3.50641 +0.04381 +2.73972
C +3.00631 −0.19146 +4.80588
C +2.36490 −2.98551 +4.02244
H +2.53292 −2.20333 +2.01573
H +4.02544 −2.18632 +2.92207
H +2.53547 −4.01323 +3.68919
H +2.84017 −2.88198 +5.00321
H +1.28945 −2.84095 +4.14372
H +3.98925 −0.55410 +5.12220
H +2.96868 +0.88715 +4.99128
H +2.26588 −0.67437 +5.45300
22
* E = +4.310 kcal/mol ; (60) 352_032_076_195_089
H +0.00000 +0.00000 +0.00000
O +0.00000 +0.00000 +0.96999
C +1.27337 +0.00000 +1.36649
C +2.27665 −0.20247 +0.21684
N +1.69145 +0.40312 −0.97985

H	+1.75682	+1.41430	−0.94366
O	+1.58016	+0.13056	+2.51393
C	+3.70493	+0.21160	+0.61652
H	+2.28855	−1.28893	+0.03307
H	+2.16392	+0.09593	−1.81994
C	+4.63066	+0.36059	−0.60398
H	+3.64612	+1.17574	+1.14208
C	+4.28805	−0.83416	+1.57063
C	+4.64746	+1.74360	−1.25214
H	+5.65100	+0.12714	−0.27838
H	+4.38244	−0.41119	−1.34807
H	+5.39832	+1.79722	−2.04541
H	+3.68762	+2.01868	−1.69690
H	+4.89490	+2.50859	−0.50920
H	+5.26405	−0.51016	+1.94284
H	+3.63465	−1.01218	+2.42540
H	+4.43624	−1.78107	+1.03572

22

∗ E = +4.333 kcal/mol ; (61) 179_162_306_109_296

H	+0.00000	+0.00000	+0.00000
O	+0.00000	+0.00000	+0.96432
C	+1.27800	+0.00000	+1.37383
C	+1.41864	+0.02797	+2.89109
N	+2.74289	−0.39203	+3.31123
H	+2.97355	−1.29506	+2.91170
O	+2.19744	−0.01422	+0.60446
C	+1.11947	+1.44837	+3.46151
H	+0.68138	−0.67737	+3.29592
H	+3.43063	+0.25620	+2.93893
C	−0.20664	+1.51469	+4.22961
H	+1.91441	+1.60052	+4.20136
C	+1.24313	+2.55825	+2.41584
C	−1.46183	+1.25544	+3.40185
H	−0.16120	+0.79316	+5.05507
H	−0.27491	+2.50697	+4.69323
H	−2.35727	+1.37694	+4.01721
H	−1.54216	+1.94704	+2.55786
H	−1.47351	+0.24206	+2.99139
H	+1.15270	+3.53764	+2.89436
H	+2.21078	+2.52070	+1.90463

H +0.46184 +2.48856 +1.65193
22
* E = +4.483 kcal/mol ; (62) 182_334_062_310_300
H +0.00000 +0.00000 +0.00000
O +0.00000 +0.00000 +0.96397
C +1.27825 +0.00000 +1.37025
C +1.43150 −0.05860 +2.87575
N +0.26981 +0.48993 +3.55107
H −0.57283 −0.02966 +3.34621
O +2.19842 −0.02159 +0.60210
C +1.87944 −1.50324 +3.24188
H +2.28203 +0.59525 +3.10275
H +0.40663 +0.49300 +4.55315
C +1.02988 −2.62354 +2.62040
H +2.88367 −1.60414 +2.80964
C +1.99773 −1.64533 +4.75606
C −0.45483 −2.64410 +2.98843
H +1.12830 −2.58197 +1.52781
H +1.48554 −3.57491 +2.92184
H −0.90893 −3.59069 +2.68293
H −0.61329 −2.54057 +4.06653
H −1.00723 −1.84887 +2.48008
H +2.44459 −2.60872 +5.01816
H +2.62759 −0.85542 +5.17983
H +1.02112 −1.59237 +5.24809
22
* E = +4.531 kcal/mol ; (63) 356_018_089_093_297
H +0.00000 +0.00000 +0.00000
O +0.00000 +0.00000 +0.97188
C +1.27262 +0.00000 +1.36066
C +2.28590 −0.08629 +0.20340
N +1.59696 +0.27961 −1.04022
H +1.64886 +1.28283 −1.18866
O +1.58549 +0.02796 +2.51439
C +3.59941 +0.69973 +0.44147
H +2.52883 −1.15649 +0.13297
H +2.02867 −0.16272 −1.84083
C +4.72547 −0.13926 +1.06417
H +3.94629 +0.94327 −0.57322
C +3.37394 +2.02497 +1.17015

C	+4.46954	−0.70548	+2.45693
H	+4.95522	−0.96524	+0.37727
H	+5.62408	+0.49051	+1.08807
H	+5.36349	−1.21893	+2.82200
H	+4.20732	+0.07677	+3.17324
H	+3.64250	−1.42014	+2.45846
H	+4.27432	+2.64353	+1.11591
H	+2.55361	+2.59679	+0.71840
H	+3.12142	+1.86562	+2.22023

22

∗ E = +4.539 kcal/mol ; (64) 179_131_056_185_099

H	+0.00000	+0.00000	+0.00000
O	+0.00000	+0.00000	+0.96466
C	+1.27401	+0.00000	+1.37503
C	+1.42658	+0.03072	+2.88328
N	+2.36921	−1.02296	+3.22098
H	+3.23836	−0.88063	+2.71708
O	+2.20280	+0.00440	+0.61661
C	+1.82806	+1.46296	+3.30629
H	+0.45509	−0.20531	+3.33564
H	+2.56734	−1.02777	+4.21314
C	+2.11299	+1.52606	+4.81976
H	+2.75408	+1.70406	+2.76274
C	+0.74362	+2.46141	+2.90294
C	+3.59840	+1.45643	+5.16850
H	+1.70123	+2.45960	+5.22014
H	+1.56486	+0.72175	+5.33131
H	+3.75970	+1.48451	+6.24966
H	+4.06561	+0.54626	+4.78087
H	+4.13303	+2.30407	+4.72808
H	+1.02303	+3.47550	+3.20191
H	+0.56659	+2.46767	+1.82324
H	−0.20549	+2.21591	+3.39401

22

∗ E = +4.761 kcal/mol ; (65) 182_057_186_194_092

H	+0.00000	+0.00000	+0.00000
O	+0.00000	+0.00000	+0.96364
C	+1.28075	+0.00000	+1.37809
C	+1.35890	−0.04411	+2.89703
N	+0.63716	−1.24527	+3.31336

H	+0.56636	−1.27952	+4.32295
O	+2.20001	+0.02644	+0.61381
C	+2.81464	−0.02168	+3.37481
H	+0.87067	+0.88392	+3.25384
H	−0.30534	−1.23353	+2.94214
C	+2.93480	−0.39960	+4.86264
H	+3.36948	−0.75700	+2.77733
C	+3.40540	+1.36764	+3.13234
C	+3.16510	−1.88962	+5.11141
H	+3.77208	+0.16154	+5.29384
H	+2.04470	−0.04645	+5.40561
H	+3.20400	−2.11494	+6.18098
H	+2.38726	−2.50473	+4.65270
H	+4.11877	−2.19848	+4.67150
H	+4.47055	+1.37896	+3.37955
H	+3.29737	+1.68122	+2.09152
H	+2.90869	+2.10670	+3.77329

22

∗ E = +4.901 kcal/mol ; (66) 179_314_059_054_270

H	+0.00000	+0.00000	+0.00000
O	+0.00000	+0.00000	+0.96397
C	+1.28010	+0.00000	+1.37695
C	+1.40133	+0.02160	+2.88678
N	+0.49322	+1.02746	+3.41614
H	−0.46322	+0.82372	+3.15095
O	+2.20077	−0.04246	+0.61223
C	+1.25775	−1.39867	+3.50033
H	+2.42976	+0.34940	+3.07296
H	+0.54034	+1.05025	+4.42775
C	+2.23806	−2.40465	+2.86534
H	+1.56349	−1.26142	+4.54759
C	−0.18210	−1.90746	+3.50498
C	+1.70611	−3.14984	+1.64035
H	+3.16222	−1.87690	+2.59759
H	+2.51438	−3.14495	+3.62498
H	+2.49156	−3.77321	+1.20559
H	+0.87179	−3.80582	+1.90503
H	+1.36230	−2.47284	+0.85359
H	−0.22865	−2.90483	+3.95373
H	−0.83545	−1.25392	+4.08952

H −0.59212 −1.97566 +2.49279
22
∗ E = +4.976 kcal/mol ; (67) 180_306_067_182_096

H	+0.00000	+0.00000	+0.00000
O	+0.00000	+0.00000	+0.96357
C	+1.27962	+0.00000	+1.37944
C	+1.36427	+0.00806	+2.89291
N	+0.57859	+1.13246	+3.38118
H	−0.40752	+0.98411	+3.19811
O	+2.20416	−0.03667	+0.62065
C	+0.97755	−1.38570	+3.43902
H	+2.41800	+0.19268	+3.13320
H	+0.70236	+1.24961	+4.37877
C	+1.01412	−1.39740	+4.97941
H	−0.05362	−1.58863	+3.11176
C	+1.90284	−2.45508	+2.85890
C	−0.34587	−1.17033	+5.63611
H	+1.40772	−2.36386	+5.31477
H	+1.73700	−0.64793	+5.33309
H	−0.27145	−1.18353	+6.72701
H	−0.78835	−0.21449	+5.34163
H	−1.04780	−1.95651	+5.33995
H	+1.61532	−3.44793	+3.21586
H	+1.88906	−2.47039	+1.76507
H	+2.93764	−2.27244	+3.17151

22
∗ E = +5.147 kcal/mol ; (68) 359_170_287_294_169

H	+0.00000	+0.00000	+0.00000
O	+0.00000	+0.00000	+0.96021
C	+1.25724	+0.00000	+1.44127
C	+2.37206	−0.02116	+0.39044
N	+3.67789	+0.21071	+0.97015
H	+3.77860	+1.18199	+1.23748
O	+1.45528	−0.00426	+2.61793
C	+2.34319	−1.36446	−0.37709
H	+2.16463	+0.78277	−0.33123
H	+3.75704	−0.32175	+1.83135
C	+2.70626	−2.52513	+0.55622
H	+1.31535	−1.53416	−0.73624
C	+3.27374	−1.28681	−1.58389

C +2.43530 −3.89476 −0.05829
H +3.76870 −2.43866 +0.81770
H +2.14345 −2.43226 +1.49481
H +2.65686 −4.69552 +0.65156
H +1.38384 −3.99014 −0.34966
H +3.04616 −4.06791 −0.94858
H +3.26002 −2.21563 −2.15923
H +2.98264 −0.47366 −2.25685
H +4.29670 −1.09616 −1.24704
22
∗ E = +5.255 kcal/mol ; (69) 182_341_053_294_272
H +0.00000 +0.00000 +0.00000
O +0.00000 +0.00000 +0.96396
C +1.27882 +0.00000 +1.36913
C +1.44296 −0.04223 +2.87689
N +0.22443 +0.37627 +3.54482
H −0.57548 −0.15047 +3.21659
O +2.19659 −0.01751 +0.59839
C +2.02238 −1.43370 +3.24615
H +2.22701 +0.69464 +3.09560
H +0.30085 +0.25775 +4.54663
C +1.04621 −2.59354 +2.96215
H +2.88818 −1.56096 +2.58399
C +2.52737 −1.43178 +4.68568
C +0.06969 −2.93895 +4.08808
H +0.48343 −2.37849 +2.04385
H +1.63902 −3.48871 +2.74140
H −0.60613 −3.73897 +3.77414
H +0.59850 −3.28998 +4.97908
H −0.54523 −2.08624 +4.38747
H +2.95198 −2.40473 +4.95267
H +3.30425 −0.67398 +4.82507
H +1.72265 −1.22636 +5.40073
22
∗ E = +5.335 kcal/mol ; (70) 002_179_298_284_058
H +0.00000 +0.00000 +0.00000
O +0.00000 +0.00000 +0.96002
C +1.25651 +0.00000 +1.44167
C +2.37789 +0.05335 +0.39344
N +3.69464 +0.06875 +0.99228

H	+3.80596	+0.89765	+1.56382
O	+1.45187	+0.00366	+2.61876
C	+2.28559	−1.05012	−0.68774
H	+2.23401	+1.01453	−0.12514
H	+3.78534	−0.71415	+1.63235
C	+2.73382	−2.42272	−0.17572
H	+1.23586	−1.14764	−1.00799
C	+3.12227	−0.64017	−1.89727
C	+1.95625	−2.93529	+1.03363
H	+2.63330	−3.13101	−1.00647
H	+3.80741	−2.37593	+0.04807
H	+2.19324	−3.98253	+1.23648
H	+2.18092	−2.36955	+1.94298
H	+0.87492	−2.86559	+0.86712
H	+3.10906	−1.42101	−2.66310
H	+2.75234	+0.28591	−2.34866
H	+4.15818	−0.47636	−1.58606

22

* E = +5.447 kcal/mol ; (71) 181_183_202_101_295

H	+0.00000	+0.00000	+0.00000
O	+0.00000	+0.00000	+0.96413
C	+1.27810	+0.00000	+1.36591
C	+1.43308	−0.03363	+2.87705
N	+2.85655	+0.04936	+3.15147
H	+3.07847	−0.32260	+4.06437
O	+2.18974	−0.01184	+0.58752
C	+0.70565	+1.11784	+3.62823
H	+0.97404	−0.99135	+3.18767
H	+3.39944	−0.43231	+2.44577
C	−0.63214	+0.72679	+4.26978
H	+1.38521	+1.33286	+4.46291
C	+0.63217	+2.39331	+2.79102
C	−1.76444	+0.32165	+3.33112
H	−0.44695	−0.09199	+4.97802
H	−0.95869	+1.58111	+4.87655
H	−2.68773	+0.17019	+3.89724
H	−1.95703	+1.08632	+2.57322
H	−1.53835	−0.60796	+2.80301
H	+0.33416	+3.24364	+3.41112
H	+1.61382	+2.61686	+2.35939

H −0.09119 +2.30360 +1.97529
22
* E = +5.513 kcal/mol ; (72) 000_158_288_065_058
H +0.00000 +0.00000 +0.00000
O +0.00000 +0.00000 +0.96074
C +1.25729 +0.00000 +1.43995
C +2.38394 −0.01185 +0.40051
N +3.62774 +0.49386 +0.94888
H +3.57369 +1.49704 +1.07766
O +1.45556 −0.01060 +2.61709
C +2.59952 −1.44496 −0.14528
H +2.09390 +0.63603 −0.43738
H +3.76183 +0.10298 +1.87644
C +1.41406 −1.99920 −0.94272
H +3.45344 −1.34370 −0.82845
C +2.98211 −2.40795 +0.97606
C +1.01314 −1.15512 −2.15143
H +1.69844 −2.99756 −1.29479
H +0.55561 −2.16218 −0.27721
H +0.25302 −1.65956 −2.75272
H +0.60486 −0.17133 −1.88646
H +1.87936 −0.96515 −2.79385
H +3.19551 −3.39945 +0.56649
H +3.87434 −2.07254 +1.51099
H +2.16873 −2.51258 +1.70305
22
* E = +5.632 kcal/mol ; (73) 000_157_287_065_165
H +0.00000 +0.00000 +0.00000
O +0.00000 +0.00000 +0.96039
C +1.25738 +0.00000 +1.43835
C +2.38502 +0.00511 +0.39900
N +3.61914 +0.53105 +0.94972
H +3.55416 +1.53539 +1.06393
O +1.45731 −0.02159 +2.61515
C +2.61563 −1.42004 −0.15686
H +2.08081 +0.64404 −0.44258
H +3.74804 +0.15446 +1.88417
C +1.41450 −1.94415 −0.95219
H +3.46168 −1.30648 −0.84779
C +3.02149 −2.38502 +0.95513

C +1.75185 −3.16543 −1.80309
H +0.59540 −2.19668 −0.26549
H +1.04550 −1.14693 −1.61687
H +0.89524 −3.47758 −2.40558
H +2.58139 −2.94730 −2.48324
H +2.04350 −4.01647 −1.18219
H +3.27451 −3.36665 +0.54757
H +3.90067 −2.02243 +1.49421
H +2.20845 −2.51666 +1.67848
22
∗ E = +5.699 kcal/mol ; (74) 180_047_187_086_299
H +0.00000 +0.00000 +0.00000
O +0.00000 +0.00000 +0.96359
C +1.28439 +0.00000 +1.37254
C +1.36260 +0.01042 +2.89223
N +0.44139 −1.01191 +3.38445
H +0.37523 −0.96496 +4.39310
O +2.19188 +0.02511 +0.59404
C +2.76181 −0.24149 +3.48288
H +1.05257 +1.03643 +3.17431
H −0.48643 −0.89028 +2.99883
C +3.61203 +1.03071 +3.60907
H +2.55058 −0.55724 +4.51549
C +3.50500 −1.39921 +2.81585
C +3.92989 +1.76620 +2.31032
H +3.10297 +1.72160 +4.29461
H +4.55007 +0.74952 +4.10401
H +4.60645 +2.60307 +2.50505
H +4.39988 +1.10932 +1.57455
H +3.02687 +2.17283 +1.84499
H +4.33520 −1.72331 +3.45042
H +2.83340 −2.25138 +2.67355
H +3.90570 −1.11526 +1.84124
22
∗ E = +5.712 kcal/mol ; (75) 001_147_299_184_305
H +0.00000 +0.00000 +0.00000
O +0.00000 +0.00000 +0.96024
C +1.25852 +0.00000 +1.43692
C +2.38723 +0.01803 +0.40076
N +3.54642 +0.74849 +0.87145

H	+3.30863	+1.71423	+1.06513
O	+1.46200	−0.02604	+2.61281
C	+2.78105	−1.43470	+0.06225
H	+2.02017	+0.50099	−0.51810
H	+3.84669	+0.34865	+1.75591
C	+3.97772	−1.47045	−0.90021
H	+3.09454	−1.89805	+1.00946
C	+1.59269	−2.22765	−0.48437
C	+3.77060	−0.66859	−2.18179
H	+4.86303	−1.08882	−0.38448
H	+4.17599	−2.52121	−1.14427
H	+4.59429	−0.83443	−2.88059
H	+2.84527	−0.94674	−2.69771
H	+3.73773	+0.40403	−1.96721
H	+1.91341	−3.22518	−0.79701
H	+0.80123	−2.35460	+0.25977
H	+1.15640	−1.73762	−1.36461

22

* E = +5.870 kcal/mol ; (76) 001_145_291_173_167

H	+0.00000	+0.00000	+0.00000
O	+0.00000	+0.00000	+0.95990
C	+1.25756	+0.00000	+1.43693
C	+2.39081	+0.01931	+0.40651
N	+3.52725	+0.78667	+0.87951
H	+3.30525	+1.77379	+0.92522
O	+1.46016	−0.03084	+2.61320
C	+2.82874	−1.42948	+0.10012
H	+2.02748	+0.47194	−0.52975
H	+3.73952	+0.49392	+1.82907
C	+3.83146	−1.42832	−1.05728
H	+3.33643	−1.79178	+1.00764
C	+1.63776	−2.34067	−0.19064
C	+4.56039	−2.75692	−1.22981
H	+3.29211	−1.17493	−1.98193
H	+4.55603	−0.62747	−0.88345
H	+5.30790	−2.69366	−2.02478
H	+5.08037	−3.03525	−0.30708
H	+3.87951	−3.57332	−1.48740
H	+1.97201	−3.35180	−0.43314
H	+0.95246	−2.41916	+0.65829

H +1.07169 −1.97141 −1.05734
22
∗ E = +6.030 kcal/mol ; (77) 356_154_029_071_166

H	+0.00000	+0.00000	+0.00000
O	+0.00000	+0.00000	+0.96056
C	+1.25531	+0.00000	+1.43611
C	+2.36576	−0.08710	+0.39380
N	+3.55115	+0.51774	+0.95937
H	+3.54542	+0.41219	+1.96793
O	+1.46168	+0.02674	+2.61145
C	+2.55272	−1.56263	−0.05453
H	+2.04795	+0.48744	−0.49226
H	+4.39609	+0.10467	+0.58716
C	+1.38467	−2.06836	−0.90692
H	+3.44620	−1.54599	−0.69850
C	+2.82268	−2.47775	+1.13736
C	+1.69718	−3.37616	−1.62881
H	+0.50276	−2.21215	−0.26838
H	+1.12362	−1.30234	−1.65287
H	+0.87127	−3.67911	−2.27705
H	+2.59232	−3.27311	−2.25091
H	+1.87426	−4.18922	−0.91970
H	+3.16950	−3.46117	+0.81153
H	+3.58482	−2.05488	+1.79812
H	+1.91258	−2.61765	+1.73210

22
∗ E = +6.344 kcal/mol ; (78) 356_155_031_069_057

H	+0.00000	+0.00000	+0.00000
O	+0.00000	+0.00000	+0.96073
C	+1.25553	+0.00000	+1.43789
C	+2.36422	−0.09658	+0.39619
N	+3.55864	+0.49588	+0.96291
H	+3.56036	+0.36651	+1.96893
O	+1.45986	+0.03176	+2.61334
C	+2.54165	−1.57674	−0.04327
H	+2.05917	+0.48888	−0.48376
H	+4.39720	+0.08177	+0.57700
C	+1.37314	−2.13430	−0.86456
H	+3.42560	−1.56491	−0.70037
C	+2.82711	−2.48128	+1.15247

C	+1.05119	−1.35098	−2.13621
H	+1.63200	−3.16282	−1.14220
H	+0.48337	−2.22303	−0.22691
H	+0.32246	−1.88307	−2.75233
H	+0.63472	−0.35512	−1.94283
H	+1.95313	−1.19978	−2.73881
H	+3.11275	−3.48199	+0.81647
H	+3.63383	−2.08716	+1.77644
H	+1.93872	−2.57950	+1.78626

22

∗ E = +6.420 kcal/mol ; (79) 355_164_029_303_180

H	+0.00000	+0.00000	+0.00000
O	+0.00000	+0.00000	+0.96025
C	+1.25550	+0.00000	+1.43997
C	+2.35419	−0.11889	+0.39159
N	+3.60452	+0.26364	+1.00377
H	+3.58559	+0.05767	+1.99610
O	+1.45790	+0.04057	+2.61487
C	+2.30938	−1.54646	−0.21916
H	+2.11504	+0.58890	−0.42083
H	+4.40018	−0.18684	+0.57447
C	+2.45745	−2.62409	+0.85793
H	+1.32090	−1.67141	−0.69029
C	+3.36570	−1.67739	−1.31593
C	+2.42528	−4.04267	+0.29768
H	+3.40020	−2.45694	+1.39565
H	+1.65899	−2.50670	+1.60076
H	+2.41608	−4.78201	+1.10214
H	+1.52900	−4.20312	−0.31197
H	+3.29657	−4.25096	−0.32974
H	+3.20955	−2.58160	−1.90888
H	+3.34216	−0.82077	−1.99715
H	+4.37325	−1.74536	−0.88834

22

∗ E = +6.460 kcal/mol ; (80) 359_170_283_313_304

H	+0.00000	+0.00000	+0.00000
O	+0.00000	+0.00000	+0.96025
C	+1.25779	+0.00000	+1.44083
C	+2.36798	−0.03074	+0.38752
N	+3.68040	+0.20748	+0.95146

H	+3.80376	+1.19333	+1.14423
O	+1.45646	−0.00917	+2.61736
C	+2.27753	−1.36465	−0.40399
H	+2.16704	+0.77638	−0.33199
H	+3.76613	−0.26629	+1.84350
C	+2.10740	−2.60688	+0.48595
H	+1.36779	−1.30343	−1.02207
C	+3.46263	−1.49002	−1.35585
C	+3.18577	−2.81173	+1.55065
H	+1.12622	−2.56966	+0.97728
H	+2.07858	−3.47866	−0.17891
H	+3.12328	−3.82026	+1.96701
H	+4.19280	−2.68059	+1.14371
H	+3.05414	−2.11541	+2.38441
H	+3.34271	−2.36757	−1.99797
H	+3.55053	−0.60455	−1.99349
H	+4.40033	−1.57978	−0.80399

22

* E = +6.893 kcal/mol ; (81) 357_140_043_187_306

H	+0.00000	+0.00000	+0.00000
O	+0.00000	+0.00000	+0.95983
C	+1.25554	+0.00000	+1.43431
C	+2.38061	−0.07843	+0.40661
N	+3.42878	+0.81105	+0.86340
H	+3.55727	+0.69501	+1.86335
O	+1.46416	+0.01973	+2.60930
C	+2.78109	−1.56631	+0.24324
H	+2.00478	+0.28274	−0.56451
H	+4.30682	+0.62607	+0.39723
C	+4.02090	−1.71624	−0.65086
H	+3.04016	−1.93142	+1.24681
C	+1.60784	−2.39102	−0.28940
C	+3.90012	−1.03838	−2.01458
H	+4.90331	−1.33122	−0.12657
H	+4.20691	−2.78791	−0.78786
H	+4.76846	−1.26152	−2.63923
H	+3.01140	−1.37649	−2.55685
H	+3.83752	+0.05155	−1.92259
H	+1.91846	−3.41957	−0.49368
H	+0.77968	−2.43170	+0.42396

H +1.22249 −1.97404 −1.22890
22
∗ E = +6.949 kcal/mol ; (82) 006_326_314_181_306
H +0.00000 +0.00000 +0.00000
O +0.00000 +0.00000 +0.96206
C +1.26785 +0.00000 +1.40901
C +2.34910 +0.13771 +0.33325
N +2.04071 −0.49733 −0.93188
H +1.66913 −1.43358 −0.87009
O +1.50864 −0.07412 +2.57568
C +2.65001 +1.63380 +0.10323
H +3.24173 −0.32963 +0.76754
H +1.51473 +0.07278 −1.57953
C +3.71038 +1.81871 −0.99601
H +1.71640 +2.10114 −0.25161
C +3.06614 +2.32639 +1.40070
C +4.99863 +1.03825 −0.75260
H +3.29852 +1.52628 −1.96770
H +3.92922 +2.89111 −1.06333
H +5.75434 +1.29674 −1.49853
H +5.42382 +1.25079 +0.23312
H +4.81987 −0.03876 −0.82555
H +3.33634 +3.36651 +1.19729
H +2.26902 +2.31873 +2.14685
H +3.93168 +1.83035 +1.85091
22
∗ E = +7.130 kcal/mol ; (83) 357_136_046_177_174
H +0.00000 +0.00000 +0.00000
O +0.00000 +0.00000 +0.95956
C +1.25487 +0.00000 +1.43411
C +2.38524 −0.07702 +0.41127
N +3.37565 +0.89524 +0.82962
H +3.53129 +0.80515 +1.82831
O +1.46392 +0.01899 +2.60916
C +2.86542 −1.54749 +0.32841
H +2.00372 +0.20955 −0.58319
H +4.25564 +0.77330 +0.34821
C +3.94907 −1.69081 −0.74386
H +3.29921 −1.78689 +1.31103
C +1.69357 −2.49104 +0.05925

C	+4.58972	−3.07545	−0.78694
H	+3.50852	−1.45184	−1.72270
H	+4.73882	−0.95039	−0.57155
H	+5.43276	−3.09669	−1.48211
H	+4.96436	−3.35997	+0.20199
H	+3.88209	−3.84361	−1.10913
H	+2.02647	−3.52869	−0.01153
H	+0.93985	−2.44652	+0.85072
H	+1.20654	−2.23752	−0.89261

22
∗ E = +7.138 kcal/mol ; (84) 359_168_288_294_271

H	+0.00000	+0.00000	+0.00000
O	+0.00000	+0.00000	+0.96020
C	+1.25706	+0.00000	+1.44097
C	+2.37435	−0.02589	+0.39244
N	+3.66959	+0.26423	+0.97110
H	+3.73180	+1.24532	+1.21522
O	+1.45524	−0.00270	+2.61788
C	+2.36530	−1.39095	−0.33719
H	+2.14849	+0.74911	−0.35545
H	+3.75368	−0.24021	+1.84954
C	+2.74432	−2.55695	+0.60220
H	+1.33042	−1.56932	−0.66460
C	+3.24632	−1.32250	−1.58054
C	+4.23880	−2.87529	+0.68600
H	+2.35146	−2.35332	+1.60762
H	+2.22077	−3.45596	+0.25752
H	+4.41795	−3.65049	+1.43563
H	+4.61618	−3.25067	−0.26963
H	+4.83504	−1.99857	+0.94818
H	+3.28230	−2.28994	−2.09075
H	+2.86535	−0.58018	−2.28924
H	+4.26559	−1.03682	−1.30693

22
∗ E = +7.185 kcal/mol ; (85) 357_162_028_303_091

H	+0.00000	+0.00000	+0.00000
O	+0.00000	+0.00000	+0.96038
C	+1.25483	+0.00000	+1.43975
C	+2.35684	−0.08177	+0.39379
N	+3.58911	+0.34788	+1.00927

H	+3.56674	+0.16433	+2.00566
O	+1.45683	+0.01636	+2.61612
C	+2.37415	−1.50926	−0.22607
H	+2.09250	+0.61562	−0.41967
H	+4.40378	−0.08398	+0.59781
C	+2.56633	−2.61258	+0.82712
H	+1.39875	−1.66485	−0.71427
C	+3.45086	−1.57947	−1.30798
C	+1.26209	−3.16066	+1.40380
H	+3.12655	−3.43671	+0.37002
H	+3.20177	−2.22555	+1.63498
H	+1.45985	−3.96363	+2.11858
H	+0.68338	−2.39617	+1.92825
H	+0.63226	−3.57164	+0.60750
H	+3.36877	−2.51565	−1.86687
H	+3.37414	−0.74797	−2.01617
H	+4.45476	−1.56136	−0.86869

22

* E = +7.405 kcal/mol ; (86) 001_144_291_057_272

H	+0.00000	+0.00000	+0.00000
O	+0.00000	+0.00000	+0.95973
C	+1.25848	+0.00000	+1.43849
C	+2.38700	+0.01264	+0.40395
N	+3.51839	+0.80197	+0.85497
H	+3.27688	+1.78545	+0.88407
O	+1.45603	−0.01490	+2.61652
C	+2.85112	−1.42364	+0.06191
H	+2.00665	+0.46158	−0.52509
H	+3.74402	+0.53653	+1.80908
C	+1.71052	−2.32301	−0.45115
H	+3.56150	−1.27588	−0.76056
C	+3.60831	−2.06627	+1.22285
C	+0.91747	−3.06826	+0.62504
H	+1.03051	−1.72871	−1.08010
H	+2.14699	−3.06581	−1.12879
H	+0.07493	−3.60154	+0.17697
H	+1.54666	−3.80953	+1.12488
H	+0.51428	−2.40799	+1.39719
H	+3.89323	−3.09118	+0.96531
H	+4.52555	−1.51497	+1.44298

H +2.99921 −2.10285 +2.13208
22
∗ E = +7.501 kcal/mol ; (87) 001_144_295_180_096
H +0.00000 +0.00000 +0.00000
O +0.00000 +0.00000 +0.96010
C +1.25850 +0.00000 +1.43642
C +2.39047 +0.02360 +0.40311
N +3.52102 +0.80570 +0.86268
H +3.26638 +1.78091 +0.96617
O +1.46250 −0.02970 +2.61225
C +2.83058 −1.42183 +0.09428
H +2.02028 +0.46924 −0.53382
H +3.78473 +0.47943 +1.78842
C +3.97148 −1.43176 −0.94250
H +3.20679 −1.84062 +1.04042
C +1.64923 −2.27212 −0.37330
C +5.36424 −1.52581 −0.32695
H +3.81999 −2.28059 −1.62019
H +3.90464 −0.52608 −1.55869
H +6.13686 −1.56361 −1.10004
H +5.56252 −0.66160 +0.31060
H +5.45771 −2.43188 +0.28130
H +1.98227 −3.28600 −0.60946
H +0.85950 −2.35110 +0.37963
H +1.21283 −1.85493 −1.29146
22
∗ E = +7.535 kcal/mol ; (88) 355_165_017_314_301
H +0.00000 +0.00000 +0.00000
O +0.00000 +0.00000 +0.96045
C +1.25496 +0.00000 +1.43900
C +2.35279 −0.10993 +0.38825
N +3.61754 +0.26190 +0.98210
H +3.54309 +0.24163 +1.99274
O +1.45772 +0.04108 +2.61471
C +2.27696 −1.52386 −0.25395
H +2.10475 +0.60891 −0.41328
H +4.37094 −0.34682 +0.69790
C +2.12954 −2.67408 +0.75465
H +1.36106 −1.52982 −0.86490
C +3.44485 −1.73983 −1.21514

C +3.24026 −2.80096 +1.79667
H +1.16737 −2.57325 +1.27337
H +2.06397 −3.60478 +0.17714
H +3.12207 −3.72935 +2.36157
H +4.23386 −2.82434 +1.33726
H +3.21038 −1.97520 +2.51128
H +3.29253 −2.65408 −1.79569
H +3.54751 −0.90313 −1.91333
H +4.39781 −1.85342 −0.68871
22
∗ E = +7.667 kcal/mol ; (89) 353_291_301_171_169
H +0.00000 +0.00000 +0.00000
O +0.00000 +0.00000 +0.96081
C +1.26190 +0.00000 +1.44370
C +2.36778 −0.17640 +0.40140
N +2.37344 −1.52197 −0.16110
H +2.50670 −2.22351 +0.55666
O +1.45573 +0.08837 +2.61714
C +2.28451 +0.87582 −0.71463
H +3.30671 −0.03183 +0.94821
H +1.50775 −1.73343 −0.64476
C +3.53321 +0.79900 −1.59916
H +1.41498 +0.63265 −1.35501
C +2.09278 +2.27159 −0.12239
C +3.42199 +1.62219 −2.87809
H +4.39139 +1.14299 −1.00531
H +3.72211 −0.25026 −1.84479
H +4.30693 +1.49228 −3.50613
H +2.55099 +1.31284 −3.46622
H +3.32188 +2.69170 −2.67275
H +2.03545 +3.03082 −0.90531
H +1.18058 +2.34793 +0.47786
H +2.93976 +2.51961 +0.52753
22
∗ E = +7.982 kcal/mol ; (90) 357_140_040_061_271
H +0.00000 +0.00000 +0.00000
O +0.00000 +0.00000 +0.95968
C +1.25555 +0.00000 +1.43590
C +2.37522 −0.07693 +0.40450
N +3.42417 +0.81765 +0.85115

H	+3.53146	+0.73650	+1.85694
O	+1.45961	+0.02996	+2.61234
C	+2.82687	−1.54752	+0.18184
H	+1.98595	+0.28730	−0.55955
H	+4.30860	+0.59094	+0.41324
C	+1.69000	−2.44809	−0.33760
H	+3.57574	−1.47333	−0.62019
C	+3.51261	−2.13434	+1.41313
C	+0.83785	−3.11884	+0.74203
H	+1.04928	−1.86517	−1.01626
H	+2.13061	−3.23250	−0.96362
H	−0.00378	−3.64853	+0.28819
H	+1.42551	−3.85261	+1.30018
H	+0.42722	−2.40914	+1.46505
H	+3.82557	−3.16469	+1.21710
H	+4.40357	−1.56165	+1.68295
H	+2.84540	−2.13947	+2.28025

22

* E = +8.418 kcal/mol ; (91) 001_165_308_111_299

H	+0.00000	+0.00000	+0.00000
O	+0.00000	+0.00000	+0.96129
C	+1.25488	+0.00000	+1.44172
C	+2.38006	+0.03055	+0.39853
N	+3.65709	+0.36742	+0.99685
H	+3.57378	+1.21970	+1.54059
O	+1.45361	−0.02569	+2.61850
C	+2.50382	−1.33010	−0.35497
H	+2.13024	+0.81838	−0.32824
H	+3.90473	−0.35440	+1.66766
C	+2.08049	−1.24194	−1.82788
H	+3.58232	−1.52519	−0.36055
C	+1.83164	−2.49606	+0.37437
C	+0.62906	−0.83929	−2.07836
H	+2.74073	−0.53154	−2.33958
H	+2.26231	−2.22038	−2.28842
H	+0.37448	−0.92953	−3.13714
H	−0.07605	−1.47147	−1.52633
H	+0.45125	+0.21442	−1.82168
H	+2.08697	−3.44077	−0.11385
H	+2.16176	−2.55470	+1.41653

H +0.74012 −2.41135 +0.38712
22
∗ E = +8.878 kcal/mol ; (92) 357_134_050_186_100
H +0.00000 +0.00000 +0.00000
O +0.00000 +0.00000 +0.95957
C +1.25570 +0.00000 +1.43391
C +2.38643 −0.08131 +0.41211
N +3.36656 +0.91399 +0.80125
H +3.58152 +0.80852 +1.78751
O +1.46584 +0.02179 +2.60853
C +2.88208 −1.54588 +0.35454
H +2.00543 +0.18188 −0.58849
H +4.22062 +0.81911 +0.26744
C +4.11966 −1.67232 −0.55596
H +3.16840 −1.81764 +1.38123
C +1.75524 −2.47084 −0.10462
C +5.44026 −1.71411 +0.21044
H +4.03046 −2.58676 −1.15354
H +4.12916 −0.84783 −1.28365
H +6.29657 −1.77796 −0.46638
H +5.57299 −0.83061 +0.84209
H +5.46911 −2.58643 +0.87096
H +2.10108 −3.50683 −0.15433
H +0.89249 −2.44169 +0.56822
H +1.41551 −2.19212 −1.11115
22
∗ E = +9.105 kcal/mol ; (93) 353_286_303_176_093
H +0.00000 +0.00000 +0.00000
O +0.00000 +0.00000 +0.96066
C +1.26093 +0.00000 +1.44282
C +2.37411 −0.16929 +0.40505
N +2.44881 −1.54180 −0.08133
H +2.58262 −2.19401 +0.68212
O +1.45510 +0.07400 +2.61767
C +2.25605 +0.82803 −0.75451
H +3.30280 +0.04609 +0.94604
H +1.60445 −1.81184 −0.57450
C +3.44209 +0.69360 −1.72853
H +1.33758 +0.60189 −1.32797
C +2.14956 +2.24859 −0.19914

C	+3.18451	−0.24004	−2.90743
H	+3.68898	+1.69166	−2.10933
H	+4.32040	+0.34904	−1.16834
H	+4.04184	−0.26370	−3.58564
H	+2.99860	−1.25999	−2.56445
H	+2.31638	+0.09570	−3.48553
H	+2.02522	+2.97207	−1.00888
H	+1.30864	+2.36696	+0.49224
H	+3.06565	+2.50719	+0.34353

22

∗ E = +9.495 kcal/mol ; (94) 003_184_208_103_296

H	+0.00000	+0.00000	+0.00000
O	+0.00000	+0.00000	+0.96077
C	+1.24643	+0.00000	+1.45040
C	+2.38543	+0.07560	+0.42792
N	+3.62450	−0.02760	+1.17425
H	+4.38787	+0.43687	+0.70302
O	+1.42950	−0.01875	+2.63022
C	+2.36021	−1.03546	−0.66502
H	+2.25736	+1.05616	−0.07189
H	+3.51606	+0.34758	+2.10865
C	+1.84200	−0.58872	−2.04041
H	+3.42416	−1.25516	−0.81208
C	+1.72191	−2.33024	−0.16215
C	+0.38380	−0.14479	−2.13231
H	+2.47871	+0.22786	−2.40361
H	+1.99622	−1.42567	−2.73202
H	+0.09096	+0.01607	−3.17272
H	−0.30449	−0.89839	−1.72938
H	+0.21510	+0.81569	−1.62645
H	+1.92575	−3.15281	−0.85354
H	+2.13964	−2.59560	+0.81452
H	+0.63570	−2.25259	−0.04855

22

∗ E = +9.735 kcal/mol ; (95) 358_159_030_107_299

H	+0.00000	+0.00000	+0.00000
O	+0.00000	+0.00000	+0.96052
C	+1.24981	+0.00000	+1.44213
C	+2.37728	−0.04927	+0.40917
N	+3.56499	+0.46025	+1.06356

H	+3.52636	+0.25716	+2.05641
O	+1.44491	−0.00331	+2.61992
C	+2.52523	−1.49470	−0.17906
H	+2.10551	+0.62902	−0.41679
H	+4.41020	+0.06868	+0.67268
C	+2.02410	−1.62127	−1.62598
H	+3.60909	−1.66077	−0.22537
C	+1.94945	−2.57734	+0.73470
C	+0.54842	−1.30651	−1.86270
H	+2.63027	−0.96934	−2.26707
H	+2.22415	−2.64791	−1.95551
H	+0.24321	−1.60612	−2.86825
H	−0.10568	−1.82982	−1.15705
H	+0.35279	−0.22663	−1.80812
H	+2.19936	−3.57131	+0.35278
H	+2.35210	−2.48881	+1.74820
H	+0.85845	−2.51263	+0.81224

6 Leu

6.1 Reference Z-matrix for Leu

Bond distances are given in Å; bond and dihedral angles, in degrees.

```
 h
 o     1  oh2
 c     2  co3       1  coh3
 c     3  cc4       2  cco4       1  dih4
 n     4  nc5       3  ncc5       2  dih5
 h     5  hn6       4  hnc6       3  dih6
 o     3  oc7       2  oco7       4  dih7
 c     4  cc8       3  ccc8       5  dih8
 h     4  hc9       3  hcc9       5  dih9
 h     5  hn10      4  hnc10      6  dih10
 c     8  cc11      4  ccc11      3  dih11
 h     8  hc12      4  hcc12     11  dih12
 h     8  hc13      4  hcc13     11  dih13
 c    11  cc14      8  ccc14      4  dih14
 c    11  cc15      8  ccc15     14  dih15
 h    11  hc16      8  hcc16     14  dih16
 h    14  hc17     11  hcc17      8  dih17
 h    14  hc18     11  hcc18     17  dih18
 h    14  hc19     11  hcc19     17  dih19
 h    15  hc20     11  hcc20      8  dih20
 h    15  hc21     11  hcc21     20  dih21
 h    15  hc22     11  hcc22     20  dih22

oh2     0.96832536
co3     1.36061769
coh3    111.84763871
cc4     1.51007648
cco4    110.64869974
dih4    181.14650294
nc5     1.46933278
ncc5    106.27183461
dih5    50.10534656
hn6     1.00204524
hnc6    113.57566548
dih6    178.8916766
```

oc7	1.20100284
oco7	121.91905647
dih7	181.53644233
cc8	1.53002213
ccc8	109.34508534
dih8	119.5904398
hc9	1.08907122
hcc9	106.25889269
dih9	238.86955186
hn10	1.00292267
hnc10	112.70505921
dih10	129.16547612
cc11	1.54197633
ccc11	114.97563831
dih11	176.54080515
hc12	1.08277665
hcc12	106.877127
dih12	121.56066815
hc13	1.08375111
hcc13	108.7746939
dih13	237.55956188
cc14	1.54175782
ccc14	108.96893328
dih14	175.4906013
cc15	1.54099722
ccc15	112.06141295
dih15	121.53907019
hc16	1.08520844
hcc16	109.22858613
dih16	242.42502163
hc17	1.08466937
hcc17	110.78397636
dih17	181.38602787
hc18	1.08446269
hcc18	110.91910538
dih18	120.32787018
hc19	1.08540983
hcc19	110.1551575
dih19	240.21937254
hc20	1.08475519

```
hcc20    110.45515901
dih20    183.00116625
hc21     1.08553504
hcc21    110.06396831
dih21    119.34443351
hc22     1.08416118
hcc22    112.02431482
dih22    239.92640505
```

6.2 Reference energy and target torsions for Leu

- Reference M08-HX/MG3S energy: -441.7142546 hartree
- Target torsions:
  - torsion1: dih4
  - torsion2: dih5
  - torsion3: dih6
  - torsion4: dih11
  - torsion5: dih14

6.3 Properties of Leu conformers

Table S6: Relative energy (kcal mol^{-1}), rotational constants (B1 to B3, GHz) and dipolar moment (Debye) for the conformers of Leu located at the M08-HX/MG3S level.

Conformer	Rel. Energy	B1	B2	B3	Dip. Mom.
1	0.000	0.792	0.865	2.841	1.162
2	0.167	0.983	1.093	2.482	5.161
3	0.248	0.917	1.053	2.360	4.844
4	0.366	0.765	0.856	2.965	5.438
5	0.440	0.987	1.100	2.393	1.436
6	0.620	0.938	1.078	2.221	1.543
7	1.002	0.968	1.088	2.200	1.606
8	1.064	0.986	1.105	2.442	1.900

9	1.158	1.006	1.120	2.324	5.069
10	1.273	1.033	1.151	2.177	1.428
11	1.322	0.983	1.093	2.458	1.562
12	1.438	0.809	0.848	2.828	1.884
13	1.560	0.801	0.865	2.809	2.433
14	1.589	0.823	0.923	2.924	1.362
15	1.590	0.755	0.857	3.038	1.505
16	1.629	0.933	1.066	2.257	2.158
17	1.812	0.821	0.950	2.615	4.835
18	1.923	0.909	1.155	2.318	4.895
19	1.931	0.732	0.895	2.832	1.311
20	1.989	0.754	0.857	3.066	2.251
21	1.993	0.982	1.093	2.511	2.159
22	2.034	0.914	1.136	2.320	4.824
23	2.046	0.798	0.991	2.718	5.461
24	2.073	0.931	1.134	2.217	1.360
25	2.126	0.890	1.083	2.388	1.260
26	2.169	0.957	1.073	2.237	2.558
27	2.340	0.909	1.144	2.244	1.564
28	2.497	1.095	1.290	2.039	1.608
29	2.563	0.896	1.100	2.348	1.640
30	2.579	1.077	1.286	2.026	1.406
31	2.583	1.064	1.259	2.135	4.806
32	2.642	0.986	1.188	2.170	1.159
33	2.660	0.799	0.982	2.714	4.992
34	2.715	0.814	0.843	2.819	2.757
35	2.817	0.845	0.957	2.541	2.270
36	2.824	1.143	1.378	2.023	5.152
37	2.921	1.057	1.180	2.416	2.011
38	3.044	1.031	1.132	2.195	1.685
39	3.044	0.823	0.917	2.942	1.707
40	3.051	1.036	1.162	2.430	5.092
41	3.107	1.054	1.172	2.365	1.521
42	3.139	0.879	1.117	2.440	1.475
43	3.163	0.905	1.136	2.272	2.165
44	3.180	0.722	0.884	2.972	1.586
45	3.195	0.891	1.094	2.351	1.736

46	3.243	0.991	1.096	2.421	1.948
47	3.259	0.889	1.129	2.421	2.302
48	3.293	0.830	1.025	2.474	5.002
49	3.352	0.746	0.882	2.829	1.697
50	3.398	0.836	1.020	2.622	2.389
51	3.425	0.726	0.881	3.003	2.263
52	3.536	1.073	1.276	2.050	2.003
53	3.686	1.089	1.282	2.063	2.738
54	3.709	0.999	1.209	2.138	1.792
55	3.741	1.056	1.180	2.436	1.975
56	3.834	1.164	1.430	1.948	2.305
57	3.914	0.809	0.977	2.704	2.178
58	3.921	0.905	1.127	2.279	2.542
59	3.943	0.892	1.112	2.313	2.772
60	3.944	0.751	0.887	2.804	2.468
61	3.976	0.907	1.125	2.355	2.236
62	3.993	1.052	1.171	2.391	1.432
63	4.134	0.889	1.082	2.418	1.678
64	4.147	0.726	0.853	3.097	2.283
65	4.355	0.830	0.914	2.886	2.623
66	4.570	0.819	0.884	3.029	2.115
67	4.637	1.156	1.400	1.953	1.575
68	4.785	0.794	0.863	2.825	3.411
69	4.838	0.982	1.185	2.193	1.738
70	4.850	0.946	1.133	2.203	2.244
71	4.864	0.760	0.873	2.832	2.720
72	5.242	0.837	0.913	2.895	2.816
73	5.404	0.989	1.093	2.379	3.326
74	5.415	0.920	1.049	2.269	3.473
75	5.522	1.072	1.194	2.332	1.972
76	5.915	0.980	1.102	2.435	4.231
77	5.926	1.035	1.153	2.174	3.136
78	6.360	0.923	1.043	2.283	4.553
79	6.420	0.969	1.131	2.338	2.371
80	6.481	0.824	0.926	2.897	3.443
81	6.728	0.981	1.184	2.231	4.036
82	6.841	0.933	1.131	2.216	3.148

83	6.850	0.734	0.895	2.812	3.384
84	6.949	0.906	1.141	2.248	3.265
85	7.361	1.162	1.434	1.920	3.219
86	7.498	1.077	1.281	2.026	3.228
87	7.838	1.058	1.179	2.400	4.284
88	7.919	0.800	0.957	2.600	4.940
89	8.076	1.059	1.171	2.350	3.356
90	8.201	1.170	1.434	1.937	4.061
91	8.906	0.751	0.886	2.791	4.786
92	9.493	0.883	0.959	2.432	4.812
93	9.707	0.872	1.056	2.477	4.950
94	10.340	0.955	1.152	2.240	4.859
95	10.987	1.072	1.283	2.014	5.056

6.4 Vibrational frequencies of Leu conformers

Harmonic vibrational zero point energy (ZPE) and frequencies for each conformer in cm^{-1}.

- Conformer (1); ZPE = 42437.987 cm^{-1}:

38.91	69.96	99.78	138.29	158.46	223.55
238.43	260.59	306.27	329.03	381.21	423.21
430.37	521.82	589.07	658.15	745.07	827.91
856.88	881.53	924.88	957.41	968.16	983.84
1000.07	1077.75	1145.40	1166.83	1195.60	1199.70
1230.00	1275.54	1316.23	1324.92	1361.92	1372.34
1390.55	1392.69	1409.32	1415.98	1467.93	1480.05
1484.49	1493.32	1505.92	1666.62	1862.87	3008.01
3019.40	3023.62	3039.19	3042.95	3070.84	3096.12
3100.17	3111.97	3115.31	3536.51	3615.82	3847.23

- Conformer (2); ZPE = 42735.631 cm^{-1}:

72.31	90.74	118.22	196.00	241.48	255.03
278.09	302.90	327.22	366.04	388.59	417.84
521.96	544.46	614.31	758.05	792.92	807.29
851.28	873.51	893.79	924.20	965.27	976.11
1025.27	1101.45	1140.35	1160.06	1195.74	1215.88
1241.72	1266.68	1303.47	1340.14	1365.06	1389.44
1396.25	1400.99	1415.17	1430.54	1466.66	1479.93
1484.36	1496.21	1504.44	1658.59	1885.96	2987.75
3027.91	3029.23	3031.32	3046.81	3084.47	3099.48
3105.20	3114.73	3116.71	3557.35	3646.03	3682.33

- Conformer (3); ZPE = 42669.404 cm^{-1}:

57.16	84.72	124.19	156.02	234.85	237.62
262.81	293.00	320.95	342.40	385.97	436.30
446.48	548.71	651.89	757.62	791.07	818.24
841.63	887.08	928.05	947.85	960.55	982.24
1022.89	1107.94	1110.11	1159.69	1197.94	1207.73
1261.94	1265.67	1312.71	1352.21	1354.04	1373.19
1392.29	1396.28	1408.98	1426.88	1468.46	1480.21
1485.44	1493.68	1507.09	1654.79	1887.25	2994.40
3019.81	3023.08	3036.28	3046.61	3059.14	3097.67
3101.65	3116.77	3127.10	3553.37	3638.39	3699.76

- Conformer (4); ZPE = 42618.463 cm^{-1}:

49.26	76.51	103.85	138.47	225.04	242.89
266.34	284.66	321.96	370.19	396.58	427.62
443.81	540.96	630.33	737.51	804.98	828.70
856.42	877.34	911.89	923.27	971.56	974.72
1027.13	1090.99	1136.63	1178.01	1195.16	1213.15
1240.39	1265.17	1292.35	1334.79	1365.77	1387.82
1394.28	1396.24	1412.46	1429.65	1468.04	1478.83
1484.48	1495.02	1501.56	1657.77	1889.52	2998.65
3015.82	3024.80	3028.81	3047.06	3081.18	3097.07
3103.91	3111.32	3115.71	3543.30	3633.43	3695.80

- Conformer (5); ZPE = 42601.878 cm^{-1}:

69.66	76.92	112.34	191.37	228.61	241.84
256.34	280.71	302.32	348.78	382.85	419.53
475.18	511.19	617.86	634.15	766.63	780.38
852.14	889.80	920.55	926.53	969.72	986.60
1008.27	1105.33	1143.90	1168.52	1180.98	1198.62
1241.85	1280.91	1313.19	1335.78	1369.30	1377.91
1393.45	1395.81	1409.05	1418.16	1466.72	1481.96
1485.36	1496.43	1507.60	1672.63	1856.96	3015.47
3020.18	3024.19	3027.23	3042.68	3089.84	3098.87
3102.37	3110.98	3115.91	3543.66	3619.32	3842.37

- Conformer (6); ZPE = 42549.051 cm^{-1}:

44.46	92.52	112.23	153.23	226.81	230.83
247.60	265.47	283.80	325.05	372.01	421.94
441.50	519.56	633.38	653.09	783.29	810.40
843.28	886.69	926.65	947.59	965.84	978.34
1041.93	1100.72	1138.97	1172.03	1177.16	1197.30
1221.99	1263.36	1302.81	1333.05	1359.32	1374.52
1387.65	1394.18	1411.03	1433.39	1468.21	1479.63
1485.19	1494.43	1507.04	1666.17	1860.83	3009.49
3015.75	3025.35	3028.66	3066.45	3083.28	3099.63
3103.32	3116.18	3122.47	3536.38	3621.35	3833.32

- Conformer (7); ZPE = 42547.635 cm^{-1}:

50.59	86.27	103.01	152.76	233.04	235.15
262.24	275.76	302.57	335.53	361.67	423.85
452.83	544.75	591.31	616.05	780.80	815.92
840.88	871.27	925.43	934.00	964.75	979.64
1033.47	1091.39	1129.48	1161.69	1197.54	1202.17
1218.24	1271.15	1322.87	1337.19	1361.51	1374.24
1384.33	1396.82	1405.74	1413.26	1467.58	1480.25
1484.96	1495.31	1506.89	1660.16	1862.67	2999.20
3019.67	3023.28	3026.90	3069.44	3091.42	3099.09
3101.76	3115.85	3120.81	3545.40	3634.20	3849.26

- Conformer (8); ZPE = 42538.981 cm^{-1}:

65.97	83.42	115.14	190.75	220.09	243.23
247.80	298.75	319.18	354.31	384.05	413.83
493.17	509.22	611.95	628.67	759.38	783.04
830.85	880.59	907.91	921.99	952.85	976.40
1010.76	1102.99	1148.19	1164.29	1194.34	1204.18
1233.46	1262.94	1311.64	1338.89	1349.18	1367.22
1392.08	1397.35	1409.08	1448.27	1470.79	1483.34
1485.08	1494.68	1513.13	1632.27	1857.00	2936.86
3021.96	3024.37	3025.58	3043.48	3074.17	3096.06
3102.88	3111.56	3116.20	3558.67	3653.20	3849.29

- Conformer (9); ZPE = 42724.741 cm^{-1}:

37.97	84.76	124.41	197.39	235.85	249.19
267.91	305.05	330.80	364.88	389.53	406.80
525.32	539.52	604.48	770.98	791.73	828.81
851.19	870.34	885.97	929.43	956.20	973.28
1044.09	1095.23	1118.54	1179.03	1199.97	1211.17
1246.82	1268.95	1310.23	1335.24	1373.90	1386.24
1393.99	1397.79	1411.33	1431.06	1459.54	1480.87
1486.22	1500.86	1507.10	1659.50	1884.63	3006.49
3013.09	3024.00	3048.02	3053.56	3087.22	3089.51
3101.01	3110.73	3116.95	3573.92	3657.63	3663.27

- Conformer (10); ZPE = 42546.183 cm^{-1}:

61.40	92.11	102.77	153.45	190.77	228.66
254.70	274.37	317.28	344.53	380.04	414.15
468.40	516.41	605.08	643.25	777.31	786.14
858.48	873.33	912.43	929.33	958.19	974.16
1027.55	1110.89	1125.57	1166.05	1186.63	1203.74
1232.07	1268.30	1299.19	1334.02	1367.68	1371.90
1388.37	1393.87	1408.90	1425.76	1461.98	1482.54
1484.73	1495.22	1516.00	1672.12	1861.69	3003.77
3022.58	3025.93	3034.87	3050.95	3088.00	3099.23
3102.13	3112.72	3114.51	3557.35	3635.84	3842.97

- Conformer (11); ZPE = 42649.765 cm^{-1}:

66.77	89.49	115.65	194.02	245.36	247.76
289.60	300.55	307.33	349.53	383.39	419.36
489.56	526.32	590.34	660.05	764.09	779.50
850.48	878.15	912.67	928.72	967.08	982.40
1003.03	1104.11	1139.85	1169.85	1190.64	1204.04
1230.65	1277.47	1316.66	1336.48	1369.57	1381.57
1389.39	1395.60	1397.57	1416.41	1463.87	1480.67
1485.01	1496.55	1506.84	1669.60	1856.09	3015.76
3023.48	3024.73	3026.92	3043.26	3093.17	3095.87
3102.17	3109.27	3113.09	3552.85	3634.26	3844.99

- Conformer (12); ZPE = 42498.257 cm^{-1}:

25.04	69.96	111.00	131.74	218.76	233.56
262.73	266.67	298.00	330.62	375.71	422.41
433.93	555.75	617.17	638.27	761.05	822.40
855.57	871.15	921.17	941.40	959.22	977.61
993.18	1075.78	1138.92	1180.62	1195.40	1204.70
1221.56	1281.94	1312.28	1332.45	1366.03	1383.71
1387.81	1391.89	1395.24	1408.76	1466.59	1479.41
1483.57	1493.79	1505.51	1662.32	1862.30	3004.21
3022.46	3023.02	3038.91	3060.51	3074.14	3096.97
3102.15	3112.84	3119.21	3544.13	3629.30	3844.01

- Conformer (13); ZPE = 42455.395 cm^{-1}:

46.11	69.61	116.68	134.42	171.11	224.47
240.13	267.73	321.63	323.15	383.73	424.12
436.06	535.32	601.03	656.50	752.23	820.35
833.24	858.04	906.88	928.30	967.51	985.21
1011.25	1124.67	1137.52	1171.33	1182.14	1198.67
1239.59	1264.46	1296.98	1337.17	1356.70	1368.43
1381.79	1396.39	1412.70	1450.30	1466.57	1480.80
1484.63	1495.43	1502.27	1636.55	1867.71	3007.20
3011.58	3022.68	3027.13	3038.27	3066.05	3096.40
3102.28	3111.16	3114.16	3550.43	3648.42	3847.44

- Conformer (14); ZPE = 42529.728 cm^{-1}:

42.42	61.62	105.43	166.29	208.07	229.16
243.45	288.67	312.87	339.40	365.09	384.49
483.94	538.98	589.82	663.94	732.76	819.94
849.72	892.93	919.22	959.16	968.73	979.64
1016.72	1087.96	1113.10	1167.83	1196.72	1212.51
1227.88	1282.95	1321.71	1346.56	1364.12	1370.82
1389.46	1391.62	1408.81	1416.09	1470.13	1476.29
1493.26	1496.40	1509.50	1665.69	1860.99	3007.62
3015.62	3024.81	3032.27	3033.35	3085.96	3100.69
3103.31	3110.81	3127.58	3530.87	3609.05	3844.65

- Conformer (15); ZPE = 42453.853 cm^{-1}:

20.32	75.02	101.88	133.44	230.19	239.00
259.62	271.66	316.22	327.03	371.63	409.97
431.44	531.67	588.64	634.79	756.10	818.91
844.33	876.44	925.04	954.89	963.58	982.26
1001.81	1074.29	1142.76	1173.80	1191.89	1200.34
1216.48	1282.91	1306.64	1328.71	1362.03	1374.90
1391.17	1396.57	1407.05	1415.96	1466.55	1478.91
1484.40	1493.97	1505.04	1657.77	1864.90	3015.36
3017.80	3022.49	3025.47	3035.04	3072.18	3094.56
3098.35	3112.02	3113.65	3538.64	3630.80	3848.44

- Conformer (16); ZPE = 42534.025 cm^{-1}:

46.78	90.37	112.82	153.88	231.39	232.08
257.52	266.96	301.79	314.23	372.32	423.53
436.61	518.08	625.18	648.53	782.58	811.16
838.02	882.48	924.10	930.38	969.31	983.36
1052.97	1117.42	1133.78	1163.65	1179.40	1196.72
1233.04	1251.37	1302.20	1335.27	1351.98	1364.16
1384.86	1392.81	1409.67	1456.55	1473.86	1479.04
1485.06	1494.59	1507.05	1640.99	1865.91	3001.45
3013.36	3024.28	3027.93	3054.21	3076.51	3098.67
3102.05	3115.49	3124.02	3537.93	3629.88	3836.44

- Conformer (17); ZPE = 42596.343 cm^{-1}:

34.74	57.29	96.17	172.91	212.86	231.70
279.28	288.37	312.95	368.50	377.13	416.24
448.81	560.98	589.48	741.65	796.48	817.24
848.65	882.05	919.04	927.61	965.90	975.08
1067.52	1096.91	1136.53	1175.83	1193.73	1200.71
1230.65	1251.11	1304.27	1341.64	1366.54	1380.54
1395.17	1400.20	1411.45	1432.04	1465.55	1476.14
1485.04	1494.86	1499.26	1657.59	1888.74	3005.72
3021.02	3023.01	3038.03	3056.23	3066.64	3095.45
3101.45	3111.42	3118.14	3540.47	3633.71	3708.28

- Conformer (18); ZPE = 42691.322 cm^{-1}:

51.66	74.08	86.27	171.73	244.96	256.50
277.29	306.98	351.36	358.81	391.51	418.31
500.33	558.46	597.43	764.13	795.64	807.00
859.93	887.68	908.93	932.32	961.96	971.45
1054.74	1091.38	1133.97	1162.03	1198.16	1208.22
1238.91	1263.93	1307.83	1335.18	1356.83	1390.77
1396.41	1398.63	1411.32	1432.90	1470.00	1477.93
1482.43	1495.07	1501.52	1655.56	1885.57	2975.44
3021.41	3024.74	3033.69	3043.38	3092.49	3095.65
3100.61	3109.81	3121.26	3541.40	3634.58	3704.19

- Conformer (19); ZPE = 42453.452 cm^{-1}:

26.97	45.73	96.29	145.76	200.48	228.81
254.37	267.28	296.13	313.74	383.95	418.97
438.90	509.50	587.87	660.76	748.11	839.02
859.55	895.19	923.51	946.85	969.51	979.91
1014.84	1085.56	1141.15	1167.14	1196.32	1200.42
1220.76	1270.42	1317.10	1321.97	1363.08	1368.48
1388.28	1392.75	1407.85	1417.26	1469.01	1481.68
1485.40	1495.32	1507.57	1667.62	1862.70	3001.25
3013.71	3023.78	3027.93	3034.32	3080.76	3095.94
3100.81	3114.49	3145.03	3532.33	3612.19	3844.53

- Conformer (20); ZPE = 42499.783 cm^{-1}:

44.07	84.26	109.01	136.35	228.85	245.81
263.79	293.97	319.85	333.02	396.90	416.04
431.76	527.12	585.15	629.67	746.61	824.47
845.41	859.84	922.86	929.45	962.67	988.01
1005.67	1123.64	1139.41	1178.32	1190.56	1195.49
1226.06	1281.26	1299.47	1336.48	1361.00	1368.87
1385.91	1396.23	1409.27	1444.37	1464.12	1478.47
1483.72	1495.07	1499.97	1651.11	1872.55	2936.35
3010.65	3022.13	3027.45	3036.84	3086.69	3094.09
3102.43	3108.02	3116.66	3556.64	3641.53	3848.13

- Conformer (21); ZPE = 42557.935 cm^{-1}:

63.12	84.69	119.03	193.94	240.94	245.49
260.09	313.10	315.40	351.12	380.74	413.86
491.24	534.12	585.35	652.84	756.13	781.51
822.87	882.16	913.92	924.49	953.63	979.06
1005.34	1101.06	1143.30	1172.21	1182.33	1197.80
1230.19	1266.65	1317.53	1344.78	1345.98	1365.49
1391.89	1396.11	1409.23	1437.98	1465.78	1481.11
1483.95	1494.08	1513.68	1647.41	1863.02	2937.99
3022.82	3025.29	3026.74	3034.32	3076.64	3097.75
3106.30	3108.76	3110.26	3557.12	3647.71	3848.36

- Conformer (22); ZPE = 42651.873 cm^{-1}:

69.37	74.90	120.83	151.44	210.25	225.77
270.81	304.20	321.16	334.32	388.41	426.43
456.05	547.37	648.47	753.26	769.50	815.60
862.70	883.81	926.42	948.04	971.23	983.63
1027.34	1104.39	1113.72	1147.27	1198.32	1210.04
1256.88	1264.61	1306.10	1346.91	1354.81	1368.02
1391.23	1395.47	1416.29	1424.48	1471.90	1480.21
1487.59	1495.74	1500.05	1654.54	1890.78	3004.13
3022.91	3025.77	3034.78	3036.37	3056.87	3097.51
3105.27	3114.19	3131.68	3552.55	3638.31	3712.70

- Conformer (23); ZPE = 42679.369 cm^{-1}:

39.02	52.87	99.30	192.63	222.01	239.43
252.20	321.01	328.04	359.36	392.84	448.60
505.02	543.38	621.58	739.06	822.14	829.46
863.28	875.07	902.65	925.13	968.63	982.83
1038.59	1082.63	1102.11	1160.25	1202.31	1208.73
1243.11	1261.13	1296.42	1349.58	1360.40	1382.80
1391.42	1396.83	1415.72	1432.61	1472.60	1474.20
1488.44	1497.58	1502.78	1659.21	1886.67	3009.05
3019.97	3027.53	3032.02	3041.07	3092.91	3093.58
3102.72	3111.66	3121.23	3557.13	3649.23	3668.96

- Conformer (24); ZPE = 42561.445 cm^{-1}:

35.80	65.82	81.54	186.13	213.46	235.49
258.55	269.51	304.19	347.42	362.92	429.13
470.40	521.80	609.06	651.34	775.83	798.71
849.64	904.21	928.12	936.11	970.99	975.19
1021.12	1110.26	1133.62	1169.56	1183.31	1201.71
1232.19	1265.98	1301.82	1331.63	1363.62	1374.27
1393.13	1396.90	1414.39	1426.39	1473.71	1480.03
1484.64	1494.31	1505.00	1671.52	1859.24	2988.21
3023.98	3028.37	3039.41	3052.46	3090.80	3097.60
3101.43	3113.66	3131.41	3533.91	3609.32	3842.60

- Conformer (25); ZPE = 42570.123 cm^{-1}:

45.00	64.54	108.02	166.41	223.49	244.09
261.25	306.50	311.95	331.44	347.62	415.61
464.78	534.40	625.10	648.36	788.73	832.72
839.29	882.39	923.76	932.41	969.74	990.16
1027.85	1105.78	1122.08	1155.84	1180.67	1197.92
1226.64	1263.94	1309.07	1322.32	1361.17	1379.69
1394.84	1402.55	1412.39	1426.54	1470.29	1482.03
1484.85	1495.28	1508.89	1653.89	1859.80	3001.91
3023.61	3024.21	3027.58	3058.38	3086.47	3099.66
3102.02	3107.03	3114.40	3532.06	3622.59	3838.24

- Conformer (26); ZPE = 42490.647 cm^{-1}:

45.68	87.19	105.56	153.58	229.17	234.84
242.13	267.75	290.72	319.37	365.07	424.94
445.32	540.77	593.06	610.86	783.74	812.69
834.71	869.81	917.43	930.22	970.97	982.76
1053.91	1099.14	1126.13	1135.54	1193.77	1197.40
1230.05	1261.99	1304.86	1342.12	1363.30	1369.41
1380.92	1395.09	1411.26	1432.27	1465.10	1479.87
1485.09	1494.24	1508.38	1649.41	1873.69	3001.64
3009.58	3022.86	3026.91	3056.45	3094.13	3098.95
3101.47	3115.33	3122.51	3541.29	3630.38	3848.50

- Conformer (27); ZPE = 42529.262 cm^{-1}:

69.49	83.90	115.30	143.70	206.62	222.22
244.28	270.83	286.79	317.84	373.50	413.39
450.62	517.73	607.67	650.26	772.18	806.85
856.56	899.66	925.29	940.13	968.91	993.68
1040.94	1092.45	1143.64	1159.86	1179.00	1199.43
1223.86	1263.51	1305.59	1322.07	1354.20	1374.55
1386.94	1396.13	1415.32	1432.96	1473.48	1475.97
1485.67	1496.32	1498.04	1665.13	1860.74	3010.81
3021.04	3025.81	3032.19	3063.54	3085.53	3099.14
3104.42	3113.79	3126.16	3536.61	3619.48	3836.75

- Conformer (28); ZPE = 42628.856 cm^{-1}:

65.94	86.29	121.66	161.31	248.11	261.90
267.99	276.20	295.20	326.27	371.94	387.26
527.18	545.31	600.89	613.09	773.26	808.86
826.34	879.30	920.34	944.57	973.35	981.93
1012.42	1089.80	1114.01	1145.04	1198.18	1211.77
1220.95	1275.03	1337.42	1339.61	1366.44	1373.12
1386.31	1398.38	1405.79	1419.29	1470.64	1476.08
1496.22	1499.83	1511.92	1658.40	1858.95	3017.32
3025.44	3028.39	3033.94	3076.43	3089.57	3100.81
3108.82	3111.50	3114.44	3541.44	3629.82	3849.73

- Conformer (29); ZPE = 42548.319 cm^{-1}:

38.69	69.13	111.12	169.84	219.54	243.63
253.34	291.16	303.81	333.87	353.90	421.61
465.56	554.65	597.66	617.06	785.46	826.59
841.13	880.97	923.82	927.60	968.25	981.97
1030.36	1082.37	1120.41	1151.30	1194.16	1203.42
1227.55	1267.87	1314.78	1348.58	1356.25	1377.79
1389.26	1396.24	1398.75	1413.77	1470.79	1482.44
1486.67	1495.57	1509.84	1655.20	1859.38	3007.23
3022.24	3026.15	3029.43	3068.02	3081.96	3100.51
3104.65	3115.15	3117.90	3536.88	3626.80	3846.61

- Conformer (30); ZPE = 42582.825 cm^{-1}:

52.50	77.34	112.51	160.16	235.53	253.26
255.46	269.00	285.99	319.62	368.67	382.09
494.56	546.13	634.50	652.11	775.16	805.02
829.16	886.87	918.09	947.58	976.20	989.93
1013.70	1091.90	1127.50	1147.27	1171.88	1211.54
1221.56	1268.43	1307.94	1346.26	1364.87	1373.12
1392.38	1396.44	1415.61	1430.95	1469.44	1473.39
1493.48	1497.13	1508.38	1660.43	1860.29	3015.04
3025.55	3026.17	3037.78	3075.95	3088.42	3100.09
3110.69	3112.13	3119.97	3531.40	3619.06	3832.07

- Conformer (31); ZPE = 42740.738 cm^{-1}:

51.66	92.57	137.61	165.86	223.81	254.48
280.76	293.40	323.07	353.68	361.16	393.12
530.70	550.97	651.71	762.33	768.33	793.94
836.42	889.12	926.35	958.95	963.40	984.49
1013.85	1105.31	1112.40	1131.83	1204.71	1218.23
1267.34	1270.13	1323.50	1349.89	1370.19	1379.36
1387.63	1399.21	1413.89	1427.14	1472.24	1476.81
1495.56	1499.20	1509.67	1656.28	1888.68	3002.53
3017.40	3027.29	3038.58	3041.76	3059.22	3103.49
3109.57	3117.74	3133.00	3550.73	3637.25	3722.01

- Conformer (32); ZPE = 42601.041 cm^{-1}:

38.94	57.21	119.15	169.86	218.74	240.77
262.24	296.90	322.44	326.91	373.06	392.43
502.69	553.19	622.45	647.44	788.33	816.45
831.05	880.97	923.22	952.32	973.32	989.95
1007.58	1093.89	1116.92	1145.37	1177.97	1207.36
1212.39	1262.32	1306.95	1348.18	1361.40	1366.14
1396.68	1408.96	1416.76	1425.21	1472.30	1477.18
1491.71	1499.30	1508.34	1652.68	1859.40	3017.44
3023.84	3030.72	3036.96	3072.61	3089.71	3093.88
3103.59	3113.93	3117.40	3529.68	3618.70	3838.59

- Conformer (33); ZPE = 42684.421 cm^{-1}:

53.57	84.61	89.57	189.27	241.91	261.54
277.97	301.12	318.62	374.43	391.99	413.98
451.65	555.92	601.41	748.14	794.26	839.35
843.00	895.48	901.87	922.97	971.35	981.42
1058.96	1116.07	1133.80	1174.41	1192.10	1201.57
1233.91	1259.62	1306.71	1335.92	1363.34	1382.95
1397.46	1401.45	1419.01	1432.24	1461.95	1471.28
1483.22	1493.70	1497.39	1656.85	1888.97	2987.10
3016.54	3026.29	3028.07	3035.84	3077.50	3101.24
3113.44	3114.83	3115.87	3542.32	3634.12	3707.37

- Conformer (34); ZPE = 42454.527 cm^{-1}:

39.42	69.50	111.48	131.25	216.05	232.59
245.75	266.83	312.92	323.18	375.71	421.44
434.07	552.77	584.85	633.01	766.40	821.64
855.93	859.52	890.70	924.40	967.00	980.49
1012.34	1106.85	1123.24	1142.85	1194.30	1203.64
1234.10	1268.99	1293.87	1342.87	1363.56	1369.80
1381.10	1394.99	1410.85	1430.40	1466.37	1479.11
1484.22	1494.90	1501.55	1648.79	1871.57	3005.66
3010.85	3025.35	3028.22	3055.83	3087.20	3099.37
3102.84	3113.16	3115.69	3543.87	3634.06	3849.81

- Conformer (35); ZPE = 42454.375 cm^{-1}:

40.84	53.55	99.21	166.97	215.09	232.23
268.31	281.22	306.31	326.87	363.28	413.60
455.18	542.54	575.47	612.61	758.02	801.57
833.64	879.85	919.73	936.23	964.44	986.77
1040.05	1099.75	1144.08	1162.08	1190.00	1198.27
1224.58	1270.37	1298.43	1326.31	1351.24	1359.52
1394.09	1399.15	1411.18	1451.72	1464.15	1477.07
1484.78	1495.25	1499.12	1648.95	1874.66	2956.44
3014.10	3022.89	3023.50	3041.22	3084.52	3096.02
3100.69	3111.29	3118.18	3552.84	3641.26	3847.49

- Conformer (36); ZPE = 42765.149 cm^{-1}:

37.25	90.62	147.72	199.53	242.96	248.77
290.49	314.13	325.01	354.08	379.45	408.41
528.90	587.58	619.16	750.91	786.40	814.05
831.61	885.45	893.06	926.69	968.55	978.10
1013.16	1081.76	1122.62	1158.69	1198.20	1220.94
1247.36	1276.51	1330.06	1357.02	1371.87	1380.09
1387.87	1398.47	1419.47	1431.13	1461.35	1473.78
1493.78	1498.50	1509.95	1656.21	1888.96	3007.81
3021.10	3023.59	3038.59	3039.48	3083.67	3096.32
3109.91	3112.97	3125.25	3570.58	3655.18	3659.20

- Conformer (37); ZPE = 42567.378 cm^{-1}:

74.74	87.70	122.18	190.66	227.75	233.65
250.93	316.89	327.25	349.73	392.91	393.83
496.90	527.35	607.03	647.58	749.19	779.26
812.11	881.86	909.46	919.00	951.17	974.07
1029.82	1095.34	1119.91	1170.38	1195.29	1209.16
1229.94	1268.89	1321.97	1340.15	1362.50	1380.04
1384.40	1390.96	1410.84	1445.95	1473.86	1478.97
1496.10	1500.12	1513.56	1629.80	1860.39	2923.23
3008.29	3028.12	3029.18	3032.99	3086.06	3097.93
3108.97	3109.81	3131.50	3552.56	3646.75	3847.82

- Conformer (38); ZPE = 42574.154 cm^{-1}:

23.95	90.60	104.68	172.68	214.48	231.69
257.94	274.85	318.46	340.96	381.19	413.20
479.37	544.46	591.10	665.12	774.97	788.24
848.27	859.63	906.22	928.50	947.66	974.49
1019.36	1104.12	1121.13	1185.11	1194.96	1210.16
1225.04	1270.61	1316.43	1334.98	1368.33	1377.77
1390.58	1396.40	1400.30	1410.90	1457.69	1481.89
1485.20	1495.12	1516.49	1667.38	1857.57	3005.92
3023.50	3026.32	3036.23	3060.46	3091.27	3099.37
3102.49	3112.82	3115.18	3564.97	3650.10	3839.45

- Conformer (39); ZPE = 42588.238 cm^{-1}:

17.43	65.34	116.15	164.81	224.16	240.82
273.85	291.52	309.43	341.10	366.24	386.25
505.48	546.55	617.34	654.31	742.77	819.95
839.27	883.16	921.89	946.41	956.62	976.95
1012.42	1089.53	1108.64	1189.56	1201.44	1208.74
1225.05	1292.21	1320.26	1350.37	1369.50	1378.35
1386.39	1391.83	1397.88	1409.51	1470.07	1476.49
1493.64	1496.90	1510.19	1665.13	1859.09	3005.32
3016.88	3026.47	3033.57	3048.43	3081.85	3102.79
3103.96	3111.82	3129.68	3539.56	3623.48	3841.67

- Conformer (40); ZPE = 42715.680 cm^{-1}:

63.75	71.33	120.48	174.60	195.43	244.18
293.19	304.35	347.27	359.91	388.28	397.21
532.20	550.15	627.45	759.85	774.30	808.20
832.13	868.75	903.37	918.48	956.79	972.69
1050.18	1094.03	1109.03	1155.80	1201.70	1211.64
1240.88	1271.08	1308.55	1354.71	1371.35	1386.11
1398.00	1402.72	1418.43	1432.57	1466.95	1475.51
1489.88	1497.55	1501.72	1657.08	1885.23	3016.87
3025.58	3031.38	3031.95	3042.52	3091.10	3093.94
3102.13	3109.77	3127.94	3572.90	3655.16	3685.09

- Conformer (41); ZPE = 42616.464 cm^{-1}:

68.06	77.10	123.19	174.72	199.43	227.99
273.04	301.23	305.41	340.15	382.87	397.96
488.00	536.71	616.29	644.45	747.40	784.11
825.69	902.34	918.04	918.45	965.22	988.19
1019.96	1099.36	1143.90	1159.30	1171.94	1216.73
1237.62	1278.96	1311.50	1358.65	1366.71	1380.70
1386.25	1399.56	1416.25	1421.25	1469.20	1478.14
1486.16	1499.34	1501.97	1673.03	1858.78	3011.79
3020.89	3025.97	3037.83	3050.12	3095.58	3098.82
3108.59	3110.13	3129.12	3543.25	3616.31	3843.29

- Conformer (42); ZPE = 42553.972 cm^{-1}:

33.15	68.49	93.79	177.91	245.79	250.18
276.25	287.99	316.51	347.53	376.77	425.28
433.16	550.72	583.04	644.09	757.29	800.30
857.11	878.95	923.14	934.85	971.09	973.80
1024.22	1104.52	1128.80	1168.35	1191.70	1203.28
1216.90	1274.08	1312.45	1332.79	1352.88	1386.05
1389.06	1397.30	1405.08	1410.91	1468.37	1479.69
1482.43	1494.04	1503.14	1663.53	1855.92	2987.03
3022.57	3023.18	3025.81	3049.63	3095.08	3099.19
3099.86	3116.18	3117.95	3543.24	3627.63	3847.91

- Conformer (43); ZPE = 42518.949 cm^{-1}:

70.53	81.84	118.01	145.32	207.01	222.37
254.74	274.83	296.06	313.74	374.86	414.62
449.53	517.39	606.64	646.19	772.04	810.22
853.91	876.04	922.69	955.91	962.82	979.74
1051.61	1119.66	1132.24	1167.37	1177.97	1198.05
1225.22	1250.97	1299.29	1332.13	1353.85	1357.06
1382.68	1395.84	1414.79	1460.44	1474.86	1478.44
1485.32	1496.15	1497.54	1641.91	1866.10	3009.58
3017.43	3024.84	3032.79	3048.19	3066.99	3098.64
3105.09	3111.82	3127.03	3538.44	3630.33	3840.16

- Conformer (44); ZPE = 42468.536 cm^{-1}:

15.02	49.97	85.54	176.68	223.99	237.47
245.12	279.95	315.93	324.72	407.07	414.24
428.91	524.93	589.78	649.56	743.28	824.53
851.17	873.24	924.03	952.48	954.88	978.96
1023.14	1083.95	1143.95	1172.97	1195.60	1204.63
1223.06	1284.61	1306.26	1338.19	1366.72	1374.60
1390.51	1394.80	1397.66	1416.09	1466.27	1471.37
1482.99	1494.57	1495.58	1662.73	1858.41	2989.49
2997.31	3012.24	3025.17	3029.27	3096.81	3098.18
3102.25	3114.29	3129.59	3545.64	3631.87	3844.82

- Conformer (45); ZPE = 42553.844 cm^{-1}:

50.83	68.79	109.35	166.55	223.60	244.77
265.29	286.86	313.17	327.02	348.32	419.39
458.40	519.25	625.98	645.63	792.93	822.82
839.44	877.20	918.48	928.17	970.80	980.96
1078.97	1088.61	1133.72	1150.19	1180.11	1195.79
1222.27	1256.22	1303.78	1332.25	1359.01	1369.83
1386.76	1394.83	1411.79	1459.39	1470.14	1480.57
1485.24	1495.60	1509.01	1644.74	1863.90	3001.78
3014.28	3026.58	3029.45	3056.33	3069.15	3101.76
3106.56	3112.84	3115.00	3532.44	3625.52	3839.29

- Conformer (46); ZPE = 42540.421 cm^{-1}:

73.40	82.97	114.75	189.74	220.73	239.49
248.34	289.40	318.31	338.20	380.76	419.48
505.17	526.91	581.71	652.05	766.85	776.47
839.38	858.94	902.30	927.43	955.18	976.92
1015.25	1105.12	1147.22	1156.06	1194.46	1204.72
1234.01	1266.76	1285.40	1333.64	1361.70	1366.72
1391.15	1396.18	1414.45	1442.82	1468.54	1480.68
1485.46	1496.62	1507.65	1652.88	1856.71	3008.30
3017.56	3024.60	3026.30	3028.78	3073.62	3099.89
3103.88	3108.25	3112.33	3547.10	3635.66	3845.49

- Conformer (47); ZPE = 42501.501 cm^{-1}:

47.87	52.40	96.50	182.62	239.43	251.93
261.60	281.72	318.39	350.34	377.28	420.86
444.94	556.79	574.22	647.32	756.45	797.41
829.46	903.14	920.60	935.08	947.30	970.46
1023.60	1095.19	1139.94	1175.83	1187.39	1197.49
1220.74	1260.89	1319.66	1332.43	1348.21	1365.27
1387.18	1398.46	1406.96	1448.18	1468.04	1479.19
1484.62	1492.13	1502.76	1646.95	1866.76	2935.85
3019.65	3021.13	3024.46	3033.91	3086.21	3094.23
3099.23	3113.44	3119.94	3553.29	3641.74	3847.96

- Conformer (48); ZPE = 42610.185 cm^{-1}:

8.31	71.57	92.41	191.04	220.61	225.60
253.59	312.00	320.58	345.19	375.79	428.68
437.57	551.84	671.02	762.88	782.36	829.22
846.55	883.38	922.02	946.78	957.97	978.28
1013.79	1097.44	1129.96	1158.45	1192.04	1207.05
1249.59	1266.25	1307.61	1348.34	1356.18	1373.46
1385.82	1397.30	1417.21	1422.28	1470.94	1476.02
1484.64	1495.38	1497.07	1656.38	1892.42	2995.83
3012.63	3028.04	3029.31	3031.79	3058.27	3101.87
3105.08	3116.97	3132.51	3548.70	3637.15	3711.36

- Conformer (49); ZPE = 42469.920 cm^{-1}:

15.32	31.16	98.46	137.31	223.24	240.52
260.61	268.37	290.06	315.93	377.75	419.73
441.00	543.43	606.63	638.79	762.01	828.46
855.30	889.84	922.81	940.01	955.00	970.26
1010.66	1087.24	1134.82	1184.13	1196.17	1198.50
1218.54	1277.71	1310.22	1332.85	1359.54	1382.04
1385.47	1392.14	1396.08	1409.09	1468.71	1481.01
1485.65	1494.92	1506.85	1661.71	1861.57	3000.14
3015.76	3024.46	3029.26	3058.50	3073.51	3096.61
3101.57	3115.90	3146.74	3538.92	3624.43	3846.45

- Conformer (50); ZPE = 42517.527 cm^{-1}:

45.54	75.06	110.03	179.77	232.75	243.10
278.13	285.41	316.62	329.43	351.70	408.25
459.61	546.68	588.32	626.62	766.76	808.96
841.23	869.47	920.46	942.52	965.54	976.65
1041.44	1097.55	1147.22	1161.00	1186.56	1197.98
1236.00	1263.98	1299.43	1325.59	1351.71	1370.46
1392.33	1396.51	1414.89	1452.18	1465.24	1476.07
1482.74	1494.49	1500.90	1650.51	1879.75	2940.15
2993.51	3026.91	3030.89	3033.14	3082.39	3102.05
3106.41	3112.32	3114.06	3551.17	3640.23	3848.70

- Conformer (51); ZPE = 42507.963 cm^{-1}:

41.04	71.29	91.82	178.85	229.42	239.04
265.76	314.72	325.83	332.50	406.71	423.29
429.59	531.66	581.63	627.26	748.67	820.02
837.96	886.11	922.98	928.91	959.05	972.63
1028.56	1101.33	1150.51	1165.47	1184.26	1199.26
1238.82	1268.89	1295.87	1334.80	1351.56	1364.00
1386.38	1393.88	1407.97	1447.14	1474.28	1482.08
1486.93	1496.50	1506.74	1649.84	1870.63	2911.06
3003.92	3015.31	3026.80	3031.28	3090.39	3098.72
3102.26	3106.26	3115.32	3562.46	3651.51	3848.20

- Conformer (52); ZPE = 42566.151 cm^{-1}:

49.86	76.07	112.38	160.26	232.71	247.55
257.88	273.89	297.73	302.52	368.97	381.08
492.76	538.67	633.62	646.15	776.43	804.82
826.57	879.58	917.15	944.63	972.74	988.42
1028.28	1101.52	1127.59	1156.50	1171.85	1210.77
1225.90	1257.72	1322.82	1332.67	1357.88	1372.81
1384.60	1396.36	1415.38	1455.19	1472.91	1477.26
1494.11	1497.05	1508.09	1641.55	1867.13	3009.11
3020.89	3025.36	3038.21	3060.25	3082.81	3100.00
3110.95	3112.43	3121.31	3533.20	3624.97	3834.45

- Conformer (53); ZPE = 42576.092 cm^{-1}:

71.31	82.58	121.37	161.55	236.28	249.71
259.79	278.71	287.93	306.39	370.68	384.43
522.64	539.08	599.66	608.76	773.91	806.66
823.35	868.93	919.51	938.21	970.84	988.76
1029.01	1097.65	1110.43	1133.19	1187.37	1211.83
1229.03	1264.75	1327.01	1342.79	1365.33	1374.98
1382.61	1398.74	1416.06	1432.76	1468.17	1476.19
1496.36	1500.73	1511.89	1648.78	1874.23	3010.51
3022.14	3024.56	3034.92	3060.95	3091.18	3100.35
3109.56	3111.46	3116.83	3539.48	3629.80	3849.53

- Conformer (54); ZPE = 42551.164 cm^{-1}:

26.79	67.85	123.21	166.37	219.52	244.66
251.41	286.39	300.45	313.97	372.02	388.17
488.82	544.25	623.64	641.49	790.98	811.90
819.78	869.55	916.11	943.70	979.17	988.95
1027.36	1089.69	1131.02	1141.55	1175.73	1209.21
1216.18	1255.71	1314.01	1339.53	1362.05	1368.17
1390.85	1397.84	1415.90	1459.26	1471.67	1477.18
1492.61	1498.36	1509.29	1642.99	1864.13	3014.66
3025.44	3026.96	3030.29	3065.19	3084.24	3101.07
3104.51	3109.63	3116.79	3530.12	3623.27	3840.74

- Conformer (55); ZPE = 42642.717 cm^{-1}:

66.49	83.06	128.27	192.08	238.14	260.01
296.55	326.33	336.13	351.98	390.69	395.92
505.46	537.70	586.86	662.81	750.66	776.91
813.27	891.22	914.60	923.53	953.63	976.11
1023.49	1091.14	1124.49	1177.52	1185.31	1205.49
1229.54	1275.01	1328.73	1348.20	1363.82	1377.85
1384.70	1390.54	1411.24	1440.92	1466.85	1478.43
1498.03	1500.46	1515.47	1647.67	1861.69	2931.74
3009.19	3024.98	3028.95	3033.42	3087.62	3097.95
3099.89	3118.12	3128.28	3551.20	3641.36	3847.75

- Conformer (56); ZPE = 42632.172 cm^{-1}:

66.18	100.60	135.70	173.44	189.66	248.06
274.82	311.13	317.86	342.02	380.77	412.64
493.60	546.43	606.91	653.75	741.86	787.50
841.57	872.51	902.60	929.71	957.08	973.04
1030.76	1090.67	1120.67	1172.39	1205.05	1214.74
1238.52	1280.77	1298.18	1335.63	1362.28	1376.31
1384.87	1396.65	1414.70	1448.02	1471.48	1478.19
1493.71	1497.54	1510.24	1649.96	1857.20	3002.00
3009.44	3024.97	3029.38	3035.05	3070.50	3105.42
3108.39	3111.81	3117.70	3559.61	3652.05	3850.05

- Conformer (57); ZPE = 42542.073 cm^{-1}:

40.91	58.24	105.01	197.70	219.64	241.40
262.08	313.54	335.84	338.67	382.29	436.17
479.18	528.18	578.29	632.07	740.68	817.88
829.91	882.71	918.62	927.32	967.19	982.40
1007.80	1083.82	1133.37	1167.18	1182.94	1207.93
1226.70	1267.12	1307.34	1332.65	1351.79	1365.51
1389.79	1394.77	1412.91	1444.68	1474.20	1479.23
1489.74	1502.30	1503.46	1649.91	1870.05	2930.36
3013.47	3024.17	3029.87	3045.48	3087.58	3100.40
3102.38	3107.65	3115.87	3563.01	3651.09	3851.72

- Conformer (58); ZPE = 42471.686 cm^{-1}:

38.39	83.57	115.60	151.40	206.39	222.39
241.20	274.02	287.33	319.10	376.10	426.86
446.75	541.89	592.46	605.16	774.35	812.88
846.20	870.07	920.33	944.62	966.00	977.49
1053.50	1099.22	1118.14	1140.79	1186.69	1198.84
1223.62	1257.59	1302.09	1346.47	1357.40	1364.38
1384.25	1394.86	1412.39	1433.21	1469.13	1476.79
1486.37	1495.59	1498.97	1649.66	1873.95	3005.84
3012.90	3023.73	3032.70	3055.36	3076.44	3097.56
3105.78	3113.99	3132.65	3542.27	3630.85	3848.89

- Conformer (59); ZPE = 42466.752 cm^{-1}:

14.69	77.30	109.80	167.23	203.35	240.07
243.97	252.88	285.92	326.81	369.57	429.27
454.75	542.11	591.93	611.11	780.79	816.72
843.03	873.80	917.98	927.63	970.65	975.72
1058.79	1089.79	1120.09	1134.38	1186.94	1192.22
1230.44	1259.36	1301.71	1351.51	1357.25	1365.81
1381.07	1394.75	1410.50	1428.25	1465.68	1479.56
1485.23	1494.43	1509.92	1648.33	1878.20	3004.52
3012.26	3025.52	3031.38	3054.21	3081.76	3100.11
3104.34	3114.77	3133.37	3539.60	3629.80	3850.55

- Conformer (60); ZPE = 42468.824 cm^{-1}:

51.53	66.25	91.35	137.59	222.68	230.48
260.30	279.86	310.80	326.80	375.95	412.47
447.62	521.10	585.32	654.92	758.00	819.91
846.95	861.58	916.91	925.22	964.13	969.71
1035.59	1120.68	1136.11	1157.49	1174.08	1198.65
1225.77	1261.74	1304.50	1331.09	1350.48	1367.03
1383.64	1394.58	1410.87	1454.96	1475.86	1477.83
1485.51	1494.20	1501.47	1633.58	1869.75	2991.08
3013.29	3025.88	3030.03	3038.93	3072.13	3100.59
3104.34	3114.69	3118.98	3549.09	3649.14	3846.59

- Conformer (61); ZPE = 42570.266 cm^{-1}:

41.32	74.82	113.51	177.08	217.99	250.41
282.88	300.19	306.20	336.62	377.16	401.37
485.15	564.38	581.07	619.51	760.83	807.47
819.50	881.65	922.43	946.73	975.47	984.70
1004.11	1097.94	1132.54	1157.32	1184.22	1207.39
1226.61	1267.73	1316.94	1327.21	1356.65	1362.42
1395.32	1397.29	1416.93	1453.14	1469.00	1474.41
1488.40	1496.40	1501.05	1650.48	1877.77	2962.16
3020.80	3025.44	3029.92	3040.74	3095.08	3096.16
3099.83	3112.37	3129.78	3550.49	3639.43	3848.60

- Conformer (62); ZPE = 42643.041 cm^{-1}:

66.64	77.53	124.75	180.62	204.54	237.47
293.61	302.53	308.77	348.23	389.80	394.84
519.05	527.29	593.74	664.62	748.04	780.32
823.35	890.58	908.26	919.48	963.22	983.58
1015.11	1097.80	1136.55	1157.22	1192.17	1215.52
1226.38	1282.42	1315.41	1358.82	1367.82	1383.51
1386.18	1394.24	1406.10	1419.49	1465.79	1478.91
1486.29	1498.06	1502.26	1670.20	1854.83	3011.57
3022.77	3023.60	3038.55	3051.50	3093.92	3098.65
3109.24	3117.80	3128.79	3552.03	3630.21	3845.50

- Conformer (63); ZPE = 42528.537 cm^{-1}:

45.78	58.19	113.42	172.89	224.05	229.03
271.09	279.19	313.14	335.67	343.16	414.85
463.58	558.68	588.80	617.29	791.10	803.68
842.43	876.42	920.47	937.16	961.99	978.19
1038.74	1096.42	1119.13	1155.18	1197.16	1218.61
1244.47	1261.18	1300.70	1352.00	1369.13	1373.50
1394.48	1401.28	1414.33	1429.09	1470.94	1482.11
1485.10	1495.37	1508.39	1651.71	1859.67	2982.66
3001.16	3022.39	3026.48	3028.93	3079.73	3101.61
3105.48	3109.54	3114.10	3545.44	3634.30	3846.35

- Conformer (64); ZPE = 42504.482 cm^{-1}:

41.46	69.52	102.39	140.12	225.00	246.59
274.90	310.93	319.75	343.63	375.94	424.58
427.98	529.88	575.46	622.27	746.21	843.09
853.92	864.88	925.91	947.08	964.72	970.81
1025.74	1120.72	1135.60	1174.22	1185.89	1199.33
1224.56	1270.64	1297.13	1332.83	1349.22	1368.83
1386.90	1394.34	1411.96	1446.86	1470.57	1477.77
1484.03	1494.48	1503.79	1654.93	1872.63	2922.02
2979.64	3024.52	3031.31	3040.99	3091.86	3099.02
3103.44	3114.03	3130.40	3553.66	3637.82	3850.30

- Conformer (65); ZPE = 42491.112 cm^{-1}:

43.14	54.85	102.70	145.69	168.11	218.06
246.35	284.18	322.78	334.72	364.73	376.59
492.51	550.01	592.19	663.89	735.29	819.16
825.37	854.33	903.27	918.21	962.08	985.30
1030.60	1095.68	1136.42	1167.81	1176.58	1207.05
1234.11	1268.38	1313.93	1340.57	1358.34	1377.06
1383.06	1398.53	1416.64	1452.39	1470.50	1474.55
1487.66	1496.28	1498.75	1634.24	1867.27	3012.62
3023.08	3026.01	3038.13	3046.15	3072.48	3097.05
3110.76	3114.08	3117.41	3557.41	3669.72	3847.40

- Conformer (66); ZPE = 42561.775 cm^{-1}:

41.58	72.68	110.70	165.68	190.91	237.81
280.26	299.40	325.89	355.49	374.74	402.72
472.27	530.83	590.88	640.31	742.94	807.32
850.97	856.49	916.90	928.19	963.60	989.17
1020.34	1093.17	1142.42	1175.91	1184.84	1205.13
1232.61	1279.10	1310.82	1347.64	1352.84	1373.68
1391.50	1399.28	1416.04	1444.08	1467.36	1475.20
1486.25	1495.89	1497.08	1654.19	1871.31	2936.81
3017.68	3023.93	3040.43	3046.57	3091.49	3096.13
3112.11	3113.77	3124.44	3559.94	3644.26	3851.59

- Conformer (67); ZPE = 42644.648 cm^{-1}:

64.20	84.71	122.24	172.63	229.41	247.34
256.30	287.87	313.01	342.78	388.30	407.58
509.32	564.68	582.60	669.27	741.62	788.21
835.90	862.86	892.17	926.26	966.79	979.60
1008.05	1094.32	1124.06	1161.22	1198.93	1218.47
1230.08	1273.94	1312.80	1360.34	1365.21	1377.07
1391.05	1396.08	1402.72	1416.22	1458.06	1471.97
1493.11	1499.63	1513.56	1669.30	1858.99	3013.32
3022.60	3038.18	3041.58	3054.45	3092.78	3095.53
3110.93	3113.77	3121.47	3564.45	3648.35	3841.07

- Conformer (68); ZPE = 42410.260 cm^{-1}:

47.57	69.75	101.87	142.33	166.98	223.24
236.64	260.85	310.10	331.61	382.94	420.42
430.66	496.61	527.32	672.68	730.69	832.74
855.81	884.92	925.91	958.81	969.12	985.11
1001.29	1076.84	1146.10	1167.20	1195.05	1199.08
1229.61	1277.02	1307.27	1327.98	1349.61	1369.18
1389.93	1392.61	1409.97	1410.95	1466.37	1478.06
1485.00	1493.50	1505.14	1669.11	1895.22	3000.72
3008.20	3016.30	3024.23	3043.47	3058.04	3095.82
3100.76	3116.17	3119.21	3534.30	3612.61	3883.94

- Conformer (69); ZPE = 42569.551 cm^{-1}:

38.55	52.36	123.88	176.02	216.94	242.79
263.45	278.47	309.07	315.51	379.97	391.98
513.26	564.07	592.83	616.29	792.54	796.87
821.77	883.15	919.03	943.64	971.99	980.82
1004.26	1097.09	1120.18	1130.53	1204.51	1209.37
1241.49	1265.53	1318.66	1356.73	1360.60	1374.42
1395.81	1404.50	1416.81	1427.92	1476.09	1476.71
1490.91	1499.29	1507.00	1651.32	1858.61	3006.06
3018.80	3026.16	3029.59	3031.94	3094.14	3097.68
3105.59	3111.30	3119.50	3545.24	3633.79	3845.74

- Conformer (70); ZPE = 42508.661 cm^{-1}:

37.07	45.68	88.04	184.80	228.98	240.77
263.36	304.37	317.74	346.72	360.80	420.30
481.87	550.72	573.13	650.89	777.55	789.40
834.57	864.53	911.21	926.97	944.87	968.53
1036.44	1093.29	1137.93	1142.90	1197.37	1198.92
1240.41	1259.85	1286.14	1337.42	1351.64	1369.55
1391.31	1393.76	1410.49	1440.35	1469.87	1478.79
1484.69	1494.47	1500.90	1653.47	1863.11	2996.30
3021.79	3025.00	3028.97	3053.40	3076.12	3097.97
3102.08	3117.38	3120.52	3548.42	3638.61	3844.80

- Conformer (71); ZPE = 42487.750 cm^{-1}:

41.56	65.79	93.97	136.77	219.79	235.09
261.27	284.47	309.83	321.87	371.20	416.25
445.43	545.16	592.93	633.06	770.32	827.68
854.60	863.50	906.33	924.03	964.46	968.91
1035.20	1108.78	1119.99	1142.20	1195.54	1197.17
1230.72	1262.56	1306.99	1341.59	1357.77	1370.35
1383.12	1393.62	1409.63	1433.27	1474.68	1477.37
1485.57	1494.35	1500.02	1648.13	1873.56	2992.16
3012.67	3027.11	3031.17	3061.53	3076.66	3101.47
3105.37	3116.97	3122.91	3543.91	3637.48	3849.62

- Conformer (72); ZPE = 42497.818 cm^{-1}:

39.99	64.65	108.10	157.52	204.21	215.85
253.48	275.56	313.84	320.85	364.67	378.21
505.28	551.28	592.27	645.94	747.34	819.20
837.29	855.46	892.41	917.23	962.02	984.76
1032.12	1085.95	1105.86	1141.69	1195.33	1209.53
1233.49	1272.17	1320.64	1345.49	1371.79	1374.03
1386.46	1396.07	1412.83	1431.43	1469.87	1474.94
1487.23	1496.08	1498.82	1647.80	1871.43	3009.74
3021.59	3027.64	3035.81	3060.48	3085.76	3098.64
3110.54	3114.66	3115.83	3550.69	3646.57	3849.25

- Conformer (73); ZPE = 42541.419 cm^{-1}:

59.83	77.35	109.90	191.41	234.03	240.49
261.71	275.73	304.64	352.89	385.19	414.18
459.40	495.72	542.50	627.87	767.92	783.55
850.93	891.53	925.46	927.20	970.66	987.04
1010.12	1107.26	1142.26	1168.05	1178.94	1199.58
1242.29	1274.63	1295.90	1331.49	1363.30	1372.50
1391.03	1397.19	1408.84	1418.76	1468.68	1480.61
1486.09	1495.82	1506.53	1673.82	1888.64	2997.12
3007.73	3020.88	3024.56	3031.04	3074.57	3099.16
3102.45	3117.81	3118.82	3542.42	3616.27	3890.51

- Conformer (74); ZPE = 42475.059 cm^{-1}:

41.97	91.13	121.59	158.01	214.25	224.79
233.10	270.92	288.19	321.25	377.47	418.87
440.35	513.81	526.30	663.69	761.80	815.14
843.40	895.88	923.58	951.01	964.12	976.22
1038.45	1097.52	1137.70	1167.97	1180.34	1195.76
1220.75	1261.94	1294.42	1337.26	1359.82	1370.62
1383.57	1397.54	1416.23	1422.23	1468.66	1475.34
1485.35	1496.13	1499.86	1670.19	1891.02	3016.77
3018.92	3022.40	3029.28	3033.43	3074.35	3085.30
3103.65	3112.06	3118.29	3534.74	3615.62	3879.79

- Conformer (75); ZPE = 42571.042 cm^{-1}:

67.43	76.24	124.70	185.52	229.85	245.91
251.97	306.86	315.32	339.53	387.46	393.42
521.99	529.46	585.43	662.31	745.82	782.33
814.40	850.45	906.07	923.91	951.04	976.55
1042.15	1090.04	1113.39	1155.15	1201.08	1212.33
1234.65	1275.36	1289.77	1359.20	1363.17	1373.54
1387.43	1393.59	1414.88	1442.15	1468.63	1479.84
1489.78	1499.24	1506.09	1651.98	1857.11	3009.07
3013.25	3026.56	3030.21	3032.78	3079.05	3098.83
3104.47	3113.28	3113.49	3554.91	3644.31	3847.35

- Conformer (76); ZPE = 42484.976 cm^{-1}:

52.17	74.94	113.94	190.59	224.39	246.64
257.16	293.83	333.65	358.61	390.56	411.84
477.15	500.67	547.65	621.14	760.79	787.53
836.50	876.90	904.62	923.21	953.30	977.39
1011.22	1101.83	1149.45	1167.88	1193.26	1199.80
1231.66	1254.79	1303.88	1317.85	1343.85	1368.26
1392.10	1394.89	1413.74	1434.62	1473.72	1484.32
1487.30	1494.96	1512.19	1631.06	1886.02	2930.04
2985.92	3021.80	3023.49	3046.16	3058.86	3095.21
3102.41	3114.39	3120.92	3557.73	3653.02	3896.20

- Conformer (77); ZPE = 42503.134 cm^{-1}:

58.33	93.21	96.25	131.37	191.83	235.31
260.69	278.41	321.89	347.74	381.44	416.28
464.87	495.82	532.95	642.39	779.07	788.78
861.05	875.59	914.42	933.22	958.53	977.23
1024.57	1109.44	1124.59	1167.51	1188.14	1204.16
1231.93	1269.52	1287.17	1331.25	1359.18	1368.30
1389.50	1395.88	1412.83	1422.69	1467.77	1483.03
1486.40	1496.95	1517.50	1671.11	1894.29	3006.76
3007.22	3020.97	3024.00	3027.37	3070.62	3099.47
3103.68	3115.63	3116.56	3556.15	3633.34	3884.15

- Conformer (78); ZPE = 42469.366 cm^{-1}:

47.41	87.61	117.91	158.98	221.87	230.94
256.36	272.49	307.04	314.62	377.32	422.90
437.07	502.96	525.47	661.44	763.39	819.94
839.06	876.76	921.54	928.47	965.99	981.93
1046.41	1122.64	1133.58	1164.73	1185.00	1196.12
1235.35	1247.28	1292.19	1332.15	1344.92	1366.88
1381.98	1397.35	1414.28	1446.65	1467.01	1475.61
1484.54	1496.52	1498.73	1641.35	1895.51	3002.90
3010.05	3021.18	3024.59	3028.84	3064.22	3090.21
3101.67	3111.96	3118.12	3538.22	3629.90	3890.58

- Conformer (79); ZPE = 42578.041 cm^{-1}:

41.37	55.37	131.66	167.27	216.95	254.38
278.43	298.93	301.76	345.62	349.26	393.90
477.59	559.35	580.37	614.91	758.34	785.23
828.75	869.82	917.15	960.75	969.69	982.69
1034.34	1120.00	1141.51	1145.74	1181.33	1210.43
1232.21	1281.32	1312.52	1338.88	1354.45	1368.44
1389.90	1393.59	1411.60	1457.40	1473.09	1480.04
1486.39	1501.53	1503.25	1647.61	1876.79	2976.71
3015.32	3026.08	3034.79	3037.69	3083.83	3101.41
3105.29	3107.73	3142.76	3551.68	3640.64	3850.26

- Conformer (80); ZPE = 42518.258 cm^{-1}:

54.89	63.07	110.61	170.39	205.18	229.10
246.70	292.98	315.49	339.78	364.48	387.14
483.91	496.95	546.64	674.44	724.43	820.10
851.37	892.93	921.60	958.65	968.52	979.12
1017.61	1090.57	1115.49	1165.60	1195.41	1211.34
1229.14	1285.19	1325.42	1325.96	1364.71	1371.47
1385.71	1392.65	1410.20	1413.73	1469.62	1476.03
1493.12	1497.07	1509.94	1671.29	1895.96	2999.53
3009.54	3020.38	3023.82	3035.19	3058.80	3098.37
3106.03	3113.76	3132.84	3531.26	3608.23	3887.09

- Conformer (81); ZPE = 42493.546 cm^{-1}:

43.16	95.58	108.76	171.67	214.06	227.45
236.78	262.44	327.63	361.69	394.97	415.80
467.98	505.56	555.22	652.94	770.91	791.77
839.71	876.30	916.87	927.59	959.79	976.32
1012.83	1107.39	1135.25	1173.51	1197.56	1205.13
1230.43	1261.81	1285.81	1317.42	1344.59	1352.63
1393.93	1396.98	1417.14	1437.24	1470.72	1480.35
1488.23	1496.05	1503.01	1646.70	1893.14	2971.76
3005.93	3017.93	3024.07	3032.08	3051.91	3097.89
3104.65	3113.59	3135.83	3550.36	3643.95	3888.34

- Conformer (82); ZPE = 42521.658 cm^{-1}:

48.92	67.46	84.43	187.74	211.08	236.12
256.72	272.80	306.70	348.09	362.68	428.66
465.13	506.19	539.04	646.77	779.07	798.94
850.18	907.10	928.37	936.35	971.04	975.37
1022.48	1110.81	1133.12	1167.26	1183.81	1200.74
1233.91	1263.54	1285.64	1334.19	1351.91	1368.84
1393.16	1399.73	1413.08	1422.60	1472.90	1480.22
1483.66	1494.54	1504.35	1673.41	1892.08	2999.28
3014.25	3023.08	3025.05	3030.43	3073.21	3098.16
3103.33	3115.23	3132.18	3532.42	3606.86	3888.91

- Conformer (83); ZPE = 42398.445 cm^{-1}:

24.60	54.67	96.55	149.08	189.67	227.78
252.89	266.96	300.17	313.82	384.52	419.03
438.91	489.51	512.72	674.39	735.67	843.38
858.21	894.58	924.10	946.20	969.64	980.12
1016.12	1086.70	1140.30	1164.20	1196.52	1198.24
1219.35	1271.08	1309.94	1320.54	1349.31	1364.07
1386.39	1393.49	1408.69	1413.86	1469.10	1480.56
1484.14	1495.56	1507.38	1669.04	1894.32	2996.71
3005.70	3013.86	3024.44	3029.77	3055.15	3097.87
3101.61	3114.06	3147.94	3529.36	3609.19	3885.14

- Conformer (84); ZPE = 42457.071 cm^{-1}:

68.77	77.70	118.63	149.76	207.80	217.88
224.70	273.60	289.89	317.23	375.67	415.37
452.58	488.46	522.93	660.98	759.41	817.29
857.30	902.60	926.65	940.59	971.45	993.90
1041.56	1085.72	1140.81	1159.42	1171.63	1198.33
1224.09	1266.32	1300.04	1320.94	1347.59	1363.72
1382.60	1396.50	1417.05	1422.23	1471.12	1476.41
1485.92	1496.97	1500.67	1668.32	1891.35	2974.82
3008.18	3022.72	3027.48	3034.41	3084.49	3101.91
3106.64	3116.49	3134.94	3534.57	3615.60	3891.43

- Conformer (85); ZPE = 42603.043 cm^{-1}:

80.45	95.41	124.90	147.11	209.05	251.56
272.12	296.21	311.74	340.88	389.15	415.55
476.32	494.70	580.58	635.24	752.40	790.59
852.88	878.57	900.06	928.40	973.30	987.93
1013.84	1097.71	1134.58	1164.06	1174.24	1221.37
1235.40	1264.89	1289.04	1337.71	1365.52	1377.10
1386.27	1398.10	1416.16	1430.79	1464.41	1474.86
1494.48	1503.66	1515.65	1673.82	1890.14	3004.89
3015.93	3021.90	3026.73	3033.06	3081.75	3101.72
3106.16	3117.80	3119.51	3552.87	3627.58	3887.29

- Conformer (86); ZPE = 42509.407 cm^{-1}:

65.06	74.73	114.83	161.10	239.30	243.78
255.72	260.53	285.12	311.07	369.23	379.73
497.23	506.82	558.67	657.65	773.27	800.92
833.70	891.99	914.56	947.88	973.97	988.21
1012.84	1087.71	1125.04	1148.99	1161.44	1208.70
1218.19	1271.71	1305.14	1345.89	1362.47	1369.74
1384.98	1396.92	1414.72	1419.81	1468.19	1470.86
1491.10	1493.18	1506.85	1662.81	1889.87	3014.87
3023.10	3024.68	3033.41	3038.23	3084.08	3090.90
3112.17	3115.10	3120.45	3529.62	3615.05	3894.99

- Conformer (87); ZPE = 42540.698 cm^{-1}:

72.30	81.00	122.19	198.52	236.27	247.11
264.75	317.89	338.33	354.78	394.20	405.31
482.91	492.81	567.34	621.78	759.56	777.17
820.61	883.41	907.06	915.87	950.12	973.66
1032.26	1095.63	1118.38	1174.92	1192.58	1207.51
1229.48	1259.69	1314.47	1320.48	1357.82	1378.64
1384.40	1392.20	1413.46	1432.74	1476.98	1486.16
1494.30	1501.16	1511.99	1629.36	1890.14	2913.83
2998.30	3014.86	3030.97	3033.43	3048.79	3102.17
3108.91	3114.79	3137.39	3554.94	3650.30	3893.01

- Conformer (88); ZPE = 42423.233 cm^{-1}:

41.21	57.03	90.28	184.26	221.72	237.21
255.83	288.31	326.08	346.41	384.96	419.61
450.99	498.36	524.51	615.60	734.93	818.28
836.47	881.34	920.40	924.22	965.38	980.54
1050.90	1094.48	1151.99	1172.10	1193.79	1208.64
1225.44	1258.71	1283.63	1318.16	1336.63	1376.96
1395.78	1396.69	1413.31	1423.19	1473.07	1478.58
1490.45	1495.73	1498.37	1635.00	1891.41	2930.90
2961.23	3025.29	3028.67	3030.72	3072.65	3102.73
3105.47	3116.69	3118.21	3553.55	3648.69	3884.73

- Conformer (89); ZPE = 42547.736 cm^{-1}:

66.86	74.18	122.56	171.99	195.75	225.95
262.86	305.07	310.33	341.95	382.05	398.65
474.74	493.91	578.15	621.94	755.57	784.34
824.28	900.51	917.95	921.43	966.40	986.53
1022.29	1102.08	1141.63	1162.66	1168.37	1217.50
1238.81	1276.57	1287.53	1353.94	1361.23	1374.96
1384.34	1401.63	1416.39	1423.80	1471.52	1477.44
1486.42	1500.07	1500.93	1674.60	1890.50	3001.46
3008.14	3021.27	3028.85	3039.50	3075.18	3102.79
3110.03	3111.35	3131.69	3540.82	3612.21	3893.01

- Conformer (90); ZPE = 42571.603 cm^{-1}:

72.28	80.26	133.68	137.37	197.14	247.65
274.68	314.53	329.46	341.49	381.20	418.66
480.47	498.83	587.91	639.44	749.99	788.61
833.57	872.56	901.26	929.26	956.72	976.07
1028.27	1091.42	1121.95	1177.84	1211.27	1214.36
1234.95	1278.63	1303.82	1322.80	1361.54	1377.58
1384.68	1400.05	1418.92	1435.30	1472.00	1476.67
1493.93	1499.36	1509.89	1654.73	1889.33	2953.04
3005.60	3017.10	3030.25	3036.80	3051.39	3106.27
3109.85	3114.72	3124.14	3555.84	3651.08	3884.70

- Conformer (91); ZPE = 42433.890 cm^{-1}:

49.14	66.83	92.18	140.02	225.20	234.22
262.88	281.10	311.86	331.45	378.03	412.26
448.77	496.39	526.94	672.48	741.64	816.71
850.54	857.64	918.73	925.02	964.15	970.69
1035.37	1120.51	1142.17	1158.04	1181.23	1199.54
1227.32	1253.20	1308.83	1322.93	1346.88	1363.50
1382.36	1395.65	1412.20	1439.03	1475.05	1477.63
1484.42	1495.20	1501.75	1631.54	1899.35	2982.19
2992.63	3012.59	3026.26	3031.25	3061.64	3101.49
3105.56	3114.48	3119.58	3548.71	3651.62	3891.20

- Conformer (92); ZPE = 42337.777 cm^{-1}:

30.43	60.97	100.90	163.16	181.02	220.53
235.26	263.32	303.01	329.99	352.39	414.19
461.17	491.42	542.98	618.12	753.61	798.99
822.97	866.09	922.89	930.13	965.49	984.16
1039.48	1094.36	1136.84	1156.18	1181.94	1196.84
1227.32	1266.63	1295.08	1300.72	1352.27	1361.14
1395.59	1400.85	1412.13	1442.30	1461.22	1477.01
1485.04	1495.54	1500.38	1652.95	1908.68	2934.84
3008.74	3023.21	3023.39	3037.26	3087.49	3096.98
3101.43	3110.62	3118.58	3549.43	3638.86	3891.03

- Conformer (93); ZPE = 42378.597 cm^{-1}:

40.22	69.04	111.34	175.93	210.75	226.74
244.69	280.75	314.75	326.76	346.85	412.46
462.94	487.41	546.69	632.30	761.17	799.04
841.28	853.59	918.90	929.38	965.01	978.10
1045.62	1093.45	1138.33	1156.87	1177.25	1196.82
1237.94	1257.19	1293.73	1300.97	1351.08	1368.17
1391.53	1396.81	1415.17	1443.60	1464.14	1476.81
1483.74	1494.94	1502.86	1652.84	1911.53	2900.94
2992.26	3026.86	3028.23	3030.48	3081.98	3099.84
3103.66	3112.70	3114.97	3547.85	3639.23	3890.76

- Conformer (94); ZPE = 42430.296 cm^{-1}:

42.73	67.51	118.39	174.17	198.78	216.20
248.63	286.21	308.51	327.17	377.06	394.93
493.63	503.00	570.66	630.01	758.94	794.21
821.05	860.17	915.11	941.48	974.60	983.42
1006.17	1093.18	1129.52	1145.90	1172.80	1205.68
1229.32	1260.01	1298.79	1313.19	1357.63	1359.77
1392.61	1397.16	1417.34	1448.12	1465.89	1474.56
1488.86	1496.57	1501.73	1654.07	1910.56	2927.01
3016.18	3025.31	3030.30	3037.62	3091.36	3096.34
3099.14	3110.73	3126.21	3546.47	3637.25	3890.68

- Conformer (95); ZPE = 42445.345 cm^{-1}:

46.58	74.80	119.59	153.16	188.67	251.26
258.17	271.94	295.90	306.22	371.61	383.88
503.25	512.15	560.30	646.57	761.94	778.27
824.09	848.78	916.29	950.53	963.55	984.16
1014.70	1105.20	1124.41	1151.67	1163.55	1211.16
1229.61	1261.82	1314.81	1325.86	1350.60	1367.38
1390.28	1396.31	1419.34	1439.46	1469.93	1471.87
1492.28	1493.53	1509.20	1656.66	1910.69	2935.59
3013.70	3020.62	3026.86	3038.56	3083.80	3090.00
3111.24	3112.72	3123.02	3553.02	3644.49	3895.12

6.5 Cartesian coordinates of Leu conformers

Each line starting by an asterisk contains the relative energy of the corresponding conformer and the dihedral angle for each of the target torsions (separated by underscores). Cartesian coordinates are given in Å.

```
22
 * E =  +0.000 kcal/mol ; (1) 179_172_306_181_183
 H   +0.00000   +0.00000   +0.00000
 O   +0.00000   +0.00000   +0.96381
 C   +1.27701   +0.00000   +1.37891
 C   +1.38723   +0.01675   +2.89638
 N   +2.74078   -0.17369   +3.36762
 H   +3.14106   -1.01657   +2.97270
 O   +2.20436   -0.00696   +0.61977
 C   +0.80696   +1.33493   +3.42613
 H   +0.76153   -0.80833   +3.26251
 H   +3.32514   +0.59098   +3.04486
 C   +0.89208   +1.47861   +4.94789
 H   +1.34121   +2.17132   +2.95173
 H   -0.24083   +1.40979   +3.10876
 C   +0.22499   +2.78537   +5.37053
 C   +0.25346   +0.28822   +5.65775
 H   +1.95196   +1.51189   +5.22787
 H   +0.32240   +2.94869   +6.44793
 H   +0.66505   +3.64576   +4.85588
 H   -0.84512   +2.76426   +5.13136
 H   +0.21322   +0.45693   +6.73806
 H   -0.77460   +0.13365   +5.30565
```

H +0.82141 −0.63083 +5.48621
22
∗ E = +0.167 kcal/mol ; (2) 357_015_093_280_053

H	+0.00000	+0.00000	+0.00000
O	+0.00000	+0.00000	+0.97204
C	+1.27348	+0.00000	+1.35541
C	+2.28706	−0.07544	+0.19870
N	+1.62011	+0.22214	−1.06943
H	+1.69532	+1.20519	−1.30377
O	+1.60375	+0.01163	+2.50481
C	+3.54697	+0.74041	+0.50844
H	+2.56635	−1.13668	+0.17670
H	+2.01257	−0.30680	−1.83583
C	+3.42131	+2.25021	+0.27509
H	+3.80296	+0.55996	+1.55948
H	+4.37685	+0.35700	−0.09758
C	+2.21660	+2.85480	+0.99657
C	+4.70896	+2.93750	+0.72531
H	+3.31268	+2.43285	−0.80524
H	+2.19601	+3.94096	+0.86652
H	+1.25962	+2.46532	+0.62861
H	+2.26372	+2.63635	+2.06887
H	+4.68056	+4.01027	+0.51281
H	+4.84431	+2.81214	+1.80550
H	+5.58571	+2.51354	+0.22562

22
∗ E = +0.248 kcal/mol ; (3) 007_336_153_056_056

H	+0.00000	+0.00000	+0.00000
O	+0.00000	+0.00000	+0.97166
C	+1.27417	+0.00000	+1.35788
C	+2.27974	+0.16719	+0.21514
N	+1.67447	−0.28179	−1.03821
H	+2.08122	+0.19329	−1.83386
O	+1.60390	−0.07991	+2.50512
C	+2.64607	+1.65150	+0.08000
H	+3.17208	−0.40431	+0.49660
H	+1.78335	−1.27822	−1.17988
C	+3.20349	+2.32854	+1.33436
H	+3.39659	+1.73022	−0.72163
H	+1.75452	+2.19220	−0.26781

C	+4.42681	+1.59227	+1.87693
C	+3.54887	+3.77909	+1.00363
H	+2.42711	+2.31964	+2.10943
H	+4.88857	+2.16103	+2.68945
H	+4.16022	+0.60802	+2.27042
H	+5.18107	+1.46670	+1.08903
H	+3.90365	+4.30963	+1.89175
H	+4.34468	+3.82156	+0.24997
H	+2.68293	+4.31977	+0.60842

22

∗ E = +0.366 kcal/mol ; (4) 357_016_092_177_181

H	+0.00000	+0.00000	+0.00000
O	+0.00000	+0.00000	+0.97157
C	+1.27306	+0.00000	+1.35771
C	+2.28716	−0.08102	+0.20732
N	+1.63417	+0.24459	−1.06115
H	+1.71918	+1.23413	−1.26790
O	+1.60200	+0.03016	+2.50682
C	+3.52272	+0.75598	+0.53256
H	+2.57000	−1.14118	+0.16774
H	+2.04541	−0.26114	−1.83447
C	+4.62157	+0.68524	−0.52971
H	+3.21186	+1.80148	+0.67189
H	+3.91648	+0.42457	+1.50069
C	+5.80837	+1.54325	−0.09726
C	+5.06910	−0.75296	−0.78623
H	+4.22702	+1.09716	−1.47059
H	+6.59031	+1.55577	−0.86228
H	+5.50459	+2.57644	+0.09753
H	+6.24711	+1.14530	+0.82499
H	+5.92301	−0.77968	−1.46952
H	+5.37553	−1.23056	+0.15226
H	+4.27707	−1.36647	−1.22916

22

∗ E = +0.440 kcal/mol ; (5) 183_184_307_284_058

H	+0.00000	+0.00000	+0.00000
O	+0.00000	+0.00000	+0.96395
C	+1.27844	+0.00000	+1.37374
C	+1.40893	−0.07606	+2.89320
N	+2.78212	+0.00909	+3.34061

H	+3.34860	−0.68183	+2.86238
O	+2.19959	+0.00666	+0.60625
C	+0.51545	+0.92954	+3.63235
H	+1.03015	−1.07828	+3.14108
H	+3.17437	+0.90993	+3.08780
C	+1.00985	+2.37924	+3.62311
H	−0.49344	+0.89099	+3.20361
H	+0.44027	+0.59291	+4.67199
C	+1.19355	+2.92064	+2.20530
C	+0.02297	+3.24950	+4.39850
H	+1.97558	+2.41646	+4.14592
H	+1.44132	+3.98621	+2.22658
H	+1.99371	+2.41086	+1.65806
H	+0.26620	+2.80419	+1.63059
H	+0.37206	+4.28441	+4.46242
H	−0.95247	+3.25546	+3.89763
H	−0.12516	+2.87616	+5.41653

22

∗ E = +0.620 kcal/mol ; (6) 178_144_295_057_059

H	+0.00000	+0.00000	+0.00000
O	+0.00000	+0.00000	+0.96461
C	+1.27676	+0.00000	+1.37929
C	+1.41216	+0.06022	+2.89478
N	+2.53355	−0.72155	+3.38025
H	+2.39865	−1.70837	+3.19636
O	+2.20361	−0.02219	+0.61908
C	+1.61568	+1.52522	+3.30383
H	+0.48813	−0.32381	+3.33933
H	+3.37559	−0.44271	+2.88647
C	+0.52990	+2.50684	+2.85874
H	+1.70182	+1.54089	+4.39762
H	+2.58750	+1.84652	+2.90347
C	−0.84277	+2.11411	+3.40313
C	+0.90335	+3.91450	+3.31870
H	+0.48145	+2.50776	+1.76011
H	−1.58995	+2.87508	+3.15808
H	−1.19131	+1.16282	+2.99191
H	−0.80451	+2.02438	+4.49590
H	+0.16385	+4.64883	+2.98586
H	+0.94708	+3.95659	+4.41348

```
 H    +1.88196    +4.21602    +2.93262
22
 * E =  +1.002 kcal/mol ; (7) 181_306_299_050_056
 H    +0.00000    +0.00000    +0.00000
 O    +0.00000    +0.00000    +0.96361
 C    +1.28012    +0.00000    +1.37943
 C    +1.37864    -0.01905    +2.89781
 N    +0.62304    +1.03888    +3.54770
 H    +0.95140    +1.95674    +3.27468
 O    +2.20407    -0.00194    +0.61592
 C    +0.89459    -1.37773    +3.41148
 H    +2.43939    +0.11315    +3.13106
 H    -0.35869    +0.97381    +3.30328
 C    +1.52772    -2.58811    +2.72162
 H    +1.09932    -1.41133    +4.48899
 H    -0.19690    -1.41779    +3.29176
 C    +3.05276    -2.55734    +2.81665
 C    +0.97453    -3.87276    +3.33326
 H    +1.24824    -2.56669    +1.65712
 H    +3.48441    -3.47827    +2.41331
 H    +3.48052    -1.71886    +2.25935
 H    +3.36567    -2.47284    +3.86494
 H    +1.37319    -4.75622    +2.82594
 H    +1.25479    -3.93990    +4.39116
 H    -0.11781    -3.90808    +3.27319
22
 * E =  +1.064 kcal/mol ; (8) 184_191_203_284_056
 H    +0.00000    +0.00000    +0.00000
 O    +0.00000    +0.00000    +0.96374
 C    +1.27745    +0.00000    +1.36900
 C    +1.41431    -0.08932    +2.87739
 N    +2.79427    +0.18995    +3.22218
 H    +3.03967    -0.20424    +4.11984
 O    +2.19789    -0.00224    +0.60014
 C    +0.44038    +0.81149    +3.65161
 H    +1.11531    -1.13456    +3.08429
 H    +3.42090    -0.17328    +2.51422
 C    +0.80114    +2.29939    +3.66916
 H    -0.56741    +0.67803    +3.23877
 H    +0.40128    +0.44788    +4.68672
```

C	+0.95915	+2.86852	+2.26158
C	−0.26655	+3.06673	+4.44555
H	+1.76296	+2.40577	+4.18468
H	+1.12058	+3.95028	+2.29922
H	+1.81733	+2.42804	+1.74416
H	+0.05629	+2.68536	+1.66577
H	−0.00806	+4.12653	+4.53089
H	−1.23477	+2.99967	+3.93473
H	−0.39442	+2.66653	+5.45679

22

* E = +1.158 kcal/mol ; (9) 359_007_106_313_184

H	+0.00000	+0.00000	+0.00000
O	+0.00000	+0.00000	+0.97319
C	+1.27442	+0.00000	+1.34919
C	+2.28788	−0.01937	+0.19353
N	+1.60133	+0.12430	−1.09485
H	+1.73474	+1.04509	−1.49142
O	+1.61657	−0.00472	+2.49595
C	+3.42236	+0.98327	+0.45938
H	+2.71599	−1.02806	+0.23016
H	+1.92710	−0.55334	−1.76932
C	+2.99666	+2.38140	+0.92504
H	+4.08221	+0.56064	+1.22398
H	+4.01480	+1.06646	−0.46320
C	+4.23214	+3.27203	+1.03190
C	+1.95256	+3.03056	+0.01666
H	+2.55809	+2.27441	+1.92476
H	+3.97502	+4.25775	+1.43061
H	+4.98792	+2.82778	+1.68673
H	+4.68711	+3.41879	+0.04462
H	+1.72174	+4.04266	+0.36176
H	+2.32753	+3.11853	−1.01271
H	+1.00851	+2.47422	+0.00222

22

* E = +1.273 kcal/mol ; (10) 179_170_297_317_181

H	+0.00000	+0.00000	+0.00000
O	+0.00000	+0.00000	+0.96396
C	+1.27773	+0.00000	+1.37907
C	+1.37883	+0.02958	+2.89763
N	+2.72004	−0.20366	+3.39209

H	+3.03475	−1.13092	+3.13300
O	+2.20473	−0.00521	+0.61954
C	+0.77661	+1.34912	+3.42093
H	+0.74113	−0.78405	+3.26412
H	+3.37539	+0.44505	+2.97391
C	+1.14750	+2.62649	+2.65726
H	−0.31516	+1.25703	+3.41683
H	+1.09491	+1.44307	+4.46660
C	+0.48140	+3.82016	+3.33932
C	+2.65668	+2.85210	+2.55179
H	+0.74032	+2.54655	+1.63818
H	+0.66492	+4.74539	+2.78515
H	−0.60092	+3.68156	+3.42437
H	+0.88341	+3.95086	+4.35100
H	+2.86719	+3.85089	+2.15701
H	+3.12794	+2.77681	+3.53944
H	+3.13151	+2.13309	+1.87681

22

∗ E = +1.322 kcal/mol ; (11) 177_012_304_280_056

H	+0.00000	+0.00000	+0.00000
O	+0.00000	+0.00000	+0.96399
C	+1.28317	+0.00000	+1.36544
C	+1.43940	+0.06543	+2.88615
N	+0.23492	−0.15416	+3.66158
H	−0.49443	+0.49325	+3.39078
O	+2.19516	−0.00338	+0.58768
C	+2.57982	−0.84898	+3.34803
H	+1.76389	+1.10076	+3.06221
H	−0.12967	−1.08683	+3.50566
C	+2.21527	−2.33350	+3.44899
H	+3.41902	−0.72793	+2.65228
H	+2.91094	−0.50089	+4.33213
C	+1.67005	−2.89021	+2.13335
C	+3.44608	−3.12463	+3.88676
H	+1.44743	−2.44600	+4.22726
H	+1.49716	−3.96786	+2.21237
H	+0.71748	−2.43057	+1.84323
H	+2.38324	−2.71741	+1.31902
H	+3.20870	−4.18360	+4.02598
H	+4.23218	−3.05390	+3.12587

H +3.85362 −2.74002 +4.82683
22
* E = +1.438 kcal/mol ; (12) 183_332_302_179_183
H +0.00000 +0.00000 +0.00000
O +0.00000 +0.00000 +0.96385
C +1.28150 +0.00000 +1.37118
C +1.41927 −0.07833 +2.88483
N +0.31941 +0.47048 +3.65195
H +0.15606 +1.44333 +3.42485
O +2.20096 +0.01611 +0.60295
C +1.63103 −1.55764 +3.23770
H +2.33428 +0.47463 +3.12395
H −0.53802 −0.03201 +3.45019
C +1.81861 −1.81803 +4.73448
H +0.76752 −2.13336 +2.87276
H +2.50951 −1.91961 +2.68728
C +2.09225 −3.30347 +4.95835
C +2.94372 −0.96496 +5.31333
H +0.88755 −1.54561 +5.24623
H +2.17320 −3.53392 +6.02480
H +1.29717 −3.92640 +4.53574
H +3.03521 −3.59558 +4.48083
H +3.14612 −1.24336 +6.35192
H +3.87010 −1.10733 +4.74248
H +2.68777 +0.09842 +5.29832
22
* E = +1.560 kcal/mol ; (13) 177_160_038_183_185
H +0.00000 +0.00000 +0.00000
O +0.00000 +0.00000 +0.96378
C +1.27587 +0.00000 +1.37585
C +1.38806 +0.08022 +2.88111
N +2.70371 −0.38844 +3.26280
H +3.39472 −0.08005 +2.58780
O +2.20392 −0.01118 +0.61782
C +1.04276 +1.52763 +3.29038
H +0.62203 −0.58732 +3.29651
H +2.96727 −0.05839 +4.18109
C +1.18786 +1.80166 +4.79009
H +1.68792 +2.21920 +2.73118
H +0.00632 +1.73518 +2.99005

C +0.71097 +3.21970 +5.09718
C +0.42133 +0.78175 +5.63014
H +2.25338 +1.74365 +5.05474
H +0.86510 +3.47058 +6.15082
H +1.24019 +3.95991 +4.48905
H −0.36012 +3.31399 +4.88330
H +0.45229 +1.04932 +6.69045
H −0.63143 +0.74932 +5.32352
H +0.82955 −0.22937 +5.53050
22
∗ E = +1.589 kcal/mol ; (14) 180_172_312_176_316
H +0.00000 +0.00000 +0.00000
O +0.00000 +0.00000 +0.96380
C +1.27743 +0.00000 +1.37783
C +1.39419 −0.00078 +2.89667
N +2.75582 −0.18639 +3.34825
H +3.18636 −0.96354 +2.85973
O +2.20302 −0.00599 +0.61642
C +0.77990 +1.29412 +3.44676
H +0.78409 −0.84519 +3.24703
H +3.30760 +0.63216 +3.11142
C +0.72165 +1.40404 +4.98151
H +1.34227 +2.14782 +3.04090
H −0.23583 +1.36769 +3.04383
C +0.24378 +0.10443 +5.62544
C +2.04067 +1.85813 +5.60900
H −0.02464 +2.18182 +5.19337
H +0.06674 +0.24623 +6.69561
H −0.68794 −0.25473 +5.17434
H +1.00608 −0.67527 +5.51576
H +1.90280 +2.06782 +6.67465
H +2.80426 +1.08251 +5.51077
H +2.41211 +2.77331 +5.13520
22
∗ E = +1.590 kcal/mol ; (15) 179_047_308_180_183
H +0.00000 +0.00000 +0.00000
O +0.00000 +0.00000 +0.96355
C +1.28157 +0.00000 +1.37687
C +1.38016 +0.01689 +2.89834
N +0.50561 −0.95008 +3.54678

H	−0.44631	−0.86293	+3.21282
O	+2.20043	−0.00355	+0.60878
C	+2.82781	−0.17536	+3.33217
H	+1.04016	+1.01864	+3.19933
H	+0.81535	−1.89558	+3.34964
C	+3.02581	−0.17589	+4.84963
H	+3.20285	−1.11635	+2.90626
H	+3.43586	+0.61817	+2.87978
C	+4.51422	−0.29414	+5.16816
C	+2.43187	+1.07443	+5.49349
H	+2.50408	−1.04816	+5.26305
H	+4.68508	−0.35876	+6.24695
H	+4.95527	−1.18015	+4.70016
H	+5.05595	+0.58361	+4.79569
H	+2.67961	+1.11901	+6.55837
H	+2.83309	+1.97998	+5.02035
H	+1.34136	+1.09010	+5.40571

22

* E = +1.629 kcal/mol ; (16) 177_142_046_059_058

H	+0.00000	+0.00000	+0.00000
O	+0.00000	+0.00000	+0.96451
C	+1.27494	+0.00000	+1.37622
C	+1.41168	+0.06874	+2.88282
N	+2.53410	−0.77776	+3.25352
H	+3.31814	−0.58458	+2.63867
O	+2.20322	−0.00894	+0.61807
C	+1.58606	+1.54654	+3.28426
H	+0.49419	−0.33042	+3.32784
H	+2.82446	−0.58825	+4.20463
C	+0.45474	+2.49809	+2.88575
H	+1.70236	+1.57502	+4.37777
H	+2.53448	+1.89506	+2.85335
C	−0.88940	+2.05177	+3.45961
C	+0.79251	+3.91199	+3.35404
H	+0.37772	+2.50916	+1.78907
H	−1.66490	+2.79567	+3.25320
H	−1.22155	+1.09990	+3.03665
H	−0.81843	+1.93956	+4.54891
H	+0.02200	+4.62532	+3.04667
H	+0.85902	+3.94378	+4.44820

H +1.75171 +4.25009 +2.94990
22
* E = +1.812 kcal/mol ; (17) 353_025_085_078_071
H +0.00000 +0.00000 +0.00000
O +0.00000 +0.00000 +0.97102
C +1.27292 +0.00000 +1.36109
C +2.28222 −0.16199 +0.21360
N +1.66086 +0.30410 −1.02833
H +1.77184 +1.30755 −1.12939
O +1.59240 +0.09225 +2.50970
C +3.62636 +0.48382 +0.56164
H +2.41537 −1.24764 +0.11089
H +2.08772 −0.12847 −1.83672
C +4.51589 −0.34082 +1.50046
H +4.17566 +0.65593 −0.37544
H +3.43628 +1.46774 +1.01004
C +5.06902 −1.57766 +0.79365
C +5.66419 +0.52647 +2.00961
H +3.90520 −0.65265 +2.35534
H +5.69025 −2.16831 +1.47334
H +4.28198 −2.23882 +0.41717
H +5.69555 −1.28188 −0.05716
H +6.32744 −0.04140 +2.66885
H +6.26620 +0.89680 +1.17025
H +5.29120 +1.39165 +2.56557
22
* E = +1.923 kcal/mol ; (18) 355_022_084_296_130
H +0.00000 +0.00000 +0.00000
O +0.00000 +0.00000 +0.97121
C +1.27236 +0.00000 +1.35974
C +2.28789 −0.11995 +0.21096
N +1.65618 +0.31866 −1.03445
H +1.71027 +1.32739 −1.13008
O +1.59770 +0.06351 +2.50907
C +3.58181 +0.59263 +0.59648
H +2.47854 −1.19697 +0.11367
H +2.10733 −0.08886 −1.84251
C +3.39405 +2.11468 +0.77933
H +3.93898 +0.14701 +1.53001
H +4.33980 +0.39266 −0.17114

C	+3.98311	+2.57651	+2.10987
C	+4.00276	+2.89918	−0.38348
H	+2.31319	+2.32927	+0.81249
H	+3.84899	+3.65318	+2.25313
H	+3.50639	+2.04909	+2.94117
H	+5.05857	+2.36505	+2.14053
H	+3.81492	+3.97246	−0.28298
H	+5.08860	+2.75020	−0.40769
H	+3.60942	+2.57384	−1.35335

22

* E = +1.931 kcal/mol ; (19) 179_169_303_172_086

H	+0.00000	+0.00000	+0.00000
O	+0.00000	+0.00000	+0.96385
C	+1.27690	+0.00000	+1.37887
C	+1.39223	+0.02203	+2.89847
N	+2.74047	−0.23355	+3.35577
H	+3.07460	−1.12044	+2.99677
O	+2.20381	−0.01061	+0.61925
C	+0.85500	+1.36204	+3.42038
H	+0.73285	−0.77129	+3.27647
H	+3.36430	+0.47311	+2.97822
C	+0.74148	+1.45082	+4.95058
H	+1.50181	+2.17325	+3.05227
H	−0.13146	+1.52049	+2.97064
C	+2.05009	+1.88592	+5.61001
C	−0.37547	+2.42371	+5.32785
H	+0.47878	+0.45143	+5.32650
H	+1.93322	+1.94808	+6.69672
H	+2.86062	+1.19049	+5.38676
H	+2.33518	+2.88314	+5.25174
H	−0.46741	+2.51759	+6.41421
H	−0.16036	+3.42141	+4.92611
H	−1.34279	+2.10373	+4.92826

22

* E = +1.989 kcal/mol ; (20) 179_041_188_181_183

H	+0.00000	+0.00000	+0.00000
O	+0.00000	+0.00000	+0.96357
C	+1.27996	+0.00000	+1.37530
C	+1.38962	+0.02636	+2.88809
N	+0.39103	−0.86210	+3.46745

H	+0.36806	−0.75930	+4.47429
O	+2.20005	+0.02147	+0.61048
C	+2.79689	−0.37127	+3.31685
H	+1.22340	+1.08838	+3.15343
H	−0.53308	−0.65374	+3.11050
C	+2.99829	−0.39917	+4.83382
H	+3.02035	−1.35935	+2.89773
H	+3.50921	+0.33197	+2.86738
C	+4.45149	−0.74653	+5.14871
C	+2.61714	+0.92997	+5.48524
H	+2.36441	−1.19354	+5.25286
H	+4.61655	−0.83034	+6.22708
H	+4.74392	−1.69273	+4.68341
H	+5.11911	+0.03459	+4.76635
H	+2.84387	+0.91961	+6.55558
H	+3.18337	+1.75292	+5.03200
H	+1.55147	+1.16054	+5.37671

22

∗ E = +1.993 kcal/mol ; (21) 177_020_186_280_054

H	+0.00000	+0.00000	+0.00000
O	+0.00000	+0.00000	+0.96374
C	+1.27960	+0.00000	+1.36862
C	+1.42644	+0.07822	+2.87995
N	+0.20749	−0.33529	+3.55497
H	+0.29079	−0.18849	+4.55299
O	+2.19667	+0.01120	+0.59779
C	+2.63156	−0.73886	+3.35785
H	+1.67166	+1.14458	+3.04201
H	−0.59845	+0.17774	+3.22198
C	+2.39052	−2.24855	+3.46133
H	+3.46308	−0.54294	+2.66964
H	+2.93810	−0.35953	+4.34127
C	+1.86281	−2.83823	+2.15571
C	+3.68857	−2.93569	+3.87944
H	+1.63417	−2.42236	+4.23719
H	+1.77676	−3.92654	+2.23384
H	+0.86769	−2.44690	+1.91799
H	+2.53869	−2.60833	+1.32379
H	+3.53862	−4.01003	+4.02298
H	+4.45516	−2.80525	+3.10646

H +4.08146 −2.51994 +4.81325
22
∗ E = +2.034 kcal/mol ; (22) 009_330_156_051_145
H +0.00000 +0.00000 +0.00000
O +0.00000 +0.00000 +0.97094
C +1.27463 +0.00000 +1.36183
C +2.27702 +0.21185 +0.22672
N +1.72239 −0.32690 −1.01265
H +2.14073 +0.11157 −1.82339
O +1.59930 −0.10937 +2.50733
C +2.54597 +1.71142 +0.03062
H +3.20726 −0.27388 +0.54830
H +1.85946 −1.32696 −1.09132
C +2.92382 +2.48648 +1.30372
H +3.35429 +1.78908 −0.70903
H +1.65550 +2.16526 −0.42879
C +3.93766 +3.58036 +0.97338
C +1.68948 +3.10379 +1.96321
H +3.37925 +1.78198 +2.01228
H +4.19365 +4.16142 +1.86424
H +4.86179 +3.16239 +0.56180
H +3.52280 +4.27417 +0.23209
H +1.95663 +3.59534 +2.90319
H +1.25410 +3.86174 +1.30041
H +0.91725 +2.36320 +2.18663
22
∗ E = +2.046 kcal/mol ; (23) 357_013_100_148_288
H +0.00000 +0.00000 +0.00000
O +0.00000 +0.00000 +0.97290
C +1.27310 +0.00000 +1.35103
C +2.28327 −0.08253 +0.19003
N +1.59395 +0.16061 −1.08074
H +1.72873 +1.11650 −1.38879
O +1.61027 +0.02370 +2.49842
C +3.49236 +0.81937 +0.46396
H +2.60634 −1.13281 +0.19814
H +1.92842 −0.44264 −1.81843
C +4.82301 +0.31886 −0.11544
H +3.26479 +1.81829 +0.06483
H +3.59616 +0.92720 +1.54830

C	+5.33998	−0.89518	+0.65510
C	+4.74738	+0.00592	−1.60927
H	+5.54574	+1.13389	+0.02148
H	+6.31839	−1.20863	+0.27855
H	+5.43563	−0.67866	+1.72280
H	+4.66391	−1.75179	+0.54945
H	+5.74232	−0.20716	−2.01124
H	+4.13389	−0.88571	−1.79060
H	+4.32154	+0.83670	−2.18233

22

∗ E = +2.073 kcal/mol ; (24) 179_171_296_294_144

H	+0.00000	+0.00000	+0.00000
O	+0.00000	+0.00000	+0.96392
C	+1.27688	+0.00000	+1.37862
C	+1.39275	+0.02092	+2.89927
N	+2.75341	−0.18334	+3.35011
H	+3.07641	−1.10550	+3.08154
O	+2.20296	−0.00250	+0.61742
C	+0.80622	+1.33958	+3.42700
H	+0.76777	−0.79907	+3.27355
H	+3.36854	+0.46960	+2.87387
C	+1.60993	+2.58925	+3.01787
H	−0.22304	+1.41961	+3.06048
H	+0.76002	+1.27347	+4.52005
C	+0.67019	+3.76528	+2.75611
C	+2.63420	+2.97307	+4.08824
H	+2.14686	+2.37025	+2.08091
H	+1.22851	+4.66878	+2.49218
H	−0.03076	+3.54506	+1.94513
H	+0.08360	+3.98657	+3.65583
H	+3.25972	+3.80649	+3.75374
H	+2.11167	+3.29300	+4.99785
H	+3.27989	+2.13520	+4.36199

22

∗ E = +2.126 kcal/mol ; (25) 185_094_302_080_181

H	+0.00000	+0.00000	+0.00000
O	+0.00000	+0.00000	+0.96429
C	+1.27866	+0.00000	+1.37748
C	+1.39768	−0.11133	+2.89263
N	+1.57476	−1.51959	+3.24786

H	+0.80160	−2.08396	+2.91496
O	+2.20195	+0.00398	+0.61236
C	+2.56912	+0.71143	+3.41563
H	+0.45866	+0.23584	+3.34009
H	+2.42421	−1.87780	+2.82349
C	+2.27658	+2.20726	+3.56131
H	+2.84756	+0.29718	+4.39075
H	+3.42489	+0.56824	+2.74033
C	+3.52393	+2.92737	+4.06688
C	+1.79203	+2.83659	+2.25543
H	+1.48132	+2.31983	+4.31303
H	+3.32663	+3.98948	+4.24036
H	+3.88731	+2.49015	+5.00209
H	+4.32925	+2.85326	+3.32667
H	+1.67274	+3.91832	+2.36994
H	+2.51325	+2.65530	+1.45000
H	+0.82227	+2.43679	+1.93780

22

* E = +2.169 kcal/mol ; (26) 181_309_065_052_056

H	+0.00000	+0.00000	+0.00000
O	+0.00000	+0.00000	+0.96363
C	+1.27789	+0.00000	+1.38071
C	+1.36600	−0.01659	+2.89283
N	+0.52932	+1.05031	+3.42744
H	−0.44529	+0.87321	+3.20997
O	+2.20508	−0.03578	+0.62418
C	+1.00136	−1.42491	+3.39350
H	+2.40860	+0.20826	+3.13411
H	+0.61767	+1.09239	+4.43535
C	+1.78187	−2.57209	+2.74557
H	+1.16734	−1.44432	+4.48043
H	−0.07513	−1.57343	+3.23206
C	+3.29096	−2.38381	+2.89692
C	+1.33927	−3.90122	+3.35243
H	+1.54430	−2.58672	+1.67138
H	+3.82895	−3.26251	+2.52882
H	+3.65134	−1.51576	+2.33778
H	+3.55380	−2.25126	+3.95422
H	+1.84345	−4.74378	+2.86999
H	+1.58646	−3.93186	+4.42040

H +0.25903 −4.04807 +3.25416
22
* E = +2.340 kcal/mol ; (27) 178_145_296_055_147
H +0.00000 +0.00000 +0.00000
O +0.00000 +0.00000 +0.96454
C +1.27584 +0.00000 +1.38069
C +1.40304 +0.05697 +2.89589
N +2.54143 −0.70052 +3.37971
H +2.43546 −1.68753 +3.17796
O +2.20427 −0.02675 +0.62200
C +1.54158 +1.51518 +3.35812
H +0.48339 −0.35840 +3.32369
H +3.37875 −0.39162 +2.89584
C +0.42588 +2.46911 +2.90270
H +1.59309 +1.47510 +4.45130
H +2.51513 +1.89259 +3.01212
C +0.18192 +3.53848 +3.96646
C +0.76438 +3.14193 +1.57062
H −0.49777 +1.88671 +2.77688
H −0.58703 +4.24833 +3.64628
H −0.13470 +3.09483 +4.91519
H +1.10225 +4.10504 +4.15200
H −0.07339 +3.74843 +1.21335
H +1.62649 +3.80674 +1.70172
H +1.02315 +2.42775 +0.78412
22
* E = +2.497 kcal/mol ; (28) 180_302_300_058_282
H +0.00000 +0.00000 +0.00000
O +0.00000 +0.00000 +0.96355
C +1.27905 +0.00000 +1.38084
C +1.37654 +0.00834 +2.89747
N +0.69436 +1.15255 +3.48807
H +1.08065 +2.02817 +3.15697
O +2.20463 +0.00701 +0.61813
C +0.79978 −1.27288 +3.50728
H +2.44322 +0.08463 +3.12891
H −0.29054 +1.13785 +3.24714
C +1.42800 −2.59920 +3.05090
H +0.92277 −1.16173 +4.59041
H −0.28104 −1.29008 +3.31025

C	+0.95090	−3.05708	+1.66983
C	+2.95527	−2.55048	+3.09905
H	+1.08874	−3.35139	+3.77512
H	+1.25701	−4.09191	+1.48774
H	−0.13925	−3.00502	+1.58477
H	+1.38806	−2.45287	+0.86831
H	+3.38077	−3.54272	+2.92241
H	+3.34712	−1.88226	+2.32298
H	+3.31643	−2.19505	+4.07020

22

∗ E = +2.563 kcal/mol ; (29) 176_287_304_072_175

H	+0.00000	+0.00000	+0.00000
O	+0.00000	+0.00000	+0.96369
C	+1.28134	+0.00000	+1.37484
C	+1.39946	+0.10800	+2.88816
N	+1.05798	+1.46665	+3.30744
H	+1.63229	+2.15422	+2.83410
O	+2.20066	−0.00839	+0.60484
C	+0.52283	−0.88602	+3.64648
H	+2.45520	−0.06851	+3.12174
H	+0.08819	+1.66734	+3.08750
C	+0.98260	−2.34539	+3.59921
H	+0.50395	−0.54561	+4.68811
H	−0.50793	−0.81639	+3.26952
C	+0.07671	−3.18840	+4.49430
C	+0.99619	−2.91493	+2.18060
H	+2.00549	−2.38915	+4.00070
H	+0.40738	−4.23084	+4.52316
H	+0.05764	−2.80694	+5.51984
H	−0.95111	−3.17505	+4.11231
H	+1.21439	−3.98709	+2.19931
H	+0.01602	−2.77997	+1.70699
H	+1.75096	−2.44212	+1.54516

22

∗ E = +2.579 kcal/mol ; (30) 180_127_296_062_284

H	+0.00000	+0.00000	+0.00000
O	+0.00000	+0.00000	+0.96475
C	+1.27783	+0.00000	+1.37839
C	+1.42254	−0.00507	+2.89366
N	+2.28699	−1.09418	+3.32790

H	+1.88535	−1.99602	+3.10079
O	+2.20393	−0.01380	+0.61675
C	+2.03667	+1.31385	+3.37547
H	+0.43262	−0.14528	+3.33896
H	+3.17628	−1.03027	+2.84216
C	+1.24079	+2.59948	+3.10757
H	+2.18280	+1.19121	+4.45428
H	+3.03499	+1.39818	+2.92399
C	+1.31482	+3.07226	+1.65363
C	−0.21462	+2.47192	+3.55711
H	+1.71817	+3.37335	+3.72307
H	+0.92761	+4.09211	+1.56551
H	+2.34249	+3.06189	+1.27794
H	+0.70810	+2.44535	+0.99289
H	−0.72374	+3.43897	+3.50653
H	−0.76301	+1.77916	+2.90781
H	−0.28737	+2.10381	+4.58615

22

* E = +2.583 kcal/mol ; (31) 010_326_160_058_277

H	+0.00000	+0.00000	+0.00000
O	+0.00000	+0.00000	+0.97061
C	+1.27379	+0.00000	+1.36295
C	+2.27198	+0.24544	+0.22980
N	+1.74354	−0.37297	−0.98696
H	+2.17234	+0.02304	−1.81399
O	+1.59431	−0.15266	+2.50522
C	+2.44207	+1.74662	−0.05690
H	+3.22845	−0.17682	+0.56132
H	+1.89732	−1.37384	−1.00292
C	+2.91224	+2.65611	+1.09008
H	+3.17451	+1.80884	−0.87408
H	+1.49140	+2.12310	−0.45965
C	+1.77263	+3.08686	+2.01602
C	+4.06627	+2.03898	+1.88018
H	+3.28814	+3.56511	+0.60133
H	+2.13018	+3.83699	+2.72826
H	+0.94892	+3.53098	+1.44774
H	+1.37803	+2.24964	+2.59722
H	+4.48248	+2.76833	+2.58119
H	+3.72194	+1.17653	+2.46013

H	+4.87572	+1.71076	+1.21802

22

∗ E = +2.642 kcal/mol ; (32) 184_098_301_079_305

H	+0.00000	+0.00000	+0.00000
O	+0.00000	+0.00000	+0.96434
C	+1.27834	+0.00000	+1.37740
C	+1.40183	−0.10259	+2.89290
N	+1.65438	−1.49829	+3.25148
H	+0.90973	−2.10376	+2.92605
O	+2.20171	−0.00032	+0.61201
C	+2.54001	+0.77321	+3.41103
H	+0.44518	+0.19100	+3.33794
H	+2.51827	−1.81247	+2.82126
C	+2.20780	+2.26731	+3.52850
H	+2.81990	+0.38293	+4.39570
H	+3.39964	+0.63271	+2.74308
C	+1.69273	+2.85592	+2.21524
C	+1.21526	+2.53725	+4.65917
H	+3.14778	+2.77525	+3.78063
H	+1.57876	+3.94106	+2.29605
H	+2.36770	+2.63967	+1.38146
H	+0.70312	+2.44981	+1.96674
H	+1.05160	+3.61203	+4.78420
H	+0.23937	+2.08511	+4.44862
H	+1.57353	+2.13433	+5.61147

22

∗ E = +2.660 kcal/mol ; (33) 354_024_086_104_174

H	+0.00000	+0.00000	+0.00000
O	+0.00000	+0.00000	+0.97099
C	+1.27281	+0.00000	+1.36091
C	+2.28385	−0.15599	+0.21420
N	+1.65945	+0.29654	−1.03144
H	+1.76115	+1.29962	−1.14176
O	+1.59518	+0.07723	+2.50943
C	+3.64674	+0.46630	+0.56420
H	+2.41011	−1.24484	+0.11657
H	+2.08639	−0.14041	−1.83733
C	+4.72892	−0.55847	+0.92232
H	+3.98738	+1.05083	−0.29949
H	+3.52893	+1.16056	+1.40575

C	+6.06878	+0.14726	+1.11234
C	+4.36495	−1.35400	+2.17392
H	+4.82035	−1.25724	+0.07575
H	+6.86411	−0.56805	+1.34177
H	+6.36098	+0.70726	+0.21816
H	+6.00683	+0.85480	+1.94736
H	+5.15101	−2.07727	+2.41180
H	+4.23996	−0.67983	+3.02777
H	+3.42322	−1.90035	+2.06435

22

∗ E = +2.715 kcal/mol ; (34) 182_321_062_181_182

H	+0.00000	+0.00000	+0.00000
O	+0.00000	+0.00000	+0.96354
C	+1.27844	+0.00000	+1.37652
C	+1.38956	−0.05618	+2.88509
N	+0.36349	+0.77296	+3.49665
H	−0.56201	+0.43043	+3.26387
O	+2.20322	−0.02256	+0.61630
C	+1.37206	−1.54485	+3.28567
H	+2.36928	+0.36897	+3.12316
H	+0.45604	+0.76705	+4.50480
C	+1.46041	−1.78384	+4.79478
H	+0.45078	−2.00248	+2.89838
H	+2.21474	−2.04935	+2.79189
C	+1.48970	−3.28480	+5.07496
C	+2.68356	−1.10158	+5.40497
H	+0.55658	−1.36963	+5.26383
H	+1.49239	−3.48787	+6.15003
H	+0.62487	−3.79058	+4.63445
H	+2.39373	−3.73365	+4.64710
H	+2.78596	−1.35930	+6.46322
H	+3.59662	−1.42566	+4.89090
H	+2.63316	−0.01002	+5.33420

22

∗ E = +2.817 kcal/mol ; (35) 181_055_181_075_073

H	+0.00000	+0.00000	+0.00000
O	+0.00000	+0.00000	+0.96365
C	+1.27932	+0.00000	+1.37898
C	+1.36132	−0.02761	+2.89639
N	+0.59568	−1.18464	+3.35332

H	+0.60741	−1.23333	+4.36423
O	+2.20194	+0.02814	+0.61795
C	+2.80943	−0.12465	+3.35669
H	+0.92733	+0.93123	+3.23309
H	−0.36915	−1.12542	+3.05145
C	+3.60462	+1.17793	+3.21947
H	+2.81694	−0.43380	+4.41201
H	+3.29374	−0.92548	+2.78721
C	+3.18343	+2.20477	+4.27070
C	+5.09783	+0.88639	+3.34313
H	+3.41617	+1.58950	+2.21936
H	+3.73750	+3.14035	+4.14808
H	+2.11692	+2.44533	+4.22143
H	+3.39464	+1.82303	+5.27721
H	+5.68989	+1.80344	+3.26622
H	+5.31697	+0.42862	+4.31574
H	+5.43173	+0.19636	+2.56291

22

∗ E = +2.824 kcal/mol ; (36) 002_351_136_301_301

H	+0.00000	+0.00000	+0.00000
O	+0.00000	+0.00000	+0.97316
C	+1.27624	+0.00000	+1.34931
C	+2.28723	+0.04433	+0.19553
N	+1.60965	−0.14368	−1.09060
H	+1.94483	+0.50068	−1.79342
O	+1.61987	−0.02533	+2.49459
C	+3.13428	+1.32536	+0.27283
H	+2.97227	−0.78804	+0.39862
H	+1.73053	−1.08305	−1.44419
C	+2.43767	+2.69702	+0.21890
H	+3.69531	+1.26913	+1.21219
H	+3.87017	+1.26849	−0.54083
C	+1.61508	+2.93165	−1.04867
C	+1.59031	+2.99979	+1.45672
H	+3.26493	+3.42024	+0.20851
H	+1.28189	+3.97260	−1.09680
H	+2.19344	+2.72994	−1.95871
H	+0.71808	+2.30260	−1.06017
H	+1.32346	+4.06097	+1.47480
H	+0.65373	+2.43324	+1.45857

H +2.12353 +2.75954 +2.38076
22
∗ E = +2.921 kcal/mol ; (37) 184_190_199_287_278
H +0.00000 +0.00000 +0.00000
O +0.00000 +0.00000 +0.96371
C +1.27893 +0.00000 +1.36819
C +1.41567 −0.10024 +2.87613
N +2.80339 +0.14246 +3.21742
H +3.02206 −0.21231 +4.13874
O +2.19806 −0.00159 +0.59857
C +0.44383 +0.79642 +3.65802
H +1.09552 −1.14222 +3.07262
H +3.41999 −0.28619 +2.53751
C +0.72106 +2.31192 +3.65513
H −0.55966 +0.60561 +3.26258
H +0.43538 +0.44450 +4.69787
C +1.77603 +2.72928 +4.68220
C +1.09065 +2.84003 +2.27008
H −0.22501 +2.78388 +3.95439
H +1.82326 +3.82070 +4.75709
H +1.53827 +2.33720 +5.67720
H +2.76369 +2.36314 +4.39196
H +1.19048 +3.92950 +2.29132
H +2.05338 +2.42743 +1.94713
H +0.33057 +2.58864 +1.52303
22
∗ E = +3.044 kcal/mol ; (38) 182_345_300_312_179
H +0.00000 +0.00000 +0.00000
O +0.00000 +0.00000 +0.96432
C +1.28180 +0.00000 +1.36721
C +1.44731 −0.03900 +2.88276
N +0.27260 +0.26599 +3.67627
H −0.06667 +1.19916 +3.47979
O +2.19743 +0.00391 +0.59332
C +2.07357 −1.39620 +3.25783
H +2.19912 +0.72852 +3.09922
H −0.48634 −0.37358 +3.48302
C +1.44709 −2.64833 +2.63165
H +3.12978 −1.36977 +2.96909
H +2.03051 −1.47165 +4.35163

C +2.22232 −3.87550 +3.10891
C −0.04035 −2.81158 +2.95077
H +1.56257 −2.57773 +1.53980
H +1.84843 −4.78836 +2.63588
H +3.28956 −3.78676 +2.88437
H +2.11575 −3.99316 +4.19389
H −0.39101 −3.80203 +2.64421
H −0.21741 −2.71412 +4.02893
H −0.65764 −2.07664 +2.42492
22
∗ E = +3.044 kcal/mol ; (39) 182_346_308_172_316
H +0.00000 +0.00000 +0.00000
O +0.00000 +0.00000 +0.96407
C +1.28195 +0.00000 +1.36668
C +1.44035 −0.03778 +2.88360
N +0.24754 +0.24894 +3.65343
H −0.18335 +1.11566 +3.35556
O +2.19777 +0.00890 +0.59348
C +2.03001 −1.41065 +3.23899
H +2.19632 +0.72586 +3.10624
H −0.44175 −0.48344 +3.52187
C +2.44523 −1.60037 +4.70903
H +1.30206 −2.18777 +2.96247
H +2.90302 −1.55360 +2.59281
C +3.18905 −0.38199 +5.25144
C +1.27770 −1.96711 +5.62690
H +3.14100 −2.45039 +4.71103
H +3.60429 −0.59261 +6.24135
H +4.01443 −0.08440 +4.59539
H +2.50340 +0.46694 +5.35341
H +1.64526 −2.23560 +6.62262
H +0.58941 −1.12495 +5.73383
H +0.71901 −2.82457 +5.23615
22
∗ E = +3.051 kcal/mol ; (40) 357_015_091_284_274
H +0.00000 +0.00000 +0.00000
O +0.00000 +0.00000 +0.97195
C +1.27293 +0.00000 +1.35639
C +2.28816 −0.06161 +0.20034
N +1.61750 +0.24081 −1.06457

H	+1.65274	+1.23001	−1.27212
O	+1.60078	−0.00083	+2.50682
C	+3.56359	+0.72132	+0.53446
H	+2.56006	−1.12518	+0.16738
H	+2.04042	−0.24958	−1.84066
C	+3.49707	+2.26058	+0.44733
H	+3.83029	+0.43568	+1.55734
H	+4.37111	+0.36629	−0.11738
C	+3.76960	+2.80991	−0.95749
C	+2.20690	+2.84267	+1.02586
H	+4.32180	+2.62090	+1.07562
H	+3.91702	+3.89372	−0.91719
H	+4.67072	+2.36450	−1.39068
H	+2.94609	+2.63408	−1.65791
H	+2.23562	+3.93600	+0.99657
H	+1.32155	+2.53303	+0.45383
H	+2.05163	+2.52840	+2.06112

22

* E = +3.107 kcal/mol ; (41) 183_180_301_287_279

H	+0.00000	+0.00000	+0.00000
O	+0.00000	+0.00000	+0.96391
C	+1.27954	+0.00000	+1.37341
C	+1.40903	−0.07293	+2.89357
N	+2.78940	−0.06443	+3.33150
H	+3.28242	−0.85253	+2.92727
O	+2.19951	+0.00605	+0.60522
C	+0.53440	+0.95950	+3.62386
H	+0.98417	−1.05494	+3.14711
H	+3.26010	+0.75933	+2.97275
C	+0.96672	+2.43720	+3.57119
H	−0.47688	+0.87227	+3.21241
H	+0.48226	+0.64795	+4.67281
C	+2.05513	+2.78883	+4.58800
C	+1.35946	+2.89289	+2.16623
H	+0.07491	+3.00954	+3.86094
H	+2.22271	+3.87066	+4.60405
H	+1.76644	+2.47583	+5.59598
H	+3.00955	+2.30704	+4.36242
H	+1.53083	+3.97322	+2.14696
H	+2.28567	+2.41376	+1.82671

H +0.57736 +2.66396 +1.43427
22
* E = +3.139 kcal/mol ; (42) 178_040_304_292_137
H +0.00000 +0.00000 +0.00000
O +0.00000 +0.00000 +0.96371
C +1.28038 +0.00000 +1.37500
C +1.39519 +0.04404 +2.89948
N +0.41763 −0.78434 +3.59015
H −0.52383 −0.53465 +3.31294
O +2.19857 −0.01293 +0.60434
C +2.82240 −0.30004 +3.30951
H +1.18844 +1.08872 +3.17194
H +0.54750 −1.76119 +3.35049
C +3.20505 −1.76777 +3.02616
H +3.49794 +0.36845 +2.76680
H +2.93095 −0.08634 +4.37862
C +4.60530 −1.85021 +2.42424
C +3.10919 −2.62267 +4.29077
H +2.50623 −2.17137 +2.27656
H +4.89745 −2.88750 +2.23246
H +4.65114 −1.29376 +1.48326
H +5.34002 −1.41805 +3.11422
H +3.29644 −3.67872 +4.07229
H +3.86162 −2.29456 +5.01777
H +2.13112 −2.53275 +4.77318
22
* E = +3.163 kcal/mol ; (43) 177_142_045_058_146
H +0.00000 +0.00000 +0.00000
O +0.00000 +0.00000 +0.96449
C +1.27379 +0.00000 +1.37750
C +1.40521 +0.06370 +2.88438
N +2.51933 −0.79220 +3.25297
H +3.30146 −0.61422 +2.63126
O +2.20385 −0.01189 +0.62090
C +1.56590 +1.53171 +3.32990
H +0.47752 −0.33134 +3.31550
H +2.81878 −0.60212 +4.20109
C +0.42226 +2.47602 +2.92384
H +1.66053 +1.50979 +4.42339
H +2.52173 +1.90635 +2.93697

C +0.19472 +3.53595 +4.00035
C +0.70962 +3.15735 +1.58433
H −0.49578 +1.87941 +2.82359
H −0.59657 +4.23367 +3.70939
H −0.08427 +3.08274 +4.95656
H +1.11028 +4.11823 +4.15900
H −0.14955 +3.74728 +1.25125
H +1.56229 +3.83833 +1.69141
H +0.95935 +2.44714 +0.79136
22
∗ E = +3.180 kcal/mol ; (44) 177_020_309_144_058
H +0.00000 +0.00000 +0.00000
O +0.00000 +0.00000 +0.96382
C +1.28238 +0.00000 +1.36736
C +1.42286 +0.07768 +2.88958
N +0.24448 −0.32486 +3.63649
H −0.58387 +0.14988 +3.29932
O +2.19338 −0.01372 +0.58908
C +2.66765 −0.68599 +3.35357
H +1.59178 +1.15022 +3.08242
H +0.08333 −1.31974 +3.52932
C +3.39769 −0.05009 +4.54007
H +2.36550 −1.70988 +3.62176
H +3.36301 −0.76709 +2.51250
C +2.50588 +0.11446 +5.76806
C +4.63001 −0.88325 +4.88452
H +3.73411 +0.94706 +4.21841
H +3.07459 +0.54322 +6.59948
H +1.64153 +0.75206 +5.56872
H +2.11727 −0.85981 +6.08813
H +5.20254 −0.42543 +5.69672
H +4.32945 −1.88521 +5.21391
H +5.29278 −0.99861 +4.02119
22
∗ E = +3.195 kcal/mol ; (45) 184_104_064_078_178
H +0.00000 +0.00000 +0.00000
O +0.00000 +0.00000 +0.96432
C +1.27251 +0.00000 +1.37504
C +1.42254 −0.09623 +2.88553
N +1.82301 −1.48576 +3.10898

H	+2.72797	−1.64824	+2.67880
O	+2.20258	+0.02587	+0.61856
C	+2.41992	+0.93987	+3.41196
H	+0.43652	+0.05451	+3.34045
H	+1.91457	−1.67159	+4.10019
C	+1.88672	+2.37070	+3.52229
H	+2.74771	+0.60521	+4.40472
H	+3.30593	+0.92359	+2.76041
C	+2.97159	+3.27859	+4.09709
C	+1.39890	+2.91236	+2.17873
H	+1.03608	+2.36059	+4.22001
H	+2.60161	+4.29835	+4.23940
H	+3.33500	+2.90989	+5.06152
H	+3.82659	+3.32575	+3.41246
H	+1.13719	+3.97159	+2.26222
H	+2.18309	+2.81494	+1.41832
H	+0.50932	+2.38610	+1.81844

22

∗ E = +3.243 kcal/mol ; (46) 179_002_055_281_058

H	+0.00000	+0.00000	+0.00000
O	+0.00000	+0.00000	+0.96406
C	+1.27879	+0.00000	+1.36164
C	+1.46319	+0.01580	+2.87109
N	+0.19117	−0.02140	+3.57100
H	−0.37180	−0.81045	+3.27589
O	+2.19355	−0.00206	+0.58595
C	+2.50332	−1.04204	+3.29003
H	+1.91730	+0.99916	+3.05810
H	+0.34104	−0.10131	+4.56909
C	+1.98700	−2.48140	+3.35108
H	+3.35031	−0.98425	+2.59417
H	+2.88488	−0.76288	+4.28060
C	+1.41642	−2.94889	+2.01246
C	+3.11709	−3.40651	+3.79836
H	+1.18729	−2.53010	+4.10416
H	+1.15847	−4.01158	+2.05273
H	+0.50727	−2.40530	+1.73317
H	+2.15124	−2.80610	+1.21132
H	+2.76591	−4.43695	+3.90834
H	+3.92452	−3.40506	+3.05693

H +3.54157 −3.08701 +4.75543
22
∗ E = +3.259 kcal/mol ; (47) 179_040_186_295_137
H +0.00000 +0.00000 +0.00000
O +0.00000 +0.00000 +0.96367
C +1.27817 +0.00000 +1.37664
C +1.39119 +0.03582 +2.89197
N +0.37095 −0.82066 +3.48176
H +0.37379 −0.72324 +4.48951
O +2.20055 +0.01810 +0.61308
C +2.79300 −0.39279 +3.31177
H +1.25778 +1.10390 +3.14869
H −0.55245 −0.58085 +3.14330
C +3.12190 −1.85892 +2.96777
H +3.50397 +0.27791 +2.81804
H +2.89937 −0.22775 +4.39243
C +4.53424 −1.96657 +2.39886
C +2.95221 −2.77020 +4.18183
H +2.41567 −2.19363 +2.19616
H +4.78759 −3.00333 +2.15585
H +4.62956 −1.36294 +1.49096
H +5.27040 −1.60584 +3.12781
H +3.16549 −3.81279 +3.92523
H +3.64558 −2.47528 +4.97921
H +1.93245 −2.71851 +4.57310
22
∗ E = +3.293 kcal/mol ; (48) 007_332_161_104_173
H +0.00000 +0.00000 +0.00000
O +0.00000 +0.00000 +0.97084
C +1.27297 +0.00000 +1.36691
C +2.27947 +0.17427 +0.22431
N +1.68695 −0.35455 −1.00455
H +2.15824 +0.00464 −1.82433
O +1.58763 −0.08268 +2.51691
C +2.59320 +1.67834 +0.05028
H +3.19546 −0.35083 +0.52735
H +1.73155 −1.36627 −1.03830
C +3.94778 +2.11277 +0.61608
H +2.56758 +1.90942 −1.02278
H +1.80083 +2.28726 +0.50734

C	+4.21886	+3.56582	+0.23364
C	+4.02086	+1.93579	+2.13029
H	+4.71891	+1.47986	+0.15095
H	+5.19412	+3.89440	+0.60429
H	+4.19983	+3.71080	−0.85133
H	+3.45866	+4.22164	+0.67415
H	+4.99136	+2.27073	+2.50882
H	+3.24198	+2.53345	+2.61895
H	+3.86564	+0.89897	+2.43820

22

∗ E = +3.352 kcal/mol ; (49) 183_332_301_171_087

H	+0.00000	+0.00000	+0.00000
O	+0.00000	+0.00000	+0.96389
C	+1.28112	+0.00000	+1.37127
C	+1.42000	−0.07300	+2.88721
N	+0.31735	+0.48506	+3.64406
H	+0.17293	+1.46174	+3.41942
O	+2.20067	+0.01500	+0.60306
C	+1.66302	−1.54592	+3.24690
H	+2.33203	+0.48684	+3.12535
H	−0.54391	−0.00297	+3.42278
C	+2.04604	−1.78685	+4.71597
H	+0.76225	−2.12825	+2.99879
H	+2.46309	−1.91086	+2.59206
C	+0.82320	−1.95832	+5.61697
C	+2.94196	−3.02089	+4.81985
H	+2.61235	−0.91077	+5.06305
H	+1.12822	−2.13673	+6.65310
H	+0.18172	−1.07595	+5.59278
H	+0.23760	−2.82726	+5.29146
H	+3.21731	−3.22259	+5.85955
H	+2.41571	−3.90453	+4.43870
H	+3.86136	−2.90249	+4.23834

22

∗ E = +3.398 kcal/mol ; (50) 182_061_181_091_178

H	+0.00000	+0.00000	+0.00000
O	+0.00000	+0.00000	+0.96366
C	+1.28050	+0.00000	+1.37871
C	+1.35246	−0.05980	+2.89739
N	+0.70422	−1.31254	+3.28572

H	+0.70692	−1.40851	+4.29330
O	+2.20162	+0.03184	+0.61745
C	+2.79135	−0.01248	+3.40336
H	+0.80909	+0.83174	+3.26338
H	−0.25831	−1.33395	+2.97076
C	+3.30722	+1.39825	+3.70205
H	+2.84638	−0.60945	+4.32219
H	+3.44491	−0.50044	+2.66990
C	+4.73123	+1.32114	+4.24661
C	+3.25738	+2.31112	+2.47803
H	+2.66060	+1.83124	+4.48083
H	+5.10784	+2.31267	+4.51531
H	+4.78848	+0.68233	+5.13373
H	+5.40310	+0.90262	+3.48832
H	+3.66408	+3.29819	+2.71918
H	+3.84354	+1.88441	+1.65735
H	+2.23625	+2.45627	+2.10985

22

∗ E = +3.425 kcal/mol ; (51) 179_042_181_142_056

H	+0.00000	+0.00000	+0.00000
O	+0.00000	+0.00000	+0.96345
C	+1.27928	+0.00000	+1.37471
C	+1.37709	+0.03465	+2.89248
N	+0.36790	−0.86103	+3.44673
H	+0.40188	−0.85117	+4.45746
O	+2.19737	+0.02459	+0.60731
C	+2.77957	−0.33625	+3.37609
H	+1.19395	+1.09918	+3.14560
H	−0.56428	−0.59553	+3.15462
C	+3.25780	+0.46424	+4.59134
H	+2.77865	−1.40835	+3.61620
H	+3.48687	−0.19254	+2.55350
C	+2.29321	+0.36807	+5.77232
C	+4.64926	−0.00847	+5.00334
H	+3.32450	+1.52019	+4.29058
H	+2.68723	+0.89915	+6.64416
H	+1.31463	+0.80360	+5.54144
H	+2.14649	−0.68063	+6.06260
H	+5.04123	+0.58345	+5.83595
H	+4.61389	−1.05612	+5.32508

H +5.35628 +0.06101 +4.17116
22
* E = +3.536 kcal/mol ; (52) 180_125_055_064_283
H +0.00000 +0.00000 +0.00000
O +0.00000 +0.00000 +0.96471
C +1.27457 +0.00000 +1.37610
C +1.42468 −0.00612 +2.88485
N +2.27320 −1.15413 +3.18842
H +3.13620 −1.07972 +2.65891
O +2.20416 +0.00745 +0.61941
C +2.01296 +1.32894 +3.37343
H +0.43885 −0.17263 +3.32972
H +2.51252 −1.16442 +4.17251
C +1.17916 +2.59885 +3.14181
H +2.17945 +1.20863 +4.45230
H +3.00133 +1.44339 +2.90804
C +1.22765 +3.09357 +1.69443
C −0.26846 +2.42276 +3.60100
H +1.64013 +3.37650 +3.76527
H +0.79658 +4.09681 +1.62089
H +2.25366 +3.13268 +1.31626
H +0.64666 +2.44877 +1.02805
H −0.80299 +3.37694 +3.57474
H −0.80425 +1.72878 +2.94293
H −0.32325 +2.03288 +4.62332
22
* E = +3.686 kcal/mol ; (53) 179_303_067_058_281
H +0.00000 +0.00000 +0.00000
O +0.00000 +0.00000 +0.96361
C +1.27714 +0.00000 +1.38227
C +1.35853 +0.01784 +2.89465
N +0.63412 +1.19899 +3.35519
H −0.35590 +1.09904 +3.15853
O +2.20599 −0.03078 +0.62735
C +0.84104 −1.29736 +3.50338
H +2.41737 +0.15166 +3.13320
H +0.74021 +1.30663 +4.35653
C +1.53982 −2.59621 +3.06734
H +0.94488 −1.18533 +4.59086
H −0.23506 −1.36773 +3.29401

C	+1.08216	−3.09123	+1.69282
C	+3.06261	−2.46514	+3.11150
H	+1.24234	−3.35765	+3.80050
H	+1.45515	−4.10403	+1.51162
H	−0.00989	−3.11392	+1.61951
H	+1.46888	−2.46165	+0.88548
H	+3.53968	−3.43546	+2.94536
H	+3.41726	−1.78526	+2.32814
H	+3.40631	−2.08171	+4.07862

22

∗ E = +3.709 kcal/mol ; (54) 184_107_064_076_301

H	+0.00000	+0.00000	+0.00000
O	+0.00000	+0.00000	+0.96439
C	+1.27254	+0.00000	+1.37527
C	+1.42382	−0.08544	+2.88572
N	+1.88708	−1.45461	+3.11604
H	+2.79687	−1.57778	+2.68273
O	+2.20271	+0.02168	+0.61863
C	+2.38735	+0.98852	+3.40658
H	+0.43240	+0.01718	+3.33794
H	+1.99218	−1.62788	+4.10823
C	+1.84138	+2.42161	+3.46676
H	+2.69239	+0.68449	+4.41648
H	+3.28540	+0.95522	+2.77541
C	+1.38479	+2.93203	+2.10004
C	+0.71490	+2.55738	+4.49136
H	+2.67520	+3.05340	+3.80058
H	+1.15749	+4.00136	+2.14337
H	+2.14913	+2.77262	+1.33283
H	+0.46773	+2.42352	+1.77858
H	+0.40890	+3.60270	+4.59632
H	−0.17187	+1.99166	+4.18420
H	+1.02274	+2.19490	+5.47759

22

∗ E = +3.741 kcal/mol ; (55) 177_018_185_284_279

H	+0.00000	+0.00000	+0.00000
O	+0.00000	+0.00000	+0.96379
C	+1.27924	+0.00000	+1.36803
C	+1.42978	+0.07977	+2.87913
N	+0.19457	−0.27590	+3.55671

H	+0.29115	−0.13633	+4.55486
O	+2.19634	+0.01182	+0.59666
C	+2.62205	−0.75758	+3.35765
H	+1.71410	+1.13857	+3.03054
H	−0.58387	+0.28213	+3.22938
C	+2.43826	−2.28660	+3.39763
H	+3.45818	−0.50789	+2.69572
H	+2.90005	−0.40880	+4.36069
C	+1.69840	−2.77591	+4.64547
C	+1.76980	−2.83388	+2.13720
H	+3.45526	−2.69941	+3.44481
H	+1.77725	−3.86459	+4.73105
H	+2.12089	−2.33997	+5.55775
H	+0.63726	−2.51933	+4.59529
H	+1.71662	−3.92618	+2.17892
H	+0.74123	−2.45942	+2.06040
H	+2.31604	−2.55287	+1.23196

22

∗ E = +3.834 kcal/mol ; (56) 178_164_021_309_309

H	+0.00000	+0.00000	+0.00000
O	+0.00000	+0.00000	+0.96372
C	+1.27315	+0.00000	+1.37976
C	+1.37074	+0.04284	+2.88808
N	+2.71147	−0.33604	+3.29294
H	+3.36087	−0.21464	+2.52465
O	+2.20505	−0.02040	+0.62425
C	+0.86449	+1.39676	+3.43813
H	+0.65434	−0.71165	+3.24466
H	+3.03143	+0.21850	+4.07431
C	+1.42091	+2.70613	+2.85069
H	−0.22762	+1.41200	+3.33782
H	+1.07356	+1.37353	+4.51623
C	+2.94784	+2.77218	+2.80772
C	+0.84826	+3.03999	+1.46958
H	+1.07395	+3.49108	+3.53690
H	+3.27246	+3.77267	+2.50473
H	+3.39736	+2.55643	+3.78272
H	+3.34838	+2.06075	+2.07778
H	+1.07145	+4.08015	+1.21294
H	+1.29456	+2.41904	+0.68624

H	−0.23762	+2.90638	+1.43978

22

∗ E = +3.914 kcal/mol ; (57) 178_040_183_144_289

H	+0.00000	+0.00000	+0.00000
O	+0.00000	+0.00000	+0.96343
C	+1.27958	+0.00000	+1.37437
C	+1.38102	+0.03776	+2.89140
N	+0.35111	−0.82871	+3.45626
H	+0.36706	−0.78366	+4.46633
O	+2.19668	+0.02428	+0.60561
C	+2.77621	−0.37377	+3.36841
H	+1.21930	+1.10627	+3.13575
H	−0.57185	−0.55748	+3.14074
C	+3.28199	+0.38514	+4.60443
H	+2.73969	−1.44990	+3.57566
H	+3.48496	−0.22536	+2.54785
C	+3.62923	+1.83597	+4.27228
C	+2.30080	+0.32735	+5.77440
H	+4.20794	−0.11517	+4.91685
H	+4.05688	+2.34453	+5.14192
H	+4.35131	+1.89406	+3.45245
H	+2.73876	+2.40116	+3.97320
H	+2.72748	+0.79460	+6.66698
H	+1.37818	+0.87369	+5.53781
H	+2.03678	−0.70502	+6.03035

22

∗ E = +3.921 kcal/mol ; (58) 180_310_062_057_147

H	+0.00000	+0.00000	+0.00000
O	+0.00000	+0.00000	+0.96362
C	+1.27778	+0.00000	+1.38181
C	+1.36798	−0.00851	+2.89253
N	+0.50566	+1.04154	+3.41730
H	−0.46040	+0.85930	+3.16892
O	+2.20566	−0.03702	+0.62635
C	+1.06144	−1.41638	+3.43546
H	+2.40856	+0.24155	+3.12420
H	+0.56481	+1.07150	+4.42781
C	+1.96105	−2.53812	+2.88983
H	+1.16619	−1.35338	+4.52650
H	+0.00575	−1.64894	+3.23395

C	+2.17417	−3.61153	+3.95597
C	+1.37541	−3.17599	+1.62869
H	+2.93642	−2.09882	+2.63367
H	+2.79882	−4.42706	+3.57913
H	+2.65256	−3.19970	+4.84993
H	+1.21168	−4.04161	+4.25842
H	+2.03891	−3.95773	+1.24674
H	+0.40856	−3.63932	+1.85899
H	+1.22236	−2.45469	+0.82225

22

∗ E = +3.943 kcal/mol ; (59) 178_297_068_065_165

H	+0.00000	+0.00000	+0.00000
O	+0.00000	+0.00000	+0.96362
C	+1.27901	+0.00000	+1.38023
C	+1.35917	+0.05450	+2.89362
N	+0.75143	+1.32384	+3.28817
H	−0.24315	+1.30785	+3.08925
O	+2.20517	−0.03440	+0.62375
C	+0.71801	−1.17017	+3.56232
H	+2.42681	+0.09257	+3.13279
H	+0.86382	+1.47241	+4.28353
C	+1.38294	−2.52679	+3.30251
H	+0.73076	−0.96976	+4.64219
H	−0.34027	−1.21878	+3.26581
C	+0.86866	−3.54251	+4.32215
C	+1.12815	−3.04572	+1.88589
H	+2.46738	−2.41002	+3.44388
H	+1.33500	−4.52132	+4.17617
H	+1.06731	−3.21896	+5.34858
H	−0.21520	−3.67086	+4.21465
H	+1.50261	−4.06870	+1.78150
H	+0.05120	−3.06120	+1.67820
H	+1.61741	−2.44261	+1.11764

22

∗ E = +3.944 kcal/mol ; (60) 176_157_047_182_098

H	+0.00000	+0.00000	+0.00000
O	+0.00000	+0.00000	+0.96388
C	+1.27635	+0.00000	+1.37569
C	+1.39275	+0.09051	+2.88091
N	+2.67911	−0.45203	+3.26323

H	+3.41260	−0.07225	+2.67507
O	+2.20441	−0.00982	+0.61828
C	+1.11332	+1.55787	+3.27336
H	+0.59914	−0.53031	+3.31672
H	+2.89117	−0.25570	+4.23184
C	+1.24228	+1.81832	+4.78610
H	+1.80608	+2.21147	+2.72435
H	+0.10084	+1.80842	+2.93398
C	+2.61138	+2.39940	+5.14448
C	+0.14171	+2.76188	+5.26888
H	+1.11919	+0.85807	+5.31087
H	+2.72367	+2.51055	+6.22738
H	+3.43640	+1.78346	+4.77531
H	+2.72086	+3.39278	+4.69356
H	+0.23469	+2.96570	+6.34015
H	+0.20676	+3.71985	+4.73971
H	−0.85259	+2.34439	+5.08425

22

∗ E = +3.976 kcal/mol ; (61) 182_059_181_092_304

H	+0.00000	+0.00000	+0.00000
O	+0.00000	+0.00000	+0.96363
C	+1.28035	+0.00000	+1.37886
C	+1.35198	−0.05264	+2.89762
N	+0.67155	−1.28372	+3.30141
H	+0.67425	−1.36622	+4.31024
O	+2.20102	+0.03273	+0.61670
C	+2.79610	−0.04481	+3.39807
H	+0.82668	+0.85302	+3.24971
H	−0.29210	−1.28273	+2.98906
C	+3.36234	+1.34867	+3.70896
H	+2.83791	−0.65136	+4.31120
H	+3.42155	−0.54795	+2.65357
C	+3.26917	+2.29939	+2.51628
C	+2.69793	+1.96316	+4.94143
H	+4.42559	+1.20628	+3.94238
H	+3.74633	+3.25625	+2.74945
H	+3.74193	+1.87683	+1.62629
H	+2.22254	+2.51373	+2.26444
H	+3.15868	+2.92298	+5.19430
H	+1.63209	+2.15412	+4.76963

H +2.78547 +1.30599 +5.81276
22
∗ E = +3.993 kcal/mol ; (62) 178_009_300_284_278
H +0.00000 +0.00000 +0.00000
O +0.00000 +0.00000 +0.96400
C +1.28263 +0.00000 +1.36526
C +1.44098 +0.05567 +2.88642
N +0.22074 −0.09593 +3.65563
H −0.44055 +0.63367 +3.41912
O +2.19469 +0.00147 +0.58707
C +2.56838 −0.88259 +3.34135
H +1.80938 +1.07663 +3.06050
H −0.23510 −0.97406 +3.43795
C +2.26719 −2.39321 +3.37068
H +3.41253 −0.70130 +2.66767
H +2.87476 −0.56550 +4.34388
C +1.53377 −2.84277 +4.63690
C +1.54840 −2.88251 +2.11308
H +3.24936 −2.88446 +3.39090
H +1.47379 −3.93544 +4.67195
H +2.05884 −2.50376 +5.53511
H +0.51502 −2.45132 +4.69399
H +1.43165 −3.97011 +2.13728
H +0.53955 −2.45652 +2.02973
H +2.09867 −2.61771 +1.20500
22
∗ E = +4.134 kcal/mol ; (63) 175_273_177_079_178
H +0.00000 +0.00000 +0.00000
O +0.00000 +0.00000 +0.96385
C +1.27412 +0.00000 +1.36954
C +1.44107 +0.11634 +2.87899
N +1.43324 +1.55558 +3.14073
H +1.49086 +1.73197 +4.13598
O +2.19727 −0.02856 +0.60343
C +0.34607 −0.56639 +3.69311
H +2.41077 −0.35929 +3.10003
H +2.22540 +2.00627 +2.69788
C +0.50722 −2.08365 +3.81839
H +0.36246 −0.13048 +4.70123
H −0.63295 −0.32085 +3.26418

C	−0.60996	−2.65014	+4.69102
C	+0.52494	−2.78241	+2.45960
H	+1.46907	−2.27779	+4.31588
H	−0.48903	−3.72715	+4.84063
H	−0.63431	−2.17004	+5.67444
H	−1.58333	−2.48595	+4.21409
H	+0.58585	−3.86760	+2.58613
H	−0.39114	−2.55671	+1.90188
H	+1.38006	−2.47907	+1.84572

22

∗ E = +4.147 kcal/mol ; (64) 179_042_189_179_094

H	+0.00000	+0.00000	+0.00000
O	+0.00000	+0.00000	+0.96357
C	+1.28004	+0.00000	+1.37577
C	+1.38788	+0.02516	+2.88963
N	+0.40163	−0.88469	+3.46068
H	+0.35920	−0.76579	+4.46547
O	+2.20007	+0.01990	+0.61109
C	+2.80811	−0.31867	+3.32212
H	+1.19396	+1.08084	+3.16500
H	−0.52060	−0.69140	+3.08935
C	+3.00888	−0.28476	+4.84776
H	+3.06654	−1.31113	+2.93192
H	+3.48215	+0.39293	+2.83389
C	+2.82585	−1.66974	+5.47362
C	+4.39562	+0.25456	+5.19512
H	+2.26248	+0.40094	+5.28074
H	+2.87443	−1.61958	+6.56594
H	+1.88187	−2.13801	+5.18370
H	+3.63113	−2.33227	+5.13502
H	+4.56362	+0.25503	+6.27666
H	+5.16814	−0.37436	+4.73730
H	+4.53374	+1.27501	+4.82530

22

∗ E = +4.355 kcal/mol ; (65) 177_158_043_177_320

H	+0.00000	+0.00000	+0.00000
O	+0.00000	+0.00000	+0.96381
C	+1.27653	+0.00000	+1.37533
C	+1.39447	+0.08225	+2.88170
N	+2.69052	−0.44849	+3.25114

H	+3.40468	−0.10271	+2.61972
O	+2.20345	−0.01102	+0.61615
C	+1.09056	+1.54602	+3.27011
H	+0.60705	−0.55627	+3.30162
H	+2.93894	−0.20322	+4.19758
C	+1.09134	+1.85915	+4.78025
H	+1.80982	+2.20077	+2.75952
H	+0.09840	+1.78380	+2.86650
C	+0.43950	+0.74846	+5.60237
C	+2.47158	+2.21575	+5.33856
H	+0.47304	+2.75913	+4.89491
H	+0.33398	+1.05249	+6.64784
H	−0.55547	+0.49534	+5.22140
H	+1.04362	−0.16710	+5.58756
H	+2.37873	+2.60893	+6.35596
H	+3.14216	+1.35272	+5.39348
H	+2.96303	+2.97792	+4.72631

22

∗ E = +4.570 kcal/mol ; (66) 179_040_193_175_317

H	+0.00000	+0.00000	+0.00000
O	+0.00000	+0.00000	+0.96352
C	+1.28087	+0.00000	+1.37400
C	+1.39324	+0.03014	+2.88902
N	+0.37165	−0.83483	+3.46707
H	+0.29200	−0.67068	+4.46194
O	+2.19749	+0.01966	+0.60501
C	+2.80823	−0.36641	+3.30024
H	+1.23917	+1.09627	+3.14563
H	−0.53289	−0.64719	+3.05192
C	+3.13127	−0.26097	+4.80188
H	+2.99372	−1.38947	+2.95221
H	+3.49122	+0.28239	+2.74168
C	+2.57679	+1.01854	+5.42743
C	+2.71433	−1.49331	+5.60777
H	+4.22602	−0.20588	+4.86605
H	+2.93678	+1.13685	+6.45366
H	+2.87387	+1.90721	+4.86038
H	+1.48018	+1.00238	+5.47056
H	+3.08637	−1.42032	+6.63491
H	+1.62900	−1.61550	+5.66011

H +3.11981 −2.40732 +5.16409
22
∗ E = +4.637 kcal/mol ; (67) 181_343_297_303_308
H +0.00000 +0.00000 +0.00000
O +0.00000 +0.00000 +0.96425
C +1.28283 +0.00000 +1.36848
C +1.44363 −0.01723 +2.88361
N +0.27511 +0.35199 +3.66151
H +0.01206 +1.31220 +3.47865
O +2.19913 +0.00458 +0.59593
C +2.05734 −1.35741 +3.33663
H +2.21380 +0.73885 +3.07666
H −0.52485 −0.22188 +3.43115
C +1.35138 −2.67321 +2.96439
H +3.07967 −1.40033 +2.94364
H +2.12627 −1.28637 +4.42769
C −0.13110 −2.71025 +3.33809
C +1.52985 −3.06559 +1.49553
H +1.86136 −3.43934 +3.56425
H −0.52066 −3.72954 +3.25588
H −0.30463 −2.35876 +4.36002
H −0.71889 −2.09187 +2.64766
H +1.22223 −4.10485 +1.34226
H +0.91098 −2.45092 +0.83431
H +2.56947 −2.96197 +1.17111
22
∗ E = +4.785 kcal/mol ; (68) 359_170_306_181_182
H +0.00000 +0.00000 +0.00000
O +0.00000 +0.00000 +0.96027
C +1.25754 +0.00000 +1.44227
C +2.36863 −0.02114 +0.38928
N +3.67950 +0.21883 +0.94703
H +3.68215 +1.07952 +1.48225
O +1.45795 +0.00724 +2.61788
C +2.34280 −1.36847 −0.34790
H +2.14671 +0.77988 −0.33246
H +3.90155 −0.51749 +1.61018
C +3.43912 −1.51762 −1.40636
H +2.43659 −2.17405 +0.39418
H +1.36513 −1.51148 −0.83203

C +3.28110 −2.86064 −2.11479
C +3.41006 −0.36653 −2.40745
H +4.40686 −1.49517 −0.89149
H +4.08956 −3.02569 −2.83286
H +3.28404 −3.69287 −1.40364
H +2.33369 −2.89553 −2.66646
H +4.11993 −0.54245 −3.22097
H +2.41240 −0.26670 −2.85545
H +3.67684 +0.58287 −1.93421
22
∗ E = +4.838 kcal/mol ; (69) 175_276_176_078_304
H +0.00000 +0.00000 +0.00000
O +0.00000 +0.00000 +0.96380
C +1.27459 +0.00000 +1.36905
C +1.44114 +0.11729 +2.87871
N +1.36221 +1.55266 +3.15325
H +1.39475 +1.72127 +4.15098
O +2.19684 −0.02973 +0.60180
C +0.37157 −0.61516 +3.68757
H +2.43474 −0.30556 +3.08985
H +2.13984 +2.04462 +2.72916
C +0.56356 −2.13513 +3.79282
H +0.38181 −0.19406 +4.70208
H −0.60838 −0.37835 +3.26059
C +0.66820 −2.80640 +2.42383
C +1.77102 −2.49784 +4.65803
H −0.33238 −2.52580 +4.29288
H +0.70868 −3.89459 +2.52979
H −0.18428 −2.55541 +1.78582
H +1.58541 −2.50150 +1.90371
H +1.83402 −3.58093 +4.80062
H +2.70866 −2.17950 +4.18923
H +1.71079 −2.02994 +5.64598
22
∗ E = +4.850 kcal/mol ; (70) 182_342_054_293_145
H +0.00000 +0.00000 +0.00000
O +0.00000 +0.00000 +0.96404
C +1.27809 +0.00000 +1.36928
C +1.44270 −0.04329 +2.87858
N +0.21361 +0.33716 +3.55133

H	−0.56736	−0.22657	+3.23715
O	+2.19718	−0.01579	+0.60004
C	+2.03662	−1.42162	+3.23708
H	+2.20554	+0.71288	+3.09877
H	+0.30735	+0.21979	+4.55235
C	+1.09570	−2.61269	+2.97887
H	+2.95767	−1.53691	+2.65343
H	+2.33092	−1.40636	+4.29514
C	+1.89486	−3.82967	+2.51504
C	+0.28037	−2.97592	+4.22140
H	+0.39508	−2.33809	+2.17490
H	+1.24326	−4.69282	+2.34672
H	+2.43647	−3.62002	+1.58803
H	+2.63112	−4.11128	+3.27735
H	−0.40846	−3.80060	+4.01334
H	+0.95225	−3.29864	+5.02586
H	−0.30588	−2.13311	+4.59698

22

* E = +4.864 kcal/mol ; (71) 182_319_064_178_095

H	+0.00000	+0.00000	+0.00000
O	+0.00000	+0.00000	+0.96356
C	+1.27815	+0.00000	+1.37733
C	+1.38432	−0.05429	+2.88709
N	+0.38104	+0.80975	+3.48731
H	−0.55372	+0.48009	+3.27428
O	+2.20373	−0.02269	+0.61824
C	+1.34988	−1.54045	+3.29647
H	+2.37328	+0.34738	+3.13272
H	+0.48804	+0.83312	+4.49339
C	+1.49692	−1.76241	+4.81234
H	+0.40867	−1.98735	+2.94473
H	+2.16216	−2.05018	+2.76320
C	+0.13836	−1.88647	+5.50460
C	+2.33094	−3.01122	+5.09453
H	+2.02904	−0.89547	+5.23438
H	+0.25522	−1.98559	+6.58824
H	−0.51095	−1.02884	+5.30888
H	−0.38149	−2.78125	+5.14222
H	+2.42789	−3.18948	+6.16999
H	+1.85238	−3.89270	+4.65181

H +3.33510 −2.92665 +4.66876
22
∗ E = +5.242 kcal/mol ; (72) 182_319_069_175_317
H +0.00000 +0.00000 +0.00000
O +0.00000 +0.00000 +0.96353
C +1.27785 +0.00000 +1.37682
C +1.38403 −0.05083 +2.88809
N +0.38474 +0.82470 +3.48034
H −0.54940 +0.45776 +3.33737
O +2.20335 −0.02471 +0.61721
C +1.33358 −1.54139 +3.28042
H +2.37480 +0.34914 +3.12436
H +0.53755 +0.92009 +4.47483
C +1.54231 −1.85747 +4.77296
H +0.37169 −1.95700 +2.94890
H +2.11529 −2.05069 +2.70166
C +2.68464 −1.04434 +5.37976
C +0.26902 −1.72775 +5.61218
H +1.83493 −2.91503 +4.81368
H +2.91295 −1.39130 +6.39170
H +3.59765 −1.12201 +4.78025
H +2.42509 +0.01890 +5.45314
H +0.43616 −2.11782 +6.62127
H −0.05567 −0.68855 +5.71759
H −0.55764 −2.29056 +5.16727
22
∗ E = +5.404 kcal/mol ; (73) 002_180_305_285_057
H +0.00000 +0.00000 +0.00000
O +0.00000 +0.00000 +0.95986
C +1.25621 +0.00000 +1.44208
C +2.37938 +0.06000 +0.39464
N +3.69642 +0.05002 +0.99113
H +3.77324 +0.80148 +1.66697
O +1.45157 +0.00304 +2.61917
C +2.27600 −1.01719 −0.69793
H +2.24269 +1.03380 −0.10054
H +3.82324 −0.80464 +1.52380
C +2.67196 −2.43436 −0.27207
H +1.25654 −1.05602 −1.11240
H +2.92603 −0.70244 −1.52150

C +1.84978 −2.93732 +0.91415
C +2.51908 −3.37581 −1.46489
H +3.73318 −2.41567 +0.01053
H +2.06644 −3.99099 +1.11321
H +2.04997 −2.38136 +1.83524
H +0.77587 −2.85377 +0.70322
H +2.85660 −4.38594 −1.21593
H +1.46637 −3.44175 −1.76564
H +3.09458 −3.02735 −2.32814
22
* E = +5.415 kcal/mol ; (74) 000_152_298_060_062
H +0.00000 +0.00000 +0.00000
O +0.00000 +0.00000 +0.96053
C +1.25818 +0.00000 +1.43909
C +2.38428 −0.00096 +0.39917
N +3.59304 +0.62476 +0.89505
H +3.42882 +1.60023 +1.11461
O +1.46010 −0.02156 +2.61496
C +2.70481 −1.45184 +0.01009
H +2.05197 +0.55240 −0.49009
H +3.85796 +0.17943 +1.76851
C +1.56575 −2.26756 −0.60645
H +3.54792 −1.41290 −0.69115
H +3.06804 −1.95972 +0.91417
C +1.08639 −1.66464 −1.92799
C +2.03899 −3.70142 −0.83844
H +0.72405 −2.30135 +0.09930
H +0.29481 −2.27408 −2.37317
H +0.69665 −0.64392 −1.83206
H +1.91672 −1.61766 −2.64265
H +1.24219 −4.31793 −1.26461
H +2.88413 −3.71474 −1.53667
H +2.36765 −4.16668 +0.09540
22
* E = +5.522 kcal/mol ; (75) 179_359_049_285_282
H +0.00000 +0.00000 +0.00000
O +0.00000 +0.00000 +0.96395
C +1.27843 +0.00000 +1.36411
C +1.45884 +0.01447 +2.87341
N +0.18316 +0.04777 +3.56850

H	−0.45436	−0.65268	+3.21135
O	+2.19405	−0.00057	+0.58943
C	+2.47417	−1.06599	+3.29914
H	+1.95896	+0.97820	+3.05000
H	+0.31612	−0.12247	+4.55708
C	+2.01665	−2.53507	+3.28795
H	+3.34138	−0.95576	+2.63751
H	+2.81715	−0.81417	+4.31109
C	+1.14364	−2.92090	+4.48577
C	+1.33631	−2.93329	+1.97909
H	+2.94008	−3.12396	+3.37158
H	+1.04525	−4.00933	+4.54931
H	+1.57808	−2.56950	+5.42779
H	+0.13254	−2.51095	+4.40574
H	+1.14014	−4.00956	+1.95913
H	+0.36789	−2.42941	+1.86804
H	+1.95141	−2.68032	+1.10984

22

∗ E = +5.915 kcal/mol ; (76) 006_190_209_287_056

H	+0.00000	+0.00000	+0.00000
O	+0.00000	+0.00000	+0.95974
C	+1.25186	+0.00000	+1.44059
C	+2.37125	+0.14515	+0.40871
N	+3.63128	−0.11577	+1.07232
H	+4.39418	+0.39147	+0.64640
O	+1.44950	−0.03728	+2.61758
C	+2.22439	−0.73311	−0.84670
H	+2.26753	+1.19734	+0.07996
H	+3.56761	+0.13104	+2.05301
C	+2.49606	−2.22625	−0.64492
H	+1.22945	−0.60415	−1.30438
H	+2.92533	−0.34688	−1.59805
C	+1.61830	−2.83344	+0.44592
C	+2.29959	−2.95829	−1.97040
H	+3.54149	−2.32582	−0.33169
H	+1.76759	−3.91603	+0.49821
H	+1.85387	−2.42331	+1.43247
H	+0.55431	−2.65679	+0.24235
H	+2.55505	−4.01771	−1.87626
H	+1.25294	−2.89770	−2.29366

H +2.92016 −2.52941 −2.76409
22
* E = +5.926 kcal/mol ; (77) 000_171_300_318_181

H	+0.00000	+0.00000	+0.00000
O	+0.00000	+0.00000	+0.96040
C	+1.25933	+0.00000	+1.44115
C	+2.36284	−0.00859	+0.37887
N	+3.68709	+0.20760	+0.92030
H	+3.73200	+1.10907	+1.38078
O	+1.45974	−0.00906	+2.61670
C	+2.27894	−1.31235	−0.44388
H	+2.15222	+0.82745	−0.30356
H	+3.89297	−0.48023	+1.63464
C	+2.00850	−2.60410	+0.33909
H	+1.49896	−1.21161	−1.21171
H	+3.23001	−1.40104	−0.98326
C	+1.97454	−3.77704	−0.63902
C	+3.03697	−2.86215	+1.44133
H	+1.01791	−2.52166	+0.81066
H	+1.72169	−4.70968	−0.12673
H	+1.24132	−3.61721	−1.43608
H	+2.95667	−3.90870	−1.10825
H	+2.90572	−3.86630	+1.85555
H	+4.05644	−2.79671	+1.04160
H	+2.93267	−2.15620	+2.27102

22
* E = +6.360 kcal/mol ; (78) 356_147_037_062_062

H	+0.00000	+0.00000	+0.00000
O	+0.00000	+0.00000	+0.96023
C	+1.25475	+0.00000	+1.43794
C	+2.37324	−0.08595	+0.40475
N	+3.50274	+0.66475	+0.91927
H	+3.56212	+0.52801	+1.92334
O	+1.46018	+0.01681	+2.61324
C	+2.68420	−1.57445	+0.14944
H	+2.03190	+0.38159	−0.53022
H	+4.36908	+0.35013	+0.50140
C	+1.54881	−2.42611	−0.42621
H	+3.53913	−1.61693	−0.54159
H	+3.02275	−2.00331	+1.10181

C	+1.10273	−1.92860	−1.80208
C	+2.00515	−3.88036	−0.52754
H	+0.69341	−2.38694	+0.26264
H	+0.32358	−2.57425	−2.21726
H	+0.70751	−0.90637	−1.79270
H	+1.94965	−1.93564	−2.49891
H	+1.20654	−4.52038	−0.91393
H	+2.86064	−3.96447	−1.20832
H	+2.31231	−4.26931	+0.44761

22

∗ E = +6.420 kcal/mol ; (79) 181_056_180_070_251

H	+0.00000	+0.00000	+0.00000
O	+0.00000	+0.00000	+0.96364
C	+1.27934	+0.00000	+1.37890
C	+1.35825	−0.03399	+2.89342
N	+0.61367	−1.21700	+3.32756
H	+0.62861	−1.28587	+4.33744
O	+2.19995	+0.03121	+0.61529
C	+2.79119	−0.12181	+3.41241
H	+0.89282	+0.90553	+3.23654
H	−0.35303	−1.16510	+3.03015
C	+3.70784	+1.12095	+3.25629
H	+2.70159	−0.36239	+4.47916
H	+3.26112	−0.99329	+2.94276
C	+4.79474	+0.90424	+2.20223
C	+2.95268	+2.42073	+2.97414
H	+4.21822	+1.25157	+4.21920
H	+5.48221	+1.75619	+2.17815
H	+5.38013	+0.00603	+2.42301
H	+4.35384	+0.78375	+1.20990
H	+3.65541	+3.25846	+2.93339
H	+2.44264	+2.37918	+2.00465
H	+2.21108	+2.64989	+3.74629

22

∗ E = +6.481 kcal/mol ; (80) 358_167_310_176_316

H	+0.00000	+0.00000	+0.00000
O	+0.00000	+0.00000	+0.96022
C	+1.25721	+0.00000	+1.44334
C	+2.37261	−0.05166	+0.39376
N	+3.67366	+0.24001	+0.95095

H	+3.63549	+1.09156	+1.50026
O	+1.45582	+0.03114	+2.61883
C	+2.35088	−1.43096	−0.28330
H	+2.13998	+0.71436	−0.36263
H	+3.93141	−0.49550	+1.60208
C	+3.34576	−1.62625	−1.44306
H	+2.52827	−2.19492	+0.48689
H	+1.33651	−1.61476	−0.66142
C	+3.37628	−0.41787	−2.37595
C	+4.75881	−1.97575	−0.97319
H	+2.97016	−2.48650	−2.01351
H	+3.97580	−0.63250	−3.26518
H	+2.37179	−0.13420	−2.71128
H	+3.82970	+0.44166	−1.86955
H	+5.38502	−2.25152	−1.82761
H	+5.22230	−1.12351	−0.47052
H	+4.74852	−2.82392	−0.28037

22

∗ E = +6.728 kcal/mol ; (81) 357_163_021_303_090

H	+0.00000	+0.00000	+0.00000
O	+0.00000	+0.00000	+0.96050
C	+1.25473	+0.00000	+1.43987
C	+2.35353	−0.06902	+0.38876
N	+3.60435	+0.33131	+0.99318
H	+3.55185	+0.23369	+2.00101
O	+1.45819	+0.01496	+2.61588
C	+2.38971	−1.47826	−0.24337
H	+2.08219	+0.64515	−0.40634
H	+4.38216	−0.21375	+0.64704
C	+2.58608	−2.62796	+0.75760
H	+1.47799	−1.65629	−0.83291
H	+3.21529	−1.46464	−0.96729
C	+1.25144	−3.16355	+1.28001
C	+3.38032	−3.76086	+0.10977
H	+3.16167	−2.23955	+1.61006
H	+1.41729	−3.97308	+1.99694
H	+0.65120	−2.40053	+1.78157
H	+0.66082	−3.57040	+0.44995
H	+3.51522	−4.59629	+0.80307
H	+2.85033	−4.14251	−0.77140

H +4.36960 −3.42392 −0.21586
22
∗ E = +6.841 kcal/mol ; (82) 359_169_296_294_143
H +0.00000 +0.00000 +0.00000
O +0.00000 +0.00000 +0.96017
C +1.25739 +0.00000 +1.44183
C +2.37389 −0.02374 +0.38958
N +3.67775 +0.22356 +0.96539
H +3.71411 +1.15974 +1.35197
O +1.45602 +0.00059 +2.61817
C +2.33968 −1.36875 −0.35645
H +2.15994 +0.77584 −0.33406
H +3.81346 −0.39877 +1.75706
C +2.69005 −2.58160 +0.52856
H +1.34229 −1.51298 −0.79384
H +3.04287 −1.30662 −1.19487
C +1.84101 −3.79073 +0.13936
C +4.17572 −2.93328 +0.42541
H +2.45829 −2.32841 +1.57473
H +2.10414 −4.66751 +0.73855
H +0.77355 −3.59186 +0.27638
H +2.00579 −4.04565 −0.91454
H +4.43726 −3.73631 +1.12111
H +4.40175 −3.28435 −0.58874
H +4.81800 −2.07189 +0.62384
22
∗ E = +6.850 kcal/mol ; (83) 358_167_303_173_087
H +0.00000 +0.00000 +0.00000
O +0.00000 +0.00000 +0.96022
C +1.25770 +0.00000 +1.44208
C +2.37337 −0.03872 +0.39109
N +3.67006 +0.27394 +0.94707
H +3.65268 +1.18655 +1.38790
O +1.45795 +0.01591 +2.61756
C +2.37431 −1.41650 −0.28861
H +2.13600 +0.71769 −0.37359
H +3.87921 −0.38650 +1.69008
C +3.32593 −1.53656 −1.49054
H +2.62790 −2.17809 +0.46372
H +1.35193 −1.64912 −0.61563

C +4.74249 −1.92826 −1.07076
C +2.77873 −2.56098 −2.48429
H +3.36979 −0.55496 −1.98438
H +5.39553 −2.00855 −1.94548
H +5.17082 −1.19805 −0.38260
H +4.72845 −2.90851 −0.57829
H +3.45002 −2.67791 −3.34022
H +2.68127 −3.54159 −2.00300
H +1.79179 −2.27441 −2.86168
22
∗ E = +6.949 kcal/mol ; (84) 359_145_298_057_149
H +0.00000 +0.00000 +0.00000
O +0.00000 +0.00000 +0.95985
C +1.25806 +0.00000 +1.43857
C +2.38539 −0.01738 +0.40275
N +3.53830 +0.74608 +0.83755
H +3.29887 +1.72215 +0.96718
O +1.45934 −0.00588 +2.61511
C +2.82548 −1.46152 +0.12432
H +2.01542 +0.41965 −0.53685
H +3.83417 +0.40110 +1.74581
C +1.72718 −2.42755 −0.34652
H +3.62191 −1.39161 −0.62418
H +3.28784 −1.85552 +1.04088
C +2.32733 −3.48824 −1.26877
C +1.01795 −3.11022 +0.82540
H +0.98449 −1.85983 −0.93111
H +1.56886 −4.20724 −1.59250
H +2.77911 −3.03913 −2.15827
H +3.10991 −4.04452 −0.73971
H +0.20628 −3.75137 +0.46846
H +1.73235 −3.74505 +1.36223
H +0.59504 −2.40588 +1.54574
22
∗ E = +7.361 kcal/mol ; (85) 002_167_290_309_311
H +0.00000 +0.00000 +0.00000
O +0.00000 +0.00000 +0.96036
C +1.25759 +0.00000 +1.44127
C +2.36480 +0.04503 +0.38665
N +3.66773 +0.34430 +0.94651

H	+3.69684	+1.30841	+1.25587
O	+1.45690	−0.03892	+2.61738
C	+2.38049	−1.23794	−0.47529
H	+2.11164	+0.86949	−0.29538
H	+3.82868	−0.21698	+1.77468
C	+2.39980	−2.60648	+0.22968
H	+1.51891	−1.22470	−1.15888
H	+3.27340	−1.14286	−1.10220
C	+3.45798	−2.71195	+1.32878
C	+1.03355	−3.02793	+0.77699
H	+2.66609	−3.32395	−0.55831
H	+3.56107	−3.75105	+1.65510
H	+4.43652	−2.36006	+0.98832
H	+3.15849	−2.12991	+2.20909
H	+1.06124	−4.07805	+1.08375
H	+0.74508	−2.44566	+1.65751
H	+0.24525	−2.92023	+0.02407

22

∗ E = +7.498 kcal/mol ; (86) 000_126_298_063_285

H	+0.00000	+0.00000	+0.00000
O	+0.00000	+0.00000	+0.95975
C	+1.25999	+0.00000	+1.43605
C	+2.39590	+0.00477	+0.40848
N	+3.32160	+1.09949	+0.66166
H	+2.86014	+1.99828	+0.58364
O	+1.46467	+0.00963	+2.61187
C	+3.18126	−1.30861	+0.48358
H	+1.99042	+0.13409	−0.60478
H	+3.66300	+1.02478	+1.61534
C	+2.42558	−2.60452	+0.15569
H	+4.02433	−1.18977	−0.20605
H	+3.60032	−1.37713	+1.49672
C	+1.48087	−3.06239	+1.26941
C	+1.68648	−2.51554	−1.17928
H	+3.20087	−3.37490	+0.05189
H	+1.13165	−4.08120	+1.07491
H	+1.97779	−3.05054	+2.24395
H	+0.59158	−2.42852	+1.34428
H	+1.30978	−3.49625	−1.48363
H	+0.81374	−1.85432	−1.10525

H +2.33247 −2.13515 −1.97771
22
∗ E = +7.838 kcal/mol ; (87) 006_189_205_290_276
H +0.00000 +0.00000 +0.00000
O +0.00000 +0.00000 +0.95972
C +1.25310 +0.00000 +1.44109
C +2.37186 +0.14402 +0.40807
N +3.63228 −0.08450 +1.08192
H +4.39846 +0.37473 +0.60887
O +1.45005 −0.02928 +2.61785
C +2.22719 −0.73849 −0.84441
H +2.25049 +1.19130 +0.06588
H +3.58159 +0.23984 +2.04064
C +2.42401 −2.25847 −0.67406
H +1.24783 −0.54861 −1.30928
H +2.95204 −0.37134 −1.58255
C +3.89778 −2.66738 −0.72386
C +1.76484 −2.80563 +0.59076
H +1.93148 −2.72044 −1.54078
H +3.98719 −3.75825 −0.74405
H +4.38935 −2.27559 −1.62096
H +4.42985 −2.29382 +0.15392
H +1.85043 −3.89580 +0.61943
H +2.26257 −2.41208 +1.48382
H +0.69997 −2.55604 +0.64911
22
∗ E = +7.919 kcal/mol ; (88) 005_201_205_092_073
H +0.00000 +0.00000 +0.00000
O +0.00000 +0.00000 +0.96038
C +1.25084 +0.00000 +1.44567
C +2.38066 +0.11665 +0.42175
N +3.58482 −0.39320 +1.04628
H +4.41575 +0.00475 +0.63045
O +1.44429 −0.04761 +2.62225
C +2.08767 −0.61710 −0.90010
H +2.43141 +1.20206 +0.20745
H +3.57585 −0.18730 +2.03875
C +1.42760 +0.24012 −1.99093
H +3.04460 −0.98436 −1.28973
H +1.49231 −1.51744 −0.69566

C +2.43369 +1.22851 −2.58018
C +0.85339 −0.65134 −3.08899
H +0.60292 +0.83602 −1.56297
H +1.95990 +1.88313 −3.31713
H +2.88988 +1.86342 −1.81445
H +3.23764 +0.68016 −3.08471
H +0.40545 −0.05791 −3.89125
H +1.64998 −1.26249 −3.52876
H +0.09024 −1.33225 −2.69914
22
* E = +8.076 kcal/mol ; (89) 002_178_299_289_278
H +0.00000 +0.00000 +0.00000
O +0.00000 +0.00000 +0.95978
C +1.25679 +0.00000 +1.44283
C +2.38128 +0.05246 +0.39531
N +3.69442 +0.09864 +1.00218
H +3.77473 +0.93274 +1.57283
O +1.45038 +0.00526 +2.61974
C +2.27703 −1.04147 −0.68371
H +2.22718 +1.00927 −0.12709
H +3.79363 −0.67466 +1.65201
C +2.57425 −2.49740 −0.27388
H +1.27015 −1.01073 −1.12417
H +2.96231 −0.75562 −1.48925
C +4.07001 −2.81994 −0.24840
C +1.90268 −2.89911 +1.03868
H +2.13687 −3.11649 −1.06905
H +4.22020 −3.89222 −0.08780
H +4.54818 −2.54875 −1.19453
H +4.59765 −2.28601 +0.54526
H +2.01572 −3.97341 +1.21023
H +2.34957 −2.38533 +1.89746
H +0.83099 −2.67111 +1.04007
22
* E = +8.201 kcal/mol ; (90) 359_164_014_310_310
H +0.00000 +0.00000 +0.00000
O +0.00000 +0.00000 +0.96060
C +1.25430 +0.00000 +1.43931
C +2.35180 −0.02554 +0.38296
N +3.61354 +0.35451 +0.98176

H	+3.54080	+0.33069	+1.99272
O	+1.45726	+0.01253	+2.61611
C	+2.37755	−1.37352	−0.37572
H	+2.06438	+0.73824	−0.36077
H	+4.36194	−0.26132	+0.69838
C	+2.37919	−2.68786	+0.42645
H	+1.52855	−1.40463	−1.07388
H	+3.27341	−1.33620	−1.01002
C	+3.43960	−2.74304	+1.52648
C	+1.00616	−3.03849	+1.00708
H	+2.62327	−3.46587	−0.31010
H	+3.45625	−3.73960	+1.97834
H	+4.44393	−2.53652	+1.14149
H	+3.21722	−2.02317	+2.32093
H	+1.00629	−4.07197	+1.36647
H	+0.74456	−2.40391	+1.85889
H	+0.21385	−2.94544	+0.25665

22

∗ E = +8.906 kcal/mol ; (91) 354_157_041_182_099

H	+0.00000	+0.00000	+0.00000
O	+0.00000	+0.00000	+0.95987
C	+1.25588	+0.00000	+1.43990
C	+2.36013	−0.14317	+0.40129
N	+3.57139	+0.41284	+0.95910
H	+3.65407	+0.15360	+1.93625
O	+1.46126	+0.04409	+2.61358
C	+2.41502	−1.63062	−0.01021
H	+2.08347	+0.44407	−0.48967
H	+4.39349	+0.11016	+0.45551
C	+3.52933	−1.94229	−1.02886
H	+2.55261	−2.23900	+0.89405
H	+1.44016	−1.91403	−0.43054
C	+4.76198	−2.53948	−0.34734
C	+3.02645	−2.89739	−2.11033
H	+3.82042	−0.99986	−1.51831
H	+5.57736	−2.68583	−1.06218
H	+5.13103	−1.91471	+0.47120
H	+4.51143	−3.51760	+0.07957
H	+3.81705	−3.13570	−2.82834
H	+2.69109	−3.83790	−1.65792

H +2.18242 −2.47131 −2.66147
22
∗ E = +9.493 kcal/mol ; (92) 008_073_181_069_069
H +0.00000 +0.00000 +0.00000
O +0.00000 +0.00000 +0.95977
C +1.26101 +0.00000 +1.44532
C +2.34785 +0.18744 +0.38485
N +2.29376 +1.58885 −0.03308
H +2.98368 +1.77316 −0.75050
O +1.45684 −0.11241 +2.61321
C +3.71981 −0.12338 +0.96229
H +2.12610 −0.51541 −0.44026
H +1.38753 +1.86226 −0.39246
C +3.96003 −1.60167 +1.28115
H +4.48274 +0.21531 +0.24569
H +3.83628 +0.48128 +1.86812
C +4.06029 −2.45057 +0.01349
C +5.23727 −1.74325 +2.10552
H +3.11848 −1.96049 +1.88847
H +4.25383 −3.49857 +0.26139
H +3.14784 −2.42151 −0.58981
H +4.88937 −2.09961 −0.61345
H +5.43745 −2.79070 +2.35013
H +6.09821 −1.36169 +1.54283
H +5.16896 −1.18003 +3.04055
22
∗ E = +9.707 kcal/mol ; (93) 008_081_182_085_180
H +0.00000 +0.00000 +0.00000
O +0.00000 +0.00000 +0.95980
C +1.26271 +0.00000 +1.44093
C +2.34466 +0.19079 +0.37243
N +2.41190 +1.62960 +0.12367
H +3.08464 +1.83707 −0.60337
O +1.46363 −0.09557 +2.60880
C +3.69725 −0.32448 +0.84689
H +2.03988 −0.39101 −0.52174
H +1.51982 +2.02405 −0.14887
C +3.90495 −1.82561 +0.62910
H +4.48048 +0.22574 +0.30995
H +3.80325 −0.07557 +1.91000

C	+5.28743	−2.23167	+1.13315
C	+2.82527	−2.66817	+1.30730
H	+3.86115	−2.01713	−0.45424
H	+5.48519	−3.29006	+0.93915
H	+6.07787	−1.64363	+0.65601
H	+5.35659	−2.07095	+2.21510
H	+3.03468	−3.73467	+1.18029
H	+2.78218	−2.44851	+2.37944
H	+1.82745	−2.48185	+0.89373

22

∗ E = +10.340 kcal/mol ; (94) 008_080_182_085_306

H	+0.00000	+0.00000	+0.00000
O	+0.00000	+0.00000	+0.95977
C	+1.26261	+0.00000	+1.44114
C	+2.34370	+0.18586	+0.37071
N	+2.39578	+1.62238	+0.09858
H	+3.06985	+1.82339	−0.62918
O	+1.46203	−0.09258	+2.60967
C	+3.70443	−0.29743	+0.86151
H	+2.03972	−0.40766	−0.51322
H	+1.50078	+2.00022	−0.18795
C	+3.95443	−1.80168	+0.68048
H	+4.47804	+0.25870	+0.31679
H	+3.78985	−0.01631	+1.91637
C	+2.85385	−2.65737	+1.30681
C	+4.15070	−2.17076	−0.79055
H	+4.89236	−2.02488	+1.20502
H	+3.10448	−3.71995	+1.23462
H	+2.69301	−2.40493	+2.35802
H	+1.89852	−2.51937	+0.78245
H	+4.40693	−3.22943	−0.89507
H	+3.23653	−2.00423	−1.37253
H	+4.95244	−1.58224	−1.24813

22

∗ E = +10.987 kcal/mol ; (95) 359_125_176_064_286

H	+0.00000	+0.00000	+0.00000
O	+0.00000	+0.00000	+0.95956
C	+1.26355	+0.00000	+1.43605
C	+2.37214	−0.03240	+0.37756
N	+3.26046	+1.09238	+0.61898

H	+4.04844	+1.06764	−0.01569
O	+1.46988	+0.00802	+2.60697
C	+3.18952	−1.32065	+0.51976
H	+1.90847	−0.00742	−0.62572
H	+2.79547	+1.98519	+0.51489
C	+2.46444	−2.64830	+0.25604
H	+4.03083	−1.23484	−0.18185
H	+3.61147	−1.31333	+1.53149
C	+1.51531	−3.05657	+1.38513
C	+1.73572	−2.65789	−1.08809
H	+3.25649	−3.40716	+0.20811
H	+1.19265	−4.09364	+1.25078
H	+1.99678	−2.96895	+2.36330
H	+0.61095	−2.44007	+1.40596
H	+1.39243	−3.66613	−1.33740
H	+0.84363	−2.01988	−1.05761
H	+2.37677	−2.30575	−1.90366

7 Pro

7.1 Reference Z-matrix for Pro

Bond distances are given in Å; bond and dihedral angles, in degrees.

c						
c	1	cc2				
c	2	cc3	1	ccc3		
c	3	cc4	2	ccc4	1	dih4
n	4	nc5	3	ncc5	2	dih5
c	1	cc6	2	ccc6	5	dih6
o	6	oc7	1	occ7	2	dih7
h	7	ho8	6	hoc8	1	dih8
o	6	oc9	1	occ9	7	dih9
h	1	hc10	2	hcc10	5	dih10
h	2	hc11	1	hcc11	3	dih11
h	2	hc12	1	hcc12	3	dih12
h	3	hc13	2	hcc13	4	dih13
h	3	hc14	2	hcc14	4	dih14
h	4	hc15	3	hcc15	5	dih15
h	4	hc16	3	hcc16	5	dih16
h	5	hn17	4	hnc17	1	dih17

cc2	1.500000
cc3	1.500000
ccc3	109.471
cc4	1.500000
ccc4	109.471
dih4	0.000
nc5	1.500000
ncc5	109.471
dih5	0.000
cc6	1.500000
ccc6	109.471
dih6	−120.000
oc7	1.500000
occ7	109.471
dih7	180.000
ho8	1.070000
hoc8	109.471

```
dih8         180.000
oc9          1.380000
occ9         109.471
dih9         180.000
hc10         1.070000
hcc10        109.471
dih10        120.000
hc11         1.070000
hcc11        109.471
dih11        120.000
hc12         1.070000
hcc12        109.471
dih12        240.000
hc13         1.070000
hcc13        109.471
dih13        120.000
hc14         1.070000
hcc14        109.471
dih14        240.000
hc15         1.070000
hcc15        109.471
dih15        120.000
hc16         1.070000
hcc16        109.471
dih16        240.000
hn17         1.030000
hnc17        109.471
dih17        120.000
```

7.2 Reference energy and target torsions for Pro

- Reference M08-HX/MG3S energy: -401.1769285 hartree
- Target torsions:
  - torsion1: dih8
  - torsion2: dih7
  - torsion3: dih5
  - torsion4: dih4

– torsion5: dih17

7.3 Properties of Pro conformers

Table S7: Relative energy (kcal mol^{-1}), rotational constants (B1 to B3, GHz) and dipolar moment (Debye) for the conformers of Pro located at the M08-HX/MG3S level.

Conformer	Rel. Energy	B1	B2	B3	Dip. Mom.
1	0.000	1.419	1.699	3.701	5.597
2	0.684	1.363	1.720	3.685	5.488
3	1.176	1.371	1.676	3.780	1.539
4	1.313	1.421	1.643	3.812	1.851
5	2.406	1.438	1.778	3.525	1.445
6	3.191	1.359	1.621	3.889	2.215
7	3.660	1.174	1.484	4.350	2.205
8	3.706	1.419	1.529	4.062	2.428
9	3.833	1.416	1.518	4.039	2.132
10	3.930	1.421	1.497	4.099	2.753
11	4.243	1.207	1.468	4.323	2.431
12	4.809	1.180	1.520	4.240	4.616
13	5.663	1.364	1.678	3.766	3.618
14	5.760	1.419	1.634	3.815	3.730
15	8.073	1.566	1.720	3.611	3.120
16	9.450	1.567	1.718	3.587	5.145

7.4 Vibrational frequencies of Pro conformers

Harmonic vibrational zero point energy (ZPE) and frequencies for each conformer in cm^{-1}.

- Conformer (1); ZPE = 32088.698 cm^{-1}:

51.62	100.19	208.42	312.82	355.56	484.22
565.11	592.58	636.69	744.85	810.63	841.56
881.39	895.90	922.80	937.72	969.52	983.04
1057.08	1098.41	1149.75	1176.67	1206.49	1245.26
1258.44	1272.01	1317.02	1318.60	1344.75	1383.32
1425.96	1448.70	1480.54	1490.20	1519.90	1888.13
3010.72	3045.36	3059.93	3077.38	3091.59	3129.52
3137.65	3615.45	3633.94			

- Conformer (2); ZPE = 32111.353 cm^{-1}:

39.66	88.78	205.15	268.94	380.71	513.74
553.98	604.60	684.83	766.32	792.90	823.80
849.92	882.77	930.38	943.83	969.74	1005.67
1059.19	1076.57	1144.83	1181.17	1217.15	1236.29
1244.36	1288.64	1307.99	1323.55	1352.30	1367.97
1424.34	1459.49	1484.88	1491.50	1509.16	1890.58
3024.11	3052.31	3078.23	3082.78	3116.43	3121.35
3133.90	3579.49	3668.40			

- Conformer (3); ZPE = 31989.351 cm^{-1}:

59.60	71.73	206.19	242.97	330.25	439.71
556.43	586.11	637.04	675.60	748.53	806.89
841.81	896.92	931.80	942.09	980.79	1014.60
1081.23	1113.36	1163.86	1193.54	1203.36	1227.87
1240.44	1278.00	1309.11	1326.04	1339.67	1369.76
1402.46	1465.07	1482.17	1501.43	1516.08	1857.25
2971.71	3047.35	3070.59	3076.34	3098.34	3120.01
3134.00	3576.15	3844.46			

- Conformer (4); ZPE = 31956.926 cm^{-1}:

52.60	66.75	189.93	277.15	314.72	437.75
552.03	593.65	617.37	702.63	751.90	793.13
837.21	898.56	929.18	942.18	965.81	988.86
1052.81	1109.66	1165.74	1176.97	1200.97	1220.51
1255.03	1293.76	1313.64	1314.73	1343.79	1372.32
1404.30	1479.48	1487.66	1496.78	1510.97	1855.83
3019.22	3039.75	3045.82	3063.25	3101.17	3117.37
3127.12	3586.82	3846.96			

- Conformer (5); ZPE = 31981.625 cm^{-1}:

39.80	65.46	185.02	269.44	366.29	507.77
513.00	593.62	626.61	675.62	766.24	778.56
853.02	893.48	921.89	941.70	972.95	1011.11
1082.40	1108.91	1157.91	1181.65	1208.67	1223.39
1254.68	1258.23	1318.54	1325.44	1329.47	1370.85
1381.48	1452.73	1481.62	1501.67	1518.77	1861.62
2948.55	3048.70	3074.65	3088.90	3096.36	3122.71
3142.05	3594.26	3847.45			

- Conformer (6); ZPE = 31902.660 cm^{-1}:

49.99	77.56	203.30	258.28	311.64	418.05
538.99	587.97	598.95	646.56	749.81	798.43
865.04	900.25	920.61	942.95	972.42	989.27
1072.26	1097.59	1152.49	1173.68	1192.28	1221.80
1251.89	1280.37	1313.66	1327.55	1341.45	1374.58
1427.68	1449.67	1472.53	1484.00	1516.39	1867.31
2970.02	3039.28	3058.29	3064.89	3081.73	3126.07
3137.67	3632.39	3847.76			

- Conformer (7); ZPE = 31889.945 cm^{-1}:

49.33	66.60	182.53	190.93	336.56	441.26
510.39	594.25	631.93	661.81	740.82	764.74
859.33	887.98	934.01	951.14	1007.16	1029.12
1075.62	1110.33	1173.20	1195.27	1211.08	1236.38
1247.73	1290.09	1310.39	1323.73	1334.12	1373.98
1420.43	1449.36	1479.25	1497.12	1515.83	1874.08
2907.14	2948.53	3074.96	3083.26	3096.52	3120.07
3138.82	3606.93	3845.76			

- Conformer (8); ZPE = 31929.263 cm^{-1}:

40.72	90.52	205.13	267.87	295.37	412.83
536.99	584.03	601.67	635.82	786.59	798.63
857.35	900.24	912.68	941.17	973.94	987.26
1066.44	1094.59	1147.31	1169.45	1215.43	1223.29
1259.55	1283.22	1321.89	1344.85	1350.24	1385.26
1397.12	1449.73	1475.40	1487.02	1517.46	1866.38
2965.58	3053.48	3064.92	3070.12	3085.84	3120.32
3131.19	3629.52	3854.11			

- Conformer (9); ZPE = 31975.540 cm^{-1}:

54.76	60.79	195.08	281.00	303.12	403.40
572.98	614.97	632.10	701.47	767.03	807.01
824.49	884.70	930.77	935.24	959.86	1006.80
1048.52	1080.43	1131.56	1173.79	1204.95	1221.48
1254.17	1296.03	1315.53	1332.45	1346.82	1359.59
1412.97	1474.87	1478.32	1485.81	1501.01	1862.62
3039.42	3046.68	3074.24	3096.13	3110.10	3115.29
3130.27	3577.27	3845.20			

- Conformer (10); ZPE = 31954.358 cm^{-1}:

48.22	78.06	206.93	267.21	304.26	423.90
561.08	566.94	618.30	695.68	755.11	809.20
827.88	886.27	924.90	937.74	953.53	985.83
1034.59	1081.31	1139.03	1180.46	1209.32	1237.29
1250.44	1294.86	1323.59	1332.66	1348.67	1374.82
1390.47	1468.55	1478.60	1487.10	1500.55	1868.53
3039.55	3046.75	3068.55	3100.90	3109.31	3114.26
3131.03	3593.70	3852.80			

- Conformer (11); ZPE = 31832.181 cm^{-1}:

20.85	78.81	170.75	183.40	308.96	415.07
525.48	576.88	592.40	650.70	726.07	794.78
863.90	898.21	911.77	941.09	976.24	1006.72
1063.97	1099.15	1162.01	1185.22	1215.35	1221.41
1252.63	1297.03	1315.26	1331.34	1339.78	1377.63
1437.06	1443.67	1479.88	1491.97	1522.41	1872.90
2966.78	3027.40	3045.58	3069.26	3072.86	3118.92
3133.66	3629.73	3849.46			

- Conformer (12); ZPE = 32044.482 cm^{-1}:

54.45	84.03	194.26	210.29	348.66	462.45
507.66	623.89	664.29	699.77	745.44	800.19
867.69	904.42	921.18	990.19	1007.14	1033.90
1034.88	1109.66	1149.88	1207.47	1214.42	1239.76
1250.59	1286.84	1320.69	1330.16	1342.85	1357.85
1417.76	1471.78	1486.91	1494.26	1509.95	1899.65
3005.70	3046.15	3067.02	3078.32	3108.25	3120.82
3135.44	3498.64	3783.36			

- Conformer (13); ZPE = 31934.236 cm^{-1}:

55.61	72.59	208.94	243.05	332.08	439.20
487.03	561.17	645.05	652.10	745.73	817.08
842.70	898.09	933.24	942.23	977.83	1015.47
1078.40	1112.76	1167.63	1195.57	1203.18	1225.30
1240.96	1287.67	1301.48	1327.03	1336.57	1371.40
1378.92	1465.95	1479.47	1498.49	1514.11	1893.03
2976.49	3016.16	3055.92	3073.23	3101.27	3112.02
3132.42	3574.15	3879.69			

- Conformer (14); ZPE = 31915.744 cm^{-1}:

54.09	73.89	194.31	282.84	311.40	436.40
503.71	562.68	622.61	685.59	763.40	792.94
836.25	899.10	932.16	941.46	961.82	991.45
1052.21	1107.31	1173.23	1179.08	1197.92	1218.58
1260.71	1295.04	1306.50	1317.06	1344.14	1373.68
1379.88	1477.32	1485.05	1496.42	1510.15	1890.82
2998.11	3021.16	3050.79	3054.82	3105.59	3107.24
3122.20	3576.34	3884.05			

- Conformer (15); ZPE = 31915.171 cm^{-1}:

48.39	76.42	164.41	237.24	316.47	405.66
487.56	587.97	622.11	740.83	760.76	791.77
849.29	878.26	926.90	949.57	979.50	989.55
1089.52	1102.64	1139.72	1163.41	1211.55	1233.33
1246.03	1288.45	1295.43	1308.00	1329.52	1363.54
1392.27	1450.48	1468.56	1489.48	1520.01	1887.65
3004.29	3033.04	3079.69	3086.15	3094.06	3129.73
3145.59	3573.31	3892.25			

- Conformer (16); ZPE = 31875.033 cm^{-1}:

48.17	107.43	148.39	238.03	317.04	424.57
465.80	583.51	650.60	721.08	764.03	778.43
821.20	873.60	919.56	933.34	950.26	999.90
1038.57	1075.40	1149.53	1174.43	1198.74	1201.22
1249.41	1270.56	1300.98	1316.57	1329.02	1364.17
1403.32	1468.27	1480.40	1488.87	1506.99	1905.44
3048.63	3062.02	3066.89	3073.62	3101.99	3109.49
3128.89	3594.15	3897.52			

7.5 Cartesian coordinates of Pro conformers

Each line starting by an asterisk contains the relative energy of the corresponding conformer and the dihedral angle for each of the target torsions (separated by underscores). Cartesian coordinates are given in Å.

```
17
 * E =  +0.000 kcal/mol ; (1) 359_121_039_319_235
 C    +0.00000    +0.00000    +0.00000
 C    +0.00000    +0.00000    +1.53552
 C    +1.49659    +0.00000    +1.85007
 C    +2.03941    -0.97494    +0.80888
 N    +1.25603    -0.65683    -0.39998
 C    -0.05550    +1.44247    -0.52889
 O    +0.97209    +1.78905    -1.29504
 H    +1.55690    +1.00992    -1.32642
 O    -0.95588    +2.18374    -0.26211
 H    -0.87981    -0.50700    -0.40851
 H    -0.55071    +0.85220    +1.93657
 H    -0.46211    -0.92016    +1.90777
 H    +1.91673    +0.99924    +1.68408
 H    +1.73059    -0.30113    +2.87257
 H    +3.10930    -0.85289    +0.61989
 H    +1.86726    -2.00887    +1.13912
 H    +1.09735    -1.46821    -0.97913
17
 * E =  +0.684 kcal/mol ; (2) 356_137_323_020_241
 C    +0.00000    +0.00000    +0.00000
 C    +0.00000    +0.00000    +1.55154
 C    +1.49281    +0.00000    +1.92765
 C    +2.17130    +0.51720    +0.65649
 N    +1.39602    -0.05646    -0.45183
 C    -0.67632    +1.26005    -0.55907
 O    -0.03157    +1.82014    -1.57848
 H    +0.78716    +1.30946    -1.70382
 O    -1.70624    +1.68503    -0.12611
 H    -0.56253    -0.84290    -0.41391
 H    -0.51897    +0.88932    +1.91709
 H    -0.52642    -0.86665    +1.95413
 H    +1.70815    +0.61157    +2.80656
 H    +1.83844    -1.01815    +2.13744
```

H	+2.08875	+1.61087	+0.60004
H	+3.22773	+0.25656	+0.56635
H	+1.67684	−1.01640	−0.60947

17

∗ E = +1.176 kcal/mol ; (3) 181_297_321_019_121

C	+0.00000	+0.00000	+0.00000
C	+0.00000	+0.00000	+1.55466
C	+1.49323	+0.00000	+1.93318
C	+2.17547	+0.48732	+0.65891
N	+1.38876	−0.18293	−0.37525
C	−0.57259	+1.29933	−0.54522
O	−1.85509	+1.47360	−0.19195
H	−2.16164	+2.30216	−0.57732
O	+0.02441	+2.08487	−1.22663
H	−0.62986	−0.80506	−0.39788
H	−0.51399	+0.88590	+1.93674
H	−0.52766	−0.86961	+1.94846
H	+1.70759	+0.62931	+2.79928
H	+1.82733	−1.01658	+2.15698
H	+2.11055	+1.58634	+0.58581
H	+3.22754	+0.19874	+0.59274
H	+1.56714	+0.20320	−1.29527

17

∗ E = +1.313 kcal/mol ; (4) 180_290_035_320_120

C	+0.00000	+0.00000	+0.00000
C	+0.00000	+0.00000	+1.54487
C	+1.49491	+0.00000	+1.86346
C	+2.02795	−0.96027	+0.79957
N	+1.19602	−0.73236	−0.39382
C	+0.02907	+1.42537	−0.51960
O	−1.12381	+2.06882	−0.28690
H	−1.04266	+2.96299	−0.63710
O	+0.95874	+1.94203	−1.07428
H	−0.91719	−0.45963	−0.38906
H	−0.55774	+0.84138	+1.96093
H	−0.46341	−0.92538	+1.90093
H	+1.91263	+1.00195	+1.70859
H	+1.71911	−0.31270	+2.88573
H	+3.08458	−0.80709	+0.56613
H	+1.91274	−1.99857	+1.13668

H +1.70358 −0.19088 −1.08249
17
∗ E = +2.406 kcal/mol ; (5) 177_143_330_005_122
C +0.00000 +0.00000 +0.00000
C +0.00000 +0.00000 +1.53952
C +1.50114 +0.00000 +1.91804
C +2.22741 +0.12607 +0.57400
N +1.30403 −0.53502 −0.34446
C −0.26871 +1.40812 −0.53890
O +0.43516 +1.67446 −1.65374
H +0.18545 +2.55545 −1.95429
O −1.04284 +2.19241 −0.07012
H −0.80157 −0.62508 −0.41297
H −0.54111 +0.86791 +1.92019
H −0.49850 −0.89537 +1.91342
H +1.76102 +0.81012 +2.60214
H +1.77107 −0.94312 +2.39864
H +2.37387 +1.19134 +0.31880
H +3.20546 −0.36101 +0.56258
H +1.53829 −0.37913 −1.31678
17
∗ E = +3.191 kcal/mol ; (6) 184_188_042_322_232
C +0.00000 +0.00000 +0.00000
C +0.00000 +0.00000 +1.53226
C +1.49139 +0.00000 +1.87520
C +2.05755 −0.92069 +0.80046
N +1.32618 −0.50285 −0.39582
C −0.12166 +1.40661 −0.56457
O −0.29781 +1.38662 −1.89364
H −0.29725 +2.29770 −2.20740
O −0.04221 +2.42383 +0.06403
H −0.81512 −0.60269 −0.41932
H −0.54060 +0.85590 +1.93918
H −0.46937 −0.91922 +1.89694
H +1.89869 +1.00904 +1.75258
H +1.70348 −0.34398 +2.88929
H +3.13446 −0.79776 +0.65303
H +1.86623 −1.97214 +1.07075
H +1.28296 −1.20296 −1.12017
17

∗ E = +3.660 kcal/mol ; (7) 183_315_025_001_235
C +0.00000 +0.00000 +0.00000
C +0.00000 +0.00000 +1.53900
C +1.50549 +0.00000 +1.90028
C +2.22864 +0.03772 +0.54219
N +1.24151 +0.66403 −0.32347
C −1.18045 +0.67611 −0.64942
O −2.33766 +0.32681 −0.06244
H −3.05409 +0.75210 −0.54698
O −1.12578 +1.40346 −1.59793
H −0.02895 −1.05246 −0.35094
H −0.49168 +0.90343 +1.90783
H −0.54319 −0.85677 +1.94052
H +1.75543 +0.88660 +2.48657
H +1.79390 −0.87509 +2.48622
H +3.15438 +0.61789 +0.56469
H +2.47683 −0.98989 +0.22189
H +1.46265 +0.64714 −1.31017
17
∗ E = +3.706 kcal/mol ; (8) 178_047_041_324_233
C +0.00000 +0.00000 +0.00000
C +0.00000 +0.00000 +1.54306
C +1.48807 +0.00000 +1.90444
C +2.07759 −0.87419 +0.80446
N +1.35256 −0.42145 −0.38138
C −0.26489 +1.37729 −0.58367
O +0.47230 +2.33126 +0.00649
H +0.28282 +3.16613 −0.43515
O −1.03402 +1.61235 −1.47248
H −0.77996 −0.65207 −0.40762
H −0.54962 +0.84545 +1.96241
H −0.47282 −0.92043 +1.89915
H +1.89362 +1.01297 +1.82668
H +1.68443 −0.38386 +2.90755
H +3.15390 −0.73003 +0.67348
H +1.90019 −1.93806 +1.03391
H +1.35327 −1.08511 −1.14099
17
∗ E = +3.833 kcal/mol ; (9) 185_207_321_025_242
C +0.00000 +0.00000 +0.00000

C +0.00000 +0.00000 +1.54928
C +1.48908 +0.00000 +1.92926
C +2.13642 +0.64152 +0.69969
N +1.40205 +0.09965 −0.44619
C −0.69178 +1.22802 −0.56803
O −1.21535 +0.99774 −1.77872
H −1.56353 +1.83151 −2.11413
O −0.73301 +2.30222 −0.03651
H −0.46627 −0.88651 −0.43637
H −0.48531 +0.91045 +1.91052
H −0.54127 −0.85769 +1.95313
H +1.68702 +0.54047 +2.85787
H +1.85980 −1.02456 +2.04879
H +1.99054 +1.72839 +0.72224
H +3.20574 +0.44434 +0.59508
H +1.74812 −0.82387 −0.67380
17
* E = +3.930 kcal/mol ; (10) 179_065_321_030_241
C +0.00000 +0.00000 +0.00000
C +0.00000 +0.00000 +1.55949
C +1.48878 +0.00000 +1.92707
C +2.10963 +0.74028 +0.74026
N +1.37862 +0.25903 −0.43314
C −0.92595 +1.07118 −0.53928
O −0.49667 +2.30610 −0.23113
H −1.11652 +2.93799 −0.61131
O −1.93760 +0.87165 −1.14945
H −0.36291 −0.94486 −0.41007
H −0.47726 +0.91061 +1.93437
H −0.54798 −0.85321 +1.96269
H +1.68493 +0.47425 +2.89176
H +1.87551 −1.02477 +1.96722
H +1.94360 +1.81988 +0.83956
H +3.18218 +0.57319 +0.61724
H +1.79083 −0.59977 −0.77199
17
* E = +4.243 kcal/mol ; (11) 182_179_335_039_233
C +0.00000 +0.00000 +0.00000
C +0.00000 +0.00000 +1.52211
C +1.49690 +0.00000 +1.83182

C	+2.06422	+0.94496	+0.76360
N	+1.10226	+0.89912	−0.35050
C	−1.28479	+0.52207	−0.60789
O	−1.22025	+0.51641	−1.94926
H	−2.04196	+0.88784	−2.28887
O	−2.24909	+0.89838	−0.00658
H	+0.14994	−1.02415	−0.38295
H	−0.47929	+0.91841	+1.87633
H	−0.54270	−0.84830	+1.94265
H	+1.72939	+0.32256	+2.84870
H	+1.90328	−1.00910	+1.69937
H	+2.13163	+1.96953	+1.14836
H	+3.06908	+0.65001	+0.44364
H	+1.52232	+0.67019	−1.23831

17

∗ E = +4.809 kcal/mol ; (12) 357_156_022_003_114

C	+0.00000	+0.00000	+0.00000
C	+0.00000	+0.00000	+1.53423
C	+1.50734	+0.00000	+1.88981
C	+2.22800	+0.07828	+0.52183
N	+1.25368	+0.63488	−0.42326
C	−1.19940	+0.65036	−0.66190
O	−0.95892	+1.08269	−1.90751
H	−0.02628	+0.93291	−2.11407
O	−2.26973	+0.75976	−0.14678
H	+0.03288	−1.03762	−0.36711
H	−0.50592	+0.89873	+1.89813
H	−0.53880	−0.85626	+1.94263
H	+1.75926	+0.86643	+2.50698
H	+1.80369	−0.88995	+2.44960
H	+3.13866	+0.68030	+0.53627
H	+2.49719	−0.92482	+0.17236
H	+1.17695	+1.63447	−0.24109

17

∗ E = +5.663 kcal/mol ; (13) 001_297_320_021_120

C	+0.00000	+0.00000	+0.00000
C	+0.00000	+0.00000	+1.55739
C	+1.49353	+0.00000	+1.93363
C	+2.16487	+0.52393	+0.66924
N	+1.39136	−0.14370	−0.37709

```
 C    −0.61650    +1.28780    −0.54739
 O    −1.90985    +1.48532    −0.23029
 H    −2.24195    +0.76890    +0.31688
 O    −0.02694    +2.07684    −1.22041
 H    −0.59854    −0.83105    −0.40003
 H    −0.49734    +0.89617    +1.94336
 H    −0.52668    −0.86652    +1.96211
 H    +1.70380    +0.60828    +2.81530
 H    +1.83270    −1.02053    +2.12963
 H    +2.07743    +1.62200    +0.61576
 H    +3.22187    +0.25612    +0.59831
 H    +1.55380    +0.27264    −1.28728
17
 * E =  +5.760 kcal/mol ; (14) 358_288_036_320_119
 C    +0.00000    +0.00000    +0.00000
 C    +0.00000    +0.00000    +1.54869
 C    +1.49492    +0.00000    +1.86791
 C    +2.02590    −0.95608    +0.79996
 N    +1.20907    −0.70318    −0.39748
 C    −0.00434    +1.43460    −0.51993
 O    −1.16442    +2.09532    −0.36292
 H    −1.83017    +1.52742    +0.03321
 O    +0.93814    +1.96805    −1.02157
 H    −0.90282    −0.49370    −0.39108
 H    −0.54562    +0.84631    +1.97611
 H    −0.46594    −0.92207    +1.91043
 H    +1.91050    +1.00250    +1.71464
 H    +1.71639    −0.31588    +2.88969
 H    +3.08564    −0.81314    +0.57600
 H    +1.89454    −1.99655    +1.12417
 H    +1.71631    −0.12321    −1.05557
17
 * E =  +8.073 kcal/mol ; (15) 008_195_345_349_125
 C    +0.00000    +0.00000    +0.00000
 C    +0.00000    +0.00000    +1.52735
 C    +1.49827    +0.00000    +1.90693
 C    +2.24936    −0.27312    +0.58934
 N    +1.20392    −0.77069    −0.31284
 C    +0.13604    +1.42247    −0.54356
 O    −0.16837    +1.57455    −1.85022
```

H	−0.55299	+0.77109	−2.20732
O	+0.56614	+2.34061	+0.08654
H	−0.88535	−0.48700	−0.42947
H	−0.53275	+0.86337	+1.92896
H	−0.49156	−0.90982	+1.87823
H	+1.79688	+0.96347	+2.32250
H	+1.71038	−0.76989	+2.65140
H	+2.71008	+0.65600	+0.22415
H	+3.03504	−1.02570	+0.69340
H	+1.47376	−0.72281	−1.28826

17

∗ E = +9.450 kcal/mol ; (16) 355_277_009_332_242

C	+0.00000	+0.00000	+0.00000
C	+0.00000	+0.00000	+1.54756
C	+1.50299	+0.00000	+1.85899
C	+2.15319	−0.70158	+0.63771
N	+1.10288	−0.85987	−0.38546
C	+0.25082	+1.43006	−0.46665
O	−0.84523	+2.21775	−0.50429
H	−1.63838	+1.71572	−0.30545
O	+1.32718	+1.87572	−0.71129
H	−0.93990	−0.36591	−0.42938
H	−0.53105	+0.84688	+1.99301
H	−0.47715	−0.92209	+1.89486
H	+1.86979	+1.02689	+1.93545
H	+1.72782	−0.50399	+2.80110
H	+2.96175	−0.08868	+0.23365
H	+2.56661	−1.68101	+0.89330
H	+0.77962	−1.81734	−0.41111

8 Val

8.1 Reference Z-matrix for Val

Bond distances are given in Å; bond and dihedral angles, in degrees.

h						
o	1	oh2				
c	2	co3	1	coh3		
c	3	cc4	2	cco4	1	dih4
n	4	nc5	3	ncc5	2	dih5
h	5	hn6	4	hnc6	3	dih6
o	3	oc7	2	oco7	4	dih7
c	4	cc8	3	ccc8	5	dih8
h	4	hc9	3	hcc9	5	dih9
h	5	hn10	4	hnc10	6	dih10
c	8	cc11	4	ccc11	3	dih11
h	8	hc12	4	hcc12	11	dih12
c	8	cc13	4	ccc13	11	dih13
h	11	hc14	8	hcc14	4	dih14
h	11	hc15	8	hcc15	14	dih15
h	11	hc16	8	hcc16	14	dih16
h	13	hc17	8	hcc17	4	dih17
h	13	hc18	8	hcc18	17	dih18
h	13	hc19	8	hcc19	17	dih19

oh2	0.96854469
co3	1.36192474
coh3	111.8246876
cc4	1.51274463
cco4	110.12713136
dih4	182.10723109
nc5	1.47456424
ncc5	104.72758912
dih5	55.71664058
hn6	1.00230174
hnc6	113.21877216
dih6	184.46418133
oc7	1.2012257
oco7	121.58390419
dih7	179.87048945

cc8	1.53604942
ccc8	110.75372346
dih8	120.51425013
hc9	1.09026534
hcc9	107.12576837
dih9	239.77841254
hn10	1.00358771
hnc10	111.96145056
dih10	127.25905083
cc11	1.53982182
ccc11	111.22362097
dih11	192.17604477
hc12	1.08197433
hcc12	107.31387701
dih12	119.24532727
cc13	1.5411751
ccc13	110.3080664
dih13	238.30189476
hc14	1.08404536
hcc14	109.73201352
dih14	184.33580924
hc15	1.08674564
hcc15	110.78905004
dih15	118.9918558
hc16	1.08237514
hcc16	111.43138163
dih16	240.74185585
hc17	1.08421321
hcc17	110.14149816
dih17	181.15263754
hc18	1.08020276
hcc18	110.86268872
dih18	119.73802805
hc19	1.08635425
hcc19	110.32626483
dih19	240.88173488

8.2 Reference energy and target torsions for Val

- Reference M08-HX/MG3S energy: -402.3968924 hartree
- Target torsions:
  - torsion1: dih4
  - torsion2: dih5
  - torsion3: dih6
  - torsion4: dih11

8.3 Properties of Val conformers

Table S8: Relative energy (kcal mol^{-1}), rotational constants (B1 to B3, GHz) and dipolar moment (Debye) for the conformers of Val located at the M08-HX/MG3S level.

Conformer	Rel. Energy	B1	B2	B3	Dip. Mom.
1	0.000	1.343	1.462	2.979	1.400
2	0.018	1.260	1.501	3.088	5.139
3	0.453	1.341	1.490	3.010	5.364
4	0.460	1.474	1.837	2.457	5.132
5	0.938	1.242	1.620	2.813	1.386
6	1.084	1.446	1.845	2.395	1.477
7	1.280	1.357	1.472	2.980	2.378
8	1.428	1.362	1.708	2.672	5.154
9	1.482	1.270	1.581	2.848	1.675
10	1.484	1.330	1.453	3.034	1.654
11	1.656	1.219	1.649	2.849	5.113
12	1.830	1.458	1.835	2.402	2.172
13	1.878	1.396	1.767	2.580	2.006
14	2.036	1.242	1.492	3.189	2.074
15	2.083	1.467	1.804	2.423	1.584
16	2.117	1.120	1.591	3.024	4.973
17	2.186	1.240	1.614	2.827	2.110
18	2.608	1.356	1.468	3.010	2.306
19	2.715	1.257	1.593	2.841	2.845

20	2.773	1.149	1.551	3.087	2.251
21	2.777	1.480	1.799	2.433	2.424
22	2.945	1.359	1.631	2.753	1.275
23	2.975	1.340	1.668	2.762	2.181
24	4.762	1.344	1.464	2.965	3.361
25	5.317	1.441	1.858	2.378	3.397
26	5.425	1.226	1.635	2.801	3.299
27	5.834	1.462	1.850	2.395	4.382
28	6.040	1.363	1.471	2.966	4.480
29	6.514	1.235	1.627	2.810	4.427
30	7.289	1.267	1.545	2.862	3.778

8.4 Vibrational frequencies of Val conformers

Harmonic vibrational zero point energy (ZPE) and frequencies for each conformer in cm^{-1}.

- Conformer (1); ZPE = 36285.075 cm^{-1}:

47.97	82.48	179.52	207.11	229.77	261.69
278.28	296.59	340.46	393.76	447.41	568.36
607.18	660.44	731.98	846.94	867.04	920.53
934.85	969.20	991.21	1074.24	1100.28	1168.55
1174.60	1211.88	1254.80	1270.48	1351.50	1363.64
1370.68	1392.88	1408.13	1429.66	1474.89	1485.38
1494.97	1504.61	1673.58	1858.39	3030.11	3037.12
3047.00	3054.82	3105.44	3113.71	3116.55	3124.90
3545.39	3625.35	3843.84			

- Conformer (2); ZPE = 36396.274 cm^{-1}:

61.65	87.49	186.91	226.67	248.19	271.69
290.34	313.56	370.16	414.11	531.05	532.21
608.35	748.72	802.90	830.11	857.89	909.86
927.41	969.65	1012.82	1050.52	1094.71	1168.16
1203.36	1219.41	1244.75	1280.73	1352.16	1361.09
1396.72	1398.24	1415.61	1429.19	1474.00	1487.31
1497.83	1502.13	1656.23	1887.57	3022.82	3024.44
3036.51	3062.83	3095.61	3101.03	3118.13	3123.92
3552.76	3645.24	3687.81			

- Conformer (3); ZPE = 36360.227 cm^{-1}:

26.48	85.34	179.60	200.54	227.70	236.72
280.76	312.93	370.51	402.83	490.91	596.74
634.73	740.02	821.94	839.10	845.53	925.16
929.38	967.70	982.88	1047.58	1094.75	1170.93
1203.02	1215.58	1261.44	1287.10	1353.18	1364.71
1382.25	1402.56	1421.33	1431.21	1471.56	1487.85
1493.98	1498.70	1654.71	1887.20	3027.61	3029.25
3046.41	3062.28	3099.31	3104.51	3118.12	3119.24
3569.72	3652.81	3664.02			

- Conformer (4); ZPE = 36423.934 cm^{-1}:

64.12	104.21	188.45	224.96	254.48	264.92
287.54	343.14	353.18	428.70	462.84	553.11
667.37	755.79	790.98	824.83	864.77	922.18
938.40	957.46	974.63	1097.90	1108.90	1128.79
1202.39	1211.97	1266.98	1309.50	1335.55	1363.80
1378.40	1401.29	1417.97	1430.28	1476.32	1482.70
1495.07	1500.65	1653.20	1886.79	2997.27	3032.49
3038.84	3042.27	3107.91	3120.18	3121.34	3128.42
3552.69	3637.23	3694.70			

- Conformer (5); ZPE = 36251.379 cm^{-1}:

66.58	75.25	175.40	224.73	239.04	248.86
274.72	301.96	359.89	374.81	419.82	528.78
611.14	672.28	764.71	845.92	866.47	925.25
928.51	962.83	1004.64	1066.25	1137.43	1163.15
1173.64	1197.08	1247.37	1283.90	1340.38	1354.15
1374.46	1390.65	1411.36	1428.62	1475.09	1481.36
1494.83	1504.28	1661.39	1859.21	3007.92	3029.83
3032.16	3056.23	3103.98	3107.35	3118.24	3141.09
3534.13	3620.29	3835.35			

- Conformer (6); ZPE = 36340.369 cm^{-1}:

44.39	98.31	183.53	223.28	254.23	277.84
294.54	300.86	351.64	391.02	447.53	533.28
618.35	678.52	752.59	829.16	861.13	927.51
936.32	968.43	975.26	1088.53	1138.95	1151.55
1175.51	1209.87	1248.48	1287.77	1325.97	1351.37
1377.32	1399.75	1416.92	1436.38	1475.09	1484.24
1497.67	1505.17	1668.04	1855.18	3032.86	3034.84
3043.69	3065.77	3106.97	3111.59	3120.92	3126.01
3540.02	3621.00	3835.57			

- Conformer (7); ZPE = 36199.956 cm^{-1}:

46.52	80.86	176.05	202.53	217.81	241.30
273.87	301.21	344.52	394.24	453.52	574.79
592.93	663.91	728.22	803.17	864.61	884.59
928.19	971.20	974.35	1056.63	1134.08	1163.64
1175.22	1198.66	1257.06	1295.10	1318.34	1358.52
1376.70	1398.07	1415.31	1449.47	1473.25	1485.81
1494.65	1501.09	1634.17	1864.77	3025.94	3027.09
3034.53	3042.08	3099.95	3108.97	3117.16	3118.97
3555.18	3653.11	3848.03			

- Conformer (8); ZPE = 36386.150 cm^{-1}:

26.96	99.52	208.98	227.13	237.87	242.59
269.02	328.51	365.56	447.43	478.96	564.77
584.30	758.35	804.40	831.65	861.91	920.45
927.69	971.55	994.68	1081.07	1125.13	1147.44
1208.23	1214.88	1244.71	1291.83	1342.03	1361.27
1396.82	1399.02	1413.72	1435.09	1472.94	1486.12
1496.02	1506.43	1653.87	1882.26	3006.85	3029.53
3039.04	3042.98	3101.72	3110.43	3122.52	3142.03
3548.78	3640.98	3676.28			

- Conformer (9); ZPE = 36239.897 cm^{-1}:

52.49	79.50	165.19	215.06	223.20	267.77
271.08	304.56	352.49	384.24	431.63	540.08
597.55	641.22	780.95	829.72	856.94	919.05
925.88	959.27	985.54	1066.95	1128.53	1152.12
1193.67	1201.97	1247.27	1300.90	1347.03	1358.35
1377.47	1391.91	1396.05	1415.06	1475.75	1479.87
1494.90	1502.68	1656.74	1867.07	3013.54	3030.34
3033.94	3066.56	3104.36	3107.79	3119.04	3141.10
3544.39	3632.71	3848.31			

- Conformer (10); ZPE = 36257.692 cm^{-1}:

40.61	73.95	177.25	209.73	226.80	265.05
285.62	301.77	334.00	391.22	472.03	563.95
608.90	649.03	732.64	838.96	844.17	914.09
930.18	965.33	978.40	1064.50	1095.68	1172.87
1198.20	1212.48	1239.14	1283.89	1360.49	1361.68
1376.81	1392.49	1396.34	1412.82	1473.47	1483.73
1495.44	1503.39	1667.83	1858.93	3027.12	3036.94
3051.93	3056.59	3100.68	3114.56	3116.86	3122.73
3556.17	3635.53	3842.40			

- Conformer (11); ZPE = 36379.162 cm^{-1}:

34.42	72.34	178.40	210.29	229.09	274.06
315.04	342.55	377.13	395.23	438.25	555.80
678.94	740.70	777.20	839.67	867.53	924.40
928.22	959.44	978.97	1083.86	1106.88	1149.36
1189.80	1222.84	1270.74	1310.93	1326.31	1352.40
1377.39	1402.59	1419.48	1420.88	1470.09	1483.43
1493.40	1502.19	1655.83	1892.61	3007.91	3025.35
3033.89	3041.53	3109.10	3115.13	3119.15	3138.02
3563.43	3648.46	3707.66			

- Conformer (12); ZPE = 36240.900 cm^{-1}:

17.91	89.02	179.07	216.99	240.25	245.44
281.84	310.70	342.43	405.69	448.07	529.87
609.43	676.58	750.60	810.54	879.37	882.25
924.86	965.20	974.86	1107.80	1126.32	1172.78
1181.15	1202.40	1245.65	1292.35	1327.85	1343.03
1369.34	1400.43	1417.21	1464.25	1473.92	1483.38
1495.00	1496.50	1642.48	1861.69	3001.90	3033.02
3034.65	3038.18	3106.90	3115.60	3118.35	3121.51
3547.16	3639.35	3840.72			

- Conformer (13); ZPE = 36234.215 cm^{-1}:

55.07	93.63	189.78	215.77	227.51	249.25
268.12	322.24	333.54	433.69	460.39	542.48
584.04	640.93	738.37	807.74	865.00	884.75
923.31	963.49	984.55	1099.84	1139.55	1157.56
1189.55	1204.56	1230.58	1307.86	1325.47	1348.03
1360.66	1391.95	1411.15	1455.11	1477.94	1486.91
1497.07	1502.39	1634.39	1860.37	2960.23	3021.88
3031.22	3039.31	3105.90	3110.10	3126.12	3134.40
3568.04	3664.10	3842.54			

- Conformer (14); ZPE = 36260.977 cm^{-1}:

43.33	94.34	193.08	219.93	234.84	266.36
283.04	317.46	347.31	404.48	482.98	533.46
605.48	641.32	735.50	819.70	866.80	899.40
927.20	972.28	976.65	1059.36	1126.58	1169.68
1187.63	1207.03	1233.56	1315.84	1331.87	1354.77
1371.88	1393.28	1410.25	1437.68	1477.51	1483.69
1500.40	1505.91	1652.36	1866.32	2924.70	3024.89
3041.39	3055.89	3098.77	3113.64	3119.48	3125.91
3567.71	3652.66	3846.39			

- Conformer (15); ZPE = 36247.241 cm^{-1}:

38.52	78.97	181.22	214.99	230.48	257.10
263.92	298.12	347.61	403.67	452.55	549.23
595.94	648.30	751.64	827.05	838.02	916.75
928.62	959.72	971.19	1084.65	1122.58	1149.01
1197.58	1212.97	1237.79	1295.37	1331.75	1361.58
1385.79	1396.96	1403.21	1419.49	1472.41	1485.45
1496.11	1499.16	1667.70	1856.86	3028.38	3037.04
3042.64	3068.99	3104.17	3116.65	3117.93	3118.83
3553.23	3632.25	3844.31			

- Conformer (16); ZPE = 36427.377 cm^{-1}:

64.62	84.09	206.27	217.15	261.69	282.90
341.07	351.98	378.15	416.88	461.99	537.47
600.18	747.19	787.07	841.01	865.54	921.82
927.02	969.55	1001.90	1087.11	1129.31	1167.23
1194.54	1209.78	1229.06	1284.29	1333.16	1374.22
1387.56	1392.07	1411.23	1424.48	1475.70	1486.82
1493.41	1508.81	1659.58	1888.42	3005.25	3020.50
3023.90	3032.42	3098.84	3102.95	3121.89	3156.83
3538.57	3633.76	3717.53			

- Conformer (17); ZPE = 36230.217 cm^{-1}:

58.92	68.39	170.03	214.62	227.79	243.22
287.59	313.70	358.72	378.68	419.39	521.88
618.98	670.24	770.75	822.07	864.35	901.94
920.80	959.39	977.48	1119.93	1138.01	1151.35
1174.61	1193.21	1237.31	1284.57	1342.17	1347.96
1367.31	1395.97	1413.41	1458.89	1472.08	1487.21
1492.02	1505.06	1637.47	1866.32	3016.42	3029.08
3032.19	3047.46	3104.44	3108.24	3117.31	3120.57
3546.34	3646.86	3837.72			

- Conformer (18); ZPE = 36231.792 cm^{-1}:

55.78	57.91	178.70	215.36	243.51	252.13
284.19	312.47	340.09	387.61	470.23	575.73
595.11	654.85	733.46	806.22	861.23	878.03
922.70	970.49	977.08	1053.17	1119.51	1138.31
1193.08	1214.22	1243.90	1289.18	1334.67	1360.13
1374.31	1394.95	1410.99	1437.93	1472.30	1487.42
1493.78	1501.53	1648.57	1863.85	3028.05	3030.45
3042.35	3053.83	3100.63	3105.27	3117.02	3118.52
3561.73	3654.40	3846.66			

- Conformer (19); ZPE = 36201.695 cm^{-1}:

53.81	69.45	164.75	214.26	223.96	248.09
278.92	320.07	353.80	384.49	425.09	534.76
588.12	640.58	775.96	816.45	850.40	904.78
923.70	960.62	975.08	1094.45	1130.02	1135.73
1188.59	1198.53	1245.17	1287.06	1347.08	1364.75
1368.93	1393.70	1412.48	1420.75	1472.69	1485.06
1493.02	1502.42	1645.97	1879.26	3015.26	3028.13
3031.88	3062.12	3103.66	3107.57	3118.00	3118.92
3548.93	3645.41	3850.72			

- Conformer (20); ZPE = 36315.827 cm^{-1}:

61.40	97.83	197.51	225.35	260.39	274.23
294.68	329.35	370.70	400.07	470.87	490.09
600.05	640.51	759.98	826.83	881.75	919.99
926.75	961.80	972.36	1104.29	1138.63	1150.75
1189.10	1197.03	1247.26	1308.55	1316.33	1341.40
1378.05	1390.28	1407.30	1447.33	1478.14	1491.15
1495.36	1511.36	1656.18	1876.97	2927.60	3019.55
3028.07	3052.89	3100.02	3106.01	3123.84	3137.30
3559.11	3642.80	3846.50			

- Conformer (21); ZPE = 36241.997 cm^{-1}:

51.56	94.42	184.47	224.07	236.03	259.04
275.79	310.93	344.28	403.31	452.36	546.46
582.97	649.51	754.48	802.26	862.46	888.78
921.57	963.63	974.28	1098.06	1113.88	1144.19
1194.76	1203.75	1246.05	1286.97	1325.55	1358.40
1370.98	1400.02	1416.31	1440.75	1471.44	1484.61
1495.31	1496.75	1649.66	1864.98	3008.22	3031.94
3037.41	3059.34	3105.36	3115.56	3119.26	3120.51
3552.64	3642.92	3845.75			

- Conformer (22); ZPE = 36312.400 cm^{-1}:

30.69	107.33	196.44	222.55	236.26	258.72
287.24	308.22	331.89	390.27	462.21	528.49
590.68	672.42	758.53	814.60	851.07	925.09
928.82	971.70	979.62	1090.33	1125.68	1144.45
1176.26	1211.41	1234.71	1310.06	1339.36	1361.41
1392.10	1397.68	1407.83	1422.12	1481.23	1490.03
1500.52	1508.51	1658.72	1860.26	3030.90	3034.54
3041.99	3043.91	3109.14	3112.94	3126.00	3130.46
3548.93	3635.99	3844.51			

- Conformer (23); ZPE = 36266.114 cm^{-1}:

36.90	106.48	210.88	223.13	233.89	260.43
290.21	321.70	327.85	423.21	482.01	522.53
578.58	641.00	740.42	805.09	869.27	906.34
928.64	964.16	984.45	1098.17	1131.76	1151.55
1186.55	1208.98	1236.36	1315.24	1334.86	1356.28
1367.87	1391.61	1405.21	1449.16	1478.56	1490.09
1494.47	1509.07	1649.27	1867.23	2926.41	2999.31
3037.93	3040.85	3109.29	3118.39	3126.87	3136.85
3560.37	3650.54	3845.97			

- Conformer (24); ZPE = 36219.043 cm^{-1}:

56.69	85.31	173.48	184.35	224.07	263.85
277.61	298.84	342.78	396.18	448.00	500.15
595.15	654.29	732.54	842.43	875.54	920.89
933.51	972.14	990.82	1075.81	1102.07	1165.24
1172.93	1216.49	1252.24	1266.13	1329.94	1354.73
1367.38	1392.63	1408.95	1426.48	1474.60	1485.82
1495.96	1505.73	1670.60	1891.22	3005.33	3020.54
3033.71	3036.56	3111.62	3112.86	3118.66	3126.68
3541.05	3620.59	3886.93			

- Conformer (25); ZPE = 36290.682 cm^{-1}:

49.94	101.08	191.36	200.49	250.64	278.43
304.07	312.98	347.53	401.79	450.17	516.00
538.86	674.01	747.79	826.13	868.08	926.43
940.59	966.36	975.07	1086.51	1141.10	1153.51
1171.09	1210.79	1247.78	1286.95	1313.85	1347.41
1371.20	1398.99	1415.34	1425.49	1478.96	1488.49
1503.98	1506.48	1671.90	1887.56	3014.83	3020.33
3036.08	3047.25	3089.87	3111.89	3117.22	3126.99
3537.40	3614.82	3889.46			

- Conformer (26); ZPE = 36220.353 cm^{-1}:

62.88	75.99	188.31	218.93	225.73	264.46
288.74	309.60	364.81	375.33	422.31	502.82
538.44	691.29	738.42	850.32	877.96	924.81
931.55	961.42	1001.58	1066.56	1137.46	1164.98
1170.81	1197.06	1245.32	1285.87	1335.62	1355.95
1359.84	1391.38	1408.93	1417.24	1478.02	1482.23
1498.38	1505.53	1665.10	1890.06	2996.51	3010.99
3021.15	3031.02	3096.85	3104.23	3124.95	3146.67
3531.28	3615.03	3889.98			

- Conformer (27); ZPE = 36225.153 cm^{-1}:

47.92	100.48	184.51	211.00	246.61	275.02
294.27	324.12	342.56	409.72	452.26	510.53
536.65	679.26	749.71	801.85	873.18	885.27
926.47	956.37	973.26	1107.80	1122.70	1174.43
1194.13	1201.16	1242.48	1292.33	1319.04	1333.50
1366.69	1397.36	1415.47	1449.75	1475.27	1484.94
1497.70	1506.93	1642.78	1892.01	2975.19	3005.45
3020.38	3037.47	3094.09	3112.00	3115.87	3125.53
3544.11	3637.23	3885.52			

- Conformer (28); ZPE = 36135.958 cm^{-1}:

48.92	85.71	176.37	199.58	212.40	233.99
276.25	305.15	349.69	396.58	454.36	492.96
594.92	658.15	729.08	796.62	861.29	890.92
926.60	971.18	974.77	1057.09	1132.00	1165.64
1186.50	1202.01	1247.69	1290.62	1319.87	1346.79
1368.17	1397.56	1415.50	1432.94	1471.25	1484.14
1493.95	1499.96	1634.98	1896.02	2968.54	3002.40
3031.94	3037.39	3104.27	3111.95	3119.86	3123.40
3552.82	3654.10	3887.07			

- Conformer (29); ZPE = 36221.284 cm^{-1}:

61.77	70.70	178.20	217.64	249.16	270.24
295.15	320.48	362.49	380.97	421.48	517.83
536.41	690.89	749.28	824.33	876.36	899.48
919.99	954.91	974.44	1124.41	1136.19	1160.67
1174.78	1192.97	1236.42	1279.43	1335.67	1342.43
1358.94	1394.54	1412.48	1437.72	1474.01	1488.32
1494.81	1506.16	1637.62	1894.72	2974.93	3014.39
3026.80	3033.93	3097.25	3106.16	3119.84	3123.52
3546.25	3646.51	3898.53			

- Conformer (30); ZPE = 36130.787 cm^{-1}:

37.59	88.71	172.78	208.29	218.66	228.60
266.85	293.15	340.75	388.60	442.49	486.73
537.40	658.02	776.34	821.66	846.76	913.19
930.16	962.75	982.68	1062.40	1125.57	1143.73
1192.26	1216.20	1242.30	1300.94	1329.99	1344.50
1365.23	1393.42	1396.98	1417.75	1477.17	1480.46
1496.60	1502.94	1654.23	1895.06	2950.71	3034.48
3036.58	3065.30	3108.65	3110.91	3123.46	3143.48
3539.45	3630.52	3878.12			

8.5 Cartesian coordinates of Val conformers

Each line starting by an asterisk contains the relative energy of the corresponding conformer and the dihedral angle for each of the target torsions (separated by underscores). Cartesian coordinates are given in Å.

```
19
 * E =  +0.000 kcal/mol ; (1) 178_169_285_293
 H    +0.00000    +0.00000    +0.00000
 O    +0.00000    +0.00000    +0.96393
 C    +1.27621    +0.00000    +1.37895
 C    +1.39179    +0.04618    +2.89553
 N    +2.74025    -0.21550    +3.35419
 H    +2.95952    -1.20055    +3.27893
 O    +2.20291    -0.00671    +0.61809
 C    +0.88674    +1.42021    +3.39535
 H    +0.71828    -0.72060    +3.29789
 H    +3.40204    +0.26799    +2.75547
 C    +1.82080    +2.53866    +2.93653
 H    -0.10421    +1.57736    +2.95207
 C    +0.76110    +1.40638    +4.91472
 H    +1.41742    +3.51475    +3.21936
 H    +2.80021    +2.43536    +3.41626
 H    +1.96951    +2.53665    +1.85091
 H    +0.42051    +2.37871    +5.28268
 H    +0.04717    +0.64705    +5.24937
 H    +1.73220    +1.18438    +5.36667
19
 * E =  +0.018 kcal/mol ; (2) 357_016_091_296
 H    +0.00000    +0.00000    +0.00000
```

O	+0.00000	+0.00000	+0.97174
C	+1.27284	+0.00000	+1.35601
C	+2.28776	−0.07427	+0.20400
N	+1.62951	+0.25424	−1.05986
H	+1.68480	+1.24698	−1.25795
O	+1.60256	+0.02774	+2.50545
C	+3.54377	+0.74366	+0.54280
H	+2.57363	−1.13388	+0.15378
H	+2.04349	−0.23807	−1.83916
C	+3.22370	+2.23028	+0.68817
H	+3.88901	+0.37389	+1.51451
C	+4.62873	+0.51515	−0.50441
H	+4.11643	+2.78357	+0.99210
H	+2.89272	+2.66858	−0.26202
H	+2.45016	+2.40232	+1.44239
H	+5.54638	+1.04597	−0.23639
H	+4.87165	−0.54759	−0.60890
H	+4.31124	+0.88852	−1.48559

19

∗ E = +0.453 kcal/mol ; (3) 002_352_133_296

H	+0.00000	+0.00000	+0.00000
O	+0.00000	+0.00000	+0.97319
C	+1.27441	+0.00000	+1.34756
C	+2.28648	+0.04014	+0.19222
N	+1.60205	−0.11798	−1.09177
H	+1.90212	+0.56902	−1.77092
O	+1.61949	−0.00023	+2.49330
C	+3.06575	+1.36419	+0.29506
H	+2.99044	−0.78056	+0.37753
H	+1.74708	−1.03380	−1.49316
C	+2.14539	+2.56903	+0.09879
H	+3.45402	+1.38907	+1.31949
C	+4.23886	+1.39435	−0.67987
H	+2.70264	+3.49949	+0.23659
H	+1.71897	+2.59329	−0.91120
H	+1.31662	+2.56434	+0.81412
H	+4.83103	+2.30258	−0.53726
H	+4.89861	+0.53253	−0.53987
H	+3.90097	+1.39372	−1.72317

19

∗ E = +0.460 kcal/mol ; (4) 004_340_152_069

H	+0.00000	+0.00000	+0.00000
O	+0.00000	+0.00000	+0.97172
C	+1.27401	+0.00000	+1.35861
C	+2.28776	+0.09695	+0.21214
N	+1.64677	−0.29841	−1.04156
H	+2.08080	+0.15528	−1.83572
O	+1.59915	−0.03296	+2.50944
C	+2.79934	+1.55028	+0.10723
H	+3.13355	−0.54515	+0.48934
H	+1.69246	−1.29837	−1.19144
C	+3.67622	+1.92726	+1.29706
H	+3.42084	+1.57519	−0.80081
C	+1.64901	+2.54375	−0.05807
H	+4.07905	+2.93510	+1.16124
H	+3.09952	+1.90797	+2.22586
H	+4.51537	+1.23488	+1.41243
H	+2.03843	+3.54842	−0.24329
H	+0.98755	+2.27725	−0.88835
H	+1.04515	+2.58515	+0.85597

19

∗ E = +0.938 kcal/mol ; (5) 178_141_291_172

H	+0.00000	+0.00000	+0.00000
O	+0.00000	+0.00000	+0.96458
C	+1.27508	+0.00000	+1.37923
C	+1.41380	+0.05531	+2.89316
N	+2.49068	−0.79488	+3.36812
H	+2.26525	−1.77454	+3.24752
O	+2.20385	−0.02278	+0.62020
C	+1.67748	+1.51504	+3.31597
H	+0.46645	−0.27128	+3.33952
H	+3.32480	−0.61195	+2.81852
C	+1.62630	+1.62547	+4.83624
H	+2.69384	+1.75442	+2.96974
C	+0.69476	+2.48708	+2.66683
H	+1.92254	+2.62724	+5.16060
H	+0.60394	+1.44859	+5.19168
H	+2.28506	+0.89229	+5.30552
H	+0.85111	+3.49758	+3.05427
H	+0.80553	+2.52672	+1.57882

H −0.33890 +2.19680 +2.88838
19
∗ E = +1.084 kcal/mol ; (6) 178_148_289_058
H +0.00000 +0.00000 +0.00000
O +0.00000 +0.00000 +0.96449
C +1.27627 +0.00000 +1.37823
C +1.41041 +0.04489 +2.89471
N +2.58651 −0.66775 +3.35781
H +2.46705 −1.66708 +3.24589
O +2.20193 −0.02196 +0.61504
C +1.45050 +1.51233 +3.37992
H +0.51529 −0.42276 +3.31965
H +3.38497 −0.40714 +2.78772
C +0.21114 +2.29389 +2.95088
H +1.46596 +1.43538 +4.47393
C +2.72327 +2.22547 +2.92869
H +0.18000 +3.26114 +3.46000
H +0.22056 +2.48982 +1.87297
H −0.71231 +1.75321 +3.18059
H +2.72138 +3.25844 +3.28871
H +3.62033 +1.74031 +3.32238
H +2.79573 +2.25123 +1.83553
19
∗ E = +1.280 kcal/mol ; (7) 177_163_037_298
H +0.00000 +0.00000 +0.00000
O +0.00000 +0.00000 +0.96375
C +1.27624 +0.00000 +1.37534
C +1.38816 +0.06675 +2.88214
N +2.72624 −0.34166 +3.24926
H +3.39880 −0.00951 +2.56799
O +2.20248 −0.00193 +0.61472
C +0.95402 +1.48734 +3.33676
H +0.65720 −0.64330 +3.29328
H +2.98604 −0.01360 +4.16904
C +1.86567 +2.56677 +2.75983
H −0.06475 +1.63656 +2.95677
C +0.91626 +1.56389 +4.86044
H +1.53434 +3.55916 +3.07815
H +2.89406 +2.43135 +3.11302
H +1.88527 +2.55047 +1.66545

H +0.52648 +2.53209 +5.18741
H +0.28408 +0.77964 +5.28839
H +1.92075 +1.46450 +5.28854
19
* E = +1.428 kcal/mol ; (8) 358_012_095_073
H +0.00000 +0.00000 +0.00000
O +0.00000 +0.00000 +0.97240
C +1.27243 +0.00000 +1.35572
C +2.28681 −0.04332 +0.19832
N +1.59360 +0.22253 −1.06540
H +1.65501 +1.20431 −1.31219
O +1.59553 +0.00770 +2.50817
C +3.50231 +0.87246 +0.44793
H +2.63710 −1.08360 +0.17270
H +1.99412 −0.30689 −1.82747
C +4.45383 +0.31854 +1.50624
H +4.03803 +0.89677 −0.51215
C +3.06750 +2.29604 +0.79708
H +5.35048 +0.94262 +1.56890
H +3.97213 +0.29974 +2.48597
H +4.76959 −0.70096 +1.26158
H +3.93277 +2.96353 +0.83140
H +2.36387 +2.71395 +0.06739
H +2.58719 +2.31431 +1.78052
19
* E = +1.482 kcal/mol ; (9) 180_303_296_171
H +0.00000 +0.00000 +0.00000
O +0.00000 +0.00000 +0.96358
C +1.28154 +0.00000 +1.37967
C +1.37392 +0.00413 +2.89723
N +0.67840 +1.13089 +3.50104
H +1.09455 +2.01385 +3.23341
O +2.20418 −0.00501 +0.61590
C +0.82785 −1.31966 +3.45835
H +2.44176 +0.07629 +3.13492
H −0.29201 +1.14264 +3.20685
C +1.13013 −1.41398 +4.95041
H −0.26196 −1.30214 +3.31010
C +1.40901 −2.51522 +2.70697
H +0.66937 −2.30724 +5.38192

H	+2.21253	−1.48633	+5.11057
H	+0.76417	−0.53462	+5.48406
H	+1.06285	−3.45218	+3.15166
H	+1.12362	−2.52130	+1.65009
H	+2.50406	−2.50331	+2.75686

19

∗ E = +1.484 kcal/mol ; (10) 181_350_292_291

H	+0.00000	+0.00000	+0.00000
O	+0.00000	+0.00000	+0.96419
C	+1.28218	+0.00000	+1.36659
C	+1.44636	−0.03429	+2.88337
N	+0.23620	+0.15625	+3.65707
H	−0.12136	+1.09680	+3.55221
O	+2.19651	+0.00169	+0.59136
C	+2.14571	−1.36086	+3.25244
H	+2.14304	+0.77959	+3.11884
H	−0.49401	−0.47093	+3.34106
C	+1.21500	−2.54893	+3.01333
H	+3.01032	−1.44750	+2.58373
C	+2.62253	−1.32344	+4.69962
H	+1.74570	−3.49081	+3.17627
H	+0.37123	−2.52556	+3.71221
H	+0.81773	−2.56279	+1.99171
H	+3.11316	−2.26368	+4.96810
H	+3.33600	−0.50955	+4.86236
H	+1.77374	−1.17020	+5.37246

19

∗ E = +1.656 kcal/mol ; (11) 008_331_157_171

H	+0.00000	+0.00000	+0.00000
O	+0.00000	+0.00000	+0.97112
C	+1.27535	+0.00000	+1.35986
C	+2.27660	+0.20347	+0.21897
N	+1.69662	−0.32660	−1.01327
H	+2.11638	+0.08777	−1.83418
O	+1.60192	−0.10987	+2.50437
C	+2.57771	+1.70752	+0.04938
H	+3.20424	−0.29896	+0.52840
H	+1.80741	−1.33047	−1.08370
C	+3.73324	+1.90745	−0.93290
H	+1.66989	+2.17001	−0.36543

C +2.91600 +2.37779 +1.37951
H +3.93494 +2.97358 −1.06783
H +4.64444 +1.44224 −0.53974
H +3.54370 +1.48932 −1.92622
H +3.21596 +3.41516 +1.20696
H +2.07412 +2.37394 +2.07448
H +3.74658 +1.85897 +1.87047
19
* E = +1.830 kcal/mol ; (12) 178_152_037_070
H +0.00000 +0.00000 +0.00000
O +0.00000 +0.00000 +0.96433
C +1.27541 +0.00000 +1.37406
C +1.40884 +0.05035 +2.88192
N +2.66038 −0.59427 +3.22807
H +3.36665 −0.38343 +2.53195
O +2.20035 −0.00535 +0.61082
C +1.28353 +1.51785 +3.37588
H +0.56778 −0.51447 +3.30480
H +2.99195 −0.28995 +4.13423
C −0.13120 +2.06901 +3.22001
H +1.51066 +1.46153 +4.45065
C +2.30752 +2.43349 +2.71130
H −0.22321 +3.03058 +3.73325
H −0.37908 +2.22822 +2.16629
H −0.87779 +1.38584 +3.63701
H +2.29568 +3.42336 +3.17608
H +3.32264 +2.03332 +2.78931
H +2.08293 +2.56196 +1.64634
19
* E = +1.878 kcal/mol ; (13) 181_183_209_079
H +0.00000 +0.00000 +0.00000
O +0.00000 +0.00000 +0.96413
C +1.27875 +0.00000 +1.36873
C +1.41694 −0.02438 +2.87908
N +2.82990 +0.06183 +3.19452
H +3.04556 −0.39302 +4.06993
O +2.19459 −0.00711 +0.59559
C +0.65672 +1.13467 +3.56917
H +0.94937 −0.97378 +3.19602
H +3.39701 −0.33513 +2.45589

C	−0.84380	+0.89425	+3.70681
H	+1.08948	+1.16716	+4.57827
C	+0.96225	+2.46167	+2.88103
H	−1.29041	+1.67036	+4.33552
H	−1.34384	+0.91941	+2.73577
H	−1.05385	−0.07562	+4.17021
H	+0.59449	+3.30404	+3.47340
H	+2.04005	+2.58028	+2.73801
H	+0.47032	+2.50880	+1.90176

19

∗ E = +2.036 kcal/mol ; (14) 178_029_195_297

H	+0.00000	+0.00000	+0.00000
O	+0.00000	+0.00000	+0.96375
C	+1.27949	+0.00000	+1.37231
C	+1.41746	+0.05731	+2.88281
N	+0.27084	−0.55324	+3.53699
H	+0.24287	−0.29882	+4.51543
O	+2.19653	+0.01053	+0.60209
C	+2.73542	−0.59773	+3.32480
H	+1.50245	+1.14141	+3.09473
H	−0.59802	−0.25956	+3.11047
C	+2.77874	−2.08076	+2.96505
H	+3.53477	−0.07703	+2.78483
C	+2.93483	−0.39802	+4.82557
H	+3.71043	−2.53015	+3.32112
H	+1.94099	−2.60781	+3.43110
H	+2.72663	−2.23082	+1.88310
H	+3.92358	−0.74903	+5.13331
H	+2.84729	+0.65640	+5.11103
H	+2.19698	−0.97408	+5.39606

19

∗ E = +2.083 kcal/mol ; (15) 182_334_297_067

H	+0.00000	+0.00000	+0.00000
O	+0.00000	+0.00000	+0.96404
C	+1.28249	+0.00000	+1.36992
C	+1.42535	−0.05613	+2.88608
N	+0.30123	+0.47877	+3.63198
H	+0.18023	+1.46745	+3.45094
O	+2.19732	+0.01294	+0.59466
C	+1.71056	−1.51266	+3.31955

```
 H    +2.31648    +0.53689    +3.11967
 H    -0.56073    +0.02458    +3.35409
 C    +3.07171    -1.99856    +2.83045
 H    +1.71491    -1.46812    +4.41536
 C    +0.60529    -2.46889    +2.87388
 H    +3.27836    -2.99872    +3.22263
 H    +3.10547    -2.04894    +1.73778
 H    +3.87561    -1.33153    +3.15591
 H    +0.77744    -3.46548    +3.28998
 H    -0.38731    -2.14266    +3.19906
 H    +0.58978    -2.56663    +1.78185
19
 * E =  +2.117  kcal/mol ; (16) 352_029_081_195
 H    +0.00000    +0.00000    +0.00000
 O    +0.00000    +0.00000    +0.97046
 C    +1.27313    +0.00000    +1.36363
 C    +2.27742    -0.18937    +0.21278
 N    +1.66902    +0.35091    -1.00412
 H    +1.76595    +1.36043    -1.04101
 O    +1.58403    +0.11802    +2.51169
 C    +3.68055    +0.32879    +0.57871
 H    +2.34821    -1.27889    +0.06737
 H    +2.10737    -0.02498    -1.83431
 C    +4.54840    +0.47434    -0.67034
 H    +3.56218    +1.31161    +1.05616
 C    +4.36464    -0.61755    +1.56634
 H    +5.56484    +0.76557    -0.39136
 H    +4.61726    -0.48269    -1.20405
 H    +4.16898    +1.22552    -1.36867
 H    +5.34700    -0.22868    +1.84967
 H    +3.77101    -0.75421    +2.47045
 H    +4.52078    -1.59651    +1.09558
19
 * E =  +2.186  kcal/mol ; (17) 178_135_052_177
 H    +0.00000    +0.00000    +0.00000
 O    +0.00000    +0.00000    +0.96458
 C    +1.27354    +0.00000    +1.37540
 C    +1.42096    +0.04636    +2.88291
 N    +2.42534    -0.94398    +3.22830
 H    +3.26137    -0.79033    +2.67443
```

O	+2.20307	−0.00126	+0.61789
C	+1.74506	+1.50217	+3.29536
H	+0.46058	−0.23714	+3.33282
H	+2.67105	−0.89639	+4.20767
C	+1.82617	+1.61406	+4.81485
H	+2.72602	+1.73912	+2.85912
C	+0.70542	+2.47932	+2.74849
H	+2.06911	+2.63802	+5.11275
H	+0.86173	+1.35520	+5.26816
H	+2.59137	+0.96043	+5.24324
H	+0.89430	+3.49022	+3.12050
H	+0.71238	+2.51926	+1.65528
H	−0.30245	+2.18587	+3.06541

19

∗ E = +2.608 kcal/mol ; (18) 182_339_056_297

H	+0.00000	+0.00000	+0.00000
O	+0.00000	+0.00000	+0.96390
C	+1.27776	+0.00000	+1.37037
C	+1.43389	−0.05648	+2.87694
N	+0.22842	+0.39108	+3.54645
H	−0.58322	−0.13789	+3.25288
O	+2.19842	−0.01922	+0.60290
C	+1.94482	−1.47796	+3.23555
H	+2.24139	+0.64706	+3.11345
H	+0.31993	+0.31664	+4.55084
C	+0.93617	−2.55578	+2.84746
H	+2.86308	−1.62319	+2.65256
C	+2.28287	−1.55940	+4.72044
H	+1.31349	−3.54802	+3.11025
H	−0.01061	−2.41376	+3.38158
H	+0.72359	−2.55288	+1.77357
H	+2.74124	−2.52310	+4.95954
H	+2.97902	−0.76920	+5.02004
H	+1.37940	−1.47208	+5.33533

19

∗ E = +2.715 kcal/mol ; (19) 180_305_068_175

H	+0.00000	+0.00000	+0.00000
O	+0.00000	+0.00000	+0.96359
C	+1.27954	+0.00000	+1.38016
C	+1.36054	+0.01044	+2.89357

N	+0.60123	+1.15493	+3.37594
H	−0.38916	+1.02485	+3.20234
O	+2.20502	−0.03935	+0.62320
C	+0.92077	−1.36580	+3.44019
H	+2.41863	+0.16427	+3.13622
H	+0.73648	+1.28887	+4.36903
C	+1.10723	−1.41393	+4.95352
H	−0.14765	−1.47709	+3.20463
C	+1.70033	−2.49422	+2.76780
H	+0.79773	−2.38499	+5.35025
H	+2.16341	−1.27039	+5.21204
H	+0.52135	−0.64667	+5.46766
H	+1.42115	−3.46316	+3.19102
H	+1.52207	−2.53394	+1.68892
H	+2.77758	−2.35776	+2.91875

19

∗ E = +2.773 kcal/mol ; (20) 182_058_187_192

H	+0.00000	+0.00000	+0.00000
O	+0.00000	+0.00000	+0.96370
C	+1.28103	+0.00000	+1.37749
C	+1.36165	−0.04714	+2.89569
N	+0.65079	−1.25350	+3.31306
H	+0.56703	−1.28364	+4.32091
O	+2.19975	+0.02654	+0.61249
C	+2.81598	−0.03978	+3.37307
H	+0.87527	+0.88009	+3.25752
H	−0.28540	−1.26391	+2.92678
C	+2.89866	−0.38771	+4.85814
H	+3.35339	−0.80059	+2.79402
C	+3.45042	+1.32732	+3.12121
H	+3.92531	−0.27406	+5.21728
H	+2.26826	+0.28827	+5.45118
H	+2.59145	−1.41758	+5.05810
H	+4.50247	+1.32162	+3.41997
H	+3.39978	+1.61087	+2.06783
H	+2.94250	+2.09689	+3.71568

19

∗ E = +2.777 kcal/mol ; (21) 182_330_053_069

H	+0.00000	+0.00000	+0.00000
O	+0.00000	+0.00000	+0.96383

C +1.27906 +0.00000 +1.37303
C +1.41523 −0.04525 +2.88151
N +0.28500 +0.61573 +3.51028
H −0.59457 +0.23980 +3.17824
O +2.19837 −0.02068 +0.60429
C +1.65533 −1.51436 +3.32535
H +2.32569 +0.52089 +3.10994
H +0.32126 +0.50105 +4.51541
C +3.02913 −2.01919 +2.89389
H +1.62464 −1.48009 +4.42400
C +0.55197 −2.44998 +2.83771
H +3.20717 −3.02493 +3.28590
H +3.10529 −2.06057 +1.80297
H +3.82781 −1.36481 +3.25639
H +0.65465 −3.43725 +3.29646
H −0.44642 −2.06964 +3.07561
H +0.61105 −2.58356 +1.75109
19
* E = +2.945 kcal/mol ; (22) 183_067_301_058
H +0.00000 +0.00000 +0.00000
O +0.00000 +0.00000 +0.96378
C +1.28242 +0.00000 +1.37748
C +1.35322 −0.07566 +2.90011
N +0.86155 −1.36837 +3.36973
H −0.09777 −1.51601 +3.08088
O +2.19688 +0.01355 +0.60247
C +2.74159 +0.22593 +3.47289
H +0.65764 +0.68738 +3.27314
H +1.41777 −2.12537 +2.98950
C +3.22415 +1.61475 +3.05675
H +2.59318 +0.21819 +4.56049
C +3.78191 −0.84292 +3.13576
H +4.13016 +1.88316 +3.60738
H +3.45787 +1.64274 +1.98882
H +2.46855 +2.38038 +3.26475
H +4.73893 −0.58560 +3.59935
H +3.49493 −1.82623 +3.52078
H +3.93809 −0.91340 +2.05608
19
* E = +2.975 kcal/mol ; (23) 178_036_189_071

H	+0.00000	+0.00000	+0.00000
O	+0.00000	+0.00000	+0.96377
C	+1.28129	+0.00000	+1.37160
C	+1.39361	+0.05231	+2.88655
N	+0.31027	−0.71587	+3.48470
H	+0.29067	−0.57095	+4.48598
O	+2.19267	+0.01540	+0.59466
C	+2.74723	−0.45733	+3.40898
H	+1.33197	+1.13432	+3.11632
H	−0.58983	−0.45191	+3.10569
C	+3.89203	+0.50644	+3.10520
H	+2.62140	−0.49582	+4.50158
C	+3.05507	−1.87160	+2.92097
H	+4.80892	+0.16922	+3.59762
H	+4.08070	+0.55668	+2.03028
H	+3.67150	+1.51777	+3.46358
H	+3.91668	−2.27960	+3.45722
H	+2.20089	−2.53430	+3.08362
H	+3.29665	−1.86341	+1.85396

19

∗ E = +4.762 kcal/mol ; (24) 358_169_283_294

H	+0.00000	+0.00000	+0.00000
O	+0.00000	+0.00000	+0.96019
C	+1.25714	+0.00000	+1.44110
C	+2.37203	−0.03821	+0.39201
N	+3.67491	+0.21862	+0.96766
H	+3.79202	+1.20612	+1.15627
O	+1.45592	−0.00206	+2.61766
C	+2.34911	−1.40830	−0.32886
H	+2.16047	+0.73918	−0.35664
H	+3.73480	−0.24580	+1.86889
C	+2.72072	−2.53092	+0.63792
H	+1.32486	−1.58648	−0.68863
C	+3.29094	−1.38368	−1.52777
H	+2.62743	−3.50397	+0.14850
H	+3.76204	−2.41991	+0.95810
H	+2.08492	−2.53628	+1.52965
H	+3.29674	−2.35261	−2.03522
H	+2.99642	−0.62001	−2.25509
H	+4.30747	−1.15958	−1.19249

19
∗ E = +5.317 kcal/mol ; (25) 000_156_288_063

H	+0.00000	+0.00000	+0.00000
O	+0.00000	+0.00000	+0.96028
C	+1.25720	+0.00000	+1.43935
C	+2.38564	+0.00419	+0.40098
N	+3.61250	+0.55650	+0.94031
H	+3.52444	+1.55791	+1.06487
O	+1.45593	−0.02029	+2.61638
C	+2.63918	−1.42681	−0.12788
H	+2.07216	+0.62311	−0.45218
H	+3.76334	+0.17391	+1.86902
C	+1.42968	−2.00899	−0.85726
H	+3.46103	−1.30720	−0.84382
C	+3.09831	−2.36373	+0.98681
H	+1.70374	−2.93822	−1.36388
H	+0.61720	−2.24880	−0.16215
H	+1.04549	−1.32377	−1.62417
H	+3.30060	−3.35988	+0.58303
H	+4.01609	−2.00643	+1.46107
H	+2.32899	−2.46442	+1.76078

19
∗ E = +5.425 kcal/mol ; (26) 001_146_292_175

H	+0.00000	+0.00000	+0.00000
O	+0.00000	+0.00000	+0.95999
C	+1.25758	+0.00000	+1.43736
C	+2.39035	+0.02047	+0.40579
N	+3.53661	+0.76892	+0.88372
H	+3.32012	+1.75558	+0.95840
O	+1.46021	−0.03264	+2.61346
C	+2.81035	−1.42893	+0.08353
H	+2.03031	+0.48670	−0.52504
H	+3.75928	+0.45167	+1.82304
C	+3.84424	−1.43199	−1.03801
H	+3.27025	−1.82252	+1.00175
C	+1.61776	−2.30995	−0.28256
H	+4.22327	−2.44358	−1.20928
H	+3.38680	−1.08551	−1.97311
H	+4.68219	−0.77372	−0.80320
H	+1.95844	−3.31086	−0.56062

H +0.90196 −2.41717 +0.53756
H +1.08707 −1.89991 −1.15322
19
* E = +5.834 kcal/mol ; (27) 356_154_030_069
H +0.00000 +0.00000 +0.00000
O +0.00000 +0.00000 +0.96043
C +1.25518 +0.00000 +1.43649
C +2.36609 −0.09402 +0.39550
N +3.54988 +0.51840 +0.95578
H +3.55143 +0.40675 +1.96376
O +1.46092 +0.02878 +2.61188
C +2.55735 −1.57322 −0.03809
H +2.04767 +0.47110 −0.49640
H +4.39550 +0.11345 +0.57604
C +1.37634 −2.10357 −0.84846
H +3.43864 −1.55557 −0.69566
C +2.85201 −2.47971 +1.15337
H +1.62088 −3.07062 −1.29617
H +0.49663 −2.25704 −0.21352
H +1.10607 −1.42375 −1.66618
H +3.12378 −3.48320 +0.81444
H +3.67257 −2.09099 +1.76299
H +1.97265 −2.56927 +1.80088
19
* E = +6.040 kcal/mol ; (28) 355_162_030_299
H +0.00000 +0.00000 +0.00000
O +0.00000 +0.00000 +0.96019
C +1.25533 +0.00000 +1.43989
C +2.35577 −0.12604 +0.39446
N +3.59473 +0.31076 +0.99228
H +3.58742 +0.11799 +1.98757
O +1.45815 +0.04188 +2.61473
C +2.34480 −1.57971 −0.15409
H +2.09997 +0.54209 −0.44561
H +4.40363 −0.11987 +0.56698
C +2.61966 −2.59933 +0.94756
H +1.33969 −1.76655 −0.56105
C +3.34651 −1.72211 −1.29727
H +2.60219 −3.61464 +0.54201
H +3.61006 −2.43214 +1.38461

H	+1.88473	−2.54085	+1.75588
H	+3.27737	−2.71632	−1.74743
H	+3.17538	−0.97842	−2.08198
H	+4.37568	−1.60666	−0.93849

19

∗ E = +6.514 kcal/mol ; (29) 357_138_044_178

H	+0.00000	+0.00000	+0.00000
O	+0.00000	+0.00000	+0.95965
C	+1.25477	+0.00000	+1.43452
C	+2.38336	−0.07874	+0.41021
N	+3.40186	+0.85456	+0.84641
H	+3.54442	+0.75229	+1.84588
O	+1.46374	+0.01762	+2.60956
C	+2.82777	−1.55675	+0.29220
H	+2.00821	+0.23875	−0.57756
H	+4.28106	+0.70214	+0.37181
C	+3.93283	−1.69968	−0.75036
H	+3.21567	−1.84263	+1.27977
C	+1.65024	−2.46321	−0.06394
H	+4.24972	−2.74319	−0.83254
H	+3.57194	−1.38313	−1.73662
H	+4.82106	−1.10998	−0.50717
H	+1.98683	−3.49308	−0.21063
H	+0.88064	−2.47126	+0.71332
H	+1.18502	−2.13801	−1.00465

19

∗ E = +7.289 kcal/mol ; (30) 353_290_301_171

H	+0.00000	+0.00000	+0.00000
O	+0.00000	+0.00000	+0.96069
C	+1.26136	+0.00000	+1.44444
C	+2.37022	−0.16909	+0.40359
N	+2.38981	−1.51278	−0.16195
H	+2.52002	−2.21600	+0.55474
O	+1.45416	+0.08418	+2.61839
C	+2.28123	+0.88157	−0.71171
H	+3.30684	−0.01637	+0.95217
H	+1.53060	−1.72817	−0.65540
C	+3.51880	+0.80895	−1.60200
H	+1.40205	+0.64505	−1.33838
C	+2.11088	+2.28156	−0.12479

H	+3.43235	+1.50401	−2.44182
H	+4.40702	+1.09051	−1.02480
H	+3.67123	−0.19991	−1.98980
H	+2.08438	+3.03270	−0.91861
H	+1.19420	+2.38077	+0.46539
H	+2.95648	+2.51713	+0.53118

9 Asn

9.1 Reference Z-matrix for Asn

Bond distances are given in Å; bond and dihedral angles, in degrees.

h						
o	1	oh2				
c	2	co3	1	coh3		
c	3	cc4	2	cco4	1	dih4
n	4	nc5	3	ncc5	2	dih5
h	5	hn6	4	hnc6	3	dih6
o	3	oc7	2	oco7	4	dih7
c	4	cc8	3	ccc8	5	dih8
h	4	hc9	3	hcc9	5	dih9
h	5	hn10	4	hnc10	6	dih10
c	8	cc11	4	ccc11	3	dih11
h	8	hc12	4	hcc12	11	dih12
h	8	hc13	4	hcc13	11	dih13
n	11	nc14	8	ncc14	4	dih14
h	14	hn15	11	hnc15	8	dih15
h	14	hn16	11	hnc16	15	dih16
o	11	oc17	8	occ17	14	dih17

oh2	0.96828128
co3	1.35610922
coh3	112.06300787
cc4	1.50639769
cco4	110.87140795
dih4	180.99893433
nc5	1.46887696
ncc5	107.44685678
dih5	50.4937386
hn6	1.00467618
hnc6	110.07676647
dih6	181.86899798
oc7	1.20255264
oco7	122.20191751
dih7	181.4370328
cc8	1.52268821
ccc8	109.09610538

dih8	118.58595143
hc9	1.08801112
hcc9	107.61191391
dih9	237.54613751
hn10	1.00384864
hnc10	112.07339306
dih10	125.91773299
cc11	1.51466461
ccc11	111.45977385
dih11	173.79855761
hc12	1.08360726
hcc12	107.82716104
dih12	119.31642523
hc13	1.0823204
hcc13	110.56072897
dih13	237.35902689
nc14	1.35143623
ncc14	114.65449897
dih14	186.95282032
hn15	0.99760905
hnc15	118.74579708
dih15	181.46051889
hn16	0.99517674
hnc16	122.54464858
dih16	179.21513827
oc17	1.22221452
occ17	122.71444657
dih17	180.5413488

9.2 Reference energy and target torsions for Asn

- Reference M08-HX/MG3S energy: -492.4713959 hartree
- Target torsions:
  - torsion1: dih4
  - torsion2: dih5
  - torsion3: dih6
  - torsion4: dih11

– torsion5: dih14

9.3 Properties of Asn conformers

Table S9: Relative energy (kcal mol^{-1}), rotational constants (B1 to B3, GHz) and dipolar moment (Debye) for the conformers of Asn located at the M08-HX/MG3S level.

Conformer	Rel. Energy	B1	B2	B3	Dip. Mom.
1	0.000	1.112	1.405	2.300	2.319
2	1.913	0.771	0.922	3.430	4.865
3	2.552	1.137	1.317	2.374	3.706
4	2.594	1.100	1.245	2.596	4.682
5	2.603	1.109	1.236	2.610	2.222
6	2.608	0.883	1.124	2.775	6.170
7	2.644	0.780	0.951	3.131	3.534
8	2.812	0.788	0.964	3.212	3.763
9	3.266	0.794	0.916	3.420	3.779
10	3.375	0.846	1.112	2.812	3.009
11	3.505	0.769	0.914	3.532	1.532
12	3.814	1.060	1.361	2.356	3.398
13	4.087	0.993	1.077	3.087	6.983
14	4.420	0.990	1.201	2.842	3.650
15	4.425	1.120	1.412	2.218	4.883
16	4.446	0.761	0.921	3.328	5.799
17	4.473	0.860	1.108	2.762	4.010
18	4.573	0.807	0.916	3.129	5.286
19	4.733	1.003	1.171	2.573	2.578
20	4.735	1.005	1.170	2.572	2.653
21	4.789	1.116	1.262	2.444	6.018
22	4.870	0.816	0.911	3.225	5.130
23	4.973	0.906	1.158	2.597	3.286
24	5.242	1.120	1.243	2.572	4.147
25	5.283	1.017	1.308	2.479	2.598
26	5.382	1.080	1.102	2.563	4.245
27	5.616	1.114	1.421	2.308	4.973
28	5.621	0.790	0.967	3.147	5.324

29	5.622	0.943	1.139	2.697	3.015
30	5.801	0.944	1.140	2.670	4.179
31	5.818	0.813	0.902	3.274	4.924
32	5.930	0.999	1.206	2.565	4.918
33	6.206	1.046	1.087	2.753	3.000
34	6.220	0.982	1.202	2.368	2.583
35	6.237	1.110	1.237	2.570	6.070
36	6.239	0.809	0.876	3.418	3.493
37	6.843	1.000	1.205	2.505	4.183
38	6.880	1.047	1.240	2.595	4.102
39	7.045	0.898	1.118	2.589	3.029
40	7.057	1.041	1.288	2.360	4.079
41	7.379	0.783	0.965	3.079	3.129
42	7.419	1.035	1.246	2.281	5.128
43	7.666	0.837	1.079	2.787	4.489
44	7.743	0.940	1.156	2.389	4.929
45	8.017	0.822	0.913	3.347	2.980
46	8.121	1.081	1.110	2.555	4.885
47	8.212	1.039	1.271	2.400	4.103
48	8.311	0.844	1.088	2.767	3.161
49	8.580	1.047	1.268	2.275	4.751
50	8.832	1.129	1.246	2.332	7.026
51	8.941	0.991	1.090	3.021	3.237
52	9.006	0.818	0.903	3.369	4.567
53	9.020	0.824	0.935	3.157	2.900
54	9.032	0.986	1.212	2.533	6.519
55	9.746	0.837	0.894	3.200	5.320
56	9.897	0.777	0.912	3.527	2.629
57	10.277	1.001	1.193	2.465	5.498
58	10.311	1.081	1.098	2.563	3.082
59	10.363	1.162	1.414	2.228	4.484
60	10.509	0.916	1.167	2.625	4.041
61	11.003	1.048	1.101	2.800	2.612
62	11.262	0.967	1.186	2.444	2.622
63	12.831	1.077	1.237	2.532	3.857
64	13.889	1.023	1.180	2.723	7.814

9.4 Vibrational frequencies of Asn conformers

Harmonic vibrational zero point energy (ZPE) and frequencies for each conformer in cm^{-1}.

- Conformer (1); ZPE = 30345.335 cm^{-1}:

51.00	68.05	135.78	191.36	261.98	315.96
361.68	425.80	454.07	472.93	533.14	546.34
570.00	620.85	747.36	805.11	820.90	833.76
891.90	928.57	951.33	1049.31	1099.97	1143.43
1181.16	1234.32	1254.85	1299.10	1329.35	1394.17
1419.54	1442.31	1474.60	1632.60	1672.34	1787.94
1871.57	3053.05	3086.89	3146.86	3508.70	3609.54
3633.55	3638.35	3739.29			

- Conformer (2); ZPE = 30157.355 cm^{-1}:

45.11	83.97	92.14	159.68	260.54	323.13
327.63	347.45	375.98	427.11	514.95	546.77
580.61	638.50	668.48	747.57	809.21	870.08
892.94	921.86	935.29	1080.81	1104.30	1154.31
1178.02	1219.40	1256.83	1282.36	1339.07	1398.48
1425.46	1439.84	1453.75	1627.18	1661.87	1798.08
1887.24	3037.40	3050.93	3097.36	3533.15	3618.05
3638.39	3691.75	3771.72			

- Conformer (3); ZPE = 30158.784 cm^{-1}:

60.75	73.62	92.62	222.11	273.37	283.20
344.09	366.87	465.21	492.25	508.26	545.43
576.82	612.24	661.60	733.39	772.08	811.46
866.07	934.43	957.01	1013.60	1118.61	1152.10
1188.78	1208.68	1240.21	1328.29	1339.23	1350.67
1404.32	1434.55	1462.45	1620.92	1629.43	1812.65
1865.96	2969.28	3061.79	3136.13	3549.12	3560.15
3643.25	3723.89	3850.63			

- Conformer (4); ZPE = 30031.885 cm^{-1}:

40.10	88.49	106.58	179.78	257.41	274.76
291.63	327.73	362.58	447.12	482.12	517.61
554.63	611.70	641.62	673.99	778.18	805.80
883.80	927.75	972.61	1050.15	1096.02	1150.56
1174.82	1198.73	1271.88	1310.24	1314.61	1390.46
1403.86	1433.71	1447.62	1625.49	1667.50	1803.19
1874.81	3032.44	3062.58	3112.99	3537.00	3616.41
3640.90	3775.20	3846.59			

- Conformer (5); ZPE = 30069.213 cm^{-1}:

39.51	72.69	112.04	194.08	262.42	294.50
308.40	327.54	357.50	451.63	496.18	531.86
551.65	606.58	641.87	676.18	781.76	806.76
880.40	914.66	959.46	1049.35	1095.10	1144.46
1194.29	1206.34	1267.89	1313.72	1318.19	1398.79
1406.77	1428.22	1442.26	1624.53	1658.93	1798.53
1854.22	3039.13	3066.05	3118.89	3548.05	3632.70
3640.91	3774.30	3849.12			

- Conformer (6); ZPE = 30136.514 cm^{-1}:

37.57	91.47	118.97	152.39	224.14	242.61
313.49	353.73	368.04	429.01	508.88	520.17
614.10	658.15	687.96	765.43	831.57	843.11
888.34	934.83	1001.35	1046.67	1110.76	1167.11
1200.99	1214.70	1251.46	1312.68	1349.36	1404.10
1443.52	1445.84	1462.93	1629.33	1675.48	1768.76
1876.09	3037.94	3056.75	3098.28	3532.81	3581.27
3611.37	3638.00	3771.49			

- Conformer (7); ZPE = 30153.831 cm^{-1}:

46.46	54.24	96.12	174.56	242.49	267.88
308.17	347.60	445.19	473.29	506.09	538.56
594.26	621.04	673.53	740.01	767.17	864.08
899.55	930.02	995.66	1016.21	1129.79	1165.10
1167.94	1196.63	1266.60	1311.86	1344.10	1377.06
1396.52	1427.59	1456.12	1618.81	1664.62	1809.93
1865.49	3036.47	3054.30	3137.34	3531.45	3564.53
3609.36	3730.36	3843.49			

- Conformer (8); ZPE = 30268.960 cm^{-1}:

26.78	70.70	97.52	168.55	240.54	336.64
346.93	349.56	374.48	448.37	533.54	553.59
602.79	657.78	735.56	759.53	837.91	878.73
893.55	931.39	988.63	1019.91	1094.14	1148.59
1181.80	1243.74	1270.42	1302.41	1356.90	1375.07
1417.99	1440.94	1474.28	1627.73	1672.32	1792.85
1889.18	3022.99	3052.18	3122.54	3526.29	3614.07
3638.76	3644.26	3775.50			

- Conformer (9); ZPE = 30034.470 cm^{-1}:

46.41	71.02	91.13	157.32	244.12	297.52
324.28	350.08	374.47	437.24	494.12	522.72
577.38	604.91	648.82	666.65	737.42	867.46
880.24	905.05	954.73	1063.30	1103.90	1165.71
1188.56	1220.76	1248.95	1261.07	1343.64	1365.39
1417.37	1441.94	1450.95	1626.00	1629.18	1803.96
1868.88	2971.48	3052.83	3106.88	3562.46	3641.56
3654.84	3776.57	3849.66			

- Conformer (10); ZPE = 30304.590 cm^{-1}:

37.61	75.29	108.36	201.99	247.53	299.50
368.61	377.62	416.29	470.44	531.69	557.11
592.41	668.21	742.04	764.16	810.94	840.38
894.41	916.89	967.19	1069.78	1118.26	1153.75
1186.33	1204.00	1251.43	1312.65	1338.06	1396.87
1406.55	1439.66	1460.39	1626.90	1661.14	1806.42
1871.79	3031.72	3062.53	3134.24	3546.10	3583.63
3637.73	3684.32	3736.22			

- Conformer (11); ZPE = 29995.381 cm^{-1}:

40.66	60.48	92.81	156.62	252.71	278.16
323.83	338.12	360.61	405.13	504.68	536.56
569.77	606.10	641.26	655.49	752.27	871.18
888.63	898.66	969.49	1059.65	1101.14	1169.63
1186.35	1205.08	1259.27	1264.90	1337.94	1368.94
1418.48	1442.13	1465.48	1625.64	1647.36	1800.48
1866.69	2957.39	3054.21	3103.81	3549.98	3635.53
3641.55	3775.57	3850.35			

- Conformer (12); ZPE = 30184.563 cm^{-1}:

24.55	49.01	143.65	191.70	253.07	311.58
322.58	393.35	444.87	473.67	499.17	544.82
553.32	602.81	640.94	742.94	794.03	815.08
866.33	904.06	966.92	1019.84	1114.83	1153.75
1185.10	1199.15	1268.23	1324.04	1334.30	1383.80
1410.79	1418.16	1470.76	1631.44	1656.41	1796.67
1842.77	2997.54	3085.44	3145.07	3539.78	3618.88
3643.82	3746.06	3844.03			

- Conformer (13); ZPE = 30278.368 cm^{-1}:

36.31	81.92	147.57	210.76	229.38	288.15
341.73	366.39	380.38	393.64	519.82	560.89
626.28	661.06	747.00	799.21	815.67	830.36
882.57	903.96	958.15	1034.14	1109.98	1145.76
1193.10	1232.54	1320.83	1323.71	1337.92	1404.28
1437.59	1440.72	1466.89	1632.03	1656.67	1765.38
1873.87	3048.01	3092.91	3104.05	3550.37	3560.29
3634.94	3639.06	3770.48			

- Conformer (14); ZPE = 30111.773 cm^{-1}:

55.56	76.48	130.27	176.97	215.38	262.81
312.84	371.89	426.36	445.21	513.42	554.88
577.31	606.01	651.74	756.15	765.38	801.52
834.58	930.25	973.19	1001.97	1118.25	1152.16
1177.35	1196.37	1238.48	1330.89	1339.63	1353.28
1420.50	1449.97	1467.08	1630.36	1645.08	1805.24
1853.01	2966.56	3081.77	3140.34	3554.66	3611.34
3657.34	3746.33	3847.40			

- Conformer (15); ZPE = 30085.546 cm^{-1}:

38.34	59.34	119.79	179.74	238.84	256.12
310.13	351.63	400.51	451.72	496.15	537.92
559.01	617.34	652.54	725.95	793.90	812.35
889.66	909.01	971.63	1011.93	1104.20	1156.63
1168.24	1186.84	1266.88	1318.15	1324.81	1374.02
1403.99	1414.35	1476.49	1625.42	1674.00	1804.07
1874.28	3000.30	3082.80	3142.79	3532.38	3621.51
3632.06	3766.46	3836.90			

- Conformer (16); ZPE = 30150.686 cm^{-1}:

20.55	61.50	92.70	169.22	272.47	307.84
327.16	364.94	389.21	461.53	515.82	571.79
583.88	621.37	653.31	737.88	769.87	861.83
891.89	926.31	991.29	1011.12	1120.98	1159.25
1163.76	1185.16	1276.10	1296.85	1338.26	1389.35
1401.30	1414.27	1456.41	1621.30	1655.23	1808.66
1873.33	3028.84	3051.00	3142.17	3535.13	3575.21
3624.18	3734.45	3846.70			

- Conformer (17); ZPE = 30306.893 cm^{-1}:

34.85	66.72	116.90	218.33	227.81	283.50
337.88	368.69	428.41	509.48	529.04	579.95
615.50	671.15	719.81	780.81	823.95	841.38
894.48	919.12	994.92	1012.41	1121.55	1154.15
1185.29	1230.78	1272.32	1322.98	1355.53	1374.59
1407.81	1438.57	1461.35	1648.91	1658.68	1804.40
1870.50	3035.49	3051.78	3113.36	3547.16	3561.01
3646.22	3656.83	3719.42			

- Conformer (18); ZPE = 30140.818 cm^{-1}:

28.50	57.85	96.29	154.86	253.61	292.41
299.35	342.70	437.28	462.10	526.30	556.33
604.04	616.84	655.96	744.78	781.39	850.55
898.05	919.53	967.05	1003.31	1123.46	1156.09
1166.70	1196.96	1277.97	1300.47	1357.26	1379.71
1398.53	1399.08	1454.46	1618.33	1656.87	1810.30
1876.86	3044.42	3085.42	3122.09	3537.33	3571.23
3621.78	3732.46	3844.74			

- Conformer (19); ZPE = 29999.728 cm^{-1}:

14.47	59.02	90.72	151.71	250.43	280.99
309.09	311.01	352.93	387.16	500.68	543.00
582.80	614.18	641.51	665.04	776.50	831.65
879.30	887.10	994.79	1056.38	1097.15	1146.37
1174.18	1185.61	1244.97	1292.49	1324.59	1392.13
1423.09	1442.16	1448.05	1625.78	1659.63	1808.08
1859.83	3053.90	3093.30	3110.56	3541.65	3632.19
3641.07	3774.60	3847.60			

- Conformer (20); ZPE = 29987.802 cm^{-1}:

9.66	58.04	88.76	150.40	251.78	280.89
304.74	309.97	355.29	386.63	498.43	544.17
580.52	611.59	638.88	668.75	775.02	830.71
879.21	887.11	994.22	1056.22	1097.02	1147.07
1174.25	1186.03	1244.60	1293.25	1324.87	1391.29
1422.21	1441.53	1449.29	1625.57	1659.66	1808.75
1859.90	3054.35	3091.50	3110.88	3541.88	3632.50
3639.67	3772.42	3846.13			

- Conformer (21); ZPE = 30175.890 cm^{-1}:

44.68	66.74	99.13	223.18	281.68	293.77
340.56	360.98	468.04	490.93	537.49	548.85
567.19	611.58	672.54	727.38	768.40	808.41
858.36	930.73	961.79	1007.94	1119.77	1153.02
1175.47	1211.00	1248.44	1335.21	1340.53	1352.89
1402.97	1433.28	1448.00	1621.07	1647.60	1815.98
1878.45	2974.35	3062.76	3134.99	3554.41	3560.57
3644.03	3721.11	3845.53			

- Conformer (22); ZPE = 29925.717 cm^{-1}:

36.87	61.42	95.94	150.10	231.74	267.03
274.02	334.95	357.52	427.58	488.76	524.75
597.82	605.84	644.78	671.55	756.83	818.92
871.29	898.63	931.11	1060.13	1099.33	1150.60
1162.75	1199.45	1251.25	1281.80	1329.11	1364.28
1416.15	1446.84	1455.49	1620.11	1623.44	1806.85
1874.04	3030.89	3068.14	3085.77	3556.89	3639.98
3658.84	3774.65	3847.17			

- Conformer (23); ZPE = 30162.447 cm^{-1}:

24.70	59.84	122.39	175.34	250.53	303.18
304.97	372.75	427.66	458.32	488.78	569.96
583.89	615.83	620.78	749.03	770.43	827.27
880.28	927.29	976.38	1051.63	1112.54	1150.51
1180.74	1205.35	1244.17	1296.40	1336.95	1369.36
1412.95	1453.79	1468.62	1630.14	1654.79	1807.20
1861.06	2963.69	3077.84	3136.99	3554.02	3614.73
3641.17	3745.71	3844.90			

- Conformer (24); ZPE = 30016.848 cm^{-1}:

36.02	77.15	109.16	190.67	259.90	288.88
312.94	346.64	348.70	465.36	501.84	528.28
554.05	608.46	641.14	680.36	784.07	800.64
852.02	886.11	936.35	1045.17	1102.51	1134.73
1191.85	1216.29	1260.19	1282.35	1322.31	1376.61
1424.73	1440.18	1459.12	1624.22	1652.97	1801.03
1847.27	3032.92	3052.60	3101.99	3551.97	3638.05
3644.38	3770.93	3850.59			

- Conformer (25); ZPE = 30171.007 cm^{-1}:

37.65	41.57	157.78	171.13	198.64	255.81
321.17	396.77	449.53	478.46	518.11	551.04
563.12	613.67	653.00	750.29	795.09	814.72
865.49	903.98	947.71	1047.15	1105.60	1154.08
1190.58	1206.19	1247.47	1297.50	1328.07	1380.12
1415.93	1442.71	1471.75	1630.24	1683.96	1788.85
1831.76	3034.56	3080.54	3139.06	3539.76	3612.38
3639.52	3741.58	3847.91			

- Conformer (26); ZPE = 30087.190 cm^{-1}:

39.39	66.95	141.46	192.60	198.95	245.28
293.53	354.31	447.51	462.73	512.54	541.20
566.85	631.49	658.81	698.87	788.53	806.91
849.87	883.05	959.40	1032.93	1112.75	1151.23
1177.52	1213.53	1264.21	1309.30	1343.16	1374.56
1398.97	1426.15	1449.95	1646.56	1668.18	1808.94
1844.29	3051.31	3070.10	3130.11	3558.13	3594.89
3641.65	3726.64	3839.07			

- Conformer (27); ZPE = 30256.049 cm^{-1}:

46.37	81.08	144.70	199.80	276.75	317.58
361.31	389.49	458.40	504.53	535.28	546.03
582.90	611.38	663.00	741.47	796.07	829.33
897.30	948.90	974.45	1029.48	1110.50	1156.54
1201.38	1219.10	1260.89	1318.59	1335.56	1352.66
1417.33	1439.56	1475.75	1629.39	1640.66	1785.45
1872.93	2979.88	3067.94	3124.79	3534.48	3567.40
3624.47	3717.94	3743.28			

- Conformer (28); ZPE = 30051.244 cm^{-1}:

14.93	65.67	85.38	166.75	229.62	299.92
315.64	327.67	341.98	441.83	508.42	541.66
597.24	609.14	658.28	728.96	772.21	869.39
878.95	895.08	950.94	1034.34	1095.93	1158.65
1170.45	1211.98	1259.99	1277.29	1341.04	1353.75
1402.80	1456.15	1471.61	1624.40	1645.16	1799.26
1875.44	3023.90	3054.18	3113.12	3541.03	3630.13
3638.95	3776.78	3846.51			

- Conformer (29); ZPE = 30005.750 cm^{-1}:

49.82	60.91	87.34	165.88	257.65	275.47
296.60	326.14	363.43	420.89	508.14	514.39
576.48	608.93	626.16	698.58	743.77	830.32
867.94	912.97	973.91	1067.36	1110.75	1147.64
1189.16	1202.63	1244.45	1273.30	1344.32	1359.12
1429.35	1444.91	1466.52	1625.13	1645.04	1809.22
1866.13	2983.90	3057.99	3109.01	3555.45	3642.65
3645.56	3779.48	3846.70			

- Conformer (30); ZPE = 29953.604 cm^{-1}:

32.54	59.40	89.68	152.72	214.93	260.29
268.43	313.18	347.73	418.77	497.39	513.50
584.52	613.50	634.56	692.54	750.20	827.03
887.82	916.06	999.73	1044.10	1096.97	1168.86
1174.54	1209.69	1227.61	1300.45	1328.32	1377.41
1415.89	1430.30	1463.04	1624.50	1672.51	1815.54
1862.33	3038.15	3050.47	3103.57	3540.23	3619.18
3645.64	3782.48	3840.90			

- Conformer (31); ZPE = 29934.955 cm^{-1}:

13.78	44.84	97.03	149.63	235.76	279.70
285.79	327.67	348.85	429.14	497.13	545.57
582.75	609.80	646.82	661.45	762.33	840.82
869.56	896.95	925.05	1063.28	1098.75	1129.13
1171.33	1217.29	1246.05	1280.83	1339.22	1370.02
1416.13	1436.27	1453.28	1624.10	1640.44	1805.72
1867.36	3030.05	3077.17	3091.51	3553.11	3639.42
3646.99	3773.81	3848.23			

- Conformer (32); ZPE = 29992.881 cm^{-1}:

31.06	67.01	109.66	149.61	248.43	261.33
276.74	319.84	350.63	391.51	502.14	561.03
576.47	610.90	641.85	657.04	779.78	829.90
876.30	897.85	987.31	1039.60	1098.86	1138.90
1163.56	1201.35	1254.69	1291.27	1351.34	1381.03
1399.41	1437.05	1454.19	1626.41	1658.42	1810.98
1876.03	3048.59	3092.24	3105.63	3536.14	3626.25
3642.50	3777.13	3847.79			

- Conformer (33); ZPE = 30053.110 cm^{-1}:

29.98	54.04	125.90	180.56	206.55	222.93
297.61	354.54	425.81	436.88	524.69	541.75
585.87	623.45	657.75	711.89	799.13	808.52
864.17	894.16	958.19	1022.13	1115.35	1146.78
1181.93	1217.46	1249.07	1278.22	1338.38	1361.94
1412.78	1457.50	1476.08	1642.76	1648.60	1805.07
1849.18	3050.37	3066.62	3124.88	3546.12	3602.07
3634.07	3735.04	3839.46			

- Conformer (34); ZPE = 30088.687 cm^{-1}:

34.28	44.37	114.21	158.65	244.06	248.79
292.78	336.14	418.28	435.17	475.39	575.87
596.31	618.81	629.31	749.12	789.95	815.66
852.68	911.75	961.18	1092.49	1097.99	1136.14
1172.03	1187.87	1239.03	1302.01	1327.57	1379.00
1401.60	1457.60	1470.80	1626.26	1654.90	1808.05
1845.39	3062.11	3078.59	3135.54	3547.57	3616.86
3642.50	3749.62	3843.05			

- Conformer (35); ZPE = 29908.709 cm^{-1}:

31.09	94.36	104.28	160.67	199.38	267.44
284.73	330.83	360.26	466.74	487.54	520.69
549.89	606.40	645.48	661.51	781.38	794.16
836.91	897.70	938.67	1039.11	1101.13	1145.54
1165.83	1206.13	1272.81	1273.98	1326.32	1349.29
1420.89	1442.65	1460.31	1625.18	1646.07	1809.53
1890.44	3010.17	3046.51	3093.97	3552.07	3640.53
3648.05	3775.89	3854.87			

- Conformer (36); ZPE = 29918.037 cm^{-1}:

22.03	43.16	69.39	141.61	227.48	250.95
281.89	319.52	374.28	386.96	498.49	548.50
563.76	597.56	636.67	696.35	753.57	835.56
888.06	918.97	958.54	1033.24	1089.26	1151.87
1170.25	1195.86	1256.32	1291.42	1325.68	1387.16
1407.53	1433.35	1457.62	1622.36	1663.22	1813.82
1859.26	3040.36	3054.53	3109.74	3547.53	3638.92
3643.22	3780.71	3849.55			

- Conformer (37); ZPE = 29935.383 cm^{-1}:

27.46	71.21	89.99	152.58	240.88	253.69
288.78	297.79	333.80	388.61	496.84	527.02
588.09	615.13	636.92	667.09	789.19	820.75
840.49	888.86	979.04	1085.48	1112.04	1127.84
1174.81	1186.83	1243.20	1280.17	1337.23	1370.69
1425.44	1443.41	1472.86	1625.35	1647.39	1808.06
1857.48	3043.11	3090.55	3106.79	3542.05	3637.43
3638.05	3772.19	3848.12			

- Conformer (38); ZPE = 29995.856 cm^{-1}:

57.61	73.79	99.47	156.85	234.79	254.26
325.69	348.07	414.69	454.33	493.95	526.55
562.41	614.24	659.89	712.99	764.28	792.64
824.66	930.97	957.37	995.92	1093.09	1147.43
1180.80	1199.47	1225.74	1322.91	1336.07	1341.87
1412.97	1434.13	1474.25	1622.63	1634.59	1823.33
1876.82	2921.55	3077.00	3132.32	3570.37	3635.21
3668.10	3768.03	3837.60			

- Conformer (39); ZPE = 30089.194 cm^{-1}:

32.60	61.18	124.46	202.41	224.41	262.07
309.65	330.40	436.53	481.95	506.64	546.18
593.66	600.63	620.51	698.97	792.73	826.18
848.16	903.30	973.35	1064.10	1118.14	1130.28
1183.34	1190.43	1252.41	1306.05	1353.09	1383.98
1397.21	1436.60	1460.89	1650.34	1653.60	1801.69
1851.96	3046.16	3074.27	3109.08	3549.25	3582.84
3638.91	3723.33	3844.48			

- Conformer (40); ZPE = 29933.365 cm^{-1}:

24.82	62.51	99.78	139.67	220.05	235.64
256.07	277.83	331.35	423.63	489.76	546.41
581.12	588.08	648.18	726.47	772.24	821.48
902.15	914.06	982.89	1036.25	1091.21	1149.57
1173.62	1182.66	1229.55	1298.69	1332.42	1373.65
1400.59	1432.27	1473.91	1624.50	1668.87	1817.68
1870.85	3056.62	3066.72	3126.39	3535.24	3616.98
3643.56	3778.23	3842.50			

- Conformer (41); ZPE = 30124.238 cm^{-1}:

46.06	63.23	90.71	184.27	241.03	267.68
315.13	348.48	444.85	469.39	496.61	510.58
552.39	620.04	693.69	730.37	761.96	870.35
904.54	931.61	991.88	1020.46	1126.02	1162.43
1169.11	1190.94	1264.65	1305.66	1329.26	1363.64
1399.23	1421.07	1462.20	1618.04	1668.45	1808.52
1895.69	3020.10	3051.77	3121.90	3527.49	3566.66
3606.82	3731.89	3881.64			

- Conformer (42); ZPE = 30032.332 cm^{-1}:

38.66	51.29	107.13	144.48	245.73	254.75
286.89	331.17	335.99	441.70	498.94	579.62
592.57	606.45	631.48	746.70	788.19	819.04
868.93	908.46	966.42	1019.64	1087.72	1136.38
1163.92	1187.31	1249.28	1303.84	1341.41	1368.94
1396.62	1402.93	1476.98	1624.93	1652.65	1819.98
1878.25	3060.30	3092.49	3144.68	3541.51	3632.23
3632.85	3766.86	3838.37			

- Conformer (43); ZPE = 30042.080 cm^{-1}:

39.83	54.19	96.46	182.52	221.47	224.96
293.56	366.16	412.42	417.05	499.68	537.19
591.50	602.46	654.28	694.41	773.58	830.94
895.72	907.93	992.88	1013.85	1120.49	1154.77
1176.60	1215.08	1234.02	1304.18	1339.05	1365.98
1390.84	1420.93	1458.20	1634.09	1671.17	1815.29
1878.27	3036.14	3051.08	3137.09	3540.29	3619.16
3624.81	3755.78	3837.87			

- Conformer (44); ZPE = 30041.984 cm^{-1}:

30.30	44.21	121.25	184.96	235.62	258.93
288.79	343.07	365.83	440.51	497.97	509.25
588.51	624.97	643.20	683.35	789.91	823.52
880.98	904.04	1003.66	1039.35	1115.10	1141.67
1169.52	1188.33	1250.65	1302.01	1340.24	1383.87
1396.68	1429.56	1453.71	1646.33	1665.05	1808.36
1876.29	3057.00	3069.21	3115.23	3535.41	3622.99
3629.47	3756.69	3828.39			

- Conformer (45); ZPE = 29912.194 cm^{-1}:

16.29	49.10	60.29	142.34	211.21	226.72
278.61	301.63	383.12	426.51	522.09	528.50
566.67	597.70	640.53	719.94	755.75	801.87
880.08	927.02	964.92	989.25	1084.96	1150.36
1186.88	1204.33	1284.28	1297.62	1350.38	1376.91
1390.59	1406.59	1473.42	1626.29	1664.23	1819.37
1858.42	3002.68	3069.28	3128.65	3554.85	3640.29
3649.32	3772.52	3842.05			

- Conformer (46); ZPE = 30064.386 cm^{-1}:

44.58	64.53	127.33	188.97	242.32	270.29
303.15	352.53	387.71	473.01	504.07	545.87
569.34	594.27	654.79	701.82	771.60	800.57
840.09	878.06	956.46	1020.32	1100.70	1148.99
1178.97	1209.75	1267.41	1324.81	1344.09	1368.50
1386.26	1403.41	1444.69	1642.21	1668.88	1813.09
1871.57	3047.09	3072.14	3134.06	3564.63	3617.89
3651.40	3743.73	3832.81			

- Conformer (47); ZPE = 29918.815 cm^{-1}:

34.00	66.74	105.13	142.70	224.33	239.75
262.10	302.86	324.89	424.85	495.66	546.02
579.63	583.18	645.12	719.90	770.63	825.98
841.88	911.27	982.50	1033.63	1102.65	1153.53
1173.87	1177.16	1238.62	1291.93	1334.67	1359.57
1392.02	1462.00	1471.97	1625.97	1641.25	1818.83
1874.49	3037.25	3050.06	3124.90	3545.13	3639.44
3640.99	3772.71	3845.88			

- Conformer (48); ZPE = 30032.009 cm^{-1}:

43.37	62.03	92.85	202.23	218.98	243.86
313.71	354.48	400.19	408.51	501.28	558.45
589.83	600.67	657.16	700.78	783.94	816.29
839.71	913.16	970.28	1020.46	1106.98	1152.34
1157.58	1202.57	1238.53	1294.78	1332.98	1355.84
1392.68	1448.93	1467.91	1634.58	1640.26	1812.09
1887.02	3033.73	3050.79	3136.37	3554.16	3623.39
3651.79	3755.84	3840.68			

- Conformer (49); ZPE = 29992.251 cm^{-1}:

47.36	68.20	92.53	139.57	245.62	249.91
281.41	320.77	323.08	444.08	497.59	572.90
586.36	601.01	619.19	740.32	792.02	811.16
849.44	911.01	960.96	1058.41	1088.74	1103.21
1168.89	1171.67	1248.41	1294.12	1341.34	1359.56
1396.38	1420.74	1476.28	1625.28	1649.90	1819.97
1895.30	3055.20	3097.19	3129.68	3546.17	3632.91
3640.06	3766.78	3843.84			

- Conformer (50); ZPE = 30145.924 cm^{-1}:

16.81	66.70	131.28	195.35	248.84	281.19
296.85	346.70	443.19	479.08	516.79	554.45
588.89	609.99	704.40	719.14	793.47	817.87
867.89	944.98	978.94	997.71	1098.39	1146.82
1165.18	1214.58	1289.36	1324.08	1341.33	1370.37
1405.93	1418.12	1453.79	1609.02	1652.17	1824.48
1898.24	3036.31	3045.22	3131.50	3531.65	3591.09
3612.46	3728.96	3802.27			

- Conformer (51); ZPE = 30055.147 cm^{-1}:

37.92	50.61	96.82	184.62	201.00	256.33
291.94	358.55	372.27	399.44	533.98	553.21
598.04	617.53	643.60	749.44	802.90	809.21
882.17	890.89	955.00	1015.67	1100.74	1107.73
1168.25	1200.79	1244.86	1303.12	1336.81	1364.92
1399.90	1439.30	1472.20	1630.09	1657.30	1811.47
1890.40	3057.58	3102.50	3138.78	3538.49	3622.76
3626.25	3757.46	3837.43			

- Conformer (52); ZPE = 29932.237 cm^{-1}:

43.63	71.40	83.07	156.46	235.48	271.41
316.89	338.46	374.71	439.27	476.84	502.41
524.33	602.81	650.41	680.56	736.71	839.14
870.17	910.34	941.64	1059.19	1108.10	1167.90
1174.67	1217.55	1237.91	1272.19	1318.25	1359.85
1410.51	1435.17	1444.46	1618.83	1627.50	1807.43
1900.37	2978.63	3034.29	3098.57	3562.88	3638.66
3660.11	3772.54	3892.77			

- Conformer (53); ZPE = 29992.872 cm^{-1}:

16.07	49.16	76.17	158.79	184.37	212.40
308.84	360.65	385.00	425.63	503.77	531.64
547.32	591.61	662.53	731.88	751.66	865.18
901.04	915.45	957.64	1039.98	1097.81	1162.26
1169.27	1185.57	1257.58	1297.58	1321.14	1346.46
1418.95	1437.26	1469.04	1629.04	1670.40	1808.78
1895.44	3042.18	3055.38	3109.22	3532.28	3626.12
3633.83	3767.44	3875.97			

- Conformer (54); ZPE = 29869.797 cm^{-1}:

23.75	90.01	96.34	150.40	221.37	248.65
262.87	280.78	339.78	391.77	494.96	549.34
578.72	598.67	628.78	671.03	781.25	812.40
833.98	893.87	964.65	1075.59	1100.72	1105.22
1167.43	1186.12	1248.96	1278.63	1351.52	1364.40
1402.11	1431.15	1450.49	1624.85	1649.20	1813.43
1899.55	3038.85	3084.32	3104.68	3542.39	3636.37
3641.06	3776.90	3852.27			

- Conformer (55); ZPE = 29909.168 cm^{-1}:

39.52	53.62	93.01	145.38	225.39	286.63
290.62	338.41	357.84	419.49	495.93	523.82
534.35	612.10	645.04	693.38	750.66	824.44
877.80	899.57	926.45	1057.22	1102.66	1146.13
1163.13	1197.27	1248.63	1280.61	1324.87	1349.72
1416.31	1440.57	1451.92	1617.85	1625.71	1807.31
1902.10	3026.51	3034.63	3081.21	3551.51	3638.68
3653.24	3773.06	3894.04			

- Conformer (56); ZPE = 29889.104 cm^{-1}:

42.73	78.99	95.32	149.18	233.28	253.38
292.08	307.33	358.41	387.54	510.25	525.87
553.41	595.17	646.36	666.55	756.90	833.61
873.63	893.83	955.40	1057.57	1101.27	1155.10
1175.99	1213.76	1235.34	1262.71	1308.64	1369.66
1409.83	1438.18	1460.74	1626.45	1636.37	1796.30
1903.35	2955.02	3059.26	3108.85	3553.42	3639.84
3645.62	3773.80	3881.95			

- Conformer (57); ZPE = 30150.014 cm^{-1}:

55.35	95.31	119.95	181.54	225.13	261.35
297.02	342.25	423.23	492.17	507.83	561.75
602.54	658.18	680.58	716.35	757.62	834.38
894.98	928.36	996.16	1036.75	1092.95	1169.78
1188.29	1210.76	1232.09	1316.03	1340.90	1363.22
1387.48	1413.37	1459.91	1622.54	1674.42	1850.43
1891.44	3024.41	3054.84	3118.14	3536.55	3590.60
3611.98	3708.93	3772.19			

- Conformer (58); ZPE = 30045.073 cm^{-1}:

42.46	68.79	144.71	192.24	204.99	246.82
297.66	353.52	443.19	480.42	495.05	517.91
549.13	586.76	659.26	695.84	798.63	803.60
863.37	884.03	959.97	1030.10	1115.61	1153.87
1176.44	1214.42	1264.06	1299.27	1345.95	1372.04
1398.07	1413.74	1445.65	1648.84	1671.10	1810.49
1876.47	3004.44	3052.47	3126.06	3556.08	3586.71
3637.61	3721.16	3881.19			

- Conformer (59); ZPE = 30023.376 cm^{-1}:

52.29	86.32	107.37	173.69	205.45	248.46
303.14	390.89	409.63	443.15	492.80	512.31
548.20	590.77	634.08	725.43	805.91	810.58
886.80	909.31	969.82	998.90	1104.61	1145.67
1169.89	1179.09	1253.31	1291.98	1324.83	1372.30
1386.57	1419.28	1471.49	1635.24	1681.70	1811.45
1907.72	2974.04	3065.70	3133.03	3535.97	3622.24
3623.71	3750.02	3881.62			

- Conformer (60); ZPE = 30011.359 cm^{-1}:

38.33	55.93	101.50	162.74	208.55	267.48
346.65	367.50	414.20	429.00	506.65	539.26
560.04	598.95	664.21	731.47	746.87	838.45
856.81	912.91	966.21	1054.03	1100.74	1152.62
1185.91	1200.38	1235.54	1272.66	1319.50	1350.31
1399.76	1429.84	1469.43	1623.83	1635.35	1823.82
1885.07	2979.54	3064.44	3125.84	3556.47	3612.51
3651.15	3743.70	3836.55			

- Conformer (61); ZPE = 30017.559 cm^{-1}:

24.55	45.82	115.72	189.78	213.90	246.33
300.07	364.16	421.72	436.79	503.19	523.66
549.25	601.22	667.85	705.22	802.92	817.02
868.89	898.09	967.77	1008.70	1119.97	1156.75
1174.88	1216.58	1248.14	1271.12	1324.92	1355.16
1416.46	1450.79	1467.55	1642.37	1648.41	1808.59
1879.09	3005.99	3046.26	3127.55	3541.27	3605.06
3628.50	3734.50	3892.61			

- Conformer (62); ZPE = 30023.561 cm^{-1}:

27.43	62.80	120.55	160.89	195.87	257.63
284.19	369.01	437.99	463.90	482.09	501.81
578.89	605.62	623.15	754.39	759.96	815.31
856.01	919.51	971.53	1050.88	1108.15	1141.64
1172.39	1209.60	1233.45	1290.11	1306.24	1369.65
1414.45	1444.46	1467.06	1630.48	1656.42	1805.60
1896.00	2952.54	3076.24	3136.33	3552.60	3613.73
3641.63	3741.94	3886.99			

- Conformer (63); ZPE = 29885.654 cm^{-1}:

61.53	73.24	89.45	150.88	215.12	259.61
303.57	351.80	412.76	462.33	492.80	519.65
544.04	587.96	603.87	695.26	756.14	801.89
818.97	927.59	950.28	996.13	1090.10	1143.37
1171.31	1206.61	1212.45	1300.75	1310.22	1345.74
1413.43	1427.29	1474.15	1630.54	1634.91	1823.74
1904.34	2920.23	3042.47	3118.34	3572.24	3634.24
3671.70	3761.65	3886.59			

- Conformer (64); ZPE = 29998.780 cm^{-1}:

54.06	70.50	112.93	197.82	237.83	257.20
295.30	358.69	391.95	438.24	487.52	531.20
553.35	600.91	681.25	728.70	766.01	809.53
844.15	925.54	955.07	1009.34	1110.57	1142.97
1159.01	1204.36	1246.02	1290.38	1338.72	1357.09
1405.92	1439.85	1451.07	1618.93	1649.19	1813.75
1918.39	2943.93	3051.07	3128.37	3547.27	3599.51
3635.43	3742.94	3895.68			

9.5 Cartesian coordinates of Asn conformers

Each line starting by an asterisk contains the relative energy of the corresponding conformer and the dihedral angle for each of the target torsions (separated by underscores). Cartesian coordinates are given in Å.

```
17
 * E =  +0.000 kcal/mol ; (1) 354_018_097_293_098
 H   +0.00000   +0.00000   +0.00000
 O   +0.00000   +0.00000   +0.97486
 C   +1.26693   +0.00000   +1.34904
 C   +2.27146   -0.14096   +0.19116
 N   +1.62157   +0.17480   -1.07460
 H   +1.80467   +1.14128   -1.33656
 O   +1.61431   +0.08215   +2.49535
 C   +3.53046   +0.68299   +0.50679
 H   +2.54602   -1.20287   +0.18835
 H   +1.95430   -0.41530   -1.82417
 C   +3.17514   +2.15878   +0.50095
 H   +3.93027   +0.36851   +1.47295
 H   +4.27730   +0.51603   -0.27199
 N   +2.94556   +2.70897   +1.71171
 H   +2.58754   +3.64922   +1.74387
 H   +2.83093   +2.11625   +2.51842
 O   +3.06679   +2.77308   -0.54509
17
 * E =  +1.913 kcal/mol ; (2) 355_023_082_168_180
 H   +0.00000   +0.00000   +0.00000
 O   +0.00000   +0.00000   +0.97213
 C   +1.27054   +0.00000   +1.36071
```

C	+2.27313	−0.11537	+0.20768
N	+1.65912	+0.33292	−1.03631
H	+1.69556	+1.34410	−1.11495
O	+1.59968	+0.05112	+2.50997
C	+3.58880	+0.55527	+0.57574
H	+2.46381	−1.19047	+0.09215
H	+2.17631	−0.04948	−1.81945
C	+4.67421	+0.20173	−0.42613
H	+3.46533	+1.64563	+0.61015
H	+3.89128	+0.24630	+1.58213
N	+5.88281	+0.76188	−0.18585
H	+6.64952	+0.53848	−0.79689
H	+6.05688	+1.30798	+0.63775
O	+4.47036	−0.52443	−1.37762

17

∗ E = +2.552 kcal/mol ; (3) 180_197_199_311_332

H	+0.00000	+0.00000	+0.00000
O	+0.00000	+0.00000	+0.96392
C	+1.26447	+0.00000	+1.38880
C	+1.35602	−0.01128	+2.90560
N	+2.70643	+0.39316	+3.26752
H	+2.92960	+0.12266	+4.21601
O	+2.21260	−0.02754	+0.65670
C	+0.29350	+0.88307	+3.55524
H	+1.12883	−1.05286	+3.19064
H	+3.37894	−0.02574	+2.63475
C	+0.17613	+2.29539	+2.98965
H	−0.69631	+0.43423	+3.45762
H	+0.50919	+0.96393	+4.62688
N	+1.31206	+2.80259	+2.45519
H	+1.28695	+3.74689	+2.10983
H	+2.19526	+2.33515	+2.60989
O	−0.87636	+2.89317	+3.01669

17

∗ E = +2.594 kcal/mol ; (4) 181_198_301_301_187

H	+0.00000	+0.00000	+0.00000
O	+0.00000	+0.00000	+0.96374
C	+1.28101	+0.00000	+1.37153
C	+1.41159	−0.02147	+2.89340
N	+2.72241	+0.39400	+3.34592

H	+3.42914	−0.21258	+2.94679
O	+2.20450	−0.05056	+0.61394
C	+0.31871	+0.79660	+3.57452
H	+1.26249	−1.07165	+3.18014
H	+2.90657	+1.32195	+2.97512
C	+0.36293	+2.24401	+3.11662
H	−0.67259	+0.39208	+3.34628
H	+0.46660	+0.74814	+4.65792
N	−0.47080	+3.08800	+3.76702
H	−0.48332	+4.05757	+3.50144
H	−1.05660	+2.77782	+4.51971
O	+1.09328	+2.60874	+2.21854

17

∗ E = +2.603 kcal/mol ; (5) 180_020_296_298_183

H	+0.00000	+0.00000	+0.00000
O	+0.00000	+0.00000	+0.96367
C	+1.27467	+0.00000	+1.36742
C	+1.42176	+0.00992	+2.88905
N	+0.27170	−0.43598	+3.64793
H	−0.51546	+0.18234	+3.49953
O	+2.20079	+0.04801	+0.60616
C	+2.66316	−0.79796	+3.25284
H	+1.61861	+1.05858	+3.14908
H	−0.00209	−1.35652	+3.31999
C	+2.50245	−2.24126	+2.80553
H	+3.54731	−0.37873	+2.76235
H	+2.81291	−0.76272	+4.33606
N	+3.52146	−3.06885	+3.12966
H	+3.48234	−4.02652	+2.82622
H	+4.34718	−2.73393	+3.59009
O	+1.52071	−2.62038	+2.19908

17

∗ E = +2.608 kcal/mol ; (6) 002_171_286_084_161

H	+0.00000	+0.00000	+0.00000
O	+0.00000	+0.00000	+0.97649
C	+1.23942	+0.00000	+1.44648
C	+2.36256	+0.05401	+0.39421
N	+3.66596	+0.25589	+0.99441
H	+3.75753	+1.21759	+1.29904
O	+1.47786	−0.05717	+2.61920

C	+2.37911	−1.23783	−0.44449
H	+2.14791	+0.88838	−0.28490
H	+3.72217	−0.29825	+1.84444
C	+1.40005	−1.22212	−1.60079
H	+3.39688	−1.36684	−0.82565
H	+2.15872	−2.11421	+0.17892
N	+1.64047	−2.11207	−2.58090
H	+1.00265	−2.16350	−3.35723
H	+2.43665	−2.72226	−2.55777
O	+0.44962	−0.45604	−1.64857

17

∗ E = +2.644 kcal/mol ; (7) 179_169_298_165_034

H	+0.00000	+0.00000	+0.00000
O	+0.00000	+0.00000	+0.96409
C	+1.26886	+0.00000	+1.38804
C	+1.36730	+0.03316	+2.90797
N	+2.72116	−0.21578	+3.37176
H	+3.02741	−1.14008	+3.09008
O	+2.21075	−0.01552	+0.64798
C	+0.84928	+1.38922	+3.41004
H	+0.70777	−0.75458	+3.29416
H	+3.35490	+0.43667	+2.91940
C	+0.53851	+1.46757	+4.90235
H	+1.59403	+2.16226	+3.17834
H	−0.07283	+1.66118	+2.89365
N	+1.33159	+0.72855	+5.70841
H	+1.18103	+0.78522	+6.70081
H	+2.12044	+0.22853	+5.32285
O	−0.36578	+2.16462	+5.30800

17

∗ E = +2.812 kcal/mol ; (8) 001_347_152_172_238

H	+0.00000	+0.00000	+0.00000
O	+0.00000	+0.00000	+0.97436
C	+1.27097	+0.00000	+1.35359
C	+2.27762	+0.02942	+0.19382
N	+1.61183	−0.25485	−1.07049
H	+2.10805	+0.19731	−1.83348
O	+1.61886	+0.01408	+2.49814
C	+2.89474	+1.43564	+0.18242
H	+3.06232	−0.69516	+0.45159

H +1.59833 −1.24902 −1.26188
C +4.05312 +1.52088 −0.79271
H +2.13229 +2.15233 −0.14542
H +3.19463 +1.69285 +1.20122
N +5.24323 +1.88312 −0.26683
H +6.03976 +1.95382 −0.87739
H +5.35488 +2.07830 +0.71068
O +3.91143 +1.26943 −1.97408
17
* E = +3.266 kcal/mol ; (9) 183_194_205_175_165
H +0.00000 +0.00000 +0.00000
O +0.00000 +0.00000 +0.96373
C +1.27892 +0.00000 +1.37197
C +1.40803 −0.07875 +2.87744
N +2.76508 +0.25183 +3.25540
H +2.98984 −0.17864 +4.14283
O +2.20025 +0.01946 +0.60724
C +0.42171 +0.84802 +3.58293
H +1.12456 −1.11378 +3.13851
H +3.41777 −0.05708 +2.54549
C +0.50963 +0.63733 +5.08559
H +0.65516 +1.88998 +3.33757
H −0.60639 +0.64367 +3.26504
N −0.07715 +1.59890 +5.83688
H −0.05503 +1.51121 +6.83815
H −0.46985 +2.42492 +5.42540
O +1.04319 −0.33648 +5.57375
17
* E = +3.375 kcal/mol ; (10) 354_023_086_093_291
H +0.00000 +0.00000 +0.00000
O +0.00000 +0.00000 +0.97248
C +1.26380 +0.00000 +1.36190
C +2.28277 −0.14327 +0.22168
N +1.66083 +0.30459 −1.02162
H +1.75292 +1.30823 −1.13540
O +1.58100 +0.08107 +2.51692
C +3.62505 +0.52634 +0.57736
H +2.44039 −1.22672 +0.12526
H +2.09027 −0.13307 −1.82595
C +4.62347 −0.44145 +1.20480

```
 H    +4.09597    +0.88662    −0.33866
 H    +3.45137    +1.37424    +1.24882
 N    +4.30808    −0.86456    +2.44580
 H    +4.91552    −1.52782    +2.89500
 H    +3.45395    −0.56032    +2.89012
 O    +5.60860    −0.80696    +0.59811
17
 * E =  +3.505  kcal/mol ; (11) 179_042_191_171_176
 H    +0.00000    +0.00000    +0.00000
 O    +0.00000    +0.00000    +0.96363
 C    +1.27563    +0.00000    +1.37744
 C    +1.38198    +0.02694    +2.88933
 N    +0.40326    −0.86992    +3.48409
 H    +0.37336    −0.69278    +4.48198
 O    +2.20054    +0.01665    +0.61587
 C    +2.78814    −0.36909    +3.30744
 H    +1.22329    +1.08241    +3.17731
 H    −0.51889    −0.69908    +3.10314
 C    +2.99419    −0.15247    +4.79500
 H    +2.95484    −1.42462    +3.06441
 H    +3.52845    +0.21350    +2.74908
 N    +4.19740    −0.55252    +5.26999
 H    +4.39590    −0.43463    +6.24831
 H    +4.88813    −0.97123    +4.67518
 O    +2.14888    +0.34933    +5.50775
17
 * E =  +3.814  kcal/mol ; (12) 178_033_318_296_108
 H    +0.00000    +0.00000    +0.00000
 O    +0.00000    +0.00000    +0.96402
 C    +1.26983    +0.00000    +1.38216
 C    +1.38840    +0.06039    +2.90477
 N    +0.34236    −0.60322    +3.65010
 H    −0.57026    −0.43387    +3.24940
 O    +2.19939    +0.00009    +0.61949
 C    +2.77203    −0.44596    +3.32715
 H    +1.34874    +1.13742    +3.13138
 H    +0.51229    −1.60285    +3.69594
 C    +2.95160    −1.91434    +2.97305
 H    +3.54626    +0.16743    +2.86247
 H    +2.84339    −0.35873    +4.41352
```

N +3.80361 −2.17205 +1.95259
H +3.85675 −3.12278 +1.62550
H +4.03583 −1.43337 +1.30970
O +2.36471 −2.78989 +3.57870
17
* E = +4.087 kcal/mol ; (13) 354_295_296_276_212
H +0.00000 +0.00000 +0.00000
O +0.00000 +0.00000 +0.97665
C +1.23866 +0.00000 +1.45678
C +2.38193 −0.15198 +0.43676
N +2.40458 −1.43703 −0.24893
H +2.57216 −2.19113 +0.40468
O +1.46139 +0.08057 +2.63159
C +2.44470 +0.98676 −0.59429
H +3.28556 −0.08388 +1.04746
H +1.52827 −1.61963 −0.72486
C +1.51817 +0.78254 −1.77654
H +2.18349 +1.94471 −0.12763
H +3.47414 +1.06954 −0.95501
N +1.94224 +1.27501 −2.95172
H +1.34756 +1.18271 −3.75836
H +2.85064 +1.68748 −3.05640
O +0.43456 +0.22305 −1.67294
17
* E = +4.420 kcal/mol ; (14) 182_058_158_277_084
H +0.00000 +0.00000 +0.00000
O +0.00000 +0.00000 +0.96395
C +1.26581 +0.00000 +1.38915
C +1.33408 −0.04719 +2.90794
N +0.62995 −1.24592 +3.33784
H +0.92007 −1.53244 +4.26529
O +2.20180 +0.04413 +0.63675
C +2.78170 −0.06069 +3.40298
H +0.86292 +0.88777 +3.25637
H −0.37377 −1.13498 +3.30530
C +3.37366 −1.46434 +3.38041
H +3.37808 +0.63099 +2.80222
H +2.80403 +0.26225 +4.44544
N +3.87806 −1.86327 +2.19319
H +4.18658 −2.81641 +2.10538

H	+3.72677	−1.30750	+1.36699
O	+3.37623	−2.15524	+4.37839

17
∗ E = +4.425 kcal/mol ; (15) 185_185_327_299_098

H	+0.00000	+0.00000	+0.00000
O	+0.00000	+0.00000	+0.96436
C	+1.28766	+0.00000	+1.37136
C	+1.41643	−0.12841	+2.88671
N	+2.77309	−0.00639	+3.35652
H	+3.42231	−0.37997	+2.67472
O	+2.20137	+0.04708	+0.60244
C	+0.49312	+0.86483	+3.61276
H	+1.03618	−1.13766	+3.10888
H	+3.00951	+0.96900	+3.51148
C	+0.86709	+2.29197	+3.25242
H	−0.54961	+0.65025	+3.36857
H	+0.64783	+0.73965	+4.68623
N	+0.11899	+2.85744	+2.27472
H	+0.37153	+3.77267	+1.94237
H	−0.60057	+2.33952	+1.80341
O	+1.80069	+2.86180	+3.77738

17
∗ E = +4.446 kcal/mol ; (16) 180_052_304_163_032

H	+0.00000	+0.00000	+0.00000
O	+0.00000	+0.00000	+0.96379
C	+1.27773	+0.00000	+1.38340
C	+1.35134	−0.00054	+2.90747
N	+0.54725	−1.07279	+3.49480
H	−0.41617	−1.01034	+3.18716
O	+2.20650	−0.00722	+0.63043
C	+2.80243	−0.07397	+3.36724
H	+0.91138	+0.95122	+3.23692
H	+0.90325	−1.97827	+3.20632
C	+3.05486	+0.31254	+4.82029
H	+3.18308	−1.09207	+3.21221
H	+3.42513	+0.57907	+2.75451
N	+2.05870	+0.02857	+5.69079
H	+2.21011	+0.23380	+6.66325
H	+1.24668	−0.48867	+5.38637
O	+4.09519	+0.83831	+5.15088

17
∗ E = +4.473 kcal/mol ; (17) 003_343_150_091_320
H +0.00000 +0.00000 +0.00000
O +0.00000 +0.00000 +0.97386
C +1.26331 +0.00000 +1.35952
C +2.28730 +0.07466 +0.21815
N +1.62354 −0.26644 −1.03818
H +2.05116 +0.19980 −1.82694
O +1.58518 −0.01721 +2.51713
C +2.85737 +1.50618 +0.14304
H +3.09877 −0.61791 +0.47623
H +1.64543 −1.26271 −1.21714
C +4.11256 +1.78459 +0.96951
H +3.14754 +1.72794 −0.88710
H +2.09227 +2.24065 +0.42196
N +4.16579 +1.22348 +2.19394
H +4.96467 +1.43656 +2.76671
H +3.37865 +0.72376 +2.58781
O +4.99327 +2.48066 +0.51096
17
∗ E = +4.573 kcal/mol ; (18) 183_317_297_164_032
H +0.00000 +0.00000 +0.00000
O +0.00000 +0.00000 +0.96392
C +1.27771 +0.00000 +1.38390
C +1.36798 −0.06636 +2.90198
N +0.42397 +0.80687 +3.59007
H +0.59984 +1.78067 +3.37367
O +2.20951 +0.01842 +0.63483
C +1.15159 −1.53003 +3.31198
H +2.38709 +0.23702 +3.16191
H −0.52372 +0.59669 +3.29417
C +1.56949 −1.89699 +4.73354
H +0.08919 −1.77956 +3.19146
H +1.71374 −2.19631 +2.65332
N +1.43257 −0.92313 +5.65998
H +1.66472 −1.14262 +6.61327
H +0.99858 −0.04514 +5.41368
O +1.98843 −3.00549 +4.98461
17
∗ E = +4.733 kcal/mol ; (19) 182_073_297_062_198

H	+0.00000	+0.00000	+0.00000
O	+0.00000	+0.00000	+0.96377
C	+1.27270	+0.00000	+1.38136
C	+1.37030	−0.05854	+2.89985
N	+1.03268	−1.38457	+3.40314
H	+0.07227	−1.62028	+3.18524
O	+2.20661	−0.02492	+0.62887
C	+2.77327	+0.32971	+3.32567
H	+0.65505	+0.66320	+3.30480
H	+1.62818	−2.09258	+2.98809
C	+3.07796	+1.75428	+2.89529
H	+2.84827	+0.25843	+4.41538
H	+3.50534	−0.36060	+2.89088
N	+4.39052	+2.08926	+2.87338
H	+4.64163	+3.00704	+2.54810
H	+5.10802	+1.39391	+2.96101
O	+2.19601	+2.54188	+2.62938

17

* E = +4.735 kcal/mol ; (20) 182_071_297_062_201

H	+0.00000	+0.00000	+0.00000
O	+0.00000	+0.00000	+0.96377
C	+1.27271	+0.00000	+1.38165
C	+1.36974	−0.05409	+2.90050
N	+0.98868	−1.36373	+3.41454
H	+0.02154	−1.57040	+3.19711
O	+2.20641	−0.02348	+0.62887
C	+2.78459	+0.29313	+3.32314
H	+0.67952	+0.69447	+3.30077
H	+1.56159	−2.09469	+3.00768
C	+3.13132	+1.70578	+2.88496
H	+2.85608	+0.22881	+4.41350
H	+3.49499	−0.42325	+2.89484
N	+4.45469	+1.98446	+2.80354
H	+4.72770	+2.89127	+2.46512
H	+5.14228	+1.25494	+2.83635
O	+2.27249	+2.53233	+2.66594

17

* E = +4.789 kcal/mol ; (21) 178_020_187_305_338

H	+0.00000	+0.00000	+0.00000
O	+0.00000	+0.00000	+0.96409

C	+1.27758	+0.00000	+1.37327
C	+1.41782	+0.03792	+2.88763
N	+0.18913	−0.39121	+3.54784
H	+0.25688	−0.24442	+4.54722
O	+2.20560	+0.01882	+0.62059
C	+2.61190	−0.82132	+3.30727
H	+1.67297	+1.08810	+3.10858
H	−0.61383	+0.12039	+3.20361
C	+2.60678	−2.27023	+2.82493
H	+3.53546	−0.38270	+2.92562
H	+2.68599	−0.83053	+4.40084
N	+1.39494	−2.78105	+2.49398
H	+1.36264	−3.74871	+2.22081
H	+0.55226	−2.30492	+2.78616
O	+3.63916	−2.89523	+2.74129

17

∗ E = +4.870 kcal/mol ; (22) 177_159_048_175_174

H	+0.00000	+0.00000	+0.00000
O	+0.00000	+0.00000	+0.96386
C	+1.27730	+0.00000	+1.37672
C	+1.38840	+0.06976	+2.88434
N	+2.68414	−0.42375	+3.27725
H	+3.42177	−0.00157	+2.72676
O	+2.20534	+0.00327	+0.62058
C	+1.08010	+1.52411	+3.28681
H	+0.61136	−0.56831	+3.31963
H	+2.83727	−0.27518	+4.26569
C	+1.05443	+1.67580	+4.79944
H	+1.83981	+2.19499	+2.86555
H	+0.10676	+1.83707	+2.88812
N	+0.90711	+2.94768	+5.24348
H	+0.86445	+3.11199	+6.23449
H	+0.81401	+3.72255	+4.61337
O	+1.15401	+0.73093	+5.55154

17

∗ E = +4.973 kcal/mol ; (23) 181_055_183_081_266

H	+0.00000	+0.00000	+0.00000
O	+0.00000	+0.00000	+0.96405
C	+1.26845	+0.00000	+1.38773
C	+1.34769	−0.02581	+2.90483

N +0.57405 −1.17507 +3.36280
H +0.56347 −1.20373 +4.37481
O +2.20071 +0.02201 +0.63231
C +2.79749 −0.14882 +3.37153
H +0.94122 +0.94004 +3.25404
H −0.38493 −1.11566 +3.04203
C +3.50841 +1.19753 +3.35879
H +2.79605 −0.48205 +4.41184
H +3.31426 −0.89528 +2.76237
N +4.20164 +1.48196 +2.23230
H +4.60696 +2.39896 +2.14887
H +4.05171 +0.93044 +1.40326
O +3.44040 +1.94963 +4.30780
17
∗ E = +5.242 kcal/mol ; (24) 181_014_061_298_183
H +0.00000 +0.00000 +0.00000
O +0.00000 +0.00000 +0.96382
C +1.26974 +0.00000 +1.35842
C +1.45873 −0.02023 +2.87010
N +0.23607 −0.34987 +3.56786
H −0.08207 −1.27248 +3.29342
O +2.19649 +0.04898 +0.59500
C +2.67109 −0.91143 +3.17056
H +1.73331 +1.01122 +3.13023
H +0.37297 −0.33418 +4.56975
C +2.42010 −2.33165 +2.69133
H +3.56058 −0.52314 +2.66244
H +2.87383 −0.91687 +4.24737
N +3.40388 −3.22161 +2.96243
H +3.31501 −4.15865 +2.60830
H +4.28089 −2.93065 +3.35318
O +1.39912 −2.64339 +2.11405
17
∗ E = +5.283 kcal/mol ; (25) 177_041_092_288_102
H +0.00000 +0.00000 +0.00000
O +0.00000 +0.00000 +0.96396
C +1.26468 +0.00000 +1.37028
C +1.41179 +0.06603 +2.89010
N +0.44678 −0.78093 +3.55910
H +0.83700 −1.70273 +3.72937

O +2.19241 +0.00503 +0.60109
C +2.88507 −0.16901 +3.27702
H +1.16513 +1.11005 +3.12751
H +0.18875 −0.39491 +4.45624
C +3.27506 −1.62503 +3.08152
H +3.53278 +0.49607 +2.70103
H +2.99796 +0.04970 +4.34113
N +3.98344 −1.90202 +1.96323
H +4.15951 −2.87059 +1.75319
H +3.99349 −1.23256 +1.21092
O +2.95436 −2.47520 +3.89049
17
∗ E = +5.382 kcal/mol ; (26) 178_160_305_287_023
H +0.00000 +0.00000 +0.00000
O +0.00000 +0.00000 +0.96448
C +1.26526 +0.00000 +1.39131
C +1.35584 +0.04731 +2.91398
N +2.61565 −0.40207 +3.45386
H +2.85089 −1.33222 +3.13280
O +2.20521 −0.02217 +0.64133
C +1.00393 +1.45126 +3.42856
H +0.56147 −0.61827 +3.27285
H +3.37601 +0.21497 +3.19537
C +2.01870 +2.57418 +3.22747
H +0.06222 +1.78700 +2.98038
H +0.84420 +1.39476 +4.50699
N +2.93037 +2.44218 +2.22960
H +3.54797 +3.22180 +2.07594
H +2.92659 +1.68922 +1.55921
O +1.98333 +3.54974 +3.94228
17
∗ E = +5.616 kcal/mol ; (27) 023_212_210_313_295
H +0.00000 +0.00000 +0.00000
O +0.00000 +0.00000 +0.96860
C +1.20876 +0.00000 +1.52361
C +2.39206 +0.52858 +0.69750
N +3.58332 −0.20809 +1.10191
H +4.41311 +0.36207 +0.99830
O +1.36945 −0.32742 +2.66310
C +2.19782 +0.45907 −0.82298

H +2.44515 +1.59406 +0.97603
H +3.49467 −0.46164 +2.08146
C +1.66600 −0.89270 −1.26696
H +1.48274 +1.21206 −1.16313
H +3.15951 +0.66794 −1.30170
N +2.48886 −1.93623 −1.05778
H +2.12722 −2.86236 −1.21713
H +3.28537 −1.80453 −0.44871
O +0.54224 −0.99134 −1.73025
17
∗ E = +5.621 kcal/mol ; (28) 178_161_037_173_242
H +0.00000 +0.00000 +0.00000
O +0.00000 +0.00000 +0.96387
C +1.27728 +0.00000 +1.37730
C +1.38473 +0.05697 +2.88704
N +2.69728 −0.38445 +3.28296
H +3.38807 −0.06950 +2.61139
O +2.20577 −0.00006 +0.62271
C +1.00902 +1.50175 +3.31283
H +0.61894 −0.61975 +3.29082
H +2.92838 −0.02857 +4.20332
C +0.93261 +1.59455 +4.82479
H +1.79568 +2.18235 +2.97056
H +0.06251 +1.79541 +2.84805
N −0.27577 +1.93420 +5.33320
H −0.37696 +1.99494 +6.33225
H −1.06558 +2.12833 +4.74694
O +1.88933 +1.35720 +5.53312
17
∗ E = +5.622 kcal/mol ; (29) 184_203_207_078_217
H +0.00000 +0.00000 +0.00000
O +0.00000 +0.00000 +0.96382
C +1.27339 +0.00000 +1.38042
C +1.39069 −0.09471 +2.88817
N +2.67497 +0.45796 +3.27761
H +3.00165 +0.04567 +4.14105
O +2.20399 +0.04467 +0.62730
C +0.24081 +0.62260 +3.59899
H +1.27690 −1.16977 +3.11019
H +3.36140 +0.27748 +2.55393

C −1.03843 −0.19834 +3.57621
H +0.52283 +0.74375 +4.65059
H +0.09830 +1.62617 +3.18632
N −2.18460 +0.50784 +3.42668
H −3.05416 +0.00429 +3.38821
H −2.17368 +1.48150 +3.18766
O −1.02547 −1.39943 +3.73404
17
∗ E = +5.801 kcal/mol ; (30) 183_194_301_078_213
H +0.00000 +0.00000 +0.00000
O +0.00000 +0.00000 +0.96396
C +1.27435 +0.00000 +1.38301
C +1.39020 −0.06457 +2.90171
N +2.71776 +0.26622 +3.37440
H +3.39597 −0.36910 +2.97055
O +2.20463 +0.04371 +0.62932
C +0.32802 +0.80526 +3.57453
H +1.15780 −1.10151 +3.18148
H +2.98373 +1.19223 +3.05636
C −1.03429 +0.12593 +3.57999
H +0.62996 +0.93549 +4.61898
H +0.27478 +1.79984 +3.11673
N −2.09899 +0.95756 +3.48000
H −3.02153 +0.55795 +3.48743
H −1.99111 +1.93574 +3.28946
O −1.14917 −1.07070 +3.71720
17
∗ E = +5.818 kcal/mol ; (31) 182_333_059_171_176
H +0.00000 +0.00000 +0.00000
O +0.00000 +0.00000 +0.96381
C +1.27592 +0.00000 +1.37389
C +1.41122 −0.05047 +2.88289
N +0.26308 +0.52681 +3.54533
H −0.59352 +0.04554 +3.30058
O +2.20088 −0.02824 +0.61258
C +1.72439 −1.51249 +3.24729
H +2.29257 +0.54781 +3.13336
H +0.40008 +0.48942 +4.54778
C +2.09150 −1.64088 +4.71703
H +0.85590 −2.14893 +3.03295

H	+2.56040	−1.88216	+2.64034
N	+2.29580	−2.90988	+5.14624
H	+2.57659	−3.05729	+6.10044
H	+2.25537	−3.69352	+4.52131
O	+2.19360	−0.68164	+5.45079

17

∗ E = +5.930 kcal/mol ; (32) 178_268_301_057_180

H	+0.00000	+0.00000	+0.00000
O	+0.00000	+0.00000	+0.96365
C	+1.28162	+0.00000	+1.37350
C	+1.41165	+0.05642	+2.89010
N	+1.51945	+1.43920	+3.34397
H	+2.32147	+1.88991	+2.91952
O	+2.20425	+0.06237	+0.61460
C	+0.25682	−0.62252	+3.60672
H	+2.34629	−0.46040	+3.12434
H	+0.69445	+1.96837	+3.08227
C	+0.12706	−2.07178	+3.17695
H	+0.42580	−0.56437	+4.68703
H	−0.68347	−0.09862	+3.39559
N	−0.87896	−2.76461	+3.76372
H	−1.01198	−3.72915	+3.51393
H	−1.49887	−2.33961	+4.42766
O	+0.87979	−2.56853	+2.36791

17

∗ E = +6.206 kcal/mol ; (33) 177_149_049_276_049

H	+0.00000	+0.00000	+0.00000
O	+0.00000	+0.00000	+0.96459
C	+1.26466	+0.00000	+1.38620
C	+1.36826	+0.08455	+2.89420
N	+2.56055	−0.61689	+3.32128
H	+3.36303	−0.32291	+2.77567
O	+2.20175	−0.01154	+0.63193
C	+1.25865	+1.56715	+3.33899
H	+0.48642	−0.42929	+3.29125
H	+2.75813	−0.43431	+4.29745
C	+2.54964	+2.37846	+3.29217
H	+0.49052	+2.08495	+2.75101
H	+0.93797	+1.59891	+4.38224
N	+3.27853	+2.31574	+2.15230

H	+4.13454	+2.84349	+2.12406
H	+3.07305	+1.67641	+1.40109
O	+2.87702	+3.06470	+4.23574

17

∗ E = +6.220 kcal/mol ; (34) 184_081_075_070_270

H	+0.00000	+0.00000	+0.00000
O	+0.00000	+0.00000	+0.96417
C	+1.26064	+0.00000	+1.38508
C	+1.37481	−0.10003	+2.90107
N	+1.22432	−1.51491	+3.21526
H	+2.06799	−2.02588	+2.98156
O	+2.20227	+0.03837	+0.63643
C	+2.69055	+0.52218	+3.38401
H	+0.52489	+0.43581	+3.33662
H	+1.03906	−1.64838	+4.20063
C	+2.68741	+2.03623	+3.23746
H	+2.80419	+0.30520	+4.44807
H	+3.51999	+0.06859	+2.83082
N	+3.15597	+2.50295	+2.05588
H	+3.10545	+3.49154	+1.87807
H	+3.36828	+1.87045	+1.30239
O	+2.26966	+2.75691	+4.11808

17

∗ E = +6.237 kcal/mol ; (35) 180_187_044_301_197

H	+0.00000	+0.00000	+0.00000
O	+0.00000	+0.00000	+0.96328
C	+1.28511	+0.00000	+1.36534
C	+1.38607	+0.00366	+2.88321
N	+2.77355	+0.17172	+3.25061
H	+3.20081	+0.89043	+2.67715
O	+2.20437	−0.04990	+0.60744
C	+0.40919	+1.01379	+3.50090
H	+1.04988	−0.99689	+3.19427
H	+2.87463	+0.41980	+4.22545
C	+0.68255	+2.42083	+2.99915
H	−0.62412	+0.75426	+3.24356
H	+0.49124	+0.97833	+4.59341
N	+0.13160	+3.42082	+3.72972
H	+0.22973	+4.36706	+3.40378
H	−0.44676	+3.23656	+4.52814

O +1.34284 +2.62512 +2.00432
17
∗ E = +6.239 kcal/mol ; (36) 178_042_293_161_140
H +0.00000 +0.00000 +0.00000
O +0.00000 +0.00000 +0.96370
C +1.27826 +0.00000 +1.37554
C +1.38161 +0.04464 +2.89424
N +0.43916 −0.81712 +3.58865
H −0.51262 −0.49774 +3.46367
O +2.20005 −0.02046 +0.60955
C +2.80043 −0.26057 +3.34597
H +1.16441 +1.08013 +3.19072
H +0.48960 −1.76817 +3.24171
C +2.99338 +0.24003 +4.76829
H +3.00393 −1.33425 +3.26062
H +3.51736 +0.25789 +2.70195
N +3.71568 −0.56680 +5.58241
H +3.86393 −0.27813 +6.53411
H +3.99553 −1.48738 +5.30156
O +2.56319 +1.31330 +5.12669
17
∗ E = +6.843 kcal/mol ; (37) 182_094_066_061_180
H +0.00000 +0.00000 +0.00000
O +0.00000 +0.00000 +0.96377
C +1.26341 +0.00000 +1.37602
C +1.40402 −0.04755 +2.88977
N +1.55399 −1.45523 +3.23713
H +2.42808 −1.81962 +2.87478
O +2.20668 −0.02437 +0.63271
C +2.57892 +0.83664 +3.30145
H +0.47566 +0.32853 +3.32704
H +1.54515 −1.58978 +4.23973
C +2.34206 +2.27776 +2.88590
H +2.72961 +0.78248 +4.38555
H +3.49242 +0.46968 +2.81721
N +3.33076 +3.14984 +3.20156
H +3.24643 +4.10281 +2.89189
H +4.20387 +2.83863 +3.58494
O +1.32521 +2.61664 +2.32059
17

∗ E = +6.880 kcal/mol ; (38) 187_203_188_286_071

H	+0.00000	+0.00000	+0.00000
O	+0.00000	+0.00000	+0.96456
C	+1.28743	+0.00000	+1.36816
C	+1.44616	−0.17257	+2.86803
N	+2.73110	+0.37240	+3.23528
H	+2.96711	+0.17571	+4.19798
O	+2.19783	+0.06209	+0.59651
C	+0.33321	+0.46571	+3.70912
H	+1.35862	−1.26863	+3.00465
H	+3.46518	+0.04902	+2.61921
C	+0.45426	+1.98166	+3.75869
H	−0.64589	+0.16098	+3.32811
H	+0.42564	+0.11520	+4.73919
N	+0.15138	+2.61439	+2.59636
H	+0.22910	+3.61612	+2.56289
H	−0.26240	+2.12366	+1.82425
O	+0.81013	+2.56598	+4.75504

17

∗ E = +7.045 kcal/mol ; (39) 182_320_062_080_338

H	+0.00000	+0.00000	+0.00000
O	+0.00000	+0.00000	+0.96423
C	+1.26498	+0.00000	+1.38747
C	+1.37133	−0.05599	+2.89905
N	+0.33704	+0.78595	+3.47645
H	−0.58550	+0.45137	+3.22291
O	+2.20061	−0.01420	+0.63229
C	+1.32503	−1.52907	+3.36462
H	+2.34773	+0.37126	+3.15132
H	+0.40509	+0.78518	+4.48643
C	+2.59610	−2.36870	+3.26753
H	+1.05202	−1.54288	+4.42351
H	+0.52637	−2.06749	+2.83871
N	+3.54380	−1.96384	+2.39209
H	+4.36091	−2.54420	+2.30378
H	+3.39260	−1.22664	+1.71957
O	+2.72567	−3.34683	+3.97155

17

∗ E = +7.057 kcal/mol ; (40) 172_154_294_056_079

H	+0.00000	+0.00000	+0.00000

O	+0.00000	+0.00000	+0.96441
C	+1.26672	+0.00000	+1.39133
C	+1.35910	+0.19779	+2.90095
N	+2.56340	−0.36070	+3.47522
H	+2.56553	−1.37036	+3.39241
O	+2.21409	−0.12057	+0.66970
C	+1.32686	+1.71007	+3.19580
H	+0.48214	−0.27759	+3.35587
H	+3.36732	−0.02918	+2.95050
C	+0.10063	+2.40033	+2.62401
H	+1.38789	+1.83347	+4.28107
H	+2.20542	+2.17700	+2.74205
N	−1.03286	+2.25048	+3.35507
H	−1.89180	+2.63258	+2.99906
H	−1.04514	+1.74358	+4.22033
O	+0.13181	+3.01397	+1.58189

17

∗ E = +7.379 kcal/mol ; (41) 359_169_294_161_045

H	+0.00000	+0.00000	+0.00000
O	+0.00000	+0.00000	+0.96090
C	+1.24668	+0.00000	+1.45688
C	+2.37717	−0.02110	+0.42299
N	+3.67255	+0.24247	+1.02270
H	+3.70932	+1.19161	+1.37707
O	+1.44136	+0.00218	+2.63331
C	+2.38319	−1.38307	−0.29328
H	+2.17232	+0.76560	−0.31791
H	+3.78207	−0.35751	+1.83601
C	+3.13091	−1.39049	−1.62320
H	+2.83818	−2.12326	+0.37684
H	+1.37159	−1.73165	−0.51465
N	+4.32639	−0.76415	−1.61423
H	+4.86285	−0.74484	−2.46426
H	+4.65536	−0.31381	−0.77167
O	+2.65943	−1.93149	−2.59992

17

∗ E = +7.419 kcal/mol ; (42) 173_286_298_063_269

H	+0.00000	+0.00000	+0.00000
O	+0.00000	+0.00000	+0.96420
C	+1.29174	+0.00000	+1.37189

C	+1.41256	+0.16271	+2.88185
N	+1.13262	+1.53970	+3.26615
H	+1.79861	+2.18240	+2.85540
O	+2.20190	−0.04713	+0.59881
C	+0.47625	−0.77312	+3.64907
H	+2.45048	−0.07017	+3.13615
H	+0.20179	+1.81487	+2.97289
C	+0.81842	−2.22970	+3.38298
H	+0.61591	−0.58192	+4.71384
H	−0.56231	−0.54929	+3.38092
N	+0.15925	−2.79266	+2.33868
H	+0.38561	−3.73667	+2.07618
H	−0.50032	−2.26849	+1.79239
O	+1.64697	−2.82270	+4.03489

17

∗ E = +7.666 kcal/mol ; (43) 179_175_304_106_312

H	+0.00000	+0.00000	+0.00000
O	+0.00000	+0.00000	+0.96432
C	+1.28190	+0.00000	+1.39046
C	+1.38115	+0.02194	+2.91046
N	+2.74476	−0.08703	+3.37658
H	+3.18174	−0.92395	+3.00843
O	+2.20495	−0.00949	+0.63220
C	+0.72229	+1.30530	+3.47691
H	+0.81839	−0.84977	+3.27112
H	+3.29349	+0.69367	+3.03169
C	−0.62356	+1.11324	+4.16537
H	+1.39005	+1.72078	+4.23259
H	+0.59307	+2.05968	+2.69126
N	−1.53585	+0.37412	+3.48491
H	−2.45040	+0.27269	+3.89031
H	−1.38172	+0.09486	+2.53120
O	−0.85103	+1.59564	+5.25104

17

∗ E = +7.743 kcal/mol ; (44) 179_133_298_072_000

H	+0.00000	+0.00000	+0.00000
O	+0.00000	+0.00000	+0.96512
C	+1.27897	+0.00000	+1.38857
C	+1.40706	+0.03324	+2.90969
N	+2.36575	−0.95347	+3.37673

H	+2.06813	−1.89418	+3.14759
O	+2.20801	−0.03261	+0.63681
C	+1.89229	+1.41341	+3.36370
H	+0.43038	−0.19128	+3.35327
H	+3.25929	−0.80137	+2.91920
C	+0.95484	+2.60921	+3.28208
H	+2.18797	+1.32558	+4.41307
H	+2.79895	+1.67961	+2.80797
N	−0.29885	+2.40165	+2.80612
H	−0.89877	+3.20647	+2.73754
H	−0.59037	+1.54762	+2.36455
O	+1.32736	+3.69475	+3.66581

17
∗ E = +8.017 kcal/mol ; (45) 179_000_321_182_287

H	+0.00000	+0.00000	+0.00000
O	+0.00000	+0.00000	+0.96437
C	+1.27508	+0.00000	+1.37278
C	+1.43000	+0.01419	+2.89197
N	+0.22403	+0.02081	+3.68232
H	−0.49532	+0.60888	+3.28421
O	+2.20207	+0.00364	+0.61308
C	+2.30399	−1.18710	+3.27893
H	+1.99417	+0.93695	+3.09724
H	−0.15040	−0.91307	+3.80261
C	+2.45658	−1.27256	+4.78813
H	+1.81918	−2.11396	+2.95926
H	+3.27153	−1.10973	+2.77565
N	+3.27395	−0.34018	+5.33773
H	+3.34146	−0.29017	+6.33948
H	+3.71447	+0.37126	+4.78448
O	+1.87958	−2.11045	+5.44256

17
∗ E = +8.121 kcal/mol ; (46) 179_348_308_285_021

H	+0.00000	+0.00000	+0.00000
O	+0.00000	+0.00000	+0.96502
C	+1.28722	+0.00000	+1.37272
C	+1.44954	+0.03204	+2.89483
N	+0.27886	+0.33439	+3.68424
H	−0.18196	+1.18054	+3.37784
O	+2.20111	−0.02288	+0.60252

C	+2.12809	−1.25663	+3.37452
H	+2.18496	+0.83159	+3.04809
H	−0.39846	−0.41762	+3.67323
C	+1.30122	−2.53636	+3.41707
H	+2.99984	−1.46139	+2.74399
H	+2.49050	−1.10608	+4.39316
N	+0.17974	−2.60000	+2.64797
H	−0.30941	−3.47939	+2.63195
H	−0.09719	−1.88752	+1.99489
O	+1.65031	−3.46273	+4.11087

17

∗ E = +8.212 kcal/mol ; (47) 172_153_038_059_075

H	+0.00000	+0.00000	+0.00000
O	+0.00000	+0.00000	+0.96430
C	+1.26548	+0.00000	+1.38829
C	+1.35835	+0.20225	+2.88948
N	+2.57493	−0.42462	+3.35378
H	+3.31294	−0.28631	+2.67150
O	+2.21340	−0.10355	+0.66505
C	+1.29132	+1.72627	+3.16635
H	+0.48449	−0.28268	+3.34328
H	+2.87557	−0.04991	+4.24354
C	+0.02193	+2.37631	+2.64101
H	+1.37497	+1.87574	+4.24906
H	+2.13895	+2.21367	+2.67844
N	−1.09508	+2.12704	+3.37151
H	−1.97969	+2.45495	+3.02376
H	−1.08258	+1.52565	+4.17425
O	+0.01038	+3.04942	+1.63619

17

∗ E = +8.311 kcal/mol ; (48) 177_165_043_110_304

H	+0.00000	+0.00000	+0.00000
O	+0.00000	+0.00000	+0.96428
C	+1.28314	+0.00000	+1.38364
C	+1.38189	+0.08164	+2.89101
N	+2.72590	−0.28844	+3.26456
H	+3.40460	+0.13757	+2.64434
O	+2.20440	−0.00617	+0.62429
C	+0.91513	+1.50619	+3.33926
H	+0.67803	−0.65372	+3.30320

H	+2.93163	−0.03115	+4.22008
C	−0.41662	+1.52873	+4.07513
H	+1.65494	+1.91700	+4.02743
H	+0.85282	+2.18731	+2.48169
N	−1.45793	+0.97577	+3.40686
H	−2.35847	+0.96496	+3.85364
H	−1.35632	+0.61047	+2.47528
O	−0.52374	+2.00218	+5.18450

17

* E = +8.580 kcal/mol ; (49) 174_293_070_061_270

H	+0.00000	+0.00000	+0.00000
O	+0.00000	+0.00000	+0.96408
C	+1.28957	+0.00000	+1.37549
C	+1.37567	+0.13633	+2.88635
N	+0.88238	+1.46489	+3.21666
H	−0.11787	+1.52495	+3.06310
O	+2.20505	−0.08803	+0.61680
C	+0.61806	−1.00827	+3.58868
H	+2.43825	+0.07847	+3.13634
H	+1.06914	+1.68869	+4.18569
C	+1.20907	−2.36022	+3.22972
H	+0.72074	−0.87785	+4.66809
H	−0.44295	−0.94866	+3.32149
N	+0.65700	−2.95075	+2.13937
H	+1.06206	−3.80387	+1.79355
H	−0.08861	−2.51476	+1.62724
O	+2.13114	−2.84225	+3.84663

17

* E = +8.832 kcal/mol ; (50) 354_320_187_302_317

H	+0.00000	+0.00000	+0.00000
O	+0.00000	+0.00000	+0.96582
C	+1.24917	+0.00000	+1.43615
C	+2.36109	−0.13774	+0.39146
N	+1.97594	−1.09912	−0.64552
H	+2.76106	−1.26764	−1.26540
O	+1.48564	+0.10365	+2.60067
C	+2.69243	+1.22725	−0.23248
H	+3.23907	−0.45518	+0.96664
H	+1.72572	−1.99334	−0.23803
C	+1.56820	+1.98915	−0.93143

H +3.07710 +1.89673 +0.53892
H +3.49671 +1.08259 −0.96622
N +0.73976 +1.25675 −1.73215
H +0.08050 +1.76822 −2.29582
H +0.96472 +0.30023 −1.95672
O +1.43848 +3.18011 −0.78460
17
* E = +8.941 kcal/mol ; (51) 178_303_063_256_078
H +0.00000 +0.00000 +0.00000
O +0.00000 +0.00000 +0.96447
C +1.28966 +0.00000 +1.37407
C +1.40532 +0.04355 +2.88411
N +0.69975 +1.21460 +3.37482
H −0.29044 +1.15439 +3.16193
O +2.19920 −0.06412 +0.60395
C +0.96916 −1.32981 +3.49198
H +2.47146 +0.17790 +3.07798
H +0.78864 +1.28574 +4.38188
C −0.41574 −1.26110 +4.11370
H +1.01934 −2.12236 +2.73666
H +1.64885 −1.59411 +4.30291
N −1.45599 −1.30761 +3.24596
H −2.38614 −1.20062 +3.61372
H −1.30893 −1.26469 +2.25271
O −0.56613 −1.14491 +5.31024
17
* E = +9.006 kcal/mol ; (52) 356_180_212_177_157
H +0.00000 +0.00000 +0.00000
O +0.00000 +0.00000 +0.95994
C +1.25391 +0.00000 +1.44851
C +2.36054 −0.10137 +0.40866
N +3.64742 −0.09847 +1.06574
H +4.34659 +0.34700 +0.48772
O +1.44824 +0.08936 +2.62095
C +2.22556 −1.38408 −0.41912
H +2.22986 +0.75143 −0.27959
H +3.58925 +0.35331 +1.96986
C +3.30435 −1.39010 −1.49361
H +2.33182 −2.25191 +0.23987
H +1.25641 −1.46356 −0.92940

N	+3.64298	−2.61070	−1.96772
H	+4.36036	−2.67193	−2.67004
H	+3.30600	−3.45364	−1.54093
O	+3.79177	−0.36274	−1.91330

17

∗ E = +9.020 kcal/mol ; (53) 355_166_277_162_122

H	+0.00000	+0.00000	+0.00000
O	+0.00000	+0.00000	+0.96128
C	+1.25279	+0.00000	+1.44615
C	+2.36231	−0.11051	+0.40151
N	+3.67078	+0.21517	+0.92652
H	+3.82550	+1.21510	+0.92390
O	+1.45789	+0.04766	+2.62054
C	+2.38357	−1.54214	−0.15822
H	+2.12933	+0.55824	−0.43790
H	+3.72010	−0.08368	+1.89646
C	+3.14817	−1.52905	−1.47201
H	+2.82896	−2.21154	+0.58456
H	+1.37314	−1.90391	−0.37810
N	+4.23013	−2.33595	−1.54381
H	+4.80132	−2.28679	−2.37091
H	+4.62003	−2.74665	−0.71550
O	+2.76956	−0.83988	−2.39432

17

∗ E = +9.032 kcal/mol ; (54) 178_280_070_056_166

H	+0.00000	+0.00000	+0.00000
O	+0.00000	+0.00000	+0.96342
C	+1.28544	+0.00000	+1.37210
C	+1.36940	+0.05146	+2.89161
N	+1.16196	+1.44674	+3.26429
H	+0.20044	+1.72290	+3.09951
O	+2.20859	+0.04222	+0.62043
C	+0.38512	−0.91191	+3.55936
H	+2.39769	−0.22658	+3.13695
H	+1.37182	+1.60096	+4.24196
C	+0.57813	−2.34215	+3.09059
H	+0.49459	−0.84951	+4.64826
H	−0.64093	−0.60730	+3.31764
N	−0.04631	−3.29108	+3.83156
H	+0.01313	−4.25024	+3.53575

H −0.61058 −3.05822 +4.62712
O +1.24063 −2.60676 +2.11251
17
∗ E = +9.746 kcal/mol ; (55) 350_148_048_176_168
H +0.00000 +0.00000 +0.00000
O +0.00000 +0.00000 +0.95980
C +1.25569 +0.00000 +1.43940
C +2.36207 −0.23171 +0.41900
N +3.53331 +0.49359 +0.84290
H +3.72257 +0.32513 +1.82401
O +1.46581 +0.09890 +2.60882
C +2.54040 −1.75782 +0.31470
H +2.05831 +0.14367 −0.56740
H +4.33350 +0.25346 +0.27260
C +3.56894 −2.10946 −0.74856
H +2.85212 −2.15188 +1.28978
H +1.59460 −2.25274 +0.05445
N +3.97712 −3.40021 −0.74894
H +4.64470 −3.69679 −1.44008
H +3.64194 −4.06338 −0.07494
O +3.98107 −1.29211 −1.54314
17
∗ E = +9.897 kcal/mol ; (56) 007_063_197_175_173
H +0.00000 +0.00000 +0.00000
O +0.00000 +0.00000 +0.96041
C +1.25780 +0.00000 +1.44633
C +2.34606 +0.16724 +0.39334
N +2.20562 +1.47095 −0.24915
H +2.73480 +1.47780 −1.11322
O +1.45615 −0.12441 +2.61412
C +3.71337 +0.06094 +1.04329
H +2.23904 −0.66790 −0.32251
H +1.24648 +1.72525 −0.44462
C +4.80672 +0.11114 −0.00754
H +3.83635 +0.88059 +1.75864
H +3.79755 −0.87454 +1.60751
N +6.06791 +0.17217 +0.47730
H +6.83574 +0.20805 −0.17066
H +6.25322 +0.22319 +1.46194
O +4.56762 +0.08272 −1.19874

17
∗ E = +10.277 kcal/mol ; (57) 001_167_302_062_043

H	+0.00000	+0.00000	+0.00000
O	+0.00000	+0.00000	+0.96638
C	+1.24067	+0.00000	+1.46578
C	+2.38614	+0.03572	+0.43822
N	+3.66861	+0.32886	+1.03680
H	+3.63429	+1.22252	+1.51402
O	+1.43858	−0.02387	+2.64235
C	+2.49272	−1.30843	−0.30255
H	+2.15610	+0.83436	−0.28151
H	+3.86067	−0.35640	+1.76145
C	+1.28848	−1.71414	−1.12894
H	+3.36516	−1.24552	−0.96333
H	+2.67878	−2.11636	+0.41019
N	+0.72137	−0.69491	−1.87949
H	+0.03978	−1.00466	−2.55726
H	+1.31682	+0.05924	−2.18513
O	+0.81887	−2.82032	−1.11246

17
∗ E = +10.311 kcal/mol ; (58) 358_160_308_287_023

H	+0.00000	+0.00000	+0.00000
O	+0.00000	+0.00000	+0.96078
C	+1.24628	+0.00000	+1.45066
C	+2.37277	−0.05799	+0.40968
N	+3.65354	+0.39593	+0.88923
H	+3.58672	+1.31035	+1.31788
O	+1.43408	+0.02672	+2.63258
C	+2.48592	−1.46866	−0.19043
H	+2.06513	+0.60665	−0.41179
H	+4.03377	−0.23735	+1.58264
C	+3.08239	−2.58180	+0.67243
H	+1.50453	−1.83021	−0.52158
H	+3.12102	−1.42008	−1.07717
N	+3.04046	−2.43790	+2.01965
H	+3.37662	−3.21256	+2.56748
H	+2.55456	−1.68869	+2.48954
O	+3.56144	−3.55451	+0.13594

17
∗ E = +10.363 kcal/mol ; (59) 350_175_329_300_091

H	+0.00000	+0.00000	+0.00000
O	+0.00000	+0.00000	+0.96084
C	+1.26611	+0.00000	+1.44434
C	+2.35645	−0.23569	+0.39315
N	+3.69183	−0.11020	+0.91359
H	+3.71972	+0.56480	+1.66830
O	+1.46876	+0.15220	+2.60611
C	+2.16183	−1.62720	−0.24628
H	+2.19362	+0.51578	−0.39766
H	+4.01750	−0.99714	+1.28561
C	+2.26451	−2.70929	+0.81794
H	+1.20561	−1.68974	−0.77583
H	+2.96919	−1.78537	−0.96369
N	+1.09670	−3.05862	+1.40989
H	+1.14052	−3.65849	+2.21712
H	+0.24766	−2.54591	+1.24457
O	+3.33287	−3.18947	+1.12892

17

∗ E = +10.509 kcal/mol ; (60) 002_194_214_083_245

H	+0.00000	+0.00000	+0.00000
O	+0.00000	+0.00000	+0.96287
C	+1.22278	+0.00000	+1.50907
C	+2.41763	+0.04671	+0.55311
N	+3.60870	−0.28658	+1.30358
H	+4.41578	+0.21864	+0.96582
O	+1.35122	−0.01410	+2.69587
C	+2.26131	−0.91793	−0.64387
H	+2.43211	+1.06963	+0.13911
H	+3.46792	−0.07758	+2.28529
C	+1.42272	−0.30706	−1.75071
H	+3.26122	−1.07518	−1.05525
H	+1.88258	−1.88770	−0.30486
N	+0.22446	−0.92095	−1.99754
H	−0.31018	−0.59118	−2.78615
H	+0.06121	−1.86221	−1.68327
O	+1.75175	+0.71103	−2.31093

17

∗ E = +11.003 kcal/mol ; (61) 353_149_039_272_061

H	+0.00000	+0.00000	+0.00000
O	+0.00000	+0.00000	+0.96020

C	+1.24461	+0.00000	+1.44784
C	+2.36293	−0.17531	+0.42902
N	+3.53454	+0.53025	+0.89461
H	+3.63196	+0.41428	+1.89782
O	+1.43438	+0.06351	+2.62831
C	+2.56252	−1.69514	+0.16049
H	+2.02799	+0.27646	−0.51590
H	+4.37619	+0.18564	+0.44852
C	+3.53326	−2.36693	+1.12699
H	+1.60073	−2.22355	+0.17385
H	+3.00182	−1.82840	−0.82979
N	+3.19723	−2.34683	+2.43733
H	+3.85739	−2.74044	+3.08692
H	+2.46059	−1.75955	+2.79522
O	+4.54171	−2.89786	+0.71467

17

* E = +11.262 kcal/mol ; (62) 009_071_186_074_258

H	+0.00000	+0.00000	+0.00000
O	+0.00000	+0.00000	+0.96040
C	+1.25181	+0.00000	+1.44892
C	+2.34996	+0.20912	+0.40590
N	+2.26974	+1.59934	−0.03720
H	+2.91379	+1.76726	−0.80007
O	+1.44458	−0.13332	+2.61874
C	+3.71947	−0.05309	+1.02176
H	+2.18875	−0.52472	−0.40518
H	+1.34296	+1.86399	−0.34747
C	+3.94165	−1.54545	+1.21922
H	+4.48751	+0.29010	+0.32370
H	+3.80340	+0.51348	+1.95221
N	+3.78808	−1.99569	+2.48553
H	+3.82119	−2.99028	+2.63595
H	+3.31103	−1.41921	+3.15937
O	+4.23233	−2.26084	+0.28356

17

* E = +12.831 kcal/mol ; (63) 356_192_198_290_065

H	+0.00000	+0.00000	+0.00000
O	+0.00000	+0.00000	+0.96031
C	+1.25693	+0.00000	+1.45366
C	+2.37562	−0.10452	+0.42120

N	+3.60253	−0.37537	+1.12486
H	+4.42319	−0.17202	+0.57288
O	+1.44158	+0.09353	+2.62568
C	+2.14225	−1.19516	−0.64059
H	+2.35656	+0.87124	−0.10343
H	+3.63833	+0.10919	+2.01207
C	+2.25029	−2.60834	−0.07688
H	+1.18367	−1.07188	−1.16161
H	+2.92300	−1.11402	−1.39982
N	+1.30699	−2.95200	+0.83491
H	+1.39001	−3.84892	+1.28213
H	+0.57978	−2.33043	+1.14100
O	+3.11555	−3.36717	−0.44404

17

∗ E = +13.889 kcal/mol ; (64) 011_064_188_303_325

H	+0.00000	+0.00000	+0.00000
O	+0.00000	+0.00000	+0.95975
C	+1.25599	+0.00000	+1.45924
C	+2.35375	+0.25432	+0.42522
N	+2.17719	+1.60396	−0.12436
H	+2.80109	+1.75587	−0.90770
O	+1.45004	−0.18280	+2.61620
C	+3.73837	+0.07203	+1.03794
H	+2.23410	−0.52574	−0.34866
H	+1.23195	+1.78482	−0.43875
C	+4.11457	+0.90058	+2.26757
H	+3.86783	−0.96909	+1.34016
H	+4.49217	+0.27624	+0.26707
N	+3.61588	+2.15467	+2.33109
H	+3.81452	+2.69504	+3.15520
H	+2.95740	+2.48222	+1.64270
O	+4.87875	+0.44541	+3.08821

10 Gln

10.1 Reference Z-matrix for Gln

Bond distances are given in Å; bond and dihedral angles, in degrees.

h						
o	1	oh2				
c	2	co3	1	coh3		
c	3	cc4	2	cco4	1	dih4
n	4	nc5	3	ncc5	2	dih5
h	5	hn6	4	hnc6	3	dih6
o	3	oc7	2	oco7	4	dih7
c	4	cc8	3	ccc8	5	dih8
h	4	hc9	3	hcc9	5	dih9
h	5	hn10	4	hnc10	6	dih10
c	8	cc11	4	ccc11	3	dih11
h	8	hc12	4	hcc12	11	dih12
h	8	hc13	4	hcc13	11	dih13
c	11	cc14	8	ccc14	4	dih14
h	11	hc15	8	hcc15	14	dih15
h	11	hc16	8	hcc16	14	dih16
n	14	nc17	11	ncc17	8	dih17
o	14	oc18	11	occ18	17	dih18
h	17	hn19	14	hnc19	11	dih19
h	17	hn20	14	hnc20	19	dih20

oh2	0.96839417
co3	1.36095498
coh3	111.85327003
cc4	1.5104121
cco4	110.78297339
dih4	180.62977284
nc5	1.46942746
ncc5	106.82438452
dih5	47.80441336
hn6	1.00244189
hnc6	113.58792693
dih6	185.18239499
oc7	1.19981567
oco7	122.0300199

dih7	181.64548422
cc8	1.52739663
ccc8	109.44209597
dih8	120.41153772
hc9	1.09195449
hcc9	106.30675066
dih9	238.83608518
hn10	1.00295882
hnc10	112.58147334
dih10	128.63658777
cc11	1.53195891
ccc11	112.32367294
dih11	177.1485837
hc12	1.08080975
hcc12	108.11309578
dih12	121.16318748
hc13	1.07938181
hcc13	109.86747905
dih13	238.19950131
cc14	1.52134064
ccc14	111.2324845
dih14	189.91072897
hc15	1.08823407
hcc15	110.57169621
dih15	119.29255116
hc16	1.08400437
hcc16	110.85947818
dih16	238.86499936
nc17	1.35747743
ncc17	114.26304057
dih17	191.20266811
oc18	1.21581111
occ18	123.00078321
dih18	181.0578807
hn19	0.99746147
hnc19	118.65168138
dih19	181.31369152
hn20	0.99455926
hnc20	122.69828739
dih20	180.57270523

10.2 Reference energy and target torsions for Gln

- Reference M08-HX/MG3S energy: -531.7888477 hartree
- Target torsions:
  - torsion1: dih4
  - torsion2: dih5
  - torsion3: dih6
  - torsion4: dih11
  - torsion5: dih14
  - torsion6: dih17

10.3 Properties of Gln conformers

Table S10: Relative energy (kcal mol^{-1}), rotational constants (B1 to B3, GHz) and dipolar moment (Debye) for the conformers of Gln located at the M08-HX/MG3S level.

Conformer	Rel. Energy	B1	B2	B3	Dip. Mom.
1	0.000	0.750	0.903	2.198	3.411
2	0.327	0.933	0.991	2.070	2.239
3	0.612	0.672	0.823	2.418	7.721
4	0.701	1.060	1.221	1.766	4.743
5	0.814	0.752	0.877	2.293	6.838
6	1.570	0.659	0.733	2.520	3.127
7	1.655	0.668	0.745	2.501	5.324
8	1.733	1.021	1.207	1.804	2.169
9	1.912	0.579	0.650	3.194	7.718
10	2.009	0.675	0.783	2.272	2.960
11	2.065	1.016	1.174	1.759	4.173
12	2.226	0.637	0.687	2.971	3.854
13	2.325	0.899	0.958	2.182	3.589
14	2.363	0.664	0.805	2.375	4.843
15	2.546	0.744	0.776	2.468	2.457
16	2.565	0.751	0.873	2.317	8.490
17	2.618	0.763	0.794	2.433	2.288

18	2.652	0.624	0.719	2.645	5.412
19	2.661	0.981	1.155	1.776	1.918
20	2.662	0.747	0.885	2.177	5.142
21	2.712	0.625	0.701	2.711	2.717
22	2.841	0.646	0.714	2.682	4.937
23	2.995	0.894	1.039	2.038	4.763
24	3.031	0.755	0.893	2.175	3.591
25	3.062	0.639	0.717	2.802	6.371
26	3.088	0.665	0.795	2.343	4.535
27	3.236	0.657	0.723	2.511	5.398
28	3.246	0.883	1.035	2.112	1.862
29	3.261	0.665	0.726	2.549	5.361
30	3.291	0.570	0.640	2.937	4.778
31	3.315	0.611	0.740	2.612	5.119
32	3.362	0.634	0.686	2.975	3.231
33	3.418	0.976	1.154	1.841	5.090
34	3.456	0.976	1.141	1.813	3.047
35	3.577	0.571	0.677	2.424	1.872
36	3.579	0.885	0.923	2.132	4.515
37	3.612	0.763	0.829	2.704	8.048
38	3.677	0.681	0.749	2.531	4.438
39	3.685	0.647	0.716	2.645	2.504
40	3.798	0.641	0.712	2.277	2.719
41	3.819	0.742	0.900	2.180	3.658
42	3.885	0.522	0.578	2.866	3.468
43	3.904	0.831	0.991	2.148	4.873
44	3.919	0.864	0.888	2.282	7.130
45	3.949	0.636	0.674	2.341	1.988
46	4.005	0.737	0.879	2.140	4.316
47	4.007	0.594	0.683	2.478	2.138
48	4.030	0.642	0.658	2.678	3.705
49	4.053	0.682	0.776	2.199	2.599
50	4.056	0.980	1.119	1.802	6.224
51	4.164	0.775	0.927	2.113	4.159
52	4.200	0.647	0.789	2.466	5.837
53	4.208	0.530	0.634	2.760	1.978
54	4.225	0.643	0.786	2.454	5.503

55	4.280	0.633	0.739	2.509	5.701
56	4.295	0.650	0.688	2.208	2.422
57	4.295	0.651	0.685	2.208	2.475
58	4.366	0.744	0.892	2.135	2.849
59	4.398	0.883	1.029	2.129	2.548
60	4.404	0.653	0.766	2.379	5.177
61	4.535	0.639	0.764	2.427	4.203
62	4.564	1.136	1.280	1.632	2.438
63	4.580	0.619	0.735	2.592	5.338
64	4.605	0.648	0.716	2.820	3.296
65	4.617	0.948	0.965	2.033	2.728
66	4.658	0.736	0.814	2.023	3.184
67	4.671	1.029	1.216	1.791	3.511
68	4.671	0.936	1.086	1.891	1.721
69	4.751	0.995	1.108	1.848	2.919
70	4.753	0.648	0.778	2.408	5.778
71	4.796	0.626	0.682	2.689	4.707
72	4.797	0.626	0.710	2.629	3.745
73	4.804	0.896	0.959	2.254	7.566
74	4.809	1.079	1.208	1.644	4.918
75	4.812	0.587	0.657	2.646	3.057
76	4.852	0.886	1.103	1.947	2.406
77	4.914	0.828	0.985	2.174	5.304
78	4.927	0.639	0.695	2.263	3.401
79	5.128	0.527	0.562	2.973	6.583
80	5.136	0.583	0.638	2.914	5.221
81	5.215	0.679	0.750	2.479	5.616
82	5.219	0.656	0.748	2.571	6.282
83	5.233	0.782	0.820	2.384	4.462
84	5.306	0.673	0.723	2.538	4.867
85	5.352	0.539	0.565	2.852	3.337
86	5.358	0.897	1.067	1.926	6.394
87	5.376	0.791	0.842	2.291	4.629
88	5.444	0.622	0.694	2.850	1.635
89	5.447	0.853	0.996	2.088	2.499
90	5.580	0.500	0.576	3.010	1.565
91	5.591	0.580	0.683	2.300	4.957

92	5.594	0.734	0.812	2.005	2.881
93	5.621	0.592	0.677	2.304	2.642
94	5.640	0.681	0.737	2.523	4.505
95	5.646	0.667	0.800	2.298	3.993
96	5.670	0.552	0.644	2.606	4.194
97	5.689	0.918	1.093	1.846	4.925
98	5.691	0.665	0.805	2.237	4.244
99	5.726	0.712	0.852	2.255	6.732
100	5.742	0.657	0.796	2.306	4.650
101	5.754	0.925	1.087	1.930	2.544
102	5.785	0.537	0.637	2.739	3.379
103	5.901	0.624	0.699	2.696	4.548
104	5.902	0.569	0.677	2.358	4.206
105	5.921	0.689	0.770	2.389	2.776
106	5.986	0.865	1.073	1.987	1.887
107	6.006	0.826	1.000	2.046	5.216
108	6.061	0.639	0.783	2.466	3.945
109	6.063	0.873	1.030	2.117	3.294
110	6.073	0.592	0.647	2.635	4.495
111	6.178	0.579	0.676	2.336	2.062
112	6.202	0.909	1.105	1.909	5.121
113	6.220	0.653	0.686	2.956	4.238
114	6.350	0.651	0.682	2.770	4.806
115	6.414	0.906	0.967	2.168	2.706
116	6.417	0.856	0.905	2.181	4.206
117	6.420	0.847	0.988	2.112	2.830
118	6.421	0.526	0.577	2.826	3.987
119	6.475	0.654	0.670	2.227	5.218
120	6.566	0.530	0.638	2.714	4.558
121	6.571	0.642	0.763	2.437	3.360
122	6.592	0.648	0.776	2.440	4.565
123	6.678	0.937	1.052	1.868	3.768
124	6.739	0.758	0.896	2.180	4.511
125	6.764	0.917	1.085	1.866	5.959
126	6.800	0.770	0.813	2.457	5.581
127	6.837	0.632	0.696	2.278	3.946
128	6.924	0.957	1.101	1.800	7.359

129	6.938	0.499	0.570	3.148	3.585
130	6.944	0.894	1.109	1.926	3.684
131	7.074	0.781	0.967	2.029	3.308
132	7.081	0.668	0.683	2.788	5.111
133	7.116	0.920	1.078	1.947	4.416
134	7.131	0.663	0.742	2.327	4.502
135	7.218	0.596	0.719	2.587	2.979
136	7.233	0.499	0.572	3.146	5.079
137	7.251	0.625	0.679	2.795	6.167
138	7.304	1.072	1.207	1.656	5.376
139	7.310	0.889	0.963	1.958	4.459
140	7.422	0.636	0.673	2.911	3.282
141	7.469	0.733	0.791	2.640	4.018
142	7.483	0.807	0.857	2.307	3.984
143	7.505	0.761	0.798	2.461	1.272
144	7.542	0.544	0.563	2.799	3.876
145	7.552	0.783	0.962	2.048	3.834
146	7.700	0.760	0.786	2.396	4.611
147	7.703	0.638	0.687	2.958	6.274
148	7.861	0.834	1.033	2.045	4.647
149	7.929	0.615	0.674	2.887	5.372
150	8.030	0.893	0.932	2.111	3.463
151	8.049	0.647	0.679	2.311	2.094
152	8.246	0.834	0.971	2.043	5.173
153	8.333	0.729	0.881	2.108	3.925
154	8.519	0.663	0.725	2.544	5.897
155	8.572	0.569	0.639	2.926	3.878
156	8.592	0.709	0.760	2.776	3.725
157	8.626	0.513	0.591	2.842	5.175
158	8.818	0.624	0.758	2.357	2.670
159	9.117	0.648	0.697	2.245	2.060
160	9.319	0.827	0.961	2.069	5.416
161	9.798	0.836	1.026	1.960	5.949
162	9.822	0.662	0.723	2.788	4.366
163	10.217	0.794	0.907	2.156	5.210
164	10.399	0.588	0.696	2.251	3.604
165	10.437	1.082	1.210	1.659	5.927

166	10.514	0.582	0.686	2.300	3.703
167	10.682	0.630	0.733	2.578	7.581
168	10.686	0.710	0.854	2.162	4.805
169	10.790	0.723	0.871	2.148	5.283
170	10.797	0.706	0.864	2.129	2.727
171	11.132	0.633	0.738	2.525	5.988
172	11.220	0.746	0.797	2.466	6.857
173	11.514	0.655	0.679	2.762	4.999
174	11.518	0.877	1.061	1.858	5.362
175	11.672	0.642	0.677	2.883	7.669
176	11.934	0.791	0.899	2.184	6.036
177	12.139	0.839	0.991	2.108	7.070
178	12.199	0.763	0.808	2.500	7.703
179	12.413	0.549	0.644	2.629	3.274
180	12.752	1.067	1.202	1.677	6.745
181	13.296	0.764	0.897	2.161	5.622

10.4 Vibrational frequencies of Gln conformers

Harmonic vibrational zero point energy (ZPE) and frequencies for each conformer in cm^{-1}.

- Conformer (1); ZPE = 36642.567 cm^{-1}:

45.12	69.69	100.34	139.69	189.51	254.72
268.34	324.12	352.29	411.50	446.41	494.46
543.46	554.68	641.60	650.21	743.68	767.75
798.42	821.72	843.12	877.66	927.46	996.65
1028.55	1067.13	1122.08	1145.76	1173.89	1198.99
1262.91	1285.37	1321.14	1337.02	1365.40	1385.18
1420.70	1435.06	1478.20	1483.92	1634.22	1658.66
1803.19	1867.14	3028.29	3037.91	3076.00	3086.68
3134.17	3558.39	3588.25	3642.27	3668.20	3727.84

- Conformer (2); ZPE = 36610.593 cm^{-1}:

59.18	76.11	96.86	168.24	173.65	261.83
309.56	330.00	362.49	385.71	424.29	482.06
532.74	546.49	613.54	657.57	706.40	771.27
797.89	840.54	862.67	891.01	918.30	958.32
1025.86	1045.99	1104.81	1149.65	1168.07	1204.71
1240.63	1284.70	1310.10	1353.42	1371.23	1374.54
1425.19	1441.00	1461.72	1477.31	1626.82	1658.89
1792.97	1890.66	3051.45	3053.36	3064.89	3099.54
3123.12	3544.80	3608.05	3631.88	3643.59	3765.52

- Conformer (3); ZPE = 36527.691 cm^{-1}:

52.20	90.84	110.61	144.43	200.75	230.72
254.62	303.90	317.65	336.15	403.51	485.36
514.78	525.95	617.83	642.28	669.34	752.25
799.00	805.80	863.94	901.59	940.22	1008.58
1017.10	1095.27	1109.06	1174.30	1181.23	1223.78
1232.53	1300.13	1322.58	1329.18	1392.97	1408.84
1436.20	1439.37	1457.38	1474.39	1629.19	1672.47
1771.06	1883.62	3035.51	3037.16	3060.24	3085.51
3110.86	3532.84	3610.08	3637.12	3651.54	3771.60

- Conformer (4); ZPE = 36643.789 cm^{-1}:

71.40	98.22	106.09	119.23	191.54	231.12
320.26	364.77	397.35	421.29	440.54	511.49
546.51	555.91	562.15	598.12	741.79	756.16
765.10	792.63	851.59	873.79	933.77	972.74
996.82	1083.27	1128.23	1136.00	1182.05	1217.46
1246.96	1251.30	1325.31	1342.72	1368.39	1401.92
1433.47	1439.09	1470.41	1489.87	1634.14	1637.89
1788.78	1876.38	3025.80	3054.98	3061.45	3101.19
3115.89	3522.66	3624.39	3644.98	3709.95	3752.28

- Conformer (5); ZPE = 36589.931 cm^{-1}:

45.65	87.21	135.80	169.92	184.37	203.74
241.96	302.96	321.08	374.81	381.72	459.52
531.90	560.96	624.62	662.59	745.30	766.47
802.45	843.61	877.74	888.33	940.58	983.54
1055.27	1069.83	1119.45	1157.32	1184.30	1198.73
1230.65	1293.64	1336.08	1348.12	1360.24	1417.60
1436.24	1444.81	1476.13	1495.38	1631.59	1670.46
1763.10	1871.95	3042.47	3057.45	3068.08	3095.52
3110.57	3528.89	3557.47	3613.19	3635.73	3772.83

- Conformer (6); ZPE = 36495.084 cm^{-1}:

42.64	47.75	87.27	143.23	178.21	201.73
265.20	305.94	344.56	405.24	444.19	467.05
519.32	546.72	589.63	650.21	660.03	738.96
764.28	783.86	857.16	919.31	973.33	995.52
1022.18	1054.96	1118.46	1161.91	1172.16	1198.77
1235.67	1290.48	1312.19	1340.42	1358.77	1392.08
1410.88	1418.34	1474.20	1483.18	1627.98	1667.22
1810.90	1862.21	3020.32	3035.42	3069.13	3110.46
3131.29	3528.18	3577.25	3602.72	3727.96	3843.14

- Conformer (7); ZPE = 36552.343 cm^{-1}:

31.42	63.73	89.60	134.51	176.55	210.65
329.00	342.22	363.93	398.67	415.75	437.58
531.05	549.03	593.73	624.33	743.05	752.96
799.27	812.56	849.56	897.33	954.94	978.77
1008.88	1076.97	1111.83	1154.94	1165.07	1218.16
1235.50	1264.87	1314.55	1342.49	1368.14	1398.13
1421.56	1437.36	1475.36	1488.77	1627.73	1657.47
1791.48	1886.77	3032.27	3045.56	3062.25	3098.78
3119.49	3522.99	3607.08	3635.89	3682.60	3771.49

- Conformer (8); ZPE = 36529.998 cm^{-1}:

70.04	87.02	113.56	126.00	181.80	229.07
290.82	350.03	367.84	383.11	429.80	457.52
532.79	548.37	554.17	585.60	624.45	749.60
756.36	786.96	854.41	898.19	947.41	970.07
1013.95	1071.05	1111.87	1155.36	1179.86	1199.68
1235.84	1259.81	1320.92	1338.73	1370.27	1393.13
1408.31	1428.46	1465.70	1488.99	1629.95	1672.65
1784.19	1845.52	3025.02	3061.41	3065.91	3111.54
3122.37	3523.09	3623.12	3634.47	3767.64	3856.21

- Conformer (9); ZPE = 36447.375 cm^{-1}:

21.37	33.98	71.84	153.46	178.41	254.49
273.67	310.00	315.00	368.13	407.04	481.46
509.51	549.99	612.03	624.58	653.11	750.32
802.65	850.61	856.38	880.60	946.22	975.96
1018.93	1080.70	1103.43	1150.66	1175.36	1241.38
1254.18	1290.93	1294.56	1337.25	1377.14	1396.51
1432.55	1444.75	1454.28	1469.41	1627.50	1667.26
1795.86	1883.19	3023.90	3031.78	3051.96	3076.91
3117.55	3533.11	3618.69	3640.71	3648.45	3775.03

- Conformer (10); ZPE = 36451.435 cm^{-1}:

33.82	46.37	63.70	110.97	208.33	285.52
288.39	296.67	327.48	359.36	380.93	495.09
501.76	557.54	569.24	634.28	644.79	741.62
791.13	807.41	836.29	883.40	946.58	972.04
1067.08	1092.72	1100.26	1159.24	1181.37	1206.77
1240.24	1264.80	1303.13	1344.22	1376.04	1411.14
1424.23	1439.60	1459.51	1462.61	1623.79	1661.36
1795.76	1878.31	3043.68	3050.34	3055.43	3097.35
3108.84	3540.90	3631.89	3640.35	3684.40	3774.77

- Conformer (11); ZPE = 36522.697 cm^{-1}:

65.71	83.32	110.39	133.50	179.84	229.57
279.95	309.95	368.73	438.22	472.06	497.35
504.59	543.52	577.36	623.96	675.34	743.89
767.95	770.88	847.18	897.66	924.31	982.68
1002.15	1068.65	1113.50	1148.82	1189.08	1204.88
1235.04	1249.55	1320.71	1346.66	1364.63	1383.19
1409.35	1448.65	1473.96	1484.83	1622.83	1628.34
1810.72	1855.73	2949.10	3049.56	3070.86	3109.24
3135.27	3545.49	3579.59	3636.23	3728.62	3852.25

- Conformer (12); ZPE = 36372.196 cm^{-1}:

49.77	52.20	61.30	129.34	178.77	252.20
294.88	322.00	332.32	346.67	387.59	462.82
511.73	528.96	577.17	595.57	648.36	673.53
745.76	810.45	848.98	903.72	948.29	990.62
1013.33	1071.81	1099.92	1162.35	1164.49	1205.99
1236.38	1295.66	1301.95	1335.53	1359.30	1397.11
1408.79	1432.33	1450.38	1474.44	1627.52	1666.08
1803.97	1866.96	3003.95	3041.82	3053.48	3090.58
3120.23	3530.88	3618.66	3638.82	3771.88	3846.79

- Conformer (13); ZPE = 36446.750 cm^{-1}:

53.02	85.03	90.66	146.92	166.19	207.55
259.25	284.91	348.73	421.73	432.67	447.04
527.45	539.97	599.54	615.68	676.51	721.52
767.34	803.19	837.01	887.35	916.92	955.66
1019.31	1063.04	1113.46	1162.71	1178.30	1190.14
1237.18	1267.24	1304.27	1331.00	1343.41	1379.62
1423.03	1447.88	1476.12	1484.09	1632.32	1655.98
1801.62	1846.32	3033.25	3050.85	3053.56	3100.24
3107.46	3555.02	3616.56	3637.75	3745.15	3844.74

- Conformer (14); ZPE = 36630.875 cm^{-1}:

54.79	74.37	101.76	117.63	187.88	232.68
307.54	327.23	378.48	426.98	444.85	483.96
544.10	563.89	588.18	640.18	727.78	771.43
790.94	814.54	864.51	880.89	934.57	958.97
1011.39	1090.82	1119.51	1143.18	1182.95	1218.79
1249.76	1288.79	1298.34	1324.31	1382.00	1396.73
1397.86	1439.16	1475.14	1484.65	1635.91	1661.07
1812.89	1870.03	3019.08	3039.91	3066.09	3108.97
3127.74	3543.00	3586.09	3638.16	3694.47	3736.87

- Conformer (15); ZPE = 36381.040 cm^{-1}:

45.86	61.90	83.40	125.17	188.04	261.50
292.27	319.01	350.74	381.13	400.50	462.00
516.51	554.01	570.60	596.81	650.49	668.25
755.65	800.51	827.48	906.58	921.77	957.23
1011.60	1067.49	1104.13	1157.42	1178.77	1192.50
1220.61	1269.09	1305.70	1336.21	1363.09	1389.39
1430.17	1453.10	1455.35	1461.84	1624.09	1641.62
1796.56	1866.90	2948.19	3049.01	3069.39	3096.26
3123.73	3546.79	3639.26	3643.90	3773.24	3849.28

- Conformer (16); ZPE = 36577.543 cm^{-1}:

44.10	88.87	137.28	170.50	187.59	241.81
260.00	318.15	325.32	370.97	376.85	460.48
532.93	559.34	621.04	659.68	743.50	767.43
799.84	833.00	860.08	875.50	902.71	1007.26
1029.61	1092.03	1109.90	1162.12	1185.05	1204.80
1235.42	1281.76	1332.35	1343.29	1366.86	1411.90
1433.69	1460.47	1474.48	1494.55	1630.80	1633.95
1765.97	1877.45	3028.58	3050.78	3057.10	3085.61
3108.59	3539.90	3569.80	3635.01	3636.48	3772.55

- Conformer (17); ZPE = 36351.935 cm^{-1}:

40.09	46.65	83.63	110.96	175.46	257.00
278.84	342.81	383.56	393.26	423.16	496.54
504.25	531.41	569.30	590.89	641.97	680.00
743.97	792.58	819.41	877.24	921.28	966.42
1004.39	1066.78	1108.71	1157.47	1183.65	1190.96
1220.64	1257.38	1305.98	1340.19	1353.63	1383.95
1428.19	1444.01	1451.09	1480.76	1627.30	1629.30
1802.32	1864.82	2947.16	3036.19	3067.52	3088.13
3126.99	3559.64	3634.30	3659.42	3763.78	3848.51

- Conformer (18); ZPE = 36502.305 cm^{-1}:

20.55	48.34	82.98	111.75	184.70	277.27
289.26	304.28	346.50	389.87	410.21	494.11
505.73	545.92	579.79	619.09	656.01	743.13
797.58	812.11	847.30	913.45	953.87	979.40
1062.71	1098.22	1107.08	1158.29	1174.42	1234.66
1244.53	1254.69	1307.96	1338.51	1388.01	1402.66
1428.65	1441.49	1458.00	1461.92	1625.30	1665.21
1791.60	1883.72	3029.82	3036.11	3058.75	3082.58
3117.34	3531.27	3612.06	3639.89	3681.63	3774.29

- Conformer (19); ZPE = 36330.296 cm^{-1}:

49.72	66.57	115.43	126.21	169.62	183.98
252.14	288.76	343.77	351.63	402.63	461.09
505.98	522.54	571.10	609.12	659.80	664.95
769.72	784.93	845.37	890.45	930.74	969.30
1002.87	1079.27	1116.40	1149.51	1168.16	1190.69
1227.90	1260.91	1303.55	1317.70	1361.95	1385.32
1428.16	1448.63	1456.85	1464.98	1626.01	1668.78
1803.25	1847.37	3044.83	3066.00	3081.42	3088.01
3123.91	3536.74	3623.95	3638.89	3770.52	3842.52

- Conformer (20); ZPE = 36487.887 cm^{-1}:

42.82	58.71	103.39	139.27	173.91	239.00
270.84	280.91	314.32	403.79	444.90	469.10
536.56	554.37	615.76	628.87	652.57	753.76
781.85	788.27	832.04	861.66	909.15	985.46
1032.27	1063.47	1123.89	1153.68	1182.27	1184.83
1219.15	1285.47	1320.92	1337.96	1350.91	1393.70
1401.36	1418.19	1477.17	1483.10	1634.65	1663.84
1807.58	1836.42	3034.53	3066.93	3068.92	3105.58
3128.25	3550.31	3594.93	3637.61	3734.80	3841.80

- Conformer (21); ZPE = 36218.580 cm^{-1}:

30.41	39.50	48.72	106.71	185.42	208.94
242.95	260.00	292.63	312.53	385.03	468.84
504.39	517.85	585.63	624.60	635.55	663.13
745.93	792.53	844.31	886.06	955.32	990.08
1003.07	1077.76	1091.82	1160.89	1170.29	1193.09
1239.84	1275.39	1297.59	1332.60	1362.35	1383.47
1425.68	1435.17	1450.91	1462.96	1621.88	1669.92
1806.55	1856.11	3037.17	3049.55	3070.84	3095.87
3116.16	3536.45	3621.09	3641.48	3777.04	3847.11

- Conformer (22); ZPE = 36388.313 cm^{-1}:

29.08	57.77	87.42	131.00	191.69	201.83
296.92	309.22	321.14	349.41	421.78	446.99
513.37	541.85	578.47	614.44	638.14	734.44
758.07	792.18	849.29	898.27	931.84	981.41
1016.23	1077.60	1112.68	1166.29	1182.64	1207.08
1223.73	1255.61	1315.34	1331.34	1360.83	1373.63
1418.89	1439.00	1471.73	1486.15	1623.82	1631.62
1790.13	1864.64	2976.46	3043.65	3061.36	3097.87
3118.99	3548.17	3637.20	3640.48	3778.96	3848.44

- Conformer (23); ZPE = 36511.183 cm^{-1}:

36.86	56.19	77.93	118.83	200.68	251.67
279.68	335.16	351.51	358.39	438.89	491.02
517.77	558.09	598.23	645.59	689.06	742.41
783.96	795.51	838.51	867.80	915.00	1013.58
1021.27	1082.47	1113.31	1131.74	1175.35	1232.45
1256.60	1287.94	1313.72	1339.49	1358.76	1385.74
1432.32	1435.96	1451.61	1480.29	1634.15	1655.29
1811.64	1872.71	3028.32	3039.42	3054.72	3091.59
3105.56	3561.27	3618.79	3647.87	3698.95	3740.76

- Conformer (24); ZPE = 36467.080 cm^{-1}:

46.49	64.14	103.99	135.63	173.17	235.63
261.25	293.32	306.44	402.83	444.00	493.99
535.61	554.77	590.01	614.98	645.22	752.21
784.73	789.80	832.10	855.17	904.63	1002.42
1032.62	1080.14	1117.27	1132.86	1175.93	1184.95
1223.87	1263.75	1319.95	1336.06	1361.59	1381.47
1417.70	1440.51	1476.37	1482.76	1636.58	1651.19
1805.51	1845.79	3033.41	3066.95	3071.50	3092.40
3126.92	3546.73	3594.37	3636.49	3730.83	3845.16

- Conformer (25); ZPE = 36518.636 cm^{-1}:

28.76	42.09	79.93	138.42	177.06	211.59
314.46	333.92	361.79	392.13	426.80	443.75
533.85	547.61	590.75	605.51	733.10	759.85
813.82	829.04	856.38	895.69	955.89	966.57
1030.13	1069.71	1101.34	1148.33	1161.68	1208.04
1245.36	1283.99	1290.43	1339.79	1367.98	1394.79
1414.14	1440.32	1474.90	1485.29	1626.98	1666.40
1797.84	1885.23	3018.67	3037.13	3056.84	3108.50
3119.97	3541.52	3626.21	3632.52	3659.39	3765.09

- Conformer (26); ZPE = 36620.362 cm^{-1}:

49.44	64.39	90.65	127.81	198.45	261.54
293.06	328.88	358.92	434.76	497.87	522.54
542.08	545.95	562.08	602.07	714.20	755.10
799.00	823.27	853.67	858.99	934.79	988.56
1021.49	1099.27	1113.34	1147.05	1170.55	1223.84
1259.85	1288.03	1299.48	1344.73	1386.04	1401.35
1404.41	1441.49	1457.54	1497.01	1654.28	1661.53
1798.85	1867.30	3030.84	3046.59	3055.61	3093.56
3104.38	3563.72	3567.80	3654.39	3661.15	3717.20

- Conformer (27); ZPE = 36472.144 cm^{-1}:

29.08	49.50	94.65	135.55	176.87	206.24
298.09	329.30	352.57	390.88	447.23	455.94
500.05	551.65	585.74	632.89	657.55	753.26
761.43	781.92	850.36	906.04	968.61	990.02
1008.65	1055.32	1109.01	1159.84	1170.90	1181.02
1228.24	1285.78	1311.91	1334.89	1353.94	1396.60
1405.74	1418.50	1472.69	1482.83	1625.32	1655.42
1812.41	1870.98	3019.95	3050.48	3069.85	3108.38
3133.59	3530.23	3586.80	3618.38	3734.23	3846.97

- Conformer (28); ZPE = 36332.290 cm^{-1}:

58.94	68.31	96.52	121.12	175.39	247.47
265.82	286.84	323.26	344.44	375.86	483.81
495.83	552.74	568.44	594.90	622.28	659.21
761.19	789.41	830.07	878.32	917.39	997.87
1029.84	1079.42	1103.20	1151.73	1177.51	1203.35
1245.13	1274.50	1306.18	1338.95	1362.68	1391.60
1409.38	1432.73	1449.14	1469.38	1629.70	1664.91
1805.51	1845.81	3043.16	3047.76	3060.53	3090.25
3112.83	3544.52	3628.79	3635.14	3764.78	3850.74

- Conformer (29); ZPE = 36476.801 cm^{-1}:

41.03	56.28	78.23	116.83	189.37	196.02
239.87	296.45	360.77	425.82	445.41	479.63
516.28	556.54	605.54	630.55	675.27	740.67
765.50	812.20	845.46	906.78	951.22	974.48
1041.78	1058.34	1136.41	1156.93	1175.31	1206.44
1223.23	1269.72	1291.89	1334.98	1355.69	1382.81
1397.62	1444.44	1469.75	1485.40	1636.96	1668.85
1803.81	1859.30	3028.91	3061.23	3072.34	3110.10
3131.61	3523.82	3540.23	3615.14	3723.01	3841.33

- Conformer (30); ZPE = 36450.916 cm^{-1}:

42.66	54.56	58.87	120.30	160.50	219.24
251.94	273.16	310.86	396.79	426.58	474.59
510.84	557.10	583.59	656.12	686.61	750.58
762.17	814.87	865.76	918.03	955.03	998.11
1028.44	1064.93	1141.14	1164.54	1167.27	1197.94
1216.08	1273.79	1318.56	1342.11	1364.23	1378.01
1399.79	1436.28	1466.69	1480.93	1631.22	1665.99
1809.59	1863.17	3034.14	3048.22	3059.94	3107.58
3136.91	3517.18	3557.09	3606.78	3730.83	3843.60

- Conformer (31); ZPE = 36586.928 cm^{-1}:

49.46	72.96	101.14	110.46	189.32	252.69
287.31	306.43	374.48	388.68	424.76	440.40
545.22	559.15	577.57	646.81	730.98	767.07
785.34	806.13	864.63	882.35	933.14	1000.08
1044.70	1091.58	1121.81	1154.60	1181.33	1199.83
1247.27	1270.78	1309.91	1334.94	1375.09	1397.67
1403.26	1437.01	1476.49	1485.40	1626.97	1659.13
1810.59	1870.87	3023.86	3042.24	3066.48	3094.95
3134.42	3543.32	3595.72	3636.08	3698.19	3742.77

- Conformer (32); ZPE = 36361.416 cm^{-1}:

8.08	51.67	62.43	132.37	179.39	258.34
299.87	315.28	320.40	362.96	400.34	476.11
511.85	532.04	583.30	595.69	654.59	673.12
750.22	807.88	840.46	885.67	941.48	972.32
1010.38	1071.17	1097.52	1158.95	1185.99	1203.10
1236.23	1295.51	1306.88	1328.24	1372.68	1391.18
1397.16	1432.41	1448.55	1474.46	1626.56	1662.02
1801.46	1857.68	3007.11	3040.77	3056.75	3089.21
3124.65	3539.74	3635.10	3640.14	3773.82	3841.54

- Conformer (33); ZPE = 36480.798 cm^{-1}:

53.18	92.73	103.69	121.96	178.11	226.51
286.06	291.83	321.18	366.70	425.70	470.37
502.05	544.45	574.77	603.09	642.74	743.57
753.34	785.11	854.90	904.42	955.85	975.33
1020.58	1072.60	1101.76	1159.03	1180.54	1192.08
1237.46	1270.97	1325.41	1339.33	1376.26	1385.86
1404.88	1429.11	1475.80	1489.93	1624.85	1672.65
1789.68	1870.81	3019.91	3060.21	3064.03	3107.55
3118.56	3517.01	3610.12	3643.96	3781.68	3841.32

- Conformer (34); ZPE = 36320.017 cm^{-1}:

62.76	67.29	116.22	120.51	171.15	224.54
276.76	298.80	344.15	356.85	397.04	465.64
499.51	526.84	571.28	603.52	654.15	674.11
766.82	780.56	822.62	868.16	924.22	953.61
996.28	1088.24	1103.71	1150.28	1179.57	1206.27
1225.85	1252.29	1308.04	1320.94	1349.72	1384.85
1430.20	1449.79	1456.87	1483.48	1627.17	1637.55
1805.62	1849.59	3042.53	3049.21	3061.40	3086.09
3104.91	3548.15	3635.58	3644.49	3766.09	3848.17

- Conformer (35); ZPE = 36326.428 cm^{-1}:

25.76	50.17	70.77	103.81	137.91	195.95
273.01	278.97	325.17	340.72	359.49	464.41
509.08	561.51	611.79	646.62	657.59	744.94
794.88	808.36	843.88	885.50	909.74	1002.60
1045.31	1085.32	1105.94	1136.43	1153.41	1227.64
1245.05	1282.27	1311.26	1333.79	1358.52	1379.97
1422.73	1440.67	1460.30	1486.68	1623.82	1656.56
1809.10	1883.89	3029.69	3037.75	3045.94	3083.09
3104.26	3557.49	3641.51	3642.37	3683.15	3776.34

- Conformer (36); ZPE = 36396.263 cm^{-1}:

51.77	60.46	108.05	147.36	159.34	191.17
236.90	263.44	342.84	414.90	424.71	444.05
507.48	546.72	616.13	621.47	673.16	745.26
766.11	792.55	832.49	872.90	907.23	948.61
1036.98	1049.35	1114.20	1161.07	1174.56	1190.97
1209.07	1284.08	1303.03	1331.87	1363.02	1380.28
1416.18	1429.27	1466.38	1480.83	1631.67	1673.88
1813.02	1842.91	3045.28	3056.58	3065.48	3113.11
3116.23	3554.21	3604.54	3634.21	3738.88	3836.28

- Conformer (37); ZPE = 36650.573 cm^{-1}:

45.94	91.73	122.04	162.24	212.32	277.17
296.69	309.67	344.53	359.96	364.95	483.38
504.37	599.66	607.98	661.40	669.30	776.01
798.28	820.36	845.32	886.42	941.60	974.32
1001.41	1078.84	1111.94	1161.38	1192.53	1215.87
1270.40	1294.59	1317.47	1330.10	1387.87	1408.99
1423.05	1437.31	1453.36	1492.27	1630.25	1658.83
1765.74	1878.61	3049.59	3060.64	3093.81	3102.48
3113.40	3549.41	3621.63	3633.88	3638.94	3770.94

- Conformer (38); ZPE = 36516.721 cm^{-1}:

40.31	67.92	71.98	124.83	174.05	191.74
309.92	316.07	326.43	360.49	410.66	433.35
545.56	554.14	597.68	658.28	734.74	753.81
792.34	801.33	856.13	910.42	962.13	978.12
1025.78	1057.13	1098.75	1142.87	1187.25	1198.20
1253.16	1288.78	1315.01	1333.02	1374.69	1388.12
1419.95	1426.12	1471.62	1492.24	1626.01	1682.14
1799.03	1893.31	3020.21	3044.57	3066.01	3095.91
3123.33	3532.77	3598.50	3636.50	3700.29	3769.75

- Conformer (39); ZPE = 36430.597 cm^{-1}:

30.36	45.61	87.10	133.84	182.39	205.30
297.92	309.36	356.52	372.36	413.67	451.17
495.28	548.13	575.08	606.31	630.52	743.88
753.22	793.58	849.30	925.66	941.39	980.27
1020.79	1071.66	1116.00	1166.86	1183.03	1201.19
1221.01	1267.13	1318.73	1330.32	1354.94	1376.31
1417.10	1449.15	1470.35	1490.19	1624.41	1665.70
1791.29	1865.04	2951.89	3055.22	3063.79	3104.04
3121.15	3539.72	3629.18	3639.96	3777.74	3849.10

- Conformer (40); ZPE = 36426.751 cm^{-1}:

31.52	49.83	76.54	93.14	167.93	193.65
282.36	296.68	308.38	361.57	430.67	457.69
532.70	553.70	612.85	650.51	689.19	761.53
801.54	816.58	842.71	898.89	919.60	979.72
1025.17	1058.59	1102.30	1141.36	1168.37	1204.17
1263.97	1277.63	1301.24	1351.49	1359.45	1377.28
1422.60	1435.59	1464.01	1482.02	1625.12	1657.62
1809.22	1891.14	3032.91	3051.42	3061.78	3080.67
3115.20	3552.87	3636.66	3641.58	3674.55	3778.07

- Conformer (41); ZPE = 36459.802 cm^{-1}:

30.24	54.18	95.55	131.68	192.47	226.69
252.45	312.86	323.33	409.62	446.47	482.32
542.17	552.86	586.69	628.96	644.73	744.61
763.00	789.49	818.40	866.27	894.72	1012.05
1018.63	1086.79	1117.82	1160.69	1179.23	1203.47
1225.79	1260.46	1320.48	1336.67	1374.01	1376.83
1416.12	1437.74	1478.38	1484.92	1630.83	1653.36
1802.23	1833.96	2968.98	3047.41	3077.17	3099.98
3132.26	3559.43	3606.51	3648.79	3739.80	3839.04

- Conformer (42); ZPE = 36131.019 cm^{-1}:

17.31	43.59	65.17	97.95	111.36	199.73
225.57	248.21	262.04	299.37	393.23	449.34
511.44	514.19	580.88	618.50	637.40	661.91
763.86	794.26	848.17	886.55	958.28	979.50
1022.89	1085.23	1113.92	1142.00	1162.01	1192.95
1237.05	1276.64	1290.89	1334.00	1352.82	1379.77
1419.91	1427.86	1464.54	1481.05	1621.70	1666.44
1812.33	1864.75	3032.24	3037.31	3053.79	3086.13
3117.83	3536.31	3617.10	3642.86	3777.37	3844.54

- Conformer (43); ZPE = 36253.057 cm^{-1}:

62.36	63.17	78.79	134.30	185.69	222.43
249.47	264.17	287.63	322.11	336.52	464.54
505.95	540.66	564.63	612.40	644.48	652.20
755.07	772.57	834.47	898.41	931.09	1003.61
1035.78	1090.33	1099.88	1150.84	1160.44	1191.79
1244.32	1276.96	1297.83	1315.69	1370.18	1380.27
1424.79	1430.05	1452.54	1472.03	1624.02	1670.89
1811.14	1877.23	3034.37	3039.30	3055.49	3084.56
3113.53	3538.15	3616.18	3641.74	3777.23	3841.85

- Conformer (44); ZPE = 36605.543 cm^{-1}:

32.29	75.43	144.84	173.69	183.91	217.56
289.97	327.71	342.92	384.41	398.44	458.58
542.80	562.29	608.52	676.15	737.21	749.74
764.79	805.92	852.27	870.38	919.95	943.31
1041.42	1051.63	1109.71	1162.15	1192.16	1198.58
1272.32	1305.50	1319.88	1356.20	1366.86	1406.84
1411.50	1437.04	1465.28	1492.55	1632.01	1662.44
1760.09	1869.44	3047.86	3051.48	3080.15	3100.11
3105.97	3562.60	3625.62	3635.89	3651.97	3772.77

- Conformer (45); ZPE = 36200.095 cm^{-1}:

23.09	52.92	59.38	110.27	162.81	207.83
248.29	255.01	290.72	354.30	426.29	488.70
503.20	511.91	581.43	629.93	633.61	650.54
761.20	787.58	832.40	855.91	921.62	966.88
1027.09	1088.67	1108.85	1140.54	1178.56	1213.58
1217.32	1255.80	1309.33	1329.56	1343.78	1372.99
1426.64	1447.53	1461.56	1480.19	1621.48	1634.98
1811.56	1863.63	2965.78	3029.10	3071.00	3074.87
3119.97	3562.51	3643.80	3657.65	3778.74	3847.34

- Conformer (46); ZPE = 36379.456 cm^{-1}:

38.82	52.99	99.27	126.91	172.79	196.14
254.71	285.80	308.84	382.30	408.24	439.78
510.15	551.57	610.74	634.90	650.83	746.74
773.46	788.33	838.06	871.65	919.49	984.71
1038.52	1065.70	1121.27	1150.73	1163.14	1186.00
1191.96	1282.04	1303.41	1326.37	1355.85	1373.53
1411.35	1424.82	1474.79	1484.12	1628.64	1666.08
1813.78	1870.93	3039.78	3062.66	3069.50	3108.30
3127.59	3538.86	3623.87	3625.06	3757.39	3825.64

- Conformer (47); ZPE = 36384.748 cm^{-1}:

27.24	31.25	52.09	111.80	169.20	204.63
261.40	295.65	299.89	372.44	429.66	478.59
537.32	548.00	568.24	620.61	698.57	755.95
786.17	806.03	842.18	878.86	915.68	972.56
1028.45	1081.76	1111.87	1144.43	1178.44	1228.35
1241.22	1262.47	1300.39	1332.29	1377.62	1396.66
1418.58	1435.57	1462.51	1475.44	1624.30	1660.12
1810.00	1883.71	3028.60	3045.75	3065.75	3093.52
3118.41	3549.38	3639.65	3641.22	3690.89	3778.11

- Conformer (48); ZPE = 36222.070 cm^{-1}:

32.48	35.58	50.14	97.66	186.24	243.72
259.47	279.23	294.88	312.65	367.44	479.16
491.67	519.74	574.73	611.96	633.47	657.42
768.55	790.43	835.75	872.98	951.41	990.68
1003.34	1076.78	1091.41	1150.08	1164.44	1180.06
1241.07	1284.41	1299.71	1326.53	1358.70	1406.19
1421.17	1425.82	1451.07	1462.46	1623.50	1656.64
1806.97	1864.59	3044.57	3052.48	3068.78	3090.40
3119.21	3536.86	3629.85	3641.81	3777.29	3850.48

- Conformer (49); ZPE = 36237.615 cm^{-1}:

35.13	50.84	56.31	101.03	191.49	242.46
275.98	290.13	293.58	330.76	367.52	482.96
507.19	521.22	562.00	600.70	635.82	638.78
751.35	782.48	824.56	890.24	917.33	993.89
1046.15	1089.49	1101.33	1152.90	1176.53	1207.80
1218.78	1281.98	1301.51	1329.42	1342.57	1403.12
1422.05	1451.51	1462.36	1464.13	1620.78	1652.47
1801.96	1863.38	2959.93	3048.46	3062.79	3095.50
3117.32	3551.20	3637.12	3642.88	3777.20	3848.90

- Conformer (50); ZPE = 36498.494 cm^{-1}:

54.57	85.68	120.56	126.19	193.78	234.74
292.67	306.36	356.17	435.01	445.67	483.34
527.98	543.72	563.00	627.93	683.25	735.55
760.96	775.43	845.57	894.29	923.82	979.49
1004.70	1067.90	1111.01	1152.75	1172.30	1193.40
1240.04	1251.64	1326.38	1338.41	1361.37	1381.44
1409.19	1439.42	1469.23	1476.72	1619.85	1644.51
1815.31	1879.14	2956.90	3049.03	3062.65	3105.14
3132.65	3547.34	3574.30	3634.12	3735.39	3849.02

- Conformer (51); ZPE = 36287.076 cm^{-1}:

39.60	49.50	62.17	132.15	198.94	242.99
253.99	260.70	288.47	325.36	343.49	460.57
510.27	532.67	603.04	627.61	635.52	664.59
773.58	781.76	825.61	874.48	931.60	966.85
1061.75	1080.36	1098.02	1158.49	1169.32	1192.29
1214.49	1287.94	1299.05	1321.49	1365.03	1370.08
1430.56	1442.71	1458.73	1468.68	1622.28	1668.62
1810.06	1859.87	3042.31	3049.00	3083.48	3092.99
3118.90	3539.40	3626.36	3642.34	3777.13	3836.91

- Conformer (52); ZPE = 36420.784 cm^{-1}:

46.04	71.62	92.70	118.23	187.83	226.21
271.65	288.08	342.48	396.93	412.32	442.32
537.98	558.25	581.97	630.71	653.96	726.74
760.50	787.15	843.77	898.17	931.33	957.86
1011.37	1065.30	1108.87	1148.47	1190.41	1206.26
1220.55	1277.61	1309.36	1342.05	1356.54	1387.41
1396.62	1453.75	1472.73	1483.97	1628.68	1649.12
1810.21	1851.34	2943.41	3055.48	3063.45	3101.98
3145.46	3556.69	3604.33	3646.04	3747.08	3842.21

- Conformer (53); ZPE = 36346.315 cm^{-1}:

27.52	42.15	53.22	114.76	146.88	215.87
275.89	291.42	304.59	357.10	373.07	473.01
526.35	562.87	569.02	623.11	670.50	736.44
794.98	806.44	855.19	885.96	904.28	1017.12
1079.18	1080.13	1111.28	1137.74	1172.78	1205.19
1243.29	1266.58	1299.21	1331.21	1368.70	1396.17
1426.04	1437.50	1463.82	1482.40	1622.65	1659.59
1807.53	1882.81	3023.55	3030.74	3042.42	3089.92
3121.27	3541.42	3634.51	3641.04	3689.30	3776.91

- Conformer (54); ZPE = 36453.821 cm^{-1}:

40.93	68.32	93.28	123.92	187.17	234.19
271.94	277.64	353.12	395.71	412.93	442.82
541.40	554.22	581.74	633.13	652.45	728.14
758.44	803.19	838.74	901.01	927.73	946.71
1023.76	1059.67	1125.35	1152.22	1182.39	1193.98
1231.14	1295.62	1308.08	1324.43	1382.21	1386.88
1403.73	1408.07	1474.95	1482.04	1629.25	1662.01
1813.92	1838.29	3023.82	3030.51	3075.14	3118.97
3135.45	3550.18	3601.48	3637.78	3745.55	3841.94

- Conformer (55); ZPE = 36431.958 cm^{-1}:

44.63	69.65	91.53	105.86	176.42	248.01
268.38	292.75	312.00	390.27	418.94	436.96
513.00	561.56	593.58	623.42	646.02	737.03
765.54	796.92	834.75	890.62	923.99	1003.22
1040.91	1060.99	1117.97	1149.68	1176.47	1189.31
1223.15	1288.59	1306.23	1318.66	1372.86	1392.85
1406.57	1421.59	1472.75	1487.47	1628.79	1655.12
1813.61	1841.22	3038.32	3045.59	3053.45	3112.91
3133.85	3543.01	3602.59	3635.84	3746.52	3842.00

- Conformer (56); ZPE = 36213.715 cm^{-1}:

17.33	46.73	57.39	104.17	143.67	204.05
219.35	249.16	268.96	331.43	414.07	439.67
519.72	525.69	596.28	628.14	649.61	661.22
776.86	788.80	844.37	895.22	914.23	953.70
1054.73	1080.62	1099.66	1162.26	1169.96	1193.52
1228.80	1271.84	1288.31	1324.70	1355.23	1375.32
1425.91	1439.08	1464.67	1477.32	1622.13	1672.64
1814.13	1854.64	3023.59	3070.77	3075.81	3078.69
3126.92	3540.60	3620.94	3643.97	3779.64	3841.23

- Conformer (57); ZPE = 36201.192 cm^{-1}:

8.40	45.98	57.11	102.79	143.62	202.93
218.61	246.14	269.37	331.68	414.39	439.73
517.76	525.23	597.21	628.90	645.41	661.62
777.16	788.54	843.35	895.08	913.51	953.96
1055.12	1081.64	1099.12	1162.52	1169.48	1192.83
1228.41	1271.39	1287.49	1324.99	1354.85	1375.11
1425.73	1438.96	1464.41	1477.06	1622.77	1672.69
1814.10	1854.35	3023.62	3071.20	3074.72	3079.10
3127.05	3541.13	3621.40	3643.00	3778.24	3841.38

- Conformer (58); ZPE = 36406.042 cm^{-1}:

36.55	55.52	96.00	119.32	173.51	257.47
274.30	297.46	310.96	388.72	404.39	440.97
510.95	551.70	600.60	624.68	647.36	742.36
775.10	788.60	836.06	867.18	901.86	1003.61
1030.06	1087.90	1112.18	1151.33	1162.07	1185.46
1210.64	1256.92	1311.39	1335.29	1343.18	1371.68
1412.66	1452.75	1473.28	1486.35	1629.58	1641.88
1810.92	1877.02	3036.27	3049.20	3070.91	3094.17
3125.26	3544.73	3622.75	3637.59	3754.09	3829.37

- Conformer (59); ZPE = 36302.718 cm^{-1}:

64.39	70.15	97.24	121.45	172.90	238.64
261.29	301.03	320.06	342.57	387.80	482.87
497.96	549.22	556.31	589.45	617.14	662.71
761.79	793.53	836.35	853.16	912.32	986.88
1065.39	1071.23	1116.41	1122.15	1178.62	1194.80
1241.36	1255.53	1306.68	1336.90	1364.28	1379.46
1430.84	1441.74	1450.54	1468.91	1630.49	1651.21
1806.43	1854.59	3041.18	3044.26	3057.54	3087.98
3100.01	3544.22	3632.30	3635.42	3764.80	3852.94

- Conformer (60); ZPE = 36285.049 cm^{-1}:

33.10	46.61	62.08	100.53	192.68	259.68
276.45	296.24	309.38	338.64	384.25	480.96
506.26	530.30	573.35	626.98	630.11	652.74
739.09	779.39	830.44	888.25	917.56	981.41
1053.69	1087.98	1101.14	1162.14	1182.29	1208.83
1229.36	1267.46	1299.50	1334.72	1342.75	1397.46
1423.90	1441.06	1460.98	1471.40	1622.93	1635.13
1801.76	1868.86	2952.38	3045.77	3067.23	3090.39
3119.12	3554.26	3641.03	3646.75	3776.77	3846.60

- Conformer (61); ZPE = 36357.898 cm^{-1}:

47.26	56.21	68.57	119.06	201.41	236.68
248.85	285.30	331.98	371.65	414.82	504.70
509.32	535.81	548.82	585.22	651.68	681.00
745.15	797.87	848.46	872.58	938.56	995.84
1004.31	1085.55	1096.88	1152.57	1172.71	1182.42
1233.74	1286.42	1313.60	1339.89	1356.09	1387.44
1391.11	1428.72	1454.62	1492.08	1636.41	1671.47
1804.50	1879.17	3035.04	3050.11	3063.81	3091.40
3109.69	3544.56	3628.38	3628.75	3755.38	3842.20

- Conformer (62); ZPE = 36454.593 cm^{-1}:

63.80	81.27	128.65	134.37	183.01	236.51
295.31	350.16	389.60	400.46	420.32	461.03
537.20	545.85	565.74	580.91	596.78	735.59
750.57	765.36	800.01	859.21	903.90	968.66
1009.11	1055.08	1112.35	1145.09	1171.79	1212.83
1236.25	1265.86	1315.36	1339.11	1362.03	1375.83
1390.11	1427.69	1473.87	1487.99	1633.97	1665.50
1773.23	1870.05	3056.31	3063.47	3068.48	3111.66
3129.80	3545.36	3624.63	3656.90	3753.89	3821.30

- Conformer (63); ZPE = 36432.721 cm^{-1}:

48.59	76.70	91.95	108.35	183.63	253.42
272.71	288.48	318.92	393.82	412.35	444.67
510.57	560.89	585.92	621.32	639.21	738.14
765.79	781.10	844.22	893.68	919.04	1014.33
1052.46	1058.58	1114.90	1148.61	1182.02	1208.30
1230.91	1281.92	1299.75	1332.49	1345.49	1389.32
1401.14	1455.66	1474.91	1486.31	1628.91	1649.86
1812.34	1855.14	2942.18	3052.33	3059.69	3101.50
3133.38	3557.94	3604.54	3646.71	3747.56	3842.76

- Conformer (64); ZPE = 36370.042 cm^{-1}:

30.06	55.34	82.69	130.62	171.33	212.76
281.77	329.58	362.65	375.11	421.71	438.76
511.83	548.46	559.28	573.77	645.40	724.56
749.44	815.54	854.85	875.61	932.98	964.35
1040.71	1058.18	1121.41	1149.80	1182.52	1191.39
1229.12	1255.11	1304.37	1338.64	1357.76	1374.47
1418.39	1438.29	1468.00	1481.50	1629.59	1639.26
1800.43	1863.12	2974.90	3044.04	3055.68	3107.49
3117.85	3556.39	3632.91	3648.42	3763.16	3848.76

- Conformer (65); ZPE = 36459.075 cm^{-1}:

21.39	69.51	89.71	168.62	189.14	224.19
282.91	294.29	313.49	352.09	422.34	466.14
533.57	535.02	552.90	600.55	636.69	743.85
749.53	787.14	847.44	884.08	940.37	946.88
1045.12	1068.30	1106.75	1174.11	1192.14	1196.48
1229.59	1288.64	1307.52	1348.17	1364.65	1397.62
1417.39	1427.52	1465.99	1485.02	1624.68	1667.20
1784.23	1847.25	3020.58	3058.41	3062.41	3106.18
3118.89	3554.22	3633.58	3640.81	3779.17	3853.73

- Conformer (66); ZPE = 36257.823 cm^{-1}:

32.47	44.58	52.64	117.66	192.69	257.38
263.91	268.07	285.73	315.03	361.62	483.36
513.07	518.73	583.60	620.86	641.51	650.78
771.62	790.21	826.89	862.20	930.47	970.87
1057.61	1080.58	1094.93	1145.94	1170.58	1181.01
1219.12	1286.58	1300.30	1322.96	1368.77	1412.86
1417.10	1429.01	1457.69	1467.20	1619.92	1655.72
1807.53	1852.13	3044.43	3051.32	3073.79	3089.85
3118.76	3539.52	3634.23	3640.42	3775.24	3844.60

- Conformer (67); ZPE = 36443.740 cm^{-1}:

77.52	85.57	107.74	123.97	179.10	227.48
286.90	341.52	352.98	400.56	431.73	470.09
525.84	548.72	553.60	581.70	636.77	751.02
753.85	779.68	835.91	851.38	929.05	966.01
992.70	1077.22	1116.00	1146.44	1183.22	1207.51
1221.55	1253.34	1316.13	1339.34	1357.74	1378.31
1424.98	1456.80	1467.34	1484.29	1632.30	1654.35
1788.34	1838.40	3004.36	3050.98	3061.57	3099.13
3115.76	3536.40	3631.25	3637.18	3762.61	3853.26

- Conformer (68); ZPE = 36328.688 cm^{-1}:

39.70	71.92	84.05	123.40	165.74	183.81
228.84	303.28	365.06	385.79	424.58	445.50
522.12	538.31	585.77	610.46	663.29	723.19
768.91	777.88	818.72	874.84	917.29	946.53
1006.91	1084.97	1103.09	1154.29	1175.26	1199.81
1216.45	1255.70	1305.55	1318.41	1343.12	1371.22
1416.90	1455.04	1470.58	1489.91	1628.25	1638.75
1805.44	1848.51	3042.03	3049.44	3053.60	3103.92
3106.73	3558.31	3626.11	3654.46	3757.16	3848.46

- Conformer (69); ZPE = 36428.287 cm^{-1}:

33.05	69.87	74.30	121.36	204.77	254.03
300.77	320.69	322.69	357.27	427.36	476.56
504.20	540.08	550.07	621.75	635.89	664.69
745.98	771.38	855.49	893.53	940.79	966.83
1055.19	1074.99	1106.60	1168.88	1186.55	1210.44
1242.00	1287.91	1316.48	1333.77	1364.39	1393.02
1404.13	1440.93	1453.62	1480.47	1626.95	1660.54
1788.30	1839.58	3017.57	3046.46	3067.72	3102.21
3117.19	3532.55	3622.81	3639.49	3772.85	3849.57

- Conformer (70); ZPE = 36471.942 cm^{-1}:

40.11	60.93	80.28	121.01	202.30	256.87
268.02	309.01	325.53	419.25	478.66	509.09
536.76	537.37	551.36	584.34	658.00	704.60
747.87	800.42	834.13	870.57	929.78	986.30
1007.64	1091.80	1103.68	1167.70	1177.25	1216.62
1232.65	1303.04	1309.85	1342.42	1385.49	1393.53
1398.99	1413.75	1454.97	1493.80	1648.70	1666.66
1798.77	1836.84	3021.21	3051.78	3060.45	3097.55
3108.24	3551.58	3592.99	3638.56	3728.77	3836.06

- Conformer (71); ZPE = 36236.093 cm^{-1}:

24.10	35.99	50.39	96.62	178.72	239.29
249.83	275.45	291.59	314.14	366.20	477.72
501.44	539.80	593.49	613.01	637.02	651.10
788.60	794.42	834.74	866.37	933.26	982.48
997.89	1073.08	1091.02	1146.61	1163.64	1190.60
1241.42	1291.89	1306.42	1330.07	1373.43	1388.07
1400.97	1426.79	1453.19	1462.29	1621.04	1656.87
1809.95	1874.62	3045.55	3050.91	3091.72	3097.95
3108.73	3540.54	3630.21	3642.22	3777.87	3850.89

- Conformer (72); ZPE = 36326.738 cm^{-1}:

22.80	42.61	60.09	109.36	182.59	275.37
287.99	299.72	344.09	354.58	414.50	487.79
502.51	522.46	561.46	614.76	625.85	653.55
743.04	790.58	840.04	921.61	930.40	974.87
1062.25	1071.61	1101.46	1158.37	1186.86	1212.57
1236.35	1274.77	1307.93	1327.54	1339.43	1394.95
1426.65	1453.87	1457.95	1470.67	1624.54	1648.46
1797.24	1861.40	2917.36	3035.78	3071.48	3083.44
3128.21	3543.42	3631.67	3640.71	3775.46	3848.45

- Conformer (73); ZPE = 36438.863 cm^{-1}:

57.79	80.10	100.77	162.13	175.12	245.81
261.63	295.53	337.99	369.61	439.38	450.10
529.77	556.53	585.32	665.46	703.64	713.72
775.51	803.54	817.74	894.98	900.81	961.33
1012.05	1043.51	1102.01	1149.54	1166.48	1195.45
1242.15	1290.43	1336.69	1349.66	1371.24	1373.89
1405.81	1417.44	1469.68	1484.13	1623.85	1657.90
1811.29	1881.02	3024.21	3033.17	3051.24	3101.11
3116.65	3555.13	3628.24	3643.92	3696.18	3759.36

- Conformer (74); ZPE = 36383.810 cm^{-1}:

57.00	71.11	96.68	104.46	175.97	248.30
256.79	276.69	328.76	355.73	421.07	460.41
516.15	539.88	564.29	598.07	640.31	712.98
739.49	779.15	852.88	903.32	944.79	974.26
1014.94	1059.15	1112.46	1151.15	1159.77	1186.00
1235.29	1272.85	1307.61	1344.26	1368.36	1375.24
1403.99	1418.00	1473.99	1484.12	1624.71	1689.42
1807.43	1873.18	3032.00	3060.21	3066.17	3107.51
3135.29	3519.21	3614.50	3635.05	3769.86	3847.38

- Conformer (75); ZPE = 36226.154 cm^{-1}:

32.43	46.37	51.78	113.42	170.41	208.08
269.68	272.92	282.75	334.83	411.65	449.97
525.28	553.57	560.35	612.31	651.73	668.34
753.30	785.20	824.66	876.71	931.67	974.19
1016.63	1086.96	1099.60	1144.46	1178.67	1201.78
1226.99	1266.36	1305.33	1337.48	1349.35	1379.49
1428.80	1448.03	1467.13	1478.43	1621.69	1648.99
1806.63	1867.43	2942.94	3042.54	3063.32	3091.09
3113.71	3557.86	3644.57	3647.13	3781.05	3846.27

- Conformer (76); ZPE = 36372.250 cm^{-1}:

56.25	73.32	86.50	128.38	164.78	197.96
257.86	292.56	313.34	376.59	405.72	453.54
502.79	546.96	565.04	573.56	630.47	753.80
774.23	811.87	842.76	884.01	913.07	1004.99
1029.35	1065.73	1097.54	1137.67	1165.50	1185.30
1236.39	1255.22	1308.95	1332.47	1360.02	1397.40
1425.93	1436.36	1471.78	1484.29	1630.74	1654.31
1796.45	1849.54	3045.89	3059.19	3075.16	3105.38
3117.22	3533.46	3623.32	3634.88	3766.73	3851.98

- Conformer (77); ZPE = 36234.615 cm^{-1}:

62.03	67.13	81.03	133.26	180.46	217.75
252.98	266.67	309.43	323.13	336.56	472.13
505.40	538.75	570.47	608.93	645.63	656.68
756.29	776.38	833.41	857.06	911.52	990.70
1064.31	1080.08	1106.56	1162.44	1168.87	1200.45
1243.32	1247.45	1302.26	1334.96	1341.82	1370.85
1429.17	1450.90	1454.66	1472.41	1623.36	1644.16
1811.57	1879.74	3014.57	3037.22	3044.94	3082.71
3099.46	3544.92	3639.52	3642.56	3777.97	3842.22

- Conformer (78); ZPE = 36198.435 cm^{-1}:

27.13	46.54	64.46	82.95	167.10	191.07
243.77	270.97	294.64	334.10	424.77	442.49
514.90	535.55	589.86	621.61	661.12	663.93
765.13	793.83	813.48	855.94	922.15	957.54
1026.71	1091.33	1103.52	1147.99	1172.32	1205.02
1240.05	1252.51	1302.25	1317.60	1338.04	1376.44
1428.68	1448.24	1458.74	1486.45	1623.90	1639.62
1812.59	1862.63	3025.92	3040.08	3059.75	3070.57
3108.75	3554.67	3642.19	3648.75	3777.26	3849.24

- Conformer (79); ZPE = 36388.449 cm^{-1}:

30.22	39.21	65.82	91.89	131.98	246.96
270.95	287.64	317.99	363.63	419.31	450.24
533.84	556.04	587.64	637.91	706.83	759.13
776.41	816.15	844.67	871.41	936.19	976.78
1040.48	1079.61	1095.98	1152.01	1157.68	1238.28
1248.81	1257.05	1296.68	1317.98	1375.68	1389.27
1420.13	1430.07	1464.85	1489.78	1626.19	1658.86
1810.73	1889.39	3029.98	3032.73	3048.68	3081.23
3117.14	3553.10	3641.67	3654.50	3679.45	3780.10

- Conformer (80); ZPE = 36452.202 cm^{-1}:

20.93	42.39	60.34	118.14	156.23	245.14
264.04	279.07	301.65	390.12	424.22	468.28
537.43	552.41	605.79	636.38	689.05	749.25
768.32	818.49	854.84	909.72	949.47	994.06
1014.89	1059.70	1132.74	1159.72	1169.55	1196.89
1220.98	1288.55	1314.60	1340.83	1372.58	1382.24
1393.27	1409.52	1468.44	1480.03	1626.31	1660.21
1809.70	1871.85	3031.05	3050.85	3075.60	3100.06
3139.93	3530.18	3574.34	3615.71	3734.92	3843.41

- Conformer (81); ZPE = 36267.133 cm^{-1}:

30.40	52.74	67.37	119.09	175.86	189.90
251.58	282.82	337.43	358.36	413.44	433.87
518.75	544.24	589.02	600.20	654.02	735.19
760.71	792.00	799.12	858.83	924.15	973.01
1007.74	1064.23	1104.53	1152.92	1155.03	1179.39
1212.47	1265.75	1307.18	1340.25	1352.78	1366.66
1413.76	1450.50	1473.78	1485.61	1624.21	1625.42
1803.29	1875.64	3029.41	3050.78	3064.53	3082.77
3126.91	3548.72	3638.00	3650.53	3773.21	3846.14

- Conformer (82); ZPE = 36468.169 cm^{-1}:

34.08	47.91	77.06	110.30	195.68	220.33
280.73	289.21	351.24	407.54	452.27	456.79
540.22	551.68	558.77	640.21	682.78	747.18
753.93	810.49	851.68	914.34	939.75	983.20
1008.28	1066.08	1129.63	1147.43	1181.21	1199.44
1235.61	1254.79	1327.33	1343.51	1349.77	1381.94
1403.93	1448.70	1467.69	1471.22	1624.62	1647.91
1809.67	1874.91	2958.44	3048.41	3057.83	3115.63
3146.04	3543.46	3583.55	3629.08	3738.98	3843.88

- Conformer (83); ZPE = 36316.033 cm^{-1}:

31.39	38.67	65.86	129.33	173.06	259.33
277.45	297.75	319.28	345.17	394.70	471.64
517.42	554.99	577.50	606.32	660.39	674.71
759.31	804.78	827.83	872.12	927.32	956.02
1011.82	1072.52	1100.99	1129.01	1176.39	1194.41
1244.08	1260.45	1306.86	1316.17	1360.19	1384.00
1425.58	1439.09	1456.41	1470.42	1623.51	1659.58
1804.27	1868.24	3039.04	3054.40	3063.56	3086.92
3109.70	3546.96	3631.17	3637.01	3773.08	3843.85

- Conformer (84); ZPE = 36496.917 cm^{-1}:

33.99	46.23	72.43	124.00	196.95	225.69
279.43	292.14	360.00	426.42	451.39	485.50
535.16	557.23	583.19	615.14	669.62	726.56
764.39	806.33	837.44	901.95	937.37	971.79
1041.18	1051.02	1130.73	1157.17	1180.56	1204.47
1230.85	1270.98	1306.84	1341.04	1377.37	1381.84
1389.94	1409.94	1468.74	1484.90	1638.83	1668.40
1803.80	1868.19	3024.33	3063.55	3079.23	3112.89
3128.85	3538.27	3556.65	3620.60	3723.16	3839.17

- Conformer (85); ZPE = 36153.710 cm^{-1}:

16.62	26.70	62.23	89.15	119.04	214.88
239.35	260.19	283.22	289.59	384.69	445.62
513.65	546.57	597.48	625.10	639.90	644.33
785.69	790.62	832.40	875.45	946.95	960.61
1018.26	1084.34	1105.16	1142.51	1161.49	1195.85
1240.24	1274.47	1302.49	1325.50	1368.26	1385.01
1393.35	1428.24	1464.09	1481.53	1622.38	1662.71
1811.91	1866.11	3036.85	3050.45	3067.73	3080.24
3105.16	3545.76	3631.70	3642.88	3777.49	3845.24

- Conformer (86); ZPE = 36589.584 cm^{-1}:

43.58	84.10	105.99	126.77	184.95	208.95
268.02	326.14	375.10	421.19	451.17	506.88
541.28	548.66	589.46	657.87	743.62	765.79
800.21	836.70	858.76	896.51	912.39	955.97
1012.61	1054.06	1104.65	1133.79	1169.90	1210.35
1221.00	1263.19	1317.20	1345.89	1363.02	1369.32
1415.66	1428.92	1478.03	1482.94	1639.27	1655.27
1813.16	1901.42	3047.95	3056.81	3060.02	3107.80
3114.23	3558.30	3614.25	3642.19	3650.96	3736.96

- Conformer (87); ZPE = 36349.205 cm^{-1}:

28.64	41.72	47.42	130.79	165.68	239.89
275.16	317.54	340.77	365.91	427.27	464.45
489.37	544.71	569.60	581.19	638.04	720.64
746.92	800.87	845.82	897.80	935.59	961.36
1035.89	1048.11	1108.51	1160.87	1167.82	1194.18
1206.77	1295.35	1299.84	1344.39	1366.08	1382.71
1410.25	1418.34	1468.88	1476.24	1627.80	1682.97
1805.88	1872.17	3029.18	3052.00	3056.80	3106.90
3113.18	3526.60	3614.92	3637.59	3769.12	3841.95

- Conformer (88); ZPE = 36349.682 cm^{-1}:

26.52	41.39	63.47	127.19	175.95	204.90
300.66	321.24	370.33	389.86	406.94	436.82
487.70	545.73	561.62	575.33	632.16	728.24
751.67	802.32	850.98	908.33	927.40	962.70
1044.59	1054.71	1115.15	1155.97	1177.02	1191.32
1238.23	1257.87	1316.51	1341.38	1348.05	1385.63
1410.52	1450.48	1466.85	1477.18	1627.20	1671.67
1798.25	1868.92	2928.88	3048.12	3053.75	3110.04
3116.35	3549.58	3635.44	3638.98	3770.65	3850.66

- Conformer (89); ZPE = 36307.161 cm^{-1}:

59.19	65.77	96.20	134.86	165.38	198.31
258.06	291.97	326.48	347.99	357.03	477.76
505.89	533.04	577.77	597.91	653.66	673.01
765.43	785.83	836.53	883.53	919.89	997.38
1034.94	1082.97	1096.84	1144.74	1169.59	1181.79
1239.61	1279.29	1303.18	1339.39	1369.94	1374.64
1392.13	1439.38	1452.64	1493.31	1631.33	1666.81
1804.34	1855.14	3025.26	3056.35	3068.09	3098.30
3107.56	3535.83	3619.79	3638.53	3766.45	3837.30

- Conformer (90); ZPE = 36349.506 cm^{-1}:

24.94	49.60	79.95	101.20	109.66	251.44
264.11	288.23	301.90	367.61	425.27	464.71
521.84	551.12	565.16	621.59	666.60	745.99
785.95	807.35	850.65	870.75	921.96	984.78
1060.62	1083.00	1108.65	1145.25	1164.60	1227.43
1253.30	1269.36	1312.12	1328.21	1385.71	1395.15
1395.39	1432.27	1459.76	1490.42	1625.36	1659.07
1809.11	1888.50	3014.78	3035.73	3043.40	3081.11
3101.56	3547.35	3642.03	3645.14	3696.95	3775.33

- Conformer (91); ZPE = 36123.130 cm^{-1}:

20.04	37.57	70.65	97.36	128.99	192.65
231.04	234.15	278.39	325.59	333.86	438.01
501.56	539.00	606.01	626.83	631.36	649.60
760.10	800.23	843.34	876.80	908.40	1015.15
1051.25	1084.95	1107.02	1142.23	1166.60	1181.50
1222.80	1278.85	1295.48	1318.17	1355.90	1376.52
1423.21	1428.98	1458.86	1481.24	1621.11	1666.76
1817.85	1865.23	3033.44	3047.71	3060.74	3075.67
3118.93	3539.29	3622.81	3642.94	3778.19	3835.36

- Conformer (92); ZPE = 36185.247 cm^{-1}:

31.12	41.42	48.00	110.04	171.79	220.47
261.28	263.36	269.80	318.89	356.24	473.08
511.56	513.75	591.52	613.83	639.14	647.35
779.81	791.97	822.14	829.47	925.70	970.23
1060.67	1092.28	1112.60	1137.16	1173.69	1182.28
1218.69	1270.25	1298.52	1327.84	1360.14	1396.43
1423.84	1448.40	1461.37	1463.37	1621.98	1650.84
1804.88	1853.09	3040.62	3052.76	3080.43	3084.73
3110.97	3540.91	3635.65	3641.67	3776.73	3845.71

- Conformer (93); ZPE = 36118.605 cm^{-1}:

20.86	33.99	53.58	103.73	124.21	197.47
231.76	240.45	278.29	325.66	334.85	455.09
502.60	553.86	599.50	616.15	626.88	632.08
761.85	800.90	832.35	876.87	889.88	1005.60
1035.24	1085.11	1104.35	1142.26	1176.53	1192.97
1231.82	1275.65	1312.49	1335.15	1349.41	1387.24
1392.91	1431.64	1462.57	1484.90	1622.31	1662.16
1814.27	1855.07	3036.91	3049.32	3064.97	3077.67
3113.46	3545.86	3631.80	3642.03	3776.56	3846.13

- Conformer (94); ZPE = 36314.050 cm^{-1}:

27.97	41.61	71.96	119.56	175.26	189.91
267.86	293.84	329.01	362.63	409.99	432.34
535.86	549.45	592.08	598.23	638.94	736.17
775.35	795.00	827.06	858.56	921.96	972.72
1007.91	1064.51	1101.03	1130.22	1158.07	1206.28
1218.08	1266.78	1315.36	1345.55	1362.16	1373.30
1414.27	1431.86	1472.92	1485.31	1625.94	1641.67
1799.57	1869.27	3030.37	3064.03	3070.16	3086.48
3127.26	3539.65	3635.69	3638.95	3774.09	3848.05

- Conformer (95); ZPE = 36415.277 cm^{-1}:

40.51	59.63	86.33	122.99	192.50	244.57
279.30	320.56	345.79	424.30	467.97	499.90
531.69	535.04	547.61	572.41	646.97	700.64
743.88	799.62	831.39	884.62	929.53	975.14
1028.10	1087.16	1107.76	1148.31	1198.08	1217.47
1228.07	1272.32	1324.02	1342.44	1361.56	1387.10
1402.43	1452.19	1455.52	1498.16	1647.59	1653.73
1799.02	1841.28	2925.04	3053.41	3061.13	3095.57
3108.14	3551.89	3590.03	3639.16	3726.91	3844.06

- Conformer (96); ZPE = 36185.236 cm^{-1}:

19.36	41.23	55.64	124.02	136.04	206.10
261.47	270.48	278.91	294.33	352.35	470.43
509.73	536.91	590.51	615.54	621.53	660.41
772.48	787.13	833.12	884.25	894.63	1022.86
1048.21	1081.12	1108.45	1142.79	1159.71	1182.48
1231.94	1272.68	1305.80	1318.18	1359.61	1397.78
1420.53	1432.89	1461.65	1476.71	1622.91	1655.25
1814.55	1858.95	3027.19	3035.68	3056.70	3088.80
3123.47	3543.34	3635.54	3643.43	3779.74	3844.95

- Conformer (97); ZPE = 36282.722 cm^{-1}:

46.79	62.68	83.47	135.96	173.17	210.40
256.61	261.89	283.50	333.07	391.70	463.65
505.24	547.97	570.36	608.05	639.38	671.42
771.41	777.96	833.39	878.02	924.16	971.50
991.12	1074.39	1103.53	1147.89	1161.60	1196.72
1244.25	1254.88	1305.73	1338.09	1377.27	1392.90
1400.57	1430.60	1458.11	1461.53	1623.55	1667.07
1810.30	1880.69	3041.04	3062.10	3082.37	3089.22
3118.34	3551.40	3630.69	3642.94	3778.60	3846.22

- Conformer (98); ZPE = 36356.642 cm^{-1}:

45.97	61.18	64.18	112.57	172.37	219.39
233.39	277.35	351.05	369.56	427.34	465.83
486.83	558.41	582.00	600.61	655.73	713.48
750.10	800.47	852.77	915.38	923.67	947.22
1030.63	1054.12	1115.06	1157.36	1165.09	1187.08
1244.54	1296.56	1302.44	1320.89	1379.23	1384.28
1386.76	1404.79	1476.31	1486.87	1626.99	1672.39
1821.60	1871.58	3014.20	3023.54	3079.18	3117.27
3132.43	3541.50	3619.94	3625.28	3758.85	3829.67

- Conformer (99); ZPE = 36401.462 cm^{-1}:

34.63	87.28	91.51	134.27	182.76	197.64
252.31	307.26	347.15	365.08	431.59	461.05
509.08	548.57	580.77	623.43	678.68	728.91
782.40	796.85	867.62	920.99	936.20	960.76
1034.47	1069.10	1112.16	1166.65	1194.39	1224.42
1249.49	1288.95	1302.05	1319.77	1380.53	1392.62
1401.49	1439.55	1461.08	1488.28	1629.82	1671.22
1784.94	1860.92	3014.30	3019.08	3069.53	3093.56
3118.03	3524.81	3600.95	3635.11	3658.67	3770.17

- Conformer (100); ZPE = 36306.719 cm^{-1}:

39.63	58.31	66.09	113.08	174.74	214.38
254.81	296.08	325.72	360.87	419.30	478.61
499.70	554.84	575.44	599.81	652.94	717.90
749.15	791.60	841.96	876.97	934.26	955.67
1004.88	1072.15	1100.96	1144.56	1178.40	1205.34
1223.18	1274.93	1302.15	1339.90	1343.75	1384.96
1395.29	1439.54	1469.82	1489.82	1624.08	1636.95
1818.08	1873.80	2941.84	3037.26	3062.33	3104.75
3152.63	3559.99	3628.06	3653.20	3761.84	3837.15

- Conformer (101); ZPE = 36332.597 cm^{-1}:

58.61	73.93	91.21	138.94	165.16	243.23
259.46	293.05	309.73	334.81	382.40	437.25
502.62	543.47	554.98	573.03	656.09	708.82
750.74	803.64	843.07	886.93	935.15	992.50
1035.62	1070.67	1098.69	1141.52	1176.11	1189.21
1228.56	1277.04	1312.88	1329.17	1369.64	1379.99
1397.26	1447.08	1467.66	1471.37	1628.02	1666.08
1801.78	1856.44	3034.83	3056.82	3078.13	3108.43
3119.91	3533.21	3621.38	3639.48	3769.96	3819.44

- Conformer (102); ZPE = 36203.344 cm^{-1}:

30.47	46.32	51.14	121.06	150.94	208.82
264.95	286.01	288.83	305.64	363.57	471.85
514.07	540.79	577.62	613.00	613.62	683.85
759.18	783.47	822.71	890.79	901.33	1012.00
1072.11	1081.21	1100.08	1145.66	1170.21	1195.26
1244.59	1266.72	1303.06	1323.41	1338.12	1385.26
1430.42	1452.58	1466.53	1480.21	1622.72	1646.51
1809.59	1870.64	2933.78	3030.56	3056.06	3097.54
3114.23	3556.10	3641.44	3645.30	3778.19	3846.54

- Conformer (103); ZPE = 36175.816 cm^{-1}:

34.10	36.07	52.80	106.89	182.32	225.75
245.76	259.65	306.43	327.25	379.02	471.62
501.85	518.83	595.78	621.20	636.28	660.53
756.29	786.91	807.21	845.79	920.70	972.07
1010.72	1086.97	1112.60	1141.92	1164.33	1196.66
1228.03	1256.03	1298.86	1323.72	1361.20	1378.19
1424.81	1452.87	1464.52	1468.53	1622.10	1630.94
1813.66	1864.32	3035.36	3039.83	3068.34	3077.48
3104.36	3552.55	3640.56	3655.41	3776.38	3849.27

- Conformer (104); ZPE = 36173.604 cm^{-1}:

24.93	34.95	76.83	96.09	150.97	190.46
219.57	265.70	300.56	319.78	337.80	443.60
512.10	544.30	606.85	625.74	646.15	662.50
762.41	802.10	840.96	869.67	886.42	1017.34
1077.00	1086.17	1102.90	1154.27	1168.85	1177.54
1229.67	1263.34	1304.00	1328.33	1342.22	1365.55
1429.10	1454.46	1461.02	1484.29	1621.47	1642.00
1813.50	1868.35	3030.16	3036.77	3048.36	3078.77
3102.83	3542.58	3633.90	3643.82	3779.56	3838.64

- Conformer (105); ZPE = 36397.227 cm^{-1}:

27.51	57.34	74.62	117.14	172.28	204.95
232.29	299.58	351.37	399.04	439.88	457.22
501.29	519.70	545.80	632.22	664.99	736.36
750.33	793.10	857.07	915.43	974.74	992.42
1018.08	1051.08	1109.25	1162.44	1171.49	1193.21
1226.48	1288.02	1307.37	1338.35	1346.42	1382.46
1409.01	1416.65	1468.57	1483.71	1629.11	1667.70
1806.06	1895.88	3003.39	3039.10	3079.16	3092.69
3137.39	3528.53	3603.25	3608.09	3741.83	3873.02

- Conformer (106); ZPE = 36375.398 cm^{-1}:

56.21	73.57	82.36	131.72	165.68	212.57
264.54	280.49	325.30	404.25	417.91	456.95
497.84	547.94	565.36	571.39	619.32	748.52
766.71	800.91	833.05	891.66	916.63	988.89
1039.41	1064.14	1116.85	1133.90	1177.91	1193.74
1235.78	1255.02	1319.01	1331.65	1340.61	1387.22
1426.35	1462.23	1469.83	1485.34	1633.28	1651.90
1799.04	1870.32	2992.68	3051.46	3057.35	3099.50
3115.07	3546.58	3631.05	3632.64	3759.98	3851.17

- Conformer (107); ZPE = 36552.314 cm^{-1}:

55.19	70.50	91.70	109.54	158.03	237.88
312.88	329.02	383.03	399.32	432.87	467.46
542.03	548.72	562.50	577.31	729.84	763.37
771.22	809.33	851.24	858.30	918.73	1008.43
1021.27	1094.25	1105.51	1155.38	1173.59	1207.70
1241.71	1252.75	1319.77	1340.54	1375.69	1394.64
1428.03	1435.43	1469.24	1483.23	1636.44	1657.56
1805.40	1884.78	3036.83	3048.28	3057.21	3094.73
3114.39	3544.63	3622.32	3638.00	3729.86	3747.01

- Conformer (108); ZPE = 36396.875 cm^{-1}:

33.21	66.46	95.34	122.34	187.34	200.47
232.53	287.51	355.42	399.33	415.10	443.08
539.57	558.73	580.90	633.32	660.00	730.75
763.72	790.88	833.36	861.97	935.29	956.02
1007.78	1093.88	1114.98	1147.73	1189.96	1202.18
1230.79	1280.97	1301.19	1318.26	1369.65	1383.55
1395.61	1452.17	1474.01	1483.22	1628.26	1664.54
1812.13	1831.65	3026.76	3034.63	3060.35	3102.52
3132.41	3546.85	3599.48	3637.06	3745.07	3843.46

- Conformer (109); ZPE = 36267.686 cm^{-1}:

58.27	65.32	78.35	119.81	177.08	195.46
266.98	299.38	328.88	347.69	385.97	484.09
496.87	552.87	573.48	590.05	615.50	660.54
764.78	781.70	820.84	853.44	899.32	993.82
1044.67	1078.67	1115.02	1149.91	1172.42	1217.09
1232.46	1261.27	1307.95	1334.18	1376.83	1383.86
1430.95	1441.24	1451.26	1475.02	1629.61	1655.43
1809.34	1841.31	2975.86	3042.67	3050.12	3089.29
3108.74	3556.77	3634.88	3646.40	3764.13	3847.54

- Conformer (110); ZPE = 36206.979 cm^{-1}:

21.08	30.40	56.15	122.56	147.33	214.28
257.21	262.08	270.56	328.92	388.01	442.40
522.22	556.31	562.24	611.39	645.64	666.60
760.59	778.67	830.45	890.01	904.05	959.42
1054.19	1071.76	1102.28	1161.82	1177.50	1185.08
1233.81	1269.07	1309.45	1326.07	1372.09	1392.63
1401.91	1430.34	1457.25	1469.05	1621.35	1665.27
1815.34	1857.57	3028.52	3034.98	3079.34	3079.92
3132.00	3551.79	3635.85	3642.29	3777.55	3849.30

- Conformer (111); ZPE = 36134.810 cm^{-1}:

17.14	40.73	62.65	94.81	135.92	194.72
229.71	252.05	297.66	318.36	334.69	457.29
506.93	553.32	601.39	613.02	624.05	643.59
764.17	804.39	845.88	864.16	880.29	1015.40
1075.83	1077.97	1106.89	1118.48	1163.59	1178.14
1242.02	1262.03	1309.35	1329.55	1360.24	1369.55
1425.17	1440.56	1458.42	1479.86	1621.43	1652.53
1812.17	1865.61	3032.36	3044.74	3064.67	3078.25
3101.02	3545.90	3633.71	3641.41	3776.49	3849.39

- Conformer (112); ZPE = 36260.455 cm^{-1}:

26.66	54.76	96.99	142.41	178.85	203.57
219.39	259.29	305.09	365.44	426.94	469.33
504.54	542.37	567.37	582.64	612.88	736.38
755.73	788.49	841.71	863.65	929.99	967.98
992.23	1074.11	1110.07	1138.19	1178.61	1194.16
1217.07	1248.96	1325.16	1331.59	1348.13	1380.18
1405.34	1444.97	1475.25	1482.38	1623.59	1644.55
1809.31	1874.28	2972.83	3048.91	3057.46	3096.74
3122.25	3558.09	3643.30	3655.96	3778.11	3846.71

- Conformer (113); ZPE = 36277.351 cm^{-1}:

36.24	43.68	53.89	116.87	168.20	240.36
263.00	297.45	322.49	337.96	377.76	471.68
517.89	538.82	587.97	605.44	645.68	675.76
767.16	814.67	842.15	861.62	927.25	957.60
1045.42	1068.31	1100.20	1123.30	1173.27	1190.28
1237.42	1279.74	1302.55	1323.03	1373.70	1378.74
1423.62	1431.14	1455.49	1471.52	1622.30	1655.36
1811.05	1868.27	3031.34	3045.07	3065.12	3082.64
3101.94	3541.43	3629.09	3632.89	3767.56	3851.33

- Conformer (114); ZPE = 36260.089 cm^{-1}:

33.19	36.38	60.27	119.17	162.92	220.33
256.65	281.36	321.83	353.09	408.25	433.85
509.80	546.47	575.08	609.18	660.58	709.13
759.64	808.24	815.45	859.97	921.14	960.51
1036.56	1058.02	1108.08	1148.07	1161.70	1179.59
1219.80	1279.15	1303.85	1338.63	1361.16	1372.02
1405.71	1453.57	1471.70	1474.79	1625.24	1640.68
1801.14	1873.28	3021.67	3048.64	3052.00	3083.03
3116.26	3554.47	3639.36	3650.90	3773.75	3844.90

- Conformer (115); ZPE = 36436.005 cm^{-1}:

55.66	86.08	93.35	152.39	169.00	245.16
258.30	294.96	358.48	432.93	434.06	450.78
528.99	534.02	540.58	604.50	672.12	720.92
767.09	800.11	837.98	893.13	915.08	955.60
1017.26	1062.93	1115.10	1168.01	1176.44	1192.12
1240.75	1266.11	1307.07	1326.40	1334.20	1378.25
1427.29	1434.88	1475.46	1479.02	1632.55	1660.03
1798.07	1879.66	2972.56	3038.60	3056.75	3089.23
3108.71	3554.06	3615.32	3637.21	3743.55	3883.14

- Conformer (116); ZPE = 36357.078 cm^{-1}:

53.19	62.33	84.12	144.78	158.43	242.55
260.02	266.12	318.03	341.04	423.94	435.01
536.34	542.45	593.56	602.60	674.60	735.66
756.14	794.97	819.00	862.63	898.22	949.12
1020.99	1048.79	1100.85	1157.09	1176.84	1195.18
1214.20	1288.55	1304.89	1338.11	1366.25	1379.11
1396.93	1415.35	1462.02	1478.54	1622.94	1674.49
1819.36	1866.93	3050.93	3057.07	3070.99	3111.16
3115.00	3560.12	3628.62	3643.36	3764.02	3830.63

- Conformer (117); ZPE = 36306.090 cm^{-1}:

52.74	67.76	98.23	135.87	165.21	250.46
263.04	303.02	319.79	341.66	351.21	484.92
507.04	533.05	572.24	602.85	652.55	672.94
765.05	787.76	834.68	850.57	903.97	986.46
1064.29	1074.74	1102.76	1151.22	1173.39	1179.14
1241.84	1256.25	1327.31	1340.20	1351.16	1365.77
1393.92	1451.36	1471.04	1493.21	1631.46	1640.36
1804.86	1858.63	3026.70	3049.71	3055.69	3077.20
3105.25	3543.75	3636.62	3637.92	3763.52	3839.83

- Conformer (118); ZPE = 36105.613 cm^{-1}:

16.02	50.17	66.77	102.22	106.56	187.48
224.47	237.13	261.59	321.27	400.19	449.13
505.48	521.79	584.38	620.34	632.72	658.76
768.41	786.16	833.72	859.49	910.09	958.21
1072.08	1090.54	1109.03	1158.47	1171.99	1193.55
1238.72	1258.69	1308.54	1318.81	1357.64	1364.56
1425.51	1450.19	1458.66	1477.15	1623.05	1640.34
1817.86	1868.26	3016.58	3026.34	3055.64	3067.72
3109.03	3550.76	3643.61	3648.96	3777.99	3848.37

- Conformer (119); ZPE = 36191.312 cm^{-1}:

19.80	31.89	63.30	88.70	141.54	201.25
240.31	251.85	277.58	328.60	420.09	443.60
512.95	547.60	590.03	619.52	635.89	665.14
777.15	780.16	827.49	890.02	896.42	947.02
1053.37	1069.27	1096.85	1157.64	1173.46	1206.93
1227.07	1269.58	1299.74	1329.74	1363.03	1378.09
1399.22	1425.17	1457.85	1473.03	1622.85	1670.98
1818.35	1864.74	3021.81	3065.65	3072.18	3080.41
3130.66	3557.26	3636.83	3643.71	3779.29	3840.00

- Conformer (120); ZPE = 36192.430 cm^{-1}:

22.26	42.83	46.92	105.81	163.12	205.69
264.78	268.07	305.30	335.97	364.92	463.49
512.91	548.45	570.27	617.69	637.61	672.38
739.16	780.90	819.86	875.51	894.67	1008.93
1075.31	1086.39	1100.33	1152.16	1183.38	1195.87
1249.03	1252.62	1299.63	1323.72	1345.52	1382.79
1429.66	1444.43	1461.06	1484.23	1621.15	1634.92
1812.37	1866.34	2919.54	3030.65	3057.13	3099.33
3119.76	3565.47	3642.41	3661.38	3779.54	3841.25

- Conformer (121); ZPE = 36307.363 cm^{-1}:

41.71	54.71	76.53	121.98	199.47	223.65
255.89	296.38	336.75	384.53	413.33	500.83
515.20	528.39	546.83	587.84	653.49	675.65
749.59	793.70	816.94	857.23	912.55	974.04
1016.23	1086.17	1110.86	1151.74	1157.15	1196.30
1228.30	1275.04	1304.21	1340.32	1360.02	1378.65
1391.98	1454.00	1456.25	1490.82	1636.27	1638.52
1806.97	1884.72	3031.37	3041.13	3055.05	3086.63
3099.04	3547.57	3627.80	3644.48	3753.59	3846.35

- Conformer (122); ZPE = 36424.010 cm^{-1}:

44.25	58.11	81.50	124.96	201.14	225.84
275.15	310.67	331.83	409.71	494.36	507.80
531.52	541.45	557.86	580.47	656.84	704.11
754.98	795.56	821.27	854.94	909.51	980.11
1019.89	1094.75	1117.34	1140.28	1170.46	1222.65
1231.23	1275.80	1311.88	1342.18	1375.00	1389.07
1401.28	1454.12	1458.05	1492.07	1650.59	1654.13
1798.83	1836.13	3031.63	3046.47	3051.97	3090.88
3099.89	3548.31	3587.31	3639.83	3724.93	3837.09

- Conformer (123); ZPE = 36300.944 cm^{-1}:

48.70	60.09	69.42	163.98	183.55	218.21
219.09	273.13	302.23	333.54	390.44	469.11
509.47	541.17	585.74	605.45	650.92	661.14
755.69	789.02	849.57	873.16	923.49	957.33
1035.98	1072.15	1101.18	1138.80	1167.48	1211.23
1229.34	1280.04	1305.76	1331.53	1363.94	1382.93
1433.00	1439.02	1462.08	1468.51	1624.16	1670.83
1802.12	1860.27	3032.98	3052.35	3073.91	3083.91
3107.34	3543.49	3625.13	3641.51	3776.82	3850.45

- Conformer (124); ZPE = 36344.237 cm^{-1}:

46.29	59.49	78.51	95.43	206.23	227.44
276.89	294.23	336.84	390.33	399.99	444.43
516.59	546.39	581.23	584.23	656.41	698.78
760.34	794.79	844.73	903.87	930.02	950.48
1014.39	1082.31	1111.65	1137.69	1174.73	1195.08
1230.62	1247.68	1301.94	1328.70	1357.33	1375.45
1425.73	1453.77	1470.72	1483.37	1627.49	1649.92
1816.49	1872.78	2961.36	3052.82	3057.50	3098.78
3105.14	3552.07	3630.83	3640.04	3760.31	3847.83

- Conformer (125); ZPE = 36235.466 cm^{-1}:

58.95	62.06	85.24	126.19	177.16	231.54
244.73	276.85	307.59	329.13	384.80	463.49
500.73	550.72	558.93	603.90	635.33	674.71
763.50	774.92	824.87	851.61	932.05	954.56
985.66	1087.81	1104.53	1112.42	1156.69	1210.17
1234.60	1248.30	1304.39	1333.96	1360.03	1390.15
1423.92	1435.09	1455.94	1463.49	1622.05	1646.23
1813.19	1892.35	3038.41	3046.64	3079.76	3083.25
3101.36	3553.31	3643.06	3646.60	3777.86	3846.17

- Conformer (126); ZPE = 36304.629 cm^{-1}:

37.99	57.71	63.83	123.14	165.66	255.70
289.16	315.95	338.45	369.42	416.32	447.41
510.63	547.97	570.44	595.07	663.68	675.79
758.62	803.97	836.40	866.02	920.78	957.35
1004.39	1061.87	1100.53	1152.44	1169.58	1184.53
1228.11	1267.93	1309.59	1326.50	1349.65	1378.13
1394.19	1456.04	1464.60	1481.19	1621.68	1644.77
1819.63	1873.70	3032.27	3042.25	3046.92	3089.69
3107.12	3541.28	3629.52	3635.42	3761.08	3847.18

- Conformer (127); ZPE = 36190.434 cm^{-1}:

27.08	37.92	67.94	80.79	161.24	190.27
240.53	266.89	297.29	323.26	423.86	432.73
527.35	554.51	581.69	608.81	656.83	678.64
758.13	796.93	815.55	850.44	919.82	953.99
1024.74	1093.27	1098.94	1121.79	1164.90	1222.78
1235.11	1248.61	1301.61	1320.41	1360.70	1376.72
1425.46	1436.34	1453.76	1480.13	1623.25	1651.06
1812.33	1868.62	3028.12	3052.38	3068.64	3075.56
3107.73	3556.88	3643.77	3647.86	3780.93	3845.98

- Conformer (128); ZPE = 36411.472 cm^{-1}:

44.34	68.40	91.74	104.52	180.09	241.94
290.66	307.31	329.18	351.21	410.72	472.75
503.11	548.43	596.93	660.30	676.71	741.74
774.80	785.84	841.17	905.28	920.16	958.78
1005.41	1067.24	1094.07	1131.05	1158.53	1207.21
1240.09	1272.74	1305.49	1353.55	1368.20	1381.17
1414.26	1429.16	1459.72	1465.74	1624.83	1655.09
1823.11	1910.28	3040.06	3044.95	3052.74	3086.60
3109.93	3546.91	3630.82	3637.14	3729.19	3771.55

- Conformer (129); ZPE = 36245.026 cm^{-1}:

32.82	47.61	85.96	96.39	106.68	252.40
254.35	296.42	302.96	331.93	413.88	459.25
516.16	554.53	555.52	590.86	634.74	665.63
750.97	782.50	848.08	889.99	915.00	972.57
1072.70	1089.66	1106.22	1162.30	1183.48	1188.37
1254.97	1268.88	1309.98	1331.29	1363.29	1380.81
1396.68	1449.11	1459.73	1484.33	1624.66	1658.18
1807.14	1872.78	2932.43	3034.59	3047.12	3081.55
3100.68	3562.47	3642.58	3645.79	3776.22	3844.89

- Conformer (130); ZPE = 36302.327 cm^{-1}:

45.94	72.56	98.57	123.70	163.73	190.23
237.88	276.92	317.49	380.58	409.85	445.73
489.48	545.37	555.24	573.07	631.45	751.19
778.10	806.27	828.89	847.27	915.09	997.41
1030.29	1078.70	1113.06	1127.74	1170.09	1183.70
1234.44	1239.40	1315.86	1334.32	1359.59	1382.49
1422.37	1462.96	1470.41	1483.72	1629.14	1650.12
1800.65	1848.46	3037.13	3054.29	3072.87	3089.75
3113.93	3539.37	3629.96	3634.82	3759.66	3853.34

- Conformer (131); ZPE = 36422.696 cm^{-1}:

50.56	66.85	104.99	129.50	177.56	220.67
263.21	295.36	316.40	381.63	442.31	470.56
530.38	544.31	605.89	653.25	690.33	710.89
765.75	795.49	841.74	877.52	931.17	975.80
1048.58	1065.13	1104.99	1156.65	1180.99	1188.81
1204.46	1285.56	1289.25	1325.30	1357.41	1364.78
1408.61	1426.73	1464.24	1477.06	1620.45	1667.88
1828.83	1886.75	3020.92	3052.69	3057.41	3103.10
3124.16	3532.97	3600.89	3618.23	3727.77	3812.71

- Conformer (132); ZPE = 36268.581 cm^{-1}:

32.38	36.40	58.19	118.04	160.62	223.36
265.93	284.60	310.43	361.13	400.33	437.96
530.86	542.79	575.73	582.87	641.04	709.35
764.53	817.61	841.46	856.30	925.51	958.10
1037.80	1051.85	1104.02	1121.62	1168.01	1181.51
1233.92	1278.73	1310.03	1339.25	1370.47	1375.35
1403.24	1430.37	1471.44	1476.10	1624.71	1656.37
1804.18	1875.57	3022.66	3048.69	3070.19	3083.36
3114.46	3547.81	3636.64	3639.82	3773.68	3849.81

- Conformer (133); ZPE = 36284.327 cm^{-1}:

54.73	77.14	85.65	138.76	164.76	243.79
253.56	287.73	309.83	333.64	379.86	436.03
503.37	543.48	556.70	574.57	651.01	711.03
753.00	803.84	842.65	861.23	912.19	998.42
1042.10	1053.16	1115.59	1143.37	1184.59	1190.25
1224.38	1255.68	1322.27	1333.82	1357.09	1367.76
1396.87	1464.11	1467.83	1482.58	1627.73	1639.39
1803.97	1860.28	3031.92	3054.96	3065.62	3089.69
3117.99	3536.41	3630.48	3639.26	3770.03	3822.48

- Conformer (134); ZPE = 36346.742 cm^{-1}:

41.13	56.00	62.05	102.52	162.84	230.81
281.47	298.59	307.68	336.97	388.70	433.59
532.80	565.07	592.59	596.27	629.73	730.18
768.28	804.57	826.33	882.32	912.78	998.63
1028.98	1052.99	1100.21	1142.01	1168.95	1193.05
1237.66	1297.18	1298.74	1342.35	1366.61	1375.02
1393.48	1409.05	1475.37	1493.87	1625.65	1654.65
1821.17	1875.61	3029.54	3048.69	3073.26	3111.99
3134.95	3544.25	3626.00	3637.71	3760.27	3832.36

- Conformer (135); ZPE = 36346.236 cm^{-1}:

40.46	52.42	66.16	108.83	189.43	248.07
289.84	296.41	322.53	335.04	407.25	442.30
511.40	553.15	570.71	606.59	635.72	725.75
754.70	776.88	841.35	882.75	913.01	1008.86
1054.88	1065.78	1106.99	1152.95	1179.00	1209.00
1238.16	1270.06	1298.28	1335.64	1346.31	1384.64
1400.86	1436.68	1480.00	1491.23	1622.28	1641.73
1820.98	1873.41	2921.65	3054.31	3062.66	3099.97
3131.99	3558.32	3629.19	3650.85	3763.67	3831.40

- Conformer (136); ZPE = 36189.793 cm^{-1}:

21.97	41.10	77.29	89.99	122.39	239.29
250.82	271.47	283.21	332.55	396.74	452.41
507.30	534.12	572.82	608.69	629.41	688.86
743.52	788.04	844.92	893.61	910.21	962.58
1071.22	1079.42	1110.04	1162.92	1176.66	1182.13
1246.71	1257.11	1302.99	1321.97	1344.35	1378.89
1421.72	1449.22	1464.47	1479.64	1623.26	1656.57
1816.42	1879.42	2933.09	3017.23	3064.67	3076.30
3126.57	3559.04	3641.57	3643.38	3782.09	3847.18

- Conformer (137); ZPE = 36160.900 cm^{-1}:

23.27	32.66	44.54	104.98	177.47	231.66
250.49	279.89	309.87	323.12	362.46	476.64
501.42	533.71	586.59	614.52	633.62	647.89
775.10	779.78	802.20	841.68	915.07	971.66
1005.38	1081.26	1096.01	1121.85	1156.61	1206.56
1228.62	1261.93	1299.98	1332.90	1367.89	1379.83
1420.15	1431.03	1460.11	1465.79	1621.53	1642.85
1815.76	1881.18	3037.28	3041.62	3078.89	3095.91
3109.74	3548.67	3637.68	3648.04	3772.84	3853.63

- Conformer (138); ZPE = 36242.543 cm^{-1}:

61.96	72.65	90.62	114.09	175.30	237.26
245.15	252.98	305.99	341.76	412.79	463.26
528.83	540.54	562.92	587.33	643.85	699.30
741.93	774.68	837.54	853.46	928.56	967.71
992.24	1064.70	1104.35	1149.35	1159.66	1192.80
1216.64	1265.79	1306.13	1329.79	1344.02	1378.24
1404.27	1446.99	1467.21	1486.49	1623.30	1648.37
1814.54	1883.69	3023.47	3045.24	3059.67	3087.10
3128.77	3534.70	3632.76	3636.24	3766.31	3851.76

- Conformer (139); ZPE = 36374.290 cm^{-1}:

34.53	50.86	77.24	152.65	195.02	257.49
279.50	308.23	347.81	410.34	417.04	485.45
531.08	543.58	552.82	599.93	630.74	691.17
744.65	770.23	816.64	879.52	919.75	971.30
993.14	1074.38	1108.08	1154.53	1191.18	1205.47
1231.26	1257.38	1332.35	1347.50	1356.10	1378.75
1409.12	1447.50	1455.60	1493.88	1638.10	1651.86
1808.96	1849.22	2926.05	3051.62	3059.18	3093.27
3123.70	3563.51	3624.51	3653.09	3754.32	3847.41

- Conformer (140); ZPE = 36266.781 cm^{-1}:

27.17	49.43	81.51	119.08	176.96	248.51
280.58	305.46	324.93	369.10	387.07	455.08
501.42	538.62	567.27	585.18	639.14	664.01
765.63	790.01	819.42	864.10	944.34	979.64
1000.03	1061.09	1087.59	1152.20	1170.17	1177.59
1240.83	1291.26	1305.78	1334.53	1365.98	1386.73
1401.15	1426.46	1454.54	1479.48	1621.19	1653.90
1825.44	1861.78	3027.20	3045.63	3049.24	3096.30
3111.78	3546.47	3627.05	3644.60	3756.73	3847.14

- Conformer (141); ZPE = 36532.244 cm^{-1}:

38.17	54.93	98.89	150.23	214.79	247.19
290.51	316.08	324.02	354.64	484.24	507.60
523.96	540.90	593.38	638.06	652.21	714.68
772.15	804.33	825.61	835.15	933.45	967.34
1017.76	1098.27	1112.91	1137.39	1176.90	1195.10
1227.64	1275.26	1317.69	1349.11	1376.98	1388.83
1401.75	1457.16	1466.57	1501.36	1649.57	1652.57
1797.86	1842.17	3051.75	3058.82	3079.76	3096.37
3103.87	3558.31	3576.60	3653.87	3722.74	3837.03

- Conformer (142); ZPE = 36294.593 cm^{-1}:

20.62	34.45	58.10	127.77	160.98	243.72
292.74	303.45	341.73	356.70	425.96	464.80
526.69	553.30	562.57	581.19	653.99	692.86
758.90	805.61	833.66	858.27	921.56	957.62
1009.99	1064.49	1097.73	1139.44	1177.22	1185.74
1233.46	1265.83	1310.44	1334.26	1367.79	1376.62
1399.12	1444.68	1470.02	1474.08	1621.91	1660.36
1810.71	1856.32	3033.53	3042.80	3047.12	3097.63
3104.03	3546.33	3632.87	3634.06	3766.91	3846.45

- Conformer (143); ZPE = 36283.784 cm^{-1}:

40.44	43.05	80.18	112.10	177.83	261.14
282.39	340.48	370.07	388.39	421.41	482.92
492.26	517.66	545.74	590.70	615.84	681.46
744.06	787.86	816.02	874.71	916.79	964.80
1000.31	1070.53	1110.82	1158.87	1177.76	1195.44
1217.65	1257.73	1305.38	1315.44	1346.95	1382.04
1424.66	1443.32	1452.65	1478.55	1627.75	1628.66
1802.50	1896.14	2928.02	3042.34	3050.43	3092.19
3098.46	3563.97	3633.31	3666.25	3762.33	3888.81

- Conformer (144); ZPE = 36131.955 cm^{-1}:

24.38	36.44	70.56	90.08	112.48	215.57
230.30	261.20	268.29	312.54	387.13	436.34
524.05	544.30	588.95	614.05	634.75	671.26
769.32	794.12	831.93	849.28	909.55	957.78
1063.89	1088.59	1101.13	1115.93	1155.38	1200.38
1244.08	1259.78	1305.23	1329.80	1364.87	1371.04
1424.91	1430.95	1457.34	1482.54	1624.49	1645.03
1815.96	1874.20	3026.11	3044.63	3066.21	3071.75
3100.80	3550.73	3642.99	3644.91	3778.05	3847.54

- Conformer (145); ZPE = 36433.450 cm^{-1}:

62.26	65.38	103.62	125.18	173.95	265.72
278.08	308.76	310.11	382.36	441.62	460.64
528.71	542.80	601.01	651.20	680.45	713.19
768.46	794.88	835.45	870.91	904.24	999.98
1033.89	1085.32	1107.21	1156.17	1174.47	1194.30
1222.77	1260.66	1291.92	1333.90	1352.54	1362.60
1407.13	1445.17	1464.59	1478.95	1620.07	1637.88
1827.05	1894.24	3004.84	3050.65	3054.23	3102.60
3111.05	3540.58	3601.39	3635.27	3728.22	3818.29

- Conformer (146); ZPE = 36323.370 cm^{-1}:

47.33	62.81	96.17	116.98	184.24	257.71
290.43	314.10	361.20	412.85	420.65	460.26
521.95	553.86	564.36	572.62	637.86	660.53
746.67	783.97	815.57	882.67	914.79	954.31
996.19	1062.60	1104.14	1158.87	1178.93	1193.04
1215.94	1263.09	1325.84	1341.37	1363.61	1379.22
1396.72	1448.87	1458.97	1472.47	1622.36	1646.02
1829.31	1869.01	2930.00	3044.81	3048.02	3107.04
3122.95	3562.14	3620.36	3657.06	3746.56	3847.32

- Conformer (147); ZPE = 36300.799 cm^{-1}:

44.18	51.95	64.10	126.96	184.18	253.73
298.78	320.08	321.85	339.88	386.60	461.09
475.74	513.73	528.97	594.03	638.45	675.26
746.05	806.87	849.42	911.72	943.54	992.56
1013.83	1074.83	1100.45	1160.76	1162.34	1206.06
1237.69	1286.86	1301.98	1334.08	1347.35	1389.23
1404.67	1434.08	1449.76	1467.46	1627.49	1670.60
1806.78	1899.26	2978.40	3042.44	3043.87	3090.12
3094.42	3531.04	3616.52	3638.21	3770.63	3890.64

- Conformer (148); ZPE = 36356.330 cm^{-1}:

36.19	68.63	77.78	139.18	165.99	215.96
283.08	298.56	308.93	334.57	399.78	468.46
516.02	540.38	556.90	585.41	627.13	747.64
764.01	796.26	832.96	890.61	922.74	984.64
1040.24	1067.91	1111.50	1127.42	1170.33	1218.81
1237.53	1253.77	1318.03	1337.95	1351.26	1397.34
1424.88	1439.50	1477.81	1486.21	1624.92	1645.63
1800.18	1868.97	2990.30	3051.55	3056.79	3099.89
3114.11	3541.84	3629.08	3641.77	3779.07	3846.28

- Conformer (149); ZPE = 36247.047 cm^{-1}:

29.06	43.74	67.78	93.13	173.52	226.42
275.77	318.86	356.17	382.11	402.00	449.67
483.77	525.80	572.89	600.29	632.25	708.84
744.81	798.06	823.69	868.80	929.95	959.29
1036.68	1062.94	1108.20	1155.31	1177.41	1183.80
1236.71	1262.76	1306.55	1318.54	1350.61	1380.21
1410.32	1444.68	1464.48	1471.09	1620.31	1665.86
1828.84	1872.81	2906.94	3032.85	3067.78	3093.98
3124.84	3557.98	3626.94	3644.60	3762.32	3851.09

- Conformer (150); ZPE = 36364.890 cm^{-1}:

51.84	60.48	107.01	136.48	160.94	167.34
245.13	270.11	348.89	425.40	444.14	447.61
511.56	522.68	547.57	624.66	664.34	747.59
765.79	789.26	841.45	880.14	908.47	950.30
1034.00	1052.42	1115.11	1160.41	1173.53	1187.05
1213.09	1290.62	1291.23	1330.73	1360.46	1375.82
1417.43	1423.01	1469.71	1482.39	1633.89	1679.07
1811.38	1877.32	3013.74	3045.21	3050.08	3103.28
3116.62	3551.40	3602.28	3629.01	3736.70	3883.59

- Conformer (151); ZPE = 36193.757 cm^{-1}:

24.37	52.48	60.37	116.96	158.13	212.18
246.81	285.19	314.62	367.62	425.20	483.64
508.01	513.87	530.50	601.55	631.93	650.09
767.20	790.31	841.83	860.54	920.77	960.04
1029.11	1085.84	1114.78	1138.06	1184.51	1200.38
1217.43	1258.10	1308.45	1320.71	1333.12	1374.81
1428.31	1436.91	1461.67	1489.09	1625.59	1634.43
1808.31	1892.81	2957.78	3025.69	3040.50	3083.07
3096.23	3558.49	3642.47	3655.15	3777.53	3883.96

- Conformer (152); ZPE = 36247.903 cm^{-1}:

30.56	48.03	84.06	131.70	156.03	244.80
256.93	270.03	300.09	325.78	361.87	492.46
503.68	533.36	576.00	606.72	617.39	659.94
762.76	788.46	829.84	873.88	910.49	989.13
1023.79	1075.11	1090.36	1141.39	1165.17	1181.07
1248.78	1276.94	1330.71	1340.43	1367.47	1381.60
1388.47	1399.42	1449.84	1494.29	1627.83	1664.89
1811.96	1875.42	3026.36	3059.13	3077.08	3096.09
3107.52	3546.59	3632.46	3642.12	3775.11	3844.42

- Conformer (153); ZPE = 36546.466 cm^{-1}:

58.23	72.22	102.01	122.53	187.69	234.75
257.17	296.70	354.68	414.71	443.15	517.04
524.90	552.94	620.21	662.62	714.93	724.98
761.45	802.37	863.66	896.33	941.60	993.63
1004.58	1066.14	1113.50	1176.25	1180.16	1203.64
1213.68	1301.92	1308.79	1323.23	1370.03	1378.48
1396.30	1429.75	1464.03	1484.10	1622.82	1673.60
1844.30	1894.82	3012.06	3047.54	3051.36	3101.94
3123.65	3537.85	3583.20	3620.34	3699.90	3744.48

- Conformer (154); ZPE = 36399.424 cm^{-1}:

27.44	59.22	83.16	113.91	167.15	195.52
239.62	306.15	361.94	425.59	443.03	466.31
502.94	520.86	561.76	632.12	670.59	736.76
763.81	815.88	848.36	907.26	952.38	972.93
1034.54	1060.51	1137.23	1159.16	1176.30	1205.79
1226.46	1262.35	1284.26	1330.70	1339.05	1385.27
1394.26	1436.07	1469.33	1482.16	1633.66	1667.85
1807.32	1890.71	3035.09	3037.62	3048.80	3095.29
3136.87	3518.26	3540.91	3615.96	3724.84	3887.52

- Conformer (155); ZPE = 36427.701 cm^{-1}:

46.55	56.61	69.75	122.15	163.87	228.33
255.16	276.02	321.07	401.56	427.06	479.91
500.32	514.79	558.44	668.91	677.02	745.40
761.31	810.22	868.15	922.52	957.53	999.39
1030.20	1064.36	1141.17	1165.47	1166.99	1194.69
1219.20	1266.72	1320.58	1337.50	1344.45	1375.71
1398.67	1429.25	1466.52	1480.17	1630.97	1668.51
1812.09	1894.70	3022.50	3037.63	3047.91	3082.77
3135.64	3512.08	3557.12	3603.84	3730.25	3885.70

- Conformer (156); ZPE = 36392.319 cm^{-1}:

44.00	46.77	70.21	148.10	213.29	263.34
282.41	291.95	331.68	349.50	372.64	500.20
518.68	534.18	585.97	596.57	631.94	679.66
775.05	800.68	815.82	845.64	935.68	942.52
991.88	1074.03	1094.32	1162.85	1173.85	1184.92
1243.14	1287.97	1315.75	1340.02	1362.59	1386.76
1388.82	1413.49	1441.65	1507.08	1632.18	1658.42
1808.93	1877.97	3052.06	3064.57	3085.38	3101.75
3122.68	3555.68	3628.72	3647.11	3756.79	3846.83

- Conformer (157); ZPE = 36126.257 cm^{-1}:

22.48	43.89	66.52	93.69	120.76	183.39
233.80	260.68	268.41	312.26	395.75	440.91
498.20	510.41	530.20	602.05	662.10	677.48
740.10	799.14	861.54	895.84	949.89	985.62
1023.37	1086.53	1106.22	1142.75	1167.36	1198.47
1243.67	1274.84	1291.70	1327.51	1345.90	1380.33
1411.50	1431.80	1460.02	1480.25	1624.67	1671.08
1808.30	1894.25	3006.61	3031.40	3042.65	3086.87
3110.39	3532.93	3611.13	3642.04	3779.36	3883.47

- Conformer (158); ZPE = 36259.895 cm^{-1}:

32.92	41.90	58.13	97.10	168.63	195.61
230.76	282.21	291.18	324.72	387.35	433.36
507.35	562.85	579.88	605.43	652.85	731.14
764.62	782.94	812.23	876.22	913.06	1009.84
1046.39	1061.82	1108.12	1162.48	1173.02	1187.14
1240.57	1257.24	1305.54	1334.45	1339.66	1376.53
1396.78	1446.03	1485.74	1492.26	1620.10	1643.19
1821.52	1874.36	3001.68	3035.54	3077.01	3094.30
3138.27	3563.31	3632.48	3660.01	3767.54	3834.43

- Conformer (159); ZPE = 36116.925 cm^{-1}:

20.57	47.68	72.02	86.64	167.21	193.26
229.38	269.98	298.21	344.20	426.60	445.69
489.68	513.42	539.51	619.20	652.38	658.78
764.77	794.23	800.94	856.54	920.41	954.38
1025.74	1089.59	1106.44	1149.00	1172.47	1208.01
1240.12	1248.06	1304.12	1315.76	1325.91	1376.70
1425.85	1440.73	1457.28	1483.58	1624.49	1638.73
1811.61	1894.81	2986.32	3030.44	3041.83	3074.09
3098.19	3551.22	3641.19	3647.46	3775.46	3882.98

- Conformer (160); ZPE = 36192.179 cm^{-1}:

32.16	47.73	89.34	131.27	158.60	232.26
257.49	262.71	297.01	326.99	356.24	486.17
506.76	531.15	567.04	600.81	613.15	654.52
764.61	788.10	832.47	836.85	906.40	976.13
1068.41	1073.08	1098.20	1106.29	1165.00	1172.61
1250.89	1258.81	1330.60	1347.08	1360.38	1366.70
1390.25	1432.72	1449.70	1492.09	1629.85	1648.20
1812.22	1887.35	3029.66	3057.32	3069.75	3083.23
3104.70	3546.43	3636.94	3641.17	3773.09	3845.69

- Conformer (161); ZPE = 36286.378 cm^{-1}:

43.49	72.85	80.82	117.81	165.56	216.41
269.69	282.81	301.79	324.43	421.57	456.83
492.20	542.45	586.14	593.15	625.31	746.20
770.63	791.00	825.43	894.76	910.12	988.10
1033.76	1062.73	1107.33	1135.13	1164.22	1190.66
1241.30	1246.57	1321.87	1325.30	1333.53	1389.37
1404.88	1454.66	1467.81	1481.80	1620.45	1653.74
1829.35	1892.40	2942.19	3035.61	3051.03	3107.89
3121.69	3550.86	3633.29	3639.82	3768.69	3845.29

- Conformer (162); ZPE = 36298.108 cm^{-1}:

39.03	60.59	89.76	122.54	169.37	217.99
255.34	315.14	357.75	359.58	422.68	435.99
502.10	522.75	548.19	561.29	648.47	726.79
750.01	812.32	853.07	868.98	936.47	960.82
1033.32	1057.27	1119.23	1153.86	1168.23	1190.77
1225.53	1254.02	1295.20	1329.96	1346.82	1368.46
1418.51	1425.56	1469.45	1481.73	1628.39	1629.16
1799.69	1895.78	2982.25	3035.47	3054.50	3087.94
3112.32	3551.99	3633.01	3652.33	3763.74	3894.71

- Conformer (163); ZPE = 36480.871 cm^{-1}:

49.20	69.28	98.08	154.44	193.65	240.21
255.13	296.05	334.51	340.53	482.00	508.45
539.38	570.30	587.20	656.31	672.93	687.98
766.82	795.99	825.39	877.01	932.33	955.91
1060.43	1088.74	1106.19	1160.87	1182.74	1190.67
1229.72	1299.57	1315.24	1338.26	1357.11	1378.49
1387.95	1427.60	1458.61	1480.89	1621.90	1671.64
1837.40	1890.56	3040.74	3051.30	3056.68	3086.41
3099.88	3531.95	3593.77	3616.89	3712.97	3797.49

- Conformer (164); ZPE = 36179.098 cm^{-1}:

25.59	43.63	83.69	101.09	141.14	190.60
232.67	292.07	293.21	329.70	338.32	439.67
495.54	515.24	554.22	608.00	659.30	685.16
752.73	809.19	842.28	891.55	910.59	1020.61
1042.13	1079.42	1110.21	1143.32	1163.81	1182.17
1230.51	1280.78	1296.86	1315.34	1359.65	1367.00
1414.59	1433.82	1461.02	1482.34	1624.67	1668.71
1817.98	1893.93	3008.31	3012.23	3047.51	3072.83
3124.91	3538.02	3621.47	3641.09	3776.89	3890.90

- Conformer (165); ZPE = 36314.072 cm^{-1}:

71.39	81.49	105.76	108.01	171.03	243.71
277.59	308.03	311.52	345.38	412.48	452.75
489.68	521.79	545.07	574.65	621.09	709.93
748.57	779.18	850.95	899.42	939.63	970.17
1013.55	1060.60	1112.30	1147.91	1159.75	1186.59
1233.68	1267.65	1296.13	1340.39	1356.97	1369.44
1407.94	1417.94	1468.84	1479.41	1627.42	1685.99
1812.54	1906.08	3009.70	3031.06	3066.11	3097.10
3136.87	3511.21	3609.23	3629.89	3760.38	3886.19

- Conformer (166); ZPE = 36185.282 cm^{-1}:

30.68	40.99	80.30	98.85	149.38	192.95
253.74	299.86	305.08	327.28	340.19	443.36
499.20	516.57	554.56	608.39	658.76	688.42
753.67	807.57	845.62	875.05	892.82	1014.29
1072.96	1085.34	1105.58	1158.21	1168.70	1183.86
1234.09	1260.58	1299.84	1322.87	1335.31	1361.84
1428.19	1446.26	1461.85	1482.47	1624.47	1643.65
1815.18	1897.76	2984.90	3013.35	3046.85	3075.63
3104.54	3538.70	3629.46	3640.77	3776.70	3893.09

- Conformer (167); ZPE = 36185.095 cm^{-1}:

33.98	56.17	63.32	105.12	181.77	273.32
279.34	299.66	323.73	343.57	418.03	453.48
487.56	499.56	521.06	599.52	620.66	650.75
728.76	789.99	832.06	888.71	921.85	970.84
1054.07	1068.16	1100.07	1161.78	1177.91	1217.92
1230.01	1261.54	1296.84	1315.93	1329.09	1392.88
1419.39	1431.26	1454.39	1485.20	1622.86	1624.08
1806.29	1899.44	2910.29	3014.12	3050.87	3076.29
3119.83	3553.95	3638.92	3648.94	3773.22	3891.83

- Conformer (168); ZPE = 36488.169 cm^{-1}:

28.18	82.72	90.30	120.98	181.96	200.17
241.29	272.09	326.11	392.13	436.77	513.03
523.71	569.51	634.27	666.19	712.05	746.83
783.01	808.03	864.92	898.37	945.23	989.19
1001.78	1070.93	1105.10	1169.66	1182.83	1206.52
1211.16	1283.21	1309.93	1323.83	1375.09	1383.47
1387.01	1417.80	1477.62	1480.12	1622.32	1672.60
1844.67	1892.77	3029.49	3044.00	3054.43	3108.39
3134.63	3530.81	3582.64	3607.71	3702.04	3736.71

- Conformer (169); ZPE = 36438.698 cm^{-1}:

61.60	65.94	104.32	122.01	186.34	238.14
267.73	308.02	352.33	404.31	438.18	501.50
526.21	550.66	612.81	665.27	705.96	713.21
758.01	798.00	807.92	875.59	914.25	967.25
1018.48	1073.02	1095.04	1162.69	1181.76	1205.52
1224.75	1276.56	1311.04	1337.83	1367.31	1378.94
1386.16	1440.29	1463.76	1488.01	1622.99	1631.97
1843.93	1901.33	2989.52	3038.72	3048.87	3095.78
3112.61	3544.57	3584.66	3645.75	3702.02	3757.98

- Conformer (170); ZPE = 36270.793 cm^{-1}:

38.50	50.73	65.99	109.04	170.64	173.47
212.53	282.58	338.30	368.06	427.40	462.66
496.97	522.98	559.73	602.10	633.33	738.99
771.50	802.40	858.25	911.35	929.47	947.18
1019.53	1047.85	1113.41	1153.76	1161.05	1187.83
1228.51	1284.78	1285.97	1301.06	1364.53	1374.34
1394.43	1408.26	1485.11	1493.40	1630.11	1672.21
1823.19	1895.03	3021.03	3026.03	3044.20	3115.53
3134.32	3537.08	3613.16	3621.66	3750.92	3879.14

- Conformer (171); ZPE = 36317.092 cm^{-1}:

51.23	80.33	91.79	109.05	166.44	182.16
250.57	279.71	319.72	394.83	433.49	444.06
506.79	523.63	566.93	610.03	646.27	737.00
753.38	786.01	817.66	884.12	914.34	1013.62
1049.84	1058.35	1111.26	1140.24	1173.74	1213.49
1224.05	1276.78	1296.45	1311.56	1339.72	1390.22
1399.75	1443.36	1472.71	1483.95	1630.93	1656.69
1812.82	1889.74	2920.37	3047.79	3058.57	3103.86
3130.85	3555.63	3600.25	3644.79	3745.07	3888.18

- Conformer (172); ZPE = 36268.414 cm^{-1}:

30.55	41.87	62.22	130.37	166.19	240.39
256.20	280.05	331.42	347.23	422.81	437.41
509.23	521.66	551.00	556.57	647.13	713.30
755.98	802.40	843.83	896.92	927.99	961.12
1030.89	1049.69	1102.74	1157.77	1163.10	1200.45
1208.62	1282.42	1294.29	1341.01	1356.66	1381.52
1407.83	1418.19	1468.96	1473.96	1624.97	1688.32
1812.44	1902.47	3012.49	3042.02	3051.02	3088.64
3106.67	3519.13	3612.02	3640.94	3774.88	3888.86

- Conformer (173); ZPE = 36183.519 cm^{-1}:

25.83	30.60	52.48	120.33	160.75	220.22
264.22	289.46	325.06	351.36	407.69	430.23
492.26	514.61	546.52	577.95	679.69	707.35
754.34	807.01	816.58	863.79	916.78	958.13
1036.41	1056.41	1107.39	1148.85	1160.95	1176.74
1223.30	1266.49	1307.13	1338.09	1342.52	1367.10
1407.95	1440.05	1470.27	1473.12	1624.58	1634.95
1801.30	1901.63	2994.83	3020.95	3051.49	3070.65
3113.76	3552.94	3638.49	3650.71	3771.94	3902.80

- Conformer (174); ZPE = 36353.700 cm^{-1}:

42.09	66.86	91.38	131.15	178.34	239.71
249.33	285.94	314.85	381.47	420.40	465.28
503.91	547.48	555.03	593.76	657.63	745.04
757.83	788.87	842.77	876.80	918.64	996.51
1034.25	1065.86	1106.74	1140.67	1150.67	1179.80
1222.46	1271.02	1299.85	1318.73	1350.71	1364.72
1417.24	1418.48	1473.15	1480.57	1636.23	1664.70
1814.82	1903.95	3003.26	3049.43	3056.19	3109.07
3117.18	3536.07	3618.77	3621.31	3741.90	3888.52

- Conformer (175); ZPE = 36148.051 cm^{-1}:

30.86	44.30	54.66	93.43	169.25	190.18
250.24	305.76	333.10	345.91	384.52	459.35
471.76	521.72	526.19	613.46	669.04	681.57
750.71	806.32	835.04	857.62	918.93	958.16
1045.25	1070.52	1108.58	1147.52	1157.99	1187.26
1225.14	1266.17	1301.46	1316.47	1343.78	1369.39
1421.81	1436.46	1458.47	1467.75	1624.34	1632.91
1819.60	1908.48	2982.06	3033.29	3037.71	3085.22
3091.41	3546.13	3632.85	3640.56	3768.00	3897.42

- Conformer (176); ZPE = 36426.704 cm^{-1}:

45.05	67.09	96.56	155.99	192.42	245.98
254.24	305.10	332.13	333.54	489.19	506.10
537.87	559.42	579.79	646.94	665.86	684.56
767.68	794.66	817.07	864.36	894.61	985.97
1031.55	1090.21	1117.44	1160.60	1183.76	1192.00
1246.13	1274.38	1319.93	1344.19	1351.81	1366.62
1383.58	1450.10	1458.04	1484.27	1622.33	1639.41
1835.80	1894.89	3021.57	3035.89	3056.45	3080.87
3098.79	3536.28	3597.57	3631.89	3718.50	3806.37

- Conformer (177); ZPE = 36159.194 cm^{-1}:

52.33	77.46	79.32	113.78	175.62	227.74
248.89	269.94	322.23	330.91	341.73	459.61
475.96	504.92	542.09	605.89	654.23	670.86
741.85	785.08	833.80	860.93	907.28	988.80
1060.17	1075.29	1106.27	1163.79	1167.00	1200.06
1240.39	1242.72	1299.14	1312.02	1337.50	1367.76
1422.28	1441.45	1461.19	1466.57	1623.21	1646.44
1825.43	1906.82	2958.17	3017.98	3045.24	3074.21
3101.43	3541.62	3636.19	3638.84	3774.56	3893.40

- Conformer (178); ZPE = 36271.345 cm^{-1}:

41.99	55.73	69.26	126.98	167.61	262.34
294.28	325.72	341.66	378.07	413.34	445.70
498.85	514.84	554.82	569.27	664.85	680.75
756.01	807.42	837.96	871.42	920.10	957.18
999.73	1063.04	1100.84	1155.06	1167.86	1186.17
1236.15	1259.03	1306.79	1317.96	1342.29	1380.27
1395.11	1445.79	1464.82	1475.72	1620.86	1642.40
1823.38	1903.88	2986.33	3029.31	3046.97	3072.59
3112.63	3539.41	3626.39	3633.83	3756.15	3895.77

- Conformer (179); ZPE = 36109.106 cm^{-1}:

32.47	47.13	61.78	120.09	147.86	184.93
208.29	286.21	287.88	293.38	366.81	470.25
504.46	514.09	547.29	609.34	614.14	690.52
753.48	789.46	804.63	884.14	896.52	1010.43
1068.06	1080.23	1099.28	1137.30	1160.30	1199.38
1238.83	1262.89	1294.75	1308.11	1332.40	1385.06
1429.31	1443.31	1465.63	1478.95	1624.57	1653.49
1810.33	1904.96	2912.35	3029.81	3054.78	3097.73
3114.20	3553.21	3642.09	3642.67	3778.65	3890.04

- Conformer (180); ZPE = 36251.574 cm^{-1}:

64.26	75.09	95.63	132.49	175.01	248.03
267.75	308.29	336.68	353.94	410.20	458.73
494.89	524.71	541.25	575.23	628.35	697.62
748.30	777.55	839.20	855.28	923.08	967.32
990.57	1068.76	1104.63	1155.06	1157.53	1202.95
1218.71	1255.88	1304.36	1313.56	1346.26	1376.42
1407.62	1438.05	1467.79	1483.60	1627.93	1643.87
1818.06	1912.75	2980.08	3021.67	3061.07	3075.58
3131.05	3533.65	3626.22	3636.91	3753.53	3890.11

- Conformer (181); ZPE = 36254.975 cm^{-1}:

29.01	59.31	90.58	97.32	196.28	217.14
250.46	267.02	326.71	379.15	422.29	453.90
498.37	512.86	551.42	582.88	677.26	701.61
759.45	791.68	831.94	896.61	931.59	946.41
1010.30	1079.25	1110.27	1132.18	1166.10	1200.03
1228.03	1252.90	1300.38	1304.71	1354.99	1380.55
1429.70	1449.10	1467.42	1481.74	1630.00	1651.37
1814.23	1905.15	2933.23	3045.77	3057.84	3095.93
3107.15	3548.65	3623.83	3638.84	3748.28	3890.75

10.5 Cartesian coordinates of Gln conformers

Each line starting by an asterisk contains the relative energy of the corresponding conformer and the dihedral angle for each of the target torsions (separated by underscores). Cartesian coordinates are given in Å.

20

∗ E = +0.000 kcal/mol ; (1) 005_340_149_060_061_253

H	+0.00000	+0.00000	+0.00000
O	+0.00000	+0.00000	+0.97346
C	+1.26748	+0.00000	+1.35562
C	+2.28116	+0.12132	+0.21306
N	+1.64304	−0.25087	−1.04666
H	+2.04520	+0.25376	−1.82620
O	+1.59189	−0.05130	+2.51171
C	+2.77041	+1.57554	+0.12255
H	+3.13196	−0.52094	+0.47638
H	+1.72906	−1.24057	−1.24052
C	+3.45543	+2.08308	+1.39210
H	+3.49008	+1.62898	−0.70329
H	+1.92026	+2.21924	−0.13385
C	+4.68354	+1.23412	+1.68818
H	+3.80585	+3.10379	+1.22178
H	+2.76316	+2.09019	+2.23732
N	+4.55779	+0.38140	+2.73042
O	+5.68511	+1.31857	+1.00728
H	+5.31586	−0.25413	+2.91458
H	+3.64358	+0.18951	+3.11322

20

∗ E = +0.327 kcal/mol ; (2) 006_344_139_308_293_134

H	+0.00000	+0.00000	+0.00000
O	+0.00000	+0.00000	+0.97625
C	+1.28085	+0.00000	+1.34287
C	+2.26614	+0.15446	+0.17470
N	+1.58502	−0.10310	−1.09211
H	+1.86032	+0.54871	−1.81849
O	+1.63290	−0.03605	+2.48517
C	+2.82087	+1.58607	+0.25057
H	+3.09028	−0.54291	+0.36190
H	+1.73377	−1.04727	−1.42029
C	+1.73477	+2.66146	+0.32434
H	+3.44581	+1.65434	+1.14426
H	+3.45284	+1.76890	−0.62486
C	+0.92180	+2.73697	−0.95687
H	+1.09140	+2.50520	+1.19783
H	+2.20880	+3.64094	+0.44822
N	−0.41874	+2.80016	−0.79989

O	+1.45640	+2.74599	−2.04889
H	−1.00095	+2.86247	−1.61789
H	−0.84646	+2.71654	+0.10478

20
* E = +0.612 kcal/mol ; (3) 000_171_295_139_290_180

H	+0.00000	+0.00000	+0.00000
O	+0.00000	+0.00000	+0.97403
C	+1.24544	+0.00000	+1.44756
C	+2.33237	−0.00299	+0.36906
N	+3.67385	+0.19511	+0.87495
H	+3.76391	+1.12220	+1.27418
O	+1.48443	−0.00526	+2.62023
C	+2.26201	−1.31527	−0.44157
H	+2.09524	+0.83339	−0.30219
H	+3.83382	−0.44837	+1.64455
C	+2.46381	−1.06170	−1.92609
H	+3.04234	−1.98363	−0.06689
H	+1.30145	−1.81878	−0.29366
C	+1.26383	−0.33731	−2.51379
H	+3.36347	−0.45353	−2.08724
H	+2.60785	−1.99556	−2.48149
N	+1.32917	−0.06487	−3.83165
O	+0.29503	−0.01895	−1.84272
H	+0.55891	+0.41079	−4.27018
H	+2.12322	−0.32857	−4.38561

20
* E = +0.701 kcal/mol ; (4) 351_026_074_282_047_097

H	+0.00000	+0.00000	+0.00000
O	+0.00000	+0.00000	+0.97117
C	+1.26536	+0.00000	+1.36156
C	+2.27977	−0.22541	+0.22709
N	+1.69781	+0.21987	−1.03047
H	+1.68006	+1.23491	−1.07755
O	+1.59180	+0.10388	+2.51069
C	+3.65865	+0.32631	+0.61093
H	+2.36877	−1.31970	+0.17325
H	+2.17536	−0.15957	−1.83554
C	+3.79598	+1.85211	+0.47676
H	+3.85660	+0.02140	+1.64319
H	+4.42014	−0.14240	−0.02089

C +2.62305 +2.57550 +1.10870
H +4.74575 +2.17430 +0.91365
H +3.80290 +2.13726 −0.57938
N +2.79265 +3.03157 +2.36816
O +1.57895 +2.71368 +0.49611
H +1.96363 +3.31502 +2.86565
H +3.57169 +2.71143 +2.91560
20
* E = +0.814 kcal/mol ; (5) 002_156_288_056_059_106
H +0.00000 +0.00000 +0.00000
O +0.00000 +0.00000 +0.97757
C +1.23675 +0.00000 +1.46345
C +2.37709 +0.04572 +0.43364
N +3.59851 +0.59769 +0.98731
H +3.50158 +1.59630 +1.12635
O +1.45467 −0.06071 +2.64080
C +2.68390 −1.37974 −0.05697
H +2.06243 +0.66122 −0.41652
H +3.74122 +0.19955 +1.91149
C +1.50601 −2.16132 −0.65915
H +3.49902 −1.29894 −0.78323
H +3.07489 −1.94672 +0.79606
C +0.91280 −1.43580 −1.84712
H +1.84410 −3.16133 −0.94570
H +0.70861 −2.28370 +0.08139
N +1.17481 −1.94693 −3.06384
O +0.25162 −0.41494 −1.71253
H +0.81760 −1.47290 −3.87669
H +1.70923 −2.78754 −3.18118
20
* E = +1.570 kcal/mol ; (6) 181_178_315_185_301_090
H +0.00000 +0.00000 +0.00000
O +0.00000 +0.00000 +0.96402
C +1.27091 +0.00000 +1.38465
C +1.37219 −0.01612 +2.90200
N +2.74778 −0.06820 +3.36455
H +3.26715 −0.75227 +2.82432
O +2.20797 +0.00329 +0.63743
C +0.63496 +1.19577 +3.48948
H +0.85487 −0.92692 +3.23705

H	+3.20212	+0.82203	+3.18356
C	+0.80164	+1.31769	+5.00832
H	+1.01675	+2.10944	+3.01516
H	−0.42537	+1.11984	+3.23433
C	+0.29698	+0.07570	+5.72694
H	+1.85165	+1.49468	+5.25881
H	+0.20992	+2.16111	+5.36903
N	+1.23591	−0.88573	+5.91112
O	−0.85425	−0.03892	+6.08844
H	+0.94823	−1.76308	+6.31006
H	+2.12474	−0.80947	+5.43753

20

∗ E = +1.655 kcal/mol ; (7) 356_020_086_181_309_270

H	+0.00000	+0.00000	+0.00000
O	+0.00000	+0.00000	+0.97253
C	+1.27270	+0.00000	+1.35550
C	+2.27386	−0.10545	+0.20005
N	+1.64621	+0.29696	−1.05304
H	+1.70543	+1.30033	−1.19064
O	+1.60740	+0.04780	+2.50346
C	+3.57108	+0.62070	+0.56219
H	+2.48493	−1.17989	+0.10744
H	+2.11375	−0.15684	−1.83135
C	+4.60704	+0.57145	−0.57076
H	+3.34743	+1.66810	+0.79835
H	+3.96955	+0.17806	+1.47995
C	+4.79977	−0.84998	−1.06205
H	+4.26012	+1.15402	−1.42837
H	+5.55214	+1.00548	−0.23038
N	+5.76753	−1.56382	−0.44309
O	+4.08702	−1.33097	−1.92242
H	+5.89375	−2.53003	−0.69313
H	+6.34861	−1.16285	+0.26961

20

∗ E = +1.733 kcal/mol ; (8) 181_032_291_280_054_100

H	+0.00000	+0.00000	+0.00000
O	+0.00000	+0.00000	+0.96338
C	+1.27270	+0.00000	+1.37181
C	+1.39451	−0.01366	+2.89713
N	+0.33739	−0.72518	+3.59160

H	−0.53985	−0.22841	+3.50476
O	+2.19935	+0.06157	+0.60942
C	+2.78086	−0.51462	+3.31348
H	+1.33186	+1.04611	+3.18242
H	+0.20793	−1.63354	+3.15326
C	+2.91874	−2.04494	+3.27114
H	+3.53120	−0.04807	+2.66820
H	+2.96464	−0.18882	+4.34068
C	+2.52818	−2.60101	+1.91788
H	+3.94199	−2.32929	+3.53431
H	+2.24246	−2.48694	+4.00807
N	+3.53838	−2.89682	+1.07068
O	+1.35813	−2.74998	+1.61190
H	+3.30321	−3.10203	+0.11390
H	+4.48036	−2.62099	+1.27968

20

∗ E = +1.912 kcal/mol ; (9) 359_359_122_166_085_180

H	+0.00000	+0.00000	+0.00000
O	+0.00000	+0.00000	+0.97573
C	+1.27366	+0.00000	+1.33965
C	+2.27283	−0.01461	+0.16680
N	+1.56551	−0.05112	−1.11006
H	+1.81203	+0.73912	−1.69754
O	+1.63365	+0.00735	+2.48197
C	+3.20875	+1.18755	+0.31901
H	+2.86326	−0.92964	+0.31179
H	+1.72844	−0.90351	−1.62538
C	+4.45738	+1.12089	−0.55481
H	+2.65309	+2.10464	+0.08811
H	+3.49939	+1.23729	+1.37115
C	+4.25660	+1.63269	−1.97365
H	+5.25801	+1.72371	−0.10963
H	+4.84445	+0.09459	−0.60558
N	+5.35738	+1.57359	−2.75865
O	+3.20006	+2.07288	−2.38107
H	+5.30241	+1.93030	−3.69720
H	+6.24004	+1.24807	−2.41010

20

∗ E = +2.009 kcal/mol ; (10) 355_022_088_075_066_190

H	+0.00000	+0.00000	+0.00000

O	+0.00000	+0.00000	+0.97220
C	+1.27225	+0.00000	+1.35592
C	+2.27903	−0.12761	+0.20616
N	+1.64153	+0.28814	−1.04246
H	+1.73826	+1.28825	−1.18271
O	+1.59576	+0.06567	+2.50803
C	+3.59582	+0.58926	+0.52561
H	+2.48360	−1.20200	+0.11302
H	+2.07498	−0.17097	−1.83314
C	+4.47353	−0.16342	+1.51676
H	+4.14566	+0.69736	−0.41472
H	+3.37990	+1.59124	+0.91336
C	+4.97674	−1.47085	+0.92635
H	+5.35187	+0.43860	+1.77793
H	+3.92863	−0.35553	+2.44635
N	+5.58211	−2.30272	+1.80762
O	+4.86068	−1.74588	−0.25092
H	+5.93533	−3.18449	+1.47860
H	+5.61726	−2.09481	+2.78839

20

∗ E = +2.065 kcal/mol ; (11) 181_201_194_283_061_274

H	+0.00000	+0.00000	+0.00000
O	+0.00000	+0.00000	+0.96394
C	+1.26217	+0.00000	+1.38964
C	+1.37269	−0.03194	+2.90689
N	+2.69501	+0.46855	+3.25436
H	+2.92131	+0.26054	+4.21862
O	+2.20971	−0.04103	+0.65402
C	+0.25927	+0.71652	+3.65327
H	+1.24572	−1.10463	+3.14244
H	+3.39404	+0.04087	+2.65700
C	+0.39488	+2.24400	+3.67102
H	−0.70499	+0.43311	+3.22241
H	+0.25810	+0.36284	+4.69060
C	+0.36391	+2.84389	+2.27504
H	−0.44557	+2.66972	+4.22214
H	+1.32428	+2.52733	+4.17414
N	+1.57290	+2.87191	+1.66218
O	−0.65640	+3.24151	+1.75554
H	+1.62276	+3.16578	+0.70152

H +2.36756 +2.41838 +2.08988
20
∗ E = +2.226 kcal/mol ; (12) 181_180_313_183_080_207
H +0.00000 +0.00000 +0.00000
O +0.00000 +0.00000 +0.96383
C +1.27791 +0.00000 +1.37818
C +1.38759 −0.02025 +2.89672
N +2.75241 −0.03187 +3.37043
H +3.29914 −0.72006 +2.86684
O +2.20437 −0.00442 +0.61967
C +0.61212 +1.15556 +3.50333
H +0.89541 −0.95126 +3.21854
H +3.18564 +0.87269 +3.20886
C +0.77209 +1.20279 +5.01789
H +0.99015 +2.09742 +3.08742
H −0.44077 +1.06844 +3.22663
C +2.09718 +1.82855 +5.42809
H −0.01415 +1.82650 +5.45907
H +0.67486 +0.20122 +5.45310
N +2.58460 +1.42417 +6.62654
O +2.64133 +2.68272 +4.76033
H +3.48344 +1.77530 +6.91021
H +2.23130 +0.59872 +7.07383
20
∗ E = +2.325 kcal/mol ; (13) 176_158_026_307_295_121
H +0.00000 +0.00000 +0.00000
O +0.00000 +0.00000 +0.96412
C +1.26791 +0.00000 +1.38386
C +1.37541 +0.08728 +2.88952
N +2.66862 −0.41746 +3.30359
H +3.34569 −0.31664 +2.55692
O +2.20220 +0.00603 +0.62627
C +1.09246 +1.55630 +3.28304
H +0.57159 −0.52993 +3.31055
H +3.01655 +0.07858 +4.11471
C +1.95370 +2.57195 +2.52982
H +0.03354 +1.77462 +3.11242
H +1.28335 +1.65644 +4.35733
C +3.42795 +2.42343 +2.87371
H +1.79180 +2.50545 +1.44821

H	+1.66817	+3.58160	+2.83992
N	+4.25394	+2.16848	+1.82901
O	+3.82440	+2.55202	+4.01324
H	+5.22593	+2.00152	+2.03071
H	+3.87903	+1.83035	+0.95838

20

* E = +2.363 kcal/mol ; (14) 354_025_085_297_141_297

H	+0.00000	+0.00000	+0.00000
O	+0.00000	+0.00000	+0.97223
C	+1.26526	+0.00000	+1.36030
C	+2.28361	−0.15664	+0.22002
N	+1.67848	+0.31929	−1.02204
H	+1.78363	+1.32432	−1.11683
O	+1.58633	+0.09514	+2.51328
C	+3.60665	+0.48741	+0.62206
H	+2.42554	−1.24078	+0.11657
H	+2.10397	−0.11023	−1.83253
C	+3.47389	+2.00636	+0.84685
H	+3.94960	+0.00242	+1.54050
H	+4.35711	+0.28146	−0.14684
C	+4.29010	+2.49440	+2.03741
H	+3.80336	+2.57914	−0.02154
H	+2.42471	+2.26805	+1.04609
N	+3.89334	+1.97446	+3.22209
O	+5.20867	+3.27567	+1.92043
H	+4.38830	+2.24231	+4.05466
H	+3.12007	+1.32734	+3.27890

20

* E = +2.546 kcal/mol ; (15) 180_050_176_287_281_157

H	+0.00000	+0.00000	+0.00000
O	+0.00000	+0.00000	+0.96358
C	+1.27692	+0.00000	+1.37956
C	+1.36694	+0.00879	+2.89707
N	+0.50380	−1.04752	+3.40819
H	+0.59293	−1.12231	+4.41536
O	+2.20108	+0.01674	+0.61736
C	+2.80808	−0.19152	+3.35543
H	+1.04381	+1.02185	+3.19873
H	−0.46448	−0.88612	+3.16404
C	+3.29980	−1.61956	+3.14942

H	+3.44539	+0.50668	+2.80781
H	+2.87666	+0.04759	+4.42135
C	+2.79295	−2.55940	+4.23141
H	+3.02259	−1.99214	+2.15730
H	+4.39458	−1.64544	+3.20214
N	+2.75209	−3.86836	+3.88514
O	+2.50326	−2.17652	+5.34737
H	+2.39716	−4.52833	+4.55543
H	+2.86051	−4.15660	+2.93062

20

* E = +2.565 kcal/mol ; (16) 001_153_036_058_057_106

H	+0.00000	+0.00000	+0.00000
O	+0.00000	+0.00000	+0.97718
C	+1.23327	+0.00000	+1.46637
C	+2.36644	+0.01757	+0.43807
N	+3.51605	+0.64845	+1.05344
H	+3.55154	+0.39618	+2.03573
O	+1.44981	−0.05927	+2.64356
C	+2.65137	−1.43288	−0.01334
H	+2.04280	+0.60812	−0.42731
H	+4.38084	+0.37197	+0.60763
C	+1.47120	−2.20355	−0.62879
H	+3.47908	−1.39766	−0.73288
H	+3.01000	−1.98662	+0.86177
C	+0.89643	−1.48421	−1.83043
H	+1.79792	−3.21151	−0.90117
H	+0.66527	−2.30617	+0.10528
N	+1.17459	−2.00668	−3.03967
O	+0.23624	−0.46171	−1.71359
H	+0.82117	−1.54360	−3.86054
H	+1.69152	−2.86002	−3.14228

20

* E = +2.618 kcal/mol ; (17) 183_191_187_289_281_149

H	+0.00000	+0.00000	+0.00000
O	+0.00000	+0.00000	+0.96368
C	+1.27845	+0.00000	+1.37234
C	+1.40409	−0.07933	+2.88307
N	+2.78136	+0.19530	+3.22643
H	+2.95737	+0.04651	+4.21178
O	+2.19923	+0.02124	+0.60601

C +0.47249 +0.91431 +3.58722
H +1.06402 −1.10071 +3.13566
H +3.42648 −0.34115 +2.66181
C +0.94411 +2.35079 +3.39138
H −0.54570 +0.78021 +3.21626
H +0.47166 +0.69843 +4.66046
C +2.09438 +2.68781 +4.32879
H +1.23464 +2.53488 +2.34923
H +0.13346 +3.04904 +3.62832
N +2.98318 +3.59892 +3.85851
O +2.16377 +2.23000 +5.45020
H +3.79380 +3.78678 +4.42405
H +3.04548 +3.77983 +2.87315
20
∗ E = +2.652 kcal/mol ; (18) 355_022_083_112_063_179
H +0.00000 +0.00000 +0.00000
O +0.00000 +0.00000 +0.97257
C +1.27324 +0.00000 +1.35530
C +2.27424 −0.12063 +0.20118
N +1.64626 +0.30312 −1.04627
H +1.68664 +1.31314 −1.13685
O +1.60599 +0.03975 +2.50462
C +3.62372 +0.53672 +0.54987
H +2.42631 −1.20483 +0.10454
H +2.17672 −0.08350 −1.81986
C +4.74851 −0.47866 +0.69309
H +3.87998 +1.23936 −0.24940
H +3.52904 +1.09506 +1.48476
C +5.00549 −1.21389 −0.61298
H +5.67946 +0.00524 +1.01045
H +4.50046 −1.21274 +1.47064
N +6.00481 −2.12587 −0.57806
O +4.35827 −1.00177 −1.61995
H +6.21761 −2.64653 −1.41143
H +6.53392 −2.29876 +0.25654
20
∗ E = +2.661 kcal/mol ; (19) 167_158_273_296_079_203
H +0.00000 +0.00000 +0.00000
O +0.00000 +0.00000 +0.96431
C +1.26186 +0.00000 +1.38679

C	+1.38580	+0.32675	+2.86578
N	+2.63613	−0.15609	+3.42856
H	+2.54271	−1.11698	+3.73165
O	+2.20813	−0.18489	+0.66851
C	+1.26628	+1.85544	+3.02689
H	+0.54149	−0.13118	+3.38994
H	+3.34777	−0.15516	+2.70393
C	+2.39636	+2.61783	+2.34240
H	+0.30871	+2.19527	+2.61981
H	+1.28629	+2.06094	+4.09997
C	+2.19026	+2.74455	+0.83867
H	+2.44225	+3.64071	+2.73404
H	+3.36775	+2.16032	+2.56416
N	+3.31509	+2.96377	+0.11598
O	+1.08853	+2.70502	+0.33208
H	+3.23374	+2.98681	−0.88625
H	+4.22306	+2.82349	+0.51881

20

* E = +2.662 kcal/mol ; (20) 183_327_300_057_064_261

H	+0.00000	+0.00000	+0.00000
O	+0.00000	+0.00000	+0.96432
C	+1.27056	+0.00000	+1.38135
C	+1.39876	−0.07543	+2.89667
N	+0.34274	+0.57699	+3.64076
H	+0.29011	+1.56634	+3.43467
O	+2.19572	+0.01310	+0.61014
C	+1.46098	−1.55761	+3.30319
H	+2.35616	+0.39799	+3.14246
H	−0.55829	+0.16873	+3.42049
C	+2.59732	−2.34991	+2.65039
H	+1.57210	−1.58187	+4.39127
H	+0.49723	−2.02349	+3.06133
C	+3.95053	−1.81550	+3.09793
H	+2.54349	−3.38849	+2.98359
H	+2.51432	−2.33640	+1.55888
N	+4.55852	−0.97545	+2.22602
O	+4.42576	−2.11516	+4.17236
H	+5.42158	−0.54343	+2.50954
H	+4.06761	−0.62691	+1.41671

20

∗ E = +2.712 kcal/mol ; (21) 178_170_287_179_293_167

H	+0.00000	+0.00000	+0.00000
O	+0.00000	+0.00000	+0.96382
C	+1.27267	+0.00000	+1.37987
C	+1.38887	+0.03838	+2.89564
N	+2.74668	−0.18514	+3.35100
H	+3.00402	−1.15805	+3.24173
O	+2.20369	−0.00412	+0.62296
C	+0.86025	+1.39426	+3.39368
H	+0.73019	−0.73886	+3.30019
H	+3.38929	+0.34255	+2.76803
C	+0.92049	+1.49934	+4.90948
H	+1.46481	+2.19524	+2.95008
H	−0.17062	+1.51841	+3.05101
C	−0.04753	+0.54198	+5.58600
H	+1.93764	+1.29639	+5.26100
H	+0.65751	+2.51381	+5.23159
N	+0.14194	+0.38792	+6.92091
O	−0.93690	−0.02525	+4.98883
H	−0.46897	−0.22825	+7.42832
H	+0.91645	+0.81249	+7.39587

20

∗ E = +2.841 kcal/mol ; (22) 182_191_213_188_303_256

H	+0.00000	+0.00000	+0.00000
O	+0.00000	+0.00000	+0.96367
C	+1.27963	+0.00000	+1.37046
C	+1.40512	−0.04943	+2.87720
N	+2.77612	+0.22028	+3.25540
H	+3.00644	−0.30774	+4.08889
O	+2.19852	+0.01229	+0.60149
C	+0.43870	+0.94940	+3.53556
H	+1.08467	−1.06774	+3.15694
H	+3.40471	−0.03635	+2.50374
C	+0.68176	+1.07608	+5.04513
H	+0.59144	+1.93581	+3.08367
H	−0.59409	+0.64856	+3.33242
C	+0.57291	−0.26445	+5.74254
H	+1.69884	+1.44377	+5.20629
H	−0.01715	+1.80170	+5.47233
N	−0.56383	−0.48181	+6.44729

O +1.44271 −1.10869 +5.64703
H −0.68408 −1.36707 +6.90937
H −1.27004 +0.22291 +6.54583
20
∗ E = +2.995 kcal/mol ; (23) 001_342_148_047_277_153
H +0.00000 +0.00000 +0.00000
O +0.00000 +0.00000 +0.97168
C +1.26381 +0.00000 +1.36098
C +2.29790 +0.03406 +0.22846
N +1.67432 −0.36673 −1.02851
H +2.08772 +0.10935 −1.81961
O +1.59191 −0.01942 +2.51571
C +2.88648 +1.44388 +0.07299
H +3.09916 −0.64455 +0.55024
H +1.73330 −1.36335 −1.19115
C +3.30982 +2.09519 +1.38420
H +3.74215 +1.36657 −0.60643
H +2.13617 +2.08847 −0.40096
C +2.11992 +2.74468 +2.08267
H +3.80316 +1.37965 +2.04959
H +4.02371 +2.90116 +1.18151
N +2.21620 +2.84368 +3.43399
O +1.19281 +3.20272 +1.45155
H +1.38505 +3.15676 +3.90988
H +2.77661 +2.16383 +3.91915
20
∗ E = +3.031 kcal/mol ; (24) 183_323_062_060_062_257
H +0.00000 +0.00000 +0.00000
O +0.00000 +0.00000 +0.96417
C +1.26733 +0.00000 +1.38482
C +1.38234 −0.06318 +2.89282
N +0.32499 +0.71668 +3.51047
H −0.58642 +0.34142 +3.27391
O +2.19790 −0.02350 +0.62175
C +1.41639 −1.54997 +3.30952
H +2.34582 +0.39571 +3.14091
H +0.41990 +0.69829 +4.51819
C +2.58141 −2.34122 +2.70617
H +1.49604 −1.58087 +4.40193
H +0.46066 −2.01305 +3.03521

C	+3.90602	−1.76147	+3.18060
H	+2.54024	−3.37332	+3.06153
H	+2.52614	−2.35226	+1.61324
N	+4.56114	−0.99412	+2.27711
O	+4.31744	−1.96595	+4.30339
H	+5.39698	−0.52232	+2.57934
H	+4.09534	−0.68406	+1.43761

20

∗ E = +3.062 kcal/mol ; (25) 356_018_092_156_057_085

H	+0.00000	+0.00000	+0.00000
O	+0.00000	+0.00000	+0.97354
C	+1.27242	+0.00000	+1.35044
C	+2.27252	−0.10467	+0.18448
N	+1.60388	+0.23606	−1.06969
H	+1.70299	+1.22322	−1.28000
O	+1.61253	+0.04187	+2.49708
C	+3.54253	+0.68559	+0.50827
H	+2.53037	−1.17209	+0.13150
H	+1.99946	−0.28303	−1.84388
C	+4.77896	+0.19678	−0.25794
H	+3.35875	+1.74860	+0.29825
H	+3.73179	+0.60313	+1.58182
C	+4.56848	+0.22912	−1.75820
H	+5.64686	+0.80403	+0.01994
H	+4.98999	−0.84173	+0.01014
N	+4.81580	+1.42193	−2.35642
O	+4.14220	−0.72631	−2.37547
H	+4.71188	+1.49005	−3.35490
H	+5.26307	+2.17381	−1.86459

20

∗ E = +3.088 kcal/mol ; (26) 358_010_101_135_294_347

H	+0.00000	+0.00000	+0.00000
O	+0.00000	+0.00000	+0.97356
C	+1.26456	+0.00000	+1.35262
C	+2.29153	−0.04910	+0.21295
N	+1.63539	+0.14118	−1.07779
H	+1.75090	+1.08608	−1.42285
O	+1.60486	−0.00425	+2.50504
C	+3.47049	+0.89771	+0.50843
H	+2.65800	−1.08460	+0.25015

H	+1.97963	−0.49330	−1.78429
C	+4.82442	+0.26289	+0.22132
H	+3.35046	+1.80292	−0.09792
H	+3.42424	+1.22553	+1.55161
C	+5.21253	−0.91686	+1.10857
H	+4.88541	−0.09970	−0.81070
H	+5.62721	+0.99963	+0.32910
N	+4.46643	−1.12852	+2.21847
O	+6.16333	−1.61128	+0.81765
H	+4.73448	−1.89997	+2.80626
H	+3.63500	−0.60799	+2.46269

20

* E = +3.236 kcal/mol ; (27) 179_049_311_183_302_091

H	+0.00000	+0.00000	+0.00000
O	+0.00000	+0.00000	+0.96376
C	+1.27918	+0.00000	+1.38196
C	+1.36537	+0.01346	+2.90277
N	+0.51659	−0.99976	+3.52952
H	−0.41970	−0.96769	+3.14224
O	+2.20518	−0.00667	+0.62467
C	+2.81766	−0.12522	+3.34710
H	+0.98132	+0.99515	+3.21700
H	+0.88542	−1.92810	+3.34905
C	+2.97700	−0.20299	+4.86935
H	+3.24622	−1.02332	+2.88638
H	+3.38614	+0.72148	+2.95242
C	+2.38108	+1.01603	+5.55528
H	+2.50685	−1.11392	+5.25173
H	+4.03879	−0.22769	+5.12094
N	+1.07819	+0.87909	+5.91163
O	+3.01897	+2.02801	+5.74976
H	+0.60199	+1.67799	+6.29452
H	+0.55004	+0.08932	+5.57109

20

* E = +3.246 kcal/mol ; (28) 190_331_301_053_279_163

H	+0.00000	+0.00000	+0.00000
O	+0.00000	+0.00000	+0.96386
C	+1.26830	+0.00000	+1.37839
C	+1.40661	−0.24320	+2.87466
N	+0.29652	+0.20349	+3.69163

H +0.13485 +1.19729 +3.58546
O +2.20419 +0.14168 +0.63681
C +1.62792 −1.74525 +3.11161
H +2.31426 +0.29314 +3.17585
H −0.55493 −0.26927 +3.40889
C +2.76914 −2.34462 +2.29895
H +1.80885 −1.86826 +4.18243
H +0.70269 −2.27957 +2.86469
C +2.35345 −2.66768 +0.86927
H +3.64013 −1.67858 +2.28648
H +3.09437 −3.28835 +2.75154
N +3.37655 −2.84841 −0.00195
O +1.19172 −2.80831 +0.55051
H +3.14216 −2.97224 −0.97244
H +4.29522 −2.50988 +0.21964
20
∗ E = +3.261 kcal/mol ; (29) 177_169_272_293_253_045
H +0.00000 +0.00000 +0.00000
O +0.00000 +0.00000 +0.96425
C +1.26915 +0.00000 +1.38639
C +1.37490 +0.06524 +2.90248
N +2.73659 −0.19861 +3.34715
H +2.88663 −1.19126 +3.47611
O +2.20830 −0.01182 +0.64089
C +0.88600 +1.44671 +3.37481
H +0.69838 −0.69189 +3.31287
H +3.38337 +0.08940 +2.61663
C +1.79767 +2.60849 +2.93274
H −0.12721 +1.60094 +2.99393
H +0.81744 +1.41665 +4.46647
C +2.64672 +3.22712 +4.03885
H +2.48224 +2.28291 +2.13614
H +1.21411 +3.43154 +2.51883
N +3.26545 +2.34511 +4.85775
O +2.74964 +4.42941 +4.15939
H +3.86694 +2.71112 +5.57516
H +3.22947 +1.35097 +4.67113
20
∗ E = +3.291 kcal/mol ; (30) 178_167_283_171_109_297
H +0.00000 +0.00000 +0.00000

O +0.00000 +0.00000 +0.96421
C +1.27005 +0.00000 +1.38649
C +1.37338 +0.05462 +2.90384
N +2.72159 −0.24104 +3.36104
H +2.90276 −1.23685 +3.32066
O +2.20884 −0.01558 +0.64176
C +0.91130 +1.43812 +3.38874
H +0.68192 −0.69397 +3.30922
H +3.38038 +0.18724 +2.71461
C +0.76370 +1.51641 +4.92183
H +1.63128 +2.18693 +3.03396
H −0.04471 +1.66861 +2.91152
C +1.83081 +2.36048 +5.60619
H −0.19344 +1.96060 +5.19607
H +0.79980 +0.50571 +5.34700
N +3.09164 +1.89098 +5.46854
O +1.55779 +3.37235 +6.21639
H +3.84516 +2.40406 +5.89148
H +3.26068 +1.03815 +4.95235
20
* E = +3.315 kcal/mol ; (31) 352_029_083_075_219_073
H +0.00000 +0.00000 +0.00000
O +0.00000 +0.00000 +0.97158
C +1.26540 +0.00000 +1.36327
C +2.28046 −0.19347 +0.22587
N +1.69571 +0.33618 −1.00416
H +1.82718 +1.34036 −1.06542
O +1.57767 +0.12513 +2.51549
C +3.64745 +0.36554 +0.62315
H +2.36227 −1.28253 +0.09660
H +2.12141 −0.07639 −1.82347
C +4.39159 −0.53473 +1.63088
H +4.25093 +0.48699 −0.28123
H +3.50270 +1.36683 +1.04757
C +5.12703 +0.28426 +2.67986
H +3.67529 −1.18714 +2.14070
H +5.12945 −1.15905 +1.12474
N +4.30887 +0.85306 +3.59599
O +6.33062 +0.42876 +2.67345
H +4.71033 +1.44048 +4.30559

H +3.31141 +0.70713 +3.55500
20
* E = +3.362 kcal/mol ; (32) 179_005_311_180_080_205
H +0.00000 +0.00000 +0.00000
O +0.00000 +0.00000 +0.96413
C +1.27925 +0.00000 +1.36760
C +1.43956 +0.01531 +2.88520
N +0.22855 −0.08684 +3.66943
H −0.47597 +0.56739 +3.35500
O +2.19786 +0.00639 +0.59718
C +2.43266 −1.08427 +3.27679
H +1.90926 +0.98665 +3.10342
H −0.15981 −1.02300 +3.61483
C +2.65144 −1.13078 +4.78273
H +2.04461 −2.05778 +2.95399
H +3.37031 −0.91030 +2.74510
C +1.52382 −1.85993 +5.49880
H +3.57131 −1.68156 +5.01177
H +2.76776 −0.12098 +5.19405
N +1.33627 −1.50709 +6.79361
O +0.88276 −2.74331 +4.96837
H +0.57820 −1.93593 +7.29625
H +1.75813 −0.67963 +7.17207
20
* E = +3.418 kcal/mol ; (33) 181_209_296_280_056_107
H +0.00000 +0.00000 +0.00000
O +0.00000 +0.00000 +0.96397
C +1.28658 +0.00000 +1.35982
C +1.46276 −0.03591 +2.87838
N +2.69481 +0.60558 +3.29645
H +3.48657 +0.09347 +2.92644
O +2.19650 −0.04049 +0.58465
C +0.26069 +0.47978 +3.67909
H +1.54813 −1.10894 +3.10340
H +2.73252 +1.52772 +2.86799
C +0.16901 +2.00882 +3.78065
H −0.66221 +0.07075 +3.25789
H +0.35945 +0.09134 +4.69682
C +0.14143 +2.66800 +2.41927
H −0.70799 +2.28387 +4.37442

H +1.05698 +2.38350 +4.29888
N −1.04197 +3.18520 +2.01847
O +1.13601 +2.70762 +1.71958
H −1.10421 +3.59427 +1.10185
H −1.86541 +3.12851 +2.58740
20
∗ E = +3.456 kcal/mol ; (34) 167_151_034_301_081_200
H +0.00000 +0.00000 +0.00000
O +0.00000 +0.00000 +0.96410
C +1.26028 +0.00000 +1.38533
C +1.37949 +0.31454 +2.86045
N +2.56843 −0.33919 +3.36966
H +3.29235 −0.35873 +2.66063
O +2.20832 −0.16614 +0.66435
C +1.36491 +1.85478 +3.01024
H +0.48865 −0.07952 +3.36201
H +2.92331 +0.11649 +4.19976
C +2.49090 +2.55086 +2.25236
H +0.40425 +2.24530 +2.65861
H +1.44455 +2.07557 +4.08008
C +2.17180 +2.72869 +0.77262
H +2.65509 +3.55525 +2.65908
H +3.43739 +2.00810 +2.36639
N +3.24607 +2.93260 −0.02854
O +1.03405 +2.74184 +0.35375
H +3.08633 +2.97268 −1.02099
H +4.17168 +2.71540 +0.29245
20
∗ E = +3.577 kcal/mol ; (35) 005_339_150_060_170_164
H +0.00000 +0.00000 +0.00000
O +0.00000 +0.00000 +0.97236
C +1.27214 +0.00000 +1.35752
C +2.28329 +0.13150 +0.21346
N +1.65789 −0.26472 −1.04518
H +2.06298 +0.22797 −1.83079
O +1.60354 −0.05128 +2.50696
C +2.73716 +1.59120 +0.11176
H +3.14543 −0.48942 +0.49064
H +1.74222 −1.25796 −1.21975
C +3.39490 +2.11987 +1.37678

H	+3.45171	+1.67855	−0.71431
H	+1.87725	+2.21751	−0.15730
C	+4.04200	+3.47226	+1.12811
H	+2.68247	+2.18629	+2.20463
H	+4.17939	+1.42978	+1.71364
N	+4.34031	+4.17394	+2.24955
O	+4.29301	+3.88410	+0.01654
H	+4.79517	+5.06543	+2.15647
H	+4.10159	+3.83653	+3.16358

20

∗ E = +3.579 kcal/mol ; (36) 177_161_299_313_290_102

H	+0.00000	+0.00000	+0.00000
O	+0.00000	+0.00000	+0.96447
C	+1.26819	+0.00000	+1.38868
C	+1.36655	+0.07385	+2.90597
N	+2.64301	−0.34561	+3.43524
H	+2.83491	−1.31208	+3.20353
O	+2.20218	−0.00093	+0.63053
C	+1.01564	+1.51758	+3.32483
H	+0.58899	−0.58311	+3.31170
H	+3.40192	+0.20887	+3.05705
C	+1.71095	+2.62102	+2.51461
H	−0.06521	+1.65208	+3.22428
H	+1.27468	+1.62056	+4.38286
C	+3.20421	+2.72294	+2.79047
H	+1.52135	+2.49514	+1.44175
H	+1.28873	+3.58414	+2.81134
N	+4.01552	+2.17103	+1.85053
O	+3.62822	+3.26220	+3.78795
H	+5.00951	+2.20182	+2.00345
H	+3.64627	+1.62234	+1.09015

20

∗ E = +3.612 kcal/mol ; (37) 356_294_286_227_070_184

H	+0.00000	+0.00000	+0.00000
O	+0.00000	+0.00000	+0.97489
C	+1.23850	+0.00000	+1.47258
C	+2.35911	−0.10169	+0.43353
N	+2.35026	−1.39929	−0.24668
H	+2.70640	−2.11463	+0.37418
O	+1.44248	+0.04421	+2.65147

C +2.30685 +1.10214 −0.55090
H +3.28931 −0.05061 +1.00298
H +1.41163 −1.67129 −0.51721
C +2.41978 +0.71898 −2.01811
H +1.37572 +1.66205 −0.41312
H +3.11605 +1.79232 −0.30047
C +1.18017 −0.00549 −2.50147
H +2.54210 +1.61914 −2.63232
H +3.28742 +0.07608 −2.19675
N +1.22007 −0.46378 −3.76548
O +0.18812 −0.15425 −1.80116
H +0.42228 −0.95432 −4.13235
H +2.04467 −0.37539 −4.33035
20
* E = +3.677 kcal/mol ; (38) 003_338_172_184_315_277
H +0.00000 +0.00000 +0.00000
O +0.00000 +0.00000 +0.97137
C +1.27191 +0.00000 +1.36953
C +2.28715 +0.07121 +0.22839
N +1.67374 −0.38296 −1.01477
H +2.30120 −0.20695 −1.79350
O +1.59139 +0.00229 +2.52109
C +2.71123 +1.54365 +0.12981
H +3.15561 −0.52947 +0.53419
H +1.55452 −1.39061 −0.99163
C +3.66704 +1.83372 −1.03750
H +1.81871 +2.17007 +0.01682
H +3.17552 +1.81060 +1.08481
C +4.76458 +0.78984 −1.10783
H +3.13398 +1.79980 −1.99079
H +4.08342 +2.84005 −0.92841
N +5.81940 +0.98819 −0.28379
O +4.66448 −0.19009 −1.81867
H +6.54274 +0.29042 −0.24299
H +5.88543 +1.79502 +0.30888
20
* E = +3.685 kcal/mol ; (39) 179_039_202_186_305_262
H +0.00000 +0.00000 +0.00000
O +0.00000 +0.00000 +0.96361
C +1.27880 +0.00000 +1.37412

C	+1.40280	+0.03609	+2.88391
N	+0.38709	−0.79172	+3.51738
H	+0.26307	−0.46911	+4.47223
O	+2.19825	+0.01167	+0.60602
C	+2.81013	−0.41400	+3.27911
H	+1.28553	+1.10398	+3.14703
H	−0.50082	−0.69044	+3.04193
C	+2.98379	−0.54784	+4.79772
H	+3.00613	−1.38757	+2.81893
H	+3.53895	+0.28931	+2.86382
C	+2.60762	+0.73410	+5.51160
H	+2.31943	−1.33283	+5.16869
H	+4.01346	−0.83913	+5.02638
N	+3.63253	+1.55430	+5.84506
O	+1.44806	+1.02640	+5.73339
H	+3.42530	+2.43522	+6.28390
H	+4.58797	+1.30764	+5.66698

20

∗ E = +3.798 kcal/mol ; (40) 003_346_148_303_195_217

H	+0.00000	+0.00000	+0.00000
O	+0.00000	+0.00000	+0.97247
C	+1.27477	+0.00000	+1.35599
C	+2.28776	+0.08189	+0.20687
N	+1.63040	−0.20035	−1.06891
H	+2.05806	+0.31940	−1.82574
O	+1.60889	−0.00190	+2.50397
C	+2.87373	+1.49708	+0.21207
H	+3.08905	−0.62736	+0.44394
H	+1.67287	−1.18297	−1.30698
C	+1.80081	+2.57775	+0.09238
H	+3.43378	+1.63385	+1.14148
H	+3.58151	+1.60351	−0.61710
C	+2.41901	+3.92168	−0.25539
H	+1.10393	+2.33603	−0.72116
H	+1.20642	+2.64020	+1.01118
N	+1.83792	+4.99915	+0.32472
O	+3.34939	+4.01719	−1.02565
H	+2.19434	+5.91438	+0.10900
H	+1.09541	+4.90629	+0.99242

20

∗ E = +3.819 kcal/mol ; (41) 180_353_186_062_058_251

H	+0.00000	+0.00000	+0.00000
O	+0.00000	+0.00000	+0.96460
C	+1.26946	+0.00000	+1.36950
C	+1.44814	+0.00802	+2.87533
N	+0.18325	+0.18145	+3.56344
H	+0.33888	+0.28400	+4.55812
O	+2.18897	+0.00493	+0.58996
C	+2.08268	−1.31286	+3.34694
H	+2.17711	+0.81653	+3.05943
H	−0.32499	+0.99241	+3.23497
C	+3.47692	−1.59383	+2.78578
H	+2.15518	−1.25168	+4.43887
H	+1.39522	−2.13209	+3.11363
C	+4.42254	−0.45970	+3.14766
H	+3.87018	−2.49971	+3.25413
H	+3.44454	−1.75095	+1.70539
N	+4.80131	+0.32886	+2.11281
O	+4.78687	−0.27769	+4.29082
H	+5.35634	+1.14257	+2.31814
H	+4.29884	+0.28040	+1.24051

20

∗ E = +3.885 kcal/mol ; (42) 178_169_296_173_177_184

H	+0.00000	+0.00000	+0.00000
O	+0.00000	+0.00000	+0.96390
C	+1.27390	+0.00000	+1.38259
C	+1.37903	+0.04776	+2.90069
N	+2.72227	−0.20588	+3.37854
H	+3.01842	−1.14376	+3.13647
O	+2.20687	−0.02261	+0.63118
C	+0.88410	+1.41279	+3.39391
H	+0.70772	−0.72803	+3.29310
H	+3.36811	+0.42123	+2.90864
C	+0.80845	+1.45803	+4.91066
H	+1.56801	+2.19348	+3.03839
H	−0.09379	+1.63933	+2.96107
C	+0.39843	+2.83140	+5.41085
H	+0.07919	+0.72463	+5.27973
H	+1.77476	+1.17899	+5.34534
N	+0.38904	+2.96635	+6.76299

O	+0.09287	+3.73691	+4.66754
H	+0.13626	+3.85512	+7.15826
H	+0.68564	+2.22560	+7.37047

20

∗ E = +3.904 kcal/mol ; (43) 175_161_302_059_276_168

H	+0.00000	+0.00000	+0.00000
O	+0.00000	+0.00000	+0.96434
C	+1.28346	+0.00000	+1.37166
C	+1.40240	+0.12753	+2.88567
N	+2.69624	−0.29838	+3.37662
H	+2.87824	−1.25989	+3.11380
O	+2.20375	−0.11783	+0.61796
C	+1.14920	+1.56868	+3.35305
H	+0.62444	−0.51548	+3.31926
H	+3.41993	+0.25444	+2.92766
C	−0.21470	+2.14450	+2.99369
H	+1.28080	+1.55885	+4.43798
H	+1.92721	+2.21538	+2.93077
C	−0.25653	+2.71283	+1.58279
H	−1.00801	+1.39519	+3.10473
H	−0.46018	+2.96795	+3.67485
N	−1.49721	+3.00878	+1.12145
O	+0.74332	+2.91039	+0.92837
H	−1.58870	+3.42928	+0.21312
H	−2.32151	+2.82155	+1.66096

20

∗ E = +3.919 kcal/mol ; (44) 351_318_298_311_296_253

H	+0.00000	+0.00000	+0.00000
O	+0.00000	+0.00000	+0.97438
C	+1.23188	+0.00000	+1.48173
C	+2.39544	−0.20845	+0.49728
N	+2.21683	−1.22454	−0.52507
H	+2.10338	−2.14146	−0.11375
O	+1.42424	+0.17339	+2.65177
C	+2.81955	+1.14699	−0.10024
H	+3.22419	−0.53257	+1.13342
H	+1.43110	−1.04631	−1.13685
C	+1.71137	+2.05168	−0.66929
H	+3.31265	+1.71769	+0.69235
H	+3.56824	+0.93716	−0.87177

C +0.98956 +1.45750 −1.85872
H +0.95543 +2.24786 +0.09963
H +2.15224 +3.01539 −0.94175
N +1.26455 +1.99084 −3.06098
O +0.19724 +0.53060 −1.74001
H +0.81883 +1.60493 −3.87682
H +1.91279 +2.74874 −3.16829
20
* E = +3.949 kcal/mol ; (45) 183_191_206_302_189_185
H +0.00000 +0.00000 +0.00000
O +0.00000 +0.00000 +0.96377
C +1.27600 +0.00000 +1.37516
C +1.39183 −0.06324 +2.88529
N +2.77115 +0.20144 +3.24742
H +3.00957 −0.22246 +4.13285
O +2.20142 +0.02211 +0.61365
C +0.44309 +0.93020 +3.56646
H +1.05761 −1.08283 +3.14547
H +3.40394 −0.13525 +2.53196
C +0.70718 +2.35758 +3.11642
H −0.59652 +0.65415 +3.37104
H +0.58692 +0.86242 +4.64922
C −0.06649 +3.36471 +3.94726
H +1.77929 +2.57548 +3.19848
H +0.43561 +2.49195 +2.06069
N +0.04604 +4.65348 +3.53023
O −0.73494 +3.05187 +4.90711
H −0.40510 +5.37475 +4.06528
H +0.64701 +4.91083 +2.76958
20
* E = +4.005 kcal/mol ; (46) 179_150_296_063_066_258
H +0.00000 +0.00000 +0.00000
O +0.00000 +0.00000 +0.96519
C +1.28857 +0.00000 +1.37384
C +1.44412 +0.03355 +2.88977
N +2.65298 −0.63072 +3.32877
H +2.63088 −1.61875 +3.10800
O +2.19698 −0.00721 +0.59559
C +1.48498 +1.49793 +3.35697
H +0.57444 −0.46362 +3.33605

H	+3.45342	−0.23885	+2.84318
C	+0.20840	+2.29976	+3.08760
H	+1.68234	+1.48119	+4.43276
H	+2.33997	+1.98555	+2.87290
C	−0.94193	+1.75692	+3.92157
H	+0.36483	+3.33299	+3.40522
H	−0.04311	+2.30750	+2.02234
N	−1.82302	+0.96486	+3.25937
O	−1.04040	+2.00359	+5.10316
H	−2.55727	+0.52566	+3.78845
H	−1.64329	+0.66483	+2.31691

20

* E = +4.007 kcal/mol ; (47) 356_019_090_302_192_223

H	+0.00000	+0.00000	+0.00000
O	+0.00000	+0.00000	+0.97184
C	+1.27152	+0.00000	+1.35755
C	+2.28681	−0.10149	+0.20852
N	+1.64516	+0.27133	−1.05180
H	+1.73139	+1.26528	−1.23226
O	+1.60134	+0.05003	+2.50701
C	+3.57077	+0.65256	+0.55497
H	+2.52186	−1.17164	+0.14463
H	+2.06122	−0.21276	−1.83573
C	+3.33418	+2.13044	+0.85609
H	+4.02762	+0.18085	+1.42995
H	+4.27775	+0.56697	−0.27610
C	+4.65278	+2.88564	+0.90245
H	+2.74681	+2.60698	+0.05921
H	+2.77278	+2.24430	+1.78858
N	+4.77957	+3.77772	+1.91437
O	+5.51721	+2.71246	+0.07160
H	+5.63404	+4.30262	+1.98975
H	+4.08472	+3.86577	+2.63213

20

* E = +4.030 kcal/mol ; (48) 181_061_298_175_291_172

H	+0.00000	+0.00000	+0.00000
O	+0.00000	+0.00000	+0.96353
C	+1.28019	+0.00000	+1.37826
C	+1.37104	−0.03466	+2.89658
N	+0.75716	−1.24241	+3.44513

H	−0.23131	−1.27642	+3.22850
O	+2.20261	−0.00626	+0.61382
C	+2.82262	+0.08312	+3.34172
H	+0.80888	+0.82882	+3.27165
H	+1.18679	−2.07184	+3.04929
C	+2.94473	+0.17611	+4.85478
H	+3.37980	−0.78526	+2.97114
H	+3.26461	+0.96724	+2.87505
C	+2.40766	+1.49429	+5.39082
H	+2.40540	−0.64977	+5.33258
H	+3.99427	+0.09775	+5.16127
N	+2.32679	+1.56897	+6.74364
O	+2.09011	+2.41803	+4.67292
H	+1.98182	+2.41510	+7.16228
H	+2.54980	+0.78633	+7.32969

20

∗ E = +4.053 kcal/mol ; (49) 180_048_186_074_068_191

H	+0.00000	+0.00000	+0.00000
O	+0.00000	+0.00000	+0.96366
C	+1.27692	+0.00000	+1.37862
C	+1.37598	+0.00576	+2.89222
N	+0.47765	−1.01212	+3.42525
H	+0.46546	−0.95336	+4.43627
O	+2.19863	+0.02439	+0.61210
C	+2.80556	−0.26699	+3.34915
H	+1.09466	+1.02789	+3.20455
H	−0.46866	−0.86886	+3.09443
C	+3.73864	+0.91740	+3.14410
H	+2.77151	−0.50600	+4.41664
H	+3.18537	−1.14492	+2.81935
C	+3.39076	+2.07707	+4.06437
H	+4.77239	+0.62590	+3.36450
H	+3.72616	+1.25034	+2.10047
N	+4.01910	+3.24461	+3.77661
O	+2.62118	+1.96958	+4.99577
H	+3.85683	+4.04162	+4.36710
H	+4.64369	+3.32934	+2.99644

20

∗ E = +4.056 kcal/mol ; (50) 176_027_183_277_062_280

H	+0.00000	+0.00000	+0.00000

O	+0.00000	+0.00000	+0.96389
C	+1.28240	+0.00000	+1.36982
C	+1.41122	+0.09553	+2.88349
N	+0.23065	−0.45298	+3.54455
H	+0.30940	−0.34272	+4.54862
O	+2.20340	+0.01585	+0.60902
C	+2.70189	−0.56741	+3.37642
H	+1.52062	+1.18158	+3.05320
H	−0.60925	+0.02626	+3.24300
C	+2.63070	−2.09024	+3.55047
H	+3.50032	−0.30747	+2.67519
H	+2.96572	−0.12563	+4.34374
C	+2.35458	−2.83946	+2.25544
H	+3.59334	−2.44879	+3.91889
H	+1.86230	−2.34712	+4.28725
N	+1.04839	−2.85276	+1.88728
O	+3.23051	−3.37389	+1.61305
H	+0.79710	−3.30466	+1.02514
H	+0.35538	−2.36623	+2.43810

20

∗ E = +4.164 kcal/mol ; (51) 179_153_289_051_059_197

H	+0.00000	+0.00000	+0.00000
O	+0.00000	+0.00000	+0.96447
C	+1.28003	+0.00000	+1.37729
C	+1.40561	+0.02208	+2.89299
N	+2.64034	−0.57875	+3.35426
H	+2.62735	−1.58251	+3.22377
O	+2.20172	+0.00771	+0.61012
C	+1.34867	+1.47652	+3.39089
H	+0.55001	−0.51453	+3.31391
H	+3.41775	−0.22066	+2.80854
C	+0.17316	+2.30024	+2.87950
H	+1.31859	+1.42507	+4.48191
H	+2.28664	+1.96961	+3.10911
C	−1.16763	+1.68739	+3.25522
H	+0.20843	+3.30010	+3.32824
H	+0.22062	+2.44794	+1.79433
N	−2.22940	+2.14600	+2.54639
O	−1.28405	+0.87889	+4.14968
H	−3.13965	+1.76944	+2.74677

H −2.11468 +2.75449 +1.75793
20
∗ E = +4.200 kcal/mol ; (52) 179_046_182_294_143_294
H +0.00000 +0.00000 +0.00000
O +0.00000 +0.00000 +0.96419
C +1.26744 +0.00000 +1.38756
C +1.36300 +0.01361 +2.90425
N +0.43236 −0.96512 +3.44688
H +0.45181 −0.94750 +4.45878
O +2.19870 +0.02414 +0.62848
C +2.79192 −0.28497 +3.33933
H +1.11814 +1.05390 +3.18954
H −0.51644 −0.78605 +3.14334
C +3.23753 −1.71752 +2.99078
H +3.45023 +0.44690 +2.86106
H +2.87298 −0.11810 +4.41839
C +4.69590 −1.77569 +2.56747
H +3.11107 −2.39303 +3.83654
H +2.62314 −2.10639 +2.17069
N +4.95449 −1.13069 +1.40244
O +5.55010 −2.34148 +3.21505
H +5.89922 −1.10992 +1.06118
H +4.21858 −0.67032 +0.88973
20
∗ E = +4.208 kcal/mol ; (53) 355_021_088_069_167_156
H +0.00000 +0.00000 +0.00000
O +0.00000 +0.00000 +0.97200
C +1.27084 +0.00000 +1.35946
C +2.28336 −0.12461 +0.20988
N +1.63501 +0.27419 −1.04070
H +1.72707 +1.27408 −1.18706
O +1.59127 +0.06216 +2.51144
C +3.58870 +0.61421 +0.50599
H +2.49284 −1.20158 +0.13717
H +2.05761 −0.18423 −1.83686
C +4.43615 −0.01651 +1.60329
H +4.18662 +0.64158 −0.41109
H +3.36406 +1.65685 +0.76501
C +5.83443 +0.57958 +1.62001
H +3.95498 +0.08003 +2.57913

H	+4.55143	−1.09287	+1.41399
N	+6.47956	+0.48751	+2.80932
O	+6.34732	+1.07331	+0.63922
H	+7.41591	+0.84625	+2.88104
H	+6.02485	+0.13168	+3.62944

20

∗ E = +4.225 kcal/mol ; (54) 179_045_306_292_144_294

H	+0.00000	+0.00000	+0.00000
O	+0.00000	+0.00000	+0.96420
C	+1.26903	+0.00000	+1.38637
C	+1.37071	+0.02842	+2.91197
N	+0.46257	−0.88585	+3.58258
H	−0.49593	−0.73577	+3.29404
O	+2.19679	−0.00586	+0.62004
C	+2.81336	−0.21566	+3.33299
H	+1.08289	+1.04971	+3.19904
H	+0.69913	−1.85021	+3.37944
C	+3.28923	−1.64668	+3.01290
H	+3.44974	+0.51266	+2.82218
H	+2.89503	−0.02749	+4.40596
C	+4.74977	−1.68929	+2.58877
H	+3.17887	−2.30722	+3.87408
H	+2.70032	−2.06404	+2.18486
N	+4.99280	−1.05855	+1.41349
O	+5.61079	−2.23417	+3.24339
H	+5.93660	−1.02238	+1.07075
H	+4.24764	−0.61421	+0.89936

20

∗ E = +4.280 kcal/mol ; (55) 183_066_303_066_217_066

H	+0.00000	+0.00000	+0.00000
O	+0.00000	+0.00000	+0.96428
C	+1.26799	+0.00000	+1.39028
C	+1.35507	−0.07649	+2.91207
N	+0.85582	−1.35718	+3.39465
H	−0.09483	−1.52670	+3.08999
O	+2.19971	+0.00933	+0.62879
C	+2.79027	+0.14404	+3.36187
H	+0.70475	+0.71069	+3.31545
H	+1.43688	−2.11925	+3.06376
C	+3.29600	+1.56721	+3.06427

H	+2.84669	−0.06882	+4.43176
H	+3.42247	−0.59460	+2.85381
C	+4.75819	+1.59290	+2.64547
H	+2.71073	+2.01104	+2.24933
H	+3.19504	+2.21230	+3.93797
N	+4.99701	+0.98539	+1.45724
O	+5.62632	+2.10519	+3.31732
H	+5.94170	+0.94346	+1.11759
H	+4.24559	+0.57430	+0.92512

20

∗ E = +4.295 kcal/mol ; (56) 177_164_283_293_174_166

H	+0.00000	+0.00000	+0.00000
O	+0.00000	+0.00000	+0.96407
C	+1.27086	+0.00000	+1.38527
C	+1.37912	+0.07716	+2.90199
N	+2.69820	−0.28769	+3.38174
H	+2.81971	−1.29255	+3.36329
O	+2.20622	−0.01550	+0.63350
C	+1.00519	+1.50174	+3.34069
H	+0.63682	−0.60997	+3.32113
H	+3.40186	+0.09107	+2.75534
C	+2.02652	+2.53127	+2.87857
H	+0.01665	+1.76909	+2.95712
H	+0.94390	+1.51388	+4.43233
C	+1.57554	+3.94672	+3.20056
H	+3.00061	+2.34525	+3.34620
H	+2.18323	+2.47270	+1.79190
N	+2.54542	+4.89185	+3.09592
O	+0.43741	+4.21778	+3.50990
H	+2.30443	+5.85252	+3.26686
H	+3.48937	+4.65862	+2.85079

20

∗ E = +4.295 kcal/mol ; (57) 177_164_283_293_175_169

H	+0.00000	+0.00000	+0.00000
O	+0.00000	+0.00000	+0.96407
C	+1.27101	+0.00000	+1.38507
C	+1.37973	+0.07556	+2.90179
N	+2.69955	−0.28814	+3.38035
H	+2.82100	−1.29302	+3.36457
O	+2.20607	−0.01409	+0.63287

C +1.00392 +1.49909 +3.34256
H +0.63858 −0.61307 +3.32050
H +3.40248 +0.08904 +2.75220
C +2.02162 +2.53077 +2.87793
H +0.01314 +1.76366 +2.96278
H +0.94604 +1.51104 +4.43426
C +1.57734 +3.94423 +3.21728
H +2.99925 +2.34117 +3.33698
H +2.17049 +2.47917 +1.78990
N +2.53325 +4.89577 +3.05565
O +0.45657 +4.20820 +3.58956
H +2.29597 +5.85529 +3.23766
H +3.46204 +4.66849 +2.75346
20
∗ E = +4.366 kcal/mol ; (58) 178_148_046_066_063_251
H +0.00000 +0.00000 +0.00000
O +0.00000 +0.00000 +0.96509
C +1.28766 +0.00000 +1.36781
C +1.44523 +0.04636 +2.87330
N +2.65030 −0.68982 +3.20197
H +3.40446 −0.41425 +2.58219
O +2.19621 +0.00726 +0.59010
C +1.46432 +1.52327 +3.32353
H +0.57756 −0.45298 +3.32295
H +2.93308 −0.50912 +4.15663
C +0.14176 +2.26568 +3.11185
H +1.69574 +1.53427 +4.39456
H +2.28092 +2.03527 +2.80159
C −0.94417 +1.62843 +3.96458
H +0.24869 +3.29813 +3.45351
H −0.14065 +2.28646 +2.05509
N −1.88593 +0.92394 +3.28822
O −0.94213 +1.73572 +5.17153
H −2.56794 +0.41291 +3.82338
H −1.75155 +0.69208 +2.31927
20
∗ E = +4.398 kcal/mol ; (59) 190_325_056_055_278_161
H +0.00000 +0.00000 +0.00000
O +0.00000 +0.00000 +0.96370
C +1.26525 +0.00000 +1.38244

C	+1.38998	−0.24675	+2.87123
N	+0.28240	+0.36092	+3.58629
H	−0.60124	+0.01863	+3.22614
O	+2.20634	+0.11259	+0.64397
C	+1.53941	−1.76678	+3.09637
H	+2.32250	+0.24434	+3.17289
H	+0.32796	+0.13131	+4.57135
C	+2.68965	−2.39748	+2.31800
H	+1.68507	−1.91987	+4.17093
H	+0.60316	−2.25976	+2.81016
C	+2.30503	−2.70173	+0.87495
H	+3.57771	−1.75404	+2.33546
H	+2.97691	−3.35229	+2.77298
N	+3.34648	−2.87226	+0.02332
O	+1.15034	−2.83927	+0.53140
H	+3.12984	−2.97945	−0.95343
H	+4.25648	−2.52330	+0.26419

20

∗ E = +4.404 kcal/mol ; (60) 182_196_200_090_067_186

H	+0.00000	+0.00000	+0.00000
O	+0.00000	+0.00000	+0.96379
C	+1.28360	+0.00000	+1.36934
C	+1.41398	−0.05748	+2.87536
N	+2.76638	+0.31501	+3.23283
H	+2.98026	−0.02316	+4.16260
O	+2.19764	+0.00533	+0.59597
C	+0.39861	+0.84652	+3.59719
H	+1.17414	−1.10584	+3.13283
H	+3.43240	−0.07007	+2.57399
C	−0.91088	+0.14423	+3.92134
H	+0.86946	+1.16149	+4.53194
H	+0.21433	+1.74870	+3.00646
C	−0.70010	−0.94960	+4.95890
H	−1.63953	+0.85545	+4.32790
H	−1.36445	−0.29013	+3.02291
N	−1.79016	−1.71368	+5.21903
O	+0.36153	−1.12531	+5.51870
H	−1.72078	−2.43789	+5.91271
H	−2.66571	−1.56089	+4.75431

20

∗ E = +4.535 kcal/mol ; (61) 179_170_292_153_288_352

H	+0.00000	+0.00000	+0.00000
O	+0.00000	+0.00000	+0.96407
C	+1.28070	+0.00000	+1.39216
C	+1.34872	+0.02414	+2.91010
N	+2.67226	−0.19203	+3.44371
H	+2.99052	−1.13692	+3.26881
O	+2.21303	−0.00624	+0.64455
C	+0.78497	+1.36265	+3.43014
H	+0.69382	−0.78635	+3.25815
H	+3.33524	+0.42845	+2.99103
C	+0.22211	+1.22446	+4.83727
H	+1.60801	+2.08574	+3.43130
H	+0.02793	+1.76359	+2.74691
C	−1.08518	+0.45467	+4.97657
H	+0.95096	+0.72413	+5.48327
H	+0.03946	+2.20665	+5.28447
N	−1.72625	+0.09570	+3.83249
O	−1.53482	+0.19162	+6.06981
H	−2.59218	−0.40665	+3.92847
H	−1.36850	+0.26670	+2.90892

20

∗ E = +4.564 kcal/mol ; (62) 347_353_295_276_046_076

H	+0.00000	+0.00000	+0.00000
O	+0.00000	+0.00000	+0.96509
C	+1.24930	+0.00000	+1.42982
C	+2.39095	−0.31865	+0.43640
N	+2.03353	−0.74886	−0.89594
H	+1.54910	−1.63416	−0.92105
O	+1.48180	+0.21371	+2.58517
C	+3.36767	+0.86964	+0.39362
H	+2.91748	−1.14374	+0.92984
H	+1.53749	−0.05042	−1.43850
C	+2.92576	+1.99511	−0.55864
H	+3.48206	+1.24371	+1.41627
H	+4.34415	+0.50683	+0.06238
C	+1.45756	+2.32453	−0.37549
H	+3.54502	+2.88198	−0.39307
H	+3.05211	+1.68188	−1.59720
N	+1.13997	+3.04262	+0.72023

O +0.60929 +1.90015 −1.14447
H +0.16794 +3.11999 +0.97279
H +1.82933 +3.24770 +1.42209
20
∗ E = +4.580 kcal/mol ; (63) 182_056_179_071_216_069
H +0.00000 +0.00000 +0.00000
O +0.00000 +0.00000 +0.96422
C +1.26748 +0.00000 +1.38942
C +1.34754 −0.03765 +2.90744
N +0.60640 −1.21263 +3.34803
H +0.64030 −1.29299 +4.35636
O +2.19982 +0.02824 +0.63273
C +2.80127 −0.11580 +3.35026
H +0.90855 +0.91475 +3.25947
H −0.36486 −1.16307 +3.06616
C +3.56322 +1.19996 +3.10946
H +2.83143 −0.37417 +4.41343
H +3.27305 −0.94460 +2.81248
C +5.00815 +0.95971 +2.70128
H +3.07820 +1.76972 +2.30807
H +3.57464 +1.82177 +4.00578
N +5.14105 +0.40649 +1.47137
O +5.94832 +1.23186 +3.41568
H +6.06181 +0.17874 +1.13993
H +4.32941 +0.17964 +0.91791
20
∗ E = +4.605 kcal/mol ; (64) 182_185_221_162_054_086
H +0.00000 +0.00000 +0.00000
O +0.00000 +0.00000 +0.96369
C +1.27736 +0.00000 +1.37270
C +1.40390 −0.04140 +2.88406
N +2.80426 +0.07685 +3.24424
H +3.04282 −0.54344 +4.00619
O +2.19796 +0.00772 +0.60554
C +0.55472 +1.06580 +3.53425
H +0.97137 −1.01254 +3.18194
H +3.38852 −0.12295 +2.44126
C +0.28359 +0.80103 +5.02096
H +1.09577 +2.01036 +3.40296
H −0.40763 +1.15179 +3.02219

C	+1.55351	+0.55967	+5.81488
H	−0.27481	+1.64104	+5.44742
H	−0.32479	−0.10119	+5.12738
N	+2.18062	+1.67114	+6.27079
O	+1.97719	−0.55997	+6.02162
H	+3.10084	+1.56495	+6.66341
H	+1.90212	+2.58294	+5.95648

20

∗ E = +4.617 kcal/mol ; (65) 180_025_288_313_313_242

H	+0.00000	+0.00000	+0.00000
O	+0.00000	+0.00000	+0.96347
C	+1.27459	+0.00000	+1.36672
C	+1.41716	−0.00911	+2.88476
N	+0.29322	−0.56480	+3.61881
H	−0.49751	+0.06503	+3.57785
O	+2.19733	+0.06676	+0.60028
C	+2.76512	−0.62459	+3.30226
H	+1.47723	+1.05466	+3.15537
H	+0.00097	−1.43573	+3.18960
C	+3.17976	−1.94137	+2.62672
H	+3.55933	+0.09810	+3.09413
H	+2.71274	−0.75532	+4.38712
C	+2.08601	−2.98443	+2.58699
H	+3.43844	−1.73639	+1.58361
H	+4.07395	−2.32963	+3.12413
N	+2.32088	−4.13676	+3.25445
O	+1.05330	−2.79748	+1.96886
H	+1.61430	−4.85258	+3.24682
H	+3.16458	−4.28502	+3.77514

20

∗ E = +4.658 kcal/mol ; (66) 184_070_299_061_065_192

H	+0.00000	+0.00000	+0.00000
O	+0.00000	+0.00000	+0.96378
C	+1.27746	+0.00000	+1.38031
C	+1.37186	−0.09806	+2.89677
N	+0.96974	−1.43163	+3.33570
H	+0.01219	−1.63125	+3.07418
O	+2.20038	+0.01650	+0.61212
C	+2.78153	+0.20020	+3.38754
H	+0.67194	+0.62339	+3.33254

H +1.56461 −2.14111 +2.92199
C +3.27345 +1.60168 +3.05096
H +2.77673 +0.05885 +4.47143
H +3.46957 −0.53263 +2.95024
C +2.46812 +2.68671 +3.74814
H +4.31479 +1.71614 +3.37505
H +3.26854 +1.77059 +1.96838
N +2.69829 +3.94626 +3.29599
O +1.69074 +2.45510 +4.64829
H +2.22834 +4.71438 +3.74261
H +3.34160 +4.13094 +2.54908
20
∗ E = +4.671 kcal/mol ; (67) 181_027_056_282_053_099
H +0.00000 +0.00000 +0.00000
O +0.00000 +0.00000 +0.96363
C +1.26812 +0.00000 +1.36126
C +1.43541 −0.01469 +2.87687
N +0.29753 −0.63683 +3.52102
H +0.15537 −1.57355 +3.15621
O +2.19383 +0.05955 +0.59453
C +2.82394 −0.56944 +3.24272
H +1.43539 +1.05231 +3.14877
H +0.42551 −0.68361 +4.52326
C +2.92787 −2.10056 +3.16168
H +3.56767 −0.10323 +2.58886
H +3.05588 −0.26789 +4.26987
C +2.47098 −2.62182 +1.81419
H +3.95422 −2.41191 +3.37847
H +2.27388 −2.55647 +3.91059
N +3.44119 −2.90445 +0.91608
O +1.28797 −2.75956 +1.56210
H +3.15887 −3.07327 −0.03528
H +4.38814 −2.61536 +1.08168
20
∗ E = +4.671 kcal/mol ; (68) 174_164_029_309_093_236
H +0.00000 +0.00000 +0.00000
O +0.00000 +0.00000 +0.96393
C +1.26122 +0.00000 +1.39247
C +1.35156 +0.15790 +2.89349
N +2.67895 −0.23700 +3.31451

H	+3.35822	−0.06575	+2.58304
O	+2.20888	−0.03866	+0.65285
C	+0.96751	+1.61677	+3.23675
H	+0.59912	−0.50336	+3.34064
H	+2.96438	+0.23750	+4.15986
C	+1.73757	+2.64757	+2.40203
H	−0.10641	+1.77233	+3.08991
H	+1.17292	+1.75673	+4.30292
C	+0.95849	+3.04968	+1.15392
H	+1.86492	+3.57186	+2.97271
H	+2.73871	+2.27703	+2.15158
N	+1.60274	+2.89063	−0.02952
O	−0.15691	+3.51511	+1.23446
H	+1.10752	+3.13160	−0.87156
H	+2.42656	+2.31881	−0.09805

20

∗ E = +4.751 kcal/mol ; (69) 178_018_306_345_309_177

H	+0.00000	+0.00000	+0.00000
O	+0.00000	+0.00000	+0.96353
C	+1.27597	+0.00000	+1.36546
C	+1.42171	+0.04886	+2.88328
N	+0.23114	−0.31463	+3.62806
H	−0.55886	+0.24799	+3.33741
O	+2.18964	+0.03127	+0.58291
C	+2.64002	−0.76029	+3.40489
H	+1.61953	+1.11243	+3.07968
H	+0.00926	−1.28262	+3.41303
C	+3.30939	−1.75765	+2.46316
H	+3.40971	−0.07131	+3.76196
H	+2.27049	−1.29504	+4.28423
C	+2.32185	−2.71666	+1.83088
H	+3.85840	−1.25154	+1.66415
H	+4.03699	−2.34944	+3.03172
N	+2.84415	−3.57977	+0.92919
O	+1.14005	−2.72261	+2.12073
H	+2.22286	−4.20253	+0.44245
H	+3.79386	−3.49620	+0.61686

20

∗ E = +4.753 kcal/mol ; (70) 178_003_300_139_289_354

H	+0.00000	+0.00000	+0.00000

O	+0.00000	+0.00000	+0.96472
C	+1.26822	+0.00000	+1.37855
C	+1.43880	+0.03697	+2.89233
N	+0.23958	+0.00136	+3.69758
H	−0.36678	+0.78863	+3.50646
O	+2.20072	−0.00475	+0.61727
C	+2.41229	−1.07813	+3.32575
H	+1.93249	+1.00392	+3.06960
H	−0.29275	−0.84259	+3.52153
C	+3.36953	−0.60676	+4.41071
H	+1.81344	−1.91451	+3.70199
H	+2.96992	−1.45697	+2.46318
C	+4.43779	+0.39269	+3.98041
H	+2.81381	−0.13854	+5.23028
H	+3.90921	−1.45097	+4.85104
N	+4.53435	+0.67339	+2.65627
O	+5.17138	+0.89909	+4.80206
H	+5.24963	+1.32477	+2.38062
H	+3.92746	+0.29805	+1.94332

20

∗ E = +4.796 kcal/mol ; (71) 180_304_293_175_290_166

H	+0.00000	+0.00000	+0.00000
O	+0.00000	+0.00000	+0.96343
C	+1.28475	+0.00000	+1.37720
C	+1.37798	+0.00687	+2.89465
N	+0.66428	+1.12807	+3.49537
H	+1.10206	+2.01091	+3.26420
O	+2.20309	+0.00330	+0.61044
C	+0.82951	−1.31467	+3.44455
H	+2.44123	+0.08285	+3.13895
H	−0.29159	+1.15880	+3.15718
C	+0.99971	−1.40955	+4.95316
H	−0.23258	−1.38934	+3.18374
H	+1.35437	−2.14645	+2.96417
C	+2.45987	−1.56595	+5.34922
H	+0.58088	−0.52093	+5.43770
H	+0.45977	−2.27978	+5.34424
N	+2.71908	−1.36526	+6.66638
O	+3.32464	−1.87061	+4.55754
H	+3.66464	−1.46208	+6.99334

H	+2.00831	−1.05678	+7.30294

20

∗ E = +4.797 kcal/mol ; (72) 178_038_188_112_063_183

H	+0.00000	+0.00000	+0.00000
O	+0.00000	+0.00000	+0.96350
C	+1.27753	+0.00000	+1.37447
C	+1.39221	+0.05319	+2.88677
N	+0.35962	−0.75946	+3.50840
H	+0.39701	−0.59224	+4.50858
O	+2.19727	+0.01965	+0.60563
C	+2.78901	−0.38449	+3.35799
H	+1.26816	+1.13094	+3.11490
H	−0.55984	−0.49880	+3.17405
C	+3.58860	+0.75334	+3.97430
H	+2.65138	−1.17566	+4.09970
H	+3.34659	−0.80389	+2.51731
C	+2.91283	+1.28959	+5.22659
H	+4.59739	+0.41979	+4.24500
H	+3.71668	+1.57561	+3.25865
N	+3.52001	+2.35526	+5.80278
O	+1.89990	+0.79878	+5.68244
H	+3.12868	+2.74120	+6.64452
H	+4.36206	+2.75031	+5.42726

20

∗ E = +4.804 kcal/mol ; (73) 357_333_163_305_299_292

H	+0.00000	+0.00000	+0.00000
O	+0.00000	+0.00000	+0.97287
C	+1.23987	+0.00000	+1.44519
C	+2.35498	−0.08399	+0.39625
N	+1.87828	−0.75922	−0.79892
H	+2.47815	−0.58856	−1.59601
O	+1.48424	+0.10564	+2.61245
C	+2.79824	+1.34716	+0.05847
H	+3.19260	−0.58860	+0.89556
H	+1.79433	−1.75871	−0.66653
C	+1.66162	+2.27714	−0.39408
H	+3.26221	+1.77510	+0.95161
H	+3.57745	+1.29146	−0.71328
C	+0.94803	+1.81626	−1.65454
H	+0.90289	+2.36736	+0.38783

H	+2.07342	+3.27797	−0.56739
N	+1.69732	+1.86930	−2.79100
O	−0.19082	+1.40371	−1.65314
H	+1.24276	+1.65109	−3.66205
H	+2.55570	+2.38979	−2.82063

20

∗ E = +4.809 kcal/mol ; (74) 184_180_298_277_051_065

H	+0.00000	+0.00000	+0.00000
O	+0.00000	+0.00000	+0.96371
C	+1.28690	+0.00000	+1.36881
C	+1.41277	−0.09551	+2.89009
N	+2.78232	−0.09526	+3.34944
H	+3.27728	−0.89743	+2.97973
O	+2.20130	+0.02090	+0.59935
C	+0.58495	+0.98370	+3.61189
H	+0.95820	−1.06464	+3.13736
H	+3.27079	+0.72520	+2.99689
C	+1.30572	+2.33445	+3.70528
H	−0.38544	+1.08413	+3.11436
H	+0.39002	+0.63420	+4.62932
C	+1.84259	+2.81419	+2.36786
H	+0.62477	+3.08627	+4.11823
H	+2.16506	+2.25382	+4.37331
N	+0.90262	+3.10429	+1.43207
O	+3.03020	+2.90221	+2.14266
H	+1.20868	+3.34940	+0.50581
H	−0.07589	+2.96060	+1.60331

20

∗ E = +4.812 kcal/mol ; (75) 178_041_186_297_196_205

H	+0.00000	+0.00000	+0.00000
O	+0.00000	+0.00000	+0.96372
C	+1.27706	+0.00000	+1.37836
C	+1.38538	+0.04748	+2.89215
N	+0.37325	−0.81550	+3.48652
H	+0.37769	−0.71577	+4.49394
O	+2.20060	+0.01016	+0.61555
C	+2.78175	−0.35304	+3.35662
H	+1.23781	+1.11524	+3.13938
H	−0.55292	−0.58904	+3.14754
C	+3.14278	−1.78778	+2.99450

H	+3.51346	+0.33817	+2.92830
H	+2.83804	−0.24254	+4.44476
C	+4.34089	−2.27558	+3.78823
H	+2.30058	−2.44817	+3.23701
H	+3.33058	−1.88229	+1.91998
N	+5.05521	−3.26805	+3.19832
O	+4.62699	−1.83775	+4.88157
H	+5.83848	−3.65912	+3.69209
H	+4.83216	−3.60646	+2.28128

20

∗ E = +4.852 kcal/mol ; (76) 181_077_301_078_308_260

H	+0.00000	+0.00000	+0.00000
O	+0.00000	+0.00000	+0.96348
C	+1.27005	+0.00000	+1.38066
C	+1.36276	−0.03702	+2.90141
N	+1.08454	−1.39262	+3.37645
H	+0.16110	−1.69237	+3.08616
O	+2.20503	−0.06293	+0.62825
C	+2.74395	+0.40091	+3.37355
H	+0.59178	+0.63287	+3.29760
H	+1.75597	−2.04715	+2.98869
C	+2.95441	+1.92205	+3.34897
H	+2.87006	+0.04240	+4.39883
H	+3.49788	−0.09549	+2.75064
C	+2.59542	+2.51636	+2.00095
H	+2.29066	+2.39219	+4.08035
H	+3.98651	+2.15945	+3.62440
N	+3.62865	+2.78663	+1.16879
O	+1.43726	+2.71684	+1.69268
H	+3.40873	+3.00708	+0.21169
H	+4.55183	+2.45115	+1.37496

20

∗ E = +4.914 kcal/mol ; (77) 173_156_033_063_275_167

H	+0.00000	+0.00000	+0.00000
O	+0.00000	+0.00000	+0.96435
C	+1.28088	+0.00000	+1.36883
C	+1.40080	+0.16812	+2.87047
N	+2.67464	−0.38633	+3.27965
H	+3.36162	−0.24121	+2.54762
O	+2.20327	−0.11608	+0.61735

C	+1.21755	+1.64973	+3.26090
H	+0.57721	−0.40534	+3.32024
H	+3.00957	+0.04601	+4.13026
C	−0.15474	+2.23365	+2.94411
H	+1.39538	+1.70916	+4.34020
H	+1.98913	+2.24587	+2.76142
C	−0.25576	+2.73917	+1.51194
H	−0.94946	+1.49922	+3.12582
H	−0.36029	+3.09011	+3.59711
N	−1.51555	+3.00906	+1.08848
O	+0.71634	+2.91451	+0.81145
H	−1.64585	+3.38493	+0.16541
H	−2.31785	+2.82799	+1.66218

20

∗ E = +4.927 kcal/mol ; (78) 177_163_030_304_196_204

H	+0.00000	+0.00000	+0.00000
O	+0.00000	+0.00000	+0.96383
C	+1.27316	+0.00000	+1.37982
C	+1.37780	+0.06263	+2.88740
N	+2.71301	−0.34193	+3.27244
H	+3.38486	−0.10371	+2.55264
O	+2.20457	+0.01200	+0.62434
C	+0.97350	+1.48320	+3.33421
H	+0.63863	−0.63892	+3.29391
H	+2.99307	+0.07526	+4.14990
C	+1.80356	+2.56638	+2.65940
H	−0.09130	+1.64169	+3.13415
H	+1.10645	+1.55817	+4.41834
C	+1.67087	+3.90379	+3.36880
H	+2.86741	+2.29253	+2.68421
H	+1.53879	+2.67256	+1.60034
N	+1.93512	+4.99040	+2.59972
O	+1.37485	+3.99422	+4.53926
H	+1.92272	+5.89962	+3.02853
H	+2.19663	+4.90656	+1.63521

20

∗ E = +5.128 kcal/mol ; (79) 358_010_102_166_162_134

H	+0.00000	+0.00000	+0.00000
O	+0.00000	+0.00000	+0.97219
C	+1.27124	+0.00000	+1.35462

C	+2.28887	−0.05706	+0.20163
N	+1.61811	+0.13389	−1.08419
H	+1.74429	+1.07834	−1.43057
O	+1.60817	+0.01341	+2.50198
C	+3.42404	+0.93143	+0.46071
H	+2.68548	−1.08092	+0.23516
H	+1.94031	−0.50684	−1.79474
C	+4.62209	+0.68698	−0.45114
H	+3.05725	+1.95193	+0.29213
H	+3.71568	+0.86575	+1.51241
C	+5.53346	+1.90280	−0.49499
H	+5.16608	−0.21458	−0.14806
H	+4.28163	+0.52932	−1.48318
N	+6.86043	+1.65417	−0.37370
O	+5.09055	+3.01811	−0.65613
H	+7.49920	+2.43076	−0.39691
H	+7.21438	+0.73523	−0.18599

20

∗ E = +5.136 kcal/mol ; (80) 184_329_291_168_109_292

H	+0.00000	+0.00000	+0.00000
O	+0.00000	+0.00000	+0.96406
C	+1.27781	+0.00000	+1.38012
C	+1.39706	−0.08650	+2.89543
N	+0.30159	+0.52521	+3.63417
H	+0.31054	+1.53293	+3.53162
O	+2.20621	+0.01895	+0.62549
C	+1.54540	−1.56914	+3.26858
H	+2.32938	+0.42705	+3.15309
H	−0.58388	+0.19843	+3.25859
C	+1.96673	−1.77830	+4.73749
H	+0.59445	−2.07834	+3.06295
H	+2.29037	−2.01234	+2.60183
C	+0.85877	−2.34008	+5.61709
H	+2.79264	−2.48702	+4.80168
H	+2.30383	−0.82458	+5.16147
N	−0.17297	−1.49110	+5.83389
O	+0.90259	−3.46310	+6.07157
H	−0.94327	−1.80287	+6.39902
H	−0.16487	−0.56942	+5.42155

20

* E = +5.215 kcal/mol ; (81) 176_157_057_186_310_278
H +0.00000 +0.00000 +0.00000
O +0.00000 +0.00000 +0.96391
C +1.27961 +0.00000 +1.37422
C +1.39392 +0.08644 +2.88076
N +2.66609 −0.45392 +3.28679
H +3.44033 +0.02543 +2.84488
O +2.20460 +0.00126 +0.61517
C +1.12414 +1.56623 +3.25195
H +0.59922 −0.53040 +3.31849
H +2.75913 −0.45486 +4.29492
C +1.35803 +1.84678 +4.74217
H +1.78972 +2.20991 +2.66378
H +0.09436 +1.81764 +2.97054
C +0.62400 +0.83458 +5.59982
H +2.41973 +1.75457 +4.98116
H +1.04430 +2.86774 +4.98254
N −0.68955 +1.09160 +5.81429
O +1.16609 −0.16962 +6.01269
H −1.23577 +0.42627 +6.33438
H −1.12651 +1.93179 +5.48366
20
* E = +5.219 kcal/mol ; (82) 178_035_196_298_252_072
H +0.00000 +0.00000 +0.00000
O +0.00000 +0.00000 +0.96401
C +1.27725 +0.00000 +1.37958
C +1.39394 +0.05184 +2.89429
N +0.30085 −0.69130 +3.51764
H +0.23692 −0.43979 +4.49760
O +2.20331 +0.00723 +0.62313
C +2.75876 −0.47628 +3.33031
H +1.34913 +1.13029 +3.12977
H −0.58536 −0.46431 +3.08237
C +3.00978 −1.94835 +2.93914
H +3.52179 +0.15924 +2.87523
H +2.84651 −0.35000 +4.41589
C +2.97686 −2.89379 +4.12958
H +2.26350 −2.27418 +2.20474
H +3.99263 −2.05831 +2.48158
N +1.74195 −3.10713 +4.64547

O	+3.98012	−3.39828	+4.58671
H	+1.64886	−3.71519	+5.44011
H	+0.93934	−2.64863	+4.24091

20

∗ E = +5.233 kcal/mol ; (83) 181_335_047_289_283_148

H	+0.00000	+0.00000	+0.00000
O	+0.00000	+0.00000	+0.96408
C	+1.28295	+0.00000	+1.36897
C	+1.44204	−0.01263	+2.87671
N	+0.28218	+0.55737	+3.53768
H	−0.58119	+0.18934	+3.15607
O	+2.19592	−0.04737	+0.59529
C	+1.81524	−1.45011	+3.30225
H	+2.31401	+0.62175	+3.07158
H	+0.30050	+0.33970	+4.52729
C	+0.65997	−2.43535	+3.15307
H	+2.67625	−1.77657	+2.71095
H	+2.11656	−1.43100	+4.35463
C	−0.35534	−2.29647	+4.27857
H	+0.16542	−2.33297	+2.17903
H	+1.03959	−3.46211	+3.20887
N	−1.63906	−2.57205	+3.93887
O	−0.03818	−1.98207	+5.40571
H	−2.34036	−2.56888	+4.65980
H	−1.89179	−2.85567	+3.01062

20

∗ E = +5.306 kcal/mol ; (84) 182_346_287_289_257_037

H	+0.00000	+0.00000	+0.00000
O	+0.00000	+0.00000	+0.96449
C	+1.27848	+0.00000	+1.37655
C	+1.42327	−0.05560	+2.89514
N	+0.21073	+0.22906	+3.64823
H	−0.01853	+1.21444	+3.61121
O	+2.20352	+0.00972	+0.61725
C	+2.00169	−1.43050	+3.26687
H	+2.17553	+0.70003	+3.14332
H	−0.57977	−0.26268	+3.24258
C	+1.01392	−2.59726	+3.08376
H	+2.88642	−1.59385	+2.64590
H	+2.34134	−1.38362	+4.30616

C	+0.37414	−3.15243	+4.35375
H	+0.19397	−2.31522	+2.40672
H	+1.50242	−3.45308	+2.61580
N	+0.07434	−2.24852	+5.31642
O	+0.15145	−4.33835	+4.47227
H	−0.38332	−2.58596	+6.14566
H	+0.16562	−1.25470	+5.15518

20

∗ E = +5.352 kcal/mol ; (85) 184_324_297_171_172_170

H	+0.00000	+0.00000	+0.00000
O	+0.00000	+0.00000	+0.96379
C	+1.27856	+0.00000	+1.37738
C	+1.39578	−0.08759	+2.89228
N	+0.36500	+0.61257	+3.63712
H	+0.39432	+1.60991	+3.46733
O	+2.20444	+0.02559	+0.61800
C	+1.39305	−1.57387	+3.27048
H	+2.37146	+0.34518	+3.14044
H	−0.55267	+0.28389	+3.35701
C	+1.73539	−1.77506	+4.73721
H	+0.40260	−1.99771	+3.06631
H	+2.10056	−2.11872	+2.63736
C	+1.55542	−3.22106	+5.16323
H	+2.76674	−1.46144	+4.94190
H	+1.08786	−1.14404	+5.35766
N	+2.05109	−3.52014	+6.39249
O	+1.00516	−4.04652	+4.46923
H	+1.92437	−4.45055	+6.75083
H	+2.48103	−2.82512	+6.97366

20

∗ E = +5.358 kcal/mol ; (86) 007_343_149_308_097_243

H	+0.00000	+0.00000	+0.00000
O	+0.00000	+0.00000	+0.97386
C	+1.28575	+0.00000	+1.35156
C	+2.27820	+0.16209	+0.19429
N	+1.60825	−0.13478	−1.07307
H	+1.99591	+0.40872	−1.83353
O	+1.62078	−0.05170	+2.49493
C	+2.79035	+1.60978	+0.20483
H	+3.11920	−0.50861	+0.40325

H +1.68371 −1.11293 −1.32213
C +1.66519 +2.66073 +0.22499
H +3.42773 +1.76201 +1.08071
H +3.42471 +1.74160 −0.67869
C +1.39539 +3.14006 +1.64908
H +1.98554 +3.55074 −0.32228
H +0.75769 +2.28705 −0.26232
N +0.16152 +2.88591 +2.15126
O +2.24863 +3.74326 +2.26049
H +0.02863 +3.07969 +3.13082
H −0.40936 +2.16384 +1.74412
20
* E = +5.376 kcal/mol ; (87) 182_187_296_299_289_287
H +0.00000 +0.00000 +0.00000
O +0.00000 +0.00000 +0.96405
C +1.28203 +0.00000 +1.37440
C +1.40507 −0.04460 +2.89647
N +2.76515 +0.12037 +3.35758
H +3.33861 −0.65020 +3.03739
O +2.20230 +0.01218 +0.61093
C +0.44951 +0.95830 +3.55614
H +1.06135 −1.04809 +3.18408
H +3.17609 +0.96288 +2.96389
C +0.71070 +2.41935 +3.16335
H −0.57628 +0.70055 +3.28036
H +0.54516 +0.83137 +4.63996
C +1.98669 +2.99357 +3.75788
H +0.79048 +2.51919 +2.07644
H −0.13502 +3.03461 +3.48979
N +1.93069 +3.26215 +5.08621
O +2.98020 +3.20353 +3.09495
H +2.77864 +3.53493 +5.55300
H +1.14062 +2.99219 +5.64273
20
* E = +5.444 kcal/mol ; (88) 179_042_196_168_068_077
H +0.00000 +0.00000 +0.00000
O +0.00000 +0.00000 +0.96361
C +1.27761 +0.00000 +1.37608
C +1.38856 +0.02626 +2.89194
N +0.40111 −0.87253 +3.47160

H	+0.28105	−0.68491	+4.46084
O	+2.19812	+0.01613	+0.61047
C	+2.81221	−0.34731	+3.29766
H	+1.21800	+1.08632	+3.16580
H	−0.49674	−0.75744	+3.01921
C	+3.12489	−0.07141	+4.77355
H	+2.96479	−1.40705	+3.05942
H	+3.50760	+0.22287	+2.67586
C	+2.35819	−0.96414	+5.73248
H	+4.19892	−0.20489	+4.94314
H	+2.87040	+0.96114	+5.02814
N	+2.83504	−2.22607	+5.85316
O	+1.38189	−0.57766	+6.34412
H	+2.29670	−2.89204	+6.38057
H	+3.57404	−2.56307	+5.26361

20

* E = +5.447 kcal/mol ; (89) 173_151_292_059_281_339

H	+0.00000	+0.00000	+0.00000
O	+0.00000	+0.00000	+0.96487
C	+1.26031	+0.00000	+1.40407
C	+1.34400	+0.17159	+2.91346
N	+2.51064	−0.46666	+3.48728
H	+2.44267	−1.47498	+3.42148
O	+2.21650	−0.07407	+0.68147
C	+1.37942	+1.67265	+3.24811
H	+0.43721	−0.25777	+3.35404
H	+3.33684	−0.19650	+2.96265
C	+0.17620	+2.48674	+2.77383
H	+1.44760	+1.72991	+4.33797
H	+2.32626	+2.09131	+2.87871
C	+0.08274	+2.90754	+1.31258
H	−0.75768	+1.95756	+2.98711
H	+0.13045	+3.42361	+3.34033
N	+1.23665	+2.94205	+0.59749
O	−0.97609	+3.24994	+0.83478
H	+1.17511	+3.21560	−0.36851
H	+2.09959	+2.54750	+0.92398

20

* E = +5.580 kcal/mol ; (90) 356_018_091_171_176_018

H	+0.00000	+0.00000	+0.00000

O	+0.00000	+0.00000	+0.97162
C	+1.26905	+0.00000	+1.36125
C	+2.28856	−0.09589	+0.21372
N	+1.64903	+0.24901	−1.05327
H	+1.73711	+1.23890	−1.25563
O	+1.59790	+0.04184	+2.51038
C	+3.53909	+0.71116	+0.56001
H	+2.55880	−1.16003	+0.16634
H	+2.03993	−0.26133	−1.83286
C	+4.66594	+0.47245	−0.43558
H	+3.27018	+1.77676	+0.58900
H	+3.83173	+0.42939	+1.57718
C	+5.93151	+1.29736	−0.25256
H	+4.97554	−0.58033	−0.40902
H	+4.33815	+0.66735	−1.46163
N	+6.10871	+1.87365	+0.96210
O	+6.73678	+1.41233	−1.14906
H	+6.95130	+2.40008	+1.11740
H	+5.45032	+1.77495	+1.71211

20

∗ E = +5.591 kcal/mol ; (91) 179_149_299_059_172_182

H	+0.00000	+0.00000	+0.00000
O	+0.00000	+0.00000	+0.96456
C	+1.28002	+0.00000	+1.37683
C	+1.41110	+0.03538	+2.89440
N	+2.60085	−0.64311	+3.36438
H	+2.58846	−1.62319	+3.10939
O	+2.20276	−0.00798	+0.61277
C	+1.45052	+1.49406	+3.36562
H	+0.52461	−0.45269	+3.31722
H	+3.41942	−0.23638	+2.92324
C	+0.22487	+2.30718	+2.97880
H	+1.56369	+1.48596	+4.45264
H	+2.35378	+1.97186	+2.96790
C	+0.24286	+3.68068	+3.63124
H	+0.15833	+2.44672	+1.89304
H	−0.69628	+1.78667	+3.27195
N	−0.84059	+4.45363	+3.35654
O	+1.14912	+4.06136	+4.33600
H	−0.88950	+5.37566	+3.75347

H −1.59032 +4.12972 +2.77464
20
* E = +5.594 kcal/mol ; (92) 185_081_077_064_067_193
H +0.00000 +0.00000 +0.00000
O +0.00000 +0.00000 +0.96382
C +1.27016 +0.00000 +1.37655
C +1.39512 −0.11095 +2.88979
N +1.24241 −1.52790 +3.20484
H +2.09514 −2.03206 +2.98881
O +2.19963 +0.05778 +0.61745
C +2.70983 +0.50468 +3.36480
H +0.54901 +0.42187 +3.33609
H +1.04917 −1.65150 +4.19018
C +2.80370 +2.00171 +3.10240
H +2.79533 +0.32094 +4.44019
H +3.53599 −0.00606 +2.85815
C +1.81248 +2.79029 +3.94534
H +3.80625 +2.36969 +3.35078
H +2.65267 +2.22322 +2.03873
N +1.64990 +4.08588 +3.57725
O +1.22364 +2.30560 +4.88747
H +1.03203 +4.66889 +4.11471
H +2.13318 +4.47676 +2.79014
20
* E = +5.621 kcal/mol ; (93) 183_325_304_061_180_184
H +0.00000 +0.00000 +0.00000
O +0.00000 +0.00000 +0.96382
C +1.27716 +0.00000 +1.37766
C +1.39961 −0.07763 +2.89337
N +0.37135 +0.62544 +3.63381
H +0.32296 +1.60225 +3.37263
O +2.20234 +0.01481 +0.61354
C +1.39347 −1.55628 +3.30047
H +2.37718 +0.35605 +3.13384
H −0.53832 +0.21714 +3.45078
C +2.55069 −2.35087 +2.71632
H +1.42255 −1.59693 +4.39283
H +0.44197 −2.01315 +3.00228
C +2.49868 −3.80624 +3.14980
H +2.55032 −2.31563 +1.61994

H	+3.51086	−1.92065	+3.02806
N	+3.54698	−4.56151	+2.73026
O	+1.59496	−4.26172	+3.81362
H	+3.56445	−5.53817	+2.96658
H	+4.28170	−4.18012	+2.16393

20

∗ E = +5.640 kcal/mol ; (94) 182_322_068_183_310_277

H	+0.00000	+0.00000	+0.00000
O	+0.00000	+0.00000	+0.96359
C	+1.27684	+0.00000	+1.37613
C	+1.38952	−0.05128	+2.88577
N	+0.35658	+0.74220	+3.51612
H	−0.56420	+0.34291	+3.38103
O	+2.20303	−0.03177	+0.61670
C	+1.42272	−1.54824	+3.27214
H	+2.35821	+0.39624	+3.13233
H	+0.54993	+0.84203	+4.50637
C	+1.46185	−1.75405	+4.79235
H	+0.53048	−2.04421	+2.87024
H	+2.29513	−2.01281	+2.79677
C	+2.55385	−0.90768	+5.41775
H	+0.51856	−1.43453	+5.24071
H	+1.60519	−2.81466	+5.02232
N	+3.80094	−1.43501	+5.37410
O	+2.32926	+0.19520	+5.87291
H	+4.57227	−0.88628	+5.71402
H	+3.97936	−2.34429	+4.98985

20

∗ E = +5.646 kcal/mol ; (95) 176_024_183_131_294_345

H	+0.00000	+0.00000	+0.00000
O	+0.00000	+0.00000	+0.96425
C	+1.26534	+0.00000	+1.38192
C	+1.40684	+0.08491	+2.88965
N	+0.21520	−0.40647	+3.56153
H	+0.30833	−0.28979	+4.56315
O	+2.19846	+0.00613	+0.62198
C	+2.65720	−0.67353	+3.37193
H	+1.58027	+1.16722	+3.05386
H	−0.61369	+0.09363	+3.26455
C	+3.51384	+0.14124	+4.33331

H +2.31112 −1.58866 +3.86203
H +3.25576 −0.99474 +2.51384
C +4.19464 +1.37878 +3.75903
H +2.92974 +0.49120 +5.19167
H +4.31521 −0.47848 +4.74954
N +4.23955 +1.48645 +2.40926
O +4.68660 +2.20778 +4.49493
H +4.70074 +2.29582 +2.02963
H +3.78900 +0.84581 +1.77339
20
* E = +5.670 kcal/mol ; (96) 184_068_306_064_173_160
H +0.00000 +0.00000 +0.00000
O +0.00000 +0.00000 +0.96382
C +1.27791 +0.00000 +1.38205
C +1.36480 −0.09377 +2.90115
N +0.91084 −1.40406 +3.35578
H −0.01711 −1.61460 +3.00907
O +2.20249 +0.01983 +0.61850
C +2.77784 +0.16995 +3.39253
H +0.67723 +0.65690 +3.31340
H +1.54121 −2.13022 +3.03424
C +3.25878 +1.58233 +3.09420
H +2.80845 −0.00773 +4.47025
H +3.46079 −0.55242 +2.92933
C +4.60029 +1.86269 +3.75210
H +3.32996 +1.75292 +2.01469
H +2.54623 +2.31865 +3.49127
N +5.28994 +2.90553 +3.22089
O +5.01222 +1.22748 +4.69615
H +6.18265 +3.14402 +3.61668
H +4.97797 +3.37924 +2.39403
20
* E = +5.689 kcal/mol ; (97) 187_320_288_292_082_195
H +0.00000 +0.00000 +0.00000
O +0.00000 +0.00000 +0.96399
C +1.28700 +0.00000 +1.36942
C +1.40306 −0.17396 +2.87750
N +0.40680 +0.56634 +3.64102
H +0.60429 +1.55895 +3.62003
O +2.20376 +0.12563 +0.61377

C	+1.37170	−1.66499	+3.25015
H	+2.39407	+0.20722	+3.13829
H	−0.51465	+0.44517	+3.23709
C	+0.03513	−2.35634	+3.00210
H	+2.15100	−2.19348	+2.69229
H	+1.60960	−1.71779	+4.31586
C	−0.14926	−2.78814	+1.55490
H	−0.02271	−3.26870	+3.60802
H	−0.80876	−1.73191	+3.31892
N	−1.42229	−3.10737	+1.21048
O	+0.77320	−2.86994	+0.77456
H	−1.59915	−3.46248	+0.28694
H	−2.18119	−3.03615	+1.86203

20

∗ E = +5.691 kcal/mol ; (98) 184_194_311_300_149_296

H	+0.00000	+0.00000	+0.00000
O	+0.00000	+0.00000	+0.96495
C	+1.29084	+0.00000	+1.36629
C	+1.44815	−0.09927	+2.88342
N	+2.78583	+0.22943	+3.32208
H	+3.47313	−0.27140	+2.77070
O	+2.19527	+0.05741	+0.58619
C	+0.40466	+0.76215	+3.59203
H	+1.24999	−1.15266	+3.12865
H	+2.97054	+1.21653	+3.17752
C	+0.51773	+2.24835	+3.20597
H	−0.59025	+0.38171	+3.34662
H	+0.53889	+0.64372	+4.66998
C	−0.82227	+2.96569	+3.24599
H	+1.19325	+2.78748	+3.87210
H	+0.91103	+2.34502	+2.18290
N	−1.72616	+2.49102	+2.34820
O	−1.06258	+3.87897	+4.00118
H	−2.64947	+2.88757	+2.33425
H	−1.51151	+1.70653	+1.75660

20

∗ E = +5.726 kcal/mol ; (99) 016_203_317_306_119_153

H	+0.00000	+0.00000	+0.00000
O	+0.00000	+0.00000	+0.97295
C	+1.20025	+0.00000	+1.54487

C	+2.42635	+0.38319	+0.69108
N	+3.65783	−0.15034	+1.24663
H	+3.64769	−0.01595	+2.25274
O	+1.32419	−0.23681	+2.71341
C	+2.30851	+0.02472	−0.78768
H	+2.47888	+1.47904	+0.76279
H	+3.69163	−1.15578	+1.10809
C	+1.98226	−1.45603	−1.04777
H	+1.54049	+0.64404	−1.26089
H	+3.25990	+0.28843	−1.25555
C	+0.66078	−1.61904	−1.78222
H	+2.78057	−1.96309	−1.59690
H	+1.86454	−2.00369	−0.10014
N	+0.53119	−2.72540	−2.53842
O	−0.23677	−0.80273	−1.66886
H	−0.35046	−2.90481	−2.98871
H	+1.27305	−3.39450	−2.62880

20

∗ E = +5.742 kcal/mol ; (100) 184_202_200_299_151_293

H	+0.00000	+0.00000	+0.00000
O	+0.00000	+0.00000	+0.96467
C	+1.28882	+0.00000	+1.36205
C	+1.45051	−0.10870	+2.86770
N	+2.75710	+0.41582	+3.20821
H	+3.06397	+0.08659	+4.11316
O	+2.19411	+0.04624	+0.58185
C	+0.34314	+0.63470	+3.61293
H	+1.33885	−1.19006	+3.07339
H	+3.44405	+0.15966	+2.50942
C	+0.33311	+2.13762	+3.28902
H	−0.61815	+0.17572	+3.36240
H	+0.48597	+0.48754	+4.68821
C	−1.06014	+2.73048	+3.39717
H	+0.99244	+2.69131	+3.95596
H	+0.69944	+2.30120	+2.26659
N	−1.92849	+2.26814	+2.45671
O	−1.37854	+3.53775	+4.24031
H	−2.88554	+2.57298	+2.48733
H	−1.65114	+1.56033	+1.79870

20

* E =	+5.754 kcal/mol ;	(101) 161_141_289_048_280_299	
H	+0.00000	+0.00000	+0.00000
O	+0.00000	+0.00000	+0.96640
C	+1.25988	+0.00000	+1.39846
C	+1.37619	+0.44752	+2.84782
N	+2.35902	−0.30030	+3.60704
H	+2.04271	−1.24657	+3.78032
O	+2.21127	−0.24514	+0.70851
C	+1.78853	+1.92936	+2.82899
H	+0.39362	+0.35197	+3.32121
H	+3.21518	−0.36860	+3.06481
C	+0.96860	+2.81376	+1.87558
H	+1.71124	+2.30052	+3.85403
H	+2.85369	+1.96143	+2.56387
C	+1.38082	+2.73164	+0.40811
H	−0.09249	+2.55505	+1.91887
H	+1.06914	+3.86058	+2.18251
N	+2.65294	+3.12023	+0.14480
O	+0.62152	+2.37869	−0.46990
H	+3.00454	+2.98288	−0.78739
H	+3.30909	+3.30894	+0.87988
20			
* E =	+5.785 kcal/mol ;	(102) 181_054_179_068_166_148	
H	+0.00000	+0.00000	+0.00000
O	+0.00000	+0.00000	+0.96374
C	+1.27622	+0.00000	+1.38160
C	+1.35864	−0.02936	+2.89849
N	+0.56646	−1.16388	+3.35830
H	+0.60041	−1.22887	+4.36777
O	+2.20150	+0.03290	+0.62194
C	+2.79546	−0.16374	+3.37914
H	+0.95737	+0.94523	+3.23718
H	−0.40152	−1.07600	+3.07555
C	+3.64677	+1.06905	+3.10306
H	+2.78939	−0.33903	+4.46048
H	+3.24264	−1.04821	+2.91391
C	+4.94858	+1.02100	+3.88620
H	+3.83625	+1.18286	+2.03230
H	+3.11836	+1.97271	+3.43719
N	+6.01134	+1.60248	+3.27497

O +5.02491 +0.53005 +4.99090
H +6.89647 +1.61086 +3.75169
H +5.95691 +1.94274 +2.33338
20
∗ E = +5.901 kcal/mol ; (103) 176_157_046_180_293_164
H +0.00000 +0.00000 +0.00000
O +0.00000 +0.00000 +0.96380
C +1.27265 +0.00000 +1.37623
C +1.39171 +0.08716 +2.88208
N +2.68674 −0.44659 +3.24919
H +3.40956 −0.07758 +2.64178
O +2.20391 −0.00697 +0.62092
C +1.13040 +1.56333 +3.25978
H +0.59453 −0.52131 +3.32430
H +2.92367 −0.25782 +4.21260
C +1.20487 +1.81310 +4.75910
H +1.86905 +2.19182 +2.74782
H +0.13405 +1.84412 +2.90354
C +0.08212 +1.11471 +5.51396
H +2.17597 +1.51251 +5.17171
H +1.11295 +2.88667 +4.96336
N +0.27597 +0.99736 +6.85269
O −0.91610 +0.70775 +4.96417
H −0.45817 +0.59989 +7.41310
H +1.09109 +1.36654 +7.30548
20
∗ E = +5.902 kcal/mol ; (104) 178_149_042_062_164_159
H +0.00000 +0.00000 +0.00000
O +0.00000 +0.00000 +0.96440
C +1.27752 +0.00000 +1.37472
C +1.40804 +0.04856 +2.88274
N +2.61268 −0.67428 +3.24097
H +3.35716 −0.44052 +2.59268
O +2.20222 +0.00659 +0.61307
C +1.40042 +1.51924 +3.33960
H +0.53025 −0.45480 +3.30833
H +2.91386 −0.43113 +4.17601
C +0.11417 +2.26810 +3.01872
H +1.54823 +1.53880 +4.42445
H +2.26351 +2.03011 +2.89594

C	+0.01032	+3.56230	+3.81196
H	+0.02280	+2.47933	+1.94775
H	−0.75646	+1.65193	+3.28230
N	−0.83599	+4.48638	+3.29009
O	+0.61652	+3.74806	+4.84292
H	−0.98365	+5.34441	+3.79269
H	−1.33358	+4.32707	+2.43428

20

∗ E = +5.921 kcal/mol ; (105) 358_169_307_181_304_104

H	+0.00000	+0.00000	+0.00000
O	+0.00000	+0.00000	+0.96122
C	+1.25044	+0.00000	+1.45133
C	+2.36655	−0.04790	+0.40741
N	+3.67941	+0.20112	+0.97022
H	+3.66955	+1.06926	+1.49489
O	+1.44998	+0.02420	+2.62717
C	+2.34232	−1.40874	−0.30616
H	+2.15732	+0.73698	−0.33661
H	+3.88900	−0.52096	+1.65393
C	+3.45382	−1.51886	−1.35326
H	+2.44956	−2.20626	+0.43918
H	+1.37558	−1.55633	−0.79972
C	+3.33331	−0.36066	−2.32833
H	+4.43272	−1.52280	−0.86841
H	+3.33673	−2.44510	−1.92012
N	+4.24249	+0.62951	−2.15448
O	+2.45826	−0.32233	−3.16784
H	+4.14715	+1.46478	−2.70773
H	+4.77876	+0.66122	−1.30205

20

∗ E = +5.986 kcal/mol ; (106) 181_066_182_083_309_260

H	+0.00000	+0.00000	+0.00000
O	+0.00000	+0.00000	+0.96356
C	+1.27271	+0.00000	+1.38065
C	+1.34681	−0.01803	+2.90064
N	+0.80277	−1.31069	+3.31974
H	+0.79234	−1.37354	+4.33027
O	+2.20496	−0.02665	+0.62911
C	+2.79085	+0.12163	+3.36930
H	+0.75528	+0.84394	+3.24941

H −0.15194 −1.41445 +2.99624
C +3.27992 +1.57706 +3.41783
H +2.87042 −0.30138 +4.37653
H +3.42733 −0.48292 +2.71546
C +3.00912 +2.31449 +2.11886
H +2.73341 +2.11956 +4.19505
H +4.34422 +1.60364 +3.67084
N +4.05839 +2.43653 +1.26958
O +1.90879 +2.76322 +1.86871
H +3.85439 +2.74345 +0.33265
H +4.88362 +1.88103 +1.40634
20
∗ E = +6.006 kcal/mol ; (107) 352_030_082_093_314_261
H +0.00000 +0.00000 +0.00000
O +0.00000 +0.00000 +0.96994
C +1.26960 +0.00000 +1.36351
C +2.28251 −0.18951 +0.22685
N +1.72286 +0.38247 −0.99628
H +1.86029 +1.38639 −1.03457
O +1.58907 +0.12673 +2.51016
C +3.66744 +0.32174 +0.65636
H +2.33195 −1.27838 +0.08568
H +2.14694 −0.01934 −1.82145
C +4.57017 −0.76935 +1.25815
H +4.17421 +0.73425 −0.22214
H +3.53905 +1.13738 +1.37583
C +3.84609 −1.63896 +2.27319
H +4.89386 −1.44812 +0.46365
H +5.46261 −0.31308 +1.69706
N +4.04856 −1.33530 +3.58041
O +3.15137 −2.56633 +1.91308
H +3.40428 −1.75393 +4.23262
H +4.38921 −0.41990 +3.81781
20
∗ E = +6.061 kcal/mol ; (108) 178_046_073_291_144_293
H +0.00000 +0.00000 +0.00000
O +0.00000 +0.00000 +0.96428
C +1.26375 +0.00000 +1.37418
C +1.40740 +0.04742 +2.89346
N +0.49771 −0.90269 +3.51119

H	+0.81507	−1.85613	+3.37793
O	+2.19107	−0.00149	+0.60406
C	+2.88315	−0.08482	+3.27190
H	+1.05559	+1.05190	+3.16848
H	+0.43437	−0.73789	+4.50734
C	+3.44675	−1.48793	+2.97636
H	+3.45392	+0.67253	+2.72493
H	+2.99305	+0.14284	+4.33654
C	+4.89413	−1.43850	+2.51036
H	+3.41139	−2.13073	+3.85704
H	+2.86084	−1.96713	+2.18085
N	+5.05523	−0.85172	+1.29967
O	+5.81187	−1.87930	+3.16705
H	+5.98450	−0.75070	+0.93114
H	+4.26353	−0.48208	+0.79566

20

∗ E = +6.063 kcal/mol ; (109) 188_335_183_055_278_161

H	+0.00000	+0.00000	+0.00000
O	+0.00000	+0.00000	+0.96420
C	+1.26526	+0.00000	+1.36842
C	+1.44283	−0.18290	+2.86417
N	+0.27438	+0.24621	+3.60663
H	+0.43202	+0.15928	+4.60233
O	+2.20034	+0.10807	+0.61828
C	+1.69753	−1.67134	+3.15524
H	+2.36491	+0.37406	+3.09922
H	+0.02294	+1.20578	+3.40716
C	+2.87021	−2.26134	+2.38174
H	+1.87657	−1.76290	+4.23221
H	+0.78559	−2.22841	+2.91892
C	+2.47777	−2.65742	+0.96329
H	+3.71756	−1.56609	+2.34975
H	+3.22324	−3.17464	+2.87408
N	+3.51417	−2.81410	+0.10105
O	+1.32797	−2.87570	+0.64921
H	+3.28583	−2.97675	−0.86534
H	+4.40447	−2.39763	+0.30554

20

∗ E = +6.073 kcal/mol ; (110) 179_046_298_290_180_198

H	+0.00000	+0.00000	+0.00000

O	+0.00000	+0.00000	+0.96367
C	+1.27944	+0.00000	+1.37767
C	+1.37985	+0.03600	+2.90126
N	+0.48941	−0.91041	+3.56031
H	−0.47806	−0.70226	+3.34694
O	+2.19936	−0.01762	+0.60902
C	+2.82400	−0.13267	+3.35812
H	+1.04521	+1.04240	+3.18685
H	+0.66526	−1.85725	+3.24540
C	+3.37210	−1.53267	+3.11602
H	+3.45466	+0.59368	+2.83773
H	+2.88193	+0.09257	+4.42538
C	+4.81208	−1.64606	+3.59006
H	+2.79637	−2.27696	+3.68199
H	+3.31119	−1.80069	+2.05466
N	+5.49874	−2.70539	+3.08701
O	+5.30336	−0.87156	+4.37912
H	+6.45864	−2.82849	+3.35887
H	+5.11539	−3.28985	+2.36810

20

∗ E = +6.178 kcal/mol ; (111) 182_320_059_061_172_168

H	+0.00000	+0.00000	+0.00000
O	+0.00000	+0.00000	+0.96364
C	+1.27514	+0.00000	+1.38064
C	+1.38339	−0.05289	+2.89023
N	+0.36714	+0.79407	+3.49202
H	−0.55909	+0.49051	+3.21337
O	+2.20348	−0.02957	+0.62275
C	+1.33199	−1.52763	+3.32822
H	+2.37206	+0.35478	+3.12936
H	+0.41971	+0.73901	+4.50176
C	+2.46524	−2.36783	+2.75834
H	+1.37546	−1.56395	+4.42163
H	+0.36196	−1.95528	+3.04641
C	+2.48271	−3.76299	+3.36094
H	+2.40543	−2.44055	+1.66634
H	+3.43309	−1.89474	+2.97242
N	+3.29873	−4.64533	+2.72933
O	+1.83111	−4.06433	+4.33581
H	+3.38293	−5.57638	+3.09854

H +3.83515 −4.38670 +1.92223
20
∗ E = +6.202 kcal/mol ; (112) 175_191_221_297_081_081
H +0.00000 +0.00000 +0.00000
O +0.00000 +0.00000 +0.96415
C +1.27699 +0.00000 +1.36770
C +1.39631 +0.13295 +2.87754
N +2.78075 +0.39259 +3.21955
H +3.07196 −0.12075 +4.03847
O +2.20332 −0.14276 +0.62481
C +0.46540 +1.21035 +3.45949
H +1.04062 −0.83900 +3.25957
H +3.38403 +0.14601 +2.44308
C +0.77704 +2.62609 +2.95192
H −0.57781 +0.94274 +3.25749
H +0.60207 +1.18962 +4.54549
C +0.18807 +2.89001 +1.57864
H +0.38593 +3.36361 +3.66077
H +1.86058 +2.75235 +2.88734
N −1.12951 +3.22454 +1.57597
O +0.82735 +2.78512 +0.55444
H −1.59142 +3.36661 +0.69476
H −1.65798 +3.30847 +2.42397
20
∗ E = +6.220 kcal/mol ; (113) 182_320_073_179_079_213
H +0.00000 +0.00000 +0.00000
O +0.00000 +0.00000 +0.96352
C +1.27298 +0.00000 +1.37957
C +1.37793 −0.05955 +2.89080
N +0.36747 +0.78496 +3.50387
H −0.55140 +0.36236 +3.42197
O +2.20549 −0.01875 +0.62709
C +1.33688 −1.54891 +3.28880
H +2.36543 +0.34641 +3.13631
H +0.55618 +0.92046 +4.48923
C +1.46259 −1.73858 +4.79579
H +0.38538 −1.98445 +2.96078
H +2.14603 −2.07597 +2.77298
C +0.15249 −1.46215 +5.52008
H +1.72329 −2.77913 +5.02157

H	+2.26726	−1.11374	+5.20451
N	+0.27836	−0.92785	+6.76355
O	−0.92642	−1.70806	+5.02925
H	−0.55012	−0.82705	+7.32550
H	+1.17515	−0.81416	+7.19894

20

∗ E = +6.350 kcal/mol ; (114) 177_155_052_177_072_068

H	+0.00000	+0.00000	+0.00000
O	+0.00000	+0.00000	+0.96395
C	+1.27649	+0.00000	+1.37730
C	+1.39172	+0.08516	+2.88530
N	+2.64986	−0.50195	+3.27566
H	+3.42001	−0.10159	+2.75419
O	+2.20552	+0.00064	+0.62223
C	+1.16738	+1.57017	+3.25418
H	+0.57128	−0.49893	+3.32103
H	+2.82306	−0.43216	+4.26994
C	+1.17350	+1.82079	+4.76657
H	+1.94334	+2.17100	+2.76027
H	+0.20251	+1.89003	+2.84483
C	+2.54998	+1.72206	+5.40390
H	+0.77204	+2.82022	+4.96949
H	+0.53395	+1.09557	+5.27659
N	+3.41861	+2.70112	+5.04860
O	+2.85756	+0.83202	+6.16873
H	+4.35077	+2.68078	+5.42488
H	+3.16027	+3.44243	+4.42398

20

∗ E = +6.414 kcal/mol ; (115) 353_159_020_309_295_122

H	+0.00000	+0.00000	+0.00000
O	+0.00000	+0.00000	+0.96069
C	+1.25051	+0.00000	+1.43939
C	+2.35311	−0.17122	+0.40355
N	+3.60066	+0.31904	+0.94288
H	+3.55722	+0.35377	+1.95468
O	+1.44981	+0.04877	+2.61858
C	+2.37365	−1.66403	−0.00269
H	+2.07515	+0.41530	−0.48772
H	+4.38208	−0.26607	+0.67482
C	+2.44092	−2.62026	+1.18937

H	+1.48865	−1.89784	−0.60633
H	+3.24991	−1.82551	−0.64049
C	+3.75300	−2.47266	+1.94573
H	+1.58018	−2.48739	+1.85354
H	+2.41499	−3.65050	+0.82199
N	+3.64484	−2.15917	+3.25795
O	+4.81823	−2.65220	+1.39126
H	+4.49448	−1.99690	+3.77281
H	+2.78425	−1.78225	+3.61945

20

∗ E = +6.417 kcal/mol ; (116) 180_334_299_305_287_108

H	+0.00000	+0.00000	+0.00000
O	+0.00000	+0.00000	+0.96491
C	+1.29391	+0.00000	+1.35881
C	+1.47056	+0.00150	+2.87434
N	+0.38843	+0.56607	+3.64997
H	+0.23737	+1.54046	+3.42414
O	+2.19231	−0.03811	+0.56908
C	+1.80217	−1.43840	+3.30856
H	+2.36868	+0.60302	+3.05012
H	−0.48562	+0.07604	+3.50594
C	+0.79736	−2.50555	+2.85526
H	+2.78340	−1.69463	+2.89895
H	+1.87186	−1.44684	+4.40055
C	−0.51554	−2.44341	+3.61941
H	+0.63086	−2.45131	+1.77296
H	+1.21891	−3.49082	+3.07070
N	−1.58118	−1.96300	+2.92210
O	−0.59704	−2.79190	+4.77437
H	−2.47379	−1.90965	+3.38285
H	−1.48372	−1.62267	+1.98191

20

∗ E = +6.420 kcal/mol ; (117) 173_147_042_062_280_340

H	+0.00000	+0.00000	+0.00000
O	+0.00000	+0.00000	+0.96481
C	+1.25941	+0.00000	+1.39913
C	+1.34948	+0.17606	+2.89954
N	+2.49665	−0.57486	+3.36534
H	+3.27793	−0.44323	+2.73214
O	+2.21503	−0.05857	+0.67428

C +1.41380 +1.68792 +3.21468
H +0.43001 −0.22763 +3.34055
H +2.77626 −0.28628 +4.29379
C +0.19792 +2.50444 +2.77635
H +1.51189 +1.76882 +4.30312
H +2.35099 +2.09105 +2.80747
C +0.07070 +2.93189 +1.31896
H −0.72900 +1.96991 +3.00762
H +0.15904 +3.43886 +3.34715
N +1.20100 +2.94066 +0.56622
O −0.99409 +3.30402 +0.87867
H +1.11127 +3.21803 −0.39660
H +2.06103 +2.51188 +0.85585
20
* E = +6.421 kcal/mol ; (118) 177_161_033_173_181_177
H +0.00000 +0.00000 +0.00000
O +0.00000 +0.00000 +0.96377
C +1.27319 +0.00000 +1.37869
C +1.37937 +0.08196 +2.88683
N +2.70247 −0.36795 +3.26946
H +3.37322 −0.11362 +2.55254
O +2.20541 −0.02246 +0.62658
C +1.01421 +1.51819 +3.30585
H +0.61891 −0.59787 +3.29769
H +2.99967 +0.02620 +4.15088
C +0.92372 +1.66337 +4.81671
H +1.76124 +2.21516 +2.90977
H +0.05306 +1.80019 +2.86430
C +0.53833 +3.07879 +5.22172
H +0.18305 +0.96438 +5.22688
H +1.87790 +1.42473 +5.30436
N +0.38630 +3.26780 +6.55835
O +0.38334 +3.96851 +4.41759
H +0.13026 +4.18121 +6.89070
H +0.50231 +2.52160 +7.21823
20
* E = +6.475 kcal/mol ; (119) 182_342_291_292_178_182
H +0.00000 +0.00000 +0.00000
O +0.00000 +0.00000 +0.96430
C +1.28452 +0.00000 +1.36745

C	+1.44187	−0.03792	+2.88545
N	+0.26799	+0.33154	+3.65320
H	+0.05790	+1.31591	+3.54827
O	+2.19751	−0.00644	+0.59259
C	+1.94190	−1.43570	+3.27543
H	+2.24457	+0.67185	+3.11012
H	−0.54865	−0.17481	+3.33244
C	+0.89672	−2.51662	+3.04134
H	+2.84652	−1.66932	+2.70705
H	+2.21628	−1.41966	+4.33299
C	+1.43247	−3.89443	+3.39963
H	+0.00350	−2.33381	+3.65246
H	+0.56542	−2.53324	+1.99370
N	+0.56929	−4.91861	+3.16914
O	+2.53802	−4.06845	+3.85724
H	+0.85879	−5.85431	+3.39471
H	−0.34812	−4.76623	+2.79425

20

∗ E = +6.566 kcal/mol ; (120) 182_196_199_076_160_152

H	+0.00000	+0.00000	+0.00000
O	+0.00000	+0.00000	+0.96418
C	+1.28172	+0.00000	+1.36590
C	+1.43333	−0.05915	+2.87406
N	+2.79082	+0.33596	+3.18673
H	+3.06231	+0.02828	+4.11006
O	+2.19347	+0.00590	+0.58951
C	+0.44303	+0.82564	+3.64025
H	+1.20866	−1.11475	+3.12749
H	+3.44340	−0.02369	+2.50135
C	−0.97855	+0.28479	+3.72926
H	+0.82331	+0.92695	+4.66196
H	+0.45250	+1.83400	+3.21262
C	−1.74772	+0.94065	+4.86646
H	−1.51968	+0.39634	+2.78666
H	−0.95514	−0.79147	+3.95080
N	−3.09208	+1.00303	+4.68769
O	−1.21007	+1.34933	+5.87123
H	−3.65959	+1.38733	+5.42313
H	−3.53000	+0.68492	+3.84381

20

∗ E = +6.571 kcal/mol ; (121) 177_162_045_155_291_347

H	+0.00000	+0.00000	+0.00000
O	+0.00000	+0.00000	+0.96396
C	+1.28098	+0.00000	+1.38748
C	+1.35069	+0.07078	+2.89391
N	+2.66605	−0.34439	+3.32346
H	+3.38119	+0.09089	+2.75170
O	+2.21275	−0.00215	+0.64009
C	+0.92347	+1.49930	+3.32265
H	+0.60913	−0.64310	+3.27802
H	+2.83148	−0.11890	+4.29559
C	+0.37351	+1.53118	+4.74192
H	+1.80502	+2.14464	+3.24488
H	+0.18233	+1.91275	+2.62762
C	−0.97054	+0.84710	+4.96855
H	+1.06964	+1.06966	+5.45123
H	+0.24452	+2.56363	+5.08337
N	−1.69126	+0.50878	+3.86808
O	−1.37887	+0.64923	+6.09108
H	−2.57264	+0.04840	+4.02009
H	−1.34168	+0.57571	+2.92795

20

∗ E = +6.592 kcal/mol ; (122) 181_349_064_141_291_352

H	+0.00000	+0.00000	+0.00000
O	+0.00000	+0.00000	+0.96466
C	+1.26342	+0.00000	+1.37776
C	+1.44613	−0.02654	+2.88110
N	+0.20787	+0.19751	+3.59547
H	−0.46404	−0.53965	+3.41783
O	+2.20057	−0.01299	+0.62077
C	+2.22920	−1.31573	+3.25029
H	+2.10029	+0.83319	+3.08208
H	+0.37227	+0.24589	+4.59296
C	+3.24852	−1.07107	+4.35386
H	+1.50186	−2.06883	+3.57196
H	+2.73283	−1.72722	+2.36836
C	+4.45218	−0.20822	+3.98472
H	+2.78555	−0.59081	+5.22394
H	+3.66053	−2.01637	+4.72122
N	+4.60684	+0.12267	+2.67947

O +5.23786 +0.13924 +4.84024
H +5.40331 +0.69194 +2.44737
H +3.93905 −0.08229 +1.95087
20
* E = +6.678 kcal/mol ; (123) 181_325_075_325_294_163
H +0.00000 +0.00000 +0.00000
O +0.00000 +0.00000 +0.96376
C +1.27612 +0.00000 +1.37499
C +1.39908 −0.03327 +2.88497
N +0.32982 +0.72629 +3.50619
H −0.54223 +0.20898 +3.46191
O +2.19948 −0.05620 +0.61112
C +1.55248 −1.52134 +3.31827
H +2.34367 +0.47198 +3.10606
H +0.53968 +0.86275 +4.48704
C +0.78095 −2.54892 +2.49173
H +2.61251 −1.78858 +3.26989
H +1.23771 −1.58950 +4.36443
C −0.72939 −2.42250 +2.60184
H +1.08001 −2.50734 +1.43633
H +1.03915 −3.55767 +2.83729
N −1.42909 −3.06291 +1.63517
O −1.27467 −1.82840 +3.50849
H −2.43355 −3.04202 +1.67194
H −0.97501 −3.53793 +0.87772
20
* E = +6.739 kcal/mol ; (124) 180_055_183_303_109_214
H +0.00000 +0.00000 +0.00000
O +0.00000 +0.00000 +0.96377
C +1.27732 +0.00000 +1.38181
C +1.36106 −0.00288 +2.89723
N +0.58323 −1.14253 +3.37950
H +0.57459 −1.15468 +4.39194
O +2.20339 +0.00983 +0.62345
C +2.80451 −0.09038 +3.37965
H +0.94124 +0.96689 +3.21746
H −0.37588 −1.09151 +3.05843
C +3.55945 −1.31173 +2.84187
H +3.35123 +0.81216 +3.08894
H +2.77854 −0.10321 +4.47438

C +4.61604 −0.91727 +1.82077
H +4.10253 −1.81286 +3.65031
H +2.86245 −2.05128 +2.43377
N +4.81373 −1.80998 +0.81289
O +5.29076 +0.07932 +1.94782
H +5.43699 −1.52698 +0.07498
H +4.08798 −2.46363 +0.58080
20
∗ E = +6.764 kcal/mol ; (125) 185_314_054_296_084_194
H +0.00000 +0.00000 +0.00000
O +0.00000 +0.00000 +0.96399
C +1.28648 +0.00000 +1.36997
C +1.39349 −0.11970 +2.87824
N +0.44084 +0.79036 +3.49737
H −0.49734 +0.64309 +3.14712
O +2.20536 +0.07013 +0.61233
C +1.27769 −1.59650 +3.31509
H +2.40500 +0.21845 +3.12093
H +0.43370 +0.68051 +4.50350
C −0.06838 −2.24931 +3.02120
H +2.06705 −2.17920 +2.82874
H +1.46484 −1.61960 +4.39438
C −0.16434 −2.77360 +1.59526
H −0.22209 −3.10820 +3.68519
H −0.90006 −1.55980 +3.21316
N −1.41759 −3.09746 +1.18856
O +0.80585 −2.91783 +0.88599
H −1.54203 −3.49167 +0.27237
H −2.21924 −2.95351 +1.77340
20
∗ E = +6.800 kcal/mol ; (126) 178_162_039_292_287_307
H +0.00000 +0.00000 +0.00000
O +0.00000 +0.00000 +0.96382
C +1.27796 +0.00000 +1.37582
C +1.38309 +0.06098 +2.88585
N +2.71312 −0.35447 +3.26833
H +3.40507 +0.01168 +2.62222
O +2.20497 +0.00149 +0.61939
C +0.97358 +1.48214 +3.33672
H +0.63863 −0.64122 +3.28283

H +2.94681 −0.04806 +4.20384
C +1.97583 +2.55479 +2.89550
H −0.01888 +1.71039 +2.93421
H +0.87242 +1.47074 +4.42845
C +3.28931 +2.56132 +3.66285
H +2.23883 +2.43859 +1.83966
H +1.52376 +3.54693 +3.01263
N +3.17079 +2.61088 +5.02359
O +4.36798 +2.52207 +3.11897
H +4.01414 +2.75277 +5.55415
H +2.29573 +2.83682 +5.46126
20
∗ E = +6.837 kcal/mol ; (127) 182_334_050_301_196_212
H +0.00000 +0.00000 +0.00000
O +0.00000 +0.00000 +0.96395
C +1.27963 +0.00000 +1.37340
C +1.42158 −0.04207 +2.88134
N +0.25696 +0.52572 +3.53508
H −0.60610 +0.13748 +3.17677
O +2.19964 −0.03883 +0.60754
C +1.77022 −1.49159 +3.27819
H +2.29215 +0.58166 +3.10974
H +0.29326 +0.36554 +4.53381
C +0.71794 −2.50295 +2.84057
H +2.74303 −1.75431 +2.84886
H +1.87115 −1.54032 +4.36733
C +0.88384 −3.82671 +3.56992
H −0.28660 −2.13415 +3.08925
H +0.73858 −2.65467 +1.75490
N +0.53293 −4.92863 +2.86067
O +1.27313 −3.88870 +4.71460
H +0.59207 −5.82796 +3.30638
H +0.23805 −4.87362 +1.90407
20
∗ E = +6.924 kcal/mol ; (128) 009_331_162_296_084_203
H +0.00000 +0.00000 +0.00000
O +0.00000 +0.00000 +0.96965
C +1.28165 +0.00000 +1.36189
C +2.27855 +0.22368 +0.21857
N +1.70719 −0.28475 −1.03002

H	+2.18626	+0.10339	−1.83370
O	+1.61189	−0.14013	+2.49718
C	+2.58954	+1.72036	+0.07644
H	+3.19997	−0.28841	+0.51947
H	+1.77952	−1.29345	−1.08899
C	+1.38736	+2.58044	−0.31287
H	+2.98708	+2.08709	+1.02753
H	+3.37835	+1.82258	−0.67780
C	+0.54393	+2.96402	+0.89917
H	+1.73596	+3.52233	−0.75169
H	+0.76941	+2.09167	−1.07551
N	−0.74639	+3.28703	+0.62833
O	+1.01632	+3.03008	+2.00920
H	−1.35328	+3.48945	+1.40462
H	−1.15760	+3.09548	−0.26600

20

∗ E = +6.938 kcal/mol ; (129) 179_044_191_178_186_350

H	+0.00000	+0.00000	+0.00000
O	+0.00000	+0.00000	+0.96383
C	+1.27604	+0.00000	+1.37937
C	+1.37957	+0.01936	+2.89421
N	+0.42342	−0.92288	+3.45825
H	+0.34850	−0.80191	+4.46009
O	+2.20277	+0.01819	+0.62251
C	+2.80409	−0.32010	+3.31520
H	+1.17072	+1.06821	+3.18168
H	−0.49772	−0.78591	+3.06108
C	+2.97563	−0.26158	+4.82755
H	+3.03197	−1.32430	+2.93658
H	+3.48180	+0.38375	+2.81981
C	+4.38831	−0.43289	+5.36607
H	+2.61805	+0.69498	+5.22625
H	+2.38039	−1.04242	+5.31506
N	+5.33146	−0.83663	+4.47909
O	+4.64058	−0.22980	+6.53276
H	+6.26972	−0.97017	+4.81472
H	+5.13140	−0.99627	+3.50944

20

∗ E = +6.944 kcal/mol ; (130) 181_083_071_076_305_265

H	+0.00000	+0.00000	+0.00000

O	+0.00000	+0.00000	+0.96340
C	+1.26101	+0.00000	+1.37816
C	+1.38218	−0.01313	+2.89726
N	+1.25383	−1.41777	+3.27903
H	+2.07776	−1.93643	+2.99477
O	+2.20585	−0.05001	+0.63537
C	+2.69532	+0.63388	+3.34653
H	+0.52256	+0.53423	+3.29951
H	+1.15763	−1.51215	+4.28186
C	+2.71424	+2.16732	+3.26128
H	+2.87432	+0.34268	+4.38718
H	+3.50670	+0.20788	+2.74318
C	+2.36456	+2.64892	+1.86689
H	+1.95742	+2.58251	+3.93298
H	+3.69296	+2.54510	+3.57367
N	+3.41019	+2.91536	+1.04689
O	+1.20870	+2.76236	+1.51364
H	+3.20913	+3.03928	+0.06837
H	+4.33952	+2.63263	+1.30054

20

∗ E = +7.074 kcal/mol ; (131) 359_149_292_058_059_234

H	+0.00000	+0.00000	+0.00000
O	+0.00000	+0.00000	+0.96437
C	+1.24860	+0.00000	+1.46188
C	+2.39720	−0.03284	+0.44562
N	+3.57536	+0.65953	+0.92383
H	+3.40977	+1.65676	+0.98591
O	+1.43430	−0.01125	+2.64170
C	+2.76782	−1.49596	+0.15832
H	+2.07194	+0.43617	−0.49456
H	+3.78371	+0.34131	+1.86559
C	+1.60615	−2.34357	−0.36333
H	+3.57110	−1.48192	−0.58238
H	+3.16335	−1.93743	+1.07992
C	+1.06925	−1.74610	−1.65339
H	+1.96627	−3.35039	−0.59616
H	+0.81337	−2.44266	+0.38648
N	−0.27891	−1.48336	−1.66904
O	+1.78820	−1.44677	−2.57500
H	−0.67669	−1.22784	−2.56008

H −0.88298 −1.95609 −1.01669
20
∗ E = +7.081 kcal/mol ; (132) 181_316_068_174_071_065
H +0.00000 +0.00000 +0.00000
O +0.00000 +0.00000 +0.96362
C +1.27779 +0.00000 +1.37802
C +1.37699 −0.03108 +2.89056
N +0.42030 +0.89129 +3.47039
H −0.53223 +0.58324 +3.31430
O +2.20428 −0.03755 +0.62099
C +1.24917 −1.50706 +3.32134
H +2.38642 +0.31668 +3.13367
H +0.56783 +1.00325 +4.46594
C +1.47445 −1.70640 +4.82386
H +0.25585 −1.87155 +3.02522
H +1.98593 −2.10049 +2.76794
C +0.35652 −1.16158 +5.69820
H +1.58488 −2.77600 +5.03658
H +2.39465 −1.20872 +5.14101
N −0.84236 −1.78200 +5.55196
O +0.51336 −0.23438 +6.46381
H −1.61143 −1.48529 +6.12779
H −0.95494 −2.58986 +4.96764
20
∗ E = +7.116 kcal/mol ; (133) 162_137_046_050_279_298
H +0.00000 +0.00000 +0.00000
O +0.00000 +0.00000 +0.96619
C +1.25716 +0.00000 +1.39702
C +1.37402 +0.43500 +2.84355
N +2.31365 −0.44461 +3.51016
H +3.14110 −0.55489 +2.93290
O +2.21226 −0.22326 +0.70488
C +1.80270 +1.91962 +2.82549
H +0.38647 +0.35015 +3.30988
H +2.59512 −0.07109 +4.40784
C +0.94803 +2.82078 +1.91734
H +1.76457 +2.28984 +3.85544
H +2.85542 +1.94375 +2.51528
C +1.32722 +2.77164 +0.43927
H −0.10810 +2.54553 +1.98087

H +1.04169 +3.86250 +2.24322
N +2.58712 +3.18573 +0.15473
O +0.55423 +2.42210 −0.42733
H +2.91915 +3.07176 −0.78777
H +3.25716 +3.37144 +0.87785
20
∗ E = +7.131 kcal/mol ; (134) 174_276_301_057_210_066
H +0.00000 +0.00000 +0.00000
O +0.00000 +0.00000 +0.96472
C +1.29419 +0.00000 +1.36624
C +1.44281 +0.14157 +2.87711
N +1.38988 +1.54464 +3.26277
H +2.11657 +2.08589 +2.81103
O +2.19667 −0.04183 +0.58261
C +0.37292 −0.64213 +3.62414
H +2.44030 −0.24300 +3.11484
H +0.49206 +1.95235 +3.02696
C +0.37338 −2.13663 +3.26500
H +0.53135 −0.50164 +4.69614
H −0.60095 −0.19461 +3.39146
C −1.01022 −2.75726 +3.36430
H +0.72681 −2.27995 +2.23465
H +1.03689 −2.70185 +3.92075
N −1.89668 −2.26573 +2.45792
O −1.29956 −3.60974 +4.17181
H −2.84662 −2.59281 +2.48104
H −1.63562 −1.53240 +1.82108
20
∗ E = +7.218 kcal/mol ; (135) 183_212_197_081_208_071
H +0.00000 +0.00000 +0.00000
O +0.00000 +0.00000 +0.96494
C +1.28926 +0.00000 +1.35966
C +1.47941 −0.07045 +2.86649
N +2.69286 +0.66482 +3.16793
H +3.03636 +0.43789 +4.09141
O +2.18944 +0.02976 +0.57269
C +0.28932 +0.48427 +3.65315
H +1.55887 −1.15476 +3.08065
H +3.41475 +0.45807 +2.48773
C −0.86588 −0.52234 +3.78859

H	+0.64575	+0.76871	+4.64739
H	−0.04769	+1.41122	+3.17675
C	−2.20791	+0.17488	+3.91869
H	−0.90098	−1.17331	+2.90830
H	−0.73238	−1.15140	+4.67039
N	−2.62929	+0.76056	+2.76669
O	−2.84019	+0.21766	+4.94941
H	−3.47805	+1.29849	+2.77421
H	−2.05927	+0.73364	+1.93908

20

∗ E = +7.233 kcal/mol ; (136) 179_044_192_181_193_216

H	+0.00000	+0.00000	+0.00000
O	+0.00000	+0.00000	+0.96365
C	+1.28078	+0.00000	+1.37580
C	+1.38595	+0.02124	+2.88993
N	+0.42417	−0.92033	+3.45405
H	+0.33530	−0.78161	+4.45253
O	+2.20185	+0.01734	+0.61441
C	+2.80811	−0.30904	+3.31890
H	+1.16925	+1.06955	+3.17418
H	−0.49232	−0.78750	+3.04453
C	+2.95299	−0.31172	+4.83564
H	+3.08208	−1.28847	+2.91255
H	+3.50189	+0.42061	+2.89356
C	+4.41527	−0.32143	+5.25560
H	+2.51683	+0.60571	+5.25683
H	+2.42207	−1.15989	+5.28234
N	+4.69432	−1.03892	+6.37417
O	+5.26347	+0.29481	+4.65262
H	+5.64391	−1.06497	+6.70377
H	+3.99904	−1.59134	+6.83910

20

∗ E = +7.251 kcal/mol ; (137) 181_313_068_183_295_163

H	+0.00000	+0.00000	+0.00000
O	+0.00000	+0.00000	+0.96336
C	+1.28216	+0.00000	+1.37618
C	+1.37441	−0.02476	+2.88861
N	+0.45640	+0.96272	+3.43485
H	−0.50987	+0.69901	+3.28147
O	+2.20342	−0.02964	+0.61473

C	+1.13991	−1.48175	+3.34068
H	+2.39741	+0.26319	+3.14344
H	+0.60881	+1.10429	+4.42404
C	+1.14500	−1.64056	+4.85416
H	+0.17497	−1.82249	+2.94595
H	+1.92436	−2.11164	+2.90845
C	+2.50095	−1.31737	+5.46485
H	+0.37000	−1.02012	+5.32068
H	+0.90744	−2.67716	+5.12170
N	+2.48846	−1.09018	+6.80450
O	+3.51669	−1.27389	+4.80897
H	+3.36549	−0.94931	+7.27597
H	+1.65196	−1.18634	+7.34954

20

∗ E = +7.304 kcal/mol ; (138) 182_171_047_279_053_061

H	+0.00000	+0.00000	+0.00000
O	+0.00000	+0.00000	+0.96342
C	+1.28757	+0.00000	+1.36576
C	+1.39596	−0.03976	+2.88013
N	+2.78170	−0.24739	+3.23097
H	+3.38730	+0.37390	+2.70234
O	+2.20173	+0.02678	+0.59848
C	+0.70997	+1.19507	+3.51839
H	+0.80894	−0.91881	+3.18083
H	+2.94150	−0.10113	+4.21877
C	+1.57823	+2.45762	+3.50720
H	−0.25297	+1.36478	+3.02250
H	+0.47664	+0.95192	+4.56052
C	+2.12627	+2.81182	+2.13448
H	+0.99718	+3.30325	+3.89154
H	+2.44837	+2.33330	+4.15464
N	+1.18798	+3.07699	+1.18797
O	+3.31324	+2.84395	+1.90097
H	+1.49545	+3.23075	+0.24266
H	+0.20612	+2.98188	+1.37372

20

∗ E = +7.310 kcal/mol ; (139) 177_035_187_260_057_028

H	+0.00000	+0.00000	+0.00000
O	+0.00000	+0.00000	+0.96390
C	+1.26890	+0.00000	+1.38146

C	+1.37565	+0.07196	+2.89711
N	+0.27950	−0.65276	+3.51232
H	+0.26904	−0.51644	+4.51483
O	+2.19683	+0.02603	+0.61873
C	+2.74308	−0.41914	+3.40931
H	+1.33150	+1.16362	+3.07740
H	−0.61610	−0.36589	+3.13997
C	+2.76247	−1.85161	+3.94627
H	+3.48017	−0.27744	+2.61142
H	+3.06304	+0.23810	+4.22393
C	+2.31938	−2.97525	+3.02377
H	+3.78605	−2.09175	+4.25794
H	+2.14041	−1.95086	+4.83823
N	+2.50495	−2.77371	+1.69524
O	+1.86837	−4.00846	+3.46589
H	+2.27189	−3.52733	+1.07182
H	+2.91189	−1.93637	+1.31578

20

∗ E = +7.422 kcal/mol ; (140) 182_061_303_182_075_039

H	+0.00000	+0.00000	+0.00000
O	+0.00000	+0.00000	+0.96373
C	+1.27634	+0.00000	+1.38314
C	+1.36101	−0.04917	+2.90392
N	+0.74583	−1.24468	+3.45524
H	−0.22324	−1.33190	+3.17852
O	+2.20491	+0.00983	+0.62542
C	+2.81154	+0.07443	+3.35278
H	+0.79174	+0.81125	+3.28230
H	+1.24618	−2.08329	+3.18338
C	+2.95772	−0.02121	+4.87092
H	+3.40782	−0.68537	+2.83252
H	+3.20417	+1.03273	+3.00001
C	+2.81774	−1.40315	+5.49005
H	+3.94665	+0.35005	+5.16724
H	+2.21781	+0.60762	+5.37348
N	+3.38126	−2.43483	+4.78557
O	+2.27796	−1.58105	+6.55491
H	+3.45504	−3.31517	+5.26821
H	+4.04133	−2.25449	+4.05054

20

∗ E = +7.469 kcal/mol ; (141) 185_092_071_232_071_011

H	+0.00000	+0.00000	+0.00000
O	+0.00000	+0.00000	+0.96458
C	+1.26032	+0.00000	+1.38527
C	+1.39380	−0.11453	+2.89608
N	+1.51704	−1.54667	+3.13933
H	+2.41051	−1.90864	+2.82779
O	+2.19936	+0.04509	+0.63203
C	+2.52554	+0.82405	+3.40963
H	+0.43791	+0.19315	+3.32870
H	+1.39821	−1.77420	+4.11673
C	+3.57063	+0.16091	+4.29899
H	+3.02256	+1.30099	+2.55744
H	+2.06387	+1.64008	+3.97322
C	+4.54481	−0.81422	+3.64994
H	+4.19756	+0.93376	+4.75693
H	+3.11337	−0.37843	+5.13421
N	+4.57062	−0.85540	+2.29504
O	+5.27150	−1.50280	+4.33454
H	+5.23561	−1.47873	+1.86876
H	+3.93650	−0.34352	+1.69812

20

∗ E = +7.483 kcal/mol ; (142) 182_351_049_294_290_300

H	+0.00000	+0.00000	+0.00000
O	+0.00000	+0.00000	+0.96407
C	+1.26959	+0.00000	+1.37601
C	+1.42738	−0.04768	+2.88734
N	+0.16123	+0.13355	+3.56734
H	−0.55909	−0.47451	+3.19368
O	+2.20074	−0.00694	+0.61931
C	+2.20278	−1.33731	+3.22601
H	+2.08371	+0.79892	+3.12695
H	+0.25328	−0.05798	+4.55708
C	+1.41435	−2.61187	+2.90229
H	+3.13813	−1.33390	+2.65798
H	+2.46861	−1.30681	+4.28978
C	+0.22800	−2.86759	+3.81797
H	+1.02158	−2.57805	+1.88135
H	+2.08258	−3.47868	+2.96377
N	+0.53768	−3.00774	+5.13727

O	−0.91290	−2.93177	+3.41732
H	−0.19942	−3.26946	+5.76978
H	+1.48862	−3.09624	+5.44639

20

∗ E = +7.505 kcal/mol ; (143) 000_180_188_290_280_149

H	+0.00000	+0.00000	+0.00000
O	+0.00000	+0.00000	+0.96016
C	+1.25480	+0.00000	+1.44556
C	+2.36065	−0.01032	+0.39293
N	+3.62964	−0.01399	+1.07698
H	+4.40705	+0.11491	+0.44283
O	+1.44950	+0.02478	+2.62157
C	+2.26121	−1.25636	−0.50448
H	+2.18244	+0.88410	−0.23485
H	+3.65924	+0.65526	+1.83399
C	+2.62160	−2.52095	+0.26736
H	+1.26055	−1.34263	−0.94243
H	+2.96450	−1.14976	−1.33699
C	+4.13186	−2.67919	+0.37325
H	+2.16561	−2.51942	+1.26481
H	+2.25004	−3.40566	−0.26139
N	+4.57345	−3.30652	+1.49040
O	+4.87542	−2.33465	−0.52165
H	+5.56872	−3.36747	+1.62455
H	+3.98382	−3.36929	+2.30054

20

∗ E = +7.542 kcal/mol ; (144) 183_318_064_173_175_155

H	+0.00000	+0.00000	+0.00000
O	+0.00000	+0.00000	+0.96362
C	+1.27529	+0.00000	+1.38037
C	+1.37512	−0.06284	+2.89132
N	+0.37430	+0.80692	+3.48683
H	−0.56149	+0.48180	+3.27208
O	+2.20613	−0.01311	+0.62796
C	+1.29517	−1.54768	+3.29190
H	+2.36814	+0.32726	+3.13970
H	+0.47566	+0.85343	+4.49174
C	+1.56458	−1.75767	+4.77377
H	+0.30200	−1.94042	+3.04661
H	+2.01882	−2.12336	+2.70303

C	+1.35211	−3.20797	+5.17966
H	+2.57830	−1.43227	+5.03783
H	+0.86968	−1.15989	+5.37850
N	+2.04931	−3.60925	+6.27318
O	+0.59545	−3.94222	+4.58644
H	+1.92180	−4.54871	+6.60786
H	+2.69459	−3.00422	+6.74512

20

∗ E = +7.552 kcal/mol ; (145) 356_145_042_061_058_236

H	+0.00000	+0.00000	+0.00000
O	+0.00000	+0.00000	+0.96402
C	+1.24949	+0.00000	+1.45304
C	+2.37795	−0.10426	+0.43164
N	+3.48195	+0.69759	+0.91539
H	+3.59562	+0.54776	+1.91252
O	+1.44848	+0.01890	+2.63001
C	+2.72230	−1.59566	+0.23935
H	+2.03577	+0.30186	−0.53262
H	+4.34496	+0.45287	+0.44700
C	+1.55318	−2.43769	−0.27861
H	+3.54263	−1.65460	−0.48360
H	+3.07795	−1.99321	+1.19591
C	+1.06850	−1.87668	−1.60485
H	+1.88951	−3.46299	−0.46045
H	+0.74086	−2.48036	+0.45515
N	−0.26398	−1.55331	−1.66084
O	+1.82075	−1.65429	−2.52244
H	−0.63191	−1.31396	−2.56901
H	−0.90254	−1.96823	−1.00216

20

∗ E = +7.700 kcal/mol ; (146) 179_042_181_289_287_320

H	+0.00000	+0.00000	+0.00000
O	+0.00000	+0.00000	+0.96375
C	+1.27441	+0.00000	+1.37988
C	+1.37362	+0.01604	+2.89799
N	+0.38670	−0.89055	+3.45314
H	+0.41529	−0.89764	+4.46458
O	+2.20235	+0.03210	+0.62379
C	+2.78410	−0.37304	+3.33533
H	+1.20877	+1.07590	+3.17166

H	−0.55002	−0.67322	+3.14107
C	+3.09698	−1.85289	+3.10393
H	+3.49390	+0.23719	+2.77045
H	+2.90780	−0.09956	+4.39011
C	+2.43806	−2.84801	+4.04416
H	+2.82051	−2.15162	+2.09003
H	+4.17921	−2.01047	+3.19353
N	+2.37613	−2.47225	+5.36541
O	+2.02145	−3.91717	+3.67207
H	+2.12788	−3.20162	+6.01417
H	+2.98117	−1.74785	+5.71019

20

∗ E = +7.703 kcal/mol ; (147) 001_176_313_184_081_207

H	+0.00000	+0.00000	+0.00000
O	+0.00000	+0.00000	+0.95976
C	+1.25687	+0.00000	+1.44568
C	+2.37284	+0.01850	+0.39574
N	+3.69214	+0.12126	+0.97273
H	+3.71215	+0.85055	+1.67588
O	+1.45289	+0.00449	+2.62086
C	+2.29099	−1.21977	−0.50931
H	+2.19874	+0.91177	−0.22780
H	+3.93030	−0.74742	+1.44347
C	+3.44963	−1.26242	−1.49946
H	+2.32865	−2.12573	+0.10745
H	+1.34032	−1.23570	−1.05302
C	+4.71652	−1.80760	−0.85419
H	+3.21107	−1.93636	−2.33037
H	+3.63676	−0.27017	−1.92609
N	+5.87810	−1.38674	−1.40923
O	+4.67987	−2.61781	+0.04718
H	+6.73834	−1.68838	−0.98396
H	+5.90027	−0.59384	−2.02325

20

∗ E = +7.861 kcal/mol ; (148) 181_243_192_085_306_254

H	+0.00000	+0.00000	+0.00000
O	+0.00000	+0.00000	+0.96397
C	+1.27844	+0.00000	+1.36247
C	+1.47635	−0.01425	+2.87272
N	+2.16675	+1.23976	+3.17455

H +2.51475 +1.22888 +4.12529
O +2.19472 +0.06308 +0.59355
C +0.18787 −0.10526 +3.69018
H +2.09132 −0.90884 +3.06318
H +2.96241 +1.34642 +2.55469
C −0.31890 −1.53844 +3.90492
H +0.39036 +0.32837 +4.67567
H −0.58224 +0.52006 +3.22956
C −0.47476 −2.31132 +2.60836
H +0.41454 −2.09340 +4.49882
H −1.25732 −1.51843 +4.46773
N −1.74479 −2.51006 +2.18123
O +0.48947 −2.72153 +1.99646
H −1.88717 −2.96343 +1.29498
H −2.53153 −2.11037 +2.65705
20
∗ E = +7.929 kcal/mol ; (149) 179_040_187_171_081_233
H +0.00000 +0.00000 +0.00000
O +0.00000 +0.00000 +0.96360
C +1.27843 +0.00000 +1.37558
C +1.38772 +0.02417 +2.89077
N +0.36712 −0.83623 +3.46736
H +0.34049 −0.76174 +4.47673
O +2.19981 +0.02816 +0.61259
C +2.79081 −0.39478 +3.31585
H +1.25376 +1.09339 +3.15582
H −0.55118 −0.60886 +3.10835
C +3.03578 −0.19282 +4.81276
H +2.94311 −1.45070 +3.06861
H +3.51140 +0.18905 +2.73834
C +2.46708 −1.32968 +5.64843
H +4.11325 −0.19192 +5.00673
H +2.64470 +0.77855 +5.14173
N +1.62453 −0.95751 +6.65897
O +2.72653 −2.48553 +5.41947
H +1.32991 −1.67301 +7.30340
H +1.57128 −0.00076 +6.95819
20
∗ E = +8.030 kcal/mol ; (150) 356_163_303_315_289_104
H +0.00000 +0.00000 +0.00000

O	+0.00000	+0.00000	+0.96067
C	+1.25187	+0.00000	+1.44396
C	+2.36614	−0.10718	+0.39946
N	+3.66946	+0.26984	+0.88951
H	+3.65731	+1.21221	+1.26027
O	+1.44196	+0.01860	+2.62544
C	+2.36158	−1.55335	−0.14473
H	+2.11348	+0.56559	−0.43289
H	+3.96445	−0.33700	+1.64591
C	+2.24826	−2.64739	+0.92640
H	+1.52595	−1.67441	−0.84334
H	+3.28529	−1.69018	−0.71541
C	+3.51438	−2.79261	+1.75954
H	+1.37297	−2.48053	+1.56519
H	+2.11280	−3.60879	+0.42506
N	+3.46304	−2.26035	+3.00658
O	+4.49728	−3.34551	+1.31817
H	+4.28506	−2.32106	+3.58376
H	+2.68786	−1.68936	+3.30533

20

∗ E = +8.049 kcal/mol ; (151) 002_190_212_303_181_167

H	+0.00000	+0.00000	+0.00000
O	+0.00000	+0.00000	+0.96073
C	+1.25071	+0.00000	+1.44668
C	+2.36546	+0.05680	+0.40423
N	+3.62838	−0.18282	+1.07299
H	+4.38848	+0.31608	+0.63329
O	+1.44626	−0.02550	+2.62371
C	+2.14557	−0.97137	−0.71496
H	+2.29520	+1.06721	−0.03653
H	+3.56698	+0.08607	+2.04809
C	+2.02249	−2.38522	−0.16868
H	+1.27180	−0.73234	−1.33466
H	+2.99662	−0.91671	−1.40152
C	+1.83183	−3.39451	−1.28604
H	+2.90766	−2.62924	+0.42832
H	+1.15780	−2.46926	+0.50525
N	+1.99818	−4.69063	−0.92348
O	+1.53547	−3.06633	−2.41439
H	+1.85104	−5.40901	−1.61099

H +2.25448 −4.94962 +0.01103
20
∗ E = +8.246 kcal/mol ; (152) 182_317_298_059_279_341
H +0.00000 +0.00000 +0.00000
O +0.00000 +0.00000 +0.96414
C +1.28764 +0.00000 +1.37037
C +1.40685 −0.04550 +2.88635
N +0.48439 +0.82737 +3.58915
H +0.64080 +1.79977 +3.35481
O +2.20509 +0.01056 +0.60405
C +1.22117 −1.48349 +3.38804
H +2.43316 +0.26559 +3.10809
H −0.47391 +0.60604 +3.34311
C +2.22489 −2.49276 +2.83190
H +1.32556 −1.43094 +4.47567
H +0.18169 −1.79565 +3.21340
C +2.00973 −3.09272 +1.44775
H +3.23115 −2.06256 +2.81252
H +2.28000 −3.35673 +3.50327
N +0.75634 −3.02691 +0.92676
O +2.91217 −3.65850 +0.87548
H +0.60760 −3.44147 +0.02282
H +0.00711 −2.51938 +1.35824
20
∗ E = +8.333 kcal/mol ; (153) 358_169_289_140_301_315
H +0.00000 +0.00000 +0.00000
O +0.00000 +0.00000 +0.96818
C +1.25336 +0.00000 +1.44804
C +2.34336 −0.04051 +0.37532
N +3.67484 +0.21529 +0.87495
H +3.77974 +1.18860 +1.13438
O +1.47435 +0.00676 +2.62089
C +2.30291 −1.40752 −0.34703
H +2.09788 +0.74880 −0.35166
H +3.81926 −0.32070 +1.72554
C +2.53900 −1.24332 −1.84857
H +3.08488 −2.03777 +0.08573
H +1.35230 −1.92071 −0.16042
C +1.49234 −0.34407 −2.48003
H +3.51024 −0.77942 −2.03580

H	+2.52530	−2.21518	−2.35619
N	+0.18299	−0.60528	−2.09544
O	+1.75644	+0.58396	−3.19927
H	−0.51188	−0.13650	−2.65945
H	−0.04936	−1.55399	−1.84166

20

∗ E = +8.519 kcal/mol ; (154) 358_170_267_294_251_049

H	+0.00000	+0.00000	+0.00000
O	+0.00000	+0.00000	+0.96025
C	+1.24820	+0.00000	+1.45018
C	+2.37597	−0.04192	+0.41593
N	+3.66647	+0.19689	+1.04683
H	+3.90580	+1.17970	+1.01530
O	+1.44356	−0.00415	+2.62775
C	+2.35702	−1.40528	−0.30250
H	+2.18438	+0.74277	−0.32758
H	+3.58992	−0.04125	+2.03355
C	+2.70265	−2.58816	+0.62454
H	+1.37271	−1.57044	−0.75538
H	+3.07207	−1.35147	−1.12912
C	+4.06562	−3.22077	+0.36228
H	+2.67675	−2.27159	+1.67658
H	+1.97632	−3.39491	+0.52397
N	+5.09428	−2.34666	+0.26704
O	+4.19773	−4.42025	+0.24821
H	+6.01897	−2.71694	+0.13216
H	+4.95138	−1.36120	+0.44809

20

∗ E = +8.572 kcal/mol ; (155) 358_167_281_171_110_298

H	+0.00000	+0.00000	+0.00000
O	+0.00000	+0.00000	+0.96025
C	+1.24962	+0.00000	+1.45068
C	+2.37657	−0.04320	+0.41497
N	+3.66490	+0.25872	+1.01600
H	+3.77292	+1.25788	+1.14405
O	+1.44577	+0.00522	+2.62724
C	+2.40210	−1.42725	−0.25561
H	+2.16429	+0.71011	−0.35717
H	+3.67213	−0.13977	+1.95300
C	+3.35984	−1.48882	−1.46283

H +2.68940 −2.16549 +0.50359
H +1.39041 −1.70088 −0.57401
C +4.60250 −2.33771 −1.22307
H +2.86411 −1.91989 −2.33305
H +3.68440 −0.47463 −1.72685
N +5.40439 −1.88725 −0.23230
O +4.83378 −3.33447 −1.87295
H +6.23859 −2.40398 −0.01501
H +5.16398 −1.04306 +0.26975
20
∗ E = +8.592 kcal/mol ; (156) 178_299_292_214_076_011
H +0.00000 +0.00000 +0.00000
O +0.00000 +0.00000 +0.96374
C +1.28753 +0.00000 +1.38466
C +1.36755 +0.04201 +2.90248
N +0.76172 +1.24458 +3.45170
H +1.29788 +2.06797 +3.21229
O +2.20663 −0.01493 +0.62056
C +0.75957 −1.26268 +3.48519
H +2.43257 +0.07669 +3.14519
H −0.19359 +1.37465 +3.14106
C +0.04058 −1.08230 +4.81685
H +0.08467 −1.72880 +2.75777
H +1.57397 −1.98103 +3.61345
C −1.33751 −0.43705 +4.78812
H −0.10665 −2.06061 +5.28724
H +0.63603 −0.48560 +5.51196
N −1.92843 −0.27308 +3.57116
O −1.89433 −0.11407 +5.81257
H −2.86374 +0.09640 +3.56013
H −1.50138 −0.53668 +2.70065
20
∗ E = +8.626 kcal/mol ; (157) 002_173_298_176_190_206
H +0.00000 +0.00000 +0.00000
O +0.00000 +0.00000 +0.96042
C +1.25186 +0.00000 +1.44940
C +2.37640 +0.04476 +0.40838
N +3.68364 +0.20722 +1.00743
H +3.73326 +1.08555 +1.51038
O +1.44718 −0.03344 +2.62563

C +2.34440 −1.21883 −0.45807
H +2.18499 +0.91305 −0.24121
H +3.81489 −0.51448 +1.71038
C +3.38132 −1.16432 −1.57033
H +2.52574 −2.09293 +0.18073
H +1.35954 −1.37678 −0.91109
C +3.18109 −2.29618 −2.56405
H +3.27763 −0.22391 −2.12880
H +4.39416 −1.16659 −1.15845
N +4.29654 −2.68484 −3.22959
O +2.09568 −2.79511 −2.76679
H +4.21658 −3.40096 −3.93082
H +5.19441 −2.28069 −3.03976
20
∗ E = +8.818 kcal/mol ; (158) 182_172_028_086_215_080
H +0.00000 +0.00000 +0.00000
O +0.00000 +0.00000 +0.96483
C +1.29100 +0.00000 +1.35955
C +1.44777 −0.04091 +2.86678
N +2.85145 −0.21879 +3.16143
H +3.43123 +0.16048 +2.42284
O +2.19004 +0.06486 +0.57394
C +0.79777 +1.22687 +3.47229
H +0.87709 −0.91231 +3.22306
H +3.10826 +0.20056 +4.04349
C −0.71701 +1.08175 +3.72144
H +1.30051 +1.44048 +4.41981
H +1.00324 +2.08486 +2.81936
C −1.43411 +2.39363 +3.46882
H −1.13329 +0.31248 +3.06386
H −0.91009 +0.78753 +4.75418
N −1.66665 +2.64227 +2.15258
O −1.73551 +3.16784 +4.34818
H −2.08749 +3.51503 +1.88548
H −1.40961 +1.97294 +1.44898
20
∗ E = +9.117 kcal/mol ; (159) 354_164_024_306_194_198
H +0.00000 +0.00000 +0.00000
O +0.00000 +0.00000 +0.96059
C +1.25374 +0.00000 +1.44115

C	+2.35249	−0.13987	+0.39604
N	+3.60908	+0.25357	+0.98885
H	+3.57767	+0.14309	+1.99575
O	+1.45689	+0.03607	+2.61682
C	+2.32148	−1.58838	−0.13587
H	+2.11029	+0.53440	−0.44070
H	+4.38937	−0.26530	+0.60969
C	+2.41004	−2.61896	+0.98029
H	+1.41586	−1.76126	−0.73078
H	+3.16204	−1.72799	−0.82341
C	+2.71588	−4.00615	+0.44030
H	+3.21483	−2.34785	+1.67697
H	+1.48870	−2.64287	+1.57490
N	+2.43410	−5.02455	+1.29036
O	+3.19252	−4.18746	−0.65818
H	+2.65804	−5.96392	+1.01097
H	+2.04423	−4.86297	+2.20039

20

∗ E = +9.319 kcal/mol ; (160) 180_313_061_060_277_343

H	+0.00000	+0.00000	+0.00000
O	+0.00000	+0.00000	+0.96415
C	+1.28583	+0.00000	+1.37392
C	+1.38943	−0.00394	+2.88484
N	+0.48254	+0.99342	+3.42734
H	−0.47714	+0.78496	+3.17723
O	+2.20627	−0.04419	+0.61479
C	+1.17189	−1.43122	+3.42572
H	+2.41939	+0.29557	+3.10540
H	+0.54737	+1.02643	+4.43700
C	+2.18052	−2.46659	+2.92722
H	+1.25359	−1.35952	+4.51621
H	+0.13383	−1.73611	+3.23452
C	+1.97462	−3.12779	+1.56930
H	+3.18658	−2.03563	+2.89385
H	+2.23463	−3.30038	+3.63548
N	+0.73698	−3.04834	+1.01385
O	+2.87048	−3.75345	+1.05163
H	+0.60881	−3.47911	+0.11411
H	+0.00923	−2.46059	+1.37521

20

```
 * E =  +9.798  kcal/mol  ;  (161)  184_070_180_085_294_090
 H    +0.00000    +0.00000    +0.00000
 O    +0.00000    +0.00000    +0.96387
 C    +1.28251    +0.00000    +1.37615
 C    +1.35549    -0.09668    +2.89417
 N    +0.93132    -1.45534    +3.22348
 H    +0.95641    -1.60264    +4.22460
 O    +2.20722    +0.02817    +0.62349
 C    +2.77055    +0.16011    +3.40114
 H    +0.67080    +0.67295    +3.29685
 H    -0.01430    -1.62729    +2.90343
 C    +3.09352    +1.64686    +3.59617
 H    +2.88462    -0.34720    +4.36535
 H    +3.48556    -0.29156    +2.70669
 C    +3.11175    +2.41357    +2.28434
 H    +2.37032    +2.09321    +4.29081
 H    +4.08968    +1.74950    +4.03082
 N    +1.91345    +2.95578    +1.91527
 O    +4.10031    +2.50051    +1.59806
 H    +1.88435    +3.49493    +1.06615
 H    +1.15504    +3.04435    +2.56608
20
 * E =  +9.822  kcal/mol  ;  (162)  356_176_230_160_048_091
 H    +0.00000    +0.00000    +0.00000
 O    +0.00000    +0.00000    +0.95993
 C    +1.25437    +0.00000    +1.44474
 C    +2.36407    -0.08903    +0.40107
 N    +3.65677    +0.02580    +1.04723
 H    +4.23724    +0.71945    +0.59684
 O    +1.44866    +0.06189    +2.61984
 C    +2.25936    -1.42210    -0.37164
 H    +2.19739    +0.73572    -0.31260
 H    +3.53176    +0.25450    +2.02680
 C    +2.98697    -1.37430    -1.72237
 H    +2.69028    -2.19402    +0.27489
 H    +1.21558    -1.70372    -0.55787
 C    +4.38956    -0.80356    -1.60579
 H    +3.01145    -2.37426    -2.16725
 H    +2.44846    -0.71216    -2.40646
 N    +5.37725    -1.70236    -1.38443
```

O +4.59796 +0.38855 −1.70975
H +6.29155 −1.35040 −1.15510
H +5.16391 −2.65492 −1.15072
20
* E = +10.217 kcal/mol ; (163) 359_152_292_038_063_001
H +0.00000 +0.00000 +0.00000
O +0.00000 +0.00000 +0.96530
C +1.24812 +0.00000 +1.45175
C +2.37242 −0.01622 +0.41068
N +3.59092 +0.60906 +0.87904
H +3.46251 +1.60707 +0.99569
O +1.45825 −0.01830 +2.62721
C +2.69419 −1.48001 +0.03558
H +2.02580 +0.53873 −0.46953
H +3.81761 +0.23976 +1.79792
C +1.52613 −2.46087 −0.07095
H +3.30003 −1.46994 −0.87823
H +3.36003 −1.85287 +0.82202
C +0.45450 −2.21808 −1.12005
H +1.91129 −3.46730 −0.26335
H +0.98985 −2.53384 +0.88254
N +0.62084 −1.11109 −1.93073
O −0.52435 −2.91423 −1.20197
H −0.01120 −1.07112 −2.71718
H +1.55685 −0.78444 −2.11094
20
* E = +10.399 kcal/mol ; (164) 356_146_298_055_170_150
H +0.00000 +0.00000 +0.00000
O +0.00000 +0.00000 +0.95995
C +1.26027 +0.00000 +1.43782
C +2.38486 −0.10600 +0.40153
N +3.56546 +0.63946 +0.78067
H +3.36626 +1.63014 +0.85078
O +1.46337 +0.03698 +2.61271
C +2.75614 −1.58451 +0.24263
H +2.02595 +0.27670 −0.56605
H +3.86650 +0.33709 +1.70199
C +1.58070 −2.48296 −0.12060
H +3.53092 −1.66765 −0.52309
H +3.20076 −1.92373 +1.18676

C	+2.05323	−3.88186	−0.49200
H	+0.84233	−2.53184	+0.68808
H	+1.05975	−2.08856	−1.00570
N	+1.17900	−4.87954	−0.20869
O	+3.11833	−4.08070	−1.02939
H	+1.43043	−5.82268	−0.45041
H	+0.32291	−4.71244	+0.28598

20

∗ E = +10.437 kcal/mol ; (165) 355_175_297_278_049_065

H	+0.00000	+0.00000	+0.00000
O	+0.00000	+0.00000	+0.95996
C	+1.26417	+0.00000	+1.44445
C	+2.37147	−0.11694	+0.38327
N	+3.70197	+0.00854	+0.92683
H	+3.83155	+0.92862	+1.32924
O	+1.46169	+0.09959	+2.61307
C	+2.24288	−1.42099	−0.43431
H	+2.20096	+0.71997	−0.31072
H	+3.83002	−0.65351	+1.68998
C	+2.85030	−2.64318	+0.26635
H	+1.19083	−1.61107	−0.68565
H	+2.76798	−1.27804	−1.38273
C	+2.39200	−2.79896	+1.70793
H	+2.60479	−3.54818	−0.29927
H	+3.93703	−2.54822	+0.29655
N	+1.06879	−3.04667	+1.88129
O	+3.16053	−2.68334	+2.63686
H	+0.69719	−2.98386	+2.81492
H	+0.42426	−3.02713	+1.11193

20

∗ E = +10.514 kcal/mol ; (166) 353_144_039_060_167_148

H	+0.00000	+0.00000	+0.00000
O	+0.00000	+0.00000	+0.95987
C	+1.25722	+0.00000	+1.43526
C	+2.37079	−0.17597	+0.40818
N	+3.48668	+0.64742	+0.82408
H	+3.57964	+0.59814	+1.83359
O	+1.46642	+0.06983	+2.60750
C	+2.69859	−1.67610	+0.30953
H	+2.01201	+0.16940	−0.57503

H +4.35094 +0.32583 +0.40671
C +1.51973 −2.54116 −0.12002
H +3.51048 −1.81245 −0.41249
H +3.07373 −2.00620 +1.28557
C +1.97632 −3.94402 −0.49614
H +0.74555 −2.58087 +0.65450
H +1.05056 −2.11880 −1.02109
N +1.08681 −4.93248 −0.23307
O +3.04679 −4.15143 −1.02092
H +1.32931 −5.87684 −0.47935
H +0.22451 −4.75789 +0.24805
20
* E = +10.682 kcal/mol ; (167) 005_192_200_110_063_183
H +0.00000 +0.00000 +0.00000
O +0.00000 +0.00000 +0.95974
C +1.25918 +0.00000 +1.44473
C +2.36325 +0.11393 +0.40132
N +3.62899 −0.18337 +1.02606
H +4.38181 +0.19676 +0.46543
O +1.45152 −0.02965 +2.62004
C +2.13958 −0.79326 −0.83363
H +2.29231 +1.16619 +0.05840
H +3.66342 +0.19155 +1.96599
C +1.80101 −0.01404 −2.09875
H +3.07414 −1.33543 −0.99295
H +1.37244 −1.55119 −0.63701
C +2.96386 +0.88676 −2.50113
H +1.58435 −0.69274 −2.93179
H +0.90736 +0.61567 −1.97623
N +2.74020 +1.67309 −3.58076
O +4.01342 +0.89519 −1.89472
H +3.48003 +2.27189 −3.90522
H +1.87742 +1.64214 −4.09157
20
* E = +10.686 kcal/mol ; (168) 357_167_301_126_277_077
H +0.00000 +0.00000 +0.00000
O +0.00000 +0.00000 +0.96853
C +1.24662 +0.00000 +1.45684
C +2.35460 −0.07918 +0.40158
N +3.67228 +0.21373 +0.92179

H	+3.69472	+1.14706	+1.31721
O	+1.46029	+0.04263	+2.63078
C	+2.34578	−1.47662	−0.26680
H	+2.12061	+0.68391	−0.35377
H	+3.86916	−0.41425	+1.69543
C	+2.25314	−1.44024	−1.80210
H	+3.27856	−1.96968	+0.01858
H	+1.52804	−2.09660	+0.11963
C	+0.80904	−1.27450	−2.22765
H	+2.88750	−0.63533	−2.19187
H	+2.59292	−2.38636	−2.22580
N	+0.29155	+0.00900	−2.10341
O	+0.10677	−2.20115	−2.53735
H	−0.62450	+0.12532	−2.51487
H	+0.92099	+0.78272	−2.25152

20

∗ E = +10.790 kcal/mol ; (169) 357_162_037_141_302_316

H	+0.00000	+0.00000	+0.00000
O	+0.00000	+0.00000	+0.96739
C	+1.25301	+0.00000	+1.44438
C	+2.33397	−0.07456	+0.37489
N	+3.58811	+0.35036	+0.94764
H	+3.66137	+0.02277	+1.90465
O	+1.47739	+0.00200	+2.61597
C	+2.31336	−1.50785	−0.23213
H	+2.05178	+0.63806	−0.41736
H	+4.38410	+0.03048	+0.41270
C	+2.53551	−1.48484	−1.74537
H	+3.09430	−2.09659	+0.25789
H	+1.36704	−2.01270	−0.00140
C	+1.49544	−0.64177	−2.46253
H	+3.50754	−1.05568	−2.00259
H	+2.50707	−2.50081	−2.15715
N	+0.18863	−0.82900	−2.03773
O	+1.77217	+0.18289	−3.29413
H	−0.50581	−0.41740	−2.64501
H	−0.05968	−1.72956	−1.65646

20

∗ E = +10.797 kcal/mol ; (170) 357_183_307_304_139_282

H	+0.00000	+0.00000	+0.00000

O	+0.00000	+0.00000	+0.96102
C	+1.25907	+0.00000	+1.45060
C	+2.38250	−0.07167	+0.40653
N	+3.69514	−0.13016	+1.00486
H	+3.81813	+0.63263	+1.66097
O	+1.44377	+0.01512	+2.62782
C	+2.15653	−1.29007	−0.49851
H	+2.31765	+0.83868	−0.20683
H	+3.78667	−0.98004	+1.55270
C	+2.04143	−2.60625	+0.30414
H	+1.25376	−1.15139	−1.10615
H	+2.99004	−1.34416	−1.20185
C	+0.94844	−3.48866	−0.27280
H	+2.97413	−3.17060	+0.27221
H	+1.81341	−2.39507	+1.35848
N	−0.31382	−3.11238	+0.07501
O	+1.17320	−4.40310	−1.03049
H	−1.08166	−3.67287	−0.25398
H	−0.48255	−2.44316	+0.80692

20

∗ E = +11.132 kcal/mol ; (171) 007_069_179_068_216_066

H	+0.00000	+0.00000	+0.00000
O	+0.00000	+0.00000	+0.96006
C	+1.24957	+0.00000	+1.45095
C	+2.35807	+0.17260	+0.41063
N	+2.27159	+1.54180	−0.08848
H	+3.00072	+1.72193	−0.76701
O	+1.43186	−0.11289	+2.62600
C	+3.71307	−0.05824	+1.05991
H	+2.19543	−0.59499	−0.37165
H	+1.38240	+1.76205	−0.51893
C	+3.91901	−1.51943	+1.49458
H	+4.49801	+0.23819	+0.35655
H	+3.78280	+0.61906	+1.91677
C	+4.70490	−1.62929	+2.79321
H	+2.94735	−2.00324	+1.65290
H	+4.45913	−2.08967	+0.73749
N	+4.05046	−1.13432	+3.87025
O	+5.81505	−2.11207	+2.84794
H	+4.51086	−1.14671	+4.76331

H	+3.12927	−0.73344	+3.77895

20

∗ E = +11.220 kcal/mol ; (172) 357_168_296_294_282_291

H	+0.00000	+0.00000	+0.00000
O	+0.00000	+0.00000	+0.95989
C	+1.25927	+0.00000	+1.44562
C	+2.37272	−0.07815	+0.39155
N	+3.68589	+0.19194	+0.92088
H	+3.74162	+1.14184	+1.26702
O	+1.45625	+0.03735	+2.61929
C	+2.33759	−1.46149	−0.28168
H	+2.14533	+0.67429	−0.37812
H	+3.88523	−0.41433	+1.71354
C	+2.68313	−2.60888	+0.68093
H	+1.34327	−1.64379	−0.70553
H	+3.04356	−1.43701	−1.11876
C	+4.17425	−2.76331	+0.95051
H	+2.19863	−2.46314	+1.65155
H	+2.31418	−3.55266	+0.26469
N	+4.90492	−3.20199	−0.10523
O	+4.67052	−2.53021	+2.03022
H	+5.90453	−3.25022	−0.00863
H	+4.49764	−3.34763	−1.01014

20

∗ E = +11.514 kcal/mol ; (173) 351_149_048_176_072_070

H	+0.00000	+0.00000	+0.00000
O	+0.00000	+0.00000	+0.95954
C	+1.25568	+0.00000	+1.44062
C	+2.36830	−0.21135	+0.41953
N	+3.53064	+0.50730	+0.87605
H	+3.71267	+0.31107	+1.85298
O	+1.46196	+0.08023	+2.61190
C	+2.51005	−1.74374	+0.26213
H	+2.05950	+0.20549	−0.55202
H	+4.35329	+0.33123	+0.31423
C	+3.53177	−2.16018	−0.80251
H	+2.77817	−2.15887	+1.24263
H	+1.53352	−2.16874	−0.00319
C	+4.97544	−1.92023	−0.39118
H	+3.40704	−3.22593	−1.02510

H	+3.36914	−1.60448	−1.73002
N	+5.43698	−2.73762	+0.58616
O	+5.66287	−1.05414	−0.89061
H	+6.37625	−2.60886	+0.92134
H	+4.86593	−3.45255	+0.99792

20

∗ E = +11.518 kcal/mol ; (174) 351_143_298_044_268_121

H	+0.00000	+0.00000	+0.00000
O	+0.00000	+0.00000	+0.96010
C	+1.27098	+0.00000	+1.43573
C	+2.37500	−0.20867	+0.39547
N	+3.56009	+0.57481	+0.66368
H	+3.36350	+1.56810	+0.64256
O	+1.47575	+0.11257	+2.60278
C	+2.76373	−1.69632	+0.36112
H	+1.98701	+0.07765	−0.59403
H	+3.90000	+0.35738	+1.59527
C	+1.58465	−2.67902	+0.36931
H	+3.39210	−1.84047	−0.52068
H	+3.38829	−1.90956	+1.23694
C	+1.22640	−3.09569	+1.79448
H	+0.71532	−2.28912	−0.17337
H	+1.88394	−3.60278	−0.13285
N	−0.04047	−2.83785	+2.20628
O	+2.04263	−3.66167	+2.48583
H	−0.23736	−3.00240	+3.18040
H	−0.59171	−2.13997	+1.73666

20

∗ E = +11.672 kcal/mol ; (175) 353_156_048_180_082_218

H	+0.00000	+0.00000	+0.00000
O	+0.00000	+0.00000	+0.95955
C	+1.25701	+0.00000	+1.44269
C	+2.36163	−0.15759	+0.40413
N	+3.57335	+0.40362	+0.95414
H	+3.72001	+0.05217	+1.89462
O	+1.46098	+0.05366	+2.61437
C	+2.41135	−1.65308	+0.01895
H	+2.08608	+0.42163	−0.49257
H	+4.38803	+0.18802	+0.39483
C	+3.49355	−1.94982	−1.01569

H	+2.61459	−2.24868	+0.91663
H	+1.43393	−1.96360	−0.36915
C	+4.86764	−2.09264	−0.37407
H	+3.28818	−2.90925	−1.50364
H	+3.50688	−1.18578	−1.80355
N	+5.90150	−1.56750	−1.08472
O	+5.02509	−2.63614	+0.69405
H	+6.83764	−1.72572	−0.75097
H	+5.77362	−1.20083	−2.00983

20

∗ E = +11.934 kcal/mol ; (176) 357_149_038_040_061_001

H	+0.00000	+0.00000	+0.00000
O	+0.00000	+0.00000	+0.96487
C	+1.24510	+0.00000	+1.45153
C	+2.36251	−0.06931	+0.41597
N	+3.50640	+0.64864	+0.93792
H	+3.58088	+0.47687	+1.93577
O	+1.45665	−0.00197	+2.62660
C	+2.65553	−1.56294	+0.10793
H	+2.01356	+0.44364	−0.48932
H	+4.36615	+0.35541	+0.49184
C	+1.46656	−2.52114	+0.00140
H	+3.28038	−1.61214	−0.79448
H	+3.28491	−1.92085	+0.93016
C	+0.41567	−2.28806	−1.07119
H	+1.83140	−3.54152	−0.15435
H	+0.91360	−2.55268	+0.94750
N	+0.61222	−1.21217	−1.91341
O	−0.57371	−2.97045	−1.14504
H	−0.00993	−1.18115	−2.70793
H	+1.55252	−0.89484	−2.08454

20

∗ E = +12.139 kcal/mol ; (177) 354_152_030_064_277_159

H	+0.00000	+0.00000	+0.00000
O	+0.00000	+0.00000	+0.95961
C	+1.25708	+0.00000	+1.43996
C	+2.36533	−0.14829	+0.39698
N	+3.54743	+0.50257	+0.91755
H	+3.54115	+0.44852	+1.93093
O	+1.46698	+0.10522	+2.60687

C	+2.59287	−1.63202	+0.04466
H	+2.03393	+0.37091	−0.51978
H	+4.39351	+0.06830	+0.57365
C	+1.41448	−2.32588	−0.63050
H	+3.46263	−1.66620	−0.62040
H	+2.84739	−2.17527	+0.96118
C	+0.38135	−2.81984	+0.37803
H	+0.94173	−1.67872	−1.38341
H	+1.76503	−3.21094	−1.17434
N	−0.86255	−3.02656	−0.12349
O	+0.66571	−3.02621	+1.53340
H	−1.56965	−3.39854	+0.48745
H	−1.06907	−2.91739	−1.09871

20

∗ E = +12.199 kcal/mol ; (178) 353_158_038_291_286_308

H	+0.00000	+0.00000	+0.00000
O	+0.00000	+0.00000	+0.95982
C	+1.25596	+0.00000	+1.44329
C	+2.35739	−0.17893	+0.40440
N	+3.58550	+0.34852	+0.94551
H	+3.65212	+0.14238	+1.93768
O	+1.45911	+0.07042	+2.61496
C	+2.39828	−1.67621	+0.01083
H	+2.07332	+0.39666	−0.49081
H	+4.39959	−0.02713	+0.47638
C	+2.89724	−2.57150	+1.15109
H	+1.39821	−2.00345	−0.29987
H	+3.04122	−1.77474	−0.87184
C	+4.39310	−2.49154	+1.41710
H	+2.40116	−2.32028	+2.09300
H	+2.66040	−3.61754	+0.92312
N	+5.19311	−2.67839	+0.32278
O	+4.85436	−2.27017	+2.51116
H	+6.18101	−2.77378	+0.49132
H	+4.82511	−3.07100	−0.52532

20

∗ E = +12.413 kcal/mol ; (179) 007_070_178_065_166_145

H	+0.00000	+0.00000	+0.00000
O	+0.00000	+0.00000	+0.95979
C	+1.25758	+0.00000	+1.44883

C	+2.35304	+0.16550	+0.39492
N	+2.27493	+1.53883	−0.09573
H	+2.99989	+1.71467	−0.78000
O	+1.44833	−0.10687	+2.61931
C	+3.72905	−0.08340	+0.98874
H	+2.16592	−0.59254	−0.39224
H	+1.38298	+1.76851	−0.51514
C	+3.92687	−1.51891	+1.45825
H	+4.49030	+0.14224	+0.23372
H	+3.87498	+0.61322	+1.82019
C	+5.39577	−1.80724	+1.72294
H	+3.31794	−1.72560	+2.34293
H	+3.61231	−2.22041	+0.67311
N	+5.64329	−2.65046	+2.75573
O	+6.27919	−1.34275	+1.03649
H	+6.59811	−2.87977	+2.97198
H	+4.91251	−2.96715	+3.36502

20

∗ E = +12.752 kcal/mol ; (180) 351_168_042_280_052_061

H	+0.00000	+0.00000	+0.00000
O	+0.00000	+0.00000	+0.96003
C	+1.26208	+0.00000	+1.44581
C	+2.35111	−0.21666	+0.39689
N	+3.62528	+0.08905	+0.99720
H	+3.67544	−0.28733	+1.93985
O	+1.46198	+0.12708	+2.61052
C	+2.22518	−1.63128	−0.23320
H	+2.14801	+0.51118	−0.40580
H	+4.39762	−0.27153	+0.45331
C	+2.82662	−2.75297	+0.62053
H	+1.17265	−1.84418	−0.46746
H	+2.74566	−1.62084	−1.19705
C	+2.34571	−2.77020	+2.06420
H	+2.60294	−3.71964	+0.15610
H	+3.91265	−2.65526	+0.66993
N	+1.00878	−2.94752	+2.23443
O	+3.11125	−2.64159	+2.99173
H	+0.63524	−2.77832	+3.15433
H	+0.37276	−2.89666	+1.45869

20

∗ E = +13.296 kcal/mol ; (181) 010_074_185_303_106_212

H	+0.00000	+0.00000	+0.00000
O	+0.00000	+0.00000	+0.95988
C	+1.25832	+0.00000	+1.44754
C	+2.34248	+0.22972	+0.39659
N	+2.28784	+1.65015	+0.04536
H	+2.93445	+1.85692	−0.70588
O	+1.44893	−0.12844	+2.61597
C	+3.73760	−0.13310	+0.89611
H	+2.10972	−0.43236	−0.45848
H	+1.36551	+1.95383	−0.24134
C	+4.17025	+0.60953	+2.16433
H	+3.79755	−1.20992	+1.08624
H	+4.43092	+0.07892	+0.07439
C	+4.13036	−0.29550	+3.38688
H	+5.21241	+0.93451	+2.07213
H	+3.56990	+1.51169	+2.31368
N	+3.81470	+0.32136	+4.55779
O	+4.44893	−1.46231	+3.32848
H	+3.67752	−0.28189	+5.35242
H	+3.25990	+1.15860	+4.52505

11 Phe

11.1 Reference Z-matrix for Phe

Bond distances are given in Å; bond and dihedral angles, in degrees.

h						
o	1	oh2				
c	2	co3	1	coh3		
c	3	cc4	2	cco4	1	dih4
n	4	nc5	3	ncc5	2	dih5
h	5	hn6	4	hnc6	3	dih6
o	3	oc7	2	oco7	4	dih7
c	4	cc8	3	ccc8	5	dih8
h	4	hc9	3	hcc9	5	dih9
h	5	hn10	4	hnc10	6	dih10
c	8	cc11	4	ccc11	3	dih11
h	8	hc12	4	hcc12	11	dih12
h	8	hc13	4	hcc13	11	dih13
c	11	cc14	8	ccc14	4	dih14
c	14	cc15	11	ccc15	8	dih15
c	15	cc16	14	ccc16	11	dih16
c	16	cc17	15	ccc17	14	dih17
c	17	cc18	16	ccc18	15	dih18
h	14	hc19	11	hcc19	15	dih19
h	15	hc20	14	hcc20	16	dih20
h	16	hc21	15	hcc21	17	dih21
h	17	hc22	16	hcc22	15	dih22
h	18	hc23	17	hcc23	16	dih23

oh2	0.96845287
co3	1.35863035
coh3	111.97964821
cc4	1.50836317
cco4	110.83273624
dih4	181.24447152
nc5	1.46659491
ncc5	106.8244627
dih5	50.24171504
hn6	1.00330432
hnc6	112.65666144

dih6	178.90134575
oc7	1.20137767
oco7	122.15471569
dih7	181.30216865
cc8	1.5353853
ccc8	109.58457843
dih8	119.34568634
hc9	1.09002875
hcc9	107.05194979
dih9	238.02917571
hn10	1.00312909
hnc10	112.61763426
dih10	128.54490677
cc11	1.51509503
ccc11	111.31728936
dih11	176.84632011
hc12	1.08115628
hcc12	107.47327649
dih12	121.195537
hc13	1.08199888
hcc13	108.98967763
dih13	237.95478727
cc14	1.38812154
ccc14	120.80889288
dih14	95.26567538
cc15	1.38499202
ccc15	120.67930383
dih15	181.53503545
cc16	1.38311998
ccc16	120.17825848
dih16	−0.09430634
cc17	1.38472344
ccc17	119.57061737
dih17	−0.09741755
cc18	1.38302078
ccc18	120.12797154
dih18	0.12310724
hc19	1.0725777
hcc19	119.33600106
dih19	180.11676847

```
hc20    1.07219975
hcc20   119.76816296
dih20   180.2380849
hc21    1.07185594
hcc21   120.23853352
dih21   180.28212352
hc22    1.07218524
hcc22   120.02485411
dih22   180.38943088
hc23    1.07346692
hcc23   119.71370651
dih23   180.5253565
```

11.2 Reference energy and target torsions for Phe

- Reference M08-HX/MG3S energy: -554.8257659 hartree
- Target torsions:
  - torsion1: dih4
  - torsion2: dih5
  - torsion3: dih6
  - torsion4: dih11

11.3 Properties of Phe conformers

Table S11: Relative energy (kcal mol^{-1}), rotational constants (B1 to B3, GHz) and dipolar moment (Debye) for the conformers of Phe located at the M08-HX/MG3S level.

Conformer	Rel. Energy	B1	B2	B3	Dip. Mom.
1	0.000	0.577	0.649	1.673	4.884
2	0.814	0.594	0.681	1.608	1.322
3	1.071	0.430	0.466	2.479	5.424
4	1.285	0.430	0.457	2.483	5.200
5	1.496	0.598	0.681	1.605	1.653
6	2.035	0.426	0.465	2.496	1.915

7	2.348	0.429	0.468	2.454	2.288
8	2.386	0.570	0.639	1.770	1.531
9	2.552	0.430	0.451	2.533	2.245
10	2.745	0.417	0.474	2.373	1.313
11	2.745	0.604	0.696	1.609	2.147
12	3.146	0.618	0.710	1.590	2.112
13	3.316	0.565	0.634	1.788	1.852
14	3.381	0.564	0.617	1.728	1.544
15	3.472	0.429	0.467	2.473	2.540
16	4.059	0.549	0.602	1.779	1.639
17	4.061	0.519	0.602	1.899	4.714
18	4.096	0.477	0.551	1.944	4.616
19	4.147	0.502	0.566	1.837	2.344
20	4.291	0.420	0.468	2.407	1.851
21	4.307	0.413	0.467	2.430	2.059
22	4.591	0.488	0.553	1.976	1.738
23	4.732	0.559	0.622	1.763	2.567
24	4.867	0.600	0.686	1.644	3.945
25	4.918	0.538	0.595	1.897	3.661
26	5.535	0.442	0.518	2.105	4.836
27	5.792	0.540	0.599	1.905	4.699
28	6.019	0.591	0.677	1.640	2.993
29	7.436	0.428	0.469	2.441	4.614
30	7.732	0.417	0.476	2.343	3.173
31	7.761	0.593	0.680	1.656	4.056
32	7.903	0.617	0.627	1.692	4.079
33	9.533	0.531	0.615	1.828	3.721
34	10.423	0.518	0.582	1.761	4.734
35	10.553	0.426	0.452	2.390	3.574

11.4 Vibrational frequencies of Phe conformers

Harmonic vibrational zero point energy (ZPE) and frequencies for each conformer in cm^{-1}.

- Conformer (1); ZPE = 42055.889 cm^{-1}:

38.56	51.55	100.69	107.30	210.73	253.70
300.50	354.34	394.40	415.55	441.64	491.54
541.76	568.98	618.07	629.76	728.51	757.87
777.03	809.47	816.17	865.09	882.01	891.93
931.94	959.27	1014.01	1020.98	1031.14	1036.35
1060.12	1099.51	1119.70	1169.61	1176.75	1200.02
1225.29	1233.85	1250.25	1292.27	1330.22	1354.28
1361.88	1399.33	1437.50	1471.57	1492.12	1535.42
1650.04	1655.38	1673.52	1881.84	3046.13	3057.14
3113.43	3160.71	3179.85	3186.84	3195.69	3207.76
3532.63	3640.95	3679.33			

- Conformer (2); ZPE = 41919.382 cm^{-1}:

38.93	51.04	86.88	107.93	195.91	253.09
259.03	295.90	386.65	412.49	418.45	494.14
497.10	566.69	611.98	629.90	649.25	727.27
753.53	783.11	797.32	869.28	880.12	911.84
943.10	958.38	1010.95	1019.32	1022.07	1029.11
1061.71	1107.25	1140.12	1161.74	1169.67	1186.38
1199.15	1235.15	1250.06	1301.39	1334.76	1350.54
1358.34	1379.52	1414.52	1474.30	1493.99	1538.26
1655.70	1666.04	1677.03	1868.01	3039.94	3064.97
3118.98	3171.71	3173.97	3187.45	3196.79	3208.08
3534.06	3614.20	3844.22			

- Conformer (3); ZPE = 42005.812 cm^{-1}:

43.44	51.02	78.42	90.12	180.47	254.53
297.83	331.59	360.76	401.43	414.75	499.29
540.11	585.30	628.35	666.85	726.02	739.90
778.56	802.28	837.99	876.97	879.12	892.18
957.39	963.79	992.60	1011.76	1021.17	1030.33
1059.23	1099.38	1133.22	1161.27	1172.65	1196.66
1233.83	1241.13	1287.25	1293.10	1332.87	1350.02
1366.90	1373.72	1427.54	1473.30	1493.50	1536.87
1654.84	1662.15	1675.71	1891.80	3030.34	3045.57
3105.20	3163.17	3167.19	3186.59	3195.59	3208.19
3543.55	3624.83	3690.19			

- Conformer (4); ZPE = 42004.058 cm^{-1}:

38.31	58.82	64.60	85.32	200.74	249.58
292.85	338.38	366.86	414.96	416.50	489.62
534.91	572.15	627.92	630.47	727.16	744.24
778.85	805.25	830.45	877.89	882.67	902.90
930.31	955.47	1011.32	1021.37	1030.09	1053.57
1061.18	1104.67	1136.47	1155.43	1173.06	1199.54
1230.57	1232.57	1262.88	1294.57	1330.21	1349.64
1364.69	1395.02	1428.08	1477.45	1493.37	1539.41
1655.66	1662.15	1677.74	1889.65	3029.84	3043.80
3106.50	3162.34	3164.22	3186.64	3195.27	3208.47
3535.30	3625.69	3702.53			

- Conformer (5); ZPE = 41946.248 cm^{-1}:

21.37	46.63	90.85	105.36	200.33	254.74
293.97	301.84	387.17	417.14	422.69	495.71
525.57	560.35	589.99	629.79	664.71	729.05
757.95	782.62	795.69	865.06	883.69	894.12
939.44	961.10	1009.59	1015.54	1021.73	1029.86
1061.48	1107.48	1135.21	1165.73	1173.37	1200.00
1206.91	1235.33	1244.89	1308.84	1334.92	1356.59
1359.68	1384.53	1394.56	1470.33	1493.48	1537.17
1653.35	1662.51	1675.96	1853.09	3044.82	3068.97
3124.51	3171.13	3173.46	3186.68	3195.06	3207.29
3541.69	3626.94	3842.93			

- Conformer (6); ZPE = 41839.663 cm^{-1}:

36.98	58.05	68.34	83.03	195.69	253.06
273.96	320.70	343.43	414.19	428.83	487.43
501.21	578.02	590.52	629.55	643.55	723.83
735.94	781.36	828.21	833.84	874.59	886.73
939.96	954.18	1007.73	1021.50	1025.35	1028.71
1059.11	1107.19	1153.92	1168.17	1188.13	1196.97
1208.51	1235.71	1247.73	1301.27	1327.01	1348.91
1357.24	1367.76	1441.79	1472.58	1492.69	1536.00
1630.31	1652.89	1674.86	1865.58	2937.98	3056.43
3116.50	3161.17	3174.22	3185.70	3193.24	3206.30
3561.55	3655.24	3848.22			

- Conformer (7); ZPE = 41814.665 cm^{-1}:

39.46	54.50	72.21	85.45	174.76	251.65
254.09	306.97	347.39	389.56	414.61	491.94
514.48	578.29	598.21	628.74	659.86	724.80
748.53	781.31	833.58	836.02	874.57	882.90
923.31	954.61	1008.88	1019.51	1023.31	1028.23
1059.75	1105.19	1143.79	1164.31	1169.13	1195.80
1197.24	1235.11	1259.44	1297.70	1327.76	1344.98
1352.84	1362.81	1455.64	1475.96	1493.24	1537.16
1635.41	1653.93	1676.34	1869.69	3033.29	3043.14
3094.04	3164.99	3166.43	3186.03	3194.83	3207.69
3542.23	3635.98	3845.76			

- Conformer (8); ZPE = 41863.943 cm^{-1}:

41.35	57.47	68.28	86.33	180.73	253.48
275.87	304.30	324.12	354.91	415.49	471.82
519.74	599.14	617.29	629.60	652.58	725.79
773.60	776.66	825.77	845.43	877.05	895.38
948.77	974.71	1009.56	1022.60	1027.15	1038.49
1059.82	1098.42	1132.29	1164.16	1171.29	1182.81
1196.02	1226.08	1252.72	1301.65	1327.41	1343.58
1354.45	1375.73	1436.86	1474.67	1493.36	1537.19
1656.23	1664.24	1678.76	1855.57	3041.12	3076.45
3108.31	3167.04	3168.10	3186.79	3195.82	3208.35
3535.15	3623.63	3840.31			

- Conformer (9); ZPE = 41875.226 cm^{-1}:

38.27	59.17	60.14	81.57	197.67	254.85
316.56	320.51	330.43	412.13	416.84	463.75
538.60	559.04	595.19	630.39	639.53	727.07
748.56	781.38	828.26	859.43	877.92	883.89
954.27	957.15	1011.29	1020.90	1022.55	1029.12
1061.76	1107.90	1152.09	1170.04	1179.42	1186.54
1199.49	1235.38	1262.93	1302.77	1331.84	1344.33
1353.01	1369.89	1447.04	1468.43	1493.78	1539.11
1647.33	1656.18	1678.39	1871.51	2929.41	3062.14
3116.61	3164.84	3171.79	3186.54	3195.13	3208.01
3551.24	3639.30	3849.89			

- Conformer (10); ZPE = 41802.028 cm^{-1}:

34.42	48.49	67.28	89.11	171.19	192.05
257.50	286.52	324.22	399.49	414.67	487.71
509.19	576.01	598.73	629.48	664.65	721.42
743.92	784.54	830.56	868.49	873.09	896.97
928.86	970.51	1004.61	1013.74	1022.61	1025.14
1060.65	1100.28	1144.81	1160.40	1169.48	1184.96
1197.12	1236.68	1261.38	1300.29	1336.20	1347.90
1356.24	1363.47	1429.47	1476.11	1493.75	1537.68
1655.30	1669.09	1677.52	1861.80	3035.91	3063.06
3099.42	3165.89	3180.50	3190.06	3197.96	3207.07
3538.49	3626.02	3843.92			

- Conformer (11); ZPE = 41830.588 cm^{-1}:

30.36	68.38	85.22	107.25	188.08	212.35
252.60	307.39	379.29	409.09	417.65	496.62
504.59	574.69	602.37	630.10	658.95	725.35
755.42	780.70	799.35	833.69	873.40	885.93
933.74	958.16	997.74	1006.91	1022.50	1026.89
1061.91	1101.56	1132.39	1163.01	1169.87	1197.27
1200.58	1233.99	1247.79	1270.26	1330.52	1339.11
1352.82	1375.57	1452.55	1479.84	1494.34	1538.72
1638.60	1656.37	1679.13	1876.09	3033.79	3046.75
3098.18	3167.66	3168.64	3187.71	3197.00	3209.19
3546.20	3641.46	3847.52			

- Conformer (12); ZPE = 41877.357 cm^{-1}:

38.38	58.15	84.93	102.81	194.40	251.32
277.54	318.90	375.77	409.61	419.22	499.55
527.37	574.17	577.93	629.67	659.96	727.09
762.91	780.32	800.95	841.19	873.99	883.40
928.78	959.20	1004.68	1011.01	1021.34	1028.64
1060.69	1097.17	1121.94	1162.10	1170.85	1199.78
1217.61	1232.11	1235.42	1269.02	1329.89	1354.55
1361.25	1375.15	1444.82	1472.08	1493.31	1536.65
1652.18	1655.19	1676.19	1856.47	3046.46	3062.28
3103.82	3165.53	3166.57	3185.50	3194.02	3207.19
3544.44	3634.75	3846.59			

- Conformer (13); ZPE = 41820.196 cm^{-1}:

40.38	51.64	68.36	87.04	181.30	248.81
286.99	298.38	307.71	358.10	415.01	475.14
519.51	593.75	616.96	629.45	648.55	724.98
772.39	780.18	825.74	840.47	865.14	875.66
944.63	974.98	1008.52	1022.38	1027.02	1048.31
1059.79	1105.03	1134.97	1160.48	1172.08	1178.54
1196.11	1228.46	1247.69	1310.95	1322.74	1337.95
1352.32	1359.62	1466.26	1476.66	1494.11	1536.57
1639.09	1656.42	1678.28	1860.46	3035.25	3066.23
3093.67	3166.13	3166.91	3187.06	3195.99	3208.73
3536.68	3629.58	3842.11			

- Conformer (14); ZPE = 41869.545 cm^{-1}:

39.82	44.58	80.63	98.06	185.16	252.58
275.25	304.93	327.87	364.38	416.02	482.98
545.67	590.73	606.45	613.23	629.59	726.28
772.04	781.07	817.37	845.81	874.90	875.48
946.22	968.83	1008.97	1020.67	1022.71	1029.86
1062.28	1097.04	1131.38	1152.12	1173.33	1190.73
1199.69	1233.69	1257.20	1323.78	1333.24	1345.51
1362.18	1381.73	1394.93	1478.02	1496.23	1541.02
1654.66	1658.95	1680.03	1872.04	3046.22	3076.50
3103.60	3164.63	3172.01	3186.13	3194.92	3207.13
3541.15	3630.86	3848.07			

- Conformer (15); ZPE = 41809.924 cm^{-1}:

35.22	45.60	61.91	82.63	170.97	249.61
269.69	302.06	343.57	383.77	414.70	492.89
541.68	566.48	591.35	628.96	644.92	725.51
757.65	784.76	831.42	858.48	875.27	882.88
909.37	954.33	1009.40	1021.29	1027.48	1029.23
1059.56	1104.42	1115.26	1147.06	1170.30	1196.53
1208.59	1233.33	1261.57	1298.20	1328.22	1344.12
1357.19	1369.35	1434.07	1475.32	1492.94	1537.03
1650.51	1654.34	1676.24	1874.93	3033.36	3071.61
3091.68	3163.11	3170.91	3186.67	3195.68	3208.15
3539.36	3628.09	3849.08			

- Conformer (16); ZPE = 41885.201 cm^{-1}:

28.65	47.33	72.15	129.52	181.66	246.85
284.57	291.83	331.07	359.65	413.47	474.97
540.68	590.52	607.04	613.75	630.37	721.18
748.22	786.39	821.72	834.99	871.74	879.37
930.53	987.28	1007.55	1021.72	1027.18	1031.57
1065.77	1105.50	1141.39	1164.77	1171.92	1191.26
1209.29	1226.53	1272.69	1315.07	1337.40	1351.49
1368.93	1388.24	1412.10	1465.49	1494.45	1543.32
1653.51	1659.89	1681.08	1866.79	3036.39	3073.94
3090.48	3169.53	3181.09	3189.70	3197.39	3208.65
3542.34	3632.92	3847.58			

- Conformer (17); ZPE = 41971.799 cm^{-1}:

34.71	42.24	84.29	102.75	184.41	286.74
301.89	327.01	348.45	359.36	413.70	498.58
532.01	605.64	629.82	654.70	720.77	750.84
761.63	777.14	826.48	846.15	870.40	901.01
942.96	981.13	1005.53	1007.42	1023.21	1026.93
1062.65	1105.78	1120.08	1149.87	1171.58	1201.26
1228.99	1235.87	1258.89	1317.12	1334.07	1351.97
1364.09	1374.08	1417.38	1481.17	1496.64	1542.19
1656.53	1660.78	1682.85	1900.06	3024.94	3039.34
3078.54	3162.35	3168.65	3187.29	3197.14	3209.15
3553.73	3638.67	3722.00			

- Conformer (18); ZPE = 41946.139 cm^{-1}:

27.59	57.69	83.66	102.98	203.96	276.33
298.67	340.08	346.77	381.02	413.47	479.04
556.34	573.57	606.57	630.76	720.33	744.73
755.63	774.28	826.43	845.99	870.80	878.77
946.80	948.86	1005.95	1022.13	1025.43	1060.76
1084.46	1100.74	1134.84	1168.94	1175.55	1197.03
1200.81	1231.12	1233.71	1318.42	1328.38	1347.77
1363.68	1389.05	1428.95	1467.40	1493.74	1537.87
1653.65	1658.84	1677.27	1892.89	3029.73	3038.61
3093.16	3151.70	3180.83	3188.76	3198.26	3207.48
3540.83	3634.37	3738.06			

- Conformer (19); ZPE = 41845.058 cm^{-1}:

38.84	49.74	92.11	100.23	196.31	266.67
276.09	286.29	346.05	362.36	415.41	462.34
546.09	575.89	607.59	617.09	630.30	723.24
762.49	776.46	815.17	835.51	873.21	888.87
948.69	974.81	1007.62	1023.41	1026.20	1041.18
1060.60	1103.75	1131.27	1164.49	1172.40	1195.86
1201.78	1231.75	1250.55	1309.74	1331.65	1333.63
1350.45	1381.78	1454.91	1470.87	1493.16	1536.96
1647.72	1657.37	1677.72	1879.21	2963.01	3041.22
3107.95	3159.08	3182.02	3190.19	3198.32	3208.61
3550.44	3638.62	3846.76			

- Conformer (20); ZPE = 41779.343 cm^{-1}:

23.40	30.18	61.34	85.78	170.56	241.00
254.93	278.00	325.84	387.87	413.73	488.89
541.27	567.07	597.44	629.48	648.10	720.63
758.18	785.34	819.56	857.04	870.36	893.62
919.67	962.64	994.49	1005.48	1022.57	1025.08
1060.26	1096.66	1138.46	1155.01	1169.42	1191.97
1197.17	1235.47	1278.22	1294.90	1334.52	1353.41
1357.16	1378.67	1395.71	1474.95	1493.34	1537.99
1655.16	1661.42	1678.03	1866.62	3033.16	3078.64
3092.73	3167.17	3179.55	3187.54	3196.34	3207.00
3547.82	3636.84	3847.81			

- Conformer (21); ZPE = 41802.676 cm^{-1}:

11.67	31.77	59.23	86.50	197.17	236.44
254.94	299.72	321.78	406.62	414.21	491.80
524.49	566.17	600.84	629.27	651.03	721.63
741.07	784.14	817.97	856.64	870.87	898.58
927.27	969.37	999.10	1006.18	1022.35	1025.67
1060.57	1097.97	1140.90	1169.39	1170.55	1198.52
1204.93	1237.21	1275.37	1295.10	1334.71	1355.63
1358.99	1379.20	1395.44	1478.34	1493.90	1538.50
1655.13	1665.41	1676.90	1858.40	3026.62	3045.81
3105.11	3163.39	3178.34	3190.26	3197.47	3206.93
3549.23	3635.14	3841.49			

- Conformer (22); ZPE = 41822.291 cm^{-1}:

34.39	45.49	74.43	106.55	201.24	254.17
291.52	330.94	343.72	357.78	414.52	487.90
500.99	583.07	604.97	630.58	643.79	721.77
743.12	768.03	820.02	833.01	870.39	873.53
940.24	965.29	1006.46	1022.70	1024.29	1051.60
1061.41	1105.71	1143.59	1167.34	1187.49	1197.77
1201.67	1229.51	1251.23	1301.13	1330.03	1347.11
1348.24	1376.63	1447.28	1475.44	1492.83	1538.73
1636.99	1656.43	1679.49	1860.55	2945.38	3044.16
3097.10	3158.11	3173.21	3185.36	3194.57	3207.64
3559.30	3654.45	3842.18			

- Conformer (23); ZPE = 41787.357 cm^{-1}:

27.38	58.09	66.63	97.90	182.60	243.75
275.95	294.07	312.28	363.63	413.64	485.73
537.08	581.25	601.14	609.39	629.44	722.99
770.23	781.62	814.99	839.65	857.28	870.08
940.29	963.89	1004.89	1021.85	1026.12	1056.21
1060.84	1095.36	1107.56	1157.18	1171.31	1172.82
1199.11	1233.87	1251.90	1315.65	1328.02	1346.96
1360.92	1363.59	1425.28	1474.34	1495.41	1538.74
1649.01	1654.50	1679.63	1889.85	3036.53	3076.37
3095.37	3162.64	3166.87	3186.26	3194.39	3208.09
3543.20	3634.58	3848.53			

- Conformer (24); ZPE = 41848.293 cm^{-1}:

44.30	54.12	87.33	101.18	199.79	260.87
314.05	381.27	394.49	412.60	420.50	494.96
502.56	531.32	585.70	620.15	629.65	653.38
728.89	760.24	781.21	808.56	869.42	884.29
937.68	959.36	992.39	1013.42	1021.63	1031.89
1060.98	1106.96	1119.22	1155.50	1170.96	1195.94
1200.84	1232.73	1252.51	1299.58	1332.33	1352.59
1356.03	1374.95	1376.16	1473.58	1494.22	1536.92
1633.91	1653.44	1677.06	1883.20	3059.45	3068.26
3122.47	3167.24	3170.22	3187.37	3196.76	3208.86
3570.73	3686.65	3841.72			

- Conformer (25); ZPE = 41836.057 cm^{-1}:

32.50	49.64	84.70	104.16	166.57	210.49
288.10	305.85	326.98	349.22	415.60	482.75
510.25	552.74	605.80	628.83	661.22	728.80
746.16	775.07	826.18	841.95	880.84	917.78
952.46	985.55	1012.68	1022.28	1030.85	1035.01
1061.49	1101.93	1148.12	1173.23	1178.96	1199.68
1200.23	1228.11	1245.43	1302.81	1323.00	1345.00
1362.66	1371.51	1412.90	1480.34	1495.28	1539.03
1654.92	1673.61	1677.67	1883.60	3023.10	3044.58
3098.37	3163.70	3177.50	3189.73	3199.08	3210.49
3535.61	3612.68	3826.70			

- Conformer (26); ZPE = 42024.895 cm^{-1}:

18.76	38.69	82.21	125.11	190.53	236.65
311.37	329.35	351.45	375.80	414.43	485.25
536.83	589.02	629.73	673.39	725.27	744.04
781.76	791.06	837.46	839.50	880.23	915.85
949.29	974.34	1009.67	1015.13	1021.92	1036.75
1064.68	1110.05	1123.65	1171.00	1181.50	1207.28
1222.63	1235.17	1267.28	1313.55	1339.67	1358.20
1363.46	1388.09	1422.05	1475.21	1494.76	1541.66
1653.45	1659.35	1679.56	1887.90	3029.50	3044.85
3076.76	3168.18	3179.76	3188.09	3196.72	3207.83
3550.19	3636.64	3700.27			

- Conformer (27); ZPE = 41812.462 cm^{-1}:

36.68	49.76	85.25	105.82	181.96	205.99
296.49	326.30	333.03	351.20	415.46	492.26
514.29	532.27	602.35	628.62	663.73	728.15
747.74	774.62	817.72	835.04	846.44	879.62
947.59	986.71	1011.97	1021.98	1031.00	1035.25
1061.37	1109.05	1166.66	1176.41	1183.30	1191.72
1200.76	1232.05	1249.05	1308.00	1324.92	1337.94
1349.29	1364.76	1440.46	1476.82	1495.63	1538.63
1641.15	1654.70	1677.09	1888.60	2990.24	3032.48
3090.56	3161.66	3176.04	3189.14	3198.23	3209.89
3547.76	3643.41	3831.86			

- Conformer (28); ZPE = 41832.934 cm^{-1}:

38.68	65.10	80.36	106.24	196.20	239.30
253.72	294.96	386.51	410.29	417.36	487.72
499.00	503.25	594.55	628.74	636.56	724.88
756.26	780.94	803.63	866.89	878.28	910.61
942.31	956.78	1008.35	1011.34	1021.77	1028.93
1060.73	1110.30	1139.83	1157.69	1169.19	1186.66
1201.58	1234.92	1243.38	1287.97	1328.68	1349.46
1356.50	1373.31	1406.16	1468.24	1495.38	1537.75
1653.78	1669.11	1678.96	1900.22	3013.93	3043.50
3105.42	3163.27	3173.40	3188.28	3197.08	3209.67
3533.71	3613.14	3885.16			

- Conformer (29); ZPE = 41782.175 cm^{-1}:

39.41	54.40	75.96	86.03	175.20	253.16
284.38	310.62	349.13	391.30	414.66	489.23
507.96	522.42	581.42	628.91	674.96	724.11
738.26	778.54	837.12	839.32	872.93	879.42
922.84	953.41	1008.04	1017.09	1021.94	1028.72
1059.77	1105.39	1146.82	1162.17	1170.07	1197.15
1202.97	1235.64	1250.83	1304.25	1325.45	1334.01
1346.01	1359.28	1437.82	1472.18	1493.53	1537.30
1634.26	1654.39	1676.50	1900.98	2986.24	3029.26
3086.61	3159.99	3168.23	3187.27	3196.10	3208.63
3542.41	3636.75	3895.23			

- Conformer (30); ZPE = 41773.556 cm^{-1}:

27.12	51.34	74.28	93.51	186.27	209.21
260.97	291.29	321.53	405.50	414.88	478.94
497.96	517.41	576.64	629.52	677.07	722.03
734.62	782.78	836.38	869.98	883.89	893.74
930.42	972.15	1005.67	1013.20	1022.38	1026.92
1060.61	1101.90	1144.70	1160.75	1170.08	1180.74
1198.16	1235.70	1262.49	1297.62	1331.96	1336.13
1352.28	1361.16	1421.65	1474.65	1493.25	1537.43
1653.88	1669.62	1677.29	1894.61	3022.16	3035.53
3083.43	3159.91	3182.53	3191.42	3200.40	3208.64
3532.42	3620.20	3886.24			

- Conformer (31); ZPE = 41794.859 cm^{-1}:

36.57	69.52	88.58	108.80	189.42	214.07
250.69	317.28	385.25	408.60	418.28	492.06
504.25	510.75	596.66	629.36	647.53	724.65
754.60	779.82	806.17	851.09	873.08	889.48
942.39	957.52	987.95	1007.69	1022.36	1028.77
1061.64	1106.29	1143.24	1163.21	1170.76	1201.81
1205.96	1234.54	1251.97	1264.28	1314.98	1331.81
1355.21	1377.30	1436.18	1476.06	1495.21	1539.32
1641.49	1655.91	1680.19	1902.81	2981.54	3029.42
3080.01	3163.68	3164.27	3187.37	3197.54	3209.64
3544.92	3638.05	3889.88			

- Conformer (32); ZPE = 41781.948 cm^{-1}:

33.26	42.19	68.68	147.05	211.12	258.74
289.41	305.49	359.32	411.46	413.11	465.89
479.57	506.34	577.24	620.05	633.73	726.57
756.05	769.85	795.42	852.21	881.79	907.28
943.10	962.53	1016.17	1019.24	1022.47	1037.22
1062.21	1104.16	1132.96	1168.30	1197.68	1200.99
1213.23	1225.41	1243.65	1306.70	1317.25	1338.81
1359.36	1381.46	1423.88	1474.05	1494.89	1535.86
1633.81	1652.05	1675.06	1882.52	2939.59	3029.02
3068.61	3159.81	3183.86	3192.47	3199.94	3208.55
3547.73	3640.81	3856.66			

- Conformer (33); ZPE = 41678.292 cm^{-1}:

31.78	34.75	79.13	89.78	181.71	257.13
298.57	308.21	331.75	354.91	413.28	491.35
498.09	534.52	588.25	615.84	629.79	676.62
720.81	754.92	775.25	833.45	848.28	871.42
943.66	974.96	990.93	1006.31	1023.06	1026.46
1061.67	1099.92	1132.87	1154.20	1170.88	1200.31
1208.44	1225.88	1240.95	1323.60	1330.74	1341.79
1355.47	1364.17	1369.94	1478.56	1496.07	1540.75
1640.87	1660.10	1681.49	1901.59	3012.29	3064.48
3088.01	3161.62	3167.05	3187.49	3197.49	3209.31
3569.01	3675.76	3858.86			

- Conformer (34); ZPE = 41729.445 cm^{-1}:

34.11	39.76	94.40	98.17	188.20	204.79
272.01	276.48	344.46	366.20	414.65	462.16
507.27	548.87	605.01	622.91	629.77	724.30
759.92	774.87	806.34	832.73	868.68	875.77
950.11	968.97	1009.59	1022.31	1028.18	1042.88
1059.92	1101.54	1123.40	1157.46	1170.82	1196.59
1207.00	1231.40	1240.47	1299.95	1312.74	1328.80
1352.24	1379.75	1443.89	1467.66	1493.09	1536.59
1652.36	1655.36	1675.24	1907.97	2924.84	3043.16
3110.23	3158.86	3180.43	3187.17	3196.06	3207.20
3549.54	3640.02	3893.26			

- Conformer (35); ZPE = 41782.255 cm^{-1}:

24.95	37.95	67.40	87.72	196.26	243.92
279.54	282.04	303.67	382.91	414.42	474.41
503.82	556.65	572.69	629.53	656.20	721.90
777.68	781.03	832.30	852.03	870.28	904.78
919.42	959.69	1005.72	1013.81	1022.66	1027.43
1061.38	1100.39	1143.16	1153.15	1172.18	1199.35
1204.54	1238.33	1280.55	1283.16	1336.12	1342.30
1357.24	1370.00	1398.11	1480.25	1495.03	1538.98
1656.14	1657.98	1677.99	1893.27	2988.55	3063.69
3081.22	3168.43	3182.18	3190.76	3198.71	3208.77
3532.59	3624.90	3882.32			

11.5 Cartesian coordinates of Phe conformers

Each line starting by an asterisk contains the relative energy of the corresponding conformer and the dihedral angle for each of the target torsions (separated by underscores). Cartesian coordinates are given in Å.

```
23
 * E =   +0.000  kcal/mol  ;  (1)  357_015_092_287
 H    +0.00000    +0.00000    +0.00000
 O    +0.00000    +0.00000    +0.97255
 C    +1.26972    +0.00000    +1.35535
 C    +2.28463    -0.08403    +0.20127
 N    +1.62227    +0.20913    -1.06536
 H    +1.68215    +1.19907    -1.28464
```

O	+1.60314	+0.02462	+2.50502
C	+3.52287	+0.77582	+0.51531
H	+2.59078	−1.13756	+0.18503
H	+2.02569	−0.30729	−1.83444
C	+3.22671	+2.24936	+0.40071
H	+3.85484	+0.52781	+1.52748
H	+4.32126	+0.50321	−0.18296
C	+2.61414	+2.93891	+1.44789
C	+2.29607	+4.28308	+1.31393
C	+2.58303	+4.95876	+0.13422
C	+3.19634	+4.28427	−0.91188
C	+3.51490	+2.93861	−0.77632
H	+2.39109	+2.41150	+2.37165
H	+1.82274	+4.80748	+2.13827
H	+2.33445	+6.01045	+0.03335
H	+3.43342	+4.80659	−1.83369
H	+4.01109	+2.41435	−1.59094

23

* E = +0.814 kcal/mol ; (2) 181_179_303_296

H	+0.00000	+0.00000	+0.00000
O	+0.00000	+0.00000	+0.96399
C	+1.28092	+0.00000	+1.37470
C	+1.40011	−0.03650	+2.89499
N	+2.77101	−0.06522	+3.34917
H	+3.25718	−0.86031	+2.95181
O	+2.20303	+0.00991	+0.61198
C	+0.64336	+1.15066	+3.52421
H	+0.89654	−0.95683	+3.21890
H	+3.25758	+0.75954	+3.01042
C	+1.24557	+2.47523	+3.13114
H	−0.40581	+1.10634	+3.21863
H	+0.69251	+1.02154	+4.60906
C	+0.81589	+3.13630	+1.98192
C	+1.39934	+4.33400	+1.59262
C	+2.42317	+4.88783	+2.34942
C	+2.85718	+4.23963	+3.49822
C	+2.27142	+3.04195	+3.88564
H	+0.00632	+2.70763	+1.39526
H	+1.05082	+4.83920	+0.69710
H	+2.87893	+5.82539	+2.04730

H	+3.65329	+4.67012	+4.09791
H	+2.60793	+2.53256	+4.78568

23

* E = +1.071 kcal/mol ; (3) 003_343_153_176

H	+0.00000	+0.00000	+0.00000
O	+0.00000	+0.00000	+0.97200
C	+1.27301	+0.00000	+1.35884
C	+2.28506	+0.08098	+0.21194
N	+1.65193	−0.27027	−1.05373
H	+2.12468	+0.17565	−1.83220
O	+1.60768	−0.01506	+2.50626
C	+2.80385	+1.52879	+0.16221
H	+3.12129	−0.57731	+0.48191
H	+1.67253	−1.26894	−1.21896
C	+3.87559	+1.69561	−0.88233
H	+1.96087	+2.19680	−0.05294
H	+3.18238	+1.77981	+1.15767
C	+3.60514	+2.32653	−2.09328
C	+4.58601	+2.43494	−3.07299
C	+5.85064	+1.91181	−2.84934
C	+6.13198	+1.28207	−1.64182
C	+5.15116	+1.17436	−0.66826
H	+2.61697	+2.74856	−2.26440
H	+4.36057	+2.93343	−4.01065
H	+6.61907	+1.99784	−3.61084
H	+7.12250	+0.87744	−1.45821
H	+5.37463	+0.68692	+0.27844

23

* E = +1.285 kcal/mol ; (4) 356_019_087_174

H	+0.00000	+0.00000	+0.00000
O	+0.00000	+0.00000	+0.97138
C	+1.27176	+0.00000	+1.36126
C	+2.28448	−0.09298	+0.21186
N	+1.65235	+0.29715	−1.04513
H	+1.70705	+1.30227	−1.17687
O	+1.59860	+0.03716	+2.51051
C	+3.56769	+0.67182	+0.56073
H	+2.52843	−1.16150	+0.13300
H	+2.12168	−0.12732	−1.83512
C	+4.61750	+0.46595	−0.49769

H +3.32682 +1.73800 +0.65855
H +3.91543 +0.33244 +1.54063
C +4.85311 +1.42799 −1.47545
C +5.78273 +1.20585 −2.48439
C +6.48854 +0.01219 −2.52773
C +6.26273 −0.95564 −1.55601
C +5.33406 −0.72890 −0.55106
H +4.31045 +2.37021 −1.43668
H +5.95816 +1.96962 −3.23573
H +7.21720 −0.16297 −3.31265
H +6.81727 −1.88871 −1.57925
H +5.16549 −1.48489 +0.21335
23
∗ E = +1.496 kcal/mol ; (5) 180_004_302_292
H +0.00000 +0.00000 +0.00000
O +0.00000 +0.00000 +0.96419
C +1.27669 +0.00000 +1.36896
C +1.43431 −0.00629 +2.89006
N +0.21295 −0.09676 +3.65944
H −0.41639 +0.66372 +3.43596
O +2.19742 +0.01250 +0.59976
C +2.40343 −1.14135 +3.27221
H +1.92871 +0.94416 +3.12854
H −0.27580 −0.95963 +3.44459
C +1.80860 −2.49903 +2.99677
H +3.32851 −1.01029 +2.70424
H +2.62672 −1.03522 +4.33732
C +1.95934 −3.09937 +1.74687
C +1.37388 −4.32930 +1.47838
C +0.62902 −4.97631 +2.45567
C +0.47556 −4.38888 +3.70453
C +1.06215 −3.15900 +3.97189
H +2.54932 −2.59533 +0.98483
H +1.50558 −4.78780 +0.50302
H +0.17448 −5.93962 +2.24684
H −0.09966 −4.89221 +4.47568
H +0.94623 −2.69760 +4.94998
23
∗ E = +2.035 kcal/mol ; (6) 183_193_200_177
H +0.00000 +0.00000 +0.00000

O	+0.00000	+0.00000	+0.96377
C	+1.27726	+0.00000	+1.37239
C	+1.40276	−0.06209	+2.88081
N	+2.77021	+0.24383	+3.23918
H	+2.99129	−0.09053	+4.16746
O	+2.20057	+0.00716	+0.60923
C	+0.43062	+0.90279	+3.58179
H	+1.09288	−1.09139	+3.14505
H	+3.41674	−0.15240	+2.56824
C	+0.54341	+0.76590	+5.07751
H	+0.68329	+1.92297	+3.27406
H	−0.59083	+0.68695	+3.25608
C	+1.41414	+1.57287	+5.80608
C	+1.55107	+1.40792	+7.17837
C	+0.81923	+0.43078	+7.83987
C	−0.05072	−0.38022	+7.12229
C	−0.18521	−0.21236	+5.75125
H	+1.99084	+2.33168	+5.28340
H	+2.23109	+2.04686	+7.73352
H	+0.92317	+0.30363	+8.91276
H	−0.63023	−1.14275	+7.63366
H	−0.87221	−0.84397	+5.19168

23

∗ E = +2.348 kcal/mol ; (7) 177_159_041_179

H	+0.00000	+0.00000	+0.00000
O	+0.00000	+0.00000	+0.96388
C	+1.27526	+0.00000	+1.37766
C	+1.38479	+0.08374	+2.88325
N	+2.67967	−0.41586	+3.27931
H	+3.39402	−0.09168	+2.63698
O	+2.20573	−0.00710	+0.62340
C	+1.09178	+1.55569	+3.28258
H	+0.59460	−0.54404	+3.31504
H	+2.91376	−0.12518	+4.22046
C	+1.15028	+1.72654	+4.77564
H	+1.83475	+2.19961	+2.79695
H	+0.10168	+1.83528	+2.90503
C	+2.28182	+2.25056	+5.39444
C	+2.34978	+2.35678	+6.77874
C	+1.28361	+1.93766	+7.56094

C	+0.14893	+1.41255	+6.95340
C	+0.08556	+1.30753	+5.57234
H	+3.11475	+2.58696	+4.78123
H	+3.23799	+2.77079	+7.24595
H	+1.33368	+2.02166	+8.64179
H	−0.69054	+1.08632	+7.55958
H	−0.80449	+0.89884	+5.09837

23

∗ E = +2.386 kcal/mol ; (8) 175_135_293_055

H	+0.00000	+0.00000	+0.00000
O	+0.00000	+0.00000	+0.96460
C	+1.26843	+0.00000	+1.38345
C	+1.40290	+0.11137	+2.89740
N	+2.37795	−0.81351	+3.44200
H	+2.06833	−1.77304	+3.34704
O	+2.20736	−0.05567	+0.63753
C	+1.84562	+1.55137	+3.21405
H	+0.42329	−0.07583	+3.34943
H	+3.24807	−0.72677	+2.92597
C	+0.91709	+2.58491	+2.63364
H	+1.91105	+1.63885	+4.30243
H	+2.85596	+1.68095	+2.80684
C	−0.25260	+2.93941	+3.30103
C	−1.13770	+3.85121	+2.74429
C	−0.86357	+4.42030	+1.50704
C	+0.29902	+4.07283	+0.83261
C	+1.18269	+3.15993	+1.39336
H	−0.46631	+2.49745	+4.27189
H	−2.04329	+4.12156	+3.27862
H	−1.55346	+5.13629	+1.07168
H	+0.52167	+4.51737	−0.13265
H	+2.09294	+2.88373	+0.86499

23

∗ E = +2.552 kcal/mol ; (9) 179_044_184_176

H	+0.00000	+0.00000	+0.00000
O	+0.00000	+0.00000	+0.96367
C	+1.27773	+0.00000	+1.37767
C	+1.37926	+0.02095	+2.89128
N	+0.42056	−0.91386	+3.45911
H	+0.43869	−0.85835	+4.47080

O	+2.20204	+0.02173	+0.61779
C	+2.80062	−0.33607	+3.32947
H	+1.18290	+1.07211	+3.17951
H	−0.51977	−0.71110	+3.14415
C	+2.94476	−0.22353	+4.82437
H	+3.01339	−1.35728	+2.99751
H	+3.50220	+0.33061	+2.81987
C	+2.80738	−1.34209	+5.64304
C	+2.89121	−1.22512	+7.02504
C	+3.11303	+0.01545	+7.60673
C	+3.25152	+1.13800	+6.79969
C	+3.16714	+1.01690	+5.42017
H	+2.63340	−2.31303	+5.18523
H	+2.78564	−2.10732	+7.64906
H	+3.18218	+0.10794	+8.68591
H	+3.43112	+2.11066	+7.24744
H	+3.28391	+1.89593	+4.78960

23

* E = +2.745 kcal/mol ; (10) 178_167_283_166

H	+0.00000	+0.00000	+0.00000
O	+0.00000	+0.00000	+0.96400
C	+1.27500	+0.00000	+1.38154
C	+1.38548	+0.05061	+2.89875
N	+2.71690	−0.25606	+3.37113
H	+2.89936	−1.25057	+3.32894
O	+2.20570	−0.01008	+0.62655
C	+0.95356	+1.45608	+3.37475
H	+0.67320	−0.67556	+3.30792
H	+3.39899	+0.18945	+2.76507
C	+0.69962	+1.48916	+4.85794
H	+1.75072	+2.15957	+3.10427
H	+0.04867	+1.75593	+2.83657
C	+1.74516	+1.64288	+5.76488
C	+1.50033	+1.63728	+7.13083
C	+0.20629	+1.47492	+7.60897
C	−0.84253	+1.31986	+6.71301
C	−0.59419	+1.32759	+5.34702
H	+2.75815	+1.75420	+5.38993
H	+2.32484	+1.75963	+7.82654
H	+0.01567	+1.47279	+8.67759

H −1.85773 +1.19732 +7.07780
H −1.41792 +1.21196 +4.64585
23
* E = +2.745 kcal/mol ; (11) 179_167_041_300
H +0.00000 +0.00000 +0.00000
O +0.00000 +0.00000 +0.96374
C +1.28037 +0.00000 +1.37236
C +1.38836 +0.03514 +2.88240
N +2.75197 −0.27282 +3.24476
H +3.39768 +0.19228 +2.61587
O +2.20392 +0.01084 +0.61285
C +0.85143 +1.40274 +3.38946
H +0.71589 −0.74148 +3.26936
H +2.95622 +0.01157 +4.19359
C +1.62715 +2.56707 +2.83687
H −0.20678 +1.48582 +3.12007
H +0.91351 +1.38955 +4.48358
C +1.25584 +3.15002 +1.62675
C +1.99581 +4.18955 +1.08224
C +3.12217 +4.66215 +1.74269
C +3.50006 +4.09270 +2.95100
C +2.75609 +3.05286 +3.49212
H +0.36869 +2.78484 +1.11322
H +1.69139 +4.63438 +0.13995
H +3.70136 +5.47642 +1.31913
H +4.37525 +4.46200 +3.47661
H +3.04672 +2.61848 +4.44607
23
* E = +3.146 kcal/mol ; (12) 184_344_058_298
H +0.00000 +0.00000 +0.00000
O +0.00000 +0.00000 +0.96396
C +1.27105 +0.00000 +1.37216
C +1.42747 −0.10273 +2.87883
N +0.18632 +0.18719 +3.56409
H −0.55189 −0.43875 +3.26158
O +2.20015 +0.01600 +0.61269
C +2.05581 −1.49275 +3.17592
H +2.16738 +0.65808 +3.15155
H +0.29873 +0.08413 +4.56475
C +1.16275 −2.62078 +2.73549

H +3.02175 −1.54852 +2.66360
H +2.23991 −1.54882 +4.25459
C +1.25903 −3.13165 +1.44123
C +0.39858 −4.13079 +1.00868
C −0.57338 −4.63361 +1.86432
C −0.67630 −4.13547 +3.15584
C +0.18732 −3.13667 +3.58633
H +2.02235 −2.73883 +0.77269
H +0.49060 −4.52314 +0.00050
H −1.24452 −5.41725 +1.52738
H −1.42790 −4.52966 +3.83292
H +0.11477 −2.75809 +4.60362
23
* E = +3.316 kcal/mol ; (13) 176_133_050_058
H +0.00000 +0.00000 +0.00000
O +0.00000 +0.00000 +0.96452
C +1.26601 +0.00000 +1.38090
C +1.40452 +0.10864 +2.88746
N +2.35833 −0.90023 +3.30863
H +3.19433 −0.83507 +2.73686
O +2.20721 −0.03704 +0.63709
C +1.83270 +1.56197 +3.20566
H +0.42708 −0.08890 +3.34062
H +2.62379 −0.76660 +4.27628
C +0.85867 +2.58179 +2.67981
H +1.93365 +1.64879 +4.29364
H +2.82366 +1.71593 +2.76240
C −0.29506 +2.89636 +3.39323
C −1.22432 +3.78969 +2.87976
C −1.01058 +4.38017 +1.64089
C +0.13568 +4.07202 +0.92050
C +1.06304 +3.17724 +1.43721
H −0.46250 +2.43781 +4.36556
H −2.11722 +4.02844 +3.44929
H −1.73490 +5.08179 +1.23945
H +0.31080 +4.53335 −0.04667
H +1.96022 +2.93121 +0.87266
23
* E = +3.381 kcal/mol ; (14) 178_294_299_055
H +0.00000 +0.00000 +0.00000

O	+0.00000	+0.00000	+0.96367
C	+1.28341	+0.00000	+1.37608
C	+1.38275	+0.04297	+2.89546
N	+0.88958	+1.29714	+3.44432
H	+1.42417	+2.08783	+3.10634
O	+2.20488	+0.00740	+0.61286
C	+0.61248	−1.12615	+3.51977
H	+2.44839	−0.05174	+3.12730
H	−0.08016	+1.44075	+3.18474
C	+1.06355	−2.45736	+2.97716
H	+0.76142	−1.06953	+4.60259
H	−0.45672	−0.98214	+3.32501
C	+2.26524	−3.01890	+3.40411
C	+2.70990	−4.22396	+2.88155
C	+1.95600	−4.88575	+1.92012
C	+0.75829	−4.33527	+1.48705
C	+0.31707	−3.12727	+2.01228
H	+2.85445	−2.50457	+4.16047
H	+3.64678	−4.65042	+3.22639
H	+2.30211	−5.82976	+1.51141
H	+0.16307	−4.84881	+0.73822
H	−0.62118	−2.69364	+1.67430

23

∗ E = +3.472 kcal/mol ; (15) 182_321_058_176

H	+0.00000	+0.00000	+0.00000
O	+0.00000	+0.00000	+0.96367
C	+1.27760	+0.00000	+1.37825
C	+1.38601	−0.05637	+2.88667
N	+0.36469	+0.76520	+3.50524
H	−0.56093	+0.46237	+3.22364
O	+2.20453	−0.02385	+0.62128
C	+1.36298	−1.55392	+3.29239
H	+2.37022	+0.35253	+3.13761
H	+0.42544	+0.69828	+4.51473
C	+1.56148	−1.70547	+4.77504
H	+0.39877	−1.98070	+2.99015
H	+2.15215	−2.08074	+2.74355
C	+0.47848	−1.89290	+5.62959
C	+0.66370	−1.97379	+7.00457
C	+1.93841	−1.86508	+7.54149

C	+3.02666	−1.67662	+6.69769
C	+2.83729	−1.59611	+5.32619
H	−0.52023	−1.98511	+5.20885
H	−0.19091	−2.12459	+7.65691
H	+2.08574	−1.93024	+8.61476
H	+4.02722	−1.59519	+7.11098
H	+3.69081	−1.45118	+4.66712

23

∗ E = +4.059 kcal/mol ; (16) 179_292_299_050

H	+0.00000	+0.00000	+0.00000
O	+0.00000	+0.00000	+0.96361
C	+1.27748	+0.00000	+1.38180
C	+1.36829	+0.01926	+2.90221
N	+0.89935	+1.27879	+3.46219
H	+1.45660	+2.06019	+3.13984
O	+2.20772	+0.02392	+0.62752
C	+0.56039	−1.12550	+3.51300
H	+2.43079	−0.08489	+3.14139
H	−0.06430	+1.44838	+3.19582
C	+0.83537	−2.48791	+2.92204
H	+0.75863	−1.12289	+4.59100
H	−0.50486	−0.89398	+3.39110
C	−0.17987	−3.44263	+2.89482
C	+0.04597	−4.71204	+2.38523
C	+1.29733	−5.04740	+1.88334
C	+2.31329	−4.10346	+1.89549
C	+2.08464	−2.83364	+2.41141
H	−1.16280	−3.18055	+3.27901
H	−0.75873	−5.44082	+2.37431
H	+1.47663	−6.03856	+1.47911
H	+3.29225	−4.35212	+1.49760
H	+2.89284	−2.10745	+2.40135

23

∗ E = +4.061 kcal/mol ; (17) 009_330_159_064

H	+0.00000	+0.00000	+0.00000
O	+0.00000	+0.00000	+0.97047
C	+1.27627	+0.00000	+1.36597
C	+2.27691	+0.21376	+0.22666
N	+1.71593	−0.31589	−1.01276
H	+2.15765	+0.09622	−1.82479

O	+1.59787	−0.10239	+2.51045
C	+2.50271	+1.72847	+0.05042
H	+3.21908	−0.25481	+0.53764
H	+1.82019	−1.32113	−1.07709
C	+3.13462	+2.38941	+1.24583
H	+3.14706	+1.86319	−0.82776
H	+1.53687	+2.19412	−0.18244
C	+4.51976	+2.42782	+1.37520
C	+5.11102	+3.00993	+2.48716
C	+4.31809	+3.56193	+3.48446
C	+2.93546	+3.52808	+3.36375
C	+2.34860	+2.94509	+2.25022
H	+5.14197	+1.99981	+0.59181
H	+6.19275	+3.03483	+2.57466
H	+4.77780	+4.01852	+4.35522
H	+2.31038	+3.95403	+4.14210
H	+1.26492	+2.90999	+2.16246

23

∗ E = +4.096 kcal/mol ; (18) 351_032_079_081

H	+0.00000	+0.00000	+0.00000
O	+0.00000	+0.00000	+0.96953
C	+1.27203	+0.00000	+1.36759
C	+2.27985	−0.21718	+0.23069
N	+1.73001	+0.37210	−0.98887
H	+1.85974	+1.37820	−0.99871
O	+1.58734	+0.14063	+2.51110
C	+3.68715	+0.25220	+0.63710
H	+2.30848	−1.30475	+0.07348
H	+2.17992	−0.00132	−1.81419
C	+4.43531	−0.75994	+1.47121
H	+4.24991	+0.43660	−0.28574
H	+3.60863	+1.20303	+1.17549
C	+5.02564	−1.85806	+0.84869
C	+5.70781	−2.81459	+1.58688
C	+5.81204	−2.67926	+2.96493
C	+5.22953	−1.58698	+3.59380
C	+4.54281	−0.63508	+2.85255
H	+4.95780	−1.95935	−0.23336
H	+6.16402	−3.66264	+1.08554
H	+6.34696	−3.42319	+3.54679

H +5.30465 −1.47711 +4.67120
H +4.06324 +0.20441 +3.34642
23
* E = +4.147 kcal/mol ; (19) 182_063_181_075
H +0.00000 +0.00000 +0.00000
O +0.00000 +0.00000 +0.96379
C +1.27803 +0.00000 +1.38071
C +1.35155 −0.05587 +2.89767
N +0.75500 −1.32740 +3.29802
H +0.76291 −1.41744 +4.30635
O +2.20463 +0.02174 +0.62560
C +2.80050 +0.03079 +3.37451
H +0.79679 +0.82277 +3.27135
H −0.20660 −1.38879 +2.98558
C +3.36857 +1.42346 +3.25801
H +2.82269 −0.28388 +4.42515
H +3.39926 −0.68539 +2.80444
C +3.02355 +2.39424 +4.19672
C +3.51945 +3.68647 +4.10145
C +4.37433 +4.02491 +3.06038
C +4.72501 +3.06536 +2.12049
C +4.22336 +1.77395 +2.21722
H +2.36284 +2.12807 +5.02003
H +3.24328 +4.42912 +4.84370
H +4.76752 +5.03374 +2.98372
H +5.39223 +3.32370 +1.30388
H +4.48145 +1.02653 +1.47268
23
* E = +4.291 kcal/mol ; (20) 183_322_291_165
H +0.00000 +0.00000 +0.00000
O +0.00000 +0.00000 +0.96377
C +1.28011 +0.00000 +1.37743
C +1.39420 −0.07535 +2.89302
N +0.39348 +0.67197 +3.62688
H +0.52172 +1.67032 +3.52482
O +2.20368 +0.01053 +0.61500
C +1.33312 −1.56659 +3.28696
H +2.38847 +0.30793 +3.14344
H −0.53593 +0.44409 +3.29231
C +1.79093 −1.79094 +4.70289

H	+0.29916	−1.90939	+3.15389
H	+1.96175	−2.14063	+2.59732
C	+0.90687	−1.68132	+5.77235
C	+1.34941	−1.85891	+7.07561
C	+2.68499	−2.14573	+7.32647
C	+3.57525	−2.25481	+6.26681
C	+3.12812	−2.07837	+4.96453
H	−0.13536	−1.44572	+5.57691
H	+0.64833	−1.77175	+7.89999
H	+3.03047	−2.28646	+8.34582
H	+4.62019	−2.48239	+6.45375
H	+3.82558	−2.16927	+4.13453

23
∗ E = +4.307 kcal/mol ; (21) 180_359_296_161

H	+0.00000	+0.00000	+0.00000
O	+0.00000	+0.00000	+0.96425
C	+1.28044	+0.00000	+1.36749
C	+1.44108	+0.00105	+2.88710
N	+0.21951	+0.02401	+3.66047
H	−0.29811	+0.88090	+3.51365
O	+2.19735	−0.00019	+0.59572
C	+2.31235	−1.20517	+3.28760
H	+2.01504	+0.90952	+3.11246
H	−0.38873	−0.73992	+3.38841
C	+2.89782	−1.04551	+4.66535
H	+1.68931	−2.10794	+3.23926
H	+3.11131	−1.31445	+2.54868
C	+2.17522	−1.39362	+5.80335
C	+2.71725	−1.21161	+7.06780
C	+3.99031	−0.67513	+7.21126
C	+4.71855	−0.32316	+6.08297
C	+4.17346	−0.50880	+4.81971
H	+1.17462	−1.80142	+5.69177
H	+2.14255	−1.48897	+7.94621
H	+4.41493	−0.53476	+8.20033
H	+5.71621	+0.09253	+6.18590
H	+4.74724	−0.23876	+3.93580

23
∗ E = +4.591 kcal/mol ; (22) 183_201_203_080

H	+0.00000	+0.00000	+0.00000

O	+0.00000	+0.00000	+0.96415
C	+1.27264	+0.00000	+1.37364
C	+1.41205	−0.06758	+2.88380
N	+2.72707	+0.44040	+3.22195
H	+3.03998	+0.09014	+4.11682
O	+2.19901	+0.02161	+0.61285
C	+0.32020	+0.72204	+3.63159
H	+1.27699	−1.13903	+3.12320
H	+3.40037	+0.18377	+2.50973
C	−1.00359	+0.01035	+3.75339
H	+0.71791	+0.91485	+4.63483
H	+0.19397	+1.69986	+3.15577
C	−1.10385	−1.14774	+4.52151
C	−2.31397	−1.81363	+4.65353
C	−3.44832	−1.32386	+4.01943
C	−3.36144	−0.16764	+3.25581
C	−2.14656	+0.49071	+3.12338
H	−0.22127	−1.52823	+5.03283
H	−2.37342	−2.71355	+5.25793
H	−4.39731	−1.84030	+4.12306
H	−4.24421	+0.22256	+2.75850
H	−2.07649	+1.38870	+2.51491

23

* E = +4.732 kcal/mol ; (23) 179_300_065_056

H	+0.00000	+0.00000	+0.00000
O	+0.00000	+0.00000	+0.96368
C	+1.28317	+0.00000	+1.37697
C	+1.36285	+0.02617	+2.89239
N	+0.69595	+1.23241	+3.35845
H	−0.29336	+1.19635	+3.13902
O	+2.20672	−0.03178	+0.62111
C	+0.78359	−1.28691	+3.46320
H	+2.42857	+0.09122	+3.13228
H	+0.79115	+1.33025	+4.36151
C	+1.46102	−2.50702	+2.89882
H	+0.89659	−1.25208	+4.55308
H	−0.28976	−1.31082	+3.24143
C	+2.69737	−2.92053	+3.38930
C	+3.35122	−4.01033	+2.83402
C	+2.77518	−4.70203	+1.77592

C	+1.54434	−4.29766	+1.27861
C	+0.89345	−3.20550	+1.83675
H	+3.14890	−2.38221	+4.22002
H	+4.31319	−4.32262	+3.22846
H	+3.28540	−5.55561	+1.34119
H	+1.08829	−4.83507	+0.45292
H	−0.07167	−2.88678	+1.44875

23

* E = +4.867 kcal/mol ; (24) 358_351_299_290

H	+0.00000	+0.00000	+0.00000
O	+0.00000	+0.00000	+0.96307
C	+1.26228	+0.00000	+1.39999
C	+2.36338	−0.04711	+0.32508
N	+1.94213	−0.28790	−1.03384
H	+1.47908	−1.16590	−1.20940
O	+1.52022	+0.04886	+2.56626
C	+3.15776	+1.27188	+0.40336
H	+3.02919	−0.85909	+0.63819
H	+1.49669	+0.49487	−1.49311
C	+2.32982	+2.44085	−0.06782
H	+3.47005	+1.41396	+1.44167
H	+4.04982	+1.16455	−0.22009
C	+1.41067	+3.04849	+0.78816
C	+0.60647	+4.08586	+0.33782
C	+0.70932	+4.53180	−0.97404
C	+1.62303	+3.93699	−1.83320
C	+2.42696	+2.89783	−1.38111
H	+1.33213	+2.70442	+1.81747
H	−0.10165	+4.55122	+1.01637
H	+0.08266	+5.34618	−1.32346
H	+1.71500	+4.28562	−2.85729
H	+3.14911	+2.43512	−2.05053

23

* E = +4.918 kcal/mol ; (25) 007_165_300_064

H	+0.00000	+0.00000	+0.00000
O	+0.00000	+0.00000	+0.96373
C	+1.24719	+0.00000	+1.45481
C	+2.38548	+0.15994	+0.43566
N	+3.64559	+0.49743	+1.05841
H	+3.56378	+1.37267	+1.56300

O	+1.44316	−0.11460	+2.62764
C	+2.55850	−1.13302	−0.38792
H	+2.10651	+0.96873	−0.25489
H	+3.86405	−0.20101	+1.76263
C	+1.36738	−1.46632	−1.24837
H	+3.44938	−0.98330	−1.00632
H	+2.77436	−1.96024	+0.29871
C	+1.03480	−0.64254	−2.32563
C	−0.08726	−0.90385	−3.10036
C	−0.89386	−1.99692	−2.80879
C	−0.57088	−2.82421	−1.74201
C	+0.55033	−2.55794	−0.96669
H	+1.67267	+0.20633	−2.56443
H	−0.32940	−0.25585	−3.93694
H	−1.77151	−2.20282	−3.41286
H	−1.19778	−3.67856	−1.50717
H	+0.79321	−3.20036	−0.12416

23

* E = +5.535 kcal/mol ; (26) 006_336_160_101

H	+0.00000	+0.00000	+0.00000
O	+0.00000	+0.00000	+0.97140
C	+1.27069	+0.00000	+1.36672
C	+2.28488	+0.13514	+0.22416
N	+1.66759	−0.34282	−1.01267
H	+2.15203	+0.01154	−1.82698
O	+1.58617	−0.05304	+2.51924
C	+2.67216	+1.62817	+0.07455
H	+3.17416	−0.43529	+0.51904
H	+1.67750	−1.35479	−1.06549
C	+4.00437	+1.99758	+0.68437
H	+2.69787	+1.87042	−0.99395
H	+1.88104	+2.25634	+0.50340
C	+4.90093	+2.77971	−0.03796
C	+6.12515	+3.14613	+0.50581
C	+6.46778	+2.72931	+1.78394
C	+5.57949	+1.94716	+2.51113
C	+4.35504	+1.58293	+1.96850
H	+4.63728	+3.10593	−1.04144
H	+6.81271	+3.75505	−0.07307
H	+7.42429	+3.01159	+2.21231

H +5.84012 +1.61816 +3.51244
H +3.66150 +0.97530 +2.54459
23
∗ E = +5.792 kcal/mol ; (27) 005_160_026_066
H +0.00000 +0.00000 +0.00000
O +0.00000 +0.00000 +0.96358
C +1.24453 +0.00000 +1.45221
C +2.37181 +0.11118 +0.42593
N +3.55943 +0.57400 +1.10279
H +3.52199 +0.31029 +2.08141
O +1.44426 −0.10169 +2.62576
C +2.55426 −1.25167 −0.29180
H +2.05726 +0.84666 −0.33181
H +4.40269 +0.20259 +0.68772
C +1.36899 −1.64353 −1.13393
H +3.44449 −1.15806 −0.92593
H +2.76694 −2.01796 +0.46184
C +1.05694 −0.91288 −2.28190
C −0.06841 −1.22007 −3.03523
C −0.89753 −2.26618 −2.65052
C −0.59483 −3.00071 −1.51192
C +0.52852 −2.68821 −0.75795
H +1.71185 −0.10122 −2.59300
H −0.29522 −0.64477 −3.92742
H −1.77719 −2.50825 −3.23809
H −1.23956 −3.81807 −1.20477
H +0.75470 −3.25547 +0.14131
23
∗ E = +6.019 kcal/mol ; (28) 358_174_304_295
H +0.00000 +0.00000 +0.00000
O +0.00000 +0.00000 +0.96014
C +1.25999 +0.00000 +1.44465
C +2.37224 −0.05150 +0.38973
N +3.69222 +0.09350 +0.95321
H +3.75346 +0.95214 +1.48777
O +1.45720 +0.03514 +2.61854
C +2.28961 −1.36835 −0.41388
H +2.19410 +0.78069 −0.30740
H +3.85822 −0.66015 +1.61425
C +2.55010 −2.57465 +0.45196

H +1.30783 −1.46218 −0.89395
H +3.03808 −1.30636 −1.20870
C +1.52975 −3.12891 +1.22243
C +1.78083 −4.21158 +2.05278
C +3.05762 −4.75328 +2.12392
C +4.08136 −4.20687 +1.36200
C +3.82797 −3.12307 +0.53174
H +0.52780 −2.70675 +1.17216
H +0.97658 −4.63307 +2.64741
H +3.25387 −5.60124 +2.77233
H +5.08145 −4.62615 +1.41261
H +4.62806 −2.68960 −0.06360
23
* E = +7.436 kcal/mol ; (29) 353_156_037_180
H +0.00000 +0.00000 +0.00000
O +0.00000 +0.00000 +0.95987
C +1.25492 +0.00000 +1.44079
C +2.35858 −0.16483 +0.40524
N +3.57348 +0.39717 +0.93802
H +3.63260 +0.21117 +1.93331
O +1.46112 +0.05836 +2.61359
C +2.43246 −1.67655 +0.05533
H +2.07625 +0.38116 −0.50944
H +4.39243 +0.02908 +0.47043
C +3.49380 −1.94273 −0.97661
H +2.64859 −2.22701 +0.97816
H +1.45604 −2.01798 −0.31205
C +4.75107 −2.41054 −0.60443
C +5.74651 −2.60398 −1.55493
C +5.49410 −2.33117 −2.89139
C +4.24196 −1.86486 −3.27424
C +3.25234 −1.67209 −2.32250
H +4.94646 −2.63381 +0.44193
H +6.72083 −2.97225 −1.24913
H +6.26928 −2.48410 −3.63529
H +4.03624 −1.65426 −4.31913
H +2.27160 −1.31192 −2.62716
23
* E = +7.732 kcal/mol ; (30) 357_165_284_164
H +0.00000 +0.00000 +0.00000

O	+0.00000	+0.00000	+0.96030
C	+1.25634	+0.00000	+1.44303
C	+2.37070	−0.07851	+0.39589
N	+3.66537	+0.27104	+0.93391
H	+3.74669	+1.27229	+1.05980
O	+1.45770	+0.02441	+2.61825
C	+2.40424	−1.51398	−0.17982
H	+2.12845	+0.61319	−0.42351
H	+3.75611	−0.14079	+1.85847
C	+3.17683	−1.59019	−1.46977
H	+2.85381	−2.16030	+0.58356
H	+1.38312	−1.87920	−0.34356
C	+4.56988	−1.60868	−1.47413
C	+5.26904	−1.64243	−2.67218
C	+4.58677	−1.65746	−3.88233
C	+3.19921	−1.64125	−3.88851
C	+2.50236	−1.60879	−2.68785
H	+5.10165	−1.58222	−0.52817
H	+6.35455	−1.65773	−2.66131
H	+5.13596	−1.68648	−4.81808
H	+2.65709	−1.66044	−4.82887
H	+1.41410	−1.61058	−2.69674

23

∗ E = +7.761 kcal/mol ; (31) 354_166_031_297

H	+0.00000	+0.00000	+0.00000
O	+0.00000	+0.00000	+0.96022
C	+1.25713	+0.00000	+1.44394
C	+2.35476	−0.14366	+0.39675
N	+3.61490	+0.21110	+1.00113
H	+3.59939	−0.02586	+1.98779
O	+1.45768	+0.05997	+2.61624
C	+2.30141	−1.57742	−0.20156
H	+2.12320	+0.55971	−0.41962
H	+4.38913	−0.27063	+0.56362
C	+2.57570	−2.64604	+0.82215
H	+1.32412	−1.74646	−0.67235
H	+3.04851	−1.62298	−1.00219
C	+1.56490	−3.08420	+1.67535
C	+1.82625	−4.03546	+2.65019
C	+3.10436	−4.56215	+2.78530

C +4.11812 −4.13477 +1.93937
C +3.85230 −3.18240 +0.96455
H +0.56212 −2.67270 +1.57631
H +1.02897 −4.36570 +3.30841
H +3.30855 −5.30724 +3.54757
H +5.11828 −4.54555 +2.03590
H +4.64536 −2.85877 +0.29357
23
* E = +7.903 kcal/mol ; (32) 020_204_205_318
H +0.00000 +0.00000 +0.00000
O +0.00000 +0.00000 +0.96201
C +1.24153 +0.00000 +1.46003
C +2.36923 +0.47015 +0.53615
N +3.60773 −0.11553 +1.01779
H +4.40728 +0.43625 +0.73674
O +1.44113 −0.26817 +2.60693
C +2.15195 +0.17849 −0.95901
H +2.34807 +1.56924 +0.65602
H +3.58491 −0.16803 +2.03056
C +1.59314 −1.18967 −1.28382
H +1.49132 +0.94236 −1.38894
H +3.12179 +0.31533 −1.45254
C +0.57409 −1.30831 −2.22709
C +0.02217 −2.54706 −2.52965
C +0.48337 −3.68465 −1.88460
C +1.49648 −3.57548 −0.93976
C +2.05081 −2.33962 −0.63934
H +0.21177 −0.41734 −2.73687
H −0.77116 −2.61980 −3.26703
H +0.05245 −4.65404 −2.11360
H +1.85848 −4.46256 −0.42919
H +2.84418 −2.25210 +0.09702
23
* E = +9.533 kcal/mol ; (33) 008_320_314_059
H +0.00000 +0.00000 +0.00000
O +0.00000 +0.00000 +0.96186
C +1.26885 +0.00000 +1.41625
C +2.34606 +0.19523 +0.34479
N +2.11605 −0.50592 −0.90174
H +1.83080 −1.46988 −0.81025

O	+1.50562	−0.10379	+2.57898
C	+2.47653	+1.69834	+0.03529
H	+3.27843	−0.15367	+0.80185
H	+1.53989	−0.01080	−1.56803
C	+2.84171	+2.53334	+1.23270
H	+3.23425	+1.79613	−0.74936
H	+1.52300	+2.04833	−0.38650
C	+4.17715	+2.76544	+1.54769
C	+4.51973	+3.50698	+2.66920
C	+3.52602	+4.02477	+3.48937
C	+2.19114	+3.79709	+3.18346
C	+1.85286	+3.05558	+2.06090
H	+4.95546	+2.36249	+0.90342
H	+5.56514	+3.68271	+2.90293
H	+3.79200	+4.60546	+4.36689
H	+1.41012	+4.19534	+3.82330
H	+0.80604	+2.87052	+1.82673

23

∗ E = +10.423 kcal/mol ; (34) 010_081_184_072

H	+0.00000	+0.00000	+0.00000
O	+0.00000	+0.00000	+0.95978
C	+1.25963	+0.00000	+1.44226
C	+2.34564	+0.23017	+0.38809
N	+2.40019	+1.66966	+0.15104
H	+3.06225	+1.88944	−0.58245
O	+1.46269	−0.12087	+2.60817
C	+3.69732	−0.26821	+0.89599
H	+2.07517	−0.35539	−0.51186
H	+1.50195	+2.05892	−0.10785
C	+3.76309	−1.77352	+0.94731
H	+4.47281	+0.11514	+0.22175
H	+3.86611	+0.17036	+1.88353
C	+4.04552	−2.49986	−0.20834
C	+4.07750	−3.88691	−0.18768
C	+3.82638	−4.56742	+0.99676
C	+3.54471	−3.85341	+2.15351
C	+3.51110	−2.46515	+2.12918
H	+4.25533	−1.96758	−1.13449
H	+4.30472	−4.43776	−1.09526
H	+3.85384	−5.65231	+1.01819

H	+3.34844	−4.37977	+3.08251
H	+3.27732	−1.90400	+3.02954

23

∗ E = +10.553 kcal/mol ; (35) 352_268_297_163

H	+0.00000	+0.00000	+0.00000
O	+0.00000	+0.00000	+0.96027
C	+1.25608	+0.00000	+1.44982
C	+2.38999	−0.18507	+0.43716
N	+2.73078	−1.59720	+0.30787
H	+3.07575	−1.96409	+1.18683
O	+1.44633	+0.06645	+2.62555
C	+2.08955	+0.40683	−0.94724
H	+3.25623	+0.32929	+0.86508
H	+1.91866	−2.14567	+0.04453
C	+3.33725	+0.57159	−1.77662
H	+1.38762	−0.25615	−1.47774
H	+1.60190	+1.38260	−0.83052
C	+3.88183	−0.50069	−2.47801
C	+5.05148	−0.34309	−3.20761
C	+5.69270	+0.88840	−3.24316
C	+5.15696	+1.96278	−2.54670
C	+3.98558	+1.80278	−1.81917
H	+3.38874	−1.46746	−2.43866
H	+5.46531	−1.18708	−3.75066
H	+6.60674	+1.01093	−3.81547
H	+5.64950	+2.92978	−2.57228
H	+3.56520	+2.64771	−1.27826

12 Trp

12.1 Reference Z-matrix for Trp

Bond distances are given in Å; bond and dihedral angles, in degrees.

h						
o	1	oh2				
c	2	co3	1	coh3		
c	3	cc4	2	cco4	1	dih4
n	4	nc5	3	ncc5	2	dih5
h	5	hn6	4	hnc6	3	dih6
o	3	oc7	2	oco7	4	dih7
c	4	cc8	3	ccc8	5	dih8
h	4	hc9	3	hcc9	5	dih9
h	5	hn10	4	hnc10	6	dih10
c	8	cc11	4	ccc11	3	dih11
h	8	hc12	4	hcc12	11	dih12
h	8	hc13	4	hcc13	11	dih13
c	11	cc14	8	ccc14	4	dih14
c	14	cc15	11	ccc15	8	dih15
n	15	nc16	14	ncc16	11	dih16
c	16	cn17	15	cnc17	14	dih17
c	14	cc18	15	ccc18	16	dih18
c	15	cc19	14	ccc19	11	dih19
h	16	hn20	15	hnc20	14	dih20
h	17	hc21	16	hcn21	15	dih21
c	18	cc22	14	ccc22	15	dih22
h	18	hc23	22	hcc23	14	dih23
h	22	hc24	18	hcc24	23	dih24
c	19	cc25	15	ccc25	14	dih25
h	19	hc26	25	hcc26	15	dih26
h	25	hc27	19	hcc27	26	dih27

oh2	0.96855279
co3	1.35891599
coh3	111.81386759
cc4	1.51940547
cco4	111.35424175
dih4	177.75260599
nc5	1.46548493

ncc5	112.51318659
dih5	35.45493749
hn6	1.00576235
hnc6	112.57719775
dih6	−54.67
oc7	1.20267844
oco7	121.9477744
dih7	181.66101407
cc8	1.53339299
ccc8	109.57058295
dih8	124.56920758
hc9	1.08418715
hcc9	106.69103153
dih9	241.9015561
hn10	1.00419829
hnc10	112.57013084
dih10	126.367
cc11	1.50370834
ccc11	113.6229495
dih11	168.27976505
hc12	1.0879213
hcc12	108.54078276
dih12	123.44462727
hc13	1.08171937
hcc13	107.7308396
dih13	238.50939645
cc14	1.45117037
ccc14	124.73544479
dih14	209.37244077
cc15	1.40380243
ccc15	107.11664785
dih15	180.20736581
nc16	1.37511404
ncc16	107.37911765
dih16	0.08222008
cn17	1.38808473
cnc17	109.0498764
dih17	0.08542624
cc18	1.39340584
ccc18	119.42201357

dih18	179.96159771
cc19	1.38970038
ccc19	121.58129849
dih19	180.22016224
hn20	0.99383215
hnc20	125.81048234
dih20	−179.85803744
hc21	1.06333266
hcn21	121.81175482
dih21	−180.64304142
cc22	1.37555383
ccc22	119.07796935
dih22	−0.05076836
hc23	1.07242294
hcc23	120.43716003
dih23	180.05815641
hc24	1.07186058
hcc24	119.90575904
dih24	0.0511883
cc25	1.37521091
ccc25	117.96411347
dih25	−0.0853439
hc26	1.07194791
hcc26	120.94367934
dih26	179.97461546
hc27	1.07213225
hcc27	119.49561404
dih27	0.00586626

12.2 Reference energy and target torsions for Trp

- Reference M08-HX/MG3S energy: -686.4077608 hartree
- Target torsions:
  - torsion1: dih4
  - torsion2: dih5
  - torsion3: dih6
  - torsion4: dih11

– torsion5: dih14

12.3 Properties of Trp conformers

Table S12: Relative energy (kcal mol^{-1}), rotational constants (B1 to B3, GHz) and dipolar moment (Debye) for the conformers of Trp located at the M08-HX/MG3S level.

Conformer	Rel. Energy	B1	B2	B3	Dip. Mom.
1	0.000	0.353	0.401	1.241	3.315
2	1.773	0.361	0.417	1.191	3.117
3	1.831	0.382	0.493	0.974	6.811
4	1.841	0.292	0.340	1.299	7.032
5	2.132	0.393	0.495	1.011	1.164
6	2.172	0.353	0.406	1.204	2.400
7	2.252	0.286	0.337	1.369	6.335
8	2.364	0.401	0.522	0.995	3.405
9	2.421	0.297	0.336	1.267	6.906
10	2.716	0.286	0.328	1.371	6.116
11	3.172	0.318	0.341	1.420	1.917
12	3.314	0.293	0.337	1.277	2.267
13	3.329	0.378	0.469	1.090	3.275
14	3.429	0.292	0.342	1.280	3.269
15	3.465	0.283	0.332	1.385	4.012
16	3.774	0.294	0.331	1.277	2.998
17	3.783	0.365	0.407	1.159	2.436
18	3.879	0.285	0.326	1.384	3.408
19	3.883	0.285	0.335	1.373	4.126
20	3.885	0.258	0.300	1.473	1.230
21	3.926	0.335	0.379	1.154	2.189
22	3.961	0.403	0.497	1.042	2.693
23	3.986	0.403	0.521	1.005	4.318
24	4.151	0.377	0.469	1.094	3.949
25	4.202	0.365	0.419	1.212	3.297
26	4.311	0.370	0.424	1.187	2.318
27	4.363	0.307	0.344	1.354	1.332
28	4.413	0.295	0.349	1.300	2.559

29	4.669	0.290	0.341	1.293	4.543
30	4.746	0.353	0.396	1.149	3.214
31	4.761	0.286	0.326	1.422	3.572
32	4.805	0.361	0.403	1.168	2.774
33	4.806	0.384	0.442	1.098	0.938
34	4.905	0.319	0.368	1.192	1.450
35	4.932	0.287	0.337	1.370	3.047
36	4.973	0.276	0.329	1.396	3.318
37	5.032	0.306	0.343	1.333	3.339
38	5.144	0.368	0.406	1.162	3.930
39	5.157	0.253	0.295	1.528	3.310
40	5.183	0.369	0.422	1.214	2.884
41	5.392	0.313	0.337	1.458	3.309
42	5.488	0.299	0.336	1.374	0.902
43	5.556	0.257	0.289	1.602	4.104
44	5.597	0.387	0.459	1.078	3.604
45	5.747	0.290	0.347	1.246	3.379
46	5.754	0.365	0.456	1.101	5.570
47	5.801	0.338	0.358	1.327	3.400
48	5.827	0.301	0.337	1.351	2.696
49	5.935	0.318	0.372	1.186	3.178
50	6.087	0.385	0.452	1.094	2.716
51	6.148	0.363	0.402	1.174	5.174
52	6.165	0.353	0.396	1.167	3.532
53	6.408	0.307	0.344	1.366	1.206
54	6.439	0.279	0.328	1.397	0.431
55	6.709	0.337	0.395	1.152	4.097
56	6.756	0.362	0.445	1.115	5.312
57	6.782	0.362	0.416	1.218	2.932
58	6.800	0.343	0.408	1.163	5.540
59	6.934	0.372	0.420	1.120	2.712
60	7.018	0.310	0.335	1.455	2.626
61	7.465	0.363	0.388	1.261	3.656
62	7.556	0.358	0.451	1.089	6.048
63	7.559	0.362	0.447	1.119	6.196
64	7.622	0.387	0.505	0.982	3.744
65	7.769	0.317	0.341	1.431	2.150

66	8.360	0.291	0.341	1.285	5.594
67	8.520	0.260	0.304	1.433	4.072
68	8.899	0.283	0.333	1.379	6.335
69	8.927	0.286	0.336	1.367	6.507
70	9.560	0.388	0.508	0.987	4.934
71	9.575	0.403	0.473	0.982	5.198
72	10.534	0.293	0.332	1.275	5.746
73	10.580	0.263	0.288	1.554	5.822
74	10.871	0.329	0.374	1.169	2.948
75	10.901	0.347	0.392	1.161	3.559
76	12.548	0.281	0.319	1.397	4.077

12.4 Vibrational frequencies of Trp conformers

Harmonic vibrational zero point energy (ZPE) and frequencies for each conformer in cm^{-1}.

- Conformer (1); ZPE = 48628.490 cm^{-1}:

39.09	51.45	80.38	98.82	153.47	196.95
226.57	304.77	316.32	342.57	380.93	434.65
460.20	474.49	493.43	543.38	554.22	576.43
587.05	602.15	667.42	715.68	765.70	777.42
778.32	798.54	813.84	850.96	874.03	884.66
894.22	896.57	932.10	965.30	1003.23	1019.66
1040.75	1098.39	1108.54	1116.57	1153.22	1164.04
1178.59	1235.10	1244.58	1261.13	1276.25	1299.20
1331.56	1361.50	1372.80	1392.74	1399.36	1437.07
1466.23	1470.87	1494.94	1541.65	1608.87	1642.37
1652.26	1691.98	1879.01	3042.92	3054.25	3111.98
3169.53	3186.60	3196.96	3209.07	3258.71	3527.44
3639.50	3667.75	3717.73			

- Conformer (2); ZPE = 48492.814 cm^{-1}:

37.21	53.31	76.93	94.57	150.71	197.65
224.69	273.37	295.03	311.15	367.40	434.82
448.97	463.33	471.80	490.93	548.75	573.27
590.45	601.86	644.85	663.89	719.15	762.43
769.82	777.00	792.16	844.74	869.48	878.12
895.36	916.93	943.58	971.68	1007.59	1012.80
1041.49	1088.61	1112.40	1144.08	1153.43	1161.35
1165.90	1189.66	1256.10	1261.28	1278.70	1307.96
1331.62	1357.74	1375.16	1378.01	1394.04	1411.97
1466.82	1470.84	1495.55	1542.85	1614.59	1644.96
1670.98	1692.14	1867.35	3030.80	3056.58	3109.99
3181.11	3187.65	3198.21	3208.63	3257.33	3529.68
3610.75	3720.31	3843.20			

- Conformer (3); ZPE = 48588.369 cm^{-1}:

18.80	41.49	89.46	111.65	154.28	192.23
224.80	286.35	306.21	340.56	377.98	436.23
458.10	467.35	481.48	539.63	546.97	586.62
588.87	601.03	665.36	715.71	767.88	773.34
778.51	797.65	826.17	833.28	863.66	881.39
881.65	893.35	930.77	976.06	1013.27	1037.08
1042.65	1071.14	1113.66	1123.30	1156.02	1166.50
1181.18	1224.16	1257.41	1259.74	1279.10	1297.96
1329.04	1357.70	1373.69	1383.50	1401.19	1437.88
1460.38	1469.17	1495.05	1542.51	1612.35	1643.69
1648.88	1690.70	1878.49	3047.64	3059.35	3101.85
3184.34	3194.18	3202.18	3211.29	3248.40	3525.79
3641.04	3660.34	3718.11			

- Conformer (4); ZPE = 48627.674 cm^{-1}:

39.15	48.71	72.08	84.19	174.95	183.75
227.05	242.47	309.58	346.26	356.30	420.21
438.79	462.91	486.17	542.52	551.69	588.61
599.59	643.06	676.51	708.41	772.51	778.18
790.26	799.28	806.25	839.96	880.34	882.18
896.32	902.79	963.04	974.99	996.10	1011.92
1042.30	1090.17	1114.15	1133.22	1152.87	1163.35
1180.35	1242.74	1256.68	1273.67	1287.36	1302.80
1336.60	1365.78	1366.98	1382.76	1394.83	1427.81
1464.20	1468.77	1497.32	1543.91	1620.50	1648.96
1657.49	1694.48	1890.77	3033.84	3046.33	3091.67
3174.77	3185.71	3198.82	3211.23	3252.31	3535.33
3619.69	3689.28	3718.45			

- Conformer (5); ZPE = 48456.647 cm^{-1}:

28.66	43.73	81.19	105.18	151.29	191.03
219.56	249.68	273.55	307.04	370.04	429.42
442.83	463.23	468.24	503.81	545.46	570.33
589.97	599.09	635.31	667.52	719.38	765.24
772.56	777.73	793.72	842.32	874.29	883.42
892.25	917.69	934.30	968.76	1005.15	1023.55
1041.67	1082.33	1111.70	1141.05	1153.73	1161.16
1170.74	1193.89	1246.09	1261.04	1274.64	1307.63
1331.40	1352.13	1369.94	1378.85	1397.76	1420.90
1465.48	1470.78	1495.77	1542.58	1613.61	1644.31
1661.52	1692.27	1867.32	3050.87	3060.18	3109.39
3181.14	3188.24	3197.65	3209.20	3254.79	3532.69
3612.22	3720.26	3839.82			

- Conformer (6); ZPE = 48498.439 cm^{-1}:

28.98	46.42	84.41	90.56	154.42	197.36
226.04	281.66	316.57	322.90	367.58	435.59
440.46	463.17	477.08	524.68	542.52	563.43
579.71	598.67	633.34	671.88	723.35	760.65
768.63	777.19	792.14	848.17	875.56	877.26
890.43	896.67	940.97	969.13	1004.65	1007.13
1041.36	1087.78	1112.97	1137.82	1152.00	1163.27
1180.54	1196.77	1253.50	1260.59	1282.37	1320.46
1331.55	1361.41	1375.28	1384.30	1389.59	1401.00
1464.29	1467.88	1495.10	1542.05	1612.00	1643.94
1662.80	1691.54	1850.79	3016.88	3061.05	3116.81
3180.17	3186.94	3197.34	3207.88	3258.52	3536.74
3626.85	3718.91	3846.51			

- Conformer (7); ZPE = 48626.712 cm^{-1}:

44.78	48.40	73.23	89.59	167.64	201.17
222.85	231.87	327.49	347.68	352.06	417.37
437.46	463.02	500.45	537.65	563.29	582.82
598.63	642.96	676.77	709.20	767.91	776.30
788.22	800.20	804.98	848.79	875.17	882.15
886.31	896.51	956.62	969.15	994.54	1007.59
1042.03	1088.01	1116.22	1128.73	1154.48	1162.40
1175.60	1239.44	1262.74	1273.82	1287.57	1302.42
1339.04	1362.81	1368.92	1377.41	1396.38	1427.89
1467.17	1472.77	1496.69	1541.95	1615.39	1645.06
1664.93	1692.82	1891.25	3025.39	3039.28	3103.23
3175.24	3187.75	3199.76	3210.66	3254.79	3543.04
3622.86	3689.07	3717.58			

- Conformer (8); ZPE = 48484.825 cm^{-1}:

38.90	57.28	79.79	101.55	148.10	191.94
224.89	260.94	288.73	306.56	358.39	428.40
441.36	462.08	469.23	533.69	545.19	575.51
590.23	597.29	631.65	672.04	721.26	769.39
775.49	784.48	795.40	831.56	872.83	882.80
890.92	894.87	924.99	972.35	1006.39	1010.69
1042.55	1079.99	1112.14	1136.97	1155.61	1162.22
1177.94	1212.40	1236.05	1261.74	1278.84	1316.58
1331.20	1359.35	1371.74	1382.73	1392.93	1402.29
1463.88	1467.35	1496.54	1540.96	1615.06	1644.37
1657.17	1691.29	1849.95	3051.79	3070.40	3107.99
3180.97	3187.26	3197.90	3208.23	3254.73	3542.03
3626.50	3720.94	3843.92			

- Conformer (9); ZPE = 48511.101 cm^{-1}:

29.78	45.53	64.85	67.36	172.59	201.07
224.10	234.29	252.32	347.46	362.92	423.54
436.20	461.05	463.55	525.45	549.47	588.58
593.33	609.52	663.66	719.06	767.58	775.96
778.83	793.56	806.73	836.06	874.85	883.67
893.04	898.05	932.25	969.28	1006.83	1041.36
1056.00	1087.64	1112.42	1131.73	1157.21	1158.03
1170.33	1230.48	1249.47	1267.79	1275.70	1297.57
1330.21	1358.33	1377.73	1389.05	1397.76	1431.69
1462.19	1470.84	1495.84	1540.63	1622.27	1646.97
1660.97	1692.42	1888.25	3031.03	3046.31	3092.45
3176.17	3186.15	3199.13	3210.56	3252.64	3535.20
3624.88	3694.55	3719.90			

- Conformer (10); ZPE = 48524.691 cm^{-1}:

29.01	56.25	62.66	74.87	173.16	198.08
221.75	235.79	294.79	349.25	366.14	416.73
437.73	460.79	462.20	526.06	552.46	587.79
592.49	604.42	668.04	720.92	765.38	775.88
777.06	793.84	805.35	847.18	871.85	880.17
894.38	903.21	933.07	967.20	1003.88	1036.03
1047.34	1096.64	1114.73	1132.68	1156.00	1159.03
1168.42	1224.75	1256.13	1260.80	1273.06	1294.65
1334.09	1357.18	1372.01	1392.03	1394.96	1429.96
1466.91	1471.39	1495.19	1541.62	1615.92	1643.74
1658.04	1692.45	1888.12	3023.63	3038.46	3105.35
3172.41	3186.39	3198.38	3209.96	3251.47	3538.33
3622.44	3698.33	3720.58			

- Conformer (11); ZPE = 48455.783 cm^{-1}:

26.76	58.68	94.54	120.10	136.25	179.55
209.40	234.51	274.87	306.07	343.05	410.60
434.48	463.03	474.69	500.41	560.81	569.09
594.93	622.79	632.44	645.74	718.34	765.53
767.04	784.75	793.28	853.46	868.58	877.39
895.93	902.56	953.66	968.20	1003.55	1019.34
1039.22	1079.78	1129.65	1150.85	1155.55	1163.37
1175.92	1205.19	1255.81	1272.01	1285.75	1321.38
1329.44	1360.58	1372.17	1381.51	1400.08	1418.91
1463.14	1475.15	1498.07	1542.06	1617.58	1646.89
1668.39	1694.01	1852.07	3014.05	3036.81	3076.20
3177.14	3185.87	3198.27	3210.79	3243.56	3549.47
3630.06	3724.11	3846.29			

- Conformer (12); ZPE = 48388.018 cm^{-1}:

33.51	46.29	64.95	70.52	176.88	195.29
222.68	230.80	239.09	330.99	358.56	430.71
436.31	460.94	481.14	494.93	545.15	582.69
592.03	599.22	631.97	665.98	720.66	768.17
769.86	777.69	792.90	822.83	842.93	877.16
893.16	897.83	933.41	969.21	1005.90	1028.79
1042.11	1094.97	1112.59	1151.47	1157.27	1165.25
1186.81	1208.83	1243.81	1260.72	1278.67	1297.78
1336.87	1350.31	1362.73	1377.21	1392.67	1440.75
1465.25	1466.01	1497.26	1542.09	1620.51	1626.23
1648.49	1693.95	1865.77	2936.73	3048.73	3102.33
3171.09	3184.25	3197.21	3209.68	3259.16	3561.56
3656.15	3719.77	3851.88			

- Conformer (13); ZPE = 48426.984 cm^{-1}:

37.95	64.92	80.98	86.08	148.89	210.74
225.18	241.60	271.09	278.73	321.78	389.20
438.83	458.75	459.59	481.89	552.49	594.98
600.84	601.68	650.63	661.48	754.75	768.35
771.13	789.04	798.55	819.92	854.19	879.71
893.74	904.51	968.70	971.13	1008.31	1031.69
1042.24	1090.40	1113.57	1132.65	1156.42	1164.34
1179.50	1184.15	1234.58	1267.18	1276.49	1304.91
1329.73	1352.27	1369.79	1373.76	1394.71	1438.84
1465.61	1473.02	1497.74	1541.59	1618.82	1646.56
1664.84	1692.31	1850.93	3024.07	3074.91	3099.49
3177.58	3184.19	3195.96	3207.57	3255.43	3533.39
3617.94	3720.29	3834.15			

- Conformer (14); ZPE = 48382.482 cm^{-1}:

36.81	46.31	70.70	71.38	171.38	181.36
225.97	232.28	238.65	312.09	347.29	404.95
436.85	461.80	479.92	520.18	541.47	577.22
593.46	598.55	640.27	668.97	720.25	769.96
778.08	791.14	793.34	833.27	846.08	877.53
892.49	895.58	916.39	971.49	1008.57	1025.67
1042.63	1094.41	1113.75	1143.44	1159.37	1161.60
1168.56	1197.98	1252.38	1266.52	1276.70	1299.96
1331.14	1352.43	1355.91	1375.18	1393.45	1454.65
1465.02	1470.21	1497.87	1543.33	1621.04	1633.17
1649.07	1694.44	1866.60	3026.25	3044.24	3081.51
3173.89	3185.50	3198.60	3210.90	3254.13	3535.82
3628.55	3719.59	3847.48			

- Conformer (15); ZPE = 48424.249 cm^{-1}:

39.39	54.14	73.01	83.71	172.77	199.11
222.77	228.74	280.56	337.16	355.57	426.57
440.51	463.00	466.52	492.90	552.15	574.03
591.81	596.98	628.58	667.30	722.15	769.79
771.49	776.47	792.79	830.56	845.74	879.76
883.55	896.68	939.85	973.56	1009.47	1022.98
1042.81	1095.96	1114.82	1151.13	1155.98	1165.27
1188.85	1210.67	1246.27	1260.93	1281.00	1298.53
1338.65	1350.39	1366.10	1375.15	1394.07	1439.11
1465.26	1467.70	1496.21	1542.74	1616.22	1629.98
1645.14	1693.08	1865.74	2934.49	3040.95	3107.72
3182.29	3190.56	3199.16	3209.07	3250.92	3555.72
3649.20	3720.95	3847.59			

- Conformer (16); ZPE = 48374.484 cm^{-1}:

30.77	49.22	60.10	67.70	175.88	193.60
223.77	234.08	280.81	318.24	355.11	402.40
434.74	452.17	458.94	508.78	550.40	573.08
591.57	598.04	625.81	661.58	740.12	764.83
766.29	777.14	792.35	836.72	854.96	874.03
893.82	895.31	950.26	966.87	1003.61	1021.75
1041.37	1092.68	1110.87	1148.42	1157.73	1164.17
1180.44	1192.64	1253.24	1263.83	1280.39	1301.37
1332.94	1347.33	1363.93	1378.00	1390.76	1448.22
1462.57	1463.56	1496.08	1540.56	1621.07	1639.12
1647.48	1692.71	1870.49	2937.17	3054.00	3104.72
3176.26	3185.95	3198.27	3209.66	3258.25	3548.41
3638.46	3721.53	3849.44			

- Conformer (17); ZPE = 48399.574 cm^{-1}:

29.02	41.94	58.74	72.39	144.05	223.24
226.01	249.24	255.66	284.20	319.19	392.67
435.57	461.78	469.58	487.78	551.38	594.79
601.87	620.66	648.81	658.08	755.32	766.26
771.92	787.44	795.02	819.52	847.58	876.23
892.35	897.29	967.07	978.82	1004.61	1033.94
1041.78	1086.28	1111.41	1132.19	1154.36	1161.94
1178.45	1181.60	1243.44	1260.47	1274.47	1306.68
1326.35	1353.05	1373.46	1378.41	1392.76	1438.85
1464.47	1468.59	1496.13	1541.81	1621.88	1649.08
1660.00	1692.91	1854.67	3035.66	3074.17	3092.08
3172.28	3184.73	3196.98	3209.16	3256.11	3533.67
3620.61	3720.20	3838.01			

- Conformer (18); ZPE = 48445.223 cm^{-1}:

35.74	51.26	71.32	79.79	175.33	198.60
222.31	230.98	318.75	341.39	353.12	405.59
438.40	457.91	463.21	508.00	548.26	573.91
589.69	597.09	624.67	663.14	741.49	766.19
770.07	776.19	793.63	844.29	863.15	880.33
882.17	895.84	955.28	974.18	1009.26	1017.80
1042.32	1095.53	1113.65	1148.38	1157.10	1167.04
1181.16	1191.32	1255.00	1262.30	1282.70	1302.94
1333.75	1348.16	1366.27	1372.98	1394.00	1446.45
1463.11	1466.50	1495.60	1541.48	1616.79	1644.38
1648.77	1691.77	1871.50	2932.04	3049.62	3109.68
3181.18	3187.84	3197.92	3208.11	3252.75	3547.61
3636.22	3720.43	3847.78			

- Conformer (19); ZPE = 48391.056 cm^{-1}:

38.94	48.16	71.86	78.95	163.12	197.39
221.98	227.59	257.43	315.69	346.10	405.25
436.41	462.23	491.08	511.66	554.97	572.39
597.11	597.86	641.69	667.97	721.49	765.52
776.90	789.64	794.78	837.37	845.00	873.37
880.68	895.40	922.13	967.04	1004.62	1016.37
1041.68	1094.88	1115.03	1142.40	1155.46	1158.12
1165.35	1201.41	1257.92	1259.87	1274.64	1298.04
1334.63	1350.64	1354.97	1373.90	1392.74	1452.76
1466.93	1473.77	1495.54	1542.03	1614.89	1632.15
1644.93	1692.63	1868.44	3023.26	3043.58	3084.84
3178.61	3187.88	3199.69	3210.65	3254.55	3543.20
3637.64	3718.91	3847.44			

- Conformer (20); ZPE = 48394.677 cm^{-1}:

25.82	44.21	55.64	100.12	154.68	199.20
213.16	215.48	230.50	306.95	326.62	385.25
433.58	461.81	472.97	498.41	560.35	589.83
598.23	603.63	637.24	663.04	722.21	766.37
776.08	784.83	794.43	864.28	874.47	877.53
894.64	923.43	964.79	989.42	1002.68	1003.95
1041.03	1077.27	1112.42	1150.07	1158.90	1166.00
1172.47	1203.02	1251.81	1265.32	1282.83	1320.60
1332.30	1346.07	1370.83	1380.25	1407.96	1411.55
1464.19	1477.65	1496.98	1541.98	1617.25	1645.91
1669.08	1693.22	1861.74	2991.89	3024.34	3079.92
3178.38	3186.57	3197.37	3208.51	3270.10	3536.89
3614.72	3722.28	3843.84			

- Conformer (21); ZPE = 48372.846 cm^{-1}:

22.91	34.58	66.95	78.17	146.31	209.86
225.24	248.77	265.44	302.79	316.58	380.73
431.92	448.38	461.77	514.14	550.81	594.52
597.38	608.82	632.66	658.49	755.43	765.35
774.43	786.08	791.75	825.08	862.80	872.39
876.66	894.16	963.76	978.52	1002.69	1028.51
1039.18	1086.58	1111.27	1133.60	1147.87	1159.06
1172.88	1181.06	1242.70	1258.30	1275.17	1309.68
1327.71	1355.03	1372.79	1391.37	1398.78	1421.86
1465.95	1469.39	1494.09	1540.54	1620.05	1645.99
1651.07	1691.18	1854.99	3042.05	3062.23	3095.13
3173.82	3185.46	3196.79	3208.00	3259.16	3536.60
3627.19	3722.47	3843.81			

- Conformer (22); ZPE = 48399.442 cm^{-1}:

29.88	53.52	92.06	104.87	152.57	188.09
223.68	239.30	277.70	314.85	361.37	430.29
442.83	464.76	474.28	514.89	545.75	570.25
586.41	597.24	634.75	670.54	719.02	765.60
772.55	779.86	793.78	813.78	843.86	874.61
888.62	898.42	923.52	969.20	1002.26	1004.63
1041.59	1078.63	1115.68	1131.59	1154.03	1162.28
1171.17	1204.22	1250.99	1261.11	1264.98	1283.80
1330.92	1342.11	1366.98	1372.24	1396.97	1452.56
1466.03	1480.43	1497.24	1541.51	1615.89	1637.32
1644.80	1692.45	1873.66	3034.14	3043.59	3089.58
3180.84	3188.07	3198.17	3209.62	3251.36	3545.69
3642.45	3721.92	3842.66			

- Conformer (23); ZPE = 48450.425 cm^{-1}:

38.40	66.39	87.64	101.51	149.47	189.61
226.92	258.61	284.43	317.76	346.89	428.15
441.43	461.86	470.26	533.15	548.92	573.90
583.58	596.37	629.10	674.95	725.93	769.98
774.45	786.12	796.44	835.23	836.63	876.57
888.80	893.08	923.96	972.40	1008.28	1010.33
1042.43	1076.86	1112.62	1117.53	1154.18	1162.01
1177.50	1224.46	1236.34	1258.95	1272.19	1284.31
1331.39	1362.77	1373.45	1377.16	1395.23	1446.53
1463.08	1472.00	1496.90	1542.00	1617.02	1645.46
1652.93	1692.01	1855.84	3033.83	3073.39	3085.19
3181.17	3187.49	3198.03	3208.51	3253.16	3537.66
3627.21	3720.51	3844.00			

- Conformer (24); ZPE = 48395.111 cm^{-1}:

38.90	64.39	80.70	86.35	148.88	211.09
225.22	236.05	277.02	297.04	306.27	387.66
438.75	457.59	460.79	486.79	551.80	595.08
598.79	601.71	647.82	659.42	752.69	768.98
771.82	789.86	798.14	821.28	851.40	856.57
880.28	894.14	970.45	972.96	1008.97	1038.45
1043.55	1092.53	1113.96	1140.24	1152.02	1161.70
1178.60	1183.56	1237.98	1263.78	1277.00	1306.85
1332.33	1343.50	1355.86	1371.91	1393.23	1464.22
1466.83	1477.93	1497.95	1541.81	1617.75	1638.78
1646.82	1692.17	1855.61	3023.19	3058.41	3079.76
3176.57	3184.23	3196.16	3207.79	3254.58	3538.12
3630.06	3720.42	3838.32			

- Conformer (25); ZPE = 48361.407 cm^{-1}:

21.27	56.34	71.33	90.50	147.20	190.39
195.23	224.35	284.20	317.32	354.39	432.74
449.51	463.79	478.07	505.10	553.83	574.30
590.17	598.86	642.81	666.57	720.23	763.02
771.08	777.31	791.90	825.82	849.24	872.49
888.25	896.16	940.27	964.53	986.53	1001.58
1040.33	1090.13	1113.67	1131.05	1154.47	1158.28
1164.79	1199.16	1250.97	1260.76	1265.88	1281.22
1326.14	1340.03	1367.62	1376.64	1394.67	1448.82
1469.87	1476.48	1495.42	1542.44	1615.79	1637.93
1644.86	1693.24	1876.22	3029.49	3040.58	3091.92
3177.48	3188.09	3199.47	3210.47	3252.03	3546.04
3639.80	3722.61	3851.32			

- Conformer (26); ZPE = 48429.413 cm^{-1}:

31.85	52.89	78.17	83.15	152.74	194.72
226.36	242.25	292.54	324.30	355.41	433.76
454.20	464.61	478.30	523.84	555.49	567.39
575.74	596.99	640.98	674.12	723.35	764.15
775.55	777.71	792.84	830.77	856.67	873.67
886.98	895.33	934.26	965.05	996.66	1002.33
1040.09	1091.86	1114.76	1120.28	1151.44	1161.38
1176.21	1219.87	1239.95	1260.41	1265.01	1282.21
1330.93	1360.38	1369.71	1376.65	1395.12	1444.73
1468.64	1470.28	1495.21	1541.88	1613.59	1643.77
1653.81	1692.56	1850.00	3039.71	3049.86	3099.03
3177.36	3187.95	3198.65	3210.03	3253.29	3539.22
3629.05	3722.53	3846.28			

- Conformer (27); ZPE = 48422.870 cm^{-1}:

27.89	50.75	84.79	101.57	138.49	203.71
229.74	258.51	280.42	292.23	323.31	331.74
431.72	444.57	464.22	535.54	569.94	593.14
597.32	609.93	620.96	636.12	755.96	764.78
775.13	789.25	800.82	824.89	855.92	872.73
877.15	895.47	964.89	987.99	1002.09	1016.13
1039.64	1080.23	1125.14	1136.99	1151.81	1160.45
1173.66	1196.36	1250.68	1268.22	1273.48	1322.54
1344.50	1355.48	1369.72	1382.86	1396.60	1413.54
1464.81	1478.34	1497.48	1541.59	1620.30	1645.88
1658.54	1694.18	1859.22	3018.98	3061.23	3079.01
3179.09	3187.57	3198.64	3210.34	3251.57	3542.47
3632.74	3721.42	3850.62			

- Conformer (28); ZPE = 48561.151 cm^{-1}:

18.32	58.38	66.60	99.24	154.30	206.30
227.45	290.25	300.45	352.25	365.43	381.08
432.62	460.16	492.83	549.98	558.39	563.98
596.05	600.80	661.69	730.99	761.35	766.90
775.27	778.61	792.65	835.51	870.59	874.52
884.16	893.89	952.04	960.52	999.05	1039.77
1073.76	1091.52	1115.55	1131.48	1154.08	1164.97
1176.64	1201.71	1233.38	1260.18	1274.79	1317.99
1333.78	1359.19	1374.86	1385.26	1396.39	1429.78
1466.92	1469.68	1495.35	1540.62	1616.57	1644.82
1657.84	1692.73	1886.10	3029.15	3037.82	3085.97
3174.86	3187.44	3198.03	3210.04	3275.56	3541.34
3633.35	3720.24	3730.21			

- Conformer (29); ZPE = 48420.951 cm^{-1}:

32.21	39.46	62.56	71.66	165.03	180.09
224.86	234.64	285.84	317.87	341.71	401.06
437.41	461.68	471.94	538.28	549.69	576.09
586.20	597.12	627.44	662.09	728.50	770.46
778.07	792.49	798.71	833.97	873.18	878.91
890.33	894.05	905.42	972.71	1009.31	1032.79
1042.12	1093.83	1112.11	1116.09	1147.32	1160.53
1171.22	1211.22	1252.24	1268.93	1277.83	1302.47
1334.57	1357.72	1367.88	1376.50	1392.33	1435.56
1464.82	1470.27	1497.30	1543.29	1621.42	1648.79
1651.98	1694.17	1871.44	3023.20	3069.85	3087.20
3173.08	3185.04	3198.07	3210.51	3255.05	3537.09
3625.19	3719.79	3850.01			

- Conformer (30); ZPE = 48365.726 cm^{-1}:

28.10	47.48	59.32	74.79	142.99	217.68
224.49	247.22	267.56	301.24	323.66	386.86
432.94	444.90	461.58	524.52	552.61	593.85
594.75	604.61	612.97	654.87	754.46	765.61
773.15	786.66	792.82	816.84	849.99	872.08
876.35	894.46	964.44	968.81	1003.91	1015.35
1039.18	1080.00	1110.24	1127.79	1146.64	1158.50
1173.03	1187.77	1252.73	1257.48	1274.93	1321.63
1338.06	1350.81	1373.46	1378.65	1392.97	1396.04
1464.44	1472.41	1494.82	1541.99	1621.12	1647.68
1654.79	1692.44	1872.15	3041.36	3076.37	3094.91
3173.32	3185.60	3197.14	3208.59	3254.66	3540.40
3630.05	3722.67	3850.73			

- Conformer (31); ZPE = 48556.296 cm^{-1}:

25.28	49.35	99.80	105.79	146.87	192.03
229.84	290.49	299.38	312.68	329.54	363.08
434.54	467.67	474.62	532.69	570.41	594.93
610.93	631.05	658.24	751.86	759.55	763.19
765.77	787.67	806.45	825.71	872.26	880.62
895.30	908.64	964.75	982.74	1000.78	1022.83
1040.57	1086.00	1117.04	1126.68	1151.92	1163.17
1172.00	1241.77	1249.22	1267.44	1277.85	1314.87
1343.51	1366.27	1370.12	1373.82	1407.29	1419.84
1466.43	1476.21	1499.28	1540.47	1623.29	1647.79
1656.37	1694.15	1889.57	2996.13	3026.53	3042.67
3172.08	3184.55	3196.85	3209.37	3263.52	3551.85
3637.93	3717.24	3723.57			

- Conformer (32); ZPE = 48393.876 cm^{-1}:

29.27	40.19	59.17	74.59	143.52	224.00
229.88	255.12	265.33	292.56	314.04	390.86
435.57	462.08	472.04	489.85	551.47	594.02
599.79	617.65	644.73	656.25	754.58	766.14
773.67	788.41	795.49	819.13	848.15	869.14
876.33	894.42	966.65	976.83	1004.33	1040.97
1051.23	1092.52	1112.43	1134.20	1150.04	1161.45
1176.45	1182.65	1242.19	1259.09	1274.12	1311.02
1331.95	1342.85	1363.51	1374.93	1392.45	1461.37
1464.15	1478.58	1496.46	1541.97	1620.93	1640.70
1649.16	1692.96	1859.58	3020.86	3064.44	3083.46
3171.18	3184.65	3197.16	3209.41	3255.93	3536.37
3628.29	3719.90	3840.98			

- Conformer (33); ZPE = 48428.368 cm^{-1}:

30.68	44.69	79.53	82.55	151.45	216.62
220.06	251.21	272.51	290.70	321.59	388.52
438.22	465.31	474.92	520.95	553.49	580.75
595.92	601.41	613.61	657.14	749.59	766.55
772.95	788.65	799.59	818.68	856.20	874.82
879.70	893.68	958.83	968.96	1004.46	1018.37
1041.34	1085.00	1116.33	1130.00	1151.36	1158.47
1171.21	1193.75	1254.21	1264.69	1278.66	1326.29
1336.25	1348.81	1373.10	1384.58	1389.47	1398.20
1468.61	1476.75	1497.02	1542.70	1616.25	1645.76
1657.15	1693.60	1871.20	3041.89	3079.07	3099.36
3180.51	3187.01	3197.62	3209.16	3251.45	3541.36
3629.82	3719.07	3846.75			

- Conformer (34); ZPE = 48372.193 cm^{-1}:

24.48	50.48	70.27	88.24	150.15	214.67
226.02	261.90	269.87	301.89	337.44	373.26
430.91	453.78	461.56	521.21	550.33	577.99
595.33	602.21	616.13	658.28	751.71	762.88
772.12	775.32	791.93	809.50	865.29	872.01
891.87	893.53	960.92	970.23	1000.10	1037.66
1041.14	1089.83	1112.69	1129.10	1151.01	1161.16
1173.61	1202.97	1247.73	1259.28	1277.43	1307.31
1329.10	1341.46	1368.32	1382.56	1394.94	1451.78
1466.34	1468.08	1494.61	1539.74	1620.20	1645.54
1649.40	1691.16	1872.59	2961.31	3033.04	3100.80
3172.54	3185.88	3196.68	3208.56	3264.67	3551.09
3638.47	3724.06	3846.77			

- Conformer (35); ZPE = 48408.202 cm^{-1}:

35.82	40.39	70.58	73.52	162.24	197.23
219.83	223.23	284.32	321.14	342.62	399.57
435.12	462.44	478.58	536.72	554.58	572.11
592.33	596.96	628.46	662.98	729.87	763.36
776.75	789.87	802.11	843.31	864.63	874.73
883.49	895.41	910.59	965.49	1002.18	1022.65
1041.82	1093.93	1112.91	1115.61	1145.54	1159.31
1170.10	1212.88	1259.38	1261.02	1275.13	1302.10
1337.75	1353.97	1367.43	1372.81	1392.81	1432.76
1466.55	1473.74	1495.22	1541.89	1614.57	1644.65
1648.46	1691.64	1872.04	3022.39	3071.29	3085.57
3178.50	3187.89	3199.26	3210.21	3255.12	3540.01
3627.11	3719.61	3848.22			

- Conformer (36); ZPE = 48321.924 cm^{-1}:

24.06	50.02	70.86	81.81	138.90	162.38
198.25	221.59	227.35	293.62	348.95	400.20
435.89	456.97	460.31	504.42	549.35	573.13
595.58	599.54	641.63	671.81	719.75	762.81
776.12	787.49	791.36	838.74	862.42	873.40
891.93	899.83	949.77	965.52	1001.49	1005.14
1040.54	1089.35	1112.59	1141.83	1155.37	1161.28
1170.48	1187.82	1254.88	1261.33	1270.57	1313.22
1331.04	1354.75	1362.89	1375.48	1393.80	1429.04
1463.48	1469.18	1493.06	1542.77	1615.38	1644.13
1667.15	1691.75	1860.81	3025.04	3066.68	3090.33
3183.33	3193.95	3204.90	3215.80	3254.95	3533.56
3622.95	3721.05	3844.90			

- Conformer (37); ZPE = 48401.133 cm^{-1}:

22.01	40.44	79.59	99.86	139.33	203.42
228.16	255.48	271.23	291.66	318.83	328.86
429.16	440.82	457.25	511.43	567.78	594.01
610.17	626.04	638.07	651.97	758.12	765.67
774.73	790.41	801.01	827.43	843.19	873.44
890.84	894.90	965.81	992.14	1003.42	1033.43
1040.23	1081.94	1121.95	1140.16	1150.25	1160.36
1173.01	1182.46	1243.50	1265.75	1271.70	1305.66
1328.64	1352.90	1368.76	1386.97	1407.82	1431.37
1462.43	1475.02	1496.51	1541.67	1622.01	1646.06
1659.30	1693.95	1864.53	3018.55	3063.95	3076.84
3179.45	3187.18	3197.96	3209.61	3260.14	3533.76
3623.71	3723.25	3832.87			

- Conformer (38); ZPE = 48404.704 cm^{-1}:

28.27	44.01	69.90	89.41	168.66	189.82
224.81	236.50	262.59	295.76	323.83	393.80
438.18	465.52	488.23	497.20	532.00	550.99
595.36	604.94	646.66	668.40	748.93	758.46
769.73	779.78	797.27	818.41	856.46	880.61
894.96	913.88	971.83	984.59	1008.07	1033.23
1042.35	1088.11	1116.15	1135.81	1159.44	1170.33
1179.24	1198.68	1243.47	1262.97	1273.44	1311.35
1328.14	1354.65	1369.13	1378.44	1387.62	1420.77
1463.61	1472.45	1496.70	1540.87	1620.87	1645.68
1670.83	1693.55	1880.75	3031.28	3040.72	3087.49
3173.85	3188.10	3199.46	3211.97	3261.23	3533.41
3612.26	3716.32	3816.90			

- Conformer (39); ZPE = 48365.829 cm^{-1}:

20.84	32.66	56.53	96.62	161.29	201.00
217.11	225.42	278.13	315.24	323.91	371.16
432.98	448.87	455.81	526.00	558.55	578.94
588.66	596.81	627.50	648.42	749.05	764.20
765.34	783.83	795.38	851.63	858.92	875.86
894.23	919.17	966.15	977.90	1002.25	1005.82
1040.13	1076.26	1111.40	1148.63	1153.06	1164.87
1168.88	1183.80	1251.28	1266.70	1289.63	1308.74
1329.39	1346.76	1369.29	1386.36	1393.16	1424.95
1467.57	1474.83	1496.58	1541.56	1617.83	1645.74
1655.48	1692.84	1866.11	3003.15	3021.85	3080.22
3178.26	3185.49	3196.97	3208.71	3273.47	3537.82
3628.96	3723.46	3849.26			

- Conformer (40); ZPE = 48426.327 cm^{-1}:

39.95	52.57	71.27	91.13	153.13	200.32
226.69	292.38	319.49	363.10	396.04	434.95
456.30	465.45	500.23	506.01	528.50	555.63
579.34	599.10	627.90	651.30	665.24	714.40
767.12	777.30	780.34	799.18	852.11	878.01
878.97	895.36	931.07	969.48	989.20	1005.77
1040.29	1080.61	1116.40	1121.14	1145.15	1158.33
1170.92	1202.65	1252.59	1261.79	1277.12	1303.88
1329.91	1356.20	1369.68	1373.92	1376.52	1395.41
1466.28	1470.03	1495.83	1540.73	1611.24	1634.36
1642.24	1691.77	1880.13	3056.96	3065.22	3119.35
3178.70	3188.36	3199.45	3210.23	3251.08	3565.14
3682.63	3718.79	3837.29			

- Conformer (41); ZPE = 48453.020 cm^{-1}:

25.86	50.85	84.90	117.41	141.29	179.55
227.54	267.90	277.16	313.21	338.57	418.77
432.65	453.78	476.02	524.99	555.53	568.72
594.15	600.46	630.53	663.51	721.04	760.07
763.92	783.19	791.28	839.71	855.92	873.94
885.85	895.91	945.63	966.59	1002.61	1012.42
1039.64	1078.48	1128.34	1142.94	1152.32	1163.40
1185.06	1199.30	1255.22	1268.84	1282.89	1328.10
1330.93	1362.57	1371.20	1383.01	1385.41	1404.64
1458.82	1469.82	1497.08	1542.82	1619.11	1647.41
1661.15	1693.79	1862.93	3024.24	3043.44	3083.95
3181.14	3187.85	3199.49	3211.06	3243.46	3560.77
3647.36	3725.35	3841.29			

- Conformer (42); ZPE = 48409.641 cm^{-1}:

28.56	49.99	94.46	102.46	139.25	203.47
230.42	260.11	290.53	293.41	311.01	324.86
433.11	450.80	465.15	530.86	569.67	593.31
595.47	602.45	620.62	636.50	755.41	765.10
778.21	789.46	801.86	824.19	862.55	867.07
875.72	895.61	964.65	978.60	1002.15	1038.08
1054.61	1095.98	1111.03	1127.20	1148.36	1160.70
1175.15	1190.71	1250.55	1261.56	1273.75	1309.99
1342.02	1358.89	1370.28	1375.93	1399.07	1430.42
1463.42	1475.15	1497.90	1540.86	1619.68	1646.17
1648.91	1693.75	1873.85	3008.13	3043.50	3081.07
3176.55	3186.50	3197.93	3210.26	3252.32	3540.39
3630.77	3721.53	3849.36			

- Conformer (43); ZPE = 48381.199 cm^{-1}:

27.78	47.59	66.67	87.93	162.95	204.94
217.48	225.74	300.02	314.76	332.69	399.94
430.15	444.95	451.60	521.56	563.95	581.36
593.92	603.32	628.20	644.21	743.85	766.13
767.63	782.03	794.44	815.88	848.24	872.01
894.23	896.97	958.73	967.83	1005.26	1029.07
1039.77	1080.79	1118.19	1148.39	1160.41	1164.22
1180.05	1194.59	1252.65	1267.20	1283.17	1301.32
1333.63	1343.92	1362.59	1373.50	1402.85	1452.72
1466.94	1472.30	1495.13	1542.64	1620.10	1641.52
1647.77	1692.73	1874.45	2924.47	3039.23	3080.34
3181.44	3188.57	3198.77	3209.43	3249.33	3559.47
3650.57	3722.97	3850.24			

- Conformer (44); ZPE = 48403.625 cm^{-1}:

22.32	38.44	77.05	107.14	157.35	192.50
218.88	230.42	278.69	318.74	361.89	432.90
450.08	462.04	477.42	521.20	549.91	562.81
575.12	596.13	629.33	670.67	721.27	763.79
766.88	778.48	791.34	826.20	842.84	871.76
891.12	909.83	934.32	966.19	996.59	1002.71
1041.89	1074.60	1114.87	1131.42	1156.45	1166.96
1176.78	1218.22	1230.75	1260.62	1278.74	1306.51
1333.09	1355.92	1367.16	1371.28	1399.31	1440.89
1463.32	1473.92	1497.08	1541.12	1613.29	1645.40
1650.81	1692.72	1864.46	2967.15	3046.93	3103.62
3180.65	3188.89	3197.81	3208.99	3271.50	3562.43
3651.55	3720.14	3845.66			

- Conformer (45); ZPE = 48354.315 cm^{-1}:

10.13	31.95	43.50	70.70	174.35	186.59
214.47	222.06	243.39	294.57	345.51	402.83
435.38	459.60	462.12	538.57	543.62	580.29
596.53	607.32	636.12	661.55	726.33	768.48
777.65	789.28	791.10	833.60	838.71	877.17
889.88	901.28	941.37	969.07	997.14	1007.22
1041.37	1086.70	1111.61	1135.86	1157.88	1165.87
1171.29	1198.09	1258.45	1272.06	1278.84	1307.77
1331.35	1359.31	1373.70	1380.37	1391.54	1401.44
1464.66	1471.04	1497.74	1540.81	1625.62	1649.51
1664.40	1694.28	1859.23	3028.93	3063.13	3082.28
3169.14	3184.15	3197.48	3209.69	3261.09	3549.90
3635.98	3722.35	3844.30			

- Conformer (46); ZPE = 48495.407 cm^{-1}:

33.73	65.25	73.58	98.78	147.96	210.37
222.77	270.63	281.68	324.18	341.15	396.69
433.15	461.20	473.39	518.81	554.43	593.13
604.27	646.23	653.27	746.15	755.35	762.80
768.17	781.44	794.40	823.56	846.37	872.33
883.46	895.99	965.39	979.98	995.78	1003.01
1042.83	1093.50	1114.44	1115.85	1143.86	1159.03
1171.72	1229.42	1249.69	1267.16	1279.68	1311.49
1341.46	1354.09	1368.47	1378.44	1397.72	1413.29
1467.22	1474.74	1497.38	1543.97	1620.40	1650.07
1654.43	1694.10	1902.08	3012.77	3041.81	3063.80
3178.21	3185.97	3198.19	3208.90	3254.35	3549.60
3634.83	3720.91	3726.13			

- Conformer (47); ZPE = 48430.133 cm^{-1}:

22.89	50.38	63.69	139.96	140.84	182.84
225.68	255.51	273.68	317.49	352.26	422.96
433.62	461.95	482.46	523.68	556.35	568.35
587.25	597.96	635.45	664.58	725.02	763.97
765.26	783.21	795.08	818.20	864.00	875.43
894.25	925.78	946.65	964.35	996.47	1000.81
1040.28	1074.95	1116.01	1143.23	1153.61	1165.93
1172.90	1215.76	1251.24	1258.73	1273.47	1325.50
1337.51	1354.25	1370.46	1372.58	1408.73	1435.96
1462.59	1473.93	1496.68	1542.54	1615.69	1646.58
1648.68	1692.81	1868.90	2960.89	3015.85	3076.27
3177.13	3184.51	3195.97	3207.85	3271.07	3555.77
3645.90	3727.47	3843.83			

- Conformer (48); ZPE = 48398.743 cm^{-1}:

23.73	40.00	82.80	100.36	139.17	203.98
228.70	252.24	286.47	293.70	308.62	327.88
432.01	448.77	456.64	508.71	567.37	594.23
604.44	622.51	629.59	650.05	756.85	766.88
778.38	790.49	801.53	825.75	848.77	867.24
875.12	895.10	966.65	990.00	1004.15	1038.02
1055.35	1105.55	1120.27	1129.75	1152.89	1161.43
1172.50	1192.62	1240.72	1259.17	1271.93	1309.56
1333.52	1346.77	1368.09	1374.36	1403.15	1460.66
1466.33	1476.78	1497.05	1541.39	1621.08	1641.39
1646.38	1693.77	1870.74	3009.49	3046.40	3069.98
3177.31	3186.45	3197.46	3209.86	3261.86	3533.95
3626.68	3722.90	3835.05			

- Conformer (49); ZPE = 48389.265 cm^{-1}:

29.70	39.57	60.15	77.03	155.67	223.08
226.89	267.40	276.24	337.66	356.60	373.84
434.46	458.77	468.59	505.52	547.68	576.31
596.20	601.73	632.86	659.68	736.70	763.69
769.73	775.04	792.86	815.37	849.68	874.85
879.02	893.96	956.35	963.87	1001.06	1039.59
1051.47	1092.34	1111.42	1139.75	1153.29	1163.33
1192.34	1205.54	1241.23	1261.14	1275.11	1302.81
1334.81	1349.87	1366.81	1382.55	1390.11	1444.75
1463.93	1474.02	1496.34	1540.75	1624.88	1638.39
1649.22	1694.43	1857.91	2941.78	3036.02	3093.82
3167.32	3185.51	3197.03	3209.93	3261.47	3556.90
3649.87	3721.84	3841.07			

- Conformer (50); ZPE = 48383.790 cm^{-1}:

30.36	47.92	79.69	82.85	150.94	216.56
220.90	247.91	270.12	291.20	307.66	389.77
438.36	463.93	476.67	518.57	552.21	585.46
595.79	599.91	607.33	656.39	748.36	765.91
773.53	788.83	801.31	815.49	849.54	856.98
877.16	893.89	957.86	969.70	1004.50	1040.84
1051.58	1089.77	1099.10	1115.75	1150.97	1159.27
1172.90	1177.31	1252.20	1262.67	1276.38	1312.95
1341.82	1346.04	1363.65	1373.51	1392.77	1427.48
1466.05	1473.57	1497.38	1542.63	1616.34	1645.08
1647.99	1693.77	1885.13	3030.61	3072.59	3090.51
3180.42	3187.56	3198.05	3209.90	3252.55	3540.42
3630.07	3719.69	3846.75			

- Conformer (51); ZPE = 48434.081 cm^{-1}:

30.32	49.19	71.92	94.24	166.20	223.63
227.49	240.48	261.79	321.85	326.01	394.24
437.41	466.08	498.26	500.90	547.01	551.70
595.04	604.02	645.91	668.48	749.58	759.81
768.92	779.34	795.75	821.12	850.87	859.37
878.76	895.00	970.05	984.74	1007.64	1036.06
1041.05	1094.06	1116.06	1152.33	1162.22	1174.31
1188.90	1195.96	1249.03	1260.31	1273.57	1309.37
1337.79	1340.14	1360.37	1376.91	1386.46	1447.06
1463.84	1471.24	1495.44	1541.12	1619.14	1642.16
1645.40	1692.67	1885.95	3002.47	3019.02	3080.09
3176.15	3189.75	3200.77	3212.81	3262.19	3544.43
3638.66	3716.21	3823.55			

- Conformer (52); ZPE = 48321.174 cm^{-1}:

27.17	43.65	53.99	78.68	141.47	221.94
226.28	249.80	266.72	282.45	313.87	388.22
434.14	453.79	461.41	516.80	552.31	580.18
593.76	603.23	605.94	652.83	752.80	766.00
774.15	787.05	794.60	814.06	844.68	857.58
875.40	894.34	959.00	966.92	1003.88	1039.78
1054.63	1089.17	1096.30	1112.16	1147.04	1160.62
1170.87	1178.91	1251.36	1258.01	1273.47	1312.81
1337.61	1353.11	1361.11	1375.37	1391.27	1425.22
1464.63	1469.10	1495.85	1542.48	1620.63	1647.93
1649.20	1693.19	1888.47	3024.28	3072.14	3090.41
3169.80	3184.52	3196.51	3208.50	3254.04	3539.74
3630.43	3720.90	3851.69			

- Conformer (53); ZPE = 48401.984 cm^{-1}:

28.98	48.62	76.29	103.01	137.80	205.67
220.37	229.35	276.43	287.20	318.15	333.33
432.77	450.96	463.15	537.03	573.74	591.67
597.51	604.47	620.80	635.35	755.23	766.62
777.28	790.08	802.81	808.94	855.53	875.17
892.01	895.71	966.98	989.82	1003.42	1023.32
1040.31	1083.54	1123.06	1129.10	1153.80	1162.60
1170.33	1233.77	1252.57	1256.09	1274.31	1310.85
1347.54	1368.39	1379.82	1383.50	1410.59	1425.97
1464.88	1480.90	1498.31	1541.67	1620.91	1646.11
1655.78	1694.13	1854.27	3004.20	3011.57	3066.72
3176.68	3185.93	3197.20	3209.66	3250.70	3550.18
3637.46	3721.79	3853.22			

- Conformer (54); ZPE = 48298.026 cm^{-1}:

20.04	22.74	66.27	70.87	153.95	191.97
210.08	221.96	245.10	284.31	341.23	391.72
432.22	448.98	459.97	536.30	552.16	570.74
593.51	596.82	630.73	661.09	728.95	760.54
776.46	786.75	803.38	833.08	848.28	869.81
890.31	898.49	937.72	960.99	985.91	998.77
1039.26	1084.24	1112.85	1135.74	1155.35	1159.15
1171.22	1193.14	1258.21	1267.37	1278.91	1308.71
1332.71	1361.33	1372.62	1378.86	1391.80	1397.74
1464.70	1470.06	1494.07	1542.79	1615.21	1644.78
1658.93	1692.66	1863.06	3020.10	3074.56	3087.71
3182.19	3189.49	3199.27	3209.59	3255.02	3545.38
3633.67	3721.64	3849.76			

- Conformer (55); ZPE = 48363.804 cm^{-1}:

34.92	43.72	76.33	91.53	155.84	210.38
217.52	255.23	263.71	301.91	333.20	360.95
430.58	443.77	462.60	517.65	553.79	584.28
593.32	602.56	616.83	655.76	755.72	765.36
773.20	777.85	794.60	808.16	841.10	874.83
877.21	895.18	968.79	977.75	1005.63	1028.60
1042.21	1087.62	1113.21	1129.34	1152.71	1160.37
1176.58	1202.06	1249.79	1259.95	1278.62	1305.77
1329.51	1342.83	1370.94	1373.61	1397.82	1454.36
1465.14	1472.52	1495.44	1543.70	1611.69	1644.27
1648.20	1692.73	1883.15	2969.93	3036.61	3099.22
3180.86	3190.66	3201.33	3210.34	3249.09	3549.08
3637.11	3723.66	3845.19			

- Conformer (56); ZPE = 48400.532 cm^{-1}:

28.62	40.21	75.19	92.57	169.42	198.95
212.27	225.69	271.40	293.60	324.93	387.61
436.45	460.90	478.49	486.97	550.83	554.70
592.66	606.38	647.55	668.10	746.73	763.95
768.27	778.78	798.50	822.79	854.95	877.33
893.91	924.40	971.31	975.87	1009.04	1032.31
1041.43	1091.42	1114.44	1140.90	1158.14	1167.66
1183.65	1204.52	1238.03	1263.48	1276.16	1307.30
1330.36	1352.14	1367.53	1374.41	1395.35	1417.71
1463.58	1478.45	1495.93	1543.50	1610.87	1647.25
1669.95	1692.22	1881.19	3022.51	3035.97	3097.39
3174.76	3185.62	3198.81	3210.27	3253.94	3534.02
3611.32	3716.20	3831.06			

- Conformer (57); ZPE = 48415.071 cm^{-1}:

36.72	58.72	66.96	96.88	149.14	197.24
222.17	254.60	282.83	312.07	371.28	434.77
449.29	460.96	486.97	496.03	500.12	555.00
579.47	597.56	628.90	657.04	714.60	766.54
775.99	777.03	794.39	844.49	870.57	877.23
895.50	914.03	939.41	969.73	1003.67	1006.94
1040.99	1088.52	1119.07	1141.45	1151.63	1157.39
1163.64	1187.81	1248.75	1263.41	1278.04	1288.45
1332.63	1354.03	1367.88	1374.53	1395.60	1405.58
1466.22	1474.07	1496.34	1542.88	1613.42	1644.99
1670.87	1692.98	1900.73	3009.22	3040.23	3093.56
3181.90	3188.94	3199.85	3209.97	3249.68	3528.02
3609.29	3719.11	3893.61			

- Conformer (58); ZPE = 48379.649 cm^{-1}:

16.52	30.08	76.53	84.41	172.45	191.19
216.70	221.12	264.26	300.93	331.92	382.72
433.74	462.24	489.12	494.67	540.91	557.26
592.18	605.47	635.68	670.40	738.47	766.19
768.04	777.89	797.83	820.19	858.48	875.48
893.55	928.11	969.93	984.04	1007.92	1022.68
1041.03	1088.78	1116.06	1149.33	1158.31	1171.27
1186.41	1213.15	1237.22	1261.03	1275.43	1311.68
1330.50	1352.18	1370.07	1377.91	1396.22	1410.92
1466.74	1483.44	1495.50	1544.17	1607.94	1645.61
1672.88	1692.68	1880.82	3015.97	3037.15	3091.53
3181.67	3188.03	3200.02	3210.78	3254.18	3533.33
3608.30	3713.78	3807.95			

- Conformer (59); ZPE = 48350.927 cm^{-1}:

19.98	36.13	72.77	83.55	153.86	208.97
217.13	219.37	259.58	290.60	314.13	379.21
436.61	459.18	464.97	529.71	553.14	579.62
594.82	600.78	604.02	655.36	754.03	766.61
773.08	788.06	796.96	807.47	845.77	875.07
881.94	895.00	968.75	979.46	1004.98	1009.17
1040.70	1088.81	1115.93	1120.33	1148.87	1158.23
1171.64	1234.94	1247.38	1261.10	1277.85	1309.91
1343.54	1369.02	1370.82	1381.10	1398.60	1425.34
1466.93	1482.12	1495.69	1543.00	1613.20	1645.46
1652.48	1692.04	1865.37	3000.64	3030.81	3093.53
3180.92	3187.30	3197.43	3208.41	3250.46	3548.19
3635.63	3717.71	3850.62			

- Conformer (60); ZPE = 48399.779 cm^{-1}:

24.85	47.93	92.35	110.81	140.93	160.25
190.62	229.12	284.67	314.45	339.02	415.86
432.46	456.44	477.63	529.25	555.08	571.03
594.03	596.67	629.28	662.64	725.74	762.92
768.29	784.06	793.66	835.42	858.57	874.40
884.71	895.93	941.93	965.85	1001.43	1022.71
1039.84	1084.27	1127.53	1128.60	1152.29	1163.03
1181.91	1231.26	1244.38	1260.57	1278.40	1284.95
1329.64	1366.02	1367.90	1375.09	1404.42	1440.85
1462.52	1474.02	1496.67	1542.21	1618.80	1647.09
1662.62	1692.81	1860.97	3022.78	3049.84	3063.89
3181.01	3187.99	3199.47	3210.80	3251.33	3548.00
3630.26	3724.53	3840.00			

- Conformer (61); ZPE = 48427.369 cm^{-1}:

20.79	43.22	68.52	125.51	164.14	191.39
224.09	286.33	312.07	336.71	383.01	417.92
433.50	459.79	476.92	510.01	517.55	562.05
584.07	594.80	611.80	665.31	718.76	763.95
771.11	782.17	797.08	854.82	870.41	879.25
893.78	922.72	952.66	964.83	1002.89	1011.99
1040.27	1070.89	1111.43	1133.86	1154.08	1165.56
1201.79	1216.11	1234.73	1255.22	1275.35	1317.61
1326.94	1340.01	1370.46	1371.10	1401.52	1428.86
1468.33	1489.97	1495.33	1539.71	1608.54	1635.69
1643.37	1691.36	1878.26	2946.11	3027.21	3061.73
3172.48	3186.92	3198.92	3211.23	3271.46	3542.65
3633.76	3715.88	3844.13			

- Conformer (62); ZPE = 48490.346 cm^{-1}:

24.13	45.93	77.23	110.57	173.81	194.69
226.17	242.36	286.48	309.06	355.23	373.46
435.16	461.70	507.13	538.36	566.18	575.82
579.82	597.94	646.52	689.74	750.16	768.42
774.13	784.98	799.16	834.46	867.04	875.72
888.98	893.11	932.26	970.28	999.33	1009.42
1040.78	1073.56	1113.97	1138.53	1153.20	1163.41
1180.41	1200.99	1252.34	1263.66	1278.30	1326.27
1333.21	1355.30	1363.30	1377.10	1390.96	1401.75
1463.50	1466.73	1494.85	1543.25	1604.84	1643.61
1649.27	1692.66	1881.21	3049.73	3090.77	3100.27
3181.70	3189.10	3200.92	3211.74	3256.67	3542.20
3630.75	3713.61	3801.36			

- Conformer (63); ZPE = 48336.056 cm^{-1}:

22.51	38.25	75.00	88.76	164.63	207.70
214.46	225.35	275.24	317.67	325.38	387.78
436.52	462.51	477.42	491.82	496.80	554.27
592.55	604.80	648.10	666.95	745.01	765.10
768.47	779.30	797.92	821.80	842.97	852.88
877.29	894.21	971.08	978.25	1009.16	1033.47
1042.25	1094.25	1114.66	1154.12	1162.58	1173.86
1189.15	1193.26	1244.42	1259.47	1276.17	1306.51
1330.59	1339.37	1349.09	1371.30	1393.63	1446.74
1463.39	1476.60	1496.01	1543.66	1610.05	1641.45
1647.38	1692.16	1883.57	2990.43	3029.17	3079.54
3175.06	3185.74	3198.88	3210.20	3253.28	3545.40
3638.87	3716.09	3842.37			

- Conformer (64); ZPE = 48354.520 cm^{-1}:

28.53	59.54	86.96	95.02	151.01	187.92
207.77	228.54	268.14	312.37	356.16	428.17
434.97	458.96	471.70	490.37	513.92	546.07
583.37	596.00	640.32	656.72	717.34	763.04
772.91	776.53	793.52	837.31	868.65	879.08
891.46	909.51	933.95	966.38	1003.84	1014.07
1043.82	1083.51	1113.92	1145.78	1155.84	1157.56
1166.91	1198.59	1242.18	1262.70	1279.21	1291.27
1332.65	1353.20	1365.64	1379.67	1394.76	1410.07
1460.53	1464.32	1496.13	1542.71	1615.18	1645.95
1659.98	1691.94	1900.56	3009.60	3030.08	3097.31
3183.14	3189.29	3199.74	3210.61	3254.67	3531.72
3612.10	3719.21	3886.86			

- Conformer (65); ZPE = 48424.158 cm^{-1}:

26.25	40.92	92.94	118.19	138.55	178.61
224.23	243.42	288.91	310.21	353.75	422.74
434.69	468.83	479.96	489.02	497.63	561.87
595.00	605.35	628.98	633.43	713.93	765.78
773.69	784.37	798.67	857.75	877.39	880.70
896.45	912.94	955.43	967.72	1004.06	1017.98
1039.59	1078.44	1132.84	1152.08	1159.32	1164.08
1173.82	1200.48	1255.49	1269.96	1282.66	1302.45
1328.60	1361.03	1372.82	1388.12	1391.79	1409.28
1465.09	1484.97	1498.39	1542.57	1617.42	1647.31
1671.10	1694.66	1882.19	2990.36	3002.05	3054.07
3176.85	3187.70	3199.54	3211.59	3242.95	3544.95
3623.63	3721.90	3885.84			

- Conformer (66); ZPE = 48358.521 cm^{-1}:

34.32	46.24	70.40	73.73	172.22	181.69
226.52	235.70	290.79	320.81	350.62	406.34
435.58	460.96	478.05	488.29	527.99	544.51
586.37	598.87	644.69	681.98	708.40	768.49
778.16	789.36	796.44	835.41	853.09	875.15
891.22	894.38	918.14	967.72	1006.68	1022.02
1041.90	1094.13	1114.94	1145.34	1159.80	1161.63
1169.74	1207.32	1246.11	1264.08	1275.27	1305.39
1327.23	1340.05	1353.44	1375.92	1392.63	1437.53
1465.54	1467.35	1497.04	1543.81	1620.46	1632.71
1648.98	1694.88	1896.56	2988.80	3014.85	3077.14
3169.72	3186.43	3199.21	3211.59	3255.22	3535.08
3628.53	3718.16	3891.18			

- Conformer (67); ZPE = 48339.223 cm^{-1}:

17.03	49.53	55.57	101.79	157.61	200.85
214.00	216.05	231.14	304.05	333.29	384.27
434.03	467.41	471.34	484.90	506.05	563.24
587.83	597.75	636.96	677.33	710.16	767.39
777.81	784.27	798.51	865.89	877.28	881.03
894.53	915.45	966.13	988.62	1004.41	1005.38
1041.50	1078.12	1112.12	1150.78	1155.11	1165.32
1166.34	1193.98	1252.95	1264.84	1279.10	1313.88
1326.68	1342.31	1365.09	1373.77	1408.08	1409.38
1466.33	1475.05	1496.94	1541.80	1614.53	1645.47
1667.41	1693.17	1896.20	3000.99	3013.93	3052.73
3176.86	3186.49	3197.70	3209.11	3273.77	3533.04
3609.94	3719.05	3879.69			

- Conformer (68); ZPE = 48363.434 cm^{-1}:

28.68	52.19	73.45	81.67	179.43	198.71
222.62	229.06	294.07	345.88	357.07	430.81
440.87	458.87	471.09	479.96	495.57	553.25
589.07	596.46	614.45	668.87	713.24	770.63
774.72	776.28	793.39	824.88	841.53	877.38
881.80	896.18	935.97	974.64	1010.79	1023.38
1043.09	1096.90	1117.14	1152.99	1158.05	1166.36
1185.68	1207.82	1243.31	1262.66	1282.47	1305.68
1320.91	1343.14	1364.35	1374.51	1395.34	1424.05
1452.97	1467.26	1496.24	1543.88	1615.74	1624.44
1645.85	1693.52	1894.44	2922.51	3019.82	3076.40
3183.31	3192.34	3200.54	3210.08	3248.73	3559.80
3655.56	3719.82	3902.23			

- Conformer (69); ZPE = 48358.090 cm^{-1}:

37.84	45.37	72.28	80.58	163.32	198.39
221.26	228.74	300.28	324.62	348.10	405.26
436.47	461.62	479.87	490.35	517.81	556.01
581.78	596.72	645.40	680.90	708.76	765.50
776.49	787.79	798.04	835.93	849.22	872.22
878.90	895.17	921.93	968.18	1005.40	1013.43
1041.56	1094.70	1115.92	1144.95	1156.46	1157.76
1167.31	1208.47	1249.97	1260.96	1274.21	1304.41
1328.94	1341.92	1351.28	1373.24	1392.01	1436.34
1464.59	1471.53	1495.37	1542.35	1614.92	1632.23
1645.12	1692.54	1897.93	2985.25	3022.15	3072.28
3178.97	3188.62	3200.49	3211.22	3253.66	3544.22
3640.28	3718.53	3893.61			

- Conformer (70); ZPE = 48324.783 cm^{-1}:

28.50	66.79	88.40	99.18	148.72	182.23
193.82	224.26	283.34	323.04	345.63	429.63
437.48	459.72	468.66	483.09	522.23	547.36
583.60	596.15	644.80	657.09	718.97	763.28
772.37	776.98	793.61	836.27	854.50	872.68
891.35	896.00	941.31	967.93	989.22	1004.66
1043.75	1084.77	1115.17	1146.22	1157.62	1167.01
1173.71	1216.79	1249.08	1263.49	1270.54	1280.19
1317.77	1334.47	1367.27	1377.07	1400.35	1440.52
1463.06	1470.05	1496.64	1543.24	1616.71	1644.03
1647.73	1692.38	1904.25	2978.59	3017.70	3069.97
3183.16	3189.14	3199.88	3210.70	3249.14	3539.06
3629.97	3719.25	3886.27			

- Conformer (71); ZPE = 48448.573 cm^{-1}:

17.84	54.27	81.37	126.72	169.93	187.06
229.44	269.31	300.68	340.78	365.74	432.52
445.35	451.41	483.64	516.73	540.48	558.06
588.84	592.65	610.90	666.31	713.21	766.47
772.59	779.58	798.03	839.05	851.00	883.91
894.80	907.88	947.63	981.75	1009.36	1021.41
1041.96	1094.51	1116.86	1135.69	1155.70	1167.67
1212.04	1234.34	1249.97	1258.46	1285.75	1323.19
1334.65	1344.19	1364.07	1384.97	1389.31	1430.42
1457.28	1470.72	1494.80	1544.03	1608.17	1635.99
1642.32	1692.10	1877.15	2940.19	3027.16	3069.06
3185.83	3196.67	3206.16	3213.23	3246.09	3541.05
3632.21	3716.75	3779.68			

- Conformer (72); ZPE = 48270.330 cm^{-1}:

32.05	49.92	63.12	69.24	174.11	189.66
211.87	226.38	236.49	292.91	355.88	379.09
436.30	458.68	459.65	460.29	522.28	553.49
581.61	596.67	632.86	666.49	754.33	764.14
767.88	777.15	793.75	831.12	846.99	876.62
893.23	894.43	949.65	968.23	1005.55	1018.59
1042.15	1091.57	1112.22	1139.75	1150.83	1160.62
1169.24	1189.35	1248.42	1265.07	1278.96	1284.95
1320.26	1345.69	1364.02	1376.10	1391.00	1442.38
1459.71	1463.25	1497.30	1541.64	1619.99	1645.74
1649.15	1693.98	1908.18	2930.15	3051.15	3106.11
3175.55	3187.25	3199.54	3211.72	3255.82	3540.11
3629.04	3719.16	3892.84			

- Conformer (73); ZPE = 48280.253 cm^{-1}:

28.86	48.72	64.13	96.20	158.08	200.50
221.03	226.07	249.52	314.74	348.91	427.25
430.87	446.93	470.26	490.47	500.25	561.68
593.52	598.33	628.67	648.63	713.07	762.64
766.32	775.50	785.04	797.63	830.74	872.51
894.10	895.34	939.41	968.18	1005.28	1034.52
1041.66	1087.37	1120.36	1150.70	1160.95	1164.98
1192.41	1218.04	1247.31	1264.49	1273.23	1308.00
1318.54	1340.88	1365.18	1375.07	1401.82	1432.05
1460.19	1466.59	1496.02	1543.70	1617.99	1621.58
1648.23	1693.89	1896.64	2905.24	2997.10	3065.42
3181.19	3188.73	3199.81	3210.87	3249.05	3573.22
3674.72	3723.10	3890.25			

- Conformer (74); ZPE = 48267.916 cm^{-1}:

25.30	45.54	72.18	85.78	147.66	194.42
217.72	226.49	261.21	299.03	329.61	382.14
431.26	461.65	469.20	493.83	523.27	551.12
595.44	601.41	621.24	659.48	753.31	760.53
765.17	776.50	791.29	806.80	856.87	871.37
878.41	893.43	960.60	966.15	1000.01	1036.87
1040.66	1088.45	1112.99	1120.98	1149.07	1158.21
1167.78	1207.65	1239.00	1258.61	1275.39	1298.75
1309.36	1340.02	1367.92	1381.29	1394.22	1441.59
1465.03	1468.02	1494.14	1539.94	1619.34	1645.04
1651.90	1691.12	1906.61	2923.35	3033.45	3104.24
3171.58	3186.26	3197.26	3208.56	3262.40	3548.53
3637.99	3721.60	3895.21			

- Conformer (75); ZPE = 48252.847 cm^{-1}:

18.93	28.60	61.37	70.46	142.84	204.64
222.77	234.90	262.01	304.46	327.69	391.60
432.76	454.70	461.55	480.38	530.31	552.52
594.28	601.80	611.96	654.43	753.07	766.48
773.19	781.96	793.24	811.18	844.74	861.13
875.40	894.50	960.74	966.91	1005.17	1011.87
1039.42	1078.36	1116.78	1126.55	1140.80	1158.21
1170.35	1200.24	1236.50	1258.96	1274.20	1319.50
1338.06	1344.67	1356.51	1373.94	1392.32	1396.08
1465.41	1483.41	1496.44	1542.97	1621.63	1648.15
1653.16	1694.29	1899.57	2997.55	3069.16	3079.04
3172.07	3185.97	3197.50	3208.67	3250.94	3540.16
3631.25	3719.37	3882.99			

- Conformer (76); ZPE = 48288.262 cm^{-1}:

25.14	30.67	67.56	73.58	168.61	189.61
219.21	223.23	258.40	275.09	351.74	361.52
432.34	459.27	467.68	492.44	536.09	549.91
587.14	594.08	630.38	666.06	757.85	762.70
776.24	784.97	796.25	833.38	851.39	869.69
892.87	902.66	932.52	962.26	999.96	1008.16
1040.29	1087.20	1115.12	1138.71	1151.88	1156.71
1173.64	1202.53	1258.95	1266.16	1279.48	1300.77
1336.07	1340.56	1366.67	1379.09	1392.36	1404.11
1464.66	1468.69	1493.32	1543.14	1615.65	1644.69
1656.62	1691.91	1891.57	2972.14	3056.32	3090.31
3184.48	3194.47	3204.21	3213.16	3255.36	3529.74
3624.40	3719.88	3880.85			

12.5 Cartesian coordinates of Trp conformers

Each line starting by an asterisk contains the relative energy of the corresponding conformer and the dihedral angle for each of the target torsions (separated by underscores). Cartesian coordinates are given in Å.

```
27
 * E =  +0.000  kcal/mol ; (1) 356_018_089_292_268
 H    +0.00000    +0.00000    +0.00000
 O    +0.00000    +0.00000    +0.97288
 C    +1.27143    +0.00000    +1.35263
 C    +2.28120    −0.10127    +0.19711
 N    +1.62186    +0.24347    −1.05939
 H    +1.68435    +1.24284    −1.23391
 O    +1.60594    +0.04883    +2.50187
 C    +3.52490    +0.73422    +0.53609
 H    +2.55906    −1.16234    +0.15267
 H    +2.03666    −0.23517    −1.84687
 C    +3.21043    +2.19757    +0.56134
 H    +3.90340    +0.40526    +1.50749
 H    +4.30144    +0.53053    −0.21008
 C    +3.32612    +3.12016    −0.53472
 C    +2.86582    +4.37507    −0.07550
 N    +2.49280    +4.21353    +1.23165
 C    +2.69512    +2.91034    +1.60841
 C    +3.77601    +3.00253    −1.85686
```

C +2.84066 +5.50135 −0.89747
H +2.11737 +4.93203 +1.82376
H +2.44951 +2.56802 +2.60451
C +3.75105 +4.11477 −2.67257
H +4.14422 +2.05003 −2.23102
H +4.09797 +4.03953 −3.69796
C +3.28616 +5.35294 −2.19522
H +2.48422 +6.45984 −0.53288
H +3.27913 +6.20940 −2.86150
27
* E = +1.773 kcal/mol ; (2) 182_184_306_300_275
H +0.00000 +0.00000 +0.00000
O +0.00000 +0.00000 +0.96386
C +1.28367 +0.00000 +1.36978
C +1.41327 −0.06133 +2.88823
N +2.78414 +0.02854 +3.33652
H +3.35173 −0.67283 +2.87502
O +2.19950 +0.02381 +0.59939
C +0.55318 +1.03753 +3.53822
H +1.00289 −1.03499 +3.19007
H +3.17242 +0.92740 +3.06454
C +0.96479 +2.41229 +3.11259
H −0.49634 +0.86509 +3.28524
H +0.65835 +0.93126 +4.62289
C +2.00522 +3.22390 +3.68529
C +2.05118 +4.42627 +2.94623
N +1.08831 +4.34358 +1.97554
C +0.44162 +3.13752 +2.07960
C +2.89111 +3.04568 +4.75674
C +2.95420 +5.44679 +3.24462
H +0.88660 +5.05162 +1.29364
H −0.36505 +2.87852 +1.40735
C +3.78306 +4.05439 +5.05505
H +2.87943 +2.12072 +5.32733
H +4.47687 +3.93248 +5.88039
C +3.81351 +5.24341 +4.30455
H +2.98112 +6.36597 +2.66752
H +4.52964 +6.01644 +4.56423
27
* E = +1.831 kcal/mol ; (3) 358_008_100_281_080

H	+0.00000	+0.00000	+0.00000
O	+0.00000	+0.00000	+0.97318
C	+1.27007	+0.00000	+1.34970
C	+2.28591	−0.04062	+0.19609
N	+1.60652	+0.11164	−1.08545
H	+1.69385	+1.06653	−1.42094
O	+1.61104	−0.00752	+2.49852
C	+3.42775	+0.96887	+0.44380
H	+2.71013	−1.05014	+0.25112
H	+1.97327	−0.51011	−1.79174
C	+3.04611	+2.37961	+0.12301
H	+3.73262	+0.87736	+1.49143
H	+4.28216	+0.67709	−0.17634
C	+2.28464	+3.29952	+0.93000
C	+2.16168	+4.49515	+0.18865
N	+2.81245	+4.30965	−1.00351
C	+3.34098	+3.04400	−1.03581
C	+1.72301	+3.23302	+2.21340
C	+1.48777	+5.61300	+0.68089
H	+2.91316	+4.99985	−1.72499
H	+3.92071	+2.70810	−1.88537
C	+1.05889	+4.33794	+2.70221
H	+1.80745	+2.32761	+2.80667
H	+0.61717	+4.30134	+3.69251
C	+0.93959	+5.51491	+1.94228
H	+1.40112	+6.52434	+0.09726
H	+0.40742	+6.36446	+2.35836

27

∗ E = +1.841 kcal/mol ; (4) 004_343_153_177_292

H	+0.00000	+0.00000	+0.00000
O	+0.00000	+0.00000	+0.97218
C	+1.27481	+0.00000	+1.35567
C	+2.28182	+0.08580	+0.20731
N	+1.64667	−0.25582	−1.05954
H	+2.12275	+0.20956	−1.82541
O	+1.61128	−0.01724	+2.50292
C	+2.79817	+1.53779	+0.16001
H	+3.11918	−0.57440	+0.46672
H	+1.68221	−1.25135	−1.24032
C	+3.84202	+1.72097	−0.89006

H	+1.94830	+2.20451	−0.03250
H	+3.18813	+1.78351	+1.15369
C	+5.16575	+1.15959	−0.87898
C	+5.79372	+1.54891	−2.08127
N	+4.89369	+2.30663	−2.78470
C	+3.72684	+2.40552	−2.06688
C	+5.87604	+0.38202	+0.04436
C	+7.10196	+1.17557	−2.38887
H	+5.06580	+2.74045	−3.67319
H	+2.89135	+2.97932	−2.44472
C	+7.16994	+0.01143	−0.25653
H	+5.41906	+0.08589	+0.98518
H	+7.73619	−0.58878	+0.44788
C	+7.77463	+0.40385	−1.46390
H	+7.57569	+1.48160	−3.31645
H	+8.79452	+0.09577	−1.67030

27

∗ E = +2.132 kcal/mol ; (5) 178_175_299_299_095

H	+0.00000	+0.00000	+0.00000
O	+0.00000	+0.00000	+0.96420
C	+1.27988	+0.00000	+1.37384
C	+1.39806	+0.05453	+2.89206
N	+2.76587	−0.06311	+3.34586
H	+3.15516	−0.95440	+3.06196
O	+2.20363	−0.03897	+0.61349
C	+0.77249	+1.36322	+3.41908
H	+0.81128	−0.78240	+3.29045
H	+3.32491	+0.64582	+2.87989
C	+1.46056	+2.57885	+2.88241
H	−0.28872	+1.38835	+3.15136
H	+0.84313	+1.33002	+4.50991
C	+1.07028	+3.34683	+1.73111
C	+2.03186	+4.36730	+1.56406
N	+2.95446	+4.22386	+2.56578
C	+2.60782	+3.15374	+3.35288
C	−0.01353	+3.28148	+0.84570
C	+1.94942	+5.30494	+0.53464
H	+3.75560	+4.81119	+2.70813
H	+3.20645	+2.87549	+4.20971
C	−0.09945	+4.20863	−0.17163

H	−0.77404	+2.51496	+0.96629
H	−0.93444	+4.17358	−0.86393
C	+0.87692	+5.20863	−0.32748
H	+2.69772	+6.08256	+0.41610
H	+0.78143	+5.92182	−1.13988

27

∗ E = +2.172 kcal/mol ; (6) 178_022_306_294_273

H	+0.00000	+0.00000	+0.00000
O	+0.00000	+0.00000	+0.96388
C	+1.27849	+0.00000	+1.36991
C	+1.42197	+0.05629	+2.89108
N	+0.27360	−0.39609	+3.64929
H	−0.56826	+0.08756	+3.36336
O	+2.19531	−0.00731	+0.59544
C	+2.68214	−0.71886	+3.29830
H	+1.58440	+1.11997	+3.11855
H	+0.11892	−1.38806	+3.49844
C	+2.56303	−2.18594	+3.02214
H	+3.53655	−0.30437	+2.75755
H	+2.84271	−0.55051	+4.36798
C	+2.00649	−3.18909	+3.89014
C	+2.09506	−4.42480	+3.21146
N	+2.67209	−4.17760	+1.99388
C	+2.94907	−2.83756	+1.88468
C	+1.44693	−3.15368	+5.17448
C	+1.64427	−5.61672	+3.77863
H	+2.86734	−4.86672	+1.29098
H	+3.40643	−2.42999	+0.99348
C	+1.00177	−4.33154	+5.73792
H	+1.35472	−2.20907	+5.70404
H	+0.56504	−4.32181	+6.73123
C	+1.10061	−5.55138	+5.04528
H	+1.71808	−6.56043	+3.24683
H	+0.74004	−6.46007	+5.51658

27

∗ E = +2.252 kcal/mol ; (7) 004_341_156_178_090

H	+0.00000	+0.00000	+0.00000
O	+0.00000	+0.00000	+0.97208
C	+1.27424	+0.00000	+1.35763
C	+2.28341	+0.09133	+0.21014

N	+1.65210	−0.28449	−1.04957
H	+2.13627	+0.12745	−1.83976
O	+1.60773	−0.01700	+2.50576
C	+2.77093	+1.55125	+0.15228
H	+3.13527	−0.54480	+0.48288
H	+1.65574	−1.28845	−1.18292
C	+3.80961	+1.77741	−0.89904
H	+1.90434	+2.19830	−0.03903
H	+3.15255	+1.81104	+1.14381
C	+3.57633	+2.15022	−2.26847
C	+4.83592	+2.22163	−2.90356
N	+5.78095	+1.90956	−1.96318
C	+5.16294	+1.64703	−0.76632
C	+2.42140	+2.43934	−3.00906
C	+4.97000	+2.56462	−4.24875
H	+6.77276	+1.89391	−2.11704
H	+5.74305	+1.39121	+0.10964
C	+2.55141	+2.77887	−4.33944
H	+1.44105	+2.39775	−2.54010
H	+1.66751	+3.00717	−4.92576
C	+3.81545	+2.83931	−4.95254
H	+5.94393	+2.61622	−4.72556
H	+3.88362	+3.11014	−6.00111

27

∗ E = +2.364 kcal/mol ; (8) 186_343_301_293_086

H	+0.00000	+0.00000	+0.00000
O	+0.00000	+0.00000	+0.96431
C	+1.27394	+0.00000	+1.37389
C	+1.42576	−0.14956	+2.88361
N	+0.24352	+0.10805	+3.67550
H	−0.10152	+1.04801	+3.52731
O	+2.20139	+0.07177	+0.61493
C	+1.94048	−1.58026	+3.16022
H	+2.21699	+0.55078	+3.17208
H	−0.49609	−0.53083	+3.40240
C	+0.92957	−2.62872	+2.81776
H	+2.85983	−1.73651	+2.58565
H	+2.19151	−1.62980	+4.22356
C	+0.70810	−3.23109	+1.52972
C	−0.37367	−4.12764	+1.66986

N	−0.78373	−4.07102	+2.97577
C	−0.00034	−3.17287	+3.65784
C	+1.33012	−3.10277	+0.28027
C	−0.85340	−4.88710	+0.60268
H	−1.52866	−4.61105	+3.37631
H	−0.15755	−2.98497	+4.71127
C	+0.85719	−3.85280	−0.77676
H	+2.16767	−2.42281	+0.14730
H	+1.32951	−3.76675	−1.74998
C	−0.22612	−4.73506	−0.61662
H	−1.68636	−5.57266	+0.72508
H	−0.57284	−5.31085	−1.46876

27

∗ E = +2.421 kcal/mol ; (9) 357_018_089_175_293

H	+0.00000	+0.00000	+0.00000
O	+0.00000	+0.00000	+0.97171
C	+1.27340	+0.00000	+1.35707
C	+2.28245	−0.08304	+0.20527
N	+1.63971	+0.28062	−1.05439
H	+1.71127	+1.28087	−1.21296
O	+1.60355	+0.03223	+2.50605
C	+3.54836	+0.71955	+0.54180
H	+2.55056	−1.14579	+0.13487
H	+2.10102	−0.16968	−1.83458
C	+4.58713	+0.56270	−0.51784
H	+3.27553	+1.77673	+0.65525
H	+3.91558	+0.38699	+1.51837
C	+5.29905	−0.64652	−0.83217
C	+6.13500	−0.36557	−1.93477
N	+5.93788	+0.94800	−2.27005
C	+5.00908	+1.49737	−1.41848
C	+5.31692	−1.92993	−0.27066
C	+6.97417	−1.32956	−2.49333
H	+6.41522	+1.44037	−3.00266
H	+4.72217	+2.53600	−1.51137
C	+6.14491	−2.88584	−0.82121
H	+4.70068	−2.16412	+0.59391
H	+6.17282	−3.88399	−0.39695
C	+6.96348	−2.58674	−1.92453
H	+7.61403	−1.10166	−3.34024

H +7.60447 −3.36066 −2.33425
27
∗ E = +2.716 kcal/mol ; (10) 356_021_084_175_087
H +0.00000 +0.00000 +0.00000
O +0.00000 +0.00000 +0.97151
C +1.27296 +0.00000 +1.36020
C +2.28297 −0.09659 +0.20982
N +1.65115 +0.31867 −1.03954
H +1.68190 +1.32856 −1.13653
O +1.59886 +0.03825 +2.51009
C +3.58627 +0.63051 +0.56670
H +2.50509 −1.16834 +0.11463
H +2.14609 −0.06050 −1.83746
C +4.63500 +0.44124 −0.48239
H +3.36770 +1.69949 +0.69527
H +3.92656 +0.26877 +1.54081
C +4.83037 +1.24352 −1.65949
C +5.89262 +0.66567 −2.38989
N +6.31827 −0.42945 −1.68728
C +5.56543 −0.55687 −0.54597
C +4.22344 +2.40648 −2.15268
C +6.34600 +1.20347 −3.59454
H +7.07719 −1.03136 −1.95005
H +5.75722 −1.35873 +0.15380
C +4.66919 +2.94220 −3.34255
H +3.42594 +2.89330 −1.59639
H +4.21044 +3.84451 −3.73321
C +5.71966 +2.34138 −4.05893
H +7.16292 +0.74768 −4.14548
H +6.04778 +2.78666 −4.99249
27
∗ E = +3.172 kcal/mol ; (11) 181_179_323_298_189
H +0.00000 +0.00000 +0.00000
O +0.00000 +0.00000 +0.96381
C +1.27223 +0.00000 +1.38167
C +1.37475 −0.02839 +2.90377
N +2.73542 −0.05677 +3.38842
H +3.32289 −0.61817 +2.78352
O +2.20365 −0.01387 +0.62500
C +0.58569 +1.12436 +3.53224

H	+0.87112	−0.96071	+3.19832
H	+3.12524	+0.87828	+3.40364
C	+1.06640	+2.49428	+3.15753
H	−0.47034	+1.01855	+3.25846
H	+0.64502	+1.00215	+4.62053
C	+0.59520	+3.71158	+3.75891
C	+1.26118	+4.78138	+3.12452
N	+2.09464	+4.23760	+2.18485
C	+1.97701	+2.86775	+2.20761
C	−0.32881	+3.98621	+4.77344
C	+1.03064	+6.11091	+3.47707
H	+2.70590	+4.75189	+1.57765
H	+2.56287	+2.26020	+1.53011
C	−0.56013	+5.29994	+5.12536
H	−0.85406	+3.17744	+5.27434
H	−1.27260	+5.53094	+5.91043
C	+0.11510	+6.35162	+4.48136
H	+1.54937	+6.92645	+2.98263
H	−0.08846	+7.37470	+4.78090

27

* E = +3.314 kcal/mol ; (12) 183_192_198_177_285

H	+0.00000	+0.00000	+0.00000
O	+0.00000	+0.00000	+0.96367
C	+1.27845	+0.00000	+1.37178
C	+1.40235	−0.06285	+2.87867
N	+2.77210	+0.22665	+3.24130
H	+2.96921	−0.08585	+4.18240
O	+2.20020	+0.00735	+0.60646
C	+0.44739	+0.91736	+3.58358
H	+1.07960	−1.08801	+3.14446
H	+3.41870	−0.20467	+2.59259
C	+0.54373	+0.76757	+5.06607
H	+0.72131	+1.93476	+3.28545
H	−0.57790	+0.73017	+3.24738
C	−0.00738	−0.30866	+5.84334
C	+0.36661	−0.08900	+7.18721
N	+1.11016	+1.06132	+7.21788
C	+1.21505	+1.56767	+5.94400
C	−0.78675	−1.42750	+5.52492
C	−0.01520	−0.95339	+8.21282

H +1.50596 +1.47777 +8.04059
H +1.77132 +2.47432 +5.75076
C −1.16481 −2.28573 +6.53680
H −1.09325 −1.61117 +4.49799
H −1.76997 −3.15676 +6.30784
C −0.78067 −2.04912 +7.86842
H +0.27561 −0.77333 +9.24323
H −1.09484 −2.74229 +8.64209
27
* E = +3.329 kcal/mol ; (13) 173_144_293_058_275
H +0.00000 +0.00000 +0.00000
O +0.00000 +0.00000 +0.96482
C +1.26756 +0.00000 +1.38400
C +1.40372 +0.18142 +2.88933
N +2.50372 −0.58276 +3.44448
H +2.32682 −1.57768 +3.37533
O +2.20623 −0.09823 +0.64093
C +1.64695 +1.67903 +3.17148
H +0.46469 −0.12052 +3.36408
H +3.34374 −0.39728 +2.90469
C +0.57082 +2.58730 +2.67042
H +1.77389 +1.77631 +4.25353
H +2.60843 +1.94762 +2.71220
C +0.45519 +3.13876 +1.34740
C −0.73393 +3.89923 +1.31398
N −1.30489 +3.81478 +2.55612
C −0.51882 +3.02960 +3.36302
C +1.24618 +3.05801 +0.19366
C −1.15314 +4.57308 +0.16702
H −2.15577 +4.26561 +2.83818
H −0.79690 +2.84505 +4.39156
C +0.83284 +3.72205 −0.94312
H +2.16560 +2.47804 +0.19273
H +1.43498 +3.67063 −1.84444
C −0.35678 +4.47202 −0.95534
H −2.06950 +5.15531 +0.15539
H −0.65279 +4.98359 −1.86560
27
* E = +3.429 kcal/mol ; (14) 176_159_040_178_288
H +0.00000 +0.00000 +0.00000

O	+0.00000	+0.00000	+0.96384
C	+1.27599	+0.00000	+1.37685
C	+1.38680	+0.08719	+2.88045
N	+2.68357	−0.40554	+3.28028
H	+3.39239	−0.09226	+2.62607
O	+2.20477	−0.00823	+0.61964
C	+1.09816	+1.56165	+3.27914
H	+0.59896	−0.54046	+3.31649
H	+2.91649	−0.07846	+4.21033
C	+1.14110	+1.73701	+4.75895
H	+1.84713	+2.20137	+2.79670
H	+0.11349	+1.84937	+2.88939
C	+0.16011	+1.25946	+5.69503
C	+0.61520	+1.59831	+6.98714
N	+1.81365	+2.24637	+6.83921
C	+2.12428	+2.32414	+5.50295
C	−1.06059	+0.58870	+5.55307
C	−0.11147	+1.28237	+8.13488
H	+2.37295	+2.61513	+7.58633
H	+3.03180	+2.81136	+5.17342
C	−1.78220	+0.27477	+6.68583
H	−1.43363	+0.32475	+4.56657
H	−2.73033	−0.24479	+6.59480
C	−1.30917	+0.61853	+7.96452
H	+0.24781	+1.54799	+9.12428
H	−1.89996	+0.35653	+8.83632

27

∗ E = +3.465 kcal/mol ; (15) 183_193_198_176_082

H	+0.00000	+0.00000	+0.00000
O	+0.00000	+0.00000	+0.96372
C	+1.27884	+0.00000	+1.37050
C	+1.40881	−0.06419	+2.87876
N	+2.77671	+0.24832	+3.22959
H	+2.99267	−0.05178	+4.17137
O	+2.19898	+0.00543	+0.60330
C	+0.43122	+0.88869	+3.58730
H	+1.10657	−1.09629	+3.14054
H	+3.42466	−0.17528	+2.57696
C	+0.49689	+0.75869	+5.07672
H	+0.68304	+1.91372	+3.28879

H	−0.58493	+0.68100	+3.24046
C	+1.44764	+1.36900	+5.96884
C	+1.11576	+0.95593	+7.27806
N	+0.02287	+0.13572	+7.18195
C	−0.34515	+0.02566	+5.86378
C	+2.53436	+2.23505	+5.78233
C	+1.83647	+1.37605	+8.39629
H	−0.44973	−0.29781	+7.95370
H	−1.20309	−0.56764	+5.57849
C	+3.24569	+2.65495	+6.88675
H	+2.81269	+2.54970	+4.78073
H	+4.08962	+3.32510	+6.75942
C	+2.90027	+2.22750	+8.18148
H	+1.57080	+1.04882	+9.39684
H	+3.48295	+2.57514	+9.02859

27

∗ E = +3.774 kcal/mol ; (16) 179_044_181_176_288

H	+0.00000	+0.00000	+0.00000
O	+0.00000	+0.00000	+0.96362
C	+1.27825	+0.00000	+1.37703
C	+1.38033	+0.02029	+2.88948
N	+0.43052	−0.92410	+3.45686
H	+0.49078	−0.89492	+4.46816
O	+2.20145	+0.02099	+0.61511
C	+2.80237	−0.33108	+3.33199
H	+1.17286	+1.06786	+3.18175
H	−0.51811	−0.69986	+3.18421
C	+2.94887	−0.22164	+4.81454
H	+3.02267	−1.35020	+2.99804
H	+3.50452	+0.33559	+2.82014
C	+2.97948	+0.99643	+5.57815
C	+3.08112	+0.63551	+6.93950
N	+3.10869	−0.73293	+6.99544
C	+3.02718	−1.24002	+5.72006
C	+2.94375	+2.35299	+5.23240
C	+3.14075	+1.58833	+7.95632
H	+3.19185	−1.28056	+7.83212
H	+3.03573	−2.30716	+5.54591
C	+3.00209	+3.29829	+6.23536
H	+2.88017	+2.65441	+4.18979

H	+2.97735	+4.35361	+5.98438
C	+3.09788	+2.91689	+7.58521
H	+3.22003	+1.29916	+8.99979
H	+3.14198	+3.68422	+8.35138

27

∗ E = +3.783 kcal/mol ; (17) 177_135_292_056_071

H	+0.00000	+0.00000	+0.00000
O	+0.00000	+0.00000	+0.96443
C	+1.26968	+0.00000	+1.38099
C	+1.40852	+0.07985	+2.89573
N	+2.37669	−0.86399	+3.42028
H	+2.05891	−1.81903	+3.30939
O	+2.20588	−0.04014	+0.62997
C	+1.86380	+1.51145	+3.23163
H	+0.42784	−0.10903	+3.34488
H	+3.24657	−0.77510	+2.90421
C	+0.94002	+2.55144	+2.69058
H	+1.95090	+1.57816	+4.32150
H	+2.86927	+1.64466	+2.81376
C	−0.38153	+2.84696	+3.16961
C	−0.91657	+3.84719	+2.32960
N	+0.03438	+4.13605	+1.38605
C	+1.14322	+3.35488	+1.60667
C	−1.15164	+2.36361	+4.23359
C	−2.19329	+4.37316	+2.52495
H	−0.05496	+4.82218	+0.65937
H	+2.01063	+3.42698	+0.96474
C	−2.41552	+2.88018	+4.42958
H	−0.75649	+1.59763	+4.89645
H	−3.02586	+2.51762	+5.25017
C	−2.93012	+3.87597	+3.58087
H	−2.59444	+5.14410	+1.87418
H	−3.92810	+4.26248	+3.76098

27

∗ E = +3.879 kcal/mol ; (18) 179_043_184_176_084

H	+0.00000	+0.00000	+0.00000
O	+0.00000	+0.00000	+0.96363
C	+1.27871	+0.00000	+1.37637
C	+1.38399	+0.02145	+2.88994
N	+0.41993	−0.90824	+3.45804

H	+0.43907	−0.85427	+4.47016
O	+2.20066	+0.02171	+0.61301
C	+2.80678	−0.33590	+3.32393
H	+1.19154	+1.07299	+3.17755
H	−0.51945	−0.69753	+3.14511
C	+2.99928	−0.22778	+4.80396
H	+3.01638	−1.35825	+2.98770
H	+3.50512	+0.32118	+2.79805
C	+2.68701	−1.22301	+5.79589
C	+3.02758	−0.68088	+7.05489
N	+3.51956	+0.57814	+6.83530
C	+3.50409	+0.84150	+5.48747
C	+2.16511	−2.52277	+5.73140
C	+2.85750	−1.39398	+8.24179
H	+3.86096	+1.20183	+7.54338
H	+3.87269	+1.78380	+5.10591
C	+1.99823	−3.23036	+6.90345
H	+1.88843	−2.95238	+4.77242
H	+1.59565	−4.23750	+6.87091
C	+2.34059	−2.66954	+8.14695
H	+3.12330	−0.96496	+9.20306
H	+2.19579	−3.25322	+9.05040

27

∗ E = +3.883 kcal/mol ; (19) 176_158_041_181_088

H	+0.00000	+0.00000	+0.00000
O	+0.00000	+0.00000	+0.96386
C	+1.27700	+0.00000	+1.37539
C	+1.39136	+0.08923	+2.87997
N	+2.68549	−0.41606	+3.27320
H	+3.40039	−0.09080	+2.63204
O	+2.20399	−0.00739	+0.61643
C	+1.09979	+1.56380	+3.27250
H	+0.60125	−0.53309	+3.31843
H	+2.92197	−0.12740	+4.21437
C	+1.20369	+1.78571	+4.74636
H	+1.81998	+2.20663	+2.74892
H	+0.10028	+1.83387	+2.91390
C	+2.39228	+2.12684	+5.48048
C	+2.03494	+2.20552	+6.84434
N	+0.69704	+1.92633	+6.92884

C	+0.20445	+1.67794	+5.67182
C	+3.71881	+2.38318	+5.10788
C	+2.96131	+2.52405	+7.83749
H	+0.15689	+1.91451	+7.77464
H	−0.84175	+1.44649	+5.52636
C	+4.63664	+2.70037	+6.08682
H	+4.01596	+2.33245	+4.06340
H	+5.66726	+2.90221	+5.81421
C	+4.25965	+2.76790	+7.44013
H	+2.67413	+2.57931	+8.88302
H	+5.00637	+3.01790	+8.18681

27

* E = +3.885 kcal/mol ; (20) 181_176_315_169_201

H	+0.00000	+0.00000	+0.00000
O	+0.00000	+0.00000	+0.96383
C	+1.27547	+0.00000	+1.37951
C	+1.39091	−0.02510	+2.89963
N	+2.76677	−0.11177	+3.34370
H	+3.26744	−0.80355	+2.79705
O	+2.20364	−0.00097	+0.62142
C	+0.68838	+1.19603	+3.50233
H	+0.84811	−0.92097	+3.23504
H	+3.23536	+0.77196	+3.17251
C	+0.52330	+1.13590	+4.98874
H	+1.25099	+2.09692	+3.21107
H	−0.29944	+1.29225	+3.03879
C	−0.44555	+1.89126	+5.73432
C	−0.29217	+1.54309	+7.09406
N	+0.71990	+0.62393	+7.16255
C	+1.20946	+0.38518	+5.90058
C	−1.42891	+2.82073	+5.37595
C	−1.08835	+2.09721	+8.09566
H	+1.06246	+0.19542	+8.00260
H	+2.02993	−0.29721	+5.73369
C	−2.21885	+3.37165	+6.36423
H	−1.56680	+3.10558	+4.33628
H	−2.98512	+4.09396	+6.10229
C	−2.04826	+3.01224	+7.71224
H	−0.96062	+1.82081	+9.13786
H	−2.68499	+3.46308	+8.46665

27
* E = +3.926 kcal/mol ; (21) 184_083_299_066_080

H	+0.00000	+0.00000	+0.00000
O	+0.00000	+0.00000	+0.96405
C	+1.27599	+0.00000	+1.38012
C	+1.38092	−0.10841	+2.89636
N	+1.28313	−1.50583	+3.30511
H	+0.39633	−1.91067	+3.03021
O	+2.20276	+0.00279	+0.61785
C	+2.70008	+0.49686	+3.36853
H	+0.53973	+0.43840	+3.33759
H	+2.02355	−2.05250	+2.87874
C	+2.75395	+1.97090	+3.13028
H	+2.80259	+0.27193	+4.43560
H	+3.52093	−0.00496	+2.84360
C	+2.13894	+2.98048	+3.94667
C	+2.39763	+4.22714	+3.33611
N	+3.12930	+3.97874	+2.20444
C	+3.33486	+2.62586	+2.08373
C	+1.39854	+2.94146	+5.13398
C	+1.93978	+5.42680	+3.87971
H	+3.45915	+4.67308	+1.55957
H	+3.87487	+2.21416	+1.24225
C	+0.94177	+4.12634	+5.67349
H	+1.18984	+1.99235	+5.62184
H	+0.36617	+4.11415	+6.59327
C	+1.21115	+5.35729	+5.05021
H	+2.14660	+6.38027	+3.40328
H	+0.83865	+6.27227	+5.49945

27
* E = +3.961 kcal/mol ; (22) 174_166_039_307_096

H	+0.00000	+0.00000	+0.00000
O	+0.00000	+0.00000	+0.96416
C	+1.27838	+0.00000	+1.37168
C	+1.38872	+0.14003	+2.87520
N	+2.74207	−0.18935	+3.25816
H	+3.39696	+0.17865	+2.57689
O	+2.20439	−0.05601	+0.61626
C	+0.92114	+1.57109	+3.26203
H	+0.68815	−0.57425	+3.32641

H	+2.96892	+0.17645	+4.17320
C	+1.64059	+2.63737	+2.50163
H	−0.15707	+1.65206	+3.08391
H	+1.07557	+1.68141	+4.34158
C	+1.17266	+3.28488	+1.30579
C	+2.19624	+4.15546	+0.87337
N	+3.22907	+4.04182	+1.76476
C	+2.89248	+3.13136	+2.73575
C	−0.02138	+3.22254	+0.57563
C	+2.06523	+4.94872	−0.26647
H	+4.09859	+4.54024	+1.71514
H	+3.57968	+2.90700	+3.53994
C	−0.15506	+4.00667	−0.55117
H	−0.82812	+2.56931	+0.89708
H	−1.07514	+3.97253	−1.12562
C	+0.88195	+4.85954	−0.96956
H	+2.86197	+5.61189	−0.58904
H	+0.74652	+5.46225	−1.86182

27

∗ E = +3.986 kcal/mol ; (23) 187_335_061_295_086

H	+0.00000	+0.00000	+0.00000
O	+0.00000	+0.00000	+0.96416
C	+1.26986	+0.00000	+1.37638
C	+1.41508	−0.17415	+2.87510
N	+0.22737	+0.26776	+3.57507
H	−0.57724	−0.27775	+3.28465
O	+2.20268	+0.05795	+0.62296
C	+1.82619	−1.65263	+3.13114
H	+2.25784	+0.46081	+3.16677
H	+0.34171	+0.15071	+4.57438
C	+0.77029	−2.63084	+2.73037
H	+2.75731	−1.85314	+2.58782
H	+2.04583	−1.74804	+4.20060
C	+0.56638	−3.19071	+1.42033
C	−0.57347	−4.01997	+1.49365
N	−1.03444	−3.96687	+2.78268
C	−0.22649	−3.13420	+3.51741
C	+1.24563	−3.07046	+0.20036
C	−1.05450	−4.72268	+0.38882
H	−1.82796	−4.46743	+3.13855

H	−0.41497	−2.97083	+4.56987
C	+0.77164	−3.76412	−0.89396
H	+2.12568	−2.43788	+0.11767
H	+1.28705	−3.68326	−1.84548
C	−0.36902	−4.58132	−0.79993
H	−1.93279	−5.35698	+0.45976
H	−0.71495	−5.11376	−1.68012

27

∗ E = +4.151 kcal/mol ; (24) 172_141_044_060_274

H	+0.00000	+0.00000	+0.00000
O	+0.00000	+0.00000	+0.96474
C	+1.26556	+0.00000	+1.38111
C	+1.40382	+0.18565	+2.87823
N	+2.48315	−0.67239	+3.32627
H	+3.26741	−0.59148	+2.68737
O	+2.20582	−0.08479	+0.63870
C	+1.64680	+1.69434	+3.14875
H	+0.46137	−0.10971	+3.35216
H	+2.79647	−0.41012	+4.25224
C	+0.54571	+2.59138	+2.68419
H	+1.80119	+1.80717	+4.22814
H	+2.59132	+1.96711	+2.65931
C	+0.38979	+3.13905	+1.36375
C	−0.81186	+3.87962	+1.35769
N	−1.35240	+3.78691	+2.61291
C	−0.53547	+3.01500	+3.40169
C	+1.15431	+3.06738	+0.19187
C	−1.26886	+4.54436	+0.21982
H	−2.20617	+4.22022	+2.91316
H	−0.78860	+2.82561	+4.43582
C	+0.70347	+3.72214	−0.93594
H	+2.08216	+2.50113	+0.17023
H	+1.28417	+3.67766	−1.85158
C	−0.49774	+4.45348	−0.92084
H	−2.19467	+5.11142	+0.22860
H	−0.82353	+4.95806	−1.82479

27

∗ E = +4.202 kcal/mol ; (25) 180_170_044_301_274

H	+0.00000	+0.00000	+0.00000
O	+0.00000	+0.00000	+0.96351

C	+1.28379	+0.00000	+1.36843
C	+1.39475	+0.01012	+2.87833
N	+2.77714	−0.21701	+3.23170
H	+3.38771	+0.34048	+2.64371
O	+2.20060	+0.03118	+0.60155
C	+0.77469	+1.33450	+3.40020
H	+0.77692	−0.81753	+3.25201
H	+2.95176	+0.02613	+4.19767
C	+1.44919	+2.54375	+2.83739
H	−0.29311	+1.34370	+3.15798
H	+0.85607	+1.32860	+4.49402
C	+2.61923	+3.20426	+3.34727
C	+2.93103	+4.25162	+2.45243
N	+1.99472	+4.22456	+1.45373
C	+1.11131	+3.20165	+1.68874
C	+3.42035	+3.02167	+4.48175
C	+4.01830	+5.10213	+2.65278
H	+1.96399	+4.84704	+0.66724
H	+0.28825	+3.01208	+1.01286
C	+4.49469	+3.86169	+4.68542
H	+3.19402	+2.23479	+5.19686
H	+5.12265	+3.73139	+5.56063
C	+4.79231	+4.89122	+3.77491
H	+4.24803	+5.90061	+1.95397
H	+5.64687	+5.53361	+3.96098

27

∗ E = +4.311 kcal/mol ; (26) 182_354_060_299_273

H	+0.00000	+0.00000	+0.00000
O	+0.00000	+0.00000	+0.96391
C	+1.27277	+0.00000	+1.36468
C	+1.45066	−0.05963	+2.87263
N	+0.18257	+0.03013	+3.56561
H	−0.42872	−0.73644	+3.30420
O	+2.19473	+0.00113	+0.59428
C	+2.29339	−1.32572	+3.16831
H	+2.05383	+0.81885	+3.13403
H	+0.32435	−0.01475	+4.56681
C	+1.60417	−2.57431	+2.71824
H	+3.25970	−1.22296	+2.66499
H	+2.48398	−1.36180	+4.24756

C	+0.65426	−3.36100	+3.45629
C	+0.23114	−4.40919	+2.60889
N	+0.89128	−4.25946	+1.41848
C	+1.71142	−3.16102	+1.48838
C	+0.13309	−3.28558	+4.75452
C	−0.69541	−5.36780	+3.01892
H	+0.80043	−4.86342	+0.62214
H	+2.32417	−2.86450	+0.64746
C	−0.78231	−4.23223	+5.16423
H	+0.45016	−2.49448	+5.42943
H	−1.19338	−4.18637	+6.16738
C	−1.19387	−5.26333	+4.30114
H	−1.01303	−6.16731	+2.35661
H	−1.91701	−5.99227	+4.65230

27

∗ E = +4.363 kcal/mol ; (27) 179_296_299_058_188

H	+0.00000	+0.00000	+0.00000
O	+0.00000	+0.00000	+0.96351
C	+1.27553	+0.00000	+1.38248
C	+1.36874	+0.02248	+2.90322
N	+0.81563	+1.24808	+3.46191
H	+1.31258	+2.06593	+3.13138
O	+2.20516	+0.02264	+0.62463
C	+0.65145	−1.17953	+3.51162
H	+2.43673	−0.01876	+3.14195
H	−0.15893	+1.34713	+3.19953
C	+1.17554	−2.50021	+3.04598
H	+0.73266	−1.08731	+4.60125
H	−0.41854	−1.10103	+3.27269
C	+0.58174	−3.77774	+3.32729
C	+1.39594	−4.76084	+2.72536
N	+2.43094	−4.10791	+2.11111
C	+2.29483	−2.75391	+2.30433
C	−0.55945	−4.17388	+4.03342
C	+1.09798	−6.12061	+2.80726
H	+3.16509	−4.54266	+1.58313
H	+3.01284	−2.06596	+1.87828
C	−0.85743	−5.51851	+4.11782
H	−1.19874	−3.43380	+4.50729
H	−1.73880	−5.84297	+4.66125

C −0.03452 −6.48180 +3.50905
H +1.73115 −6.86835 +2.33956
H −0.29522 −7.53196 +3.59306
27
∗ E = +4.413 kcal/mol ; (28) 351_032_081_082_092
H +0.00000 +0.00000 +0.00000
O +0.00000 +0.00000 +0.97003
C +1.27237 +0.00000 +1.36396
C +2.27726 −0.21721 +0.22406
N +1.71732 +0.36091 −0.99682
H +1.86002 +1.36488 −1.02425
O +1.58748 +0.14190 +2.50889
C +3.68053 +0.26482 +0.62954
H +2.31489 −1.30541 +0.07505
H +2.15118 −0.03178 −1.82172
C +4.43764 −0.74013 +1.44004
H +4.24120 +0.47127 −0.29025
H +3.58928 +1.21178 +1.17301
C +5.33470 −1.72969 +0.90991
C +5.80446 −2.49485 +2.00097
N +5.21715 −1.98849 +3.12946
C +4.39591 −0.94298 +2.78945
C +5.78756 −2.03677 −0.37886
C +6.70264 −3.54822 +1.83664
H +5.34526 −2.33943 +4.06093
H +3.81048 −0.42302 +3.53374
C +6.67570 −3.07972 −0.54602
H +5.44865 −1.46091 −1.23646
H +7.03501 −3.32829 −1.53933
C +7.12802 −3.82892 +0.55359
H +7.05637 −4.12723 +2.68427
H +7.82669 −4.64309 +0.39057
27
∗ E = +4.669 kcal/mol ; (29) 183_322_058_175_289
H +0.00000 +0.00000 +0.00000
O +0.00000 +0.00000 +0.96362
C +1.27892 +0.00000 +1.37615
C +1.39395 −0.06270 +2.88213
N +0.36193 +0.73493 +3.51429
H −0.55802 +0.42472 +3.22148

O	+2.20278	−0.02318	+0.61479
C	+1.39342	−1.56356	+3.28109
H	+2.37166	+0.36112	+3.13350
H	+0.42186	+0.63082	+4.52106
C	+1.62391	−1.73007	+4.74445
H	+0.42861	−1.99870	+2.99231
H	+2.17416	−2.08150	+2.70902
C	+2.86311	−1.49540	+5.43478
C	+2.62686	−1.72330	+6.80740
N	+1.30897	−2.07513	+6.94017
C	+0.71332	−2.07471	+5.70186
C	+4.14612	−1.12549	+5.01279
C	+3.63228	−1.58838	+7.76457
H	+0.85271	−2.31018	+7.80248
H	−0.33022	−2.33549	+5.59031
C	+5.14265	−0.99059	+5.95672
H	+4.35275	−0.95285	+3.95945
H	+6.14258	−0.70447	+5.64788
C	+4.88536	−1.21906	+7.32011
H	+3.43977	−1.76696	+8.81796
H	+5.69100	−1.10381	+8.03800

27

∗ E = +4.746 kcal/mol ; (30) 177_292_299_056_074

H	+0.00000	+0.00000	+0.00000
O	+0.00000	+0.00000	+0.96344
C	+1.28642	+0.00000	+1.37153
C	+1.39420	+0.07316	+2.88863
N	+0.95287	+1.35944	+3.40711
H	+1.51605	+2.12052	+3.04836
O	+2.20178	−0.00812	+0.60069
C	+0.57854	−1.05235	+3.53469
H	+2.45631	−0.05825	+3.11928
H	−0.01215	+1.53412	+3.14910
C	+0.99324	−2.39918	+3.04092
H	+0.71099	−0.97007	+4.61917
H	−0.48322	−0.87974	+3.32360
C	+2.19634	−3.09821	+3.39812
C	+2.21362	−4.30296	+2.66335
N	+1.07727	−4.32758	+1.89786
C	+0.35274	−3.18308	+2.12576

C	+3.25031	−2.81626	+4.27492
C	+3.25141	−5.22708	+2.78091
H	+0.81640	−5.06229	+1.26634
H	−0.58289	−3.00551	+1.61336
C	+4.27907	−3.72720	+4.39341
H	+3.25490	−1.89548	+4.85317
H	+5.10462	−3.52465	+5.06776
C	+4.27785	−4.92139	+3.65124
H	+3.25479	−6.15125	+2.21122
H	+5.10247	−5.61758	+3.76556

27

∗ E = +4.761 kcal/mol ; (31) 008_328_162_072_172

H	+0.00000	+0.00000	+0.00000
O	+0.00000	+0.00000	+0.97062
C	+1.27132	+0.00000	+1.36760
C	+2.27775	+0.20713	+0.22850
N	+1.72125	−0.39105	−0.98404
H	+2.18709	−0.04204	−1.81186
O	+1.58529	−0.13142	+2.51407
C	+2.48296	+1.70504	−0.03274
H	+3.22630	−0.23906	+0.55453
H	+1.80624	−1.40034	−0.98072
C	+3.25095	+2.45710	+1.00604
H	+3.01685	+1.79338	−0.99081
H	+1.49946	+2.16876	−0.19809
C	+3.68697	+3.81754	+0.85353
C	+4.39393	+4.16334	+2.02510
N	+4.38418	+3.06342	+2.84025
C	+3.69693	+2.04371	+2.22882
C	+3.54629	+4.76657	−0.16490
C	+4.95728	+5.42590	+2.20384
H	+4.78625	+3.01287	+3.75825
H	+3.54826	+1.09693	+2.72829
C	+4.10323	+6.01759	+0.00776
H	+3.00375	+4.52430	−1.07515
H	+4.00153	+6.76575	−0.77156
C	+4.80187	+6.34254	+1.18299
H	+5.49803	+5.68133	+3.10996
H	+5.22740	+7.33511	+1.29015

27

∗ E = +4.805 kcal/mol ; (32) 177_133_050_058_071

H	+0.00000	+0.00000	+0.00000
O	+0.00000	+0.00000	+0.96432
C	+1.26758	+0.00000	+1.37791
C	+1.41076	+0.08024	+2.88484
N	+2.37257	−0.93105	+3.28521
H	+3.20817	−0.84523	+2.71539
O	+2.20540	−0.02242	+0.62867
C	+1.83202	+1.53001	+3.22384
H	+0.43565	−0.13351	+3.33574
H	+2.63761	−0.81149	+4.25498
C	+0.85368	+2.54807	+2.74046
H	+1.95445	+1.59488	+4.31291
H	+2.81630	+1.70136	+2.77190
C	−0.46050	+2.78694	+3.26893
C	−1.05815	+3.78328	+2.46718
N	−0.15066	+4.12325	+1.49850
C	+0.99174	+3.37876	+1.66655
C	−1.17835	+2.25642	+4.34702
C	−2.34522	+4.25984	+2.71453
H	−0.28935	+4.81810	+0.78798
H	+1.83422	+3.49513	+0.99847
C	−2.45189	+2.72437	+4.59493
H	−0.73766	+1.48965	+4.97953
H	−3.02203	+2.32381	+5.42665
C	−3.02867	+3.71749	+3.78387
H	−2.79457	+5.02757	+2.09211
H	−4.03314	+4.06452	+4.00380

27

∗ E = +4.806 kcal/mol ; (33) 183_299_298_050_260

H	+0.00000	+0.00000	+0.00000
O	+0.00000	+0.00000	+0.96380
C	+1.28016	+0.00000	+1.38071
C	+1.36686	−0.06433	+2.89982
N	+0.72810	+1.06308	+3.55940
H	+1.16918	+1.93996	+3.31115
O	+2.20675	+0.06174	+0.62562
C	+0.73899	−1.37438	+3.39792
H	+2.43517	−0.05882	+3.13673
H	−0.24873	+1.12011	+3.29303

C	+1.26117	−2.57557	+2.67306
H	+0.91834	−1.43543	+4.47552
H	−0.34752	−1.30698	+3.25973
C	+0.61143	−3.27693	+1.59959
C	+1.49070	−4.29632	+1.17344
N	+2.61402	−4.21354	+1.95123
C	+2.47290	−3.18199	+2.84481
C	−0.63443	−3.14753	+0.97166
C	+1.16512	−5.17631	+0.14098
H	+3.41966	−4.80712	+1.87585
H	+3.26104	−2.95391	+3.54910
C	−0.96028	−4.01645	−0.04839
H	−1.32866	−2.37282	+1.28514
H	−1.92256	−3.93060	−0.54274
C	−0.06585	−5.02081	−0.46119
H	+1.85238	−5.95414	−0.17737
H	−0.35156	−5.68756	−1.26838

27

* E = +4.905 kcal/mol ; (34) 182_062_180_074_081

H	+0.00000	+0.00000	+0.00000
O	+0.00000	+0.00000	+0.96368
C	+1.27808	+0.00000	+1.38037
C	+1.35387	−0.05885	+2.89700
N	+0.73092	−1.31645	+3.30133
H	+0.74927	−1.40758	+4.30947
O	+2.20297	+0.02529	+0.62166
C	+2.80880	−0.00044	+3.35904
H	+0.82253	+0.83385	+3.27239
H	−0.23603	−1.35325	+3.00190
C	+3.39255	+1.36924	+3.22902
H	+2.84141	−0.31422	+4.41059
H	+3.38576	−0.73270	+2.78698
C	+3.21273	+2.45153	+4.15648
C	+3.90255	+3.56872	+3.63580
N	+4.46721	+3.17692	+2.45076
C	+4.15423	+1.86144	+2.20997
C	+2.53516	+2.57657	+5.37489
C	+3.93247	+4.79524	+4.29796
H	+5.01440	+3.76018	+1.84480
H	+4.48189	+1.36338	+1.30841

C	+2.56055	+3.78950	+6.03246
H	+1.99851	+1.73011	+5.79678
H	+2.04009	+3.90236	+6.97793
C	+3.25409	+4.88844	+5.49685
H	+4.46807	+5.64664	+3.88913
H	+3.25641	+5.82945	+6.03740

27

∗ E = +4.932 kcal/mol ; (35) 183_321_058_177_088

H	+0.00000	+0.00000	+0.00000
O	+0.00000	+0.00000	+0.96363
C	+1.27835	+0.00000	+1.37748
C	+1.38981	−0.06161	+2.88510
N	+0.36968	+0.75914	+3.50835
H	−0.55532	+0.46701	+3.21391
O	+2.20322	−0.02326	+0.61717
C	+1.37224	−1.56098	+3.28422
H	+2.37414	+0.34571	+3.13657
H	+0.41821	+0.66877	+4.51694
C	+1.53948	−1.74993	+4.75679
H	+0.41700	−1.99371	+2.95743
H	+2.16787	−2.08025	+2.73788
C	+0.49644	−1.76056	+5.74652
C	+1.11426	−1.92913	+7.00525
N	+2.46301	−2.01623	+6.78559
C	+2.71187	−1.90974	+5.43984
C	−0.89950	−1.65553	+5.68267
C	+0.38121	−1.98641	+8.19081
H	+3.16155	−2.14613	+7.49443
H	+3.72298	−1.96498	+5.06051
C	−1.62760	−1.71420	+6.85214
H	−1.39847	−1.53396	+4.72459
H	−2.70924	−1.63559	+6.81837
C	−0.99050	−1.87582	+8.09558
H	+0.86890	−2.11431	+9.15229
H	−1.59141	−1.91580	+8.99829

27

∗ E = +4.973 kcal/mol ; (36) 178_167_282_164_080

H	+0.00000	+0.00000	+0.00000
O	+0.00000	+0.00000	+0.96398
C	+1.27644	+0.00000	+1.37984

C	+1.39116	+0.05337	+2.89678
N	+2.72555	−0.25591	+3.36075
H	+2.89610	−1.25331	+3.34691
O	+2.20406	−0.01115	+0.62075
C	+0.95778	+1.45899	+3.37128
H	+0.68023	−0.67109	+3.30943
H	+3.40358	+0.16133	+2.73018
C	+0.65815	+1.52552	+4.83440
H	+1.76372	+2.15929	+3.11334
H	+0.07049	+1.76684	+2.80776
C	+1.58115	+1.62589	+5.93366
C	+0.81255	+1.67835	+7.11705
N	−0.50637	+1.60573	+6.75262
C	−0.59159	+1.52114	+5.38556
C	+2.97678	+1.69466	+6.03002
C	+1.39321	+1.79336	+8.38066
H	−1.28711	+1.63282	+7.38214
H	−1.55370	+1.47413	+4.89377
C	+3.55447	+1.81726	+7.27585
H	+3.58606	+1.62941	+5.13491
H	+4.63466	+1.87131	+7.36483
C	+2.76898	+1.86362	+8.44164
H	+0.78673	+1.82927	+9.28055
H	+3.25529	+1.95455	+9.40773

27

∗ E = +5.032 kcal/mol ; (37) 181_119_295_064_191

H	+0.00000	+0.00000	+0.00000
O	+0.00000	+0.00000	+0.96469
C	+1.27898	+0.00000	+1.37493
C	+1.43417	−0.02199	+2.89284
N	+2.15006	−1.21397	+3.32404
H	+1.62461	−2.05554	+3.12082
O	+2.20253	−0.02646	+0.61211
C	+2.21492	+1.21431	+3.33211
H	+0.43969	−0.02441	+3.35187
H	+3.03176	−1.27741	+2.82475
C	+1.52257	+2.50964	+3.05225
H	+2.42433	+1.10400	+4.40248
H	+3.18583	+1.19560	+2.81697
C	+2.12206	+3.81164	+3.15110

C	+1.12547	+4.75898	+2.83449
N	−0.02052	+4.06297	+2.55635
C	+0.22245	+2.71579	+2.68803
C	+3.40777	+4.25569	+3.47975
C	+1.37962	+6.13019	+2.83547
H	−0.89832	+4.46761	+2.28766
H	−0.56465	+1.99867	+2.49963
C	+3.66334	+5.61139	+3.48320
H	+4.19015	+3.54335	+3.72706
H	+4.65500	+5.97295	+3.73463
C	+2.65644	+6.53885	+3.16363
H	+0.60521	+6.85022	+2.58933
H	+2.88860	+7.59896	+3.17483

27

∗ E = +5.144 kcal/mol ; (38) 007_159_296_059_063

H	+0.00000	+0.00000	+0.00000
O	+0.00000	+0.00000	+0.96446
C	+1.25016	+0.00000	+1.44652
C	+2.37770	+0.17455	+0.42031
N	+3.60695	+0.65038	+1.01539
H	+3.48059	+1.58153	+1.39445
O	+1.45709	−0.12854	+2.61695
C	+2.64962	−1.16852	−0.29123
H	+2.04287	+0.90430	−0.32961
H	+3.83772	+0.05863	+1.80811
C	+1.45585	−1.72225	−0.99617
H	+3.47563	−0.98867	−0.98827
H	+3.01253	−1.88346	+0.45713
C	+0.76537	−1.11229	−2.10220
C	−0.35605	−1.92300	−2.39172
N	−0.33695	−2.96989	−1.51002
C	+0.74325	−2.84048	−0.67349
C	+0.99585	+0.03505	−2.87581
C	−1.24751	−1.61533	−3.41959
H	−1.01769	−3.70632	−1.46489
H	+0.92295	−3.56009	+0.11307
C	+0.11391	+0.34196	−3.89073
H	+1.86383	+0.66241	−2.68913
H	+0.27925	+1.22459	−4.49954
C	−0.99837	−0.47632	−4.15700

H	−2.10446	−2.24648	−3.63323
H	−1.67387	−0.20638	−4.96216

27

∗ E = +5.157 kcal/mol ; (39) 179_048_311_168_202

H	+0.00000	+0.00000	+0.00000
O	+0.00000	+0.00000	+0.96359
C	+1.28049	+0.00000	+1.37843
C	+1.37219	+0.01573	+2.90185
N	+0.49879	−0.96911	+3.53030
H	−0.44088	−0.90940	+3.15691
O	+2.20088	−0.00558	+0.61273
C	+2.81897	−0.16444	+3.34014
H	+1.02776	+1.01200	+3.21675
H	+0.84293	−1.90661	+3.35316
C	+3.06207	+0.12809	+4.78725
H	+3.13535	−1.18753	+3.08619
H	+3.44692	+0.48934	+2.72547
C	+4.33826	+0.47834	+5.34782
C	+4.14638	+0.68890	+6.73067
N	+2.81919	+0.47594	+6.98995
C	+2.17334	+0.13576	+5.82461
C	+5.61877	+0.63797	+4.80536
C	+5.19344	+1.04962	+7.57803
H	+2.38345	+0.54249	+7.89118
H	+1.11842	−0.09635	+5.81361
C	+6.65767	+0.99460	+5.64058
H	+5.79141	+0.48109	+3.74396
H	+7.65624	+1.12127	+5.23522
C	+6.44515	+1.19766	+7.01513
H	+5.03310	+1.20897	+8.64004
H	+7.28258	+1.47731	+7.64633

27

∗ E = +5.183 kcal/mol ; (40) 358_355_296_297_272

H	+0.00000	+0.00000	+0.00000
O	+0.00000	+0.00000	+0.96327
C	+1.26429	+0.00000	+1.39472
C	+2.35962	−0.05321	+0.31440
N	+1.92153	−0.20844	−1.05295
H	+1.44261	−1.06732	−1.27514
O	+1.52569	+0.05882	+2.56053

C	+3.18469	+1.23880	+0.45925
H	+2.99541	−0.90569	+0.58006
H	+1.48391	+0.60688	−1.46179
C	+2.34822	+2.45162	+0.19078
H	+3.58461	+1.27162	+1.47578
H	+4.02561	+1.18744	−0.23962
C	+2.11142	+3.08424	−1.07866
C	+1.20686	+4.14635	−0.85259
N	+0.91322	+4.15046	+0.48483
C	+1.59521	+3.13465	+1.10471
C	+2.58274	+2.85409	−2.37889
C	+0.76392	+4.97477	−1.88354
H	+0.29272	+4.79231	+0.94380
H	+1.49265	+2.95728	+2.16717
C	+2.14450	+3.67171	−3.40001
H	+3.27802	+2.04130	−2.57429
H	+2.50076	+3.50748	−4.41172
C	+1.24315	+4.72234	−3.15277
H	+0.06956	+5.78824	−1.69703
H	+0.91847	+5.34786	−3.97796

27

∗ E = +5.392 kcal/mol ; (41) 177_005_315_292_194

H	+0.00000	+0.00000	+0.00000
O	+0.00000	+0.00000	+0.96432
C	+1.28659	+0.00000	+1.36274
C	+1.45963	+0.08098	+2.88107
N	+0.25722	+0.03812	+3.68302
H	−0.46780	+0.63579	+3.30894
O	+2.19539	−0.02007	+0.58358
C	+2.46248	−0.98337	+3.33429
H	+1.94044	+1.05774	+3.03108
H	−0.10868	−0.90440	+3.73943
C	+1.97739	−2.39431	+3.19070
H	+3.38690	−0.85010	+2.76069
H	+2.69981	−0.78656	+4.38591
C	+2.60142	−3.53923	+3.79460
C	+1.87447	−4.67913	+3.39145
N	+0.85858	−4.24568	+2.58308
C	+0.92361	−2.87653	+2.46617
C	+3.70883	−3.69679	+4.63582

C	+2.22069	−5.96460	+3.80756
H	+0.16828	−4.82993	+2.14869
H	+0.19599	−2.34917	+1.86280
C	+4.05525	−4.96620	+5.04939
H	+4.28532	−2.83307	+4.95551
H	+4.91073	−5.10658	+5.70185
C	+3.31597	−6.08908	+4.63761
H	+1.65342	−6.83466	+3.49159
H	+3.61414	−7.07492	+4.97975

27

∗ E = +5.488 kcal/mol ; (42) 179_301_067_063_182

H	+0.00000	+0.00000	+0.00000
O	+0.00000	+0.00000	+0.96357
C	+1.27518	+0.00000	+1.38262
C	+1.35232	+0.01913	+2.89962
N	+0.66176	+1.22188	+3.34910
H	−0.32860	+1.15611	+3.14095
O	+2.20558	−0.01141	+0.62916
C	+0.79511	−1.28336	+3.48976
H	+2.41469	+0.11530	+3.14583
H	+0.76340	+1.33550	+4.34992
C	+1.55094	−2.51792	+3.11324
H	+0.78828	−1.16872	+4.58279
H	−0.25719	−1.37557	+3.18575
C	+1.19718	−3.85626	+3.49808
C	+2.17542	−4.71979	+2.96030
N	+3.07547	−3.93941	+2.28509
C	+2.69791	−2.62144	+2.37766
C	+0.14659	−4.39622	+4.24791
C	+2.12803	−6.10050	+3.14868
H	+3.87542	−4.27359	+1.77988
H	+3.27610	−1.84965	+1.88791
C	+0.09623	−5.76220	+4.43748
H	−0.61726	−3.74968	+4.67188
H	−0.71236	−6.19758	+5.01543
C	+1.07945	−6.60516	+3.89148
H	+2.88563	−6.75559	+2.72962
H	+1.01246	−7.67556	+4.05744

27

∗ E = +5.556 kcal/mol ; (43) 180_047_176_172_164

H	+0.00000	+0.00000	+0.00000
O	+0.00000	+0.00000	+0.96354
C	+1.27807	+0.00000	+1.37787
C	+1.36783	+0.00314	+2.89322
N	+0.46445	−1.01050	+3.41863
H	+0.55841	−1.07742	+4.42432
O	+2.20248	+0.02927	+0.61890
C	+2.79655	−0.26721	+3.34137
H	+1.09198	+1.03378	+3.19208
H	−0.49982	−0.81016	+3.18736
C	+2.98959	−0.08111	+4.81445
H	+3.05347	−1.29531	+3.05771
H	+3.47376	+0.38550	+2.77903
C	+4.11294	−0.55526	+5.57446
C	+3.92538	−0.14076	+6.91006
N	+2.74416	+0.54991	+6.95785
C	+2.18751	+0.58160	+5.69979
C	+5.25698	−1.28929	+5.23995
C	+4.84423	−0.44181	+7.91559
H	+2.34030	+0.95936	+7.77990
H	+1.24626	+1.08914	+5.53444
C	+6.16861	−1.58791	+6.23083
H	+5.42166	−1.61558	+4.21679
H	+7.06097	−2.15602	+5.98956
C	+5.96187	−1.16694	+7.55646
H	+4.68952	−0.11885	+8.94050
H	+6.69806	−1.41793	+8.31338

27

∗ E = +5.597 kcal/mol ; (44) 180_010_200_303_105

H	+0.00000	+0.00000	+0.00000
O	+0.00000	+0.00000	+0.96405
C	+1.27712	+0.00000	+1.36825
C	+1.44912	−0.00101	+2.87609
N	+0.19858	−0.22808	+3.58044
H	+0.26583	+0.09996	+4.53493
O	+2.19864	+0.02945	+0.60343
C	+2.47745	−1.08901	+3.23653
H	+1.89920	+0.98135	+3.09928
H	−0.57686	+0.24424	+3.13465
C	+2.08422	−2.44274	+2.73320

H	+3.44514	−0.80604	+2.81099
H	+2.59111	−1.09010	+4.32661
C	+2.65661	−3.13388	+1.60917
C	+1.97659	−4.36521	+1.48560
N	+1.03638	−4.40994	+2.48142
C	+1.10106	−3.25605	+3.22135
C	+3.67063	−2.82216	+0.69520
C	+2.28830	−5.29117	+0.49004
H	+0.39219	−5.16257	+2.64037
H	+0.42453	−3.08510	+4.04592
C	+3.98261	−3.73579	−0.28980
H	+4.18361	−1.86630	+0.75063
H	+4.76390	−3.50700	−1.00733
C	+3.29860	−4.96048	−0.38975
H	+1.75743	−6.23485	+0.40820
H	+3.56759	−5.65706	−1.17746

27

∗ E = +5.747 kcal/mol ; (45) 183_339_293_162_285

H	+0.00000	+0.00000	+0.00000
O	+0.00000	+0.00000	+0.96400
C	+1.28119	+0.00000	+1.36982
C	+1.43472	−0.06805	+2.88326
N	+0.28554	+0.33564	+3.66290
H	+0.09611	+1.32459	+3.56412
O	+2.19940	+0.01056	+0.59936
C	+1.84314	−1.51405	+3.24640
H	+2.28043	+0.58599	+3.12565
H	−0.54330	−0.16211	+3.35871
C	+2.43128	−1.59290	+4.61321
H	+0.95618	−2.15488	+3.16619
H	+2.56913	−1.86677	+2.50400
C	+3.74952	−1.15058	+4.97272
C	+3.89397	−1.35227	+6.36223
N	+2.71817	−1.88935	+6.81707
C	+1.84520	−2.02635	+5.76463
C	+4.81362	−0.60648	+4.24391
C	+5.07029	−1.02976	+7.03766
H	+2.52144	−2.14022	+7.76831
H	+0.85057	−2.42003	+5.91973
C	+5.97829	−0.28356	+4.91009

H +4.72271 −0.44484 +3.17209
H +6.81395 +0.13878 +4.36172
C +6.10328 −0.49424 +6.29434
H +5.17293 −1.19164 +8.10636
H +7.03240 −0.23027 +6.78926
27
∗ E = +5.754 kcal/mol ; (46) 010_328_159_059_273
H +0.00000 +0.00000 +0.00000
O +0.00000 +0.00000 +0.97032
C +1.27945 +0.00000 +1.36430
C +2.27238 +0.24405 +0.22584
N +1.73076 −0.31671 −1.00920
H +2.16213 +0.10060 −1.82439
O +1.60351 −0.13210 +2.50421
C +2.45030 +1.76220 +0.02367
H +3.22864 −0.18938 +0.54280
H +1.86411 −1.31903 −1.06305
C +2.94946 +2.50655 +1.21759
H +3.14264 +1.89801 −0.81707
H +1.48292 +2.17362 −0.29702
C +2.15163 +3.05519 +2.27897
C +3.03542 +3.67980 +3.18262
N +4.30473 +3.51359 +2.69144
C +4.24641 +2.81318 +1.51375
C +0.77710 +3.07245 +2.54350
C +2.58617 +4.32485 +4.33471
H +5.14502 +3.85108 +3.12354
H +5.14849 +2.58827 +0.96137
C +0.32863 +3.71142 +3.67941
H +0.07703 +2.57530 +1.87622
H −0.73285 +3.72958 +3.90305
C +1.22633 +4.33239 +4.56599
H +3.27690 +4.80066 +5.02403
H +0.84087 +4.82303 +5.45400
27
∗ E = +5.801 kcal/mol ; (47) 178_008_183_301_158
H +0.00000 +0.00000 +0.00000
O +0.00000 +0.00000 +0.96412
C +1.28126 +0.00000 +1.36236
C +1.45414 +0.03973 +2.87280

N	+0.18941	−0.12746	+3.56975
H	+0.32678	−0.04207	+4.56882
O	+2.19793	+0.01207	+0.59281
C	+2.45681	−1.04368	+3.27987
H	+1.92981	+1.01770	+3.06230
H	−0.49143	+0.56320	+3.28020
C	+2.06168	−2.43319	+2.88322
H	+3.41634	−0.80077	+2.80986
H	+2.61413	−0.97458	+4.36545
C	+2.97476	−3.53227	+2.73291
C	+2.21866	−4.65408	+2.32914
N	+0.91450	−4.24854	+2.23689
C	+0.82213	−2.91914	+2.57464
C	+4.35805	−3.66686	+2.89746
C	+2.80446	−5.89699	+2.09219
H	+0.14007	−4.83077	+1.97670
H	−0.12824	−2.40524	+2.58331
C	+4.94186	−4.89532	+2.66500
H	+4.96245	−2.81705	+3.20291
H	+6.01325	−5.01530	+2.78845
C	+4.16991	−6.00040	+2.26689
H	+2.21198	−6.75217	+1.78142
H	+4.65805	−6.95369	+2.09123

27

∗ E = +5.827 kcal/mol ; (48) 181_120_056_067_187

H	+0.00000	+0.00000	+0.00000
O	+0.00000	+0.00000	+0.96465
C	+1.27499	+0.00000	+1.37314
C	+1.43856	−0.01542	+2.88454
N	+2.18323	−1.23188	+3.18365
H	+3.05701	−1.22691	+2.66711
O	+2.20254	−0.00592	+0.61521
C	+2.13970	+1.27316	+3.33173
H	+0.44748	−0.08698	+3.34567
H	+2.40260	−1.28071	+4.17091
C	+1.33782	+2.52121	+3.14081
H	+2.40592	+1.15695	+4.39148
H	+3.08699	+1.34525	+2.77926
C	+1.82384	+3.85893	+3.33850
C	+0.75091	+4.73856	+3.08189

N	−0.33071	+3.96930	+2.74486
C	+0.02541	+2.64175	+2.78137
C	+3.06607	+4.38544	+3.70953
C	+0.88714	+6.12261	+3.18413
H	−1.23897	+4.31603	+2.49701
H	−0.69661	+1.87619	+2.53343
C	+3.20515	+5.75417	+3.81300
H	+3.90634	+3.72612	+3.90995
H	+4.16179	+6.17949	+4.09808
C	+2.12359	+6.61340	+3.55223
H	+0.05489	+6.79032	+2.98354
H	+2.26449	+7.68577	+3.64153

27

∗ E = +5.935 kcal/mol ; (49) 183_203_202_084_069

H	+0.00000	+0.00000	+0.00000
O	+0.00000	+0.00000	+0.96417
C	+1.27827	+0.00000	+1.36255
C	+1.44511	−0.06121	+2.86954
N	+2.75080	+0.48973	+3.18056
H	+3.08017	+0.16621	+4.07984
O	+2.19256	+0.02036	+0.58665
C	+0.33899	+0.68993	+3.63900
H	+1.34967	−1.13656	+3.11274
H	+3.42371	+0.22955	+2.46919
C	−0.90225	−0.11136	+3.85606
H	+0.77166	+0.95984	+4.61058
H	+0.11715	+1.63364	+3.13083
C	−1.01798	−1.24775	+4.72764
C	−2.34622	−1.71758	+4.63185
N	−2.99951	−0.90174	+3.74625
C	−2.12821	+0.05556	+3.28494
C	−0.12326	−1.90277	+5.58194
C	−2.79689	−2.81769	+5.35997
H	−3.96145	−0.98834	+3.47466
H	−2.44651	+0.79076	+2.55918
C	−0.56476	−2.99286	+6.30371
H	+0.90241	−1.55412	+5.67900
H	+0.11650	−3.51114	+6.97056
C	−1.89073	−3.44547	+6.19125
H	−3.82086	−3.16958	+5.27929

H −2.20873 −4.30549 +6.77166
27
∗ E = +6.087 kcal/mol ; (50) 183_303_063_051_264
H +0.00000 +0.00000 +0.00000
O +0.00000 +0.00000 +0.96388
C +1.28133 +0.00000 +1.37774
C +1.36122 −0.06713 +2.89115
N +0.61092 +1.04862 +3.44637
H −0.36928 +0.97220 +3.19811
O +2.20726 +0.02298 +0.62344
C +0.88684 −1.46131 +3.36309
H +2.42026 +0.05611 +3.13656
H +0.67753 +1.05703 +4.45660
C +1.57301 −2.58364 +2.65178
H +1.04588 −1.51871 +4.44613
H −0.19587 −1.52413 +3.19589
C +1.06906 −3.29493 +1.50895
C +2.07175 −4.20390 +1.10702
N +3.12689 −4.04868 +1.96526
C +2.82490 −3.07648 +2.88548
C −0.13938 −3.25736 +0.80112
C +1.90396 −5.06313 +0.02112
H +3.99178 −4.55579 +1.92265
H +3.53898 −2.80400 +3.65036
C −0.30889 −4.10595 −0.27279
H −0.92769 −2.57018 +1.09633
H −1.24018 −4.09129 −0.82973
C +0.70627 −4.99917 −0.65996
H +2.68430 −5.75601 −0.27825
H +0.54243 −5.65266 −1.51075
27
∗ E = +6.148 kcal/mol ; (51) 005_156_030_062_063
H +0.00000 +0.00000 +0.00000
O +0.00000 +0.00000 +0.96410
C +1.24745 +0.00000 +1.44473
C +2.36498 +0.10931 +0.41072
N +3.53478 +0.65645 +1.05931
H +3.53471 +0.39039 +2.03836
O +1.45658 −0.10365 +2.61719
C +2.60182 −1.28370 −0.22938

H	+2.01997	+0.79378	−0.37933
H	+4.38930	+0.33002	+0.62823
C	+1.41288	−1.81434	−0.95970
H	+3.45432	−1.17830	−0.91394
H	+2.90803	−1.97635	+0.56298
C	+0.79271	−1.22876	−2.11942
C	−0.35001	−2.00586	−2.41696
N	−0.41118	−3.00902	−1.48727
C	+0.64023	−2.88566	−0.61485
C	+1.10183	−0.13107	−2.93634
C	−1.18640	−1.71294	−3.49434
H	−1.12470	−3.71337	−1.43602
H	+0.75858	−3.57429	+0.21013
C	+0.27564	+0.16061	−4.00113
H	+1.98387	+0.47341	−2.74057
H	+0.50195	+1.00575	−4.64262
C	−0.85920	−0.62361	−4.27470
H	−2.06093	−2.31794	−3.71240
H	−1.49009	−0.36616	−5.11917

27

∗ E = +6.165 kcal/mol ; (52) 178_299_066_057_073

H	+0.00000	+0.00000	+0.00000
O	+0.00000	+0.00000	+0.96343
C	+1.28510	+0.00000	+1.37460
C	+1.36644	+0.04134	+2.88912
N	+0.72176	+1.26529	+3.34258
H	−0.27193	+1.23481	+3.14248
O	+2.20464	−0.03914	+0.61394
C	+0.75959	−1.25616	+3.46507
H	+2.43337	+0.08894	+3.12819
H	+0.83612	+1.38091	+4.34173
C	+1.42258	−2.48237	+2.93185
H	+0.85162	−1.20995	+4.55801
H	−0.31120	−1.26692	+3.23007
C	+2.73522	−2.95469	+3.27364
C	+2.98513	−4.09934	+2.48681
N	+1.87831	−4.30447	+1.70584
C	+0.94764	−3.33106	+1.97436
C	+3.71293	−2.51815	+4.17493
C	+4.17927	−4.81387	+2.57727

H +1.76675 −5.04367 +1.03659
H −0.00244 −3.31381 +1.45797
C +4.89552 −3.22164 +4.26759
H +3.54192 −1.63927 +4.79193
H +5.66467 −2.89639 +4.96032
C +5.12477 −4.35929 +3.47379
H +4.36134 −5.69290 +1.96673
H +6.06648 −4.89037 +3.56784
27
* E = +6.408 kcal/mol ; (53) 180_290_170_060_187
H +0.00000 +0.00000 +0.00000
O +0.00000 +0.00000 +0.96344
C +1.26819 +0.00000 +1.37695
C +1.39620 +0.00885 +2.89542
N +0.95768 +1.31250 +3.37804
H +0.87719 +1.30464 +4.38731
O +2.20253 +0.00419 +0.62235
C +0.53105 −1.08140 +3.51957
H +2.45713 −0.20166 +3.09814
H +1.61465 +2.04047 +3.12633
C +0.89364 −2.46146 +3.07305
H +0.62450 −1.00383 +4.61238
H −0.51715 −0.86439 +3.27996
C +0.16578 −3.65839 +3.39013
C +0.84638 −4.73627 +2.78441
N +1.93149 −4.21443 +2.13261
C +1.95676 −2.85132 +2.30795
C −0.99545 −3.91139 +4.12885
C +0.39788 −6.05189 +2.89670
H +2.59880 −4.73794 +1.59651
H +2.73873 −2.25644 +1.85480
C −1.44209 −5.21182 +4.24325
H −1.53624 −3.09589 +4.60163
H −2.34146 −5.42569 +4.81140
C −0.74959 −6.27141 +3.63180
H +0.92898 −6.87345 +2.42585
H −1.12620 −7.28356 +3.73939
27
* E = +6.439 kcal/mol ; (54) 183_323_291_166_085
H +0.00000 +0.00000 +0.00000

O	+0.00000	+0.00000	+0.96371
C	+1.27977	+0.00000	+1.37844
C	+1.39470	−0.08143	+2.89403
N	+0.38194	+0.64409	+3.63223
H	+0.48570	+1.64512	+3.52728
O	+2.20344	+0.01239	+0.61524
C	+1.36311	−1.57703	+3.27428
H	+2.38157	+0.31672	+3.14795
H	−0.54379	+0.39558	+3.30176
C	+1.78641	−1.83910	+4.68331
H	+0.34142	−1.94600	+3.10777
H	+2.01546	−2.12539	+2.58527
C	+0.98093	−1.78184	+5.87244
C	+1.81651	−2.12198	+6.95817
N	+3.06586	−2.36527	+6.45079
C	+3.03947	−2.20059	+5.08853
C	−0.36603	−1.49196	+6.11997
C	+1.34800	−2.17687	+8.27125
H	+3.86915	−2.63908	+6.98593
H	+3.92887	−2.36436	+4.49540
C	−0.83620	−1.55231	+7.41441
H	−1.02426	−1.21250	+5.30316
H	−1.87820	−1.33010	+7.62039
C	+0.01562	−1.88971	+8.48169
H	+2.00450	−2.43766	+9.09578
H	−0.38375	−1.92353	+9.49028

27

∗ E = +6.709 kcal/mol ; (55) 183_066_179_072_255

H	+0.00000	+0.00000	+0.00000
O	+0.00000	+0.00000	+0.96381
C	+1.28004	+0.00000	+1.37843
C	+1.35712	−0.06807	+2.89390
N	+0.82865	−1.37634	+3.27294
H	+0.86064	−1.49099	+4.27838
O	+2.20455	+0.02113	+0.62190
C	+2.79876	+0.08835	+3.37510
H	+0.75736	+0.77338	+3.28066
H	−0.13490	−1.47592	+2.97682
C	+3.32968	+1.48242	+3.23057
H	+2.82875	−0.21556	+4.42888

H	+3.42621	−0.61881	+2.82334
C	+4.26469	+1.96931	+2.25237
C	+4.47582	+3.33867	+2.52201
N	+3.70211	+3.66545	+3.60455
C	+3.02296	+2.54989	+4.02681
C	+4.94791	+1.37389	+1.18407
C	+5.34326	+4.12421	+1.76180
H	+3.65157	+4.57156	+4.03228
H	+2.36901	+2.59927	+4.88712
C	+5.80974	+2.14502	+0.43429
H	+4.78020	+0.32892	+0.94386
H	+6.34431	+1.69841	−0.39777
C	+6.00501	+3.50876	+0.72033
H	+5.49353	+5.17682	+1.98162
H	+6.68858	+4.08786	+0.10753

27

∗ E = +6.756 kcal/mol ; (56) 011_160_299_060_269

H	+0.00000	+0.00000	+0.00000
O	+0.00000	+0.00000	+0.96359
C	+1.25260	+0.00000	+1.43761
C	+2.36609	+0.26670	+0.41630
N	+3.59883	+0.69597	+1.03993
H	+3.45604	+1.56498	+1.54149
O	+1.47082	−0.18927	+2.59784
C	+2.64691	−0.99751	−0.42169
H	+2.01995	+1.05633	−0.26492
H	+3.86495	+0.01479	+1.74488
C	+1.49096	−1.51329	−1.22063
H	+3.48827	−0.74933	−1.07521
H	+3.00092	−1.78193	+0.26086
C	+0.52373	−2.49267	−0.80002
C	−0.35689	−2.70387	−1.88071
N	+0.05432	−1.88939	−2.90484
C	+1.15990	−1.18134	−2.50636
C	+0.32431	−3.19283	+0.39651
C	−1.42329	−3.60085	−1.80404
H	−0.37379	−1.83137	−3.81089
H	+1.65321	−0.50031	−3.18644
C	−0.72573	−4.08112	+0.47491
H	+0.97532	−3.02431	+1.25127

H −0.89923 −4.62983 +1.39446
C −1.58945 −4.28349 −0.61741
H −2.09564 −3.75638 −2.64199
H −2.40826 −4.98914 −0.52110
27
∗ E = +6.782 kcal/mol ; (57) 357_178_306_301_271
H +0.00000 +0.00000 +0.00000
O +0.00000 +0.00000 +0.96001
C +1.26264 +0.00000 +1.44100
C +2.36987 −0.06739 +0.38185
N +3.69596 −0.01928 +0.94955
H +3.79014 +0.79130 +1.55068
O +1.46096 +0.04359 +2.61449
C +2.20782 −1.35424 −0.45502
H +2.23822 +0.79955 −0.28320
H +3.83187 −0.82816 +1.55026
C +2.27322 −2.58741 +0.39012
H +1.26529 −1.33411 −1.01560
H +3.01258 −1.35975 −1.19716
C +3.45420 −3.32671 +0.74273
C +3.04772 −4.37685 +1.59430
N +1.69072 −4.27196 +1.74927
C +1.23220 −3.19840 +1.03109
C +4.81120 −3.19273 +0.41977
C +3.95434 −5.29554 +2.12322
H +1.12094 −4.87107 +2.31835
H +0.18231 −2.93668 +1.03495
C +5.70905 −4.10028 +0.94114
H +5.14539 −2.37693 −0.21593
H +6.76392 −4.01094 +0.70320
C +5.28272 −5.14245 +1.78395
H +3.63009 −6.09875 +2.77761
H +6.01583 −5.83829 +2.17903
27
∗ E = +6.800 kcal/mol ; (58) 008_174_309_069_257
H +0.00000 +0.00000 +0.00000
O +0.00000 +0.00000 +0.96484
C +1.24689 +0.00000 +1.45172
C +2.37749 +0.18912 +0.43072
N +3.67345 +0.32018 +1.05958

H	+3.63369	+1.02598	+1.78654
O	+1.44697	−0.12237	+2.62381
C	+2.42692	−0.96677	−0.59047
H	+2.15975	+1.11416	−0.12279
H	+3.89966	−0.54442	+1.54172
C	+1.28296	−1.02596	−1.55771
H	+3.37176	−0.84676	−1.12963
H	+2.49831	−1.91691	−0.04523
C	+0.15904	−1.92543	−1.52463
C	−0.64334	−1.63501	−2.64844
N	−0.03620	−0.61030	−3.32656
C	+1.11292	−0.24934	−2.67257
C	−0.24163	−2.93650	−0.64060
C	−1.82462	−2.32784	−2.91737
H	−0.37635	−0.18913	−4.17207
H	+1.74767	+0.53358	−3.06483
C	−1.40576	−3.62376	−0.90455
H	+0.34969	−3.16512	+0.24195
H	−1.73242	−4.40791	−0.22986
C	−2.18854	−3.32194	−2.03438
H	−2.43338	−2.09480	−3.78534
H	−3.10099	−3.88202	−2.21120

27

* E = +6.934 kcal/mol ; (59) 181_285_171_055_252

H	+0.00000	+0.00000	+0.00000
O	+0.00000	+0.00000	+0.96368
C	+1.27118	+0.00000	+1.37798
C	+1.39406	−0.01400	+2.89572
N	+1.07894	+1.32315	+3.38250
H	+1.00183	+1.32279	+4.39223
O	+2.20413	+0.02885	+0.62566
C	+0.43230	−1.03138	+3.51627
H	+2.42950	−0.32780	+3.09574
H	+1.79786	+1.98857	+3.12661
C	+0.61407	−2.40868	+2.95676
H	+0.58909	−1.02542	+4.60232
H	−0.59121	−0.68509	+3.33928
C	−0.27578	−3.09378	+2.05992
C	+0.30798	−4.34488	+1.76558
N	+1.49238	−4.41067	+2.44966

C	+1.67182	−3.24984	+3.15897
C	−1.51096	−2.76667	+1.48574
C	−0.30292	−5.27033	+0.91847
H	+2.13650	−5.17984	+2.42708
H	+2.55482	−3.11044	+3.76758
C	−2.11859	−3.67932	+0.64993
H	−1.97355	−1.80573	+1.69250
H	−3.07626	−3.44187	+0.19808
C	−1.51819	−4.92041	+0.36859
H	+0.15840	−6.22853	+0.69987
H	−2.02207	−5.61576	−0.29505

27

∗ E = +7.018 kcal/mol ; (60) 179_353_069_289_198

H	+0.00000	+0.00000	+0.00000
O	+0.00000	+0.00000	+0.96438
C	+1.28187	+0.00000	+1.35792
C	+1.47940	+0.02505	+2.86706
N	+0.23547	+0.21631	+3.58673
H	−0.36194	−0.59816	+3.50488
O	+2.19300	−0.02362	+0.57966
C	+2.33288	−1.18945	+3.28273
H	+2.09765	+0.91628	+3.03089
H	+0.42565	+0.34785	+4.57247
C	+1.64306	−2.51485	+3.18263
H	+3.24642	−1.18978	+2.67459
H	+2.64868	−1.03210	+4.32228
C	+2.08212	−3.71476	+3.84103
C	+1.18878	−4.74599	+3.48157
N	+0.25382	−4.19709	+2.64660
C	+0.52848	−2.86077	+2.47104
C	+3.15005	−4.00453	+4.69834
C	+1.33132	−6.04976	+3.95626
H	−0.51628	−4.68696	+2.22989
H	−0.10691	−2.25666	+1.83729
C	+3.29483	−5.29246	+5.17047
H	+3.85489	−3.22815	+4.98329
H	+4.11741	−5.53408	+5.83554
C	+2.39198	−6.30503	+4.80124
H	+0.63727	−6.83503	+3.67287
H	+2.53351	−7.30898	+5.18839

27
∗ E = +7.465 kcal/mol ; (61) 021_210_203_311_130

H	+0.00000	+0.00000	+0.00000
O	+0.00000	+0.00000	+0.96282
C	+1.23779	+0.00000	+1.46639
C	+2.37996	+0.48509	+0.56647
N	+3.58122	−0.22578	+0.97309
H	+4.41236	+0.28724	+0.70924
O	+1.43218	−0.28970	+2.61012
C	+2.13128	+0.31367	−0.93643
H	+2.43576	+1.56872	+0.77888
H	+3.57734	−0.33964	+1.98118
C	+1.66710	−1.05984	−1.31681
H	+1.38803	+1.05059	−1.26966
H	+3.05786	+0.58479	−1.45723
C	+0.52963	−1.35194	−2.14557
C	+0.38977	−2.75785	−2.18096
N	+1.39713	−3.28103	−1.41722
C	+2.15583	−2.26547	−0.89359
C	−0.38863	−0.55816	−2.84674
C	−0.63208	−3.38315	−2.89393
H	+1.54409	−4.25782	−1.23792
H	+2.99386	−2.46259	−0.24142
C	−1.40412	−1.17478	−3.54812
H	−0.29904	+0.52547	−2.85070
H	−2.12227	−0.57368	−4.09593
C	−1.52222	−2.57514	−3.57168
H	−0.72796	−4.46432	−2.91430
H	−2.33100	−3.02976	−4.13436

27
∗ E = +7.556 kcal/mol ; (62) 350_296_302_286_101

H	+0.00000	+0.00000	+0.00000
O	+0.00000	+0.00000	+0.96492
C	+1.24733	+0.00000	+1.46156
C	+2.38227	−0.24152	+0.46159
N	+2.33314	−1.55953	−0.15510
H	+2.36319	−2.29278	+0.54249
O	+1.43418	+0.14401	+2.63344
C	+2.45657	+0.83939	−0.63514
H	+3.29481	−0.17941	+1.06035

H	+1.48780	−1.68037	−0.70181
C	+1.38132	+0.76504	−1.67954
H	+2.44567	+1.82925	−0.16495
H	+3.43278	+0.72248	−1.11541
C	+0.19084	+1.57286	−1.76740
C	−0.49811	+1.17789	−2.93440
N	+0.23843	+0.18421	−3.52375
C	+1.35958	−0.05879	−2.77385
C	−0.34988	+2.58720	−0.96430
C	−1.70308	+1.76608	−3.32107
H	−0.00049	−0.28969	−4.37608
H	+2.08736	−0.79791	−3.08042
C	−1.53791	+3.17142	−1.34530
H	+0.15296	+2.89658	−0.05186
H	−1.97190	+3.95548	−0.73405
C	−2.20712	+2.76369	−2.51395
H	−2.22327	+1.45303	−4.22089
H	−3.14227	+3.24363	−2.78345

27

* E = +7.559 kcal/mol ; (63) 008_156_029_063_268

H	+0.00000	+0.00000	+0.00000
O	+0.00000	+0.00000	+0.96314
C	+1.24864	+0.00000	+1.43743
C	+2.35943	+0.19211	+0.40746
N	+3.51391	+0.73317	+1.08614
H	+3.51413	+0.42963	+2.05408
O	+1.46598	−0.15960	+2.60246
C	+2.63871	−1.15265	−0.31197
H	+1.99670	+0.90472	−0.34979
H	+4.37807	+0.44389	+0.64814
C	+1.49437	−1.69862	−1.10606
H	+3.50099	−0.98566	−0.96820
H	+2.95309	−1.88134	+0.44605
C	+0.48905	−2.61530	−0.63688
C	−0.37597	−2.87821	−1.71865
N	+0.07989	−2.15208	−2.78940
C	+1.19849	−1.44941	−2.41874
C	+0.24463	−3.22024	+0.60242
C	−1.47054	−3.73600	−1.60132
H	−0.32971	−2.14567	−3.70579

H	+1.72337	−0.83176	−3.13480
C	−0.83359	−4.06944	+0.72121
H	+0.88298	−3.00847	+1.45715
H	−1.04243	−4.54424	+1.67395
C	−1.68104	−4.32552	−0.37256
H	−2.13059	−3.93225	−2.44048
H	−2.52285	−4.99816	−0.24376

27

∗ E = +7.622 kcal/mol ; (64) 358_169_306_291_084

H	+0.00000	+0.00000	+0.00000
O	+0.00000	+0.00000	+0.96030
C	+1.26293	+0.00000	+1.44207
C	+2.36438	−0.05125	+0.37945
N	+3.68064	+0.19830	+0.91258
H	+3.69309	+1.07053	+1.42818
O	+1.46463	+0.03874	+2.61490
C	+2.35023	−1.41558	−0.34892
H	+2.13080	+0.72616	−0.36354
H	+3.90429	−0.53174	+1.58284
C	+2.77394	−2.55383	+0.52351
H	+1.35132	−1.61570	−0.75899
H	+3.03421	−1.32743	−1.19808
C	+1.96192	−3.30542	+1.44327
C	+2.80151	−4.26804	+2.04411
N	+4.05632	−4.10190	+1.51774
C	+4.03388	−3.07612	+0.60835
C	+0.60996	−3.26033	+1.80726
C	+2.33186	−5.17997	+2.98906
H	+4.86384	−4.64897	+1.75382
H	+4.92749	−2.78562	+0.07300
C	+0.14191	−4.16141	+2.73957
H	−0.05837	−2.52012	+1.37598
H	−0.90143	−4.13429	+3.03576
C	+0.99594	−5.11227	+3.32506
H	+2.99037	−5.91388	+3.44331
H	+0.59605	−5.80459	+4.05891

27

∗ E = +7.769 kcal/mol ; (65) 006_182_324_301_189

H	+0.00000	+0.00000	+0.00000
O	+0.00000	+0.00000	+0.96024

C	+1.25103	+0.00000	+1.44481
C	+2.37631	+0.14225	+0.40845
N	+3.69475	+0.08688	+0.99664
H	+3.69114	+0.53426	+1.90622
O	+1.44511	−0.06118	+2.62227
C	+2.25903	−0.87343	−0.73405
H	+2.23082	+1.14357	−0.02727
H	+3.96947	−0.87791	+1.14404
C	+2.28456	−2.30982	−0.30791
H	+1.35271	−0.68250	−1.32869
H	+3.09348	−0.67728	−1.41853
C	+2.37151	−3.42478	−1.20979
C	+2.34418	−4.59906	−0.42852
N	+2.24880	−4.21080	+0.88096
C	+2.21276	−2.84019	+0.95122
C	+2.46826	−3.53080	−2.60194
C	+2.41065	−5.86980	−0.99894
H	+2.20771	−4.82664	+1.67243
H	+2.13641	−2.34633	+1.91133
C	+2.53405	−4.78606	−3.17039
H	+2.49314	−2.63986	−3.22385
H	+2.61036	−4.88714	−4.24798
C	+2.50494	−5.94405	−2.37378
H	+2.38902	−6.76692	−0.38807
H	+2.55836	−6.91727	−2.85096

27

∗ E = +8.360 kcal/mol ; (66) 353_157_035_179_288

H	+0.00000	+0.00000	+0.00000
O	+0.00000	+0.00000	+0.95996
C	+1.25607	+0.00000	+1.43870
C	+2.35555	−0.16380	+0.40087
N	+3.57950	+0.37845	+0.93396
H	+3.62370	+0.20710	+1.93285
O	+1.46265	+0.05760	+2.61206
C	+2.42304	−1.67435	+0.03874
H	+2.07665	+0.39248	−0.50868
H	+4.38594	−0.03291	+0.47982
C	+3.46905	−1.94242	−0.98908
H	+2.63686	−2.23210	+0.95803
H	+1.44150	−2.01134	−0.32364

C	+3.40243	−1.59353	−2.38189
C	+4.62451	−1.98544	−2.96948
N	+5.39167	−2.54155	−1.97972
C	+4.69572	−2.51145	−0.79637
C	+2.42070	−0.99933	−3.18377
C	+4.89019	−1.79493	−4.32519
H	+6.31299	−2.92099	−2.10025
H	+5.12622	−2.91518	+0.10986
C	+2.67858	−0.80910	−4.52510
H	+1.46658	−0.69777	−2.75852
H	+1.92773	−0.35047	−5.15989
C	+3.90489	−1.20312	−5.08851
H	+5.83410	−2.10057	−4.76573
H	+4.07876	−1.03920	−6.14699

27

∗ E = +8.520 kcal/mol ; (67) 358_168_307_166_210

H	+0.00000	+0.00000	+0.00000
O	+0.00000	+0.00000	+0.96062
C	+1.25596	+0.00000	+1.44495
C	+2.36947	−0.04824	+0.39329
N	+3.67467	+0.23187	+0.95138
H	+3.65554	+1.10825	+1.46098
O	+1.45604	+0.02907	+2.62014
C	+2.36542	−1.42240	−0.29375
H	+2.14492	+0.71468	−0.36802
H	+3.90038	−0.48025	+1.63927
C	+3.17797	−1.47905	−1.54952
H	+2.71356	−2.16505	+0.43947
H	+1.33434	−1.71323	−0.53363
C	+2.90807	−2.34115	−2.66699
C	+3.89379	−2.07934	−3.64370
N	+4.71612	−1.10967	−3.13638
C	+4.28769	−0.75366	−1.88107
C	+1.92597	−3.30333	−2.93245
C	+3.92121	−2.74655	−4.86784
H	+5.51480	−0.72036	−3.60284
H	+4.80950	−0.01178	−1.29465
C	+1.95005	−3.96596	−4.14230
H	+1.16007	−3.53156	−2.19586
H	+1.19671	−4.71549	−4.36148

C +2.94006 −3.68879 −5.10091
H +4.68457 −2.53516 −5.61038
H +2.93271 −4.22770 −6.04282
27
∗ E = +8.899 kcal/mol ; (68) 003_189_201_178_083
H +0.00000 +0.00000 +0.00000
O +0.00000 +0.00000 +0.95929
C +1.25341 +0.00000 +1.44556
C +2.37215 +0.06862 +0.40925
N +3.62671 −0.15099 +1.08843
H +4.41046 +0.20526 +0.55804
O +1.44470 −0.00511 +2.62246
C +2.20503 −0.96277 −0.72506
H +2.28375 +1.07571 −0.04380
H +3.61164 +0.25779 +2.01450
C +3.28237 −0.84602 −1.75804
H +2.21826 −1.96320 −0.27638
H +1.23628 −0.84235 −1.22829
C +4.60722 −1.40610 −1.70638
C +5.26224 −1.03190 −2.89975
N +4.38013 −0.28194 −3.63263
C +3.19648 −0.17841 −2.94700
C +5.29042 −2.19731 −0.77200
C +6.57241 −1.41926 −3.18300
H +4.56124 +0.11026 −4.53846
H +2.36084 +0.36219 −3.37043
C +6.58345 −2.58527 −1.05209
H +4.80773 −2.47729 +0.15955
H +7.12662 −3.19759 −0.33972
C +7.21856 −2.19796 −2.24606
H +7.06414 −1.12219 −4.10418
H +8.23843 −2.51866 −2.43300
27
∗ E = +8.927 kcal/mol ; (69) 353_156_037_182_088
H +0.00000 +0.00000 +0.00000
O +0.00000 +0.00000 +0.95978
C +1.25733 +0.00000 +1.43720
C +2.35697 −0.16913 +0.39860
N +3.57132 +0.40346 +0.92221
H +3.63836 +0.22171 +1.91770

O	+1.46507	+0.05854	+2.60995
C	+2.42914	−1.68315	+0.05628
H	+2.07053	+0.36847	−0.51938
H	+4.39052	+0.03956	+0.45196
C	+3.50483	−1.99333	−0.93318
H	+2.61148	−2.23024	+0.99024
H	+1.45620	−2.01970	−0.32438
C	+4.87989	−2.28980	−0.63393
C	+5.54320	−2.48700	−1.86435
N	+4.61442	−2.31940	−2.85676
C	+3.39705	−2.02698	−2.29495
C	+5.60360	−2.41813	+0.55960
C	+6.90122	−2.79861	−1.93425
H	+4.79106	−2.40909	−3.84072
H	+2.52740	−1.87338	−2.91909
C	+6.94531	−2.72839	+0.49406
H	+5.11243	−2.27597	+1.51898
H	+7.51950	−2.83216	+1.40874
C	+7.58749	−2.91451	−0.74344
H	+7.40030	−2.94568	−2.88704
H	+8.64539	−3.15558	−0.76068

27

* E = +9.560 kcal/mol ; (70) 354_162_030_293_082

H	+0.00000	+0.00000	+0.00000
O	+0.00000	+0.00000	+0.96027
C	+1.25964	+0.00000	+1.44090
C	+2.35130	−0.13373	+0.38833
N	+3.60344	+0.29000	+0.96507
H	+3.58924	+0.10520	+1.96329
O	+1.46458	+0.06247	+2.61228
C	+2.35603	−1.58389	−0.17562
H	+2.08152	+0.53444	−0.44606
H	+4.38241	−0.21541	+0.56294
C	+2.76895	−2.62275	+0.81399
H	+1.36444	−1.82317	−0.58528
H	+3.04849	−1.59080	−1.02574
C	+1.95457	−3.25039	+1.82054
C	+2.78369	−4.15333	+2.51958
N	+4.03503	−4.07350	+1.96555
C	+4.01856	−3.15938	+0.94399

C	+0.60638	−3.14317	+2.18593
C	+2.30883	−4.94459	+3.56549
H	+4.83363	−4.60955	+2.25165
H	+4.90985	−2.96609	+0.36206
C	+0.13267	−3.92578	+3.21678
H	−0.05506	−2.44654	+1.67796
H	−0.90773	−3.84907	+3.51461
C	+0.97731	−4.81762	+3.90087
H	+2.96005	−5.63331	+4.09485
H	+0.57319	−5.41589	+4.71115

27

∗ E = +9.575 kcal/mol ; (71) 024_208_205_324_307

H	+0.00000	+0.00000	+0.00000
O	+0.00000	+0.00000	+0.96580
C	+1.24547	+0.00000	+1.44551
C	+2.35563	+0.55147	+0.54320
N	+3.59257	−0.11496	+0.91523
H	+4.39569	+0.45520	+0.68434
O	+1.47340	−0.32369	+2.57380
C	+2.11832	+0.49057	−0.98203
H	+2.36849	+1.62393	+0.81224
H	+3.59207	−0.28233	+1.91608
C	+1.37987	−0.69973	−1.52060
H	+1.57484	+1.39067	−1.29434
H	+3.11036	+0.55970	−1.44550
C	+1.64207	−2.10594	−1.34123
C	+0.64878	−2.80283	−2.06382
N	−0.17068	−1.87200	−2.64763
C	+0.26839	−0.61705	−2.31696
C	+2.61997	−2.83699	−0.65063
C	+0.59903	−4.19602	−2.11299
H	−0.97435	−2.07612	−3.21317
H	−0.23303	+0.26444	−2.69481
C	+2.57356	−4.21392	−0.70129
H	+3.38330	−2.31337	−0.08446
H	+3.32109	−4.79442	−0.17063
C	+1.57255	−4.88680	−1.42368
H	−0.17407	−4.71499	−2.67097
H	+1.56518	−5.97206	−1.43784

27

∗ E = +10.534 kcal/mol ; (72) 006_067_176_179_289

H	+0.00000	+0.00000	+0.00000
O	+0.00000	+0.00000	+0.95956
C	+1.25901	+0.00000	+1.44874
C	+2.35316	+0.15351	+0.39676
N	+2.26196	+1.49683	−0.16822
H	+3.02199	+1.63745	−0.82506
O	+1.45085	−0.10840	+2.61821
C	+3.72980	−0.03455	+1.03437
H	+2.20268	−0.64254	−0.35849
H	+1.39199	+1.66761	−0.65655
C	+4.81919	+0.08089	+0.01880
H	+3.84327	+0.72042	+1.81797
H	+3.75761	−1.01288	+1.52710
C	+5.12389	−0.87003	−1.01611
C	+6.19128	−0.33851	−1.77251
N	+6.51417	+0.87392	−1.22249
C	+5.68823	+1.12009	−0.15186
C	+4.60244	−2.12365	−1.35943
C	+6.73997	−1.01836	−2.85974
H	+7.24829	+1.48068	−1.53879
H	+5.78485	+2.02883	+0.42622
C	+5.14224	−2.79902	−2.43430
H	+3.79320	−2.56178	−0.78045
H	+4.75155	−3.77253	−2.71156
C	+6.20046	−2.24793	−3.17817
H	+7.56152	−0.60033	−3.43328
H	+6.60416	−2.80387	−4.01819

27

∗ E = +10.580 kcal/mol ; (73) 003_188_198_173_162

H	+0.00000	+0.00000	+0.00000
O	+0.00000	+0.00000	+0.95977
C	+1.25175	+0.00000	+1.44760
C	+2.37255	+0.06351	+0.41272
N	+3.62615	−0.13198	+1.09989
H	+4.41887	+0.17551	+0.55618
O	+1.44360	+0.00487	+2.62420
C	+2.22375	−0.98033	−0.70163
H	+2.26148	+1.06846	−0.04521
H	+3.61837	+0.30574	+2.01200

C	+3.22661	−0.79035	−1.79744
H	+2.35085	−1.97151	−0.25009
H	+1.21574	−0.96168	−1.14282
C	+3.58661	−1.77562	−2.77835
C	+4.52949	−1.18205	−3.64379
N	+4.73039	+0.10011	−3.20665
C	+3.94774	+0.32952	−2.10019
C	+3.19562	−3.10175	−2.99824
C	+5.09079	−1.87517	−4.71634
H	+5.36146	+0.76415	−3.61618
H	+3.96839	+1.29443	−1.61083
C	+3.74702	−3.79003	−4.05828
H	+2.47303	−3.58015	−2.34282
H	+3.45687	−4.81905	−4.24279
C	+4.68609	−3.17995	−4.90880
H	+5.81701	−1.40904	−5.37501
H	+5.10210	−3.74903	−5.73391

27

∗ E = +10.871 kcal/mol ; (74) 009_079_182_070_081

H	+0.00000	+0.00000	+0.00000
O	+0.00000	+0.00000	+0.95992
C	+1.26074	+0.00000	+1.44041
C	+2.34432	+0.22869	+0.38447
N	+2.37003	+1.66170	+0.10505
H	+3.04955	+1.87426	−0.61457
O	+1.46369	−0.12831	+2.60627
C	+3.69720	−0.23172	+0.92181
H	+2.09209	−0.39079	−0.49819
H	+1.47210	+2.01972	−0.19642
C	+3.76626	−1.71906	+1.04360
H	+4.47857	+0.12624	+0.23881
H	+3.85806	+0.25535	+1.88758
C	+4.04294	−2.64443	−0.01966
C	+3.95493	−3.94496	+0.52466
N	+3.63780	−3.80670	+1.85012
C	+3.51960	−2.47318	+2.15398
C	+4.35686	−2.49310	−1.37549
C	+4.17155	−5.08643	−0.24605
H	+3.49617	−4.56025	+2.49758
H	+3.24646	−2.14558	+3.14746

C	+4.56943	−3.62056	−2.14221
H	+4.43605	−1.50199	−1.81613
H	+4.81444	−3.51991	−3.19453
C	+4.47689	−4.90562	−1.58025
H	+4.10336	−6.08111	+0.18380
H	+4.65035	−5.77340	−2.20835

27

* E = +10.901 kcal/mol ; (75) 356_290_304_056_079

H	+0.00000	+0.00000	+0.00000
O	+0.00000	+0.00000	+0.96008
C	+1.26366	+0.00000	+1.44285
C	+2.37492	−0.09527	+0.39184
N	+2.44381	−1.40283	−0.24396
H	+2.56247	−2.14459	+0.43492
O	+1.45627	+0.02283	+2.61843
C	+2.22716	+0.99574	−0.67532
H	+3.30511	+0.07320	+0.94431
H	+1.61503	−1.60744	−0.79061
C	+2.19457	+2.36330	−0.07614
H	+3.06848	+0.88957	−1.36897
H	+1.32412	+0.81642	−1.27956
C	+3.33995	+3.10856	+0.36502
C	+2.86218	+4.30820	+0.93332
N	+1.49512	+4.28442	+0.84141
C	+1.10055	+3.11520	+0.24242
C	+4.71840	+2.86857	+0.32816
C	+3.72200	+5.27117	+1.46075
H	+0.87510	+4.99484	+1.18493
H	+0.05114	+2.89842	+0.09473
C	+5.57177	+3.81789	+0.85004
H	+5.10792	+1.94971	−0.10337
H	+6.64331	+3.64858	+0.83155
C	+5.07560	+5.00808	+1.41085
H	+3.34403	+6.19116	+1.89570
H	+5.77321	+5.73464	+1.81470

27

* E = +12.548 kcal/mol ; (76) 352_273_296_162_081

H	+0.00000	+0.00000	+0.00000
O	+0.00000	+0.00000	+0.96043
C	+1.25822	+0.00000	+1.44871

C	+2.38185	−0.20025	+0.42920
N	+2.63031	−1.62163	+0.21551
H	+2.96351	−2.06157	+1.06505
O	+1.44822	+0.07941	+2.62386
C	+2.11563	+0.49578	−0.91371
H	+3.27774	+0.23259	+0.88289
H	+1.78085	−2.10176	−0.06371
C	+3.34344	+0.68075	−1.74794
H	+1.38143	−0.09639	−1.48634
H	+1.66136	+1.47718	−0.72684
C	+3.99567	−0.27889	−2.59744
C	+5.10300	+0.37318	−3.18208
N	+5.12128	+1.65749	−2.70479
C	+4.06267	+1.83721	−1.85240
C	+3.74262	−1.61469	−2.93388
C	+5.95706	−0.26635	−4.08115
H	+5.79451	+2.36043	−2.94903
H	+3.88990	+2.79470	−1.38019
C	+4.57841	−2.24807	−3.82832
H	+2.91232	−2.14475	−2.47917
H	+4.39422	−3.28320	−4.09671
C	+5.67844	−1.57964	−4.39503
H	+6.80633	+0.24937	−4.51871
H	+6.32124	−2.10889	−5.09110

13 Tyr

13.1 Reference Z-matrix for Tyr

Bond distances are given in Å; bond and dihedral angles, in degrees.

```
h
o    1  oh2
c    2  co3     1  coh3
c    3  cc4     2  cco4     1  dih4
n    4  nc5     3  ncc5     2  dih5
h    5  hn6     4  hnc6     3  dih6
o    3  oc7     2  oco7     4  dih7
c    4  cc8     3  ccc8     5  dih8
h    4  hc9     3  hcc9     5  dih9
h    5  hn10    4  hnc10    6  dih10
c    8  cc11    4  ccc11    3  dih11
h    8  hc12    4  hcc12   11  dih12
h    8  hc13    4  hcc13   11  dih13
c   11  cc14    8  ccc14    4  dih14
c   14  cc15   11  ccc15    8  dih15
c   15  cc16   14  ccc16   11  dih16
c   16  cc17   15  ccc17   14  dih17
c   17  cc18   16  ccc18   15  dih18
h   14  hc19   11  hcc19   15  dih19
h   15  hc20   14  hcc20   16  dih20
o   16  oc21   15  occ21   17  dih21
h   17  hc22   16  hcc22   15  dih22
h   18  hc23   17  hcc23   16  dih23
h   21  ho24   16  hoc24   15  dih24

oh2      0.96847042
co3      1.35864705
coh3     111.97501043
cc4      1.50866699
cco4     110.93820265
dih4     181.1207533
nc5      1.46646889
ncc5     106.98340445
dih5     49.61119113
hn6      1.00323256
```

hnc6	112.65255916
dih6	180.13398177
oc7	1.20145719
oco7	122.11622808
dih7	181.4526646
cc8	1.53540831
ccc8	109.56827282
dih8	119.46074334
hc9	1.09033397
hcc9	106.97624226
dih9	238.01246394
hn10	1.00307122
hnc10	112.64562447
dih10	128.51156468
cc11	1.51451332
ccc11	111.54443156
dih11	177.31744306
hc12	1.08135651
hcc12	107.43156048
dih12	121.21241788
hc13	1.08210802
hcc13	108.94263758
dih13	237.75949879
cc14	1.39119803
ccc14	121.02807612
dih14	93.60458368
cc15	1.3790168
ccc15	121.22004292
dih15	181.42980449
cc16	1.38371791
ccc16	120.07429513
dih16	−0.14587878
cc17	1.38108791
ccc17	119.46985985
dih17	−0.06125244
cc18	1.38415458
ccc18	120.08519755
dih18	0.09683998
hc19	1.07236186
hcc19	119.2770432

```
dih19    180.03932385
hc20     1.06969896
hcc20    121.45439924
dih20    180.32426862
oc21     1.37673905
occ21    117.29906782
dih21    180.08149534
hc22     1.07312921
hcc22    120.20028446
dih22    180.37328095
hc23     1.07326513
hcc23    119.14503059
dih23    180.50485137
ho24     0.96414541
hoc24    112.97039082
dih24    178.61031165
```

13.2 Reference energy and target torsions for Tyr

- Reference M08-HX/MG3S energy: -630.0483669 hartree
- Target torsions:
  - torsion1: dih4
  - torsion2: dih5
  - torsion3: dih6
  - torsion4: dih11
  - torsion5: dih24

13.3 Properties of Tyr conformers

Table S13: Relative energy (kcal mol^{-1}), rotational constants (B1 to B3, GHz) and dipolar moment (Debye) for the conformers of Tyr located at the M08-HX/MG3S level.

Conformer	Rel. Energy	B1	B2	B3	Dip. Mom.

1	0.000	0.434	0.473	1.529	3.576
2	0.381	0.432	0.471	1.534	6.196
3	1.076	0.443	0.489	1.497	0.884
4	1.085	0.443	0.489	1.499	2.469
5	1.368	0.325	0.344	2.425	5.302
6	1.383	0.325	0.344	2.427	6.002
7	1.691	0.325	0.340	2.398	4.995
8	1.729	0.443	0.485	1.492	1.815
9	1.734	0.326	0.339	2.404	5.821
10	1.772	0.446	0.490	1.489	2.320
11	2.690	0.324	0.345	2.402	2.306
12	2.706	0.424	0.462	1.701	1.498
13	2.710	0.423	0.463	1.704	2.421
14	2.712	0.324	0.345	2.402	3.071
15	2.919	0.325	0.336	2.455	2.264
16	2.980	0.324	0.336	2.454	2.865
17	2.995	0.317	0.348	2.315	0.400
18	3.023	0.450	0.498	1.508	2.635
19	3.087	0.451	0.500	1.500	2.377
20	3.097	0.317	0.348	2.317	2.574
21	3.424	0.459	0.507	1.488	1.505
22	3.491	0.460	0.509	1.489	3.234
23	3.645	0.420	0.460	1.714	1.541
24	3.653	0.420	0.461	1.718	2.886
25	3.670	0.420	0.449	1.658	0.705
26	3.711	0.418	0.448	1.658	2.734
27	3.813	0.325	0.345	2.418	1.873
28	3.853	0.325	0.345	2.419	3.684
29	4.179	0.360	0.401	1.835	3.544
30	4.284	0.411	0.441	1.700	0.754
31	4.320	0.379	0.413	1.732	1.340
32	4.537	0.360	0.401	1.838	5.771
33	4.555	0.378	0.413	1.733	3.535
34	4.579	0.318	0.345	2.349	3.063
35	4.603	0.318	0.345	2.352	0.878
36	4.944	0.448	0.497	1.521	2.828
37	4.983	0.367	0.403	1.875	1.775

38	5.045	0.414	0.451	1.691	3.086
39	5.045	0.418	0.452	1.688	2.574
40	5.049	0.367	0.404	1.876	2.569
41	5.067	0.400	0.434	1.822	3.359
42	5.228	0.446	0.495	1.529	5.164
43	5.289	0.396	0.429	1.836	4.588
44	5.606	0.336	0.382	1.991	3.775
45	5.606	0.336	0.382	1.991	3.775
46	5.918	0.335	0.381	1.999	5.978
47	5.924	0.403	0.438	1.826	4.475
48	6.088	0.442	0.490	1.524	2.773
49	6.155	0.397	0.432	1.841	5.401
50	6.259	0.441	0.489	1.523	3.621
51	6.351	0.332	0.372	2.005	2.039
52	6.379	0.332	0.373	2.003	2.454
53	6.576	0.406	0.439	1.706	2.663
54	7.713	0.324	0.346	2.392	4.427
55	7.816	0.324	0.346	2.393	5.354
56	7.827	0.442	0.491	1.542	3.633
57	7.862	0.454	0.462	1.563	3.491
58	7.916	0.316	0.349	2.286	2.968
59	8.084	0.316	0.349	2.285	3.907
60	8.087	0.442	0.491	1.540	4.742
61	8.164	0.454	0.459	1.565	4.908
62	10.484	0.389	0.425	1.663	3.437
63	10.764	0.322	0.335	2.320	4.269
64	10.805	0.321	0.335	2.322	3.383
65	10.849	0.387	0.423	1.668	6.030

13.4 Vibrational frequencies of Tyr conformers

Harmonic vibrational zero point energy (ZPE) and frequencies for each conformer in cm^{-1}.

- Conformer (1); ZPE = 43035.978 cm^{-1}:

42.61	48.38	85.97	100.86	176.25	213.51
314.53	328.89	342.36	350.95	384.16	422.99
430.62	433.32	515.80	538.54	559.43	607.16
653.10	737.40	745.05	792.68	816.54	844.26
847.84	860.44	883.30	895.73	930.73	981.43
1001.11	1033.71	1038.81	1104.95	1127.32	1173.37
1193.20	1193.75	1228.32	1234.15	1248.40	1291.91
1310.85	1339.81	1358.28	1362.02	1397.28	1434.93
1467.85	1485.08	1563.46	1654.61	1662.75	1690.77
1881.41	3046.68	3057.60	3112.39	3160.79	3171.48
3192.57	3210.01	3531.99	3639.35	3675.41	3910.72

- Conformer (2); ZPE = 42996.877 cm^{-1}:

37.99	48.10	84.55	99.45	175.71	212.37
301.56	307.54	341.96	349.24	383.53	423.24
429.30	432.45	515.56	538.45	558.88	607.26
652.85	736.79	744.85	792.29	809.95	834.59
850.14	863.88	886.02	893.38	929.68	968.50
1011.61	1032.59	1040.58	1103.62	1128.81	1173.45
1188.46	1192.05	1227.78	1234.71	1248.56	1292.44
1310.20	1339.43	1357.73	1362.06	1396.72	1433.99
1467.74	1484.20	1563.07	1653.66	1659.40	1690.22
1882.45	3047.28	3057.19	3111.47	3158.81	3177.06
3186.34	3210.60	3531.39	3638.90	3681.36	3907.75

- Conformer (3); ZPE = 42902.690 cm^{-1}:

39.37	48.27	79.08	91.74	166.46	212.77
260.72	289.03	327.09	370.28	380.86	420.86
425.98	433.23	482.95	508.87	557.42	607.59
643.23	653.75	736.91	748.70	777.85	836.73
850.51	862.39	886.15	914.16	945.19	979.64
996.81	1019.51	1035.88	1121.72	1139.56	1162.88
1179.68	1189.81	1200.03	1236.94	1250.08	1302.65
1309.63	1342.29	1355.47	1358.11	1379.54	1413.83
1474.02	1486.55	1564.81	1665.30	1667.27	1693.17
1867.77	3041.61	3063.74	3116.89	3167.26	3172.49
3183.82	3211.94	3532.70	3613.63	3840.67	3909.53

- Conformer (4); ZPE = 42877.182 cm^{-1}:

38.39	47.49	78.37	90.99	166.81	212.36
260.38	288.47	322.19	333.33	380.65	421.42
425.33	432.64	482.56	509.13	556.68	606.72
642.83	653.73	736.92	748.32	777.46	841.08
844.32	862.54	886.81	913.55	945.77	976.10
999.37	1018.28	1035.70	1121.96	1139.48	1161.97
1180.18	1189.85	1199.18	1237.03	1250.22	1302.67
1309.38	1340.95	1355.19	1359.28	1378.56	1413.29
1473.36	1486.57	1564.85	1664.27	1668.09	1693.49
1867.51	3041.03	3064.55	3117.61	3166.61	3176.84
3182.25	3210.40	3533.37	3614.29	3842.07	3911.31

- Conformer (5); ZPE = 42980.242 cm^{-1}:

43.10	48.70	64.08	83.09	170.26	188.54
302.35	312.30	347.17	351.28	360.94	414.53
424.11	427.01	493.35	536.69	571.57	652.27
665.60	719.10	750.09	802.96	809.45	839.80
854.92	866.88	881.59	904.88	962.79	975.85
993.32	996.88	1032.54	1112.20	1134.13	1160.73
1186.11	1196.11	1235.83	1241.94	1285.55	1293.86
1310.62	1336.43	1357.07	1367.76	1370.83	1428.40
1472.43	1484.90	1563.02	1662.00	1664.48	1690.52
1891.67	3028.31	3044.06	3102.99	3163.74	3165.26
3180.34	3211.51	3543.23	3625.17	3692.44	3908.87

- Conformer (6); ZPE = 42972.982 cm^{-1}:

41.46	49.31	63.70	83.37	170.15	187.86
302.88	310.55	342.65	351.57	361.01	413.61
424.48	426.65	493.58	536.84	571.56	652.13
665.67	719.11	749.62	799.99	809.54	838.02
856.08	866.57	881.62	904.04	962.79	974.35
993.16	999.78	1032.62	1112.83	1134.49	1161.66
1186.08	1192.35	1235.16	1242.80	1287.21	1293.00
1311.50	1338.16	1354.92	1368.75	1372.57	1428.74
1471.73	1484.64	1563.54	1661.36	1662.28	1690.68
1892.19	3029.28	3043.46	3101.26	3162.47	3169.32
3180.23	3209.58	3542.58	3624.24	3692.44	3908.17

- Conformer (7); ZPE = 42956.301 cm^{-1}:

36.62	53.52	58.76	72.04	170.99	203.60
283.30	306.09	344.99	358.25	366.25	419.98
424.94	429.45	495.26	540.05	541.59	621.68
653.55	732.58	749.53	796.94	797.73	840.77
853.21	866.96	895.32	902.79	931.34	976.81
993.93	1034.21	1055.04	1115.37	1136.72	1155.95
1189.33	1193.91	1228.86	1234.25	1263.60	1293.11
1310.37	1334.85	1357.24	1361.24	1393.59	1428.66
1476.48	1483.75	1565.62	1660.40	1666.22	1692.82
1889.09	3029.10	3043.15	3106.46	3163.51	3165.84
3179.97	3209.64	3533.87	3624.02	3702.25	3915.34

- Conformer (8); ZPE = 42892.070 cm^{-1}:

17.81	46.10	76.69	93.64	169.29	215.52
287.94	296.28	329.30	343.79	378.79	424.02
425.28	432.37	501.37	521.54	546.08	586.08
651.26	652.93	736.55	751.00	776.18	840.94
843.57	861.34	884.17	891.18	940.77	983.15
999.44	1011.59	1035.54	1121.70	1133.56	1166.81
1190.23	1195.26	1204.63	1236.70	1248.94	1307.99
1312.23	1342.82	1357.30	1361.42	1382.70	1394.29
1467.08	1485.73	1562.65	1661.54	1665.18	1690.84
1851.57	3036.97	3069.47	3123.84	3167.70	3174.07
3185.14	3208.28	3541.50	3628.23	3846.32	3909.93

- Conformer (9); ZPE = 42940.586 cm^{-1}:

34.11	50.55	58.04	72.40	169.65	203.33
281.37	305.86	346.65	357.69	366.22	419.75
425.45	427.66	495.24	540.02	540.84	621.82
653.27	732.71	748.07	797.58	798.97	837.97
855.19	865.76	895.46	901.44	931.29	971.72
997.65	1033.14	1055.57	1114.85	1137.05	1156.49
1189.22	1192.21	1229.39	1233.73	1263.35	1294.65
1310.49	1332.00	1355.31	1364.13	1394.02	1429.35
1475.62	1482.98	1564.74	1660.86	1663.33	1690.86
1889.19	3029.46	3043.16	3104.45	3160.79	3164.12
3177.97	3208.35	3533.84	3623.86	3703.35	3915.57

- Conformer (10); ZPE = 42886.246 cm^{-1}:

23.37	45.59	75.57	91.67	167.94	214.32
280.01	293.72	328.47	344.42	377.72	423.06
426.52	432.20	498.80	527.03	550.63	584.52
652.59	653.56	737.09	751.94	777.59	833.70
850.85	862.42	885.05	890.23	939.30	977.88
1002.58	1011.52	1035.31	1121.46	1133.22	1165.80
1189.82	1193.43	1206.31	1236.76	1245.79	1307.77
1311.47	1342.60	1357.17	1359.62	1383.48	1394.56
1467.36	1484.85	1562.91	1661.92	1663.50	1691.17
1852.09	3043.49	3068.96	3123.31	3165.81	3175.94
3181.83	3209.96	3541.56	3626.89	3843.89	3910.63

- Conformer (11); ZPE = 42788.192 cm^{-1}:

39.84	51.25	62.93	73.45	166.41	185.63
271.17	297.54	322.02	328.51	356.61	402.48
423.72	426.94	487.72	517.17	560.52	595.09
652.23	657.75	728.82	755.59	805.03	834.45
845.68	853.94	867.55	894.13	924.98	971.51
994.48	1024.38	1033.94	1117.57	1144.88	1164.34
1184.32	1187.61	1202.36	1235.88	1259.25	1298.19
1310.49	1330.52	1351.52	1355.30	1360.36	1455.41
1475.26	1484.06	1563.65	1635.83	1663.30	1690.99
1868.51	3031.40	3041.70	3091.09	3164.88	3168.40
3180.95	3210.25	3542.38	3636.17	3847.17	3908.95

- Conformer (12); ZPE = 42796.786 cm^{-1}:

37.43	56.03	59.03	73.81	147.71	236.03
244.66	271.27	309.95	325.77	350.15	389.58
422.17	427.42	470.26	527.49	563.14	611.65
648.80	655.47	743.78	771.69	801.95	819.33
838.30	860.79	877.03	887.78	970.21	972.68
992.25	1033.96	1038.48	1110.60	1132.83	1165.45
1181.95	1187.72	1194.12	1228.26	1254.11	1302.31
1308.27	1329.29	1352.42	1356.43	1374.93	1436.65
1473.60	1485.54	1563.80	1660.44	1666.76	1692.19
1855.10	3039.23	3075.74	3105.22	3164.82	3169.73
3179.97	3209.87	3533.62	3621.20	3836.22	3909.15

- Conformer (13); ZPE = 42801.775 cm^{-1}:

38.05	58.51	60.43	74.44	148.72	235.04
243.27	271.42	309.95	326.00	348.89	390.48
422.23	427.65	469.89	527.57	563.14	609.99
649.20	655.56	743.53	771.14	802.09	818.92
837.58	862.74	877.26	889.26	967.12	972.92
993.55	1033.51	1038.65	1111.69	1133.19	1166.75
1181.45	1187.13	1197.06	1228.55	1253.15	1301.83
1308.35	1332.22	1351.16	1357.16	1374.28	1436.26
1473.93	1485.57	1564.76	1660.79	1666.27	1693.45
1855.88	3038.64	3075.49	3105.34	3164.78	3169.52
3180.77	3209.67	3533.42	3620.70	3835.74	3909.88

- Conformer (14); ZPE = 42801.726 cm^{-1}:

40.86	51.58	63.32	73.42	166.54	185.97
273.26	297.94	324.89	341.64	356.82	402.79
423.36	427.19	487.68	516.98	560.47	595.36
652.26	657.68	728.87	755.47	804.98	836.63
843.76	854.70	868.24	894.77	925.49	973.20
992.52	1024.81	1033.37	1117.76	1144.77	1164.20
1185.80	1188.85	1200.66	1236.40	1259.87	1297.86
1309.82	1330.80	1350.28	1356.27	1359.86	1455.00
1475.25	1484.56	1563.11	1636.43	1664.77	1690.87
1868.91	3031.16	3041.06	3091.28	3164.18	3169.55
3180.17	3210.93	3542.70	3636.21	3846.90	3910.44

- Conformer (15); ZPE = 42815.176 cm^{-1}:

36.60	53.30	58.05	62.31	172.36	201.43
295.79	313.66	322.55	334.56	360.69	404.00
424.59	428.79	489.98	516.73	546.37	592.07
631.53	653.26	740.35	755.94	789.96	837.02
853.27	861.73	867.19	896.70	956.10	976.21
994.34	1023.14	1034.71	1120.50	1152.38	1177.61
1180.27	1188.35	1196.67	1236.36	1262.85	1303.17
1308.41	1335.03	1344.61	1355.27	1369.48	1446.38
1468.03	1483.99	1563.88	1645.13	1664.36	1691.59
1872.29	2927.26	3059.65	3113.65	3165.99	3167.63
3182.14	3208.94	3550.33	3638.45	3849.96	3914.41

- Conformer (16); ZPE = 42832.194 cm^{-1}:

38.68	53.90	58.71	62.77	173.07	201.84
295.51	314.43	321.37	345.72	362.16	404.92
425.02	429.49	489.63	516.79	547.00	592.41
631.61	653.38	740.11	756.32	790.15	838.53
850.09	861.38	868.86	898.00	955.89	973.78
997.54	1023.21	1035.00	1121.30	1152.15	1178.53
1179.31	1188.77	1199.05	1236.94	1262.15	1303.83
1308.67	1334.59	1345.68	1356.98	1368.26	1445.88
1468.59	1484.52	1564.74	1645.66	1665.65	1692.77
1872.20	2925.54	3059.49	3113.84	3163.67	3174.67
3179.78	3209.56	3550.55	3638.81	3850.32	3914.67

- Conformer (17); ZPE = 42744.012 cm^{-1}:

31.33	46.03	64.05	69.59	170.55	182.20
192.65	274.58	302.56	310.34	364.09	398.81
422.09	427.69	486.75	507.97	556.88	599.59
652.15	663.41	725.92	756.45	801.33	835.50
855.47	863.23	881.42	896.76	945.97	979.15
991.46	1014.09	1033.87	1114.44	1144.81	1160.13
1182.69	1188.58	1195.76	1237.95	1261.60	1300.46
1307.05	1343.21	1351.98	1355.62	1362.50	1429.32
1473.51	1484.66	1563.18	1663.31	1667.99	1690.88
1861.07	3032.76	3064.04	3095.87	3166.81	3168.82
3196.00	3208.12	3537.14	3623.99	3842.08	3907.77

- Conformer (18); ZPE = 42794.673 cm^{-1}:

28.36	60.36	77.72	95.79	161.57	202.49
220.97	301.37	323.36	349.27	369.60	422.78
423.71	431.45	480.56	516.86	566.51	599.21
651.47	655.55	735.63	749.80	781.16	826.01
837.90	853.61	863.13	894.41	940.89	974.13
989.30	997.11	1034.96	1113.96	1134.76	1160.50
1188.98	1191.61	1203.50	1236.30	1248.58	1269.35
1310.11	1332.22	1341.26	1358.75	1376.33	1452.44
1478.45	1486.83	1565.39	1638.70	1666.41	1694.23
1875.40	3033.63	3045.08	3095.51	3164.21	3171.40
3181.05	3210.95	3545.59	3640.20	3850.92	3909.73

- Conformer (19); ZPE = 42787.191 cm^{-1}:

26.78	59.79	79.25	96.58	160.58	202.23
219.75	302.13	318.77	329.95	370.88	421.75
424.87	431.86	481.38	516.59	567.56	600.61
651.97	655.85	735.68	749.99	781.69	823.38
834.85	859.18	862.70	894.63	938.75	970.09
993.82	999.88	1035.10	1113.65	1135.36	1162.87
1186.84	1190.39	1205.31	1236.24	1249.72	1270.25
1310.49	1333.71	1343.03	1357.17	1376.34	1453.77
1478.72	1486.87	1565.66	1638.83	1665.53	1694.15
1875.49	3032.91	3043.90	3095.34	3165.41	3168.14
3181.62	3211.81	3546.81	3642.15	3848.24	3908.82

- Conformer (20); ZPE = 42746.556 cm^{-1}:

32.39	46.06	64.56	70.59	170.42	182.77
192.84	274.08	301.48	313.87	364.02	398.88
422.64	427.34	486.76	507.90	556.93	599.37
652.02	663.37	725.70	756.53	801.35	835.53
855.87	862.43	879.78	897.22	945.99	976.96
993.67	1012.90	1033.54	1114.53	1144.65	1160.20
1183.87	1187.77	1193.00	1238.15	1261.82	1300.54
1307.83	1342.38	1352.26	1356.26	1362.68	1428.30
1473.58	1484.26	1563.26	1663.48	1667.86	1690.13
1861.56	3032.26	3063.43	3095.43	3162.63	3178.61
3191.42	3210.97	3537.64	3624.16	3842.47	3909.97

- Conformer (21); ZPE = 42829.132 cm^{-1}:

36.81	53.75	74.53	91.84	161.22	213.70
269.43	310.12	320.65	329.96	369.60	423.60
425.46	432.63	488.15	534.06	565.17	575.75
648.09	653.40	736.75	754.00	786.09	831.40
843.02	851.85	863.28	891.01	933.48	979.97
993.55	1005.72	1035.04	1106.38	1127.72	1162.61
1190.95	1194.67	1220.16	1233.11	1236.15	1268.63
1308.96	1338.57	1357.13	1362.28	1374.60	1444.39
1470.96	1486.03	1563.15	1654.58	1664.08	1691.56
1854.44	3044.42	3059.46	3101.52	3165.53	3168.44
3180.73	3208.75	3543.46	3633.92	3848.24	3909.60

- Conformer (22); ZPE = 42814.911 cm^{-1}:

35.46	53.48	73.95	90.81	160.99	212.77
266.97	308.90	315.71	327.85	369.50	421.99
426.17	432.13	487.22	533.91	566.32	576.07
648.18	653.27	736.73	753.76	786.72	830.19
835.47	859.95	863.38	890.55	931.75	969.44
1001.71	1006.15	1034.84	1105.14	1128.19	1161.15
1189.40	1193.84	1219.76	1232.78	1236.45	1268.56
1309.08	1338.35	1357.50	1361.62	1374.48	1444.04
1470.43	1485.61	1563.39	1653.78	1662.30	1691.88
1855.27	3044.29	3061.22	3101.12	3163.52	3171.06
3179.60	3209.39	3543.24	3633.83	3847.65	3909.58

- Conformer (23); ZPE = 42793.892 cm^{-1}:

35.67	54.44	59.06	75.45	148.36	235.06
253.87	284.79	304.86	331.17	336.39	388.69
421.94	427.60	474.15	525.47	559.62	613.09
645.94	654.38	743.29	774.45	802.10	821.57
838.26	859.09	870.19	880.55	965.26	978.98
990.81	1034.05	1052.39	1118.82	1134.64	1163.42
1177.41	1187.89	1195.06	1230.25	1247.96	1307.72
1314.49	1323.53	1345.99	1354.87	1361.60	1466.25
1476.32	1487.01	1563.31	1640.66	1666.40	1691.90
1859.95	3032.71	3066.10	3090.66	3164.16	3168.72
3179.44	3209.94	3537.11	3630.28	3840.09	3912.14

- Conformer (24); ZPE = 42799.143 cm^{-1}:

37.19	52.82	60.18	76.21	149.12	234.36
252.33	285.58	301.75	330.89	353.01	389.91
422.21	427.76	474.00	525.70	559.77	611.74
646.27	654.43	743.28	773.17	802.11	820.46
837.55	860.57	869.85	880.11	964.12	975.99
994.52	1034.19	1050.53	1120.03	1135.72	1164.16
1176.87	1187.22	1197.96	1230.49	1247.40	1308.12
1313.77	1324.63	1345.39	1356.17	1360.14	1466.40
1475.91	1487.21	1564.30	1640.52	1665.75	1693.21
1860.02	3032.35	3064.38	3090.14	3164.28	3167.81
3180.33	3209.66	3536.91	3629.93	3839.05	3910.39

- Conformer (25); ZPE = 42800.674 cm^{-1}:

31.95	41.09	72.24	83.79	149.03	240.61
251.92	280.63	319.85	325.70	335.84	380.65
422.16	428.05	498.48	543.85	561.04	594.55
609.03	653.05	741.91	772.75	800.48	816.94
837.12	859.62	876.10	879.12	963.10	970.95
992.39	1021.61	1034.54	1109.17	1133.43	1151.98
1188.56	1190.05	1196.09	1234.59	1258.18	1309.14
1325.32	1335.43	1355.07	1358.52	1382.48	1394.66
1476.25	1486.20	1564.36	1655.65	1664.20	1692.69
1872.79	3046.09	3075.40	3102.42	3161.60	3173.19
3178.00	3207.66	3541.47	3631.35	3850.30	3908.91

- Conformer (26); ZPE = 42820.721 cm^{-1}:

34.79	41.36	73.22	83.53	149.64	239.49
251.60	279.58	323.29	336.28	340.47	380.76
422.71	428.00	498.59	544.14	561.59	592.56
609.03	653.02	742.75	773.39	800.20	817.07
838.03	859.32	876.21	878.21	962.47	974.77
993.75	1020.52	1034.98	1108.82	1133.40	1151.51
1188.46	1190.60	1195.63	1234.65	1258.41	1309.59
1325.23	1336.12	1355.27	1360.18	1381.29	1394.92
1476.86	1485.98	1565.32	1655.36	1665.92	1691.65
1873.26	3045.80	3076.53	3102.62	3164.88	3166.40
3182.35	3209.85	3541.72	3631.65	3849.56	3916.32

- Conformer (27); ZPE = 42768.469 cm^{-1}:

34.90	44.57	57.14	63.54	162.68	184.19
274.07	293.19	318.72	332.20	353.63	395.47
423.03	426.45	492.44	532.54	560.62	586.48
635.39	652.52	734.36	759.64	807.66	840.17
851.36	857.38	871.20	895.59	911.03	976.25
993.92	1031.05	1032.60	1113.42	1118.78	1149.49
1187.30	1188.94	1210.76	1235.31	1261.72	1299.64
1310.07	1331.89	1352.67	1355.29	1369.43	1433.73
1474.30	1483.46	1562.60	1650.55	1661.76	1690.53
1873.93	3031.20	3069.90	3089.13	3160.79	3172.25
3178.69	3207.20	3538.17	3626.62	3849.61	3911.82

- Conformer (28); ZPE = 42787.685 cm^{-1}:

35.69	44.95	57.49	64.12	162.79	184.07
274.60	293.91	319.76	347.75	357.84	395.32
424.06	426.69	492.70	532.88	560.94	585.99
635.50	652.55	734.58	759.73	807.76	838.16
854.14	857.65	871.73	895.58	910.56	972.16
998.02	1030.51	1034.40	1113.45	1119.00	1149.40
1186.83	1191.04	1210.91	1234.81	1261.95	1300.50
1311.02	1331.82	1353.15	1356.66	1369.56	1433.99
1474.92	1483.91	1563.14	1650.74	1662.64	1689.71
1874.45	3030.97	3070.53	3089.07	3162.54	3165.12
3181.41	3211.04	3538.12	3626.73	3849.47	3912.16

- Conformer (29); ZPE = 42917.922 cm^{-1}:

25.62	52.13	79.70	85.47	170.00	232.89
286.70	318.66	331.01	339.94	370.17	381.24
420.68	427.96	512.75	530.63	565.72	587.94
653.67	739.43	743.30	758.60	797.98	826.93
840.15	858.26	876.13	882.72	948.77	975.32
990.60	1034.37	1082.64	1114.55	1136.53	1174.44
1187.74	1191.49	1203.40	1231.13	1235.58	1309.53
1317.01	1335.25	1355.31	1360.83	1389.93	1428.88
1467.93	1485.64	1564.31	1656.89	1664.32	1692.98
1891.85	3029.03	3037.39	3091.34	3155.99	3170.24
3201.80	3207.68	3540.54	3633.52	3735.38	3909.29

- Conformer (30); ZPE = 42822.638 cm^{-1}:

20.72	46.46	64.66	116.54	136.03	243.31
256.74	283.26	313.55	320.60	344.04	363.04
422.91	423.81	509.72	540.70	554.01	584.64
607.23	655.37	729.19	773.21	797.21	818.06
834.85	852.60	872.45	879.94	954.78	986.19
996.10	1029.87	1035.67	1118.41	1142.51	1163.87
1187.65	1195.91	1198.40	1229.11	1270.78	1311.63
1317.29	1334.53	1358.92	1365.94	1388.84	1409.60
1467.99	1486.80	1568.05	1656.90	1667.22	1693.87
1866.19	3036.36	3073.80	3090.21	3170.53	3174.10
3191.41	3209.06	3542.27	3632.26	3849.75	3907.67

- Conformer (31); ZPE = 42814.868 cm^{-1}:

38.01	47.52	81.97	87.76	161.61	234.54
277.90	288.29	329.86	334.84	343.15	360.29
421.69	427.92	501.86	524.89	564.03	586.01
614.09	653.34	741.95	765.85	796.02	810.17
838.57	860.76	868.28	897.32	969.72	979.43
990.73	1033.58	1042.12	1115.96	1130.82	1165.00
1187.08	1191.69	1203.80	1233.76	1252.26	1304.74
1309.42	1332.30	1338.51	1354.52	1380.75	1454.13
1469.06	1484.31	1564.63	1650.84	1666.72	1692.12
1878.41	2962.20	3038.96	3103.47	3160.85	3171.00
3196.46	3207.79	3552.05	3640.47	3851.92	3909.69

- Conformer (32); ZPE = 42897.180 cm^{-1}:

24.74	51.98	78.60	86.01	169.25	233.28
285.34	315.72	318.13	331.39	369.07	379.94
420.78	427.34	512.65	530.30	565.91	587.43
653.57	739.29	741.63	755.30	797.88	826.75
834.35	861.30	878.67	882.82	948.42	964.79
999.10	1034.62	1083.21	1114.24	1136.71	1174.35
1188.25	1190.30	1201.47	1230.59	1236.03	1308.43
1316.98	1336.38	1356.18	1358.78	1389.22	1428.75
1467.62	1486.30	1563.25	1657.01	1664.76	1691.70
1893.13	3028.65	3037.06	3091.09	3154.10	3174.29
3195.77	3212.04	3540.43	3633.18	3738.68	3909.13

- Conformer (33); ZPE = 42809.992 cm^{-1}:

37.05	46.36	81.82	87.83	161.46	233.93
277.13	286.73	330.07	341.08	347.18	361.56
421.53	427.93	501.30	524.74	563.57	585.46
613.48	653.16	741.36	765.78	795.92	810.01
832.11	865.02	868.29	896.10	964.52	976.16
996.72	1033.61	1041.44	1115.56	1130.56	1165.63
1185.70	1191.36	1205.29	1233.58	1252.04	1304.59
1309.14	1331.54	1337.49	1355.30	1379.71	1454.02
1468.87	1484.68	1563.29	1650.61	1666.20	1690.97
1879.44	2961.98	3038.83	3103.92	3157.55	3175.47
3190.24	3212.19	3551.88	3640.42	3851.04	3910.48

- Conformer (34); ZPE = 42767.146 cm^{-1}:

19.79	34.94	56.66	68.76	163.60	188.38
245.48	277.46	297.87	348.75	361.44	389.93
421.70	427.15	490.05	528.76	561.82	598.50
640.49	653.12	734.08	760.07	808.38	830.32
839.24	857.54	876.37	893.65	932.84	971.80
989.05	997.47	1034.08	1110.98	1139.89	1154.03
1187.49	1192.85	1196.07	1237.15	1280.48	1295.13
1307.70	1343.95	1355.69	1358.26	1378.53	1395.76
1474.54	1484.67	1564.96	1660.21	1666.65	1692.54
1866.24	3029.73	3076.92	3090.29	3169.66	3171.58
3197.26	3211.03	3547.47	3636.18	3848.75	3912.09

- Conformer (35); ZPE = 42774.443 cm^{-1}:

23.11	35.34	57.90	69.12	164.28	188.56
245.06	277.29	297.79	347.70	362.50	390.89
422.09	427.37	490.13	528.86	561.56	599.75
640.64	653.08	733.52	759.83	807.93	830.55
839.87	854.64	875.69	894.49	933.47	972.68
988.29	996.35	1033.90	1111.21	1140.17	1155.33
1187.26	1192.80	1198.03	1237.53	1280.47	1295.17
1308.39	1343.94	1355.22	1359.40	1379.35	1395.75
1475.16	1484.78	1564.73	1660.36	1666.84	1691.87
1865.92	3029.32	3076.10	3090.14	3165.16	3180.91
3192.08	3212.87	3548.02	3636.75	3847.92	3915.73

- Conformer (36); ZPE = 42829.970 cm^{-1}:

45.49	53.74	78.07	88.52	170.34	212.53
311.02	332.19	363.58	374.74	390.30	422.02
427.11	431.90	482.03	512.53	538.58	575.03
626.52	649.97	657.72	739.23	744.77	794.17
846.40	848.61	862.17	886.30	937.43	982.06
993.49	998.72	1034.09	1117.37	1120.12	1154.69
1190.29	1192.08	1199.85	1234.63	1253.50	1299.63
1310.79	1339.07	1351.57	1359.34	1375.21	1375.77
1472.99	1484.43	1563.48	1631.84	1663.69	1690.37
1882.15	3059.05	3068.24	3121.90	3167.26	3169.45
3181.77	3210.80	3570.33	3685.02	3843.06	3908.86

- Conformer (37); ZPE = 42764.928 cm^{-1}:

32.86	40.19	64.09	90.69	165.76	237.76
263.49	309.62	330.50	336.68	340.80	380.39
421.43	426.42	489.32	522.12	553.52	586.30
634.99	653.90	733.24	748.71	796.47	811.06
835.81	854.73	862.04	886.50	954.52	973.33
989.71	1032.98	1053.60	1117.50	1142.56	1179.15
1185.69	1194.57	1201.12	1230.73	1251.36	1301.08
1308.55	1332.70	1345.91	1354.23	1375.97	1445.83
1475.14	1484.38	1564.90	1636.10	1665.20	1695.03
1859.29	2943.06	3042.66	3095.64	3160.54	3168.07
3185.07	3208.67	3558.33	3653.52	3843.64	3910.17

- Conformer (38); ZPE = 42767.472 cm^{-1}:

31.40	51.32	64.04	84.62	149.07	235.05
241.94	273.93	294.33	334.85	339.37	385.33
422.43	427.72	495.85	537.81	559.96	601.61
606.86	653.22	743.39	773.38	801.54	814.56
839.13	845.91	866.19	877.41	956.07	973.00
992.81	1034.84	1058.31	1095.15	1120.54	1158.34
1171.48	1190.04	1197.05	1236.18	1253.52	1309.44
1318.13	1332.47	1355.01	1359.57	1363.50	1424.81
1473.39	1486.84	1565.60	1649.60	1665.65	1693.62
1887.80	3035.50	3076.83	3094.56	3162.98	3167.44
3179.38	3207.56	3542.30	3633.76	3848.87	3910.79

- Conformer (39); ZPE = 42779.913 cm^{-1}:

33.34	50.91	64.51	85.79	148.26	236.93
245.17	273.13	294.95	334.79	348.58	385.51
422.27	428.12	496.34	537.80	559.44	597.04
604.95	653.23	742.98	772.65	801.98	814.18
836.89	847.91	866.52	877.95	956.96	971.49
992.62	1035.25	1058.28	1095.66	1121.71	1159.45
1171.30	1189.85	1198.26	1236.21	1253.41	1309.98
1318.73	1332.69	1356.03	1359.36	1364.71	1425.62
1472.71	1487.51	1565.76	1649.89	1666.89	1694.08
1887.54	3035.37	3075.43	3094.11	3163.13	3169.86
3179.37	3209.91	3542.25	3633.54	3849.00	3913.82

- Conformer (40); ZPE = 42755.576 cm^{-1}:

32.18	39.60	63.99	90.75	165.82	237.14
263.18	309.61	324.90	333.06	340.33	380.20
421.30	425.96	489.27	522.01	553.36	586.19
634.46	653.91	732.71	748.12	796.60	811.16
833.55	854.02	863.46	886.34	955.80	969.39
992.52	1033.32	1052.99	1117.54	1142.44	1179.33
1185.90	1195.47	1200.98	1230.49	1251.88	1301.26
1307.08	1331.99	1346.44	1354.58	1374.34	1446.20
1475.20	1485.09	1563.89	1635.89	1667.20	1692.86
1859.08	2942.32	3042.81	3095.86	3157.58	3175.64
3178.64	3211.20	3558.40	3653.82	3843.89	3908.69

- Conformer (41); ZPE = 42779.648 cm^{-1}:

26.59	46.34	67.02	96.96	167.79	183.82
253.24	293.87	308.62	319.77	355.56	391.63
421.74	427.86	469.75	529.27	554.79	568.16
651.60	659.85	736.61	752.56	796.71	818.50
844.08	865.50	877.31	916.03	972.20	984.08
996.09	1031.16	1033.83	1114.05	1146.94	1176.34
1188.83	1192.91	1201.69	1230.19	1245.71	1304.88
1313.56	1323.71	1353.20	1359.97	1370.92	1413.87
1478.79	1484.26	1563.83	1661.08	1671.93	1689.45
1882.61	3020.35	3043.04	3096.87	3164.20	3171.04
3186.39	3211.43	3533.89	3610.38	3824.08	3909.98

- Conformer (42); ZPE = 42787.271 cm^{-1}:

42.54	53.56	76.82	86.28	170.22	210.91
308.85	311.66	334.87	373.91	389.09	421.37
425.80	430.97	486.44	520.91	540.02	573.02
618.79	648.88	659.08	738.70	744.52	793.59
836.95	852.59	864.86	886.90	937.41	971.92
993.50	1006.32	1033.91	1117.33	1121.71	1155.89
1186.58	1190.59	1196.93	1234.49	1252.80	1300.02
1309.98	1338.97	1352.47	1357.25	1373.83	1375.40
1472.93	1483.39	1563.16	1631.56	1661.56	1689.55
1882.54	3058.80	3067.66	3121.00	3164.76	3171.13
3180.19	3211.97	3568.59	3683.12	3844.80	3908.48

- Conformer (43); ZPE = 42787.609 cm^{-1}:

29.76	44.55	67.96	96.85	168.53	193.03
253.48	295.30	307.08	321.21	349.92	390.47
421.99	427.88	470.08	529.06	547.17	567.45
651.71	658.64	735.27	751.28	797.13	818.22
844.05	865.96	877.56	921.61	971.66	985.88
995.95	1031.25	1033.84	1116.09	1149.31	1177.87
1189.38	1195.28	1203.99	1230.86	1243.97	1304.29
1313.83	1325.74	1355.56	1358.89	1371.79	1412.36
1479.29	1485.51	1563.55	1661.09	1671.79	1689.43
1884.42	3018.87	3042.57	3095.54	3162.69	3178.40
3180.74	3210.69	3534.39	3609.82	3825.37	3910.11

- Conformer (44); ZPE = 43018.977 cm^{-1}:

23.83	36.66	88.09	100.01	172.90	192.52
304.33	320.65	333.00	346.80	356.49	388.17
424.87	430.54	498.90	541.10	558.72	652.00
669.10	724.60	768.78	783.26	806.12	836.53
848.80	859.86	872.31	920.14	967.29	981.88
1005.64	1022.29	1035.03	1118.31	1130.70	1180.92
1192.69	1199.10	1224.07	1237.93	1269.28	1309.72
1317.77	1341.05	1363.98	1366.60	1385.33	1425.42
1474.91	1486.55	1566.14	1657.98	1664.84	1692.78
1887.89	3025.44	3045.43	3072.38	3166.34	3168.75
3186.28	3208.44	3550.80	3638.16	3702.28	3908.45

- Conformer (45); ZPE = 43019.027 cm^{-1}:

23.85	36.67	88.10	100.02	172.90	192.52
304.35	320.65	333.04	346.81	356.52	388.17
424.86	430.54	498.91	541.10	558.72	652.00
669.10	724.60	768.78	783.32	806.14	836.54
848.80	859.87	872.32	920.16	967.30	981.89
1005.64	1022.29	1035.03	1118.30	1130.71	1180.93
1192.69	1199.11	1224.07	1237.93	1269.28	1309.73
1317.78	1341.05	1363.99	1366.60	1385.33	1425.44
1474.91	1486.56	1566.15	1657.99	1664.84	1692.79
1887.89	3025.44	3045.43	3072.37	3166.34	3168.75
3186.28	3208.43	3550.73	3638.12	3702.14	3908.45

- Conformer (46); ZPE = 42986.575 cm^{-1}:

22.73	35.32	84.01	98.18	173.27	192.96
300.03	321.03	324.23	332.38	347.95	388.96
425.14	429.33	498.38	541.68	557.71	651.62
669.42	723.30	767.50	783.91	806.92	835.03
839.63	867.87	874.72	921.08	960.76	975.22
1005.94	1026.33	1034.67	1117.35	1132.08	1182.12
1187.96	1195.98	1221.60	1238.82	1269.02	1309.98
1316.94	1342.29	1362.86	1365.37	1386.47	1423.89
1476.16	1485.17	1566.21	1656.70	1662.81	1693.36
1889.24	3026.52	3046.69	3073.31	3162.36	3178.44
3179.46	3208.37	3550.20	3637.32	3701.92	3908.94

- Conformer (47); ZPE = 42787.969 cm^{-1}:

33.19	48.90	67.58	99.83	165.99	210.64
251.58	311.61	318.30	329.77	351.48	396.08
422.21	428.25	478.80	525.38	532.44	566.25
651.29	662.24	739.94	753.22	797.17	818.33
839.98	845.98	866.90	878.07	967.87	988.85
995.92	1031.35	1037.34	1121.26	1168.25	1181.89
1188.82	1192.41	1197.02	1234.33	1248.93	1310.03
1314.04	1325.83	1339.96	1353.48	1363.31	1441.36
1475.92	1484.61	1564.08	1641.72	1660.67	1690.32
1888.10	2987.56	3031.67	3088.74	3165.12	3170.13
3184.71	3209.31	3549.09	3644.14	3833.01	3913.35

- Conformer (48); ZPE = 42800.791 cm^{-1}:

41.67	60.18	72.91	90.98	166.96	209.64
229.40	289.42	324.86	336.48	378.60	420.49
424.45	430.68	477.88	511.23	518.60	576.45
634.04	653.00	738.69	743.69	788.24	841.12
843.81	862.41	885.53	908.71	943.67	977.60
995.01	1010.07	1034.57	1125.01	1140.37	1157.11
1179.29	1193.50	1197.59	1237.37	1243.73	1287.43
1312.60	1334.84	1352.31	1360.52	1372.84	1409.46
1468.55	1487.65	1565.25	1663.98	1668.85	1692.56
1898.70	3011.94	3040.86	3105.03	3163.70	3176.48
3181.44	3209.64	3531.78	3612.08	3886.93	3911.14

- Conformer (49); ZPE = 42782.383 cm^{-1}:

32.22	46.56	67.87	94.77	167.42	180.76
251.63	313.01	317.77	334.28	364.06	395.99
422.32	428.56	479.93	531.73	539.06	565.99
651.40	661.66	737.37	750.93	798.35	816.67
838.59	844.90	867.14	878.55	968.60	985.89
998.71	1032.10	1035.98	1122.82	1171.24	1184.71
1190.24	1196.90	1199.99	1235.10	1247.70	1310.81
1314.66	1326.20	1338.06	1355.85	1363.34	1438.04
1476.07	1485.88	1563.50	1643.88	1661.76	1689.00
1885.90	2980.68	3030.99	3088.20	3162.89	3175.97
3179.74	3212.08	3549.05	3645.33	3826.23	3911.19

- Conformer (50); ZPE = 42773.163 cm^{-1}:

40.12	58.82	71.33	90.16	165.43	208.02
218.07	289.77	320.23	334.35	377.76	420.92
423.33	429.93	476.68	501.25	517.90	576.88
633.48	652.84	738.21	743.72	787.89	833.90
850.59	862.90	884.75	909.00	942.71	973.93
997.86	1010.94	1034.71	1125.39	1140.14	1157.14
1179.43	1191.74	1197.28	1237.49	1243.32	1286.26
1311.81	1335.25	1351.90	1359.40	1373.87	1409.61
1467.61	1486.97	1565.10	1663.88	1666.91	1692.11
1898.90	3013.52	3040.24	3104.35	3165.15	3167.72
3183.82	3213.50	3531.31	3610.79	3887.82	3908.22

- Conformer (51); ZPE = 42746.149 cm^{-1}:

21.18	33.67	56.39	93.97	173.62	205.95
221.73	281.63	309.94	334.03	358.01	401.13
422.76	426.38	498.06	510.01	547.97	587.91
641.12	654.25	718.96	756.75	794.25	830.45
837.21	860.27	869.65	895.94	947.56	978.89
1001.51	1031.37	1036.40	1118.36	1134.89	1183.35
1188.47	1190.13	1218.21	1239.35	1245.28	1293.57
1307.24	1333.27	1342.76	1353.77	1375.28	1440.95
1475.19	1484.41	1563.62	1634.69	1663.12	1689.99
1866.13	2947.31	3056.28	3108.49	3165.96	3181.93
3182.81	3207.88	3555.66	3648.93	3848.32	3907.80

- Conformer (52); ZPE = 42780.682 cm^{-1}:

22.77	37.57	56.18	95.18	174.59	205.81
229.31	309.60	325.93	336.03	357.93	399.74
422.56	426.39	498.00	509.74	548.04	589.50
641.07	654.52	719.50	756.22	794.50	829.39
840.66	856.59	867.82	896.03	949.79	983.61
994.49	1032.26	1035.65	1117.90	1135.15	1185.07
1189.27	1193.35	1218.47	1239.91	1245.06	1294.12
1307.40	1331.52	1342.58	1356.11	1374.40	1440.75
1475.64	1485.04	1562.60	1634.43	1664.40	1688.91
1866.11	2946.24	3055.49	3108.62	3166.45	3173.21
3190.86	3210.09	3555.77	3649.14	3848.18	3912.14

- Conformer (53); ZPE = 42777.824 cm^{-1}:

20.43	42.74	53.29	120.99	136.51	204.20
248.69	264.39	297.16	324.31	334.26	357.51
421.54	422.09	514.92	543.65	552.30	596.07
601.20	655.15	728.41	776.39	792.15	809.38
834.62	851.22	869.94	891.76	961.56	989.78
1001.77	1025.60	1037.03	1122.85	1127.71	1167.89
1193.55	1196.67	1222.33	1240.82	1264.04	1308.21
1311.74	1343.66	1359.98	1379.19	1404.85	1422.76
1470.00	1486.64	1567.95	1653.07	1665.31	1695.28
1863.70	3013.66	3016.53	3083.19	3166.06	3182.57
3185.85	3210.60	3547.70	3634.68	3851.97	3913.65

- Conformer (54); ZPE = 42744.068 cm^{-1}:

39.75	50.55	64.04	75.66	166.92	186.30
278.65	294.23	326.19	340.99	359.15	402.47
423.72	427.34	487.63	510.28	521.16	562.85
652.62	673.39	718.03	751.94	808.69	831.93
840.68	851.87	868.98	891.08	924.44	972.29
991.47	1020.42	1033.47	1118.21	1148.32	1161.34
1185.75	1190.50	1206.64	1236.64	1249.97	1304.78
1312.27	1327.90	1336.00	1355.45	1356.61	1437.42
1470.95	1484.94	1564.45	1633.20	1664.52	1691.18
1899.63	2984.73	3026.34	3083.38	3164.00	3167.45
3182.71	3212.33	3541.29	3635.71	3894.28	3910.06

- Conformer (55); ZPE = 42745.424 cm^{-1}:

39.35	50.47	64.21	75.82	167.02	186.55
275.33	293.77	326.48	350.31	361.03	402.63
423.07	427.65	487.50	509.79	520.76	562.58
652.59	672.93	717.90	751.21	808.48	831.75
836.50	853.72	869.67	892.00	924.78	969.72
992.91	1020.34	1032.99	1118.63	1148.30	1160.82
1186.30	1191.77	1205.38	1237.10	1249.85	1304.15
1311.42	1329.07	1334.70	1354.71	1356.60	1436.85
1470.80	1485.12	1563.90	1633.23	1664.55	1691.63
1900.09	2982.96	3026.51	3083.66	3161.84	3172.41
3180.90	3211.44	3541.24	3635.52	3895.10	3912.51

- Conformer (56); ZPE = 42750.652 cm^{-1}:

36.68	62.08	77.79	101.28	165.10	193.15
213.67	311.45	325.04	336.89	368.55	421.32
425.15	430.67	481.37	506.19	523.53	581.96
642.81	653.68	736.20	743.89	790.42	838.74
841.38	857.80	865.90	896.09	946.55	976.93
988.10	991.38	1034.07	1119.59	1143.88	1166.14
1190.56	1193.59	1208.38	1235.94	1253.24	1265.65
1311.54	1318.69	1337.97	1359.58	1377.74	1437.47
1475.56	1486.67	1565.65	1641.30	1665.12	1692.93
1901.89	2980.95	3027.87	3078.54	3163.03	3165.81
3181.26	3209.04	3543.10	3636.04	3890.02	3910.76

- Conformer (57); ZPE = 42754.086 cm^{-1}:

23.89	41.69	62.09	135.07	158.31	248.01
274.30	299.62	323.91	341.04	370.96	412.17
423.86	426.26	456.55	489.56	523.69	567.15
616.77	655.62	730.42	739.68	784.02	843.36
848.39	855.83	862.95	917.92	944.00	982.48
1007.13	1023.04	1034.86	1120.27	1134.88	1192.05
1194.88	1200.63	1217.26	1228.66	1245.09	1308.24
1310.43	1320.19	1351.43	1362.35	1378.17	1424.60
1472.92	1490.50	1561.88	1633.22	1658.68	1690.00
1880.95	2939.26	3027.35	3065.59	3158.92	3169.76
3192.16	3208.92	3545.55	3638.07	3853.08	3907.63

- Conformer (58); ZPE = 42731.175 cm^{-1}:

25.52	47.51	71.42	75.09	176.96	189.02
195.72	272.76	305.37	354.07	369.65	399.38
422.22	429.24	485.66	501.29	512.64	556.79
652.52	675.93	714.79	753.21	804.97	833.99
853.87	866.44	885.95	893.05	947.97	980.49
986.26	1012.29	1033.94	1115.42	1144.54	1159.93
1179.16	1188.57	1194.91	1237.48	1261.37	1298.19
1309.03	1333.54	1343.93	1356.36	1360.60	1420.17
1472.65	1483.98	1564.35	1665.40	1668.74	1691.59
1893.37	3022.22	3033.15	3079.33	3163.21	3171.45
3205.40	3211.80	3529.68	3617.13	3888.27	3911.45

- Conformer (59); ZPE = 42736.009 cm^{-1}:

24.61	47.11	71.39	76.05	180.97	191.26
203.97	271.93	304.50	348.64	370.11	399.55
422.57	429.54	482.54	497.91	509.73	556.79
652.30	675.66	714.26	753.15	805.13	829.45
857.79	866.65	884.25	893.77	947.19	974.24
995.14	1011.34	1033.89	1116.69	1145.22	1159.54
1179.40	1188.30	1195.06	1237.73	1261.23	1297.83
1309.75	1333.11	1343.55	1355.58	1364.08	1420.48
1472.62	1483.97	1564.63	1664.34	1669.58	1691.66
1894.13	3022.09	3032.67	3078.49	3160.66	3180.13
3199.98	3213.96	3530.79	3618.49	3887.28	3915.66

- Conformer (60); ZPE = 42719.045 cm^{-1}:

33.72	61.58	77.29	98.57	163.73	183.01
209.79	310.70	313.91	333.10	368.42	422.55
423.11	429.92	480.39	500.91	523.11	582.22
642.66	653.47	735.55	743.57	789.96	834.76
836.34	863.38	865.84	895.63	944.24	967.59
989.24	998.90	1034.09	1120.04	1144.31	1166.90
1186.52	1192.27	1209.18	1236.48	1252.73	1266.34
1311.44	1318.97	1338.92	1358.67	1377.95	1437.41
1475.39	1485.70	1565.73	1641.00	1663.95	1692.31
1903.15	2980.96	3027.86	3078.16	3161.91	3164.41
3178.58	3213.80	3542.79	3636.20	3887.83	3909.00

- Conformer (61); ZPE = 42742.009 cm^{-1}:

23.50	43.11	60.92	134.06	160.02	250.01
274.68	298.65	309.52	340.18	371.31	413.98
423.22	424.68	460.44	488.67	524.13	567.68
619.13	655.48	730.15	740.11	783.57	837.19
846.60	862.45	869.66	918.46	946.55	968.82
1014.55	1025.94	1033.94	1120.34	1133.55	1185.59
1190.51	1200.66	1219.18	1229.41	1245.00	1308.24
1311.01	1320.57	1351.42	1357.92	1380.56	1425.37
1471.70	1487.75	1561.23	1634.99	1657.71	1686.56
1880.91	2941.51	3027.68	3066.69	3157.54	3176.54
3189.53	3211.14	3545.24	3637.13	3842.97	3906.46

- Conformer (62); ZPE = 42655.990 cm^{-1}:

32.16	38.06	78.37	87.98	160.85	180.65
233.42	265.30	321.03	332.61	342.59	366.26
421.91	428.08	490.91	502.10	529.53	571.84
622.11	653.14	744.02	756.96	786.57	808.95
839.49	849.68	862.26	883.26	966.53	976.97
992.25	1033.64	1042.09	1112.75	1126.12	1156.08
1188.92	1190.23	1208.26	1233.06	1240.52	1295.85
1308.76	1312.55	1337.14	1355.08	1378.67	1444.15
1468.65	1484.67	1563.91	1651.94	1663.82	1690.83
1909.14	2922.49	3041.93	3109.43	3159.53	3170.97
3191.70	3205.51	3549.14	3639.61	3890.78	3908.17

- Conformer (63); ZPE = 42769.634 cm^{-1}:

26.26	36.48	57.26	74.95	178.97	195.53
264.38	272.65	297.22	324.08	362.10	372.50
422.57	428.59	492.01	524.90	534.09	561.23
645.05	652.96	745.68	778.93	799.86	836.62
842.33	862.91	876.39	905.30	935.71	974.94
991.67	1015.42	1034.40	1116.61	1144.21	1155.88
1191.99	1195.90	1206.41	1241.01	1282.12	1285.38
1311.14	1343.56	1347.44	1359.13	1372.71	1400.84
1479.54	1487.03	1565.34	1656.90	1665.73	1691.53
1891.38	2985.27	3060.75	3083.00	3167.95	3170.03
3197.32	3211.37	3530.81	3624.13	3885.09	3907.79

- Conformer (64); ZPE = 42738.508 cm^{-1}:

26.41	35.45	56.76	74.89	178.78	194.07
261.28	272.24	293.84	317.13	361.92	371.13
422.72	427.47	490.39	512.73	533.75	561.09
644.97	652.95	744.15	778.15	800.56	833.58
842.23	860.18	875.01	905.05	933.29	972.34
991.85	1013.77	1033.82	1116.39	1143.60	1154.76
1190.83	1193.41	1203.51	1240.86	1281.42	1284.49
1311.51	1341.74	1347.85	1357.11	1372.81	1400.32
1478.61	1486.21	1565.22	1657.02	1664.86	1691.66
1893.25	2983.79	3060.51	3084.67	3165.08	3180.78
3191.74	3212.02	3530.97	3624.75	3883.35	3908.02

- Conformer (65); ZPE = 42626.203 cm^{-1}:

25.75	37.50	78.68	87.56	161.04	186.81
231.49	265.46	309.94	323.77	342.68	365.43
421.93	427.21	477.89	499.88	529.57	571.38
622.11	652.94	743.45	757.01	786.67	809.18
833.06	850.97	866.29	883.32	963.53	968.45
1001.13	1033.15	1042.67	1111.65	1125.99	1155.74
1186.03	1188.30	1208.45	1232.78	1239.92	1295.13
1308.30	1311.69	1335.33	1354.72	1377.70	1443.82
1468.27	1483.70	1562.39	1652.03	1662.04	1689.31
1910.21	2921.62	3042.24	3109.50	3156.48	3174.14
3186.07	3212.12	3549.41	3640.16	3890.51	3908.71

13.5 Cartesian coordinates of Tyr conformers

Each line starting by an asterisk contains the relative energy of the corresponding conformer and the dihedral angle for each of the target torsions (separated by underscores). Cartesian coordinates are given in Å.

```
24
* E =  +0.000  kcal/mol  ;  (1)  357_015_091_288_000
H    +0.00000    +0.00000    +0.00000
O    +0.00000    +0.00000    +0.97265
C    +1.27035    +0.00000    +1.35431
C    +2.28395    -0.08338    +0.19963
N    +1.61957    +0.21509    -1.06496
H    +1.67943    +1.20675    -1.27708
```

O	+1.60382	+0.02638	+2.50414
C	+3.52027	+0.77809	+0.51405
H	+2.58956	−1.13712	+0.17936
H	+2.02470	−0.29571	−1.83698
C	+3.22043	+2.25236	+0.42402
H	+3.86530	+0.51557	+1.51820
H	+4.31318	+0.51745	−0.19523
C	+2.62579	+2.93275	+1.48572
C	+2.30500	+4.27735	+1.38043
C	+2.57350	+4.96921	+0.20419
C	+3.17001	+4.31037	−0.86364
C	+3.48672	+2.96616	−0.74407
H	+2.41334	+2.39861	+2.40804
H	+1.84505	+4.79508	+2.21894
O	+2.27942	+6.28522	+0.04963
H	+3.38436	+4.86515	−1.77074
H	+3.96984	+2.45936	−1.57748
H	+1.87696	+6.63179	+0.84715

24

∗ E = +0.381 kcal/mol ; (2) 356_015_092_288_180

H	+0.00000	+0.00000	+0.00000
O	+0.00000	+0.00000	+0.97247
C	+1.27009	+0.00000	+1.35495
C	+2.28463	−0.08614	+0.20122
N	+1.62257	+0.20992	−1.06516
H	+1.68733	+1.20051	−1.28067
O	+1.60336	+0.02678	+2.50463
C	+3.52246	+0.77388	+0.51400
H	+2.58904	−1.14025	+0.18415
H	+2.02616	−0.30578	−1.83470
C	+3.22997	+2.24874	+0.41000
H	+3.86170	+0.51867	+1.52207
H	+4.31775	+0.50404	−0.18920
C	+2.62086	+2.94041	+1.46059
C	+2.30866	+4.28346	+1.34800
C	+2.60100	+4.96840	+0.17254
C	+3.20963	+4.30096	−0.88231
C	+3.51820	+2.95229	−0.75527
H	+2.39280	+2.41055	+2.38181
H	+1.84000	+4.82251	+2.16441

O	+2.27617	+6.28499	+0.11317
H	+3.44995	+4.83585	−1.79802
H	+4.01097	+2.44055	−1.57975
H	+2.52800	+6.65297	−0.73477

24

∗ E = +1.076 kcal/mol ; (3) 181_179_303_296_180

H	+0.00000	+0.00000	+0.00000
O	+0.00000	+0.00000	+0.96400
C	+1.28111	+0.00000	+1.37422
C	+1.40166	−0.03453	+2.89418
N	+2.77417	−0.05220	+3.34538
H	+3.26607	−0.84224	+2.94483
O	+2.20258	+0.01011	+0.61049
C	+0.63961	+1.14974	+3.52169
H	+0.90558	−0.95821	+3.22024
H	+3.25095	+0.77715	+3.00371
C	+1.22826	+2.47913	+3.12563
H	−0.41062	+1.09625	+3.22092
H	+0.69138	+1.02270	+4.60683
C	+0.79161	+3.14403	+1.97977
C	+1.35425	+4.34484	+1.58256
C	+2.37797	+4.90717	+2.33637
C	+2.82621	+4.26287	+3.48321
C	+2.25154	+3.05997	+3.86897
H	−0.01629	+2.71250	+1.39285
H	+1.01167	+4.86448	+0.69420
O	+2.89936	+6.08722	+1.91178
H	+3.62233	+4.70554	+4.07727
H	+2.60368	+2.55947	+4.76800
H	+3.59339	+6.37627	+2.50553

24

∗ E = +1.085 kcal/mol ; (4) 182_179_303_296_359

H	+0.00000	+0.00000	+0.00000
O	+0.00000	+0.00000	+0.96394
C	+1.28182	+0.00000	+1.37350
C	+1.40299	−0.04248	+2.89316
N	+2.77462	−0.06685	+3.34527
H	+3.26661	−0.85370	+2.93879
O	+2.20201	+0.01554	+0.60832
C	+0.64444	+1.14163	+3.52550

H	+0.90327	−0.96598	+3.21426
H	+3.25404	+0.76572	+3.01543
C	+1.24297	+2.46985	+3.14057
H	−0.40481	+1.09537	+3.22012
H	+0.69113	+1.00780	+4.61004
C	+0.82321	+3.13879	+1.99469
C	+1.40195	+4.33966	+1.61048
C	+2.42071	+4.89257	+2.37691
C	+2.85201	+4.24169	+3.52717
C	+2.26408	+3.04400	+3.89798
H	+0.01942	+2.71625	+1.39579
H	+1.05920	+4.85219	+0.71474
O	+3.02303	+6.06569	+2.05190
H	+3.64377	+4.69011	+4.11772
H	+2.60179	+2.53626	+4.79859
H	+2.64977	+6.41743	+1.24275

24

∗ E = +1.368 kcal/mol ; (5) 004_342_154_177_179

H	+0.00000	+0.00000	+0.00000
O	+0.00000	+0.00000	+0.97197
C	+1.27339	+0.00000	+1.35849
C	+2.28435	+0.08571	+0.21172
N	+1.65373	−0.27364	−1.05266
H	+2.12933	+0.16865	−1.83160
O	+1.60775	−0.01700	+2.50610
C	+2.79094	+1.53805	+0.15589
H	+3.12633	−0.56459	+0.48360
H	+1.67519	−1.27317	−1.21258
C	+3.85808	+1.71770	−0.89070
H	+1.94021	+2.19644	−0.05923
H	+3.16546	+1.79695	+1.15093
C	+3.58205	+2.33278	−2.10969
C	+4.55125	+2.45931	−3.09349
C	+5.82861	+1.96458	−2.86510
C	+6.12532	+1.34848	−1.65272
C	+5.14504	+1.22857	−0.68110
H	+2.58679	+2.73444	−2.28903
H	+4.33729	+2.94341	−4.04027
O	+6.74836	+2.11079	−3.85175
H	+7.12748	+0.96772	−1.47039

H	+5.38766	+0.75113	+0.26589
H	+7.59302	+1.75042	−3.57848

24

∗ E = +1.383 kcal/mol ; (6) 004_342_154_177_001

H	+0.00000	+0.00000	+0.00000
O	+0.00000	+0.00000	+0.97194
C	+1.27361	+0.00000	+1.35837
C	+2.28415	+0.08581	+0.21128
N	+1.65253	−0.27071	−1.05360
H	+2.12859	+0.17396	−1.83093
O	+1.60790	−0.01680	+2.50590
C	+2.79213	+1.53769	+0.15729
H	+3.12526	−0.56628	+0.48134
H	+1.67525	−1.26986	−1.21589
C	+3.85131	+1.72012	−0.89688
H	+1.94081	+2.19826	−0.04875
H	+3.17447	+1.79215	+1.15059
C	+3.57671	+2.37116	−2.09347
C	+4.54436	+2.50070	−3.08315
C	+5.81173	+1.97236	−2.88045
C	+6.10628	+1.31751	−1.68699
C	+5.13243	+1.19629	−0.71371
H	+2.58934	+2.79845	−2.25492
H	+4.31376	+3.01816	−4.01119
O	+6.80091	+2.06683	−3.80417
H	+7.10431	+0.91775	−1.54256
H	+5.37148	+0.68981	+0.21913
H	+6.49546	+2.54881	−4.57386

24

∗ E = +1.691 kcal/mol ; (7) 356_019_087_173_179

H	+0.00000	+0.00000	+0.00000
O	+0.00000	+0.00000	+0.97141
C	+1.27212	+0.00000	+1.36069
C	+2.28497	−0.08858	+0.21116
N	+1.64995	+0.29653	−1.04615
H	+1.69937	+1.30190	−1.17835
O	+1.59885	+0.03364	+2.51022
C	+3.56558	+0.68226	+0.55602
H	+2.53429	−1.15601	+0.13384
H	+2.12236	−0.12528	−1.83571

C	+4.62416	+0.47142	−0.49250
H	+3.32043	+1.74879	+0.64162
H	+3.90814	+0.35680	+1.54243
C	+4.83860	+1.40111	−1.50742
C	+5.77290	+1.18187	−2.50756
C	+6.51638	+0.00827	−2.50662
C	+6.31923	−0.93339	−1.50207
C	+5.38053	−0.69713	−0.50929
H	+4.27053	+2.32913	−1.50738
H	+5.94401	+1.91055	−3.29264
O	+7.42192	−0.16660	−3.50277
H	+6.90641	−1.84865	−1.49516
H	+5.23938	−1.43598	+0.27690
H	+7.88633	−0.99700	−3.39010

24

∗ E = +1.729 kcal/mol ; (8) 179_009_303_292_000

H	+0.00000	+0.00000	+0.00000
O	+0.00000	+0.00000	+0.96411
C	+1.27763	+0.00000	+1.36804
C	+1.43445	+0.02013	+2.88919
N	+0.22354	−0.17444	+3.65659
H	−0.47661	+0.51574	+3.41640
O	+2.19583	+0.00425	+0.59544
C	+2.50335	−1.01322	+3.28779
H	+1.83374	+1.01741	+3.11785
H	−0.17599	−1.08580	+3.45808
C	+2.04107	−2.42706	+3.04294
H	+3.41258	−0.80723	+2.71658
H	+2.71903	−0.86576	+4.34971
C	+2.25633	−3.04958	+1.81576
C	+1.79606	−4.33639	+1.57491
C	+1.10708	−5.02390	+2.56687
C	+0.88518	−4.42084	+3.79954
C	+1.35148	−3.13653	+4.02681
H	+2.79874	−2.51781	+1.03776
H	+1.97839	−4.81189	+0.61390
O	+0.63596	−6.28548	+2.38666
H	+0.35293	−4.97394	+4.56606
H	+1.18086	−2.66735	+4.99321
H	+0.85627	−6.59892	+1.50857

24
∗ E = +1.734 kcal/mol ; (9) 356_019_087_174_001

H	+0.00000	+0.00000	+0.00000
O	+0.00000	+0.00000	+0.97141
C	+1.27230	+0.00000	+1.36041
C	+2.28471	−0.08792	+0.21060
N	+1.64968	+0.29922	−1.04622
H	+1.70360	+1.30435	−1.17869
O	+1.59942	+0.03496	+2.50973
C	+3.56456	+0.68366	+0.55643
H	+2.53380	−1.15522	+0.13162
H	+2.12034	−0.12491	−1.83557
C	+4.61641	+0.49135	−0.50234
H	+3.31612	+1.74801	+0.65771
H	+3.91532	+0.34710	+1.53634
C	+4.86071	+1.45963	−1.46843
C	+5.79419	+1.25521	−2.47727
C	+6.50050	+0.06106	−2.53118
C	+6.27066	−0.92192	−1.57288
C	+5.33829	−0.70085	−0.57533
H	+4.32157	+2.40367	−1.42758
H	+5.97697	+2.02759	−3.22050
O	+7.42658	−0.19937	−3.48899
H	+6.83726	−1.84554	−1.62420
H	+5.17114	−1.47028	+0.17592
H	+7.51530	+0.54909	−4.08048

24
∗ E = +1.772 kcal/mol ; (10) 180_004_302_292_179

H	+0.00000	+0.00000	+0.00000
O	+0.00000	+0.00000	+0.96420
C	+1.27706	+0.00000	+1.36858
C	+1.43591	−0.00704	+2.88931
N	+0.21496	−0.11043	+3.65832
H	−0.42286	+0.64291	+3.43452
O	+2.19700	+0.01205	+0.59826
C	+2.41171	−1.13712	+3.26802
H	+1.92195	+0.94728	+3.12995
H	−0.26302	−0.97864	+3.44051
C	+1.82743	−2.49909	+2.99291
H	+3.33626	−0.99860	+2.70080

H	+2.63697	−1.03004	+4.33275
C	+1.97196	−3.10323	+1.74267
C	+1.40006	−4.33389	+1.46746
C	+0.66533	−4.98836	+2.44973
C	+0.51257	−4.40590	+3.70203
C	+1.09183	−3.17194	+3.96426
H	+2.55221	−2.59731	+0.97435
H	+1.51824	−4.80791	+0.49886
O	+0.12420	−6.19429	+2.13523
H	−0.05556	−4.92034	+4.47362
H	+0.97198	−2.72001	+4.94623
H	−0.34273	−6.55533	+2.88981

24

* E = +2.690 kcal/mol ; (11) 177_159_041_179_000

H	+0.00000	+0.00000	+0.00000
O	+0.00000	+0.00000	+0.96386
C	+1.27538	+0.00000	+1.37744
C	+1.38565	+0.08539	+2.88244
N	+2.67969	−0.41699	+3.27916
H	+3.39371	−0.09650	+2.63449
O	+2.20542	−0.00719	+0.62230
C	+1.09866	+1.55859	+3.28099
H	+0.59411	−0.53983	+3.31548
H	+2.91410	−0.11933	+4.21815
C	+1.16686	+1.73947	+4.77218
H	+1.84021	+2.19876	+2.78779
H	+0.10827	+1.84081	+2.90569
C	+2.29722	+2.26555	+5.38644
C	+2.37736	+2.38468	+6.76862
C	+1.31342	+1.97191	+7.55920
C	+0.17294	+1.44164	+6.96287
C	+0.11004	+1.32926	+5.58614
H	+3.13141	+2.59858	+4.77322
H	+3.26746	+2.80299	+7.23240
O	+1.32886	+2.06520	+8.91350
H	−0.65070	+1.12798	+7.59525
H	−0.78552	+0.91646	+5.12629
H	+2.15632	+2.44473	+9.21210

24

* E = +2.706 kcal/mol ; (12) 176_134_293_055_179

H	+0.00000	+0.00000	+0.00000
O	+0.00000	+0.00000	+0.96455
C	+1.26876	+0.00000	+1.38293
C	+1.40526	+0.10098	+2.89717
N	+2.36540	−0.84276	+3.43636
H	+2.04139	−1.79697	+3.33649
O	+2.20666	−0.04991	+0.63490
C	+1.86991	+1.53240	+3.21931
H	+0.42289	−0.07389	+3.34810
H	+3.23772	−0.76580	+2.92265
C	+0.96690	+2.58580	+2.63521
H	+1.93282	+1.61664	+4.30824
H	+2.88543	+1.64408	+2.81929
C	−0.20368	+2.96798	+3.28814
C	−1.06816	+3.89689	+2.73604
C	−0.77033	+4.46347	+1.50141
C	+0.38986	+4.09415	+0.83265
C	+1.24690	+3.16131	+1.40071
H	−0.43982	+2.53111	+4.25607
H	−1.97616	+4.19971	+3.24638
O	−1.64475	+5.37343	+0.99761
H	+0.62565	+4.54047	−0.13067
H	+2.15240	+2.87205	+0.87142
H	−1.33064	+5.70319	+0.15479

24

∗ E = +2.710 kcal/mol ; (13) 175_135_293_055_000

H	+0.00000	+0.00000	+0.00000
O	+0.00000	+0.00000	+0.96463
C	+1.26876	+0.00000	+1.38332
C	+1.40464	+0.11215	+2.89682
N	+2.37842	−0.81388	+3.44185
H	+2.06895	−1.77332	+3.34564
O	+2.20700	−0.05592	+0.63642
C	+1.84857	+1.55240	+3.21075
H	+0.42498	−0.07360	+3.34938
H	+3.24956	−0.72662	+2.92768
C	+0.92948	+2.58987	+2.62317
H	+1.91225	+1.64258	+4.29913
H	+2.86173	+1.67697	+2.80845
C	−0.23282	+2.97169	+3.28387

C	−1.11217	+3.88509	+2.72160
C	−0.83607	+4.43146	+1.47363
C	+0.32031	+4.06197	+0.79712
C	+1.18857	+3.14914	+1.37297
H	−0.45504	+2.55093	+4.26204
H	−2.01448	+4.17763	+3.25346
O	−1.65905	+5.33125	+0.87399
H	+0.52374	+4.50248	−0.17313
H	+2.09156	+2.85872	+0.83966
H	−2.41458	+5.51389	+1.43393

24

∗ E = +2.712 kcal/mol ; (14) 177_159_041_179_180

H	+0.00000	+0.00000	+0.00000
O	+0.00000	+0.00000	+0.96387
C	+1.27579	+0.00000	+1.37702
C	+1.38643	+0.08543	+2.88203
N	+2.68122	−0.41495	+3.27850
H	+3.39483	−0.09366	+2.63382
O	+2.20527	−0.00758	+0.62134
C	+1.09566	+1.55756	+3.28151
H	+0.59599	−0.54154	+3.31471
H	+2.91603	−0.11756	+4.21749
C	+1.16476	+1.73813	+4.77284
H	+1.83510	+2.20023	+2.78847
H	+0.10440	+1.83725	+2.90668
C	+2.30090	+2.25953	+5.38790
C	+2.38777	+2.37991	+6.76627
C	+1.32266	+1.97256	+7.55924
C	+0.17901	+1.44861	+6.96511
C	+0.10896	+1.33456	+5.58564
H	+3.13465	+2.58761	+4.77121
H	+3.27081	+2.79016	+7.24427
O	+1.44694	+2.11040	+8.90413
H	−0.65712	+1.13344	+7.58486
H	−0.79049	+0.92617	+5.12974
H	+0.65469	+1.80192	+9.34574

24

∗ E = +2.919 kcal/mol ; (15) 179_044_184_177_001

H	+0.00000	+0.00000	+0.00000
O	+0.00000	+0.00000	+0.96367

C	+1.27807	+0.00000	+1.37762
C	+1.38014	+0.01992	+2.89095
N	+0.42546	−0.92002	+3.45791
H	+0.44481	−0.86248	+4.46950
O	+2.20193	+0.02176	+0.61708
C	+2.80238	−0.33122	+3.33040
H	+1.17780	+1.06954	+3.18071
H	−0.51592	−0.71997	+3.14423
C	+2.94730	−0.23644	+4.82633
H	+3.02358	−1.34691	+2.98671
H	+3.50101	+0.34397	+2.82772
C	+2.82400	−1.36093	+5.63534
C	+2.91055	−1.26604	+7.01845
C	+3.12309	−0.02963	+7.61449
C	+3.24805	+1.10724	+6.82282
C	+3.15924	+0.99515	+5.44627
H	+2.65718	−2.33090	+5.17304
H	+2.81669	−2.15628	+7.63599
O	+3.22061	+0.12576	+8.96068
H	+3.42051	+2.06423	+7.30364
H	+3.26638	+1.88714	+4.83238
H	+3.13377	−0.72130	+9.39976

24

∗ E = +2.980 kcal/mol ; (16) 179_044_184_176_179

H	+0.00000	+0.00000	+0.00000
O	+0.00000	+0.00000	+0.96366
C	+1.27805	+0.00000	+1.37760
C	+1.38049	+0.01846	+2.89102
N	+0.42297	−0.91851	+3.45757
H	+0.44051	−0.86155	+4.46920
O	+2.20181	+0.02368	+0.61696
C	+2.80224	−0.33663	+3.32946
H	+1.18184	+1.06876	+3.18115
H	−0.51754	−0.71732	+3.14215
C	+2.95117	−0.23800	+4.82484
H	+3.01870	−1.35430	+2.98852
H	+3.50231	+0.33426	+2.82318
C	+2.79171	−1.35651	+5.64177
C	+2.88026	−1.26322	+7.02163
C	+3.13286	−0.03127	+7.61282

C	+3.29536	+1.09678	+6.81668
C	+3.20342	+0.98605	+5.43696
H	+2.59458	−2.32179	+5.18117
H	+2.76104	−2.13471	+7.65653
O	+3.21498	+0.01476	+8.96822
H	+3.49990	+2.06027	+7.27773
H	+3.34005	+1.87269	+4.82145
H	+3.40442	+0.90752	+9.25946

24

* E = +2.995 kcal/mol ; (17) 178_167_284_166_000

H	+0.00000	+0.00000	+0.00000
O	+0.00000	+0.00000	+0.96399
C	+1.27507	+0.00000	+1.38140
C	+1.38596	+0.05336	+2.89815
N	+2.71768	−0.25667	+3.36957
H	+2.89692	−1.25181	+3.32642
O	+2.20542	−0.01063	+0.62569
C	+0.95929	+1.46116	+3.37214
H	+0.67206	−0.66961	+3.30980
H	+3.39934	+0.18599	+2.76078
C	+0.70774	+1.50675	+4.85490
H	+1.75728	+2.16104	+3.09365
H	+0.05481	+1.76253	+2.83392
C	+1.75323	+1.62298	+5.76534
C	+1.51394	+1.62961	+7.13133
C	+0.21356	+1.51579	+7.60953
C	−0.84347	+1.39840	+6.71597
C	−0.58807	+1.39474	+5.35430
H	+2.77206	+1.69582	+5.39719
H	+2.34108	+1.72315	+7.83133
O	−0.07707	+1.51899	+8.93768
H	−1.85320	+1.31594	+7.10354
H	−1.42045	+1.30739	+4.65934
H	+0.72703	+1.60805	+9.45066

24

* E = +3.023 kcal/mol ; (18) 179_167_041_299_359

H	+0.00000	+0.00000	+0.00000
O	+0.00000	+0.00000	+0.96368
C	+1.28115	+0.00000	+1.37155
C	+1.39023	+0.02916	+2.88108

N +2.75411 −0.28099 +3.24179
H +3.39929 +0.19142 +2.61768
O +2.20302 +0.01729 +0.60984
C +0.85772 +1.39744 +3.39015
H +0.71722 −0.74821 +3.26577
H +2.95658 −0.00217 +4.19274
C +1.64146 +2.56152 +2.84923
H −0.19940 +1.48583 +3.11743
H +0.91264 +1.37896 +4.48475
C +1.29505 +3.15023 +1.63678
C +2.04274 +4.18726 +1.09998
C +3.16288 +4.65433 +1.77730
C +3.52363 +4.08399 +2.99225
C +2.76515 +3.04946 +3.51398
H +0.41598 +2.79351 +1.10383
H +1.75579 +4.63762 +0.15281
O +3.93243 +5.66737 +1.30216
H +4.39535 +4.46580 +3.51269
H +3.04676 +2.61681 +4.47146
H +3.59571 +5.97274 +0.45886
24
∗ E = +3.087 kcal/mol ; (19) 178_167_041_301_180
H +0.00000 +0.00000 +0.00000
O +0.00000 +0.00000 +0.96377
C +1.28062 +0.00000 +1.37179
C +1.38969 +0.03856 +2.88131
N +2.75489 −0.26554 +3.24265
H +3.39809 +0.19850 +2.61043
O +2.20356 +0.01019 +0.61134
C +0.85379 +1.40826 +3.38251
H +0.71950 −0.73844 +3.27168
H +2.95932 +0.02333 +4.19005
C +1.61395 +2.57377 +2.81172
H −0.20864 +1.48349 +3.12724
H +0.92801 +1.40360 +4.47620
C +1.21990 +3.15973 +1.60872
C +1.93677 +4.19752 +1.04005
C +3.07884 +4.67281 +1.67475
C +3.48737 +4.10754 +2.87629
C +2.75588 +3.06765 +3.43302

H +0.32305 +2.79523 +1.11180
H +1.62775 +4.65467 +0.10627
O +3.75062 +5.69376 +1.08268
H +4.37492 +4.48468 +3.37873
H +3.07677 +2.64137 +4.38076
H +4.51840 +5.93295 +1.60331
24
* E = +3.097 kcal/mol ; (20) 178_167_284_166_179
H +0.00000 +0.00000 +0.00000
O +0.00000 +0.00000 +0.96399
C +1.27565 +0.00000 +1.38081
C +1.38725 +0.05213 +2.89770
N +2.71884 −0.25658 +3.36922
H +2.90335 −1.25031 +3.31693
O +2.20518 −0.01051 +0.62427
C +0.95833 +1.45860 +3.37328
H +0.67451 −0.67258 +3.30839
H +3.40067 +0.19589 +2.76795
C +0.70261 +1.50368 +4.85551
H +1.75655 +2.15954 +3.09792
H +0.05520 +1.76025 +2.83296
C +1.74876 +1.60539 +5.77201
C +1.51087 +1.61599 +7.13527
C +0.20725 +1.52067 +7.61003
C −0.84852 +1.41785 +6.71384
C −0.59356 +1.41058 +5.34901
H +2.76862 +1.66503 +5.40427
H +2.32318 +1.69769 +7.84961
O +0.02226 +1.53770 +8.95684
H −1.86928 +1.34843 +7.08234
H −1.42676 +1.33541 +4.65383
H −0.91116 +1.47552 +9.16293
24
* E = +3.424 kcal/mol ; (21) 184_345_058_298_000
H +0.00000 +0.00000 +0.00000
O +0.00000 +0.00000 +0.96390
C +1.27156 +0.00000 +1.37098
C +1.43158 −0.09364 +2.87768
N +0.18539 +0.17521 +3.56322
H −0.53957 −0.46860 +3.26598

O	+2.19904	+0.01057	+0.60890
C	+2.08417	−1.47045	+3.17846
H	+2.15641	+0.68334	+3.14636
H	+0.30193	+0.07969	+4.56422
C	+1.21594	−2.61867	+2.74081
H	+3.05294	−1.50877	+2.66975
H	+2.26877	−1.51989	+4.25754
C	+1.31979	−3.13749	+1.45236
C	+0.48441	−4.15668	+1.01961
C	−0.47782	−4.67611	+1.87800
C	−0.59562	−4.17448	+3.16867
C	+0.24706	−3.15739	+3.58645
H	+2.07231	−2.73628	+0.77679
H	+0.58429	−4.55636	+0.01307
O	−1.32034	−5.67574	+1.50952
H	−1.34449	−4.59760	+3.82957
H	+0.15934	−2.77935	+4.60276
H	−1.14042	−5.94618	+0.60822

24

∗ E = +3.491 kcal/mol ; (22) 184_344_058_298_180

H	+0.00000	+0.00000	+0.00000
O	+0.00000	+0.00000	+0.96397
C	+1.27119	+0.00000	+1.37246
C	+1.42772	−0.10526	+2.87862
N	+0.18581	+0.18362	+3.56404
H	−0.55077	−0.44354	+3.25991
O	+2.20010	+0.01745	+0.61263
C	+2.05101	−1.49766	+3.17164
H	+2.16781	+0.65477	+3.15335
H	+0.29850	+0.07800	+4.56449
C	+1.16108	−2.62445	+2.72227
H	+3.02057	−1.55233	+2.66582
H	+2.23091	−1.55818	+4.25098
C	+1.25525	−3.13202	+1.42503
C	+0.40302	−4.12555	+0.97579
C	−0.57160	−4.63518	+1.82711
C	−0.67977	−4.14797	+3.12347
C	+0.18322	−3.15141	+3.55948
H	+2.01805	−2.73734	+0.75694
H	+0.48105	−4.52527	−0.02968

O	−1.38388	−5.61045	+1.34345
H	−1.43424	−4.55298	+3.79373
H	+0.10000	−2.78538	+4.58043
H	−2.00848	−5.88672	+2.01527

24

∗ E = +3.645 kcal/mol ; (23) 176_132_051_057_179

H	+0.00000	+0.00000	+0.00000
O	+0.00000	+0.00000	+0.96447
C	+1.26653	+0.00000	+1.37976
C	+1.40854	+0.09419	+2.88672
N	+2.34444	−0.93645	+3.29795
H	+3.18540	−0.87368	+2.73315
O	+2.20627	−0.02998	+0.63331
C	+1.86275	+1.53645	+3.21450
H	+0.42839	−0.09074	+3.33930
H	+2.60577	−0.82015	+4.26909
C	+0.91442	+2.58154	+2.69177
H	+1.96498	+1.61485	+4.30318
H	+2.85848	+1.67226	+2.77552
C	−0.23477	+2.92890	+3.39959
C	−1.14311	+3.84437	+2.89732
C	−0.91190	+4.43250	+1.65870
C	+0.22616	+4.09749	+0.93545
C	+1.12724	+3.17757	+1.45320
H	−0.41980	+2.47537	+4.37105
H	−2.03483	+4.11981	+3.45010
O	−1.82693	+5.32862	+1.20545
H	+0.40966	+4.56064	−0.03128
H	+2.01494	+2.91498	+0.88136
H	−1.55937	+5.67339	+0.35262

24

∗ E = +3.653 kcal/mol ; (24) 176_133_050_057_000

H	+0.00000	+0.00000	+0.00000
O	+0.00000	+0.00000	+0.96455
C	+1.26643	+0.00000	+1.38038
C	+1.40713	+0.10863	+2.88642
N	+2.36192	−0.90028	+3.30675
H	+3.19576	−0.83607	+2.73164
O	+2.20664	−0.03659	+0.63512
C	+1.83465	+1.56224	+3.20286

H	+0.43022	−0.08942	+3.34069
H	+2.63184	−0.76233	+4.27267
C	+0.86765	+2.58600	+2.67228
H	+1.93539	+1.65025	+4.29095
H	+2.82790	+1.71306	+2.76312
C	−0.28005	+2.92473	+3.38012
C	−1.20557	+3.81930	+2.86307
C	−0.99117	+4.39002	+1.61407
C	+0.14991	+4.06320	+0.89062
C	+1.06408	+3.16841	+1.42086
H	−0.45510	+2.48489	+4.35955
H	−2.09602	+4.07777	+3.43123
O	−1.86027	+5.27357	+1.05749
H	+0.30442	+4.52194	−0.08019
H	+1.95440	+2.91063	+0.85057
H	−2.60224	+5.42384	+1.64454

24

∗ E = +3.670 kcal/mol ; (25) 178_294_299_054_359

H	+0.00000	+0.00000	+0.00000
O	+0.00000	+0.00000	+0.96367
C	+1.28323	+0.00000	+1.37636
C	+1.38224	+0.03979	+2.89564
N	+0.88872	+1.29208	+3.44856
H	+1.42095	+2.08484	+3.11167
O	+2.20482	+0.00922	+0.61303
C	+0.61323	−1.13257	+3.51493
H	+2.44816	−0.05468	+3.12671
H	−0.08176	+1.43461	+3.19120
C	+1.04799	−2.46190	+2.95516
H	+0.76863	−1.08576	+4.59747
H	−0.45696	−0.98061	+3.33119
C	+2.25055	−3.03947	+3.35068
C	+2.68192	−4.24170	+2.81236
C	+1.90581	−4.88924	+1.85736
C	+0.70185	−4.32910	+1.44955
C	+0.28619	−3.12600	+1.99662
H	+2.86213	−2.54216	+4.10049
H	+3.62285	−4.68138	+3.13453
O	+2.27629	−6.06961	+1.29648
H	+0.10701	−4.84905	+0.70631

H −0.65574 −2.68815 +1.67457
H +3.12283 −6.35150 +1.64522
24
∗ E = +3.711 kcal/mol ; (26) 178_294_299_055_180
H +0.00000 +0.00000 +0.00000
O +0.00000 +0.00000 +0.96362
C +1.28453 +0.00000 +1.37462
C +1.38627 +0.05224 +2.89331
N +0.89688 +1.31152 +3.43415
H +1.43234 +2.09914 +3.09040
O +2.20378 +0.00233 +0.60861
C +0.61479 −1.11181 +3.52504
H +2.45213 −0.04326 +3.12411
H −0.07299 +1.45536 +3.17537
C +1.06003 −2.44893 +2.99330
H +0.76146 −1.04637 +4.60780
H −0.45425 −0.96587 +3.32988
C +2.25069 −3.02718 +3.43269
C +2.69718 −4.23438 +2.92665
C +1.94833 −4.89151 +1.95572
C +0.75988 −4.33316 +1.50414
C +0.32647 −3.11999 +2.02233
H +2.83862 −2.52038 +4.19511
H +3.62134 −4.68623 +3.27054
O +2.42331 −6.07523 +1.48852
H +0.17211 −4.84862 +0.74825
H −0.60464 −2.68598 +1.66572
H +1.83292 −6.43009 +0.82277
24
∗ E = +3.813 kcal/mol ; (27) 182_321_058_176_180
H +0.00000 +0.00000 +0.00000
O +0.00000 +0.00000 +0.96366
C +1.27783 +0.00000 +1.37793
C +1.38759 −0.05862 +2.88574
N +0.36581 +0.76056 +3.50749
H −0.55932 +0.46016 +3.22152
O +2.20412 −0.02427 +0.61988
C +1.36726 −1.55591 +3.29128
H +2.37158 +0.35143 +3.13612
H +0.42422 +0.68341 +4.51654

C	+1.56356	−1.71426	+4.77342
H	+0.40385	−1.98366	+2.98722
H	+2.15531	−2.08174	+2.73957
C	+0.48332	−1.90536	+5.63234
C	+0.65590	−1.99545	+7.00475
C	+1.93169	−1.89051	+7.54434
C	+3.02388	−1.69786	+6.70457
C	+2.83347	−1.61012	+5.33429
H	−0.51699	−1.99469	+5.21468
H	−0.18505	−2.14995	+7.67221
O	+2.05755	−1.98678	+8.89275
H	+4.02353	−1.62076	+7.12558
H	+3.69395	−1.46223	+4.68515
H	+2.97705	−1.90264	+9.14833

24

∗ E = +3.853 kcal/mol ; (28) 182_321_058_176_000

H	+0.00000	+0.00000	+0.00000
O	+0.00000	+0.00000	+0.96365
C	+1.27827	+0.00000	+1.37746
C	+1.38812	−0.05841	+2.88526
N	+0.36781	+0.76314	+3.50653
H	−0.55778	+0.46296	+3.22178
O	+2.20397	−0.02471	+0.61892
C	+1.36317	−1.55544	+3.29135
H	+2.37283	+0.34966	+3.13587
H	+0.42751	+0.68680	+4.51555
C	+1.55629	−1.71375	+4.77385
H	+0.39922	−1.98072	+2.98543
H	+2.15106	−2.08365	+2.74162
C	+0.47902	−1.92224	+5.62695
C	+0.65508	−2.01353	+7.00215
C	+1.92769	−1.89222	+7.54404
C	+3.01912	−1.68070	+6.70659
C	+2.82606	−1.59243	+5.33993
H	−0.51997	−2.02461	+5.20950
H	−0.19843	−2.18253	+7.65434
O	+2.16555	−1.97517	+8.87811
H	+4.00694	−1.59301	+7.14611
H	+3.68413	−1.43038	+4.69079
H	+1.34823	−2.12984	+9.35343

24
∗ E = +4.179 kcal/mol ; (29) 351_032_080_082_180

H	+0.00000	+0.00000	+0.00000
O	+0.00000	+0.00000	+0.96971
C	+1.27238	+0.00000	+1.36603
C	+2.27884	−0.21593	+0.22773
N	+1.72364	+0.36956	−0.99175
H	+1.85719	+1.37504	−1.00726
O	+1.58785	+0.14094	+2.50987
C	+3.68711	+0.25668	+0.62847
H	+2.30966	−1.30370	+0.07259
H	+2.16724	−0.00975	−1.81776
C	+4.45000	−0.75441	+1.44935
H	+4.24077	+0.45313	−0.29749
H	+3.60651	+1.20415	+1.17281
C	+5.09725	−1.81427	+0.81610
C	+5.79133	−2.77460	+1.53246
C	+5.85080	−2.68128	+2.91784
C	+5.21555	−1.62973	+3.56738
C	+4.51993	−0.67866	+2.83566
H	+5.06601	−1.88652	−0.26981
H	+6.29720	−3.59679	+1.03790
O	+6.54308	−3.64022	+3.58681
H	+5.26012	−1.55830	+4.65163
H	+4.00275	+0.12519	+3.35007
H	+6.50966	−3.47409	+4.52976

24
∗ E = +4.284 kcal/mol ; (30) 180_294_299_049_000

H	+0.00000	+0.00000	+0.00000
O	+0.00000	+0.00000	+0.96358
C	+1.27718	+0.00000	+1.38271
C	+1.36575	+0.00779	+2.90320
N	+0.84746	+1.23794	+3.48303
H	+1.37205	+2.04642	+3.17285
O	+2.20797	+0.02560	+0.62892
C	+0.60473	−1.18105	+3.49224
H	+2.43150	−0.05918	+3.14166
H	−0.12222	+1.37262	+3.21873
C	+0.93462	−2.51379	+2.86345
H	+0.80349	−1.19053	+4.57009

H −0.46858 −0.98804 +3.37352
C +2.21082 −2.81918 +2.40084
C +2.49743 −4.05928 +1.84620
C +1.50222 −5.02190 +1.74651
C +0.21865 −4.73473 +2.19722
C −0.05137 −3.49258 +2.74345
H +3.00588 −2.08054 +2.45553
H +3.49965 −4.27546 +1.48400
O +1.72445 −6.25087 +1.21143
H −0.55197 −5.49270 +2.10594
H −1.05950 −3.27257 +3.08653
H +2.63769 −6.33020 +0.93300
24
* E = +4.320 kcal/mol ; (31) 182_063_180_074_180
H +0.00000 +0.00000 +0.00000
O +0.00000 +0.00000 +0.96373
C +1.27804 +0.00000 +1.38070
C +1.35176 −0.05668 +2.89742
N +0.75769 −1.33024 +3.29591
H +0.77179 −1.42411 +4.30385
O +2.20410 +0.02156 +0.62449
C +2.79986 +0.03345 +3.37549
H +0.79469 +0.82020 +3.27172
H −0.20575 −1.39110 +2.98907
C +3.37415 +1.42192 +3.24708
H +2.82008 −0.27521 +4.42815
H +3.39742 −0.69002 +2.81300
C +3.04395 +2.40835 +4.17556
C +3.54070 +3.69668 +4.07449
C +4.39253 +4.01999 +3.02467
C +4.73543 +3.05142 +2.08977
C +4.22557 +1.76569 +2.20343
H +2.38593 +2.15920 +5.00628
H +3.28643 +4.46195 +4.79998
O +4.86171 +5.29427 +2.96084
H +5.40121 +3.30469 +1.26788
H +4.48078 +1.01642 +1.45990
H +5.43930 +5.39496 +2.20321
24
* E = +4.537 kcal/mol ; (32) 351_032_080_081_000

H	+0.00000	+0.00000	+0.00000
O	+0.00000	+0.00000	+0.96957
C	+1.27252	+0.00000	+1.36655
C	+2.27924	−0.21738	+0.22888
N	+1.72691	+0.37235	−0.98988
H	+1.85785	+1.37829	−0.99981
O	+1.58795	+0.14205	+2.50994
C	+3.68837	+0.25054	+0.63131
H	+2.30696	−1.30495	+0.07162
H	+2.17486	−0.00140	−1.81608
C	+4.44575	−0.75845	+1.46020
H	+4.24506	+0.44035	−0.29429
H	+3.61048	+1.20107	+1.17069
C	+5.08137	−1.82738	+0.83753
C	+5.77365	−2.78266	+1.56816
C	+5.84180	−2.67150	+2.95143
C	+5.21545	−1.60795	+3.59101
C	+4.52367	−0.66679	+2.84835
H	+5.04614	−1.91504	−0.24702
H	+6.26772	−3.60974	+1.06391
O	+6.50768	−3.57022	+3.72308
H	+5.27636	−1.53819	+4.67176
H	+4.01366	+0.14790	+3.35298
H	+6.89452	−4.25486	+3.17611

24

* E = +4.555 kcal/mol ; (33) 182_063_180_074_000

H	+0.00000	+0.00000	+0.00000
O	+0.00000	+0.00000	+0.96377
C	+1.27838	+0.00000	+1.38028
C	+1.35274	−0.05683	+2.89698
N	+0.76425	−1.33319	+3.29478
H	+0.77739	−1.42746	+4.30268
O	+2.20424	+0.02105	+0.62419
C	+2.80064	+0.03920	+3.37473
H	+0.79198	+0.81732	+3.27211
H	−0.19832	−1.39896	+2.98623
C	+3.36970	+1.43015	+3.24742
H	+2.82242	−0.27076	+4.42697
H	+3.40111	−0.68101	+2.81118
C	+3.04157	+2.41039	+4.17858

C +3.53810 +3.70170 +4.07421
C +4.38438 +4.02964 +3.02180
C +4.72493 +3.06412 +2.08229
C +4.21731 +1.78076 +2.19867
H +2.38850 +2.15991 +5.01264
H +3.27463 +4.45422 +4.81379
O +4.90583 +5.27567 +2.86774
H +5.38614 +3.33714 +1.26680
H +4.47033 +1.03200 +1.45373
H +4.60353 +5.84971 +3.57246
24
* E = +4.579 kcal/mol ; (34) 183_322_291_164_000
H +0.00000 +0.00000 +0.00000
O +0.00000 +0.00000 +0.96375
C +1.28065 +0.00000 +1.37693
C +1.39602 −0.07795 +2.89187
N +0.39284 +0.66669 +3.62669
H +0.52244 +1.66537 +3.52898
O +2.20330 +0.01103 +0.61332
C +1.33823 −1.57003 +3.28472
H +2.38916 +0.30735 +3.14306
H −0.53471 +0.44203 +3.28443
C +1.81163 −1.80533 +4.69320
H +0.30219 −1.91026 +3.15912
H +1.95674 −2.14367 +2.58554
C +0.96377 −1.63800 +5.78269
C +1.41938 −1.82461 +7.07926
C +2.74371 −2.18143 +7.30464
C +3.60628 −2.35211 +6.22903
C +3.13520 −2.16336 +4.94030
H −0.06878 −1.34676 +5.61429
H +0.74421 −1.69163 +7.92142
O +3.24254 −2.37906 +8.55365
H +4.63580 −2.63502 +6.42027
H +3.81429 −2.30206 +4.10185
H +2.55889 −2.23389 +9.20879
24
* E = +4.603 kcal/mol ; (35) 183_324_291_164_180
H +0.00000 +0.00000 +0.00000
O +0.00000 +0.00000 +0.96377

C +1.28027 +0.00000 +1.37677
C +1.39808 −0.07963 +2.89167
N +0.38115 +0.63950 +3.63175
H +0.48597 +1.64122 +3.53596
O +2.20311 +0.01132 +0.61311
C +1.37496 −1.57433 +3.27843
H +2.38325 +0.32595 +3.14261
H −0.54276 +0.39316 +3.29476
C +1.85505 −1.80622 +4.68530
H +0.34671 −1.93773 +3.15309
H +2.00527 −2.13022 +2.57552
C +1.00502 −1.65567 +5.77973
C +1.45930 −1.84021 +7.07391
C +2.78929 −2.17893 +7.29653
C +3.65304 −2.33190 +6.21970
C +3.18109 −2.14552 +4.92769
H −0.03166 −1.37947 +5.61101
H +0.79856 −1.72556 +7.92654
O +3.19027 −2.35233 +8.58380
H +4.69265 −2.60090 +6.39110
H +3.86294 −2.27167 +4.08967
H +4.11817 −2.58868 +8.61188
24
∗ E = +4.944 kcal/mol ; (36) 358_351_299_291_001
H +0.00000 +0.00000 +0.00000
O +0.00000 +0.00000 +0.96311
C +1.26300 +0.00000 +1.39887
C +2.36258 −0.03770 +0.32273
N +1.93945 −0.27530 −1.03639
H +1.47921 −1.15440 −1.21424
O +1.52070 +0.04681 +2.56548
C +3.14272 +1.28950 +0.40399
H +3.03535 −0.84576 +0.63118
H +1.48677 +0.50767 −1.48848
C +2.30005 +2.45558 −0.04707
H +3.46722 +1.42470 +1.43953
H +4.02957 +1.19651 −0.22938
C +1.38521 +3.05238 +0.81857
C +0.56628 +4.08649 +0.39157
C +0.65049 +4.54382 −0.91905

C +1.55878 +3.96395 −1.79678
C +2.37106 +2.93087 −1.35623
H +1.31451 +2.70350 +1.84680
H −0.14009 +4.54369 +1.08040
O −0.12544 +5.55330 −1.38955
H +1.61943 +4.33836 −2.81306
H +3.08718 +2.48420 −2.04297
H −0.70078 +5.88142 −0.69713
24
* E = +4.983 kcal/mol ; (37) 183_202_203_081_180
H +0.00000 +0.00000 +0.00000
O +0.00000 +0.00000 +0.96418
C +1.27440 +0.00000 +1.37034
C +1.42263 −0.06849 +2.87958
N +2.73249 +0.45878 +3.20937
H +3.05103 +0.12076 +4.10703
O +2.19700 +0.02273 +0.60485
C +0.32390 +0.70001 +3.63925
H +1.30582 −1.14287 +3.11688
H +3.40764 +0.20318 +2.49855
C −0.98180 −0.03958 +3.78204
H +0.73298 +0.90818 +4.63491
H +0.16968 +1.67283 +3.16113
C −1.06190 −1.17571 +4.58593
C −2.24669 −1.87477 +4.74071
C −3.39108 −1.43578 +4.08498
C −3.33494 −0.30273 +3.28315
C −2.13671 +0.38156 +3.13606
H −0.17407 −1.51953 +5.11398
H −2.30704 −2.75685 +5.36914
O −4.53497 −2.14778 +4.26455
H −4.23016 +0.04271 +2.77124
H −2.09698 +1.25962 +2.49682
H −5.25267 −1.75059 +3.76974
24
* E = +5.045 kcal/mol ; (38) 179_300_065_056_181
H +0.00000 +0.00000 +0.00000
O +0.00000 +0.00000 +0.96361
C +1.28402 +0.00000 +1.37613
C +1.36480 +0.03639 +2.89104

N	+0.70178	+1.24872	+3.34843
H	−0.28884	+1.21069	+3.13502
O	+2.20573	−0.03850	+0.61818
C	+0.78215	−1.27074	+3.47057
H	+2.43083	+0.10022	+3.12999
H	+0.80192	+1.35497	+4.35019
C	+1.45463	−2.49969	+2.92042
H	+0.89001	−1.22583	+4.56077
H	−0.29125	−1.29215	+3.24758
C	+2.67699	−2.93555	+3.42969
C	+3.33322	−4.02927	+2.89430
C	+2.76878	−4.71211	+1.82213
C	+1.55172	−4.29405	+1.29955
C	+0.90673	−3.19452	+1.84833
H	+3.12332	−2.40705	+4.26946
H	+4.28278	−4.37106	+3.29183
O	+3.44627	−5.78115	+1.32939
H	+1.10862	−4.82981	+0.46347
H	−0.04615	−2.87015	+1.43562
H	+2.97366	−6.16020	+0.58730

24

∗ E = +5.045 kcal/mol ; (39) 179_300_065_055_359

H	+0.00000	+0.00000	+0.00000
O	+0.00000	+0.00000	+0.96368
C	+1.28308	+0.00000	+1.37722
C	+1.36230	+0.02490	+2.89252
N	+0.70507	+1.23677	+3.35928
H	−0.28487	+1.20670	+3.14140
O	+2.20666	−0.03040	+0.62113
C	+0.77374	−1.28417	+3.46139
H	+2.42863	+0.08236	+3.13202
H	+0.80126	+1.33234	+4.36251
C	+1.42279	−2.51170	+2.88022
H	+0.89758	−1.25727	+4.55055
H	−0.30278	−1.29097	+3.25356
C	+2.66356	−2.94711	+3.33362
C	+3.29358	−4.04158	+2.76175
C	+2.68285	−4.72071	+1.71349
C	+1.44341	−4.30112	+1.24653
C	+0.82827	−3.20515	+1.82816

H +3.14845 −2.42317 +4.15454
H +4.26220 −4.37161 +3.12955
O +3.25076 −5.80014 +1.11600
H +0.98003 −4.84385 +0.42968
H −0.14141 −2.87713 +1.45983
H +4.10478 −5.98583 +1.50827
24
∗ E = +5.049 kcal/mol ; (40) 183_202_203_080_000
H +0.00000 +0.00000 +0.00000
O +0.00000 +0.00000 +0.96419
C +1.27310 +0.00000 +1.37275
C +1.41704 −0.06682 +2.88266
N +2.72655 +0.45908 +3.21565
H +3.04370 +0.12010 +4.11339
O +2.19812 +0.02136 +0.61008
C +0.31709 +0.70390 +3.63836
H +1.29813 −1.14078 +3.12092
H +3.40272 +0.20476 +2.50534
C −0.99337 −0.02959 +3.77103
H +0.72150 +0.90798 +4.63676
H +0.16993 +1.67857 +3.16183
C −1.08607 −1.16235 +4.57262
C −2.28102 −1.85327 +4.71357
C −3.41625 −1.40794 +4.04740
C −3.34603 −0.27481 +3.24591
C −2.14326 +0.39804 +3.11332
H −0.20675 −1.51323 +5.10994
H −2.33433 −2.73564 +5.34693
O −4.61587 −2.03947 +4.14717
H −4.24180 +0.06101 +2.73444
H −2.09025 +1.27659 +2.47556
H −4.54373 −2.79738 +4.72853
24
∗ E = +5.067 kcal/mol ; (41) 007_166_301_065_180
H +0.00000 +0.00000 +0.00000
O +0.00000 +0.00000 +0.96387
C +1.24903 +0.00000 +1.45073
C +2.38226 +0.17025 +0.42806
N +3.64754 +0.49147 +1.04922
H +3.57068 +1.35438 +1.57537

O	+1.44886	−0.12177	+2.62236
C	+2.54334	−1.10840	−0.42008
H	+2.10296	+0.99229	−0.24681
H	+3.87095	−0.22381	+1.73467
C	+1.35121	−1.41972	−1.28706
H	+3.43740	−0.95530	−1.03313
H	+2.75298	−1.94999	+0.25129
C	+1.03069	−0.59386	−2.36738
C	−0.08613	−0.82819	−3.15144
C	−0.91224	−1.91101	−2.86521
C	−0.60988	−2.74599	−1.79620
C	+0.51113	−2.49502	−1.01755
H	+1.67887	+0.24749	−2.60511
H	−0.33156	−0.18913	−3.99280
O	−1.99365	−2.10256	−3.65949
H	−1.25548	−3.59064	−1.56880
H	+0.73158	−3.14662	−0.17590
H	−2.49117	−2.86882	−3.37031

24

∗ E = +5.228 kcal/mol ; (42) 358_350_299_290_179

H	+0.00000	+0.00000	+0.00000
O	+0.00000	+0.00000	+0.96296
C	+1.26232	+0.00000	+1.40070
C	+2.36442	−0.04315	+0.32751
N	+1.94649	−0.29605	−1.03079
H	+1.48161	−1.17514	−1.19708
O	+1.51839	+0.04995	+2.56739
C	+3.14129	+1.28661	+0.39638
H	+3.03893	−0.84559	+0.64653
H	+1.49378	+0.48290	−1.49000
C	+2.30461	+2.44473	−0.08518
H	+3.45267	+1.43941	+1.43360
H	+4.03639	+1.18446	−0.22387
C	+1.36315	+3.04610	+0.75376
C	+0.55280	+4.07351	+0.30433
C	+0.67132	+4.52465	−1.00662
C	+1.60253	+3.94165	−1.85680
C	+2.40869	+2.91007	−1.39222
H	+1.26917	+2.70291	+1.78220
H	−0.17531	+4.54391	+0.95638

```
 O    −0.14275    +5.53720    −1.39976
 H    +1.70283    +4.29863    −2.87905
 H    +3.14170    +2.46169    −2.05943
 H    +0.03593    +5.77473    −2.31057
24
 * E =  +5.289  kcal/mol ; (43) 007_168_303_066_000
 H    +0.00000    +0.00000    +0.00000
 O    +0.00000    +0.00000    +0.96390
 C    +1.24862    +0.00000    +1.45147
 C    +2.38219    +0.16507    +0.42822
 N    +3.65833    +0.43372    +1.05226
 H    +3.59666    +1.26546    +1.62843
 O    +1.44770    −0.11439    +2.62383
 C    +2.50604    −1.08883    −0.46179
 H    +2.12224    +1.01559    −0.21877
 H    +3.87952    −0.32139    +1.69425
 C    +1.32279    −1.32299    −1.36568
 H    +3.41790    −0.95095    −1.05196
 H    +2.66814    −1.96282    +0.18071
 C    +1.05202    −0.44344    −2.41261
 C    −0.05086    −0.61979    −3.23592
 C    −0.90791    −1.69431    −3.02208
 C    −0.65529    −2.58245    −1.98240
 C    +0.44770    −2.39051    −1.16756
 H    +1.72395    +0.39248    −2.59681
 H    −0.24280    +0.07372    −4.05099
 O    −1.99919    −1.92149    −3.79296
 H    −1.33514    −3.41290    −1.82604
 H    +0.63253    −3.08382    −0.35090
 H    −2.07345    −1.25443    −4.47679
24
 * E =  +5.606  kcal/mol ; (44) 006_334_161_098_180
 H    +0.00000    +0.00000    +0.00000
 O    +0.00000    +0.00000    +0.97135
 C    +1.27061    +0.00000    +1.36711
 C    +2.28321    +0.14771    +0.22437
 N    +1.67203    −0.35365    −1.00625
 H    +2.15678    −0.00897    −1.82456
 O    +1.58354    −0.06403    +2.52003
 C    +2.63475    +1.64677    +0.05549
```

H	+3.18591	−0.39930	+0.52313
H	+1.68700	−1.36628	−1.04346
C	+3.92160	+2.07817	+0.71918
H	+2.71388	+1.85660	−1.01749
H	+1.79713	+2.25820	+0.41582
C	+4.82099	+2.88683	+0.02826
C	+6.00281	+3.31901	+0.60766
C	+6.30552	+2.93824	+1.90847
C	+5.42187	+2.12860	+2.61274
C	+4.24049	+1.70497	+2.02243
H	+4.59328	+3.18768	−0.99190
H	+6.70184	+3.94729	+0.06628
O	+7.47432	+3.37923	+2.44212
H	+5.65787	+1.82863	+3.63122
H	+3.55731	+1.07512	+2.58632
H	+7.56954	+3.05840	+3.33985

24

∗ E = +5.606 kcal/mol ; (45) 006_334_161_098_359

H	+0.00000	+0.00000	+0.00000
O	+0.00000	+0.00000	+0.97136
C	+1.27061	+0.00000	+1.36711
C	+2.28320	+0.14777	+0.22437
N	+1.67202	−0.35360	−1.00625
H	+2.15674	−0.00890	−1.82456
O	+1.58354	−0.06407	+2.52003
C	+2.63468	+1.64685	+0.05550
H	+3.18592	−0.39922	+0.52312
H	+1.68703	−1.36625	−1.04347
C	+3.92148	+2.07832	+0.71925
H	+2.71388	+1.85666	−1.01748
H	+1.79702	+2.25824	+0.41576
C	+4.24040	+1.70507	+2.02247
C	+5.42172	+2.12879	+2.61281
C	+6.30529	+2.93857	+1.90861
C	+6.00255	+3.31941	+0.60783
C	+4.82078	+2.88714	+0.02839
H	+3.55729	+1.07510	+2.58631
H	+5.65775	+1.82877	+3.63127
O	+7.47403	+3.37967	+2.44232
H	+6.70151	+3.94780	+0.06650

H +4.59305 +3.18804 −0.99175
H +7.56929 +3.05876 +3.34001
24
∗ E = +5.918 kcal/mol ; (46) 006_335_161_101_001
H +0.00000 +0.00000 +0.00000
O +0.00000 +0.00000 +0.97131
C +1.27082 +0.00000 +1.36675
C +2.28487 +0.13635 +0.22442
N +1.66861 −0.34610 −1.01120
H +2.15545 +0.00361 −1.82608
O +1.58535 −0.05366 +2.51954
C +2.66526 +1.63119 +0.07229
H +3.17682 −0.42932 +0.52048
H +1.67631 −1.35836 −1.05965
C +3.98589 +2.01250 +0.69832
H +2.70753 +1.86469 −0.99783
H +1.85925 +2.25442 +0.48061
C +4.89795 +2.78091 −0.01448
C +6.11235 +3.16151 +0.54121
C +6.43005 +2.76795 +1.83397
C +5.52997 +1.99595 +2.56109
C +4.32174 +1.62570 +1.99703
H +4.66016 +3.09292 −1.02883
H +6.81418 +3.76157 −0.03300
O +7.60194 +3.10426 +2.43305
H +5.79314 +1.69661 +3.57005
H +3.62321 +1.02547 +2.57498
H +8.13237 +3.64418 +1.84582
24
∗ E = +5.924 kcal/mol ; (47) 005_160_026_066_180
H +0.00000 +0.00000 +0.00000
O +0.00000 +0.00000 +0.96362
C +1.24624 +0.00000 +1.44855
C +2.36933 +0.11772 +0.41913
N +3.55841 +0.58068 +1.09379
H +3.52603 +0.31115 +2.07097
O +1.44917 −0.10742 +2.62120
C +2.55029 −1.24167 −0.30530
H +2.05064 +0.85559 −0.33484
H +4.40082 +0.21371 +0.67295

C +1.36531 −1.63348 −1.14730
H +3.44189 −1.14591 −0.93737
H +2.76476 −2.01067 +0.44542
C +1.05946 −0.92317 −2.31046
C −0.06070 −1.22264 −3.06767
C −0.90421 −2.25473 −2.66846
C −0.61642 −2.97454 −1.51456
C +0.50706 −2.65906 −0.76434
H +1.72065 −0.12218 −2.63577
H −0.29529 −0.67394 −3.97343
O −1.98770 −2.51403 −3.44033
H −1.27614 −3.77905 −1.19917
H +0.71512 −3.21795 +0.14451
H −2.49804 −3.23629 −3.07127
24
* E = +6.088 kcal/mol ; (48) 358_174_304_295_359
H +0.00000 +0.00000 +0.00000
O +0.00000 +0.00000 +0.96021
C +1.26089 +0.00000 +1.44342
C +2.37063 −0.05823 +0.38703
N +3.69195 +0.08717 +0.94704
H +3.75569 +0.94578 +1.48133
O +1.45834 +0.03428 +2.61751
C +2.28356 −1.38321 −0.40315
H +2.19217 +0.76924 −0.31578
H +3.85828 −0.66698 +1.60744
C +2.54500 −2.58231 +0.47131
H +1.30123 −1.47775 −0.88242
H +3.02977 −1.32962 −1.20086
C +1.53675 −3.12599 +1.26210
C +1.78772 −4.19749 +2.10502
C +3.06484 −4.74289 +2.16966
C +4.08474 −4.21285 +1.38787
C +3.81901 −3.14215 +0.55062
H +0.53384 −2.70499 +1.22375
H +0.98994 −4.61070 +2.71726
O +3.36817 −5.79227 +2.97508
H +5.07495 −4.65126 +1.44917
H +4.61883 −2.72437 −0.05629
H +2.59725 −6.06026 +3.47696

24
∗ E = +6.155 kcal/mol ; (49) 005_163_021_067_000

H	+0.00000	+0.00000	+0.00000
O	+0.00000	+0.00000	+0.96380
C	+1.24577	+0.00000	+1.44947
C	+2.36808	+0.12667	+0.41931
N	+3.57955	+0.51746	+1.10032
H	+3.51057	+0.28708	+2.08556
O	+1.44763	−0.10502	+2.62252
C	+2.50791	−1.19671	−0.37535
H	+2.06354	+0.90936	−0.29508
H	+4.39676	+0.06942	+0.71007
C	+1.32968	−1.49802	−1.26416
H	+3.41515	−1.10103	−0.98494
H	+2.67846	−2.01559	+0.33262
C	+1.06896	−0.70680	−2.38145
C	−0.03847	−0.93737	−3.18553
C	−0.90936	−1.97762	−2.87957
C	−0.66649	−2.77756	−1.76794
C	+0.44007	−2.53193	−0.97333
H	+1.75099	+0.10184	−2.63674
H	−0.22307	−0.31308	−4.05631
O	−2.00515	−2.25534	−3.62697
H	−1.35750	−3.58199	−1.54060
H	+0.61618	−3.15403	−0.09939
H	−2.07475	−1.64545	−4.36272

24
∗ E = +6.259 kcal/mol ; (50) 358_174_303_296_181

H	+0.00000	+0.00000	+0.00000
O	+0.00000	+0.00000	+0.96016
C	+1.26022	+0.00000	+1.44409
C	+2.37149	−0.05467	+0.38898
N	+3.69174	+0.08742	+0.95359
H	+3.75802	+0.95169	+1.47844
O	+1.45776	+0.03259	+2.61816
C	+2.28617	−1.37516	−0.40848
H	+2.19584	+0.77642	−0.31006
H	+3.84655	−0.65966	+1.62481
C	+2.54304	−2.58028	+0.45884
H	+1.30543	−1.46674	−0.89162

H +3.03498 −1.31691 −1.20346
C +1.52445 −3.14080 +1.23000
C +1.76319 −4.21990 +2.06200
C +3.04235 −4.76045 +2.13818
C +4.07124 −4.21404 +1.38067
C +3.81640 −3.13188 +0.54989
H +0.52086 −2.72234 +1.18010
H +0.97242 −4.65664 +2.66208
O +3.22956 −5.82077 +2.96449
H +5.07121 −4.63722 +1.43858
H +4.62438 −2.70386 −0.03860
H +4.14608 −6.09919 +2.94626
24
* E = +6.351 kcal/mol ; (51) 183_192_204_114_000
H +0.00000 +0.00000 +0.00000
O +0.00000 +0.00000 +0.96380
C +1.27804 +0.00000 +1.37500
C +1.39889 −0.07075 +2.88374
N +2.77420 +0.22550 +3.23484
H +3.01765 −0.17536 +4.13032
O +2.20039 +0.00598 +0.61087
C +0.40992 +0.87490 +3.61867
H +1.10114 −1.10885 +3.12166
H +3.40725 −0.13073 +2.52841
C −0.63891 +0.15884 +4.43182
H +1.01304 +1.51276 +4.27020
H −0.07476 +1.53726 +2.89343
C −0.73650 +0.35103 +5.80410
C −1.69564 −0.31095 +6.56072
C −2.57801 −1.18585 +5.94268
C −2.49678 −1.39244 +4.56921
C −1.53759 −0.72394 +3.82945
H −0.04993 +1.03459 +6.29753
H −1.75630 −0.14672 +7.63402
O −3.53637 −1.86436 +6.62720
H −3.19638 −2.07693 +4.10115
H −1.48520 −0.88234 +2.75495
H −3.49977 −1.64055 +7.55796
24
* E = +6.379 kcal/mol ; (52) 183_193_204_113_180

H	+0.00000	+0.00000	+0.00000
O	+0.00000	+0.00000	+0.96382
C	+1.27890	+0.00000	+1.37352
C	+1.40381	−0.06661	+2.88218
N	+2.77747	+0.24324	+3.22753
H	+3.02541	−0.14640	+4.12674
O	+2.19940	+0.00402	+0.60723
C	+0.40993	+0.87216	+3.61873
H	+1.11586	−1.10677	+3.12373
H	+3.41228	−0.11477	+2.52365
C	−0.64998	+0.15160	+4.41403
H	+1.00833	+1.49918	+4.28495
H	−0.06321	+1.54691	+2.89721
C	−0.76281	+0.33749	+5.78941
C	−1.72680	−0.32230	+6.53575
C	−2.60474	−1.19348	+5.90508
C	−2.50952	−1.39448	+4.53240
C	−1.54112	−0.72519	+3.79945
H	−0.07907	+1.01839	+6.29063
H	−1.81203	−0.17448	+7.60700
O	−3.53426	−1.82293	+6.67147
H	−3.19877	−2.07446	+4.03674
H	−1.48055	−0.88133	+2.72534
H	−4.08284	−2.38952	+6.12770

24

∗ E = +6.576 kcal/mol ; (53) 179_284_169_052_000

H	+0.00000	+0.00000	+0.00000
O	+0.00000	+0.00000	+0.96352
C	+1.27051	+0.00000	+1.37627
C	+1.39761	+0.01941	+2.89383
N	+1.09727	+1.37306	+3.34848
H	+0.99571	+1.39119	+4.35598
O	+2.20461	+0.00292	+0.62422
C	+0.42015	−0.95570	+3.54519
H	+2.43355	−0.28300	+3.10201
H	+1.83921	+2.01608	+3.10113
C	+0.47566	−2.36387	+3.00161
H	+0.62353	−0.95902	+4.62524
H	−0.58989	−0.55281	+3.41315
C	−0.67011	−3.15333	+3.01469

C −0.65662 −4.45752 +2.54567
C +0.52043 −4.99795 +2.04047
C +1.67232 −4.22400 +2.00698
C +1.64385 −2.92317 +2.48534
H −1.59845 −2.73609 +3.39703
H −1.56432 −5.05621 +2.56436
O +0.59431 −6.26777 +1.56252
H +2.58172 −4.65301 +1.60023
H +2.55833 −2.33793 +2.44009
H −0.25754 −6.69973 +1.63604
24
∗ E = +7.713 kcal/mol ; (54) 353_156_037_180_000
H +0.00000 +0.00000 +0.00000
O +0.00000 +0.00000 +0.95987
C +1.25517 +0.00000 +1.44025
C +2.35819 −0.16586 +0.40478
N +3.57372 +0.39683 +0.93622
H +3.63203 +0.21316 +1.93201
O +1.46134 +0.05795 +2.61328
C +2.43406 −1.67784 +0.05733
H +2.07562 +0.37914 −0.51046
H +4.39076 +0.02313 +0.46958
C +3.50296 −1.95393 −0.96363
H +2.64102 −2.22608 +0.98384
H +1.45894 −2.01942 −0.31394
C +4.75275 −2.43168 −0.58671
C +5.75774 −2.63827 −1.52338
C +5.51878 −2.36518 −2.86362
C +4.27339 −1.88680 −3.26100
C +3.28495 −1.68572 −2.31534
H +4.94444 −2.65648 +0.45993
H +6.72814 −3.01677 −1.21155
O +6.45641 −2.54953 −3.82656
H +4.10182 −1.68404 −4.31266
H +2.31282 −1.31630 −2.63606
H +7.26907 −2.88212 −3.44315
24
∗ E = +7.816 kcal/mol ; (55) 353_156_037_180_180
H +0.00000 +0.00000 +0.00000
O +0.00000 +0.00000 +0.95983

C	+1.25561	+0.00000	+1.43987
C	+2.35785	−0.16486	+0.40330
N	+3.57479	+0.39375	+0.93550
H	+3.63247	+0.20969	+1.93125
O	+1.46197	+0.05772	+2.61275
C	+2.42996	−1.67543	+0.04916
H	+2.07511	+0.38410	−0.50966
H	+4.39153	+0.01906	+0.46912
C	+3.50006	−1.95032	−0.97114
H	+2.63426	−2.22874	+0.97322
H	+1.45433	−2.01259	−0.32483
C	+4.75320	−2.42675	−0.59129
C	+5.76095	−2.63324	−1.52005
C	+5.52491	−2.36148	−2.86185
C	+4.28030	−1.88601	−3.26236
C	+3.28414	−1.68423	−2.32013
H	+4.94058	−2.64976	+0.45663
H	+6.73482	−3.00823	−1.22437
O	+6.53737	−2.58106	−3.73775
H	+4.09227	−1.67862	−4.31305
H	+2.31303	−1.31696	−2.64594
H	+6.26268	−2.36298	−4.62932

24

∗ E = +7.827 kcal/mol ; (56) 354_166_031_297_359

H	+0.00000	+0.00000	+0.00000
O	+0.00000	+0.00000	+0.96026
C	+1.25778	+0.00000	+1.44292
C	+2.35365	−0.14411	+0.39498
N	+3.61545	+0.20862	+0.99754
H	+3.59901	−0.02448	+1.98506
O	+1.45801	+0.05718	+2.61570
C	+2.29653	−1.57947	−0.19924
H	+2.12188	+0.55923	−0.42150
H	+4.38711	−0.27956	+0.56244
C	+2.57100	−2.64829	+0.82331
H	+1.31898	−1.74560	−0.67119
H	+3.04182	−1.62761	−1.00156
C	+1.57350	−3.07557	+1.69515
C	+1.83394	−4.02302	+2.67221
C	+3.11036	−4.56110	+2.79278

C +4.11864 −4.15093 +1.92906
C +3.84150 −3.20301 +0.95808
H +0.57123 −2.65902 +1.61478
H +1.04419 −4.34438 +3.34676
O +3.42250 −5.49142 +3.73039
H +5.10716 −4.58528 +2.03156
H +4.63283 −2.89461 +0.27789
H +2.65881 −5.68226 +4.27663
24
∗ E = +7.862 kcal/mol ; (57) 021_205_203_317_181
H +0.00000 +0.00000 +0.00000
O +0.00000 +0.00000 +0.96235
C +1.24269 +0.00000 +1.45640
C +2.36635 +0.48747 +0.53644
N +3.60425 −0.11859 +0.99610
H +4.40657 +0.42473 +0.70629
O +1.44770 −0.28164 +2.59949
C +2.14077 +0.23484 −0.96365
H +2.35313 +1.58311 +0.68599
H +3.59569 −0.17650 +2.00875
C +1.58399 −1.12601 −1.32048
H +1.47205 +1.00669 −1.36614
H +3.10611 +0.39534 −1.45962
C +0.57931 −1.23501 −2.28105
C +0.02534 −2.45982 −2.61855
C +0.47530 −3.61032 −1.98426
C +1.47503 −3.52123 −1.02093
C +2.02377 −2.29157 −0.69387
H +0.22219 −0.33796 −2.78374
H −0.75694 −2.54100 −3.36538
O −0.09257 −4.79042 −2.33785
H +1.82453 −4.42206 −0.52177
H +2.80581 −2.22670 +0.05702
H +0.27945 −5.50678 −1.82156
24
∗ E = +7.916 kcal/mol ; (58) 357_165_285_164_000
H +0.00000 +0.00000 +0.00000
O +0.00000 +0.00000 +0.96030
C +1.25660 +0.00000 +1.44247
C +2.36996 −0.07816 +0.39474

N	+3.66493	+0.27279	+0.93296
H	+3.73917	+1.27308	+1.07112
O	+1.45813	+0.02349	+2.61786
C	+2.40555	−1.51331	−0.18103
H	+2.12777	+0.61334	−0.42472
H	+3.75905	−0.14832	+1.85309
C	+3.17546	−1.59795	−1.47164
H	+2.85249	−2.15847	+0.58538
H	+1.38438	−1.87979	−0.34245
C	+4.56716	−1.57366	−1.48935
C	+5.26578	−1.61747	−2.68606
C	+4.57785	−1.68625	−3.89261
C	+3.18921	−1.71441	−3.89483
C	+2.50532	−1.67077	−2.69063
H	+5.10844	−1.50473	−0.55110
H	+6.35320	−1.59911	−2.68459
O	+5.21334	−1.73365	−5.09219
H	+2.66550	−1.77674	−4.84258
H	+1.41773	−1.70612	−2.70133
H	+6.16275	−1.71462	−4.96523

24

∗ E = +8.084 kcal/mol ; (59) 357_165_286_163_179

H	+0.00000	+0.00000	+0.00000
O	+0.00000	+0.00000	+0.96028
C	+1.25737	+0.00000	+1.44149
C	+2.36924	−0.07925	+0.39212
N	+3.66490	+0.27395	+0.92636
H	+3.73551	+1.27348	+1.07184
O	+1.45983	+0.02479	+2.61656
C	+2.40417	−1.51553	−0.18084
H	+2.12485	+0.61075	−0.42799
H	+3.76708	−0.15420	+1.84232
C	+3.15962	−1.60412	−1.48001
H	+2.86143	−2.15695	+0.58273
H	+1.38254	−1.88609	−0.32970
C	+4.55357	−1.54815	−1.51708
C	+5.24064	−1.60060	−2.71669
C	+4.53970	−1.71110	−3.91324
C	+3.15265	−1.77101	−3.89747
C	+2.47712	−1.71807	−2.68528

H +5.10231 −1.44825 −0.58597
H +6.32420 −1.55909 −2.74793
O +5.25999 −1.76185 −5.06375
H +2.60156 −1.86587 −4.82998
H +1.39079 −1.77942 −2.68388
H +4.67578 −1.84658 −5.81823
24
∗ E = +8.087 kcal/mol ; (60) 354_166_030_297_181
H +0.00000 +0.00000 +0.00000
O +0.00000 +0.00000 +0.96023
C +1.25739 +0.00000 +1.44319
C +2.35405 −0.14048 +0.39522
N +3.61543 +0.21071 +0.99992
H +3.59446 −0.01732 +1.98855
O +1.45829 +0.05694 +2.61576
C +2.29942 −1.57304 −0.20508
H +2.12227 +0.56568 −0.41883
H +4.38649 −0.28241 +0.56952
C +2.56372 −2.64833 +0.81332
H +1.32513 −1.73572 −0.68487
H +3.05022 −1.61809 −1.00252
C +1.55318 −3.08680 +1.66970
C +1.79579 −4.04253 +2.63909
C +3.07119 −4.58200 +2.77099
C +4.09107 −4.16090 +1.92722
C +3.83042 −3.20090 +0.95909
H +0.55252 −2.66846 +1.57783
H +1.01103 −4.38243 +3.30599
O +3.26163 −5.51883 +3.73440
H +5.08675 −4.58675 +2.02422
H +4.63219 −2.88709 +0.29398
H +4.17549 −5.80620 +3.74383
24
∗ E = +8.164 kcal/mol ; (61) 022_207_204_317_359
H +0.00000 +0.00000 +0.00000
O +0.00000 +0.00000 +0.96276
C +1.24339 +0.00000 +1.45501
C +2.36547 +0.50457 +0.54172
N +3.59757 −0.14088 +0.96241
H +4.40529 +0.40625 +0.69543

O	+1.45237	−0.29488	+2.59392
C	+2.12314	+0.32471	−0.96655
H	+2.37321	+1.59281	+0.73841
H	+3.59451	−0.24676	+1.97130
C	+1.54930	−1.01115	−1.38414
H	+1.45912	+1.12179	−1.32477
H	+3.08639	+0.49736	−1.46241
C	+0.49686	−1.06039	−2.29269
C	−0.06893	−2.26848	−2.68137
C	+0.41920	−3.45543	−2.15326
C	+1.46938	−3.42498	−1.23969
C	+2.02636	−2.21719	−0.86215
H	+0.11021	−0.13531	−2.71655
H	−0.89090	−2.28564	−3.39274
O	−0.08803	−4.66796	−2.48807
H	+1.83461	−4.36270	−0.83447
H	+2.84569	−2.19464	−0.14969
H	−0.80906	−4.56890	−3.11119

24

∗ E = +10.484 kcal/mol ; (62) 009_081_183_071_180

H	+0.00000	+0.00000	+0.00000
O	+0.00000	+0.00000	+0.95983
C	+1.26012	+0.00000	+1.44131
C	+2.34545	+0.22641	+0.38607
N	+2.40183	+1.66530	+0.14517
H	+3.06758	+1.88205	−0.58591
O	+1.46310	−0.11909	+2.60766
C	+3.69624	−0.27436	+0.89348
H	+2.07297	−0.36122	−0.51199
H	+1.50533	+2.05482	−0.11932
C	+3.75810	−1.77858	+0.96255
H	+4.47112	+0.10093	+0.21373
H	+3.87118	+0.17468	+1.87539
C	+4.03803	−2.52822	−0.17945
C	+4.06657	−3.91205	−0.14992
C	+3.81158	−4.57556	+1.04491
C	+3.53238	−3.84703	+2.19452
C	+3.50507	−2.46038	+2.14819
H	+4.25051	−2.01444	−1.11534
H	+4.29059	−4.49474	−1.03698

O +3.85059 −5.93367 +1.03317
H +3.33387 −4.36613 +3.12930
H +3.27199 −1.89418 +3.04547
H +3.66394 −6.27693 +1.90796
24
* E = +10.764 kcal/mol ; (63) 352_268_297_163_000
H +0.00000 +0.00000 +0.00000
O +0.00000 +0.00000 +0.96031
C +1.25641 +0.00000 +1.44916
C +2.38960 −0.18346 +0.43561
N +2.73589 −1.59605 +0.31500
H +3.08068 −1.95508 +1.19747
O +1.44700 +0.06539 +2.62503
C +2.08509 +0.39827 −0.95199
H +3.25454 +0.33659 +0.85893
H +1.92360 −2.14754 +0.05792
C +3.32582 +0.57280 −1.78904
H +1.38476 −0.27275 −1.47540
H +1.58841 +1.37020 −0.84014
C +3.92026 −0.50411 −2.43870
C +5.08207 −0.33875 −3.17708
C +5.67111 +0.91692 −3.27418
C +5.09050 +2.00392 −2.63251
C +3.92916 +1.82341 −1.89996
H +3.47459 −1.49054 −2.35442
H +5.53550 −1.18930 −3.68053
O +6.80719 +1.13576 −3.98482
H +5.55853 +2.97832 −2.72066
H +3.47855 +2.67993 −1.40353
H +7.11441 +0.31884 −4.37990
24
* E = +10.805 kcal/mol ; (64) 352_270_297_163_180
H +0.00000 +0.00000 +0.00000
O +0.00000 +0.00000 +0.96036
C +1.25685 +0.00000 +1.44901
C +2.38692 −0.18938 +0.43359
N +2.70088 −1.60601 +0.28096
H +3.04039 −1.99420 +1.15292
O +1.44759 +0.07057 +2.62451
C +2.09507 +0.42999 −0.94065

H	+3.26299	+0.30169	+0.86820
H	+1.87765	−2.13426	+0.01053
C	+3.33879	+0.59679	−1.77477
H	+1.38158	−0.21444	−1.47958
H	+1.61739	+1.40850	−0.80634
C	+3.91136	−0.48049	−2.45000
C	+5.07213	−0.32909	−3.18731
C	+5.68761	+0.91615	−3.26004
C	+5.13169	+2.00121	−2.59480
C	+3.96560	+1.83417	−1.86070
H	+3.44275	−1.45777	−2.38447
H	+5.51884	−1.16518	−3.71452
O	+6.82358	+1.01545	−3.99707
H	+5.60776	+2.97695	−2.65336
H	+3.53582	+2.69100	−1.34693
H	+7.15575	+1.91386	−3.97733

24

∗ E = +10.849 kcal/mol ; (65) 009_080_184_072_000

H	+0.00000	+0.00000	+0.00000
O	+0.00000	+0.00000	+0.95980
C	+1.26027	+0.00000	+1.44122
C	+2.34501	+0.22763	+0.38560
N	+2.39637	+1.66648	+0.14272
H	+3.05341	+1.88420	−0.59584
O	+1.46377	−0.11950	+2.60722
C	+3.69833	−0.26744	+0.89239
H	+2.07391	−0.36187	−0.51166
H	+1.49625	+2.05468	−0.11119
C	+3.77359	−1.77168	+0.95177
H	+4.47145	+0.11864	+0.21677
H	+3.86823	+0.17655	+1.87752
C	+4.08884	−2.50778	−0.18595
C	+4.13245	−3.89439	−0.15987
C	+3.85804	−4.56860	+1.02402
C	+3.54208	−3.85069	+2.17148
C	+3.50027	−2.46714	+2.12897
H	+4.31816	−1.98777	−1.11420
H	+4.38725	−4.45405	−1.05678
O	+3.88850	−5.92404	+1.11398
H	+3.33217	−4.39291	+3.08722

H	+3.24167	−1.90767	+3.02359
H	+4.12358	−6.30742	+0.26818

14 Arg

14.1 Reference Z-matrix for Arg

Bond distances are given in Å; bond and dihedral angles, in degrees.

```
h
o    1  oh2
c    2  co3      1  coh3
c    3  cc4      2  cco4      1  dih4
n    4  nc5      3  ncc5      2  dih5
h    5  hn6      4  hnc6      3  dih6
o    3  oc7      2  oco7      4  dih7
c    4  cc8      3  ccc8      5  dih8
h    4  hc9      3  hcc9      5  dih9
h    5  hn10     4  hnc10     6  dih10
c    8  cc11     4  ccc11     3  dih11
h    8  hc12     4  hcc12    11  dih12
h    8  hc13     4  hcc13    11  dih13
c   11  cc14     8  ccc14     4  dih14
h   11  hc15     8  hcc15    14  dih15
h   11  hc16     8  hcc16    14  dih16
n   14  nc17    11  ncc17     8  dih17
h   14  hc18    11  hcc18    17  dih18
h   14  hc19    11  hcc19    17  dih19
c   17  cn20    14  cnc20    11  dih20
h   17  hn21    14  hnc21    20  dih21
n   20  nc22    17  ncn22    14  dih22
n   20  nc23    22  ncn23    17  dih23
h   22  hn24    20  hnc24    23  dih24
h   23  hn25    20  hnc25    22  dih25
h   23  hn26    20  hnc26    25  dih26
```

oh2	0.994975
co3	1.373572
coh3	113.013
cc4	1.530224
cco4	109.176
dih4	181.454
nc5	1.481107
ncc5	105.630

dih5	122.539
hn6	1.018117
hnc6	111.503
dih6	67.383
oc7	1.205666
oco7	120.563
dih7	181.577
cc8	1.542738
ccc8	108.112
dih8	124.806
hc9	1.127470
hcc9	108.779
dih9	244.182
hn10	1.015523
hnc10	110.927
dih10	120.386
cc11	1.530565
ccc11	111.600
dih11	173.727
hc12	1.109501
hcc12	110.045
dih12	122.997
hc13	1.110114
hcc13	109.390
dih13	238.273
cc14	1.537919
ccc14	110.668
dih14	182.551
hc15	1.108640
hcc15	110.213
dih15	122.049
hc16	1.109067
hcc16	110.716
dih16	238.638
nc17	1.497564
ncc17	108.120
dih17	184.510
hc18	1.112434
hcc18	110.261
dih18	119.648

hc19	1.112903
hcc19	110.630
dih19	237.054
cn20	1.447738
cnc20	115.050
dih20	171.646
hn21	1.034856
hnc21	110.569
dih21	125.767
nc22	1.298738
ncn22	118.914
dih22	118.813
nc23	1.432319
ncn23	126.952
dih23	180.028
hn24	1.012986
hnc24	120.573
dih24	−7.833
hn25	1.013592
hnc25	116.381
dih25	159.734
hn26	1.011997
hnc26	115.590
dih26	226.748

14.2 Reference energy and target torsions for Arg

- Reference M08-HX/MG3S energy: -606.5841510 hartree
- Target torsions:
  - torsion1: dih4
  - torsion2: dih5
  - torsion3: dih6
  - torsion4: dih11
  - torsion5: dih14
  - torsion6: dih17

- torsion7: dih20
- torsion8: dih22
- torsion9: dih24

14.3 Properties of Arg conformers

Table S14: Relative energy (kcal mol^{-1}), rotational constants (B1 to B3, GHz) and dipolar moment (Debye) for the conformers of Arg located at the M08-HX/MG3S level.

Conformer	Rel. Energy	B1	B2	B3	Dip. Mom.
1	0.000	0.450	0.589	1.427	8.100
2	0.491	0.683	0.796	1.359	2.816
3	0.895	0.504	0.523	1.548	6.046
4	0.896	0.732	0.846	1.281	3.380
5	1.305	0.686	0.833	1.260	4.905
6	1.442	0.443	0.521	1.623	7.908
7	1.734	0.355	0.400	2.184	8.675
8	1.801	0.657	0.835	1.316	3.061
9	1.804	0.436	0.461	2.032	7.948
10	1.812	0.633	0.772	1.339	3.515
11	1.961	0.559	0.695	1.491	4.301
12	1.969	0.704	0.873	1.261	3.667
13	2.151	0.522	0.651	1.234	7.880
14	2.191	0.518	0.697	1.293	6.212
15	2.237	0.454	0.502	1.515	7.056
16	2.458	0.455	0.501	1.459	7.861
17	2.546	0.640	0.780	1.340	5.138
18	2.577	0.441	0.519	1.640	8.723
19	2.579	0.719	0.872	1.171	5.311
20	2.601	0.580	0.732	1.441	2.044
21	2.601	0.560	0.695	1.494	6.107
22	2.626	0.355	0.388	2.365	8.609
23	2.662	0.470	0.547	1.646	2.820
24	2.713	0.684	0.820	1.267	2.452
25	2.735	0.658	0.815	1.243	5.432
26	2.782	0.422	0.502	1.752	7.899

27	2.831	0.586	0.700	1.344	4.554
28	2.843	0.612	0.738	1.201	2.844
29	2.874	0.352	0.393	2.201	8.819
30	2.880	0.640	0.783	1.305	1.924
31	2.962	0.606	0.753	1.403	3.503
32	2.965	0.699	0.849	1.281	4.695
33	2.969	0.615	0.703	1.257	6.867
34	2.983	0.455	0.529	1.871	5.606
35	3.032	0.482	0.541	1.685	7.733
36	3.123	0.588	0.613	1.362	7.937
37	3.140	0.699	0.866	1.223	4.872
38	3.209	0.499	0.572	1.518	8.000
39	3.253	0.557	0.714	1.225	6.502
40	3.273	0.355	0.381	2.341	8.765
41	3.362	0.498	0.616	1.396	8.370
42	3.424	0.672	0.789	1.349	4.072
43	3.443	0.581	0.733	1.441	1.286
44	3.480	0.616	0.736	1.203	4.040
45	3.489	0.584	0.627	1.295	7.800
46	3.530	0.584	0.746	1.395	2.730
47	3.536	0.532	0.594	1.291	5.190
48	3.545	0.654	0.794	1.302	3.470
49	3.602	0.493	0.543	1.668	8.067
50	3.606	0.647	0.749	1.191	4.295
51	3.725	0.640	0.781	1.309	0.848
52	3.735	0.598	0.775	1.352	2.090
53	3.740	0.620	0.751	1.347	2.548
54	3.810	0.695	0.879	1.252	4.583
55	3.811	0.517	0.607	1.647	5.130
56	3.827	0.623	0.732	1.341	2.221
57	3.836	0.627	0.789	1.340	3.172
58	3.837	0.470	0.519	1.628	7.699
59	3.878	0.583	0.731	1.415	2.786
60	3.907	0.466	0.523	1.475	3.313
61	3.994	0.350	0.391	2.291	5.030
62	4.018	0.455	0.530	1.873	6.729
63	4.029	0.503	0.671	1.305	6.793

64	4.032	0.634	0.762	1.245	1.547
65	4.035	0.703	0.864	1.226	5.128
66	4.045	0.451	0.479	1.965	3.051
67	4.047	0.556	0.670	1.303	7.534
68	4.060	0.586	0.702	1.344	6.245
69	4.092	0.465	0.538	1.659	5.448
70	4.143	0.461	0.513	1.433	4.190
71	4.160	0.632	0.779	1.351	2.303
72	4.180	0.502	0.518	1.531	3.656
73	4.185	0.586	0.638	1.406	7.790
74	4.198	0.575	0.773	1.137	3.712
75	4.208	0.443	0.487	1.474	6.761
76	4.273	0.706	0.873	1.214	4.193
77	4.298	0.393	0.429	1.903	1.902
78	4.306	0.726	0.887	1.232	4.945
79	4.308	0.463	0.520	1.474	5.223
80	4.333	0.432	0.469	1.454	3.788
81	4.342	0.500	0.511	1.547	3.955
82	4.344	0.416	0.484	1.748	3.154
83	4.356	0.503	0.565	1.603	5.613
84	4.357	0.536	0.605	1.321	4.454
85	4.389	0.491	0.561	1.524	6.888
86	4.392	0.682	0.787	1.354	4.667
87	4.394	0.608	0.757	1.404	4.996
88	4.405	0.661	0.747	1.276	5.787
89	4.416	0.417	0.453	1.996	7.681
90	4.425	0.436	0.474	1.442	2.010
91	4.435	0.429	0.474	1.866	8.154
92	4.467	0.517	0.571	1.390	1.739
93	4.505	0.518	0.588	1.324	4.106
94	4.529	0.458	0.504	1.554	3.323
95	4.530	0.643	0.776	1.239	1.571
96	4.584	0.518	0.572	1.391	2.190
97	4.592	0.531	0.598	1.305	4.231
98	4.594	0.647	0.794	1.248	3.282
99	4.599	0.586	0.613	1.366	9.014
100	4.603	0.348	0.388	2.303	4.781

101	4.606	0.450	0.478	1.956	4.402
102	4.607	0.517	0.693	1.301	7.861
103	4.612	0.408	0.479	1.665	8.370
104	4.613	0.488	0.578	1.530	7.013
105	4.617	0.396	0.472	1.739	4.424
106	4.619	0.589	0.681	1.374	2.091
107	4.620	0.634	0.770	1.299	4.246
108	4.626	0.348	0.382	2.330	4.553
109	4.627	0.585	0.697	1.449	5.280
110	4.630	0.651	0.803	1.247	7.100
111	4.640	0.654	0.794	1.305	5.326
112	4.688	0.411	0.450	2.076	3.636
113	4.703	0.538	0.660	1.394	6.862
114	4.708	0.553	0.658	1.309	6.700
115	4.721	0.502	0.641	1.436	3.815
116	4.737	0.626	0.738	1.334	3.903
117	4.745	0.593	0.639	1.306	5.545
118	4.756	0.519	0.602	1.659	6.985
119	4.767	0.457	0.502	1.503	3.202
120	4.783	0.565	0.734	1.236	6.138
121	4.792	0.520	0.604	1.277	5.923
122	4.855	0.655	0.761	1.348	4.388
123	4.864	0.464	0.590	1.379	4.187
124	4.878	0.350	0.382	2.373	5.507
125	4.883	0.652	0.818	1.340	4.142
126	4.903	0.692	0.872	1.213	4.615
127	4.911	0.391	0.427	1.897	3.968
128	4.919	0.470	0.547	1.619	3.035
129	4.943	0.417	0.444	2.072	4.672
130	4.977	0.480	0.522	1.628	5.460
131	4.982	0.613	0.704	1.355	3.426
132	4.988	0.438	0.519	1.610	3.068
133	5.012	0.404	0.441	1.566	4.151
134	5.012	0.466	0.517	1.438	2.829
135	5.053	0.527	0.560	1.510	6.599
136	5.056	0.636	0.765	1.245	2.486
137	5.063	0.500	0.643	1.424	7.065

138	5.095	0.344	0.384	2.317	4.449
139	5.097	0.450	0.541	1.626	5.446
140	5.139	0.387	0.457	1.772	7.287
141	5.174	0.593	0.684	1.374	3.072
142	5.193	0.429	0.448	2.024	5.517
143	5.206	0.607	0.700	1.303	3.794
144	5.228	0.429	0.499	1.742	7.835
145	5.236	0.522	0.679	1.385	7.074
146	5.245	0.446	0.512	1.847	2.078
147	5.258	0.634	0.779	1.355	4.230
148	5.269	0.479	0.537	1.517	6.113
149	5.281	0.449	0.520	1.847	5.670
150	5.290	0.625	0.677	1.253	6.505
151	5.308	0.391	0.449	1.850	3.462
152	5.322	0.414	0.479	1.753	5.562
153	5.341	0.441	0.521	1.531	6.276
154	5.368	0.482	0.557	1.421	3.625
155	5.383	0.342	0.375	2.414	4.057
156	5.386	0.554	0.735	1.248	6.319
157	5.390	0.432	0.506	1.748	5.160
158	5.395	0.465	0.545	1.633	3.147
159	5.400	0.421	0.493	1.773	4.265
160	5.410	0.390	0.438	1.928	4.852
161	5.449	0.594	0.754	1.371	5.329
162	5.459	0.362	0.375	1.738	4.737
163	5.466	0.636	0.784	1.355	3.551
164	5.472	0.555	0.609	1.431	5.689
165	5.473	0.622	0.673	1.289	3.350
166	5.480	0.408	0.477	1.851	7.655
167	5.487	0.628	0.776	1.349	3.225
168	5.489	0.418	0.497	1.777	5.151
169	5.490	0.553	0.621	1.392	3.497
170	5.501	0.355	0.381	2.343	5.482
171	5.510	0.641	0.711	1.274	5.226
172	5.524	0.467	0.490	1.696	5.830
173	5.530	0.376	0.387	1.734	5.040
174	5.537	0.432	0.550	1.481	5.992

175	5.553	0.479	0.561	1.663	6.439
176	5.557	0.492	0.546	1.677	6.498
177	5.558	0.413	0.471	1.748	8.091
178	5.560	0.466	0.507	1.625	3.170
179	5.566	0.493	0.550	1.387	6.328
180	5.579	0.528	0.603	1.250	3.187
181	5.582	0.350	0.374	2.369	5.384
182	5.585	0.504	0.574	1.502	9.000
183	5.592	0.553	0.649	1.357	5.991
184	5.607	0.647	0.757	1.344	3.630
185	5.607	0.438	0.533	1.586	6.580
186	5.608	0.345	0.381	2.182	3.057
187	5.640	0.460	0.503	1.739	5.648
188	5.676	0.427	0.462	1.838	6.824
189	5.680	0.535	0.641	1.359	4.084
190	5.686	0.719	0.883	1.139	2.127
191	5.717	0.587	0.676	1.267	6.289
192	5.719	0.414	0.467	1.424	3.316
193	5.721	0.529	0.617	1.683	3.880
194	5.725	0.647	0.795	1.250	5.226
195	5.757	0.450	0.493	1.779	5.329
196	5.766	0.498	0.554	1.421	1.649
197	5.777	0.365	0.416	1.760	5.404
198	5.780	0.435	0.508	1.454	5.936
199	5.793	0.538	0.656	1.401	3.483
200	5.804	0.458	0.510	1.447	4.691
201	5.808	0.470	0.491	1.916	3.790
202	5.831	0.352	0.389	2.314	4.935
203	5.842	0.413	0.438	2.087	4.628
204	5.846	0.344	0.368	2.365	3.312
205	5.846	0.479	0.501	1.897	2.361
206	5.860	0.461	0.540	1.635	5.365
207	5.862	0.398	0.465	1.608	4.405
208	5.863	0.473	0.504	1.699	7.826
209	5.864	0.465	0.541	1.622	4.663
210	5.874	0.383	0.448	1.676	7.301
211	5.875	0.514	0.538	1.534	3.142

212	5.882	0.426	0.453	1.611	3.300
213	5.887	0.560	0.599	1.389	6.114
214	5.887	0.424	0.486	1.419	4.549
215	5.894	0.460	0.529	1.646	3.623
216	5.894	0.352	0.389	2.304	5.524
217	5.927	0.408	0.476	1.593	3.405
218	5.928	0.418	0.456	1.856	4.910
219	5.939	0.554	0.710	1.170	6.286
220	5.939	0.683	0.918	1.187	2.482
221	5.941	0.455	0.523	1.728	4.231
222	5.963	0.481	0.614	1.300	1.987
223	5.965	0.467	0.516	1.644	3.812
224	5.968	0.291	0.314	2.512	7.465
225	5.968	0.415	0.450	1.664	2.859
226	5.975	0.345	0.384	2.184	2.494
227	5.990	0.464	0.527	1.590	1.855
228	6.000	0.530	0.604	1.536	6.848
229	6.007	0.377	0.417	1.845	3.893
230	6.011	0.505	0.592	1.353	3.939
231	6.014	0.502	0.589	1.355	4.271
232	6.017	0.494	0.572	1.433	6.802
233	6.018	0.732	0.887	1.137	4.588
234	6.033	0.502	0.576	1.466	6.415
235	6.034	0.470	0.497	1.504	2.778
236	6.034	0.342	0.366	2.386	3.539
237	6.036	0.589	0.679	1.383	1.503
238	6.044	0.558	0.598	1.408	3.700
239	6.046	0.435	0.531	1.575	7.425
240	6.051	0.556	0.679	1.413	3.768
241	6.062	0.441	0.528	1.662	8.573
242	6.063	0.463	0.516	1.491	4.115
243	6.077	0.521	0.599	1.554	7.134
244	6.079	0.603	0.743	1.341	1.286
245	6.080	0.439	0.462	1.427	4.060
246	6.083	0.510	0.538	1.461	6.217
247	6.086	0.563	0.682	1.481	3.435
248	6.102	0.514	0.588	1.323	1.439

249	6.109	0.422	0.447	1.884	3.479
250	6.112	0.390	0.448	1.831	4.901
251	6.115	0.430	0.494	1.405	4.322
252	6.115	0.373	0.407	2.109	3.829
253	6.122	0.477	0.526	1.724	3.845
254	6.127	0.400	0.465	1.621	2.595
255	6.130	0.403	0.471	1.861	9.008
256	6.130	0.493	0.527	1.498	3.388
257	6.132	0.402	0.467	1.819	2.712
258	6.133	0.459	0.511	1.492	5.506
259	6.135	0.569	0.688	1.249	4.825
260	6.141	0.416	0.472	1.920	3.144
261	6.142	0.348	0.385	2.310	4.868
262	6.163	0.426	0.449	1.634	6.454
263	6.164	0.490	0.578	1.366	3.833
264	6.177	0.392	0.458	1.654	3.406
265	6.178	0.576	0.711	1.372	2.807
266	6.181	0.565	0.637	1.380	6.681
267	6.190	0.618	0.768	1.327	3.089
268	6.196	0.538	0.595	1.414	2.791
269	6.199	0.655	0.700	1.263	3.695
270	6.199	0.555	0.581	1.444	3.974
271	6.204	0.378	0.424	1.918	6.755
272	6.212	0.611	0.752	1.321	3.447
273	6.215	0.411	0.438	1.896	4.313
274	6.222	0.343	0.381	1.729	3.789
275	6.233	0.553	0.580	1.445	4.558
276	6.242	0.497	0.613	1.257	4.883
277	6.243	0.425	0.482	1.743	2.611
278	6.251	0.427	0.465	1.822	6.443
279	6.256	0.397	0.430	1.898	4.106
280	6.260	0.381	0.428	1.912	6.697
281	6.262	0.470	0.499	1.836	4.207
282	6.267	0.387	0.423	1.929	4.443
283	6.272	0.340	0.377	1.744	3.273
284	6.273	0.453	0.523	1.727	3.038
285	6.278	0.614	0.666	1.267	7.474

286	6.279	0.416	0.439	2.062	4.915
287	6.309	0.542	0.642	1.458	2.084
288	6.312	0.625	0.750	1.161	3.097
289	6.313	0.503	0.562	1.556	4.338
290	6.319	0.428	0.491	1.798	5.214
291	6.319	0.550	0.576	1.424	3.207
292	6.321	0.306	0.328	2.581	2.295
293	6.326	0.439	0.509	1.732	6.352
294	6.329	0.428	0.494	1.783	5.779
295	6.339	0.400	0.466	1.826	2.654
296	6.357	0.488	0.531	1.632	4.322
297	6.358	0.444	0.470	2.001	7.758
298	6.358	0.506	0.623	1.422	5.282
299	6.365	0.698	0.885	1.216	4.116
300	6.371	0.455	0.520	1.685	3.717
301	6.387	0.355	0.375	2.354	5.950
302	6.389	0.487	0.496	1.583	4.123
303	6.408	0.426	0.484	1.858	3.575
304	6.416	0.481	0.632	1.343	6.100
305	6.422	0.456	0.498	1.641	4.745
306	6.423	0.465	0.521	1.758	3.392
307	6.427	0.623	0.688	1.245	5.199
308	6.427	0.352	0.380	2.284	5.272
309	6.428	0.486	0.517	1.573	1.242
310	6.430	0.618	0.755	1.385	3.262
311	6.430	0.559	0.686	1.234	2.739
312	6.430	0.506	0.521	1.532	4.297
313	6.445	0.590	0.648	1.401	8.803
314	6.455	0.615	0.716	1.296	7.590
315	6.455	0.434	0.481	1.810	4.321
316	6.456	0.524	0.596	1.402	5.819
317	6.460	0.377	0.450	1.758	5.476
318	6.460	0.447	0.469	2.005	3.569
319	6.494	0.548	0.589	1.720	4.928
320	6.495	0.493	0.553	1.488	1.435
321	6.502	0.609	0.702	1.299	5.702
322	6.502	0.442	0.501	1.618	6.300

323	6.503	0.432	0.550	1.476	7.589
324	6.524	0.393	0.455	1.924	7.677
325	6.525	0.531	0.576	1.383	6.420
326	6.526	0.373	0.415	1.801	6.797
327	6.529	0.388	0.452	1.663	2.798
328	6.531	0.415	0.445	1.393	1.825
329	6.532	0.423	0.437	2.026	2.943
330	6.537	0.564	0.679	1.222	3.374
331	6.539	0.455	0.509	1.441	5.524
332	6.544	0.639	0.803	1.304	1.712
333	6.547	0.522	0.639	1.279	4.909
334	6.548	0.444	0.530	1.538	7.284
335	6.550	0.379	0.453	1.757	4.313
336	6.553	0.422	0.456	2.007	3.681
337	6.554	0.343	0.379	2.176	3.653
338	6.556	0.386	0.438	1.687	5.488
339	6.557	0.503	0.614	1.462	4.501
340	6.565	0.386	0.423	1.542	2.296
341	6.566	0.351	0.379	2.289	5.549
342	6.567	0.460	0.526	1.612	4.068
343	6.570	0.374	0.402	1.905	4.396
344	6.573	0.492	0.599	1.389	3.193
345	6.576	0.418	0.461	1.841	4.689
346	6.585	0.379	0.452	1.769	6.435
347	6.588	0.652	0.728	1.285	6.787
348	6.589	0.384	0.402	1.913	4.898
349	6.590	0.686	0.859	1.213	6.934
350	6.599	0.371	0.396	1.904	5.125
351	6.603	0.525	0.621	1.442	5.774
352	6.609	0.454	0.520	1.675	3.731
353	6.609	0.488	0.560	1.575	4.401
354	6.610	0.475	0.547	1.631	1.136
355	6.613	0.459	0.527	1.637	3.018
356	6.614	0.450	0.501	1.530	7.489
357	6.626	0.424	0.461	1.817	2.777
358	6.630	0.467	0.489	1.832	4.123
359	6.631	0.456	0.519	1.738	6.340

360	6.638	0.357	0.410	1.794	2.728
361	6.639	0.391	0.462	1.414	4.028
362	6.639	0.489	0.497	1.560	4.236
363	6.646	0.465	0.517	1.490	2.965
364	6.647	0.486	0.571	1.312	8.946
365	6.649	0.342	0.377	2.200	3.106
366	6.658	0.566	0.675	1.240	3.178
367	6.666	0.593	0.683	1.382	3.781
368	6.668	0.477	0.557	1.664	8.262
369	6.669	0.546	0.574	1.577	4.107
370	6.670	0.437	0.501	1.463	3.997
371	6.674	0.380	0.394	1.633	3.205
372	6.680	0.545	0.667	1.393	3.569
373	6.686	0.386	0.421	1.924	4.914
374	6.705	0.387	0.440	1.691	4.493
375	6.711	0.537	0.649	1.398	5.174
376	6.717	0.292	0.309	2.327	2.368
377	6.719	0.495	0.536	1.712	6.272
378	6.719	0.575	0.688	1.257	3.724
379	6.721	0.353	0.368	1.780	2.334
380	6.725	0.401	0.428	1.625	4.921
381	6.731	0.363	0.377	1.721	2.725
382	6.732	0.344	0.372	2.421	1.723
383	6.736	0.532	0.623	1.673	3.819
384	6.739	0.340	0.375	2.378	1.799
385	6.743	0.446	0.498	1.792	3.403
386	6.746	0.432	0.461	1.806	2.412
387	6.749	0.533	0.577	1.359	4.091
388	6.750	0.475	0.547	1.614	6.718
389	6.752	0.398	0.409	1.803	2.128
390	6.753	0.525	0.655	1.334	7.175
391	6.753	0.514	0.701	1.249	4.854
392	6.759	0.490	0.574	1.721	2.271
393	6.760	0.668	0.810	1.184	5.172
394	6.762	0.416	0.464	1.382	4.714
395	6.770	0.462	0.509	1.679	2.661
396	6.772	0.616	0.694	1.289	4.628

397	6.774	0.476	0.524	1.728	3.384
398	6.776	0.379	0.448	1.501	4.945
399	6.782	0.376	0.396	1.909	3.461
400	6.783	0.436	0.441	1.479	3.036
401	6.786	0.464	0.480	1.742	4.136
402	6.793	0.487	0.638	1.336	4.982
403	6.796	0.434	0.496	1.469	4.100
404	6.799	0.352	0.400	1.615	3.290
405	6.801	0.403	0.441	1.877	2.823
406	6.802	0.397	0.405	1.886	7.718
407	6.802	0.321	0.329	1.827	3.433
408	6.807	0.377	0.396	1.899	3.227
409	6.808	0.305	0.327	2.579	2.220
410	6.810	0.455	0.497	1.619	4.375
411	6.816	0.502	0.619	1.377	9.965
412	6.820	0.510	0.579	1.568	5.578
413	6.823	0.562	0.679	1.225	3.158
414	6.824	0.448	0.518	1.861	6.741
415	6.829	0.436	0.486	1.485	4.393
416	6.831	0.528	0.602	1.554	3.898
417	6.839	0.531	0.603	1.386	6.520
418	6.840	0.628	0.777	1.350	5.080
419	6.841	0.437	0.489	1.754	6.498
420	6.843	0.395	0.444	1.847	3.991
421	6.849	0.324	0.345	2.038	3.670
422	6.849	0.364	0.378	1.712	3.626
423	6.857	0.390	0.452	1.931	7.680
424	6.864	0.380	0.454	1.768	4.927
425	6.871	0.646	0.827	1.222	4.355
426	6.878	0.536	0.574	1.413	5.516
427	6.884	0.592	0.724	1.404	3.763
428	6.896	0.305	0.327	2.568	2.050
429	6.900	0.344	0.386	1.669	3.578
430	6.901	0.349	0.365	1.824	2.020
431	6.902	0.453	0.501	1.485	2.712
432	6.906	0.429	0.472	1.514	3.696
433	6.906	0.540	0.656	1.408	2.862

434	6.917	0.417	0.499	1.641	7.750
435	6.918	0.422	0.449	1.617	1.954
436	6.920	0.513	0.614	1.419	5.521
437	6.922	0.503	0.646	1.392	4.183
438	6.922	0.291	0.301	2.472	7.827
439	6.924	0.557	0.611	1.429	7.201
440	6.928	0.437	0.500	1.459	3.430
441	6.929	0.442	0.514	1.669	8.138
442	6.934	0.626	0.737	1.250	5.159
443	6.944	0.533	0.606	1.528	4.531
444	6.959	0.518	0.591	1.448	5.934
445	6.960	0.426	0.538	1.467	2.337
446	6.961	0.303	0.339	1.815	3.625
447	6.962	0.439	0.467	1.975	5.447
448	6.965	0.411	0.442	1.669	3.528
449	6.969	0.439	0.503	1.448	3.342
450	6.977	0.317	0.339	2.164	3.422
451	6.978	0.428	0.446	2.025	5.535
452	6.980	0.415	0.478	1.809	3.011
453	6.989	0.342	0.383	1.674	2.702
454	6.991	0.321	0.348	2.213	3.847
455	6.993	0.387	0.448	1.807	3.720
456	6.997	0.324	0.331	1.811	1.658
457	7.003	0.508	0.621	1.436	4.935
458	7.009	0.491	0.504	1.546	4.133
459	7.011	0.666	0.708	1.283	4.949
460	7.016	0.455	0.492	1.849	5.130
461	7.018	0.500	0.517	1.562	6.037
462	7.018	0.456	0.503	1.487	3.066
463	7.021	0.503	0.555	1.511	3.154
464	7.023	0.421	0.477	1.748	5.148
465	7.028	0.502	0.569	1.429	3.533
466	7.037	0.305	0.313	2.344	3.057
467	7.041	0.385	0.455	1.479	5.285
468	7.051	0.436	0.489	1.735	5.027
469	7.052	0.393	0.406	1.886	4.865
470	7.058	0.551	0.706	1.170	4.374

471	7.061	0.382	0.438	1.703	6.273
472	7.063	0.420	0.458	1.365	3.284
473	7.067	0.549	0.606	1.446	2.906
474	7.070	0.613	0.704	1.248	8.458
475	7.074	0.369	0.380	1.736	2.526
476	7.075	0.424	0.499	1.748	2.318
477	7.076	0.406	0.470	1.698	2.804
478	7.076	0.343	0.385	1.799	3.462
479	7.082	0.347	0.393	1.632	2.430
480	7.085	0.478	0.522	1.702	4.332
481	7.091	0.427	0.540	1.461	2.031
482	7.092	0.517	0.590	1.319	4.164
483	7.095	0.482	0.493	1.650	4.368
484	7.096	0.331	0.365	1.978	3.863
485	7.097	0.449	0.502	1.638	2.416
486	7.100	0.443	0.527	1.528	6.067
487	7.102	0.501	0.519	1.555	6.390
488	7.102	0.429	0.546	1.468	4.970
489	7.103	0.415	0.484	1.559	2.098
490	7.106	0.372	0.417	1.790	4.058
491	7.107	0.256	0.269	2.688	3.524
492	7.113	0.615	0.750	1.327	5.608
493	7.126	0.357	0.392	2.006	2.331
494	7.126	0.559	0.596	1.364	5.291
495	7.128	0.599	0.655	1.397	4.471
496	7.131	0.432	0.473	1.619	4.242
497	7.134	0.349	0.385	2.050	4.058
498	7.137	0.659	0.745	1.226	3.765
499	7.147	0.443	0.482	1.554	4.337
500	7.151	0.524	0.574	1.375	1.755
501	7.158	0.386	0.426	1.534	2.351
502	7.162	0.382	0.439	1.710	4.352
503	7.162	0.378	0.396	1.952	7.602
504	7.166	0.443	0.471	1.951	1.828
505	7.167	0.463	0.527	1.493	8.436
506	7.184	0.453	0.530	1.475	2.840
507	7.185	0.424	0.472	1.894	7.574

508	7.186	0.592	0.638	1.311	3.916
509	7.190	0.443	0.506	1.735	7.846
510	7.193	0.409	0.446	1.851	3.225
511	7.194	0.651	0.757	1.307	4.164
512	7.198	0.420	0.450	1.832	3.591
513	7.198	0.426	0.510	1.724	4.735
514	7.199	0.560	0.671	1.506	4.021
515	7.209	0.507	0.597	1.671	5.703
516	7.214	0.613	0.759	1.323	4.439
517	7.215	0.464	0.535	1.479	7.922
518	7.215	0.365	0.377	1.777	2.220
519	7.215	0.367	0.423	1.809	3.873
520	7.216	0.591	0.751	1.302	3.519
521	7.216	0.417	0.431	1.709	2.382
522	7.219	0.314	0.319	1.931	3.102
523	7.219	0.368	0.411	1.819	4.359
524	7.224	0.310	0.331	2.137	3.799
525	7.226	0.293	0.312	2.461	3.431
526	7.229	0.306	0.316	2.321	2.408
527	7.232	0.495	0.558	1.485	3.934
528	7.234	0.463	0.512	1.570	5.497
529	7.235	0.541	0.621	1.449	4.643
530	7.235	0.339	0.375	1.738	4.162
531	7.238	0.397	0.464	1.776	7.958
532	7.241	0.567	0.693	1.410	5.266
533	7.243	0.290	0.307	2.321	1.782
534	7.245	0.306	0.342	1.794	3.810
535	7.253	0.384	0.425	1.557	2.180
536	7.258	0.476	0.534	1.530	2.945
537	7.260	0.446	0.468	1.995	4.460
538	7.261	0.433	0.463	1.835	7.440
539	7.261	0.479	0.589	1.536	7.333
540	7.261	0.392	0.411	1.811	3.526
541	7.263	0.399	0.429	1.795	7.030
542	7.272	0.312	0.318	1.982	3.416
543	7.272	0.346	0.388	1.799	3.791
544	7.273	0.474	0.515	1.635	3.812

545	7.281	0.418	0.487	1.774	5.844
546	7.283	0.463	0.480	1.741	3.113
547	7.285	0.622	0.766	1.317	5.226
548	7.285	0.416	0.490	1.658	3.063
549	7.288	0.392	0.399	1.914	6.239
550	7.295	0.371	0.383	1.729	4.282
551	7.298	0.422	0.479	1.540	4.211
552	7.298	0.631	0.717	1.213	5.628
553	7.304	0.288	0.310	2.497	4.577
554	7.306	0.412	0.475	1.817	3.235
555	7.307	0.420	0.433	1.710	1.021
556	7.307	0.427	0.451	1.636	4.638
557	7.310	0.321	0.349	2.212	3.223
558	7.312	0.257	0.271	2.682	2.761
559	7.313	0.555	0.633	1.306	3.241
560	7.313	0.420	0.477	1.551	4.430
561	7.313	0.375	0.428	1.695	3.744
562	7.318	0.392	0.454	1.646	1.934
563	7.320	0.455	0.524	1.741	1.190
564	7.322	0.458	0.546	1.368	3.325
565	7.335	0.501	0.593	1.275	2.644
566	7.343	0.395	0.443	1.847	3.463
567	7.346	0.619	0.770	1.309	4.699
568	7.349	0.380	0.418	1.570	3.242
569	7.351	0.287	0.308	2.541	4.806
570	7.353	0.401	0.465	1.836	4.345
571	7.356	0.576	0.703	1.203	3.688
572	7.357	0.495	0.532	1.714	3.363
573	7.357	0.478	0.537	1.673	3.900
574	7.361	0.491	0.551	1.632	4.630
575	7.368	0.386	0.459	1.423	3.510
576	7.371	0.296	0.331	1.880	3.841
577	7.373	0.433	0.474	1.593	4.489
578	7.373	0.346	0.366	2.372	3.078
579	7.377	0.425	0.477	1.697	3.211
580	7.384	0.485	0.549	1.458	2.771
581	7.392	0.483	0.511	1.575	3.029

582	7.393	0.483	0.546	1.652	5.195
583	7.395	0.374	0.389	1.986	6.098
584	7.398	0.503	0.523	1.542	5.187
585	7.399	0.511	0.594	1.369	2.585
586	7.401	0.631	0.679	1.234	1.634
587	7.402	0.336	0.374	2.325	1.580
588	7.402	0.361	0.373	1.728	4.244
589	7.403	0.376	0.394	1.880	4.152
590	7.405	0.401	0.458	1.695	2.406
591	7.406	0.478	0.500	1.601	3.885
592	7.406	0.404	0.437	1.412	2.614
593	7.407	0.438	0.457	1.408	1.555
594	7.408	0.369	0.401	1.846	6.973
595	7.410	0.426	0.474	1.855	4.791
596	7.413	0.355	0.366	1.783	2.604
597	7.414	0.537	0.615	1.448	4.612
598	7.415	0.347	0.364	2.019	5.633
599	7.417	0.420	0.447	1.603	4.568
600	7.417	0.340	0.376	1.737	4.465
601	7.422	0.463	0.589	1.383	5.453
602	7.425	0.520	0.600	1.297	5.067
603	7.432	0.440	0.462	1.410	2.135
604	7.433	0.358	0.367	1.739	3.984
605	7.439	0.368	0.376	1.683	3.062
606	7.443	0.423	0.459	1.829	2.935
607	7.445	0.366	0.390	1.784	2.097
608	7.449	0.505	0.555	1.610	2.781
609	7.451	0.423	0.445	1.888	1.451
610	7.457	0.539	0.638	1.460	2.256
611	7.458	0.565	0.618	1.258	4.932
612	7.459	0.399	0.460	1.617	4.010
613	7.462	0.492	0.631	1.408	3.782
614	7.464	0.435	0.463	1.409	1.398
615	7.473	0.397	0.435	1.884	3.400
616	7.473	0.502	0.521	1.552	6.025
617	7.474	0.453	0.512	1.506	6.784
618	7.474	0.598	0.734	1.367	3.739

619	7.475	0.524	0.633	1.236	2.363
620	7.476	0.314	0.335	2.158	3.196
621	7.477	0.453	0.494	1.512	4.133
622	7.478	0.315	0.324	1.962	5.449
623	7.479	0.293	0.310	2.728	2.332
624	7.479	0.566	0.638	1.445	5.918
625	7.479	0.310	0.319	1.983	3.362
626	7.484	0.443	0.517	1.497	3.201
627	7.485	0.465	0.508	1.559	0.988
628	7.486	0.349	0.390	2.267	4.480
629	7.491	0.407	0.479	1.705	2.386
630	7.493	0.354	0.386	2.310	4.672
631	7.495	0.419	0.432	1.706	2.883
632	7.498	0.426	0.479	1.482	7.591
633	7.502	0.472	0.530	1.535	3.202
634	7.504	0.361	0.384	1.812	2.277
635	7.504	0.420	0.459	1.428	6.006
636	7.505	0.484	0.574	1.514	4.454
637	7.510	0.433	0.480	1.552	4.415
638	7.512	0.289	0.306	2.349	1.803
639	7.513	0.351	0.388	2.171	5.190
640	7.515	0.347	0.372	1.968	7.520
641	7.517	0.332	0.367	1.973	4.096
642	7.523	0.360	0.402	1.853	7.195
643	7.524	0.498	0.613	1.250	7.328
644	7.526	0.453	0.497	1.491	2.841
645	7.527	0.358	0.372	1.751	4.223
646	7.528	0.525	0.550	1.450	3.934
647	7.530	0.354	0.386	2.322	5.340
648	7.530	0.349	0.389	2.281	5.273
649	7.532	0.474	0.523	1.392	5.445
650	7.541	0.462	0.516	1.457	3.756
651	7.542	0.458	0.485	1.797	3.706
652	7.543	0.371	0.378	1.651	4.168
653	7.545	0.343	0.364	1.829	3.504
654	7.546	0.313	0.318	1.988	1.106
655	7.546	0.345	0.365	2.391	2.863

656	7.567	0.399	0.463	1.845	4.426
657	7.568	0.415	0.425	1.747	5.899
658	7.568	0.395	0.461	1.647	3.347
659	7.568	0.346	0.371	1.949	6.082
660	7.569	0.468	0.548	1.710	6.069
661	7.577	0.387	0.446	1.791	3.281
662	7.580	0.390	0.432	1.764	3.912
663	7.586	0.422	0.436	1.683	3.074
664	7.594	0.354	0.391	1.737	4.049
665	7.600	0.468	0.506	1.415	2.373
666	7.600	0.378	0.420	1.983	5.574
667	7.602	0.398	0.409	1.852	3.759
668	7.606	0.575	0.669	1.270	2.896
669	7.613	0.301	0.323	2.626	7.957
670	7.615	0.472	0.596	1.449	5.041
671	7.617	0.379	0.419	1.592	3.104
672	7.618	0.616	0.670	1.338	7.010
673	7.624	0.610	0.781	1.400	4.647
674	7.627	0.426	0.470	1.909	1.673
675	7.630	0.410	0.478	1.794	5.894
676	7.633	0.308	0.329	2.153	5.436
677	7.634	0.511	0.554	1.434	4.023
678	7.638	0.317	0.324	1.969	4.830
679	7.639	0.383	0.398	1.941	1.329
680	7.644	0.587	0.717	1.264	6.718
681	7.645	0.321	0.344	2.177	5.981
682	7.645	0.549	0.574	1.465	3.481
683	7.646	0.386	0.422	1.562	3.869
684	7.649	0.508	0.607	1.474	4.961
685	7.649	0.392	0.425	1.475	7.423
686	7.651	0.460	0.570	1.523	2.320
687	7.651	0.430	0.547	1.465	4.885
688	7.654	0.442	0.470	1.886	1.759
689	7.659	0.361	0.392	1.861	5.138
690	7.662	0.600	0.685	1.277	3.970
691	7.663	0.416	0.430	1.696	1.818
692	7.664	0.632	0.763	1.144	2.177

693	7.665	0.480	0.524	1.704	4.045
694	7.668	0.422	0.472	1.847	2.025
695	7.671	0.408	0.441	1.858	3.527
696	7.674	0.375	0.416	1.968	4.331
697	7.675	0.471	0.542	1.632	3.090
698	7.676	0.509	0.593	1.449	3.369
699	7.677	0.401	0.431	1.734	2.799
700	7.679	0.419	0.472	1.500	3.314
701	7.680	0.530	0.545	1.451	4.064
702	7.681	0.306	0.332	2.328	6.049
703	7.681	0.411	0.487	1.663	1.848
704	7.685	0.288	0.308	2.492	4.314
705	7.686	0.397	0.416	1.831	2.724
706	7.687	0.355	0.389	2.012	3.597
707	7.692	0.457	0.503	1.490	1.155
708	7.694	0.345	0.381	2.070	3.828
709	7.700	0.354	0.388	2.027	3.807
710	7.706	0.352	0.388	1.948	6.609
711	7.707	0.566	0.663	1.400	2.461
712	7.710	0.489	0.521	1.849	0.906
713	7.711	0.309	0.314	2.288	7.433
714	7.715	0.446	0.499	1.756	1.606
715	7.718	0.534	0.656	1.355	7.649
716	7.720	0.346	0.382	2.052	3.862
717	7.722	0.617	0.735	1.210	2.389
718	7.722	0.416	0.456	1.841	2.960
719	7.732	0.373	0.386	1.679	4.025
720	7.735	0.494	0.538	1.651	2.153
721	7.735	0.562	0.635	1.443	5.557
722	7.738	0.490	0.597	1.573	2.313
723	7.745	0.427	0.480	1.518	4.752
724	7.750	0.384	0.418	1.574	6.701
725	7.752	0.488	0.530	1.690	1.428
726	7.753	0.296	0.333	1.957	5.018
727	7.759	0.388	0.451	1.884	7.438
728	7.763	0.367	0.406	2.026	4.583
729	7.764	0.353	0.395	1.887	5.435

730	7.765	0.430	0.460	1.824	3.072
731	7.766	0.415	0.466	1.716	2.237
732	7.768	0.401	0.432	1.454	7.587
733	7.768	0.576	0.682	1.259	5.211
734	7.775	0.438	0.465	1.946	1.495
735	7.775	0.284	0.307	2.209	4.837
736	7.777	0.455	0.500	1.491	2.248
737	7.779	0.499	0.582	1.365	2.529
738	7.784	0.293	0.313	2.475	4.978
739	7.785	0.340	0.386	1.913	4.013
740	7.790	0.450	0.490	1.762	6.082
741	7.797	0.423	0.470	1.896	8.333
742	7.798	0.358	0.404	2.001	7.283
743	7.799	0.289	0.300	2.434	6.522
744	7.802	0.453	0.460	1.462	2.516
745	7.804	0.359	0.373	1.750	2.322
746	7.809	0.365	0.378	1.730	3.703
747	7.810	0.415	0.429	1.707	1.968
748	7.810	0.518	0.613	1.447	7.792
749	7.816	0.286	0.309	2.207	2.769
750	7.817	0.411	0.457	1.921	6.981
751	7.817	0.304	0.311	2.091	4.757
752	7.821	0.288	0.298	2.481	6.711
753	7.821	0.548	0.573	1.468	4.927
754	7.822	0.298	0.310	2.718	3.439
755	7.822	0.357	0.375	1.900	2.354
756	7.823	0.361	0.384	1.798	1.575
757	7.825	0.547	0.621	1.247	3.882
758	7.826	0.303	0.309	2.093	5.321
759	7.827	0.388	0.447	1.658	4.010
760	7.828	0.326	0.341	1.901	2.340
761	7.831	0.408	0.441	1.684	1.387
762	7.835	0.421	0.473	1.490	4.723
763	7.837	0.488	0.509	1.640	4.418
764	7.838	0.350	0.363	2.015	3.946
765	7.838	0.287	0.307	2.530	4.356
766	7.842	0.351	0.369	1.910	2.088

767	7.844	0.395	0.410	1.829	3.568
768	7.852	0.420	0.474	1.504	2.714
769	7.853	0.549	0.622	1.325	1.629
770	7.857	0.414	0.501	1.692	4.787
771	7.868	0.444	0.506	1.511	1.905
772	7.874	0.552	0.581	1.609	2.726
773	7.876	0.502	0.570	1.495	3.930
774	7.876	0.486	0.536	1.643	1.910
775	7.879	0.480	0.551	1.386	1.881
776	7.885	0.292	0.308	2.719	2.957
777	7.891	0.354	0.387	1.761	4.873
778	7.897	0.438	0.476	1.382	2.080
779	7.898	0.308	0.324	2.593	3.306
780	7.900	0.618	0.757	1.382	4.964
781	7.902	0.398	0.414	1.847	3.287
782	7.902	0.421	0.473	1.530	4.698
783	7.903	0.415	0.488	1.619	7.779
784	7.906	0.346	0.381	2.098	4.123
785	7.908	0.569	0.630	1.450	3.781
786	7.908	0.379	0.419	1.755	3.670
787	7.912	0.345	0.379	1.960	4.507
788	7.913	0.479	0.526	1.684	2.280
789	7.915	0.481	0.577	1.398	4.154
790	7.921	0.400	0.472	1.728	2.083
791	7.921	0.575	0.701	1.323	4.421
792	7.922	0.333	0.360	2.077	7.164
793	7.922	0.506	0.605	1.356	7.671
794	7.923	0.469	0.529	1.682	3.500
795	7.924	0.384	0.402	1.886	4.475
796	7.927	0.446	0.474	1.870	1.428
797	7.928	0.363	0.385	2.015	1.661
798	7.928	0.411	0.459	1.582	3.704
799	7.930	0.332	0.360	2.044	5.377
800	7.932	0.593	0.731	1.393	3.872
801	7.939	0.251	0.265	2.879	6.136
802	7.941	0.362	0.377	1.903	3.315
803	7.941	0.413	0.425	1.751	6.347

804	7.943	0.366	0.387	1.950	4.801
805	7.947	0.411	0.470	1.863	4.440
806	7.948	0.379	0.414	1.605	6.726
807	7.951	0.308	0.334	2.314	7.484
808	7.953	0.396	0.462	1.389	1.473
809	7.956	0.284	0.308	2.210	4.467
810	7.956	0.525	0.601	1.476	2.755
811	7.958	0.362	0.367	1.878	1.513
812	7.962	0.294	0.323	2.509	6.783
813	7.963	0.365	0.388	1.772	1.627
814	7.964	0.350	0.365	1.803	2.818
815	7.968	0.629	0.733	1.165	3.560
816	7.972	0.463	0.517	1.654	1.787
817	7.972	0.461	0.499	1.528	3.692
818	7.975	0.346	0.362	1.929	2.522
819	7.978	0.364	0.401	1.699	2.303
820	7.980	0.321	0.342	2.043	3.710
821	7.981	0.349	0.351	1.770	3.593
822	7.981	0.356	0.397	1.881	5.312
823	7.997	0.407	0.464	1.600	7.483
824	7.999	0.363	0.376	1.698	4.298
825	8.004	0.435	0.473	1.881	2.165
826	8.009	0.449	0.516	1.401	1.294
827	8.013	0.405	0.431	1.929	3.495
828	8.018	0.413	0.450	1.849	1.478
829	8.020	0.477	0.557	1.416	6.009
830	8.022	0.346	0.380	1.713	1.559
831	8.023	0.353	0.387	2.063	2.135
832	8.026	0.353	0.396	1.631	4.395
833	8.027	0.383	0.446	1.567	2.806
834	8.027	0.629	0.750	1.245	2.860
835	8.029	0.544	0.682	1.332	3.560
836	8.031	0.373	0.407	2.076	4.124
837	8.031	0.441	0.500	1.432	7.014
838	8.034	0.422	0.495	1.775	5.329
839	8.034	0.388	0.407	1.866	4.672
840	8.035	0.303	0.322	2.639	3.321

841	8.042	0.482	0.542	1.519	3.779
842	8.042	0.386	0.454	1.845	8.258
843	8.043	0.351	0.353	1.772	1.961
844	8.044	0.471	0.588	1.442	4.731
845	8.045	0.250	0.267	2.877	6.151
846	8.046	0.314	0.320	1.904	5.803
847	8.051	0.373	0.390	1.933	2.158
848	8.055	0.477	0.517	1.399	7.383
849	8.057	0.595	0.764	1.357	1.359
850	8.059	0.411	0.441	1.897	2.564
851	8.060	0.405	0.439	1.515	1.886
852	8.061	0.346	0.384	1.680	4.311
853	8.062	0.443	0.467	1.440	4.068
854	8.068	0.355	0.378	2.290	4.921
855	8.076	0.345	0.386	2.244	5.312
856	8.079	0.311	0.331	2.088	3.651
857	8.081	0.579	0.704	1.197	4.284
858	8.084	0.420	0.454	1.807	7.314
859	8.098	0.355	0.392	1.717	3.556
860	8.100	0.476	0.496	1.598	3.195
861	8.101	0.346	0.381	1.679	1.924
862	8.101	0.399	0.421	1.755	2.542
863	8.102	0.413	0.452	1.405	4.240
864	8.103	0.374	0.408	1.604	7.747
865	8.103	0.511	0.565	1.574	2.712
866	8.103	0.473	0.597	1.471	1.593
867	8.105	0.507	0.566	1.482	3.870
868	8.109	0.557	0.656	1.349	2.413
869	8.109	0.415	0.499	1.714	5.207
870	8.111	0.465	0.511	1.462	6.004
871	8.112	0.427	0.448	1.408	2.572
872	8.113	0.450	0.509	1.681	1.875
873	8.113	0.447	0.486	1.398	3.201
874	8.114	0.461	0.506	1.470	5.392
875	8.117	0.365	0.382	1.822	3.637
876	8.117	0.459	0.509	1.605	1.911
877	8.120	0.396	0.469	1.745	2.284

878	8.121	0.370	0.419	1.844	4.228
879	8.121	0.533	0.623	1.348	4.083
880	8.123	0.432	0.514	1.589	4.427
881	8.124	0.363	0.394	2.220	3.752
882	8.125	0.394	0.441	1.584	3.666
883	8.127	0.372	0.392	1.908	4.188
884	8.130	0.344	0.385	2.249	4.518
885	8.134	0.367	0.399	1.620	6.829
886	8.135	0.428	0.483	1.501	3.980
887	8.135	0.369	0.404	1.636	7.855
888	8.136	0.432	0.470	1.417	5.857
889	8.138	0.341	0.358	1.822	3.378
890	8.138	0.295	0.308	2.349	4.426
891	8.139	0.313	0.316	1.924	5.652
892	8.139	0.528	0.557	1.438	1.940
893	8.140	0.337	0.369	1.881	1.976
894	8.141	0.457	0.571	1.376	3.822
895	8.143	0.562	0.663	1.345	3.486
896	8.147	0.385	0.419	1.760	2.189
897	8.148	0.360	0.374	1.729	4.185
898	8.151	0.423	0.449	1.495	3.676
899	8.152	0.346	0.362	1.907	3.093
900	8.152	0.510	0.531	1.426	6.103
901	8.157	0.420	0.453	1.842	2.316
902	8.157	0.308	0.330	2.135	3.014
903	8.159	0.316	0.350	2.290	2.655
904	8.161	0.360	0.397	2.040	4.392
905	8.162	0.417	0.443	1.648	3.123
906	8.163	0.314	0.333	2.079	3.072
907	8.166	0.424	0.480	1.475	2.945
908	8.167	0.513	0.598	1.447	3.171
909	8.167	0.337	0.370	1.887	3.728
910	8.168	0.289	0.314	2.084	3.975
911	8.169	0.688	0.868	1.217	6.579
912	8.171	0.456	0.519	1.573	4.865
913	8.173	0.430	0.484	1.490	4.024
914	8.176	0.515	0.614	1.418	2.835

915	8.177	0.306	0.307	2.300	5.780
916	8.181	0.289	0.312	2.625	4.397
917	8.181	0.340	0.361	1.829	2.404
918	8.183	0.544	0.575	1.413	4.755
919	8.193	0.561	0.599	1.370	2.125
920	8.199	0.491	0.514	1.525	4.793
921	8.201	0.381	0.412	1.911	5.508
922	8.204	0.547	0.678	1.324	6.396
923	8.209	0.489	0.596	1.269	2.839
924	8.213	0.489	0.607	1.261	4.141
925	8.219	0.320	0.340	2.068	3.339
926	8.219	0.361	0.366	1.793	0.582
927	8.223	0.414	0.466	1.550	2.433
928	8.225	0.359	0.376	1.840	3.488
929	8.234	0.357	0.394	2.059	6.307
930	8.234	0.348	0.366	1.903	2.946
931	8.245	0.574	0.621	1.259	5.116
932	8.249	0.612	0.763	1.361	2.437
933	8.252	0.507	0.607	1.438	7.051
934	8.252	0.525	0.556	1.432	1.997
935	8.256	0.392	0.400	1.894	2.481
936	8.260	0.363	0.396	1.652	6.832
937	8.261	0.316	0.322	1.953	2.480
938	8.261	0.321	0.345	2.165	1.369
939	8.261	0.345	0.382	1.664	2.779
940	8.264	0.313	0.321	1.952	1.367
941	8.264	0.352	0.390	1.741	3.730
942	8.265	0.406	0.440	1.413	1.427
943	8.271	0.421	0.497	1.342	1.596
944	8.275	0.375	0.438	1.735	8.459
945	8.275	0.408	0.470	1.841	4.664
946	8.275	0.325	0.349	2.154	2.421
947	8.278	0.304	0.314	2.773	7.629
948	8.280	0.404	0.456	1.473	3.918
949	8.289	0.434	0.462	1.456	4.262
950	8.290	0.537	0.653	1.394	1.319
951	8.290	0.586	0.629	1.329	4.994

952	8.293	0.338	0.358	1.869	4.271
953	8.297	0.450	0.500	1.518	5.171
954	8.297	0.316	0.335	2.035	2.215
955	8.300	0.389	0.452	1.876	8.369
956	8.301	0.535	0.591	1.357	2.847
957	8.302	0.305	0.338	1.790	3.756
958	8.312	0.324	0.339	1.909	3.102
959	8.313	0.338	0.356	1.865	2.649
960	8.313	0.439	0.475	1.351	4.095
961	8.318	0.384	0.418	1.558	3.885
962	8.319	0.293	0.306	2.336	3.560
963	8.320	0.270	0.290	2.510	5.084
964	8.321	0.481	0.571	1.300	4.657
965	8.322	0.376	0.386	1.658	4.826
966	8.327	0.483	0.505	1.581	1.543
967	8.329	0.306	0.323	2.597	4.391
968	8.331	0.352	0.391	1.611	3.050
969	8.331	0.291	0.297	2.426	2.628
970	8.333	0.335	0.343	1.830	3.603
971	8.333	0.346	0.361	1.831	2.961
972	8.336	0.368	0.399	1.987	6.084
973	8.338	0.292	0.320	2.324	5.806
974	8.347	0.392	0.418	1.490	8.068
975	8.351	0.495	0.559	1.435	1.718
976	8.354	0.583	0.668	1.262	1.641
977	8.355	0.540	0.578	1.492	4.468
978	8.356	0.289	0.316	2.090	4.001
979	8.359	0.297	0.321	2.315	4.565
980	8.360	0.322	0.332	1.827	3.904
981	8.360	0.343	0.376	1.682	3.300
982	8.360	0.490	0.548	1.519	4.631
983	8.362	0.411	0.452	1.846	1.832
984	8.362	0.429	0.477	1.689	2.768
985	8.363	0.431	0.476	1.693	2.801
986	8.363	0.419	0.486	1.785	5.357
987	8.365	0.308	0.336	2.384	5.119
988	8.371	0.413	0.451	1.378	3.845

989	8.373	0.390	0.403	1.873	4.467
990	8.374	0.343	0.359	1.908	1.000
991	8.375	0.387	0.459	1.751	5.048
992	8.376	0.509	0.553	1.441	2.752
993	8.377	0.609	0.744	1.197	2.665
994	8.377	0.396	0.419	1.483	8.157
995	8.380	0.301	0.320	2.641	4.648
996	8.381	0.474	0.505	1.852	3.648
997	8.384	0.474	0.565	1.599	4.204
998	8.386	0.448	0.513	1.506	3.685
999	8.388	0.411	0.416	1.621	5.167
1000	8.390	0.448	0.473	1.412	3.186
1001	8.394	0.385	0.422	1.557	3.387
1002	8.396	0.338	0.367	1.713	3.412
1003	8.397	0.494	0.546	1.654	3.550
1004	8.398	0.374	0.414	1.776	4.816
1005	8.403	0.381	0.415	1.569	8.028
1006	8.408	0.369	0.400	1.893	2.660
1007	8.409	0.299	0.321	2.607	6.906
1008	8.409	0.442	0.472	1.425	3.422
1009	8.415	0.458	0.492	1.411	4.861
1010	8.415	0.347	0.375	2.004	2.851
1011	8.417	0.366	0.378	1.875	4.825
1012	8.420	0.365	0.377	1.736	4.311
1013	8.421	0.306	0.323	2.588	4.587
1014	8.427	0.291	0.298	2.522	3.574
1015	8.427	0.374	0.434	1.917	7.371
1016	8.429	0.403	0.448	1.652	4.780
1017	8.430	0.487	0.511	1.529	4.828
1018	8.430	0.363	0.393	2.219	4.659
1019	8.433	0.376	0.396	1.986	3.807
1020	8.433	0.433	0.461	1.414	3.044
1021	8.436	0.433	0.439	1.557	0.970
1022	8.436	0.434	0.455	1.415	3.495
1023	8.438	0.358	0.380	1.843	3.856
1024	8.438	0.305	0.327	2.189	2.468
1025	8.442	0.303	0.328	2.403	4.777

1026	8.447	0.483	0.537	1.694	3.482
1027	8.447	0.415	0.481	1.831	3.414
1028	8.448	0.363	0.387	1.821	3.863
1029	8.449	0.519	0.645	1.377	4.394
1030	8.455	0.591	0.724	1.406	5.371
1031	8.459	0.387	0.398	1.909	3.915
1032	8.461	0.401	0.426	1.795	3.373
1033	8.462	0.313	0.336	2.466	3.175
1034	8.467	0.356	0.361	1.922	3.054
1035	8.467	0.672	0.782	1.222	4.404
1036	8.468	0.602	0.741	1.357	1.544
1037	8.473	0.302	0.312	2.312	3.268
1038	8.473	0.476	0.591	1.508	3.619
1039	8.474	0.606	0.742	1.362	1.585
1040	8.475	0.397	0.403	1.736	3.761
1041	8.476	0.359	0.364	1.889	2.946
1042	8.478	0.302	0.321	2.619	4.609
1043	8.478	0.351	0.387	1.762	4.862
1044	8.487	0.383	0.422	1.575	3.225
1045	8.488	0.309	0.341	1.763	1.922
1046	8.488	0.294	0.311	2.457	2.091
1047	8.491	0.342	0.378	1.678	4.384
1048	8.492	0.407	0.431	1.801	2.337
1049	8.492	0.433	0.490	1.471	2.862
1050	8.493	0.454	0.486	1.932	4.812
1051	8.496	0.389	0.460	1.765	5.757
1052	8.496	0.477	0.519	1.639	4.508
1053	8.497	0.375	0.435	1.577	1.583
1054	8.498	0.496	0.549	1.467	1.829
1055	8.504	0.423	0.446	1.656	5.220
1056	8.507	0.357	0.379	2.008	2.601
1057	8.508	0.299	0.321	2.601	6.785
1058	8.509	0.362	0.387	2.060	3.138
1059	8.510	0.363	0.376	1.754	4.193
1060	8.511	0.464	0.480	1.956	4.010
1061	8.514	0.362	0.402	2.137	4.035
1062	8.515	0.411	0.434	1.764	3.778

1063	8.522	0.646	0.788	1.195	2.208
1064	8.524	0.420	0.489	1.539	0.612
1065	8.526	0.353	0.360	1.781	3.406
1066	8.527	0.444	0.483	1.760	5.449
1067	8.528	0.312	0.331	2.088	3.059
1068	8.534	0.365	0.405	1.968	3.888
1069	8.536	0.474	0.528	1.545	5.231
1070	8.536	0.369	0.405	1.570	4.008
1071	8.536	0.375	0.413	1.596	2.968
1072	8.538	0.498	0.513	1.361	3.508
1073	8.539	0.302	0.311	2.411	4.769
1074	8.541	0.319	0.337	1.918	3.455
1075	8.541	0.417	0.470	1.835	3.777
1076	8.541	0.395	0.416	1.715	2.618
1077	8.546	0.394	0.444	1.645	3.390
1078	8.547	0.367	0.373	1.737	2.817
1079	8.548	0.321	0.335	1.948	3.292
1080	8.551	0.417	0.483	1.648	5.326
1081	8.551	0.425	0.493	1.514	4.039
1082	8.552	0.359	0.376	1.822	4.510
1083	8.559	0.360	0.375	1.749	4.025
1084	8.560	0.379	0.427	1.732	3.386
1085	8.560	0.364	0.372	1.766	2.110
1086	8.561	0.417	0.437	1.452	7.766
1087	8.566	0.388	0.425	1.521	3.221
1088	8.568	0.341	0.358	1.877	3.236
1089	8.571	0.293	0.315	2.055	3.227
1090	8.573	0.391	0.438	1.667	3.153
1091	8.577	0.387	0.455	1.800	3.550
1092	8.579	0.483	0.502	1.684	2.901
1093	8.580	0.249	0.265	2.980	4.888
1094	8.583	0.394	0.455	1.640	0.788
1095	8.583	0.493	0.507	1.566	3.792
1096	8.587	0.366	0.404	1.680	2.226
1097	8.590	0.258	0.268	2.673	3.813
1098	8.591	0.370	0.404	1.875	0.936
1099	8.592	0.495	0.556	1.556	3.083

1100	8.592	0.368	0.406	1.975	2.445
1101	8.594	0.527	0.628	1.384	4.412
1102	8.599	0.300	0.307	2.162	4.818
1103	8.599	0.316	0.333	2.081	2.187
1104	8.600	0.443	0.510	1.524	3.917
1105	8.603	0.484	0.543	1.503	5.600
1106	8.603	0.540	0.640	1.467	3.246
1107	8.603	0.352	0.375	2.108	3.706
1108	8.603	0.471	0.588	1.431	5.348
1109	8.605	0.380	0.434	1.728	4.857
1110	8.608	0.492	0.600	1.559	1.270
1111	8.609	0.345	0.374	1.989	2.043
1112	8.610	0.326	0.335	1.810	4.256
1113	8.612	0.488	0.543	1.368	3.766
1114	8.612	0.405	0.467	1.849	4.884
1115	8.615	0.280	0.298	2.272	4.684
1116	8.616	0.300	0.306	2.170	5.042
1117	8.617	0.396	0.400	1.623	4.957
1118	8.617	0.314	0.337	2.451	3.886
1119	8.623	0.431	0.507	1.714	5.794
1120	8.624	0.396	0.404	1.829	3.623
1121	8.627	0.362	0.380	1.807	4.583
1122	8.628	0.475	0.553	1.461	7.893
1123	8.629	0.368	0.414	1.982	2.577
1124	8.629	0.460	0.534	1.691	3.269
1125	8.636	0.367	0.413	1.982	2.507
1126	8.643	0.416	0.468	1.839	3.814
1127	8.648	0.495	0.575	1.554	4.753
1128	8.649	0.653	0.755	1.307	5.922
1129	8.651	0.465	0.587	1.369	4.260
1130	8.653	0.379	0.394	1.949	2.912
1131	8.654	0.476	0.511	1.752	3.695
1132	8.655	0.416	0.431	2.075	3.161
1133	8.655	0.560	0.622	1.204	2.640
1134	8.656	0.427	0.474	1.817	2.975
1135	8.661	0.520	0.565	1.457	3.728
1136	8.662	0.418	0.465	1.497	7.521

1137	8.662	0.490	0.630	1.405	3.846
1138	8.664	0.429	0.499	1.587	2.822
1139	8.665	0.340	0.369	1.746	1.834
1140	8.665	0.377	0.451	1.750	4.814
1141	8.667	0.509	0.521	1.427	4.833
1142	8.668	0.417	0.429	1.716	3.892
1143	8.668	0.463	0.516	1.552	7.579
1144	8.669	0.430	0.459	1.637	2.716
1145	8.670	0.344	0.378	2.111	2.351
1146	8.670	0.361	0.400	2.151	2.872
1147	8.674	0.365	0.403	1.700	2.061
1148	8.675	0.437	0.479	1.492	6.728
1149	8.678	0.518	0.642	1.388	4.450
1150	8.680	0.516	0.658	1.286	5.570
1151	8.681	0.371	0.381	2.016	3.264
1152	8.681	0.415	0.421	1.641	3.512
1153	8.683	0.359	0.392	2.278	3.833
1154	8.684	0.442	0.494	1.611	3.897
1155	8.685	0.353	0.360	1.791	4.057
1156	8.686	0.343	0.374	1.723	1.722
1157	8.690	0.367	0.392	2.158	4.216
1158	8.690	0.565	0.624	1.457	4.690
1159	8.693	0.432	0.451	1.438	4.249
1160	8.694	0.616	0.752	1.393	5.032
1161	8.694	0.373	0.383	1.682	7.004
1162	8.699	0.355	0.359	1.826	3.040
1163	8.700	0.467	0.570	1.308	4.306
1164	8.703	0.340	0.383	1.792	3.088
1165	8.705	0.335	0.358	2.031	2.637
1166	8.707	0.358	0.367	1.723	2.779
1167	8.707	0.372	0.410	1.618	3.251
1168	8.710	0.365	0.379	1.705	4.287
1169	8.711	0.385	0.423	1.544	3.462
1170	8.712	0.305	0.318	2.271	2.362
1171	8.715	0.316	0.349	2.287	3.344
1172	8.715	0.336	0.358	2.060	3.582
1173	8.716	0.344	0.382	1.678	3.124

1174	8.717	0.473	0.477	1.541	1.963
1175	8.717	0.319	0.335	1.920	3.242
1176	8.718	0.300	0.322	2.299	3.356
1177	8.720	0.417	0.454	1.839	2.058
1178	8.722	0.513	0.525	1.579	3.665
1179	8.723	0.502	0.590	1.430	3.650
1180	8.724	0.619	0.693	1.273	4.261
1181	8.724	0.417	0.429	1.715	5.205
1182	8.725	0.284	0.303	2.659	4.033
1183	8.729	0.418	0.472	1.855	2.176
1184	8.730	0.549	0.577	1.412	3.983
1185	8.731	0.664	0.751	1.263	7.988
1186	8.731	0.517	0.594	1.433	6.148
1187	8.733	0.344	0.379	2.092	2.315
1188	8.735	0.259	0.267	2.667	3.955
1189	8.736	0.597	0.620	1.260	4.276
1190	8.736	0.302	0.308	2.128	4.854
1191	8.739	0.373	0.382	1.715	4.271
1192	8.743	0.303	0.325	2.261	2.674
1193	8.745	0.447	0.512	1.378	2.083
1194	8.745	0.424	0.480	1.833	4.425
1195	8.747	0.605	0.783	1.388	4.807
1196	8.750	0.430	0.497	1.793	5.221
1197	8.750	0.394	0.410	1.859	2.538
1198	8.751	0.462	0.543	1.543	3.513
1199	8.752	0.371	0.386	2.036	3.817
1200	8.753	0.347	0.384	1.626	4.571
1201	8.753	0.289	0.296	2.417	3.548
1202	8.753	0.452	0.565	1.379	3.745
1203	8.754	0.271	0.290	2.524	3.699
1204	8.756	0.553	0.727	1.170	3.137
1205	8.758	0.342	0.376	1.985	2.668
1206	8.759	0.303	0.324	2.249	4.591
1207	8.761	0.426	0.440	1.636	3.759
1208	8.763	0.367	0.398	1.979	3.573
1209	8.764	0.358	0.394	2.056	7.592
1210	8.765	0.419	0.464	1.861	4.229

1211	8.765	0.357	0.371	2.105	3.178
1212	8.774	0.359	0.397	2.070	4.621
1213	8.774	0.337	0.342	1.965	3.989
1214	8.776	0.351	0.384	2.065	1.903
1215	8.776	0.382	0.451	1.822	3.907
1216	8.776	0.486	0.493	1.570	2.889
1217	8.780	0.427	0.440	1.435	7.722
1218	8.782	0.350	0.383	2.081	2.289
1219	8.782	0.545	0.576	1.622	4.262
1220	8.783	0.423	0.470	1.849	4.055
1221	8.784	0.317	0.338	2.179	3.173
1222	8.785	0.448	0.575	1.398	3.162
1223	8.785	0.344	0.359	1.868	2.664
1224	8.786	0.305	0.331	2.378	3.418
1225	8.788	0.370	0.418	1.770	2.653
1226	8.789	0.259	0.269	2.660	2.716
1227	8.789	0.357	0.380	1.830	2.631
1228	8.793	0.576	0.609	1.493	4.644
1229	8.793	0.407	0.463	1.535	7.067
1230	8.795	0.395	0.412	1.845	2.089
1231	8.800	0.614	0.752	1.372	1.299
1232	8.800	0.383	0.424	1.768	2.422
1233	8.800	0.411	0.478	1.802	6.293
1234	8.801	0.435	0.454	1.458	4.444
1235	8.801	0.487	0.558	1.347	7.004
1236	8.807	0.432	0.547	1.468	3.936
1237	8.808	0.442	0.484	1.803	4.080
1238	8.810	0.349	0.391	2.278	7.999
1239	8.811	0.431	0.439	1.608	3.228
1240	8.811	0.325	0.361	2.354	5.668
1241	8.813	0.395	0.421	1.865	4.256
1242	8.814	0.400	0.466	1.792	3.058
1243	8.816	0.426	0.536	1.477	1.006
1244	8.816	0.425	0.471	1.818	3.391
1245	8.833	0.304	0.318	2.181	2.807
1246	8.836	0.309	0.345	1.772	3.675
1247	8.837	0.350	0.354	1.877	2.554

1248	8.838	0.426	0.471	1.901	7.083
1249	8.843	0.361	0.385	2.089	3.485
1250	8.843	0.509	0.548	1.408	2.728
1251	8.843	0.550	0.617	1.410	2.930
1252	8.843	0.533	0.604	1.266	3.381
1253	8.844	0.371	0.415	1.672	6.890
1254	8.847	0.345	0.347	1.791	1.567
1255	8.848	0.309	0.332	2.139	4.306
1256	8.849	0.434	0.491	1.478	4.426
1257	8.849	0.521	0.559	1.482	3.564
1258	8.850	0.404	0.470	1.787	3.297
1259	8.851	0.529	0.580	1.379	2.780
1260	8.851	0.379	0.392	1.634	4.492
1261	8.851	0.420	0.469	1.853	4.152
1262	8.852	0.488	0.589	1.391	3.788
1263	8.854	0.292	0.310	2.440	4.645
1264	8.860	0.303	0.314	2.398	3.595
1265	8.864	0.291	0.304	2.349	4.736
1266	8.864	0.405	0.443	1.413	3.767
1267	8.866	0.296	0.311	2.470	4.505
1268	8.870	0.385	0.402	1.900	3.833
1269	8.877	0.379	0.415	1.765	4.095
1270	8.878	0.349	0.391	1.637	3.204
1271	8.879	0.407	0.425	1.602	3.833
1272	8.880	0.314	0.333	2.039	1.749
1273	8.883	0.386	0.414	1.686	3.587
1274	8.883	0.532	0.701	1.276	2.458
1275	8.887	0.386	0.435	1.696	2.621
1276	8.891	0.551	0.617	1.399	4.212
1277	8.894	0.474	0.528	1.543	6.828
1278	8.894	0.326	0.360	2.347	5.636
1279	8.895	0.338	0.355	1.823	4.489
1280	8.901	0.395	0.416	1.763	1.741
1281	8.903	0.305	0.321	2.118	2.789
1282	8.906	0.310	0.339	2.366	4.761
1283	8.910	0.448	0.514	1.521	4.589
1284	8.914	0.471	0.526	1.417	6.552

1285	8.918	0.467	0.552	1.522	5.363
1286	8.919	0.306	0.342	1.791	1.839
1287	8.920	0.437	0.476	1.388	3.307
1288	8.921	0.316	0.334	1.922	3.656
1289	8.922	0.459	0.517	1.555	2.970
1290	8.924	0.326	0.329	1.758	2.059
1291	8.926	0.567	0.665	1.398	4.170
1292	8.927	0.313	0.347	2.343	3.539
1293	8.927	0.408	0.484	1.719	5.012
1294	8.929	0.345	0.377	1.798	7.190
1295	8.930	0.282	0.299	2.267	3.360
1296	8.936	0.367	0.395	2.083	5.155
1297	8.940	0.290	0.309	2.473	4.575
1298	8.943	0.371	0.384	1.679	1.844
1299	8.947	0.614	0.775	1.355	2.763
1300	8.951	0.298	0.309	2.726	3.227
1301	8.952	0.310	0.330	2.113	1.958
1302	8.954	0.411	0.444	1.428	4.137
1303	8.963	0.589	0.720	1.410	4.284
1304	8.966	0.394	0.431	1.891	2.203
1305	8.968	0.407	0.442	1.438	3.547
1306	8.969	0.367	0.423	1.710	3.278
1307	8.969	0.388	0.395	1.598	3.189
1308	8.969	0.519	0.612	1.414	5.011
1309	8.973	0.445	0.456	1.420	2.763
1310	8.978	0.508	0.528	1.592	2.052
1311	8.978	0.490	0.545	1.553	3.594
1312	8.980	0.287	0.306	2.519	1.790
1313	8.981	0.419	0.442	1.614	3.131
1314	8.981	0.361	0.384	1.811	2.720
1315	8.984	0.413	0.427	1.723	1.283
1316	8.986	0.336	0.352	1.852	4.452
1317	8.986	0.375	0.384	1.996	3.937
1318	8.988	0.368	0.408	1.809	4.282
1319	8.988	0.322	0.326	1.778	1.110
1320	8.989	0.594	0.770	1.309	3.954
1321	8.989	0.313	0.332	2.102	3.334

1322	8.993	0.354	0.370	1.931	3.266
1323	8.994	0.288	0.294	2.456	3.742
1324	8.997	0.394	0.398	1.632	7.230
1325	8.999	0.412	0.482	1.653	4.161
1326	9.003	0.333	0.363	2.044	2.719
1327	9.004	0.447	0.496	1.726	3.452
1328	9.010	0.286	0.313	2.613	3.466
1329	9.011	0.461	0.513	1.560	7.445
1330	9.011	0.391	0.451	1.748	2.383
1331	9.013	0.289	0.297	2.502	2.671
1332	9.015	0.476	0.539	1.434	5.192
1333	9.015	0.373	0.430	1.724	2.911
1334	9.019	0.301	0.307	2.174	2.987
1335	9.019	0.333	0.351	1.904	3.743
1336	9.022	0.430	0.482	1.826	3.455
1337	9.024	0.384	0.398	1.907	4.051
1338	9.025	0.346	0.379	1.687	3.052
1339	9.028	0.427	0.486	1.845	2.666
1340	9.028	0.457	0.523	1.499	3.713
1341	9.029	0.376	0.389	1.971	4.033
1342	9.032	0.412	0.450	1.592	5.690
1343	9.033	0.514	0.620	1.446	4.986
1344	9.033	0.292	0.306	2.335	3.124
1345	9.034	0.459	0.515	1.441	6.055
1346	9.038	0.422	0.442	1.720	4.619
1347	9.040	0.305	0.322	2.112	3.792
1348	9.041	0.306	0.312	2.369	3.818
1349	9.042	0.451	0.515	1.419	2.209
1350	9.042	0.359	0.373	1.927	4.109
1351	9.045	0.339	0.356	1.923	2.729
1352	9.046	0.498	0.587	1.425	2.458
1353	9.048	0.366	0.412	1.851	2.177
1354	9.049	0.438	0.483	1.815	5.087
1355	9.052	0.403	0.467	1.752	2.794
1356	9.056	0.385	0.393	1.611	4.116
1357	9.058	0.509	0.525	1.488	5.288
1358	9.059	0.343	0.376	1.817	7.040

1359	9.059	0.574	0.765	1.184	2.194
1360	9.060	0.500	0.552	1.639	3.995
1361	9.061	0.306	0.333	1.856	1.773
1362	9.064	0.327	0.345	2.369	2.782
1363	9.067	0.304	0.310	2.100	3.801
1364	9.069	0.413	0.491	1.704	4.238
1365	9.070	0.334	0.340	1.978	1.954
1366	9.071	0.291	0.305	2.365	2.926
1367	9.075	0.351	0.373	2.120	4.091
1368	9.078	0.368	0.392	2.151	4.774
1369	9.080	0.345	0.352	1.853	4.478
1370	9.081	0.365	0.403	1.639	4.717
1371	9.083	0.348	0.384	2.182	4.169
1372	9.084	0.502	0.565	1.327	3.962
1373	9.085	0.390	0.451	1.774	2.795
1374	9.085	0.437	0.505	1.716	6.046
1375	9.090	0.488	0.592	1.577	5.127
1376	9.095	0.450	0.514	1.657	3.021
1377	9.097	0.396	0.420	1.814	3.771
1378	9.101	0.352	0.360	1.916	6.020
1379	9.101	0.353	0.371	1.933	4.085
1380	9.102	0.346	0.404	1.706	5.438
1381	9.105	0.256	0.270	2.640	1.882
1382	9.113	0.468	0.499	1.550	5.346
1383	9.113	0.611	0.758	1.375	2.486
1384	9.113	0.350	0.357	1.854	2.318
1385	9.114	0.286	0.303	2.609	3.595
1386	9.114	0.359	0.397	2.065	4.948
1387	9.116	0.581	0.728	1.387	5.506
1388	9.118	0.484	0.496	1.361	4.061
1389	9.121	0.288	0.311	2.129	3.651
1390	9.122	0.350	0.385	1.658	3.534
1391	9.123	0.286	0.310	2.127	2.197
1392	9.124	0.301	0.321	2.667	4.617
1393	9.125	0.290	0.303	2.374	4.789
1394	9.127	0.351	0.388	1.742	4.282
1395	9.128	0.288	0.295	2.541	3.062

1396	9.129	0.311	0.330	2.092	2.085
1397	9.131	0.285	0.305	2.557	1.885
1398	9.133	0.654	0.691	1.227	4.790
1399	9.136	0.396	0.446	1.671	6.299
1400	9.138	0.339	0.373	1.976	1.911
1401	9.138	0.370	0.406	1.559	6.716
1402	9.139	0.427	0.537	1.470	3.022
1403	9.143	0.512	0.673	1.345	4.242
1404	9.144	0.284	0.300	2.666	3.118
1405	9.147	0.319	0.326	1.865	4.304
1406	9.147	0.354	0.358	1.819	2.533
1407	9.148	0.451	0.479	1.966	2.835
1408	9.148	0.293	0.319	2.377	5.108
1409	9.152	0.285	0.311	2.129	2.257
1410	9.153	0.437	0.493	1.585	1.828
1411	9.154	0.303	0.316	2.205	3.840
1412	9.155	0.410	0.467	1.679	3.547
1413	9.159	0.360	0.396	1.716	3.679
1414	9.161	0.333	0.363	2.018	1.487
1415	9.163	0.286	0.312	2.613	2.746
1416	9.164	0.455	0.485	1.923	4.266
1417	9.166	0.537	0.628	1.211	7.542
1418	9.171	0.420	0.452	1.439	4.112
1419	9.172	0.417	0.464	1.931	5.075
1420	9.172	0.374	0.435	1.630	2.818
1421	9.174	0.446	0.485	1.394	4.930
1422	9.175	0.425	0.504	1.659	1.938
1423	9.180	0.407	0.454	1.624	3.860
1424	9.182	0.362	0.400	1.943	2.423
1425	9.182	0.426	0.451	1.410	3.609
1426	9.187	0.465	0.506	1.467	3.381
1427	9.188	0.440	0.475	1.819	4.449
1428	9.189	0.336	0.355	1.865	2.831
1429	9.189	0.625	0.737	1.373	4.903
1430	9.191	0.362	0.401	1.964	3.705
1431	9.191	0.395	0.403	1.758	4.327
1432	9.193	0.286	0.313	2.591	3.522

1433	9.199	0.426	0.493	1.506	3.350
1434	9.200	0.580	0.716	1.296	6.821
1435	9.200	0.475	0.511	1.647	4.718
1436	9.206	0.520	0.589	1.325	6.223
1437	9.208	0.358	0.399	1.852	2.889
1438	9.208	0.425	0.484	1.842	3.094
1439	9.214	0.307	0.320	2.008	4.798
1440	9.216	0.420	0.489	1.646	3.064
1441	9.223	0.358	0.397	2.169	2.781
1442	9.226	0.335	0.341	1.840	1.615
1443	9.226	0.399	0.471	1.840	5.679
1444	9.226	0.366	0.421	1.725	1.424
1445	9.226	0.328	0.335	1.785	4.773
1446	9.228	0.386	0.432	1.673	4.194
1447	9.231	0.416	0.477	1.812	4.667
1448	9.231	0.370	0.405	1.920	3.659
1449	9.233	0.284	0.302	2.659	3.898
1450	9.237	0.386	0.445	1.823	7.145
1451	9.246	0.460	0.541	1.535	4.185
1452	9.248	0.608	0.697	1.265	5.784
1453	9.248	0.448	0.493	1.503	2.471
1454	9.251	0.323	0.357	1.802	2.603
1455	9.252	0.306	0.315	2.369	4.478
1456	9.254	0.423	0.443	1.709	4.081
1457	9.254	0.419	0.441	1.702	7.493
1458	9.255	0.435	0.491	1.449	2.945
1459	9.255	0.370	0.414	1.694	7.806
1460	9.256	0.470	0.503	1.603	2.102
1461	9.260	0.401	0.436	1.471	4.480
1462	9.261	0.374	0.433	1.751	6.373
1463	9.262	0.366	0.428	1.921	3.914
1464	9.263	0.360	0.397	1.957	3.036
1465	9.266	0.303	0.332	1.873	2.830
1466	9.269	0.362	0.381	2.012	4.412
1467	9.273	0.302	0.309	2.343	1.895
1468	9.279	0.488	0.537	1.463	5.873
1469	9.279	0.304	0.322	2.245	2.480

1470	9.281	0.397	0.433	1.559	3.143
1471	9.281	0.429	0.484	1.483	3.198
1472	9.286	0.403	0.433	1.652	3.905
1473	9.289	0.342	0.368	1.905	7.386
1474	9.291	0.500	0.513	1.602	1.796
1475	9.292	0.308	0.322	2.004	2.721
1476	9.292	0.373	0.415	1.846	3.299
1477	9.293	0.346	0.359	1.947	1.939
1478	9.294	0.349	0.386	1.766	4.274
1479	9.297	0.334	0.351	1.900	2.736
1480	9.297	0.492	0.502	1.562	1.990
1481	9.299	0.488	0.574	1.513	7.497
1482	9.302	0.411	0.455	1.683	2.743
1483	9.304	0.248	0.267	2.988	5.152
1484	9.306	0.416	0.463	1.429	2.368
1485	9.308	0.420	0.472	1.518	4.343
1486	9.310	0.570	0.639	1.407	1.537
1487	9.311	0.295	0.328	2.014	4.841
1488	9.313	0.362	0.384	1.851	5.234
1489	9.315	0.501	0.535	1.720	5.208
1490	9.318	0.337	0.372	2.198	6.265
1491	9.319	0.353	0.359	2.091	2.624
1492	9.320	0.317	0.340	2.191	4.397
1493	9.320	0.319	0.345	2.249	4.401
1494	9.320	0.276	0.292	2.423	5.084
1495	9.322	0.518	0.562	1.376	2.955
1496	9.324	0.249	0.266	2.989	5.142
1497	9.326	0.402	0.435	1.810	3.138
1498	9.328	0.295	0.309	2.803	3.884
1499	9.330	0.448	0.482	1.492	2.248
1500	9.332	0.311	0.320	2.231	3.996
1501	9.332	0.415	0.437	1.702	3.765
1502	9.332	0.407	0.445	1.859	4.888
1503	9.334	0.404	0.460	1.577	2.830
1504	9.339	0.366	0.404	1.996	1.830
1505	9.342	0.394	0.466	1.795	5.352
1506	9.342	0.302	0.310	2.696	3.253

1507	9.343	0.469	0.517	1.501	2.648
1508	9.347	0.450	0.487	1.408	2.955
1509	9.348	0.309	0.344	1.774	3.238
1510	9.351	0.558	0.619	1.236	2.164
1511	9.362	0.419	0.468	1.667	5.971
1512	9.367	0.429	0.452	1.425	6.617
1513	9.377	0.321	0.351	2.137	4.305
1514	9.377	0.364	0.403	1.665	4.827
1515	9.377	0.346	0.365	2.220	7.118
1516	9.381	0.430	0.463	1.848	4.442
1517	9.382	0.295	0.303	2.397	5.388
1518	9.385	0.311	0.321	2.223	4.105
1519	9.386	0.437	0.493	1.468	4.323
1520	9.395	0.644	0.658	1.265	3.951
1521	9.396	0.575	0.707	1.392	7.504
1522	9.398	0.281	0.301	2.607	3.352
1523	9.399	0.302	0.324	2.519	3.537
1524	9.399	0.321	0.364	1.998	7.743
1525	9.399	0.375	0.421	1.736	4.384
1526	9.403	0.354	0.388	1.972	4.619
1527	9.404	0.514	0.534	1.581	3.838
1528	9.407	0.386	0.400	1.871	2.238
1529	9.409	0.612	0.757	1.372	2.477
1530	9.409	0.445	0.570	1.398	3.659
1531	9.412	0.348	0.362	2.098	2.886
1532	9.413	0.296	0.307	2.717	4.060
1533	9.418	0.455	0.501	1.593	4.195
1534	9.423	0.353	0.372	1.889	5.120
1535	9.424	0.441	0.472	1.555	3.460
1536	9.424	0.488	0.499	1.611	4.391
1537	9.425	0.514	0.591	1.439	7.891
1538	9.426	0.372	0.407	1.905	3.192
1539	9.428	0.298	0.331	1.989	3.435
1540	9.430	0.333	0.368	1.987	2.764
1541	9.432	0.442	0.463	1.428	5.319
1542	9.437	0.494	0.632	1.413	4.554
1543	9.438	0.369	0.426	1.688	3.783

1544	9.440	0.396	0.424	1.661	2.863
1545	9.442	0.258	0.273	2.640	4.256
1546	9.445	0.303	0.320	2.260	3.861
1547	9.446	0.304	0.318	2.524	3.067
1548	9.448	0.344	0.381	1.841	0.808
1549	9.449	0.398	0.433	1.690	4.245
1550	9.449	0.567	0.751	1.240	2.516
1551	9.456	0.399	0.482	1.475	6.423
1552	9.457	0.373	0.402	1.929	2.010
1553	9.458	0.386	0.403	1.867	2.182
1554	9.459	0.369	0.431	1.850	7.807
1555	9.462	0.361	0.394	1.648	2.972
1556	9.463	0.258	0.274	2.628	4.086
1557	9.463	0.410	0.432	1.452	4.431
1558	9.465	0.358	0.395	2.177	3.796
1559	9.467	0.371	0.401	1.940	2.369
1560	9.467	0.398	0.450	1.504	4.519
1561	9.469	0.376	0.409	1.804	2.929
1562	9.470	0.282	0.302	2.592	4.615
1563	9.471	0.336	0.358	2.227	6.090
1564	9.474	0.429	0.448	2.026	7.418
1565	9.479	0.427	0.483	1.487	3.694
1566	9.484	0.355	0.389	1.891	2.802
1567	9.485	0.396	0.405	1.861	5.161
1568	9.485	0.446	0.493	1.708	3.347
1569	9.488	0.440	0.476	1.352	1.215
1570	9.489	0.413	0.437	1.695	3.176
1571	9.491	0.424	0.436	1.536	3.157
1572	9.495	0.346	0.388	1.798	2.713
1573	9.496	0.409	0.430	1.754	4.456
1574	9.500	0.419	0.451	1.559	7.055
1575	9.502	0.450	0.484	1.770	4.215
1576	9.506	0.307	0.330	2.135	2.774
1577	9.506	0.419	0.440	1.702	3.129
1578	9.506	0.423	0.445	1.706	4.441
1579	9.508	0.395	0.467	1.846	6.020
1580	9.510	0.436	0.493	1.473	5.463

1581	9.511	0.402	0.465	1.594	2.451
1582	9.514	0.536	0.564	1.406	4.769
1583	9.515	0.401	0.435	1.469	4.755
1584	9.516	0.385	0.405	1.723	4.577
1585	9.517	0.551	0.576	1.406	5.516
1586	9.518	0.315	0.326	1.903	4.002
1587	9.524	0.453	0.521	1.513	5.972
1588	9.525	0.438	0.482	1.815	5.591
1589	9.528	0.420	0.442	1.699	4.032
1590	9.531	0.309	0.331	2.470	2.800
1591	9.535	0.343	0.379	1.842	2.590
1592	9.540	0.371	0.407	1.808	1.870
1593	9.541	0.481	0.567	1.527	5.067
1594	9.549	0.533	0.624	1.443	4.434
1595	9.549	0.380	0.414	1.580	5.149
1596	9.551	0.550	0.607	1.225	4.770
1597	9.556	0.349	0.367	2.172	7.206
1598	9.556	0.305	0.319	2.506	3.042
1599	9.557	0.315	0.322	1.958	4.457
1600	9.559	0.399	0.451	1.571	1.665
1601	9.560	0.408	0.430	1.450	4.324
1602	9.560	0.435	0.475	1.486	3.631
1603	9.562	0.330	0.354	2.157	3.413
1604	9.567	0.354	0.357	2.123	4.241
1605	9.570	0.388	0.438	1.540	2.838
1606	9.575	0.391	0.453	1.789	7.809
1607	9.575	0.338	0.379	2.276	1.792
1608	9.577	0.306	0.315	2.329	4.045
1609	9.579	0.350	0.357	1.744	2.404
1610	9.580	0.390	0.441	1.631	4.830
1611	9.580	0.378	0.427	1.828	2.442
1612	9.585	0.397	0.470	1.782	5.093
1613	9.585	0.348	0.381	2.323	7.564
1614	9.586	0.525	0.571	1.443	4.727
1615	9.590	0.444	0.552	1.564	2.648
1616	9.590	0.402	0.446	1.643	2.490
1617	9.590	0.310	0.329	2.104	2.098

1618	9.596	0.620	0.759	1.368	3.153
1619	9.597	0.432	0.458	1.443	5.264
1620	9.602	0.355	0.370	1.936	5.186
1621	9.602	0.352	0.357	2.057	4.760
1622	9.604	0.294	0.324	2.256	3.138
1623	9.607	0.376	0.450	1.529	3.491
1624	9.608	0.436	0.463	1.597	4.713
1625	9.609	0.342	0.391	1.831	7.898
1626	9.611	0.556	0.695	1.281	6.351
1627	9.612	0.257	0.272	2.660	4.005
1628	9.614	0.457	0.503	1.859	6.196
1629	9.621	0.577	0.663	1.304	4.873
1630	9.622	0.378	0.420	1.586	1.389
1631	9.623	0.455	0.480	1.916	3.665
1632	9.624	0.495	0.561	1.492	1.589
1633	9.625	0.346	0.403	1.694	7.288
1634	9.627	0.351	0.371	1.885	4.990
1635	9.629	0.295	0.321	2.363	4.781
1636	9.635	0.405	0.431	1.639	4.170
1637	9.638	0.509	0.569	1.565	2.189
1638	9.640	0.307	0.322	2.580	4.530
1639	9.640	0.477	0.510	1.494	6.001
1640	9.640	0.420	0.473	1.830	3.076
1641	9.642	0.288	0.310	2.108	1.708
1642	9.643	0.359	0.389	1.690	2.500
1643	9.643	0.341	0.354	2.306	4.158
1644	9.645	0.377	0.418	1.595	2.221
1645	9.650	0.387	0.419	1.927	3.352
1646	9.659	0.291	0.312	2.101	1.731
1647	9.659	0.379	0.418	1.621	3.077
1648	9.662	0.304	0.317	2.096	3.982
1649	9.663	0.373	0.397	2.066	4.872
1650	9.666	0.466	0.479	1.370	3.885
1651	9.666	0.399	0.426	1.683	2.622
1652	9.669	0.373	0.402	1.794	2.960
1653	9.672	0.315	0.332	2.086	3.321
1654	9.672	0.300	0.311	2.785	5.873

1655	9.675	0.352	0.359	1.740	3.940
1656	9.676	0.345	0.369	1.983	4.044
1657	9.677	0.289	0.311	2.105	1.771
1658	9.677	0.276	0.293	2.427	3.798
1659	9.679	0.439	0.527	1.489	1.701
1660	9.680	0.460	0.495	1.572	5.941
1661	9.681	0.411	0.433	1.743	4.382
1662	9.682	0.411	0.455	1.388	5.480
1663	9.683	0.305	0.328	2.184	2.935
1664	9.685	0.461	0.530	1.495	5.663
1665	9.685	0.373	0.397	2.065	4.646
1666	9.686	0.389	0.449	1.645	2.342
1667	9.687	0.364	0.376	1.725	2.746
1668	9.690	0.546	0.575	1.459	1.933
1669	9.699	0.358	0.381	1.851	4.923
1670	9.699	0.298	0.310	2.806	5.859
1671	9.703	0.402	0.440	1.863	4.770
1672	9.704	0.390	0.407	1.892	4.096
1673	9.705	0.370	0.379	1.700	2.240
1674	9.711	0.374	0.417	1.620	3.359
1675	9.713	0.500	0.621	1.223	2.247
1676	9.724	0.322	0.337	1.851	1.911
1677	9.726	0.374	0.387	1.649	2.674
1678	9.734	0.321	0.335	1.846	1.825
1679	9.736	0.556	0.593	1.401	4.234
1680	9.739	0.299	0.319	2.659	4.683
1681	9.740	0.367	0.392	2.186	1.986
1682	9.743	0.343	0.380	2.163	4.536
1683	9.748	0.435	0.462	1.423	4.774
1684	9.750	0.506	0.659	1.311	3.487
1685	9.750	0.454	0.483	1.471	1.989
1686	9.750	0.385	0.418	1.846	4.242
1687	9.755	0.443	0.465	1.407	4.696
1688	9.759	0.387	0.448	1.564	4.070
1689	9.762	0.399	0.438	1.678	2.844
1690	9.764	0.252	0.271	2.805	2.355
1691	9.765	0.593	0.761	1.346	3.378

1692	9.767	0.317	0.325	1.977	4.480
1693	9.774	0.299	0.320	2.658	4.777
1694	9.775	0.382	0.449	1.779	3.204
1695	9.778	0.440	0.454	1.427	2.124
1696	9.778	0.420	0.440	2.072	7.333
1697	9.780	0.370	0.420	1.811	3.068
1698	9.782	0.392	0.427	1.893	1.696
1699	9.787	0.404	0.452	1.627	6.638
1700	9.788	0.562	0.640	1.322	4.558
1701	9.790	0.422	0.467	1.860	5.271
1702	9.794	0.552	0.675	1.391	5.074
1703	9.798	0.278	0.297	2.450	3.393
1704	9.798	0.315	0.320	1.973	5.279
1705	9.805	0.518	0.646	1.464	4.196
1706	9.806	0.314	0.317	2.155	2.650
1707	9.808	0.326	0.345	2.102	3.597
1708	9.809	0.333	0.341	2.021	3.697
1709	9.812	0.344	0.376	1.688	4.097
1710	9.817	0.438	0.482	1.819	4.656
1711	9.819	0.364	0.375	1.746	2.203
1712	9.820	0.310	0.341	1.755	1.211
1713	9.826	0.493	0.554	1.573	4.630
1714	9.827	0.337	0.365	2.219	4.379
1715	9.829	0.297	0.326	2.231	3.102
1716	9.835	0.433	0.446	1.566	3.135
1717	9.837	0.351	0.382	2.366	8.115
1718	9.844	0.362	0.405	1.653	4.055
1719	9.846	0.368	0.408	1.868	1.998
1720	9.847	0.550	0.578	1.599	4.024
1721	9.855	0.325	0.346	1.953	5.571
1722	9.855	0.311	0.327	1.967	4.701
1723	9.862	0.664	0.754	1.222	4.091
1724	9.866	0.392	0.430	1.747	2.704
1725	9.866	0.315	0.333	2.465	3.810
1726	9.867	0.366	0.378	2.057	4.077
1727	9.870	0.382	0.408	1.612	7.781
1728	9.870	0.591	0.727	1.398	2.897

1729	9.874	0.295	0.309	2.437	2.508
1730	9.876	0.350	0.405	1.666	4.352
1731	9.880	0.414	0.435	1.740	3.332
1732	9.881	0.366	0.401	1.662	7.630
1733	9.882	0.301	0.317	2.087	6.696
1734	9.884	0.381	0.413	1.965	7.005
1735	9.888	0.339	0.372	1.905	7.999
1736	9.889	0.367	0.392	2.182	1.144
1737	9.890	0.423	0.438	1.606	4.633
1738	9.901	0.341	0.359	1.757	3.115
1739	9.901	0.402	0.446	1.629	3.375
1740	9.903	0.403	0.414	1.701	0.975
1741	9.904	0.404	0.457	1.839	8.228
1742	9.909	0.363	0.382	1.910	2.542
1743	9.911	0.355	0.408	1.758	4.461
1744	9.913	0.620	0.770	1.366	4.401
1745	9.920	0.365	0.390	1.812	2.676
1746	9.924	0.334	0.361	2.232	4.742
1747	9.926	0.398	0.420	1.523	6.697
1748	9.927	0.274	0.291	2.465	4.308
1749	9.929	0.370	0.380	1.694	2.697
1750	9.937	0.373	0.383	1.980	2.394
1751	9.937	0.604	0.748	1.362	2.842
1752	9.940	0.346	0.375	1.952	7.256
1753	9.941	0.488	0.575	1.496	7.006
1754	9.943	0.308	0.331	2.477	4.188
1755	9.944	0.350	0.364	1.866	3.393
1756	9.944	0.371	0.381	1.695	2.694
1757	9.946	0.394	0.428	1.586	4.323
1758	9.951	0.316	0.334	2.453	2.528
1759	9.954	0.439	0.492	1.422	3.100
1760	9.955	0.362	0.375	2.079	2.543
1761	9.960	0.294	0.308	2.470	2.325
1762	9.962	0.395	0.429	1.718	2.755
1763	9.962	0.427	0.475	1.820	2.248
1764	9.963	0.320	0.342	2.172	2.739
1765	9.963	0.375	0.425	1.998	5.752

1766	9.964	0.402	0.450	1.541	3.088
1767	9.969	0.405	0.470	1.796	2.348
1768	9.970	0.361	0.396	1.702	3.907
1769	9.972	0.311	0.321	1.963	2.978
1770	9.975	0.307	0.338	1.777	1.908
1771	9.979	0.395	0.421	1.514	6.327
1772	9.980	0.398	0.437	1.862	4.422
1773	9.981	0.383	0.448	1.797	3.917
1774	9.988	0.502	0.543	1.666	4.183
1775	9.989	0.329	0.347	1.917	4.337
1776	9.994	0.376	0.384	1.770	3.680
1777	9.994	0.523	0.556	1.420	1.534
1778	9.994	0.407	0.490	1.692	3.021
1779	10.000	0.400	0.465	1.800	3.734
1780	10.001	0.355	0.388	1.688	4.827
1781	10.003	0.385	0.420	1.547	4.677
1782	10.005	0.413	0.446	1.855	3.723
1783	10.005	0.342	0.347	1.980	3.281
1784	10.009	0.342	0.360	1.747	3.031
1785	10.010	0.394	0.417	1.584	4.642
1786	10.011	0.345	0.364	2.217	6.655
1787	10.017	0.390	0.396	1.900	4.662
1788	10.019	0.392	0.418	1.694	4.012
1789	10.020	0.390	0.396	1.625	4.318
1790	10.020	0.545	0.703	1.339	6.141
1791	10.021	0.371	0.420	1.835	4.504
1792	10.023	0.456	0.460	1.619	1.118
1793	10.023	0.489	0.595	1.563	3.974
1794	10.025	0.555	0.609	1.230	1.815
1795	10.027	0.616	0.746	1.383	1.240
1796	10.029	0.409	0.441	1.958	5.370
1797	10.031	0.310	0.328	2.545	4.375
1798	10.033	0.286	0.311	2.705	3.451
1799	10.035	0.344	0.374	1.981	3.171
1800	10.044	0.392	0.428	1.884	3.479
1801	10.046	0.361	0.384	2.187	3.440
1802	10.047	0.407	0.445	1.652	4.249

1803	10.047	0.513	0.537	1.575	3.330
1804	10.048	0.481	0.503	1.889	3.213
1805	10.053	0.436	0.487	1.791	2.196
1806	10.056	0.460	0.497	1.503	2.920
1807	10.057	0.323	0.344	2.168	3.980
1808	10.058	0.334	0.343	2.077	3.646
1809	10.060	0.438	0.493	1.429	3.156
1810	10.061	0.351	0.366	1.852	3.204
1811	10.063	0.413	0.446	1.426	1.987
1812	10.064	0.463	0.520	1.551	4.575
1813	10.065	0.481	0.493	1.372	4.296
1814	10.069	0.544	0.590	1.261	2.719
1815	10.069	0.419	0.503	1.699	3.558
1816	10.073	0.360	0.396	1.719	3.683
1817	10.073	0.473	0.531	1.419	4.166
1818	10.075	0.417	0.479	1.871	4.271
1819	10.076	0.396	0.400	1.591	3.831
1820	10.077	0.363	0.385	2.172	0.902
1821	10.080	0.345	0.367	2.081	3.990
1822	10.082	0.293	0.310	2.688	3.682
1823	10.084	0.375	0.415	1.654	3.191
1824	10.087	0.532	0.620	1.207	3.720
1825	10.089	0.433	0.457	1.975	4.471
1826	10.095	0.380	0.409	1.935	3.251
1827	10.096	0.436	0.492	1.494	4.570
1828	10.097	0.377	0.402	2.024	3.607
1829	10.102	0.405	0.424	1.782	6.345
1830	10.107	0.648	0.737	1.230	3.150
1831	10.112	0.344	0.378	1.674	4.429
1832	10.113	0.312	0.343	1.947	6.299
1833	10.114	0.337	0.361	1.883	7.628
1834	10.115	0.414	0.459	1.880	2.558
1835	10.115	0.535	0.591	1.512	5.000
1836	10.118	0.311	0.330	2.531	3.097
1837	10.122	0.417	0.428	1.624	5.924
1838	10.122	0.381	0.417	1.572	4.721
1839	10.123	0.329	0.346	1.929	4.180

1840	10.123	0.434	0.490	1.422	3.676
1841	10.127	0.401	0.449	1.544	2.773
1842	10.128	0.470	0.527	1.580	1.566
1843	10.129	0.377	0.386	1.761	3.850
1844	10.134	0.556	0.622	1.440	2.734
1845	10.136	0.325	0.362	1.919	3.925
1846	10.137	0.339	0.364	1.924	3.229
1847	10.144	0.334	0.344	2.012	1.410
1848	10.147	0.514	0.574	1.480	5.480
1849	10.153	0.413	0.447	1.432	2.109
1850	10.157	0.502	0.604	1.371	2.542
1851	10.161	0.362	0.398	2.000	2.120
1852	10.164	0.422	0.501	1.338	3.351
1853	10.166	0.410	0.443	1.446	4.298
1854	10.173	0.369	0.380	1.965	3.735
1855	10.174	0.349	0.408	1.692	3.909
1856	10.180	0.387	0.403	1.913	4.021
1857	10.183	0.301	0.310	2.428	5.161
1858	10.184	0.407	0.443	1.457	3.863
1859	10.184	0.386	0.430	1.607	4.423
1860	10.188	0.530	0.570	1.451	5.618
1861	10.194	0.440	0.478	1.340	4.716
1862	10.198	0.356	0.396	1.871	4.765
1863	10.198	0.286	0.311	2.703	4.714
1864	10.199	0.438	0.492	1.589	3.314
1865	10.203	0.349	0.358	1.908	6.266
1866	10.208	0.622	0.689	1.274	4.819
1867	10.210	0.386	0.401	1.930	3.891
1868	10.210	0.465	0.496	1.558	6.496
1869	10.211	0.508	0.537	1.447	4.859
1870	10.214	0.530	0.614	1.225	4.760
1871	10.219	0.377	0.424	1.550	4.298
1872	10.226	0.423	0.477	1.494	4.210
1873	10.230	0.323	0.332	1.769	4.273
1874	10.231	0.320	0.329	1.785	2.766
1875	10.233	0.345	0.353	1.854	3.324
1876	10.233	0.445	0.475	1.861	3.175

1877	10.235	0.328	0.352	2.156	4.116
1878	10.239	0.477	0.489	1.553	4.330
1879	10.242	0.292	0.301	2.384	5.154
1880	10.243	0.364	0.409	1.791	5.388
1881	10.246	0.294	0.314	2.091	1.683
1882	10.247	0.486	0.525	1.640	3.891
1883	10.247	0.423	0.448	1.506	4.689
1884	10.249	0.374	0.430	1.774	4.428
1885	10.252	0.285	0.297	2.736	2.589
1886	10.257	0.520	0.649	1.465	4.536
1887	10.264	0.503	0.523	1.538	1.792
1888	10.266	0.388	0.404	1.938	4.458
1889	10.270	0.378	0.434	1.577	3.060
1890	10.272	0.294	0.319	2.467	3.380
1891	10.272	0.461	0.519	1.554	4.594
1892	10.274	0.324	0.346	1.956	7.338
1893	10.278	0.287	0.313	2.153	7.229
1894	10.281	0.347	0.357	1.918	6.084
1895	10.282	0.257	0.268	2.651	2.715
1896	10.284	0.358	0.361	1.894	4.405
1897	10.285	0.434	0.477	1.705	4.751
1898	10.289	0.307	0.324	2.223	2.994
1899	10.293	0.378	0.414	1.670	4.285
1900	10.294	0.473	0.475	1.561	2.398
1901	10.295	0.473	0.497	1.893	2.611
1902	10.302	0.360	0.396	1.974	3.229
1903	10.303	0.291	0.300	2.416	5.145
1904	10.304	0.449	0.505	1.448	7.569
1905	10.310	0.439	0.495	1.431	5.088
1906	10.317	0.410	0.425	1.564	2.383
1907	10.318	0.490	0.568	1.502	5.761
1908	10.326	0.359	0.398	1.712	3.585
1909	10.328	0.358	0.394	1.988	1.681
1910	10.333	0.445	0.490	1.523	3.833
1911	10.338	0.516	0.623	1.491	4.846
1912	10.338	0.465	0.527	1.410	4.148
1913	10.339	0.352	0.375	2.357	7.802

1914	10.342	0.307	0.333	2.434	2.735
1915	10.343	0.418	0.500	1.347	3.531
1916	10.344	0.438	0.481	1.818	5.924
1917	10.347	0.389	0.422	1.703	3.073
1918	10.347	0.305	0.320	2.574	3.772
1919	10.348	0.384	0.428	1.768	2.752
1920	10.348	0.332	0.362	2.157	5.618
1921	10.349	0.302	0.311	2.183	3.360
1922	10.350	0.345	0.370	2.074	3.497
1923	10.357	0.493	0.604	1.445	3.347
1924	10.358	0.431	0.470	1.496	4.459
1925	10.364	0.328	0.351	2.172	3.824
1926	10.368	0.542	0.613	1.270	5.057
1927	10.369	0.306	0.323	2.237	3.816
1928	10.374	0.501	0.603	1.374	3.520
1929	10.381	0.274	0.291	2.704	2.966
1930	10.382	0.275	0.289	2.611	4.741
1931	10.386	0.360	0.363	1.867	4.392
1932	10.388	0.544	0.671	1.339	7.895
1933	10.391	0.366	0.400	1.960	3.151
1934	10.392	0.366	0.414	1.847	3.417
1935	10.392	0.387	0.409	1.555	4.231
1936	10.395	0.350	0.377	2.258	3.636
1937	10.396	0.469	0.525	1.586	2.257
1938	10.398	0.374	0.401	1.915	3.271
1939	10.400	0.410	0.432	1.762	1.808
1940	10.401	0.507	0.542	1.458	2.406
1941	10.402	0.404	0.422	1.789	7.559
1942	10.413	0.308	0.321	2.127	3.924
1943	10.415	0.350	0.373	2.095	4.108
1944	10.415	0.444	0.516	1.564	4.758
1945	10.417	0.415	0.430	1.709	3.251
1946	10.418	0.434	0.496	1.575	2.509
1947	10.419	0.510	0.554	1.490	5.460
1948	10.424	0.508	0.524	1.576	0.882
1949	10.425	0.514	0.556	1.453	5.165
1950	10.431	0.381	0.421	1.574	3.455

1951	10.441	0.511	0.596	1.386	5.177
1952	10.445	0.289	0.305	2.228	6.581
1953	10.446	0.381	0.398	1.867	3.285
1954	10.446	0.335	0.384	1.864	3.270
1955	10.447	0.493	0.537	1.538	4.693
1956	10.448	0.357	0.381	1.676	3.241
1957	10.449	0.647	0.656	1.272	3.920
1958	10.449	0.299	0.310	2.432	5.156
1959	10.450	0.407	0.448	1.610	4.314
1960	10.451	0.305	0.318	2.143	4.314
1961	10.456	0.310	0.322	1.864	2.890
1962	10.460	0.303	0.315	2.235	2.555
1963	10.462	0.394	0.443	1.927	3.148
1964	10.466	0.546	0.576	1.606	3.705
1965	10.468	0.391	0.449	1.851	3.372
1966	10.477	0.354	0.367	2.140	4.023
1967	10.478	0.304	0.318	2.155	2.873
1968	10.481	0.305	0.320	2.563	3.510
1969	10.497	0.471	0.555	1.554	5.505
1970	10.499	0.577	0.687	1.418	3.274
1971	10.501	0.389	0.437	1.931	3.833
1972	10.501	0.291	0.318	2.055	3.485
1973	10.505	0.380	0.421	1.574	4.322
1974	10.506	0.354	0.376	1.697	4.389
1975	10.507	0.300	0.318	2.345	1.723
1976	10.511	0.302	0.313	2.261	1.760
1977	10.512	0.524	0.555	1.416	2.732
1978	10.512	0.364	0.410	1.512	4.377
1979	10.520	0.367	0.398	1.967	0.733
1980	10.520	0.412	0.435	1.749	3.027
1981	10.521	0.331	0.351	1.871	2.866
1982	10.522	0.362	0.395	1.991	1.020
1983	10.524	0.381	0.414	1.896	1.444
1984	10.527	0.486	0.507	1.369	2.625
1985	10.530	0.393	0.409	1.888	5.796
1986	10.534	0.364	0.417	1.528	1.214
1987	10.543	0.399	0.452	1.750	4.000

1988	10.546	0.300	0.311	2.810	4.674
1989	10.548	0.501	0.532	1.492	4.433
1990	10.549	0.566	0.709	1.491	5.218
1991	10.549	0.521	0.560	1.382	3.604
1992	10.557	0.555	0.620	1.439	1.825
1993	10.559	0.364	0.375	1.752	3.972
1994	10.560	0.392	0.443	1.658	3.049
1995	10.561	0.367	0.414	1.491	3.969
1996	10.562	0.399	0.412	1.812	5.182
1997	10.564	0.319	0.321	1.967	4.504
1998	10.568	0.306	0.309	2.290	4.188
1999	10.569	0.353	0.384	1.911	1.427
2000	10.573	0.324	0.340	1.971	2.911
2001	10.574	0.484	0.561	1.421	5.548
2002	10.574	0.460	0.482	1.636	2.917
2003	10.578	0.440	0.488	1.410	2.093
2004	10.583	0.353	0.354	1.769	5.101
2005	10.587	0.420	0.457	1.630	4.608
2006	10.592	0.306	0.320	2.136	3.120
2007	10.600	0.350	0.409	1.679	5.350
2008	10.601	0.387	0.460	1.745	5.027
2009	10.602	0.358	0.404	1.664	4.476
2010	10.603	0.607	0.767	1.337	5.038
2011	10.604	0.369	0.398	1.888	2.759
2012	10.609	0.339	0.371	1.889	2.010
2013	10.609	0.359	0.394	1.830	6.891
2014	10.610	0.421	0.476	1.569	7.402
2015	10.610	0.414	0.429	1.715	4.436
2016	10.626	0.594	0.664	1.295	4.500
2017	10.630	0.365	0.403	1.658	7.707
2018	10.637	0.416	0.474	1.795	3.936
2019	10.647	0.315	0.328	1.921	3.007
2020	10.652	0.298	0.316	2.374	2.238
2021	10.655	0.402	0.444	1.639	2.897
2022	10.655	0.338	0.357	1.939	2.807
2023	10.656	0.391	0.437	1.695	4.668
2024	10.656	0.374	0.415	1.606	2.550

2025	10.659	0.446	0.490	1.524	4.343
2026	10.659	0.317	0.335	2.063	4.533
2027	10.663	0.308	0.327	2.165	5.894
2028	10.664	0.302	0.329	2.435	4.771
2029	10.664	0.369	0.424	1.772	4.739
2030	10.665	0.347	0.372	2.152	4.316
2031	10.667	0.389	0.390	1.734	4.144
2032	10.669	0.368	0.402	1.946	4.263
2033	10.672	0.395	0.454	1.753	4.558
2034	10.680	0.400	0.402	1.703	4.473
2035	10.685	0.437	0.467	1.545	3.740
2036	10.689	0.342	0.375	2.401	5.288
2037	10.695	0.355	0.387	1.887	2.975
2038	10.696	0.357	0.385	1.712	3.500
2039	10.696	0.373	0.414	1.613	3.292
2040	10.696	0.366	0.422	1.508	2.346
2041	10.699	0.308	0.319	2.282	3.072
2042	10.705	0.286	0.297	2.280	3.271
2043	10.716	0.364	0.422	1.709	2.552
2044	10.719	0.386	0.421	1.844	2.891
2045	10.720	0.308	0.319	2.291	4.594
2046	10.727	0.556	0.583	1.445	4.514
2047	10.727	0.299	0.315	2.358	3.088
2048	10.729	0.300	0.335	1.928	8.044
2049	10.731	0.361	0.382	1.925	1.410
2050	10.736	0.487	0.590	1.287	3.851
2051	10.737	0.302	0.315	2.132	3.026
2052	10.740	0.323	0.342	2.042	4.598
2053	10.748	0.481	0.556	1.517	3.879
2054	10.752	0.310	0.333	2.526	5.475
2055	10.755	0.507	0.535	1.413	5.749
2056	10.755	0.606	0.717	1.224	3.461
2057	10.756	0.352	0.358	1.716	4.229
2058	10.756	0.371	0.380	1.699	4.641
2059	10.762	0.460	0.478	1.383	4.187
2060	10.765	0.414	0.438	2.081	3.457
2061	10.766	0.310	0.317	2.186	3.891

2062	10.766	0.332	0.351	1.865	4.101
2063	10.772	0.476	0.560	1.539	3.192
2064	10.773	0.519	0.623	1.347	3.532
2065	10.776	0.305	0.329	1.844	4.778
2066	10.789	0.511	0.540	1.387	3.764
2067	10.792	0.385	0.441	1.666	3.486
2068	10.795	0.467	0.522	1.581	4.367
2069	10.796	0.365	0.387	1.816	4.916
2070	10.797	0.345	0.376	1.971	2.234
2071	10.798	0.413	0.482	1.647	3.972
2072	10.812	0.410	0.451	1.393	4.537
2073	10.814	0.456	0.484	1.660	4.084
2074	10.815	0.374	0.447	1.775	5.360
2075	10.816	0.272	0.296	2.463	7.030
2076	10.819	0.641	0.787	1.195	3.910
2077	10.821	0.302	0.307	2.199	5.471
2078	10.823	0.312	0.339	2.062	3.872
2079	10.824	0.389	0.412	1.932	4.429
2080	10.824	0.251	0.269	2.918	3.300
2081	10.825	0.312	0.342	1.738	4.639
2082	10.830	0.348	0.357	1.766	3.791
2083	10.837	0.302	0.324	2.291	4.832
2084	10.840	0.385	0.397	1.781	3.476
2085	10.841	0.422	0.486	1.488	2.256
2086	10.847	0.333	0.349	2.272	4.550
2087	10.848	0.554	0.581	1.449	5.567
2088	10.859	0.347	0.400	1.830	5.390
2089	10.862	0.402	0.416	1.783	2.868
2090	10.862	0.379	0.410	1.490	3.164
2091	10.869	0.621	0.753	1.379	4.360
2092	10.870	0.277	0.288	2.522	5.300
2093	10.877	0.286	0.296	2.641	4.293
2094	10.884	0.307	0.332	2.382	5.161
2095	10.886	0.302	0.310	2.191	4.074
2096	10.887	0.466	0.528	1.580	3.582
2097	10.898	0.367	0.428	1.742	4.175
2098	10.899	0.289	0.313	2.476	1.905

2099	10.910	0.360	0.407	1.637	5.613
2100	10.916	0.427	0.442	1.582	2.375
2101	10.918	0.411	0.487	1.791	8.417
2102	10.919	0.299	0.313	2.143	4.686
2103	10.919	0.297	0.314	2.073	4.593
2104	10.925	0.278	0.298	2.663	4.223
2105	10.929	0.386	0.419	1.733	3.560
2106	10.929	0.295	0.315	2.076	4.540
2107	10.942	0.283	0.299	2.609	4.486
2108	10.942	0.409	0.434	1.612	2.872
2109	10.945	0.279	0.298	2.651	4.809
2110	10.950	0.346	0.368	2.347	4.168
2111	10.957	0.399	0.444	1.415	4.234
2112	10.957	0.253	0.267	2.896	3.575
2113	10.960	0.430	0.456	1.543	3.522
2114	10.963	0.358	0.366	1.771	2.400
2115	10.971	0.295	0.314	2.273	5.567
2116	10.989	0.361	0.378	1.934	2.080
2117	10.990	0.347	0.373	2.286	3.751
2118	11.004	0.383	0.448	1.487	3.949
2119	11.009	0.407	0.464	1.694	4.957
2120	11.010	0.369	0.393	1.795	5.257
2121	11.012	0.634	0.752	1.288	4.239
2122	11.013	0.334	0.366	2.025	2.261
2123	11.014	0.308	0.316	2.528	4.440
2124	11.015	0.389	0.414	1.790	2.781
2125	11.022	0.250	0.267	2.951	5.311
2126	11.035	0.599	0.776	1.342	3.781
2127	11.037	0.351	0.365	1.899	4.433
2128	11.039	0.483	0.536	1.620	5.368
2129	11.050	0.291	0.314	2.352	5.356
2130	11.050	0.342	0.363	1.881	4.575
2131	11.052	0.263	0.269	2.605	4.540
2132	11.052	0.362	0.386	1.835	5.337
2133	11.054	0.297	0.314	2.708	4.460
2134	11.065	0.301	0.322	2.318	3.793
2135	11.073	0.422	0.471	1.568	5.615

2136	11.073	0.365	0.383	2.182	4.051
2137	11.077	0.440	0.489	1.332	2.879
2138	11.080	0.318	0.335	2.063	4.002
2139	11.084	0.297	0.308	2.724	4.702
2140	11.090	0.422	0.448	1.883	5.765
2141	11.094	0.434	0.477	1.827	6.978
2142	11.102	0.324	0.357	1.802	1.565
2143	11.104	0.343	0.366	2.369	4.517
2144	11.112	0.383	0.408	1.891	3.666
2145	11.113	0.401	0.404	1.725	2.481
2146	11.113	0.309	0.317	2.517	4.104
2147	11.118	0.299	0.315	2.686	3.096
2148	11.119	0.408	0.449	1.431	4.338
2149	11.119	0.375	0.437	1.720	4.058
2150	11.120	0.500	0.633	1.314	4.427
2151	11.124	0.385	0.400	1.940	2.638
2152	11.125	0.441	0.444	1.495	2.248
2153	11.126	0.362	0.394	2.006	4.389
2154	11.127	0.342	0.347	2.069	2.823
2155	11.134	0.514	0.614	1.354	4.412
2156	11.134	0.381	0.411	1.904	4.321
2157	11.134	0.360	0.417	1.765	4.317
2158	11.140	0.442	0.496	1.737	2.532
2159	11.149	0.385	0.460	1.451	6.178
2160	11.150	0.352	0.389	2.303	2.584
2161	11.162	0.366	0.383	2.172	3.180
2162	11.162	0.555	0.665	1.396	4.435
2163	11.164	0.386	0.436	1.559	4.649
2164	11.170	0.386	0.402	1.920	2.792
2165	11.172	0.352	0.367	1.891	4.593
2166	11.181	0.309	0.329	2.535	2.836
2167	11.181	0.425	0.438	2.012	2.828
2168	11.182	0.403	0.415	1.668	7.646
2169	11.183	0.364	0.414	1.763	5.734
2170	11.186	0.355	0.402	1.607	3.756
2171	11.186	0.554	0.607	1.239	2.102
2172	11.190	0.342	0.371	1.800	3.572

2173	11.194	0.422	0.456	2.007	4.748
2174	11.198	0.309	0.323	2.540	5.751
2175	11.201	0.455	0.475	1.837	3.752
2176	11.205	0.426	0.456	1.585	5.385
2177	11.209	0.306	0.330	2.136	2.314
2178	11.210	0.618	0.695	1.352	3.319
2179	11.212	0.465	0.549	1.532	3.905
2180	11.218	0.418	0.439	2.054	3.111
2181	11.218	0.259	0.275	2.583	4.003
2182	11.222	0.384	0.408	1.884	4.378
2183	11.225	0.323	0.337	1.957	4.623
2184	11.232	0.292	0.310	2.128	2.604
2185	11.234	0.312	0.321	2.253	3.603
2186	11.250	0.369	0.425	1.811	5.278
2187	11.259	0.394	0.414	1.573	3.524
2188	11.260	0.451	0.562	1.392	5.271
2189	11.260	0.461	0.517	1.435	4.475
2190	11.270	0.360	0.389	2.015	3.062
2191	11.273	0.298	0.309	2.816	4.694
2192	11.278	0.279	0.294	2.459	5.100
2193	11.288	0.312	0.316	2.203	3.236
2194	11.291	0.618	0.734	1.239	4.489
2195	11.293	0.372	0.397	1.942	2.334
2196	11.293	0.437	0.476	1.503	2.485
2197	11.302	0.352	0.388	2.292	4.413
2198	11.304	0.395	0.398	1.595	2.031
2199	11.305	0.370	0.377	2.002	1.101
2200	11.305	0.414	0.468	1.602	4.475
2201	11.310	0.295	0.319	2.410	5.785
2202	11.311	0.411	0.439	1.893	5.231
2203	11.316	0.355	0.382	1.909	3.466
2204	11.316	0.365	0.379	1.886	5.455
2205	11.317	0.408	0.451	1.871	3.234
2206	11.319	0.420	0.434	2.048	3.348
2207	11.322	0.397	0.400	1.589	3.612
2208	11.329	0.324	0.343	1.825	6.315
2209	11.338	0.297	0.308	2.843	4.612

2210	11.349	0.389	0.403	2.010	5.459
2211	11.349	0.644	0.765	1.209	6.433
2212	11.353	0.251	0.270	2.920	2.776
2213	11.355	0.472	0.557	1.531	5.604
2214	11.362	0.282	0.292	2.433	2.907
2215	11.363	0.384	0.405	1.569	3.130
2216	11.368	0.390	0.406	1.856	3.640
2217	11.373	0.289	0.316	2.083	3.491
2218	11.377	0.339	0.382	1.810	1.416
2219	11.379	0.389	0.423	1.906	3.313
2220	11.387	0.341	0.347	2.068	2.631
2221	11.389	0.411	0.445	1.444	1.791
2222	11.402	0.285	0.312	2.669	5.057
2223	11.404	0.472	0.553	1.541	2.842
2224	11.408	0.310	0.323	2.216	4.179
2225	11.413	0.464	0.546	1.492	3.749
2226	11.424	0.391	0.448	1.832	5.004
2227	11.431	0.399	0.410	1.851	6.099
2228	11.437	0.309	0.318	2.158	2.317
2229	11.437	0.309	0.322	2.231	2.698
2230	11.440	0.348	0.385	2.301	2.679
2231	11.441	0.446	0.517	1.646	4.660
2232	11.443	0.299	0.323	2.339	4.308
2233	11.450	0.363	0.373	2.068	3.963
2234	11.457	0.441	0.495	1.743	1.402
2235	11.458	0.563	0.620	1.241	3.168
2236	11.458	0.494	0.514	1.551	4.788
2237	11.461	0.399	0.419	1.833	4.394
2238	11.471	0.426	0.475	1.852	3.352
2239	11.477	0.276	0.300	2.381	5.471
2240	11.480	0.593	0.624	1.474	4.679
2241	11.484	0.285	0.312	2.671	4.797
2242	11.494	0.368	0.382	1.853	4.592
2243	11.495	0.340	0.345	1.943	6.666
2244	11.501	0.501	0.555	1.578	6.095
2245	11.501	0.464	0.481	1.771	5.929
2246	11.503	0.435	0.455	1.586	4.915

2247	11.509	0.372	0.430	1.522	2.292
2248	11.527	0.289	0.315	2.087	2.440
2249	11.528	0.305	0.345	1.813	3.636
2250	11.531	0.327	0.345	1.892	4.404
2251	11.533	0.373	0.380	1.981	3.274
2252	11.537	0.347	0.391	1.637	1.622
2253	11.543	0.364	0.377	1.730	3.541
2254	11.548	0.356	0.367	1.722	1.859
2255	11.558	0.297	0.318	2.244	4.440
2256	11.565	0.415	0.430	1.679	3.576
2257	11.568	0.409	0.444	1.451	3.328
2258	11.571	0.321	0.336	2.010	1.671
2259	11.583	0.470	0.525	1.583	3.047
2260	11.595	0.407	0.421	1.540	3.163
2261	11.600	0.484	0.527	1.471	2.949
2262	11.602	0.478	0.569	1.587	6.970
2263	11.603	0.290	0.301	2.644	3.918
2264	11.604	0.372	0.416	1.611	5.080
2265	11.607	0.419	0.491	1.341	5.233
2266	11.609	0.378	0.414	1.485	0.536
2267	11.611	0.277	0.286	2.682	3.969
2268	11.613	0.469	0.524	1.590	0.983
2269	11.614	0.369	0.382	1.759	6.481
2270	11.617	0.365	0.419	1.689	3.275
2271	11.644	0.401	0.421	1.825	3.018
2272	11.658	0.477	0.517	1.634	4.092
2273	11.677	0.380	0.389	1.734	1.746
2274	11.677	0.420	0.435	1.712	3.078
2275	11.680	0.379	0.410	1.934	4.037
2276	11.680	0.357	0.406	1.701	3.212
2277	11.682	0.381	0.400	1.885	5.922
2278	11.698	0.307	0.316	2.175	3.889
2279	11.707	0.513	0.582	1.477	3.315
2280	11.710	0.421	0.435	1.717	3.348
2281	11.718	0.353	0.379	2.271	3.585
2282	11.719	0.412	0.428	1.866	4.076
2283	11.720	0.340	0.360	1.901	5.803

2284	11.725	0.349	0.362	1.901	3.079
2285	11.725	0.423	0.453	1.423	2.979
2286	11.736	0.618	0.687	1.349	5.437
2287	11.738	0.358	0.416	1.749	4.550
2288	11.742	0.399	0.418	1.835	3.596
2289	11.753	0.394	0.417	1.892	3.992
2290	11.755	0.351	0.374	1.733	4.699
2291	11.757	0.386	0.410	1.875	2.304
2292	11.760	0.316	0.332	2.410	1.851
2293	11.762	0.413	0.482	1.734	1.336
2294	11.765	0.504	0.533	1.411	5.208
2295	11.769	0.377	0.415	1.593	4.715
2296	11.774	0.289	0.300	2.640	1.833
2297	11.778	0.390	0.398	2.079	2.826
2298	11.781	0.327	0.338	2.064	4.807
2299	11.783	0.374	0.385	1.733	4.859
2300	11.791	0.376	0.416	1.596	4.883
2301	11.793	0.402	0.436	1.674	4.202
2302	11.805	0.458	0.543	1.578	4.451
2303	11.808	0.457	0.500	1.623	5.511
2304	11.810	0.324	0.351	2.187	4.184
2305	11.812	0.361	0.411	1.531	3.865
2306	11.812	0.290	0.302	2.764	4.336
2307	11.813	0.393	0.414	1.902	5.302
2308	11.818	0.306	0.318	2.290	5.020
2309	11.819	0.474	0.507	1.814	6.467
2310	11.821	0.323	0.350	2.200	5.272
2311	11.829	0.325	0.331	1.792	1.795
2312	11.834	0.462	0.526	1.603	4.714
2313	11.836	0.346	0.374	2.344	2.924
2314	11.842	0.381	0.401	1.881	5.958
2315	11.846	0.427	0.450	1.416	3.853
2316	11.853	0.365	0.378	1.792	6.686
2317	11.853	0.361	0.384	1.852	6.617
2318	11.857	0.387	0.418	1.863	1.678
2319	11.859	0.309	0.319	2.272	5.048
2320	11.861	0.332	0.344	2.086	4.284

2321	11.862	0.319	0.340	2.157	3.924
2322	11.864	0.426	0.487	1.684	6.678
2323	11.866	0.376	0.384	1.758	3.258
2324	11.874	0.314	0.357	2.069	3.139
2325	11.876	0.311	0.354	2.098	2.057
2326	11.893	0.526	0.652	1.372	6.051
2327	11.905	0.352	0.379	2.277	4.378
2328	11.916	0.446	0.501	1.427	4.774
2329	11.928	0.312	0.325	1.956	5.981
2330	11.928	0.479	0.502	1.673	2.269
2331	11.929	0.369	0.393	1.810	6.590
2332	11.943	0.401	0.420	1.803	2.089
2333	11.950	0.334	0.360	2.056	4.783
2334	11.953	0.330	0.342	2.098	4.597
2335	11.958	0.401	0.418	1.830	3.006
2336	11.961	0.439	0.491	1.439	1.641
2337	11.963	0.353	0.358	1.749	1.702
2338	11.963	0.315	0.331	2.420	1.674
2339	11.967	0.399	0.414	1.807	1.994
2340	11.972	0.361	0.404	1.672	3.128
2341	11.975	0.512	0.599	1.408	2.325
2342	11.979	0.483	0.491	1.601	5.444
2343	11.979	0.296	0.307	2.782	3.331
2344	11.980	0.416	0.456	1.568	4.944
2345	11.984	0.401	0.425	1.640	4.149
2346	11.989	0.322	0.329	1.813	3.334
2347	12.001	0.402	0.425	1.751	2.347
2348	12.001	0.397	0.461	1.803	3.651
2349	12.004	0.364	0.414	1.508	3.989
2350	12.005	0.305	0.327	2.568	3.157
2351	12.027	0.295	0.306	2.710	3.351
2352	12.032	0.299	0.331	1.986	5.575
2353	12.033	0.334	0.359	2.009	4.101
2354	12.035	0.366	0.385	1.869	4.063
2355	12.039	0.363	0.386	1.799	2.809
2356	12.039	0.306	0.328	2.556	3.045
2357	12.041	0.380	0.409	1.927	5.113

2358	12.048	0.292	0.316	2.056	3.985
2359	12.049	0.431	0.457	1.461	2.740
2360	12.051	0.515	0.560	1.434	4.930
2361	12.052	0.294	0.306	2.744	3.136
2362	12.052	0.408	0.473	1.693	5.164
2363	12.059	0.652	0.755	1.225	4.642
2364	12.065	0.349	0.363	1.898	3.360
2365	12.087	0.433	0.485	1.499	6.083
2366	12.092	0.478	0.489	1.678	5.546
2367	12.093	0.440	0.467	1.950	2.510
2368	12.093	0.294	0.309	2.119	5.153
2369	12.096	0.507	0.558	1.495	6.046
2370	12.097	0.356	0.369	1.779	2.540
2371	12.098	0.383	0.452	1.763	4.083
2372	12.100	0.391	0.433	1.922	5.107
2373	12.104	0.393	0.401	1.836	2.190
2374	12.105	0.299	0.327	2.238	4.357
2375	12.113	0.460	0.548	1.473	0.627
2376	12.114	0.486	0.493	1.574	5.729
2377	12.124	0.344	0.383	1.797	6.057
2378	12.137	0.571	0.631	1.457	3.608
2379	12.138	0.327	0.344	1.770	4.224
2380	12.139	0.297	0.306	2.792	2.975
2381	12.150	0.291	0.315	2.337	2.095
2382	12.156	0.555	0.584	1.608	1.984
2383	12.162	0.297	0.316	2.570	3.743
2384	12.164	0.438	0.476	1.490	4.567
2385	12.169	0.427	0.454	1.473	4.395
2386	12.171	0.438	0.491	1.447	3.225
2387	12.173	0.370	0.410	1.796	6.161
2388	12.180	0.388	0.402	1.914	2.519
2389	12.181	0.313	0.328	1.941	3.709
2390	12.199	0.308	0.318	2.298	1.551
2391	12.204	0.386	0.442	1.716	3.837
2392	12.205	0.292	0.309	2.293	2.888
2393	12.207	0.294	0.313	2.446	6.162
2394	12.212	0.482	0.554	1.379	4.134

2395	12.222	0.367	0.391	1.766	2.658
2396	12.228	0.339	0.349	2.040	4.548
2397	12.229	0.314	0.320	1.936	2.938
2398	12.237	0.317	0.330	1.945	4.048
2399	12.252	0.338	0.347	2.048	4.678
2400	12.256	0.515	0.611	1.383	3.826
2401	12.260	0.420	0.464	1.819	4.968
2402	12.266	0.307	0.314	2.329	2.981
2403	12.269	0.293	0.310	2.724	5.129
2404	12.270	0.366	0.377	1.705	3.140
2405	12.272	0.305	0.312	2.389	1.969
2406	12.275	0.260	0.273	2.583	2.454
2407	12.298	0.342	0.346	1.842	0.983
2408	12.301	0.303	0.338	1.805	3.713
2409	12.307	0.376	0.448	1.475	5.816
2410	12.309	0.368	0.420	1.554	5.439
2411	12.325	0.436	0.483	1.511	6.250
2412	12.325	0.547	0.570	1.468	5.053
2413	12.329	0.528	0.601	1.452	6.235
2414	12.330	0.340	0.361	1.851	1.663
2415	12.347	0.377	0.419	1.755	6.194
2416	12.351	0.257	0.269	2.672	4.166
2417	12.365	0.312	0.327	1.944	3.015
2418	12.369	0.430	0.487	1.484	3.479
2419	12.372	0.369	0.385	1.718	4.308
2420	12.387	0.363	0.377	1.724	3.778
2421	12.390	0.397	0.412	1.819	3.238
2422	12.400	0.297	0.313	2.292	7.015
2423	12.404	0.317	0.332	2.025	4.273
2424	12.405	0.376	0.392	1.667	4.843
2425	12.417	0.291	0.309	2.310	3.104
2426	12.426	0.672	0.770	1.234	5.341
2427	12.429	0.378	0.394	1.885	5.635
2428	12.431	0.447	0.494	1.683	3.242
2429	12.435	0.359	0.371	1.760	4.194
2430	12.435	0.355	0.356	1.734	0.703
2431	12.442	0.345	0.380	2.051	4.813

2432	12.444	0.337	0.357	1.890	1.503
2433	12.448	0.526	0.549	1.461	5.484
2434	12.462	0.425	0.478	1.699	6.083
2435	12.478	0.399	0.415	1.805	3.119
2436	12.483	0.413	0.435	1.883	4.075
2437	12.486	0.442	0.482	1.786	6.484
2438	12.499	0.429	0.479	1.828	3.766
2439	12.517	0.359	0.371	1.734	3.734
2440	12.539	0.424	0.456	1.426	4.492
2441	12.548	0.336	0.351	1.953	4.901
2442	12.557	0.258	0.270	2.672	5.249
2443	12.571	0.414	0.433	1.842	1.826
2444	12.574	0.304	0.312	2.402	3.934
2445	12.574	0.348	0.377	1.898	2.855
2446	12.593	0.546	0.570	1.475	6.531
2447	12.600	0.315	0.331	1.843	3.652
2448	12.614	0.416	0.429	1.704	2.486
2449	12.615	0.355	0.393	1.979	2.795
2450	12.618	0.294	0.314	2.470	7.411
2451	12.618	0.357	0.370	1.780	3.960
2452	12.622	0.464	0.546	1.526	5.215
2453	12.629	0.395	0.402	1.879	3.028
2454	12.634	0.370	0.393	1.906	4.106
2455	12.634	0.503	0.571	1.498	5.015
2456	12.678	0.390	0.408	1.869	6.265
2457	12.696	0.354	0.383	1.877	3.603
2458	12.715	0.373	0.414	1.779	7.320
2459	12.721	0.308	0.320	2.604	2.810
2460	12.724	0.327	0.335	1.736	3.025
2461	12.726	0.425	0.453	1.422	5.256
2462	12.727	0.354	0.356	1.731	3.440
2463	12.730	0.467	0.530	1.458	4.800
2464	12.732	0.526	0.599	1.456	7.191
2465	12.735	0.366	0.409	1.982	4.407
2466	12.739	0.417	0.467	1.539	6.508
2467	12.739	0.670	0.767	1.234	7.022
2468	12.745	0.516	0.582	1.450	7.420

2469	12.749	0.376	0.397	1.879	6.699
2470	12.756	0.503	0.517	1.496	6.748
2471	12.762	0.376	0.417	1.996	5.982
2472	12.763	0.359	0.361	1.924	3.940
2473	12.765	0.524	0.538	1.543	5.407
2474	12.772	0.466	0.520	1.644	3.401
2475	12.781	0.366	0.378	1.679	4.267
2476	12.791	0.363	0.367	1.886	2.942
2477	12.814	0.415	0.484	1.730	5.506
2478	12.815	0.376	0.410	1.557	5.097
2479	12.834	0.297	0.318	2.369	3.021
2480	12.843	0.472	0.527	1.537	5.789
2481	12.857	0.360	0.383	1.823	4.243
2482	12.858	0.401	0.422	1.817	3.711
2483	12.860	0.317	0.324	1.951	2.456
2484	12.865	0.413	0.482	1.744	2.697
2485	12.875	0.382	0.405	1.905	2.408
2486	12.875	0.312	0.333	2.085	5.293
2487	12.877	0.400	0.419	1.827	2.496
2488	12.880	0.383	0.405	1.903	3.016
2489	12.887	0.307	0.319	2.624	3.926
2490	12.899	0.375	0.434	1.782	4.820
2491	12.906	0.288	0.314	2.099	3.782
2492	12.909	0.344	0.382	1.807	6.983
2493	12.909	0.373	0.408	1.580	4.756
2494	12.921	0.373	0.393	1.906	6.327
2495	12.931	0.425	0.437	1.654	1.391
2496	12.942	0.396	0.418	1.754	2.873
2497	12.944	0.415	0.438	1.744	4.761
2498	12.951	0.298	0.310	2.702	4.650
2499	12.955	0.324	0.347	2.154	2.961
2500	12.958	0.288	0.308	2.482	5.226
2501	12.978	0.353	0.372	1.896	1.600
2502	12.979	0.422	0.503	1.725	7.211
2503	12.980	0.377	0.416	1.958	6.697
2504	12.987	0.515	0.557	1.429	6.390
2505	13.009	0.371	0.385	1.992	2.582

2506	13.009	0.292	0.309	2.703	3.594
2507	13.017	0.417	0.458	1.836	5.834
2508	13.018	0.310	0.332	2.081	4.153
2509	13.021	0.445	0.515	1.504	4.736
2510	13.028	0.362	0.380	1.891	5.294
2511	13.029	0.407	0.452	1.868	5.581
2512	13.030	0.497	0.514	1.568	7.629
2513	13.042	0.287	0.307	2.519	5.343
2514	13.046	0.468	0.523	1.543	5.943
2515	13.058	0.289	0.313	2.106	4.439
2516	13.072	0.369	0.404	1.622	5.144
2517	13.074	0.314	0.323	1.951	2.175
2518	13.085	0.430	0.446	1.614	3.462
2519	13.090	0.399	0.421	1.754	2.232
2520	13.096	0.418	0.445	1.424	4.644
2521	13.113	0.365	0.389	1.792	3.993
2522	13.116	0.345	0.379	2.063	5.435
2523	13.126	0.292	0.319	2.493	2.739
2524	13.136	0.566	0.624	1.470	5.660
2525	13.140	0.490	0.511	1.537	6.712
2526	13.149	0.482	0.523	1.636	5.181
2527	13.162	0.410	0.430	1.797	2.086
2528	13.165	0.339	0.358	1.869	3.281
2529	13.172	0.499	0.565	1.505	6.020
2530	13.183	0.398	0.415	1.828	1.557
2531	13.190	0.396	0.412	1.847	1.945
2532	13.192	0.289	0.313	2.090	3.872
2533	13.202	0.442	0.478	1.348	5.562
2534	13.216	0.393	0.415	1.796	1.700
2535	13.232	0.380	0.441	1.737	4.118
2536	13.270	0.336	0.354	1.911	3.019
2537	13.271	0.409	0.415	1.640	6.459
2538	13.275	0.343	0.379	1.788	5.965
2539	13.284	0.365	0.376	1.749	4.871
2540	13.287	0.345	0.382	1.762	5.934
2541	13.288	0.494	0.575	1.354	6.669
2542	13.291	0.348	0.390	1.646	3.364

2543	13.293	0.528	0.563	1.525	4.781
2544	13.295	0.365	0.416	1.557	3.910
2545	13.354	0.421	0.440	1.419	5.176
2546	13.359	0.310	0.317	2.502	4.059
2547	13.365	0.473	0.530	1.541	6.166
2548	13.395	0.352	0.355	1.747	2.865
2549	13.402	0.399	0.460	1.812	5.388
2550	13.410	0.413	0.465	1.510	6.012
2551	13.416	0.364	0.376	1.771	4.595
2552	13.418	0.592	0.697	1.255	7.246
2553	13.418	0.369	0.409	1.803	5.884
2554	13.423	0.424	0.443	1.708	5.499
2555	13.446	0.403	0.434	1.659	4.445
2556	13.469	0.376	0.387	1.760	2.852
2557	13.475	0.364	0.382	1.737	4.451
2558	13.489	0.503	0.565	1.471	8.602
2559	13.493	0.355	0.361	1.931	3.046
2560	13.502	0.289	0.312	2.609	4.450
2561	13.510	0.403	0.438	1.676	5.582
2562	13.511	0.399	0.428	1.657	2.074
2563	13.512	0.380	0.417	1.936	3.688
2564	13.517	0.365	0.387	1.839	3.279
2565	13.520	0.363	0.418	1.529	1.699
2566	13.538	0.441	0.486	1.694	6.279
2567	13.551	0.438	0.469	1.359	5.972
2568	13.565	0.434	0.517	1.579	4.789
2569	13.573	0.308	0.316	2.517	3.183
2570	13.583	0.373	0.405	1.968	3.755
2571	13.588	0.481	0.543	1.542	4.109
2572	13.602	0.426	0.489	1.764	6.404
2573	13.604	0.504	0.631	1.380	5.992
2574	13.606	0.402	0.433	1.650	2.197
2575	13.629	0.415	0.421	1.644	4.082
2576	13.669	0.486	0.506	1.356	5.022
2577	13.685	0.287	0.312	2.124	3.303
2578	13.699	0.314	0.336	2.451	2.297
2579	13.699	0.361	0.401	2.133	3.675

2580	13.700	0.588	0.625	1.486	6.156
2581	13.711	0.346	0.380	2.010	3.680
2582	13.715	0.362	0.402	2.125	5.368
2583	13.717	0.310	0.330	2.101	3.862
2584	13.723	0.615	0.690	1.349	6.621
2585	13.752	0.367	0.384	1.702	4.885
2586	13.762	0.307	0.324	2.080	1.339
2587	13.776	0.357	0.386	1.722	2.883
2588	13.778	0.446	0.500	1.607	5.906
2589	13.784	0.347	0.386	1.817	5.297
2590	13.786	0.408	0.455	1.575	4.343
2591	13.806	0.298	0.305	2.483	4.120
2592	13.811	0.420	0.440	1.909	2.804
2593	13.813	0.377	0.410	1.581	5.486
2594	13.828	0.316	0.336	2.165	3.408
2595	13.837	0.315	0.337	2.437	3.997
2596	13.839	0.419	0.462	1.866	5.893
2597	13.849	0.358	0.391	2.294	4.879
2598	13.849	0.575	0.608	1.501	6.030
2599	13.883	0.364	0.419	1.522	4.303
2600	13.897	0.375	0.409	1.599	5.192
2601	13.905	0.354	0.383	1.735	4.926
2602	13.911	0.400	0.441	1.760	4.517
2603	13.927	0.292	0.307	2.334	4.407
2604	13.940	0.500	0.569	1.454	4.225
2605	13.948	0.293	0.308	2.298	4.457
2606	13.948	0.412	0.433	1.745	5.424
2607	13.956	0.307	0.324	2.078	3.489
2608	13.964	0.338	0.371	2.078	3.385
2609	13.978	0.417	0.444	1.434	5.661
2610	13.982	0.403	0.449	1.634	1.929
2611	13.985	0.374	0.418	1.613	2.101
2612	13.986	0.426	0.451	2.101	5.082
2613	14.010	0.337	0.353	2.326	5.611
2614	14.030	0.350	0.367	1.920	3.336
2615	14.057	0.293	0.311	2.423	6.275
2616	14.076	0.291	0.310	2.461	6.288

2617	14.076	0.399	0.431	1.651	2.538
2618	14.084	0.463	0.568	1.544	7.373
2619	14.087	0.323	0.327	1.768	2.795
2620	14.117	0.384	0.390	1.642	5.475
2621	14.121	0.351	0.358	1.814	1.174
2622	14.132	0.344	0.380	1.781	6.792
2623	14.136	0.409	0.434	1.605	5.709
2624	14.138	0.342	0.377	1.807	6.704
2625	14.149	0.403	0.449	1.623	3.828
2626	14.164	0.414	0.444	1.423	5.072
2627	14.176	0.384	0.390	1.641	5.817
2628	14.177	0.306	0.341	1.790	3.503
2629	14.184	0.399	0.418	1.814	2.842
2630	14.193	0.307	0.313	2.358	4.564
2631	14.199	0.360	0.388	2.128	6.707
2632	14.199	0.321	0.347	2.222	5.354
2633	14.208	0.427	0.449	1.691	4.571
2634	14.236	0.318	0.340	2.152	4.003
2635	14.244	0.397	0.441	1.595	4.518
2636	14.256	0.438	0.478	1.469	5.994
2637	14.257	0.257	0.270	2.633	4.008
2638	14.270	0.303	0.326	2.125	4.032
2639	14.272	0.318	0.340	2.182	3.723
2640	14.289	0.419	0.440	1.431	6.132
2641	14.291	0.417	0.450	1.812	3.639
2642	14.301	0.306	0.324	2.224	1.450
2643	14.313	0.438	0.470	1.724	5.766
2644	14.314	0.407	0.457	1.856	4.605
2645	14.322	0.412	0.434	1.743	6.491
2646	14.332	0.489	0.499	1.577	4.929
2647	14.337	0.321	0.329	1.844	4.918
2648	14.358	0.422	0.444	1.692	3.190
2649	14.359	0.396	0.428	1.677	3.445
2650	14.364	0.400	0.460	1.818	6.673
2651	14.405	0.405	0.447	1.850	3.484
2652	14.418	0.401	0.441	1.737	3.912
2653	14.429	0.453	0.525	1.726	1.955

2654	14.431	0.423	0.446	1.686	4.465
2655	14.432	0.417	0.470	1.841	5.836
2656	14.438	0.290	0.301	2.747	1.608
2657	14.440	0.424	0.479	1.494	3.797
2658	14.441	0.404	0.437	1.440	5.893
2659	14.443	0.361	0.397	1.655	4.414
2660	14.446	0.315	0.317	2.270	0.926
2661	14.471	0.428	0.452	1.686	5.189
2662	14.477	0.305	0.318	2.506	1.240
2663	14.483	0.409	0.442	1.773	3.679
2664	14.510	0.306	0.319	2.484	1.949
2665	14.532	0.486	0.505	1.363	5.714
2666	14.534	0.346	0.379	2.023	3.218
2667	14.542	0.546	0.624	1.330	5.820
2668	14.547	0.352	0.365	1.845	4.057
2669	14.553	0.329	0.336	1.773	6.212
2670	14.578	0.353	0.387	1.900	3.518
2671	14.578	0.406	0.458	1.870	3.454
2672	14.600	0.360	0.395	1.650	5.551
2673	14.605	0.367	0.410	1.639	4.318
2674	14.610	0.420	0.434	1.702	4.823
2675	14.612	0.417	0.468	1.528	6.854
2676	14.612	0.451	0.514	1.585	6.279
2677	14.623	0.400	0.445	1.639	4.302
2678	14.648	0.304	0.322	2.243	3.243
2679	14.655	0.356	0.383	1.738	4.314
2680	14.656	0.263	0.270	2.586	1.287
2681	14.661	0.736	0.868	1.114	4.334
2682	14.662	0.324	0.339	1.932	3.843
2683	14.664	0.433	0.490	1.476	5.164
2684	14.690	0.375	0.430	1.738	5.373
2685	14.693	0.466	0.482	1.368	5.846
2686	14.733	0.310	0.321	2.211	3.871
2687	14.743	0.426	0.491	1.514	2.697
2688	14.743	0.352	0.368	1.834	4.060
2689	14.748	0.259	0.273	2.618	3.261
2690	14.769	0.413	0.436	1.747	3.780

2691	14.773	0.353	0.381	1.749	6.008
2692	14.773	0.313	0.322	1.964	5.527
2693	14.774	0.341	0.373	2.072	3.021
2694	14.785	0.394	0.417	1.573	5.086
2695	14.818	0.369	0.399	1.931	6.017
2696	14.826	0.373	0.417	1.625	3.718
2697	14.826	0.396	0.421	1.694	2.326
2698	14.849	0.355	0.378	1.949	5.459
2699	14.861	0.339	0.372	1.895	5.816
2700	14.868	0.292	0.309	2.674	6.362
2701	14.881	0.421	0.476	1.501	5.810
2702	14.883	0.374	0.382	1.986	1.469
2703	14.884	0.392	0.396	1.630	6.039
2704	14.929	0.328	0.347	2.167	5.602
2705	14.982	0.420	0.433	1.705	6.294
2706	15.043	0.365	0.420	1.523	2.526
2707	15.055	0.467	0.494	1.704	7.159
2708	15.091	0.356	0.384	1.823	5.153
2709	15.095	0.293	0.310	2.112	2.223
2710	15.097	0.395	0.399	1.607	6.399
2711	15.117	0.414	0.437	1.742	5.555
2712	15.137	0.464	0.470	1.421	4.726
2713	15.164	0.353	0.378	1.930	3.124
2714	15.173	0.337	0.366	1.831	4.917
2715	15.177	0.342	0.360	2.007	6.210
2716	15.215	0.358	0.392	1.929	5.032
2717	15.240	0.411	0.444	1.442	5.909
2718	15.253	0.308	0.325	2.212	3.245
2719	15.275	0.342	0.360	1.970	4.119
2720	15.280	0.277	0.294	2.486	3.401
2721	15.297	0.360	0.394	1.915	4.152
2722	15.333	0.309	0.323	2.091	4.667
2723	15.415	0.316	0.334	2.025	2.197
2724	15.417	0.307	0.335	2.090	3.573
2725	15.442	0.308	0.323	2.092	3.165
2726	15.450	0.322	0.338	1.940	2.038
2727	15.462	0.625	0.720	1.263	5.809

2728	15.468	0.366	0.409	1.651	5.445
2729	15.469	0.307	0.324	2.226	4.244
2730	15.471	0.404	0.438	1.464	6.247
2731	15.506	0.366	0.422	1.514	4.716
2732	15.518	0.533	0.589	1.391	3.822
2733	15.525	0.288	0.311	2.671	2.974
2734	15.542	0.315	0.330	1.932	5.036
2735	15.617	0.319	0.321	1.972	6.303
2736	15.618	0.410	0.462	1.855	3.737
2737	15.656	0.418	0.430	1.606	7.934
2738	15.661	0.462	0.482	1.376	6.557
2739	15.664	0.319	0.334	1.986	2.069
2740	15.675	0.454	0.548	1.448	5.129
2741	15.742	0.381	0.418	1.792	4.972
2742	15.744	0.388	0.414	1.777	5.335
2743	15.762	0.346	0.360	1.993	4.026
2744	15.828	0.423	0.477	1.511	3.673
2745	15.850	0.388	0.409	1.599	5.280
2746	15.852	0.278	0.298	2.321	6.371
2747	15.859	0.361	0.394	1.922	5.970
2748	15.874	0.393	0.414	1.576	4.922
2749	15.883	0.473	0.532	1.665	3.761
2750	15.900	0.346	0.361	1.985	2.134
2751	15.953	0.427	0.439	1.715	2.542
2752	15.954	0.344	0.371	2.021	4.860
2753	15.961	0.288	0.315	2.116	6.095
2754	15.979	0.297	0.320	2.329	6.738
2755	15.996	0.358	0.390	1.958	6.406
2756	16.007	0.351	0.358	1.713	5.594
2757	16.015	0.390	0.459	1.775	6.620
2758	16.042	0.423	0.460	1.759	2.951
2759	16.043	0.366	0.376	1.688	4.047
2760	16.051	0.387	0.444	1.684	3.049
2761	16.068	0.419	0.497	1.694	6.868
2762	16.136	0.373	0.389	1.994	6.387
2763	16.156	0.263	0.270	2.563	3.112
2764	16.165	0.358	0.391	1.942	6.065

2765	16.170	0.361	0.394	1.924	4.322
2766	16.187	0.383	0.444	1.738	4.777
2767	16.207	0.315	0.332	2.051	2.250
2768	16.251	0.468	0.490	1.732	6.364
2769	16.343	0.375	0.410	1.815	2.365
2770	16.344	0.394	0.433	1.738	1.998
2771	16.372	0.415	0.430	1.663	2.515
2772	16.384	0.280	0.297	2.318	5.204
2773	16.408	0.299	0.322	2.311	7.402
2774	16.454	0.442	0.475	1.570	7.524
2775	16.470	0.307	0.326	2.216	4.032
2776	16.519	0.336	0.379	1.829	7.452
2777	16.520	0.329	0.339	1.789	2.090
2778	16.553	0.402	0.433	1.847	7.287
2779	16.555	0.311	0.321	2.241	4.501
2780	16.646	0.375	0.417	1.591	5.559
2781	16.653	0.430	0.493	1.560	9.218
2782	16.664	0.345	0.369	2.135	7.186
2783	16.693	0.366	0.421	1.724	7.067
2784	16.707	0.422	0.461	1.834	4.303
2785	16.712	0.344	0.356	2.015	4.200
2786	16.827	0.410	0.462	1.498	5.677
2787	16.851	0.333	0.369	1.834	6.356
2788	16.878	0.343	0.357	2.006	2.705
2789	16.911	0.341	0.367	2.028	4.710
2790	16.922	0.401	0.432	1.836	5.795
2791	16.926	0.342	0.367	2.138	6.641
2792	16.929	0.410	0.461	1.502	5.269
2793	17.077	0.504	0.565	1.465	8.113
2794	17.085	0.338	0.362	2.057	4.351
2795	17.092	0.339	0.356	1.788	4.510
2796	17.142	0.340	0.365	2.323	4.186
2797	17.226	0.336	0.346	2.059	5.965
2798	17.243	0.350	0.373	2.038	5.206
2799	17.428	0.338	0.356	1.795	6.651
2800	17.814	0.392	0.430	1.712	7.102
2801	17.964	0.373	0.396	1.954	3.443

2802	17.971	0.379	0.435	1.661	4.106
2803	18.086	0.286	0.296	2.321	4.090
2804	18.172	0.373	0.434	1.706	6.151
2805	18.182	0.415	0.451	1.832	3.942
2806	18.317	0.303	0.321	2.148	5.362
2807	18.700	0.353	0.379	1.954	6.322
2808	18.910	0.342	0.366	1.951	6.958
2809	19.065	0.363	0.392	1.679	5.116
2810	19.089	0.413	0.435	1.610	5.052
2811	19.216	0.324	0.333	1.883	5.083

14.4 Vibrational frequencies of Arg conformers

Harmonic vibrational zero point energy (ZPE) and frequencies for each conformer in cm^{-1}.

- Conformer (1); ZPE = 49413.058 cm^{-1}:

56.34	62.10	99.28	109.93	172.32	175.58
198.44	221.60	242.70	288.45	325.40	358.90
372.21	406.14	418.02	457.90	502.63	530.68
548.19	620.50	672.83	730.18	743.41	771.08
792.61	859.88	865.68	892.16	915.80	953.55
981.90	1024.25	1068.72	1102.96	1118.86	1150.10
1166.81	1175.15	1206.05	1238.95	1244.10	1290.34
1322.38	1343.04	1370.50	1379.18	1396.16	1411.23
1421.32	1460.06	1472.43	1474.90	1477.26	1602.96
1638.24	1671.38	1706.66	1877.37	3029.67	3036.60
3045.96	3050.09	3080.77	3090.34	3114.42	3478.33
3529.23	3572.38	3611.61	3618.48	3678.82	3731.69

- Conformer (2); ZPE = 49385.773 cm^{-1}:

75.36	105.86	145.33	166.26	183.68	195.03
201.33	223.71	260.05	291.07	318.25	341.58
356.13	415.39	491.30	502.76	535.26	543.05
603.14	607.14	625.01	721.77	728.60	767.50
796.84	820.27	858.61	874.32	904.07	943.81
977.85	1025.37	1068.63	1115.17	1123.20	1141.70
1163.26	1195.91	1211.27	1238.52	1244.60	1277.29
1312.15	1331.05	1363.10	1387.88	1390.67	1403.55
1458.41	1461.52	1469.11	1494.98	1513.67	1536.35
1629.47	1655.90	1703.00	1827.27	2983.16	3003.15
3035.65	3048.27	3056.15	3085.35	3107.71	3310.09
3555.65	3590.33	3630.59	3651.70	3653.97	3736.46

- Conformer (3); ZPE = 49544.868 cm^{-1}:

60.60	72.38	81.46	101.48	128.13	181.17
222.51	294.71	323.78	325.89	335.23	365.20
379.68	425.00	440.14	516.59	541.01	551.23
568.05	598.76	665.41	729.29	734.66	788.52
795.96	808.27	831.20	890.20	906.63	949.82
976.03	1043.69	1065.65	1106.90	1129.06	1139.64
1148.69	1181.30	1205.11	1236.70	1243.17	1260.18
1320.33	1336.07	1352.13	1378.31	1389.81	1402.36
1432.82	1442.88	1464.68	1474.24	1493.44	1571.73
1639.76	1689.51	1722.21	1878.75	3033.66	3038.46
3051.93	3063.32	3078.70	3099.90	3112.22	3429.30
3582.78	3609.11	3631.61	3641.16	3662.11	3717.35

- Conformer (4); ZPE = 49345.999 cm^{-1}:

80.58	94.18	141.53	159.83	177.95	195.27
209.57	240.32	254.15	282.94	287.19	339.95
361.59	428.47	475.98	494.95	503.68	528.56
577.67	595.39	617.24	638.16	749.78	776.38
784.75	795.97	840.83	890.77	901.47	955.36
976.57	1033.37	1056.79	1100.32	1119.90	1156.71
1164.59	1188.41	1219.04	1239.01	1250.28	1279.68
1296.70	1351.09	1364.11	1383.74	1391.76	1405.29
1431.09	1451.63	1477.02	1479.60	1515.49	1600.09
1626.15	1661.82	1693.29	1828.95	2949.20	3017.30
3032.33	3038.11	3080.59	3099.25	3109.93	3541.45
3567.21	3580.49	3603.54	3614.66	3630.26	3704.75

- Conformer (5); ZPE = 49402.812 cm^{-1}:

42.73	70.87	118.41	156.16	172.03	186.21
209.60	254.64	259.07	285.02	322.80	352.87
400.56	431.92	436.85	499.21	510.39	512.27
577.54	607.42	621.94	654.16	749.94	781.08
794.26	818.70	844.83	890.01	908.42	948.82
978.31	1022.16	1063.64	1095.10	1119.02	1158.79
1184.07	1197.51	1211.55	1228.79	1239.07	1280.32
1298.14	1342.65	1359.80	1370.15	1387.89	1403.56
1421.90	1452.06	1474.68	1478.73	1509.34	1571.35
1630.87	1636.41	1720.16	1837.02	2934.42	2988.36
3036.66	3038.80	3052.39	3086.34	3096.53	3546.46
3580.37	3584.42	3635.03	3636.00	3686.00	3812.08

- Conformer (6); ZPE = 49406.330 cm^{-1}:

52.30	61.44	94.20	118.23	165.73	169.21
206.74	224.20	288.40	299.39	318.04	330.05
345.75	370.51	420.07	451.66	522.13	529.30
564.04	663.21	671.29	729.48	743.73	765.79
780.37	829.43	897.11	911.96	924.17	952.45
997.69	1056.72	1075.45	1103.08	1120.16	1155.50
1160.88	1191.50	1202.32	1212.52	1241.20	1288.69
1335.05	1340.37	1357.74	1369.82	1393.75	1409.78
1423.48	1436.03	1472.60	1482.95	1501.85	1587.11
1638.56	1668.83	1713.45	1864.84	3028.91	3042.79
3047.17	3061.10	3078.42	3091.09	3112.94	3424.84
3527.04	3575.68	3605.51	3616.60	3669.95	3728.31

- Conformer (7); ZPE = 49511.995 cm^{-1}:

37.83	71.43	74.34	100.50	128.66	138.66
212.95	253.54	311.32	331.46	358.76	364.10
398.26	431.32	441.29	473.68	533.75	547.00
567.11	621.96	661.33	732.32	736.64	755.72
792.77	830.49	834.61	901.77	935.52	951.03
999.47	1039.40	1088.01	1105.05	1120.88	1144.37
1174.02	1182.99	1190.36	1238.83	1253.90	1269.05
1307.16	1339.29	1350.48	1378.83	1392.88	1402.91
1427.20	1438.29	1467.12	1478.09	1487.22	1572.35
1641.04	1684.37	1724.51	1885.79	3030.31	3038.75
3042.60	3057.64	3077.63	3093.54	3115.29	3464.90
3570.78	3578.01	3607.94	3655.32	3660.87	3716.43

- Conformer (8); ZPE = 49142.339 cm^{-1}:

65.96	81.43	117.13	141.31	175.26	193.76
231.49	257.18	278.10	325.68	340.43	347.96
379.05	398.82	439.22	488.61	527.93	555.95
582.28	583.25	684.44	694.27	751.98	761.61
788.93	809.13	856.66	881.51	909.75	950.62
979.02	1015.73	1063.75	1097.41	1116.65	1148.65
1162.21	1198.49	1212.38	1234.47	1264.28	1291.42
1307.35	1346.75	1376.33	1386.34	1402.30	1408.35
1445.64	1458.53	1470.22	1478.35	1504.72	1518.55
1632.48	1653.03	1695.05	1829.32	2959.08	2974.20
2986.16	3026.10	3037.87	3052.31	3088.14	3099.44
3552.93	3572.30	3595.67	3643.97	3645.00	3754.03

- Conformer (9); ZPE = 49556.689 cm^{-1}:

41.06	71.83	92.55	92.96	132.35	169.73
183.67	271.04	307.51	331.34	347.04	377.21
403.87	443.32	466.26	479.47	534.58	550.60
559.97	625.45	657.09	727.43	765.57	780.09
809.99	842.67	855.86	866.12	889.00	973.07
1001.09	1022.78	1068.42	1091.16	1112.08	1143.38
1154.05	1186.16	1198.47	1224.66	1247.00	1280.59
1320.54	1363.11	1366.37	1372.43	1387.79	1404.48
1412.48	1436.97	1469.47	1477.06	1489.08	1593.29
1642.01	1692.45	1724.02	1886.74	3023.56	3026.82
3045.45	3051.85	3068.68	3104.57	3136.59	3430.77
3586.89	3598.52	3608.27	3627.99	3669.56	3717.00

- Conformer (10); ZPE = 49303.856 cm^{-1}:

45.66	72.90	110.42	138.64	154.93	169.81
192.12	207.06	247.37	291.55	331.31	355.15
377.27	435.68	458.54	478.55	514.80	524.92
554.23	628.21	656.48	713.30	766.86	782.16
792.92	815.62	876.69	881.21	907.99	938.77
968.60	1024.27	1061.00	1089.60	1110.41	1145.49
1158.04	1187.56	1210.05	1222.75	1243.85	1275.33
1301.14	1331.12	1346.42	1380.50	1389.33	1395.61
1449.36	1469.98	1477.32	1484.76	1492.23	1528.08
1634.44	1672.82	1719.92	1834.92	3032.08	3046.17
3047.69	3070.59	3085.02	3090.71	3107.90	3340.66
3536.84	3595.20	3611.69	3628.48	3647.10	3741.57

- Conformer (11); ZPE = 49357.181 cm^{-1}:

63.56	92.41	108.26	139.75	157.13	173.05
192.31	201.31	248.40	286.53	295.53	333.51
390.95	437.67	455.05	497.05	533.21	548.66
571.80	662.12	691.23	720.64	752.83	775.07
799.46	832.96	858.79	884.31	905.61	948.83
967.14	1038.02	1075.57	1087.84	1118.35	1134.91
1159.33	1200.34	1204.86	1227.51	1259.58	1275.24
1308.54	1327.18	1356.01	1380.19	1391.38	1403.94
1464.41	1473.01	1477.70	1497.60	1508.71	1528.53
1637.15	1671.36	1712.09	1810.74	3003.56	3039.79
3044.24	3074.78	3082.88	3092.48	3106.14	3314.80
3539.14	3595.91	3599.17	3616.23	3619.76	3730.26

- Conformer (12); ZPE = 49341.260 cm^{-1}:

63.96	104.68	139.88	151.58	178.71	197.61
221.87	244.59	294.99	325.68	343.02	374.64
384.45	438.93	458.18	486.15	529.36	551.92
571.48	573.77	685.61	730.97	751.55	764.23
799.01	806.50	837.01	858.97	880.28	944.28
972.18	1004.37	1042.59	1092.88	1107.27	1142.05
1164.19	1195.66	1214.19	1232.11	1254.35	1282.92
1305.32	1352.72	1372.72	1383.85	1395.35	1404.43
1441.44	1454.05	1465.13	1475.92	1484.85	1528.61
1637.36	1639.72	1706.74	1844.82	2965.03	3023.46
3029.46	3043.72	3088.80	3095.17	3104.75	3257.75
3548.52	3597.16	3627.12	3630.30	3636.18	3743.42

- Conformer (13); ZPE = 49371.375 cm^{-1}:

54.54	63.93	95.96	116.22	129.79	186.00
219.46	221.99	246.04	277.19	318.66	335.32
358.92	400.36	408.11	451.15	495.43	522.13
531.39	622.12	664.39	725.70	740.51	761.62
820.70	855.04	870.66	873.21	911.28	965.34
994.64	1020.43	1066.02	1103.06	1111.97	1152.94
1171.50	1191.22	1198.79	1231.71	1242.98	1305.01
1322.73	1337.85	1372.41	1385.72	1399.82	1414.21
1417.55	1456.89	1464.28	1473.70	1477.64	1602.23
1638.76	1671.53	1706.51	1876.34	3030.46	3034.11
3039.08	3070.37	3081.02	3085.94	3098.69	3501.27
3531.61	3570.94	3610.33	3618.28	3686.64	3732.41

- Conformer (14); ZPE = 49399.919 cm^{-1}:

48.88	85.97	100.63	108.53	155.99	185.99
204.81	224.48	251.63	293.80	335.72	369.56
388.95	409.53	463.61	476.64	518.16	542.94
549.57	600.11	683.58	732.73	750.21	775.45
798.85	859.11	887.91	902.27	928.25	945.59
966.75	1015.80	1064.15	1101.91	1108.23	1148.21
1157.90	1173.10	1206.24	1222.40	1239.66	1285.51
1318.53	1333.45	1363.68	1377.55	1392.17	1401.11
1408.45	1433.33	1471.06	1482.84	1487.08	1572.99
1624.00	1671.93	1703.93	1867.38	3031.08	3039.95
3061.94	3065.37	3079.96	3088.70	3107.06	3414.40
3525.57	3589.15	3609.58	3617.27	3657.71	3733.30

- Conformer (15); ZPE = 49511.551 cm^{-1}:

41.43	60.67	77.00	101.76	136.96	162.82
213.71	275.38	317.83	338.25	351.68	369.83
386.08	428.94	469.41	513.84	540.72	559.74
563.19	579.59	661.46	732.28	739.58	783.38
788.98	823.21	843.91	866.07	930.30	948.95
1011.01	1032.34	1078.19	1106.89	1112.74	1141.77
1147.27	1183.15	1201.67	1223.74	1241.92	1277.02
1314.62	1327.10	1339.26	1375.95	1390.12	1402.13
1417.62	1442.12	1470.93	1475.79	1491.95	1571.30
1640.58	1680.74	1728.46	1883.44	3022.91	3033.57
3037.23	3054.22	3079.18	3105.58	3109.55	3415.34
3583.67	3610.89	3625.73	3653.27	3654.68	3720.49

- Conformer (16); ZPE = 49473.508 cm^{-1}:

32.30	59.44	79.26	113.53	152.97	156.22
199.05	245.06	315.00	330.76	350.62	379.15
398.97	424.16	440.28	464.41	528.91	538.03
542.72	606.04	629.90	726.45	746.24	778.40
833.74	845.67	855.76	862.78	908.25	959.98
992.03	1048.06	1067.35	1091.51	1114.83	1141.42
1151.19	1177.80	1203.24	1237.54	1250.44	1281.61
1301.04	1331.61	1368.53	1379.81	1396.53	1400.89
1413.60	1446.50	1472.51	1478.98	1484.25	1588.41
1640.80	1683.65	1725.00	1881.16	3014.13	3024.37
3043.26	3045.03	3059.55	3109.14	3137.85	3430.08
3590.61	3607.23	3611.14	3634.85	3663.65	3721.76

- Conformer (17); ZPE = 49296.432 cm^{-1}:

54.48	81.00	114.97	141.37	152.43	193.14
206.94	229.16	256.28	312.21	315.18	346.96
373.12	431.18	460.93	476.98	512.67	532.91
557.78	622.93	660.14	714.71	766.50	781.00
787.42	807.35	848.96	889.05	899.50	911.81
975.86	1002.39	1075.81	1086.66	1111.28	1139.34
1163.95	1189.67	1212.64	1224.28	1254.03	1284.84
1301.71	1303.98	1347.37	1382.04	1391.41	1397.33
1464.48	1471.63	1482.20	1487.52	1497.14	1530.95
1634.25	1637.07	1719.53	1836.51	3030.03	3036.73
3041.15	3044.97	3073.95	3086.45	3090.71	3325.05
3549.62	3596.29	3631.06	3646.06	3651.28	3744.56

- Conformer (18); ZPE = 49392.777 cm^{-1}:

53.43	62.64	94.23	119.63	167.01	170.85
223.49	225.00	291.80	310.31	319.89	336.88
345.96	371.19	422.80	456.35	525.89	532.00
562.55	663.43	668.70	729.34	744.00	762.25
780.80	828.04	856.35	908.65	921.36	936.63
991.18	1067.45	1084.15	1102.86	1113.79	1159.73
1168.90	1191.38	1204.71	1208.00	1251.06	1290.91
1318.14	1340.12	1349.89	1375.32	1389.98	1404.23
1429.05	1453.25	1472.53	1477.95	1501.34	1585.59
1638.26	1639.41	1715.52	1869.24	3008.82	3022.95
3045.73	3058.77	3074.92	3092.08	3112.40	3424.76
3538.38	3575.99	3616.32	3632.55	3668.52	3727.93

- Conformer (19); ZPE = 49600.839 cm^{-1}:

70.32	102.34	105.72	127.35	148.96	177.52
221.12	270.00	290.90	338.50	353.79	375.06
415.54	437.80	502.99	520.15	542.30	561.38
579.30	604.15	649.13	751.45	773.18	789.77
792.18	831.06	845.29	852.28	879.11	924.32
958.24	996.81	1039.53	1105.60	1132.69	1139.55
1161.45	1191.83	1214.74	1240.09	1250.42	1255.28
1316.83	1331.38	1377.75	1391.51	1397.46	1408.61
1432.68	1460.27	1469.66	1477.44	1486.88	1508.63
1627.95	1663.83	1745.71	1866.91	2987.20	3026.00
3042.58	3048.21	3077.78	3089.67	3098.12	3532.04
3565.75	3588.81	3641.50	3655.64	3670.35	3695.29

- Conformer (20); ZPE = 49479.341 cm^{-1}:

60.69	94.34	118.73	122.59	134.35	170.35
192.58	248.60	269.35	282.09	315.85	364.41
383.08	393.95	458.18	499.85	528.59	558.07
625.19	649.70	671.93	742.54	756.41	773.83
800.48	832.34	848.94	870.31	914.64	965.97
988.10	1031.27	1072.97	1091.54	1093.78	1129.34
1147.64	1173.08	1196.60	1214.40	1241.05	1278.52
1307.35	1336.99	1340.63	1373.63	1379.38	1383.10
1407.53	1458.43	1467.27	1484.55	1486.65	1581.69
1649.81	1662.34	1744.29	1819.09	3005.93	3040.81
3050.25	3075.96	3088.89	3102.67	3124.70	3535.16
3575.48	3582.33	3620.84	3633.48	3650.85	3682.39

- Conformer (21); ZPE = 49352.443 cm^{-1}:

65.21	92.87	120.19	141.99	172.33	186.78
203.25	223.54	252.56	287.31	307.76	327.42
392.01	443.85	455.37	501.63	537.73	550.29
571.72	663.49	692.50	720.90	752.76	773.06
782.94	838.73	840.80	857.92	894.84	944.56
966.78	1019.38	1074.73	1092.93	1118.88	1135.15
1159.53	1199.95	1207.68	1237.79	1249.50	1294.95
1300.73	1313.10	1355.98	1380.18	1395.38	1404.55
1469.61	1475.69	1486.22	1501.91	1512.59	1528.20
1635.98	1637.80	1713.82	1815.35	3002.62	3030.97
3033.71	3052.48	3072.39	3079.51	3090.31	3325.93
3551.23	3595.52	3598.17	3615.15	3647.36	3728.89

- Conformer (22); ZPE = 49507.677 cm^{-1}:

55.12	65.00	84.49	99.22	128.94	153.18
196.02	240.48	314.65	331.36	352.13	359.01
373.59	394.06	441.80	481.39	533.92	564.07
579.67	663.00	670.54	731.16	746.30	752.10
788.68	805.62	834.61	911.74	921.79	949.39
981.72	1035.29	1082.93	1101.10	1112.38	1137.90
1163.53	1176.77	1192.25	1237.35	1267.19	1295.35
1324.29	1334.92	1347.25	1373.46	1390.52	1399.02
1425.11	1431.91	1470.79	1474.18	1496.91	1571.19
1639.34	1688.91	1727.60	1890.32	3017.00	3036.53
3042.76	3056.48	3078.98	3093.65	3108.92	3468.80
3578.44	3582.29	3607.89	3656.31	3678.44	3716.37

- Conformer (23); ZPE = 49422.109 cm^{-1}:

38.55	66.23	68.55	85.44	111.99	170.44
198.69	261.03	307.75	323.36	326.68	368.77
390.47	430.46	493.58	533.46	539.23	549.47
571.14	595.39	653.60	727.21	741.55	784.41
789.30	809.08	841.90	870.44	899.35	932.97
979.58	1003.85	1064.13	1100.92	1113.88	1131.51
1136.36	1171.80	1201.04	1233.33	1239.87	1256.56
1302.83	1331.30	1363.45	1379.32	1390.26	1400.78
1420.18	1436.76	1472.65	1475.04	1492.10	1579.43
1642.82	1655.68	1728.55	1868.18	3021.62	3027.19
3038.16	3050.54	3090.27	3094.53	3097.93	3552.28
3579.27	3605.39	3618.80	3641.35	3657.24	3716.96

- Conformer (24); ZPE = 49454.631 cm^{-1}:

67.30	80.19	123.03	141.93	161.60	183.21
209.33	226.74	257.12	266.94	311.62	370.60
388.80	430.64	473.25	495.12	502.24	542.05
584.11	613.01	621.33	665.44	756.27	789.18
802.03	824.34	846.32	886.11	925.92	951.85
967.55	1009.93	1072.23	1104.14	1108.87	1163.92
1184.32	1194.02	1200.66	1216.55	1259.59	1270.90
1308.63	1339.74	1363.44	1377.99	1389.65	1396.96
1411.02	1422.70	1468.24	1480.83	1493.72	1548.91
1632.92	1670.27	1735.33	1839.60	3003.58	3014.20
3027.82	3053.77	3080.64	3087.39	3102.31	3542.62
3575.72	3584.69	3617.81	3630.25	3679.66	3778.52

- Conformer (25); ZPE = 49470.372 cm^{-1}:

49.30	76.42	87.78	136.35	146.68	149.68
186.67	238.36	256.13	280.17	310.03	355.37
366.69	458.30	474.35	484.77	514.17	548.30
625.28	636.58	663.05	744.88	777.16	781.87
809.77	824.94	826.89	839.38	912.71	935.49
979.28	1012.09	1075.75	1081.55	1121.81	1141.03
1145.84	1182.30	1194.99	1221.66	1256.17	1284.74
1310.16	1335.99	1349.03	1370.30	1382.70	1387.81
1416.23	1439.07	1461.81	1481.12	1491.15	1586.14
1654.11	1670.76	1724.01	1859.33	3031.68	3040.89
3046.52	3054.40	3080.31	3090.92	3112.25	3567.47
3569.43	3579.38	3642.03	3657.41	3669.81	3683.80

- Conformer (26); ZPE = 49479.362 cm^{-1}:

49.44	61.71	70.87	94.41	134.52	167.73
187.79	283.50	311.72	339.44	350.98	356.93
398.09	402.37	422.29	469.77	532.98	560.94
564.54	579.36	619.10	731.20	741.76	772.27
789.97	823.21	859.90	878.00	902.36	947.30
1002.58	1038.50	1084.47	1109.40	1119.08	1147.80
1161.50	1184.15	1197.75	1211.71	1237.34	1260.32
1302.79	1349.56	1364.45	1381.87	1384.73	1405.59
1417.78	1437.35	1471.91	1475.91	1480.00	1591.36
1638.99	1653.44	1718.87	1880.67	3029.69	3036.85
3050.90	3054.95	3082.45	3096.60	3116.90	3525.46
3572.14	3606.29	3608.56	3672.07	3674.97	3716.56

- Conformer (27); ZPE = 49364.540 cm^{-1}:

61.02	92.77	122.07	146.00	165.42	172.77
189.99	247.85	256.24	289.66	307.70	333.24
384.23	397.80	455.23	493.89	534.14	552.35
571.30	642.75	692.88	722.21	749.98	773.34
776.76	814.31	879.72	891.76	906.11	959.43
985.72	1032.47	1072.50	1090.26	1119.61	1141.01
1148.37	1187.13	1205.04	1236.66	1268.66	1302.57
1315.66	1334.79	1342.52	1381.13	1391.60	1394.54
1453.75	1476.32	1479.96	1495.80	1509.64	1528.57
1638.09	1663.11	1712.18	1815.78	3004.62	3015.68
3037.27	3074.03	3076.10	3089.23	3101.40	3322.52
3538.33	3595.95	3599.28	3616.34	3622.18	3729.80

- Conformer (28); ZPE = 49539.529 cm^{-1}:

45.81	78.63	86.90	105.18	119.40	153.59
238.53	263.42	295.17	305.16	326.72	368.31
380.40	426.82	469.02	510.26	538.76	550.75
594.52	607.11	687.78	746.76	751.60	785.60
793.74	834.69	860.86	882.49	889.60	925.71
981.93	1031.15	1065.08	1100.92	1109.42	1129.38
1161.52	1164.36	1198.87	1228.40	1254.13	1279.25
1289.19	1320.12	1360.78	1380.89	1389.06	1413.19
1419.49	1444.53	1463.92	1481.13	1483.22	1604.42
1650.89	1658.76	1724.71	1857.27	3032.75	3039.73
3052.85	3061.71	3080.60	3090.15	3117.59	3555.13
3572.19	3584.90	3606.65	3645.13	3649.02	3721.36

- Conformer (29); ZPE = 49515.639 cm^{-1}:

38.44	71.67	84.90	111.44	118.11	162.31
194.16	264.79	302.00	326.75	342.20	369.63
394.52	425.44	470.78	480.73	538.26	543.63
565.06	617.09	662.55	734.06	747.05	756.24
792.18	832.88	845.87	892.33	938.80	953.71
1011.97	1044.23	1090.12	1111.35	1115.35	1150.39
1171.90	1174.26	1189.86	1222.37	1244.53	1277.72
1314.81	1328.77	1340.71	1376.20	1391.10	1402.31
1416.84	1438.08	1470.91	1479.19	1487.35	1573.62
1640.87	1685.18	1723.84	1886.49	3007.92	3022.40
3034.38	3057.32	3079.48	3106.28	3123.24	3459.89
3570.77	3581.92	3609.74	3657.74	3664.36	3717.96

- Conformer (30); ZPE = 49508.415 cm^{-1}:

61.85	95.48	118.05	135.34	143.44	172.29
198.89	213.90	258.21	287.13	338.30	356.93
378.95	442.09	458.20	506.52	538.59	559.68
621.45	630.71	673.13	744.78	763.33	785.11
802.74	821.36	832.01	856.04	902.27	933.46
980.47	1014.79	1074.48	1079.48	1094.54	1132.66
1158.21	1174.79	1206.96	1228.69	1268.01	1279.00
1308.23	1328.91	1351.51	1377.31	1388.47	1390.99
1407.94	1433.72	1469.62	1477.93	1482.98	1583.75
1648.94	1667.94	1742.02	1818.11	3032.11	3038.75
3052.74	3065.28	3088.60	3102.71	3125.16	3550.42
3575.77	3581.49	3629.70	3630.32	3661.37	3681.75

- Conformer (31); ZPE = 49300.805 cm^{-1}:

56.15	73.93	119.80	129.23	148.90	182.96
191.19	268.46	276.76	301.90	305.82	330.35
380.39	398.56	410.69	469.52	512.59	537.74
551.65	618.74	634.98	710.91	752.85	767.39
790.07	829.06	869.89	901.59	914.02	941.83
995.90	1015.90	1074.83	1084.34	1109.43	1136.04
1155.21	1189.65	1213.57	1216.44	1273.84	1295.95
1311.59	1328.37	1340.02	1382.71	1389.44	1393.63
1436.91	1469.27	1482.06	1487.35	1493.22	1533.43
1636.37	1663.31	1716.86	1837.71	3014.27	3042.52
3043.90	3075.91	3083.18	3090.63	3101.53	3310.41
3539.86	3595.32	3624.35	3636.37	3651.58	3750.46

- Conformer (32); ZPE = 49443.727 cm^{-1}:

75.30	94.61	110.90	142.91	174.84	194.53
222.18	248.89	271.59	309.44	340.01	357.82
392.38	429.19	466.17	528.73	540.40	557.48
571.11	586.45	593.73	650.32	753.31	790.02
793.26	820.19	838.29	884.40	907.17	944.90
969.16	1011.94	1063.67	1091.72	1126.79	1158.69
1172.90	1187.08	1201.99	1227.29	1237.72	1282.74
1305.68	1344.13	1362.69	1379.87	1391.13	1400.59
1443.54	1464.64	1473.67	1489.77	1499.23	1516.20
1632.96	1641.57	1718.49	1843.91	2953.85	3012.49
3039.31	3041.97	3056.38	3088.94	3098.32	3548.58
3552.75	3588.60	3598.68	3637.17	3700.83	3739.29

- Conformer (33); ZPE = 49479.068 cm^{-1}:

55.08	77.41	95.01	132.72	153.67	174.02
199.80	264.77	271.74	282.66	318.89	335.59
380.04	424.44	474.96	489.78	520.47	548.76
560.40	662.26	682.65	737.59	762.12	789.04
793.51	857.44	896.01	915.94	926.74	969.56
993.25	1027.11	1083.02	1097.33	1115.75	1137.99
1155.42	1185.35	1213.70	1219.82	1246.09	1298.29
1310.01	1363.52	1381.04	1388.20	1392.30	1408.91
1434.40	1462.56	1474.63	1488.36	1497.84	1532.31
1638.01	1659.49	1717.62	1855.21	3018.41	3027.00
3037.02	3078.56	3083.10	3086.92	3102.35	3268.24
3526.45	3600.00	3617.24	3620.36	3623.75	3738.10

- Conformer (34); ZPE = 49297.726 cm^{-1}:

60.48	63.07	108.09	112.37	158.40	176.03
228.23	246.06	282.91	288.27	315.55	326.41
342.43	351.51	412.29	445.16	531.10	541.76
578.31	637.84	652.91	723.62	745.70	763.41
777.45	799.63	858.13	894.11	923.52	957.07
1004.66	1044.73	1075.98	1105.23	1115.47	1150.47
1166.90	1186.61	1204.52	1223.49	1242.67	1281.86
1322.31	1339.04	1345.74	1362.68	1387.74	1394.69
1429.96	1456.55	1478.23	1486.69	1498.86	1599.06
1640.05	1665.75	1716.09	1833.85	3016.13	3030.35
3038.93	3070.79	3092.60	3103.17	3130.71	3305.64
3529.59	3595.36	3611.41	3613.96	3674.62	3720.47

- Conformer (35); ZPE = 49354.206 cm^{-1}:

56.78	69.65	105.11	128.08	135.02	189.43
211.01	223.34	287.11	312.86	334.69	354.50
394.92	409.97	453.53	509.62	516.40	537.69
549.10	589.86	625.51	717.09	759.43	774.11
807.40	847.58	864.44	903.72	908.29	939.44
973.16	1013.88	1084.59	1109.83	1111.13	1138.73
1155.87	1190.88	1210.53	1231.39	1244.17	1275.22
1308.36	1331.86	1343.79	1363.96	1382.05	1404.57
1416.49	1438.71	1483.03	1490.55	1492.79	1601.16
1627.85	1643.61	1718.96	1849.41	2952.15	3029.52
3053.41	3056.16	3075.07	3104.82	3130.59	3341.36
3555.72	3594.33	3613.33	3652.07	3675.38	3722.29

- Conformer (36); ZPE = 49399.513 cm^{-1}:

45.02	73.44	85.78	126.65	144.47	160.01
184.97	231.74	261.64	284.29	300.20	313.67
348.38	374.30	421.80	479.28	488.36	524.58
583.26	618.54	665.63	716.36	749.42	760.88
784.68	854.78	870.87	910.71	935.86	982.57
993.31	1021.00	1091.96	1106.70	1122.93	1157.03
1159.87	1176.84	1203.89	1220.98	1250.19	1287.69
1323.82	1350.08	1364.59	1387.22	1392.71	1419.98
1427.75	1448.94	1468.38	1487.00	1505.09	1602.63
1641.83	1663.14	1705.84	1863.61	3029.87	3039.51
3042.23	3071.40	3086.73	3103.59	3158.46	3377.49
3524.13	3598.93	3612.92	3617.05	3682.49	3727.04

- Conformer (37); ZPE = 49387.168 cm^{-1}:

64.81	77.30	123.10	129.22	166.79	171.85
204.87	237.14	263.98	282.21	342.33	357.35
409.56	434.81	458.63	486.57	513.29	525.14
573.53	594.34	622.87	626.20	750.15	765.05
782.22	802.52	839.20	866.52	880.94	944.74
976.82	1002.73	1057.15	1093.83	1113.13	1158.57
1181.17	1199.57	1208.01	1220.34	1250.56	1280.27
1310.15	1351.62	1356.85	1375.59	1391.65	1396.27
1409.97	1448.87	1474.44	1480.97	1489.21	1556.49
1631.91	1633.84	1721.64	1832.32	2938.90	3014.27
3024.47	3045.54	3082.26	3089.71	3103.12	3542.56
3589.38	3589.82	3632.66	3677.37	3707.10	3836.02

- Conformer (38); ZPE = 49581.318 cm^{-1}:

63.17	88.41	100.64	133.29	152.59	183.22
216.90	268.89	283.27	322.49	335.07	346.27
372.83	416.58	435.22	463.90	512.15	541.15
560.41	652.02	681.02	725.16	735.43	775.10
791.77	806.14	847.28	858.07	913.67	953.57
983.77	1044.78	1072.58	1086.91	1111.18	1163.04
1168.81	1195.93	1208.35	1211.43	1270.40	1311.83
1314.62	1335.32	1349.42	1373.78	1391.58	1399.10
1409.79	1426.89	1468.17	1487.76	1502.94	1590.72
1640.31	1682.64	1706.47	1866.09	3036.12	3043.67
3067.33	3078.85	3089.39	3095.01	3132.58	3513.57
3576.59	3584.91	3615.99	3618.47	3670.31	3729.59

- Conformer (39); ZPE = 49496.804 cm^{-1}:

52.15	86.92	97.86	112.62	144.09	179.71
206.32	221.78	247.68	296.88	306.73	349.68
397.21	437.88	463.94	490.14	527.76	543.00
568.75	672.67	710.38	736.92	748.88	775.25
780.34	874.76	879.49	903.76	924.04	935.95
985.38	1014.37	1064.19	1098.01	1114.04	1124.49
1177.58	1185.38	1190.71	1232.02	1245.75	1291.53
1317.08	1339.80	1363.33	1377.60	1391.48	1407.93
1426.27	1461.96	1475.23	1482.64	1498.07	1522.03
1631.31	1671.81	1714.30	1871.60	3029.99	3039.01
3045.48	3050.76	3075.57	3093.50	3103.37	3515.43
3531.18	3560.06	3609.76	3622.07	3625.05	3742.93

- Conformer (40); ZPE = 49535.007 cm^{-1}:

51.29	57.95	88.58	100.56	113.65	158.27
191.34	282.34	302.96	331.02	353.59	357.71
375.23	428.02	448.92	479.85	534.16	566.63
575.54	658.81	670.83	734.08	741.17	769.74
795.37	813.75	848.39	900.72	932.08	966.67
991.74	1051.56	1082.33	1095.20	1111.50	1140.87
1157.06	1183.86	1190.86	1236.78	1250.31	1290.82
1312.45	1334.73	1339.15	1372.32	1386.30	1394.85
1417.90	1429.43	1476.59	1480.94	1495.69	1572.62
1641.64	1696.52	1730.94	1890.83	3008.10	3022.26
3035.79	3056.43	3078.29	3101.27	3109.95	3451.85
3578.26	3583.88	3608.38	3658.48	3675.68	3716.38

- Conformer (41); ZPE = 49592.569 cm^{-1}:

62.62	91.86	104.80	121.58	165.98	197.47
214.22	247.97	297.40	315.94	327.44	354.59
387.93	420.13	425.58	458.03	486.84	539.28
570.93	622.67	677.24	730.92	759.40	769.30
803.55	833.97	840.54	868.84	904.80	959.29
975.30	1015.74	1069.20	1094.89	1114.29	1153.40
1171.96	1183.87	1206.30	1241.16	1281.92	1288.59
1309.76	1338.64	1373.49	1379.33	1397.31	1403.30
1422.60	1433.91	1464.15	1485.17	1489.40	1602.42
1638.05	1657.01	1698.50	1877.60	3026.26	3048.86
3051.51	3070.46	3079.26	3094.30	3154.24	3517.01
3571.34	3590.09	3618.68	3621.50	3679.09	3734.17

- Conformer (42); ZPE = 49400.878 cm^{-1}:

79.11	91.06	116.39	139.94	164.31	182.86
190.23	243.03	276.55	294.83	313.08	353.61
385.98	389.09	420.71	487.57	526.38	545.59
593.88	614.93	635.49	660.72	734.42	794.12
812.66	816.67	848.58	857.34	914.51	935.55
965.72	1019.20	1066.01	1104.18	1115.45	1150.97
1192.00	1199.76	1206.92	1234.66	1236.40	1265.63
1314.22	1332.72	1355.22	1377.43	1384.25	1395.69
1411.64	1447.34	1468.38	1478.44	1524.81	1574.71
1631.68	1648.43	1714.67	1843.43	2972.22	3002.83
3031.08	3049.35	3085.38	3090.94	3104.20	3557.72
3565.63	3578.71	3647.13	3667.46	3680.27	3689.67

- Conformer (43); ZPE = 49467.242 cm^{-1}:

60.33	94.16	120.60	122.91	134.38	171.56
192.99	255.18	271.13	299.64	309.74	364.56
383.00	390.47	459.16	499.78	528.35	557.28
625.08	647.03	670.34	741.48	756.23	774.15
801.02	834.25	844.68	868.64	891.85	962.95
1007.74	1012.24	1076.63	1092.31	1109.91	1133.65
1147.25	1171.49	1198.52	1207.29	1237.07	1282.00
1306.21	1325.38	1347.60	1368.51	1375.40	1385.09
1407.69	1466.23	1476.17	1486.25	1495.12	1581.93
1640.16	1649.83	1744.69	1824.73	3008.85	3039.11
3049.11	3063.39	3076.48	3092.56	3124.90	3536.13
3576.09	3582.85	3627.26	3631.80	3652.81	3683.13

- Conformer (44); ZPE = 49574.093 cm^{-1}:

45.55	76.55	89.24	109.98	124.58	153.97
237.89	266.50	294.87	306.01	350.48	375.65
391.26	423.27	469.55	511.17	548.40	550.70
591.56	608.16	697.72	750.96	752.90	780.25
799.33	835.86	857.85	881.85	887.06	916.54
971.03	1027.70	1064.13	1101.97	1112.04	1149.54
1163.99	1174.71	1204.48	1231.97	1254.83	1276.94
1289.88	1318.73	1363.69	1385.54	1388.71	1413.29
1416.78	1445.55	1475.49	1478.90	1488.64	1589.41
1633.34	1658.37	1733.17	1857.25	3031.96	3036.14
3044.34	3063.32	3081.20	3084.59	3093.08	3557.64
3568.76	3592.28	3610.90	3648.35	3652.73	3727.19

- Conformer (45); ZPE = 49339.701 cm^{-1}:

50.19	69.06	95.48	122.80	143.76	180.88
209.66	230.41	244.84	272.04	307.26	331.29
338.58	375.84	393.65	446.76	488.05	512.37
551.91	656.10	673.71	733.73	738.76	774.02
809.57	827.86	890.69	907.81	921.21	938.97
986.76	1012.93	1087.34	1100.20	1122.30	1145.57
1164.54	1185.39	1211.98	1222.03	1233.02	1294.64
1320.44	1347.64	1365.18	1381.74	1396.17	1408.56
1413.73	1438.41	1464.87	1476.63	1501.31	1594.71
1638.63	1669.19	1707.77	1864.83	3025.61	3043.84
3057.97	3069.91	3089.77	3092.32	3121.89	3428.77
3526.63	3575.99	3605.52	3619.43	3694.72	3733.26

- Conformer (46); ZPE = 49380.372 cm^{-1}:

35.80	85.86	96.35	130.37	155.87	169.55
186.23	203.79	245.04	280.61	299.26	336.51
[truncated: 559,459 more chars]
